# LEGO-Lipophosphonoxins: A Novel Approach in Designing Membrane Targeting Antimicrobials

**DOI:** 10.1021/acs.jmedchem.2c00684

**Published:** 2022-07-15

**Authors:** Duy Dinh Do Pham, Viktor Mojr, Michaela Helusová, Gabriela Mikušová, Radek Pohl, Eva Dávidová, Hana Šanderová, Dragana Vítovská, Kateřina Bogdanová, Renata Večeřová, Miroslava Htoutou Sedláková, Radovan Fišer, Petra Sudzinová, Jiří Pospíšil, Oldřich Benada, Tomáš Křížek, Adéla Galandáková, Milan Kolář, Libor Krásný, Dominik Rejman

**Affiliations:** † Institute of Organic Chemistry and Biochemistry, Czech Academy of Sciences v.v.i., Flemingovo nám. 2, 166 10 Prague 6, Czech Republic; ‡ Department of Genetics and Microbiology, Faculty of Science, Charles University, Viničná 5, 128 43 Prague 2, Czech Republic; § Institute of Microbiology, Czech Academy of Sciences v.v.i., Vídečská 1083, 142 20 Prague 4, Czech Republic; ∥ Department of Microbiology, Faculty of Medicine and Dentistry, Palacký University Olomouc, Hněvotínská 3, 775 15 Olomouc, Czech Republic; ⊥ Department of Analytical Chemistry, Faculty of Science, Charles University, Albertov 6, 128 43 Prague 2, Czech Republic; # Department of Medical Chemistry and Biochemistry, Faculty of Medicine and Dentistry, Palacký University Olomouc, Hněvotínská 3, 775 15 Olomouc, Czech Republic

## Abstract

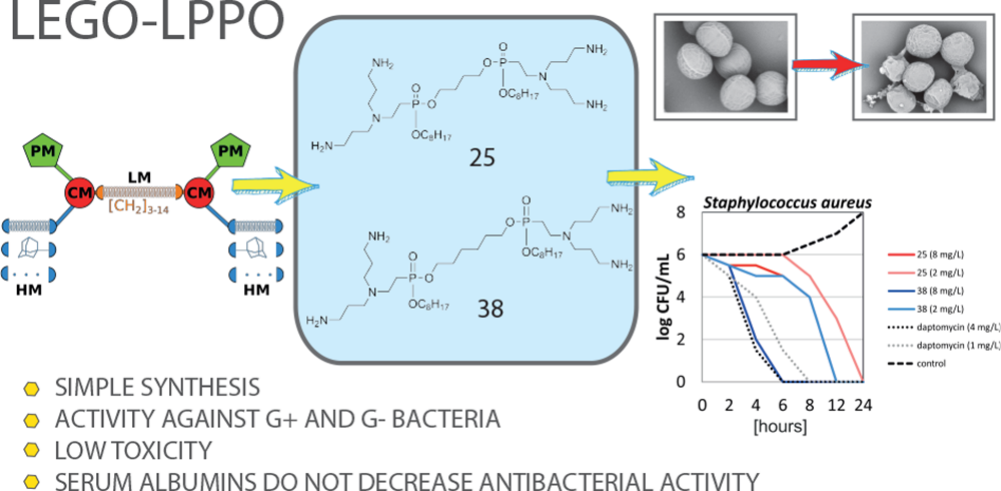

The alarming rise of bacterial antibiotic resistance requires the development of new compounds. Such compounds, lipophosphonoxins (LPPOs), were previously reported to be active against numerous bacterial species, but serum albumins abolished their activity. Here we describe the synthesis and evaluation of novel antibacterial compounds termed LEGO-LPPOs, loosely based on LPPOs, consisting of a central linker module with two attached connector modules on either side. The connector modules are then decorated with polar and hydrophobic modules. We performed an extensive structure–activity relationship study by varying the length of the linker and hydrophobic modules. The best compounds were active against both Gram-negative and Gram-positive species including multiresistant strains and persisters. LEGO-LPPOs act by first depleting the membrane potential and then creating pores in the cytoplasmic membrane. Importantly, their efficacy is not affected by the presence of serum albumins. Low cytotoxicity and low propensity for resistance development demonstrate their potential for therapeutic use.

## Introduction

Most of the antibiotics in use today are derivatives of natural products of actinomycetes and fungi.^1^ Medicinal chemistry has played a key role in modifying natural products to optimize their pharmacological properties while minimizing toxicity.^2^ Nevertheless, bacterial pathogens resistant to currently available drugs already cause at least 700,000 deaths globally a year, including 230,000 deaths from multidrug-resistant tuberculosis, a figure that could increase to 10 million deaths globally per year by 2050 under the most alarming scenario if no action is taken.^3^

Many current antibiotics were developed during the golden era of antibiotic drug discovery (1940s–1980s), and most target five biosynthetic processes that occur in actively growing bacteria: the biosynthesis of proteins, RNA, DNA, peptidoglycan, and folic acid. Most of these classical antimicrobial strategies are not effective for eradicating persistent infections in which bacteria are quiescent, and strains resistant to these antibiotics readily emerge.^4^ An attractive target for the development of antibacterial compounds is the cytoplasmic membrane as the composition of bacterial and mammalian cell membranes differs, resulting in different biophysical properties.^5^ In contrast to majority of classical antibiotics requiring metabolically active bacterial cells, membrane targeting antimicrobials are capable of also killing persistent (dormant) bacteria. A number of membrane-active compounds are already known.

Antimicrobial peptides (AMPs) and host defense peptides (HDPs) are examples of membrane-active compounds with enhanced affinity for the negatively charged prokaryotic membranes with strong electrical potential gradients as prerequisites for cellular entry or direct disruption of the bacterial cell membrane.^6,7^ These peptides are the first line of defense in many multicellular organisms and possess a broad range of biological activities, including antibacterial, antifungal, antiviral, anticancer, antiplasmodial, antiprotistal, insecticidal, spermicidal, and immunomodulatory activities. As AMPs target the cell membrane of the microorganisms for direct antimicrobial action, bacteria find it difficult to develop resistance.

**Figure 1:**
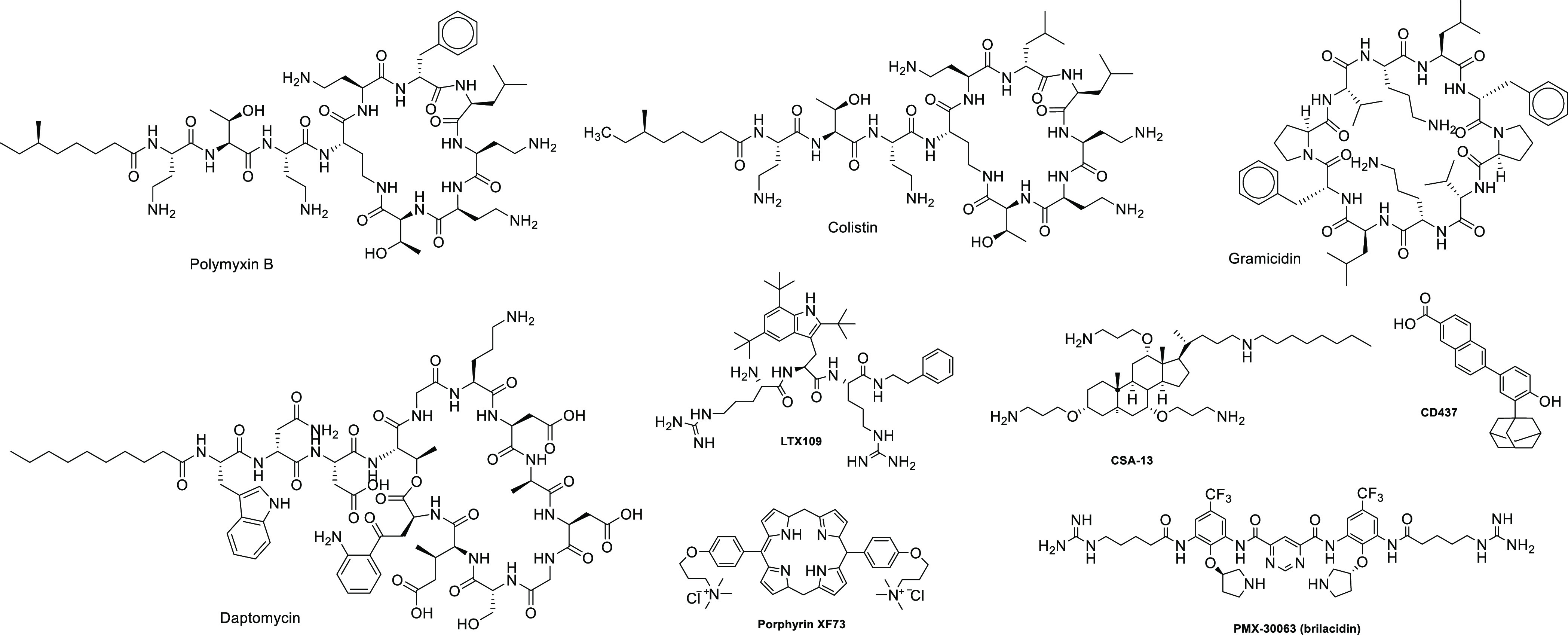
Examples of important antibiotics in clinical practice that act via the disruption of bacterial membrane and small AMP mimetics SMMTAs.

Despite so many advantages, peptide antibiotics have had relatively little clinical success largely due to their *in vivo* toxicity, limited bioavailability, and large production costs. The only peptide antibiotics that are being used clinically are shown in Figure 1 and include polymyxin B (a lipopeptide obtained from *Bacillus polymyxa*), colistin (polymyxin E, also from *B. polymyxa*), gramicidin (a linear polypeptide derived from *Bacillus brevis*), and daptomycin (a cyclic anionic lipopeptide produced by *Streptomyces roseosporus*).^5^

To overcome the limitations of AMPs, many researchers have turned their focus to peptidomimetics (small molecule membrane targeting agents [SMMTAs]) that reproduce the critical biophysical characteristics of AMPs, such as positive charge, hydrophobicity, and amphipathicity, while being relatively simple to synthesize and exhibiting better pharmacokinetic properties. Examples of SMMTAs are (i) LTX-109 (Figure 1), a first-in-class chemically synthesized, peptide-mimetic drug that is stable against protease degradation and represents a new approach to the serious challenge of *Staphylococcus aureus* nasal decolonization;^8,9^ (ii) ceragenin CSA-13 that is active against a broad spectrum of Gram-negative and Gram-positive bacteria;^10,11^ (iii) synthetic retinoid CD437 that exhibits potent *in vitro* bactericidal activity against *S. aureus* strains including the MRSA strain MW2 but not against Gram-negative species;^12^ (iv) XF-73 (exeporfinium chloride), which is a novel antistaphylococcal membrane-active photosensitizing porphyrin derivative that is active against a broad spectrum of Gram-positive bacteria;^13,14^ and (v) brilacidin (PMX-30063) that belongs to arylamide foldamers and has shown therapeutic benefits in clinical trials.^15,16^

**Figure 2:**
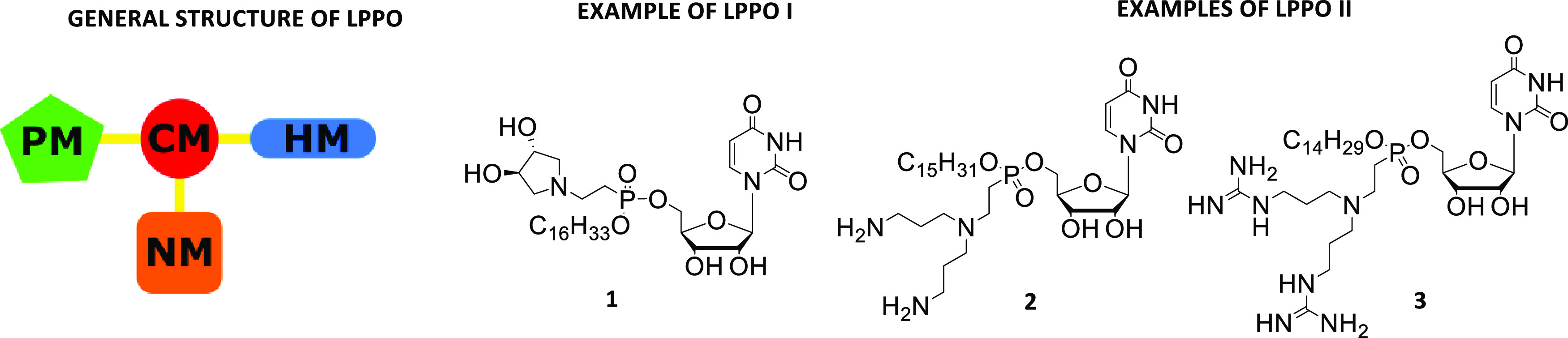
General structure of LPPO (PM = polar module, CM = connector module, HM = hydrophobic module, NM = nucleoside module) and examples of first- and second-generation LPPO.

Finally, lipophosphonoxins (LPPOs) are promising antibacterial compounds that belong among SMMTAs and that we developed several years ago. LPPOs are small amphiphilic molecules bearing positive charge(s). Their general structure (Figure 2) consists of four modules: (i) a nucleoside module (**NM**), (ii) a polar module (**PM**), (iii) a hydrophobic module (**HM**), and (iv) a phosphonate connector module (**CM**) that holds together modules i–iii. This first-generation LPPO (LPPO I)^17,18^ demonstrated excellent bactericidal activity against various Gram-positive species, including multiresistant strains such as vancomycin-resistant enterococci or methicillin-resistant *S. aureus*. The minimum inhibitory concentration (MIC) values were in the 1–12 mg/L range, while their cytotoxic concentrations against human cell lines were above this range (IC_50_ 60–100 mg/L). We have shown that at their bactericidal concentrations, LPPOs act via the disruption of the cytoplasmic membrane.^17^

However, LPPO I compounds are ineffective against Gram-negative bacteria. By redesigning the iminosugar module so that it bears more positive charges, we developed the second generation of LPPOs (LPPO II) with increased efficacy (MIC <1–6 mg/L) against Gram-positive species and an extended antibacterial activity range that now also includes serious Gram-negative pathogens such as clinically relevant strains of *Escherichia coli*, *Pseudomonas aeruginosa*, and *Salmonella* Enteritidis.^19^ LPPO II cause serious damage to the bacterial cell membrane, efflux of the bacterial cytosol, and cell disintegration.^20^ Employing model membranes (liposomes and black lipid membranes), we demonstrated that LPPO II act by creating pores in the membrane. Furthermore, LPPO II were shown to be well tolerated by live mice when administered orally (2000 mg/kg) and to cause no skin irritation in rabbits.

Importantly, using several of the most potent LPPO I and LPPO II (Figure 2), we failed to select *Bacillus subtilis*, *Enterococcus faecalis*, or *Streptococcus agalactiae* strains resistant against compound **1** (LPPO I) and, in addition, a *P. aeruginosa* strain resistant to LPPO II compound **2**, while strains resistant to known conventional antibiotics (rifampicin and ciprofloxacin, respectively) readily emerged in control experiments. Recently, LPPO II were evaluated as additives in polymethylmethacrylate (PMMA) bone cements, preventing infections,^21^ and as an antibacterial component of a polycaprolactone electrospun nanofiber dressing capable of reducing *S. aureus* induced wound infection in mice.^22^

Nevertheless, despite all the beneficial properties of LPPOs, their antibacterial activity is abolished in the presence of serum albumins. To address this limitation, we performed structure–activity relationship (SAR) studies. First, we designed compounds lacking the nucleoside module (LPPO III). The antibacterial activities of these compounds, however, were also inhibited by serum albumins. Subsequent SAR studies inspired by symmetrical peptidomimetics^15,23−30^ resulted in new modular structures that were loosely based on LPPOs. The pivotal part of these structures was the linker module (**LM**) connecting the two parts of the molecule. Hence, these compounds were termed **l**inker-**e**volved-**g**roup-**o**ptimized-**LPPO**s (LEGO-LPPOs). LEGO-LPPOs can be synthesized in a few easy steps. The best-performing LEGO-LPPOs displayed equal or better antimicrobial properties and significantly better selectivity than known LPPOs. Importantly, the antibacterial activity of LEGO-LPPOs was not affected by serum albumins. LEGO-LPPOs acted by depolarizing the microbial membrane and displayed pore forming activities. Similar to LPPO I and II, resistance to LEGO-LPPOs was not detected. Finally, additional tests demonstrated their safety and potential as therapeutics.

## Results

### LPPO III

As the first step in the optimization process of LPPOs, we removed the nucleoside module (5′-uridyl moiety) from LPPO II and created a set of asymmetrical compounds, LPPO III.

**Scheme 1:**
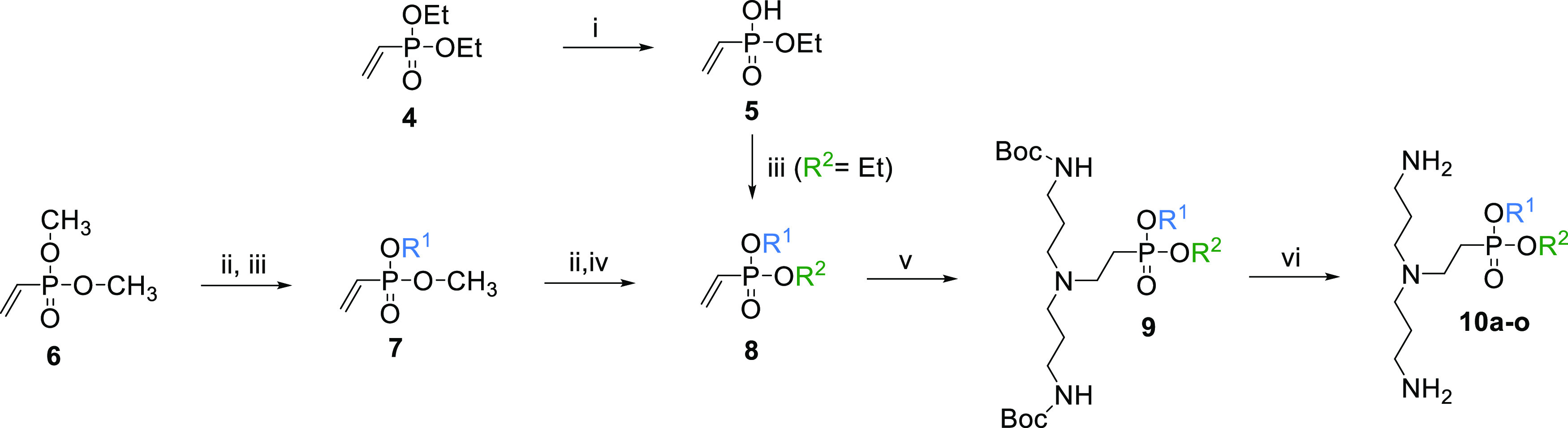
Synthesis of Compounds **10a–o** (i) aq. 2 M NaOH; (ii) 60% aq. pyridine, 60 °C; (iii) R^1^OH, TPSCl, methylimidazole, DCM or (1) oxalylchloride, DMF, DCM (2) R^1^OH, Et_3_N; (iv) R^2^OH, TPSCl, methylimidazole or (1) oxalylchloride, DMF, DCM (2) R^2^OH, Et_3_N; (v) di-*tert*-butyl (azanediylbis(propane-3,1-diyl))dicarbamate, BuOH, 105 °C; (vi) 0.5 M HCl in MeOH, rt.

In LPPO III, **NM** was replaced with various simple ester groups to obtain a series of new derivatives **10a**–**o** (Table 1) employing the same chemistry (Scheme 1) as in our original study.^19^ Diethyl (**4**) or dimethyl vinylphosphonate (**6**) served as starting material. **R^1^** and **R^2^** groups were subsequently installed by reaction of monoethyl (**5**) or monomethyl vinylphosphonate with the appropriate hydroxyl derivative **R^1^**OH and **R^2^**OH either using TPSCl (2,4,6-triisopropylbenzenesulfonylchloride) as condensing agent or via phosphonochloridate generated from monomethyl vinylphosphonate with oxalylchloride/DMF. Next, protected **PM** was introduced by Michael addition to the vinylphosphonate **8** double bonds. Finally, protecting groups were removed from **PM** by treatment with 0.5 M methanolic HCl to afford final LPPO **10a**–**o**.

**Table 1:**
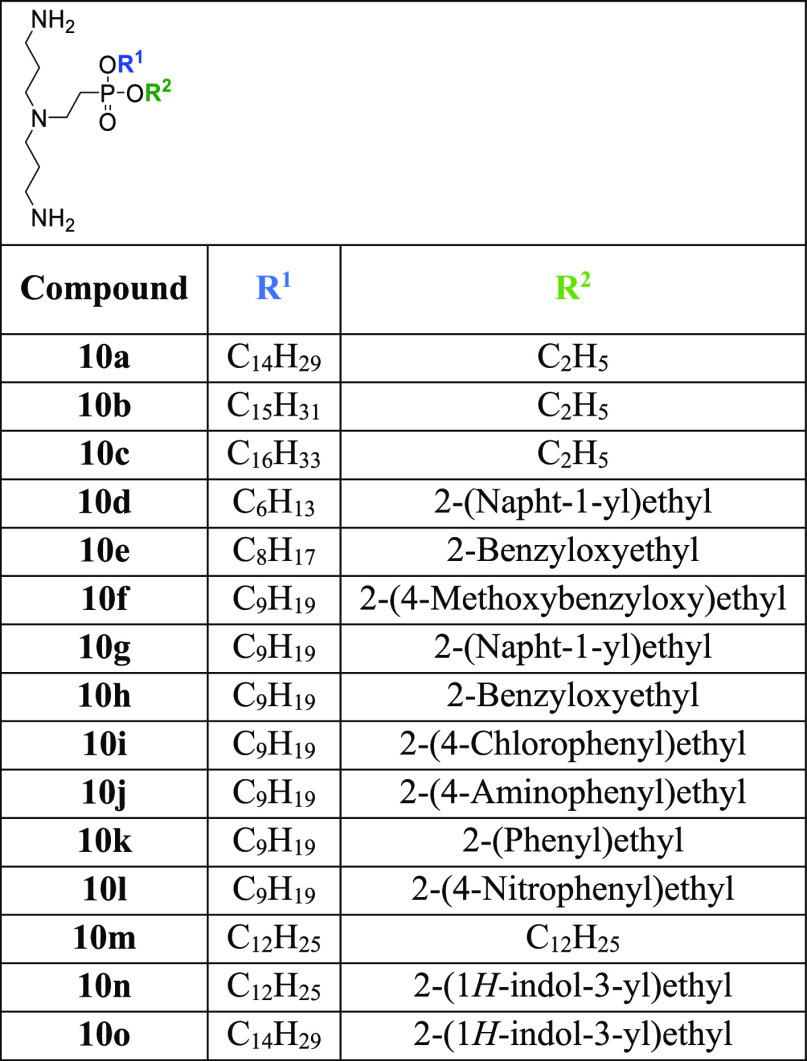
Structures of LPPO III Compounds

Next, tests of biological activities of these compounds were performed. Some of the derivatives (e.g., **10a**–**c**) retained their antibacterial activity (Table 2). However, their hemolytic activities were also relatively high, decreasing the selectivity of these compounds. Unfortunately, as in the case of original LPPO I and LPPO II, the antibacterial activities of LPPO III were lost in the presence of bovine serum albumins (BSA).

**Table 2:** Antibacterial and Hemolytic Activities of LEGO-LPPO^a^

cmpd	MIC (mg/L)						HC_50_ ± SD (mg/L)
	Gram-positive			Gram-negative			
	Efa	Sau	Sau BSA	Eco	Eco BSA	Pae	
**1**^b^	6.25	6.25	>200	>200	>200	>200	nd
**2**^c^	50	6.25	>200	6.25	>200	3.13	16
**3**^c^	6.25	3.13	>200	1.56	>200	3.13	30
**10a**	4	4	>128	4	>128	4	12.2 ± 0.8
**10b**	2	2	>128	1	>128	2	6.9 ± 0.4
**10c**	2	2	128	2	>128	4	9.4 ± 0.3
**10d**	64	64	>128	>128	>128	64	>100
**10e**	128	128	>128	>128	>128	>128	>100
**10f**	16	16	128	32	>128	16	55.0 ± 2.4
**10g**	2	2	64	4	64	4	12.8 ± 0.1
**10h**	32	32	>128	64	>128	64	79.8 ± 8.4
**10i**	2	2	64	2	64	8	17.3 ± 2.2
**10j**	128	64	>128	128	>128	128	>100
**10k**	32	16	nd	32	nd	16	58.7 ± 5.6
**10l**	16	8	64	16	128	8	50.0 ± 4.3
**10m**	25	100	nd	100	nd	100	11.3 ± 0.5
**10n**	4	2	nd	4	nd	8	12.1 ± 0.3
**10o**	4	2	nd	2	nd	2	12.4 ± 0.9

a MIC value as well as HC_50_ experiments were performed in triplicates. *Enterococcus faecalis* ATCC 29212 = CCM 4224 (Efa), *Staphylococcus aureus* ATCC 29213 = CCM 4223 (Sau)*,Escherichia coli* ATCC 25922 = CCM 3954 (Eco), *Pseudomonas aeruginosa* ATCC 27853 = CCM 3955 (Pae), BSA: in the presence of 4% BSA.

b According to ref (18).

c According to ref (19).

### LEGO-LPPO

**Figure 3:**
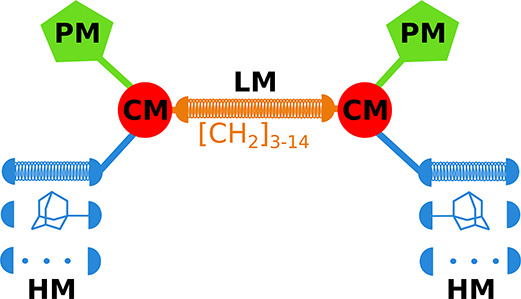
General structure of LEGO-LPPO (PM = polar module, CM = connector module, HM = hydrophobic module, LM = linker module). The coiled springs indicate variable lengths or different modules.

The failure of LPPO III prompted us to redesign the skeleton of LPPOs. We were inspired by dimeric, often symmetrical peptidomimetics. Examples of these peptidomimetics are fatty acids comprising lysine conjugates,^23^ phenylalanine conjugated lipophilic norspermidine derivatives,^24^ antimicrobial arylamide oligomers^25,26^ including brilacidin,^15^ and others.^27−30^ This resulted in symmetrical LEGO-LPPO structures, which were based on a new modular system depicted in Figure 3. We varied mostly **HM** and/or **LM**. As **PM,** bis(3-aminopropyl)amino was used in the majority of the compounds, and ethylphosphonate was exclusively used as **CM**.

**Scheme 2:**
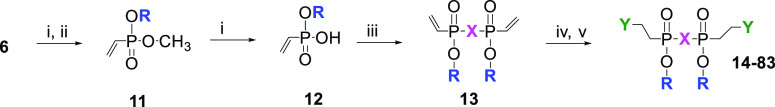
Synthesis of LEGO-LPPO Compounds (i) 60% aq. pyridine, 60 °C; (ii) ROH, TPSCl, methylimidazole, DCM or (1) oxalylchloride, DMF, DCM (2) ROH, Et_3_N; (iii) Br-X-Br, Bu_4_NOH, DMF, 90 °C, (iv) secondary amine, BuOH, 105 °C; (v) 0.5 M HCl in MeOH, rt.

The synthesis of LEGO-LPPO is depicted in Scheme 2. Dimethyl vinylphosphonate (**6**) served again as the starting material. First, **HM** (**R**) was attached by the reaction of monomethyl vinylphosphonate with the appropriate hydroxyl derivative **R**OH either using TPSCl as condensing agent or via phosphonochloridate generated from monomethyl vinylphosphonate with oxalylchloride/DMF. Tetrabutylammonium salt of monoester **12** obtained by aqueous pyridine promoted demethylation of **11** reacted with α,ω-dibromoalkane, introducing **LM** (**X**). Next, protected **PM** (**Y**) was introduced by Michael addition to the vinylphosphonate **13** double bonds. Finally, protecting groups from **PM** were removed by treatment with 0.5 M methanolic HCl to yield final LEGO-LPPO **14**–**83** (Scheme 2 and Table 3). For all compounds, cLogD values at pH 7.4 were calculated, and the gradient chromatography hydrophobicity index (CHIg) of the compounds was measured by the linear gradient HPLC method and calculated based on the retention time and acetonitrile composition as described previously.^31^

**Table 3:**
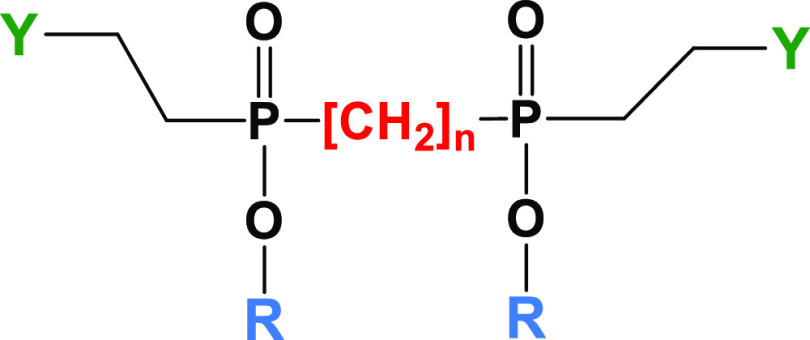

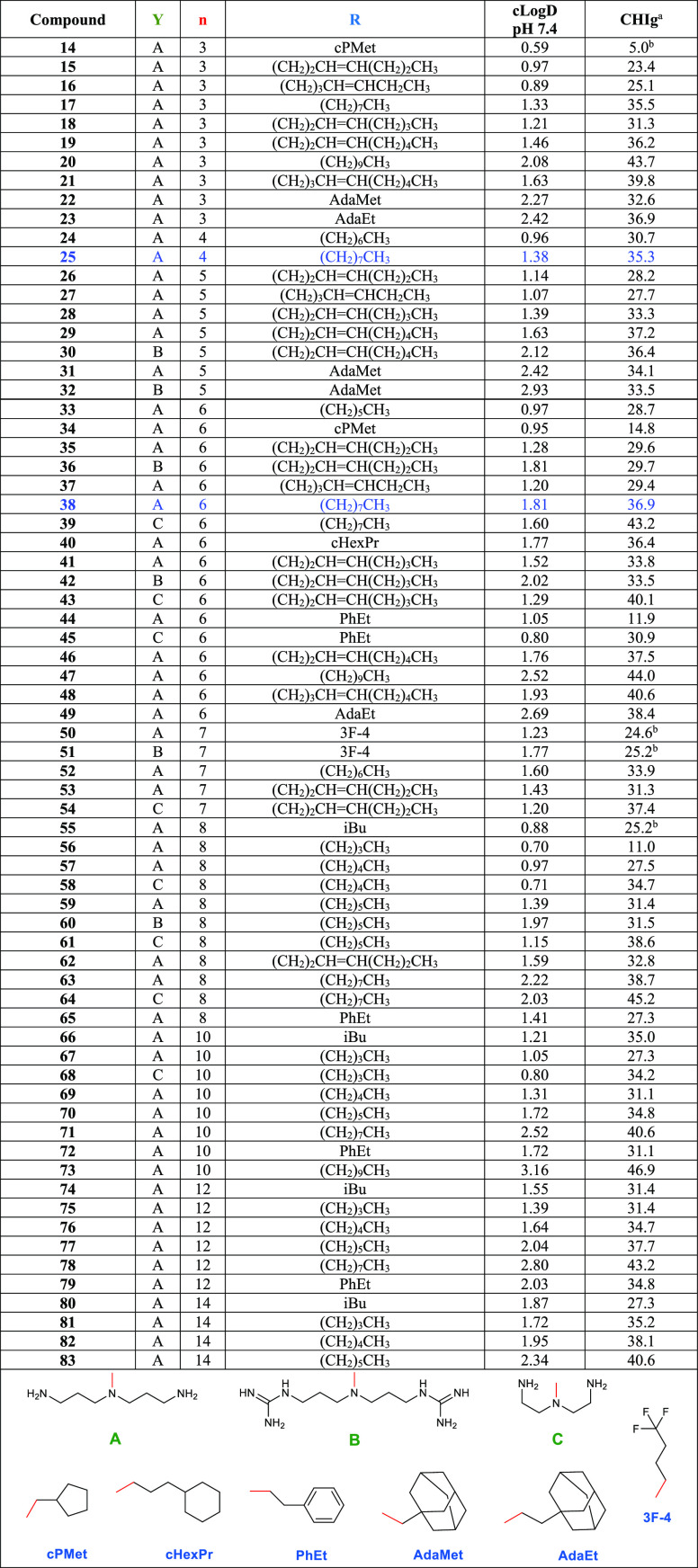
Series of LEGO-LPPO Compounds^a^^*,*^^c^

a Gradient chromatography hydrophobicity index.

b Measured using gradient B (for more polar compounds).

c Compounds **25** and **38** used in most subsequent experiments are highlighted in blue.

### Antibacterial Activities of LEGO-LPPO

#### MIC Values and Hemolytic Activity

All LEGO-LPPO compounds were tested against a panel of Gram-positive and -negative species and evaluated for cytotoxicity by determining their hemolytic activity against erythrocytes (HC_50_) (Table 4 and Table S4 for MBC values). The best compounds displayed excellent MICs ranging from <1 to 8 mg/L, while their HC_50_ were at least 20–100 times above their respective MIC values. Importantly, the presence of serum albumin only slightly, if at all, affected their antibacterial activity. To extend the testing to real clinical isolates, 24 strains of wild-type *S. aureus* and methicillin-resistant *S. aureus* (MRSA) were challenged with selected best-performing LEGO-LPPOs (**25**, **28**, **38**, and **60**; excellent antimicrobial activity and selectivity) and clinically used antibiotics (Table 5). The known antibiotics displayed a wide range of MIC values depending on the strain, and each strain was resistant to at least one antibiotic. On the contrary, the MIC values of all tested LEGO-LPPOs were uniform for each compound, irrespective of the strain, suggesting no predisposed resistance within the tested group of strains.

**Table 4:** Antibacterial and Hemolytic Activities of LEGO-LPPO^a^

cmpd	MIC (mg/L)											HC_50_ ± SD (*n* = 3) (mg/L)
	Gram-positive						Gram-negative					
	Efa	Sau	Sau BSA	Sep	SauR	EfnR	Eco	Eco BSA	EcoR	Pae	PaeR	
**14**	>128	>128	>128	>128	>128	>128	>128	>128	>128	>128	>128	>1000
**15**	>128	64	64	8	32	>128	>128	>128	>128	128	128	>200
**16**	>128	128	>128	32	64	>128	>128	>128	>128	>128	>128	>200
**17**	8	2	2	2	2	64	2	4	4	16	8	>500
**18**	128	16	32	8	16	>128	128	128	128	64	128	>200
**19**	8	2	4	2	2	64	2	4	4	8	4	>500
**20**	1	1	1	1	1	4	1	4	1	2	2	31.6
**21**	2	1	2	1	1	8	1	2	1	2	2	271
**22**	8	4	4	2	4	64	8	8	16	64	16	>500
**23**	2	1	2	2	1	32	2	1	2	16	4	196
**24**	64	4	8	2	4	>128	8	32	64	128	128	>200
**25**	4	2	2	1	2	64	2	1	2	4	4	263
**26**	>128	32	64	128	32	>128	128	128	>128	>128	>128	>250
**27**	>128	16	64	8	32	>128	128	>128	>128	>128	>128	>200
**28**	32	2	8	0.5	2	128	8	4	4	32	32	70
**29**	4	1	2	1	1	32	2	1	1	4	4	>500
**30**	4	1	1	1	1	8	1	1	1	8	8	121
**31**	4	2	2	2	2	64	2	2	4	32	8	415
**32**	4	1	0.5	0.5	1	8	1	2	1	8	8	259
**33**	128	8	16	4	16	>128	32	64	128	>128	>128	634
**34**	>128	128	>128	64	128	>128	>128	>128	>128	>128	>128	>1000
**35**	128	4	16	1	4	>128	16	32	32	128	128	>200
**36**	64	2	2	2	2	>128	2	2	2	64	64	>500
**37**	>128	8	16	4	16	>128	64	128	64	>128	>128	>200
**38**	1	0.5	1	0.5	1	8	0.5	0.5	1	2	2	162
**39**	1	0.5	0.5	0.5	0.5	4	1	1	1	2	2	79
**40**	2	1	1	0.5	1	16	1	0.5	1	4	4	183
**41**	16	1	4	0.5	1	64	2	4	2	16	16	34.8
**42**	8	1	0.2	1	1	32	4	0.5	2	16	16	169
**43**	4	1	2	0.5	1	32	1	2	2	16	8	254
**44**	>128	64	>128	64	64	>128	>128	>128	>128	>128	>128	>200
**45**	>128	32	128	16	64	>128	>128	>128	>128	>128	>128	>200
**46**	2	0.5	2	0.5	1	8	1	0.5	1	2	2	262
**47**	1	1	0.2	0.5	0.5	1	1	1	1	1	2	16
**48**	1	0.5	1	0.5	0.5	2	1	1	1	2	2	43
**49**	1	0.5	0.2	0.5	0.5	2	0.5	1	1	1	2	72.2
**50**	>128	>128	>128	128	>128	>128	>128	>128	>128	>128	>128	>200
**51**	>128	8	16	8	8	>128	128	128	128	>128	>128	>200
**52**	4	1	1	0.5	1	64	1	1	2	16	8	287
**53**	64	4	8	2	4	128	8	64	16	128	128	>200
**54**	16	2	4	0.5	1	128	4	16	32	32	32	>500
**55**	>128	>128	>128	32	>128	>128	>128	>128	>128	>128	>128	>1000
**56**	>128	>128	>128	64	>128	>128	>128	>128	>128	>128	>128	>100
**57**	>128	16	32	4	8	>128	128	>128	>128	>128	128	>100
**58**	>128	8	8	2	8	>128	>128	>128	>128	>128	>128	>200
**59**	16	1	2	0.5	2	>128	2	8	8	16	64	>500
**60**	8	0.5	0.5	0.5	0.5	16	1	2	0.5	8	8	139
**61**	16	2	1	2	2	128	16	16	64	32	64	231
**62**	64	8	8	2	4	>128	8	64	16	128	128	>200
**63**	1	0.5	0.25	0.5	0.5	4	1	0.5	1	2	2	68.5
**64**	1	0.5	0.2	0.2	0.5	2	1	0.5	1	1	1	19
**65**	128	16	32	8	16	128	64	128	128	128	128	>200
**66**	64	32	16	8	16	128	128	>128	128	128	64	>500
**67**	128	16	16	4	8	>128	>128	>128	>128	>128	>128	>500
**68**	64	64	>128	64	128	128	>128	>128	>128	64	128	55.0
**69**	32	2	8	1	2	128	64	>128	128	16	8	159
**70**	4	0.5	0.5	0.5	1	32	2	1	8	4	8	74.5
**71**	0.5	0.5	0.125	0.5	0.5	1	0.5	0.5	1	1	1	6.4
**72**	32	2	4	1	2	128	16	32	64	64	64	>500
**73**	2	2	1	1	1	2	2	16	2	4	4	5.2
**74**	32	4	16	1	4	128	>128	>128	>128	32	16	170
**75**	32	4	16	1	4	>128	128	128	>128	32	16	>500
**76**	2	1	8	0.5	1	16	16	16	32	4	4	443.5
**77**	0.5	0.25	0.5	0.125	0.5	2	1	0.5	2	2	2	63.1
**78**	1	1	0.25	0.5	1	1	1	1	1	2	2	4.0
**79**	4	1	2	0.5	4	32	4	8	16	8	8	358.0
**80**	4	2	32	0.5	4	16	16	32	16	4	8	164.7
**81**	4	2	32	0.5	2	16	16	32	32	4	4	348.5
**82**	1	1	32	0.5	1	2	2	16	2	4	2	56.4
**83**	1	0.5	2	0.5	1	2	1	4	1	2	4	10.6

a MIC value experiments were performed in triplicates. *Enterococcus faecalis* ATCC 29212 = CCM 4224 (Efa), *Staphylococcus aureus* ATCC 29213 = CCM 4223 (Sau), *Staphylococcus epidermidis* CCM 7221 (Sep), methicillin-resistant *Staphylococcus aureus* 4591 (SauR), vancomycin-resistant *Enterococcus faecium419/ANA* (EfnR), *Escherichia coli* ATCC 25922 = CCM 3954 (Eco), multiresistant *Escherichia coli* CE5556 (EcoR), *Pseudomonas aeruginosa* ATCC 27853 = CCM 3955 (Pae), multiresistant *Pseudomonas aeruginosa* R (PaeR), BSA: in the presence of 4% BSA.

**Table 5:** MIC Values (mg/L) of Four LEGO-LPPOs (**25**, **28**, **38**, and **60**) and Selected Antibiotics against 24 Wild-Type *Staphylococcus aureus* Strains

*Staphylococcus aureus*												
	PEN	OXA	AMS	CMP	ERY	CLI	CIP	GEN	**25**	**28**	**38**	**60**
4557/A	0.25^a^	0.25	0.5	2	0.25	0.125	0.25	1	1	2	0.5	0.5
4463/A	0.25^a^	0.25	0.5	2	0.25	0.125	0.25	0.25	2	4	1	0.5
4904/C	0.25^a^	0.25	0.5	2	0.25	0.125	0.25	0.25	1	4	0.5	0.5
4862/C	1^a^	0.25	1	4	0.25	0.125	0.25	0.5	1	4	0.5	0.5
4883/C	0.25^a^	0.25	0.5	4	0.25	0.125	0.25	0.25	1	2	1	0.5
4880/C	0.25^a^	0.25	0.5	2	0.25	0.125	0.25	0.5	1	4	0.5	0.5
4460/A	1^a^	0.25	1	2	0.25	0.125	0.125	0.25	1	4	1	0.5
4482/A	2^a^	0.25	1	2	0.125	0.063	0.063	0.25	1	4	0.5	0.5
4566/A	0.25^a^	0.25	0.5	4	0.25	0.125	0.25	0.25	1	4	0.5	0.5
4504/A	0.25^a^	0.25	0.5	2	0.125	0.063	0.125	>32^a^	2	4	0.5	0.25
4740/B	0.25^a^	0.25	0.5	1	>16^a^	0.125	0.5	0.25	1	4	0.5	0.5
4717/B	0.5^a^	0.25	0.5	4	0.25	0.125	0.25	0.25	1	2	0.5	0.25
4738/B	1^a^	0.25	1	2	0.25	0.125	0.25	0.25	1	4	0.5	0.5
4461/A	1^a^	0.25	1	2	0.25	0.125	0.25	0.25	2	4	0.5	0.5
4679/B	0.5^a^	0.25	1	4	0.25	0.125	0.25	>32^a^	2	4	0.5	0.5
4502/A	0.25^a^	0.25	0.5	2	0.125	0.063	0.125	>32^a^	2	4	1	0.5
4515/A	0.5^a^	0.25	0.5	2	0.25	0.125	0.125	0.25	1	4	1	0.5
5079/C	0.25^a^	0.25	0.5	2	0.25	0.063	0.25	0.25	1	4	0.5	0.5
4788/B	0.25^a^	0.125	0.5	2	0.125	0.063	0.063	0.25	1	4	0.5	0.5
78/CF	0.25^a^	0.25	0.5	1	0.125	0.063	0.25	1	2	8	1	0.5
77/CF	0.5^a^	0.25	0.5	1	>16^a^	>8^a^	0.5	>32^a^	2	4	1	0.5
MRSA 4561/A	>2^a^	>8^a^	16^a^	4	>4^a^	>4^a^	>8^a^	0.25	2	4	0.5	0.5
MRSA 4968/C	>4^a^	>16^a^	16^a^	4	>16^a^	>8^a^	>8^a^	0.25	2	4	1	0.5
MRSA 5017/C	>4^a^	>16^a^	16^a^	4	>16^a^	4^a^	>8^a^	0.5	1	4	0.5	0.5

a Strain is resistant to antibiotic (MIC value above the clinical breakpoint according to EUCAST). MRSA, methicillin-resistant *S. aureus*; PEN, penicillin; OXA, oxacillin; AMS, ampicillin/sulbactam; CMP, chloramphenicol; ERY, erythromycin; CLI, clindamycin; CIP, ciprofloxacin; GEN, gentamicin.

#### Time-Kill Kinetics

**Figure 4:**
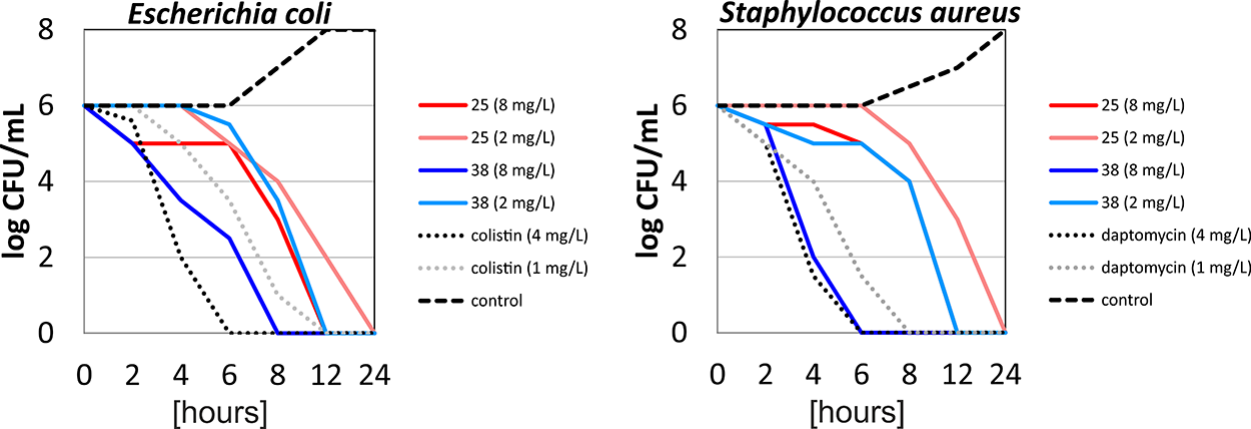
Time-kill experiments with selected LEGO-LPPOs (**25**, **38**) against *Escherichia coli* CCM 3954 and *Staphylococcus aureus* CCM 4223. In both sets of experiments, LEGO-LPPOs were tested in two concentrations: minimal bactericidal concentration (1× MBC) and a 4-times higher concentration (4× MBC). Also, membrane-acting antibiotics against Gram-positive and negative bacteria were tested where appropriate (colistin and daptomycin) also at concentrations corresponding to 1× MBC and 4× MBC.

Subsequently, we selected **25** and **38**, based on their high antibacterial activities, and determined the kinetic profiles of their bactericidal effect against *E. coli* and *S. aureus* in a time-kill assay. The results are depicted in Figure 4. Briefly, LEGO-LPPOs were typically able to reduce viable cell counts to zero within several hours. The effect was concentration dependent. In the assay, compound **38** showed kinetics comparable to those of control antibiotics, daptomycin, and colistin, respectively.

#### Persister Killing Assay CCCP

**Figure 5:**
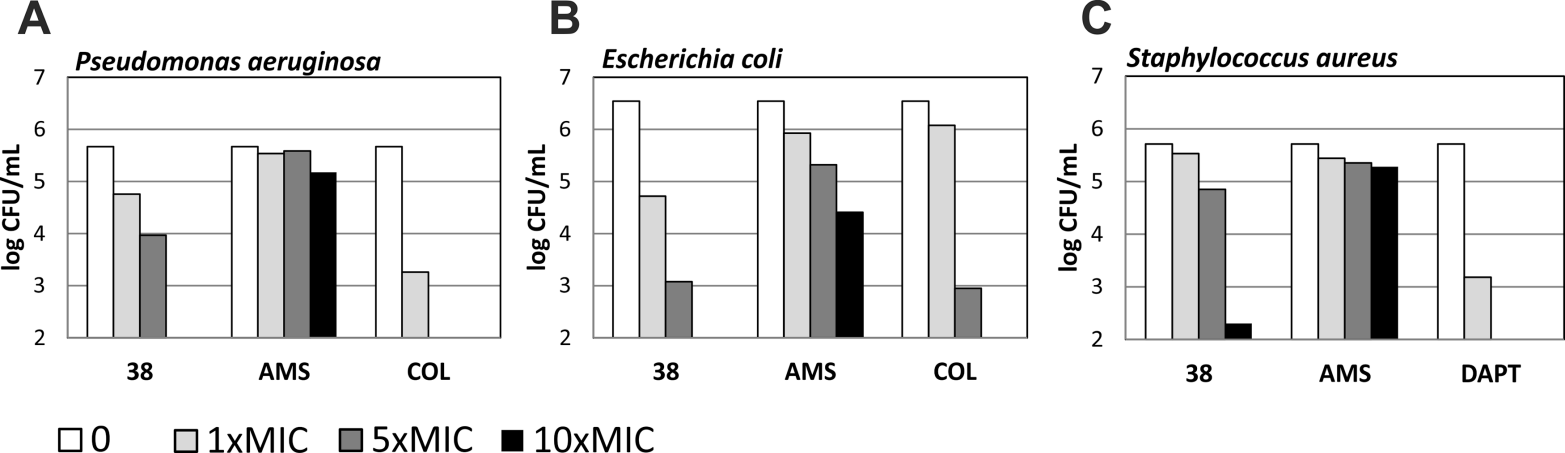
Activity of LEGO-LPPO **38** and antibiotics (ampicillin/sulbactam, daptomycin, and colistin) against CCCP-induced persisters^32^ of (A) *Pseudomonas aeruginosa* CCM 3955, (B) *Escherichia coli* CCM 3954, and (C) *Staphylococcus aureus* CCM 4223. Antimicrobial activity was evaluated by CFU/mL counting after 3 h of incubation with persisters. The detection limit used was 10^2^ CFU/mL. AMS, ampicillin/sulbactam; COL, colistin; DAPT, daptomycin.

Next, we characterized the ability of the compound with the most pronounced antibacterial activity, **38**, and three different comparable antibiotics (colistin, daptomycin, and cell wall-acting ampicillin/sulbactam [AMS]) to kill persister cells. Three bacterial species were used (*E. coli,P. aeruginosa*, and *S. aureus*). Concentrations of the tested substances corresponded to 1×, 5×, and 10× MIC. Figure 5 shows that **38** was able to kill persister cells, superior to AMS in all cases, equal to colistin in the case of *E. coli*, and less efficient than colistin with *P. aeruginosa* and daptomycin with *S. aureus*. Still, the detected activity is of note and reveals a potential for the use of **38** also against persister cells.

#### Resistance to LEGO-LPPO Is Difficult to Emerge

**Figure 6:**
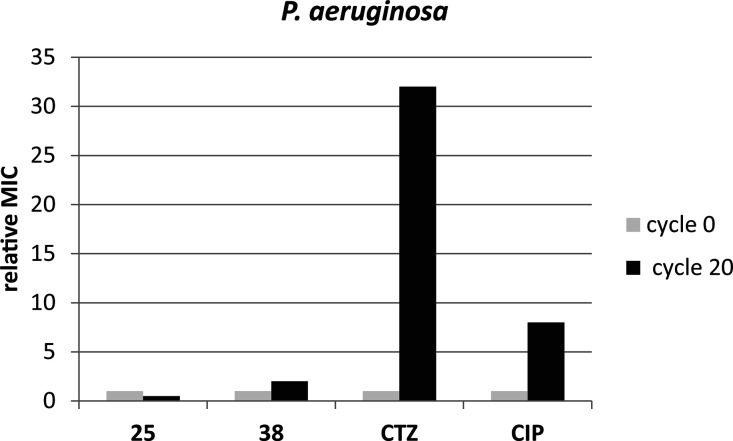
Selection of resistance. Experimental development of resistance is depicted as changes in relative MIC at the beginning of experiment and at the end (after 20 cycles). The MIC values of tested compounds were set as 1 at the beginning of the experiment to simplify the comparison. CIP, ciprofloxacin; CTZ, ceftazidime.

To evaluate the potential of LEGO-LPPOs for long-term use, we used two of the already characterized LEGO-LPPO, **25** and **38**, and attempted to select bacterial strains resistant to these compounds using the clinically relevant pathogen *P. aeruginosa* (Figure 6). With LEGO-LPPO, we were unable to select resistant strains. On the contrary, with control antibiotics, a 4-fold increase in MIC was observed by the end of the selection experiment for ciprofloxacin (from 0.5 to 2 mg/L), and a 32-fold increase of MIC was seen for ceftazidime (from 4 to 128 mg/L).

#### Effect of LEGO-LPPO on Cell Integrity

**Figure 7:**
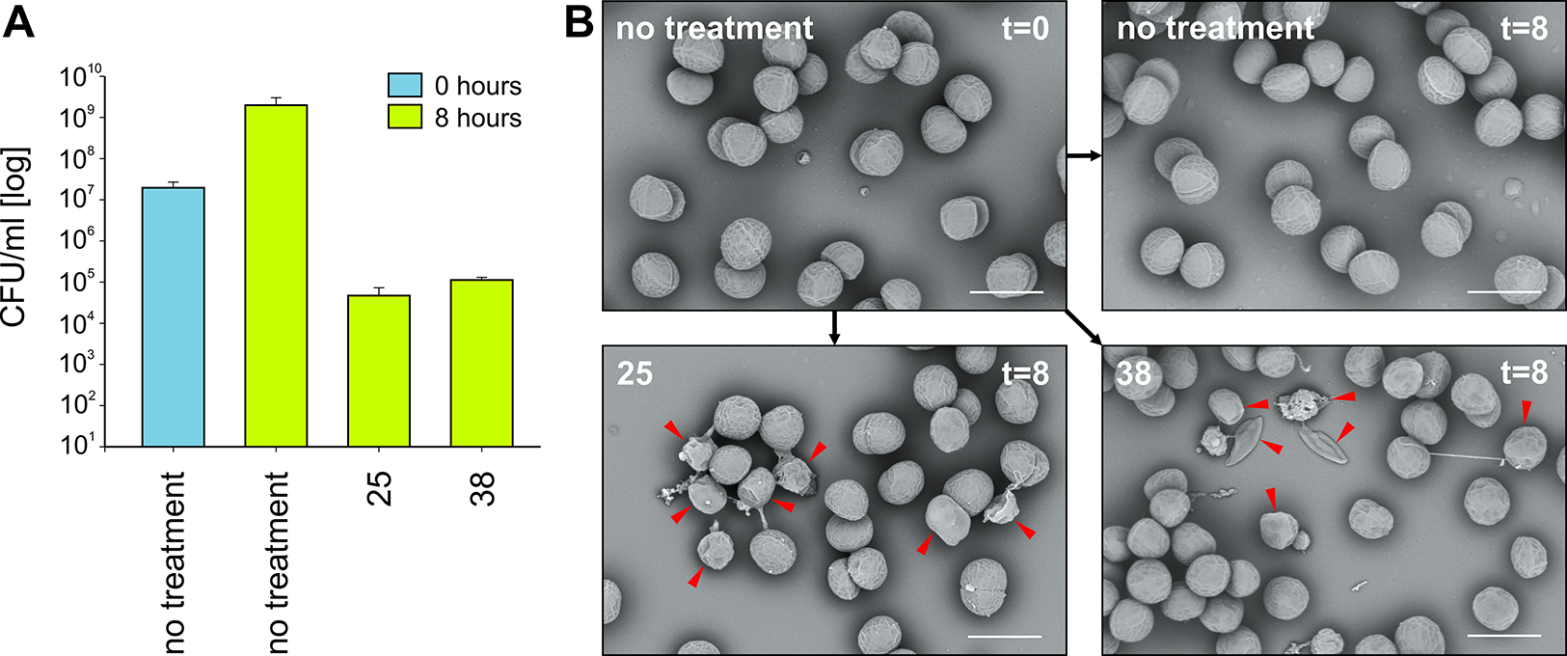
Scanning Electron Microscopy. (A) *S. aureus* cells were grown in a Mueller–Hinton (MH) medium overnight. Bacteria were inoculated into 10 mL of fresh MH medium without (no treatment) or with indicated LEGO-LPPO (**25** and **38**; 8 mg/L each). Untreated cells were collected at time points 0 and after 8 h (treated cells only after 8 h), serially diluted, and plated on MH agar dishes. Next day, CFU/mL values were determined. (B) Cells were grown in the same manner as in panel A and fixed and observed by SEM. Red arrows indicate damaged cells (empty vessels, cell wall defects) after LEGO-LPPO treatment. Scale bar = 1 μm.

As LEGO-LPPOs are derived from LPPOs that function by compromising the cell envelope, we next addressed the effect of LEGO-LPPOs on cell integrity. As the first approach, we used scanning electron microscopy (SEM) to assess the cell envelope damage, testing the effect of compounds **25** and **38** on *S. aureus*. Compared to untreated controls, visible damage of the cells induced by the presence of the compounds was detected (Figure 7), including loss of integrity and cytoplasmic content (empty vessels) and the presence of tubular structures, possibly membranous nanotubes that extrude from dying/dead cells.^33^ This result was consistent with LEGO-LPPOs targeting the cytoplasmic membrane.

#### Membrane Potential

Next, to characterize the mechanistic action of LEGO-LPPOs, we selected three compounds, each representing a different class of LEGO-LPPO with respect to activity/selectivity: (i) compound **33** with activity only against Gram-positive bacteria and excellent selectivity, (ii) compound **38** with a broad spectrum of antibacterial activity and good selectivity, and (iii) compound **71** with a broad spectrum of antibacterial activity and poor selectivity (strong hemolytic activity).

**Figure 8:**
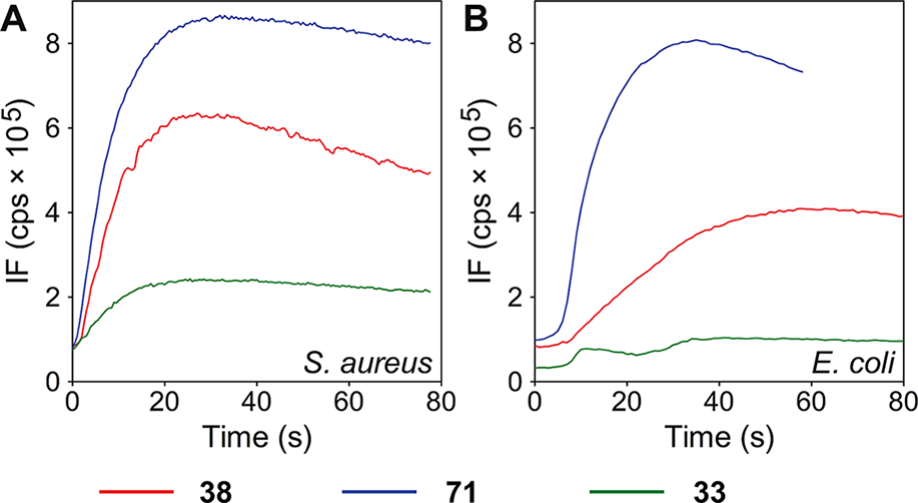
Effect of LEGO-LPPO on bacterial membrane potential. Each LEGO-LPPO was added to bacterial suspension of (A) *Staphylococcus aureus* CCM 4223 or (B) *Escherichia coli* CCM 3954 at a concentration of 2.5 mg/L. Variations in membrane potential were observed by changes in the intensity of the 3,3′-dipropylthiadicarbocyanine iodide (DiSC_3_(5)) fluorescence. The increase in intensity represents the depolarization of the bacterial membrane. Both **38** and **71** were able to depolarize rapidly the plasmatic membrane of both Gram-positive and Gram-negative bacteria within a minute after their addition. In contrast, **33** was virtually ineffective in promoting changes in bacterial membrane potential.

We started by looking at the effect of LEGO-LPPOs on the membrane potential–electrical potential gradient that bacteria maintain across the plasma membrane. We rationalized that if LEGO-LPPOs act on the cell membrane, their effect could be first detected as loss of the membrane potential. Both **38** and **71** were able to rapidly depolarize the plasmatic membrane of both *S. aureus* and *E. coli* cells within a few seconds after the addition at a concentration of 2.5 mg/L (Figure 8). In contrast, **33** was almost ineffective in promoting changes in bacterial membrane potential within the time course of the experiment. This is consistent with its relatively high MIC values compared to **38** and **71**.

We note that the loss of the membrane potential did not cause immediate cells death as Figure 4 shows no decrease in cell count within the first few hours of the experiment. This is consistent with the notion that cells generate the largest membrane potential when metabolically active^34^ and suggests that LEGO-LPPO first cause metabolic arrest followed by a slower killing effect.

#### Membrane Permeabilization: PI Assay

**Figure 9:**
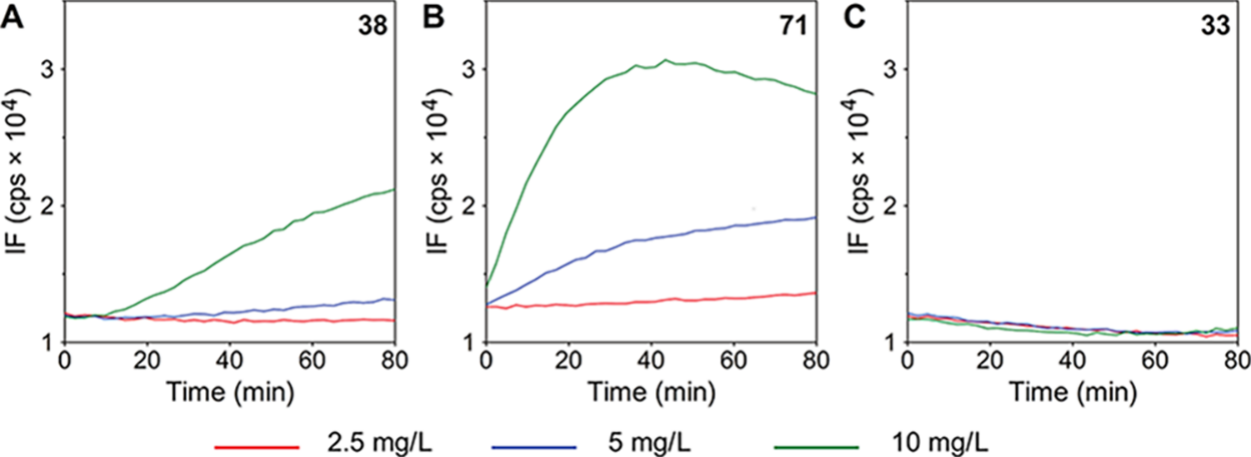
Permeabilization of *Escherichia coli* (CCM 3954) for the propidium cation by (A) **38**, (B) **71**, and (C) **33** indicated by the increase of propidium intensity of fluorescence (IF). Three different concentrations of the compounds were used. The color coding is shown below the graph.

Next, we tested the ability of the same LEGO-LPPOs as used in the previous experiment (**33**, **38**, **71**) to permeabilize *E. coli* membranes to allow passage of the large molecules of propidium iodide (PI). Both **38** and **71** promoted a relatively slow PI influx (Figure 9) in a concentration-dependent manner. Surprisingly, at the LEGO-LPPO concentration, which had been effective in membrane depolarization (2.5 mg/L, Figure 8), the propidium cation did not pass through the *E. coli* membranes. When higher concentrations were used, we observed a slow influx of PI into the bacterial cells. **33** was unable to permeabilize the *E. coli* plasmatic membrane for PI even at the highest concentration used (10 mg/mL). This was not surprising as the MIC value of **33** against *E. coli* is 32 mg/L. The dramatic differences between **38** and **71** vs **33** then may be due to variability in the affinity of these compounds for target membranes.

From the functional perspective, the permeabilizing effect requires higher concentrations or longer times than 80 min used in this experiment. This is consistent with the time interval in time-kill assays (Figure 4) where it took ∼12 h for **38** (at 2 mg/L) to reduce the bacterial population by 6 orders of magnitude.

#### LEGO-LPPOs Make Pores in the Planar Phospholipid Bilayer

**Figure 10:**
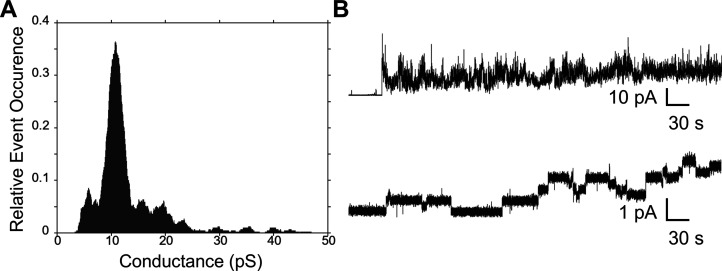
Typical recordings of pores in planar phospholipid bilayers [DPhPG in a decane/butanol (9:1) mixture] made by **38** (2.5 mg/L). (A) Histogram of single-pore conductance of **38** (*n* = 453) (B). Buffer: 1 M KCl, 10 mM Tris, pH 7.4. Membrane voltage = 50 mV.

As both **38** and **71** showed the ability to depolarize bacterial membranes (*i.e.*, promote the fluxes of small inorganic ions) but a relatively slow membrane permeabilization for propidium iodide, we characterized the pore-forming activity of **38** on planar phospholipid bilayers (made of negatively charged 1,2-diphytanoyl-*sn*-glycero-3-phospho-(1′-rac-glycerol) (DPhPG)) that are more susceptible to permeabilizing effects than natural membranes (Figure 10). In the presence of **38** at 2.5 mg/L, we observed pores of two different types: highly fluctuating unstable pores (Figure 10B, upper line) or regular pores (Figure 10B, lower line) of distinct conductance about 10 pS in 1 M KCl. At lower concentrations of **38**, we observed mostly regular pores (not shown). The most frequent conductance of ∼10 pS (Figure 10A) suggested the presence of relatively narrow and well-defined membrane pores. This was consistent with the slower efflux rate of the cellular contents than in the case of more aggressive pore-forming compounds.

#### Interaction of LEGO-LPPO with a Model Membrane: Leakage from Phospholipid Vesicles

**Figure 11:**
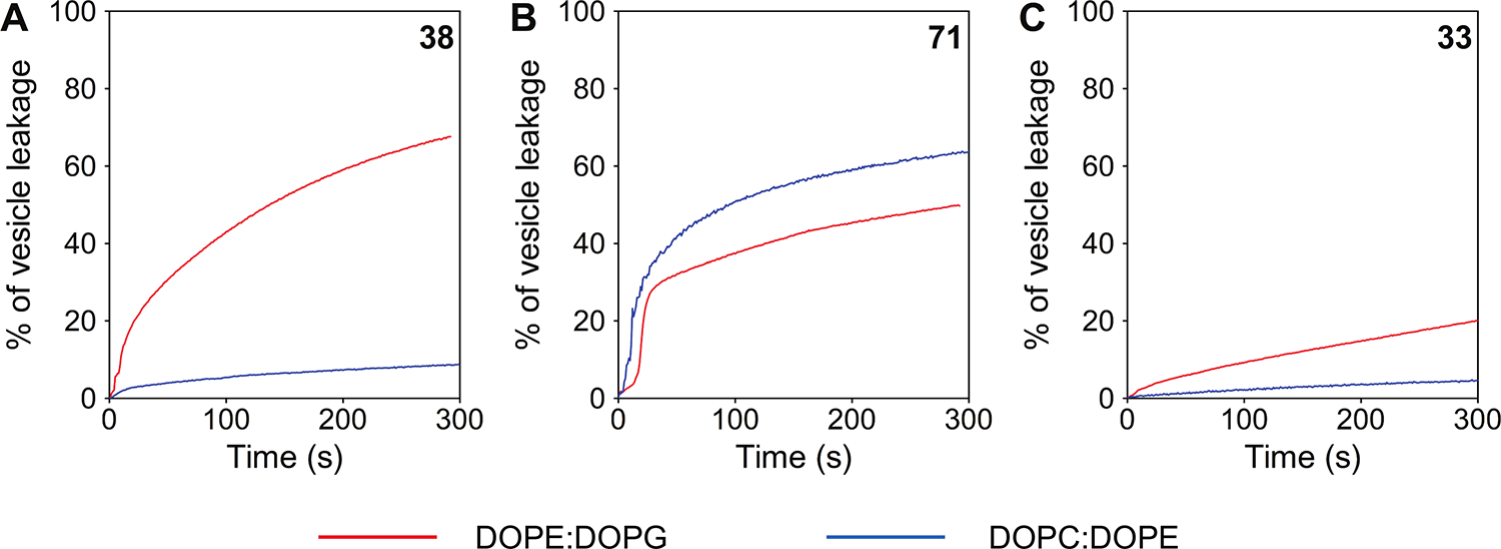
Effect of LEGO-LPPOs on liposomal membranes mimicking the composition of the plasmatic membrane of Gram-negative bacteria [DOPE/DOPG (2:1; red line)] or mammalian cells [DOPC/DOPE (2:1; blue line)]. LEGO-LPPOs were tested at a concentration of 2.5 mg/L; the identity of the compounds is indicated in the upper right hand corner of each graph. (A) For compound **38**, (B) for compound **71**, and (C) for compound **33**.

Next, we tested the relationship between the LEGO-LPPO activity and specific membrane composition. We used phospholipid vesicles created by binary mixtures without solvents using dioleoylphosphatidylethanolamine (DOPE), dioleoylphosphatidylglycerol (DOPG), and dioleoylphosphatidylcholine (DOPC). We selected the combinations of DOPE/DOPG (2:1) and DOPC/DOPE (2:1) mimicking the composition of plasmatic membrane of Gram-negative bacteria and mammalian cells, respectively. The unilamellar vesicles loaded with carboxyfluorescein (CF) revealed membrane permeabilization by increases in CF fluorescence after its leakage. In DOPE/DOPG vesicles, we observed a higher membrane disrupting activity of **38** and **71** in comparison to **33** (Figure 11), which corresponds with the ability of these molecules to permeabilize the *E. coli* plasmatic membrane (cf. Figure 8 and Figure 9). The leakage kinetics of **38** displayed a monophasic behavior (Figure 11A), whereas **71** showed typical sigmoidal kinetics (Figure 11B), suggesting differences in the mechanisms of membrane permeabilization by these two molecules. When tested on DOPC/DOPE (2:1) vesicles, **71** showed dramatically enhanced membrane disruptive activity (Figure 11B) in terms of the initial leakage rate and final maximum leakage, which explains its high hemolytic activity (lysis of red blood cells containing phosphatidylcholine in their membrane).

#### Tests of Cytotoxicity of LEGO-LPPO on Mammalian Cell Cultures, and Skin and Eye Irritation Tests

To conclude the characterization of LEGO-LPPOs, we performed a final block of experiments, further addressing the level of their cytotoxicity and safety as potential therapeutics, using several approaches. For the first approach, we selected a large set of LEGO-LPPOs to detect their potential for cytotoxicity toward HepG2 cells. These cells are nontumorigenic, display high proliferation rates, and are routinely used in drug metabolism and hepatotoxicity studies.^35,36^ The results are summarized in Table 6; the cytotoxic concentrations of the compounds were significantly above the respective MIC values.

**Table 6:** Cytotoxicity of Selected LEGO toward HepG2 Cells

compound	**15**	**22**	**23**	**25**	**29**	**31**	**35**	**38**
aCC_50_ μM (mg/L)	>99.0 (>92)	>99.0 (>102)	93.6 (99.4)	95.9 (93.6)	97.8 (99.2)	>99.0 (>105)	>99.0 (>95.9)	33.1 (33.2)

compound	**39**	**41**	**46**	**52**	**59**	**68**	**70**	**74**
aCC_50_ μM (mg/L)	80.6 (76.4)	92.5 (92.2)	30.8 (31.7)	89.5 (88.6)	>99.0 (>96.6)	>99.0 (>88.3)	17.5 (17.6)	56.2 (54.8)

a Cytotoxic dose 50%.

Next, as LEGO-LPPOs have a high potential also as topical drugs, we performed *in vitro* skin and eye irritation tests using two compounds with excellent antibacterial activities and low hemolytic, cytotoxic activities, **25** and **38**. The compounds were tested at concentrations of 20 and 200 mg/L in both tests. For potential skin irritability, we used the reconstructed human epidermis model test (RhEM).^37^ For potential eye irritability, we used the reconstructed human cornea-like epithelium (RhCE)^38^ test method designed to identify chemicals not requiring classification and labeling for eye irritation or serious eye damage. In both tests, LEGO-LPPOs performed excellently, showing no detrimental effects (Tables S1 and S2). According to the RhEM test, both tested LEGO-LPPOs could be considered to be nonirritant to skin in accordance with the UN GHS ″no category″. The tissue viabilities after 1 h exposure and 42 h post-treatment incubation were determined as higher than 60% compared to the negative control, and identical ″no category″ results were also obtained for LEGO-LPPOs in the RhCE test.

Next, we assessed the *in vivo* effect of compounds **25** and **38** at concentrations of 100 and 200 mg/L in the standard skin irritation test (according to OECD 404 and EN ISO 10993-1:2003) and at a concentration of 100 mg/L in the standard eye irritation test (according to OECD 405) performed with rabbits. For both **25** and **38**, the skin or eyes of all animals were without any adverse effects (for details see the Experimental Section and SI).

Finally, the most promising compound **25** was used in tests of the maximum tolerated dose (MTD) in mice, probing its compatibility with systemic use. The compound was administered orally (p.o.) and subcutaneously (s.c.). MTD for p.o. administration was >200 mg/kg of body weight and >15 mg/kg of body weight for s.c. administration. During the observation period after both p.o. and s.c. administration, no clinical signs were observed in the animals, and during the necropsy, no gross pathological changes were detected.

## Discussion

In this study, we present the design, synthesis, and characterization of novel antibacterial compounds termed LEGO-LPPOs. The modular structure of LEGO-LPPO allows easy, inexpensive synthesis in a few steps, an important property for prospective therapeutics.^39^ LEGO-LPPOs are active against both Gram-positive and -negative bacteria with cytotoxicity levels significantly above their MIC values. Furthermore, LEGO-LPPOs are also active against multiresistant strains of *S. aureus* as well as clinical isolates, and resistance to these compounds was not detected in the *P. aeruginosa* model. Importantly, antibacterial activities of LEGO-LPPOs are virtually unaffected by serum albumins, unlike their predecessors, LPPO II.^19^ Finally, *in vitro* and *in vivo* skin and eye irritability tests with selected LEGO-LPPOs proved their safety for potential topical applications. Furthermore, oral and subcutaneous administration of **25** demonstrated its potential for systemic use.

Based on mechanistic studies, the action of LEGO-LPPOs can be likened to the hunting strategy of spiders: trap first, kill later. LPPO-LEGO first act by depleting the membrane potential that is important for generating energy, active metabolism, and cell division.^40^ This is the fast step, the trapping. It is followed by a slower step, the killing, where LPPO-LEGOs form pores in the membrane, compromising its integrity. Both the membrane-potential-depleting and pore-forming activities of the tested compounds correlated with respective MIC values. The pore-forming activity makes LPPO-LEGOs members of SMMTAs.^5,9,11,41,42^

The activity and selectivity of LPPO-LEGOs depend on their structure. While polar modules **A**, **B**, and **C** appear to have equivalent effects, it is the hydrophobicity index of the compounds that is most correlated with their activity and selectivity. The least hydrophobic compounds **14**, **16**, **27**, **33**, **44**, **45**, **50**, **55**, **56**, and **65** (CHIg < 30) exhibited no or very low antibacterial activity. Compounds with high lipophilicity **20**, **47**, **64**, **71**, **73**, **78**, and **83** (cLogD > 2; CHIg > 40) exhibited low selectivity (high hemolytic activity). In between, there are two groups of compounds. The first group exhibits selective activity against Gram-positive bacterial strains (*S. aureus* and *S. epidermidis*) and includes compounds **24**, **36**, **54**, **61**, **69**, **72**, **74**, and **75**. The second group contains several promising broad-spectrum and low-hemolytic antibacterials: **17**, **19**, **21**, **25**, **30**, **31**, **32**, **38**, **39**, **40**, **42**, **43**, **46**, **49**, **52**, **59**, **60**, **63**, **70**, **76**, **77**, and **81**.

As **HM**, we have evaluated saturated and unsaturated linear alkyl chains, aromatic phenylethyl groups, and cyclic moieties including cyclohexyl and adamantyl. The aromatic phenethyl **HM** appeared to have too low hydrophobicity, so only compound **72** with a 10 carbon atom long **LM** exhibited some antibacterial activity (and only against *S. aureus* and *S. epidermidis*), albeit with very low hemolytic activity (HC_50_ > 500 mg/L). Cyclohexylpropyl **HM** used in combination with a 6 carbon atom long **LM** in compound **40** exhibited very good antibacterial activity (MIC 0.5–4 mg/L against most of the bacterial strains except for *E. faecium* where MIC = 16 mg/L) with low hemolytic activity (HC_50_ = 183 mg/L). Among the compounds with linear alkyl chains as **LM**, compound **25** was the best performer with MIC 1–4 mg/L against most of the bacterial strains except for *E. faecium* where MIC = 64 mg/L and with very low hemolytic activity (HC_50_ = 264 mg/L). Promising antibacterial activity (broad spectrum) and good selectivity was exhibited by compounds **23**, **31**, **32**, and **49** containing the adamantyl moiety in their LM. Finally, LEGO-LPPOs with low MIC and high hemolytic values generally displayed cLogD between 1 and 2, suggesting good solubility and permeability,^43^ similarly to a number of newly approved drugs.^44^

To summarize, we discovered a new scaffold based on original LPPOs upon which we synthesized compounds with significantly better selectivity than second-generation LPPOs (where best compounds exhibited HC_50_ values in the range of 16–30 mg/L).

To conclude, LEGO-LPPOs are a new class of compounds with broad-spectrum antibacterial activities suitable for further development into potential therapeutics. We tested in detail several selected LEGO-LPPOs, of which the two most promising compounds, **25** and **38**, were tested most thoroughly**.** Of these two compounds, **25** showed a higher therapeutic potential due to its low cytotoxicity. However, other LEGO-LPPO compounds also displayed favorable properties (*e.g.*, **23**, **31**, and **32**) and will be included in future evaluations as experiments on animal models are already being designed. These most promising compounds will undergo a more detailed SAR study focused mostly on the connector module to obtain fully stereochemically defined or completely achiral species.

## Experimental Section

### Synthesis

#### General Conditions and Used Materials

Unless stated otherwise, all used solvents were anhydrous. TLC was performed on silica gel precoated aluminum plates TLC Silica gel 60 F_254_ (Supelco), and compounds were detected by UV light (254 nm), by heating (detection of dimethoxytrityl group, orange color), by spraying with a 1% solution of ninhydrine to visualize amines, and by spraying with a 1% solution of 4-(4-nitrobenzyl)pyridine in ethanol followed by heating and treating with gaseous ammonia (blue color of mono- and diesters of phosphonic acid). Preparative column chromatography was carried out on a silica gel (40–63 μm, Fluorochem), and elution was performed at the flow rate of 60–80 mL/min. The following solvent systems were used for TLC and preparative chromatography: toluene/ethyl acetate 1:1 (T), chloroform/ethanol 9:1 (C1), ethyl acetate/acetone/ethanol/water 6:1:1:0.5 (H3), and ethyl acetate/acetone/ethanol/water 4:1:1:1 (H1). The concentrations of solvent systems are stated in volume percents (%, v/v). LC–MS (checking reaction mixtures and purity of intermediates) was performed by the Waters AutoPurification System with a 2545 Quarternary Gradient Module and 3100 Single Quadrupole Mass Detector using a LUNA C18 column (Phenomenex, 100 × 4.6 mm, 3 μm) at a flow rate of 1 mL/min. Typical conditions: mobile phase, A: 50 mM NH_4_HCO_3_, B: 50 mM NH_4_HCO_3_ in 50% aq. CH_3_CN, C: CH_3_CN, A → B/10 min, B → C/10 min, C/5 min. Preparative RP HPLC was performed on an LC5000 Liquid Chromatograph (INGOS-PIKRON, CR) using a Luna C18 (2) column (250 × 21.2 mm, 5 μm) at a flow rate of 10 mL/min by a gradient elution of methanol in 0.1% TFA (A = 0.1% TFA, B = 0.1% TFA in 50% aq. methanol, C = methanol) or without buffer. All final compounds were lyophilized from water. The purity of the final compounds was greater than 95%. Purity of final compounds was determined via LC–MS analysis using an Acquity UPLC coupled with a Xevo G2 XS QTof (Waters) and column XBridge 50 × 2.1 mm, 1.7 μm (Waters). Mass spectra were recorded on an LTQ Orbitrap XL (Thermo Fisher Scientific) using ESI ionization. NMR spectra were measured on Bruker AVANCE III HD 400 MHz (^1^H at 400.1 MHz, ^13^C at 100.6 MHz, and ^31^P at 162.0 MHz), Bruker Avance III HD 400 MHz Prodigy (^1^H at 401.0 MHz, ^13^C at 100.8 MHz, and ^31^P at 162.0 MHz), Bruker Avance III HD 500 MHz (^1^H at 500.0 MHz, ^13^C at 125.7 MHz, and ^31^P at 202.4 MHz), and JEOL JNM-ECZR 500 MHz (^1^H at 500.2 MHz, ^13^C at 125.8 MHz, and ^31^P at 202.5 MHz) spectrometers. D_2_O (reference (dioxane) = ^1^H 3.75 ppm, ^13^C 69.3 ppm. Chemical shifts (in ppm, δ scale) were referenced to TMS as internal standard, and coupling constants (*J*) are given in Hz. All intermediates were determined by LC–MS.

#### General Methods

##### General Method A1: Removal of the Phosphonate Methyl Ester Group

Methyl vinylphosphonate (1 mmol) was dissolved in 60% aqueous pyridine (20 mL), and the reaction mixture was stirred at 60 °C for 24 h. The reaction mixture was concentrated *in vacuo* at a temperature below 40 °C, and the residue was dissolved in ethanol (20 mL) and passed through a column of Dowex 50 H^+^ form (5 g). The column was washed with EtOH (40 mL). The solvent was removed *in vacuo*. The product was obtained by column chromatography on silica gel using a linear gradient of solvent system H1 (ethyl acetate/acetone/ ethanol/water 4:1:1:1) in ethyl acetate.

##### General Method A2: Removal of the Phosphonate Ethyl Ester Group

Diethyl vinylphosphonate (1 mmol) was dissolved in 1 M aqueous NaOH (20 mL), and the reaction mixture was stirred at rt for 24 h. The reaction mixture was diluted with water (20 mL) and passed through a column of Dowex 50 H^+^ form (20 g). The column was washed with water (20 mL) and ethanol (40 mL). Acidic eluate was concentrated *in vacuo* and co-evaporated with ethanol (2 × 20 mL).

##### General Method B1: Esterification of Monomethyl Vinylphosphonate Using Oxalylchloride

Mono alkyl vinylphosphonate (1 mmol) was rendered dry by co-evaporation with EtOH (10 mL/mmol) and toluene (10 mL), dissolved in DCM (3 mL), and cooled to −78 °C under an argon atmosphere. Oxalyl chloride (2 M in DCM) (0.3 mL) was slowly added, and the reaction mixture was stirred at rt for 30 min. A catalytic ammount of DMF (50 μL) was added, and the reaction mixture was stirred until gas evolution ceased. Hydroxyderivative (1 mmol) was then added followed by addition of triethylamine (1.1 mmol). The reaction mixture was stirred at rt for 12 h under an argon atmosphere. The reaction mixture was extracted with sat. soln. NaHCO_3_ (10 mL) and sat. soln. NaCl (10 mL). The organic phase was dried over Na_2_SO_4_ and concentrated *in vacuo*. The product was obtained by column chromatography using a linear gradient of acetone in toluene or a linear gradient of C1 in chloroform.

##### General Method B2: Esterification of Monomethyl Vinylphosphonate Using TPSCl

Mono alkyl vinylphosphonate (1 mmol) and hydroxyderivative (2 mmol) were rendered anhydrous by co-evaporation with DCM (2 × 10 mL) and dissolved in the same solvent (5 mL). Methylimidazole (3 mmol) and TPSCl (2 mmol) were added, and the reaction mixture was stirred at rt under an argon atmosphere for 24–48 h. The progress of the reaction was followed by TLC using a mixture of acetone/toluene (1:1). The reaction mixture was diluted with DCM (10 mL) and washed subsequently with sat. soln. NaHCO_3_ (10 mL) and brine (10 mL). Organic phases were combined, dried over Na_2_SO_4_, and concentrated *in vacuo*. The product was obtained by column chromatography using a linear gradient of acetone in toluene or a linear gradient of C1 in chloroform.

##### General Method C: Reaction of Monoalkyl Vinylphosphonate with α,ω-Dibromoalkane

Mono alkyl vinylphosphonate (1 mmol) and tetrabutylammonium hydroxide (1 mmol) were rendered anhydrous by co-evaporation with ethanol (2 × 10 mL) and DMF (10 mL) and dissolved in DMF (5 mL). α,ω-Dibromoalkane (0.36 mmol) was added, and the reaction mixture was stirred under an argon atmosphere at 90 °C for 24–48 h. The progress of the reaction was followed by TLC using a mixture of acetone/toluene (1:1). The reaction mixture was concentrated *in vacuo*, and the product was obtained by column chromatography using a linear gradient of acetone in toluene.

##### General Method D: Michael Addition

The mixture of vinylphosphonate dimer (1 mmol) and secondary amine (3 mmol) in *n*-butanol (50 mL/mmol) was stirred at 105 °C for 24–72 h in a sealed flask. The progress of the reaction was followed by TLC using mixture C1. The reaction mixture was concentrated *in vacuo*, and the product was obtained by column chromatography using a linear gradient of C1 in chloroform.

##### General Method E: Removal of Boc Protecting Groups

The starting Boc derivative (1 mmol) was dissolved in 0.5 M methanolic HCl (60 mL). The reaction mixture was stirred at rt for 24 h. The reaction mixture was concentrated *in vacuo*, and the product was obtained by precipitation from anhydrous ethyl acetate. If necessary, the final product is repurified by preparative HPLC on reversed phase using a linear gradient of methanol in 0.1% aqueous TFA followed by several codistillations with 0.5 M methanolic hydrogen chloride.

##### General Method F: Guanidination

1*H*-Pyrazole-1-carboxamidine hydrochloride (3 mmol) was added to the mixture of LPPO (1 mmol) and ethyldiisopropylamine (6 mmol) in DMF (10 mL) and stirred at rt for 24 h. The reaction mixture was concentrated *in vacuo* and purified by HPLC on reversed phase using a linear gradient of methanol in 0.1% aqueous TFA followed by several codistillations with 0.5 M methanolic hydrogen chloride.

#### Ethyl Tetradecyl (2-(Bis(3-aminopropyl)amino)ethyl)phosphonate Trihydrochloride (**10a**)

The title compound was prepared from diethyl vinylphosphonate (1.33 g, 8.1 mmol) according to general methods **A2**, **B2**, **D**, and **E** in 40% overall yield (1.74 g, 3.24 mmol) as a white amorphous solid.

^1^H NMR (500.0 MHz, CD_3_OD): 4.26–4.09 (m, 4H, C*H*_2_O), 3.50–3.38 (m, 6H, NC*H*_2_), 3.12 (t, 4H, *J* = 7.4 Hz, C*H*_2_NH_2_), 2.61–2.51 (m, 2H, PC*H*_2_), 2.27–2.18 (m, 4H, C*H*_2_CH_2_NH_2_), 1.75–1.68 (m, 2H, CH_3_(CH_2_)_11_C*H*_2_), 1.45–1.22 (m, 25H, CH_3_(C*H*_2_)_11_, C*H*_3_CH_2_O), 0.90 (m, 3H, C*H*_3_(CH_2_)_13_).

^31^C NMR (125.7 MHz, CD_3_OD): 68.18 (d, *J* = 6.7 Hz, CH_3_(CH_2_)_12_*C*H_2_O), 64.32 (d, *J* = 6.5 Hz, CH_3_*C*H_2_O), 51.00 (*C*H_2_(CH_2_)_2_NH_2_), 48.93 (*C*H_2_CH_2_P), 37.89 (*C*H_2_NH_2_), 33.05 (CH_3_(*C*H_2_)_11_), 31.56 (d, *J* = 6.0 Hz, CH_3_(CH_2_)_11_*C*H_2_CH_2_O), 30.77, 30.76, 30.74, 30.73, 30.70, 30.65, 30.45, 30.28 26.58, 23.71 (CH_3_(*C*H_2_)_11_), 23.17 (*C*H_2_CH_2_NH_2_), 21.38 (d, *J* = 140.2 Hz, P*C*H_2_), 16.79 (d, *J* = 5.9 Hz, *C*H_3_CH_2_O), 14.45 (*C*H_3_(CH_2_)_13_).

^31^P{^1^H} NMR (202.4 MHz, CD_3_OD): 27.17.

**IR***ν*_max_ (KBr) 3100–2500 (vs–s), 2960 (vs), 2922 (vs), 2852 (vs), 2750–2546 (s), 2025 (w, br), 1605 (m), 1593 (m), 1509 (m), 1484 (m), 1446 (s), 1227 (s), 1164 (m), 1095 (w), 1052 (s, sh), 1037 (s), 1025 (s), 1005 (s), 967 (m, sh), 808 (w).

**HR-MS** (ESI^+^): for C_24_H_55_N_3_O_3_P (M + H)^+^ calculated 464.39756, found 464.39783.

#### Ethyl Pentadecyl (2-(Bis(3-aminopropyl)amino)ethyl)phosphonate Trihydrochloride (**10b**)

The title compound was prepared from diethyl vinylphosphonate (1.98 g, 12.05 mmol) according to general methods **A2**, **B2**, **D**, and **E** in 43% overall yield (3.04 g, 5.18 mmol) as a white amorphous solid.

^1^H NMR (500.0 MHz, CD_3_OD): 4.26–4.16 (m, 2H, CH_3_C*H*_2_O), 4.16–4.09 (m, 2H, CH_3_(CH_2_)_13_C*H*_2_O), 3.49–3.43 (m, 2H, PCH_2_C*H*_2_), 3.42–3.36 (m, 4H, C*H*_2_(CH_2_)_2_NH_2_), 3.10 (t, 4H, *J* = 7.5 Hz, C*H*_2_NH_2_), 2.58–2.48 (m, 2H, PC*H*_2_), 2.25–2.16 (m, 4H, C*H*_2_CH_2_NH_2_), 1.76–1.68 (m, 2H, CH_3_(CH_2_)_12_C*H*_2_CH_2_O), 1.45–1.22 (m, 27H, CH_3_(C*H*_2_)_12_(CH_2_)_2_O, C*H*_3_CH_2_O), 0.90 (m, 3H, C*H*_3_(CH_2_)_14_).

^31^C NMR (125.7 MHz, CD_3_OD): 68.19 (d, *J* = 6.8 Hz, CH_3_(CH_2_)_13_*C*H_2_O), 64.32 (d, *J* = 6.6 Hz, CH_3_*C*H_2_O), 51.02 (*C*H_2_(CH_2_)_2_NH_2_), 48.80 (*C*H_2_CH_2_P), 37.85 (*C*H_2_NH_2_), 33.06 (CH_3_(*C*H_2_)_12_), 31.57 (d, *J* = 5.9 Hz, CH_3_(CH_2_)_12_*C*H_2_CH_2_O), 30.79, 30.78, 30.76, 30.75, 30.72, 30.66, 30.46, 30.29, 26.59, 23.72 CH_3_(*C*H_2_)_12_), 23.22 (*C*H_2_CH_2_NH_2_), 21.29 (d, *J* = 140.5 Hz, P*C*H_2_), 16.77 (d, *J* = 5.9 Hz, *C*H_3_CH_2_O), 14.44 (*C*H_3_(CH_2_)_14_).

^31^P{^1^H} NMR (202.4 MHz, CD_3_OD): 27.22.

**IR** ν_max_ (KBr) 2923 (vs), 2854 (vs), 2500–2800, 2022 (w), 1602 (m), 1468 (s), 1394 (m), 1379 (w), 1227 (s), 1060 (s, sh), 1018 (vs), 990 (s, sh), 722 (w).

**HR-MS** (ESI^+^): for C_25_H_57_N_3_O_3_P (M + H)^+^ calculated 478.41321, found 478.41332.

#### Ethyl Hexadecyl (2-(Bis(3-aminopropyl)amino)ethyl)phosphonate Trihydrochloride (**10c**)

The title compound was prepared from diethyl vinylphosphonate (1.45 g, 8.81 mmol) according to general methods **A2**, **B2**, **D**, and **E** in 27% overall yield (1.43 g, 2.38 mmol) as a white amorphous solid.

^1^H NMR (500.0 MHz, CD_3_OD): 4.26–4.16 (m, 2H, CH_3_C*H*_2_O), 4.16–4.08 (m, 2H, CH_3_(CH_2_)_14_C*H*_2_O), 3.49–3.43 (m, 2H, PCH_2_C*H*_2_), 3.43–3.37 (m, 4H, C*H*_2_(CH_2_)_2_NH_2_), 3.11 (t, 4H, *J* = 7.5 Hz, C*H*_2_NH_2_), 2.59–2.49 (m, 2H, PC*H*_2_), 2.26–2.16 (m, 4H, C*H*_2_CH_2_NH_2_), 1.75–1.68 (m, 2H, C*H*_2_CH_2_O), 1.45–1.22 (m, 29H, CH_3_(C*H*_2_)_13_(CH_2_)_2_O, C*H*_3_CH_2_O), 0.90 (m, 3H, C*H*_3_(CH_2_)_15_).

^31^C NMR (125.7 MHz, CD_3_OD): 68.17 (d, *J* = 6.7 Hz, CH_3_(CH_2_)_14_*C*H_2_O), 64.30 (d, *J* = 6.6 Hz, CH_3_*C*H_2_O), 51.00 (*C*H_2_(CH_2_)_2_NH_2_), 48.80 (PCH_2_*C*H_2_), 37.84 (*C*H_2_NH_2_), 33.05 (CH_3_(*C*H_2_)_13_), 31.56 (d, *J* = 6.0 Hz, *C*H_2_CH_2_O), 30.77, 30.76, 30.74, 30.72, 30.66, 30.46, 30.29, 26.58, 23.72 (CH_3_(*C*H_2_)_13_), 23.19 (*C*H_2_CH_2_NH_2_), 21.30 (d, *J* = 140.4 Hz, P*C*H_2_), 16.77 (d, *J* = 5.9 Hz, *C*H_3_CH_2_O), 14.45 (*C*H_3_(CH_2_)_15_).

^31^P{^1^H} NMR (202.4 MHz, CD_3_OD): 27.21.

**IR** ν_max_ (KBr) 3100–2500 (vs-s), 2990 (vs), 2960 (vs), 2916 (vs, br), 2852 (vs), 2725 (s), 2676 (s), 2617 (s), 2545 (s), 2488 (s), 2033 (w, br), 1604 (s), 1543 (m), 1508 (m), 1485 (s), 1468 (s), 1401 (m), 1390 (m), 1367 (m), 1227 (vs), 1164 (m), 1095 (m), 1052 (s, sh), 1036 (s, sh), 1025 (vs), 1010 (vs), 968 (s, sh), 807 (m), 721 (m).

**HR-MS** (ESI^+^): for C_26_H_59_N_3_O_3_P (M + H)^+^ calculated 492.42886, found 492.42893.

#### Hexyl 2-(Naphthalen-1-yl)ethyl (2-(Bis(3-aminopropyl)amino)ethyl)phosphonate Trihydrochloride (**10d**)

The title compound was prepared according to general methods **A1**, **B2**, **D**, and **E** from mono methyl vinylphosphonate (1.66 g, 12.2 mmol) in 7% overall yield (0.52 g, 0.88 mmol) as a white solid.

^1^H NMR (401 MHz, CD_3_OD): 8.17–8.11 (m, 1H, Naph*H*), 7.93–7.88 (m, 1H, Naph*H*), 7.84–7.78 (m, 1H, Naph*H*), 7.61–7.43 (m, 4H, Naph*H*), 4.53–4.42 (m 2H, OC*H*_2_CH_2_Naph), 3.94–3.77 (m, 2H, (CH_2_)_4_C*H*_2_O), 3.53 (t, 2H, *J* = 6.6 Hz, C*H*_2_Naph), 3.31–3.22 (m, 6H, C*H*_2_N), 3.07 (t, 4H *J* = 7.5 Hz, C*H*_2_NH_2_), 2.49–2.36 (m, 2H, PC*H*_2_), 2.22–2.07 (m, 4H, C*H*_2_CH_2_NH_2_), 1.57–1.46 (m, 2H, CH_2_C*H*_2_CH_2_O), 1.34–1.19 (m, 6H, CH_3_(C*H*_2_)_3_), 0.96–0.83 (m, 3H, C*H*_3_).

^13^C NMR (101 MHz, CD_3_OD): 135.44, 134.63, 133.36, 129.96, 128.71, 128.67, 126.84, 126.64, 124.63 (*C*_Naph_), 68.07 (d, *J* = 6.8 Hz, *C*H_2_O), 50.99 (*C*H_2_(CH_2_)_2_NH_2_), 48.64 (PCH_2_*C*H_2_), 37.84 (*C*H_2_NH_2_), 34.62 (d, *J* = 6.2 Hz, *C*H_2_Naph), 32.43 (CH_3_CH_2_*C*H_2_), 31.37 (d, *J* = 6.1 Hz, CH_2_*C*H_2_CH_2_O), 26.13 (CH_3_(CH_2_)_2_*C*H_2_), 23.57 (CH_3_*C*H_2_), 23.21 (*C*H_2_CH_2_NH_2_), 21.14 (d, *J* = 140.6 Hz, P*C*H_2_), 14.34 (*C*H_3_).

^31^P{^1^H} NMR (162 MHz, CD_3_OD): 28.53.

**IR** ν_max_ (KBr) 3200–2600 (vs, vbr), 2600–2500 (m), 2956 (vs), 2930 (vs), 2859 (s), 1620 (w), 1598 (w), 1547 (w), 1470 (m), 1397 (w), 1239 (m), 1043 (m), 1050–1000 (m), 801 (w), 776 (w), 624 (w), 588 (w), 555 (w).

**HR-MS** (ESI^+^): for C_26_H_45_N_3_O_3_P (M + H)^+^*m*/*z* calculated 478.31931, found 478.31909.

#### 2-(Benzyloxy)ethyl Octyl (2-(Bis(3-aminopropyl)amino)ethyl)phosphonate Trihydrochloride (**10e**)

The title compound was prepared according to general methods **A1**, **B2**, **D**, and **E** from mono methyl vinylphosphonate (0.86 g, 6.30 mmol) in 8% overall yield (0.28 g, 0.47 mmol) as a white solid.

^1^H NMR (400 MHz, CD_3_OD): 7.44–7.27 (m, 5H, Ph*H*), 4.60 (d, 2H, *J* = 1.9 Hz, C*H*_2_Ph), 4.37–4.24 (m, 2H, POC*H*_2_CH_2_O), 4.16–4.04 (m, 2H, (CH_2_)_6_C*H*_2_O), 3.81–3.71 (m, 2H, POCH_2_C*H*_2_O), 3.51–3.38 (m, 2H, PCH_2_C*H*_2_), 3.30–3.22 (m, 4H, C*H*_2_(CH_2_)_2_NH_2_), 3.06 (t, 4H, *J* = 7.2 Hz, C*H*_2_NH_2_), 2.61–2.43 (m, 2H, PC*H*_2_), 2.24–2.08 (m, 4H, C*H*_2_CH_2_NH_2_), 1.74–1.61 (m, 2H, CH_2_C*H*_2_CH_2_O), 1.42–1.21 (m, 10H, CH_3_(C*H*_2_)_5_), 0.96–0.86 (m, 3H, C*H*_3_).

^13^C NMR (101 MHz, CD_3_OD): 139.36 (*C*_quat_), 129.55, 129.05, 128.94 (*C*_Ph_), 74.05 (*C*H_2_Ph), 70.35 (d, *J* = 5.6 Hz, POCH_2_*C*H_2_O), 68.12 (d, *J* = 6.9 Hz, CH_3_(CH_2_)_6_*C*H_2_O), 67.35 (d, *J* = 6.6 Hz, PO*C*H_2_CH_2_O), 51.06 (*C*H_2_(CH_2_)_2_NH_2_), 48.70 (PCH_2_*C*H_2_), 37.87 (*C*H_2_NH_2_), 32.96 (CH_3_CH_2_*C*H_2_), 31.51 (d, *J* = 6.2 Hz, CH_2_*C*H_2_CH_2_O), 30.32, 30.24, 26.54 ((*C*H_2_)_3_(CH_2_)_2_O), 23.69 (CH_3_*C*H_2_), 23.27 (*C*H_2_CH_2_NH_2_), 21.33 (d, *J* = 141.5 Hz, P*C*H_2_), 14.42 (*C*H_3_).

^31^P{^1^H} NMR (162 MHz, CD_3_OD): 28.96.

**IR** ν_max_ (KBr) 3700–3000 (s, br), 2952 (vs), 2935 (vs), 2855 (vs), 2700–2500 (m, br), 1602 (m), 1540 (w), 1492 (m), 1465 (m), 1456 (m), 1337 (w), 1226 (s), 1100–100 (vs), 1053 (vs), 1023 (vs), 1004 (s), 902 (w), 878 (w), 733 (m), 697 (m), 556 (w).

**HR-MS** (ESI^+^): for C_30_H_66_N_4_O_6_P_2_ (M + H)^+^*m*/*z* calculated 486.34552, found 486.34590.

#### 2-((4-Methoxybenzyl)oxy)ethyl Nonyl (2-(Bis(3-aminopropyl)amino)ethyl)phosphonate Trihydrochloride (**10f**)

The title compound was prepared according to general methods **A1**, **B2**, **D**, and **E** from mono methyl vinylphosphonate (1.95 g, 14.3 mmol) in 10% overall yield (0.88 g, 1.37 mmol) as a white solid.

^1^H NMR (400 MHz, CD_3_OD): 7.30–7.14 (m, 2H, *o*-Ph*H*), 7.01–6.82 (m, 2H, *m*-Ph*H*), 4.36–4.25 (m, 2H, POC*H*_2_CH_2_O), 4.04–3.89 (m, 2H, (CH_2_)_7_C*H*_2_O), 3.78 (s, 3H, C*H*_3_O), 3.37–3.27 (10H, m, C*H*_2_N, CH_3_OPhC*H*_2_O), 3.09 (t, 4H, *J* = 7.5 Hz, C*H*_2_NH_2_), 2.96 (t, 2H, *J* = 6.6 Hz, POCH_2_C*H*_2_O), 2.52–2.35 (m, 2H, PC*H*_2_), 2.24–2.10 (m, 4H, C*H*_2_CH_2_NH_2_), 1.69–1.58 (m, 2H, CH_2_C*H*_2_CH_2_O), 1.39–1.25 (m, 12H, CH_3_(C*H*_2_)_6_), 0.95–0.85 (m, 3H, C*H*_3_).

^13^C NMR (101 MHz, CD_3_OD): 160.05 (CH_3_O*C*), 131.23 (*o*-Ph*C*), 130.68 (OCH_2_*C*qPh), 115.09 (*m*-Ph*C*), 68.83 (d, *J* = 6.9 Hz, PO*C*H_2_CH_2_O), 68.08 (d, *J* = 6.8 Hz, (CH_2_)_6_*C*H_2_O), 55.78 (*C*H_3_O), 51.05 (*C*H_2_(CH_2_)_2_NH_2_), 48.65, 48.63 (PCH_2_*C*H_2_, CH_3_OPh*C*H_2_O), 37.87 (*C*H_2_NH_2_), 36.86 (d, *J* = 6.4 Hz, POCH_2_*C*H_2_O), 33.04 (CH_3_CH_2_*C*H_2_), 31.53 (d, *J* = 6.3 Hz, (CH_2_)_6_*C*H_2_CH_2_O), 30.64, 30.39, 30.28, 26.53 ((*C*H_2_)_4_(CH_2_)_2_O), 23.72 (CH_3_*C*H_2_), 23.29 (*C*H_2_CH_2_NH_2_), 21.15 (d, *J* = 140.9 Hz, P*C*H_2_), 14.43 (*C*H_3_).

^31^P{^1^H} NMR (162 MHz, CD_3_OD): 28.25.

**IR** ν_max_ (KBr) 3100–2500 (vs, vbr), 2956 (s), 2926 (vs), 2856 (s), 1612 (m), 1584 (w), 1514 (m), 1444 (w, sh), 1247 (s), 1180 (w), 1068 (m), 1041 (m), 1007 (m), 827 (w), 811 (w), 758 (w), 703 (w), 562 (w), 522 (w).

**HR-MS** (ESI^+^): for C_26_H_51_N_3_O_4_P (M + H)^+^*m*/*z* calculated 500.36117, found 500.36069.

#### 2-(Naphthalen-1-yl)ethyl Nonyl (2-(Bis(3-aminopropyl)amino)ethyl)phosphonate Trihydrochloride (**10g**)

The title compound was prepared according to general methods **A1**, **B2**, **D**, and **E** from mono methyl vinylphosphonate (0.52 g, 3.82 mmol) in 9% overall yield (0.21 g, 0.34 mmol) as a white solid.

^1^H NMR (400 MHz, CD_3_OD): 8.18–8.10 (m, 1H, Naph*H*), 7.92–7.90 (m, 1H, Naph*H*), 7.86–7.78 (m, 1H, Naph*H*), 7.62–7.42 (m, 4H, Naph*H*), 4.54–4.40 (m, 2H, C*H*_2_CH_2_Naph), 3.96–3.77 (m, 2H, (CH_2_)_7_C*H*_2_O), 3.53 (t, 2H, *J* = 6.6 Hz, C*H*_2_Naph), 3.29–3.22 (m, 6H, C*H*_2_N), 3.06 (t, 4H, *J* = 7.6 Hz, C*H*_2_NH_2_), 2.49–2.30 (m, 2H, PC*H*_2_), 2.19–2.04 (m, 4H, C*H*_2_CH_2_NH_2_), 1.57–1.46 (m, 2H, CH_2_C*H*_2_CH_2_O), 1.36–1.17 (m, 12H, CH_3_(C*H*_2_)_6_, 0.95–0.84 (m, 3H, C*H*_3_).

^13^C NMR (101 MHz, CD_3_OD): 135.44, 134.61, 133.36, 129.97, 128.72, 128.65, 127.38, 126.84, 126.63, 124.62 (Naph*C*), 68.08 (d, *J* = 6.9 Hz), 68.05 (d, *J* = 6.8 Hz, *C*H_2_O), 50.99 (*C*H_2_(CH_2_)_2_NH_2_), 48.60 (PCH_2_*C*H_2_), 37.84 (*C*H_2_NH_2_), 34.63 (d, *J* = 6.2 Hz, *C*H_2_Naph), 33.02 (CH_3_CH_2_*C*H_2_), 31.41 (d, *J* = 6.0 Hz, CH_2_*C*H_2_CH_2_O), 30.58, 30.37, 30.22, 26.44 ((*C*H_2_)_4_(CH_2_)_2_O), 23.72 (CH_3_*C*H_2_), 23.24 (*C*H_2_CH_2_NH_2_), 21.14 (d, *J* = 140.6 Hz, P*C*H_2_), 14.44 (*C*H_3_).

^31^P{^1^H} NMR (162 MHz, CD_3_OD): 28.58.

**IR** ν_max_ (KBr) 3445 (m, br), 2957 (vs), 2925 (vs), 2855 (s), 2667 (m), 2627 (m), 2558 (m), 1611 (w), 1599 (w), 1511 (w), 1473 (m), 1396 (w), 1257 (m), 1238 (m), 1049 (m), 1020 (m), 1002 (m), 801 (m), 776 (m), 589 (w), 556 (w).

**HR-MS** (ESI^+^): for C_29_H_51_N_3_O_3_P (M + H)^+^*m*/*z* calculated 520.36626, found 520.36578.

#### 2-(Benzyloxy)ethyl Nonyl (2-(Bis(3-aminopropyl)amino)ethyl)phosphonate Trihydrochloride (**10h**)

The title compound was prepared according to general methods **A1**, **B2**, **D**, and **E** from mono methyl vinylphosphonate (0.98 g, 7.20 mmol) in 9% overall yield (0.40 g, 0.66 mmol) as a white solid.

^1^H NMR (400 MHz, CD_3_OD) δ 7.46–7.27 (m, 5H, Ph*H*), 4.59 (s, 2H, C*H*_2_Ph), 4.36–4.25 (m, 2H, POC*H*_2_CH_2_O), 4.19–4.05 (m, 2H, (CH_2_)_7_C*H*_2_O), 3.76 (t, 2H, *J* = 4.4 Hz, POCH_2_C*H*_2_O), 3.51–3.38 (m, 2H, PCH_2_C*H*_2_), 3.30–3.22 (m, 4H, C*H*_2_(CH_2_)_2_NH_2_), 3.05 (t, 4H, *J* = 7.5 Hz, C*H*_2_NH_2_), 2.61–2.42 (m, 2H, PC*H*_2_), 2.25–2.06 (m, 4H, C*H*_2_CH_2_NH_2_), 1.74–1.61 (m, 2H, CH_2_C*H*_2_CH_2_O), 1.29–1.33 (m, 12H, CH_3_(C*H*_2_)_6_, 0.96–0.84 (m, 3H, C*H*_3_).

^13^C NMR (101 MHz, CD_3_OD): 139.36, 129.56, 129.05, 128.96 (*C*_Ph_), 74.06 (*C*H_2_Ph), 70.36 (d, *J* = 5.4 Hz, POCH_2_*C*H_2_O), 68.13 (d, *J* = 7.0 Hz, (CH_2_)_6_*C*H_2_O), 67.35 (d, *J* = 6.7 Hz, PO*C*H_2_CH_2_O), 51.07 (*C*H_2_(CH_2_)_2_NH_2_), 48.65 (PCH_2_*C*H_2_), 37.86 (*C*H_2_NH_2_), 33.03 (CH_3_CH_2_*C*H_2_), 31.52 (d, *J* = 6.1 Hz, CH_2_*C*H_2_CH_2_O), 30.62, 30.39, 30.29, 26.55 ((*C*H_2_)_4_(CH_2_)_2_O), 23.72 (CH_3_*C*H_2_), 23.29 (*C*H_2_CH_2_NH_2_), 21.29 (*J* = 142 Hz, P*C*H_2_), 14.43 (*C*H_3_).

^31^P{^1^H} NMR (162 MHz, CD_3_OD): 29.87.

**IR** ν_max_ (KBr) 3700–3000 (s, br), 2956 (vs), 2925 (vs), 2855 (vs), 2700–2500 (m, br), 1604 (m), 1544 (w), 1496 (m), 1467 (m), 1456 (m), 1337 (w), 1226 (s), 1100–100 (vs), 1050 (vs), 1026 (vs), 1004 (s), 902 (w), 878 (w), 733 (m), 697 (m), 556 (w).

**HR-MS** (ESI^+^): for C_26_H_51_N_3_O_6_P (M + H)^+^*m*/*z* calculated 500.36117, found 500.36134.

#### 4-Chlorophenethyl Nonyl (2-(Bis(3-aminopropyl)amino)ethyl)phosphonate Trihydrochloride (**10i**)

The title compound was prepared according to general methods **A1**, **B2**, **D**, and **E** from mono methyl vinylphosphonate (1.24 g, 9.14 mmol) in 11% overall yield (0.59 g, 0.96 mmol) as a white solid.

^1^H NMR (400 MHz, CD_3_OD): 7.36–7.32 (m, 2H, *m*-Ph*H*), 7.32–7.28 (m, 2H, *o*-Ph*H*), 4.39–4.29 (m, 2H, C*H*_2_CH_2_PhCl), 4.05–3.88 (m, 2H, (CH_2_)_7_C*H*_2_O), 3.40–3.31 (m, 6H, C*H*_2_N), 3.09 (t, 4H, *J* = 7.5 Hz, C*H*_2_NH_2_), 3.02 (t, 2H, *J* = 6.4 Hz, C*H*_2_PhCl), 2.54–2.38 (m, 2H, PC*H*_2_), 2.26–2.10 (m, 4H, C*H*_2_CH_2_NH_2_), 1.69–1.56 (m, 2H, CH_2_C*H*_2_CH_2_O), 1.39–1.24 (m, 12H, CH_3_(C*H*_2_)_6_), 0.96–0.85 (m, 3H, C*H*_3_).

^13^C NMR (101 MHz, CD_3_OD): 137.74 (CH_2_*C*_quat_), 133.61 (*C*_quat_Cl), 131.90 (*o*-*C*_Ph_), 129.60 (*m*-*C*_Ph_), 68.28 (d, *J* = 6.6 Hz, *C*H_2_CH_2_PhCl), 68.15 (d, *J* = 6.7 Hz, (CH_2_)_7_*C*H_2_O), 51.07 (*C*H_2_(CH_2_)_2_NH_2_), 48.66 (PCH_2_*C*H_2_), 37.87 (*C*H_2_NH_2_), 36.96 (d, *J* = 6.4 Hz, ClPh*C*H_2_), 33.04 (CH_3_CH_2_*C*H_2_), 31.52 (d, *J* = 6.0 Hz, CH_2_*C*H_2_CH_2_O), 30.64, 30.39, 30.29, 26.53 ((*C*H_2_)_4_(CH_2_)_2_O), 23.72 (CH_3_*C*H_2_), 23.30 (*C*H_2_CH_2_NH_2_), 21.18 (d, *J* = 140.5 Hz, P*C*H_2_), 14.43 (*C*H_3_).

^31^P{^1^H} NMR (162 MHz, CD_3_OD): 28.29.

**IR** ν_max_ (KBr) 3200–2500 (vs, br), 2959 (vs), 2927 (vs), 2856 (s), 1598 (w), 1469 (m), 1493 (m), 1231 (w), 1190 (w, sh), 1107 (w), 1091 (w), 1051 (m), 1009 (m), 821 (w), 723 (w).

**HR-MS** (ESI^+^): for C_25_H_48_N_3_O_3_ClP (M + H)^+^*m*/*z* calculated 504.31163, found 504.31108.

#### 4-Aminophenethyl Nonyl (2-(Bis(3-aminopropyl)amino)ethyl)phosphonate Tetrahydrochloride (**10j**)

The title compound was prepared according to general methods **A1**, **B2**, **D**, and **E** from mono methyl vinylphosphonate (2.05 g, 15.1 mmol) in 9% overall yield (0.86 g, 1.36 mmol) as a white solid.

^1^H NMR (401 MHz, CD_3_OD): 7.54–7.47 (m, 2H, *o*-Ph*H*), 7.42–7.35 (m, 2H, *m*-Ph*H*), 4.45–4.29 (m, 2H, H_2_NPhCH_2_C*H*_2_O), 4.11–3.97 (m, 2H, (CH_2_)_7_C*H*_2_O), 3.40–3.32 (m, 6H, C*H*_2_N), 3.15–3.05 (m, 6H, C*H*_2_NH_2_, H_2_NPhC*H*_2_), 2.57–2.42 (m, 2H, PC*H*_2_), 2.25–2.13 (m, 4H, C*H*_2_CH_2_NH_2_), 1.73–1.61 (m, 2H, CH_2_C*H*_2_CH_2_O), 1.44–1.23 (m, 12H, CH_3_(C*H*_2_)_6_), 0.94–0.85 (m, 3H, C*H*_3_).

^13^C NMR (101 MHz, CD_3_OD): 140.07 (*C*_quat_NH_2_), 132.03 (*o*-*C*_Ph_**)**, 130.85 (CH_2_*C*_quat_), 124.18 (*m*-*C*_Ph_), 68.22 (d, *J* = 6.8 Hz, (CH_2_)_7_*C*H_2_O), 68.02 (d, *J* = 6.6 Hz, H_2_NPhCH_2_*C*H_2_), 51.00 (*C*H_2_(CH_2_)_2_NH_2_), 48.64 (PCH_2_*C*H_2_), 37.89 (*C*H_2_NH_2_), 37.07 (d, *J* = 6.5 Hz, H_2_NPh*C*H_2_), 33.03 (CH_3_CH_2_*C*H_2_), 31.54 (d, *J* = 6.0 Hz, CH_2_*C*H_2_CH_2_O), 30.64, 30.41, 30.30, 26.59 ((*C*H_2_)_4_(CH_2_)_2_O), 23.73 (CH_3_*C*H_2_), 23.28 (*C*H_2_CH_2_NH_2_), 21.22 (d, *J* = 140.2 Hz, P*C*H_2_), 14.44 (*C*H_3_).

^31^P{^1^H} NMR (162 MHz, CD_3_OD): 28.82.

**IR** ν_max_ (KBr) 3434 (m), 3100–2500 (m), 2956 (m), 2929 (m), 2857 (m), 1624 (w), 1513 (w), 1468 (w), 1226 (w), 1180 (w, sh), 1065 (w), 1007 (m), 824 (w), 558 (w).

**HR-MS** (ESI^+^): for C_25_H_50_N_4_O_3_P (M + H)^+^*m*/*z* calculated 485.36150, found 485.36085.

#### Nonyl Phenethyl (2-(Bis(3-aminopropyl)amino)ethyl)phosphonate Trihydrochloride (**10k**)

The title compound was prepared according to general methods **A1**, **B2**, **D**, and **E** from mono methyl vinylphosphonate (3.47 g, 25.5 mmol) in 11% yield (1.56 g, 2.73 mmol) as a white solid.

^1^H NMR (400 MHz, CD_3_OD): 7.46–7.16 (m, 5H, Ph*H*), 4.44–4.28 (m, 2H, C*H*_2_CH_2_Ph), 4.11–3.88 (m, 2H, (CH_2_)_7_C*H*_2_O), 3.34–3.27 (m, 6H, C*H*_2_N), 3.08 (t, 4H, *J* = 7.5 Hz, C*H*_2_NH_2_), 3.03 (t, 2H, *J* = 6.6 Hz, PhC*H*_2_), 2.54–2.31 (m, 2H, PC*H*_2_), 2.26–2.07 (m, 4H, C*H*_2_CH_2_NH_2_), 1.71–1.53 (m, 2H, CH_2_C*H*_2_CH_2_O), 1.44–1.23 (m, 12H, CH_3_(C*H*_2_)_6_), 0.98–0.83 (m, 3H, C*H*_3_).

^13^C NMR (101 MHz, CD_3_OD): 138.81, 130.22, 129.65, 127.84 (*C*_Ph_), 68.54 (d, *J* = 6.7 Hz), 68.04 (d, *J* = 6.8 Hz, *C*H_2_O), 51.01 (*C*H_2_(CH_2_)_2_NH_2_), 48.61 (PCH_2_*C*H_2_), 37.91 (*C*H_2_NH_2_), 37.71 (d, *J* = 6.3 Hz, Ph*C*H_2_), 33.03 (CH_3_CH_2_*C*H_2_), 31.50 (d, *J* = 6.0 Hz, CH_2_*C*H_2_CH_2_O), 30.62, 30.38, 30.26, 26.53 ((*C*H_2_)_4_(CH_2_)_2_O), 23.72 (CH_3_*C*H_2_), 23.31 (*C*H_2_CH_2_NH_2_), 21.21 (d, *J* = 140.3 Hz, P*C*H_2_), 14.44 (*C*H_3_).

^31^P{^1^H} NMR (162 MHz, CD_3_OD): 28.24.

**IR** ν_max_ (KBr) 3100–2600 (vs, br), 3026 (s, sh), 2958 (vs), 2926 (vs), 2856 (s), 1603 (w), 1587 (w, sh), 1500 (w, sh), 1468 (m), 1455 (m), 1230 (m), 1096 (w, sh), 1053 (m), 1010 (m), 881 (w), 815 (w), 751 (w), 724 (w), 700 (w), 573 (vw), 548 (vw), 489 (vw).

**HR-MS** (ESI^+^): for C_25_H_49_N_3_O_3_P (M + H)^+^*m*/*z* calculated 470.35061, found 470.35038.

#### 4-Nitrophenethyl Nonyl (2-(Bis(3-aminopropyl)amino)ethyl)phosphonate Trihydrochloride (**10l**)

The title compound was prepared according to general methods **A1**, **B2**, **D**, and **E** from mono methyl vinylphosphonate (0.91 g, 6.68 mmol) in 9% overall yield (0.37 g, 0.59 mmol) as a white solid.

^1^H NMR (400 MHz, CD_3_OD): 8.32–8.15 (m, 2H, *m*-Ph*H*), 7.69–7.51 (m, 2H, *o*-Ph*H*), 4.54–4.34 (m, 2H, O_2_NPhCH_2_C*H*_2_), 4.08–3.89 (m, 2H, (CH_2_)_7_C*H*_2_O), 3.48–3.25 (m, 6H, C*H*_2_N), 3.23–3.14 (m, 2H, O_2_NPhC*H*_2_), 3.14–3.01 (m, 4H, C*H*_2_NH_2_), 2.63–2.40 (m, 2H, PC*H*_2_), 2.29–2.07 (m, 4H, C*H*_2_CH_2_NH_2_), 1.70–1.51 (m, 2H, CH_2_C*H*_2_CH_2_O), 1.43–1.17 (m, 12H, CH_3_(C*H*_2_)_6_), 1.00–0.83 (m, 3H, C*H*_3_).

^13^C NMR (101 MHz, CD_3_OD): 148.37 (*C*_quat_NO_2_), 147.08 (CH_2_*C*_quat_), 131.48 (*o*-*C*_Ph_), 124.62 (*m*-*C*_Ph_), 68.21 (d, *J* = 6.8 Hz, (CH_2_)_7_*C*H_2_O), 67.67 (d, *J* = 6.5 Hz, *C*H_2_CH_2_PhNO_2_), 51.03 (*C*H_2_(CH_2_)_2_NH_2_), 48.72 (PCH_2_*C*H_2_), 37.89 (*C*H_2_NH_2_), 37.33 (d, *J* = 6.5 Hz, *C*H_2_PhNO_2_), 33.01 (CH_3_CH_2_*C*H_2_), 31.49 (d, *J* = 5.9 Hz, CH_2_*C*H_2_CH_2_O), 30.61, 30.36, 30.26, 26.51 ((*C*H_2_)_4_(CH_2_)_2_O), 23.70 (CH_3_*C*H_2_), 23.28 (*C*H_2_CH_2_NH_2_), 21.27 (d, *J* = 140.2 Hz, P*C*H_2_), 14.42 (*C*H_3_).

^31^P{^1^H} NMR (162 MHz, CD_3_OD): 28.70.

**IR** ν_max_ (KBr) 3200–2700 (vs, br), 2958 (vs), 2927 (vs), 2856 (m), 1601 (m), 1521 (m), 1469 (m), 1347 (vs), 1230 (w), 1206 (w), 1180 (w, sh), 1109 (w), 1051 (w), 1010 (m), 857 (m).

**HR-MS** (ESI^+^): for C_25_H_49_N_4_O_5_P (M + H)^+^*m*/*z* calculated 515.33568, found 515.33523.

#### Didodecyl (2-(Bis(3-aminopropyl)amino)ethyl)phosphonate Trihydrochloride (**10m**)

The title compound was prepared according to general methods **A1**, **B1**, **D**, and **E** from mono methyl vinylphosphonate (0.97 g, 7.1 mmol) in 7% overall yield (0.35 g, 0.51 mmol) as a white solid.

^1^H NMR (401 MHz, CD_3_OD): 4.13 (dtd, *J* = 8.3, 6.6, 1.7 Hz, 4H, OC*H*_2_), 3.50–3.42 (m, 2H, PCH_2_C*H*_2_), 3.38 (dd, *J* = 10.3, 6.3 Hz, 4H, C*H*_2_(CH_2_)_2_NH_2_), 3.10 (t, *J* = 7.5 Hz, 4H, C*H*_2_NH_2_), 2.58–2.46 (m, 2H, PC*H*_2_), 2.26–2.13 (m, 4H, C*H*_2_CH_2_NH_2_), 1.71 (dt, *J* = 8.3, 6.4 Hz, 4H, OCH_2_C*H*_2_), 1.48–1.22 (m, 36H, (C*H*_2_)_9_CH_3_), 0.96–0.84 (m, 6H, C*H*_3_).

^13^C NMR (101 MHz, CD_3_OD): 68.24 (d, *J* = 6.7 Hz, O*C*H_2_), 51.03 (*C*H_2_(CH_2_)_2_NH_2_), 48.75 (PCH_2_*C*H_2_), 37.86 (*C*H_2_NH_2_), 33.09, 31.61 (d, *J* = 5.8 Hz, OCH_2_*C*H_2_), 30.82, 30.79, 30.76, 30.73, 30.50, 30.32, 26.65, 23.75, 23.26 (*C*H_2_CH_2_NH_2_), 21.21 (d, *J* = 140.8 Hz, P*C*H_2_), 14.46 (*C*H_3_).

^31^P{^1^H} NMR (162 MHz, CD_3_OD): 28.70.

**IR** ν_max_ (KBr) 3100–2500 (m, vbr), 2958 (s), 2922 (s), 2853 (s), 2740 (m, br), 2673 (m), 2615 (m, br), 2547 (m), 2030 (w, vbr), 1606 (m), 1543 (w), 1510 (w, sh), 1485 (s, sh), 1468 (s), 1400 (m, sh), 1379 (m), 1227 (s), 1071 (s, sh), 1030 (s, sh), 998 (vs), 721 (m).

**HR-MS** (ESI^+^): for C_32_H_71_N_3_O_3_P (M + H)^+^ calculated 576.52276, found 576.52234.

#### Dodecyl 2-(Indol-3-yl)ethyl (2-(Bis(3-aminopropyl)amino)ethyl)phosphonate Tetrahydrochloride (**10n**)

The title compound was prepared according to general methods **A1**, **B2**, **D**, and **E** from mono methyl vinylphosphonate (0.45 g, 3.32 mmol) in 22% overall yield (0.48 g, 0.73 mmol) as a white amorphous solid.

^1^H NMR (401 MHz, CD_3_OD): 7.60 (dt, *J* = 7.9, 1.1 Hz, 1H, C^4^*H*), 7.40 (dt, *J* = 8.1, 0.9 Hz, 1H, C^7^*H*), 7.19 (s, 1H, C^2^*H*), 7.13 (ddd, *J* = 8.1, 7.0, 1.2 Hz, 1H, C^6^*H*), 7.05 (ddd, *J* = 8.0, 7.0, 1.1 Hz, 1H, C^5^*H*), 4.38 (qd, *J* = 6.6, 2.2 Hz, 2H, OC*H*_2_CH_2_Ar), 4.01–3.89 (m, 2H, OC*H*_2_C_11_H_23_), 3.20–3.08 (m, 8H, OCH_2_C*H*_2_Ar, NC*H*_2_), 3.03 (t, *J* = 7.5 Hz, 4H, C*H*_2_NH_2_), 2.38–2.26 (m, 2H, PC*H*_2_), 2.13–2.03 (m, 4H, C*H*_2_CH_2_NH_2_), 1.59 (p, *J* = 6.7 Hz, 2H, OCH_2_C*H*_2_C_10_H_21_), 1.28 (s, 18H, (C*H*_2_)_9_CH_3_), 0.92–0.86 (m, 3H, CH_3_).

^13^C NMR (101 MHz, CD_3_OD): 138.02 (*C*^7a^), 128.79 (*C*^3a^), 124.44 (*C*^2^), 122.58 (*C*^6^), 119.96 (*C*^5^), 119.42 (*C*^7^), 112.57 (*C*^**4**^), 111.53 (*C*^3^), 68.49 (d, *J* = 7.0 Hz, O*C*H_2_CH_2_Ar), 68.03 (d, *J* = 6.8 Hz, O*C*H_2_C_11_H_23_), 50.89 (*C*H_2_(CH_2_)_2_NH_2_), 48.53 (PCH_2_*C*H_2_), 37.81 (*C*H_2_NH_2_), 33.07, 31.48 (d, *J* = 5.9 Hz, OCH_2_*C*H_2_C_10_H_21_), 30.80, 30.78, 30.76, 30.71, 30.65, 30.47, 30.25, 27.57 (d, *J* = 6.5 Hz, OCH_2_*C*H_2_Ar), 26.50, 23.73, 23.16 (*C*H_2_CH_2_NH_2_), 21.03 (d, *J* = 140.9 Hz, P*C*H_2_), 14.44 (*C*H_3_).

^31^P{^1^H} NMR (162 MHz, CD_3_OD): 28.43.

**IR** ν_max_ (KBr) 3426 (m, br), 3256 (m, vbr), 3100–2500 (m-w, vbr), 2955 (s), 2924 (vs), 2854 (s), 2745 (w, br, sh), 2634 (w, br), 2558 (w, br), 2035 (vw, vbr), 1618 (w), 1610 (w, sh), 1491 (w, sh), 1467 (m), 1458 (m), 1433 (w, sh), 1379 (vw), 1352 (vw), 1302 (vw), 1252 (w), 1226 (m), 1180 (w), 1148 (vw), 1083 (w, sh), 1058 (m), 1007 (m), 964 (w, sh), 936 (w, sh), 876 (vw), 741 (w), 721 (w), 430 (w).

**HR-MS** (ESI^+^): for C_30_H_56_N_4_O_3_P (M + H)^+^ calculated 551.40845, found 551.40785.

#### Tetradecyl 2-(Indol-3-yl)ethyl (2-(Bis(3-aminopropyl)amino)ethyl)phosphonate Tetrahydrochloride (**10o**)

The title compound was prepared according to general methods **A1**, **B2**, **D**, and **E** from mono methyl vinylphosphonate (0.64 g, 4.7 mmol) in 21% overall yield (0.87 g, 0.99 mmol) as a white amorphous solid.

^1^H NMR (401 MHz, CD_3_OD): 7.60 (dt, *J* = 7.9, 1.1 Hz, 1H, C^7^*H*), 7.40 (dt, *J* = 8.1, 1.0 Hz, 1H, C^4^*H*), 7.19 (s, 1H, C^2^*H*), 7.13 (ddd, *J* = 8.2, 7.0, 1.2 Hz, 1H, C^6^*H*), 7.05 (ddd, *J* = 8.0, 7.0, 1.1 Hz, 1H, C^5^*H*), 4.46–4.32 (m, 2H, OC*H*_2_CH_2_Ar), 4.03–3.87 (m, 2H, OC*H*_2_C_11_H_23_), 3.24–3.09 (m, 8H, NC*H*_2_, OCH_2_C*H*_2_Ar), 3.04 (t, *J* = 7.5 Hz, 4H, C*H*_2_NH_2_), 2.39–2.24 (m, 2H, PC*H*_2_), 2.14–2.02 (m, 4H, C*H*_2_CH_2_NH_2_), 1.64–1.53 (m, 2H, OCH_2_C*H*_2_C_10_H_21_), 1.28 (s, 22H, (C*H*_2_)_9_CH_3_), 0.95–0.85 (m, 3H, C*H*_3_).

^13^C NMR (101 MHz, CD_3_OD): 138.01 (*C*^7a^), 128.79 (*C*^3a^), 124.44 (*C*^2^), 122.57 (*C*^6^), 119.96 (*C*^5^), 119.42 (*C*^7^), 112.57 (*C*^4^), 111.53 (*C*^3^), 68.48 (d, *J* = 7.0 Hz, *C*H_2_CH_2_Ar), 68.02 (d, *J* = 6.9 Hz, O*C*H_2_C_11_H_23_), 50.88 (*C*H_2_(CH_2_)_2_NH_2_), 48.55 (PCH_2_*C*H_2_), 37.80 (*C*H_2_NH_2_), 33.06, 31.48 (d, *J* = 6.0 Hz, OCH_2_*C*H_2_C_10_H_21_), 30.77, 30.74, 30.70, 30.64, 30.47, 30.25, 27.56 (d, *J* = 6.5 Hz, OCH_2_*C*H_2_Ar), 26.49, 23.73, 23.14 (*C*H_2_CH_2_NH_2_), 21.04 (d, *J* = 140.9 Hz, P*C*H_2_), 14.44 (*C*H_3_).

^31^P{^1^H} NMR (162 MHz, CD_3_OD): 28.41.

**IR** ν_max_ (KBr) 3422 (s, br), 3246 (m, br), 3000–2500 (m, vbr), 2956 (s), 2924 (vs), 2854 (s), 2745 (m, br, sh), 2460 (m, br), 2559 (m, br), 2040 (w, vbr), 1617 (m), 1610 (m, sh), 1548 (w, sh), 1520 (w, sh), 1490 (m, sh), 1467 (m), 1458 (s), 1435 (m, sh), 1379 (w), 1352 (w), 1300 (vw, sh), 1252 (m, sh), 1227 (m), 1181 (m), 1149 (w), 1084 (m, sh), 1058 (m), 1005 (s), 962 (m, sh), 934 (w, sh), 877 (vw), 741 (m), 721 (m), 427 (m).

**HR-MS** (ESI^+^): for C_32_H_60_N_4_O_3_P (M + H)^+^ calculated 579.44030, found 579.44012.

#### Bis(cyclopentylmethyl) Propane-1,3-diyl Bis((2-(bis(3-aminopropyl)amino)ethyl)phosphonate) Hexahydrochloride (**14**)

The title compound was prepared according to general methods **A1**, **B2**, **C**, **D**, and **E** from mono methyl vinylphosphonate (1.3 g, 10.63 mmol) in 19% overall yield (1.82 g, 2.02 mmol) as an amorphous white solid.

Mixture of diastereoisomers (A, B).

^1^H NMR (500.2 MHz, CD_3_OD): 4.40–4.20 (m, 8H, OC*H*_2_CH_2_-A,B), 4.09–3.99 (m, 8H, OC*H*_2_-cyclopent-A,B), 3.55–3.47 (m, 8H, PCH_2_C*H*_2_-A,B), 3.47–3.40 (m, 16H, C*H*_2_(CH_2_)_2_NH_2_-A,B), 3.13 (bt, 16H, *J* = 7.3 Hz, C*H*_2_NH_2_-A,B), 2.71–2.60 (m, 8H, PC*H*_2_-A,B), 2.34–2.19 (m, 20H, *H*-1-cyclopent, C*H*_2_CH_2_NH_2_-A,B), 2.16–2.10 (m, 4H, OCH_2_C*H*_2_-A,B), 1.85–1.77 (m, 8H, *H*-2a,5a-cyclopent-A,B), 1.70–1.56 (m, 16H, *H*-3,4-cyclopent-A,B), 1.38–1.30 (m, 8H, *H*-2b,5b-cyclopent-A,B).

^13^C NMR (125.8 MHz, CD_3_OD): 71.93 (d, *J* = 7.1 Hz, O-*C*H_2_-cyclopent-A), 71.87 (d, *J* = 7.5 Hz, O-*C*H_2_-cyclopent-B), 64.24 (d, *J* = 6.4 Hz, O*C*H_2_CH_2_-B), 63.89 (d, *J* = 6.3 Hz, O*C*H_2_CH_2_-A), 51.03 (*C*H_2_(CH_2_)_2_NH_2_-A,B), 48.96 (PCH_2_*C*H_2_-A,B, overlapped by CD_3_OD), 41.33 (d, *J* = 6.1 Hz, *C*H-1-cyclopent-A,B), 38.00 (*C*H_2_NH_2_-B), 37.91 (*C*H_2_NH_2_-A), 32.24 (t, *J* = 6.5 Hz, OCH_2_*C*H_2_-B), 31.87 (t, *J* = 6.9 Hz, OCH_2_*C*H_2_-A), 29.98 (d, *J* = 2.4 Hz, *C*H_2_–2,5-cyclopent-A,B), 26.34 (*C*H_2_–3,4-cyclopent-A,B), 23.18 (*C*H_2_CH_2_NH_2_-A,B), 21.54 (d, *J* = 140.1 Hz, P*C*H_2_-A), 21.44 (d, *J* = 139.8 Hz, P*C*H_2_-B).

^31^P{^1^H} NMR (202.5 MHz, CD_3_OD): 26.92 (B), 26.94 (A).

**IR** ν_max_ (KBr) 2955 (vs), 2950 (s, vbr), 2906 (s, sh), 2870 (s), 2744 (m, br, sh), 2635 (m, vbr), 2559 (m, vbr), 2017 (w, vbr), 1602 (w, br), 1505 (w, br, sh), 1520 (w, br, sh), 1471 (m), 1405 (w, br), 1253 (m, br, sh), 1224 (m), 1076 (w, sh), 1023 (s), 1005 (s).

**HR-MS** (ESI^+^): for C_31_H_69_N_6_O_6_P_2_ (M + H)^+^*m*/*z* calculated 683.47483, found 683.47472.

#### Bis((*Z*)-hept-3-en-1-yl) Propane-1,3-diyl Bis((2-(bis(3-aminopropyl)amino)ethyl)phosphonate) Hexahydrochloride (**15**)

The title compound was prepared according to general methods **A1**, **B2**, **C**, **D**, and **E** from mono methyl vinylphosphonate (0.50 g, 3.69 mmol) in 16% overall yield (0.54 g, 0.58 mmol) as a white solid.

Mixture of diastereoisomers.

^1^H NMR (400 MHz, CD_3_OD): 5.62–5.52 (m, 2H, CH_3_(CH_2_)_2_C*H*), 5.49–5.39 (m, 2H, C*H***(**CH_2_)_2_O), 4.40–4.19 (m, 8H, OC*H*_2_CH_2_C*H*_2_O), 4.19–4.10 (m, 8H, CHCH_2_C*H*_2_O), 3.57–3.38 (m, 12H, C*H*_2_(CH_2_)_2_NH_2_, PCH_2_C*H*_2_), 3.13 (t, 8H, *J* = 7.4 Hz, C*H*_2_NH_2_), 2.73–2.58 (m, 4H, PC*H*_2_), 2.54–2.45 (m, 4H, CHC*H*_2_CH_2_O), 2.24 (p, 8H, *J* = 7.6 Hz, C*H*_2_CH_2_NH_2_), 2.17–2.03 (m, 6H, OCH_2_C*H*_2_CH_2_O, CH_3_CH_2_C*H*_2_), 1.41 (h, 4H, *J* = 7.4 Hz, CH_3_C*H*_2_), 0.93 (t, 6H, *J* = 7.4 Hz, C*H*_3_).

^13^C NMR (101 MHz, CD_3_OD): 134.14 (CH_3_(CH_2_)_2_*C*H), 125.19 (*C*H(CH_2_)_2_O), 67.78 (d, *J* = 6.9 Hz), 67.72 (d, *J* = 6.8 Hz, CHCH_2_*C*H_2_O), 64.23 (d, *J* = 6.4 Hz), 63.86 (d, *J* = 6.5 Hz, O*C*H_2_CH_2_*C*H_2_O), 51.03 (*C*H_2_(CH_2_)_2_NH_2_), 48.91, 48.88 (PCH_2_*C*H_2_), 37.93 (*C*H_2_NH_2_), 32.21 (t, *J* = 6.7 Hz), 31.86 (t, *J* = 7.0 Hz, OCH_2_*C*H_2_CH_2_O), 30.44 (CH_3_CH_2_*C*H_2_), 29.64 (d, *J* = 6.1 Hz, CH*C*H_2_CH_2_O), 23.76 (CH_3_*C*H_2_), 23.21 (*C*H_2_CH_2_NH_2_), 21.56 (d, *J* = 140.1, 11.40 Hz, P*C*H_2_), 21.45 (d, *J* = 139.7 Hz, P*C*H_2_), 14.13 (*C*H_3_).

^31^P{^1^H} NMR (162 MHz, CD_3_OD): 28.64.

**IR** ν_max_ 3010 (vs), 2959 (vs), 2925 (s), 2897 (s), 2869 (s), 2043 (w), 1700 (w), 1609 (m), 1467 (s), 1380 (m), 1230 (s), 720 (w).

**HR-MS** (ESI^+^): for C_33_H_74_O_6_N_6_P_2_ (M + 2H)^2+^*m/*2*z* calculated 356.25671, found 356.25688.

#### Bis((*Z*)-hept-4-en-1-yl) Propane-1,3-diyl Bis((2-(bis(3-aminopropyl)amino)ethyl)phosphonate) Hexahydrochloride (**16**)

The title compound was prepared according to general methods **A1**, **B2**, **C**, **D**, and **E** from mono methyl vinylphosphonate (0.16 g, 1.18 mmol) in 14% overall yield (0.16 g, 0.17 mmol) as a white solid.

^1^H NMR (401 MHz, CD_3_OD): 5.50–5.41 (m, 2H, CH_3_CH_2_C*H*), 5.40–5.31 (m, 2H, C*H*(CH_2_)_3_O), 4.42–4.10 (m, 8H, C*H*_2_O), 3.52–3.47 (m, 4H, PCH_2_C*H*_2_), 3.46–3.38 (m, 8H, C*H*_2_(CH_2_)_2_NH_2_), 3.12 (t, 8H, *J* = 7.5 Hz, C*H*_2_NH_2_), 2.73–2.60 (m, 4H, PC*H*_2_), 2.29–2.02 (m, 18H, OCH_2_C*H*_2_CH_2_O, CH_3_C*H*_2_(CH)_2_C*H*_2_, C*H*_2_CH_2_NH_2_), 1.85–1.73 (m, 4H, CHCH_2_C*H*_2_), 0.98 (t, 6H, *J* = 7.5 Hz, C*H*_3_).

^13^C NMR (101 MHz, CD_3_OD): 133.89 (CH_3_CH_2_*C*H), 128.45 (*C*H(CH_2_)_3_O), 67.85 (d, *J* = 6.6 Hz, CH(CH_2_)_2_*C*H_2_O), 63.86 (d, *J* = 6.2 Hz, O*C*H_2_CH_2_*C*H_2_O), 51.03 (*C*H_2_(CH_2_)_2_NH_2_), 48.88 (PCH_2_*C*H_2_), 37.93 (*C*H_2_NH_2_), 31.91 (t, *J* = 6.9 Hz, OCH_2_*C*H_2_CH_2_O), 31.68 (d, *J* = 6.0 Hz, CHCH_2_*C*H_2_CH_2_O), 24.02 (CH*C*H_2_(CH_2_)_2_O), 23.27 (*C*H_2_CH_2_NH_2_), 21.50 (CH_3_*C*H_2_), 21.53 (d, *J* = 140.0 Hz, P*C*H_2_CH_2_N), 14.72 (*C*H_3_).

^31^P{^1^H} NMR (162 MHz, CD_3_OD): 28.97.

**IR** ν_max_ 3013 (s, sh), 2962 (vs), 2935 (s), 2875 (s, sh), 2010 (w), 1653 (w), 1405 (m), 1380 (w, sh), 1222 (m), 1016 (s), 987 (s), 841 (w), 756 (w).

**HR-MS** (ESI^+^): for C_33_H_74_O_6_N_6_P_2_ (M + 2H)^2+^*m/*2*z* calculated 356.25671, found 356.25693.

#### Dioctyl Propane-1,3-diyl Bis((2-(bis(3-aminopropyl)amino)ethyl)phosphonate) Hexahydrochloride (**17**)

The title compound was prepared according to general methods **A1**, **B2**, **C**, **D**, and **E** from mono methyl vinylphosphonate (0.28 g, 2.07 mmol) in 14% overall yield (0.28 g, 0.29 mmol) as a white solid.

^1^H NMR (401 MHz, CD_3_OD): 4.42–4.08 (m, 8H, C*H*_2_O), 3.57–3.36 (m, 12H, C*H*_2_N), 3.12 (t, 8H, *J* = 7.4 Hz, C*H*_2_NH_2_), 2.72–2.56 (m, 4H, PC*H*_2_), 2.23 (p, 8H, *J* = 8.2 Hz, C*H*_2_CH_2_NH_2_), 2.13 (p, 2H, *J* = 5.7 Hz OCH_2_C*H*_2_CH_2_O), 1.79–1.68 (m, 4H, CH_3_(CH_2_)_5_C*H*_2_), 1.47–1.25 (m, 20H, CH_3_(C*H*_2_)_5_), 0.95–0.87 (m, 6H, C*H*_3_).

^13^C NMR (101 MHz, CD_3_OD): 68.43 (d, *J* = 6.6 Hz, O*C*H_2_CH_2_*C*H_2_O), 63.83 (d, *J* = 6.3 Hz, CH_3_(CH_2_)_6_*C*H_2_O), 51.02 (*C*H_2_(CH_2_)_2_NH_2_), 48.92 (PCH_2_*C*H_2_), 37.91 (*C*H_2_NH_2_), 33.00 (CH_3_CH_2_*C*H_2_), 31.88 (t, *J* = 7.3 Hz, OCH_2_*C*H_2_CH_2_O), 31.64 (d, *J* = 6.0 Hz, CH_3_(CH_2_)_5_*C*H_2_), 30.38, 30.33, 26.65, 23.73 (CH_3_*C*H_2_), 23.23 (*C*H_2_CH_2_NH_2_), 21.51 (d, *J* = 140.1 Hz, P*C*H_2_), 14.44 (*C*H_3_).

^31^P{^1^H} NMR (162 MHz, CD_3_OD): 28.86.

**IR** ν_max_ 3000 (vs, vbr), 2957 (vs), 2927 (vs), 2856 (s), 2740 (s, sh), 2636 (s, br), 2559 (s, br), 2019 (w, vbr), 1602 (m), 1516 (m, sh), 1468 (s), 1379 (m), 1255 (s, sh), 1227 (s), 1073 (s, sh), 1014 (s, br), 987 (s).

**HR-MS** (ESI^+^): for C_35_H_82_O_6_N_6_P_2_ (M + 2H)^2+^*m*/2*z* calculated 372.28801, found 372.28777.

#### Bis((*Z*)-oct-3-en-1-yl) Propane-1,3-diyl Bis((2-(bis(3-aminopropyl)amino)ethyl)phosphonate) Hexahydrochloride (**18**)

The title compound was prepared according to general methods **A1**, **B2**, **C**, **D**, and **E** from mono methyl vinylphosphonate (0.40 g, 2.95 mmol) in 16% overall yield (0.44 g, 0.46 mmol) as a white solid.

Mixture of diastereoisomers.

^1^H NMR (400 MHz, CD_3_OD): 5.64–5.50 (m, 2H, CH_3_(CH_2_)_3_C*H*), 5.50–5.36 (m, 2H, C*H*(CH_2_)_2_O), 4.43–4.19 (m, 4H, OC*H*_2_CH_2_C*H*_2_O), 4.19–4.06 (m, 4H, CHCH_2_C*H*_2_O), 3.59–3.46 (m, 4H, PCH_2_C*H*_2_), 3.46–3.37 (m, 8H, C*H*_2_(CH_2_)_2_NH), 3.12 (t, 8H, *J* = 7.5 Hz, C*H*_2_NH_2_), 2.75–2.56 (m, 4H, PC*H*_2_), 2.49 (q, 4H, *J* = 6.9 Hz, CHC*H*_2_CH_2_O), 2.273 (p, 8H, *J* = 6.9 Hz, C*H*_2_CH_2_NH_2_), 2.17–2.03 (m, 6H, CH_3_(CH_2_)_2_C*H*_2_, OCH_2_C*H*_2_CH_2_O), 1.44–1.29 (m, 8H, CH_3_(C*H*_2_)_2_), 1.00–0.88 (m, 6H, C*H*_3_).

^13^C NMR (101 MHz, CD_3_OD): 134.37 (CH_3_(CH_2_)_3_*C*H), 124.95 (*C*H(CH_2_)_2_O), 67.79 (d, *J* = 6.5 Hz), 67.73 (d, *J* = 6.3 Hz CHCH_2_*C*H_2_O), 64.23 (d, *J* = 6.6 Hz), 63.86 (d, *J* = 6.2 Hz, O*C*H_2_CH_2_*C*H_2_O), 51.05 (*C*H_2_(CH_2_)_2_NH_2_), 48.89, 48.85 (PCH_2_*C*H_2_N), 37.93 (*C*H_2_NH_2_), 32.91 (CH_3_CH_2_*C*H_2_), 32.23 (t, *J* = 5.8 Hz), 31.87 (t, *J* = 7.2 Hz, OCH_2_*C*H_2_CH_2_O), 29.63 (d, *J* = 6.3 Hz, CH*C*H_2_CH_2_O), 28.12 (CH_3_(CH_2_)_2_*C*H_2_), 23.38 (CH_3_*C*H_2_), 23.24 (*C*H_2_CH_2_NH), 21.55 (d, *J* = 140.4 Hz), 21.44 (d, *J* = 140.3 Hz, P*C*H_2_), 14.35 (*C*H_3_).

^31^P{^1^H} NMR (162 MHz, CD_3_OD): 28.66.

**IR** ν_max_ (KBr) 3428 (m, br), 3015 (vs, sh), 2958 (vs), 2929 (vs), 2873 (s), 2559 (s, br), 1607 (m), 1467 (m), 1380 (w, sh), 1229 (m), 1011 (s), 1070 (s).

**HR-MS** (ESI^+^): for C_35_H_78_O_6_N_6_P_2_ (M + 2H)^2+^*m*/2*z* calculated 370.27236, found 370.27233.

#### Bis((*Z*)-non-3-en-1-yl) Propane-1,3-diyl Bis((2-(Bis(3-aminopropyl)amino)ethyl)phosphonate) Hexahydrochloride (**19**)

The title compound was prepared according to general methods **A1**, **B2**, **C**, **D**, and **E** from mono methyl vinylphosphonate (0.69 g, 5.10 mmol) in 20% overall yield (1.03 g, 1.04 mmol) as a white solid.

Mixture of diastereoisomers.

^1^H NMR (401 MHz, CD_3_OD): 5.64–5.51 (m, 2H, CH_2_(CH_2_)_4_C*H*), 5.48–5.36 (m, 2H, C*H*(CH_2_)_2_O), 4.41–4.19 (m, 4H, OC*H*_2_CH_2_C*H*_2_O), 4.19–4.10 (m, 4H, CHCH_2_C*H*_2_O), 3.56–3.46 (m, 4H, PCH_2_C*H*_2_), 3.43 (t, 8H, *J* = 8.2 Hz, C*H*_2_(CH_2_)_2_NH_2_), 3.13 (t, 8H, *J* = 7.5 Hz, C*H*_2_NH_2_), 2.74–2.58 (m, 4H, PC*H*_2_), 2.49 (q, 4H, *J* = 6.8 Hz, CHC*H*_2_CH_2_O), 2.27–2.19 (p, 8H, *J* = 7.9 Hz, C*H*_2_CH_2_NH_2_), 2.17–2.04 (m, 6H, OCH_2_C*H*_2_CH_2_O, CH_2_(CH_2_)_3_C*H*_2_), 1.47–1.23 (m, 12H, CH_2_(C*H*_2_)_3_), 0.97–0.86 (m, 6H, C*H*_3_).

^13^C NMR (101 MHz, CD_3_OD): 134.40 (CH_3_(CH_2_)_4_*C*H), 124.93 (*C*H(CH_2_)_2_O), 67.78, (d, *J* = 6.4 Hz), 67.72 (d, *J* = 6.5 Hz, CHCH_2_*C*H_2_O), 64.21 (d, *J* = 6.3 Hz), 63.83 (d, *J* = 6.5 Hz, O*C*H_2_CH_2_*C*H_2_O), 51.01 (*C*H_2_(CH_2_)_2_NH_2_), 48.90, 48.84 (PCH_2_*C*H_2_), 37.91 (*C*H_2_NH_2_), 32.66 (CH_3_CH_2_*C*H_2_), 32.21 (t, *J* = 6.9 Hz), 31.85 (t, *J* = 6.7 Hz, OCH_2_*C*H_2_CH_2_O), 30.40 (CH_3_(CH_2_)_2_*C*H_2_), 29.64 (d, *J* = 6.0 Hz, CH*C*H_2_CH_2_O), 28.38 (CH_3_(CH_2_)_3_*C*H_2_), 23.64 (CH_3_*C*H_2_), 23.21 (*C*H_2_CH_2_NH_2_), 21.54 (d, *J* = 139.9 Hz, P*C*H_2_), 21.42 (d, *J* = 139.7 Hz, P*C*H_2_), 14.44 (*C*H_3_).

^31^P{^1^H} NMR (162 MHz, CD_3_OD): 28.88, 28.87.

**IR** ν_max_ 3200–2800 (s), 2958 (vs), 2928 (w), 2873 (s), 2858 (s), 2045 (w), 1650 (sh), 1612 (m), 1516 (sh), 1406 (m), 1380 (m), 1231 (s), 725 (sh).

**HR-MS** (ESI^+^): for C_37_H_82_O_6_N_6_P_2_ (M + 2H)^2+^*m*/2*z* calculated 384.28801, found 384.28792.

#### Didecyl Propane-1,3-diyl Bis((2-(bis(3-aminopropyl)amino)ethyl)phosphonate) Hexahydrochloride (**20**)

The title compound was prepared according to general methods **A1**, **B2**, **C**, **D**, and **E** from mono methyl vinylphosphonate (1.2 g, 9.89 mmol) in 9% overall yield (0.91 g, 0.89 mmol) as an amorphous white solid.

Mixture of diastereoisomers.

^1^H NMR (500.2 MHz, CD_3_OD): 4.43–4.19 (m, 4H, OC*H*_2_CH_2_C*H*_2_O), 4.19–4.11 (m, 4H, OC*H*_2_(CH_2_)_8_CH_3_), 3.51 (dq, 4H, *J* = 11.5, 6.3, 5.3 Hz, PCH_2_C*H*_2_), 3.46–3.40 (m, 8H, C*H*_2_(CH_2_)_2_NH_2_), 3.13 (t, 8H, *J* = 7.4 Hz, C*H*_2_NH_2_), 2.71–2.57 (m, 4H, PC*H*_2_), 2.23 (p, 8H, *J* = 8.2, 7.6 Hz, C*H*_2_CH_2_NH_2_), 2.13 (tt, 2H, *J* = 8.7, 4.4 Hz, OCH_2_C*H*_2_CH_2_O), 1.77–1.69 (m, 4H, C*H*_2_(CH_2_)_7_CH_3_), 1.47–1.38 (m, 4H, C*H*_2_(CH_2_)_6_CH_3_), 1.38–1.24 (m, 24H, (C*H*_2_)_6_CH_3_), 0.93–0.87 (m, 6H, C*H*_3_).

^13^C NMR (125.8 MHz, CD_3_OD): 68.43 (d, *J* = 7.1 Hz), 68.37 (d, *J* = 6.9 Hz, O*C*H_2_(CH_2_)_8_CH_3_), 64.23 (d, *J* = 6.4 Hz), 63.85 (d, *J* = 6.4 Hz, O*C*H_2_CH_2_*C*H_2_O), 51.04 (*C*H_2_(CH_2_)_2_NH_2_), 48.95, 48.90 (PCH_2_*C*H_2_), 37.92 (*C*H_2_NH_2_), 33.06 (*C*H_2_CH_2_CH_3_), 32.24 (t, *J* = 7.0 Hz), 31.90 (t, *J* = 7.0 Hz, OCH_2_*C*H_2_CH_2_O), 31.64 (d, *J* = 6.1 Hz, *C*H_2_(CH_2_)_7_CH_3_), 30.71, 30.46, 30.36, 26.64, 23.73 (*C*H_2_)_6_CH_3_), 23.22 (*C*H_2_CH_2_NH_2_), 21.53 (d, *J* = 140.1 Hz), 21.42 (d, *J* = 139.9 Hz, P*C*H_2_), 14.43 (*C*H_3_).

^31^P{^1^H} NMR (202.5 MHz, CD_3_OD) δ 26.81.

**IR** ν_max_ (KBr) 2957 (vs), 2926 (vs), 2855 (vs), 2745 (s, sh), 2639 (s), 2559 (m), 2040 (w, br), 1609 (m), 1517 (m, br, sh), 1468 (s), 1402 (m), 1379 (m), 1257 (s, br, sh), 1230 (s), 1062 (s, sh), 1015 (vs), 988 (vs).

**HR-MS** (ESI^+^): for C_39_H_89_N_6_O_6_P_2_ (M + H)^+^*m*/*z* calculated 799.63133, found 799.63190.

#### Bis((*Z*)-dec-4-en-1-yl) Propane-1,3-diyl Bis((2-(bis(3-aminopropyl)amino)ethyl)phosphonate) Hexahydrochloride (**21**)

The title compound was prepared according to general methods **A1**, **B2**, **C**, **D**, and **E** from mono methyl vinylphosphonate (0.59 g, 4.35 mmol) in 12% overall yield (0.51 g, 0.50 mmol) as an amorphous white solid.

Mixture of diastereoisomers.

^1^H NMR (401 MHz, CD_3_OD): 5.51–5.33 (m, 4H, C*H*C*H*(CH_2_)_3_O), 4.42–4.12 (m, 8H, C*H*_2_O), 3.56–3.37 (m, 12H, C*H*_2_(CH_2_)_2_NH_2_, PCH_2_C*H*_2_), 3.13 (t, 8H, *J* = 7.4 Hz, C*H*_2_NH_2_), 2.74–2.56 (m, 4H, PC*H*_2_), 2.30–2.10 (m, 14H, CHC*H*_2_(CH_2_)_2_O, OCH_2_C*H*_2_CH_2_O, C*H*_2_CH_2_NH_2_), 2.06 (q, 4H, *J* = 6.8 Hz, CH_3_(CH_2_)_3_C*H*_2_), 1.78 (p, 4H, *J* = 6.8 Hz, CHCH_2_C*H*_2_CH_2_O), 1.43–1.26 (m, 12H, CH_3_(C*H*_2_)_3_), 0.97–0.84 (m, 6H, C*H*_3_).

^13^C NMR (101 MHz, CD_3_OD): 132.22 (CH_3_(CH_2_)_4_*C*H), 129.07 (*C*H(CH_2_)_3_O), 67.91 (d, *J* = 6.4 Hz), 67.85 (d, *J* = 6.3 Hz, CH(CH_2_)_2_*C*H_2_O), 64.21 (d, *J* = 6.2 Hz), 63.81 (d, *J* = 6.5 Hz, O*C*H_2_CH_2_*C*H_2_O), 48.24 (*C*H_2_(CH_2_)_2_NH_2_), 48.91, 48.89 (PCH_2_*C*H_2_), 37.92 (*C*H_2_NH_2_), 32.66 (CH_3_CH_2_*C*H_2_), 32.26 (t, *J* = 5.1 Hz), 31.91 (t, *J* = 6.6 Hz, OCH_2_*C*H_2_CH_2_O), 31.72 (d, *J* = 6.1 Hz, CHCH_2_*C*H_2_CH_2_O), 30.49 (CH_3_(CH_2_)_2_*C*H_2_), 28.19 (CH_3_(CH_2_)_3_*C*H_2_), 24.17 (CHC*H*_2_(CH_2_)_2_O), 23.65 (CH_3_*C*H_2_), 22.23 (*C*H_2_CH_2_NH_2_), 21.54 (d, *J* = 140.4 Hz), 21.42 (d, *J* = 139.5 Hz, P*C*H_2_), 14.45 (*C*H_3_).

^31^P{^1^H} NMR (162 MHz, CD_3_OD): 28.96, 28.93.

**IR** ν_max_ 3200–2400 (s), 3013 (s, sh), 2956 (s), 2926 (vs), 2873 (s), 2011 (w), 1657 (vw), 1600 (w), 1467 (m), 1379 (w), 1224 (m), 843 (m), 754 (m).

**HR-MS** (ESI^+^): for C_39_H_85_O_6_N_6_P_2_ (M + H)^+^*m*/*z* calculated 795.60003, found 795.60019.

#### Bis((adamantan-1-yl)methyl) Propane-1,3-diyl Bis((2-(bis(3-aminopropyl)amino)ethyl)phosphonate) Hexahydrochloride (**22**)

The title compound was prepared according to general methods **A1**, **B2**, **C**, **D**, and **E** from mono methyl vinylphosphonate (0.58 g, 4.79 mmol) in 24% overall yield (1.16 g, 1.12 mmol) as an amorphous white solid.

Mixture of diastereoisomers.

^1^H NMR (500.2 MHz, CD_3_OD): 4.41–4.21 (m, 8H, OC*H*_2_CH_2_-A,B), 3.76–3.67 (m, 8H, O-CH_2_-adamantane-A,B), 3.55–3.47 (m, 8H, PCH_2_C*H*_2_-A,B), 3.47–3.40 (m, 16H, C*H*_2_(CH_2_)_2_NH_2_-A,B), 3.13 (bt, 16H, *J* = 7.3 Hz, C*H*_2_NH_2_-A,B), 2.72–2.58 (m, 8H, PC*H*_2_-A,B), 2.28–2.20 (m, 16H, C*H*_2_CH_2_NH_2_-A,B), 2.18–2.11 (m, 4H, OCH_2_C*H*_2_-A,B), 2.03–1.98 (m, 12H, H-3,5,7-adamantane-A,B), 1.74–1.68, 1.83–1.76 (2 × m, 2 × 12H, H-4,6,10-adamantane-A,B), 1.65–1.58 (m, 24H, H-2,8,9-adamantane-A,B).

^13^C NMR (125.8 MHz, CD_3_OD): 77.55 (d, *J* = 7.2 Hz, O-*C*H_2_-adamantane-A), 77.47 (d, *J* = 7.3 Hz, O-*C*H_2_-adamantane-B), 64.34 (d, *J* = 6.4 Hz, O*C*H_2_CH_2_-B), 63.96 (d, *J* = 6.4 Hz, O*C*H_2_CH_2_-A), 51.05 (*C*H_2_(CH_2_)_2_NH_2_-A,B), 48.98 (PCH_2_*C*H_2_-A,B, overlapped by CD_3_OD), 39.87 (*C*H-2,8,9-adamantane-A,B), 37.92 (*C*H-4,6,10-adamantane-A,B, *C*H_2_NH_2_-A,B), 32.33 (t, *J* = 6.6 Hz, OCH_2_*C*H_2_-B), 31.95 (t, *J* = 7.0 Hz, OCH_2_*C*H_2_-A), 29.45 (*C*H-3,5,7-adamantane-A,B), 29.45 (*C*H-3,5,7-adamantane-A,B), 23.24 (*C*H_2_CH_2_NH_2_-A,B), 21.39 (d, *J* = 140.1 Hz, P*C*H_2_-A), 21.29 (d, *J* = 139.4 Hz, P*C*H_2_-B).

^31^P{^1^H} NMR (202.5 MHz, CD_3_OD): 27.32.

**IR** ν_max_ (KBr) 2975 (s, br, sh), 2932 (s, sh), 2903 (vs), 2847 (s), 2675, 2656, 2636, 2035 (w, vbr), 1617 (m, br), 1517 (w, br, sh), 1464 (m), 1455 (m), 1404 (w, br), 1365 (vw), 1344 (vw), 1262 (m, sh), 1240 (m), 1226 (m), 1106 (vw), 1061 (m), 1015 (s), 999 (s), 987 (s), 975 (m, sh), 945 (w, sh), 923 (w), 809 (w), 437 (w).

**HR-MS** (ESI^+^): for C_41_H_81_N_6_O_6_P_2_ (M + H)^+^*m*/*z* calculated 815.56873, found 815.56785.

#### Bis(2-(adamantan-1-yl)ethyl) Propane-1,3-diyl Bis((2-(bis(3-aminopropyl)amino)ethyl)phosphonate) Hexahydrochloride (**23**)

The title compound was prepared according to general methods **A1**, **B2**, **C**, **D**, and **E** from mono methyl vinylphosphonate (0.32 g, 2.65 mmol) in 17% overall yield (0.48 g, 0.45 mmol) as an amorphous white solid.

^1^H NMR (401 MHz, CD_3_OD): 4.41–4.25 (m, 4H, OC*H*_2_CH_2_C*H*_2_O), 4.28–4.16 (m, 4H, OC*H*_2_CH_2_C_quat_), 3.54–3.46 (m, 4H, PCH_2_C*H*_2_), 3.46–3.39 (m, 8H, C*H*_2_(CH_2_)_2_NH_2_), 3.12 (t, *J* = 7.3 Hz, 8H, C*H*_2_NH_2_), 2.63 (dq, 4H, *J* = 16.2, 5.6 Hz, PC*H*_2_), 2.22 (q, *J* = 8.0 Hz, 8H, C*H*_2_CH_2_NH_2_), 2.17–2.10 (m, 2H, OCH_2_C*H*_2_CH_2_O), 2.01–1.91 (m, 6H, C*H*), 1.81–1.65 (m, 12H, C_quat_C*H*_2_CH), 1.60 (d, 12H, *J* = 2.8 Hz, *c*-(CHC*H*_2_)_3_), 1.58–1.51 (m, 4H, OCH_2_C*H*_2_C_quat_).

^13^C NMR (101 MHz, CD_3_OD): 64.83 (d, *J* = 6.4 Hz), 64.78 (d, *J* = 6.6 Hz, O*C*H_2_CH_2_C_quat_), 64.27 (d, *J* = 6.4 Hz), 63.93 (d, *J* = 6.4 Hz, O*C*H_2_CH_2_*C*H_2_O), 51.03 (*C*H_2_(CH_2_)_2_NH_2_), 48.91 (PCH_2_*C*H_2_), 45.56 (d, *J* = 5.5 Hz, OCH_2_*C*H_2_C_quat_), 43.60 (*c*-(CH*C*H_2_)_3_), 38.01 (C_quat_*C*H_2_CH), 37.92 (*C*H_2_NH_2_), 32.91 (*C*_quat_), 32.32 (d, *J* = 6.1 Hz), 31.98 (d, *J* = 5.4 Hz, OCH_2_*C*H_2_CH_2_O), 30.03 (*C*H), 23.22 (*C*H_2_CH_2_NH_2_), 21.57 (d, *J* = 139.9 Hz), 21.47 (d, *J* = 139.4 Hz, P*C*H_2_).

^31^P{^1^H} NMR (162 MHz, CD_3_OD): 28.93.

**IR** ν_max_ (KBr) 2950 (s, br), 2926 (vs, sh), 1906 (vs), 2847 (s), 1997 (vw, vbr), 2674, 1656, 2635, 2558, 1598 (w, br), 1509 (w, sh), 1470 (m), 1452 (m), 1404 (w, br), 1365 (vw, sh), 1345 (vw), 1252 (m, sh), 1228 (m), 1106 (w), 1072 (m), 1045 (m), 1013 (s), 998 (s), 987 (s), 972 (m, sh), 816 (w).

**HR-MS** (ESI^+^): for C_43_H_85_N_6_O_6_P_2_ (M + H)^+^*m*/*z* calculated 843.60003, found 843.59928.

#### Butane-1,4-diyl Diheptyl Bis((2-(bis(3-aminopropyl)amino)ethyl)phosphonate) Hexahydrochloride (**24**)

The title compound was prepared according to general methods **A1**, **B2**, **C**, **D**, and **E** from mono methyl vinylphosphonate (1.25 g, 10.2 mmol) in 3% overall yield (0.29 g, 0.31 mmol) as an amorphous white solid.

^1^H NMR (401 MHz, CD_3_OD): 4.34–4.07 (m, 8H, OC*H*_2_), 3.48 (dt, 4H, *J* = 10.4, 7.6 Hz, PCH_2_C*H*_2_), 3.44–3.39 (m, 8H, C*H*_2_(CH_2_)_2_NH_2_), 3.12 (t, *J* = 7.5 Hz, 8H, C*H*_2_NH_2_), 2.59 (ddt, 4H, *J* = 20.8, 7.4, 3.5 Hz, PC*H*_2_), 2.30–2.14 (m, 8H, C*H*_2_CH_2_NH_2_), 1.94–1.81 (m, 4H, OCH_2_(C*H*_2_)_2_CH_2_O), 1.72 (dt, 4H, *J* = 8.1, 6.4 Hz, C*H*_2_(CH_2_)_4_CH_3_), 1.48–1.24 (m, 16H, O(CH_2_)_2_(C*H*_2_)_4_CH_3_), 0.97–0.85 (m, 6H, C*H*_3_).

^13^C NMR (101 MHz, CD_3_OD): 68.30 (d, *J* = 6.7 Hz), 68.29 (d, *J* = 6.7 Hz, O*C*H_2_(CH_2_)_5_CH_3_), 67.65 (d, *J* = 6.6 Hz), 67.61 (d, *J* = 6.5 Hz, O*C*H_2_(CH_2_)_2_*C*H_2_O), 51.04 (*C*H_2_(CH_2_)_2_NH_2_), 48.87 (PCH_2_*C*H_2_), 37.90 (*C*H_2_NH_2_), 32.92 (*C*H_2_(CH_2_)_3_CH_3_), 31.62 (d, *J* = 6.0 Hz, *C*H_2_(CH_2_)_4_CH_3_), 30.00, 27.86 (d, *J* = 6.3 Hz), 27.82 (d, *J* = 6.2 Hz, OCH_2_(*C*H_2_)_2_CH_2_O), 26.58, 23.68, 23.25 (*C*H_2_CH_2_NH_2_), 21.42 (d, *J* = 140.3 Hz), 21.38 (d, *J* = 139.7 Hz, P*C*H_2_), 14.43 (*C*H_3_).

^31^P{^1^H} NMR (162 MHz, CD_3_OD): 28.73.

**IR** ν_max_ (KBr) 2957 (vs), 2930 (vs), 2859 (s), 2740 (s, sh), 2627 (m), 2556 (m), 2002 (w, vbr), 1599 (m), 1469 (s), 1395 (m, br), 1254 (s, sh), 1229 (s), 1065 (s), 1007 (s), 984 (s, sh), 728 (w).

**HR-MS** (ESI^+^): for C_34_H_79_N_6_O_6_P_2_ (M + H)^+^*m*/*z* calculated 729.55308, found 729.55334.

#### Butane-1,4-diyl Dioctyl Bis((2-(bis(3-aminopropyl)amino)ethyl)phosphonate) Hexahydrochloride (**25**)

The title compound was prepared according to general methods **A1**, **B2**, **C**, **D**, and **E** from mono methyl vinylphosphonate (0.61 g, 4.48 mmol) in 14% overall yield (0.62 g, 0.64 mmol) as a white solid.

Mixture of diastereoisomers.

^1^H NMR (401 MHz, CD_3_OD): 4.31–4.08 (m, 8H, C*H*_2_O), 3.54–3.33 (m, 12H, C*H*_2_N), 3.12 (t, 8H, *J* = 7.5 Hz C*H*_2_NH_2_), 2.68–2.51 (m, 4H, PC*H*_2_), 2.29–2.13 (m, 8H, C*H*_2_CH_2_NH_2_), 1.93–1.82 (m, 4H, OCH_2_(C*H*_2_)_2_CH_2_O), 1.77–1.68 (m, 4H, CH_3_(CH_2_)_5_C*H*_2_), 1.48–1.24 (m, 20H, CH_3_(C*H*_2_)_5_), 0.96–0.84 (m, 6H, C*H*_3_).

^13^C NMR (101 MHz, CD_3_OD): 68.29 (d, *J* = 6.6 Hz), 68.28 (d, *J* = 6.6 Hz, CH_3_(CH_2_)_6_*C*H_2_O), 67.64 (d, *J* = 6.6 Hz), 67.59 (d, *J* = 6.5 Hz, O*C*H_2_(CH_2_)_2_*C*H_2_O), 51.04 (*C*H_2_(CH_2_)_2_NH_2_), 48.86 (PCH_2_*C*H_2_), 37.92 (*C*H_2_NH_2_), 32.99 (CH_3_CH_2_*C*H_2_), 31.62 (d, *J* = 5.9 Hz, CH_3_(CH_2_)_5_*C*H_2_CH_2_O), 30.37, 30.30, 26.63, 27.86 (d, *J* = 6.2 Hz), 27.82 (d, *J* = 6.2 Hz, OCH_2_(*C*H_2_)_2_CH_2_O), 23.72 (CH_3_*C*H_2_), 23.26 (*C*H_2_CH_2_NH_2_), 21.44 (d, *J* = 139.7 Hz), 21.40 (d, *J* = 139.8 Hz, P*C*H_2_), 14.45 (*C*H_3_).

^31^P{^1^H} NMR (162 MHz, CD_3_OD): 28.76, 28.60.

**IR** ν_max_ (KBr) 3504 (w, br), 3000 (w, br), 2959 (s), 2933 (s), 2857 (s), 2745 (s, br), 2643 (s, br), 2561 (s, br), 2051 (w, br), 1609 (m), 1521 (w, sh), 1510 (m), 1229 (s, br), 1071 (s), 997 (s, br).

**HR-MS** (ESI^+^): for C_36_H_83_N_6_O_6_P_2_ (M + H)^+^*m*/*z* calculated 757.58438, found 757.58384.

#### Bis((*Z*)-hept-3-en-1-yl) Pentane-1,5-diyl Bis((2-(bis(3-aminopropyl)amino)ethyl)phosphonate) Hexahydrochloride (**26**)

The title compound was prepared according to general methods **A1**, **B2**, **C**, **D**, and **E** from mono methyl vinylphosphonate (0.53 g, 3.93 mmol) in 17% overall yield (0.65 g, 0.68 mmol) as a white solid.

^1^H NMR (401 MHz, CD_3_OD): 5.61–5.52 (m, 2H, CH_3_(CH_2_)_2_C*H*), 5.48–5.39 (m, 2H, C*H*(CH_2_)_2_O), 4.26–4.08 (m, 8H, C*H*_2_O), 3.53–3.36 (m, 12H, C*H*_2_N), 3.12 (t, 8H, *J* = 7.5 Hz, C*H*_2_NH_2_), 2.66–2.45 (m, 8H, CHC*H*_2_CH_2_O, PC*H*_2_), 2.28–2.17 (m, 8H, C*H*_2_CH_2_NH_2_), 2.07 (qd, *J* = 7.4, 1.5 Hz, 4H, CH_3_CH_2_C*H*_2_), 1.85–1.73 (m, 4H, CH_2_C*H*_2_CH_2_O), 1.61–1.51 (m, 2H, C*H*_2_(CH_2_)_2_O), 1.41 (h, 4H, *J* = 7.4 Hz, CH_3_C*H*_2_), 0.93 (t, 6H, *J* = 7.4 Hz, C*H*_3_).

^13^C NMR (101 MHz, CD_3_OD): 134.14 (CH_3_(CH_2_)_2_*C*H), 125.19 (*C*H(CH_2_)_2_O), 67.95 (d, *J* = 6.9 Hz), 67.61 (d, *J* = 6.6 Hz, *C*H_2_O), 51.07 (*C*H_2_(CH_2_)_2_NH_2_), 37.95 (*C*H_2_NH_2_), 30.99 (d, *J* = 6.0 Hz), 30.96 (d, *J* = 6.4 Hz, CH_2_*C*H_2_CH_2_O), 30.45 (CH_3_CH_2_*C*H_2_), 30.97 (d, *J* = 6.2 Hz, CH*C*H_2_CH_2_O), 23.78 (CH_3_*C*H_2_), 23.35 (*C*H_2_CH_2_NH_2_), 22.82 (*C*H_2_(CH_2_)_2_O), 21.41 (d, *J* = 139.2 Hz (P*C*H_2_), 14.15 (*C*H_3_).

^31^P{^1^H} NMR (162 MHz, CD_3_OD): 28.65.

**IR** ν_max_ 3013 (s), 3100–2500 (vs, br), 2958 (vs), 2933 (s), 2873 (s), 2017 (w), 1655 (w), 1601 (m), 1466 (m), 1379 (w), 1230 (s), 720 (w).

**HR-MS** (ESI^+^): for C_35_H_78_O_6_N_6_P_2_ (M + 2H)^2+^*m*/2*z* calculated 370.27236, found 370.27250.

#### Bis((*Z*)-hept-4-en-1-yl) Pentane-1,5-diyl Bis((2-(bis(3-aminopropyl)amino)ethyl)phosphonate) Hexahydrochloride (**27**)

The title compound was prepared according to general methods **A1**, **B2**, **C**, **D**, and **E** from mono methyl vinylphosphonate (0.29 g, 2.13 mmol) in 19% overall yield (0.38 g, 0.40 mmol) as a white solid.

Mixture of diastereoisomers.

^1^H NMR (401 MHz, CD_3_OD): 5.49–5.41 (m, 2H, CH_3_CH_2_C*H*), 5.39–5.31 (m, 2H, C*H*(CH_2_)_3_O), 4.25–4.09 (m, 8H, C*H*_2_O), 3.54–3.37 (m, 12H, C*H*_2_(CH_2_)_2_NH_2_, PCH_2_C*H*_2_), 3.12 (t, 8H, *J* = 7.5 Hz, C*H*_2_NH_2_), 2.67–2.51 (m, 4H, PC*H*_2_), 2.29–2.13 (m, 12H, C*H*_2_CH_2_NH_2_, CHC*H*_2_(CH_2_)_2_O), 2.13–2.03 (m, 4H, CH_3_C*H*_2_), 1.85–1.73 (m, 8H, C*H*_2_CH_2_O), 1.62–1.51 (m, 2H, O(CH_2_)_2_C*H*_2_(CH_2_)_2_O), 0.98 (t, 6H, *J* = 7.5 Hz, C*H*_3_).

^13^C NMR (101 MHz, CD_3_OD): 133.87 (CH_3_CH_2_*C*H), 128.47 (*C*H(CH_2_)_3_O), 68.00 (d, *J* = 6.7 Hz, O*C*H_2_(CH_2_)_3_*C*H_2_O), 67.71 (d, *J* = 6.7 Hz, CH(CH_2_)_2_*C*H_2_O), 51.05 (*C*H_2_(CH_2_)_2_NH_2_), 48.87 (PCH_2_*C*H_2_), 37.89 (*C*H_2_NH_2_), 31.67 (d, *J* = 6.1 Hz, CHCH_2_*C*H_2_CH_2_O), 31.00 (d, *J* = 6.2 Hz), 30.97 (d, *J* = 5.9 Hz, OCH_2_*C*H_2_CH_2_*C*H_2_CH_2_O), 24.03 (CH*C*H_2_(CH_2_)_2_O), 23.26 (*C*H_2_CH_2_NH_2_), 22.81 (d, *J* = 2.9 Hz, O(CH_2_)_2_*C*H_2_(CH_2_)_2_O), 21.50 (CH_3_*C*H_2_), 21.36 (d, *J* = 140.2 Hz), 21.35 (d, *J* = 140.0 Hz, P*C*H_2_), 14.72 (*C*H_3_).

^31^P{^1^H} NMR (162 MHz, CD_3_OD): 28.72, 28.68.

**IR** ν_max_ 3010 (s, sh), 2961 (vs), 2931 (s), 2875 (s), 2040 (w), 1654 (w), 1404 (m), 1380 (w, sh), 1280 (w, sh), 1228 (m), 1067 (s), 997 (s), 840 (w), 756 (w).

**HR-MS** (ESI^+^): for C_35_H_78_O_6_N_6_P_2_ (M + 2H)^2+^*m*/2*z* calculated 370.27236, found 370.27254.

#### Bis((*Z*)-oct-3-en-1-yl) Pentane-1,5-diyl Bis((2-(bis(3-aminopropyl)amino)ethyl)phosphonate) Hexahydrochloride (**28**)

The title compound was prepared according to general methods **A1**, **B2**, **C**, **D**, and **E** from mono methyl vinylphosphonate (0.43 g, 3.16 mmol) in 17% overall yield (0.54 g, 0.54 mmol) as a white solid.

^1^H NMR (400 MHz, CD_3_OD) δ 5.60–5.50 (m, 2H, CH_3_(CH_2_)_3_C*H*), 5.46–5.37 (m, 2H, C*H*(CH_2_)_2_O), 4.25–4.06 (m, 8H, C*H*_2_O), 3.52–3.37 (m, 12H, C*H*_2_N), 3.14 (t, *J* = 7.3 Hz, 8H, C*H*_2_NH_2_), 2.68–2.54 (m, 4H, PC*H*_2_), 2.48 (q, *J* = 6.9 Hz, 4H, CHC*H*_2_CH_2_O), 2.30–2.18 (m, 8H, C*H*_2_CH_2_NH_2_), 2.12–2.04 (m, 4H, CH_3_(CH_2_)_2_C*H*_2_), 1.83–1.72 (m, 4H, CH_2_C*H*_2_CH_2_O), 1.63–1.47 (m, 2H, C*H*_2_(CH_2_)_2_O), 1.41–1.26 (m, 8H, CH_3_(C*H*_2_)_2_), 0.97–0.85 (m, 6H, C*H*_3_).

^13^C NMR (101 MHz, CD_3_OD) δ 134.20 (CH_3_(CH_2_)_3_*C*H), 125.03 (*C*H(CH_2_)_2_O), 67.91 (d, *J* = 6.7 Hz, (CH_2_)_2_*C*H_2_O), 67.57 (d, *J* = 6.7 Hz, CHCH_2_*C*H_2_O), 50.97 (*C*H_2_(CH_2_)_2_NH_2_), 48.88 (PCH_2_*C*H_2_), 37.93 (*C*H_2_NH_2_), 32.85 (CH_3_CH_2_*C*H_2_), 30.90 (d, *J* = 6.0 Hz), 30.88 (d, *J* = 6.1 Hz, CH_2_*C*H_2_CH_2_O), 29.58 (d, *J* = 6.0 Hz, CH*C*H_2_CH_2_O), 28.03 (CH_3_(CH_2_)_2_*C*H_2_), 23.31 (CH_3_*C*H_2_), 23.13 (*C*H_2_CH_2_NH_2_), 22.77 (*C*H_2_(CH_2_)_2_O), 22.75, 21.44 (d, *J* = 139.8 Hz), 21.43 (d, *J* = 139.7 Hz, P*C*H_2_), 14.35 (*C*H_3_).

^31^P{^1^H} NMR (162 MHz, CD_3_OD): 28.45.

**IR** ν_max_ (KBr) 3435 (s, br), 3015 (m, sh), 2957 (m), 2927 (m), 2872 (m), 2857 (m), 2500–2300 (m, br), 1626 (w), 1467 (w), 1379 (w), 1275 (w, sh), 1226 (w), 1004 (m).

**HR-MS** (ESI^+^): for C_37_H_82_O_6_N_6_P_2_ (M + 2H)^2+^*m*/2*z* calculated 384.28801, found 384.28824.

#### Bis((*Z*)-non-3-en-1-yl) Pentane-1,5-diyl Bis((2-(bis(3-aminopropyl)amino)ethyl)phosphonate) Hexahydrochloride (**29**)

The title compound was prepared according to general methods **A1**, **B2**, **C**, **D**, and **E** from mono methyl vinylphosphonate (0.78 g, 5.71 mmol) in 14% overall yield (0.81 g, 0.80 mmol) as a white solid.

Mixture of diastereoisomers.

^1^H NMR (401 MHz, CD_3_OD): 5.63–5.51 (m, 2H, CH_3_(CH_2_)_4_C*H*), 5.47–5.37 (m, 2H, C*H*(CH_2_)_2_O), 4.27–4.07 (m, 8H, C*H*_2_O), 3.53–3.34 (m, 12H, C*H*_2_N), 3.11 (t, 8H, *J* = 7.5 Hz, C*H*_2_NH_2_), 2.67–2.43 (m, 4H, CHC*H*_2_CH_2_O), 2.30–2.15 (m, 8H, C*H*_2_CH_2_NH_2_), 2.15–2.04 (m, 4H, CH_3_(CH_2_)_3_C*H*_2_), 1.85–1.71 (m, 4H, CH_2_C*H*_2_CH_2_O), 1.62–1.49 (m, 2H, C*H*_2_(CH_2_)_2_O), 1.45–1.25 (m, 12H, CH_3_(C*H*_2_)_3_), 0.97–0.86 (m, 6H, C*H*_3_).

^13^C NMR (101 MHz, CD_3_OD): 134.41 (CH_3_(CH_2_)_4_*C*H), 124.95 (*C*H(CH_2_)_2_O), 67.97 (d, *J* = 6.8 Hz, O*C*H_2_(CH_2_)_3_*C*H_2_O), 67.65 (d, *J* = 6.9 Hz, CHCH_2_*C*H_2_O), 49.65 (*C*H_2_(CH_2_)_2_NH_2_), 47.44 (PCH_2_*C*H_2_), 37.88 (*C*H_2_NH_2_), 32.66 (CH_3_CH_2_*C*H_2_), 30.98 (d, *J* = 6.1 Hz), 30.95 (d, *J* = 6.3 Hz, CH_2_*C*H_2_CH_2_O**)**, 30.40 (CH_3_(CH_2_)_2_*C*H_2_), 29.65 (d, *J* = 6.0 Hz, CH*C*H_2_CH_2_O), 28.37 (CH_3_(CH_2_)_3_*C*H_2_), 23.64 (CH_3_*C*H_2_), 23.25 (*C*H_2_CH_2_NH_2_), 22.82, 22.79 (*C*H_2_(CH_2_)_2_O), 21.37 (d, *J* = 140.1 Hz), 21.35 (d, *J* = 140.0 Hz, P*C*H_2_), 14.44 (*C*H_3_).

^31^P{^1^H} NMR (162 MHz, CD_3_OD): 28.67, 28.63.

**IR** ν_max_ 3200–2800 (s), 2959 (s), 2929 (s), 2873 (s), 2859 (s), 2048 (m), 2800–2400 (s), 1650 (m, sh), 1617 (m), 1468 (m), 1380 (m), 1229 (m), 725 (m).

**HR-MS** (ESI^+^): for C_39_H_85_O_6_N_6_P_2_ (M + H)^+^*m*/*z* calculated 795.60003, found 795.59979.

#### Bis((*Z*)-non-3-en-1-yl) Pentane-1,5-diyl Bis((2-(bis(3-guanidinopropyl)amino)ethyl)phosphonate) Hexahydrochloride (**30**)

The title compound was prepared according to general method **F** from compound **29** (0.30 g, 0.30 mmol) in 66% yield (0.23 g, 0.20 mmol) as a white solid.

Mixture of diastereoisomers.

^1^H NMR (401 MHz, CD_3_OD): 5.63–5.51 (m, 2H, CH_3_(CH_2_)_4_C*H*), 5.49–5.36 (m, 2H, C*H*(CH_2_)_2_O), 4.24–4.05 (m, 8H, C*H*_2_O), 3.52–3.40 (m, 4H, PCH_2_C*H*_2_), 3.41–3.32 (m, 16H, C*H*_2_CH_2_C*H*_2_NH), 2.63–2.44 (m, 8H, CHC*H*_2_CH_2_O, PC*H*_2_), 2.16–2.03 (m, 12H, CH_3_(CH_2_)_3_C*H*_2_, C*H*_2_CH_2_NH), 1.78 (p, 4H, *J* = 6.6 Hz, CH_2_C*H*_2_CH_2_O), 1.61–1.49 (m, 2H, C*H*_2_(CH_2_)_2_O), 1.44–1.26 (m, 12H, CH_3_(C*H*_2_)_3_), 0.97–0.85 (m, 6H, C*H*_3_).

^13^C NMR (101 MHz, CD_3_OD): 158.69 (*C*=NH), 134.42 (CH_3_(CH_2_)_4_*C*H), 125.03 (*C*H(CH_2_)_2_O), 67.94 (d, *J* = 6.7 Hz, CHCH_2_*C*H_2_O), 67.63 (d, *J* = 6.7 Hz, O*C*H_2_(CH_2_)_3_*C*H_2_O), 51.57 (*C*H_2_NH), 48.68 (PCH_2_*C*H_2_), 39.69 (*C*H_2_(CH_2_)_2_NH), 32.66 (CH_3_CH_2_*C*H_2_), 31.01 (d, *J* = 6.2 Hz), 30.99 (d, *J* = 5.8 Hz, CH_2_*C*H_2_CH_2_O), 30.41 (CH_3_(CH_2_)_2_*C*H_2_), 29.65 (d, *J* = 6.1 Hz, CH*C*H_2_CH_2_O), 28.38 (CH_3_(CH_2_)_3_*C*H_2_), 24.78 (*C*H_2_CH_2_NH_2_), 23.64, 22.81 (CH_3_(*C*H_2_)_2_), 22.78 (*C*H_2_(CH_2_)_2_O), 21.35 (d, *J* = 140.4 Hz, P*C*H_2_), 14.46 (*C*H_3_).

^31^P{^1^H} NMR (162 MHz, CD_3_OD): 28.84, 28.81.

**IR** ν_max_ 3400–2400 (s), 3009 (m), 2956 (m), 2930 (m), 2871 (m), 2859 (s), 1666 (s), 1646 (s), 1619 (s), 1467 (m), 1378 (w), 1229 (m), 1203 (m), 1009 (m), 721 (w).

**HR-MS** (ESI^+^): for C_43_H_93_O_6_N_14_P_2_ (M + H)^+^*m*/*z* calculated 963.68723, found 963.68764.

#### Bis((adamantan-1-yl)methyl) Pentane-1,5-diyl Bis((2-(bis(3-aminopropyl)amino)ethyl)phosphonate) Hexahydrochloride (**31**)

The title compound was prepared according to general methods **A1**, **B1**, **C**, **D**, and **E** from mono methyl vinylphosphonate (0.52 g, 4.28 mmol) in 29% overall yield (1.32 g, 1.24 mmol) as a white amorphous solid.

^1^H NMR (401 MHz, CD_3_OD): 4.26–4.10 (m, 4H, OC*H*_2_C_quat_), 3.70 (qd, 4H 9.6, 5.6 Hz, OC*H*_2_CH_2_), 3.51–3.36 (m, 12H, NC*H*_2_), 3.13 (t, 8H, *J* = 7.4 Hz, C*H*_2_NH_2_), 2.61 (dtd, 4H, *J* = 17.5, 9.0, 8.5, 4.8 Hz, PC*H*_2_), 2.30–2.16 (m, 8H, C*H*_2_CH_2_NH_2_), 2.01 (p, 6H, *J* = 3.1 Hz, C*H*), 1.85–1.67 (m, 16H, OCH_2_C*H*_2_, C_quat_C*H*_2_CH), 1.63–1.52 (m, 14H, *c*-(CHC*H*_2_)_3_, O(CH_2_)_2_C*H*_2_).

^13^C NMR (101 MHz, CD_3_OD): 77.41 (d, *J* = 7.0 Hz, O*C*H_2_C_quat_), 68.02 (d, *J* = 6.8 Hz, O*C*H_2_CH_2_), 51.03 (*C*H_2_(CH_2_)_2_NH_2_), 48.93 (PCH_2_*C*H_2_), 39.88 (*c*-(CH*C*H_2_)_3_), 37.94 (C_quat_*C*H_2_CH), 37.89 (*C*H_2_NH_2_), 34.96 (d, *J* = 6.7 Hz, *C*_quat_), 31.01 (d, *J* = 6.1 Hz), 30.97 (d, *J* = 6.1 Hz, OCH_2_*C*H_2_), 29.44 (*C*H), 23.22 (*C*H_2_CH_2_NH_2_), 22.87, 22.84 (O(CH_2_)_2_*C*H_2_), 21.23 (d, *J* = 140.1 Hz), 21.22 (d, *J* = 140.1 Hz, P*C*H_2_).

^31^P{^1^H} NMR (162 MHz, CD_3_OD) δ 28.77, 28.73.

**IR** ν_max_ (KBr) 3000 (vs, vbr), 2932 (vs, sh), 2903 (vs), 2847 (vs), 2800–2400 (s, vbr), 2676, 1656, 2043 (w, vbr), 1610 (m, br), 1515 (m, br, sh), 1474 (m, sh), 1464 (m), 1454 (m), 1397 (m, br), 1365 (w), 1344 (w), 1241 (m, sh), 1225 (m), 1155 (m), 1106 (m), 1061 (s), 1013 (s), 998 (s), 988 (s), 943 (m), 823 (m), 810 (w), 435 (w).

**HR-MS** (ESI^+^): for C_43_H_85_N_6_O_6_P_2_ (M + H)^+^*m*/*z* calculated 843.60003, found 843.59950.

#### Bis((adamantan-1-yl)methyl) Pentane-1,5-diyl Bis((2-(bis(3-guanidinopropyl)amino)ethyl)phosphonate) Hexahydrochloride (**32**)

The title compound was prepared according to general method **F** from compound **31** (0.30 g, 0.28 mmol) in 72% yield (0.25 g, 0.20 mmol) as a white solid.

Mixture of diastereoisomers.

^1^H NMR (500 MHz, CD_3_OD) δ 4.25–4.12 (m, 4H, OC*H*_2_CH_2_), 3.69 (qd, *J* = 9.6, 5.5 Hz, 4H, OC*H*_2_C), 3.50–3.41 (m, 4H, PCH_2_C*H*_2_), 3.40–3.32 (m, 16H, C*H*_2_CH_2_C*H*_2_NH), 2.64–2.50 (m, 4H, PC*H*_2_), 2.15–2.05 (m, 8H, C*H*_2_CH_2_NH), 2.01 (p, *J* = 3.1 Hz, 6H, CH_2_C*H*), 1.84–1.67 (m, 16H, OCH_2_C*H*_2_, CHC*H*_2_CH), 1.61 (d, *J* = 2.8 Hz, 12H), 1.59–1.51 (m, 2H, O(CH_2_)_2_C*H*_2_).

^13^C NMR (126 MHz, CD_3_OD) δ 158.68 (*C*=NH), 77.38 (d, *J* = 7.2 Hz, O*C*H_2_C), 68.01 (d, *J* = 6.7 Hz, O*C*H_2_CH_2_), 51.57 (*C*H_2_NH), 48.62 (PCH_2_*C*H_2_), 39.92 (C*C*H_2_CH), 39.68 (*C*H_2_(CH_2_)_2_NH), 37.95 (CH*C*H_2_CH), 34.98 (d, *J* = 6.8 Hz, OCH_2_*C*), 31.06 (d, *J* = 5.8 Hz), 31.03 (d, *J* = 5.9 Hz, OCH_2_*C*H_2_), 29.46 (*C*H), 24.84, 22.83, 22.81 (O(CH_2_)_2_*C*H_2_), 21.14 (d, *J* = 140.1 Hz, P*C*H_2_).

^31^P{^1^H} NMR (202 MHz, CD_3_OD) δ 26.30, 26.28.

**IR** ν_max_ 3400–2400 (w-s), 3322 (m), 3261 (m), 3143 (m), 2903 (m), 2847 (m), 1669 (s), 1646 (s), 1619 (s), 1453 (w), 1390 (w), 1366 (w), 1220 (m), 1014 (m), 721 (w).

**HR-MS** (ESI^+^): for C_47_H_94_O_6_N_14_P_2_ (M + 2H)^2+^*m*/2*z* calculated 506.34725, found 506.34692.

#### Hexane-1,6-diyl Dihexyl Bis((2-(bis(3-aminopropyl)amino)ethyl)phosphonate) Hexahydrochloride (**33**)

The title compound was prepared according to general methods **A1**, **B1**, **C**, **D**, and **E** from mono methyl vinylphosphonate (0.38 g, 3.15 mmol) in 13% overall yield (0.39 g, 0.41 mmol) as a white amorphous solid.

Mixture of diastereoisomers.

^1^H NMR (500.2 MHz, CD_3_OD): 4.21–4.09 (m, 8H, C*H*_2_O), 3.50–3.43 (m, 4H, PCH_2_C*H*_2_), 3.43–3.36 (m, 8H, C*H*_2_(CH_2_)_2_NH_2_), 3.43–3.36 (m, 8H, C*H*_2_NH_2_), 3.11 (t, 8H, *J* = 7.5 Hz, C*H*_2_CH_2_NH_2_), 2.61–2.50 (m, 4H, PC*H*_2_), 1.80–1.68 (m, 8H, C*H*_2_CH_2_O), 1.52–1.32 (m, 16H, CH_3_(C*H*_2_)_2_, C*H*_2_(CH_2_)_2_O), 0.95–0.90 (m, 6H, C*H*_3_).

^13^C NMR (125.8 MHz, CD_3_OD): 68.25 (d, *J* = 6.7 Hz), 68.07 (d, *J* = 6.6 Hz), 68.05 (d, *J* = 6.6 Hz, *C*H_2_O), 51.07 (*C*H_2_(CH_2_)_2_NH_2_), 48.87 (PCH_2_*C*H_2_), 37.90 (*C*H_2_NH_2_), 32.49 (CH_3_CH_2_*C*H_2_), 31.55 (d, *J* = 5.9 Hz), 31.40 (d, *J* = 5.7 Hz), 31.37 (d, *J* = 5.7 Hz, *C*H_2_CH_2_O), 26.29. 26.15, 26.14, 23.81 (CH_3_*C*H_2_), 23.26 (*C*H_2_CH_2_NH_2_), 21.35 (d, *J* = 140.4 Hz), 21.33 (d, *J* = 140.7 Hz, P*C*H_2_), 14.37 (*C*H_3_).

^31^P{^1^H} NMR (202.5 MHz, CD_3_OD): 26.62.

**IR** ν_max_ (CHCl_3_) 2957 (vs), 2933 (vs), 2860 (s), 2745 (s, br), 2640 (m, br), 2558 (m, br), 2051 (w, br), 1615 (m, br), 1513 (w, br), 1468 (m), 1397 (w, br), 1379 (w, sh), 1265 (m, sh), 1229 (m, br), 1060 (m), 1040 (m, sh), 998 (s).

**HR-MS** (ESI^+^): for C_34_H_80_O_6_P_2_ (M + 2H)^2+^*m*/2*z* calculated 365.28018, found 365.28030.

#### Bis(cyclopentylmethyl) Hexane-1,6-diyl Bis((2-(bis(3-aminopropyl)amino)ethyl)phosphonate) Hexahydrochloride (**34**)

The title compound was prepared according to general methods **A1**, **B1**, **C**, **D**, and **E** from mono methyl vinylphosphonate (1.6 g, 13.1 mmol) in 10% overall yield (1.26 g, 1.31 mmol) as a white amorphous solid.

Mixture of diastereoisomers.

^1^H NMR (500.0 MHz, CD_3_OD): 4.22–4.10 (m, 4H, OC*H*_2_CH_2_), 4.06–3.97 (m, 4H, OC*H*_2_-cyclopent), 3.50–3.45 (m, 4H, PCH_2_C*H*_2_), 3.45–3.39 (m, 8H, C*H*_2_(CH_2_)_2_NH_2_), 3.12 (t, 8H, *J* = 7.4 Hz, C*H*_2_NH_2_), 2.63–2.52 (m, 4H, PC*H*_2_), 2.28 (sep, 2H, *J* = 7.6 Hz, H-1-cyclopent), 2.27–2.18 (m, 8H, C*H*_2_CH_2_NH_2_), 1.84–1.72 (m, 8H, H-2a,5a-cyclopent, OCH_2_C*H*_2_), 1.70–1.56 (m, 8H, *H*-3,4-cyclopent), 1.53–1.44 (m, 4H, O(CH_2_)_2_C*H*_2_), 1.39–1.29 (m, 4H, H-2b,5b-cyclopent).

^13^C NMR (125.7 MHz, CD_3_OD): 71.77 (d, *J* = 6.9 Hz, OCH_2_-cyclopent), 68.08 (d, *J*_C,P_ = 6.7 Hz), 68.06 (d, *J*_C,P_ = 6.7 Hz, O*C*H_2_CH_2_), 51.06 (*C*H_2_(CH_2_)_2_NH_2_), 48.92 (PCH_2_*C*H_2_), 41.33 (d, *J* = 6.1 Hz, CH-1-cyclopent), 37.88 (*C*H_2_NH_2_), 31.37 (d, *J* = 5.9 Hz), 31.35 (d, *J* = 5.9 Hz, OCH_2_*C*H_2_), 29.97, 29.96 (CH_2_-2,5-cyclopent), 26.34 (CH_2_-3,4-cyclopent), 26.13, 26.12 (O(CH_2_)_2_*C*H_2_), 23.21 (*C*H_2_CH_2_NH_2_), 21.36 (d, *J* = 140.0 Hz), 21.35 (d, *J* = 140.2 Hz, P*C*H_2_).

^31^P{^1^H} NMR (202.4 MHz, CD_3_OD): 26.70.

**IR** ν_max_ (KBr) 3000 (s, vbr), 2953 (vs), 2912 (vs, sh), 2868 (s), 2740 (s, br, sh), 2636 (s, vbr), 2559 (m, vbr), 2022 (w, vbr), 1604 (m, br), 1505 (m, sh, br), 1470 (m), 1396 (w), 1255 (m, br, sh), 1223 (m), 1075 (m, sh), 1025 (s, sh), 1000 (s).

**HR-MS** (ESI^+^): for C_34_H_75_N_6_O_6_P_2_ (M + H)^+^*m*/*z* calculated 725.52178, found 725.52100.

#### Bis((*Z*)-hept-3-en-1-yl) Hexane-1,6-diyl Bis((2-(bis(3-aminopropyl)amino)ethyl)phosphonate) Hexahydrochloride (**35**)

The title compound was prepared according to general methods **A1**, **B2**, **C**, **D**, and **E** from mono methyl vinylphosphonate (0.61 g, 4.45 mmol) in 15% overall yield (0.58 g, 0.65 mmol) as a white amorphous solid.

Mixture of diastereoisomers.

^1^H NMR (401 MHz, CD_3_OD): 5.62–5.52 (m, 2H, CH_3_(CH_2_)_2_C*H*), 5.48–5.39 (m, 2H, C*H*(CH_2_)_2_O), 4.24–4.06 (m, 8H, C*H*_2_O), 3.52–3.35 (m, 12H, NC*H*_2_), 3.11 (t, 8H, *J* = 7.6 Hz, C*H*_2_NH_2_), 2.63–2.45 (m, 8H, CHC*H*_2_CH_2_O, PC*H*_2_), 2.27–2.15 (m, 8H, C*H*_2_CH_2_NH_2_), 2.12–2.03 (m, 4H, CH_3_CH_2_C*H*_2_), 1.81–1.70 (m, 4H, CH_2_C*H*_2_CH_2_O), 1.51–1.46 (m, 4H, C*H*_2_(CH_2_)_2_O), 1.46–1.36 (m, 4H, CH_3_C*H*_2_), 0.93 (t, 6H, *J* = 7.4 Hz, C*H*_3_).

^13^C NMR (101 MHz, CD_3_OD): 134.14 (CH_3_(CH_2_)_2_*C*H), 125.20 (*C*H(CH_2_)_2_O), 68.08 (d, *J* = 6.7 Hz), 68.06 (d, *J* = 6.7 Hz, (CH_2_)_2_*C*H_2_O), 67.63 (d, *J* = 7.2 Hz, CHCH_2_*C*H_2_O), 51.05 (*C*H_2_(CH_2_)_2_NH_2_), 48.83, 48.82 (PCH_2_*C*H_2_), 37.88 (*C*H_2_NH_2_), 31.39 (d, *J* = 6.1 Hz), 31.36 (d, *J* = 6.0 Hz, CH_2_*C*H_2_CH_2_O), 30.45, 29.66 (d, *J* = 6.2 Hz, CH*C*H_2_CH_2_O), 26.15 (*C*H_2_(CH_2_)_2_O), 23.78 (CH_3_*C*H_2_), 23.26 (*C*H_2_CH_2_NH_2_), 21.34 (d, *J* = 140.4 Hz), 21.32 (d, *J* = 140.4 Hz, P*C*H_2_),14.15 (*C*H_3_).

^31^P{^1^H} NMR (162 MHz, CD_3_OD): 28.67.

**IR** ν_max_ (KBr) 3100–2500 (vs, vbr), 3013 (vs), 2957 (vs), 2932 (vs), 2872 (s), 1653 (w), 1609 (m), 1465 (m), 1379 (w, sh), 1230 (m), 1001 (s), 720 (w).

**HR-MS** (ESI^+^): for C_36_H_79_O_6_N_6_P_2_ (M + H)^+^*m*/*z* calculated 753.55308, found 753.55327.

#### Bis((*Z*)-hept-3-en-1-yl) Hexane-1,6-diyl Bis((2-(bis(3-guanidinopropyl)amino)ethyl)phosphonate) Hexahydrochloride (**36**)

The title compound was prepared according to general method **F** from **35** (0.31 g, 0.34 mmol) in 65% yield (0.23 g, 0.22 mmol) as a white amorphous solid.

Mixture of diastereoisomers.

^1^H NMR (401 MHz, CD_3_OD) δ 5.61–5.52 (m, 2H, C*H*(CH_2_)_2_CH_3_), 5.48–5.39 (m, 2H, O(CH_2_)_2_C*H*), 4.18–4.08 (m, 8H, OC*H*_2_), 3.48–3.40 (m, 4H, PCH_2_C*H*_2_), 3.40–3.31 (m, 16H, C*H*_2_CH_2_C*H*_2_NH), 2.60–2.45 (m, 8H, PC*H*_2_, OCH_2_C*H*_2_CH), 2.16–2.04 (m, 12H, CH_3_CH_2_C*H*_2_, C*H*_2_CH_2_NH), 1.75 (p, *J* = 7.1, 6.4 Hz, 4H, OCH_2_C*H*_2_CH_2_), 1.52–1.45 (m, 4H, OCH_2_CH_2_C*H*_2_), 1.41 (q, *J* = 7.4 Hz, 4H, CH_3_C*H*_2_), 0.93 (t, *J* = 7.4 Hz, 6H, C*H*_3_).

^13^C NMR (101 MHz, CD_3_OD) δ 158.70 (*C*=NH), 134.18 (*C*H(CH_2_)_2_CH_3_), 125.27 (O(CH_2_)_2_*C*H), 68.08 (d, *J* = 6.5 Hz), 68.05 (d, *J* = 6.7 Hz, O*C*H_2_(CH_2_)_2_), 67.60 (d, *J* = 6.7 Hz, O*C*H_2_CH_2_CH), 51.59 (*C*H_2_NH), 48.64 (PCH_2_*C*H_2_), 39.69 (*C*H_2_(CH_2_)_2_NH, 31.44 (d, *J* = 5.9 Hz), 31.41 (d, *J* = 6.0 Hz, OCH_2_*C*H_2_CH_2_), 30.46 (*C*H_2_CH_2_CH_3_), 29.65 (d, *J* = 6.0 Hz, OCH_2_*C*H_2_CH), 26.18 (d, *J* = 1.9 Hz, O(CH_2_)_2_*C*H_2_), 24.80 (*C*H_2_CH_2_NH), 23.80 (*C*H_2_CH_3_), 21.31 (d, *J* = 140.4 Hz, P*C*H_2_), 14.16 (*C*H_3_).

^31^P{^1^H} NMR (162 MHz, CD_3_OD) δ 28.95, 28.92.

**IR** ν_max_ 3400–3100 (s, br), 3008 (m), 2959 (m), 2925 (m), 2870 (m), 1670 (s), 1646 (s), 1625 (s, sh), 1465 (m), 1376 (m), 1224 (m), 1003 (m), 720 (w).

**HR-MS** (ESI^+^): for C_40_H_88_O_6_N_14_P_2_ (M + 2H)^2+^*m*/2*z* calculated 461.32378, found 461.32363.

#### Bis((*Z*)-hept-4-en-1-yl) Hexane-1,6-diyl Bis((2-(bis(3-aminopropyl)amino)ethyl)phosphonate) Hexahydrochloride (**37**)

The title compound was prepared according to general methods **A1**, **B2**, **C**, **D**, and **E** from mono methyl vinylphosphonate (0.30 g, 2.23 mmol) in 21% overall yield (0.44 g, 0.46 mmol) as a white amorphous solid.

Mixture of diastereoisomers.

^1^H NMR (401 MHz, CD_3_OD): 5.49–5.40 (m, 2H, CH_3_CH_2_C*H*), 5.39–5.31 (m, 2H, C*H*(CH_2_)_3_O), 4.22–4.08 (m, 8H, C*H*_2_O), 3.52–3.35 (m, 12H, C*H*_2_NH_2_, PCH_2_C*H*_2_), 3.11 (t, 8H, *J* = 7.5 Hz, C*H*_2_(CH_2_)_2_NH), 2.64–2.47 (m, 4H, PC*H*_2_), 2.29–2.13 (m, 12H, C*H*_2_CH_2_NH_2_, CHC*H*_2_(CH_2_)_2_O), 2.13–2.01 (m, 4H, CH_3_C*H*_2_), 1.84–1.70 (m, 8H, C*H*_2_CH_2_O), 1.52–1.45 (m, 4H, O(CH_2_)_2_(C*H*_2_)_2_(CH_2_)_2_O), 0.98 (t, 6H, *J* = 7.5 Hz, C*H*_3_).

^13^C NMR (101 MHz, CD_3_OD): 133.87 (CH_3_CH_2_*C*H), 128.47 (*C*H(CH_2_)_3_O), 68.11 (d, *J* = 6.8 Hz), 68.09 (d, *J* = 6.9 Hz, O*C*H_2_(CH_2_)_4_*C*H_2_O), 67.68 (d, *J* = 6.7 Hz, CH(CH_2_)_2_*C*H_2_O), 51.06 (*C*H_2_(CH_2_)_2_NH_2_), 48.84 (PCH_2_*C*H_2_), 37.89 (*C*H_2_NH_2_), 31.67 (d, *J* = 6.0 Hz, CHCH_2_*C*H_2_CH_2_O), 31.43 (d, *J* = 6.2 Hz), 31.40 (d, *J* = 6.1 Hz, OCH_2_*C*H_2_(CH_2_)_2_*C*H_2_CH_2_O), 26.18, 26.16 (O(CH_2_)_2_(*C*H_2_)_2_(CH_2_)_2_O), 24.03 (CH*C*H_2_(CH_2_)_2_O), 23.29 (*C*H_2_CH_2_NH_2_), 21.50 (CH_3_*C*H_2_), 21.31 (d, *J* = 140.4 Hz), 21.30 (d, *J* = 140.1 Hz, P*C*H_2_), 14.73 (*C*H_3_).

^31^P{^1^H} NMR (162 MHz, CD_3_OD): 28.75.

**IR** ν_max_ 3010 (s, sh), 2961 (vs), 2935 (s), 2874 (s), 2034 (w), 1653 (w), 1404 (m), 1380 (w, sh), 1280 (sh), 1228 (m), 1066 (m), 1001 (s), 841 (w), 755 (w).

**HR-MS** (ESI^+^): for C_36_H_80_O_6_N_6_P_2_ (M + 2H)^2+^*m*/2*z* calculated 377.28018, found 377.28046.

#### Hexane-1,6-diyl Dioctyl Bis((2-(bis(3-aminopropyl)amino)ethyl)phosphonate) Hexahydrochloride (**38**)

The title compound was prepared according to general methods **A1**, **B1**, **C**, **D**, and **E** from mono methyl vinylphosphonate (1.7 g, 13.9 mmol) in 26% overall yield (1.44 g, 1.44 mmol) as a white amorphous solid.

Mixture of diastereoisomers.

^1^H NMR (500.0 MHz, CD_3_OD): 4.23–4.07 (m, 8H, C*H*_2_O), 3.51–3.42 (m, 4H, PCH_2_C*H*_2_), 3.45–3.36 (m, 8H, C*H*_2_(CH_2_)_2_NH_2_), 3.11 (t, 8H, *J* = 7.5 Hz, C*H*_2_NH_2_), 2.62–2.52 (m, 4H, PC*H*_2_), 2.27–2.17 (m, 8H, C*H*_2_CH_2_NH_2_), 1.79–1.67 (m, 8H, C*H*_2_CH_2_O), 1.52–1.26 (m, 24H, CH_3_(C*H*_2_)_4_, C*H*_2_(CH_2_)_2_O), 0.93–0.88 (m, 6H, C*H*_3_).

^13^C NMR (125.7 MHz, CD_3_OD): 68.22, 68.03, 68.01 (d, *J* = 6.6 Hz, *C*H_2_O), 50.98 (*C*H_2_(CH_2_)_2_NH_2_), 48.83 (PCH_2_*C*H_2_), 37.86 (*C*H_2_NH_2_), 32.99 (CH_3_CH_2_*C*H_2_), 31.60(d, *J* = 5.9 Hz), 31.39(d, *J* = 5.9 Hz), 31.36 (d, *J* = 6.0 Hz, *C*H_2_CH_2_O), 30.37, 30.28, 26.63, 26.16, 23.73 (CH_3_(*C*H_2_)_4_, *C*H_2_(CH_2_)_2_O), 23.21 (*C*H_2_CH_2_NH_2_), 21.31(d, *J* = 140.2 Hz), 21.28 (d, *J* = 140.3 Hz, P*C*H_2_), 14.47 (*C*H_3_).

^31^P{^1^H} NMR (202.4 MHz, CD_3_OD): 27.32.

**IR** ν_max_ (KBr) 2956 (vs), 2926 (vs), 2856 (s), 2748 (m, br, sh), 2644 (m, br), 2559 (m, br), 2055 (w, br), 1626 (s, br), 1513 (m, br), 1468 (s), 1398 (m, br), 1385 (vw), 1257 (m, sh), 1230 (s), 1071 (s, br, sh), 1001 (s, br), 724 (w).

**HR-MS** (ESI^+^): for C_38_H_88_N_6_O_6_P_2_ (M + 2H)^2+^*m*/2*z* calculated 393.31148, found 393.31156.

#### Hexane-1,6-diyl Dioctyl Bis((2-(bis(2-aminoethyl)amino)ethyl)phosphonate) Hexahydrochloride (**39**)

The title compound was prepared according to general methods **A1**, **B2**, **C**, **D**, and **E** from mono methyl vinylphosphonate in 8% overall yield (0.47 g, 0.52 mmol) as a white solid.

Mixture of diastereoisomers.

^1^H NMR (401 MHz, CD_3_OD): 4.20–4.07 (m, 8H, C*H*_2_O), 3.44–3.31 (m, 20H, C*H*_2_N, C*H*_2_NH_2_), 2.50–2.35 (m, 4H, PC*H*_2_), 1.81–1.67 (m, 8H, C*H*_2_CH_2_O), 1.53–1.46 (m, 4H, O(CH_2_)_2_(C*H*_2_)_2_(CH_2_)_2_O), 1.46–1.27 (m, 20H, CH_3_(C*H*_2_)_5_(CH_2_)_2_O), 0.95–0.87 (m, 6H, C*H*_3_(CH_2_)_7_O).

^13^C NMR (101 MHz, CD_3_OD): 67.97 (d, *J* = 6.9 Hz, CH_3_(CH_2_)_6_*C*H_2_O), 67.79 (d, *J* = 6.6 Hz), 67.77 (d, *J* = 6.9 Hz, O*C*H_2_(CH_2_)_4_*C*H_2_O), 51.46 (*C*H_2_CH_2_NH_2_), 48.50 (PCH_2_*C*H_2_), 36.85 (*C*H_2_NH_2_)_,_ 32.98 (CH_3_CH_2_*C*H_2_), 31.61 (d, *J* = 6.0 Hz, CH_3_(CH_2_)_5_*C*H_2_), 31.42 (d, *J* = 6.2 Hz), 31.40 (d, *J* = 6.0 Hz, OCH_2_*C*H_2_(CH_2_)_2_*C*H_2_CH_2_O), 30.36, 30.28, 26.66 (CH_3_(CH_2_)_2_(*C*H_2_**)**_3_), 26.19 (O(CH_2_)_2_(*C*H_2_)_2_(CH_2_)_2_O), 23.72 (CH_3_*C*H_2_), 22.23 (d, *J* = 139.3 Hz, P*C*H_2_), 14.45 *C*H_3_).

^31^P{^1^H} NMR (162 MHz, CD_3_OD): 32.26.

**IR** ν_max_ 3300–2500 (s), 2957 (s), 2928 (vs), 2856 (s), 2051 (w), 1609 (m), 1468 (s), 1379 (w), 1231 (m), 1002 (s), 848 (w), 770 (w), 724 (w).

**HR-MS** (ESI^+^): for C_34_H_80_O_6_N_6_P_2_ (M + 2H)^2+^*m*/2*z* calculated 365.28018, found 365.28057.

#### Bis(3-cyclohexylpropyl) Hexane-1,6-diyl Bis((2-(bis(3-aminopropyl)amino)ethyl)phosphonate) Hexahydrochloride (**40**)

The title compound was prepared according to general methods **A1**, **B1**, **C**, **D**, and **E** from mono methyl vinylphosphonate (0.86 g, 7.07 mmol) in 10% overall yield (1.02 g, 0.99 mmol) as a white amorphous solid.

Mixture of diastereoisomers.

^1^H NMR (500.2 MHz, CD_3_OD): 4.21–4.08 (m, 8H, OC*H*_2_), 3.50–3.44 (m, 4H, PCH_2_C*H*_2_), 3.44–3.37 (m, 8H, C*H*_2_(CH_2_)_2_NH_2_), 3.12 (t, 8H, *J* = 7.4 Hz, C*H*_2_NH_2_), 2.61–2.50 (m, 4H, PC*H*_2_), 2.28–2.16 (m, 8H, C*H*_2_CH_2_NH_2_), 1.80–1.63 (m, 18H, 2a,3a,4a,5a,6a-Cy*H*, OCH_2_C*H*_2_), 1.52–1.45 (m, 4H, O(CH_2_)_2_(C*H*_2_)_2_(CH_2_)_2_O), 1.33–1.13 (m, 12H, 1,3b,4b,5b-Cy*H*, O(CH_2_)C*H*_2_Cy), 0.98–0.88 (m, 4H, 2b,6b-Cy*H*).

^13^C NMR (125.8 MHz, CD_3_OD): 68.60 (d, *J* = 6.7 Hz, O*C*H_2_(CH_2_)Cy), 68.09, 68.06 (d, *J* = 6.6 Hz, O*C*H_2_(CH_2_)_4_*C*H_2_O), 51.06 (*C*H_2_(CH_2_)_2_NH_2_), 48.89 (PCH_2_*C*H_2_), 38.61 (1-*C*H_Cy_), 37.88 (*C*H_2_NH_2_), 34.46, 34.44 (2,6-(*C*H_2_)_Cy_), 34.26 (*C*H_2_Cy), 31.40, 31.37 (d, *J* = 5.9 Hz, OCH_2_*C*H_2_(CH_2_)_2_*C*H_2_CH_2_O), 29.03 (d, *J* = 5.9 Hz, *C*H_2_CH_2_Cy), 27.69 (4-(*C*H_2_)_Cy_), 27.42 (3,5-(*C*H_2_)_Cy_), 26.17, 26.15 (O(CH_2_)_2_(*C*H_2_)_2_(CH_2_)_2_O), 23.23 (*C*H_2_CH_2_NH_2_), 21.33, 21.31 (d, *J* = 140.0 Hz, P*C*H_2_).

^31^P{^1^H} NMR (202.5 MHz, CD_3_OD): 26.92.

**IR** ν_max_ (KBr) 3000 (v, vbr2970 (s, sh), 2923 (vs), 2851 (s), 2744 (m, br, sh), 2640 (m, vbr), 2559 (m, vbr), 2030 (w, vbr), 1608 (w, br), 1515 (w, sh), 1505 (w, sh), 1396 (w, br), 1346 (vw), 1260 (w, br, sh), 1227 (m), 1069 (m), 1053 (m, sh), 1002 (s).

**HR-MS** (ESI^+^): for C_40_H_87_N_6_O_6_P_2_ (M + H)^+^*m*/*z* calculated 809.61568, found 809.61578.

#### Hexane-1,6-diyl Bis((*Z*)-oct-3-en-1-yl) Bis((2-(bis(3-aminopropyl)amino)ethyl)phosphonate) Hexahydrochloride (**41**)

The title compound was prepared according to general methods **A1**, **B2**, **C**, **D**, and **E** from mono methyl vinylphosphonate (0.51 g, 3.75 mmol) in 18% overall yield (0.66 g, 0.66 mmol) as a white solid.

Mixture of diastereoisomers.

^1^H NMR (400 MHz, CD_3_OD): 5.62–5.52 (m, 2H, CH_3_(CH_2_)_3_C*H*), 5.48–5.35 (m, 2H, C*H*(CH_2_)_2_O), 4.23–4.05 (m, 8H, C*H*_2_O), 3.52–3.33 (m, 12H, C*H*_2_N), 3.11 (t, *J* = 7.5 Hz, 8H, C*H*_2_NH_2_), 2.61–2.51 (m, 4H, PC*H*_2_), 2.51–2.44 (m, 4H, CHC*H*_2_CH_2_O), 2.28–2.15 (m, 8H, C*H*_2_CH_2_NH_2_), 2.15–2.03 (m, 4H, CH_3_(CH_2_)_2_C*H*_2_), 1.81–1.70 (m, 4H, CH_2_C*H*_2_CH_2_O), 1.54–1.44 (m, 4H, C*H*_2_(CH_2_)_2_O), 1.42–1.29 (m, 8H, CH_3_(C*H*_2_)_2_), 0.97–0.88 (m, 6H, C*H*_3_).

^13^C NMR (101 MHz, CD_3_OD): 134.22 (CH_3_(CH_2_)_3_*C*H), 125.02 (*C*H(CH_2_)_2_O), 68.01 (d, *J* = 6.6 Hz, (CH_2_)_2_*C*H_2_O), 67.99 (d, *J* = 6.6 Hz, (CH_2_)_2_*C*H_2_O), 67.56 (d, *J* = 6.8 Hz, CHCH_2_*C*H_2_O), 50.99 (*C*H_2_(CH_2_)_2_NH_2_), 48.88 (PCH_2_*C*H_2_), 37.93 (*C*H_2_NH_2_), 32.87 (CH_3_CH_2_*C*H_2_), 31.33 (d, *J* = 6.0 Hz, CH_2_*C*H_2_CH_2_O), 31.30 (d, *J* = 6.1 Hz, CH*C*H_2_CH_2_O), 29.61 (d, *J* = 6.0 Hz, CH*C*H_2_CH_2_O), 28.05 (CH_3_(CH_2_)_2_*C*H_2_), 26.11 (*C*H_2_(CH_2_)_2_O), 23.33 (CH_3_*C*H_2_), 23.17 (*C*H_2_CH_2_NH), 21.41 (d, *J* = 140.0 Hz), 21.39 (d, *J* = 140.2 Hz, P*C*H_2_), 14.35 (*C*H_3_).

^31^P{^1^H} NMR (162 MHz, CD_3_OD): 28.41.

**IR** ν_max_ (KBr) 3432 (m, br), 3015 (s), 2957 (vs), 2931 (vs), 2872 (s), 2500–2300 (vs, vbr), 1611 (m), 1467 (m), 1380 (w, sh), 1228 (m), 1055 (s), 1003 (s).

**HR-MS** (ESI^+^): for C_38_H_84_O_6_N_6_P_2_ (M + 2H)^2+^*m*/2*z* calculated 391.29583, found 391.29589.

#### Hexane-1,6-diyl Bis((*Z*)-oct-3-en-1-yl) Bis((2-(bis(3-guanidinopropyl)amino)ethyl)phosphonate) Hexahydrochloride (**42**)

The title compound was prepared according to general method **F** from **41** (0.20 g, 0.20 mmol) in 67% yield (0.14 g, 0.13 mmol) as a white solid.

Mixture of diastereoisomers.

^1^H NMR (400 MHz, CD_3_OD): 5.62–5.51 (m, 2H, CH_3_**(**CH_2_)_3_C*H*), 5.47–5.36 (m, 2H, C*H*(CH_2_)_2_O), 4.22–4.07 (m, 8H, C*H*_2_O), 3.50–3.41 (m, 4H, PCH_2_C*H*_2_), 3.41–3.32 (m, 16H, C*H*_2_CH_2_C*H*_2_NH), 2.64–2.43 (m, 8H, CHC*H*_2_CH_2_O, PC*H*_2_), 2.18–2.03 (m, 12H, CH_3_(CH_2_)_2_C*H*_2_, C*H*_2_CH_2_NH), 1.80–1.69 (m, 4H, OCH_2_C*H*_2_CH_2_), 1.52–1.43 (m, 4H, O(CH_2_)_2_C*H*_2_), 1.41–1.30 (m, 8H, CH_3_(C*H*_2_)_2_, 0.96–0.88 (m, 6H, C*H*_3_).

^13^C NMR (101 MHz, CD_3_OD): 158.67 (*C*=NH), 134.33 (CH_3_(CH_2_)_2_*C*H), 125.07 (*C*H(CH_2_)_2_O), 68.05 (d, *J* = 6.6 Hz), 68.02 (d, *J* = 6.6 Hz, (CH_2_)_2_*C*H_2_O), 67.60 (d, *J* = 6.6 Hz, CHCH_2_*C*H_2_O), 51.55 (*C*H_2_NH), 48.69 (PCH_2_C*H*_2_), 39.69 (*C*H_2_(CH_2_)_2_NH), 32.93 (CH_3_*C*H_2_), 31.41 (d, *J* = 6.2 Hz), 31.38 (d, *J* = 6.2 Hz, CH_2_*C*H_2_CH_2_O), 29.64 (d, *J* = 6.0 Hz, CH*C*H_2_CH_2_O), 28.11 (CH_3_(CH_2_)_2_*C*H_2_), 26.19, 26.17 (*C*H_2_(CH_2_)_2_O), 24.78 (*C*H_2_CH_2_NH), 23.39 (CH_3_*C*H_2_), 21.35 (d, *J* = 140.2 Hz, P*C*H_2_), 14.38 (*C*H_3_).

^31^P{^1^H} NMR (162 MHz, CD_3_OD): 28.80.

**IR** ν_max_ 3400–3200 (vs), 3316 (s), 3258 (s), 3148 (s), 3013 (w), 2956 (m), 2932 (m), 2871 (w), 1666 (vs), 1646 (s), 1619 (s, sh), 1466 (m), 1377 (m), 1224 (m), 1006 (s), 721 (w).

**HR-MS** (ESI^+^): for C_42_H_92_O_6_N_14_P_2_ (M + 2H)^2+^*m*/2*z* calculated 475.33943, found 475.33956.

#### Hexane-1,6-diyl Bis((*Z*)-oct-3-en-1-yl) Bis((2-(bis(2-aminoethyl)amino)ethyl)phosphonate) Hexahydrochloride (**43**)

The title compound was prepared according to general methods **A1**, **B2**, **C**, **D**, and **E** from mono methyl vinylphosphonate (0.33 g, 2.41 mmol) in 13% overall yield (0.30 g, 0.32 mmol) as a white solid.

^1^H NMR (401 MHz, CD_3_OD): 5.61–5.51 (m, 2H, CH_3_(CH_2_)_3_C*H*), 5.47–5.37 (m, 2H, C*H*(CH_2_)_2_O), 4.17–4.03 (m, 8H, C*H*_2_O), 3.24–3.14 (m, 8H, C*H*_2_NH_2_), 3.09–2.93 (m, 12H, PCH_2_C*H*_2_, C*H*_2_N), 2.47 (q, 4H, *J* = 6.9 Hz, CHC*H*_2_CH_2_O), 2.33–2.18 (m, 4H, PC*H*_2_), 2.15–2.04 (m, 4H, CH_3_(CH_2_)_2_C*H*_2_, 1.80–1.69 (m, 4H, CH_2_C*H*_2_CH_2_O), 1.50–1.45 (m, 4H, C*H*_2_(CH_2_)_2_O), 1.40–1.32 (m, 8H, CH_3_(C*H*_2_)_2_), 0.96–0.89 (m, 6H, C*H*_3_).

^13^C NMR (101 MHz, CD_3_OD): 134.30 (CH_3_(CH_2_)_3_*C*H), 125.12 (*C*H(CH_2_)_2_O), 67.64 (d, *J* = 6.6 Hz, (CH_2_)_2_*C*H_2_O), 67.19 (d, *J* = 6.7 Hz, CHCH_2_*C*H_2_O), 51.51 (*C*H_2_CH_2_NH_2_), 37.67 (*C*H_2_NH_2_), 32.93 (CH_3_CH_2_*C*H_2_), 31.49 (d, *J* = 6.1 Hz), 31.47 (d, *J* = 5.9 Hz, CH_2_*C*H_2_CH_2_O), 29.65 (d, *J* = 6.2 Hz, CH*C*H_2_CH_2_O), 28.11 (CH_3_(CH_2_)_2_*C*H_2_), 26.24 (*C*H_2_(CH_2_)_2_O), 23.40 (CH_3_*C*H_2_), 22.68 (d, *J* = 137.2 Hz, P*C*H_2_), 14.37 (*C*H_3_).

^31^P{^1^H} NMR (162 MHz, CD_3_OD): 33.79.

**IR** ν_max_ (KBr) 3250–2500 (s), 3007 (s), 2959 (vs), 2930 (s), 2871 (s), 1652 (w), 1592 (m), 1469 (m), 1385 (w), 1227 (m), 1055 (s), 1007 (s), 790 (w).

**HR-MS** (ESI^+^): for C_34_H_75_O_6_N_6_P_2_ (M + H)^+^*m*/*z* calculated 725.52178, found 725.52136.

#### Hexane-1,6-diyl Diphenethyl Bis((2-(bis(3-aminopropyl)amino)ethyl)phosphonate) Hexahydrochloride (**44**)

The title compound was prepared according to general methods **A1**, **B1**, **C**, **D**, and **E** from mono methyl vinylphosphonate (1.21 g, 9.92 mmol) in 13% overall yield (1.27 g, 1.29 mmol) as a white amorphous solid.

^1^H NMR (500.2 MHz, CD_3_OD): 7.42–7.18 (m, 10H, Ph), 4.42–4.29 (m, 4H, C*H*_2_CH_2_Ph), 4.10–3.90 (m, 4H, OC*H*_2_(CH_2_)_2_), 3.38–3.30 (m, 12H, NC*H*_2_), 3.10 (t, 8H, *J* = 7.5 Hz, C*H*_2_NH_2_), 3.03 (t, 4H, *J* = 6.6 Hz, C*H*_2_Ph), 2.48 (ddd, 4H, *J* = 21.5, 10.4, 5.6 Hz, PC*H*_2_), 2.24–2.11 (m, 8H, C*H*_2_CH_2_NH_2_), 1.65 (p, *J* = 6.5 Hz, 4H, OCH_2_C*H*_2_CH_2_), 1.38 (td, *J* = 7.3, 6.8, 2.5 Hz, 4H, O(CH_2_)_2_C*H*_2_).

^13^C NMR (125.8 MHz, CD_3_OD): 138.84 (*C*_quat_), 130.27 (*C*_ortho_), 129.71 (*C*_meta_), 127.89 (*C*_para_), 68.62 (d, *J* = 6.7 Hz, O*C*H_2_CH_2_Ph), 67.94 (d, *J* = 6.7 Hz), 67.91 (d, *J* = 7.1 Hz, O*C*H_2_(CH_2_)_2_), 51.05 (*C*H_2_(CH_2_)_2_NH_2_), 48.72 (PCH_2_*C*H_2_), 37.87 (*C*H_2_NH_2_), 37.72 (d, *J* = 6.2 Hz, *C*H_2_Ph), 31.28 (d, *J* = 5.8 Hz), 31.25 (d, *J* = 5.8 Hz, OCH_2_*C*H_2_CH_2_), 26.04, 26.02 (O(CH_2_)_2_*C*H_2_), 23.23 (*C*H_2_CH_2_NH_2_), 21.25 (d, *J* = 140.1 Hz), 21.24 (d, *J* = 140.7 Hz, P*C*H_2_).

^31^P{^1^H} NMR (202.5 MHz, CD_3_OD): 26.43.

**IR** ν_max_ (KBr) 2950 (vs, vbr), 2930 (vs), 2858 (vs), 2033 (w, vbr), 2740 (s, br, sh), 2638 (s, br), 2559 (s, br), 1604 (s), 1521 (m, sh), 1497 (s), 1469 (s), 1454 (s), 1408 (m), 1395 (m), 1256 (s, br, sh), 1226 (s, br), 1157 (m), 1060 (s), 1009 (vs), 1000 (vs), 907 (m), 769 (m, sh), 753 (s), 702 (s), 621 (vw), 574 (m), 491 (m).

**HR-MS** (ESI^+^): for C_38_H_71_N_6_O_6_P_2_ (M + H)^+^*m*/*z* calculated 769.49048, found 769.48989.

#### Hexane-1,6-diyl Diphenethyl Bis((2-(bis(2-aminoethyl)amino)ethyl)phosphonate) Hexahydrochloride (**45**)

The title compound was prepared according to general methods **A1**, **B1**, **C**, **D**, and **E** from mono methyl vinylphosphonate (0.24 g, 2.0 mmol) in 14% overall yield (0.26 g, 0.28 mmol) as a white amorphous solid.

^1^H NMR (401 MHz, CD_3_OD): 7.38–7.19 (m, 10H, Ph), 4.31 (q, 4H, *J* = 6.8 Hz, CH_2_CH_2_Ph), 4.04–3.87 (m, 4H, OC*H*_2_(CH_2_)_4_C*H*_2_O), 3.27–3.13 (m, 8H, (C*H*_2_NH_2_), 3.12–2.90 (m, 16H, NC*H*_2_, C*H*_2_Ph), 2.29–2.12 (m, 4H, PC*H*_2_), 1.70–1.56 (m, 4H, OCH_2_C*H*_2_CH_2_), 1.40–1.34 (m, 4H, O(CH_2_)_2_C*H*_2_).

^13^C NMR (101 MHz, CD_3_OD): 138.94 (C_quat_), 130.25 (C_ortho_), 129.67 (C_meta_), 127.87 (C_para_), 68.31 (d, *J* = 6.6 Hz, *C*H_2_CH_2_Ph), 67.55 (d, *J* = 7.0 Hz), 67.54 (d, *J* = 6.8 Hz, O*C*H_2_(CH_2_)_4_*C*H_2_O), 51.47 (*C*H_2_CH_2_NH_2_), 47.95 (PCH_2_*C*H_2_), 37.75 (d, *J* = 6.5 Hz, *C*H_2_Ph), 37.30 (*C*H_2_NH_2_), 31.33 (d, *J* = 6.2 Hz), 31.31 (d, *J* = 6.3 Hz, OCH_2_*C*H_2_CH_2_), 26.09, 26.08 (O(CH_2_)_2_*C*H_2_), 22.41 (d, *J* = 138.5 Hz, P*C*H_2_).

^31^P{^1^H} NMR (162 MHz, CD_3_OD): 32.84.

**IR***ν*_max_ (KBr) 3023 (vs), 2958 (vs), 2950 (vs, vbr), 2933 (vs), 2863 (s), 2635 (m, br, sh), 2554 (m, vbr), 1978 (vw, vbr)1602 (w), 1593 (w, br), 1496 (m), 1470 (m), 1454 (m), 1392 (w, br), 1258 (m, sh), 1225 (s, sh), 1212 (s), 1052 (s), 1013 (vs), 1001 (vs), 769 (w), 753 (m), 702 (m), 621 (vw), 574 (w), 489 (w).

**HR-MS** (ESI^+^): for C_34_H_63_N_6_O_6_P_2_ (M + H)^+^*m*/*z* calculated 713.42788, found 713.42754.

#### Hexane-1,6-diyl Bis((*Z*)-non-3-en-1-yl) Bis((2-(bis(3-aminopropyl)amino)ethyl)phosphonate) Hexahydrochloride (**46**)

The title compound was prepared according to general methods **A1**, **B1**, **C**, **D**, and **E** from mono methyl vinylphosphonate (3 g, 24.58 mmol) in 4% overall yield (0.95 g, 0.92 mmol) as a white amorphous solid.

^1^H NMR (500.2 MHz, CD_3_OD): 5.56 (dtt, 1H, *J* = 10.5, 7.3, ^4^*J* = 1.6 Hz, CH_3_(CH_2_)_4_C*H*), 5.42 (dtt, 1H, *J* = 10.5, 6.9, ^4^*J* = 1.6 Hz, C*H*(CH_2_)_2_O), 4.21–4.09 (m, 8H, CHCH_2_C*H*_2_O, OC*H*_2_(CH_2_)_2_), 3.50–3.44 (m, 4H, PCH_2_C*H*_2_), 3.44–3.37 (m, 8H, C*H*_2_(CH_2_)_2_NH_2_), 3.14–3.09 (m, 8H, C*H*_2_NH_2_), 2.62–2.52 (m, 4H, PC*H*_2_), 2.48 (qd, 4H, *J* = 6.9, ^4^*J* = 1.6 Hz, CHC*H*_2_CH_2_O), 2.27–2.18 (m, 8H, C*H*_2_CH_2_NH_2_), 2.08 (qd, 4H, *J* = 7.3, ^4^*J* = 1.6, CH_3_(CH_2_)_3_C*H*_2_), 1.81–1.72 (m, 4H, OCH_2_C*H*_2_CH_2_), 1.45–1.51 (m, 4H, O(CH_2_)_2_C*H*_2_), 1.42–1.27 (m, 12H, CH_3_(C*H*_2_)_3_), 0.93–0.89 (m, 6H, C*H*_3_).

^13^C NMR (125.8 MHz, CD_3_OD): 68.08, 68.06 (d, *J* = 6.7 Hz, O*C*H_2_(CH_2_)_2_), 67.63 (d, *J* = 6.8 Hz, CHCH_2_*C*H_2_O), 51.06 (*C*H_2_(CH_2_)_2_NH_2_), 48.87 (PCH_2_*C*H_2_), 37.88 (*C*H_2_NH_2_), 32.62 (CH_3_*C*H_2_), 31.37, 31.34 (d, *J* = 5.9 Hz, OCH_2_*C*H_2_CH_2_), 30.36 (CH_3_(CH_2_)_2_*C*H_2_), 29.64 (d, *J* = 6.0 Hz, CH*C*H_2_CH_2_O), 28.34 (CH_3_(CH_2_)_3_*C*H_2_), 26.13, 26.12 (O(CH_2_)_2_*C*H_2_), 23.60 (CH_3_CH_2_*C*H_2_), 23.21 (*C*H_2_CH_2_NH_2_), 21.36, 21.35 (d, *J* = 140.3 Hz, P*C*H_2_), 14.42 (*C*H_3_).

^31^P{^1^H} NMR (202.5 MHz, CD_3_OD): 26.94.

**IR** ν_max_ (KBr) 3010 (s, sh), 2957 (vs), 2930 (vs), 2872 (s), 2859 (s), 2009 (w, vbr), 2735 (m, sh), 2633 (m), 2558 (m), 1655 (vw, sh), 1601 (m), 1511 (m, sh), 1467 (m), 1406 (w), 1380 (w), 1252 (m, sh), 1227 (m), 1070 (s, sh), 1056 (s), 1003 (s), 808 (w).

**HR-MS** (ESI^+^): for C_40_H_88_N_6_O_6_P_2_ (M + 2H)^+^*m*/2*z* calculated 405.31148, found 405.31180.

#### Didecyl Hexane-1,6-diyl Bis((2-(bis(3-aminopropyl)amino)ethyl)phosphonate) Hexahydrochloride (**47**)

The title compound was prepared according to general methods **A1**, **B1**, **C**, **D**, and **E** from mono methyl vinylphosphonate (0.75 g, 6.15 mmol) in 13% overall yield (0.85 g, 0.8 mmol) as a white amorphous solid.

Mixture of diastereoisomers.

^1^H NMR (401 MHz, CD_3_OD) δ 4.25–4.06 (m, 8H, OC*H*_2_), 3.54–3.44 (m, 4H, PCH_2_C*H*_2_), 3.44–3.35 (m, 8H, C*H*_2_(CH_2_)_2_NH_2_), 3.12 (t, *J* = 7.5 Hz, 8H, C*H*_2_NH_2_), 2.64–2.50 (m, 4H, PC*H*_2_), 2.22 (tt, *J* = 8.5, 6.0 Hz, 8H, C*H*_2_CH_2_NH_2_), 1.84–1.65 (m, 8H, OCH_2_C*H*_2_), 1.49 (dt, *J* = 9.1, 2.9 Hz, 4H, O(CH_2_)_2_(C*H*_2_)_2_(CH_2_)_2_O), 1.44–1.36 (m, 4H, C*H*_2_(CH_2_)_6_CH_3_), 1.36–1.21 (m, 24H, (C*H*_2_)_6_CH_3_), 0.96–0.84 (m, 6H, C*H*_3_).

^13^C NMR (101 MHz, CD_3_OD) δ 68.24 (d, *J* = 6.8 Hz), 68.04 (d, *J* = 6.7 Hz, O*C*H_2_(CH_2_)_4_*C*H_2_O), 68.01 (d, *J* = 6.5 Hz, O*C*H_2_(CH_2_)_8_CH_3_), 51.01 (N*C*H_2_(CH_2_)_2_NH_2_), 48.87 (PCH_2_*C*H_2_), 37.88 (*C*H_2_NH_2_), 33.06 (*C*H_2_(CH_2_)_5_CH_3_), 31.61 (d, *J* = 5.9 Hz, *C*H_2_(CH_2_)_7_CH_3_), 31.39 (d, *J* = 5.8 Hz), 31.35 (d, *J* = 5.6 Hz, OCH_2_*C*H_2_(CH_2_)_2_*C*H_2_CH_2_O), 30.69, 30.45, 30.31, 26.62 (O(CH_2_)_2_(*C*H_2_)_2_(CH_2_)_2_O), 26.13 (*C*H_2_(CH_2_)_6_CH_3_), 23.73, 23.21 (*C*H_2_CH_2_NH_2_), 21.32 (d, *J* = 139.1 Hz), 21.30 (d, *J* = 140.4 Hz, P*C*H_2_), 14.45 (*C*H_3_).

^31^P{^1^H} NMR (162 MHz, CD_3_OD) δ 28.65, 28.64.

**IR** ν_max_ (KBr) 2956 (vs), 2926 (vs), 2855 (vs), 2740 (s, sh), 2629 (s, br), 2556 (m), 2010 (w, vbr), 1603 (m), 1521 (w, sh), 1468 (s), 1395 (m), 1254 (s, sh), 1228 (s), 1060 (s, sh), 1000 (vs, br), 723 (w).

**HR-MS** (ESI^+^): for C_42_H_95_N_6_O_6_P_2_ (M + H)^+^*m*/*z* calculated 841.67828, found 841.67850.

#### Bis((*Z*)-dec-4-en-1-yl) Hexane-1,6-diyl Bis((2-(bis(3-aminopropyl)amino)ethyl)phosphonate) Hexahydrochloride (**48**)

The title compound was prepared according to general methods **A1**, **B1**, **C**, **D**, and **E** from mono methyl vinylphosphonate (1.63 g, 13.4 mmol) in 2% overall yield (0.29 g, 0.29 mmol) as a white solid.

^1^H NMR (500 MHz, CD_3_OD) δ 5.49–5.35 (m, 4H, C*H*=C*H*), 4.22–4.09 (m, 8H, OC*H*_2_), 3.50–3.42 (m, 4H, PCH_2_C*H*_2_), 3.42–3.35 (m, 8H, PCH_2_CH_2_NC*H*_2_), 3.14–3.07 (m, 8H, C*H*_2_NH_2_), 2.55 (dtd, *J* = 19.3, 7.9, 7.4, 2.4 Hz, 4H, PC*H*_2_), 2.27–2.14 (m, 12H, C*H*_2_CH_2_NH_2_), 2.06 (q, *J* = 7.0 Hz, 4H, CH_3_(CH_2_)_3_C*H*_2_), 1.83–1.72 (m, 8H, OCH_2_C*H*_2_), 1.49 (tt, *J* = 4.4, 1.9 Hz, 4H, O(CH_2_)_2_(C*H*_2_)_2_), 1.44–1.24 (m, 12H, CH_3_(C*H*_2_)_3_), 0.95–0.89 (m, 6H, C*H*_3_).

^13^C NMR (126 MHz, CD_3_OD) δ 132.26 (CH_3_(CH_2_)_4_*C*H), 129.08 (O(CH_2_)_3_*C*H), 68.08 (dd, *J* = 6.7, 2.8 Hz, O*C*H_2_(CH_2_)_4_*C*H_2_O), 67.75 (d, *J* = 6.7 Hz, O*C*H_2_(CH_2_)_2_CH), 51.09 (PCH_2_CH_2_N*C*H_2_), 48.83 (PCH_2_*C*H_2_), 37.92 (*C*H_2_NH_2_), 32.67 (*C*H_2_CH_3_), 31.71 (d, *J* = 5.9 Hz, OCH_2_*C*H_2_CH_2_CH), 31.43 (dd, *J* = 5.8, 3.5 Hz, OCH_2_*C*H_2_(CH_2_)_2_*C*H_2_CH_2_O), 30.49 (CH_3_(CH_2_)_2_*C*H_2_), 28.20 (CH_3_(CH_2_)_3_*C*H_2_), 26.18 (d, *J* = 1.7 Hz, (*C*H_2_)_2_(CH_2_)_2_O), 24.19 (O(CH_2_)_2_*C*H_2_CH), 23.65 (CH_3_CH_2_*C*H_2_), 23.32 (*C*H_2_CH_2_NH_2_), 21.35 (d, *J* = 139.9 Hz, P*C*H_2_), 14.45 (*C*H_3_).

^31^P{^1^H} NMR (202 MHz, CD_3_OD) δ 27.07.

**IR** ν_max_ (film) 3395 (w), 3007 (s), 2956 (vs), 2927 (vs), 2872 (s), 2852 (s), 2560–2741 (m), 1655 (w), 1608 (m), 1467 (m), 1404 (m), 1379 (m), 1230 (m), 1003 (vs), 845 (m), 758 (w), 720 (w).

**HR-MS** (ESI^+^): for C_42_H_91_O_6_N_6_P_2_ (M + H)^+^*m*/*z* calculated 837.64698, found 837.64717. For C_42_H_92_O_6_N_6_P_2_ (M + 2H)^2+^*m*/2*z* calculated 419.32713, found 419.32717.

#### Bis(2-(adamantan-1-yl)ethyl) Hexane-1,6-diyl Bis((2-(bis(3-aminopropyl)amino)ethyl)phosphonate) Hexahydrochloride (**49**)

The title compound was prepared according to general methods **A1**, **B1**, **C**, **D**, and **E** from mono methyl vinylphosphonate (3.39 g, 27.7 mmol) in 8% overall yield (2.45 g, 2.22 mmol) as a white amorphous solid.

Mixture of diastereoisomers.

^1^H NMR (401 MHz, CD_3_OD): 4.25–4.09 (m, 8H, OC*H*_2_), 3.46 (dd, 4H, *J* = 10.7, 5.9 Hz, PCH_2_C*H*_2_), 3.44–3.36 (m, 8H, C*H*_2_(CH_2_)_2_NH_2_), 3.11 (t, 8H, *J* = 7.5 Hz, C*H*_2_NH_2_), 2.64–2.48 (m, 4H, PC*H*_2_), 2.27–2.15 (m, 8H, C*H*_2_CH_2_NH_2_), 1.96 (p, 6H, *J* = 3.2 Hz, C*H*), 1.81–1.65 (m, 16H, C_quat_C*H*_2_CH, OCH_2_C*H*_2_CH_2_), 1.59 (d, 12H, *J* = 2.9 Hz, *c*-(CHC*H*_2_)_3_), 1.57–1.45 (m, 8H, O(CH_2_)_2_C*H*_2_, OCH_2_C*H*_2_C_quat_).

^13^C NMR (101 MHz, CD_3_OD): 68.09 (d, *J* = 4.6 Hz), 68.06 (d, *J* = 3.9 Hz, O*C*H_2_(CH_2_)_2_), 64.62 (d, *J* = 6.7 Hz, O*C*H_2_CH_2_C_quat_), 51.04 (*C*H_2_(CH_2_)_2_NH_2_), 48.87 (d, *J* = 2.3 Hz, PCH_2_*C*H_2_), 45.55 (d, *J* = 5.7 Hz, OCH_2_*C*H_2_C_quat_), 43.62 (*c*-(CH*C*H_2_)_3_), 38.02 (C_quat_*C*H_2_CH), 37.88 (*C*H_2_NH_2_), 32.92 (*C*_quat_), 31.42 (d, *J* = 5.5 Hz), 31.40 (d, *J* = 5.1 Hz, OCH_2_*C*H_2_CH_2_), 30.04 (*C*H), 26.22 (O(CH_2_)_2_*C*H_2_), 23.26 (*C*H_2_CH_2_NH_2_), 21.35 (d, *J* = 139.2 Hz), 21.33 (d, *J* = 140.2 Hz, P*C*H_2_).

^31^P{^1^H} NMR (162 MHz, CD_3_OD): 28.74.

**IR** ν_max_ (KBr) 2921 (vs), 2904 (vs), 2848 (vs), 2750 (s, br, sh), 2660 (s, br, sh), 2560 (m, br, sh), 2059 (w, vbr), 1507 (w, br), 1451 (m), 1345 (w), 1230 (m, vbr), 1099 (w), 1045 (m), 1010 (br, sh), 997 (s, vbr), 973 (m, sh), 813 (w).

**HR-MS** (ESI^+^): for C_46_H_91_N_6_O_6_P_2_ (M + H)^+^*m*/*z* calculated 885.64698, found 885.64665.

#### Heptane-1,7-diyl Bis(4,4,4-trifluorobutyl) Bis((2-(bis(3-aminopropyl)amino)ethyl)phosphonate) Hexahydrochloride (**50**)

The title compound was prepared according to general methods **A1**, **B2**, **C**, **D**, and **E** from mono methyl vinylphosphonate (0.61 g, 4.51 mmol) in 14% overall yield (0.66 g, 0.65 mmol) as a white solid.

^1^H NMR (500 MHz, CD_3_OD) δ 4.27–4.10 (m, 8H, C*H*_2_O), 3.51–3.43 (m, 4H, PCH_2_C*H*_2_), 3.43–3.36 (m, 8H, C*H*_2_(CH_2_)_2_NH_2_), 3.10 (t, *J* = 7.5 Hz, 8H, C*H*_2_NH_2_), 2.63–2.52 (m, 4H, PC*H*_2_), 2.41–2.28 (m, 4H, C*H*_2_CF_3_), 2.26–2.16 (m, 8H, C*H*_2_CH_2_NH_2_), 2.03–1.94 (m, 4H, C*H*_2_CH_2_CF_3_), 1.79–1.70 (m, 4H, OCH_2_C*H*_2_(CH_2_)_3_C*H*_2_CH_2_O), 1.51–1.37 (m, 6H, O(CH_2_)_2_(C*H*_2_)_3_(CH_2_)_2_O).

^13^C NMR (126 MHz, CD_3_OD) δ 128.66 (q, *J* = 275.2 Hz, *C*F_3_), 68.38 (d, *J* = 6.8 Hz, O*C*H_2_(CH_2_)_5_*C*H_2_O), 66.41 (d, *J* = 6.4 Hz, O*C*H_2_(CH_2_)_2_CF_3_), 51.06 (PCH_2_*C*H_2_), 37.87 (*C*H_2_CH_2_NH_2_), 31.47 (d, *J* = 5.8 Hz, OCH_2_*C*H_2_(CH_2_)_2_), 30.94 (q, *J* = 29.2 Hz, *C*H_2_CF_3_), 29.74 (O(CH_2_)_3_*C*H_2_), 26.48 (O(CH_2_)_2_*C*H_2_CH_2_), 24.46 (dq, *J* = 6.3, 3.1 Hz, *C*H_2_CH_2_CF_3_), 23.28 (*C*H_2_CH_2_NH_2_), 21.23 (d, *J* = 140.3 Hz, P*C*H_2_).

^31^P{^1^H} NMR (202 MHz, CD_3_OD) δ 27.67.

^19^F NMR (377 MHz, CD_3_OD): −67.78 (t, *J* = 11.2 Hz).

**IR** ν_max_ 3100–2500 (vs), 2944 (vs), 2864 (s), 2019 (w), 1605 (m), 1519 (m, sh), 1393 (m), 1340 (m), 1256 (s), 1154 (s), 1134 (m), 1100–1000 (vs), 990 (s).

**HR-MS** (ESI^+^): for C_31_H_68_O_6_N_6_F_6_P_2_ (M + 2H)^2+^*m*/2*z* calculated 398.22844, found 398.22800.

#### Heptane-1,7-diyl Bis(4,4,4-trifluorobutyl) Bis((2-(bis(3-guanidinopropyl)amino)ethyl)phosphonate) Hexahydrochloride (**51**)

The title compound was prepared according to general method F from **50** (0.28 g, 0.28 mmol) in 64% yield (0.21 g, 0.18 mmol) as a white solid.

^1^H NMR (401 MHz, CD_3_OD): 4.26–4.09 (m, 8H, C*H*_2_O), 3.51–3.42 (m, 4H, PCH_2_C*H*_2_), 3.42–3.32 (m, 16H, N(CH_2_)_2_C*H*_2_NH, NC*H*_2_(CH_2_)_2_NH), 2.68–2.52 (m, 4H, PC*H*_2_CH_2_N), 2.42–2.26 (m, 4H, CF_3_C*H*_2_(CH_2_)_2_O), 2.17–2.06 (m, 8H, NCH_2_C*H*_2_CH_2_NH_2_), 2.03–1.92 (m, 4H, CF_3_CH_2_C*H*_2_CH_2_O), 1.81–1.68 (m, 4H, OCH_2_C*H*_2_(CH_2_)_3_C*H*_2_CH_2_O), 1.50–1.39 (m, 6H, O(CH_2_)_2_(C*H*_2_)_3_(CH_2_)_2_O).

^13^C NMR (101 MHz, CD_3_OD): 158.65 (*C*=NH), 128.65 (q, *J* = 275.3 Hz, *C*F_3_), 68.31 (d, *J* = 6.7 Hz, O*C*H_2_(CH_2_)_3_), 66.37 (d, *J* = 6.3 Hz, CF_3_(CH_2_)_2_*C*H_2_O), 51.51 (*C*H_2_NH_2_), 48.64 (PCH_2_C*H*_2_), 39.67 (*C*H_2_(CH_2_)_2_NH), 31.42 (d, *J* = 6.2 Hz, OCH_2_*C*H_2_(CH_2_)_2_), 30.94 (q, *J* = 29.2 Hz, CF_3_*C*H_2_), 29.71 (O(CH_2_)_3_*C*H_2_), 26.47 (O(CH_2_)_2_*C*H_2_CH_2_), 24.74 (*C*H_2_CH_2_NH_2_), 24.44 (dq, *J* = 6.2, 3.1 Hz, CF_3_CH_2_*C*H_2_), 21.29 (d, *J* = 140.2 Hz, P*C*H_2_).

^31^P{^1^H} NMR (162 MHz, CD_3_OD): 29.13.

^19^F NMR (377 MHz, CD_3_OD): −67.70 (t, *J* = 11.1 Hz).

**IR** ν_max_ 3387 (s, vbr), 3159 (s, br), 2955 (m), 2859 (w), 2709 (w, vbr), 2597 (w, br), 2495 (w, br), 1671 (vs), 1645 (vs), 1620 (s), 1390 (m), 1341 (m), 1257 (s), 1236 (s), 1154 (s), 1132 (s), 1020 (s, br).

**HR-MS** (ESI^+^): for C_35_H_75_O_6_N_14_F_6_P_2_ (M + H)^+^*m*/*z* calculated 963.53679, found 963.53701.

#### Heptane-1,7-diyl Diheptyl Bis((2-(bis(3-aminopropyl)amino)ethyl)phosphonate) Hexahydrochloride (**52**)

The title compound was prepared according to general methods **A1**, **B1**, **C**, **D**, and **E** from mono methyl vinylphosphonate (175 mg, 1.44 mmol) in 20% overall yield (0.29 g, 0.29 mmol) as a white solid.

^1^H NMR (401 MHz, CD_3_OD) δ 4.13 (q, *J* = 7.0 Hz, 8H, OC*H*_2_), 3.43 (t, *J* = 6.5 Hz, 4H, PCH_2_C*H*_2_), 3.40–3.34 (m, 8H, P(CH_2_)_2_NC*H*_2_), 3.09 (t, *J* = 7.5 Hz, 8H, C*H*_2_NH_2_), 2.50 (dtd, *J* = 16.8, 8.3, 4.8 Hz, 4H, PC*H*_2_), 2.25–2.14 (m, 8H, C*H*_2_CH_2_NH_2_), 1.78–1.67 (m, 8H, OCH_2_C*H*_2_), 1.49–1.25 (m, 22H), 0.97–0.87 (m, 6H, C*H*_3_).

^13^C NMR (101 MHz, CD_3_OD) δ 68.26 (d, *J* = 6.7 Hz), 68.22 (d, *J* = 6.5 Hz, O*C*H_2_), 51.10 (*C*H_2_(CH_2_)_2_NH_2_), 48.72 (PCH_2_*C*H_2_), 37.86 (*C*H_2_NH_2_), 32.93 (*C*H_2_CH_2_CH_3_), 31.62 (d, *J* = 6.1 Hz), 31.55 (d, *J* = 6.1 Hz, OCH_2_*C*H_2_), 30.11, 29.98, 27.20, 26.60 (O(CH_2_)_2_(*C*H_2_)_2_), 23.67 (*C*H_2_CH_3_), 23.32 (*C*H_2_CH_2_NH_2_), 21.19 (d, *J* = 140.9 Hz, P*C*H_2_), 14.42 (*C*H_3_).

^31^P{^1^H} NMR (162 MHz, CD_3_OD) δ 28.68.

**IR** ν_max_ (film) ∼3000–2500 (m–s, vbr), 2954 (vs), 2931 (vs), 2858 (s), 2748 (m), 2633 (m), 2559 (w), 2021 (w, vbr), 1602 (w), 1467 (m), 1395 (w), 1378 (w), 1251 (m), 1227 (m), 1063 (m), 1004 (s), 726 (w).

**HR-MS** (ESI^+^): for C_37_H_85_O_6_N_6_P_2_ (M + H)^+^*m*/*z* calculated 771.60003, found 771.59984. For C_37_H_86_O_6_N_6_P_2_ (M + 2H)^2+^*m*/2*z* calculated 386.30366, found 386.30356.

#### Bis((*Z*)-hept-3-en-1-yl) Heptane-1,7-diyl Bis((2-(bis(3-aminopropyl)amino)ethyl)phosphonate) Hexahydrochloride (**53**)

The title compound was prepared according to general methods **A1**, **B2**, **C**, **D**, and **E** from mono methyl vinylphosphonate (0.42 g, 3.08 mmol) in 14% overall yield (0.43 g, 0.44 mmol) as a white solid.

Mixture of diastereoisomers.

^1^H NMR (401 MHz, CD_3_OD): 5.61–5.50 (m, 2H, CH_3_(CH_2_)_2_C*H*), 5.49–5.37 (m, 2H, C*H***(**CH_2_)_2_O), 4.21–4.07 (m, 8H, C*H*_2_O), 3.52–3.35 (m, 12H, C*H*_2_N), 3.12 (t, 8H, *J* = 7.3 Hz, C*H*_2_NH_2_), 2.65–2.44 (m, 8H, CHC*H*_2_CH_2_O, PC*H*_2_), 2.28–2.17 (m, 8H, C*H*_2_CH_2_NH_2_), 2.07 (qd, 4H, *J* = 7.4, 1.4 Hz, CH_3_CH_2_C*H*_2_), 1.78–1.68 (m, 4H, CH_2_C*H*_2_CH_2_O), 1.50–1.34 (m, 10H, CH_3_C*H*_2_, (C*H*_2_)_2_(CH_2_)_2_O), 0.93 (t, 6H, *J* = 7.4 Hz, C*H*_3_).

^13^C NMR (101 MHz, CD_3_OD): 134.07 (CH_3_(CH_2_)_2_*C*H), 125.22 (*C*H(CH_2_)_2_O), 68.14 (d, *J* = 6.8 Hz, (CH_2_)_2_*C*H_2_O), 67.58 (d, *J* = 6.7 Hz, CHCH_2_*C*H_2_O), 51.00 (*C*H_2_(CH_2_)_2_NH_2_), 48.86 (PCH_2_*C*H_2_), 37.88 (*C*H_2_NH_2_), 31.43 (d, *J* = 6.0 Hz), 31.42 (d, *J* = 6.0 Hz, CH_2_*C*H_2_CH_2_O), 30.41 (CH_3_CH_2_*C*H_2_), 29.73, 29.71 (*C*H_2_(CH_2_)_3_O), 29.64 (d, *J* = 6.0 Hz, CH*C*H_2_CH_2_O), 26.48, 26.47 (*C*H_2_(CH_2_)_2_O), 23.75 (CH_3_*C*H_2_), 23.18 (*C*H_2_CH_2_NH), 21.32 (d, *J* = 140.2 Hz, P*C*H_2_), 14.14 (*C*H_3_).

^31^P{^1^H} NMR (162 MHz, CD_3_OD): 28.63.

**IR** ν_max_ (KBr) 3100–2500 (vs, vbr), 3010 (s, sh), 2957 (vs), 2932 (s), 2871 (s), 2027 (w), 1652 (vw), 1604 (m), 1465 (m), 1379 (m), 1226 (m), 1003 (vs).

**HR-MS** (ESI^+^): for C_37_H_81_O_6_N_6_P_2_ (M + H)^+^*m*/*z* calculated 767.56873, found 767.65846.

#### Bis((*Z*)-hept-3-en-1-yl) Heptane-1,7-diyl Bis((2-(bis(2-aminoethyl)amino)ethyl)phosphonate) Hexahydrochloride (**54**)

The title compound was prepared according to general methods **A1**, **B2**, **C**, **D**, and **E** from mono methyl vinylphosphonate (0.39 g, 2.89 mmol) in 9% overall yield (0.24 g, 0.26 mmol) as a white solid.

Mixture of diastereoisomers.

^1^H NMR (401 MHz, CD_3_OD): 5.61–5.52 (m, 2H, CH_3_(CH_2_)_2_C*H*), 5.48–5.38 (m, 2H, C*H*(CH_2_)_2_O), 4.19–4.06 (m, 8H, C*H*_2_O), 3.40–3.21 (m, 20H, C*H*_2_N, C*H*_2_NH_2_), 2.53–2.33 (m, 8H, CHC*H*_2_CH_2_O, PC*H*_2_), 2.07 (q, 4H, *J* = 7.4 Hz, CH_3_CH_2_C*H*_2_), 1.79–1.68 (m, 4H, CH_2_C*H*_2_CH_2_O), 1.52–1.36 (m, 10H, CH_3_C*H*_2_, (C*H*_2_)_2_(CH_2_)_2_O), 0.93 (t, 6H, *J* = 7.4 Hz, C*H*_3_).

^13^C NMR (101 MHz, CD_3_OD): 134.11 (CH_3_(CH_2_)_2_*C*H, 125.28 (*C*H(CH_2_)_2_O), 67.93 (d, *J* = 6.6 Hz, (CH_2_)_2_*C*H_2_O), 67.38 (d, *J* = 6.7 Hz, CHCH_2_*C*H_2_O), 51.48 (*C*H_2_CH_2_NH_2_), 48.84 (PCH_2_*C*H_2_), 36.72 (*C*H_2_NH_2_), 31.47 (d, *J* = 6.0 Hz), 31.46 (d, *J* = 6.1 Hz, CH_2_*C*H_2_CH_2_O), 30.44 (CH_3_CH_2_*C*H_2_), 29.77, 29.75 (*C*H_2_(CH_2_)_2_O), 29.66 (d, *J* = 6.2 Hz, CH*C*H_2_CH_2_O), 26.53 (*C*H_2_(CH_2_)_3_O), 23.78 (CH_3_*C*H_2_), 22.18 (d, *J* = 139.0 Hz, P*C*H_2_), 14.15 (*C*H_3_).

^31^P{^1^H} NMR (162 MHz, CD_3_OD): 31.44.

**IR** ν_max_ (KBr) 3100–2500 (s, vbr), 3010 (s), 2958 (vs), 2932 (s), 2871 (s), 2046 (w), 1653 (vw), 1601 (w), 1466 (m), 1379 (w), 1233 (m), 1004 (vs).

**HR-MS** (ESI^+^): for C_33_H_74_O_6_N_6_P_2_ (M + 2H)^2+^*m*/2*z* calculated 356.25671, found 356.25675.

#### Diisobutyl Octane-1,8-diyl Bis((2-(bis(3-aminopropyl)amino)ethyl)phosphonate) Hexahydrochloride (**55**)

The title compound was prepared according to general methods **A1**, **B1**, **C**, **D**, and **E** from mono methyl vinylphosphonate (260 mg, 2.13 mmol) in 18% overall yield (165 mg, 0.386 mmol) as a white solid.

^1^H NMR (401 MHz, CD_3_OD) δ 4.14 (t, *J* = 7.9, 6.6 Hz, 4H, OC*H*_2_CH_2_), 3.99–3.86 (m, 4H, OC*H*_2_CH), 3.49–3.42 (m, 4H, PCH_2_C*H*_2_), 3.39 (dd, *J* = 9.2, 7.1 Hz, 8H, C*H*_2_(CH_2_)_2_NH_2_), 3.10 (t, *J* = 7.5 Hz, 8H, C*H*_2_NH_2_), 2.54 (dtd, *J* = 16.9, 8.4, 4.7 Hz, 4H, PC*H*_2_), 2.27–2.14 (m, 8H, C*H*_2_CH_2_NH_2_), 1.98 (dh, *J* = 13.3, 6.7 Hz, 2H, OCH_2_C*H*), 1.73 (p, *J* = 6.6 Hz, 4H, OCH_2_C*H*_2_), 1.49–1.36 (m, 8H, O(CH_2_)_2_(C*H*_2_)_2_), 0.99 (d, *J* = 6.7 Hz, 12H, C*H*_3_).

^13^C NMR (101 MHz, CD_3_OD) δ 73.92 (d, *J* = 7.0 Hz, O*C*H_2_CH), 68.25 (d, *J* = 6.7 Hz, O*C*H_2_CH_2_), 51.07 (*C*H_2_(CH_2_)_2_NH_2_), 48.79 (PCH_2_*C*H_2_), 37.87 (*C*H_2_NH_2_), 31.56 (d, *J* = 5.9 Hz, OCH_2_*C*H_2_), 30.46 (d, *J* = 6.3 Hz, OCH_2_*C*H), 30.16, 26.54 (O(CH_2_)_2_(*C*H_2_)_2_), 23.31 (*C*H_2_CH_2_NH_2_), 21.20 (d, *J* = 140.6 Hz, P*C*H_2_), 19.03 (*C*H_3_).

^31^P{^1^H} NMR (162 MHz, CD_3_OD) δ 28.60.

**IR** ν_max_ 2959–2558 (m, vbr), 2953 (vs), 2875 (s), 2754–2558 (m, vbr), 2046 (w), 1608 (w), 1517 (sh, w), 1470 (m), 1396 (w), 1369 (vw), 1227 (m), 1009 (vs), 913 (vw).

**HR-MS** (ESI^+^): for C_32_H_75_O_6_N_6_P_2_ (M + H)^+^*m*/*z* calculated 701.52178, found 701.52204, for C_32_H_76_O_6_N_6_P_2_ (M + 2H)^2+^*m*/2*z* calculated 351.26453, found 351.26459.

#### Dibutyl Octane-1,8-diyl Bis((2-(bis(3-aminopropyl)amino)ethyl)phosphonate) Hexahydrochloride (**56**)

The title compound was prepared according to general methods **A1**, **B2**, **C**, **D**, and **E** from mono methyl vinylphosphonate (0.51 g, 3.77 mmol) in 15% overall yield (0.53 g, 0.57 mmol) as a white solid.

^1^H NMR (400 MHz, CD_3_OD): 4.19–4.09 (m, 8H, C*H*_2_O), 3.49–3.42 (m, 4H, PCH_2_C*H*_2_), 3.42–3.34 (m, 8H, C*H*_2_(CH_2_)_2_NH_2_), 3.10 (t, 8H, *J* = 7.5 Hz, C*H*_2_NH_2_), 2.60–2.46 (m, 4H, PC*H*_2_), 2.27–2.14 (m, 8H, C*H*_2_CH_2_NH_2_), 1.78–1.66 (m, 8H, C*H*_2_CH_2_O), 1.51–1.34 (m, 12H, CH_3_C*H*_2_, (C*H*_2_)_2_(CH_2_)_2_O), 0.98 (t, 6H, *J* = 7.4 Hz, C*H*_3_).

^13^C NMR (101 MHz, CD_3_OD): 68.21 (d, *J* = 6.9 Hz), 67.91 (d, *J* = 6.7 Hz, *C*H_2_O), 51.07 (*C*H_2_(CH_2_)_2_NH_2_), 48.84 (PCH_2_*C*H_2_), 37.90 (*C*H_2_NH_2_), 33.61 (d, *J* = 5.9 Hz), 31.54 (d, *J* = 5.8 Hz, *C*H_2_CH_2_O), 30.14, 26.53 ((*C*H_2_)_2_(CH_2_)_2_O), 23.29 (*C*H_2_CH_2_NH_2_), 21.29 (d, *J* = 140.8 Hz, P*C*H_2_), 19.79 (CH_3_*C*H_2_), 13.96 (*C*H_3_).

^31^P{^1^H} NMR (162 MHz, CD_3_OD): 28.65.

**IR** ν_max_ (KBr) 2954 (vs), 2740 (s, sh), 2632 (s), 2558 (s), 2007 (w), 1599 (m), 1510 (m, sh), 1467 (s), 1223 (s, br), 1064 (s), 1018 (vs, br), 995 (s, br).

**HR-MS** (ESI^+^): for C_32_H_76_N_6_O_6_P_2_ (M + 2H)^2+^*m*/2*z* calculated 351.26453, found 351.26434.

#### Octane-1,8-diyl Dipentyl Bis((2-(bis(3-aminopropyl)amino)ethyl)phosphonate) Hexahydrochloride (**57**)

The title compound was prepared according to general methods **A1**, **B2**, **C**, **D**, and **E** from mono methyl vinylphosphonate (0.51 g, 3.68 mmol) in 16% overall yield (0.54 g, 0.57 mmol) as a white solid.

^1^H NMR (400 MHz, CD_3_OD): 4.20–4.07 (m, 8H, C*H*_2_O), 3.49–3.43 (m, 4H, PCH_2_C*H*_2_), 3.43–3.36 (m, 8H, C*H*_2_(CH_2_)_2_NH_2_), 3.11 (t, 8H, *J* = 7.5 Hz, C*H*_2_NH_2_), 2.61–2.47 (m, 4H, PC*H*_2_), 2.27–2.15 (m, 8H, C*H*_2_CH_2_NH_2_), 1.79–1.67 (m, 8H, C*H*_2_CH_2_O), 1.48–1.32 (m, 16H, (C*H*_2_)_2_(CH_2_)_2_O), 0.99–0.90 (m, 6H, C*H*_3_).

^13^C NMR (101 MHz, CD_3_OD): 68.22 (d, *J* = 6.7 Hz, *C*H_2_O), 51.07 (*C*H_2_(CH_2_)_2_NH_2_), 48.84 (PCH_2_*C*H_2_), 37.89 (*C*H_2_NH_2_), 31.56 (d, *J* = 5.9 Hz), 31.29 (d, *J* = 5.9 Hz, *C*H_2_CH_2_O), 30.16, 30.15, 28.79, 26.54, 23.29, 23.28 (*C*H_2_CH_2_NH_2_), 21.28 (d, *J* = 140.4 Hz, P*C*H_2_), 14.34 (*C*H_3_).

^31^P{^1^H} NMR (162 MHz, CD_3_OD): 28.40.

**IR** ν_max_ (KBr) 2933 (s), 2634 (s), 2558 (m), 1600 (m), 1467 (m), 1224 (m), 1173 (m, sh), 1070 (m, sh), 1047 (s), 997 (s).

**HR-MS** (ESI^+^): for C_34_H_79_N_6_O_6_P_2_ (M + H)^+^*m*/*z* calculated 729.55308, found 729.55290.

#### Octane-1,8-diyl Dipentyl Bis((2-(bis(2-aminoethyl)amino)ethyl)phosphonate) Hexahydrochloride (**58**)

The title compound was prepared according to general methods **A1**, **B2**, **C**, **D**, and **E** from mono methyl vinylphosphonate (0.53 g, 3.86 mmol) in 13% overall yield (0.45 g, 0.51 mmol) as a white solid.

^1^H NMR (401 MHz, CD_3_OD): 4.20–4.07 (m, 8H, C*H*_2_O), 3.36–3.13 (m, 20H, C*H*_2_NC*H*_2_C*H*_2_NH_2_), 2.44–2.29 (m, 4H, PC*H*_2_), 1.79–1.70 (m, 8H, C*H*_2_CH_2_O), 1.50–1.37 (m, 16H, (C*H*_2_)_2_(CH_2_)_2_O), 1.00–0.93 (m, 6H, C*H*_3_).

^13^C NMR (101 MHz, CD_3_OD): 67.90 (d, *J* = 6.6 Hz, *C*H_2_O), 51.50 (*C*H_2_CH_2_NH_2_), 37.03 (*C*H_2_NH_2_), 31.57 (d, *J* = 6.1 Hz, CH_3_(CH_2_)_2_*C*H_2_), 31.30 (d, *J* = 5.9 Hz, OCH_2_*C*H_2_(CH_2_)_4_*C*H_2_CH_2_O), 30.18, 28.83, 26.59, 23.31 ((*C*H_2_)_2_(CH_2_)_2_O), 22.30 (d, *J* = 138.7 Hz, P*C*H_2_), 14.35 (*C*H_3_).

^31^P{^1^H} NMR (162 MHz, CD_3_OD): 31.93.

**IR** ν_max_ 3000 (s, br), 2956 (vs), 2932 (vs), 2870 (s), 2859 (s), 2640 (m, br), 2543 (m, br), 2005 (w, br), 1666 (vs), 1594 (w), 1566 (w, sh), 1467 (m), 1390 (w), 1227 (m), 1017 (s), 1000 (s).

**HR-MS** (ESI^+^): for C_30_H_70_O_6_N_6_NaP_2_ (M + Na)^+^*m*/*z* calculated 695.47243, found 695.47269.

#### Dihexyl Octane-1,8-diyl Bis((2-(bis(3-aminopropyl)amino)ethyl)phosphonate) Hexahydrochloride (**59**)

The title compound was prepared according to general methods **A1**, **B2**, **C**, **D**, and **E** from mono methyl vinylphosphonate (1.10 g, 5.45 mmol) in 17% overall yield (0.88 g, 0.90 mmol) as a white solid.

^1^H NMR (400 MHz, CD_3_OD): 4.20–4.07 (m, 8H, C*H*_2_O), 3.50–3.42 (m, 4H, PCH_2_C*H*_2_), 3.42–3.34 (m, 8H, C*H*_2_(CH_2_)_2_NH_2_), 3.11 (t, 8H, *J* = 7.5 Hz, C*H*_2_NH_2_), 2.62–2.45 (m, 4H, PC*H*_2_), 2.28–2.14 (m, 8H, C*H*_2_CH_2_NH_2_), 1.81–1.65 (m, 8H, C*H*_2_CH_2_O), 1.50–1.28 (m, 20H, CH_3_C*H*_2_, (C*H*_2_)_2_(CH_2_)_2_O), 0.98–0.88 (m, 6H, C*H*_3_).

^13^C NMR (101 MHz, CD_3_OD): 68.24 (d, *J* = 6.8 Hz), 68.22 (d, *J* = 6.8 Hz, *C*H_2_O), 51.08 (*C*H_2_(CH_2_)_2_NH_2_), 48.82 (PCH_2_*C*H_2_), 37.89 (*C*H_2_NH_2_), 32.51 (CH_3_CH_2_*C*H_2_), 31.57 (d, *J* = 6.0 Hz, *C*H_2_CH_2_O), 30.18, 26.56, 26.31, 23.63 (CH_3_*C*H_2_), 23.29 (*C*H_2_CH_2_NH_2_), 21.27 (d, *J* = 140.1 Hz, P*C*H_2_), 14.38 (*C*H_3_).

^31^P{^1^H} NMR (162 MHz, CD_3_OD): 28.45.

**IR** ν_max_ (KBr) 2966 (m), 2931 (m), 2872 (m), 2623 (w), 2553 (w), 1470 (m), 1384 (w), 1236 (w), 1050 (m), 1000 (m).

**HR-MS** (ESI^+^): for C_36_H_84_N_6_O_6_P_2_ (M + 2H)^2+^*m*/2*z* calculated 379.29583, found 379.29553.

#### Dihexyl Octane-1,8-diyl Bis((2-(bis(3-guanidinopropyl)amino)ethyl)phosphonate) Hexahydrochloride (**60**)

The title compound was prepared according to general method F from **59** (0.20 g, 0.21 mmol) in 84% yield (0.20 g, 0.17 mmol) as a white solid.

^1^H NMR (401 MHz, CD_3_OD): 4.21–4.06 (m, 8H, C*H*_2_O), 3.52–3.39 (m, 4H, PCH_2_C*H*_2_), 3.39–3.32 (m, 16H, C*H*_2_CH_2_C*H*_2_NH), 2.60–2.43 (m, 4H, PC*H*_2_), 2.18–2.02 (m, 8H, C*H*_2_CH_2_NH), 1.79–1.66 (m, 8H, C*H*_2_CH_2_O), 1.50–1.26 (m, 20H, CH_3_C*H*_2_, (C*H*_2_)_2_(CH_2_)_2_O), 0.98–0.86 (m, 6H, C*H*_3_).

^13^C NMR (101 MHz, CD_3_OD): 158.68 (*C*=NH), 68.21 (d, *J* = 6.8 Hz, *C*H_2_O), 51.58 (*C*H_2_NH), 48.63 (PCH_2_C*H*_2_), 39.67 (*C*H_2_(CH_2_)_2_NH), 32.50 (CH_3_CH_2_*C*H_2_), 31.57 (d, *J* = 6.1 Hz), 31.55 (d, *J* = 6.0 Hz, *C*H_2_CH_2_O), 30.20, 26.58, 26.32, 24.78 (*C*H_2_CH_2_NH_2_), 23.64 (CH_3_*C*H_2_), 21.25 (d, *J* = 140.6 Hz, P*C*H_2_), 14.40 (*C*H_3_).

^31^P{^1^H} NMR (162 MHz, CD_3_OD): 28.80.

**IR** ν_max_ (KBr) 3316 (s), 3260 (s), 3147 (s), 2955 (m), 2932 (m), 2858 (m), 1667 (vs), 1646 (s), 1619 (s, sh), 1467 (m), 1376 (w), 1218 (m), 1070–1000 (s), 723 (vw).

**HR-MS** (ESI^+^): for C_40_H_92_N_14_O_6_P_2_ (M + 2H)^2+^*m*/*z* calculated 463.33943, found 463.33887.

#### Dihexyl Octane-1,8-diyl Bis((2-(bis(2-aminoethyl)amino)ethyl)phosphonate) Hexahydrochloride (**61**)

The title compound was prepared according to general methods **A1**, **B2**, **C**, **D**, and **E** from mono methyl vinylphosphonate (1.26 g, 9.25 mmol) in 9% overall yield (0.79 g, 0.86 mmol) as a white solid.

^1^H NMR (401 MHz, CD_3_OD): 4.14–4.03 (m, 8H, C*H*_2_O), 3.22–3.11 (m, 8H, C*H*_2_NH_2_), 3.05–2.88 (m, 12H, C*H*_2_N), 2.28–2.14 (m, 4H, PC*H*_2_), 1.79–1.64 (m, 8H, C*H*_2_CH_2_O), 1.46–1.23 (m, 20H, CH_3_C*H*_2_, (C*H*_2_)_2_(CH_2_)_2_O), 0.99–0.88 (m, 6H, C*H*_3_).

^13^C NMR (101 MHz, CD_3_OD): 67.73 (d, *J* = 7.0 Hz), 67.72 (d, *J* = 6.7 Hz, *C*H_2_O), 51.52 (*C*H_2_CH_2_NH_2_), 47.66 (PCH_2_*C*H_2_), 37.76 (*C*H_2_NH_2_), 31.62 (d, *J* = 6.0 Hz), 31.58 (d, *J* = 6.1 Hz, *C*H_2_CH_2_O), 32.53, 30.27, 26.64, 26.36, 23.65 (CH_3_*C*H_2_, (*C*H_2_)_2_(CH_2_)_2_O), 22.71 (d, *J* = 139.2 Hz, P*C*H_2_), 14.39 (*C*H_3_).

^31^P{^1^H} NMR (162 MHz, CD_3_OD): 31.90.

**IR** ν_max_ 2926 (m), 2793 (s), 2677 (m), 2617 (m), 2542 (s), 2438 (m), 1514 (m), 1467 (m), 1390 (m), 1228 (s), 1050 (s), 990 (s).

**HR-MS** (ESI^+^): for C_32_H_76_O_6_N_6_P_2_ (M + 2H)^2+^*m*/2*z* calculated 351.26453, found 351.26459.

#### Bis((*Z*)-hept-3-en-1-yl) Octane-1,8-diyl Bis((2-(bis(3-aminopropyl)amino)ethyl)phosphonate) Hexahydrochloride (**62**)

The title compound was prepared according to general methods **A1**, **B2**, **C**, **D**, and **E** from mono methyl vinylphosphonate (0.40 g, 2.92 mmol) in 19% overall yield (0.54 g, 0.54 mmol) as a white solid.

^1^H NMR (401 MHz, CD_3_OD): 5.63–5.51 (m, 2H, CH_3_(CH_2_)_2_C*H*), 5.49–5.39 (m, 2H, C*H*(CH_2_)_2_O), 4.20–4.05 (m, 8H, C*H*_2_O), 3.52–3.34 (m, 12H, C*H*_2_N), 3.10 (t, 8H, *J* = 7.5 Hz, C*H*_2_NH_2_), 2.63–2.42 (m, 8H, CHC*H*_2_CH_2_O, PC*H*_2_), 2.27–2.13 (m, 8H, C*H*_2_CH_2_NH_2_), 2.12–2.02 (m, 4H, CH_3_CH_2_C*H*_2_), 1.73 (p, 4H, *J* = 6.8 Hz, CH_2_C*H*_2_CH_2_O), 1.49–1.33 (m, 12H, CH_3_C*H*_2_, (C*H*_2_)_2_(CH_2_)_2_O), 0.93 (t, 6H, *J* = 7.4 Hz, C*H*_3_).

^13^C NMR (101 MHz, CD_3_OD): 134.15 (CH_3_(CH_2_)_2_*C*H), 125.18 (*C*H(CH_2_)_2_O), 68.23 (d, *J* = 6.8 Hz, (CH_2_)_2_*C*H_2_O), 67.61 (d, *J* = 6.9 Hz, CHCH_2_*C*H_2_O), 51.06 (*C*H_2_**(**CH_2_)_2_NH_2_), 48.83 (PCH_2_*C*H_2_), 37.87 (*C*H_2_NH_2_), 31.55 (d, *J* = 6.0 Hz, CH_2_*C*H_2_CH_2_O), 30.45 (CH_3_CH_2_*C*H_2_), 30.18 (d, *J* = 1.2 Hz, *C*H_2_(CH_2_)_2_O), 29.67 (d, *J* = 5.9 Hz, CH*C*H_2_CH_2_O), 26.55 (*C*H_2_(CH_2_)_3_O), 23.79 (CH_3_*C*H_2_), 23.28 (*C*H_2_CH_2_NH), 21.25 (d, *J* = 140.5 Hz, P*C*H_2_), 14.15 (*C*H_3_).

^31^P{^1^H} NMR (162 MHz, CD_3_OD): 28.67.

**IR** ν_max_ (KBr) 3100–2500 (vs, br), 3007 (s, sh), 2957 (vs), 2932 (s), 2871 (s), 2028 (w), 1657 (vw), 1605 (w), 1466 (m), 1228 (m), 1004 (s).

**HR-MS** (ESI^+^): for C_38_H_84_O_6_N_6_P_2_ (M + 2H)^2+^*m*/2*z* calculated 391.29583, found 391.29558.

#### Octane-1,8-diyl Dioctyl Bis((2-(bis(3-aminopropyl)amino)ethyl)phosphonate) Hexahydrochloride (**63**)

The title compound was prepared according to general methods **A1**, **B2**, **C**, **D**, and **E** from mono methyl vinylphosphonate (0.90 g, 3.65 mmol) in 19% overall yield (0.64 g, 0.69 mmol) as a white solid.

^1^H NMR (400 MHz, CD_3_OD): 4.16–4.10 (C*H*_2_O), 3.49–3.45 (m, 4H, PCH_2_C*H*_2_), 3.41–3.36 (m, 8H, C*H*_2_(CH_2_)_2_NH_2_), 3.11 (t, 8H, *J* = 7.6 Hz, C*H*_2_NH_2_), 2.57–2.48 (m, 4H, PC*H*_2_), 2.25–2.16 (m, 8H, C*H*_2_CH_2_NH_2_), 1.77–1.61 (m, 8H, C*H*_2_CH_2_O), 1.42–1.30 (m, 28H, (C*H*_2_)_2_(CH_2_)_2_O, CH_3_(C*H*_2_)_3_), 0.92–0.89 (m, 6H, C*H*_3_).

^13^C NMR (101 MHz, CD_3_OD): 68.28 (d, *J* = 6.9 Hz), 68.25 (d, *J* = 6.8 Hz, *C*H_2_O), 51.07 (*C*H_2_(CH_2_)_2_NH_2_), 48.94 (PCH_2_*C*H_2_), 37.84 (*C*H_2_NH_2_), 32.94 (CH_3_CH_2_*C*H_2_), 31.57 (d, *J* = 5.8 Hz), 31.54 (d, *J* = 6.0 Hz, *C*H_2_CH_2_O), 30.32, 30.22, 30.17 (d, *J* = 1.7 Hz), 26.60, 26.54, 23.68 (CH_3_*C*H_2_), 23.24 (*C*H_2_CH_2_NH_2_), 21.25 (d, *J* = 139.8 Hz, P*C*H_2_), 14.45 (*C*H_3_).

^31^P{^1^H} NMR (162 MHz, CD_3_OD): 28.52.

**IR** ν_max_ (KBr) 3000–2500 (vs, vbr), 2955 (s), 2925 (vs), 2855 (s), 2025 (w), 1599 (w), 1467 (m), 1378 (w), 1227 (m), 1015 (m), 996 (m), 721 (vw).

**HR-MS** (ESI^+^): for C_40_H_92_N_6_O_6_P_2_ (M + 2H)^2+^*m*/2*z* calculated 407.32713, found 407.32687.

#### Octane-1,8-diyl Dioctyl Bis((2-(bis(2-aminoethyl)amino)ethyl)phosphonate) Hexahydrochloride (**64**)

The title compound was prepared according to general methods **A1**, **B2**, **C**, **D**, and **E** from mono methyl vinylphosphonate (0.55 g, 4.03 mmol) in 12% overall yield (0.47 g, 0.48 mmol) as a white solid.

^1^H NMR (401 MHz, CD_3_OD): 4.16–4.05 (m, 8H, C*H*_2_O), 3.29–3.23 (m, 8H, C*H*_2_NH_2_), 3.23–3.05 (m, 12H, C*H*_2_N), 2.40–2.25 (m, 4H, PC*H*_2_), 1.77–1.66 (m, 8H, C*H*_2_CH_2_O), 1.49–1.27 (m, 28H, CH_3_(C*H*_2_)_3_, (C*H*_2_)_2_(CH_2_)_2_O), 0.94–0.87 (m, 6H, C*H*_3_).

^13^C NMR (101 MHz, CD_3_OD): 67.89 (d, *J* = 6.8 Hz), 67.86 (d, *J* = 6.8 Hz, *C*H_2_O), 51.50 (*C*H_2_CH_2_NH_2_), 48.24 (d, *J* = 4.6 Hz, PCH_2_*C*H_2_), 36.84 (*C*H_2_NH_2_), 32.99 (CH_3_CH_2_*C*H_2_), 31.61 (d, *J* = 5.9 Hz), 31.59 (d, *J* = 6.0 Hz, *C*H_2_CH_2_O), 30.37, 30.28, 30.22, 26.67, 26.62, 23.72 (CH_3_*C*H_2_), 22.34 (d, *J* = 139.8 Hz, P*C*H_2_), 14.46 *C*H_3_).

^31^P{^1^H} NMR (162 MHz, CD_3_OD): 32.43.

**IR** ν_max_ (KBr) 3300–2500 (s, br), 2956 (s), 2928 (s), 2856 (s), 1591 (m), 1468 (m), 1394 (w), 1378 (w), 1209 (m), 1071 (m), 1014 (s), 724 (w).

**HR-MS** (ESI^+^): for C_36_H_84_N_6_O_6_P_2_ (M + 2H)^2+^*m*/2*z* calculated 379.29583, found 379.29571.

#### Octane-1,8-diyl Diphenethyl Bis((2-(bis(3-aminopropyl)amino)ethyl)phosphonate) Hexahydrochloride (**65**)

The title compound was prepared according to general methods **A1**, **C**, **C**, **D**, and **E** from mono methyl vinylphosphonate (0.65 g, 5.33 mmol) in 3% overall yield (0.16 g, 0.16 mmol) as a white amorphous solid.

^1^H NMR (401 MHz, CD_3_OD) δ 7.36–7.22 (m, 10H, *Ph*), 4.39–4.31 (m, 4H, OC*H*_2_CH_2_Ph), 4.06–3.90 (m, 4H, OC*H*_2_(CH_2_)_3_), 3.39–3.31 (m, 12H, NC*H*_2_), 3.09 (t, *J* = 7.5 Hz, 8H, C*H*_2_NH_2_), 3.03 (t, *J* = 6.6 Hz, 4H, C*H*_2_Ph), 2.52–2.40 (m, 4H, PC*H*_2_), 2.17 (ddd, *J* = 15.6, 9.1, 6.5 Hz, 8H, C*H*_2_CH_2_NH_2_), 1.64 (p, *J* = 6.7 Hz, 4H, OCH_2_C*H*_2_CH_2_), 1.39–1.28 (m, 8H, O(CH_2_)_2_(C*H*_2_)_2_).

^13^C NMR (101 MHz, CD_3_OD) δ 138.84 (*C*_quat_), 130.26 (*C*_ortho_), 129.70 (*C*_meta_), 127.89 (*C*_para_), 68.60 (d, *J* = 6.8 Hz, O*C*H_2_CH_2_Ph), 68.07 (d, *J* = 6.9 Hz, O*C*H_2_(CH_2_)_3_), 51.02 (*C*H_2_(CH_2_)_2_NH_2_), 48.66 (d, *J* = 1.7 Hz, PCH_2_*C*H_2_), 37.85 (*C*H_2_NH_2_), 37.72 (d, *J* = 6.3 Hz, *C*H_2_Ph), 31.45 (d, *J* = 6.1 Hz, OCH_2_*C*H_2_CH_2_), 30.09 (d, *J* = 1.5 Hz, O(CH_2_)_2_*C*H_2_), 26.45 (O(CH_2_)_3_*C*H_2_), 23.24 (*C*H_2_CH_2_NH_2_), 21.16 (d, *J* = 140.6 Hz, P*C*H_2_).

^31^P{^1^H} NMR (162 MHz, CD_3_OD) δ 28.50.

**IR** ν_max_ (KBr) 2960 (vs, sh), 2935 (vs), 2859 (vs), 2742 (s, sh), 2633 (s, br), 2557 (m), 2019 (w, vbr), 1604 (m), 1522 (m, sh), 1497 (m), 1468 (s), 1454 (s), 1405 (w), 1394 (w), 1258 (m, sh), 1227 (s), 1156 (w), 1060 (s), 1010 (vs), 970 (s, sh), 905 (w), 752 (m), 728 (w, sh), 701 (m), 574 (w), 491 (w).

**HR-MS** (ESI^+^): for C_40_H_75_N_6_O_6_P_2_ (M + H)^+^*m*/*z* calculated 797.52178, found 797.52232.

#### Decane-1,10-diyl Diisobutyl Bis((2-(bis(3-aminopropyl)amino)ethyl)phosphonate) Hexahydrochloride (**66**)

The title compound was prepared according to general methods **A1**, **B1**, **C**, **D**, and **E** from mono methyl vinylphosphonate (330 mg, 2.71 mmol) in 10% overall yield (258 mg, 0.272 mmol) as a white solid.

^1^H NMR (401 MHz, CD_3_OD) δ 4.14 (q, *J* = 6.7 Hz, 4H, OC*H*_2_CH_2_), 3.90 (tt, *J* = 6.1, 3.2 Hz, 4H, OC*H*_2_CH), 3.51–3.43 (m, 4H, PCH_2_C*H*_2_), 3.43–3.35 (m, 8H, C*H*_2_(CH_2_)_2_NH_2_), 3.11 (t, *J* = 7.5 Hz, 8H, C*H*_2_NH_2_), 2.74–2.47 (m, 4H, PC*H*_2_), 2.21 (h, *J* = 6.4 Hz, 8H, C*H*_2_CH_2_NH_2_), 1.98 (dt, *J* = 13.3, 6.6 Hz, 2H, OCH_2_C*H*), 1.83–1.65 (m, 4H, OCH_2_C*H*_2_), 1.53–1.27 (m, 12H, O(CH_2_)_2_(C*H*_2_)_3_), 0.99 (d, *J* = 6.7 Hz, 12H, C*H*_3_).

^13^C NMR (101 MHz, CD_3_OD) δ 73.92 (d, *J* = 7.1 Hz, O*C*H_2_CH), 68.29 (d, *J* = 6.7 Hz, O*C*H_2_CH_2_), 51.09 (*C*H_2_(CH_2_)_2_NH_2_), 37.86 (*C*H_2_NH_2_), 31.63 (d, *J* = 5.9 Hz, OCH_2_*C*H_2_), 30.65, 30.47 (d, *J* = 6.4 Hz, OCH_2_*C*H), 30.30, 26.63 (O(CH_2_)_2_(*C*H_2_)_2_), 23.33 (*C*H_2_CH_2_NH_2_), 21.16 (d, *J* = 139.8 Hz, P*C*H_2_), 19.02 (*C*H_3_).

^31^P{^1^H} NMR (162 MHz, CD_3_OD) δ 28.59.

**IR** ν_max_ (KBr) 2961 (s), 2929 (s), 2855 (m), 3200–2700 (vs, vbr), 2700–2500 (m, vbr), 1610 (m), 1513 (m), 1468 (s), 1401 (m), 1369 (m), 1227 (vs), 1005 (vs), ∼869 (m, sh), 851 (m), 767 (m), 725 (w).

**HR-MS** (ESI^+^): for C_34_H_79_O_6_N_6_P_2_ (M + H)^+^*m*/*z* calculated 729.55308, found 729.55268.

#### Dibutyl Decane-1,10-diyl Bis((2-(bis(3-aminopropyl)amino)ethyl)phosphonate) Hexahydrochloride (**67**)

The title compound was prepared according to general methods **A1**, **B2**, **C**, **D**, and **E** from mono methyl vinylphosphonate (0.76 g, 5.58 mmol) in 12% overall yield (0.63 g, 0.67 mmol) as a white solid.

^1^H NMR (401 MHz, CD_3_OD) δ 4.17–4.10 (m, 8H, C*H*_2_O), 3.50–3.42 (m, 4H, PCH_2_C*H*_2_), 3.39 (dd, *J* = 10.2, 6.3 Hz, 8H, C*H*_2_(CH_2_)_2_NH_2_), 3.10 (t, *J* = 7.5 Hz, 8H, C*H*_2_NH_2_), 2.60–2.46 (m, 4H, PC*H*_2_), 2.28–2.14 (m, 8H, C*H*_2_CH_2_NH_2_), 1.78–1.66 (m, 8H, C*H*_2_CH_2_O), 1.51–1.31 (m, 16H, CH_3_C*H*_2_, (C*H*_2_)_3_(CH_2_)_2_O), 0.98 (t, *J* = 7.4 Hz, 6H, C*H*_3_).

^13^C NMR (101 MHz, CD_3_OD) δ 68.24 (d, *J* = 6.8 Hz), 67.90 (d, *J* = 6.7 Hz, *C*H_2_O), 51.05 (*C*H_2_(CH_2_)_2_NH_2_), 48.81 (PCH_2_*C*H_2_), 37.86 (*C*H_2_NH_2_), 33.61 (d, *J* = 6.0 Hz), 31.60 (d, *J* = 5.8 Hz, *C*H_2_CH_2_O), 30.62, 30.28, 26.61 ((*C*H_2_)_3_(CH_2_)_2_O), 23.27 (*C*H_2_CH_2_NH_2_), 21.23 (d, *J* = 140.6 Hz, P*C*H_2_), 19.80 (CH_3_*C*H_2_), 13.96 (*C*H_3_).

^31^P{^1^H} NMR (162 MHz, CD_3_OD) δ 28.66.

**IR** ν_max_ (KBr) 2932 (vs), 2632 (m), 2558 (m), 1598 (m), 1467 (m), 1224 (m), 1172 (m sh), 1070 (m, sh), 1063 (m), 1018 (vs), 997 (m, sh).

**HR-MS** (ESI^+^): for C_34_H_79_N_6_O_6_P_2_ (M + H)^+^*m*/*z* calculated 729.55308, found 729.55255.

#### Dibutyl Decane-1,10-diyl Bis((2-(bis(2-aminoethyl)amino)ethyl)phosphonate) Hexahydrochloride (**68**)

The title compound was prepared according to general methods **A1**, **B2**, **C**, **D**, and **E** from mono methyl vinylphosphonate (0.84 g, 6.19 mmol) in 8% overall yield (0.40 g, 0.49 mmol) as a white solid.

^1^H NMR (400 MHz, CD_3_OD): 4.17–4.04 (m, 8H, C*H*_2_O), 3.29–3.20 (m, 8H, C*H*_2_NH_2_), 3.17–3.04 (m, 12H, C*H*_2_N), 2.36–2.22 (m, 4H, PC*H*_2_), 1.78–1.63 (m, 8H, C*H*_2_CH_2_O), 1.52–1.30 (m, 16H, CH_3_C*H*_2_, (C*H*_2_)_3_(CH_2_)_2_O), 0.98 (t, 6H, *J* = 7.4 Hz, C*H*_3_).

^13^C NMR (101 MHz, CD_3_OD): 67.96 (d, *J* = 6.8 Hz), 67.64 (d, *J* = 6.7 Hz, *C*H_2_O), 51.50 (*C*H_2_CH_2_NH_2_), 48.49 (PCH_2_*C*H_2_), 36.81 (*C*H_2_NH_2_), 33.62 (d, *J* = 6.1 Hz), 31.59 (d, *J* = 6.1 Hz, *C*H_2_CH_2_O), 30.59, 30.26, 26.63 ((*C*H_2_)_3_(CH_2_)_2_O), 20.81 (d, *J* = 139.2 Hz, P*C*H_2_), 19.82 (CH_3_*C*H_2_), 13.96 (*C*H_3_).

^31^P{^1^H} NMR (162 MHz, CD_3_OD): 28.44.

**IR***ν*_max_ (KBr) 2961 (m), 2926 (m), 2674 (m, sh), 2551 (m, br), 1613, (m), 1467 (m), 1391 (m), 1260 (m), 1236 (m), 1066 (m, sh), 991 (m, sh).

**HR-MS** (ESI^+^): for C_30_H_71_N_6_O_6_P_2_ (M + H)^+^*m*/*z* calculated 673.49048, found 673.49047.

#### Decane-1,10-diyl Dipentyl Bis((2-(bis(3-aminopropyl)amino)ethyl)phosphonate) Hexahydrochloride (**69**)

The title compound was prepared according to general methods **A1**, **B2**, **C**, **D**, and **E** from mono methyl vinylphosphonate (0.74 g, 5.40 mmol) in 14% overall yield (0.73 g, 0.75 mmol) as a white solid.

^1^H NMR (400 MHz, CD_3_OD): 4.19–4.07 (m, 8H, C*H*_2_O), 3.49–3.42 (m, 4H, PCH_2_C*H*_2_), 3.42–3.35 (m, 8H, C*H*_2_(CH_2_)_2_NH_2_), 3.10 (t, 8H, *J* = 7.5 Hz, C*H*_2_NH_2_), 2.61–2.45 (m, 4H, PC*H*_2_), 2.28–2.14 (m, 8H, C*H*_2_CH_2_NH_2_), 1.79–1.66 (m, 8H, C*H*_2_CH_2_O), 1.48–1.30 (m, 20H, CH_3_(C*H*_2_)_2_, (C*H*_2_)_3_(CH_2_)_2_O), 1.00–0.89 (m, 6H, C*H*_3_).

^13^C NMR (101 MHz, CD_3_OD): 69.20 (d, *J* = 6.8 Hz), 68.27 (d, *J* = 6.8 Hz, *C*H_2_O), 51.07 (*C*H_2_(CH_2_)_2_NH_2_), 48.83 (PCH_2_*C*H_2_), 37.87 (*C*H_2_NH_2_), 31.61 (d, *J* = 5.9 Hz), 31.29 (d, *J* = 5.9 Hz, *C*H_2_CH_2_O), 30.63, 30.29, 28.80, 26.62, 23.30 (CH_3_(*C*H_2_)_2_, (*C*H_2_)_3_(CH_2_)_2_O), 23.27 (*C*H_2_CH_2_NH_2_), 21.25 (d, *J* = 140.5 Hz, P*C*H_2_), 14.35 (*C*H_3_).

^31^P{^1^H} NMR (162 MHz, CD_3_OD): 28.41.

**IR** ν_max_ (KBr) 2929 (s), 2831 (m, sh), 2677–2543 (m), 1611 (m), 1514 (m), 1467 (m), 1390 (m), 1263 (m), 1228 (s), 1077–991 (s).

**HR-MS** (ESI^+^): for C_36_H_83_N_6_O_6_P_2_ (M + H)^+^*m*/*z* calculated 379.29583, found 379.29576.

#### Decane-1,10-diyl Dihexyl Bis((2-(bis(3-aminopropyl)amino)ethyl)phosphonate) Hexahydrochloride (**70**)

The title compound was prepared according to general methods **A1**, **B2**, **C**, **D**, and **E** from mono methyl vinylphosphonate (0.61 g, 4.45 mmol) in 17% overall yield (0.70 g, 0.76 mmol) as a white solid.

^1^H NMR (400 MHz, CD_3_OD): 4.14 (t, *J* = 6.6 Hz, 4H), 4.12 (t, *J* = 6.5 Hz, 4H, C*H*_2_O), 3.50–3.41 (m, 4H, PCH_2_C*H*_2_), 3.41–3.33 (m, 8H, C*H*_2_(CH_2_)_2_NH_2_), 3.10 (t, 8H, *J* = 7.5 Hz, C*H*_2_NH_2_), 2.59–2.44 (m, 4H, PC*H*_2_), 2.27–2.13 (m, 8H, C*H*_2_CH_2_NH_2_), 1.78–1.67 (m, 8H, C*H*_2_CH_2_O), 1.49–1.29 (m, 24H, (C*H*_2_)_3_(CH_2_)_2_O), 0.97–0.88 (m, 6H, C*H*_3_).

^13^C NMR (101 MHz, CD_3_OD): 66.85 (d, *J* = 7.2 Hz, *C*H_2_O), 51.08 (*C*H_2_(CH_2_)_2_NH_2_), 48.83 (PCH_2_*C*H_2_), 37.88 (*C*H_2_NH_2_)_,_ 32.51 (CH_3_CH_2_*C*H_2_), 31.63 (d, *J* = 5.6 Hz), 31.57 (d, *J* = 5.7 Hz, *C*H_2_CH_2_O), 30.66, 30.31, 26.64, 26.31, 23.64 (CH_3_*C*H_2_), 23.31 (*C*H_2_CH_2_NH_2_), 21.24 (d, *J* = 140.6 Hz, P*C*H_2_), 14.38 (*C*H_3_).

^31^P{^1^H} NMR (162 MHz, CD_3_OD): 28.45.

**IR***ν*_max_ (KBr) 2974 (m), 2927 (w), 2895 (w), 2635 (w), 2510 (w), 1601 (w, sh), 1383 (w), 1273 (w), 1089 (m), 1050 (m).

**HR-MS** (ESI^+^): for C_38_H_87_N_6_O_6_P_2_ (M + H)^+^*m*/*z* calculated 785.61568, found 785.61479.

#### Decane-1,10-diyl Dioctyl Bis((2-(bis(3-aminopropyl)amino)ethyl)phosphonate) Hexahydrochloride (**71**)

The title compound was prepared according to general methods **A1**, **B2**, **C**, **D**, and **E** from mono methyl vinylphosphonate (0.66 g, 5.45 mmol) in 19% overall yield (1.1 g, 1.04 mmol) as a white amorphous solid.

^1^H NMR (500.2 MHz, CD_3_OD): 4.16–4.10 (m, 8H, C*H*_2_O), 3.49–3.43 (m, 4H, PCH_2_C*H*_2_), 3.43–3.36 (m, 8H, C*H*_2_(CH_2_)_2_NH_2_), 3.11 (t, 8H, *J* = 7.5 Hz, C*H*_2_NH_2_), 2.59–2.49 (m, 4H, PC*H*_2_), 2.26–2.17 (m, 8H, C*H*_2_CH_2_NH_2_), 1.76–1.68 (m, 8H, C*H*_2_CH_2_O), 1.46–1.26 (m, 32H, CH_3_(C*H*_2_)_2_, (C*H*_2_)_3_(CH_2_)_2_O), 0.93–0.88 (m, 6H, C*H*_3_).

^13^C NMR (125.8 MHz, CD_3_OD): 68.23 (d, *J* = 6.7 Hz, *C*H_2_O), 51.04 (*C*H_2_(CH_2_)_2_NH_2_), 48.85 (PCH_2_*C*H_2_), 37.88 (*C*H_2_NH_2_), 32.96 (CH_3_CH_2_*C*H_2_), 31.59, 31.58 (d, *J* = 5.8 Hz, *C*H_2_CH_2_O), 30.62, 30.34, 30.29, 30.24, 26.64, 26.62 ((*C*H_2_)_3_(CH_2_)_2_O), 23.69 (CH_3_*C*H_2_), 23.22 (*C*H_2_CH_2_NH_2_), 21.28 (d, *J* = 140.2 Hz, P*C*H_2_), 14.45 (*C*H_3_).

^31^P{^1^H} NMR (202.5 MHz, CD_3_OD): 26.64.

**IR** ν_max_ (KBr) 2956 (s), 2925 (vs), 2675 (m), 2620 (m), 2546 (m), 2055 (w, br), 1626 (w), 1608 (w, sh), 1543 (w), 1511 (w), 1468 (m), 1460 (m), 1401 (w), 1390 (w), 1378 (w, sh), 1338 (vw), 1305 (vw), 1263 (m), 1227 (s), 1075 (m), 1022 (s, sh), 996 (s), 722 (w).

**HR-MS** (ESI^+^): for C_42_H_96_N_6_O_6_P_2_ (M + 2H)^2+^*m*/2*z* calculated 421.34278, found 421.34273.

#### Decane-1,10-diyl Diphenethyl Bis((2-(bis(3-aminopropyl)amino)ethyl)phosphonate) Hexahydrochloride (**72**)

The title compound was prepared according to general methods **A1**, **C**, **C**, **D**, and **E** from mono methyl vinylphosphonate (1.3 g, 11 mmol) in 3% overall yield (0.34 g, 0.33 mmol) as a white amorphous solid.

^1^H NMR (401 MHz, CD_3_OD) δ 7.41–7.21 (m, 10H, Ph), 4.43–4.28 (m, 4H, C*H*_2_CH_2_Ph), 3.97 (dddd, *J* = 19.6, 10.0, 7.5, 3.3 Hz, 4H, OC*H*_2_(CH_2_)_8_C*H*_2_O), 3.38–3.31 (m, 12H, NC*H*_2_), 3.09 (t, *J* = 7.5 Hz, 8H, C*H*_2_NH_2_), 3.03 (t, *J* = 6.6 Hz, 4H, C*H*_2_Ph), 2.57–2.40 (m, 4H, PC*H*_2_), 2.27–2.11 (m, 8H, C*H*_2_CH_2_NH_2_), 1.69–1.58 (m, 4H, OCH_2_C*H*_2_CH_2_), 1.48–1.25 (m, 12H, O(CH_2_)_2_(C*H*_2_)_3_).

^13^C NMR (101 MHz, CD_3_OD) δ 138.83 (*C*_quat_), 130.25(*C*_ortho_), 129.68(*C*_meta_), 127.87(*C*_para_), 68.58 (d, *J* = 6.9 Hz, C*H*_2_CH_2_Ph), 68.08 (d, *J* = 6.9 Hz, O*C*H_2_(CH_2_)_4_), 51.00 (*C*H_2_(CH_2_)_2_NH_2_), 48.67 (PCH_2_*C*H_2_), 37.85 (*C*H_2_NH_2_), 37.71 (d, *J* = 6.2 Hz, *C*H_2_Ph), 31.49 (d, *J* = 5.6 Hz, OCH_2_*C*H_2_CH_2_), 30.57 (O(CH_2_)_2_*C*H_2_), 30.23 (O(CH_2_)_3_*C*H_2_), 26.53 (O(CH_2_)_4_*C*H_2_), 23.21 (*C*H_2_CH_2_NH_2_), 21.16 (d, *J* = 140.5 Hz, P*C*H_2_).

^31^P{^1^H} NMR (162 MHz, CD_3_OD) δ 28.52.

**IR** ν_max_ (KBr) 2965 (vs, sh), 2927 (vs), 2854 (vs), 2745 (s, sh), 2633 (s, br), 2558 (s), 2027 (w, vbr), 1615 (m, sh), 1605 (m), 1520 (m, sh), 1497 (m), 1470 (s), 1454 (s), 1412 (m), 1391 (m), 1256 (m), 1236 (s), 1156 (w), 1062 (s), 1088 (m, sh), 1009 (vs), 967 (s, sh), 902 (m), 750 (m), 725 (w, sh), 700 (m), 574 (w), 495 (m).

**HR-MS** (ESI^+^): for C_42_H_79_N_6_O_6_P_2_ (M + H)^+^*m*/*z* calculated 825.55308, found 825.55369.

#### Decane-1,10-diyl Didecyl Bis((2-(bis(3-aminopropyl)amino)ethyl)phosphonate) Hexahydrochloride (**73**)

The title compound was prepared according to general methods **A1**, **B2**, **C**, **D**, and **E** from mono methyl vinylphosphonate (0.32 g, 2.65 mmol) in 23% overall yield (0.68 g, 0.61 mmol) as a white amorphous solid.

^1^H NMR (500.2 MHz, CD_3_OD): 4.16–4.10 (m, 8H, C*H*_2_O), 3.49–3.42 (m, 4H, PCH_2_C*H*_2_), 3.42–3.36 (m, 8H, C*H*_2_(CH_2_)_2_NH_2_), 3.11 (t, 8H, *J* = 7.5 Hz, C*H*_2_NH_2_), 2.58–2.49 (m, 4H, PC*H*_2_), 2.25–2.16 (m, 8H, C*H*_2_CH_2_NH_2_), 1.76–1.68 (m, 8H, C*H*_2_CH_2_O), 1.46–1.25 (m, 40H, CH_3_(C*H*_2_)_4_, (C*H*_2_)_3_(CH_2_)_2_O), 0.93–0.88 (m, 6H, C*H*_3_).

^13^C NMR (125.8 MHz, CD_3_OD): 68.24 (d, *J* = 6.6 Hz, *C*H_2_O), 51.06 (*C*H_2_(CH_2_)_2_NH_2_), 48.83 (PCH_2_*C*H_2_), 37.87 (*C*H_2_NH_2_), 33.05 (CH_3_CH_2_*C*H_2_), 31.62, 31.59 (d, *J* = 5.8 Hz, CH_3_(CH_2_)_7_*C*H_2_CH_2_O, OCH_2_*C*H_2_(CH_2_)_6_*C*H_2_CH_2_O), 30.69, 30.66, 30.45, 30.31, 30.29, 26.64, 26.62, 23.72 (CH_3_(*C*H_2_)_7_(CH_2_)_2_O, O(CH_2_)_2_(*C*H_2_)_6_(CH_2_)_2_O), 23.26 (NCH_2_*C*H_2_CH_2_NH_2_), 21.26 (d, *J* = 140.3 Hz, P*C*H_2_CH_2_N), 14.46 (*C*H_3_(CH_2_)_8_CH_2_O).

^31^P{^1^H} NMR (202.5 MHz, CD_3_OD): 26.65.

**IR** ν_max_ (KBr) 2956 (vs), 2925 (vs), 2854 (vs), 2675 (m, br), 2620 (m, br), 2546 (s, br), 2059 (w, br), 1628 (m, sh), 1607 (m), 1542 (m), 1510 (m), 1484 (m), 1468 (m), 1456 (m, sh), 1401 (w), 1390 (w), 1377 (w, sh), 1338 (vw), 1305 (w), 1227 (s), 1075 (m), 1022 (s, sh), 992 (s), 722 (w).

**HR-MS** (ESI^+^): for C_46_H_104_N_6_O_6_P_2_ (M + 2H)^2+^*m*/2*z* calculated 449.37408, found 449.37398.

#### Diisobutyl Dodecane-1,12-diyl Bis((2-(bis(3-aminopropyl)amino)ethyl)phosphonate) Hexahydrochloride (**74**)

The title compound was prepared according to general methods **A1**, **B1**, **C**, **D**, and **E** from mono methyl vinylphosphonate (349 mg, 2.85 mmol) in 2% overall yield (43 mg, 43.6 μmol) as a white solid.

^1^H NMR (401 MHz, CD_3_OD) δ 4.15 (dt, *J* = 7.8, 6.6 Hz, 4H, OC*H*_2_CH_2_), 3.92 (td, *J* = 6.6, 2.4 Hz, 4H, OC*H*_2_CH), 3.55–3.42 (m, 4H, PCH_2_C*H*_2_), 3.43–3.34 (m, 8H, C*H*_2_(CH_2_)_2_NH_2_), 3.11 (t, *J* = 7.5 Hz, 8H, C*H*_2_NH_2_), 2.67–2.46 (m, 4H, PC*H*_2_), 2.33–2.15 (m, 8H, C*H*_2_CH_2_NH_2_), 1.99 (dp, *J* = 13.3, 6.7 Hz, 2H, OCH_2_C*H*), 1.86–1.65 (m, 4H, OCH_2_C*H*_2_), 1.53–1.28 (m, 16H, O(CH_2_)_2_(C*H*_2_)_4_), 1.00 (d, *J* = 6.7 Hz, 12H, C*H*_3_).

^13^C NMR (101 MHz, CD_3_OD) δ 73.92 (d, *J* = 6.6 Hz, O*C*H_2_CH), 68.29 (d, *J* = 6.7 Hz, O*C*H_2_CH_2_), 51.09 (*C*H_2_(CH_2_)_2_NH_2_), 37.87 (*C*H_2_NH_2_), 31.63 (d, *J* = 5.5 Hz, OCH_2_*C*H_2_), 30.74, 30.71, 30.47 (d, *J* = 6.4 Hz, OCH_2_*C*H), 30.32, 26.62 (O(CH_2_)_2_(*C*H_2_)_2_), 23.32 (*C*H_2_CH_2_NH_2_), 21.19 (d, *J* = 140.6 Hz, P*C*H_2_), 19.03 (*C*H_3_).

^31^P{^1^H} NMR (162 MHz, CD_3_OD) δ 27.12.

**IR** ν_max_(KBr) 3200–2700 (vs, vbr), 2961 (s), 2927 (s), 2854 (m), 2700–2500 (m), 1607 (m), 1510 (w), 1470 (m), 1400 (w), 1369 (w), 1226 (m), 1002 (s), ∼869 (w, sh), 851 (m), 768 (w), 724 (vw).

**HR-MS** (ESI^+^): for C_36_H_83_O_6_N_6_P_2_ (M + H)^+^*m*/*z* calculated 757.58438, found 757.58375.

#### Dibutyl Dodecane-1,12-diyl Bis((2-(bis(3-aminopropyl)amino)ethyl)phosphonate) Hexahydrochloride (**75**)

The title compound was prepared according to general methods **A1**, **B2**, **C**, **D**, and **E** from mono methyl vinylphosphonate (0.82 g, 6.05 mmol) in 12% overall yield (0.73 g, 0.75 mmol) as a white solid.

^1^H NMR (400 MHz, CD_3_OD): 4.20–4.07 (m, 8H, C*H*_2_O), 3.49–3.41 (m, 4H, PCH_2_C*H*_2_), 3.41–3.35 (m, 8H, C*H*_2_(CH)_2_NH_2_), 3.10 (t, 8H, *J* = 7.5 Hz, C*H*_2_NH_2_), 2.61–2.43 (m, 4H, PC*H*_2_), 2.28–2.10 (m, 8H, C*H*_2_CH_2_NH_2_), 1.80–1.64 (m, 8H, C*H*_2_CH_2_O), 1.53–1.28 (m, 20H, CH_3_C*H*_2_, (C*H*_2_)_4_(CH_2_)_2_O), 0.98 (t, 6H, *J* = 7.4 Hz, C*H*_3_).

^13^C NMR (101 MHz, CD_3_OD): 68.24 (d, *J* = 6.7 Hz), 67.90 (d, *J* = 6.8 Hz *C*H_2_O), 51.05 (*C*H_2_(CH_2_)_2_NH_2_), 48.84 (PCH_2_*C*H_2_), 37.87 (*C*H_2_NH_2_), 33.60 (d, *J* = 6.0 Hz), 31.59 (d, *J* = 6.0 Hz, *C*H_2_CH_2_O), 30.70, 30.67, 30.29, 26.60 ((*C*H_2_)_4_(CH_2_)_2_O), 23.24 (*C*H_2_CH_2_NH_2_), 21.27 (d, *J* = 140.8 Hz, P*C*H_2_), 19.79 (CH_3_*C*H_2_), 13.96 (*C*H_3_).

^31^P{^1^H} NMR (162 MHz, CD_3_OD): 28.35.

**IR** ν_max_ (KBr) 2930 (s), 2780 (m), 2630 (m), 2557 (m), 1597 (m), 1467 (m), 1226 (m), 1168 (m, sh), 1070 (m, sh), 1064 (m), 1020 (s), 1004 (s).

**HR-MS** (ESI^+^): for C_36_H_83_N_6_O_6_P_2_ (M + H)^+^*m*/*z* calculated 757.58438, found 757.58402.

#### Dodecane-1,12-diyl Dipentyl Bis((2-(bis(3-aminopropyl)amino)ethyl)phosphonate) Hexahydrochloride (**76**)

The title compound was prepared according to general methods **A1**, **B2**, **C**, **D**, and **E** from mono methyl vinylphosphonate (0.62 g, 4.56 mmol) in 18% overall yield (0.78 g, 0.84 mmol) as a white solid.

^1^H NMR (400 MHz, CD_3_OD): 4.19–4.08 (m, 8H, C*H*_2_O), 3.49–3.42 (m, 4H, PCH_2_C*H*_2_), 3.42–3.34 (m, 8H, C*H*_2_(CH_2_)_2_NH_2_), 3.10 (t, 8H, *J* = 7.5 Hz, C*H*_2_NH_2_), 2.61–2.45 (m, 4H, PC*H*_2_), 2.28–2.13 (m, 8H, C*H*_2_CH_2_NH_2_), 1.79–1.66 (m, 8H, C*H*_2_CH_2_O), 1.48–1.26 (m, 24H, CH_3_(C*H*_2_)_2_, (C*H*_2_)_4_(CH_2_)_2_O), 0.99–0.89 (m, 6H, C*H*_3_).

^13^C NMR (101 MHz, CD_3_OD): 68.26 (d, *J* = 6.8 Hz), 68.20 (d, *J* = 6.8 Hz, *C*H_2_O), 51.05 (*C*H_2_(CH_2_)_2_NH_2_), 48.86 (PCH_2_*C*H_2_), 37.87 (*C*H_2_NH_2_), 31.60 (d, *J* = 5.8 Hz), 31.29 (d, *J* = 5.9 Hz, *C*H_2_CH_2_O), 30.71, 30.68, 30.30, 28.79, 26.62, 23.29 (CH_3_(*C*H_2_)_2_, (*C*H_2_)_4_(CH_2_)_2_O), 23.24 (*C*H_2_CH_2_NH_2_), 21.27 (d, *J* = 140.3 Hz, P*C*H_2_), 14.35 (*C*H_3_).

^31^P{^1^H} NMR (162 MHz, CD_3_OD): 28.38.

**IR***ν*_max_ (KBr) 2930 (vs), 2631 (m, sh), 2557 (m, sh), 1599 (m), 1467 (m), 1226 (s), 1170 (m, sh), 1065 (m, sh), 1052 (s, sh), 995 (s).

**HR-MS** (ESI^+^): for C_38_H_88_N_6_O_6_P_2_ (M + 2H)^2+^*m*/2*z* calculated 393.31148, found 393.31141.

#### Dodecane-1,12-diyl Dihexyl Bis((2-(bis(3-aminopropyl)amino)ethyl)phosphonate) Hexahydrochloride (**77**)

The title compound was prepared according to general methods **A1**, **B1**, **C**, **D**, and **E** from mono methyl vinylphosphonate (0.6 g, 4.88 mmol) in 17% overall yield (0.8 g, 0.78 mmol) as a white amorphous solid.

^1^H NMR (401 MHz, CD_3_OD) δ 4.13 (dt, *J* = 7.8, 6.6 Hz, 8H, OC*H*_2_), 3.52–3.35 (m, 12H, NC*H*_2_), 3.12 (t, *J* = 7.5 Hz, 8H, C*H*_2_NH_2_), 2.65–2.50 (m, 4H, PC*H*_2_), 2.23 (tt, *J* = 9.0, 6.1 Hz, 8H, C*H*_2_CH_2_NH_2_), 1.71 (dt, *J* = 8.4, 6.5 Hz, 8H, OCH_2_C*H*_2_), 1.47–1.25 (m, 28H, O(CH_2_)_2_(C*H*_2_)_8_(CH_2_)_2_O, O(CH_2_)_2_(C*H*_2_)_3_CH_3_), 0.99–0.86 (m, 6H, C*H*_3_).

^13^C NMR (101 MHz, CD_3_OD) δ 68.18 (d, *J* = 6.8 Hz, O*C*H_2_), 50.95 (*C*H_2_(CH_2_)_2_NH_2_), 48.86 (d, *J* = 2.1 Hz, PCH_2_*C*H_2_), 37.86 (*C*H_2_NH_2_), 32.48, 31.57 (d, *J* = 4.4 Hz, OCH_2_*C*H_2_), 31.52 (d, *J* = 4.5 Hz, OCH_2_*C*H_2_), 30.69, 30.66, 30.28, 26.60 (O(CH_2_)_2_*C*H_2_), 26.28 (O(CH_2_)_2_*C*H_2_), 23.61, 23.13 (*C*H_2_CH_2_NH_2_), 21.27 (d, *J* = 140.1 Hz, P*C*H_2_), 14.40 (*C*H_3_).

^31^P{^1^H} NMR (162 MHz, CD_3_OD) δ 28.64.

**IR** ν_max_ (KBr) 3100–2500 (m-s, vbr), 2960 (m, br, sh), 2923 (m, br), 2855 (m), 2674 (m), 2617 (m), 2545 (m, br), 2040 (m, vbr), 1604 (s), 1542 (m), 1508 (m), 1483 (s), 1468 (s), 1459 (s), 1401 (m), 1379 (m, sh), 1226 (vs), 1074 (s), 1040 (s, sh), 1025 (vs, sh), 995 (vs), 964 (s, sh), 724 (w).

**HR-MS** (ESI^+^): for C_40_H_91_N_6_O_6_P_2_ (M + H)^+^*m*/*z* calculated 813.64698, found 813.64686.

#### Dodecane-1,12-diyl Dioctyl Bis((2-(bis(3-aminopropyl)amino)ethyl)phosphonate) Hexahydrochloride (**78**)

The title compound was prepared according to general methods **A1**, **B2**, **C**, **D**, and **E** from mono methyl vinylphosphonate (1.07 g, 8.77 mmol) in 7% overall yield (0.67 g, 0.61 mmol) as a white amorphous solid.

^1^H NMR (500.2 MHz, CD_3_OD): 4.09–4.16 (m, 8H, C*H*_2_O), 3.41–3.48 (m, 4H, PCH_2_C*H*_2_), 3.34–3.41 m, 8H, C*H*_2_(CH_2_)_2_NH_2_), 3.10 (t, 8H, *J*_vic_ = 7.5, C*H*_2_NH_2_), 2.46–2.57 (m, 4H, PC*H*_2_), 2.15–2.25 (m, 8H, C*H*_2_CH_2_NH_2_), 1.68–1.76 (m, 8H, C*H*_2_CH_2_O), 1.26–1.46 (m, 36H, CH_3_C*H*_2_, O(CH_2_)_2_(C*H*_2_)_4_), 0.88–0.93 (m, 6H, C*H*_3_).

^13^C NMR (125.8 MHz, CD_3_OD): 68.26 (d, *J*_C,P_ = 6.7, *C*H_2_O), 51.08 (*C*H_2_(CH_2_)_2_NH_2_), 48.80 (PCH_2_*C*H_2_), 37.88 (*C*H_2_NH_2_), 32.98 (CH_3_CH_2_*C*H_2_), 31.63 (d, *J*_C,P_ = 5.5), 31.61 (d, *J*_C,P_ = 5.7, *C*H_2_CH_2_O), 30.77 (CH_3_(CH_2_)_5_CH_2_CH_2_O, O(CH_2_)_2_(CH_2_)_4_), 30.74, 30.36, 30.35, 30.26, 26.64, 26.64, 23.71 (CH_3_*C*H_2_), 23.29 (*C*H_2_CH_2_NH_2_), 21.24 (d, *J*_C,P_ = 140.4, P*C*H_2_), 14.45 (*C*H_3_).

^31^P{^1^H} NMR (202.5 MHz, CD_3_OD): 26.67.

**IR** ν_max_ (KBr) 2956 (vs), 2925 (vs), 2854 (vs), 2675 (m), 2643 (m), 2619 (m), 2547 (m), 2059 (w, br), 1623 (m, sh), 1608 (m), 1543 (m), 1510 (m), 1484 (m), 1468 (m), 1460 (m), 1401 (w), 1390 (m), 1378 (w, sh), 1262 (m), 1227 (s), 1075 (m), 1022 (s), 995 (s), 722 (w).

**HR-MS**(ESI^+^) For C_44_H_100_N_6_O_6_P_2_ (M + 2H)^2+^*m*/2*z* calculated 435.35843, found 435.35823.

#### Dodecane-1,12-diyl Diphenethyl Bis((2-(bis(3-aminopropyl)amino)ethyl)phosphonate) Hexahydrochloride (**79**)

The title compound was prepared according to general methods **A1**, **B1**, **C**, **D**, and **E** from mono methyl vinylphosphonate (0.60 g, 4.91 mmol) in 15% overall yield (0.78 g, 0.73 mmol) as a white solid.

^1^H NMR (500.2 MHz, CD_3_OD): 7.37–7.22 (m, 10H, Ph*H*) 4.30–4.40 (m, 4H, OC*H*_2_CH_2_Ph), 3.91–4.03 (m, 4H, OC*H*_2_(CH_2_)_5_), 3.30–3.36 (m, 12H, C*H*_2_(CH_2_)_2_NH_2_, PCH_2_C*H*_2_), 3.09 (t, 8H, *J*_vic_ = 7.5, C*H*_2_NH_2_), 3.03 (t, 4H, *J*_vic_ = 6.6, C*H*_2_Ph), 2.41–2.51 (m, 4H, PC*H*_2_) , 2.13–2.22 (m, 8H, C*H*_2_CH_2_NH_2_), 1.59–1.67 (m, 4H, OCH_2_C*H*_2_CH_2_), 1.28–1.37 (m, 16H, O(CH_2_)_2_(C*H*_2_)_4_).

^13^C NMR (125.8 MHz, CD_3_OD): 138.83 (CH_2_*C*_quat_), 130.25, 129.68, 127.87 (H*C*_Ph_), 68.58 (d, *J*_C,P_ = 6.8, O*C*H_2_CH_2_Ph), 68.11 (d, *J*_C,P_ = 6.8, O*C*H_2_(CH_2_)_5_), 51.04 (*C*H_2_(CH2)_2_NH_2_), 48.68 (PCH_2_*C*H_2_N), 37.86 (*C*H_2_NH_2_), 37.71 (d, *J*_C,P_ = 6.3, OCH_2_*C*H_2_Ph), 31.50 (d, *J*_C,P_ = 6.0, OCH_2_*C*H_2_CH_2_) , 30.68, 30.64, 30.25, 26.53 (O(CH_2_)_2_(*C*H_2_)_4_), 23.23 (*C*H_2_CH_2_NH_2_), 21.18 (d, *J*_C,P_ = 140.5, P*C*H_2_).

^31^P{^1^H} NMR (202.5 MHz, CD_3_OD): 26.48.

**IR** ν_max_ 3087 (m), 3024 (s), 2961–2555 (m, vbr), 2924 (vs), 2853 (s), 2746–2555 (w, vbr), 2009 (w), 1603 (w), 1496 (w), 1466 (m), 1454 (m), 1394 (w), 1226 (m), 1203 (m), 1155 (vw), 1092 (sh, w), 1057–972 (w–m, br), 905 (vw), 750 (w), 699 (m), 550 (vw).

**HR-MS** (ESI^+^): for C_44_H_84_O_6_N_6_P_2_ (M + H)^+^*m*/2*z* calculated 427.29583, found 427.29547.

#### Diisobutyl Tetradecane-1,14-diyl Bis((2-(bis(3-aminopropyl)amino)ethyl)phosphonate) Hexahydrochloride (**80**)

The title compound was prepared according to general methods **A1**, **B1**, **C**, **D**, and **E** from mono methyl vinylphosphonate (0.35 g, 2.89 mmol) in 14% overall yield (409 mg, 0.407 mmol) as a white solid.

^1^H NMR (401 MHz, CD_3_OD) δ 4.15 (dt, *J* = 7.7, 6.6 Hz, 4H, OC*H*_2_CH_2_), 3.92 (td, *J* = 6.6, 2.5 Hz, 4H, OC*H*_2_CH), 3.52–3.43 (m, 4H, PCH_2_C*H*_2_), 3.43–3.36 (m, 8H, C*H*_2_(CH_2_)_2_NH_2_), 3.11 (t, *J* = 7.5 Hz, 8H, C*H*_2_NH_2_), 2.64–2.45 (m, 4H, PC*H*_2_), 2.22 (h, *J* = 7.7, 7.1 Hz, 8H, C*H*_2_CH_2_NH_2_), 1.99 (dp, *J* = 13.3, 6.6 Hz, 2H, OCH_2_C*H*), 1.84–1.64 (m, 4H, OCH_2_C*H*_2_), 1.53–1.22 (m, 20H, O(CH_2_)_2_(C*H*_2_)_5_), 1.00 (d, *J* = 6.7 Hz, 12H, C*H*_3_).

^13^C NMR (101 MHz, CD_3_OD) δ 73.92 (d, *J* = 7.0 Hz, O*C*H_2_CH), 68.29 (d, *J* = 6.8 Hz, O*C*H_2_CH_2_), 51.07 (*C*H_2_(CH_2_)_2_NH_2_), 37.86 (*C*H_2_NH_2_), 31.62 (d, *J* = 5.8 Hz, OCH_2_*C*H_2_), 30.81, 30.76, 30.71, 30.46 (d, *J* = 6.3 Hz, OCH_2_*C*H), 30.31, 26.62 (O(CH_2_)_2_(*C*H_2_)_5_), 23.30 (*C*H_2_CH_2_NH_2_), 21.18 (d, *J* = 140.6 Hz, P*C*H_2_), 19.03 (*C*H_3_).

^31^P{^1^H} NMR (162 MHz, CD_3_OD) δ 28.60.

**IR** ν_max_ (KBr) 3200–2700 (vs, vbr), 2960 (s), 2926 (vs), 2854 (s), 2700–2500 (m), 1608 (m), 1511 (m), 1468 (m), 1400 (m), 1369 (w), 1227 (s), 1004 (vs), 852 (m), 768 (w), ∼725 (w, sh).

**HR-MS** (ESI^+^): for C_38_H_87_O_6_N_6_P_2_ (M + H)^+^*m*/*z* calculated 785.61568, found 785.61523.

#### Dibutyl Tetradecane-1,14-diyl Bis((2-(bis(3-aminopropyl)amino)ethyl)phosphonate) Hexahydrochloride (**81**)

The title compound was prepared according to general methods **A1**, **B2**, **C**, **D**, and **E** from mono methyl vinylphosphonate (0.37 g, 2.72 mmol) in 18% overall yield (0.50 g, 0.50 mmol) as a white solid.

^1^H NMR (400 MHz, CD_3_OD): 4.20–4.07 (m, 8H, C*H*_2_O), 3.53–3.42 (m, 4H, PCH_2_C*H*_2_), 3.42–3.34 (m, 8H, C*H*_2_(CH_2_)_2_NH_2_), 3.10 (t, 8H, *J* = 7.5 Hz, C*H*_2_NH_2_), 2.60–2.45 (m, 4H, PC*H*_2_), 2.27–2.13 (m, 8H, C*H*_2_CH_2_NH_2_), 1.77–1.65 (m, 8H, C*H*_2_CH_2_O), 1.51–1.27 (m, 24H, CH_3_C*H*_2_, (C*H*_2_)_5_(CH_2_)_2_O), 0.98 (t, 6H, *J* = 7.4 Hz, C*H*_3_).

^13^C NMR (101 MHz, CD_3_OD): 68.27 (d, *J* = 6.7 Hz), 67.92 (d, *J* = 6.8 Hz, *C*H_2_O), 51.08 (*C*H_2_(CH_2_)_2_NH_2_), 48.85 (PCH_2_*C*H_2_), 37.86 (*C*H_2_NH_2_), 33.61 (d, *J* = 6.0 Hz), 31.59 (d, *J* = 5.9 Hz, *C*H_2_CH_2_O), 30.79, 30.75, 30.70, 30.30, 26.61 ((*C*H_2_)_5_(CH_2_)_2_O), 23.28 (*C*H_2_CH_2_NH_2_), 21.24 (d, *J* = 140.7 Hz, P*C*H_2_), 19.80 (CH_3_*C*H_2_), 13.96 (*C*H_3_).

^31^P{^1^H} NMR (162 MHz, CD_3_OD): 28.40.

**IR** ν_max_ (KBr) 2966 (m), 2928 (m), 2625 (m), 1604 (m), 1468 (m), 1384 (m), 1230 (m), 1050 (m), 1021 (m).

**HR-MS** (ESI^+^): for C_38_H_87_N_6_O_6_P_2_ (M + H)^+^ calculated 785.61568, found 785.61506.

#### Dipentyl Tetradecane-1,14-diyl Bis((2-(bis(3-aminopropyl)amino)ethyl)phosphonate) Hexahydrochloride (**82**)

The title compound was prepared according to general methods **A1**, **B2**, **C**, **D**, and **E** from mono methyl vinylphosphonate (0.48 g, 3.50 mmol) in 19% overall yield (0.68 g, 0.65 mmol) as a white solid.

^1^H NMR (400 MHz, CD_3_OD): 4.19–4.07 (m, 8H, C*H*_2_O), 3.50–3.42 (m, 4H, PCH_2_C*H*_2_), 3.42–3.35 (m, 8H, C*H*_2_(CH_2_)_2_NH_2_), 3.10 (t, 8H, *J* = 7.5 Hz, C*H*_2_NH_2_), 2.62–2.45 (m, 4H, PC*H*_2_), 2.28–2.14 (m, 8H, C*H*_2_CH_2_NH_2_), 1.79–1.66 (m, 8H, C*H*_2_CH_2_O), 1.48–1.25 (m, 28H, CH_3_(C*H*_2_)_2_, (C*H*_2_)_5_(CH_2_)_2_O), 1.00–0.89 (m, 6H, C*H*_3_).

^13^C NMR (101 MHz, CD_3_OD): 68.27 (d, *J* = 6.8 Hz), 68.21 (d, *J* = 6.7 Hz, *C*H_2_O), 51.06 (*C*H_2_(CH_2_)_2_NH_2_), 48.83 (PCH_2_*C*H_2_), 37.87 (*C*H_2_NH_2_), 31.61 (d, *J* = 5.8 Hz), 31.29 (d, *J* = 5.9 Hz, *C*H_2_CH_2_O), 30.79, 30.74, 30.70, 30.31, 28.80, 26.62, 23.30 (CH_3_(*C*H_2_)_2_, (*C*H_2_)_5_(CH_2_)_2_O), 23.26 (*C*H_2_CH_2_NH_2_), 21.25 (d, *J* = 140.5 Hz, P*C*H_2_), 14.34 (*C*H_3_).

^31^P{^1^H} NMR (162 MHz, CD_3_OD): 28.41.

**IR***ν*_max_ (KBr) 2928 (s), 2623 (m), 2553 (m), 1598 (m), 1468 (m), 1230 (m), 1171 (m), 1080 (m, sh), 1047 (m), 995 (vs).

**HR-MS** (ESI^+^): for C_40_H_91_N_6_O_6_P_2_ (M + H)^+^*m*/*z* calculated 813.64698, found 813.64643.

#### Dihexyl Tetradecane-1,14-diyl Bis((2-(bis(3-aminopropyl)amino)ethyl)phosphonate) Hexahydrochloride (**83**)

The title compound was prepared according to general methods **A1**, **B2**, **C**, **D**, and **E** from mono methyl vinylphosphonate (0.71 g, 5.22 mmol) in 18% overall yield (0.93 g, 0.95 mmol) as a white solid.

^1^H NMR (400 MHz, CD_3_OD): 4.18–4.06 (m, 8H, C*H*_2_O), 3.50–3.42 (m, 4H, PCH_2_C*H*_2_), 3.42–3.35 (m, 8H, C*H*_2_(CH_2_)_2_NH_2_), 3.10 (t, 8H, *J* = 7.5 Hz, C*H*_2_NH_2_), 2.61–2.45 (m, 4H, PC*H*_2_), 2.28–2.14 (m, 8H, C*H*_2_CH_2_NH_2_), 1.79–1.66 (m, 8H, C*H*_2_CH_2_O), 1.49–1.25 (m, 32H, CH_3_(C*H*_2_)_3_, (C*H*_2_)_5_(CH_2_)_2_O), 0.97–0.87 (m, 6H, C*H*_3_).

^13^C NMR (101 MHz, CD_3_OD) δ 68.26 (d, *J* = 6.9 Hz), 68.24 (d, *J* = 6.7 Hz, *C*H_2_O), 51.06 (*C*H_2_(CH_2_)_2_NH_2_), 48.83 (PCH_2_*C*H_2_), 37.88 (*C*H_2_NH_2_), 32.51 (CH_3_CH_2_*C*H_2_), 31.61 (d, *J* = 5.8 Hz), 31.57 (d, *J* = 6.0 Hz, *C*H_2_CH_2_O), 30.80, 30.76, 30.71, 30.32, 26.63, 26.31, 23.64 (CH_3_*C*H_2_), 23.27 (*C*H_2_CH_2_NH_2_), 21.25 (d, *J* = 140.5 Hz, P*C*H_2_), 14.39 (*C*H_3_).

^31^P{^1^H} NMR (162 MHz, CD_3_OD): 28.40.

**IR***ν*_max_ (KBr) 2925 (s), 2957 (s), 2857 (s), 2678 (m), 2488 (m, sh), 1608 (m ,sh). 1489 (m), 1467 (m), 1391 (m), 1227 (s), 1037 (m), 992 (s).

**HR-MS** (ESI^+^): for C_49_H_96_N_6_O_6_P_2_ (M + 2H)^2+^*m*/2*z* calculated 421.34278, found 421.34226.

### Determination of MIC Values

The antimicrobial activity of the tested compounds against aerobic and facultative anaerobic bacteria was assessed using the standard microdilution method determining the minimum inhibitory concentration (MIC).^45^ Disposable microtitration plates were used for the tests. The compounds were diluted in a MH medium (Mueller–Hinton, BioRad, France) to yield a concentration range between 128 and 0.06 mg/L. The plates were inoculated with a standard amount of the tested microbe; the inoculum density in each well was equal to 10^6^ CFU/mL. The plates were incubated for 24 h at 35 ± 1 °C, and MICs were determined as the lowest concentration of tested compound that visibly inhibited bacterial growth. The minimum bactericidal concentration (MBC) is characterized as the minimum concentration of the sample required to achieve irreversible inhibition, *i.e.*, killing the bacterium after a defined period of incubation. To determine MBCs, the contents of the wells with visibly inhibited growth were inoculated onto blood agar (Trios, Czech Republic)—1 μL for each well—and incubated for an additional 24 h at 35 ± 1 °C. Negative growth of microbial colonies determined the MBCs. Standard reference bacterial strains (*E. faecalis* CCM 4224 = ATCC 29212, *S. aureus* CCM 4223 = ATCC 29213, *E. coli* CCM 3954 = ATCC 25922, *P. aeruginosa* 3955 = ATCC 27853, and *S. epidermidis* CCM 7221; test strain for detection of a biofilm and *ica* operon) from the Czech Collection of Microorganisms (CCM), Faculty of Science, Masaryk University, Brno, were tested. Furthermore, multiresistant bacterial strains were tested, including methicillin-resistant *S. aureus* (MRSA) 4591/A (PBP2a positive), vancomycin-resistant *E. faecium* (VRE) VanA phenotype 419/ANA, ESBL-positive *E. coli* CE5556 (extended-spectrum beta-lactamase positive strain, CTX-M-15) also resistant to fluoroquinolones (DNA gyrase mutation) and resistant to aminoglycosides, and PDC (*Pseudomonas*-derived cephalosporinase)-positive *P. aeruginosa* 21425/C. Strains were obtained from the culture collection of the Department of Microbiology (Faculty of Medicine and Dentistry, Palacký University Olomouc). All tested microorganisms were identified by the MALDI-TOF Biotyper system (Bruker Daltonics, Germany) and stored in cryotubes (ITEST plus, Czech Republic) at −80 °C. All experiments were performed in triplicates, and the value that appeared with most frequency (mode) is shown in the result table.

### Determination of the Antimicrobial Activity of LPPO/LEGO-LPPOs in the Presence of 4% BSA^30^

Disposable microtitration plates were used for the tests. The selected samples were diluted in a MH broth (Mueller–Hinton, BioRad) to yield a concentration range between 128 mg/L and 0.06 g/L. BSA (VWR Chemicals) was added to prepared plates at the final concentrations of 4% w/v.^30^ The plates were inoculated with a standard amount of the tested microbe; the inoculum density in each well was equal to 10^6^ CFU/mL. Standard reference bacterial strains (*E. faecalis* CCM 4224, *S. aureus* CCM 4223, *E. coli* CCM 3954, and *P. aeruginosa* CCM 3955*)* from the Czech Collection of Microorganisms (CCM), Faculty of Science, Masaryk University, Brno, were tested. The MIC was determined after 24 h of incubation at 35 ± 1 °C as described above.

### Determination of MIC Values in 24 Strains of Wild-Type *Staphylococcus aureus*

The antimicrobial activity of selected LEGO-LPPOs was determined in 24 strains of *S. aureus* obtained from the culture collection of Department of Microbiology (Faculty of Medicine and Dentistry, Palacký University Olomouc) (for the list of *S. aureus* strains, see Table 5). All tested microorganisms were stored in cryotubes (ITEST plus, Czech Republic) at −80 °C. First, the MICs of selected antibiotics were tested as described above. These antibiotics were penicillin (Biotika, Slovakia), oxacillin (Bristol-Myers Squibb, United States), ampicillin/sulbactam (Pfizer, United States), chloramphenicol (Sigma-Aldrich, United States), tetracycline (Sigma-Aldrich, United States), erythromycin (Serva, Deutschland), clindamycin (Pfizer, United States), ciprofloxacin (Sigma-Aldrich, United States), gentamicin (Lek Pharmaceuticals d.d., Slovenia), teicoplanin (Sanofi, France), and vancomycin (Mylan, United States). Consequently, the antimicrobial activity of four LEGO-LPPOs (**29**, **60**, **25**, and **38**) was tested in 24 *S. aureus* wild-type strains by the means of MIC determination (as described above).

### Evaluation of the Bactericidal Effect of LEGO-LPPOs in Time (Kill-Time Assay)

The experiments were performed in 200 μL of MH broth in sterile microtiter plates with bacterial suspensions in each well corresponding to 10^6^ CFU/mL for each bacterial strain (*E. coli* CCM 3954 and *S. aureus* CCM 4223). The LPPOs **29**, **60**, **25**, and **38** were diluted in MH broth with bacterial suspension at concentrations equivalent to the minimal bactericidal concentration (MBC) and 4 times MBC. Also, two antibiotics were used, colistin (Sigma-Aldrich, United States) in case of *E. coli* and daptomycin (Sigma-Aldrich, United States) for *S. aureus*, also at concentrations corresponding to MBC and 4× MBC (determined as described above). The concentrations of LEGO-LPPO and antibiotics are depicted in Table 7. The prepared mixtures of LPPOs and bacteria were incubated for 24 h at 35 ± 1 °C, and at determined points of time (0, 2, 4, 6, 8, 12, and 24 h), 10 μL was transported on a MH (Mueller–Hinton, TRIOS) cultivation agar and spread with bacteriological loop. After incubation for another 24 h at 35 ± 1 °C, bacterial growth was assessed. The obtained data were used to plot kill-time curves referring to individual tested substances and their concentrations for each bacterial strain (*E. coli* CCM 3954, *S. aureus* CCM 4223).

**Table 7:** Concentrations of LPPO and Antibiotics Used in Time-Kill Experiment (mg/L)

	*Escherichia coli* CCM 3954		*Staphylococcus aureus* CCM 4223	
LPPO	MBC (mg/L)	4× MBC (mg/L)	MBC (mg/L)	4× MBC (mg/L)
**25**	2	8	2	8
**38**	2	8	2	8
antibiotic	MBC (mg/L)	4× MBC (mg/L)	MBC (mg/L)	4× MBC (mg/L)
colistin	1	4		
daptomycin			1	4

### Persister Killing Assay

The experiment was performed as described by Grassi et al.^32^ The three reference bacterial strains, *P. aeruginosa* CCM 3955, *E. coli* CCM 3954, and *S. aureus* CCM 4223, were used in the study. Carbonyl cyanide *m*-chlorphenylhydrazone (Sigma-Aldrich, United States) was diluted in DMSO (stock solution 40 mg/mL) and stored at −20 °C. Antibiotics used in the experiment were colistin, daptomycin (Sigma-Aldrich, United States), and ampicillin/sulbactam (Pfizer, United States). Minimum inhibitory concentrations of tested compounds were determined (as described above in Determination of MIC Values), and concentrations corresponding to MIC, 5× MIC, and 10× MIC were used in the experiment. The bacterial suspensions were cultivated overnight in MH broth at 35 ± 1 °C with shaking. One milliliter of the suspension was transferred into a microtube and incubated for 3 h at 35 ± 1 °C with 10 μL of [(3-chlorophenyl)hydrazono]malononitrile (CCCP) (200 mg/L) in case of *P. aeruginosa* and *E. coli* and 20 μL in case of *S. aureus*. After the treatment, the bacteria were washed twice in saline solution (1700*g* for 10 min) and resuspended in saline at a final density of 5 × 10^8^ CFU/mL. To evaluate the activity of LPPOs, colistin, and ampicillin/sulbactam against CCCP-induced persisters, CCCP-treated and untreated bacteria were diluted in SPB (phosphate-buffered saline) supplemented with 1% MH broth to a final density of 10^6^ CFU/mL. In case of daptomycin, the bacteria were diluted to a final density of 10^6^ CFU/mL in deionized water supplemented with 1% MH broth and 75 mg/L CaCl_2_ (Sigma-Aldrich, United States). After 3 h of incubation with gentle shaking at 35 ± 1 °C, samples were exponentially diluted in a microtiter plate in six wells that were inoculated and spread on MH agar (10 μL each well). After additional incubation for 24 h at 35 ± 1 °C, the bacterial cells were counted to determine CFU/mL.

### Selection for Resistant Bacteria

Induction of resistance was performed in microtitration plates by repeated exposure of bacterial strains to subinhibitory concentrations of tested substances **25** and **38**. Tested substances were diluted in a Mueller–Hinton broth (Biorad) exponentially (64, 32, 16, 8, 4, 2, and 1, 0.5 mg/L for **25** and 32, 16, 8, 4, 2, 1, 0.5, and 0.25 mg/L for **38**). As a comparison method, the induction resistance testing was performed with antibiotics ciprofloxacin and ceftazidime (Sigma-Aldrich, United States) in the following concentration range: 128, 64, 32, 16, 8, 4, 2, and 1 mg/L for **CTZ** and 16, 16, 8, 4, 2, 1, 0.5, 0.25, and 0.12 mg/L for **CIP**. Prepared microtitration plates were stored at −20 °C and were defrosted one after another at the day of use. The wells of microtitration plate were inoculated with *P. aeruginosa* CCM 3955, obtained from the Czech Collection of Microorganisms, Masaryk University, Brno. The final concentration of the bacterial inoculum in the well was 10^6^ CFU/mL. Incubation was carried out at 35 ± 1 °C for 24 h. After incubation, the values of minimal inhibition concentration (MIC) were noted. Ten microliters of the bacterial suspension from the wells with subinhibitory concentrations (*i.e.*, from the wells next to the wells with MIC) was cultivated on blood agar (TRIOS) for 24 h at 35 ± 1 °C. These grown bacterial cultures were then diluted in the Mueller–Hinton Broth and inoculated at 10^6^ CFU/mL into the microtitration plate containing dilution series of the tested compounds for the next cycle. The described procedure was considered to be one cycle of induction. Overall, the whole experiment consisted of 20 cycles of induction. After the 10th and 20th round, MICs of original strains were determined and compared with MICs of the tested strains.

### Determination of Hemolytic Activity

The hemolytic activity of LPPO was determined as the amount of hemoglobin released from red blood cells (RBCs) according to Drabkin’s method.^46^ Blood was aseptically collected from volunteer human donors at the Transfusion Department (University Hospital Olomouc). The blood acquisition protocol adhered to the requirements of the Ethics Committee of the University Hospital Olomouc and Faculty of Medicine and Dentistry, Palacký University, in Olomouc. All patients had signed written informed consent. For experiments, stock solutions of tested compounds (0.32–200 g/L) were prepared in DMSO. Before application, the solutions were diluted in 150 mM NaCl so that the final concentration of DMSO in NaCl was 0.5% (v/v). RBCs incubated with NaCl containing the respective aliquot of Triton-X100 (1%, v/v) were used as the positive control (100% hemolytic activity), and RBCs incubated with NaCl containing the respective aliquot of DMSO (0.5%, v/v) were used as the negative control (0% hemolytic activity). The collected blood was centrifuged (500*g*, 4 °C, 10 min), the supernatant was discarded, and RBCs were washed three times with 150 mM NaCl and resuspended to the concentration of 4% (v/v) in NaCl. Then, 250 μL of 4% (v/v) RBCs was added to 250 μL of the solution of the tested compound (final concentration 1.6–1000 mg/L) in NaCl as well as to positive and negative control and incubated for 3 h at 37 °C. After incubation, the mixture was centrifuged (500 *g*, 4 °C, 10 min). Then 20 μL of supernatant were transferred to 96-well plates and 200 μL of Drabkin’s solution was added. After incubation (10 min, room temperature), the absorbance was measured at 540 nm on a microplate reader (INFINITE M200, Tecan, Switzerland). The hemolytic activity was expressed as the concentration of tested compound that causes lysis of 50% RBCs (HC_50_).

The experiments were performed as three independent examinations with at least three replicates for each sample. Data were expressed as means of HC_50_ ± S.D.

### Hydrophobicity Index (CHIg)

The gradient chromatography hydrophobicity index (CHIg) was measured by the linear gradient HPLC method and calculated based on retention time and acetonitrile composition, as described before.^31^ Majority of samples were measured using gradient A; for more polar samples, gradient B was used. Analytes were identified by mass spectrometry in full scan mode. The mobile phase was adjusted with 0.1% formic acid to help on elution and ionization of analytes; the final pH was 3.7. HPLC method: CHIg was measured on UPLC-qTof (Waters, Milford, USA) using a C18 UPLC column (Waters XBridge 50 × 2.1 mm, 1.9 μm) with the following gradients: Gradient A: 10% B hold for 0.5 min, 95% B in 5.5 min, hold till 6 min; gradient B: 5% B hold for 1 min, 95% B in 5 min, hold till 5.5 min. A = 0.1% formic acid, B = 0.1% formic acid in acetonitrile. Flow rate was set to 0.5 mL, and injection volume was 0.5 μL. Output signal was monitored by mass spectrometry with positive electrospray ionization in full scan mode.

### Membrane Potential Measurements

The change in membrane potential by LEGO-LPPOs was monitored with a DiSC_3_(5) fluorescent probe as described previously.^47^ The probe was incorporated to polarized membranes, which leads to a reduction of its fluorescence intensity. When the membrane potential is disrupted by the action of the membrane active substance, the probe is released from the membrane and the fluorescence intensity increases.^48^ The bacterial cells (*E. coli* cells CCM 3954 or *S. aureus* cells CCM 4223) were grown to OD_450_ = 0.2 (corresponding to 4–5 × 10^7^ cells/mL), centrifuged (5000*g*, 25 °C, 10 min), and washed twice in glucose buffer (10 mM HEPES, 0.5% glucose). EDTA solution was added to the *E. coli* suspension to a final concentration of 10 mM to disrupt the outer membrane and facilitate access of the staining of the cytoplasmic membrane. The suspension was incubated with EDTA for 20 min on a roller tube mixer, and the suspension was centrifuged to remove EDTA. The supernatant was discarded, and the resulting pellet was resuspended in glucose buffer to a final OD_450_ = 0.2. The DiSC_3_(5) probe (1 mM stock solution in DMSO) was added to this suspension to a final concentration of 1 μM, and the aerated suspension was labeled for 90 min in the dark. The preparation of *S. aureus* cells was similar, omitting the step with EDTA. Fluorescence intensity was measured at 25 °C using a FluoroMax-3 spectrofluorometer (Jobin Yvon, Horiba) with 600 nm excitation and 670 nm emission wavelengths. The RPB590-610 and RPE650LP optical filters were used (Omega Optical) in the excitation and emission paths, respectively. The cell suspension was measured in 10 × 10 mm quartz cuvettes in a volume of 2 mL with continuous stirring by a magnetic stirrer. The LEGO-LPPO from the stock solution was added to the cuvette to the desired concentrations. As a positive control for membrane depolarization, 5 μM melittin (Sigma) was added to the cuvette (not shown). Representative kinetics are shown (*n* = 10).

### Membrane Permeabilization Assay

*E. coli* CCM 3954 cells were grown aerobically in an LB medium at 37 °C to mid log phase (OD_450_ = 0.5), harvested (8000*g*, 25 °C, 10 min), washed, and resuspended (final OD_450_ = 0.1) in a buffer containing 10 mM HEPES (pH 7.2), 0.5% glucose, and 10 μM propidium iodide (PI, Invitrogen). LPPOs were added to 2 mL of the bacterial suspension in a 10 × 10 mm quartz cuvette, and PI uptake into cells (indicating membrane permeabilization) was monitored as the increase in fluorescence intensity (excitation at 515 nm, emission at 620 nm with bandpass 5 and 5 nm, respectively) at 25 °C using a FluoroMax-3 spectrofluorometer (Jobin Yvon, Horriba). The optical filters 3RD500-530 and 3RD570LP (Omega Optical) were used in excitation and emission paths for suppression of light scattered by the cells. The bacterial suspension was continuously stirred by the magnetic stirrer during the measurements. As a positive control for cell permeabilization, 5 μM melittin (Sigma) was added to the cuvette. Representative kinetics are shown (*n* > 5).

### Planar Lipid Bilayer Experiments

Black lipid bilayer membranes were formed by painting a solution of 3% w/v 1,2-diphytanoyl-*sn*-glycero-3-phospho-(1′-*rac*-glycerol) (DPhPG, Avanti Polar Lipids) in *n*-decane/butanol (9:1, v/v) across the aperture (0.5 mm in diameter) in the diaphragm dividing the Teflon chamber into two compartments. Both compartments contained 1.5 mL of 1 M KCl and 10 mM Tris, pH 7.4. The temperature was kept at 25 °C. LPPO was added to the *cis* side of the membrane in the concentration of 1.25, 2.5, or 5.0 mg/L. The membrane current was registered with Ag/AgCl electrodes (Theta) with a membrane voltage of 50 mV, amplified by an LCA-200-100GV amplifier (Femto), and digitized by a KPCI-3108 card (Keithley). The signal was processed with the QuB software.^49^ The histograms of membrane currents were created using kernel density estimation (rectangular kernel with a 5 pS width).

### Liposome Preparation and Liposome Leakage Assay

Dioleylphosphatidylglycerol (DOPG), dioleoylphosphatidylethanolamine (DOPE), and dioleoylphosphatidylcholine (DOPC) were purchased from Avanti Polar Lipids. Liposomes for the carboxyfluorescein (CF) leakage assay were prepared by mixing the appropriate amount of lipids (0.5 mg/mL) in chloroform/methanol 2:1 (v/v). The solvent was subsequently evaporated *in vacuo* to form a thin film on the walls of a glass tube. The multilamellar vesicles were prepared by hydration of lipids in a buffer containing 50 mM 5(6)-carboxyfluorescein (CF) and 5 mM HEPES (pH 7.4) for 90 min. Large unilamellar vesicles (LUVs) were prepared by repeated extrusion of the multilamellar vesicles through 100 nm polycarbonate filters (Avestin) using a Mini-Extruder apparatus (Avanti Polar Lipids). Vesicles were separated from the nonencapsulated dye by gel filtration on Sephadex G-50 using 100 mM NaCl, 0.5 mM Na_2_EDTA, and 5 mM HEPES (pH 7.4) as the elution buffer. Fractions with the highest content of entrapped dye were put together and diluted in the same buffer to give a final phospholipid concentration of 10 μM according to the assessed content of inorganic phosphate. The leakage of CF from suspension of liposomes in 2 mL cuvette was initiated by LPPO addition and monitored as the increase in CF fluorescence intensity (excitation at 480 nm, emission at 520 nm with 2 nm bandpasses) at 25 °C using a FluoroMax-3 spectrofluorometer (Jobin Yvon, Horriba). The maximum intensity (100%) was achieved by addition of 0.2% Triton X-100 to liposomal suspension. Representative kinetics are shown (*n* > 8).

### Scanning Electron Microscopy

All bacterial samples were essentially processed as described in Šiková et al.^50^ with some modifications (Pospíšil et al.).^33^ The bacterial suspension in the Mueller–Hinton medium was briefly prefixed in 3% glutaraldehyde for 15 min. Prefixed cells were centrifuged at 5250*g* for 10 min at room temperature, resuspended in 3% glutaraldehyde in cacodylate buffer (pH 7.2–7.4), and stored in a refrigerator for 24 h. Fixed bacteria were extensively washed in cacodylate buffer and sedimented overnight onto the circular, poly-l-lysine treated glass coverslips. Washed coverslips were post-fixed in 1% OsO_4_ in ddH_2_O at room temperature for 1 h, dehydrated in graded ethanol series, and critical point dried in a K850 Critical Point Dryer (Quorum Technologies Ltd., Ringmer, UK). Dried coverslips sputter-coated with 3 nm of platinum (Q150T Turbo-Pumped Sputter Coater; Quorum Technologies Ltd., Ringmer, UK) were examined in an FEI Nova NanoSEM scanning electron microscope (FEI, Brno, Czech Republic) at 3 kV using CBS and TLD detectors.

### Determination of Cytotoxicity

Cytotoxicity was assessed by the alamarBlue assay (Invitrogen) with human liver HepG2 cells in a 384-well plate format. Cells were incubated with test compound concentrations (0.005–99.0 μM) for 24 h in a supplemented RPMI1640 medium, the medium was removed, and alamarBlue was added followed by incubation for 4 h. Metabolic formation of the fluorescent resorufin was measured on a plate reader (excitation 550 nm, emission 595 nm). The fluorescence signal is proportional to metabolically active and viable cells. Finally, the cytotoxic dose at 50% viability (CTD_50_) values were calculated reflecting the test compound concentration that reduced cell viability by 50%. Mean values of four replicates are shown.

### *In Vitro* Skin Irritation Test

#### Test Method

The test was performed in compliance with Commission Regulation (EC) No. 640/2012 of 6 July 2012 amending, for the purpose of its adaptation to technical progress, Regulation (EC) No. 440/2008 laying down test methods pursuant to Regulation (EC) No. 1907/2006 of the European Parliament and of the Council on the Registration, Evaluation, Authorisation and Restriction of Chemicals (REACH) (Method B.46 *In Vitro* Skin Irritation: Reconstructed Human Epidermis Model Test)–OECD TG 439–In Vitro Skin Irritation: Reconstructed Human Epidermis Test Method.

#### Principle of the Test and Data Interpretation

The test chemical is applied topically to a three-dimensional reconstructed human epidermal model composed of a functional epidermis and stratum corneum. The method is based on the prerequisite that irritant chemicals penetrate the stratum corneum by diffusion and exert cytotoxic effects on the cells in the tissue layers. Cell viability is determined by enzymatic conversion of the vital dye MTT (3-(4,5-dimethylthiazol-2-yl)-2,5-diphenyltetrazolium bromide) into blue formazan salt that is quantitatively measured after extraction from tissues. Irritant chemicals are identified by their ability to decrease cell viability below defined threshold levels (*i*.*e*., ≤50%, for UN GHS Category 2).

##### Test Material Application

Application of 30 μL per tissue.

##### Skin Model

Skin model EpiDerm, an organotypic model of human epidermis consisting of reconstructed epidermis and functional stratum corneum, produced by MatTek In Vitro Life Science Laboratories, Bratislava, Slovakia.

##### Test Procedure

The test was performed according to the Protocol for ″In Vitro EPIDERM Skin Irritation Test, EPl-200-SIT″ (MatTek Corporation, USA) in current wording. Upon receipt of the EpiDerm EPl-200-SIT Kit (containing 24 tissues, cultivation medium, and MTT assay kit), all components were placed in the refrigerator, with the MTT concentrate placed in the freezer. Twenty-four hours before testing, the tissues were transferred into six-well plates containing the assay medium and preincubated for 1 h (humidified incubator HERAcell (Heraeus), temperature 37 °C, humidity 95%, CO_2_ 5%). At the end of the first preincubation, the tissues were transferred into six-well plates with a fresh assay medium and further preincubated overnight. On the day of experiment, the tissues were exposed to the test materials and concurrent negative (PBS) and positive (5% SDS) controls for 1 h. For each tested material, three tissues were used. The test materials and controls were applied using an automatic pipette (volume 30 μL). After exposure, the tissues were rinsed with sterile PBS, blotted, and dried with a cotton swab. The tissues were then incubated for a further 42 h. After incubation, the tissues were placed into a medium containing MTT for 3 h and then rinsed with PBS, and the blue formazan crystals produced by enzymatic reduction of MTT were extracted by isopropyl alcohol for 2 h. The formazan concentration was determined by measurement of optical density at 570 nm (OD570, Spectrophotometer Varian UV–Vis Cary 1E). The OD values obtained with each test sample were used to calculate the percentage of viability compared to NC, which is set at 100%.

##### Interpretation of Results

The test substance is considered to be irritant to skin in accordance with UN GHS category 2 if the tissue viability after exposure and post-treatment incubation is less than or equal to 50%. Depending on classification requirements, the test substance may be considered to have no category if the tissue viability after exposure and post-treatment incubation is more than 50%.

##### Assay Acceptability Criteria

Assay acceptance criterion 1: negative control: The absolute OD of the negative control (NC) tissues (treated with sterile DPBS) in the MTT test is an indicator of tissue viability obtained in the testing laboratory after shipping and storing procedures and under specific conditions of use. The assay meets the acceptance criterion if the mean OD570 of the NC tissues is ≥1.0 and ≤ 2.5.
Assay acceptance criterion 2: positive control: A 5% SOS (in H_2_O) solution is used as positive control (PC) and tested concurrently with the test chemicals. The assay meets the acceptance criterion if the mean viability of PC tissues expressed as % of the negative control tissues is ≤20%.
Assay acceptance criterion 3: standard deviation (SD): Since in each test skin irritancy potential is predicted from the mean viability determined on three single tissues, the variability of tissue replicates should be acceptably low. The assay meets the acceptance criterion if the SD calculated from individual % tissue viabilities of the three identically treated test material replicates is <18%.

### *In Vitro* Eye Irritation Test

#### Test Method

The test was performed in compliance with OECD Guideline for the Testing of Chemicals 492: Reconstructed Human Cornea-Like Epithelium (RhCE) test method for identifying chemicals not requiring classification and labeling for eye irritation or serious eye damage.

#### Principle of the Test and Data Interpretation

The test chemical is applied topically to 30 reconstructed human cornea-like tissue constructs, and tissue viability is measured following exposure and a post-treatment incubation period. The tissues are reconstructed from primary human epidermal keratinocytes, which have been cultured for several days to form a stratified, highly differentiated squamous epithelium morphologically similar to that found in the human cornea. Cell viability is determined by enzymatic conversion of the vital dye MTT (3-(4,5-dimethylthiazol-2-yl)-2,5-diphenyltetrazolium bromide) into blue formazan salt that is quantitatively measured after extraction from tissues. Chemicals not requiring classification and labeling according to UN GHS (No Category) are identified as those that do not decrease tissue viability below a defined threshold (*i*.*e*., tissue viability >60% in EpiOcular Eye Irritation Test).

##### Test Material Application

Application of 50 μL per tissue.

##### Eye Model

The EpiOcular RhCE tissue construct, produced by MatTek In Vitro Life Science Laboratories, Bratislava, Slovakia, consists of at least three viable layers of cells and a nonkeratinized surface showing a cornea-like structure analogous to that found *in vivo*.

##### Test Procedure

The test was performed according to the Protocol for ″EpiOcular Eye Irritation Test, OCL-200-EIT″ (MatTek Corporation, USA) in current wording. Upon receipt of the EpiOcular OCL-200-EIT Kit (containing tissues, cultivation medium, and MTT assay kit), all components were placed in the refrigerator, with the MTT concentrate placed in the freezer. Twenty-four hours before testing, the tissues were transferred into six-well plates containing the assay medium and preincubated for 1 h (humidified incubator HERAcell (Heraeus), temperature 37 °C, humidity 95%, CO_2_ 5%). At the end of the first preincubation, the tissues were transferred into six-well plates with a fresh assay medium and further preincubated overnight. On the day of experiment, the tissues were exposed to the test materials and concurrent negative (deionized water) and positive (methyl acetate) controls for 30 min. For each tested material, two tissues were used. Thirty minutes prior to treatment, the tissues were prewetted with 20 μL of the phosphate buffer saline (PBS). The test materials and controls were applied using an automatic pipette (volume 50 μL). After exposure, the tissues were rinsed with sterile PBS, immersed in 5 mL of the fresh medium for 12 min, transferred into six-well plates with the fresh assay medium, and incubated for an additional 120 min. After incubation, the tissues were placed into a medium containing MTT for 3 h then blotted and placed into wells with 2 mL of isopropyl alcohol. The blue formazan crystals produced by enzymatic reduction of MTT were extracted by isopropyl alcohol overnight. The formazan concentration was determined by measurement of OD570 (Spectrophotometer Varian UV–Vis Cary 1E). The OD values obtained with each test sample were used to calculate the percentage of viability compared to NC, which is set at 100%.

##### Interpretation of Results

The test chemical is identified as not requiring classification and labeling according to UN GHS (No Category) if the mean percent tissue viability after exposure and post-exposure incubation is more than the established percentage tissue viability cutoff value, *i*.*e*., tissue viability >60% in the EpiOcular Eye Irritation Test. In this case, no further testing in other test methods is required. If the mean percent tissue viability after exposure and post-exposure incubation is less than or equal to the established percentage tissue viability cutoff value, *i*.*e*., tissue viability ≤60% in the EpiOcular Eye Irritation Test, no prediction can be made from this result in isolation. This is because in case of a true positive, the method cannot resolve between UN GHS Categories 1 and 2. Furthermore, RhCE test methods show a high percentage of false-positive results; therefore, further information will be required for classification purposes according to the IATA guidance document (OECD, 2018. Guidance Document on an Integrated Approach on Testing and Assessment for Serious Eye Damage and Eye irritation. Series on Testing and Assessment No. 263. ENV Publications, Organisation for Economic Cooperation and Development, Paris).

##### Assay Acceptability Criteria

Assay acceptance criterion 1: negative control: The absolute OD of the negative control (NC) tissues (treated with sterile DPBS) in the MTT test is an indicator of tissue viability obtained in the testing laboratory after shipping and storing procedures and under specific conditions of use. The assay meets the acceptance criterion if the mean OD570 of the NC tissues is ≥1.0 and ≤2.5.
Assay acceptance criterion 2: positive control: A 5% SOS (in H_2_O) solution is used as positive control (PC) and tested concurrently with the test chemicals. The assay meets the acceptance criterion if the mean viability of PC tissues expressed as % of the negative control tissues is ≤20%.
Assay acceptance criterion 3: standard deviation (SD): Since in each test skin irritancy potential is predicted from the mean viability determined on three single tissues, the variability of tissue replicates should be acceptably low. The assay meets the acceptance criterion if the SD calculated from individual % tissue viabilities of the three identically treated test material replicates is <18%.

*In vitro* skin and eye irritation tests were carried out by The National Institute of Public Health, Czech Republic, on a commercial basis.^51^

### Skin and Eye Irritation Tests in Rabbits

#### Ethics Statement

##### Facilities Management and Animal Husbandry

Animal care was performed in compliance with the SOPs of the OBK IPHYS CAS; the European convention for the protection of vertebrate animals used for experimental and other scientific purposes (ETS 123); the Czech Collection of Laws No. 246/1992, inclusive of the amendments, on the Protection of animals against cruelty, and Public Notice of the Ministry of Agriculture of the Czech Republic; Collection of laws No. 419/2012 as amended, on keeping and exploitation of experimental animals. The Department of Biological Controls, IPHYS CAS is a holder of the Accreditation Certificate CZ 11760522 for users issued by Central Committee for Animal Protection of the Czech Republic.

##### Animal Welfare Act Compliance

The study was prepared for this type of experiment and approved by the Institutional Animal Care and Use Committee (IACUC) and the Committee for Animal Protection of the Ministry of Health of the Czech Republic (No. 55/2019, June 12, 2019). The procedures used in this study were designed to conform to accepted practices and to minimize or avoid causing pain, distress, or discomfort to the animals. The number of animals selected for use in this study was considered to be the minimum (OECD Principles) number to meet the end point for this type of study.

Rabbits domestic broiler hybrid (Chovné a dodavatelské zařízení Václav Robeš, Náves 85,683 52 Saratice, Czech Republic), three animals (males) per compound were used.

#### Skin Irritation Tests in Rabbits

The animals were dermally (topically) administered with two administrations (test site nos. 1 and 2) of TI at a fixed volume of 0.5 mL (in total, each animal received TI in 1.0 mL of the application solution). Water (0.5 mL) for injection was applied dermally (topically) to two control sites (control site nos. 3 and 4). In total, each animal received 1.0 mL of water for injection. Animals were shaved 24 h before the test item administration. The fur was removed by closely clipping the dorsal area of the trunk of the animals of approx. size 10 × 15 cm. The test item was applied to two small areas (2.5 × 2.5 cm) of the skin. The concentration of the test item aqueous solution was 200 mg/L.

On D1, the animals were dermally (topically) administered with two administrations (test site nos. 1 and 2) of TI at a fixed volume of 0.5 mL (in total, each animal received TI in 1.0 mL of the application solution). Water (0.5 mL) for injection was applied dermally (topically) to two control sites (control site nos. 3 and 4). In total, each animal received 1.0 mL of water for injection.

The test item (0.5 mL) in solution of concentration 100 or 200 mg/L and 0.5 mL of control solution were applied directly to each test and control site on the skin. The administration sites were covered with 2.5 × 2.5 cm permeable gauze and then bandaged for 4 h.

At the end of the contact time, the covers are removed and the position of the administration sites is marked with indelible ink. Animals were observed at 1, 24, 48, and 72 h after administration to calculate and evaluate skin irritation. In total, the animals were observed for 7 days.

The M9 animal was observed for only 48 h. After 48 h, it was humanely euthanized for constipation. Subsequently, the M9 animal was replaced with the M20 animal.

#### Eye Irritation Tests in Rabbits

Rabbits domestic broiler hybrid (Chovné a dodavatelské zařízení Václav Robeš, Náves 85,683 52 Saratice, Czech Republic), three animals (males) per tested item were used.

One day before the administration, both eyes of all the animals were macroscopically examined by an ophthalmoscope. Only healthy animals with healthy eyes were used.

On day one, 100 μL of the test item application solution was instilled into the conjunctiva sac of the right eye of each rabbit after gently pulling the lower lid away from the eyeball. Then, the lids were gently held for 1 s to prevent the loss of the Tl solution. The left eyes of the animals, which remained Tl untreated, served as control. The concentration of the test item aqueous solution was 100 mg/L.

All rabbits were observed daily for clinical signs, morbidity, or mortality during the acclimation and observation period. Clinical observations included general condition; signs of toxicity; changes in the skin and fur, eye, and mucous membranes; respiratory, circulatory, autonomic, and central nervous system; somatomotor activity; and behavior pattern (attitude toward food, water, and hygiene). Attention was directed to observations of tremors; convulsion; salivation; diarrhea; lethargy; sleep; coma; changes in gait, posture, and response to handling; and the presence of clonic or tonic movements and stereotypes. The onset, duration, and severity of any signs were recorded. The rabbits were observed for 8 days after administration (including the day of necropsy). The eye irritability evaluation was carried out 1, 24, 48, and 72 h after the administration and then 7 days after administration.

### MTD Study

The study was carried out according to ICH M3 (R2) Non-Clinical Safety Studies for the Conduct of Human Clinical Trials and Marketing Authorization for Pharmaceuticals, OECD Guideline for Testing of Chemicals No. 420, and relevant Test Facility SOPs. The test facility is a holder of Certificate of Good Laboratory Practice for Medicinal Products for Human Use (Act No. 378/2007 Coll). The study was not performed in GLP compliance but followed standards of the Quality Assurance Program.

Animal care was in compliance with the SOPs of the OBK IPHYS CAS; the European convention for the protection of vertebrate animals used for experimental and other scientific purposes (ETS 123); the Czech, Collection of Laws No. 246/1992, inclusive of the amendments, on the Protection of animals against cruelty, and Public Notice of the Ministry of Agriculture of the Czech Republic; Collection of Laws No. 419/2012 as amended, on keeping and exploitation of experimental animals. Department of Biological Controls, IPHYS CAS is a holder of the Accreditation Certificate MZE-23481/2021-18134 for users issued by Ministry of Agriculture of the Czech Republic.

The study was prepared for this type of experiment and approved by the Institutional Animal Care and Use Committee (IACUC) and the Committee for Animal Protection of the Ministry of Health of the Czech Republic (No. 55/2019). Procedures used in this plan were designed to conform to accepted practices and to minimize or avoid causing pain, distress, or discomfort to the animals.

The number of animals selected for use in this study is considered to be the minimum number necessary to meet scientific and regulatory guidelines for this type of study (OECD and 3R principles).

Animals were divided into 10 dose groups and 2 control groups (Table 8); each group was composed of three animals (females).

**Table 8:** Allocation and Dosing

test item	administration route	group designation	dose (mg/kg) single administration	number of animals
compound **25**	p.o.	G1	10	F1, F2, F3
		G2	50	F4, F5, F6
		G3	100	F7, F8, F9
		G4	150	F10, F11, F12
		G5	200	F13, F14, F15
	s.c.	G6	0.5	F19, F20, F21
		G7	2.5	F22, F23, F24
		G8	5.0	F25, F26, F27
		G9	7.5	F28, F29, F30
		G10	10	F31, F32, F33
		G11	12.5	F37, F38, F39
		G12	15	F40, F41, F42
vehicle only	p.o.	C1	n/a	F16, F17, F18
	s.c.	C2	n/a	F34, F35, F36

#### Groups Administered Orally

All doses were administered by a single oral administration (gavage) in a volume of 1 mL/100 g of body weight. The next dose level was administered minimally 3 days (72 h) after the previous dose with the same design, depending on the presence or absence of signs of toxicity or mortality.

The first dose (10 mg/kg) of the test item was administered to females from the G1 dose group. Based on the absence of signs of toxicity, the next dose was increased to 50 mg/kg for dose group G2. After dosing of 50 mg/kg, no signs of toxicity were observed, so the next dose was increased to 100 mg/kg for dose group G3. No signs of toxicity were observed after dosing of 100 mg/kg b.w. The next dose was increased to 150 mg/kg b.w. for dose group G4. The dose of 150 mg/kg did not cause any signs of toxicity, so the next dose was increased to 200 mg/kg for dose group G5.

In addition, one group of control animals (three females) was used. The animals of the C1 group were given a single oral administration of vehicle only in a volume of 1 mL/100 g of body weight.

#### Groups Administered Subcutaneously

All doses were administered by single s.c. administration in a volume of 0.1 mL/ 30 g b.w. The next dose level was administered minimally 3 days (72 h) after the previous dose with the same design, depending on the presence or absence of signs of toxicity or mortality.

The first dose (0.5 mg/kg) of the test item was administered to the females from the G6 dose group. Based on the absence of signs of toxicity, the next dose was increased to 2.5 mg/kg for dose group G7. After dosing of 2.5 mg/kg, no signs of toxicity were observed, so the next dose was increased to 5 mg/kg for dose group G8. No signs of toxicity were observed after dosing of 5 mg/kg b.w. The next dose was increased to 7.5 mg/kg b.w. for dose group G9. The dose of 7.5 mg/kg did not cause any signs of toxicity, so the next dose was increased to 10 mg/kg for dose group G10.

In addition, one group of control animals (three females) was used. The animals of the C2 group were given a single s.c. administration of vehicle only in a volume of 0.1 mL/ 30 g b.w.

The decision was made to establish an additional dose group (G11) from the three animals of the originally control group C1 and an additional dose group (G12) from the three animals of the originally control group C2 to determine the MTD level. The dose of 12.5 mg/kg b.w. was subcutaneously administered to the G11 dose group, and the dose of 15 mg/kg b.w. was subcutaneously administered to the G12 dose group.

Skin and eye irritation test in rabbits as well as MTD tests were carried out by the Institute of Physiology CAS on a commercial basis.^49^

## Supporting Information Available

Molecular formula strings (CSV)
Detailed synthetic procedures and characterization data for selected intermediates; ^1^H, ^13^C, and ^31^P NMR spectra of final compounds; selected intermediates; and additional tables (PDF)

## Abbreviations

AMP: antimicrobial peptide
BSA: bovine serum albumin
CIP: ciprofloxacin
CM: connector module
CTZ: ceftazidime
HM: hydrophobic module
HDP: host defense peptide
LEGO-LPPO: linker-evolved-group-optimized-LPPO
LPPO: lipophosphonoxin
MRSA: methicillin-resistant *Staphylococcus aureus*
NM: nucleoside module
PM: polar module
RhCE: human cornea-like epithelium
RhEM: human epidermis model
SMMTA: small molecule membrane targeting agent
