# Supplementary material for: LEGO-Lipophosphonoxins: A Novel Approach in Designing Membrane Targeting Antimicrobials
Source: J Med Chem. 2022 Jul 15;65(14):10045–78. doi: 10.1021/acs.jmedchem.2c00684 (PMC9580004; doi:10.1021/acs.jmedchem.2c00684)
Supplement: Supplementary file 2 — jm2c00684_si_002.pdf [file jm2c00684_si_002.pdf]

Supporting information

LEGO-Lipophosphonoxins – a Novel Approach in Designing Membrane Targeting Antimicrobials

Duy Dinh Do Pham[1,†], Viktor Mojr[1,†], Michaela Helusová[2], Gabriela Mikušová[1,2], Radek Pohl[1], Eva Dávidová[1], Hana Šanderová[3], Dragana Vítovská[3], Kateřina Bogdanová[4], Renata Večeřová[4], Miroslava Htoutou Sedláková[4], Radovan Fišer[2], Petra Sudzinová[3], Jiří Pospíšil[3], Oldřich Benada[3], Tomáš Křížek[5], Adéla Galandáková[6], Milan Kolář[4], Libor Krásný*[3], Dominik Rejman*[1]

[1]Institute of Organic Chemistry and Biochemistry, Czech Academy of Sciences v.v.i., Flemingovo nám. 2, 166 10 Prague 6, Czech Republic.
[2]Department of Genetics and Microbiology, Faculty of Science, Charles University, Viničná 5, 128 43 Prague 2, Czech Republic.
[3]Institute of Microbiology, Czech Academy of Sciences v.v.i., Vídeňská 1083, 142 20 Prague 4, Czech Republic.
[4]Department of Microbiology, Faculty of Medicine and Dentistry, Palacký University Olomouc, Hněvotínská 3, 775 15 Olomouc, Czech Republic.
[5]Department of Analytical Chemistry, Faculty of Science, Charles University, Albertov 6, 128 43 Prague 2, Czech Republic.
[6]Department of Medical Chemistry and Biochemistry, Faculty of Medicine and Dentistry, Palacký University Olomouc, Hněvotínská 3, 775 15 Olomouc, Czech Republic.

Author Contributions[†] D.D.D.P. and V.M. contributed equally.
*Corresponding authors: rejman@uochb.cas.cz, krasny@biomed.cas.cz

Contents

Synthetic details for intermediates and final compounds including NMR spectra (and LC-MS traces for final compounds) 2

1. General procedures and used materials 2
2. General methods 4
3. Synthetic procedures and characterizations 6
4. Tables S2 and S3 512
5. Table S4 (MBC values) 513

Synthetic details for intermediates and final compounds including NMR spectra (and LC-MS traces for final compounds)

1. General procedures and used materials

Unless stated otherwise, all used solvents were anhydrous. TLC was performed on silica gel pre-coated aluminium plates TLC Silica gel 60 F_{254} (Supelco), and compounds were detected by UV light (254 nm), by heating (detection of dimethoxytrityl group, orange color), by spraying with 1% solution of ninhydrine to visualize amines, and by spraying with 1% solution of 4-(4-nitrobenzyl)pyridine in ethanol followed by heating and treating with gaseous ammonia (blue color of mono- and diesters of phosphonic acid). Preparative column chromatography was carried out on silica gel (40–63µm, Fluorochem), and elution was performed at the flow rate of 60–80 mL/min. The following solvent systems were used for TLC and preparative chromatography: toluene-ethyl acetate 1:1 (T), chloroform-ethanol 9:1 (C1), ethyl acetate-acetone-ethanol-water 6:1:1:0.5 (H3), ethyl acetate-acetone-ethanol-water 4:1:1:1 (H1). The concentrations of solvent systems are stated in volume percents (%, v/v). LC-MS (checking reaction mixtures and purity of intermediates) was performed on Waters AutoPurification System with 2545 Quarternary Gradient Module and 3100 Single Quadrupole Mass Detector using LUNA C18, column (Phenomenex, 100 × 4.6 mm, 3 µm) at flow rate 1 mL/min. Typical conditions: mobile phase, A – 50mM NH_4HCO_3, B – 50mM NH_4HCO_3 in 50% *aq.* CH_3CN, C – CH_3CN, A→B/10 min, B→C/10 min, C/5 min. Preparative RP HPLC was performed on LC5000 Liquid Chromatograph (INGOS-PIKRON, CR) using Luna C18 (2) column (250 × 21.2 mm, 5 µm) at flow rate of 10 mL/min by a gradient elution of methanol in 0.1M TEAB pH 7.5 (A = 0.1M TEAB, B = 0.1M TEAB in 50% aq. methanol, C = methanol) or without buffer. All final compounds were lyophilized from water. Mass spectra were recorded on LTQ Orbitrap XL (Thermo Fisher Scientific) using ESI ionization. NMR spectra were measured on Bruker AVANCE III™ HD 400 MHz (^{1}H at 400.1 MHz, ^{13}C at 100.6 MHz and ^{31}P at 162.0 MHz), Bruker Avance III™ HD 400 MHz Prodigy (^{1}H at 401.0 MHz, ^{13}C at 100.8 MHz and ^{31}P at 162.0 MHz), Bruker Avance III™ HD 500 MHz (^{1}H at 500.0 MHz, ^{13}C at 125.7 MHz and ^{31}P at 202.4 MHz) and JEOL JNM-ECZR 500 MHz (^{1}H at 500.2 MHz, ^{13}C at 125.8 MHz and ^{31}P at 202.5 MHz) spectrometers. D_2O (reference (dioxane) = ^{1}H 3.75 ppm, ^{13}C 69.3 ppm. Chemical shifts (in ppm, δ scale) were referenced to TMS as internal standard, coupling constants (*J*) are given in Hz. Purity of final compounds was determined via LC-MS analysis using Acquity UPLC coupled with Xevo G2 XS QTof (Waters). Chromatograms for all the compounds are shown in two ways – scan mode is the upper and single mass is in the bottom. Scan mode for majority of the compounds covers the range of 600–900 Da. For compounds with higher molecular mass than 900 Da, the scan is displayed in the range 300–600 Da, where masses $[M+2H]^{2+}$ are observed. Double peak at t_r = 3.21 min is the internal standard (IS). For compounds **14**, **50**, **51**, and **55**, Gradient B was used, so r_t of IS is shifted.

Table S1. Gradient programming for the LC-MS analysis of final compounds

GradientA:	time (min)	%C	%D
	0	90	10
	0.5	90	10
	5.5	5	95
	6	5	95
	6.3	90	10
	8.3	90	10
Flow:	0.5	ml/min	

GradientB:	time (min)	%C	%D
	0	95	5
	1	95	5
	5	5	95
	5.5	5	95
	6	95	5
	8	95	5

C = 0.1% formic acid, D = ACN + 0.1% formic acid;

2. General methods

General method A – removal of phosphonate methyl ester group

Methyl vinylphosphonate (1 mmol) was dissolved in 60% aqueous pyridine (20 mL) and the reaction mixture was stirred at 60 °C for 24 h. The reaction mixture was concentrated *in vacuo* at temperature below 40 °C, the residue was dissolved in ethanol (20 mL) and passed through a column of Dowex 50 H^+ form (5 g). The column was washed with EtOH (40 mL). The solvent was removed *in vacuo*. Product was obtained by column chromatography on silica gel using linear gradient of solvent system H1 (ethyl acetate, acetone, ethanol, water 4:1:1:1) in ethyl acetate.

General method B1 – Esterification of monomethyl vinylphosphonate using oxalyl chloride

Mono alkyl vinylphosphonate (1 mmol) was randered dry by co-evaporation with EtOH (10 mL/mmol) and toluene (10 mL), dissolved in DCM (3 mL) and cooled to –78 °C under argon atmosphere. Oxalyl chloride (2M in DCM, 0.3 mL) was slowly added and the reaction mixture was stirred at rt for 30 min. Catalytic ammount of DMF (50 µL) was added and the reaction mixture was stirred until gass evolution ceassed. Hydroxyderivative (1 mmol) was then added folowed by addition of triethylamine (1.1 mmol). The reaction mixture was stirred at rt for 12 h under argon atmosphere. The reaction mixture was extracted with sat. soln. $NaHCO_3$ (10 mL) and sat. soln. NaCl (10 mL). Organic phase was dried over Na_2SO_4 and concentrated *in vacuo*. Product was obtained by column chromatography using linear gradient of acetone in toluene or linear gradient of C1 in chloroform.

General method B2 – Esterification of monomethyl vinylphosphonate using TPSCl

Mono alkyl vinylphosphonate (1 mmol) and hydroxyderivative (2 mmol) was randered anhydrous by co-evaporation with DCM (2 × 10 mL) and dissolved in the same solvent (5 mL). Methylimidazole (3 mmol) and TPSCl (2 mmol) were added and the reaction mixture was stirred at rt under argon atmosphere for 24–48 h. Progress of the reaction was followed by TLC using mixture of acetone/toluene (1:1). The reaction mixture was diluted with DCM (10 mL) and washed subsequently with sat. soln $NaHCO_3$ (10 mL) and brine (10 mL). Organic phases were combined, dried over Na_2SO_4 and concentrated *in vacuo*. Product was obtained by column chromatography using linear gradient of acetone in toluene or linear gradient of C1 in chloroform.

General method C – Reaction of monoalkyl vinylphosphonate with α,ω-dibromoalkane

Mono alkyl vinylphosphonate (1 mmol) and tetrabutylammonium hydroxide (1 mmol) was randered anhydrous by co-evaporation with ethanol (2 × 10 mL) and DMF (10 mL) and dissolved in DMF (5 mL). α,ω-Dibromoalkane (0.36 mmol) was added and the reaction mixture was stirred under argon atmosphere at 90 °C for 24–48 h. Progress of the reaction was followed by TLC using mixture of acetone/toluene (1:1). The reaction mixture was concentrated *in vacuo* and product was obtained by column chromatography using linear gradient of acetone in toluene.

General method D – Michael addition

The mixture of vinylphosphonate dimer (1 mmol) and secondary amine (3 mmol) in n-butanol (50 mL/mmol) was stirred at 105 °C for 24–72 h in sealed flask. Progress of the reaction was followed by TLC using mixture C1. The reaction mixture was concentrated *in vacuo* and product was obtained by column chromatography using linear gradient of C1 in chloroform.

General method E – Removal of Boc protecting groups

Starting Boc derivative (1 mmol) was dissolved in 0.5M methanolic HCl (60 mL). The reaction mixture was stirred at rt for 24 h. The reaction mixture was concentrated *in vacuo* and product was obtained by precipitation from anhydrous ethyl acetate. If necessary, the final product is repurified by preparative HPLC on reversed phase using linear gradient of methanol in 0.1% aqueous TFA, followed by several codistillations with 0.5M methanolic hydrogen chloride.

General method F – Guanidination

1*H*-pyrazole-1-carboxamidine hydrochloride (3 mmol) was added to the mixture of LPPO (1 mmol) and ethyldiispropylamine (6 mmol) in DMF (10 mL) and stirred at rt for 24 h. The reaction mixture was concentrated *in vacuo* and purified by HPLC on reversed phase using linear gradient of methanol in 0.1% aqueous TFA, followed by several codistillations with 0.5M methanolic hydrogen chloride.

3. Synthetic procedures and characterizations

Isobutyl methyl vinylphosphonate (S1)

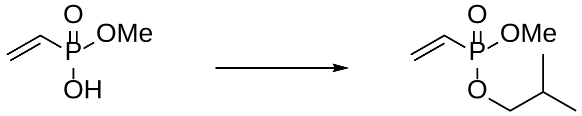

The title compound was prepared according to general method **B1** from mono methyl vinylphosphonate (4.89 g, 40.0 mmol) and isobutyl alcohol (3.71 mL, 40.0 mmol) in 38% yield (2.71 g, 15.2 mmol) as a clear oil.

^{1}H NMR (401 MHz, $CDCl_3$) δ 6.43–5.94 (m, 3H, C*H*=C*H*$_2$), 3.79 (tq, *J* = 6.6, 1.0 Hz, 2H, OC*H*$_2$), 3.72 (dt, *J* = 11.1, 0.8 Hz, 3H, OC*H*$_3$), 1.94 (dp, *J* = 13.2, 6.6 Hz, 1H, OCH$_2$C*H*), 0.93 (dd, *J* = 6.7, 1.0 Hz, 6H, C*H*$_3$).

^{13}C NMR (101 MHz, $CDCl_3$) δ 136.04 (d, *J* = 2.1 Hz, =*C*H$_2$), 125.31 (d, *J* = 184.7 Hz, P*C*H), 72.03 (d, *J* = 6.3 Hz, O*C*H$_2$), 52.47 (d, *J* = 6.1 Hz, O*C*H$_3$), 29.29 (d, *J* = 6.4 Hz, OCH$_2$*C*H), 18.81 (*C*H$_3$).

^{31}P{^{1}H} NMR (162 MHz, $CDCl_3$) δ 21.29.

IR ν_{max}($CHCl_3$) 3082 (w), 2959 (vs), 2925 (vs), 2873 (m), 2854 (m), 1615 (w), 1465 (m), 1400 (m), 1378 (m), 1277 (w), 1205 (m), 1033 (s), 986 (m), 851 (m), 726 (w), 612 (w).

HR-MS(APCI$^+$): For $C_7H_{16}O_3P$ $(M+H)^+$ *m/z* calculated 179.0837, found 179.0834.

^{1}H NMR spectrum of compound **S1**

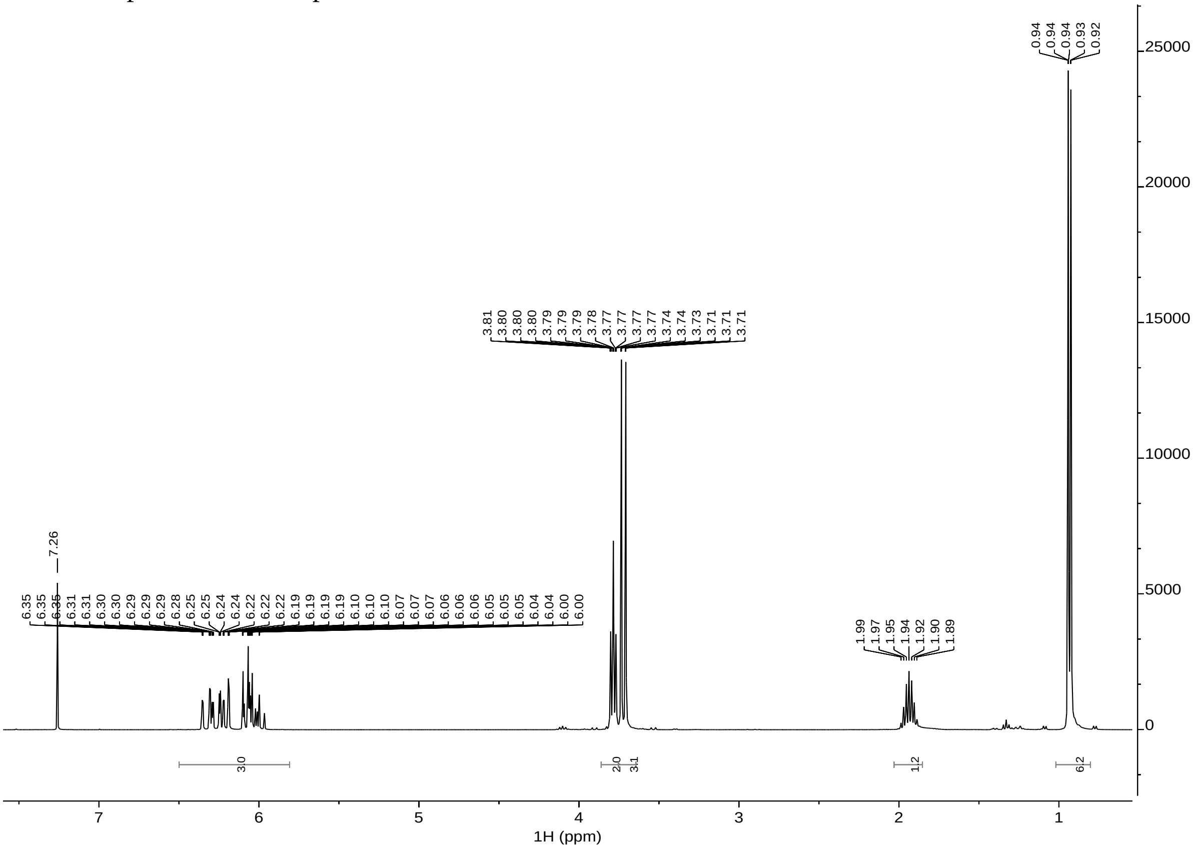

^{13}C NMR spectrum of compound **S1**

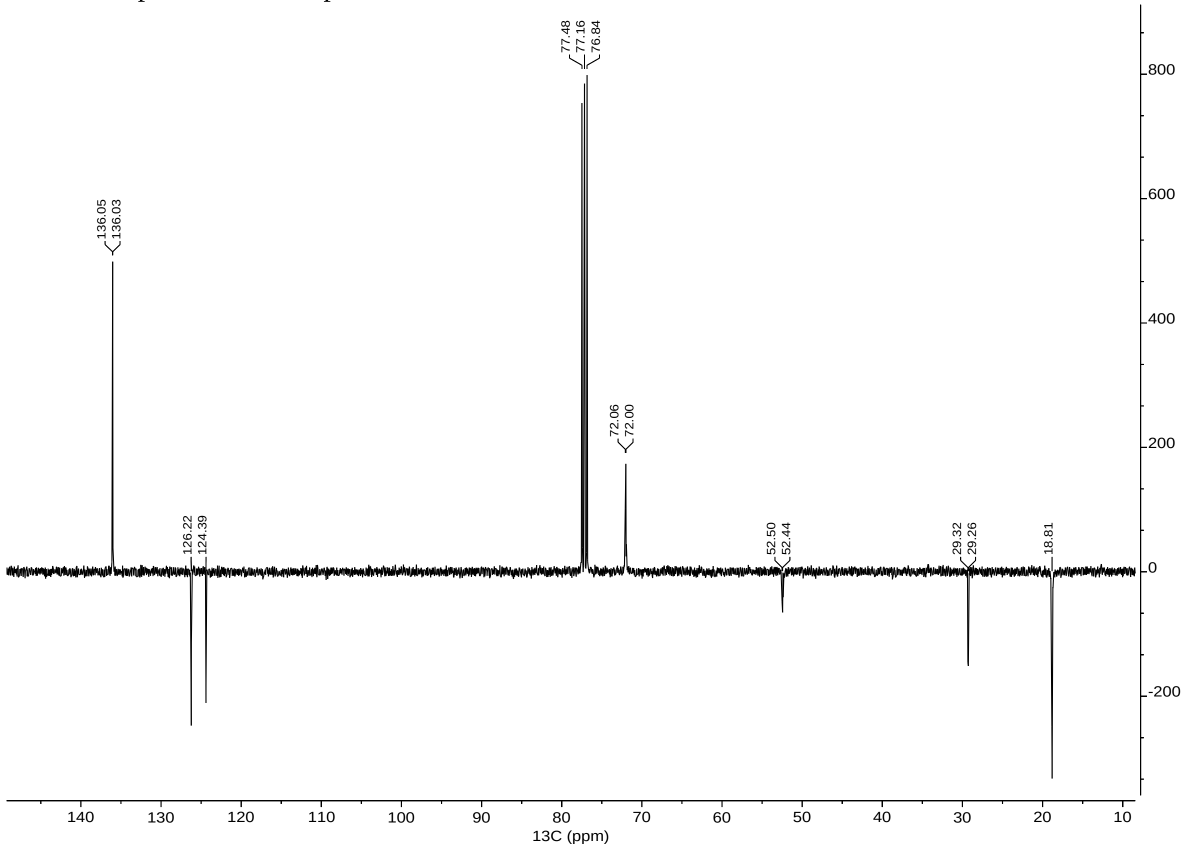

^{31}P{^{1}H} NMR spectrum of compound **S1**

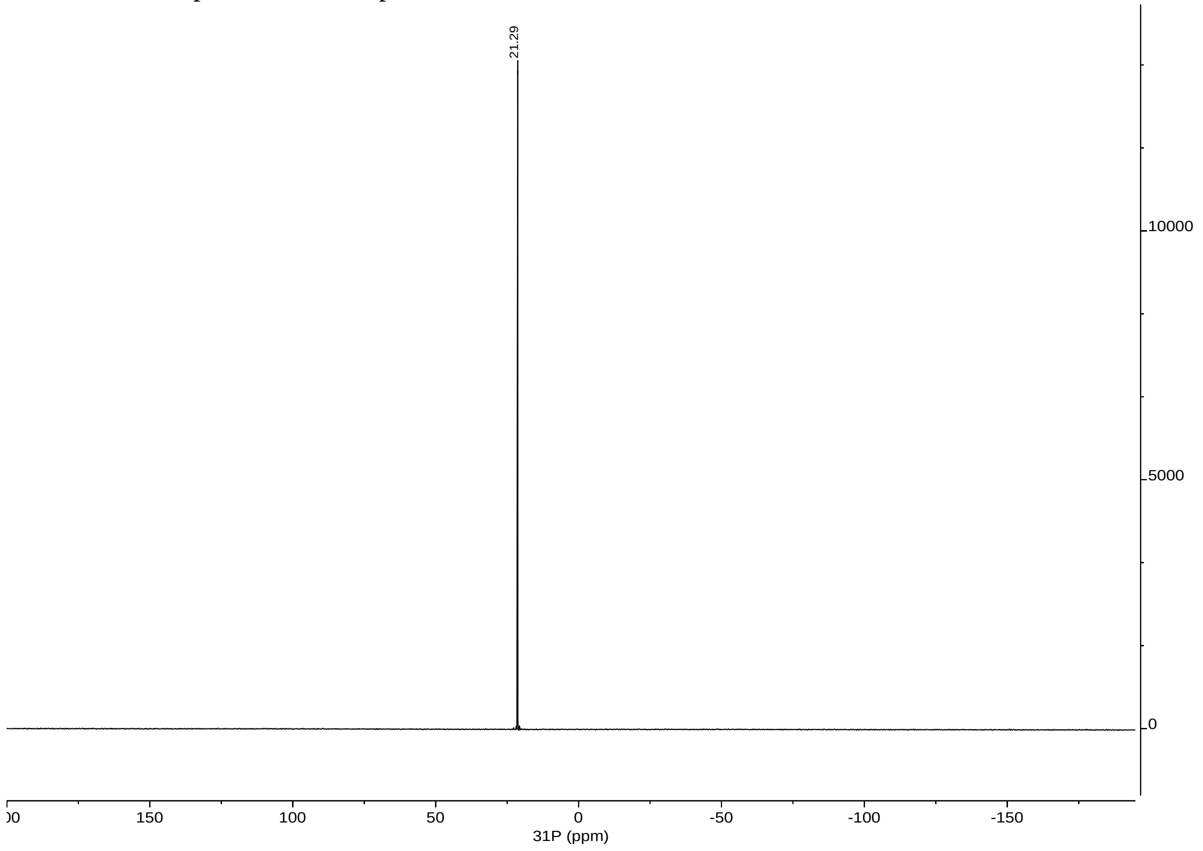

Butyl methyl vinylphosphonate (S2)

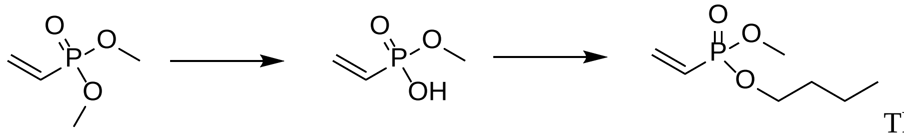

The title compound was prepared according to general methods **A** and **B2** from dimethyl vinylphosphonate (9.16 g, 67.3 mmol) in 50% overall yield (6.02 g, 33.8 mmol) as a colourless oil.

^{1}H NMR (400 MHz, $CDCl_3$): 6.36–5.97 (m, 3H, C*H*=C*H*$_2$), 4.07–4.0 (m, 2H, $CH_3(CH_2)_2CH_2C$*H*$_2$O), 3.72 (d, 3H, *J* = 12.0 Hz, OC*H*$_3$), 1.70–1.62 (m, 2H, CH_3CH_2C*H*$_2CH_2O$), 1.45–1.36 (m, 2H, CH_3C*H*$_2CH_2CH_2O$), 0.93 (t, 3H, *J* = 7.4 Hz, C*H*$_3(CH_2)_2CH_2O$).

^{13}C NMR (101 MHz, $CDCl_3$): 136.07 (d, *J* = 1.9 Hz, CH=*C*H$_2$), 125.36 (d, *J* = 183.4 Hz, *C*H=CH$_2$), 65.95 (d, *J* = 5.7 Hz, $CH_3CH_2CH_2$*C*H$_2$O), 52.47 (d, *J* = 5.4 Hz, OC*H*$_3$), 32.61 (d, *J* = 6.3 Hz, CH_3CH_2*C*H$_2CH_2O$), 18.87 (CH_3*C*H$_2CH_2CH_2O$), 13.73 (*C*H$_3(CH_2)_2CH_2O$).

^{31}P{^{1}H} NMR (162 MHz, $CDCl_3$): 21.13.

IR ν_{max} (KBr) 3091 (w), 3002 (m), 2964 (m), 2937 (m), 2876 (w), 2852 (w), 1614 (w), 1466 (w), 1448 (w), 1400 (w), 1242 (m), 1067 (m, sh), 1050 (m), 1026 (s), 852 (w).

HR-MS(CI$^+$): For $C_7H_{15}O_3P$ (M+H)$^+$ *m*/*z* calculated 179.08370, found 179.08360.

^{1}H NMR spectrum of compound **S2**

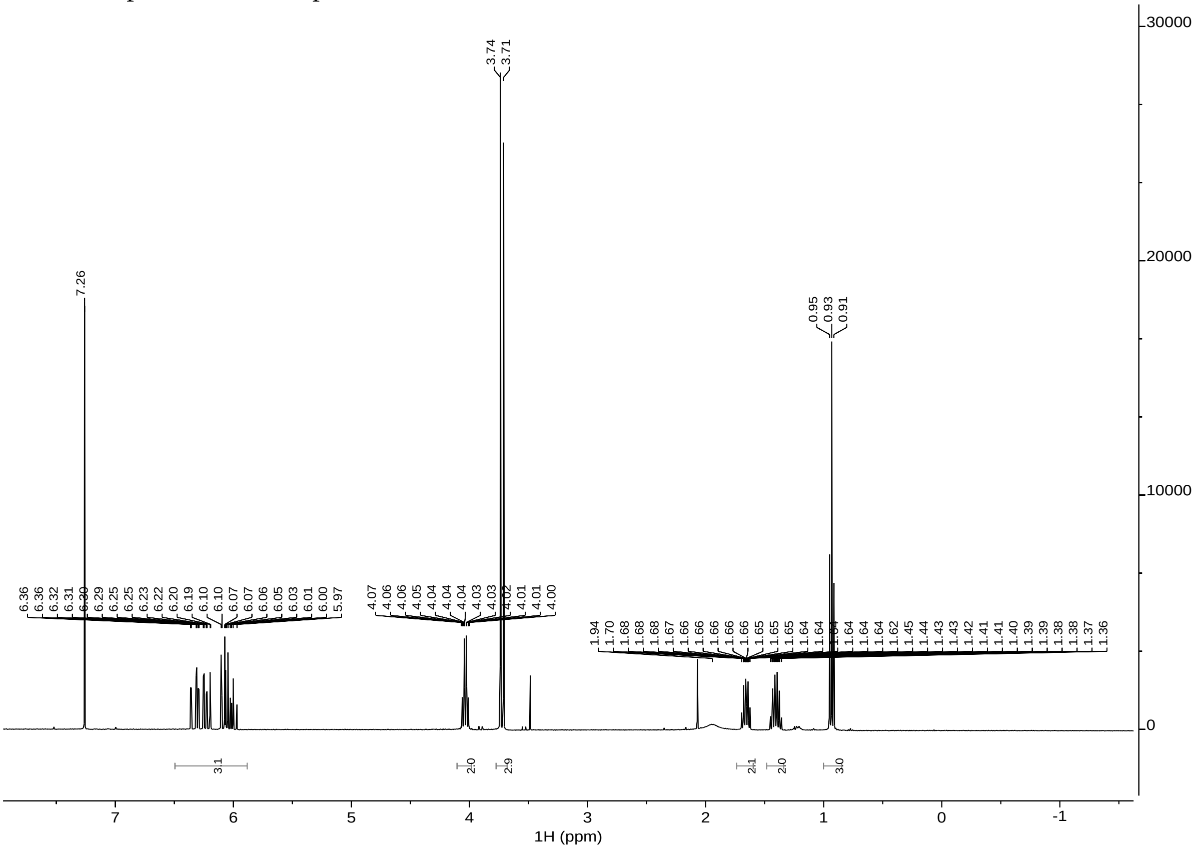

^{13}C NMR spectrum of compound **S2**

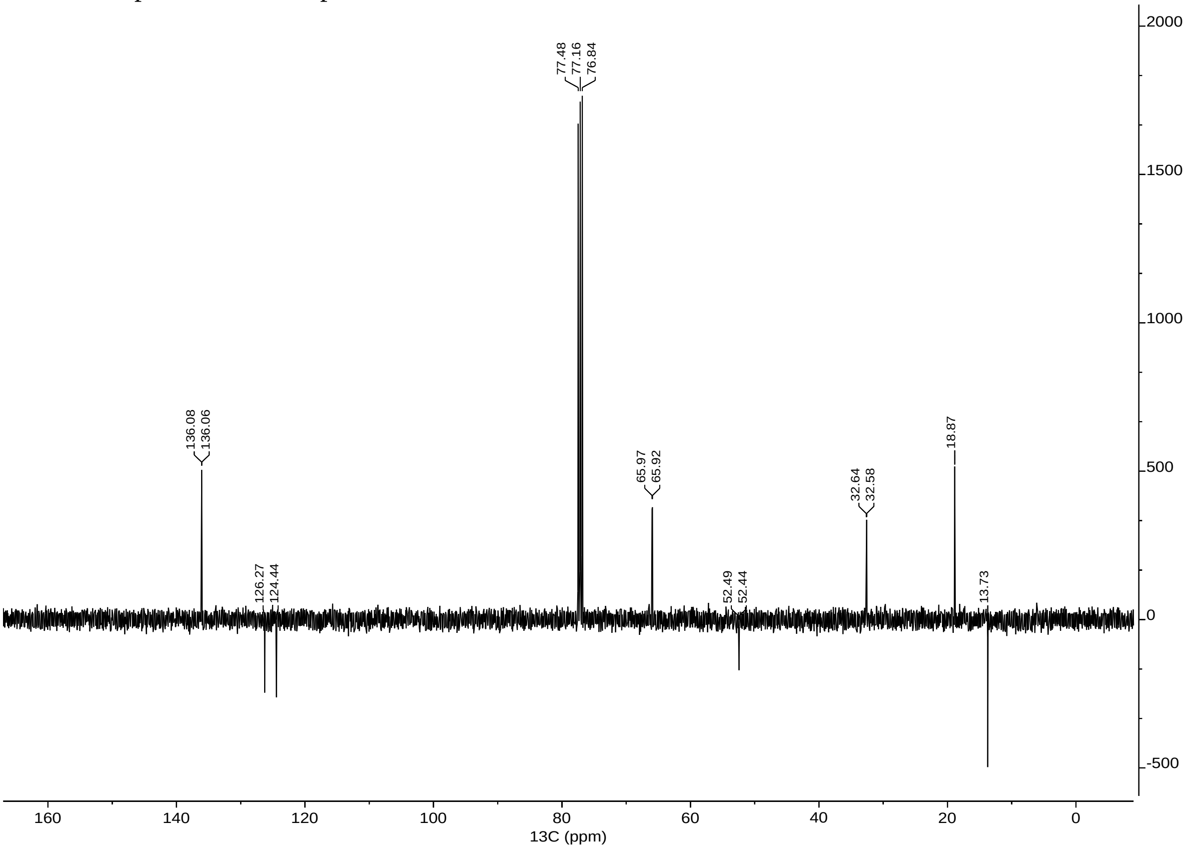

^{31}P{^{1}H} NMR spectrum of compound **S2**

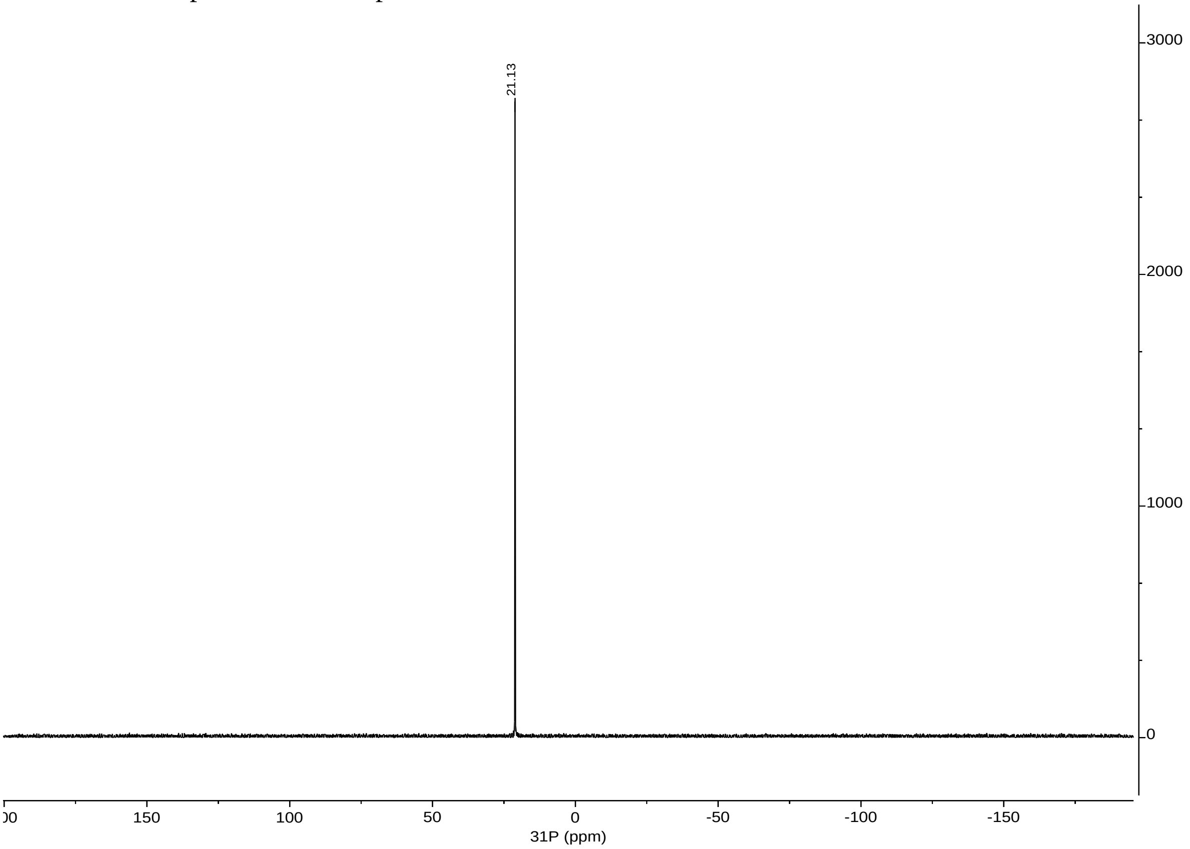

Methyl 4,4,4-trifluorobutyl vinylphosphonate (S3)

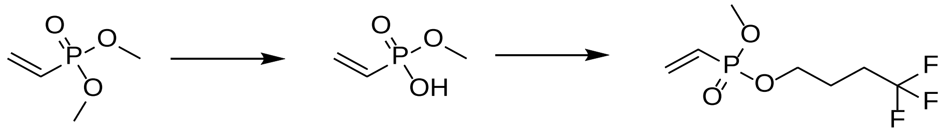

The title compound was prepared according to general methods **A** and **B2** from dimethyl vinylphosphonate (6.25 g, 45.9 mmol in 48% overall yield (5.1 g, 22.0 mmol) as a colourless oil.

^{1}H NMR (400 MHz, $CDCl_3$) δ 6.41–5.94 (m, 3H, C*H*=C*H*$_2$), 4.09 (q, *J* = 6.5 Hz , 2H, $CF_3CH_2CH_2C$*H*$_2$O), 3.73 (d, *J* = 11.1 Hz, 3H, OC*H*$_3$), 2.30–2.13 (m, 2H, CF_3C*H*$_2CH_2CH_2O$), 2.00–2.18 (m, 2H, CF_3CH_2C*H*$_2CH_2O$).

^{13}C NMR (101 MHz, $CDCl_3$) δ 136.77 (d, *J* = 2.2 Hz, CH=*C*H$_2$), 127.07 (q, *J* = 275.6 Hz, *C*F$_3$), 124.83 (d, *J* = 185.0 Hz, *C*H=CH$_2$), 64.18 (d, *J* = 5.5 Hz, $CF_3CH_2CH_2$*C*H$_2$O), 52.65 (d, *J* = 5.6 Hz, O*C*H$_3$), 30.45 (q, *J* = 29.4 Hz, CF_3*C*H$_2CH_2CH_2O$), 23.44 (dq, *J* = 6.2, 3.1 Hz, CF_3CH_2*C*H$_2CH_2O$).

^{31}P{^{1}H} NMR (162 MHz, $CDCl_3$): 21.53.

^{19}F NMR (376 MHz, $CDCl_3$) δ -66.36 (t, *J* = 10.8 Hz).

IR ν_{max} 3091 (vw), 2955 (m), 2853 (w), 1613 (w), 1475 (w, sh), 1460 (w, sh), 1451 (m), 1443 (w), 1400 (m), 1340 (m), 1256 (vs), 1239 (s), 1157 (vs), 1136 (vs), 1058–1021 (vs), 987 (m).

HR-MS(ESI$^+$): For $C_7H_{13}O_3F_3P$ $(M+H)^+$ *m/z* calculated 233.05489, found 233.05470.

^{1}H NMR spectrum of compound **S3**

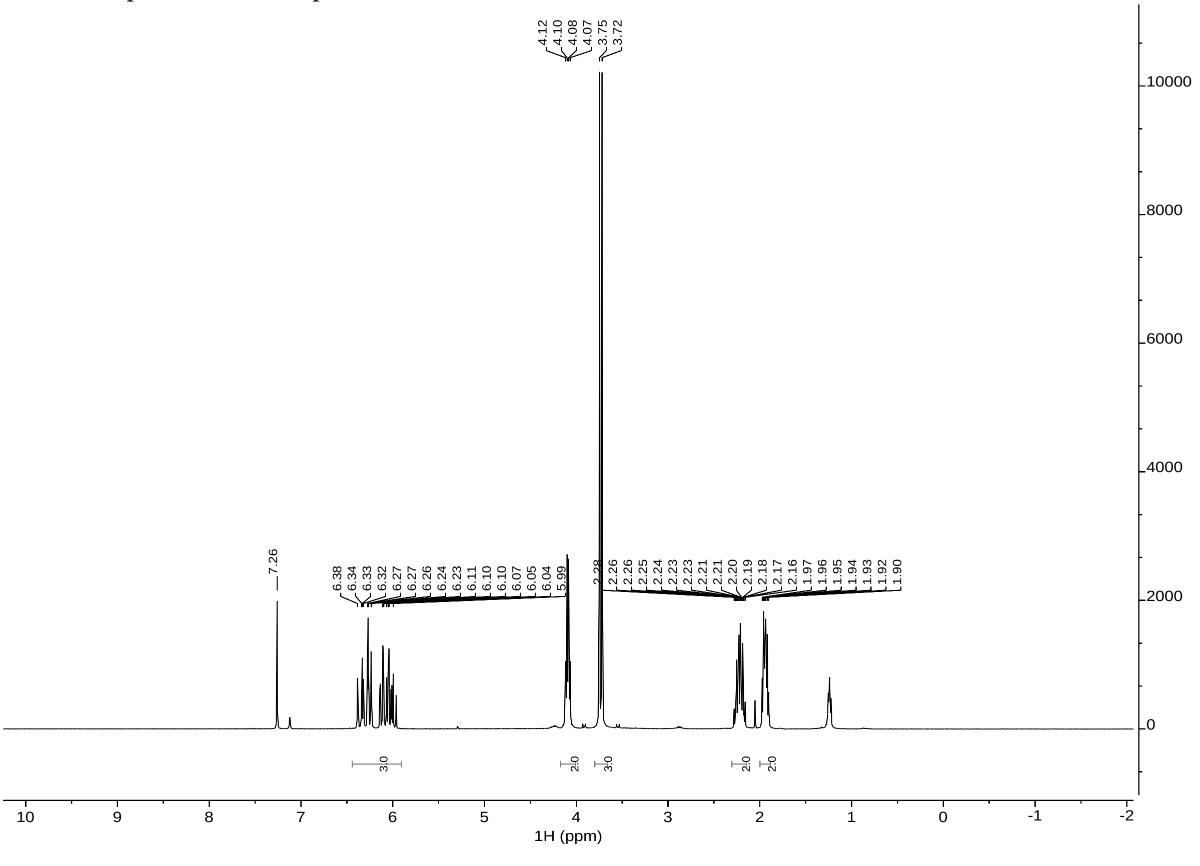

^{13}C NMR spectrum of compound **S3**

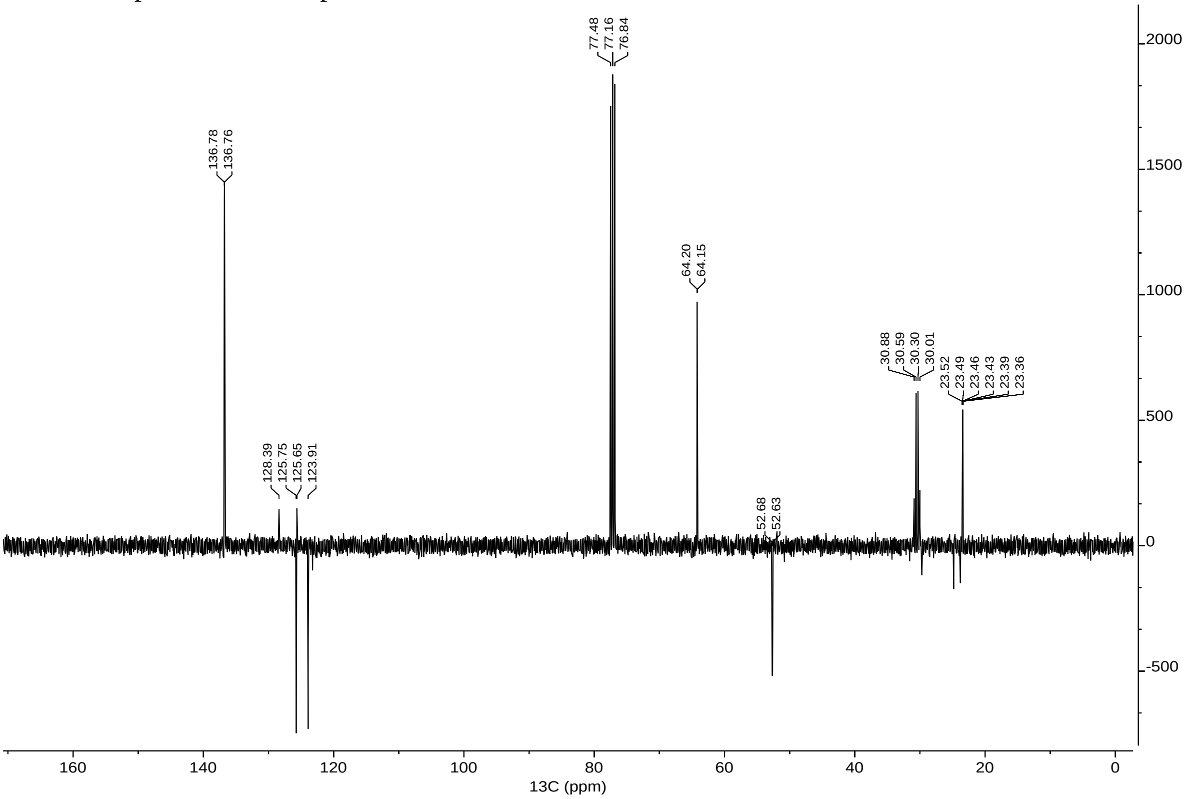

^{31}P{^{1}H} NMR spectrum of compound **S3**

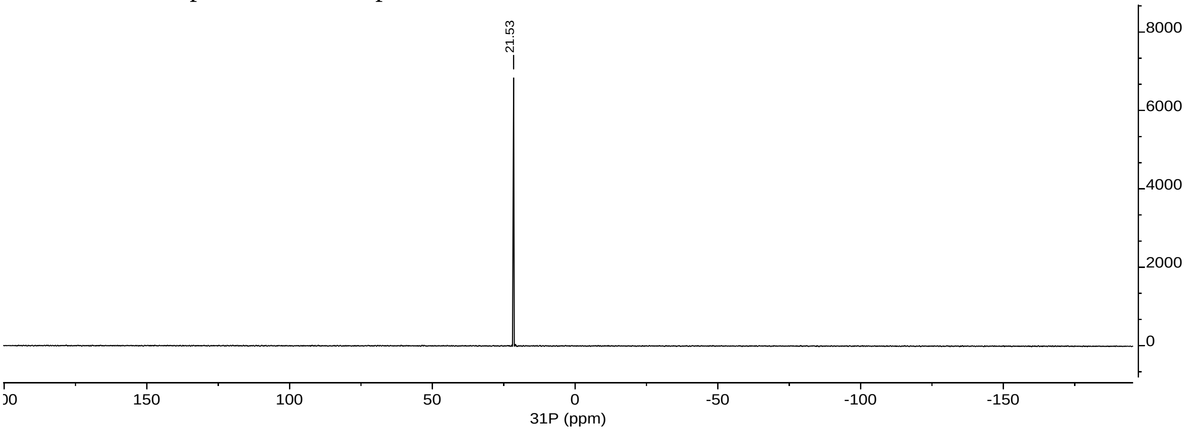

^{19}F NMR spectrum of compound **S3**

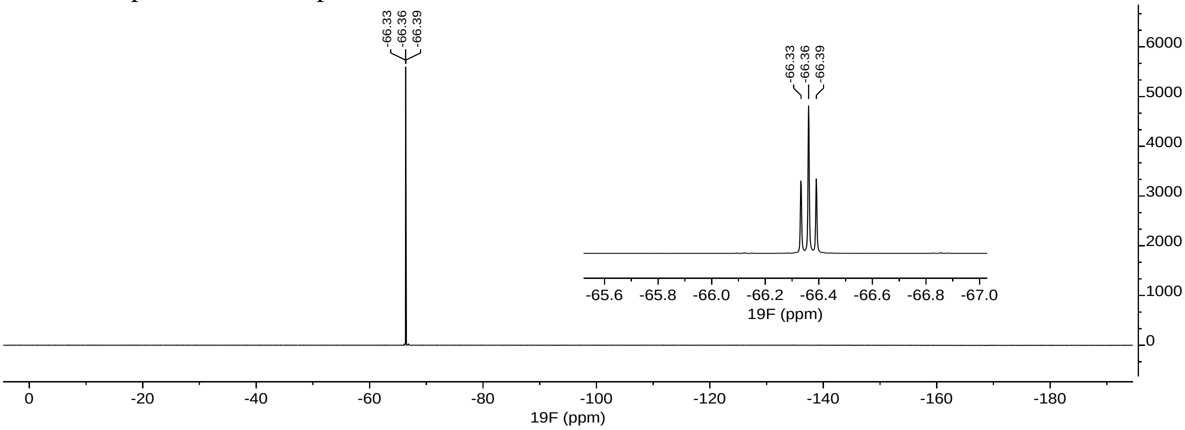

Methyl pentyl vinylphosphonate (S4)

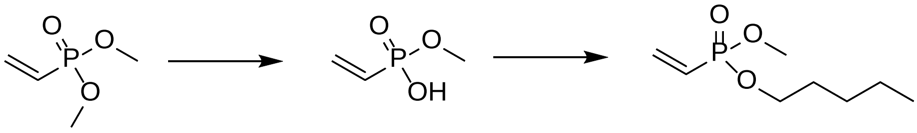

The title compound was prepared according to general methods **A** and **B2** from dimethyl vinylphosphonate (8.52 g, 62.6 mmol) in 52% overall yield (5.80 g, 32.5 mmol) as a colourless oil.

^{1}H NMR (400 MHz, $CDCl_3$): 6.36–5.97 (m, 3H, C*H*=C*H*$_2$), 4.05–3.98 (m, 2H, $CH_3(CH_2)_2CH_2C$*H*$_2$O), 3.72 (d, 3H, *J* = 8.0 Hz, OC*H*$_3$), 1.71–1.64 (m, 2H, $CH_3(CH_2)_2C$*H*$_2CH_2O$), 1.39–1.28 (m, 4H, $CH_3(C$*H*$_2)_2CH_2CH_2O$), 0.93–0.87 (m, 3H, C*H*$_3(CH_2)_3CH_2O$).

^{13}C NMR (101 MHz, $CDCl_3$): 136.04 (d, *J* = 2.2 Hz, CH=*C*H$_2$), 125.35 (d, *J* = 183.5 Hz, *C*H=CH$_2$), 66.08 (d, *J* = 5.9 Hz, $CH_3(CH_2)_2CH_2$*C*H$_2$O), 52.31 (d, *J* = 5.7 Hz, OC*H*$_3$), 30.27 (d, *J* = 6.2 Hz, $CH_3(CH_2)_2$*C*H$_2CH_2O$), 27.75, 22.35 (CH_3(*C*H$_2)_2CH_2CH_2O$), 14.07 (*C*H$_3(CH_2)_3CH_2O$).

^{31}P{^{1}H} NMR (162 MHz, $CDCl_3$): 21.11.

IR ν_{max} (KBr) 3090 (vw), 2959 (s), 2934 (s), 2874 (m), 2861 (m), 1613 (w), 1467 (w), 1461 (w), 1435 (vw), 1400 (m), 1381 (w), 1280 (w), 1242 (s), 1185 (w), 1057 (vs), 1042 (vs), 1022 (vs), 1004 (s), 989 (s), 856 (w), 814 (m).

HR-MS(APCI$^+$): For $C_8H_{18}O_3P$ $(M+H)^+$ *m/z* calculated 193.09881, found 193.09872.

^{1}H NMR spectrum of compound **S4**

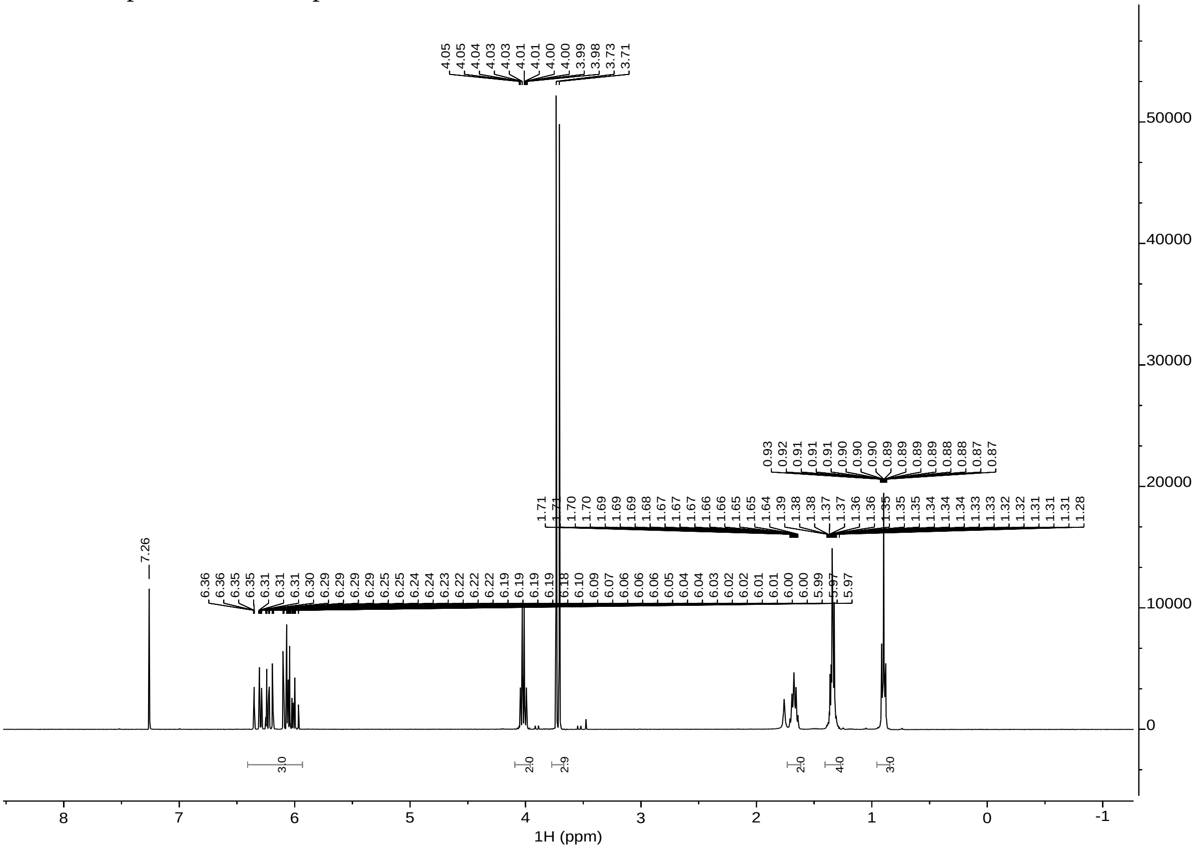

^{13}C NMR spectrum of compound **S4**

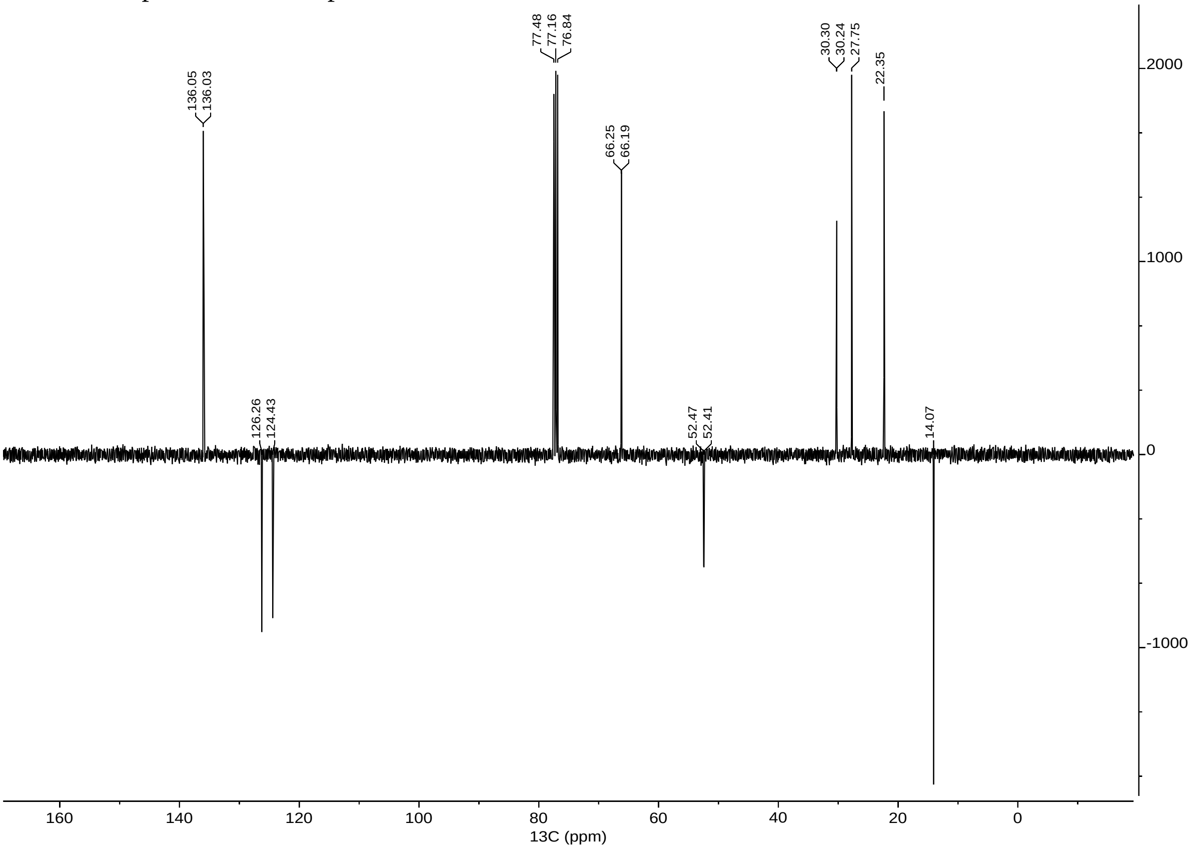

^{31}P{^{1}H} NMR spectrum of compound **S4**

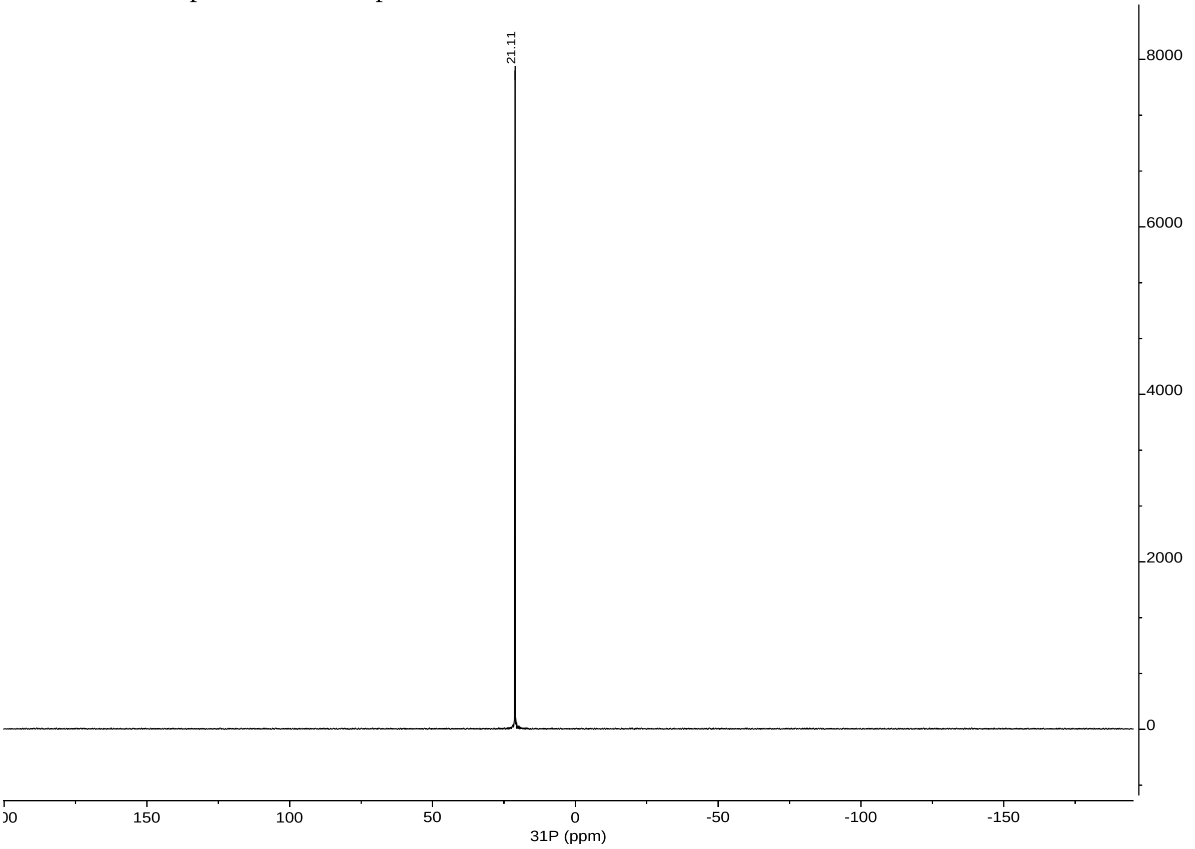

Hexyl methyl vinylphosphonate (S5)

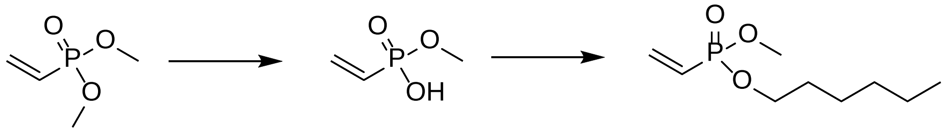

The title compound was prepared according to general methods **A** and **B2** from dimethyl vinylphosphonate (8.81 g, 64.7 mmol) in 42% overall yield (5.59 g, 27.1 mmol) as a colourless oil.

^{1}H NMR (400 MHz, CDCl$_3$): 6.36–5.97 (m, 3H, C*H*=C*H*$_2$), 4.05–3.98 (m, 2H, CH$_3$(CH$_2$)$_3$CH$_2$C*H*$_2$O), 3.72 (d, 3H, *J* = 8.1 Hz, OC*H*$_3$), 1.70–1.63 (m, 2H, CH$_3$(CH$_2$)$_3$C*H*$_2$CH$_2$O), 1.22–1.40 (m, 6H, CH$_3$(C*H*$_2$)$_3$CH$_2$CH$_2$O), 0.90–0.86 (m, 3H, C*H*$_3$(CH$_2$)$_4$CH$_2$O).

^{13}C NMR (101 MHz, CDCl$_3$): 136.01 (d, *J* = 2.1 Hz, CH=*C*H$_2$), 125.40 (d, *J* = 184.2 Hz, *C*H=CH$_2$), 66.23 (d, *J* = 5.9 Hz, CH$_3$(CH$_2$)$_3$CH$_2$*C*H$_2$O), 52.44 (d, *J* = 5.5 Hz, OC*H*$_3$), 30.57 (d, *J* = 6.4 Hz, CH$_3$(CH$_2$)$_3$*C*H$_2$CH$_2$O), 31.46, 25.30, 22.66 (CH$_3$(*C*H$_2$)$_3$CH$_2$CH$_2$O), 14.12 (*C*H$_3$(CH$_2$)$_4$CH$_2$O).

^{31}P{^{1}H} NMR (162 MHz, CDCl$_3$): 21.10.

IR ν_{max} (KBr) 3091 (vw), 2958 (s), 2933 (s), 2873 (w), 2860 (w), 1614 (w), 1468 (w), 1461 (w), 1435 (w), 1400 (m), 1380 (w), 1279 (w), 1243 (s), 1185 (w), 1061 (s), 1051 (s), 1041 (s), 1017 (s), 988 (s), 819 (s), 602 (w).

HR-MS(APCI$^+$): For $C_9H_{20}O_3P$ (M+H)$^+$ *m/z* calculated 207.11446, found 207.11441.

^{1}H NMR spectrum of compound **S5**

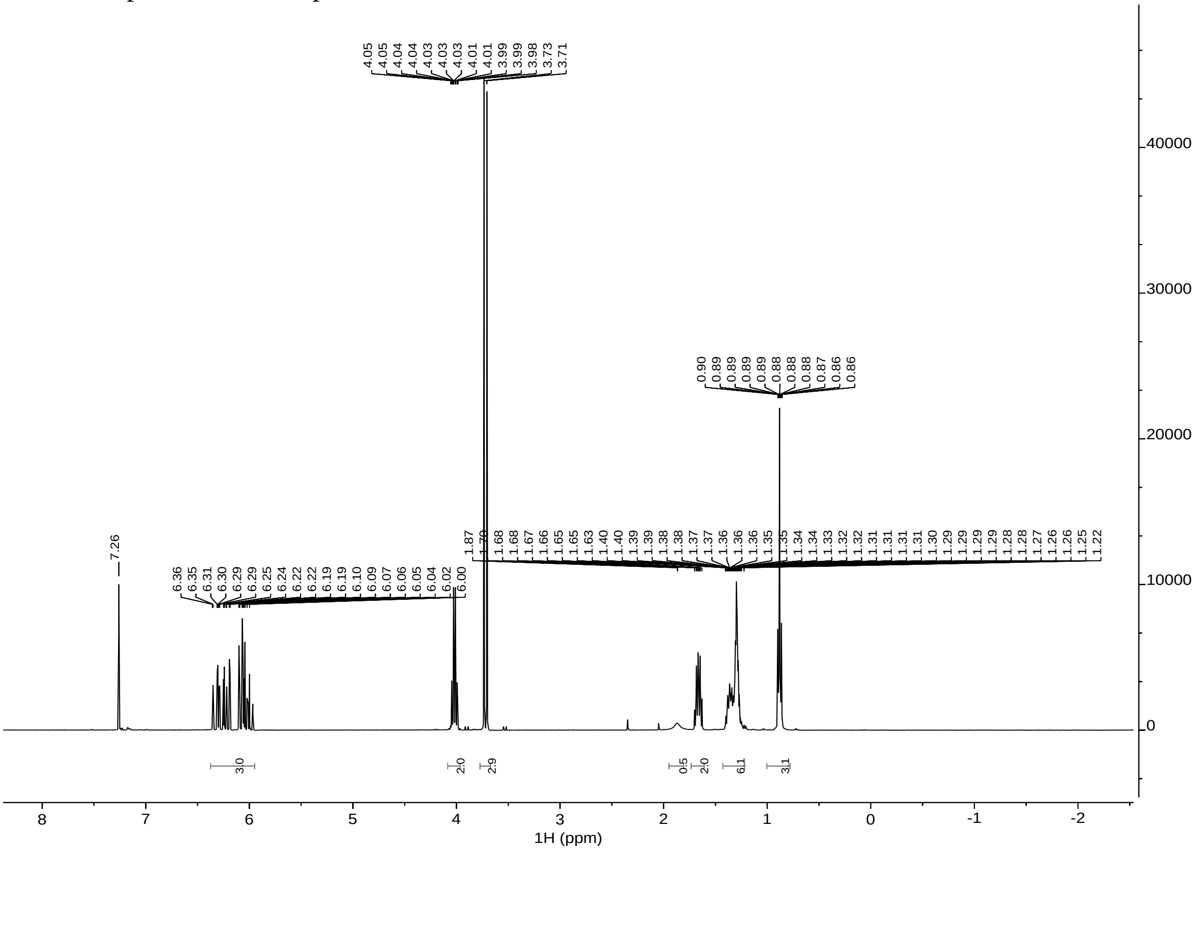

^{13}C NMR spectrum of compound **S5**

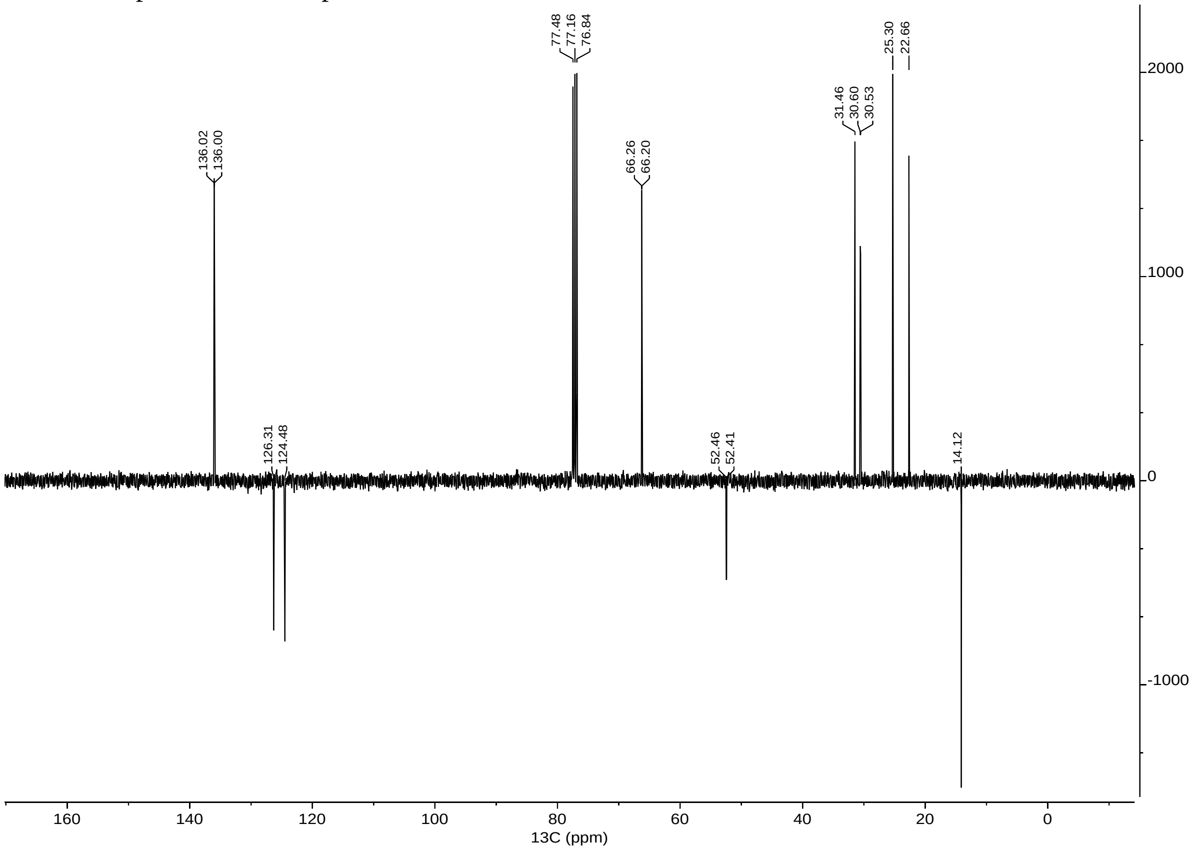

$^{31}P\{^1H\}$ NMR spectrum of compound **S5**

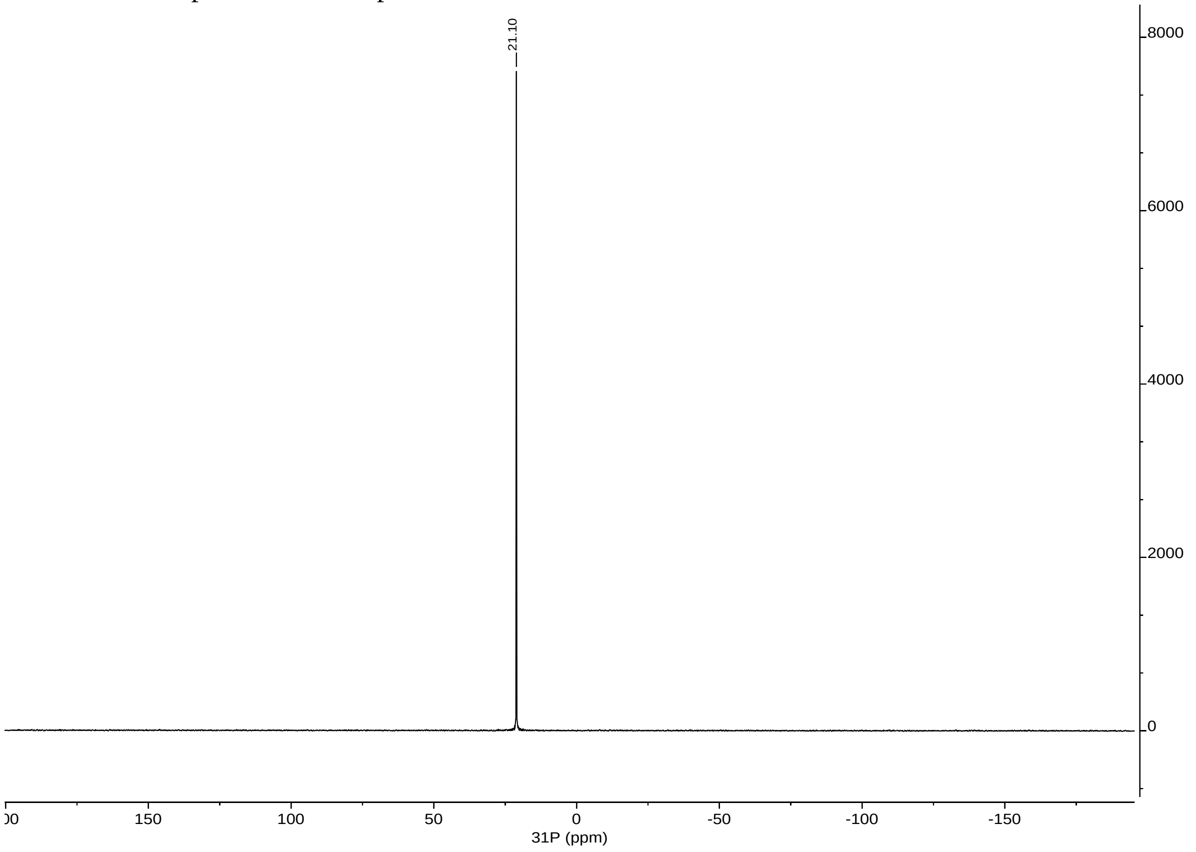

(*Z*)-Hept-3-en-1-yl methyl vinylphosphonate (S6)

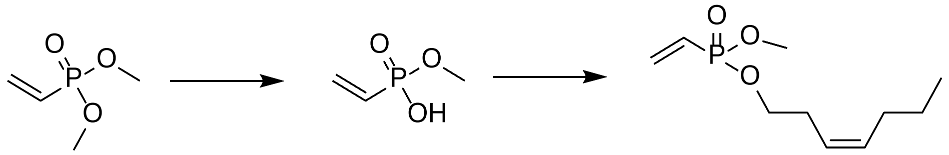

The title compound was prepared according to general methods **A** and **B2** from dimethyl vinylsphosphonate (7.73 g, 56.8 mmol) in 42% overall yield (5.19 g, 23.8 mmol) as a colourless oil.

^{1}H NMR (400 MHz, $CDCl_3$): 6.36–5.99 (m, 3H, C*H*=C*H*$_2$), 5.55–5.48 (m, 1H, $CH_3(CH_2)_2$C*H*CHCH$_2$CH$_2$O), 5.39–5.32 (m, 1H, $CH_3(CH_2)_2$CHC*H*CH$_2$CH$_2$O), 4.05–3.98 (m, 2H, CH$_3$CH$_2$CH$_2$CHCHCH$_2$C*H*$_2$O), 3.72 (d, 3H, *J* = 11.3 Hz, OC*H*$_3$), 2.46–2.40 (m, 2H, CH$_3$CH$_2$CH$_2$CHCHC*H*$_2$CH$_2$O), 2.05–1.98 (m, 2H, CH$_3$CH$_2$C*H*$_2$CHCHCH$_2$CH$_2$O), 1.42–1.32 (m, 2H, CH$_3$C*H*$_2$CH$_2$CHCHCH$_2$CH$_2$O), 0.89 (m, 3H, C*H*$_3$(CH$_2$)$_2$CHCHCH$_2$CH$_2$O).

^{13}C NMR (101 MHz, $CDCl_3$): 136.08 (d, *J* = 2.0 Hz, CH=*C*H$_2$), 133.25 (CH$_3$(CH$_2$)$_2$*C*HCHCH$_2$CH$_2$O), 125.34 (d, *J* = 184.5 Hz, *C*H=CH$_2$), 124.0 (CH$_3$(CH$_2$)$_3$CH*C*HCH$_2$CH$_2$O), 65.52 (d, *J* = 5.9 Hz, CH$_3$(CH$_2$)$_2$CHCHCH$_2$*C*H$_2$O), 52.47 (d, *J* = 5.7 Hz, O*C*H$_3$), 29.53 CH$_3$CH$_2$*C*H$_2$CHCHCH$_2$CH$_2$O), 28.83 (d, *J* = 6.3 Hz, CH$_3$(CH$_2$)$_2$CHCH*C*H$_2$CH$_2$O), 22.80 (CH$_3$*C*H$_2$CH$_2$CHCHCH$_2$CH$_2$O), 13.87 (*C*H$_3$(CH$_2$)$_2$CHCHCH$_2$CH$_2$O).

^{31}P{^{1}H} NMR (162 MHz, $CDCl_3$): 21.37.

IR ν_{max} (KBr) 3086 (w), 3011 (m), 2959 (s), 2931 (m), 2872 (m), 1655 (w), 1465 (m), 1399 (m), 1379 (w), 1253 (s), 1184 (m), 1048 (s), 1014 (vs), 989 (s, sh), 815 (s), 723 (m), 605 (m).

HR-MS(ESI$^+$): For $C_9H_{20}O_3NaP$ $(M+Na)^+$ *m/z* calculated 241.09640, found 241.09645.

^{1}H NMR spectrum of compound **S6**

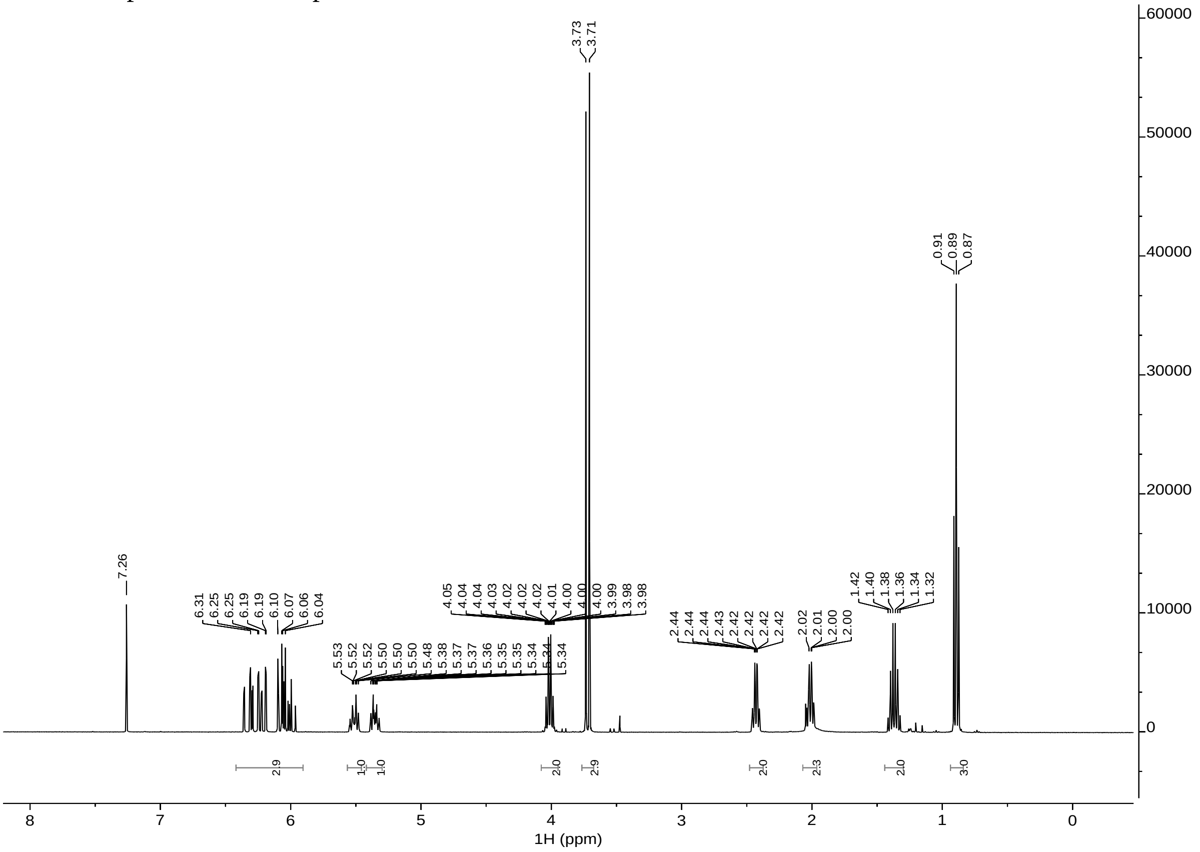

^{13}C NMR spectrum of compound **S6**

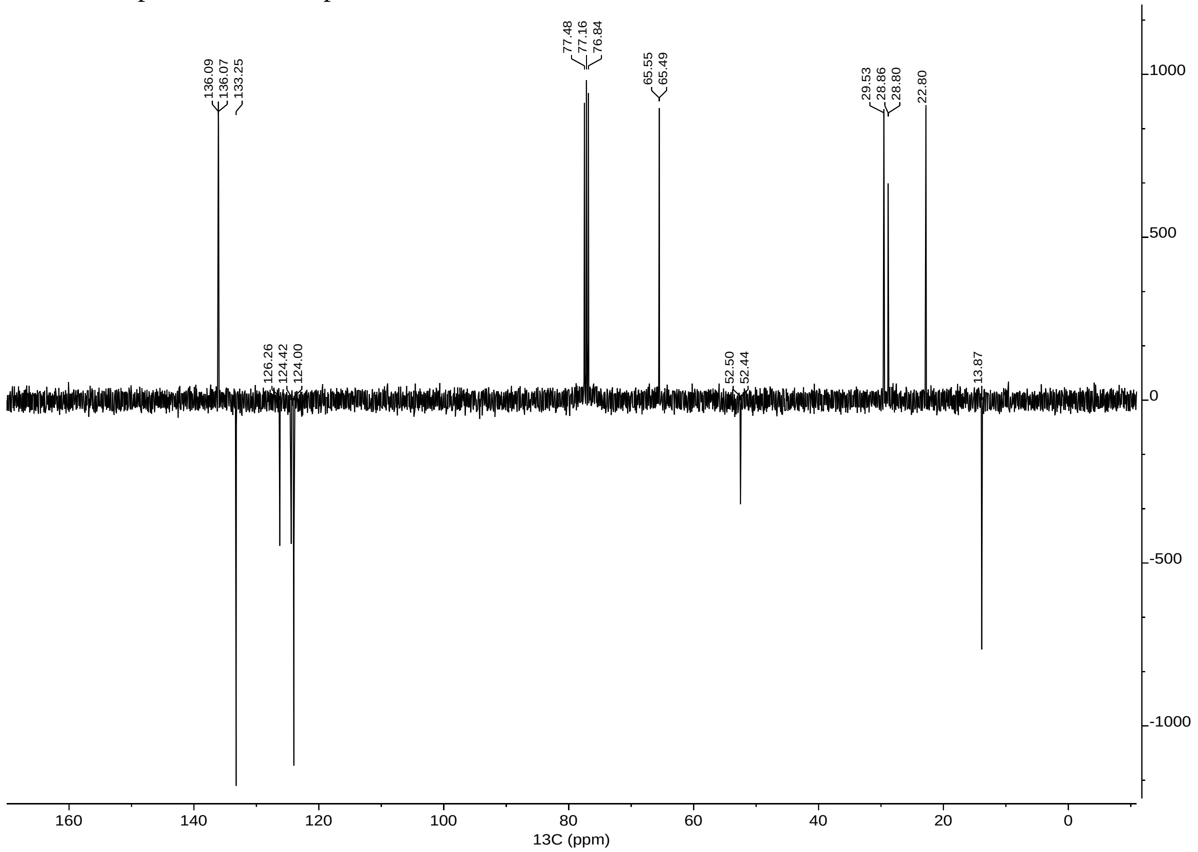

^{31}P{^{1}H} NMR spectrum of compound **S6**

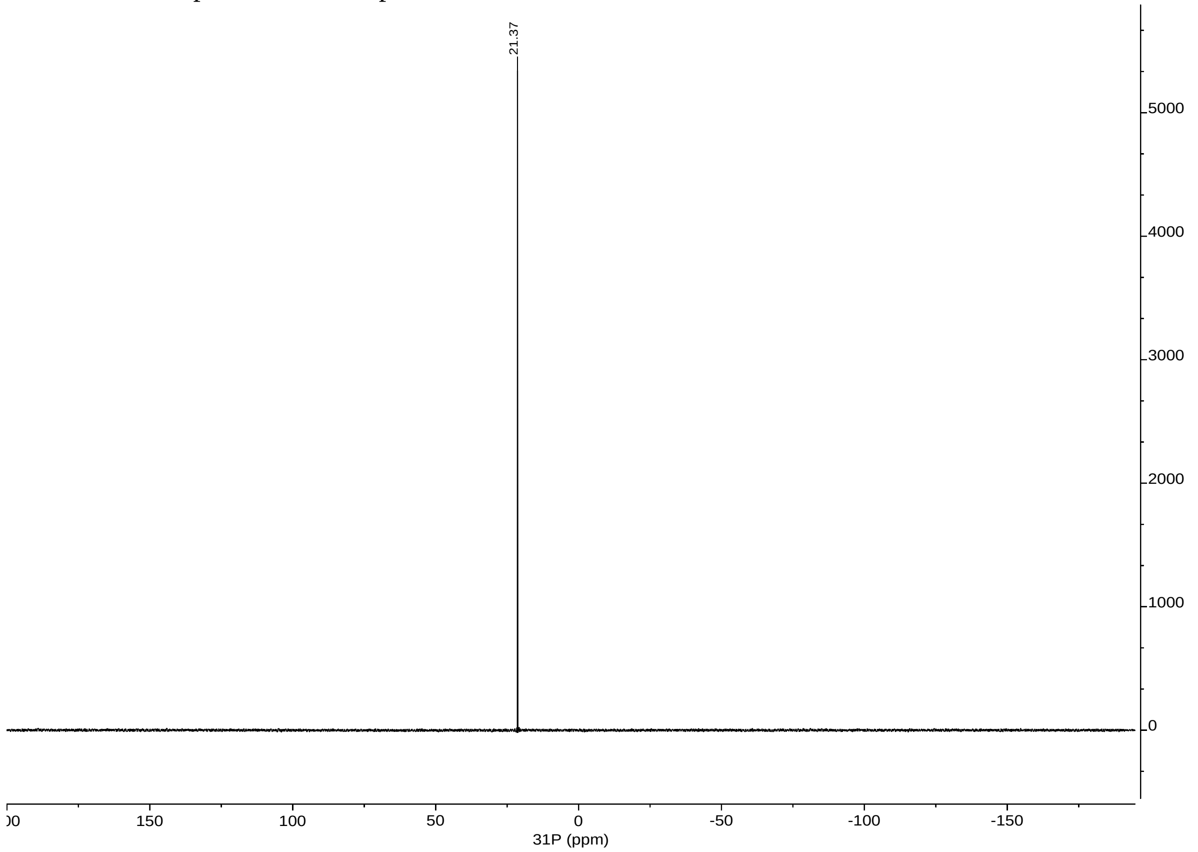

Cyclopentylmethyl methyl vinylphosphonate (S7)

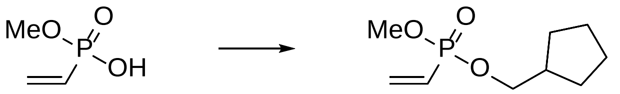

The title compound was prepared according to general method **B2** from mono methyl vinylphosphonate (8.53 g, 69.9 mmol) and cyclopentylmethanol (11.2 mL, 99.8 mmol) in 86% yield (12.3 g, 60.2 mmol) as a clear oil.

^{1}H NMR (401 MHz, $CDCl_3$) δ 6.29–5.89 (m, 3H, C*H*=C*H*$_2$), 3.83 (td, *J* = 6.9, 0.7 Hz, 2H, OC*H*$_2$), 3.65 (d, *J* = 11.1 Hz, 3H, OC*H*$_3$), 2.16 (hept, *J* = 7.4 Hz, 1H, *H*-1-cyclopent), 1.74–1.62 (m, 2H, *H*-2a,5a-cyclopent), 1.58–1.41 (m, 4H, *H*-3,4-cyclopent), 1.27–1.14 (m, 2H, *H*-2b,5b-cyclopent).

^{13}C NMR (101 MHz, $CDCl_3$) δ 135.85 (d, *J* = 2.0 Hz, =*C*H$_2$), 125.23 (d, *J* = 184.2 Hz, P*C*H), 69.71 (d, *J* = 6.0 Hz, O*C*H$_2$), 52.26 (d, *J* = 5.7 Hz, O*C*H$_3$), 39.96 (d, *J* = 6.4 Hz, OCH$_2$*C*H), 28.96 (d, *J* = 4.3 Hz, *C*H$_2$-2,5-cyclopent), 25.34 (*C*H$_2$-3,4-cyclopent).

^{31}P{^{1}H} NMR (162 MHz, $CDCl_3$) δ 21.14.

IR ν_{max} ($CHCl_3$) 3090 (w), 2956 (vs), 2871 (m), 2854 (m), 1613 (w), 1466 (m), 1453 (m), 1400 (m), 1279 (m), 1241 (vs), 1055 (vs), 1024 (vs), 1010 (vs), 989 (s), 815 (s).

HR-MS(ESI$^+$): For $C_9H_{17}O_3NaP$ (M+Na)$^+$ *m/z* calculated 227.08075, found 227.08049.

^{1}H NMR spectrum of compound **S7**

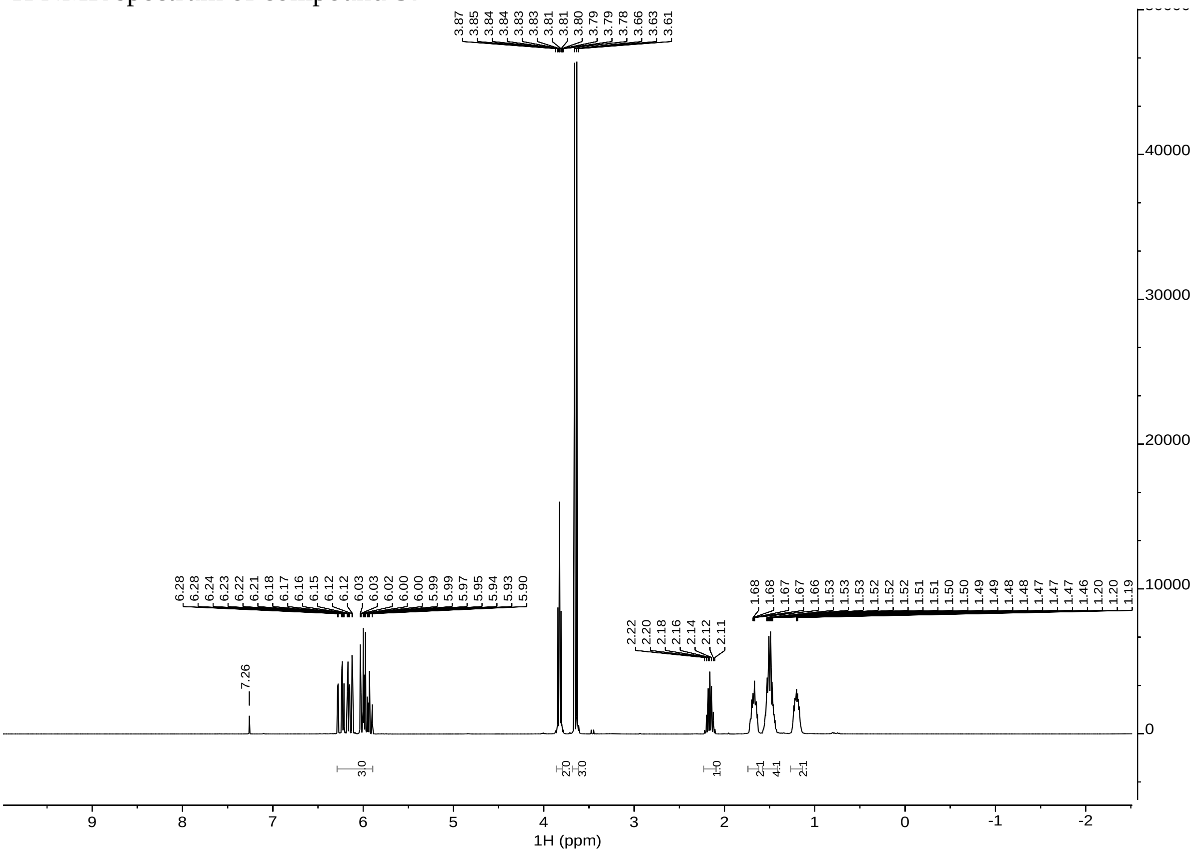

^{13}C NMR spectrum of compound **S7**

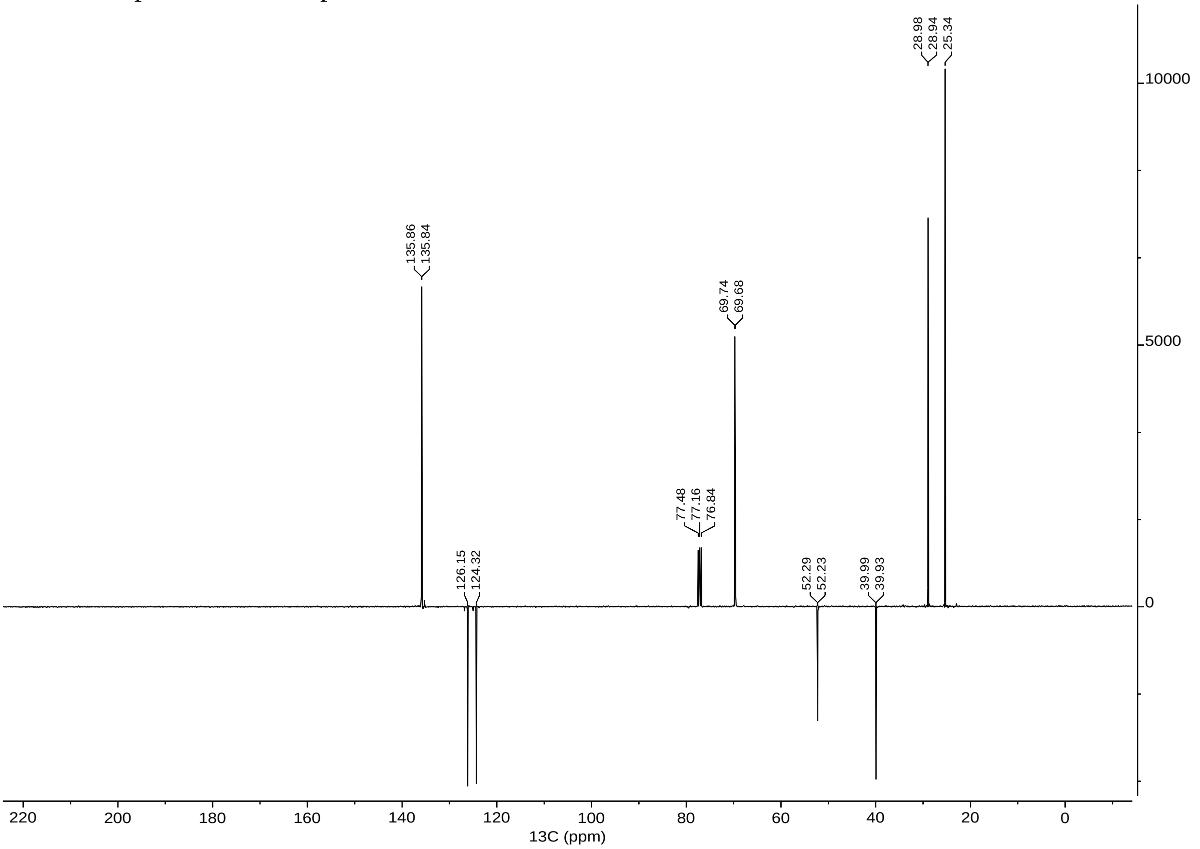

^{31}P{^{1}H} NMR spectrum of compound **S7**

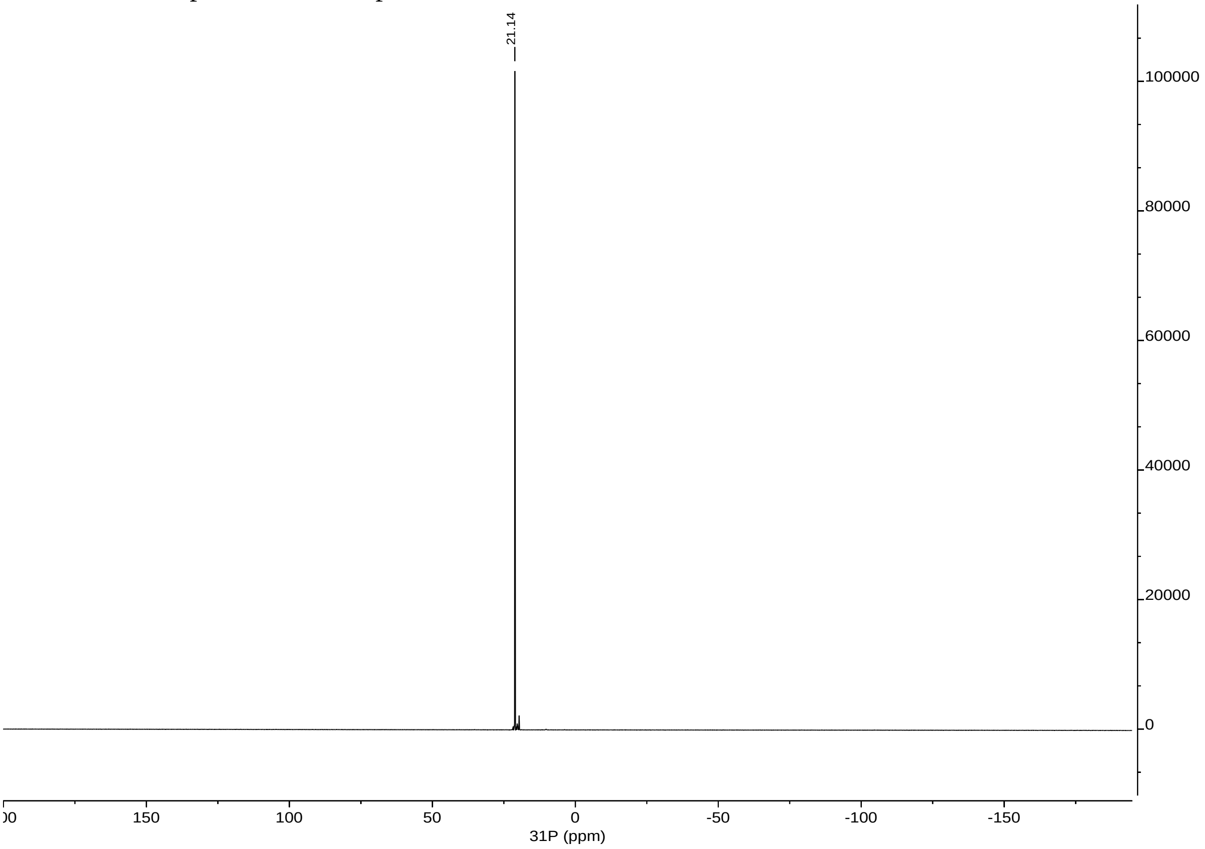

Heptyl methyl vinylphosphonate (S8)

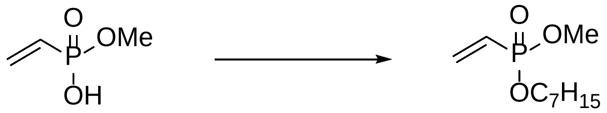

The title compound was prepared according to general method **B1** from mono methyl vinylphosphonate (9.65 g, 79.1 mmol) and *n*-heptanol (11.2 mL, 79.1 mmol) in 83% yield (14.5 g, 65.7 mmol) as a clear oil.

^{1}H NMR (401 MHz, $CDCl_3$) δ 6.39–5.95 (m, 3H, C*H*=C*H*$_2$), 4.02 (q, *J* = 6.9 Hz, 2H, OC*H*$_2$), 3.72 (d, *J* = 11.1 Hz, 3H, OC*H*$_3$), 1.67 (dt, *J* = 14.7, 6.7 Hz, 2H, OCH_2C*H*$_2$), 1.40–1.23 (m, 8H, (C*H*$_2$)$_4$$CH_3$), 0.92–0.84 (m, 3H, CH_2C*H*$_3$).

^{13}C NMR (101 MHz, $CDCl_3$) δ 136.09 (=*C*H_2), 125.33 (d, *J* = 184.1 Hz, P*C*H), 66.24 (d, *J* = 6.2 Hz, O*C*H_2), 52.45 (d, *J* = 5.6 Hz, O*C*H_3), 31.83 (*C*$H_2CH_2CH_3$), 30.59 (d, *J* = 6.2 Hz, OCH_2*C*H_2), 28.94, 25.57 (O(CH_2)$_2$(*C*H_2)$_2$), 22.69 (*C*H_2CH_3), 14.18 (CH_2*C*H_3).

^{31}P{^{1}H} NMR (162 MHz, $CDCl_3$) δ 21.37.

IR ν_{max}(film) 3086 (w), 2956 (s), 2930 (s), 2858 (m), 1613 (w), 1468 (m), 1398 (m), 1380 (w), 1254 (s), 1185 (m), 1049 (s), 1014 (vs), 987 (s), 858 (m, sh), 811 (s), 725 (m).

HR-MS(APCI$^+$): For $C_{10}H_{22}O_3P$ $(M+H)^+$ *m/z* calculated 221.13011, found 221.13002. (ESI$^+$): For $C_{10}H_{21}O_3NaP$ $(M+Na)^+$ *m/z* calculated 243.11205, found 243.11183.

^{1}H NMR spectrum of compound **S8**

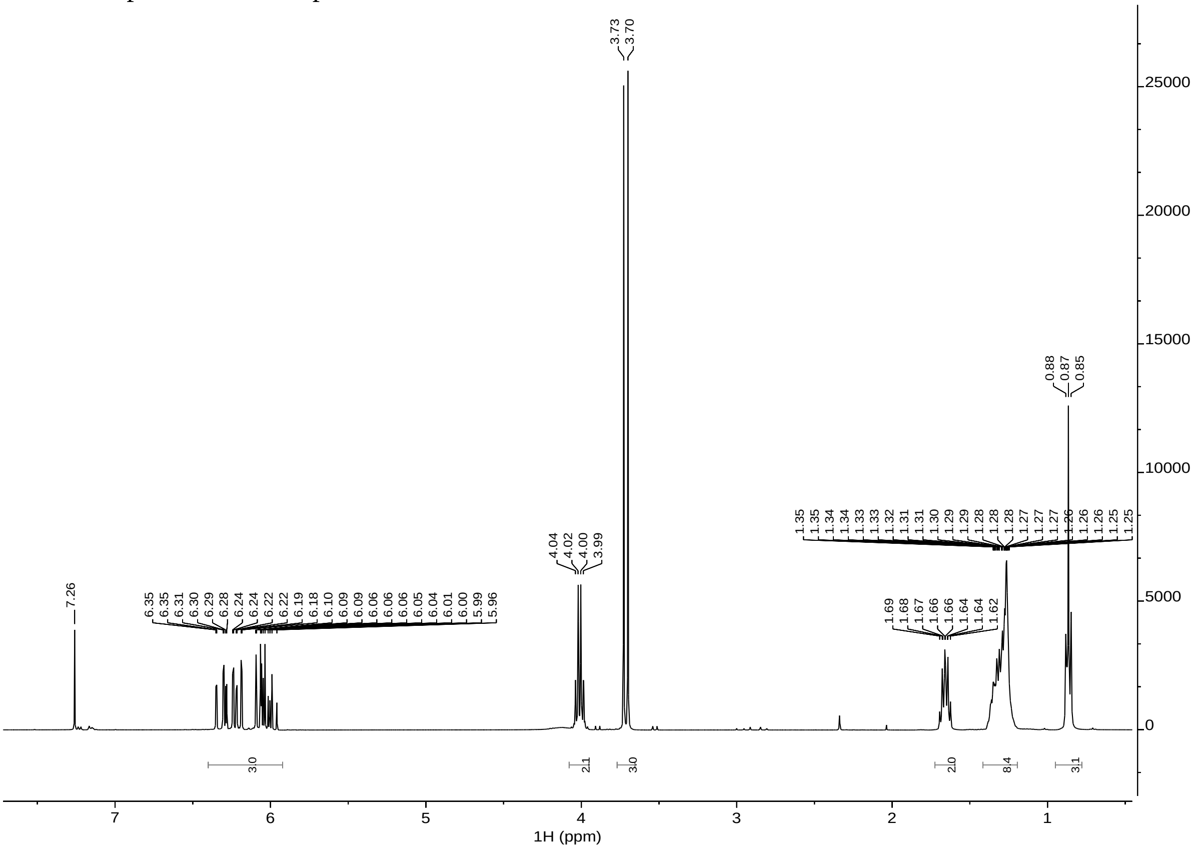

^{13}C NMR spectrum of compound **S8**

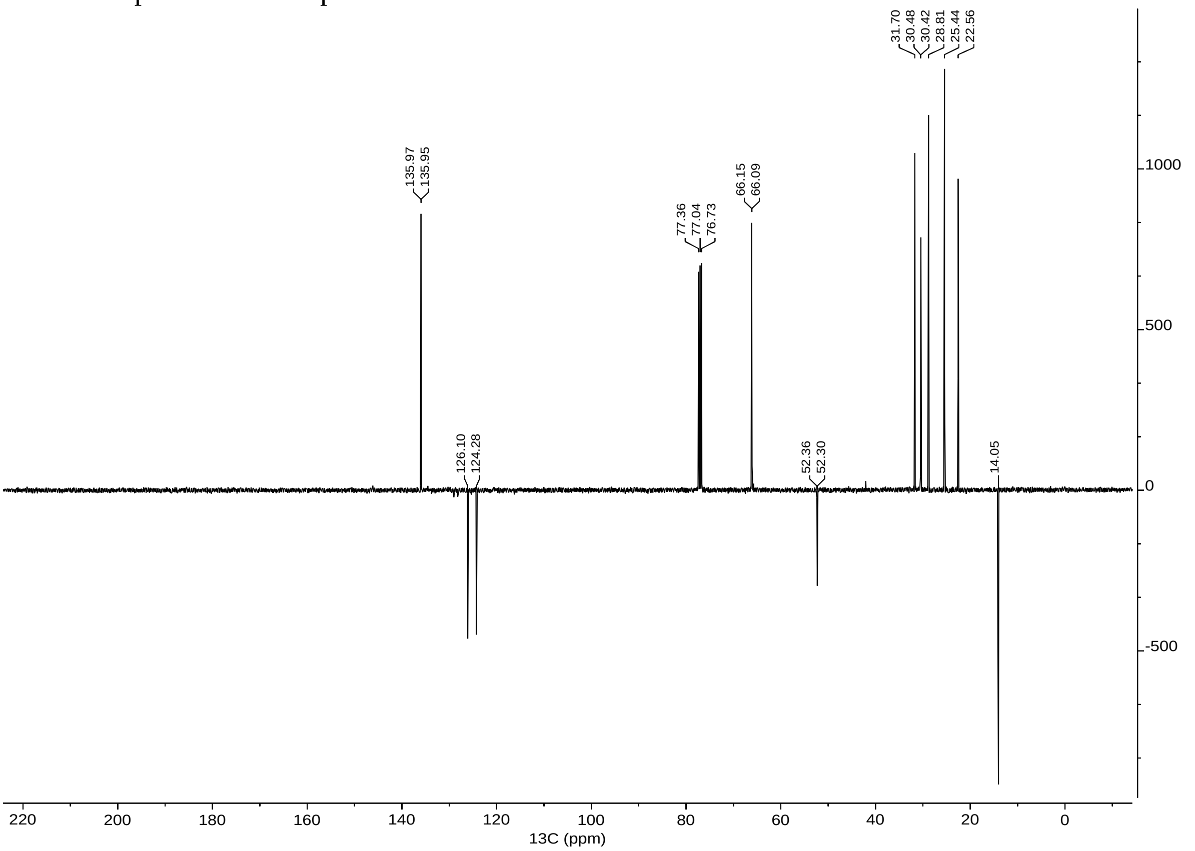

$^{31}P\{^{1}H\}$ NMR spectrum of compound **S8**

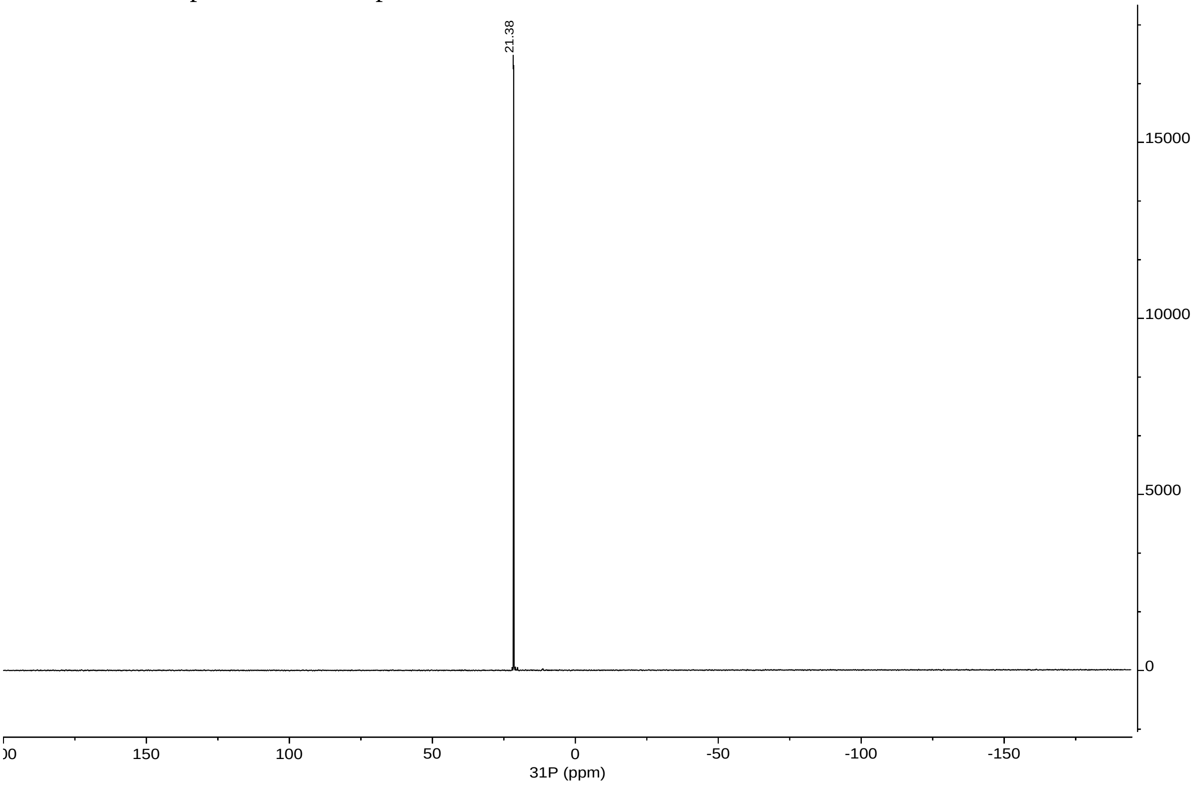

(*Z*)-Hept-4-en-1-yl methyl vinylphosphonate (S9)

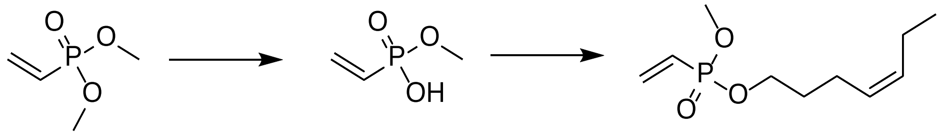

The title compound was prepared according to general methods **A** and **B2** from dimethyl vinylphosphonate (3.93 g, 28.9 mmol) and *cis*-4-hepten-1-ol (4.21 g, 36.87 mmol) in 42% overall yield (2.65 g, 12.14 mmol) as a colourless oil.

^{1}H NMR (401 MHz, $CDCl_3$): 6.36–5.97 (m, 3H, $C\mathit{H}=C\mathit{H}_2$), 5.44–5.37 (m, 1H, $CH_3CH_2C\mathit{H}CHCH_2CH_2CH_2O$), 5.25–5.32–5.25 (m, 1H, $CH_3CH_2CHC\mathit{H}CH_2CH_2CH_2O$), 4.06–3.99 (m, 2H, $CH_3CH_2CHCHCH_2CH_2C\mathit{H}_2O$), 3.73 (d, 3H, *J* = 12.0 Hz, $OC\mathit{H}_3$), 2.17–2.09 (m, 2H, $CH_3CH_2CHCHC\mathit{H}_2CH_2CH_2O$), 2.07–1.99 (m, 2H, $CH_3C\mathit{H}_2CHCHCH_2CH_2CH_2O$), 1.76–1.69 (m, 2H, $CH_3CH_2CHCHCH_2C\mathit{H}_2CH_2O$), 0.95 (t, 3H, *J* = 8.2 Hz, $C\mathit{H}_3CH_2CHCHCH_2CH_2CH_2O$).

^{13}C NMR (101 MHz, $CDCl_3$): 137.0 (d, *J* = 2.1 Hz, $CH=\mathit{C}H_2$), 133.04 ($CH_3CH_2\mathit{C}HCHCH_2CH_2CH_2O$), 127.46 ($CH_3CH_2CH\mathit{C}HCH_2CH_2CH_2O$), 125.32 (d, *J* = 182.9 Hz, $\mathit{C}H=CH_2$), 65.58 (d, *J* = 5.7 Hz, $CH_3CH_2CHCHCH_2CH_2\mathit{C}H_2O$), 52.46 (d, *J* = 5.6 Hz, $O\mathit{C}H_3$), 30.62 (d, *J* = 6.2 Hz, $CH_3CH_2CHCHCH_2\mathit{C}H_2CH_2O$), 23.19 ($CH_3CH_2CHCH\mathit{C}H_2CH_2CH_2O$), 20.63 ($CH_3\mathit{C}H_2CHCHCH_2CH_2CH_2O$), 14.41 ($\mathit{C}H_3CH_2CHCHCH_2CH_2CH_2O$).

^{31}P{^{1}H} NMR (162 MHz, $CDCl_3$): 21.40.

IR ν_{max} (KBr) 3091 (w), 3011 (w), 2965 (s), 2935 (s), 2875 (m), 2852 (m), 1653 (w), 1614 (m), 1463 (m), 1457 (m), 1405 (sh), 1376 (w), 1280 (m), 1242 (vs), 1185 (m), 1080-1000 (vs), 815 (s).

HR-MS(ESI$^+$): For $C_{10}H_{19}O_3NaP$ $(M+Na)^+$ *m*/*z* calculated 241.09640, found 243.09646.

^{1}H NMR spectrum of compound **S9**

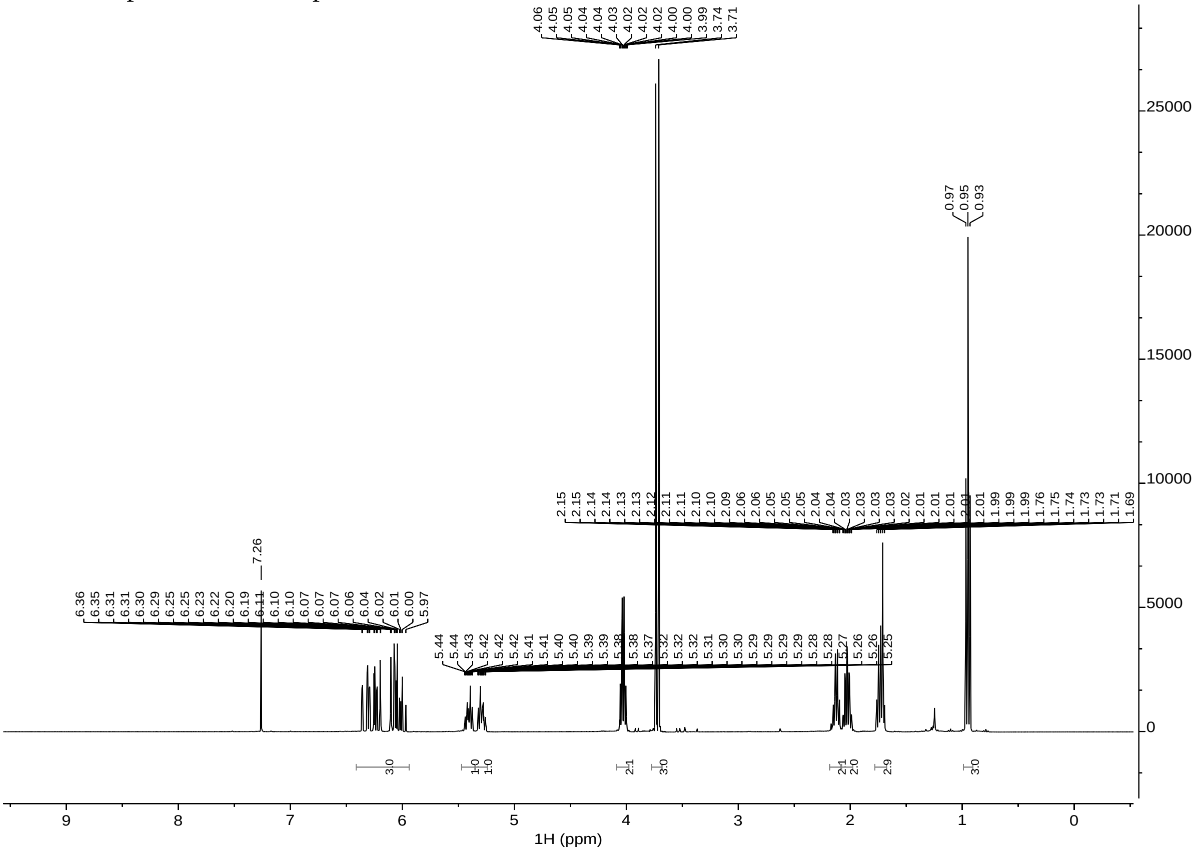

^{13}C NMR spectrum of compound **S9**

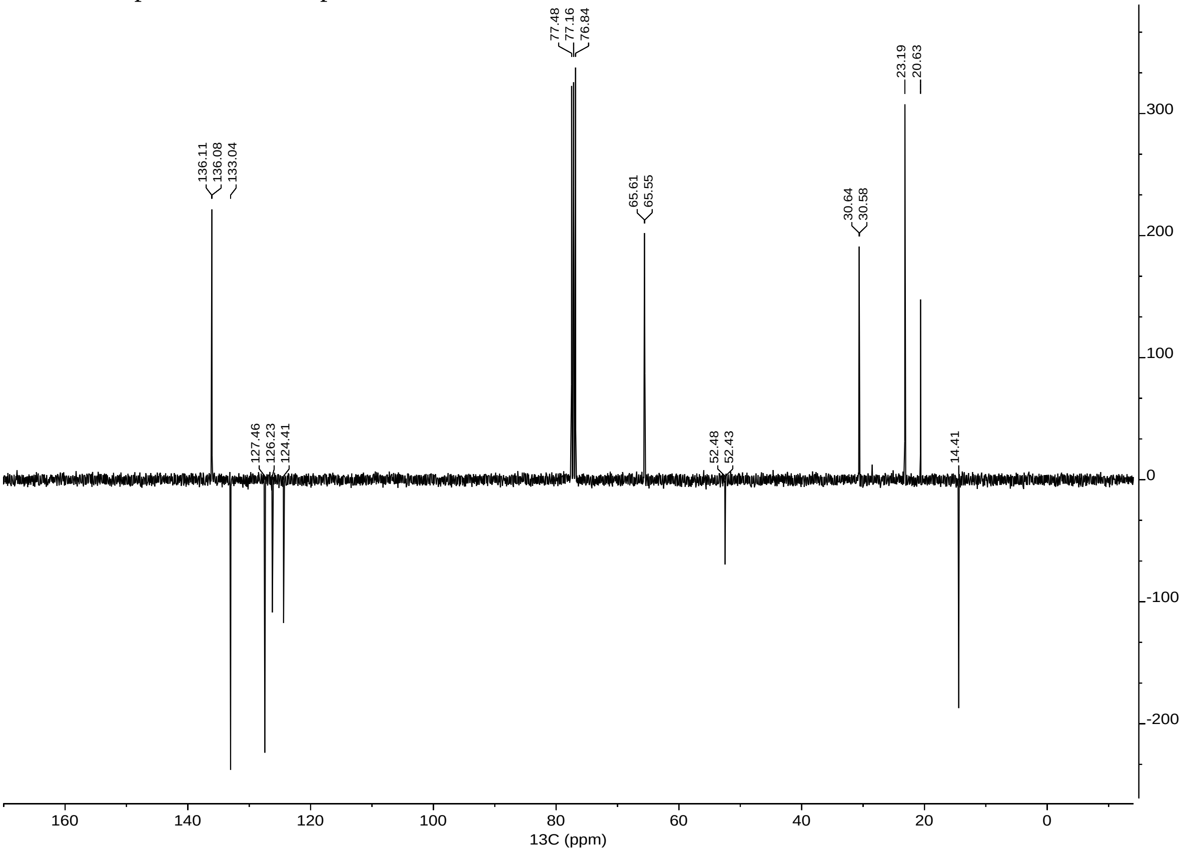

^{31}P{^{1}H} NMR spectrum of compound **S9**

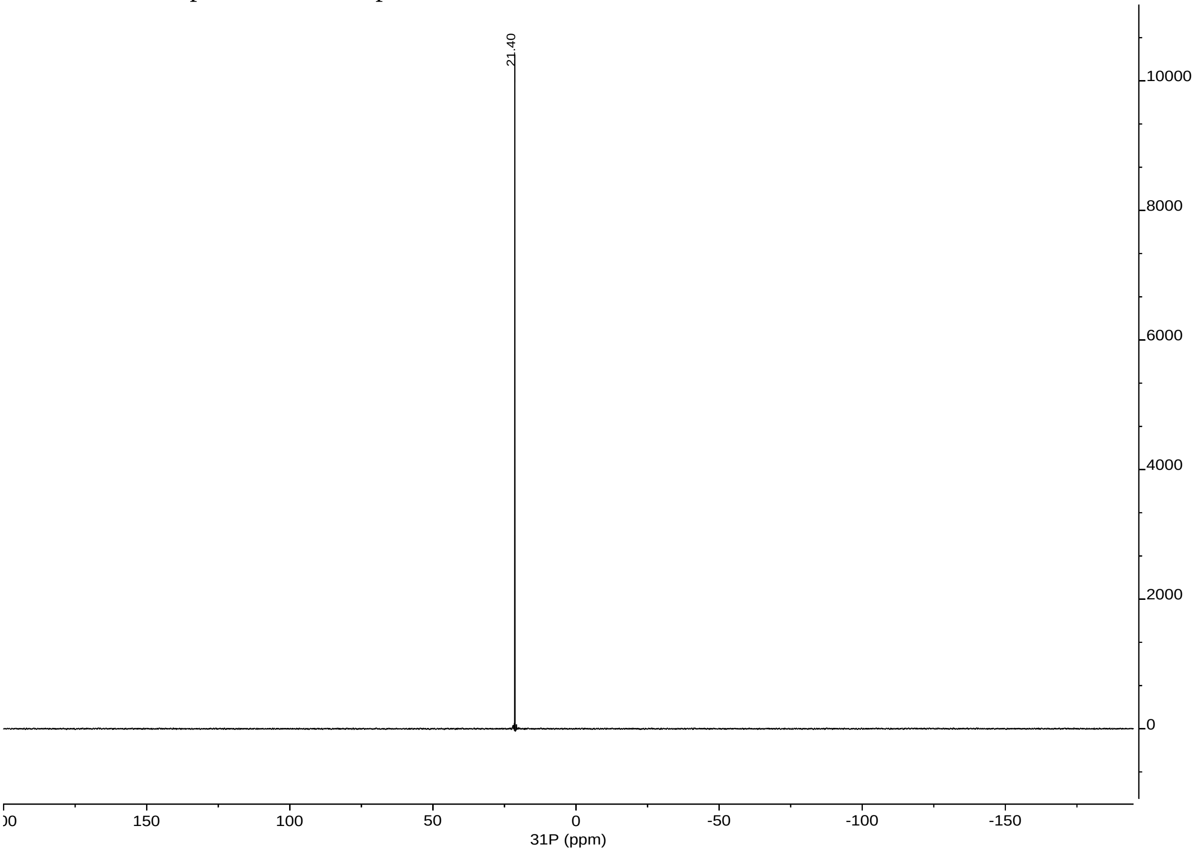

Methyl octyl vinylphosphonate (S10)

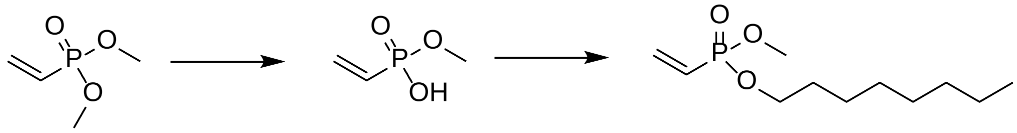

The title compound was prepared according to general methods **A** and **B2** from dimethyl vinylphosphonate (34.49 g, 253.4 mmol) in 46% overall yield (7.36 g, 27.1 mmol) as a colourless oil.

^{1}H NMR (400 MHz, $CDCl_3$): 6.36–5.97 (m, 3H, C*H*=C*H*$_2$), 4.05–3.98 (m, 2H, $CH_3(CH_2)_3CH_2C\mathit{H}_2O$), 3.72 (d, 3H, *J* = 8.2 Hz, OC*H*$_3$), 1.69–1.63 (m, 2H, $CH_3(CH_2)_5C\mathit{H}_2CH_2O$), 1.37–1.25 (m, 10H, $CH_3(C\mathit{H}_2)_5CH_2CH_2O$), 0.89–0.86 (m, 3H, $C\mathit{H}_3(CH_2)_6CH_2O$).

^{13}C NMR (101 MHz, $CDCl_3$): 136.0 (d, *J* = 2.0 Hz, CH=*C*H$_2$), 125.41 (d, *J* = 184.6 Hz, *C*H=CH$_2$), 66.23 (d, *J* = 6.3 Hz, $CH_3(CH_2)_5CH_2\mathit{C}H_2O$), 52.44 (d, *J* = 5.9 Hz, OC*H*$_3$), 30.61 (d, *J* = 6.5 Hz, $CH_3(CH_2)_5\mathit{C}H_2CH_2O$), 31.90, 29.24, 28.30, 25.63, 22.76 ($CH_3(\mathit{C}H_2)_5CH_2CH_2O$), 14.21 ($C\mathit{H}_3(CH_2)_6CH_2O$).

^{31}P{^{1}H} NMR (162 MHz, $CDCl_3$): 21.11.

IR ν_{max} (KBr) 3094 (w), 2956 (s), 2930 (s), 2872 (m), 2857 (m), 1614 (w), 1467 (m), 1400 (m), 1379 (w), 1280 (m), 1242 (s), 1053 (s), 1021 (s), 856 (m, sh), 602 (w).

HR-MS(ESI$^+$): For $C_{11}H_{24}O_3P$ $(M+H)^+$ *m/z* calculated 235.14576, found 235.14539.

^{1}H NMR spectrum of compound **S10**

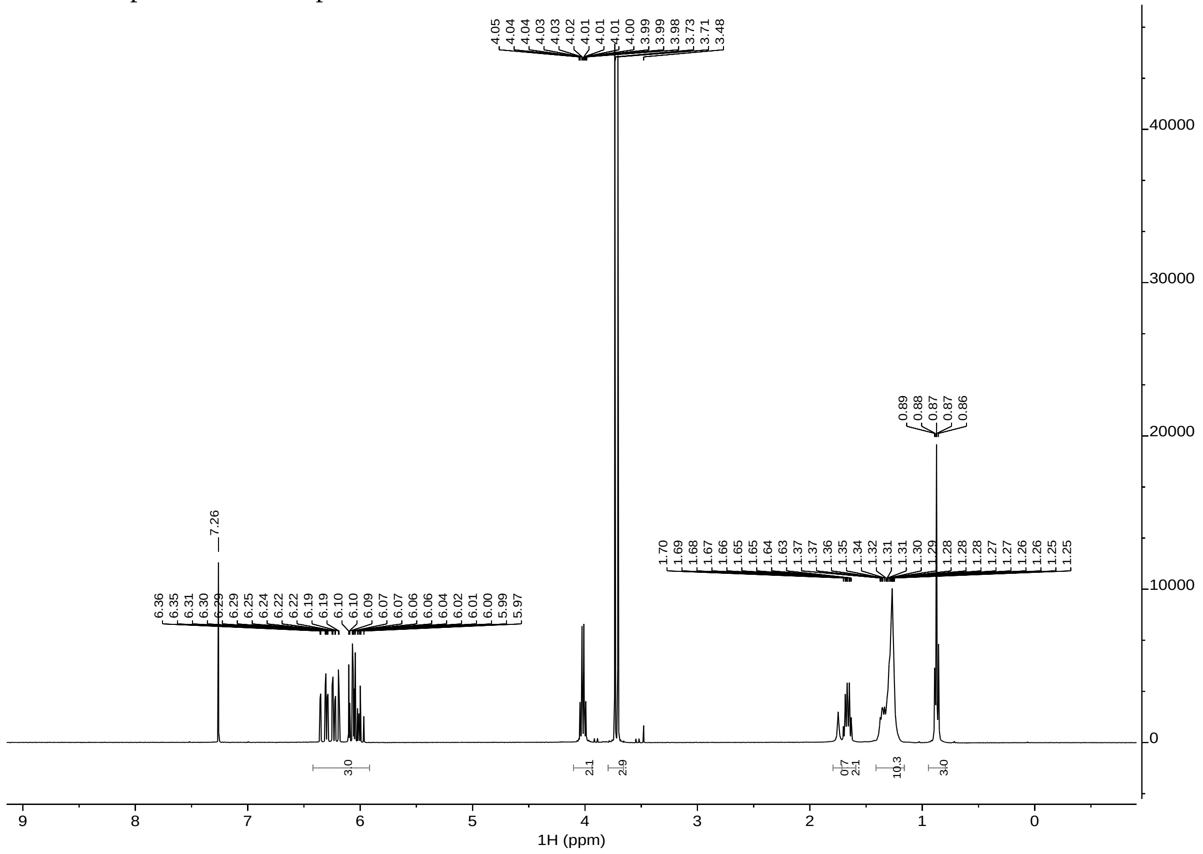

^{13}C NMR spectrum of compound **S10**

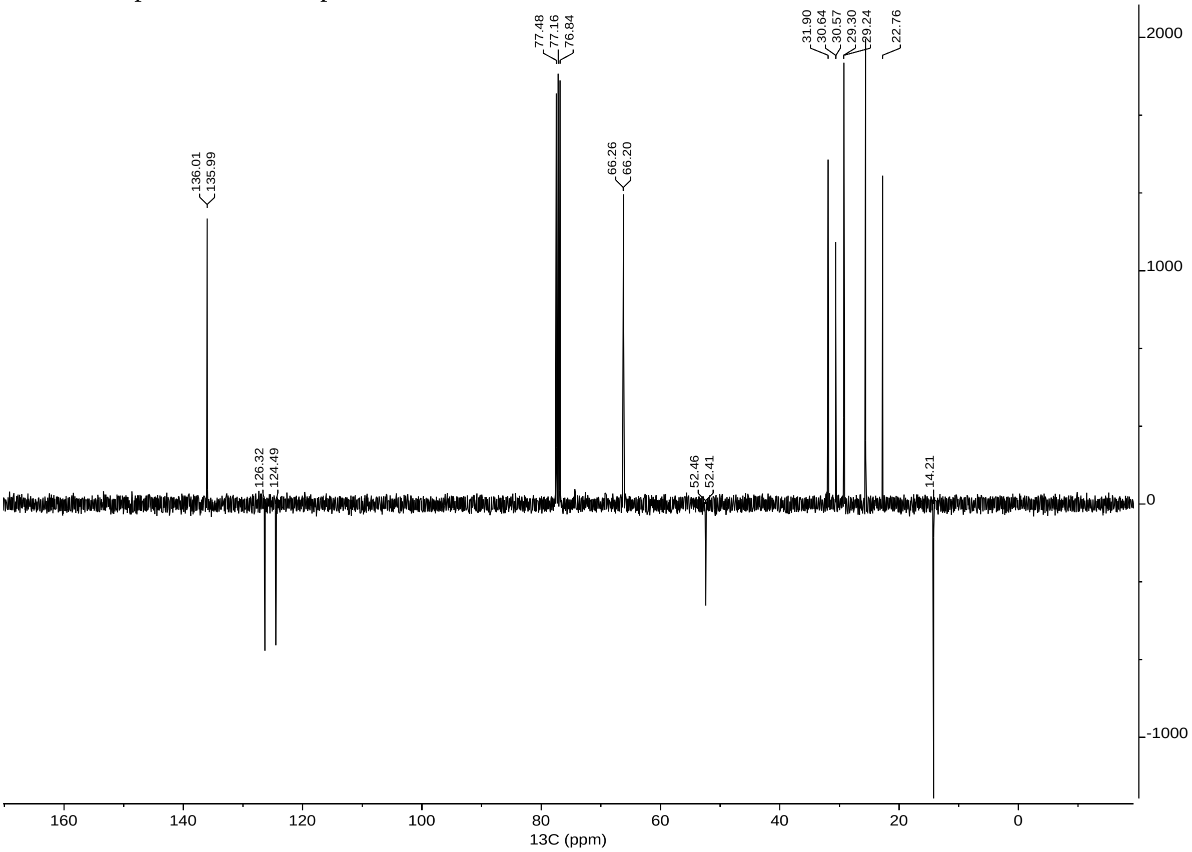

$^{31}P\{^{1}H\}$ NMR spectrum of compound **S10**

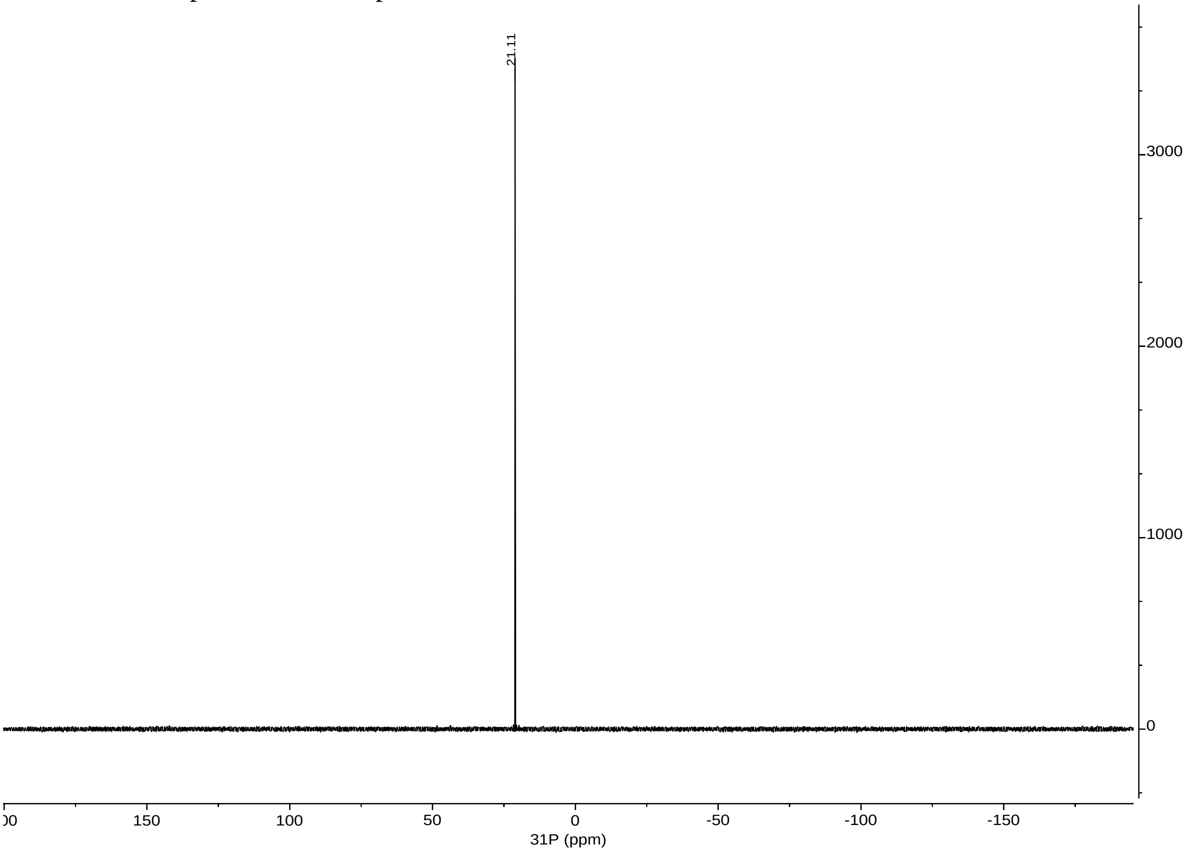

3-Cyclohexylpropyl methyl vinylphosphonate (S11)

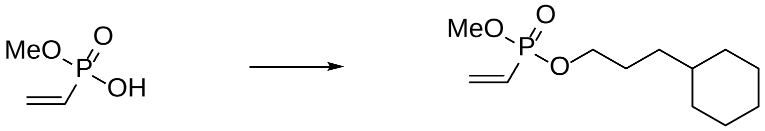

The title compound was prepared according to general method **B2** from mono methyl vinylphosphonate (5.00 g, 41.0 mmol) and 3-cyclohexylpropan-1-ol (9.1 mL, 58.5 mmol) in 34% yield (3.46 g, 14.0 mmol) as a clear oil.

^{1}H NMR (401 MHz, Chloroform-*d*) δ 6.34–5.93 (m, 3H, C*H*=C*H*$_2$), 4.02–3.93 (m, 2H, OC*H*$_2$), 3.69 (d, *J* = 11.1 Hz, 3H, OC*H*$_3$), 1.72–1.56 (m, 7H, 2a,3a,4a,5a,6a-Cy*H*, OCH$_2$C*H*$_2$), 1.26–1.03 (m, 6H, 1,3b,4b,5b-Cy*H*, O(CH$_2$)C*H*$_2$Cy), 0.92–0.77 (m, 2H, 2b,6b-Cy*H*).

^{13}C NMR (101 MHz, Chloroform-*d*) δ 135.98 (d, *J* = 2.1 Hz, =*C*H$_2$), 125.32 (d, *J* = 184.2 Hz, P*C*H), 66.52 (d, *J* = 5.9 Hz, O*C*H$_2$), 52.38 (d, *J* = 5.7 Hz, O*C*H$_3$), 37.34 (1-*C*H$_{Cy}$), 33.34 (2,6-(*C*H$_2$)$_{Cy}$), 33.17 (*C*H$_2$Cy), 27.96 (d, *J* = 6.2 Hz, *C*H$_2$CH$_2$Cy), 26.68 (4-(*C*H$_2$)$_{Cy}$), 26.39 (3,5-(*C*H$_2$)$_{Cy}$).

^{31}P{^{1}H} NMR (162 MHz, Chloroform-*d*) δ 21.30.

IR ν_{max} (CHCl$_3$) 3090 (vw), 2926 (vs), 2853 (s), 1614 (w), ~1474 (w, sh), 1462 (w), 1449 (m), 1400 (w), 1279 (w), 1242 (s), 1068 (s), 1057 (s), 1046 (s), 1010 (vs), 988 (m), 817 (m).

HR-MS(ESI^{+}): For $C_{12}H_{24}O_3P$ (M+H)$^{+}$ *m*/*z* calculated 247.14576, found 247.14564. For $C_{12}H_{23}O_3NaP$ (M+Na)$^{+}$ *m*/*z* calculated 269.12770, found 269.12759.

^{1}H NMR spectrum of compound **S11**

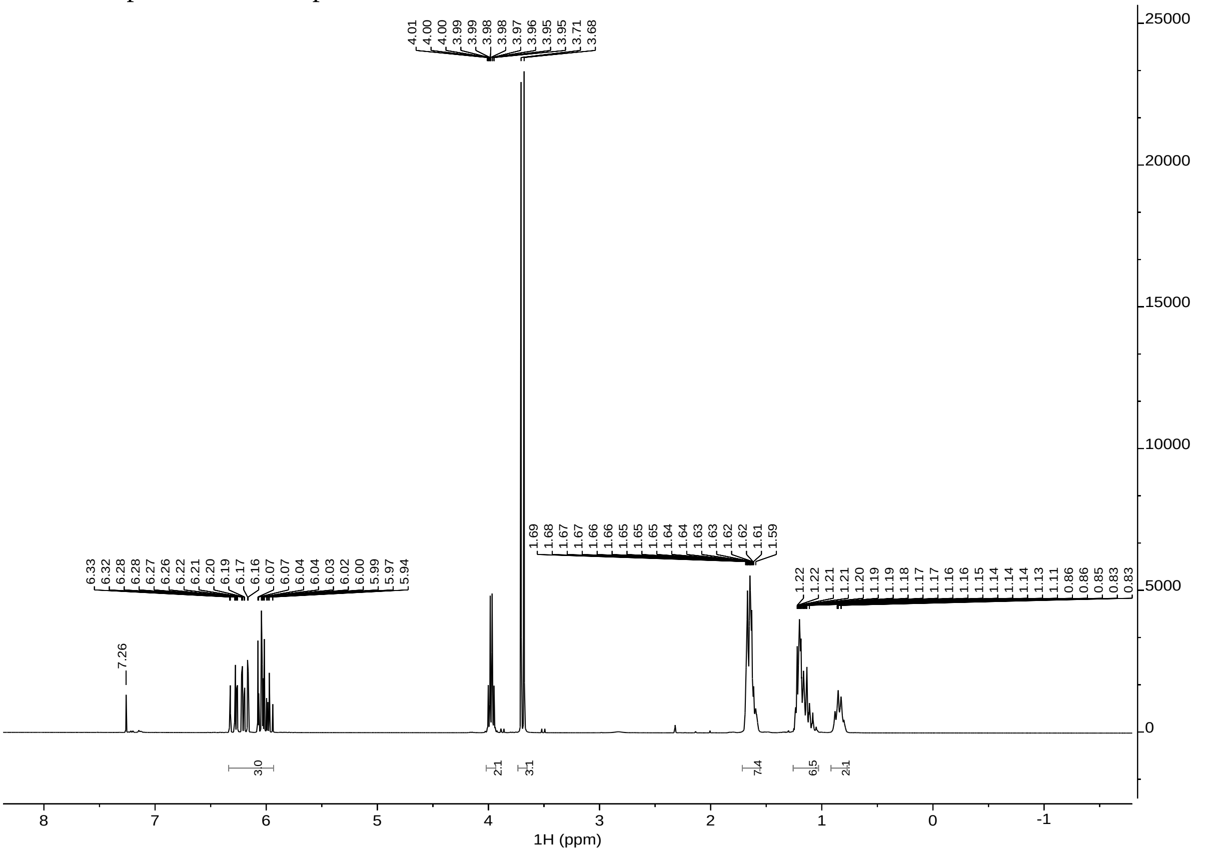

^{13}C NMR spectrum of compound **S11**

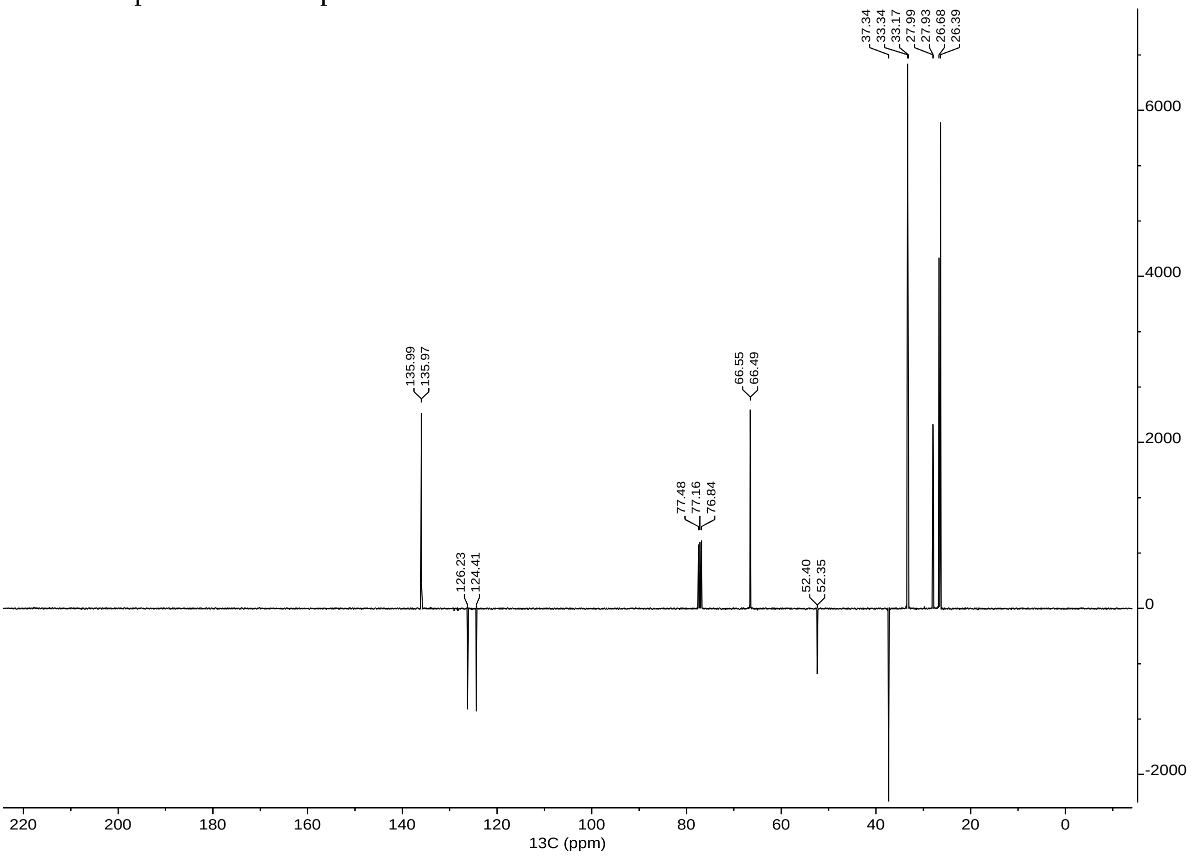

$^{31}P\{^{1}H\}$ NMR spectrum of compound **S11**

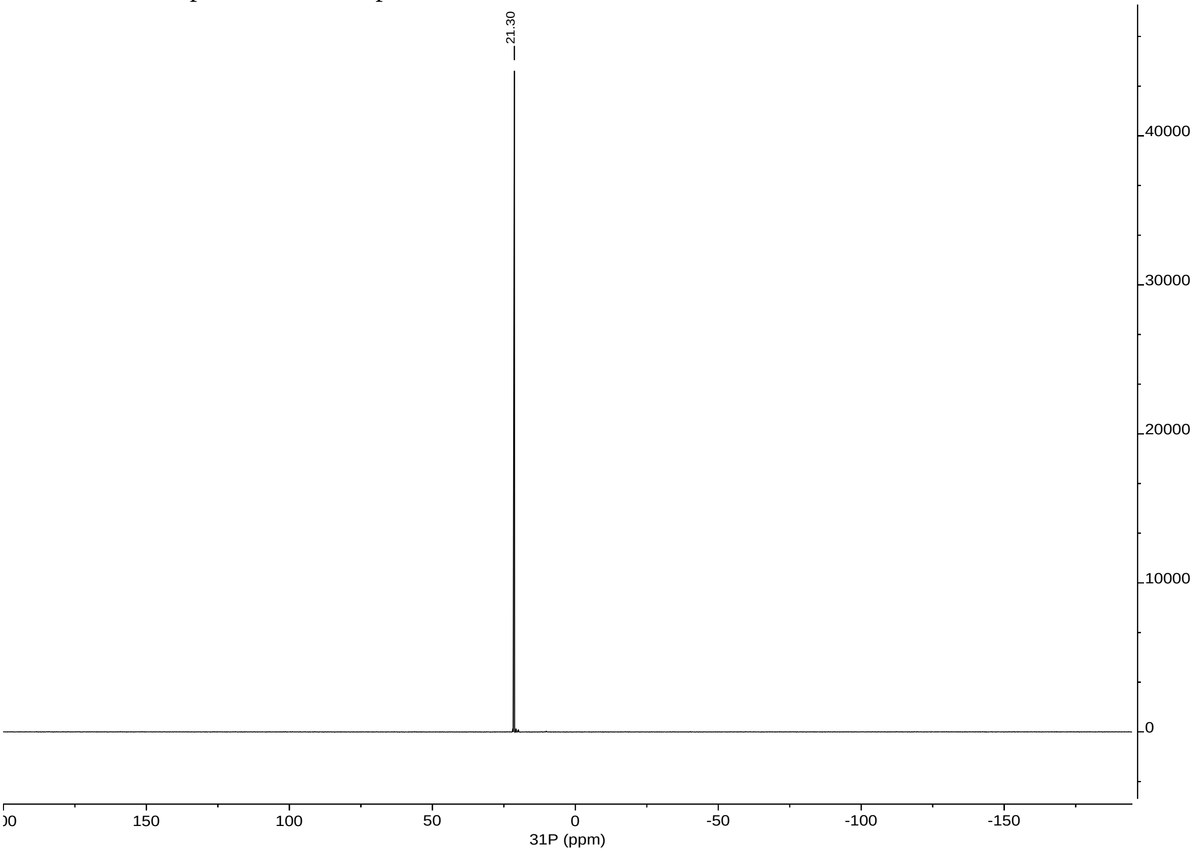

Methyl (*Z*)-oct-3-en-1-yl vinylphosphonate (S12)

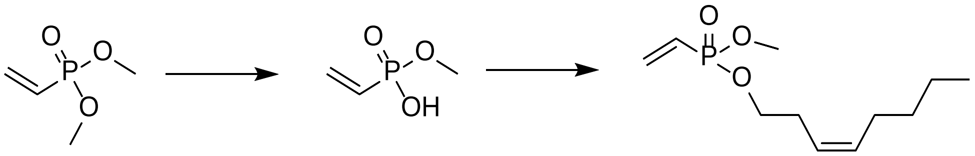

The title compound was prepared according to general methods **A** and **B2** from dimethyl vinylphosphonate (5.24 g, 38.5 mmol) in 53% overall yield (4.72 g, 20.3 mmol) as a colourless oil.

^{1}H NMR (400 MHz, $CDCl_3$): 6.36–5.96 (m, 3H, C*H*=C*H*$_2$), 5.54–5.47 (m, 1H, $CH_3(CH_2)_3C$*H*$CHCH_2CH_2O$), 5.37–5.30 (m, 1H, $CH_3(CH_2)_3CHC$*H*CH_2CH_2O), 4.04–3.97 (m, 2H, $CH_3(CH_2)_2CH_2CHCHCH_2C$*H*$_2$O), 3.72 (d, 3H, *J* =11.0 Hz, OC*H*$_3$), 2.46–2.39 (m, 2H, $CH_3(CH_2)_2CH_2CHCHC$*H*$_2CH_2O$), 2.06–2.00 (m, 2H, $CH_3(CH_2)_2C$*H*$_2CHCHCH_2CH_2O$), 1.36–1.27 (m, 4H, $CH_3(C$*H*$_2)_2CH_2CHCHCH_2CH_2O$), 0.93–0.85 (m, 3H, C*H*$_3(CH_2)_3CHCHCH_2CH_2O$).

^{13}C NMR (101 MHz, $CDCl_3$): 136.09 (d, *J* = 2.1 Hz, CH=*C*H$_2$), 133.45 ($CH_3(CH_2)_3$*C*$HCHCH_2CH_2O$), 125.32 (d, *J* = 184.3 Hz, *C*H=CH$_2$), 123.78 ($CH_3(CH_2)_3CH$*C*HCH_2CH_2O), 65.52 (d, *J* = 6.0 Hz, $CH_3(CH_2)_3CHCHCH_2$*C*H_2O), 52.46 (d, *J* = 6.2 Hz, O*C*H$_3$), 31.84 (CH_3CH_2*C*$H_2CH_2CHCHCH_2CH_2O$), 28.80 (d, *J* = 6.7 Hz, $CH_3(CH_2)_3CHCH$*C*H_2CH_2O), 27.20 14.08 (*C*$H_3(CH_2)_3CHCHCH_2CH_2O$), ($CH_3CH_2CH_2$*C*$H_2CHCHCH_2CH_2O$), 22.44 (CH_3*C*$H_2CH_2CH_2CHCHCH_2CH_2O$).

^{31}P{^{1}H} NMR (162 MHz, $CDCl_3$): 21.12.

IR ν_{max} (KBr) 3086 (w), 3011 (m), 2957 (s), 2929 (m), 2873 (m), 2859 (m), 1467 (m), 1398 (m), 1380 (w), 1254 (vs), 1184 (m), 1050 (s), 1017 (vs), 987 (m, sh), 815 (s), 723 (m).

HR-MS(ESI$^+$): For $C_{11}H_{21}O_3NaP$ $(M+Na)^+$ *m/z* calculated 255.11205, found 255.11199.

^{1}H NMR spectrum of compound **S12**

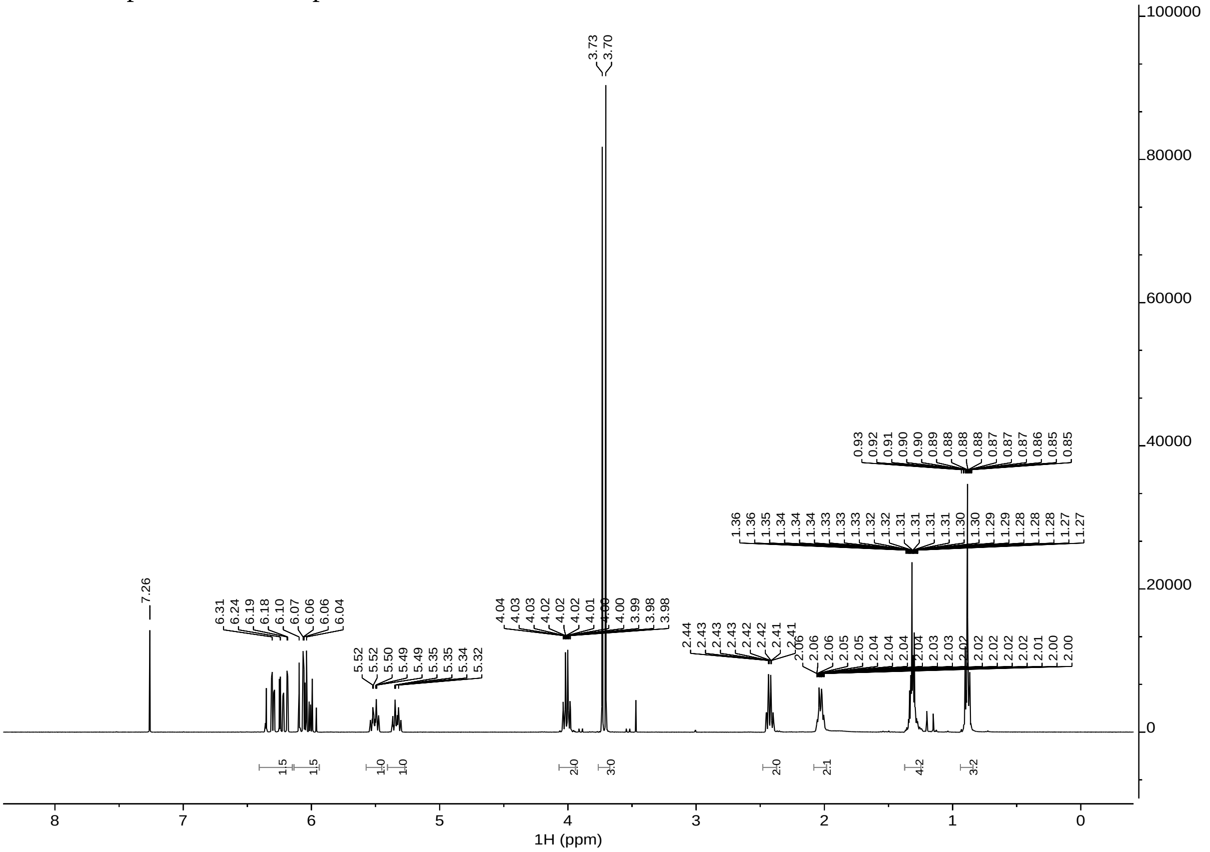

^{13}C NMR spectrum of compound **S12**

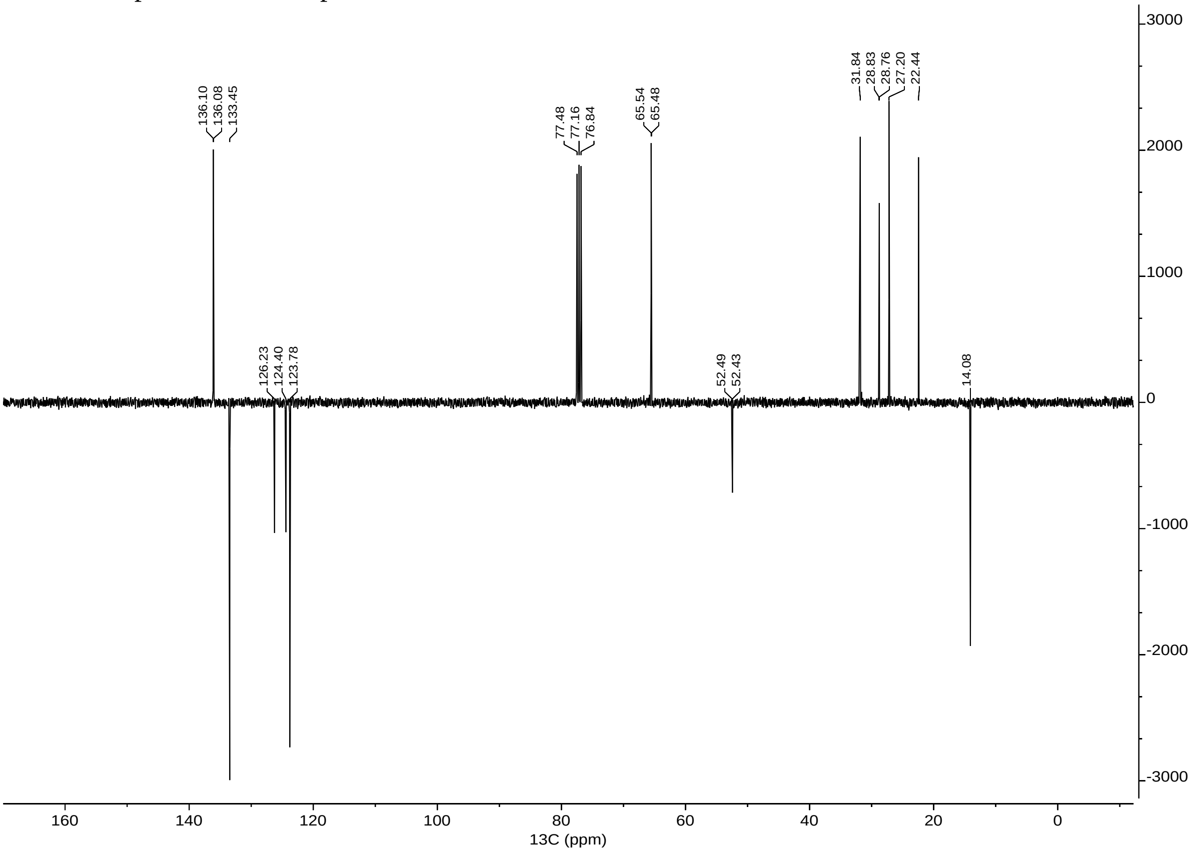

$^{31}P\{^1H\}$ NMR spectrum of compound **S12**

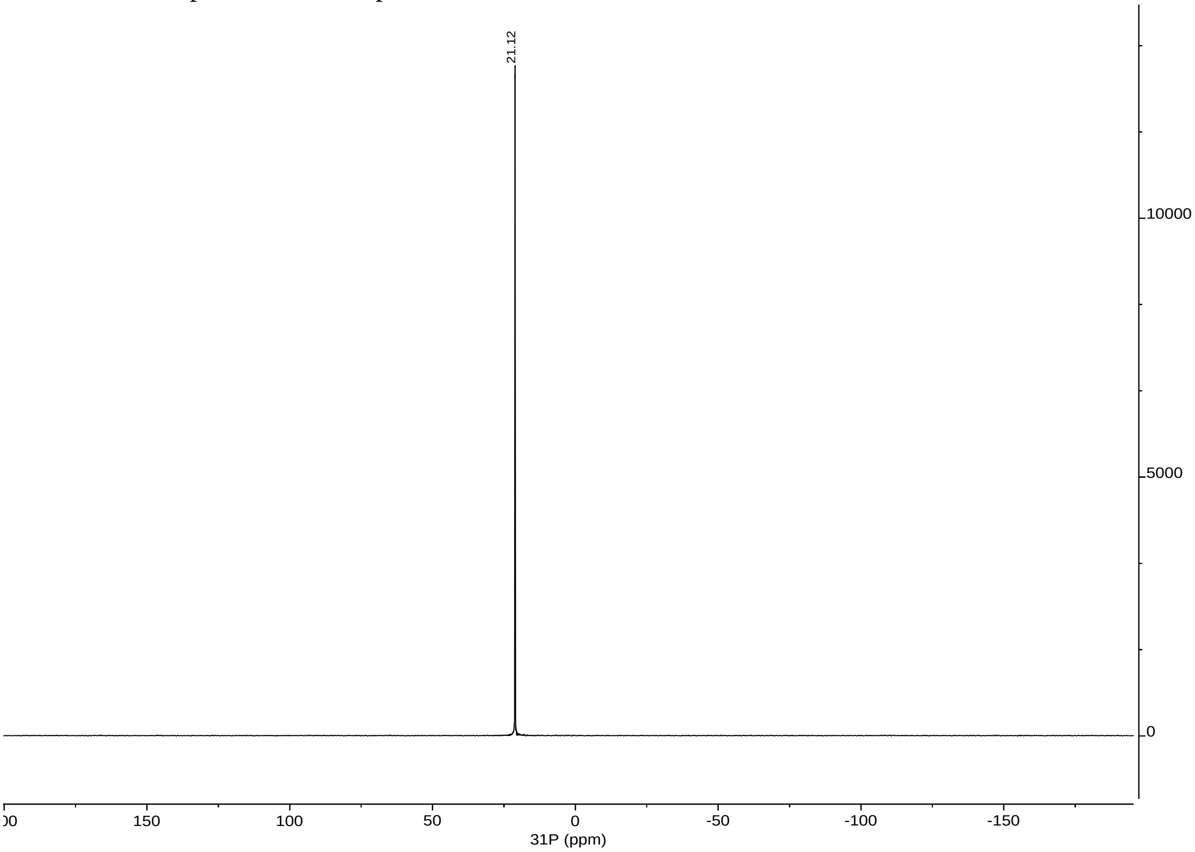

Methyl phenethyl vinylphosphonate (S13)

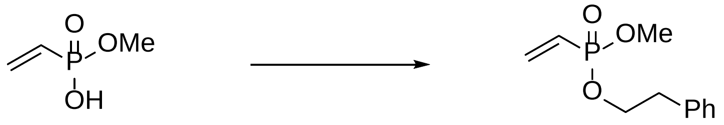

The title compound was prepared according to general method **B2** from mono methyl vinylphosphonate (2.74 g, 22.4 mmol) and 2-phenylethanol (5.4 mL, 44.9 mmol) in 28% yield (1.4 g, 6.19 mmol) as a clear oil.

^{1}H NMR (401 MHz, $CDCl_3$) δ 7.37–7.12 (m, 5H, Ph*H*), 6.37–5.84 (m, 3H, C*H*=C*H*$_2$), 4.36–4.12 (m, 2H, OC*H*$_2$), 3.75–3.57 (m, 3H, C*H*$_3$), 2.99 (t, *J* = 7.0 Hz, 2H, C*H*$_2$Ph).

^{13}C NMR (101 MHz, $CDCl_3$) δ 137.35 (CH_2C_{quat}), 136.12 (d, *J* = 2.1 Hz, =*C*H$_2$), 129.15, 128.62, 126.81 (C_{Ph}), 125.10 (d, *J* = 184.3 Hz, P*C*H), 66.41 (d, *J* = 5.8 Hz, O*C*H$_2$), 52.37 (d, *J* = 5.8 Hz, *C*H$_3$), 37.08 (d, *J* = 6.3 Hz, *C*H$_2$Ph).

^{31}P{^{1}H} NMR (162 MHz, $CDCl_3$) δ 21.36.

IR ν_{max}(film) 3087 (w), 3063 (w), 3029 (w), 2953 (m), 2850 (w), 1612 (w), 1605 (w), 1584 (vw), 1498 (m), 1468 (w), 1455 (m), 1398 (m), 1280 (m, sh), 1250 (vs), 1183 (m), 1088 (m), 1060 (vs), 1046 (vs), 1015 (vs), 1002 (vs), 817 (s), 751 (m), 701 (s), 494 (m).

HR-MS(APCI^{+}): For $C_{11}H_{16}O_3P$ $(M+H)^+$ *m/z* calculated 227.08316, found 227.08280.

^{1}H NMR spectrum of compound **S13**

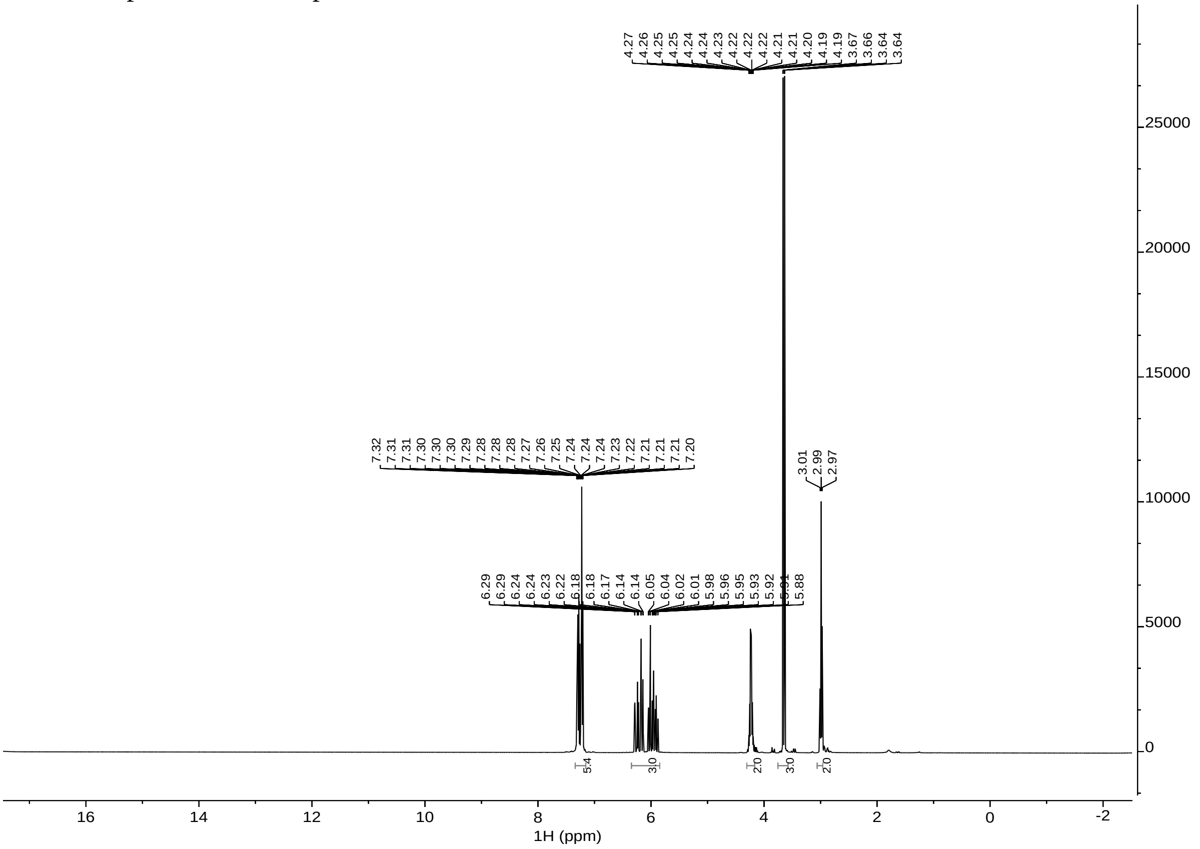

^{13}C NMR spectrum of compound **S13**

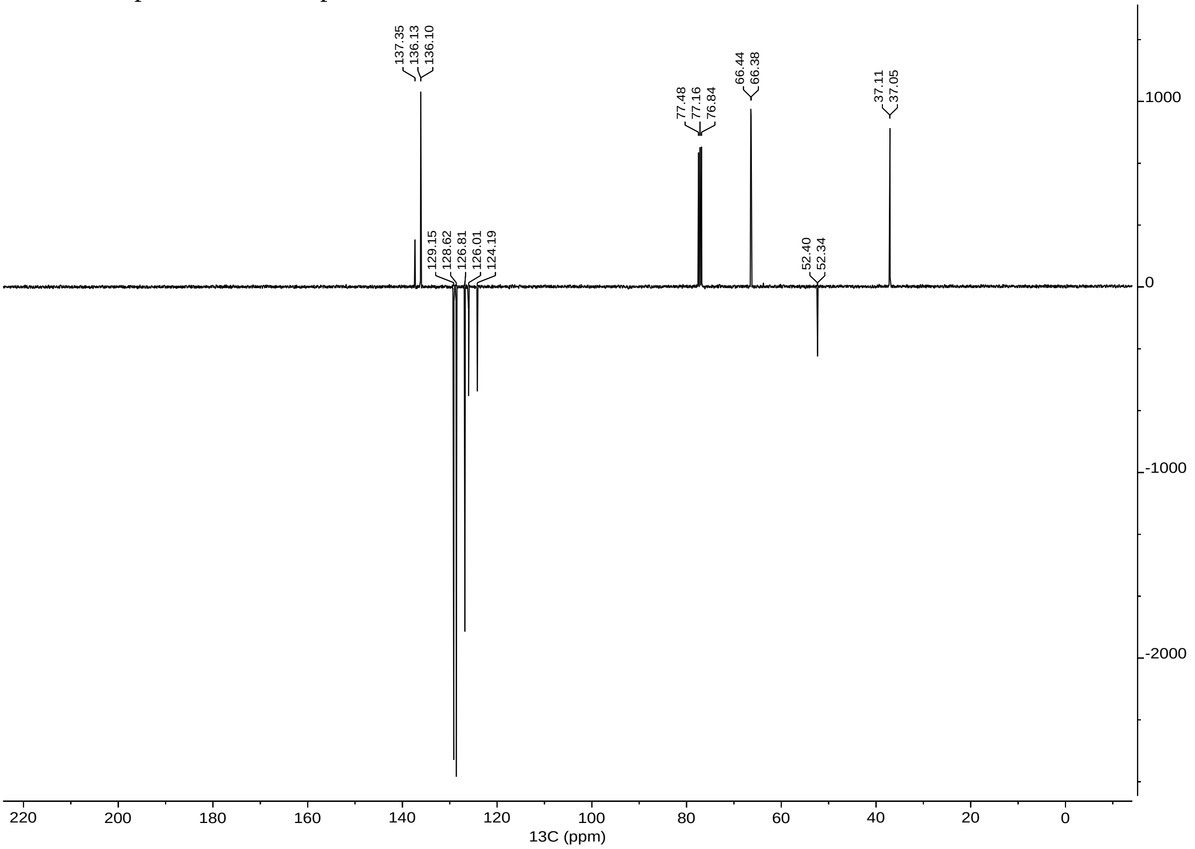

$^{31}P\{^1H\}$ NMR spectrum of compound **S13**

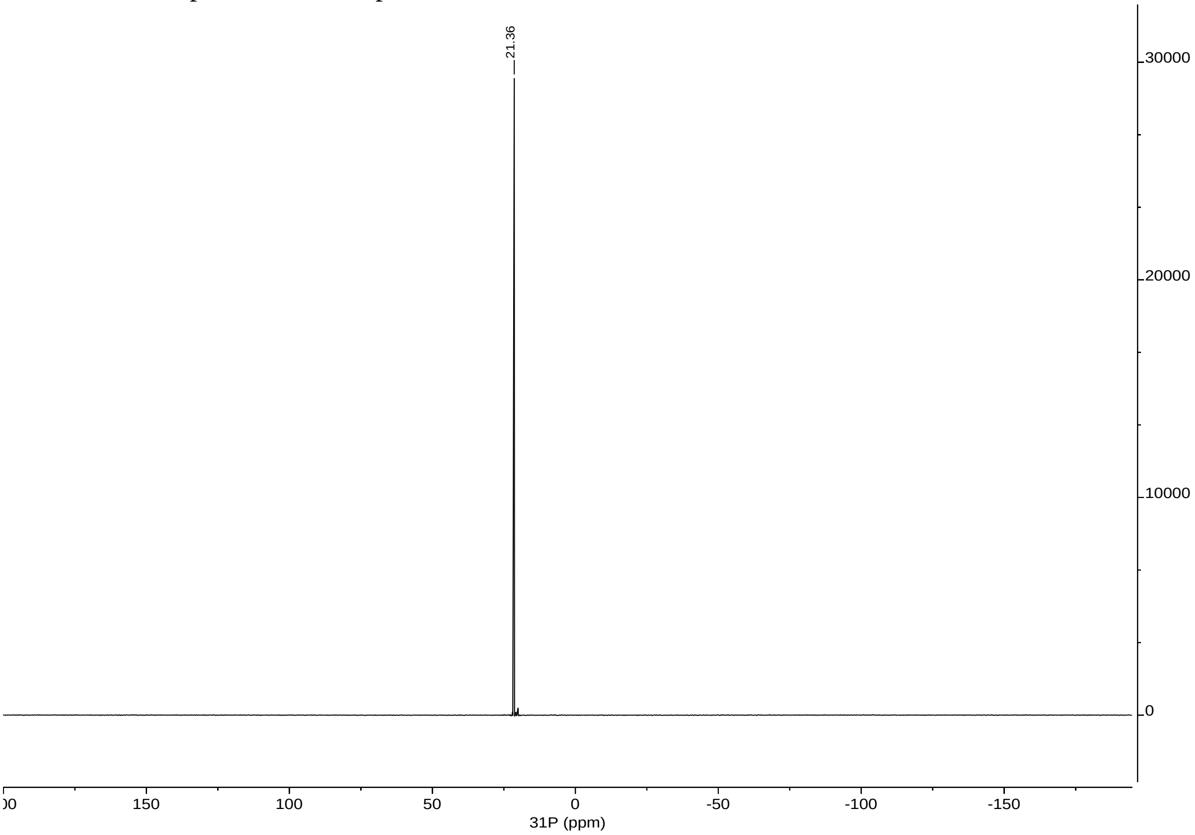

Methyl (*Z*)-non-3-en-1-yl vinylphosphonate (S14)

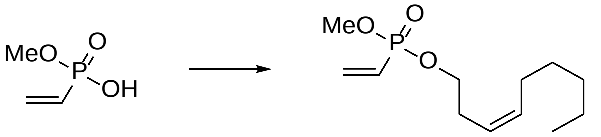

The title compound was prepared according to general method **B2** from mono methyl vinylphosphonate (3.00 g, 24.6 mmol) and (*Z*)-non-3-en-1-ol (8.3 mL, 49.2 mmol) in 70% yield (4.25 g, 17.3 mmol) as a clear oil.

^{1}H NMR (401 MHz, $CDCl_3$) δ 6.34–5.94 (m, 3H, C*H*=C*H*$_2$), 5.53–5.44 (m, 1H, C*H*$(CH_2)_4CH_3$), 5.35–5.27 (m, 1H, $O(CH_2)_2C$*H*), 3.99 (qd, *J* = 7.1, 1.3 Hz, 2H, OC*H*$_2$), 3.69 (d, *J* = 11.1 Hz, 3H, OC*H*$_3$), 2.44–2.36 (m, 2H, OCH_2C*H*$_2$), 2.04–1.96 (m, 2H, C*H*$_2(CH_2)_3CH_3$), 1.36–1.18 (m, 6H, (C*H*$_2$)$_3CH_3$), 0.88–0.81 (m, 3H, CH_2C*H*$_3$).

^{13}C NMR (101 MHz, $CDCl_3$) δ 136.04 (d, *J* = 2.1 Hz, =*C*H$_2$), 133.45 (*C*H$(CH_2)_4CH_3$), 125.28 (d, *J* = 184.4 Hz, P*C*H), 123.72 ($O(CH_2)_2$*C*H), 65.43 (d, *J* = 5.9 Hz, O*C*H$_2$), 52.39 (d, *J* = 5.7 Hz, O*C*H$_3$), 31.54 (*C*H$_2CH_2CH_3$), 29.28 (*C*H$_2(CH_2)_2CH_3$), 28.74 (d, *J* = 6.3 Hz, OCH_2*C*H$_2$), 27.40 (*C*H$_2(CH_2)_3CH_3$), 22.62 (*C*H$_2CH_3$), 14.12 (CH_2*C*H$_3$).

^{31}P{^{1}H} NMR (162 MHz, $CDCl_3$) δ 21.32.

IR ν_{max} ($CHCl_3$) 3090 (vw), 2958 (s), 2930 (s), 2873 (m), 2856 (m), ~1654 (vw), 1614 (w), 1467 (m), 1460 (m), ~1447 (w, sh), 1400 (m), 1380 (w), 1279 (m), 1242 (vs), 1185 (m), 1053 (vs, br), 1018 (vs, br), 988 (s), 820 (s).

HR-MS(APCI$^+$): For $C_{12}H_{24}O_3P$ $(M+H)^+$ *m*/*z* calculated 247.14576, found 247.14566.

^{1}H NMR spectrum of compound **S14**

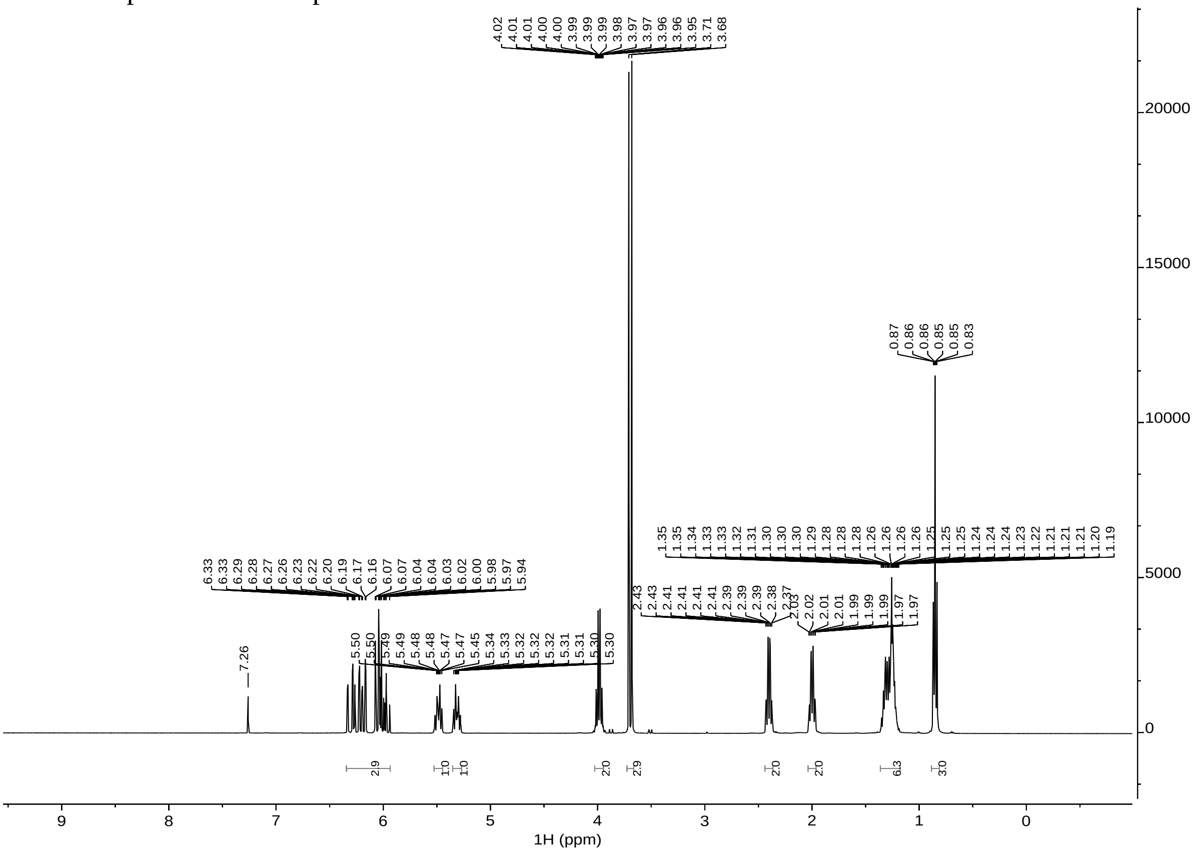

^{13}C NMR spectrum of compound **S14**

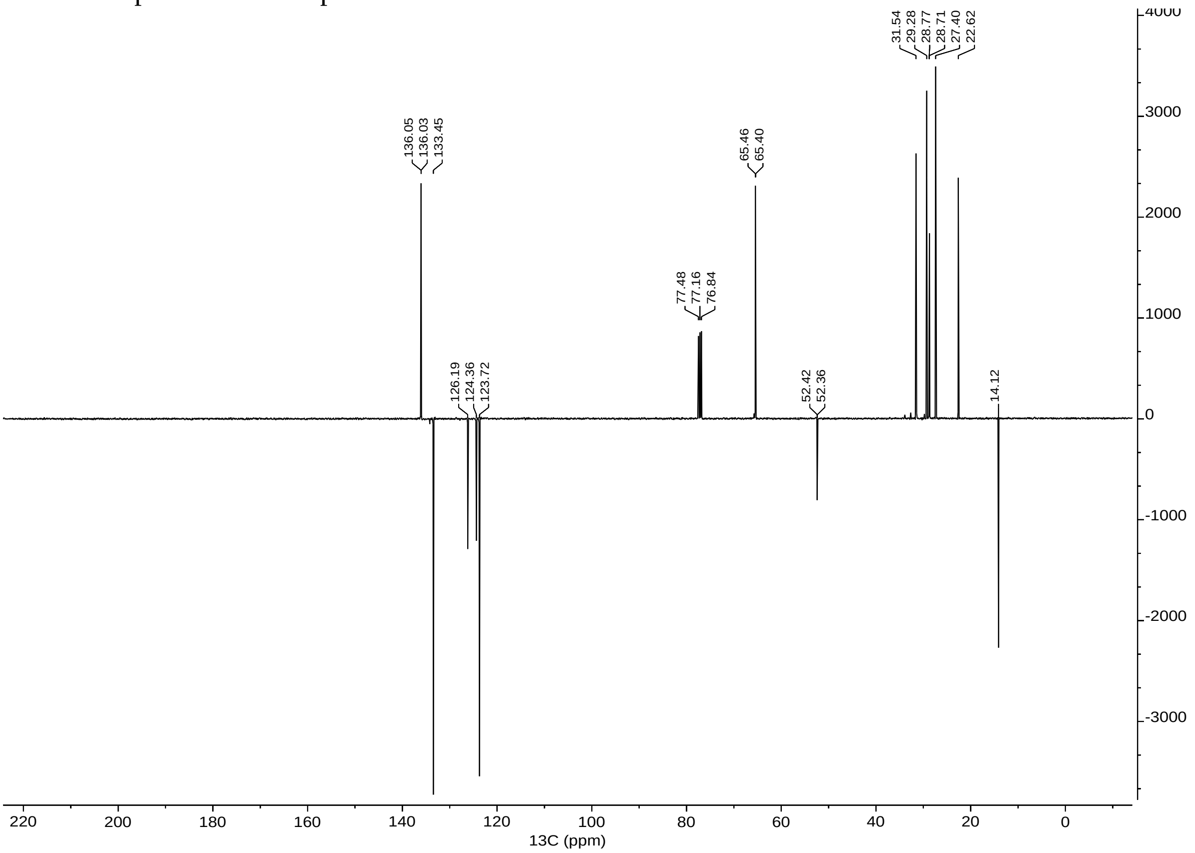

$^{31}P\{^{1}H\}$ NMR spectrum of compound **S14**

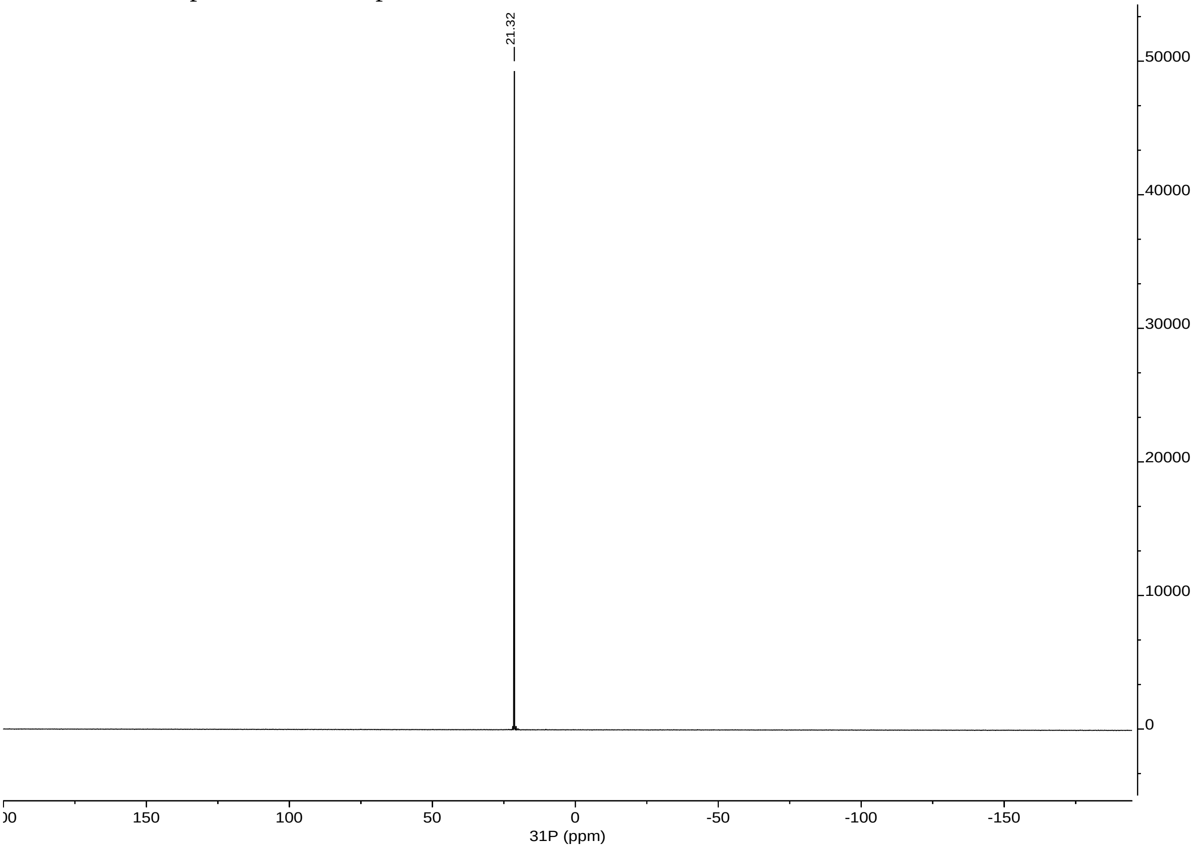

Decyl methyl vinylphosphonate (S15)

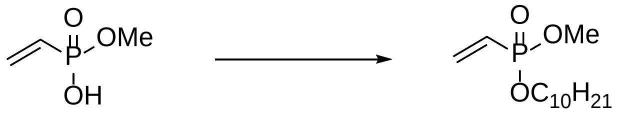

The title compound was prepared according to general method **B1** from mono methyl vinylphosphonate (9.98 g, 81.8 mmol) and *n*-decanol (12.9 mL, 81.8 mmol) in 54% yield (11.7 g, 44.5 mmol) as a clear oil.

^{1}H NMR (401 MHz, $CDCl_3$) δ 6.41–5.90 (m, 3H, C*H*=C*H*$_2$), 4.01 (q, *J* = 6.9 Hz, 2H, OC*H*$_2$), 3.72 (d, *J* = 11.1 Hz, 3H, OC*H*$_3$), 1.66 (p, *J* = 6.7 Hz, 2H, OCH_2C*H*$_2$), 1.48–1.15 (m, 14H, (C*H*$_2$)$_7$$CH_3$), 1.02–0.77 (m, 3H, CH_2C*H*$_3$).

^{13}C NMR (101 MHz, $CDCl_3$) δ 136.06 (d, *J* = 2.1 Hz, =*C*H_2), 125.35 (d, *J* = 184.2 Hz, P*C*H), 66.24 (d, *J* = 5.9 Hz, O*C*H_2), 52.44 (d, *J* = 5.6 Hz, O*C*H_3), 32.01 (*C*$H_2CH_2CH_3$), 30.58 (d, *J* = 6.3 Hz, OCH_2*C*H_2), 29.63, 29.63, 29.41, 29.27, 25.61, 22.80 (*C*H_2CH_3), 14.23 (CH_2*C*H_3).

^{31}P{^{1}H} NMR (162 MHz, $CDCl_3$) δ 21.37.

IR ν_{max}($CHCl_3$) 3090 (vw), 2956 (vs), 2928 (vs), 2871 (s), 2856 (s), 1614 (w), 1467 (m), ~1455 (m, sh), ~1447 (m, sh), 1400 (m), 1379 (w), 1279 (m), 1242 (vs), 1185 (m), 1055 (vs), 1022 (vs), 989 (s), 818 (s).

HR-MS(ESI$^+$): For $C_{13}H_{27}O_3NaP$ $(M+Na)^+$ *m*/*z* calculated 285.15900, found 285.15904.

^{1}H NMR spectrum of compound **S15**

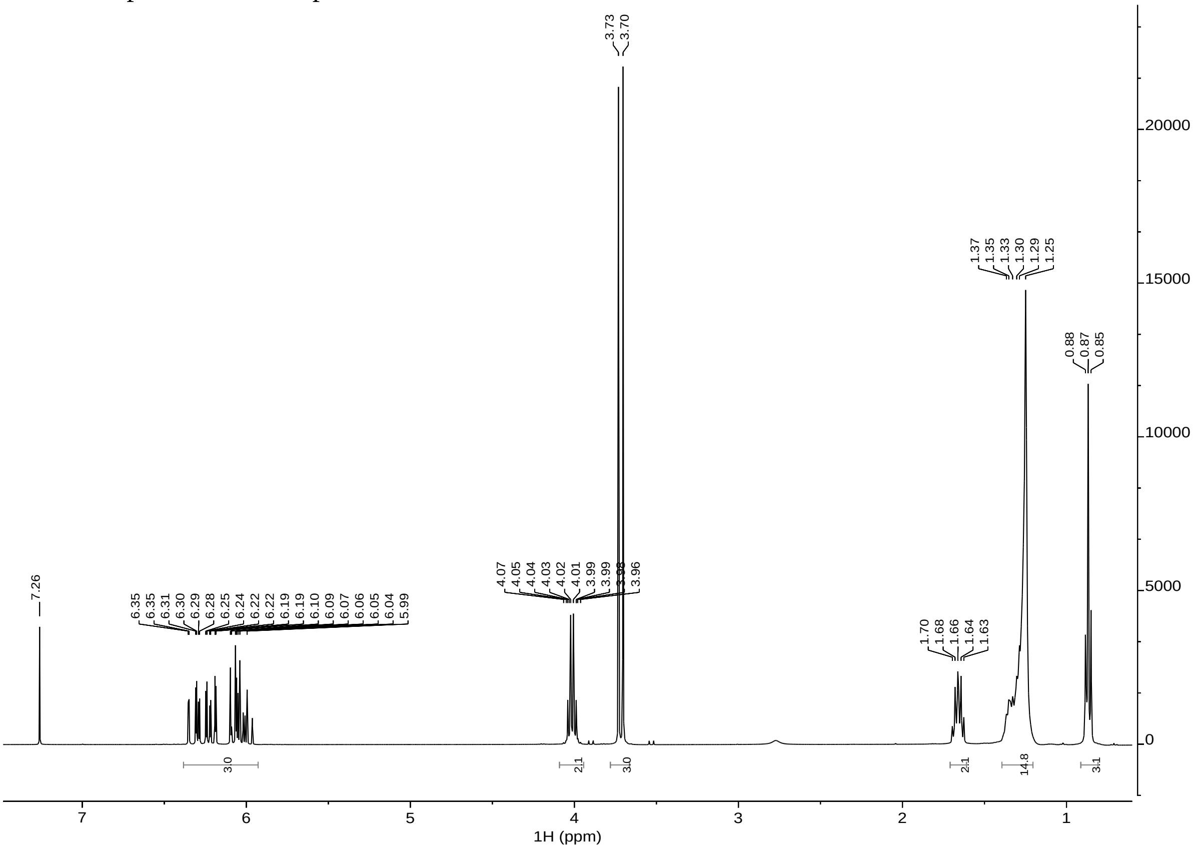

^{13}C NMR spectrum of compound **S15**

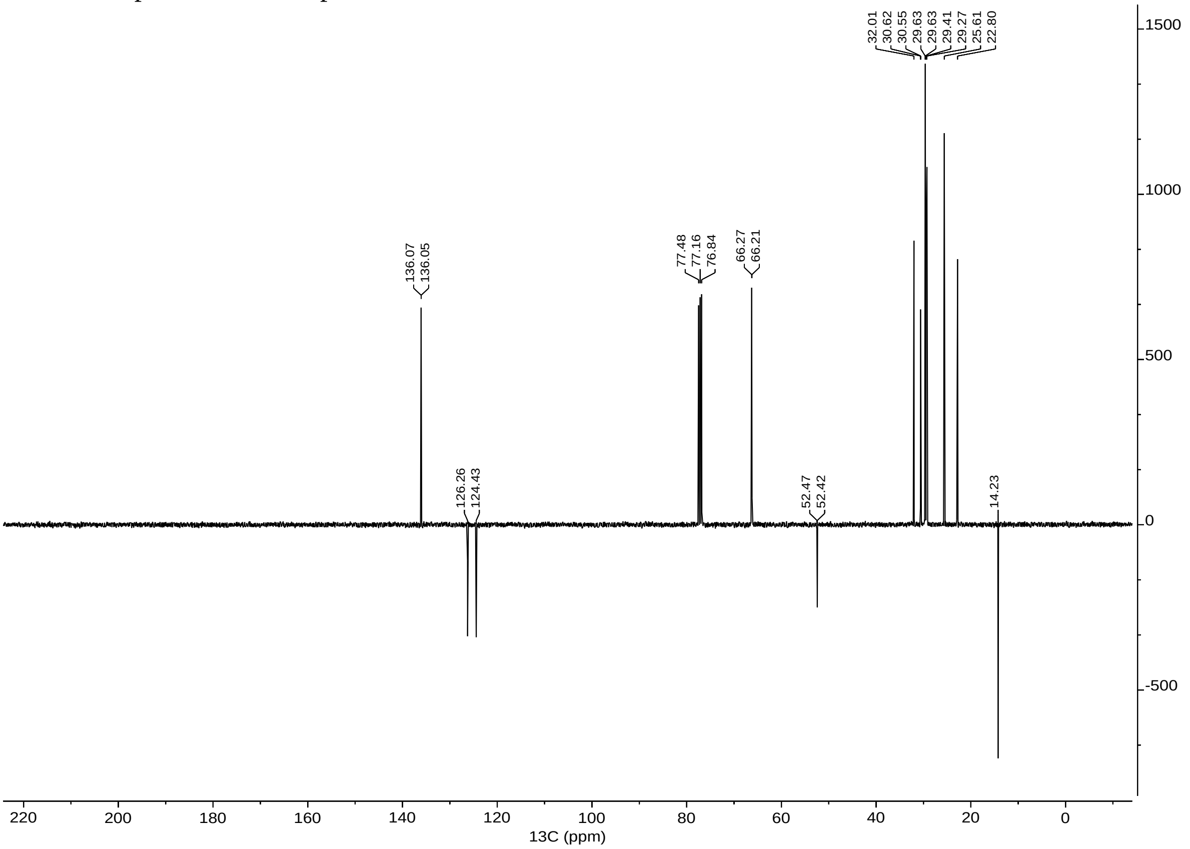

^{31}P{^{1}H} NMR spectrum of compound **S15**

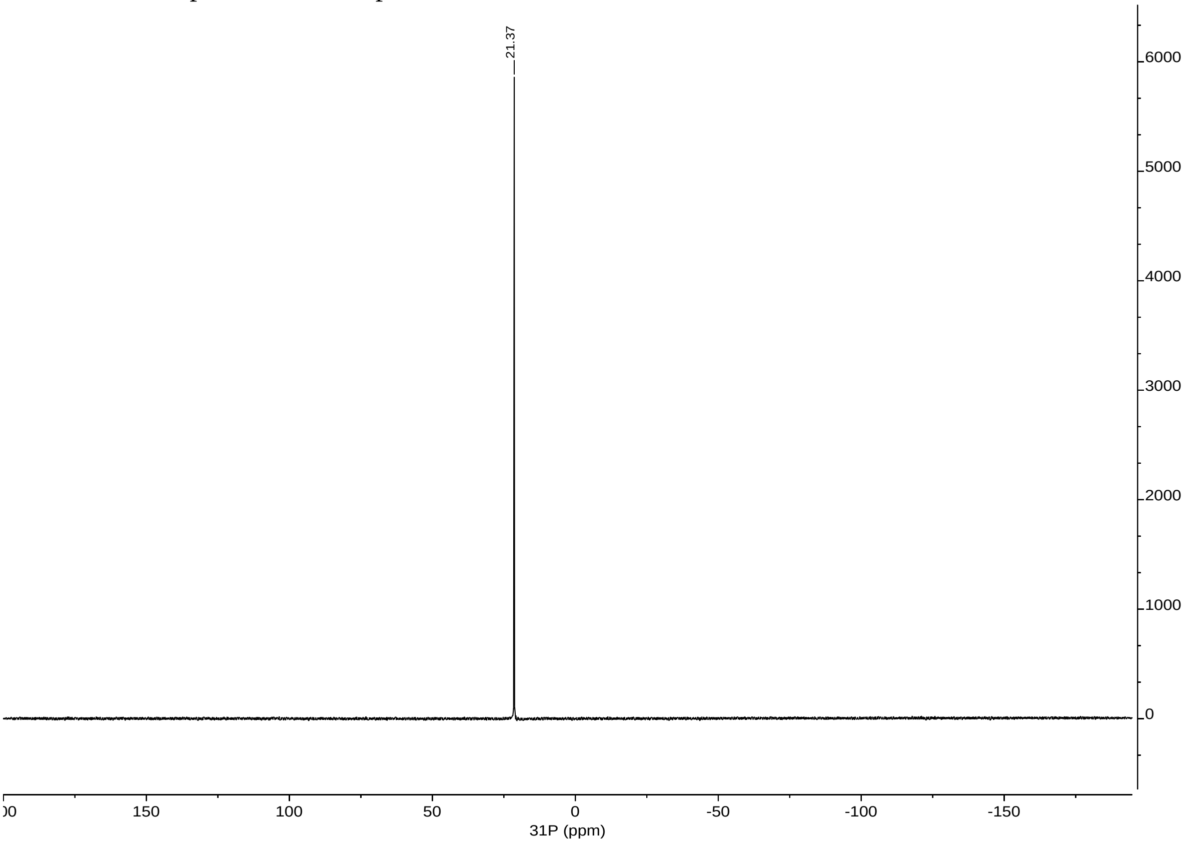

(*Z*)-Dec-4-en-1-yl methyl vinylphosphonate (S16)

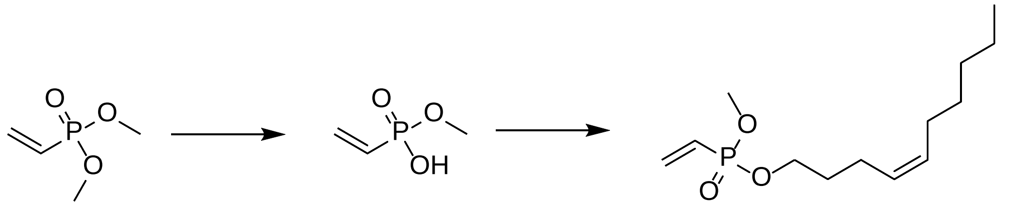

The title compound was prepared according to general methods **A** and **B2** from dimethyl vinylphosphonate (2.61 g, 19.2 mmol) in 38% overall yield (1.89 g, 7.26 mmol) as a colourless oil.

^{1}H NMR (401 MHz, $CDCl_3$): 6.36–5.97 (m, 3H, CH=CH_2), 5.44–5.38 (m, 1H, $CH_3(CH_2)_4$CH), 5.35–5.28 (m, 1H, C$H$$(CH_2)_3$O), 4.06–4.00 (m, 2H, $CH_3(CH_2)_4CHCHCH_2CH_2C\mathit{H}_2O$), 3.72 (d, 3H, *J* =11.0 Hz, OCH_3), 2.12 (tdd, 2H, *J* = 8.0, 6.8, 1.3 Hz, $CH_3(CH_2)_4CHCHC\mathit{H}_2CH_2CH_2O$), 2.01 (td, 2H, *J* = 7.1, 5.7 Hz, $CH_3(CH_2)_3C\mathit{H}_2CHCHCH_2CH_2CH_2O$), 1.73 (dq, 2H, *J* = 8.3, 6.7 Hz, $CH_3(CH_2)_4CHCHCH_2C\mathit{H}_2CH_2O$), 1.37–1.22 (m, 6H, $CH_3(C\mathit{H}_2)_3CH_2CHCHCH_2CH_2CH_2O$, 0.90–0.86 (m, 3H, C$H_3$$CH_2$).

^{13}C NMR (101 MHz, $CDCl_3$): 136.09 (d, *J* = 2.0 Hz, CH=*C*H_2), 131.46 ($CH_3(CH_2)_4\mathit{C}HCHCH_2CH_2CH_2O$), 128.01 ($CH_3(CH_2)_4CH\mathit{C}HCH_2CH_2CH_2O$), 125.34 (d, *J* = 184.6 Hz, *C*H=CH_2), 65.63 (d, *J* = 5.8 Hz, $CH_3(CH_2)_4CHCHCH_2CH_2\mathit{C}H_2O$), 52.46 (d, *J* = 5.4 Hz, OCH_3), 31.65 ($CH_3CH_2\mathit{C}H_2CH_2CH_2CHCHCH_2CH_2CH_2O$), 30.63 (d, *J* = 6.6 Hz, $CH_3(CH_2)_4CHCHCH_2\mathit{C}H_2CH_2O$), 29.47 ($CH_3CH_2CH_2\mathit{C}H_2CH_2CHCHCH_2CH_2O$), 27.32 $CH_3(CH_2)_3\mathit{C}H_2CHCHCH_2CH_2CH_2O$), 23.31 ($CH_3(CH_2)_4CHCH\mathit{C}H_2CH_2CH_2O$), 22.70 ($CH_3\mathit{C}H_2CH_2CH_2CH_2CHCHCH_2CH_2CH_2O$), 14.21 (*C*$H_3(CH_2)_4CHCHCH_2CH_2CH_2O$).

^{31}P{^{1}H} NMR (162 MHz, $CDCl_3$): 21.14.

IR ν_{max} (KBr) 3093 (w), 3011 (w), 2957 (s), 2929 (s), 2873 (m), 2855 (m), 1653 (vw), 1614 (w), 1467 (m), 1459 (m), 1400 (m), 1379 (w), 1280 (m), 1243 (s), 1070–1021 (vs), 987 (s), 815 (s).

HR-MS(ESI^+): For $C_{13}H_{25}O_3NaP$ $(M+Na)^+$ *m/z* calculated 283.14335, found 283.14328.

^{1}H NMR spectrum of compound **S16**

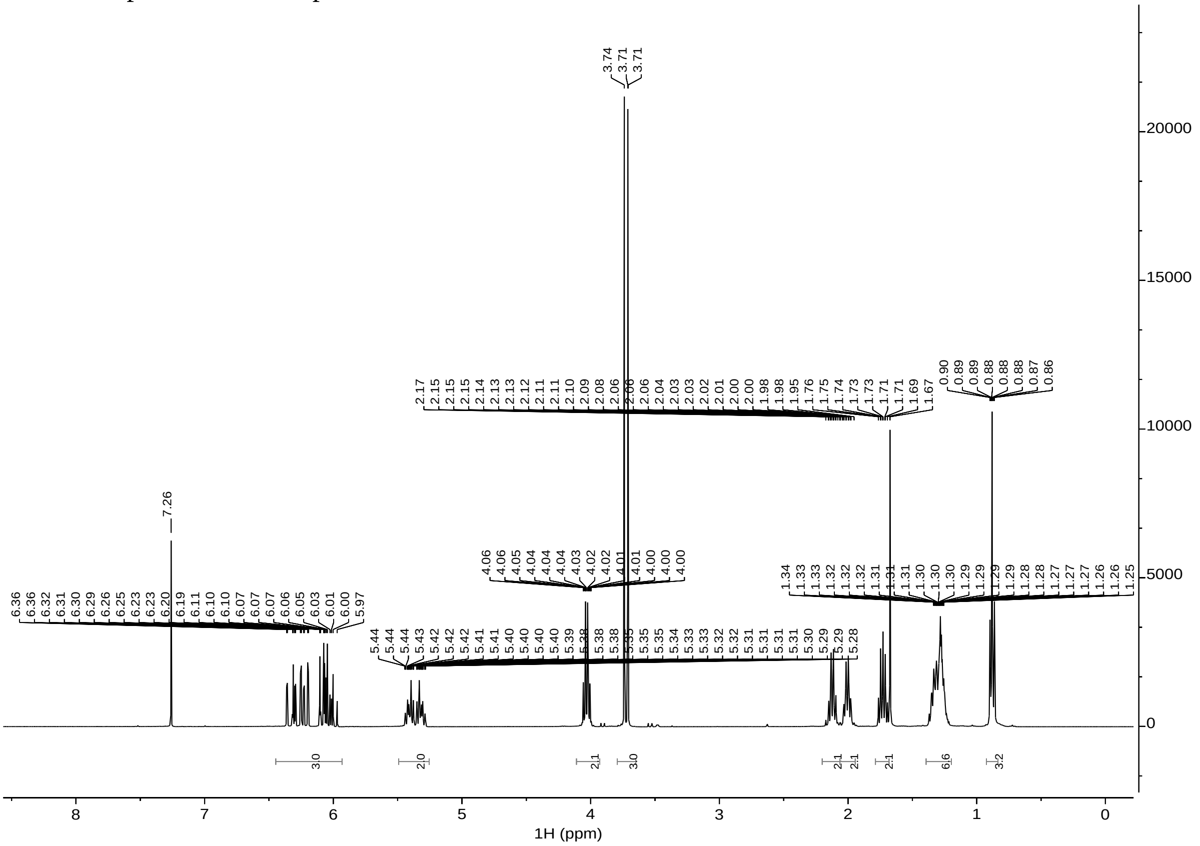

^{13}C NMR spectrum of compound **S16**

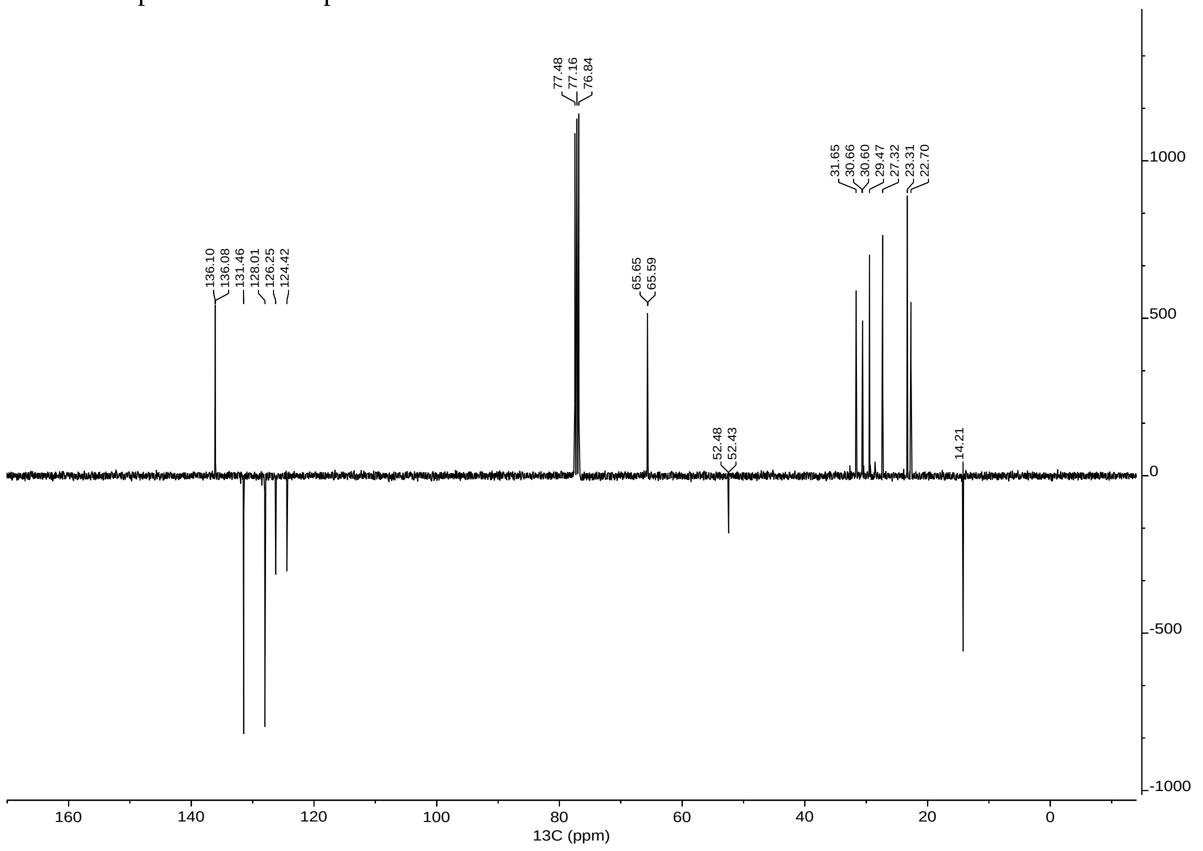

$^{31}P\{^{1}H\}$ NMR spectrum of compound **S16**

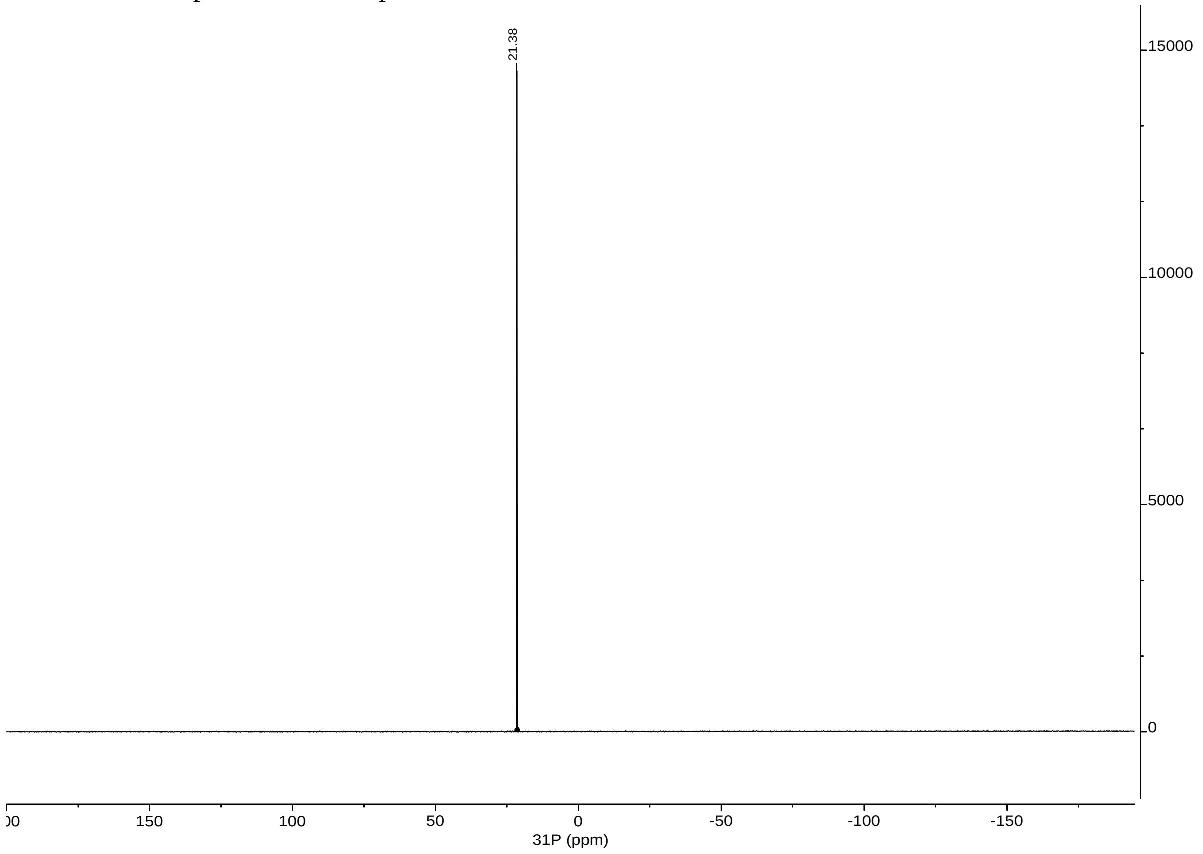

(Adamantan-1-yl)methyl methyl vinylphosphonate (S17)

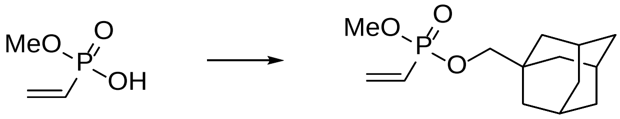

The title compound was prepared according to general method **B2** from mono methyl vinylphosphonate (4.89 g, 40.1 mmol) and (adamantan-1-yl)methanol (10.0 g, 60.1 mmol) in 84% yield (9.07 g, 33.6 mmol) as a clear oil.

^{1}H NMR (401 MHz, $CDCl_3$) δ 6.33–5.93 (m, 3H, C*H*=C*H*$_2$), 3.69 (d, J = 11.0 Hz, 3H, OC*H*$_3$), 3.54 (d, J = 5.4 Hz, 2H, OC*H*$_2$), 1.95 (p, J = 3.1 Hz, 3H, $C_{quat}CH_2C$*H*), 1.73–1.56 (m, 6H, $C_{quat}C$*H*$_2$CH), 1.50 (d, J = 2.5 Hz, 6H, *c*-(CHC*H*$_2$)$_3$).

^{13}C NMR (101 MHz, $CDCl_3$) δ 135.94 (d, J = 2.1 Hz, =*C*H$_2$), 125.18 (d, J = 184.4 Hz, P*C*H), 75.52 (d, J = 6.3 Hz, O*C*H$_2$), 52.39 (d, J = 5.7 Hz, O*C*H$_3$), 38.92 (*c*-(CH*C*H$_2$)$_3$), 36.98 ($C_{quat}CH_2CH$), 33.88 (d, J = 7.1 Hz, *C*$_{quat}$), 28.04 ($C_{quat}CH_2$*C*H).

^{31}P{^{1}H} NMR (162 MHz, $CDCl_3$) δ 21.48.

IR ν_{max} ($CHCl_3$) 3091 (vw), ~2940 (s, sh), 2907 (vs), 2851 (s), 2677 (w), 2660 (vw), 1614 (w), 1462 (m), 1453 (m), 1400 (m), 1386 (w), 1365 (w), 1279 (m), 1243 (s), 1106 (w), 1057 (s), 1020 (vs), 1004 (s), 988 (s), 943 (w), 924 (w), 817 (s), 808 (m), 433 (w).

HR-MS(ESI$^+$): For $C_{14}H_{24}O_3P$ $(M+H)^+$ *m*/*z* calculated 271.14576, found 271.14581. For $C_{14}H_{23}O_3NaP$ $(M+Na)^+$ *m*/*z* calculated 293.12770, found 293.12772.

^{1}H NMR spectrum of compound **S17**

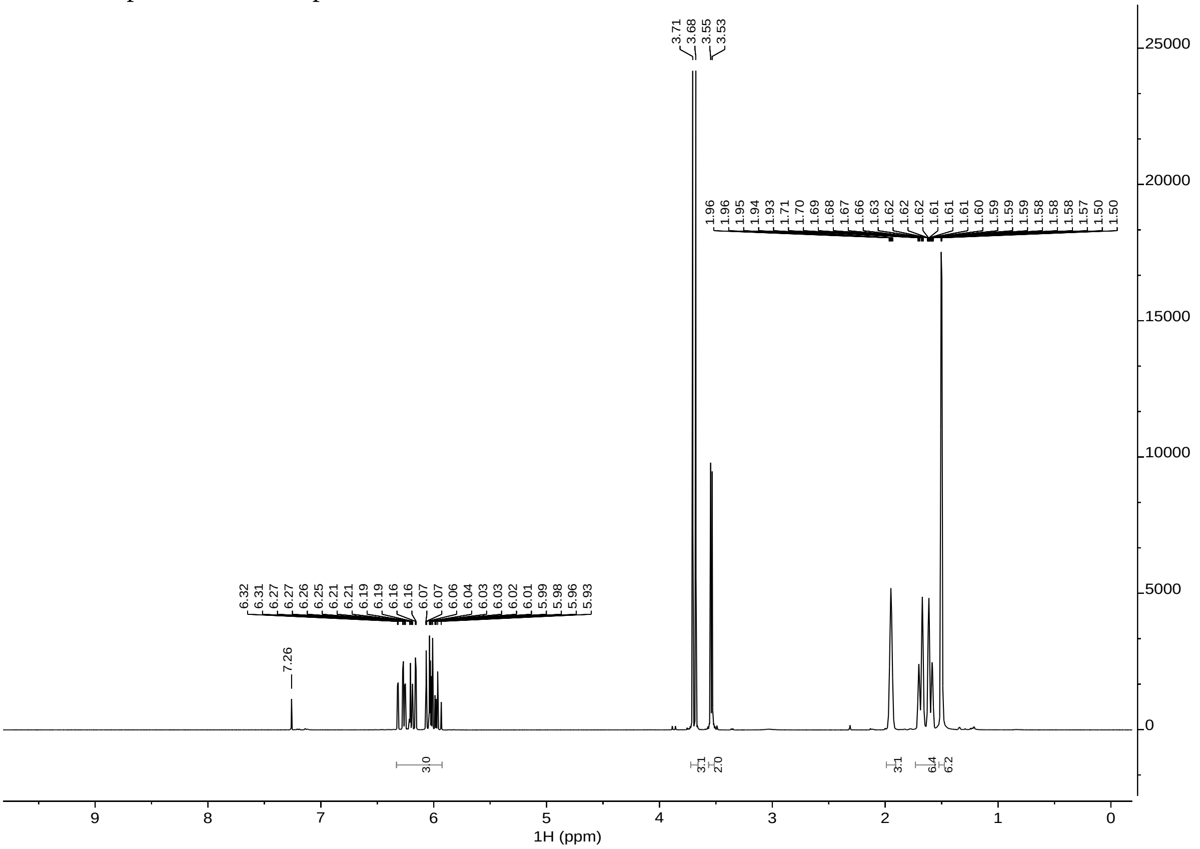

^{13}C NMR spectrum of compound **S17**

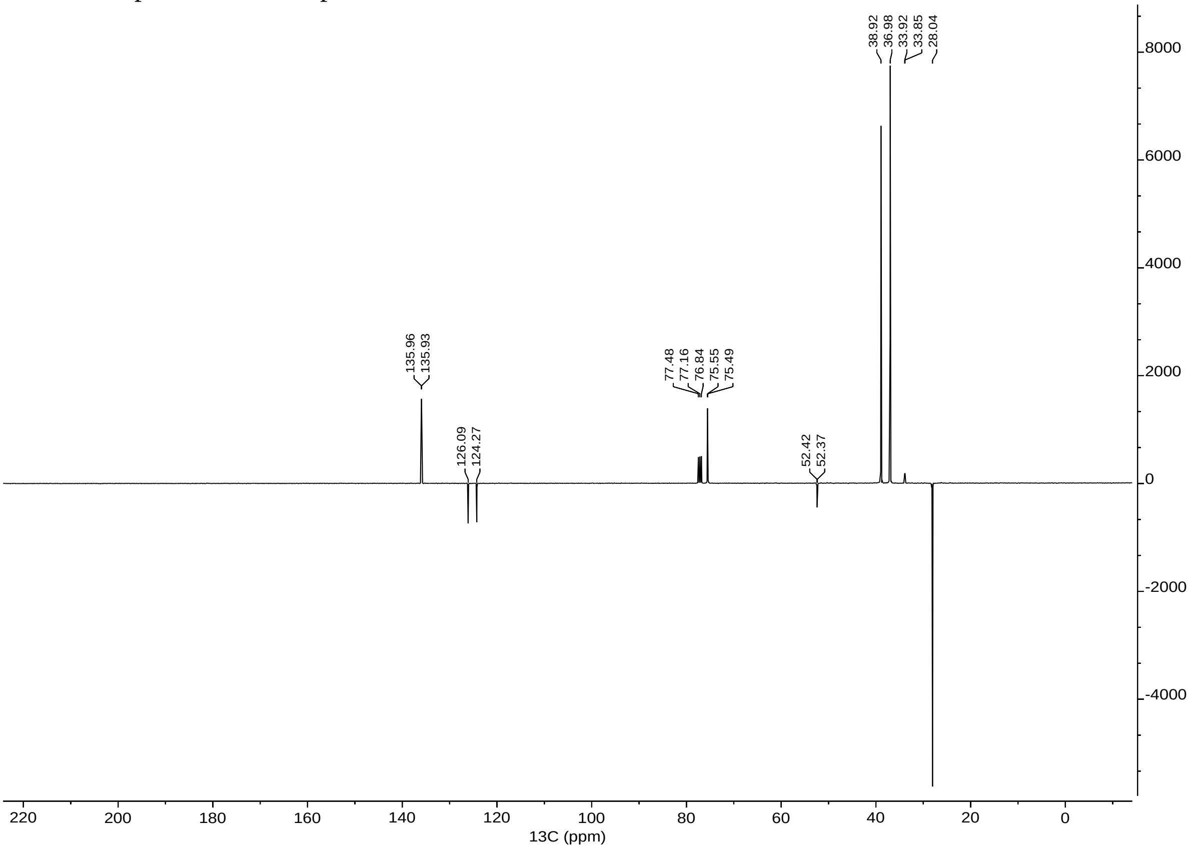

$^{31}P\{^1H\}$ NMR spectrum of compound **S17**

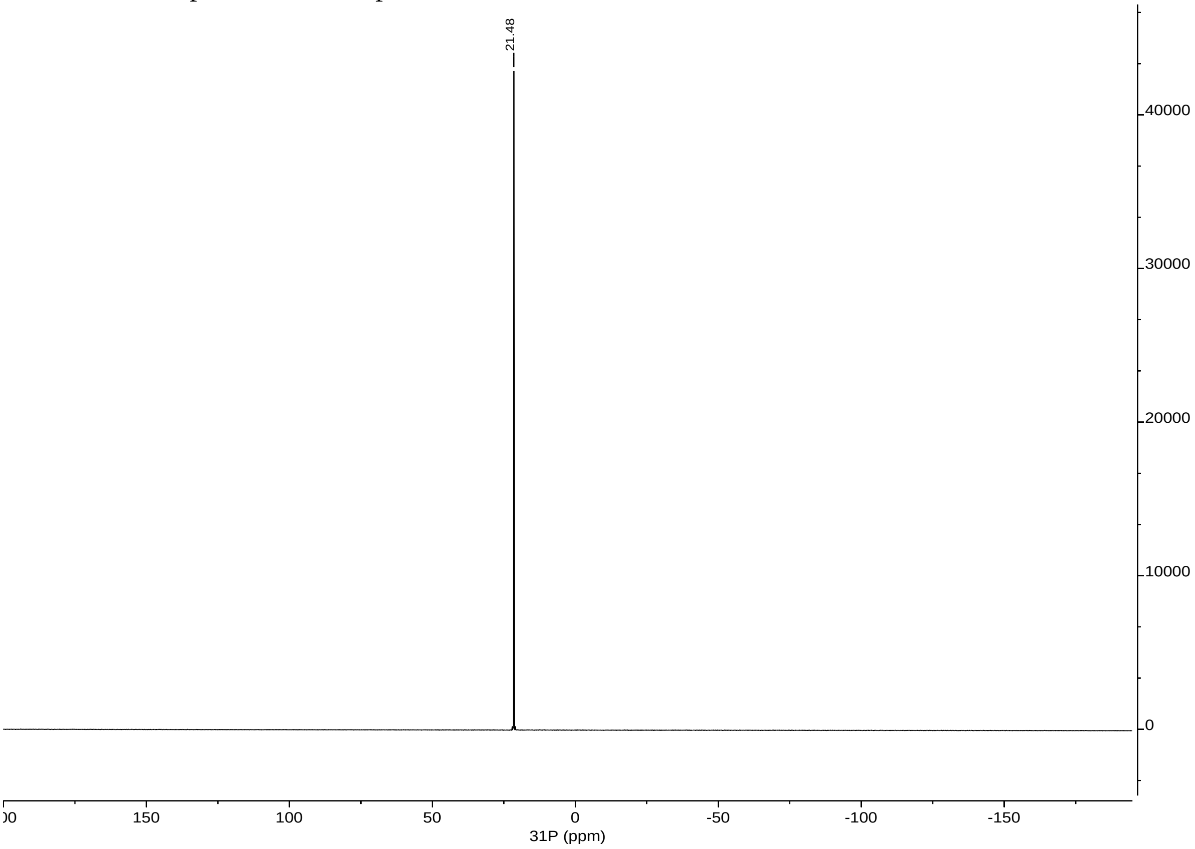

2-(Adamantan-1-yl)ethyl methyl vinylphosphonate (S18)

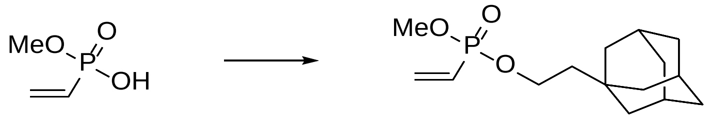

The title compound was prepared according to general method **B2** from mono methyl vinylphosphonate (5.0 g, 27.7 mmol) and 2-(adamatan-1-yl)ethanol (3.39 g, 27.7 mmol) in 62% yield (4.85 g, 17.0 mmol) as a clear oil.

^{1}H NMR (401 MHz, $CDCl_3$) δ 6.39–5.94 (m, 3H, C*H*=C*H*$_2$), 4.09 (dtd, *J* = 8.1, 7.1, 1.0 Hz, 2H, OC*H*$_2$CH$_2$), 3.72 (d, *J* = 11.0 Hz, 3H, OC*H*$_3$), 1.94 (p, *J* = 3.1 Hz, 3H, C*H*), 1.74–1.58 (m, 6H, C$_{quat}$C*H*$_2$CH), 1.53–1.46 (m, 8H, *c*-(CHC*H*$_2$)$_3$, OCH$_2$C*H*$_2$).

^{13}C NMR (101 MHz, $CDCl_3$) δ 136.09 (d, *J* = 2.0 Hz, =*C*H$_2$), 125.39 (d, *J* = 183.9 Hz, P*C*H), 62.63 (d, *J* = 5.8 Hz, O*C*H$_2$), 52.50 (d, *J* = 5.6 Hz, O*C*H$_3$), 44.43 (d, *J* = 6.2 Hz, OCH$_2$*C*H$_2$), 42.63 (*c*-(CH*C*H$_2$)$_3$), 37.10 (C$_{quat}$*C*H$_2$CH), 31.92 (*C*$_{quat}$), 28.67 (*C*H).

^{31}P{^{1}H} NMR (162 MHz, $CDCl_3$) δ 21.44.

IR ν_{max}($CHCl_3$) 3091 (vw), ~3000 (s), 2953 (m), 2927 (vs), 2905 (vs), 2850 (s), ~2800 (vw, sh), ~2687 (vw, sh), ~2680 (w), ~2660 (w), ~2640 (vw), 1614 (w), 1451 (m), 1400 (m), 1347 (w), 1242 (vs), 1186 (m), 1099 (m), 1056 (vs), ~1027 (vs, sh), 1015 (vs), 998 (s), 973 (m), 883 (w), 820 (s), 815 (s), ~810 (s, sh).

HR-MS(ESI$^+$): For $C_{15}H_{26}O_3P$ (M+H)$^+$ *m/z* calculated 285.16141, found 285.16116.

^{1}H NMR spectrum of compound **S18**

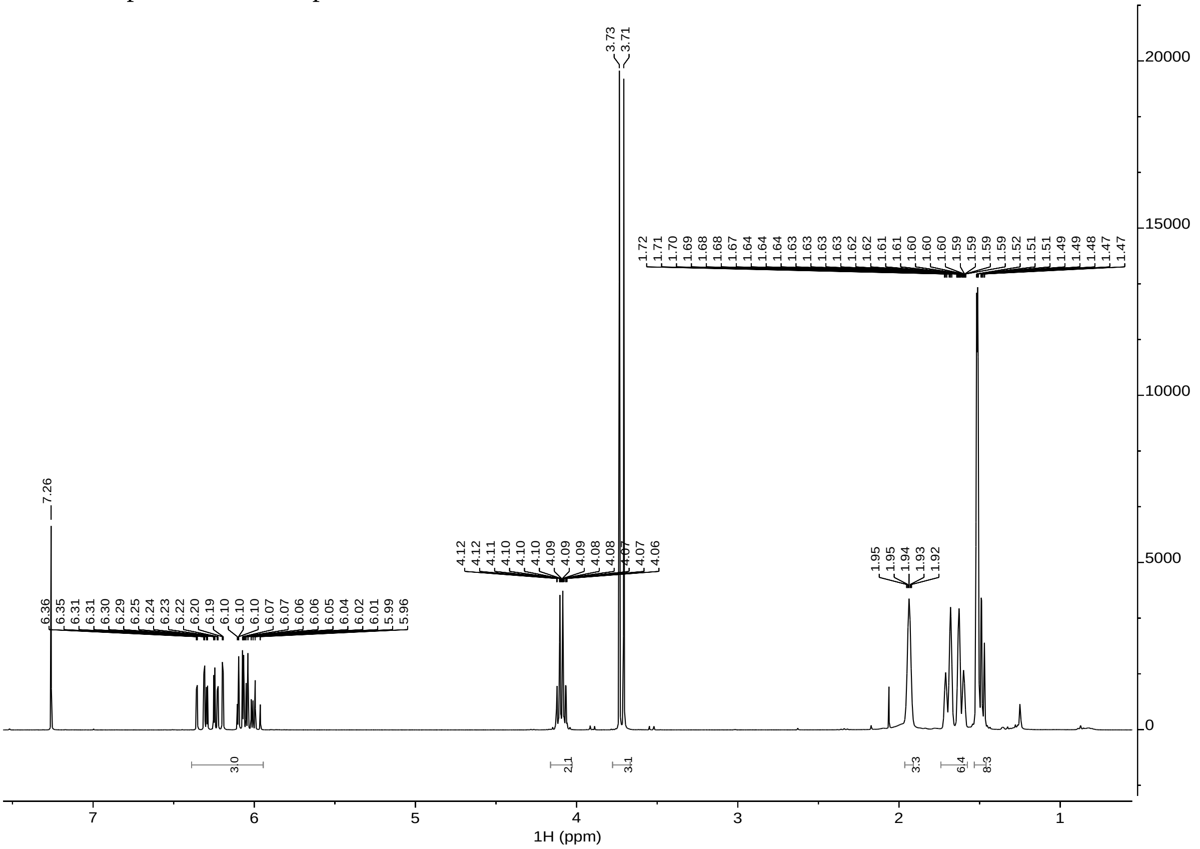

^{13}C NMR spectrum of compound **S18**

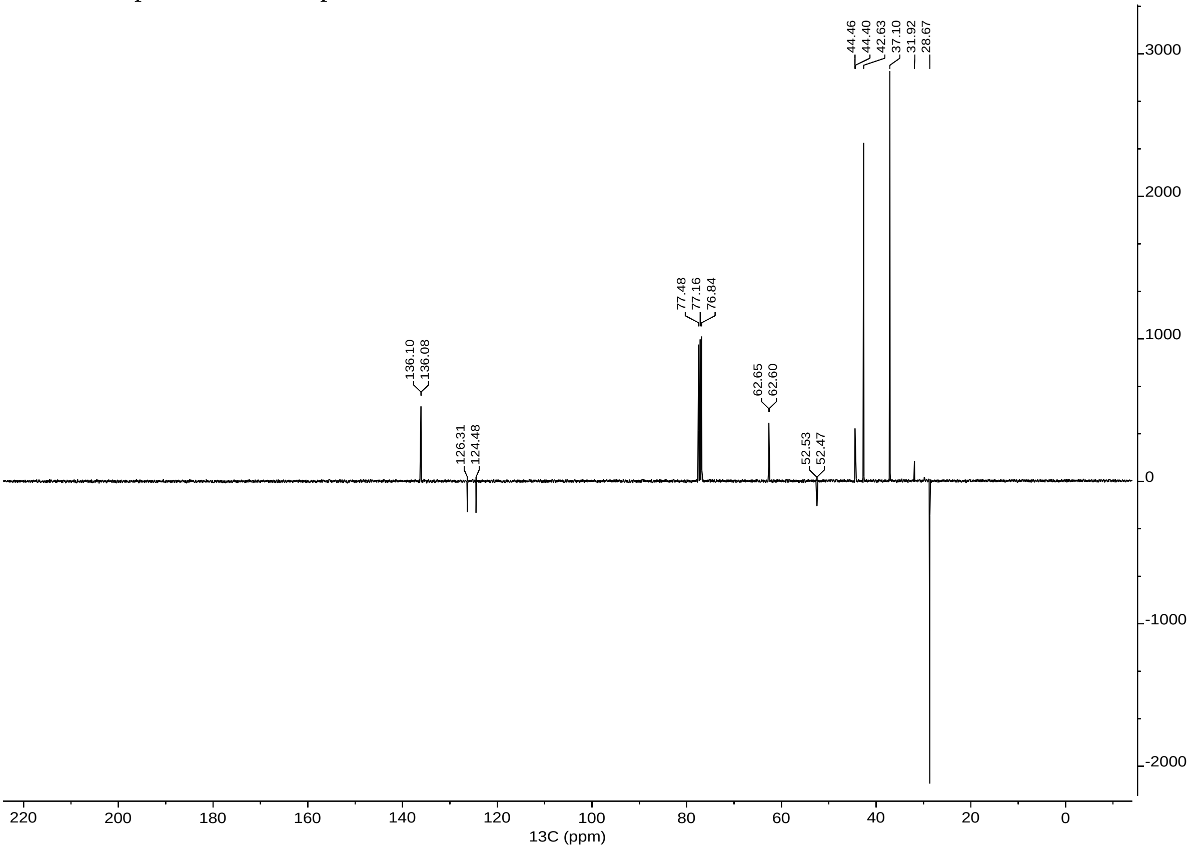

^{31}P{^{1}H} NMR spectrum of compound **S18**

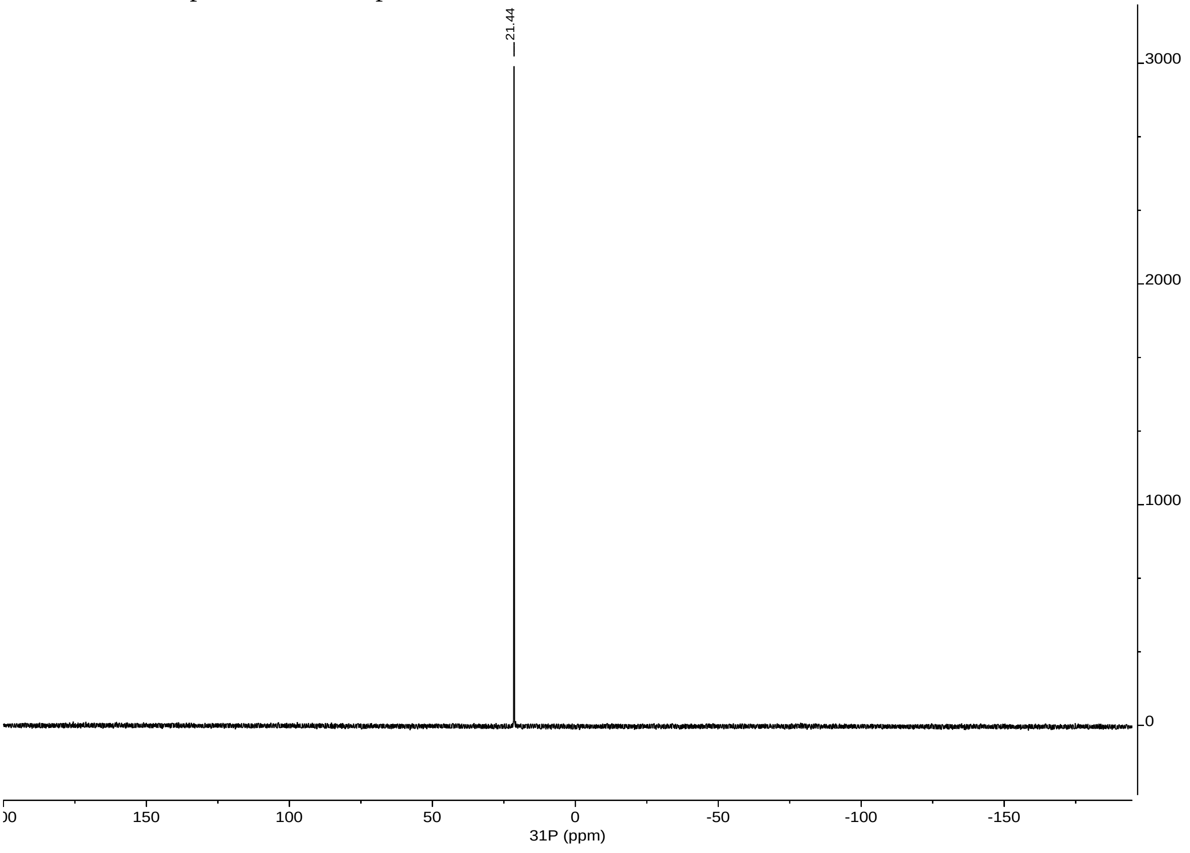

Mono isobutyl vinylphosphonate (S19)

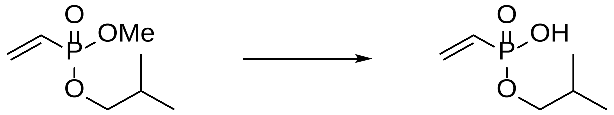

The title compound was prepared according to general method **A** from isobutyl methyl vinylphosphonate **S1** (2.25 g, 12.6 mmol) in 97% yield (2.00 g, 12.2 mmol) as a colourless oil.

^{1}H NMR (401 MHz, $CDCl_3$) δ 6.45 (brs, 1H, O*H*), 6.36–5.98 (m, 3H, C*H*=C*H*$_2$), 3.77 (t, *J* = 6.6 Hz, 2H, OC*H*$_2$), 1.95 (dt, *J* = 13.3, 6.7 Hz, 1H, OCH_2C*H*), 0.94 (d, *J* = 6.7 Hz, 6H, C*H*$_3$).

^{13}C NMR (101 MHz, $CDCl_3$) δ 134.83 (=*C*H_2), 126.25 (d, *J* = 189.6 Hz, P*C*H), 71.77 (d, *J* = 6.6 Hz, O*C*H_2), 29.20 (d, *J* = 7.0 Hz, OCH_2*C*H), 18.83 (d, *J* = 1.8 Hz, *C*H_3).

^{31}P{^{1}H} NMR (162 MHz, $CDCl_3$) δ 22.26.

IR ν_{max}($CHCl_3$) 3088 (w), 2962 (m), 2877 (m), 2853 (w), 2585 (m, br), 2278 (m, br), 1673 (m), 1615 (m), 1472 (m), 1403 (m), 1370 (w), 1278 (m), 1206 (s), 1032 (vs), 989 (s), 854 (m), 732 (m), 610 (m).

HR-MS(ESI$^+$): For $C_6H_{13}O_3NaP$ $(M+Na)^+$ *m/z* calculated 187.04945, found 187.04957.

^{1}H NMR spectrum of compound **S19**

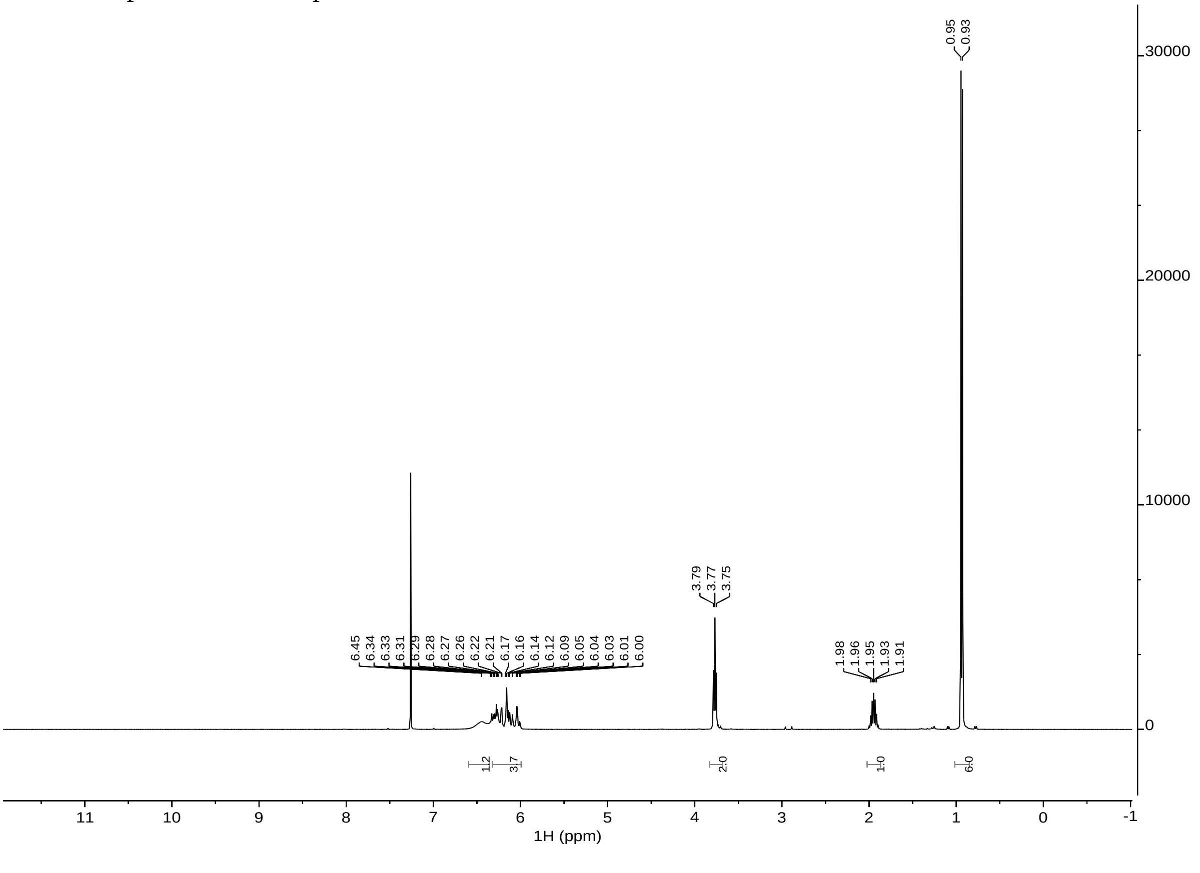

^{13}C NMR spectrum of compound **S19**

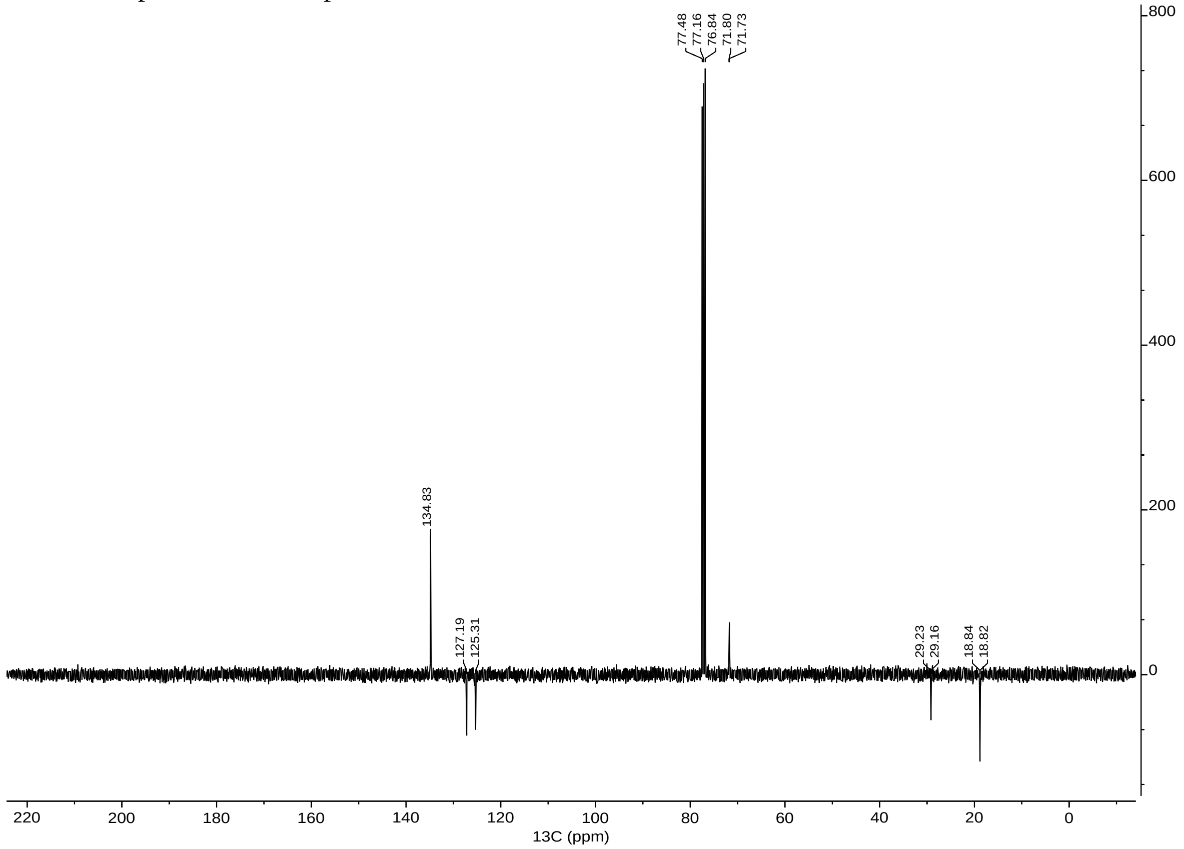

^{31}P{^{1}H} NMR spectrum of compound **S19**

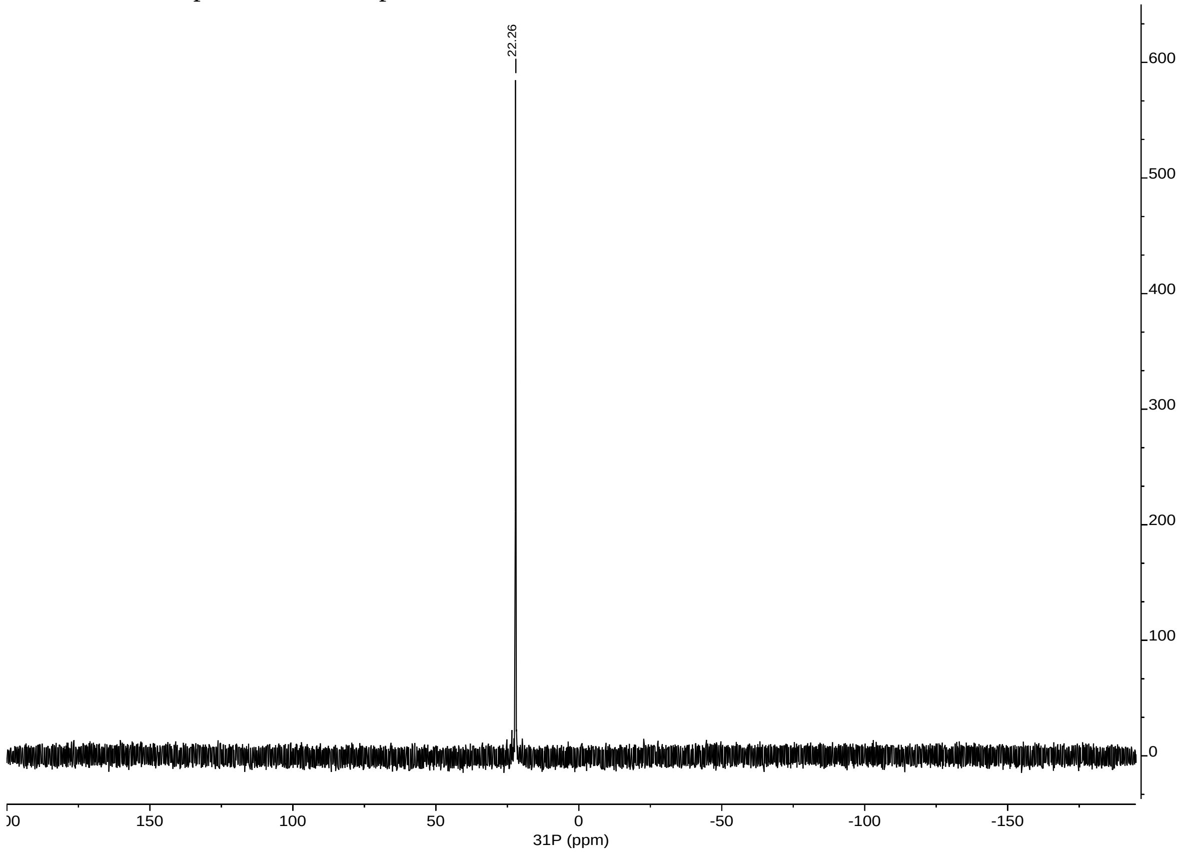

Mono butyl vinylphosphonate (S20)

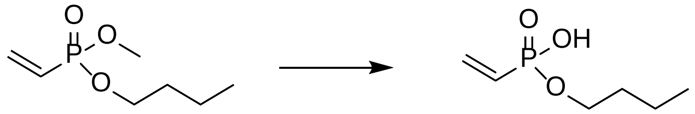

The title compound was prepared according to general method **A** from butyl methyl vinylphosphonate **S2** (5.80 g, 32.6 mmol) in 82% yield (4.39 g, 26.7 mmol) as a colourless oil.

^{1}H NMR (400 MHz, $CDCl_3$): 9.78 (s, 1H, PO*H*), 6.34–5.99 (m, 3H, C*H*=C*H*$_2$), 4.04–3.98 (m, 2H, $CH_3(CH_2)_2CH_2CH_2O$), 1.69–1.62 (m, 2H, $CH_3CH_2CH_2CH_2O$), 1.45–1.35 (m, 2H, $CH_3CH_2CH_2CH_2O$), 0.95–0.90 (m, 3H, $CH_3(CH_2)_2CH_2O$).

^{13}C NMR (101 MHz, $CDCl_3$): 134.76 (d, J = 2.0, CH=*C*H$_2$), 126.34 (d, J = 189.4 Hz, *C*H=CH$_2$), 65.46 (d, J = 6.0 Hz, $CH_3CH_2CH_2CH_2O$), 32.49 (d, J = 7.2 Hz, $CH_3CH_2CH_2CH_2O$), 18.84 ($CH_3CH_2CH_2CH_2O$), 13.71 ($CH_3(CH_2)_2CH_2O$).

^{31}P{^{1}H} NMR (162 MHz, $CDCl_3$): 21.72.

IR ν_{max} (KBr) 3090 (vw), 2963 9s), 2935 (s), 2876 (m), 2847 (m), 2739 (w, vbr), 2671 (w, vbr), 2337 (m, vbr), 2177 (w, vbr), 1666 (m, vbr), 1615 (m), 1476 (w), 1466 (m), 1459 (w), 1403 (m), 1276 (w), 1199 (s), 1062 (s), 1031 (vs), 986 (vs), 899 (w).

HR-MS(ESI$^+$): For $C_6H_{13}O_3NaP$ (M+Na)$^+$ m/z calculated 187.04945, found 187.04951.

^{1}H NMR spectrum of compound **S20**

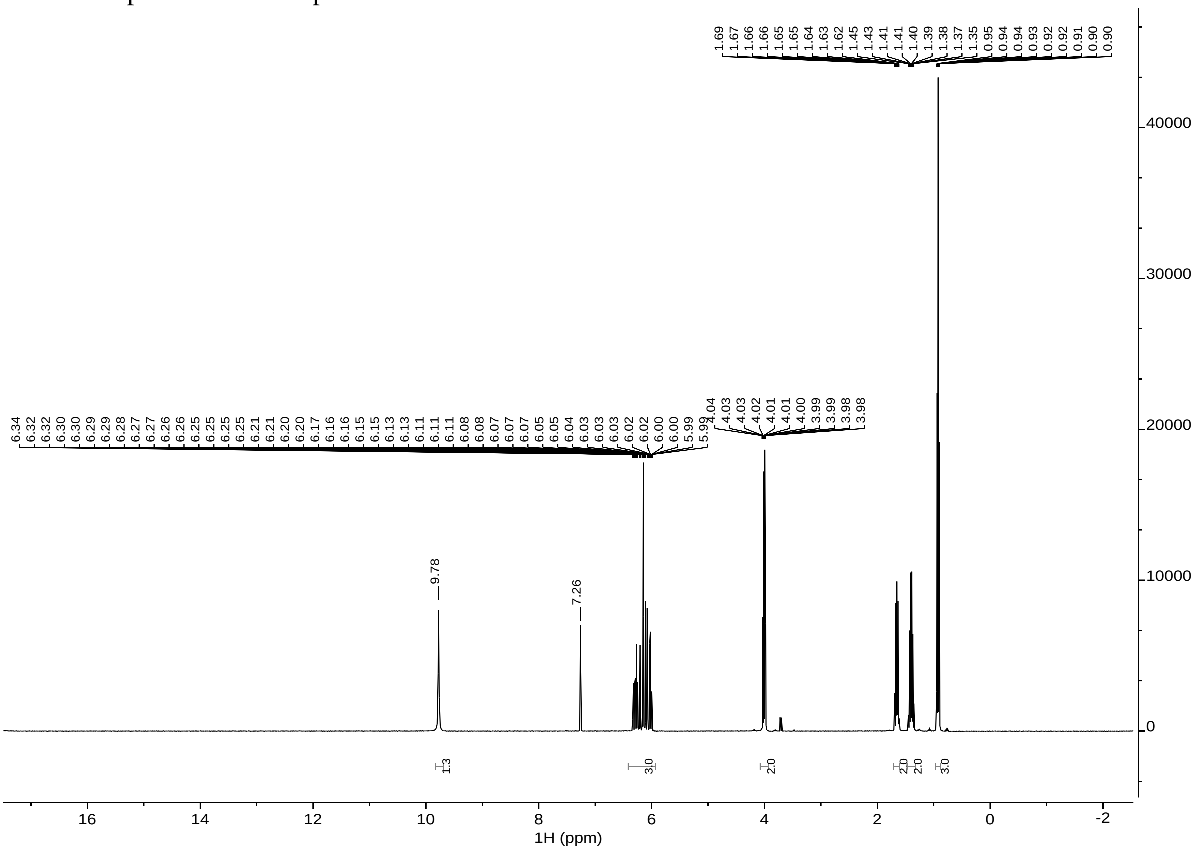

^{13}C NMR spectrum of compound **S20**

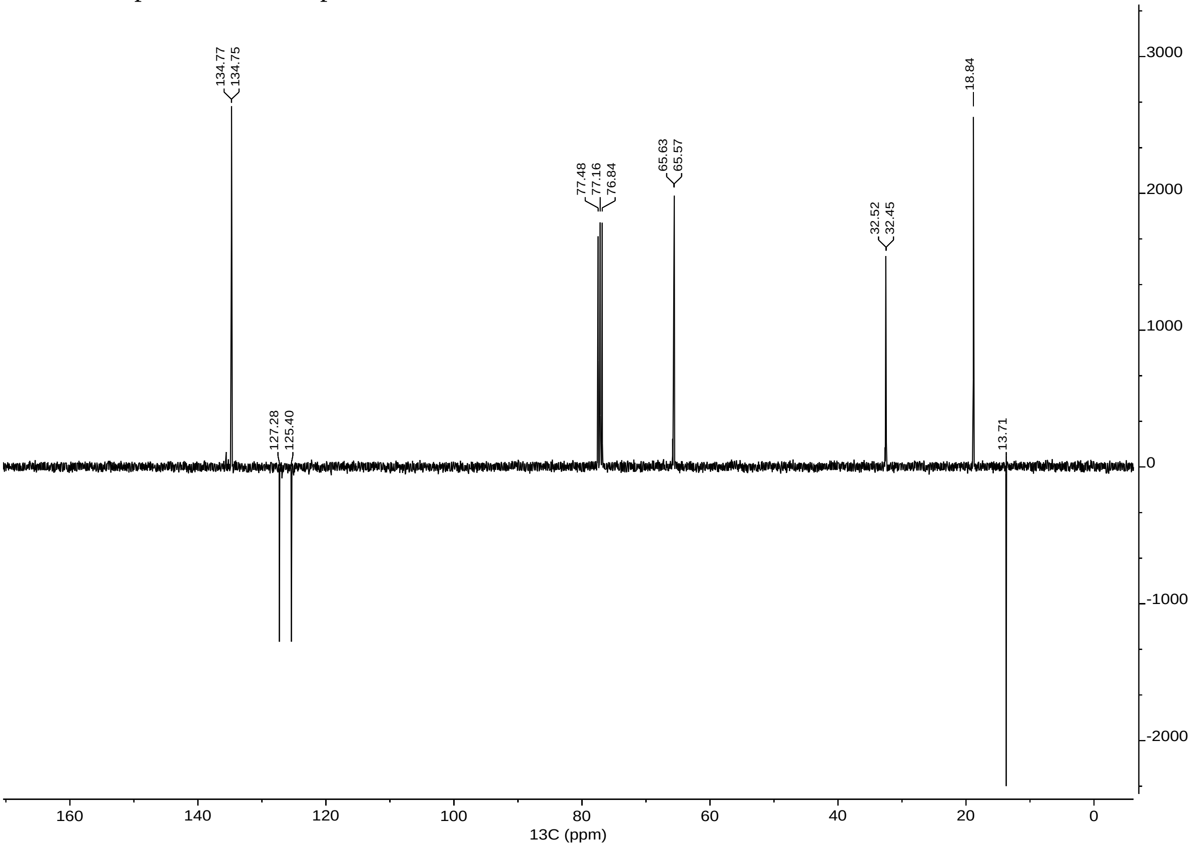

$^{31}P\{^{1}H\}$ NMR spectrum of compound **S20**

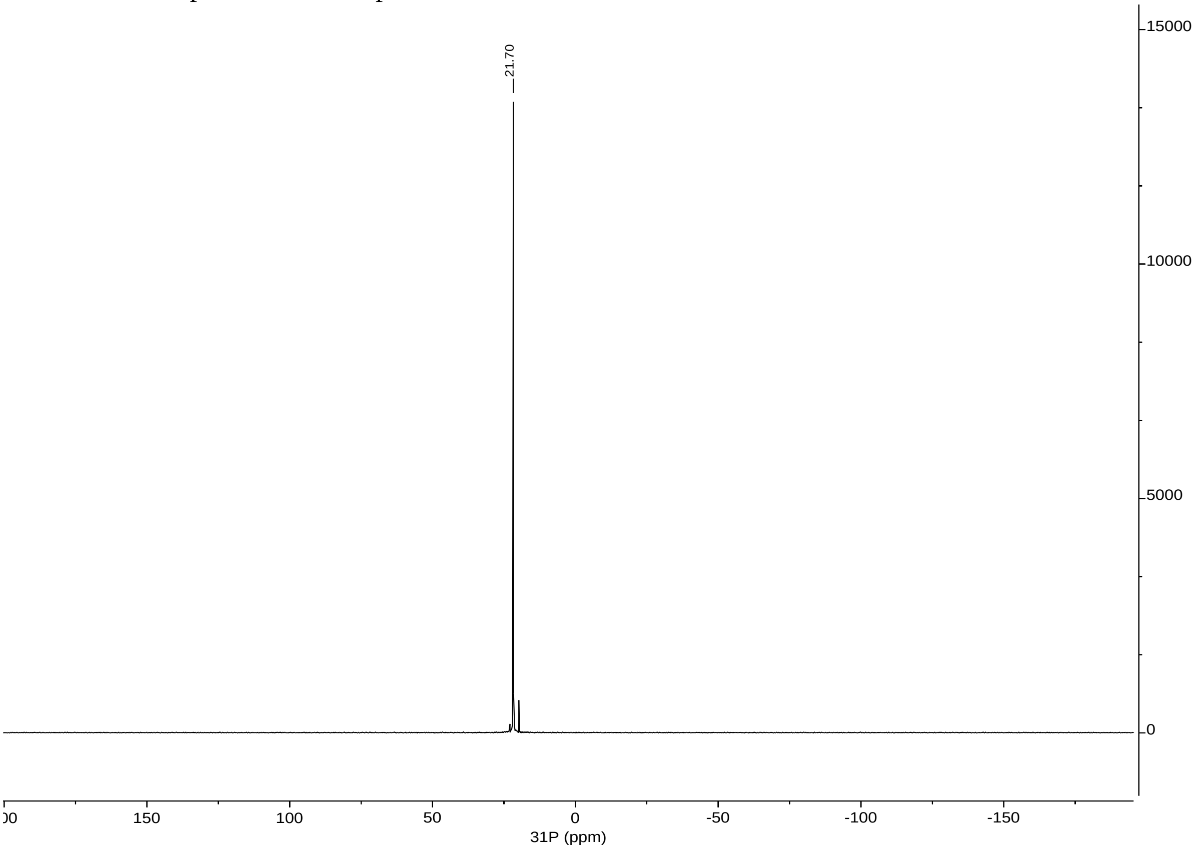

Mono 4,4,4-trifluorobutyl vinylphosphonate (S21)

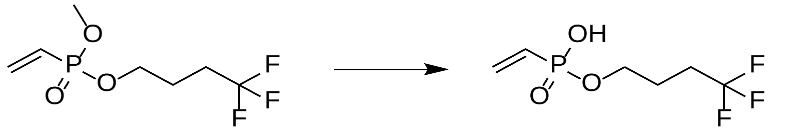

The title compound was prepared according to general method **A** from methyl 4,4,4-trifluorobutyl vinylphosphonate **S3** (5.10 g, 22.0 mmol) in 81% yield (3.90 g, 17.9 mmol) as a colourless oil.

^{1}H NMR (400 MHz, $CDCl_3$): 12.31 (s, 1H, O*H*), 6.35–6.02 (m, 3H, C*H*=C*H*$_2$), 4.09–4.04 (m, 2H, $CF_3CH_2CH_2C$*H*$_2$O), 2.28–2.17 (m, 2H, CF_3C*H*$_2CH_2CH_2O$), 1.97–1.90 (m, 2H, CF_3CH_2C*H*$_2CH_2O$).

^{13}C NMR (101 MHz, $CDCl_3$) δ 135.64 (d, *J* = 1.9 Hz, CH=*C*H$_2$), 127.06 (q, *J* = 275.8 Hz, *C*F$_3$), 125.63 (d, *J* = 189.5 Hz, *C*H=CH$_2$), 64.05 (d, *J* = 5.7 Hz, O*C*H$_2$), 30.45 (q, *J* = 29.3 Hz, *C*H$_2$CF$_3$), 23.33 (dq, *J* = 6.2, 3.1 Hz, OCH$_2$*C*H$_2$).

^{31}P{^{1}H} NMR (162 MHz, $CDCl_3$) δ 21.75.

^{19}F NMR (376 MHz, $CDCl_3$) δ -66.36 (t, *J* = 10.8 Hz).

IR ν_{max} 3097 (w), 3019 (w), 2965 (m), 2871 (w), 2589 (w, br), 2579 (w, br), 1616 (m), 1475 (w, sh), 1453 (m), 1403 (m), 1341 (m), 1257 (s), 1239 (s), 1156 (vs), 1135 (vs), 1083–1026 (vs), 991 (s).

HR-MS(ESI^{+}): For $C_6H_{11}O_3F_3P$ $(M+H)^{+}$ *m/z* calculated 219.03924, found 219.03930.

^{1}H NMR spectrum of compound **S21**

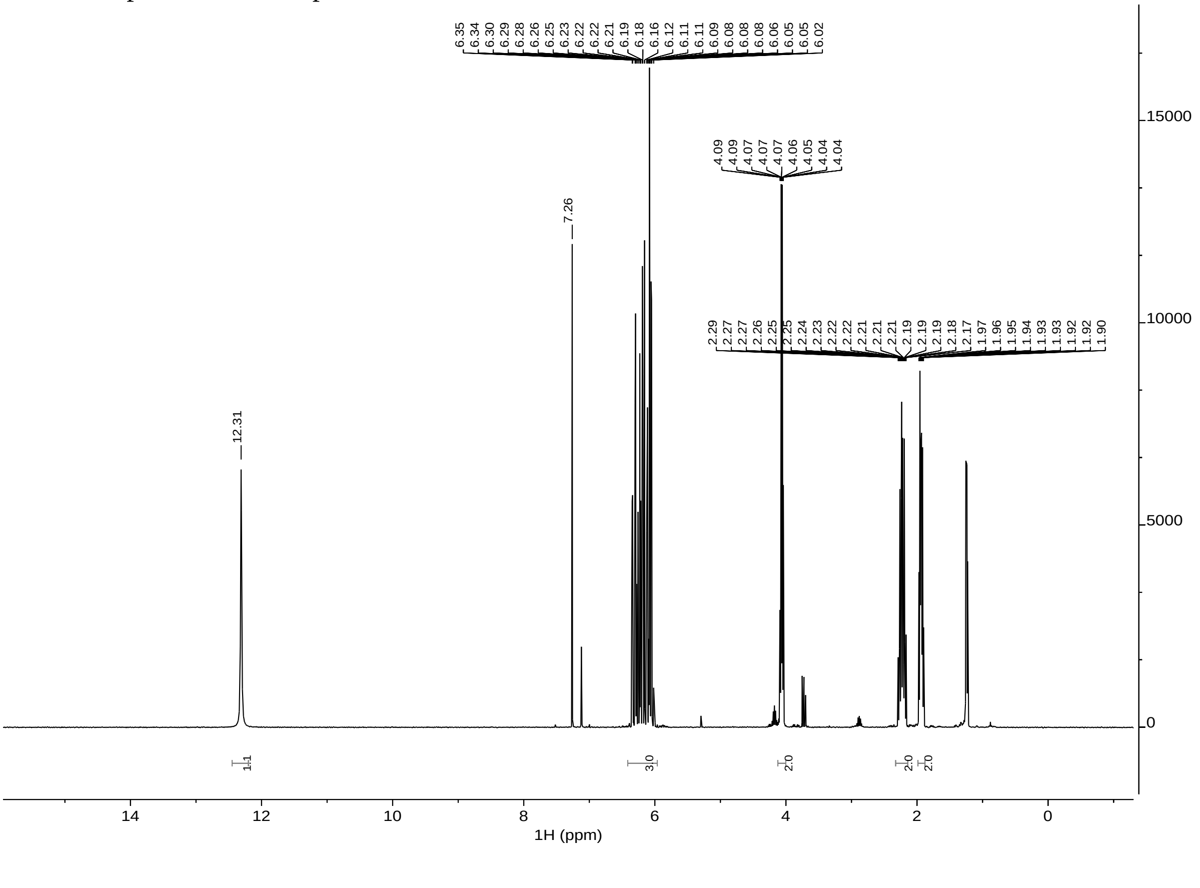

^{13}C NMR spectrum of compound **S21**

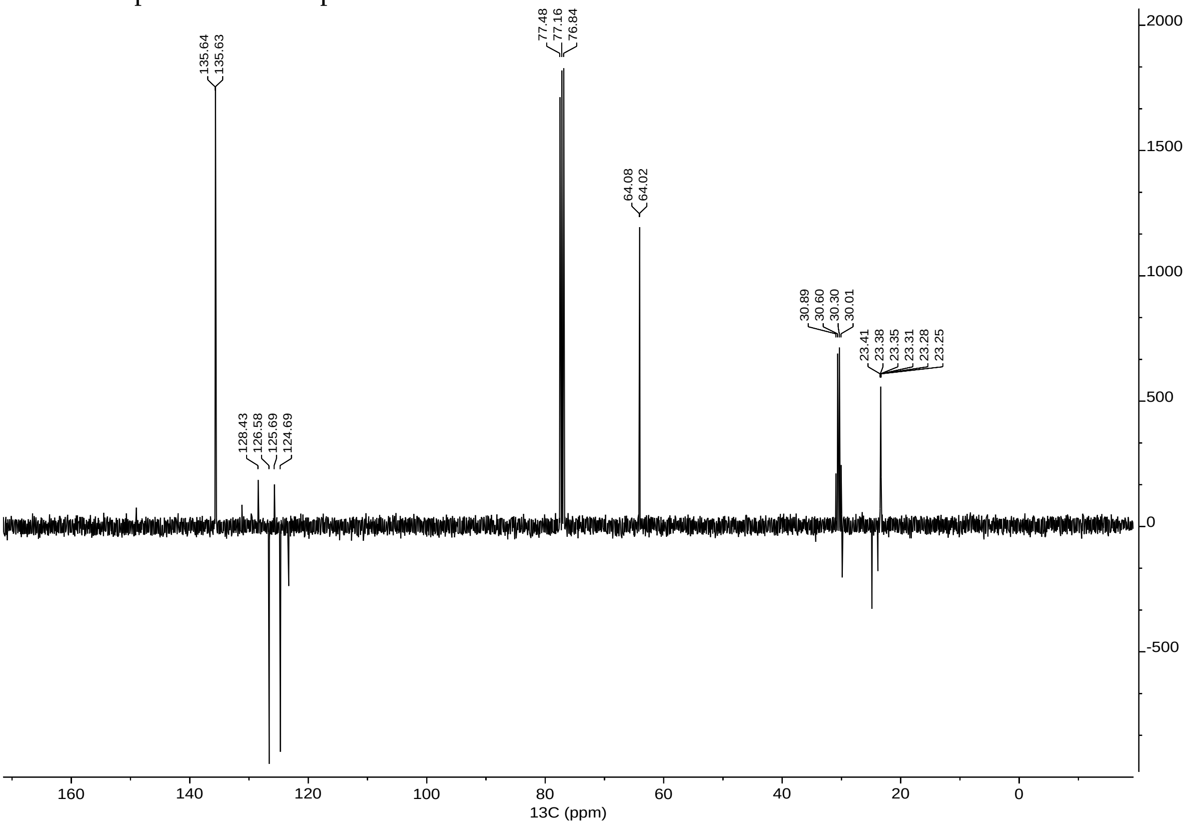

$^{31}P\{^{1}H\}$ NMR spectrum of compound **S21**

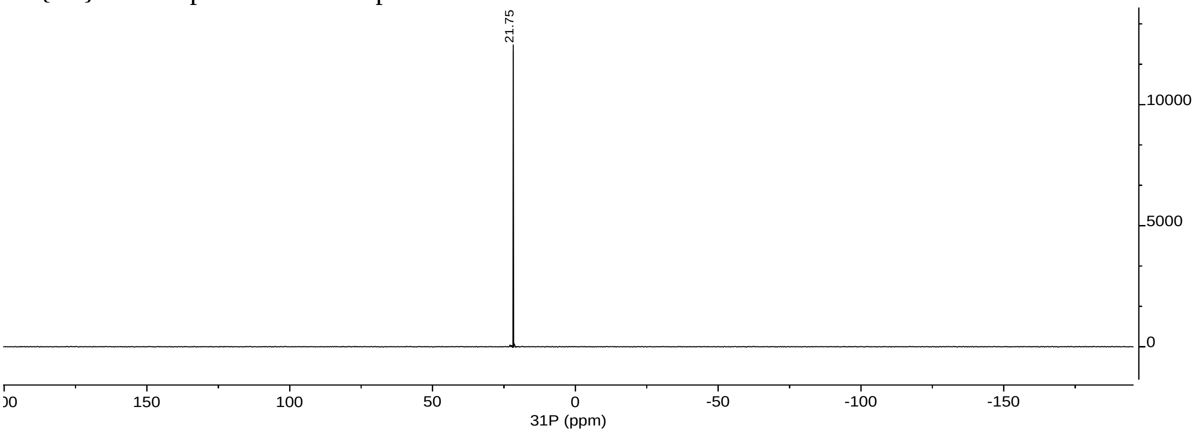

^{19}F NMR spectrum of compound **S21**

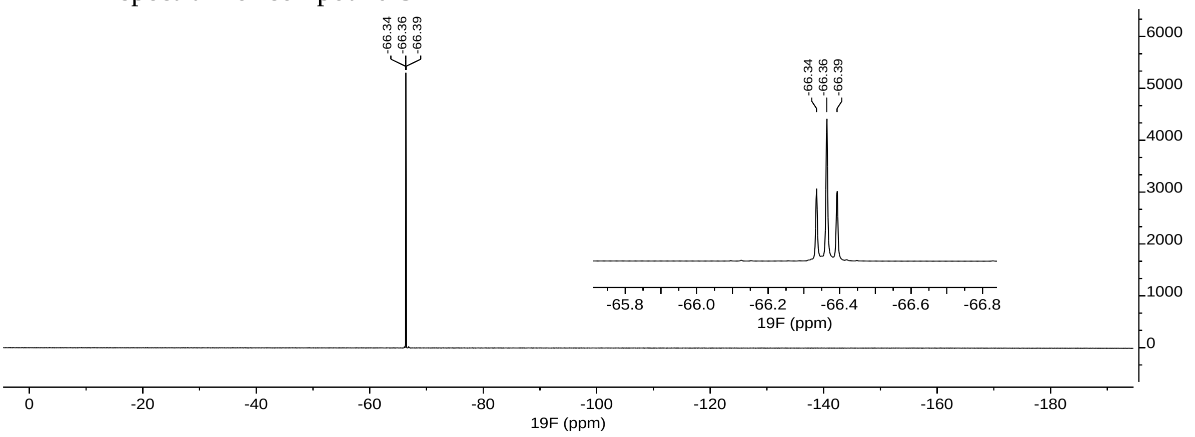

Mono pentyl vinylphosphonate (S22)

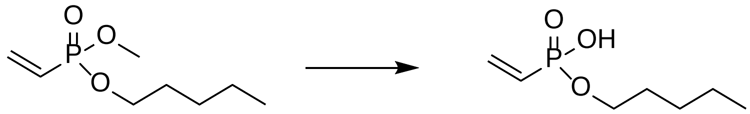

The title compound was prepared according to general method **A** from methyl pentyl vinylphosphonate **S4** (5.90 g, 30.7 mmol) in 79% yield (4.43 g, 24.9 mmol) as a colourless oil.

^{1}H NMR (400 MHz, $CDCl_3$): 10.12 (s, 1H, PO*H*), 6.33–5.98 (m, 3H, C*H*=C*H*$_2$), 4.01–3.96 (m, 2H, $CH_3(CH_2)_2CH_2C$*H*$_2$O), 1.70–1.64 (m, 2H, $CH_3(CH_2)_2C$*H*$_2CH_2O$), 1.39–1.28 (m, 4H, $CH_3(C$*H*$_2)_2CH_2CH_2O$), 0.92–0.87 (m, 3H, C*H*$_3(CH_2)_3CH_2O$).

^{13}C NMR (101 MHz, $CDCl_3$): 134.70 (d, *J* = 1.5 Hz, CH=*C*H$_2$), 126.40 (d, *J* = 189.2 Hz, *C*H=CH$_2$), 65.89 (d, *J* = 6.2 Hz, $CH_3(CH_2)_2CH_2$*C*H$_2$O), 30.18 (d, *J* = 6.4 Hz, $CH_3(CH_2)_2$*C*H$_2CH_2O$), 27.75, 22.35 (CH_3(*C*H$_2)_2CH_2CH_2O$), 14.07 (*C*H$_3(CH_2)_3CH_2O$).

^{31}P{^{1}H} NMR (162 MHz, $CDCl_3$): 21.44.

IR ν_{max} (KBr) 3089 (w), 3085 (w), 2960 (s), 2933 (s), 2874 (m), 2862 (m), 2735 (w, vbr), 2673 (w, vbr), 2338 (m, vbr), 2170 (w, vbr), 1642 (m, vbr), 1615 (m), 1468 (m), 1460 (w), 1403 (m), 1381 (w), 1277 (w), 1230–1200 (s), 1051 (s), 1024 (vs), 985 (vs).

HR-MS(APCI$^+$): For $C_7H_{16}O_3P$ $(M+H)^+$ *m/z* calculated 179.08316, found 179.08300.

^{1}H NMR spectrum of compound **S22**

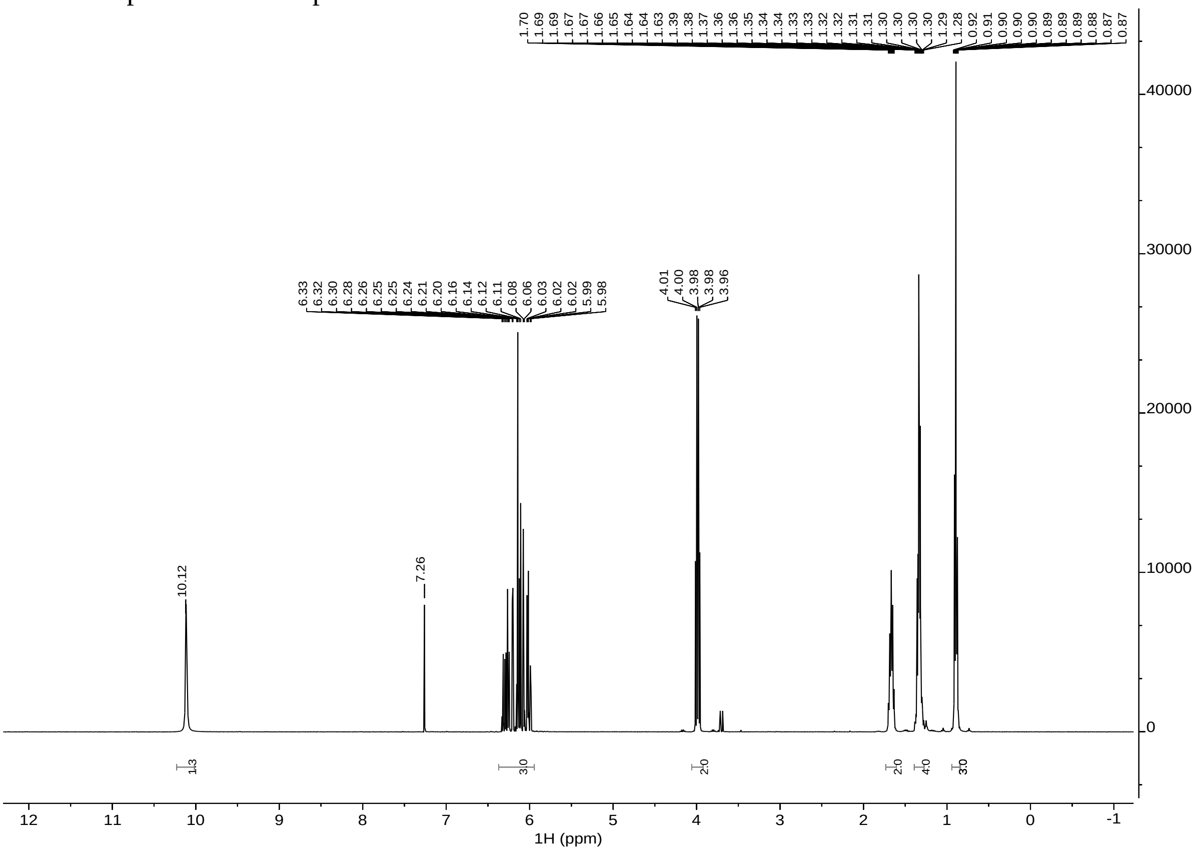

^{13}C NMR spectrum of compound **S22**

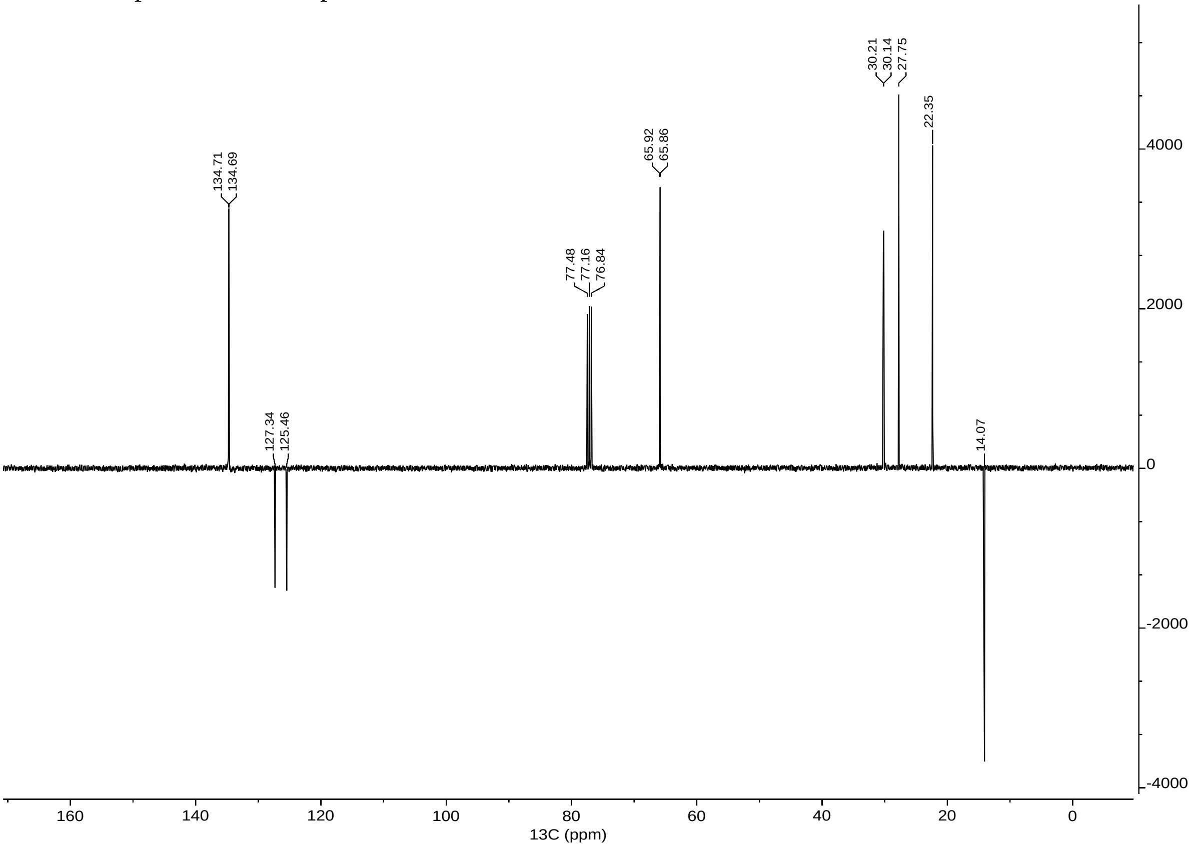

^{31}P{^{1}H} NMR spectrum of compound **S22**

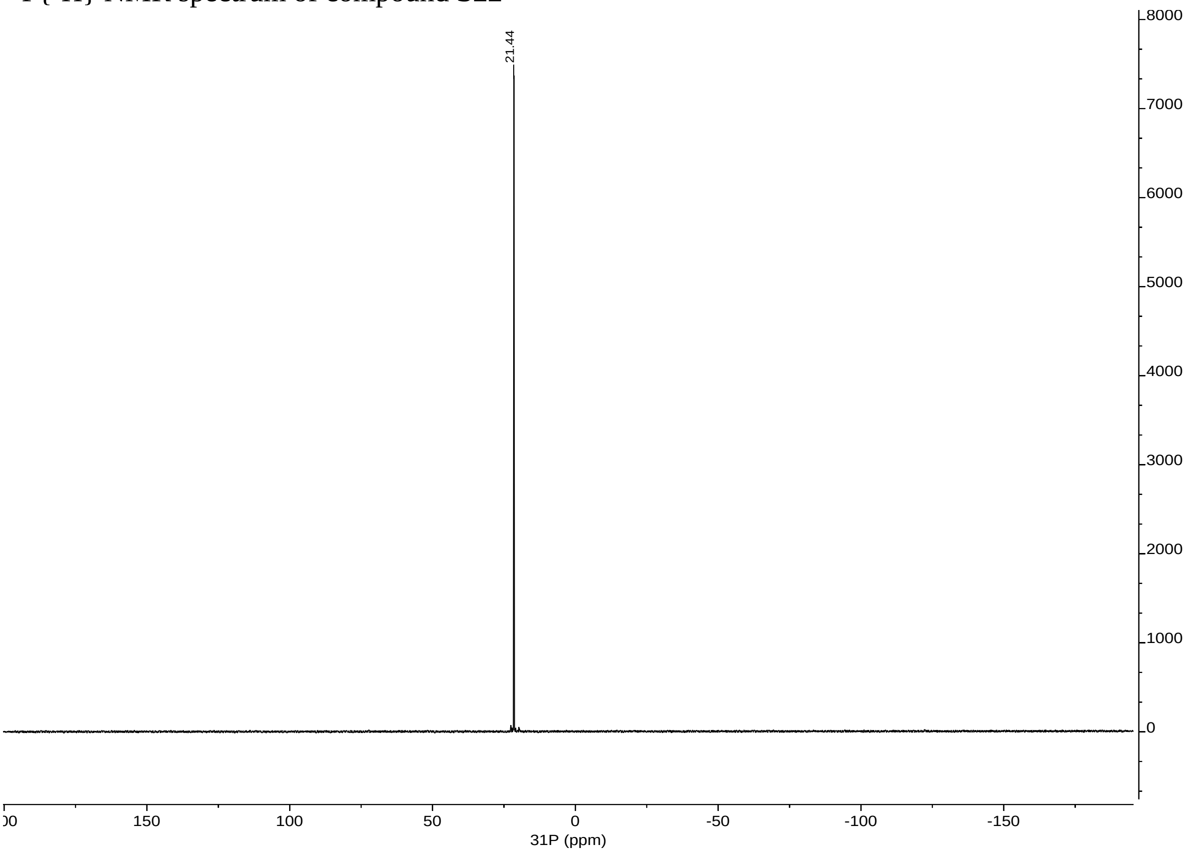

Mono hexyl vinylphosphonate (S23)

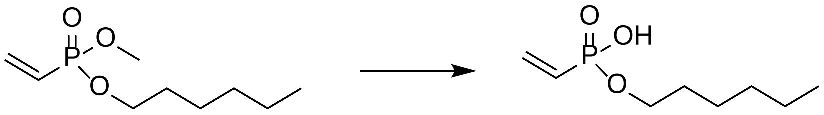

The title compound was prepared according to general method **A** from hexyl methyl vinylphosphonate **S5** (5.12 g, 24.8 mmol) in 79% yield (3.77 g, 19.6 mmol) as a colourless oil.

^{1}H NMR (400 MHz, CDCl$_3$): 10.53 (s, 1H, PO*H*), 6.34–5.99 (m, 3H, C*H*=C*H*$_2$), 4.02–3.97 (m, 2H, CH$_3$(CH$_2$)$_3$CH$_2$C*H*$_2$O), 1.70–1.63 (m, 2H, CH$_3$(CH$_2$)$_3$C*H*$_2$CH$_2$O), 1.40–1.23 (m, 6H, CH$_3$(C*H*$_2$)$_3$CH$_2$CH$_2$O), 0.90–0.86 (m, 3H, C*H*$_3$(CH$_2$)$_4$CH$_2$O).

^{13}C NMR (101 MHz, CDCl$_3$): 134.75 (d, J = 2.1 Hz, CH=*C*H$_2$), 126.34 (d, J = 190.0 Hz, *C*H=CH$_2$), 65.93 (d, J = 6.0 Hz, CH$_3$(CH$_2$)$_3$CH$_2$*C*H$_2$O), 30.45 (d, J = 6.7 Hz, CH$_3$(CH$_2$)$_3$*C*H$_2$CH$_2$O), 31.47, 25.29, 22.66 (CH$_3$(*C*H$_2$)$_3$CH$_2$CH$_2$O), 14.12 (*C*H$_3$(CH$_2$)$_4$CH$_2$O).

^{31}P{^{1}H} NMR (162 MHz, CDCl$_3$): 21.48.

IR ν_{max} (KBr) 3089 (vw), 2959 (s), 2932 (s), 2873 (s), 2861 (s), 2732 (m, vbr), 2672 (m, vbr), 2337 (m,vbr), 2181 (m, vbr), 1672 (m, vbr), 1615 (w), 1468 (m), 1462 (m), 1458 (m), 1276 (m), 1199 (s), 1059 (s), 1042 (vs), 1020 (vs), 986 (vs), 895 (w).

HR-MS(ESI$^+$): For $C_8H_{17}O_3NaP$ (M+Na)$^+$ *m/z* calculated 215.08075, found 241.08069.

^{1}H NMR spectrum of compound **S23**

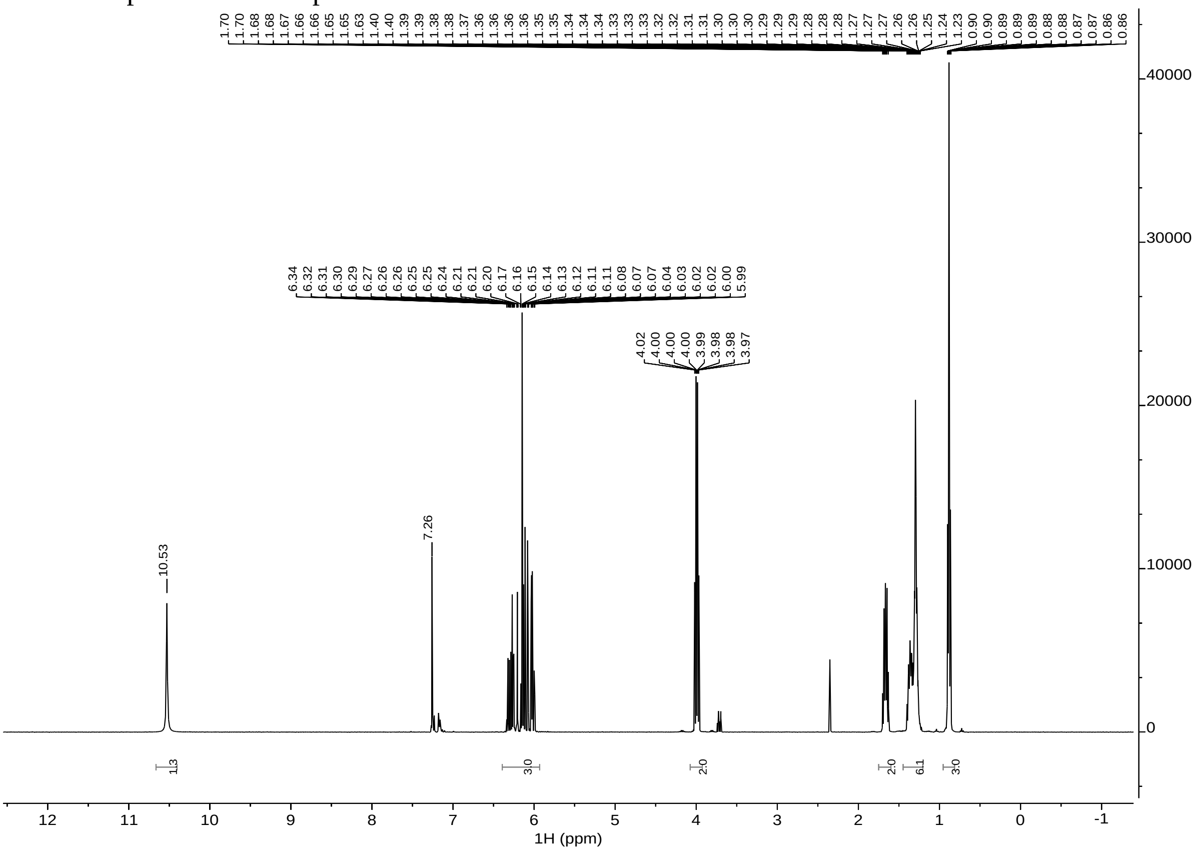

^{13}C NMR spectrum of compound **S23**

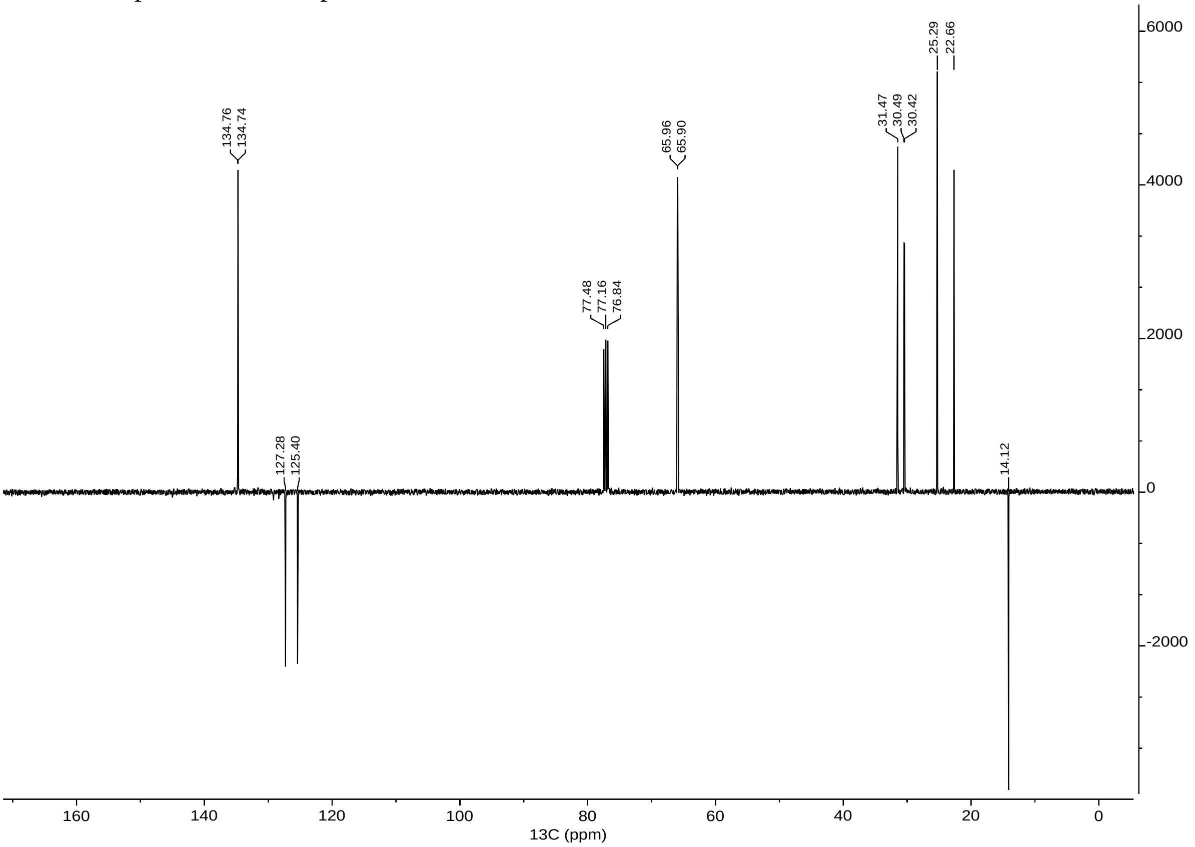

^{31}P{^{1}H} NMR spectrum of compound **S23**

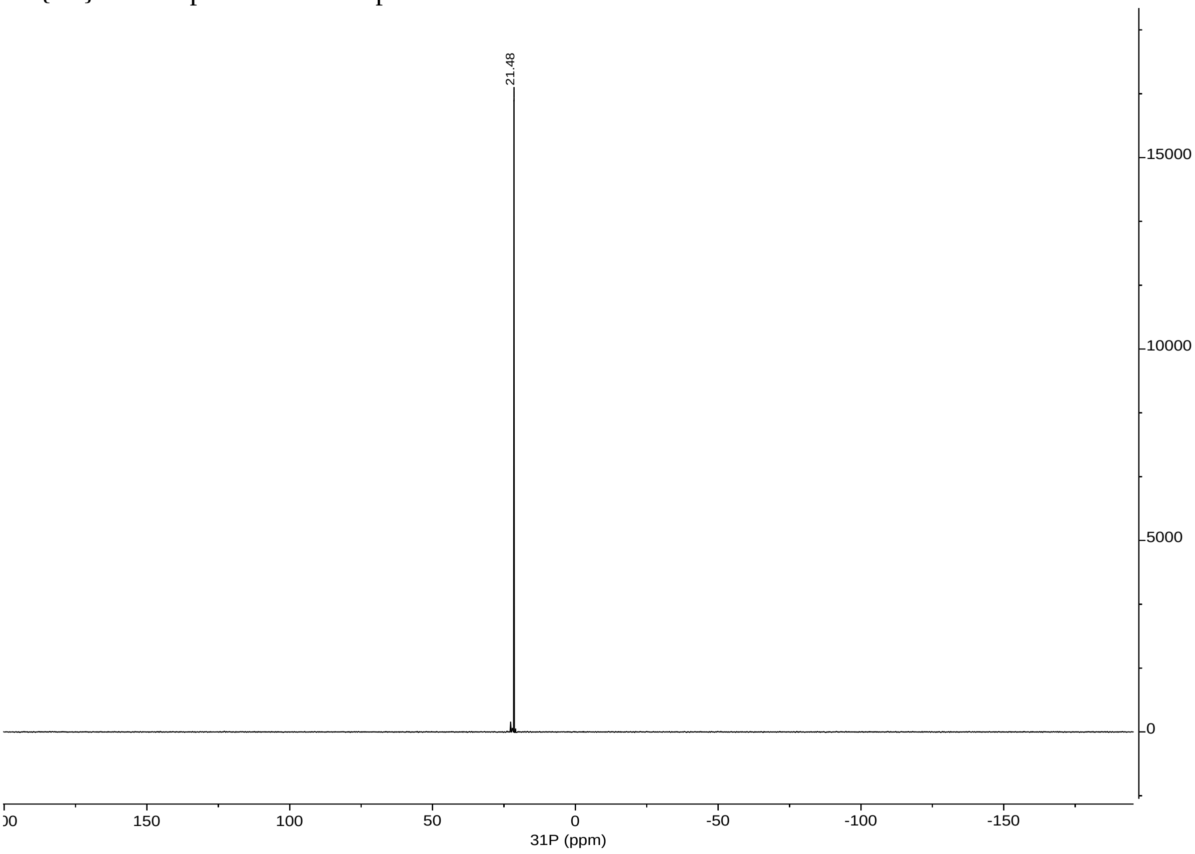

Mono cyclopentylmethyl vinylphosphonate (S24)

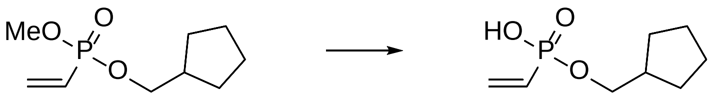

The title compound was prepared according to general method **A** from cyclopentylmethyl methyl vinylphosphonate **S7** (12.3 g, 60.2 mmol) in 77% yield (8.8 g, 46.3 mmol) as a colourless oil.

^{1}H NMR (401 MHz, $CDCl_3$) δ 9.17 (s, 1H, PO*H*), 6.30–5.89 (m, 3H, C*H*=C*H*$_2$), 3.82 (t, *J* = 6.7 Hz, 2H, OC*H*$_2$), 2.21 (p, *J* = 7.5 Hz, 1H, *H*-1-cyclopent), 1.79–1.67 (m, 2H, *H*-2a,5a-cyclopent), 1.65–1.47 (m, 4H, *H*-3,4-cyclopent), 1.32–1.20 (m, 2H, *H*-2b,5b-cyclopent).

^{13}C NMR (101 MHz, $CDCl_3$) δ 133.84 (=*C*H$_2$), 127.31 (d, *J* = 187.2 Hz, P*C*H), 69.31 (d, *J* = 5.9 Hz, O*C*H$_2$), 40.08 (d, *J* = 6.7 Hz, OCH$_2$*C*H), 29.15 (*C*H$_2$-2,5-cyclopent), 25.50 (*C*H$_2$-3,4-cyclopent).

^{31}P{^{1}H} NMR (162 MHz, $CDCl_3$) δ 17.65.

IR ν_{max} ($CHCl_3$) 3086 (vw), 2957 (s), 2870 (m), 2757 (w, vbr), 2337 (w, vbr), ~1655 (m, vbr), 1615 (m), 1467 (m), 1453 (m), 1402 (m), 1277 (m), 1200 (s), 1033 (vs), 1009 (vs), 987 (vs), ~977 (vs, sh), 900 (m).

HR-MS(CI$^+$): For $C_8H_{16}O_3P$ (M+H)$^+$ *m*/*z* calculated 191.0837, found 191.0836.

^{1}H NMR spectrum of compound **S24**

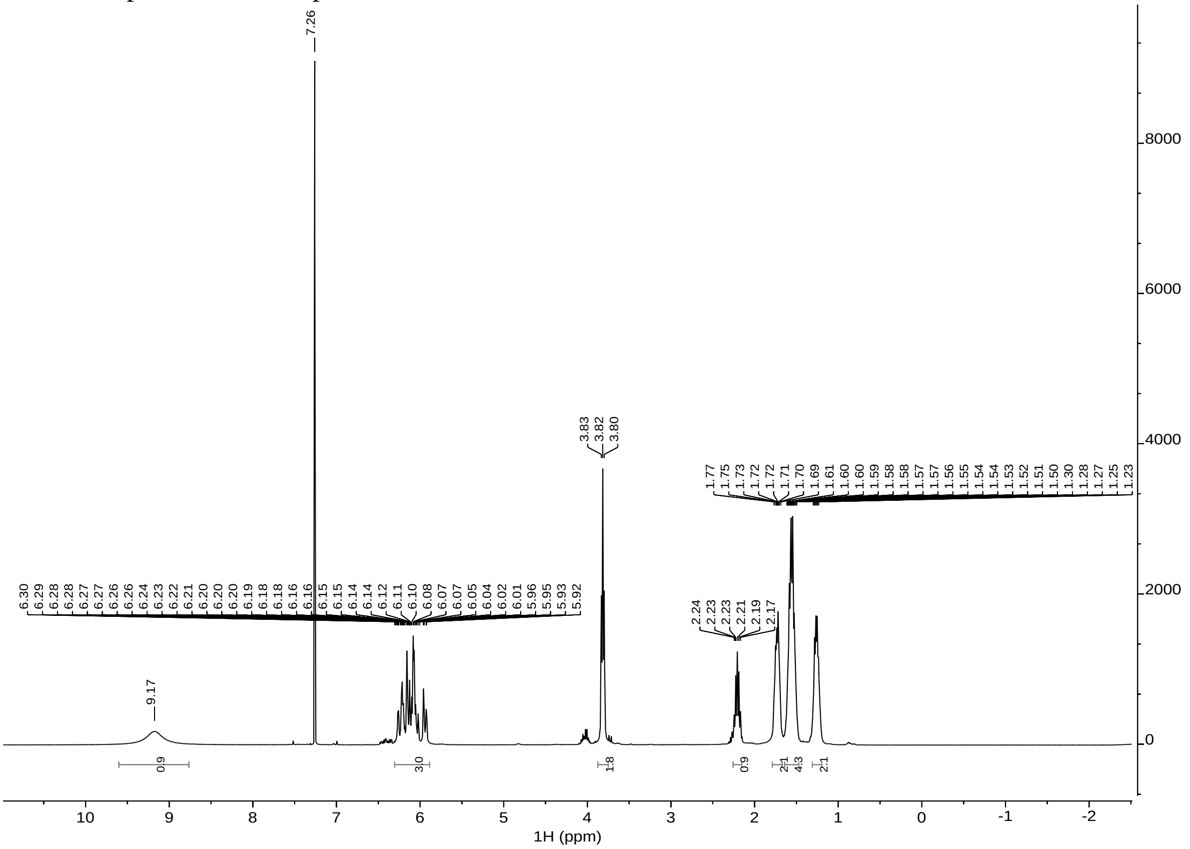

^{13}C NMR spectrum of compound **S24**

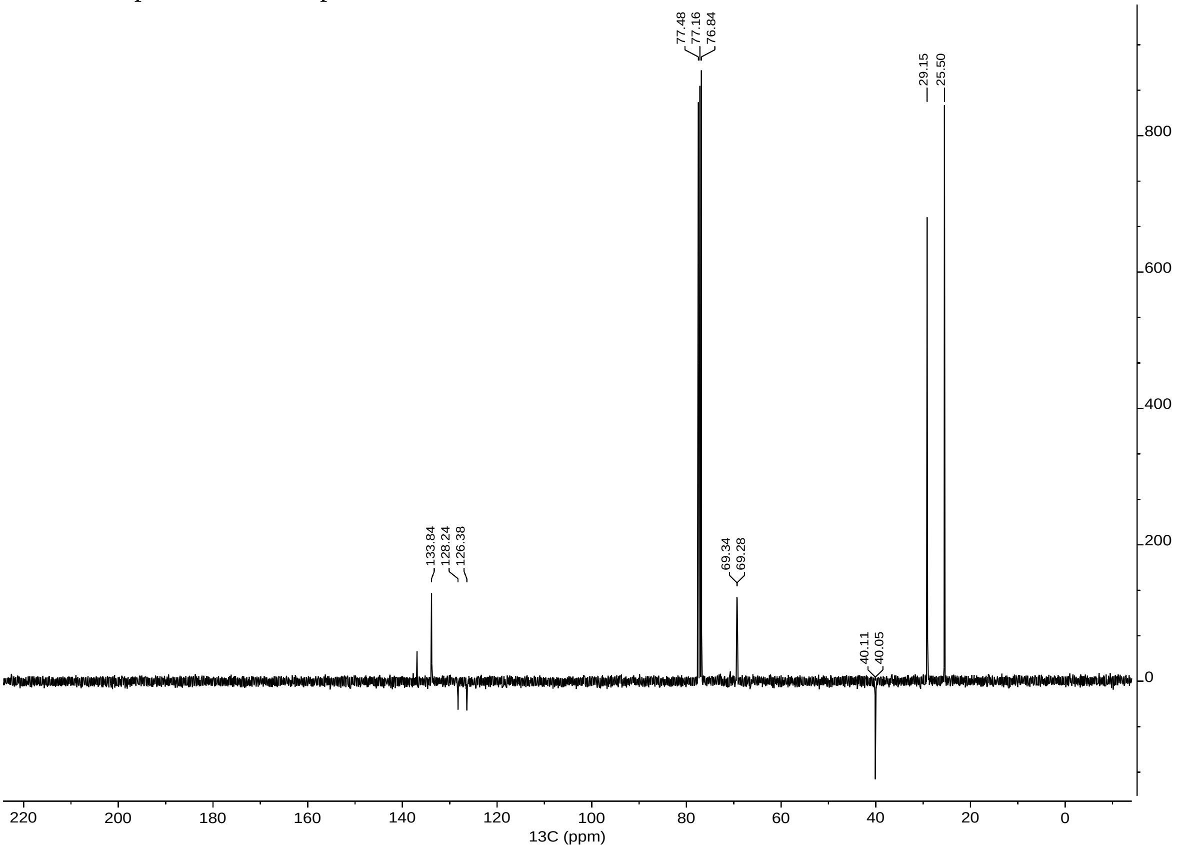

^{31}P{^{1}H} NMR spectrum of compound **S24**

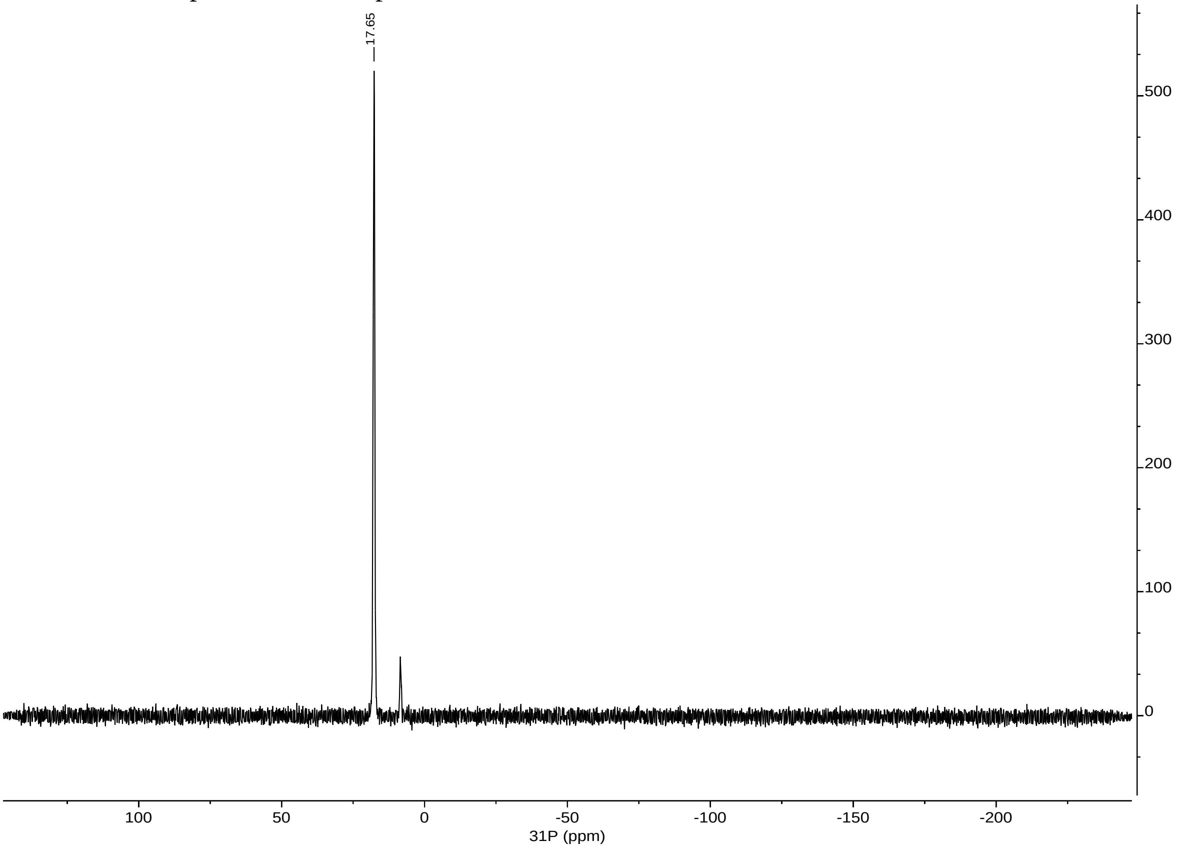

Mono heptyl vinylphosphonate (S25)

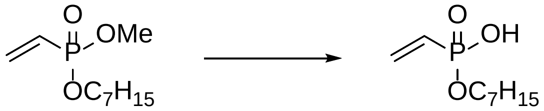

The title compound was prepared according to general method **A** from heptyl methyl vinylphosphonate **S8** (14.5 g, 65.7 mmol) in 85% yield (11.5 g, 55.5 mmol) as a colourless oil.

^{1}H NMR (401 MHz, $CDCl_3$) δ 10.83 (s, 1H, O*H*), 6.34–5.98 (m, 3H, C*H*=C*H*$_2$), 3.99 (q, *J* = 6.7 Hz, 2H, OC*H*$_2$), 1.66 (p, *J* = 6.6 Hz, 2H, OCH_2CH_2), 1.41–1.19 (m, 8H, $(CH_2)_4CH_3$), 0.92–0.83 (m, 3H, C*H*$_3$).

^{13}C NMR (101 MHz, $CDCl_3$) δ 134.92 (=CH_2), 126.20 (d, *J* = 189.2 Hz, P*C*H), 66.02 (d, *J* = 6.1 Hz, O*C*H$_2$), 31.84 ($CH_2CH_2CH_3$), 30.48 (d, *J* = 7.0 Hz, OCH_2CH_2), 28.96, 25.57 ($O(CH_2)_2(CH_2)_2$), 22.71 (CH_2CH_3), 14.19 (CH_3).

^{31}P{^{1}H} NMR (162 MHz, $CDCl_3$) δ 21.89.

IR ν_{max}(film) 3086 (w), 2957 (m), 2930 (s), 2872 (m), 2858 (m), 2594 (w, br), 2280 (w), 1615 (m), 1468 (m), 1457 (m), 1402 (m), 1276 (m), 1208 (s), 1064 (s), 1053 (s), 1013 (vs), 988 (vs), 850 (m), 727 (m), 607 (m).

HR-MS(ESI$^-$): For $C_9H_{18}O_3P$ $(M-H)^-$ *m*/*z* calculated 205.09990, found 205.10001.

^{1}H NMR spectrum of compound **S25**

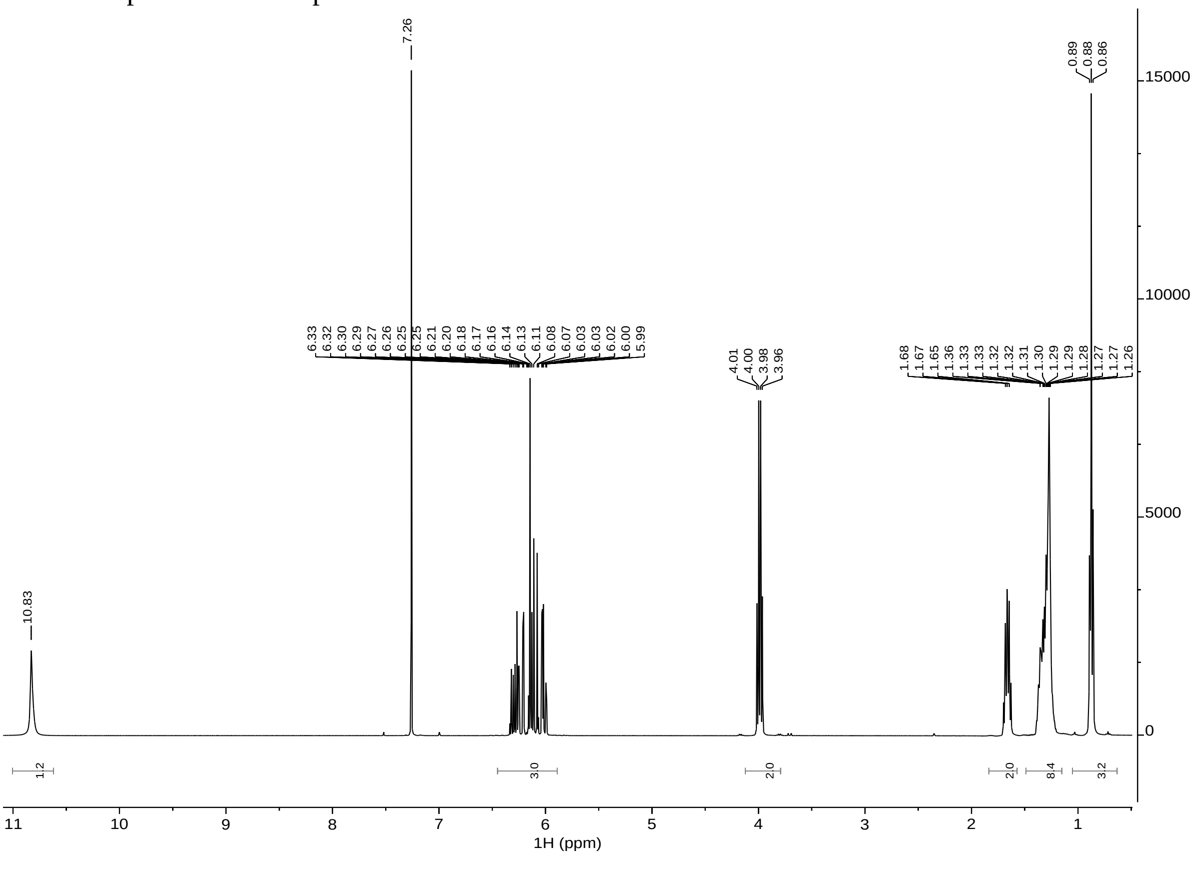

^{13}C NMR spectrum of compound **S25**

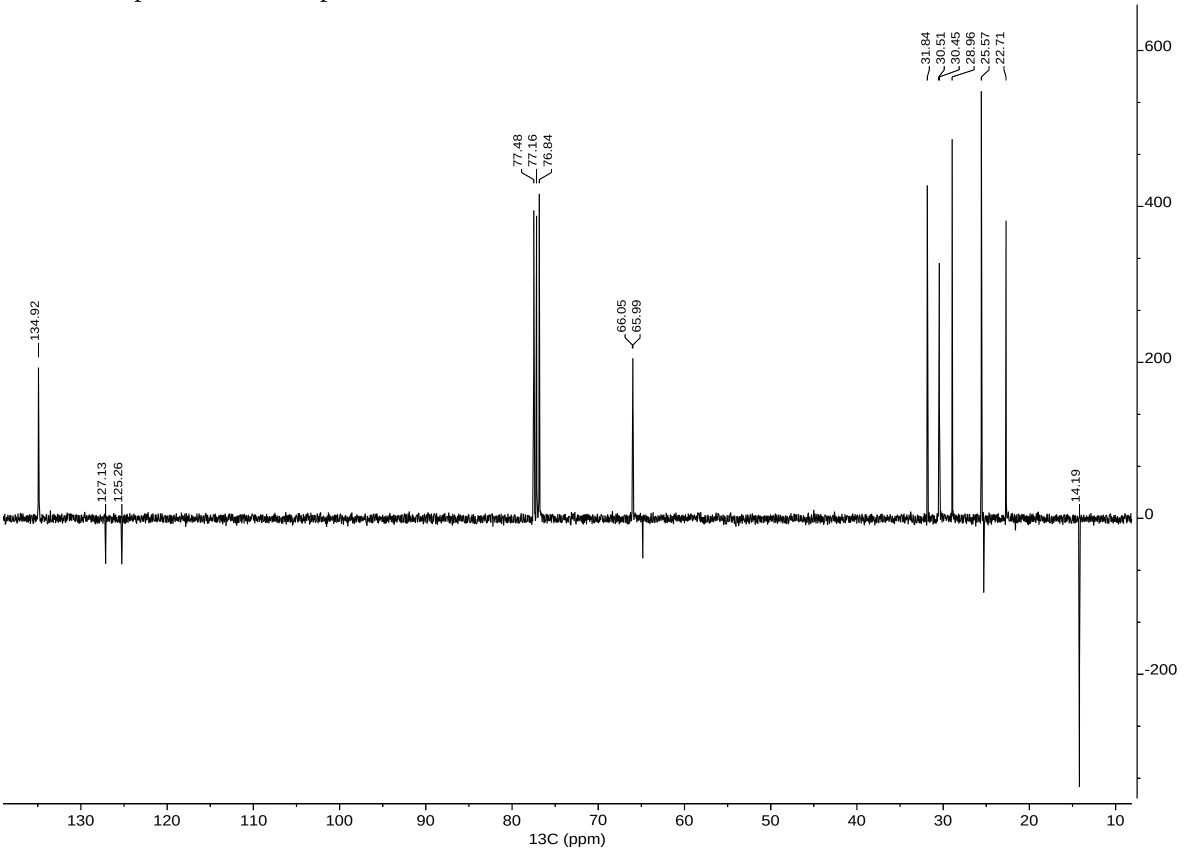

^{31}P{^{1}H} NMR spectrum of compound **S25**

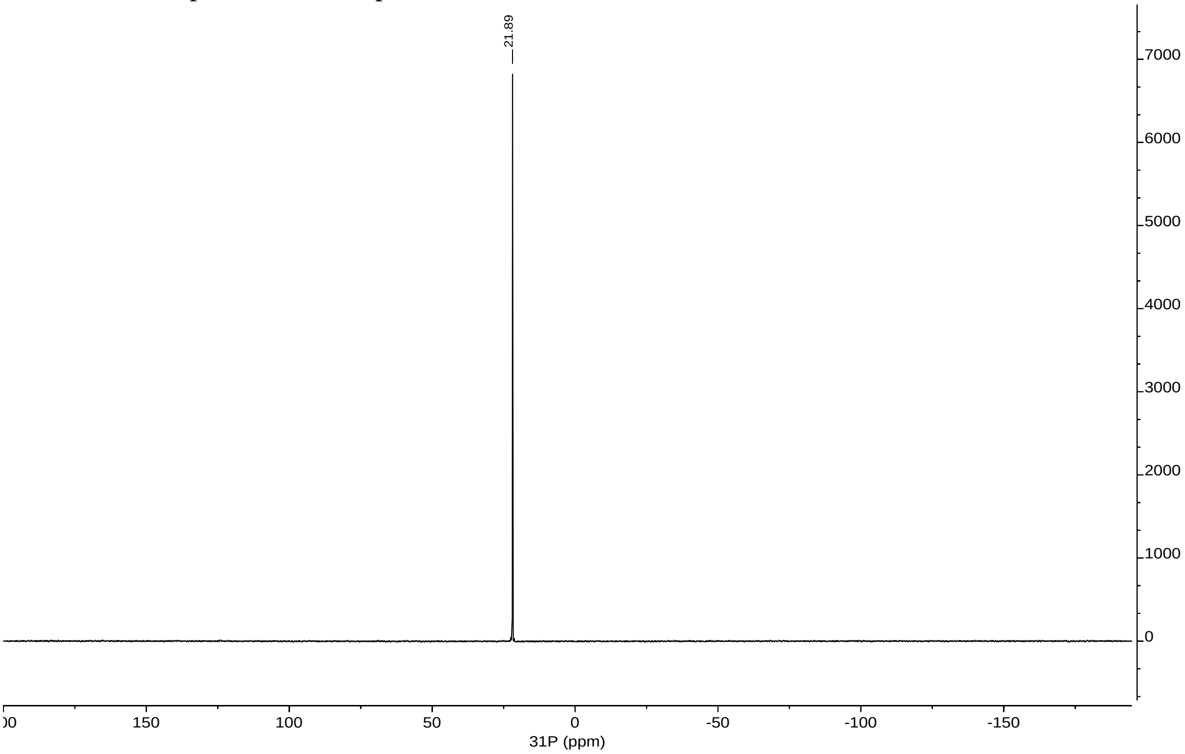

Mono (*Z*)-hept-3-en-1-yl vinylphosphonate (S26)

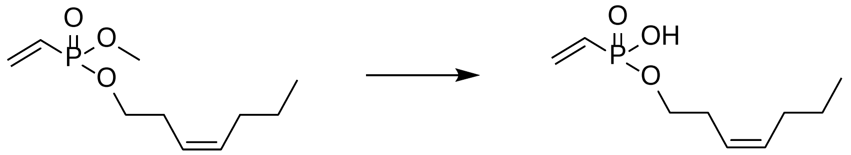

The title compound was prepared according to general method **A** from (*Z*)-hept-3-en-1-yl methyl vinylphosphonate **S6** (4.50 g, 20.6 mmol) in 80% yield (3.38 g, 16.5 mmol) as a colourless oil.

^{1}H NMR (400 MHz, $CDCl_3$): 11.10 (s, 1H, PO*H*), 6.34–5.99 (m, 3H, $C\mathit{H}{=}C\mathit{H}_2$), 5.54–5.47 (m, 1H, $CH_3(CH_2)_2C\mathit{H}CHCH_2CH_2O$), 5.38–5.31 (m, 1H, $CH_3(CH_2)_2CHC\mathit{H}CH_2CH_2O$), 3.98 (q, 2H, *J* = 7.2 Hz, $CH_3CH_2CH_2CHCHCH_2C\mathit{H}_2O$), 2.46–2.35 (m, 2H, $CH_3(H_2CH_2CHCHC\mathit{H}_2CH_2O$), 2.04–1.98 (m, 2H, $CH_3CH_2C\mathit{H}_2CHCHCH_2CH_2O$), 1.41–1.32 (m, 2H, $CH_3C\mathit{H}_2CH_2CHCHCH_2CH_2O$), 0.89 (t, *J* = 7.3 Hz, 3H, $C\mathit{H}_3(CH_2)_2CHCHCH_2CH_2O$).

^{13}C NMR (101 MHz, $CDCl_3$): 134.86 (d, *J* = 1.9 Hz, $CH{=}\mathit{C}H_2$), 133.18 ($CH_3(CH_2)_2\mathit{C}HCHCH_2CH_2O$), 126.27 (d, *J* = 190.2 Hz, $\mathit{C}H{=}CH_2$), 123.94 ($CH_3(CH_2)_3CH\mathit{C}HCH_2CH_2O$), 65.20 (d, *J* = 6.1 Hz, $CH_3(CH_2)_2CHCHCH_2\mathit{C}H_2O$), 29.50 $CH_3CH_2\mathit{C}H_2CHCHCH_2CH_2O$), 28.72 (d, *J* = 6.7 Hz, $CH_3(CH_2)_2CHCH\mathit{C}H_2CH_2O$), 22.80 ($CH_3\mathit{C}H_2CH_2CHCHCH_2CH_2O$), 13.86 ($\mathit{C}H_3(CH_2)_2CHCHCH_2CH_2O$).

^{1}P{^{1}H} NMR (162 MHz, $CDCl_3$): 21.58.

IR ν_{max} (KBr) 3091 (w), 3013 (m), 2960 (m), 2931 (m), 2873 (s), 2700–2100 (w, vbr), 1665 (w), 1657 (w), 1615 (w), 1466 (w), 1458 (w), 1403 (m), 1379 (vw), 1277 (w), 1210 (m), 1144 (m, sh), 1066 (m), 1016 (vs), 1023 (vs), 989 (vs), 849 (w).

HR-MS(ESI$^-$): For $C_9H_{16}O_3P$ $(M{-}H)^-$ *m/z* calculated 203.08425, found 203.08435.

^{1}H NMR spectrum of compound **S26**

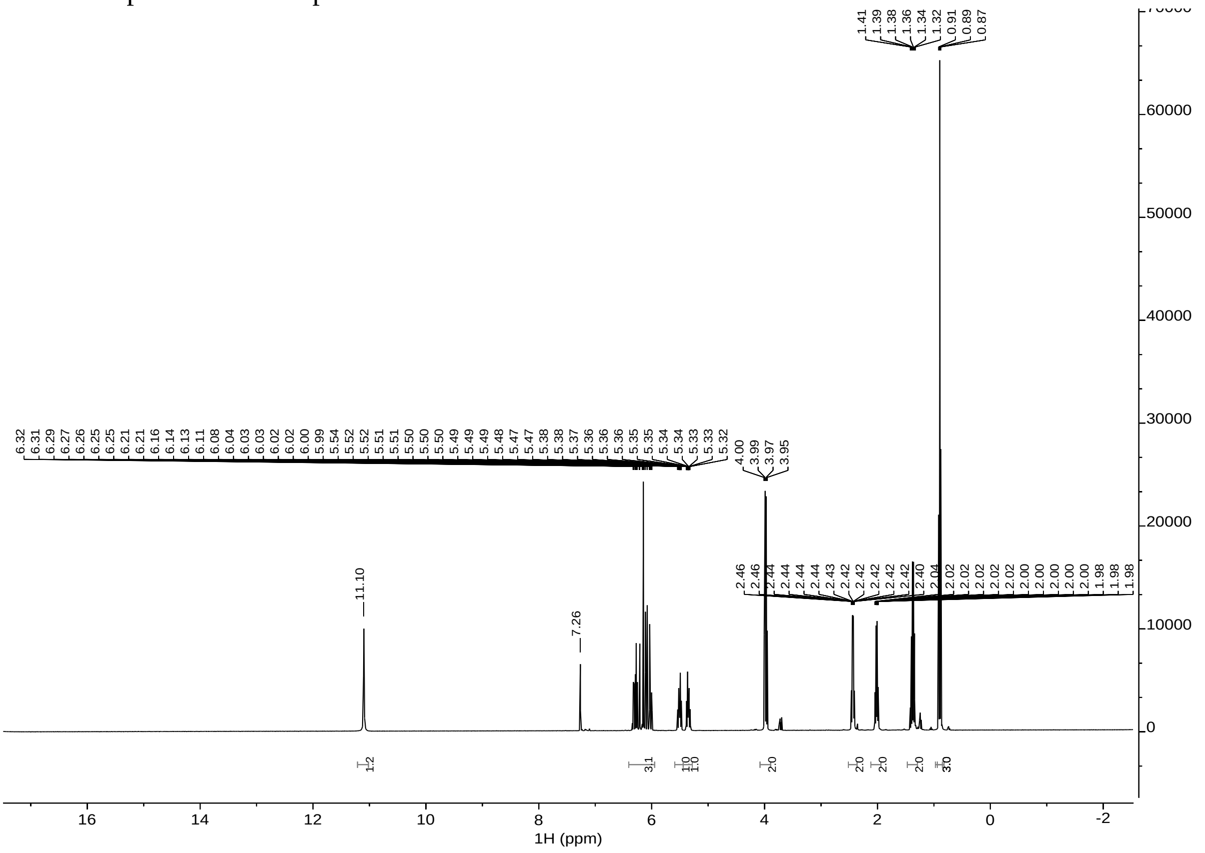

^{13}C NMR spectrum of compound **S26**

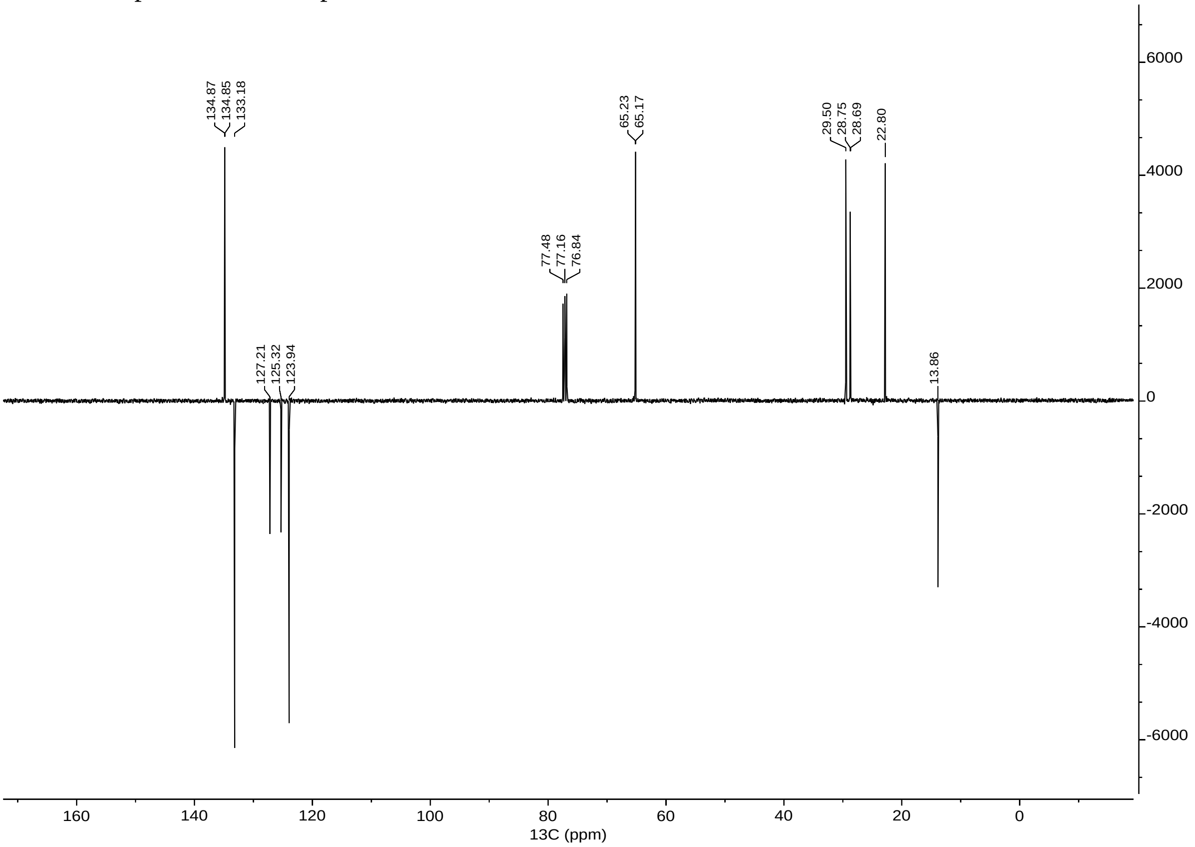

^{31}P{^{1}H} NMR spectrum of compound **S26**

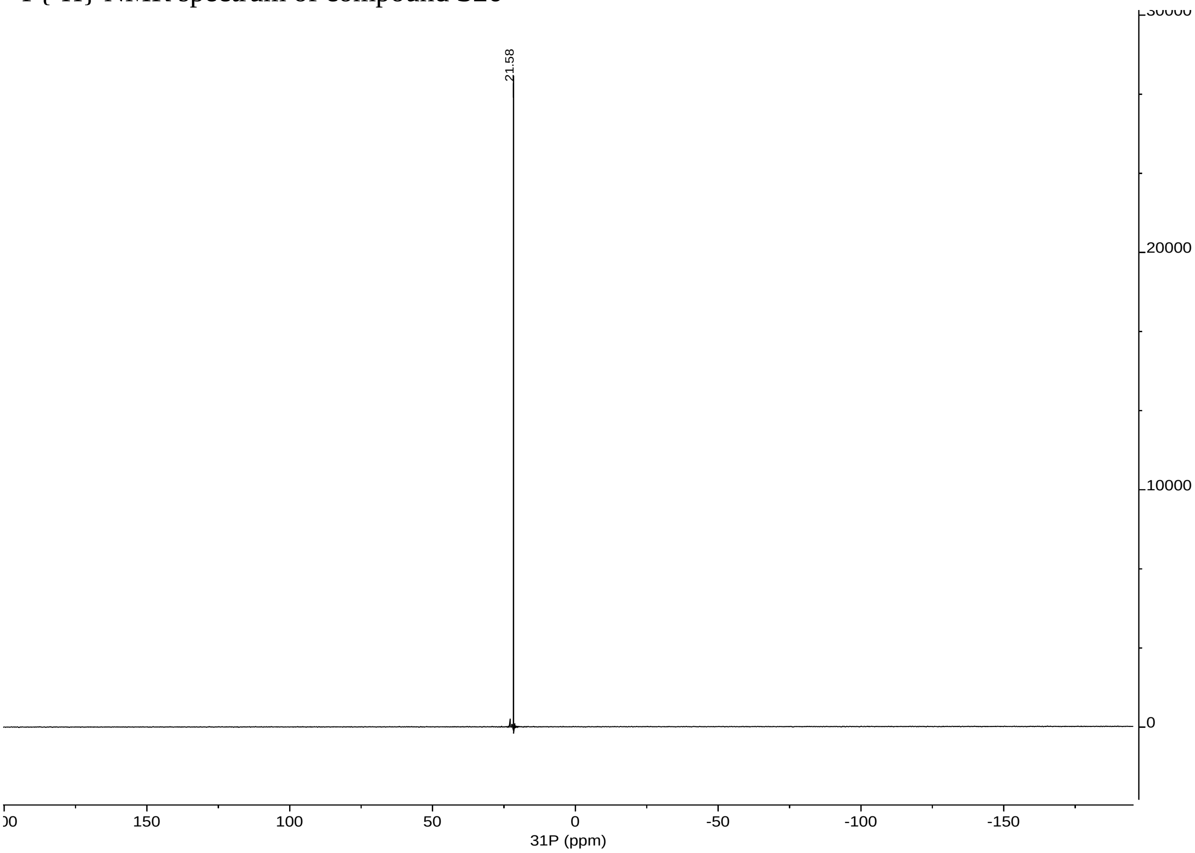

Mono (*Z*)-hept-4-en-1-yl vinylphosphonate (S27)

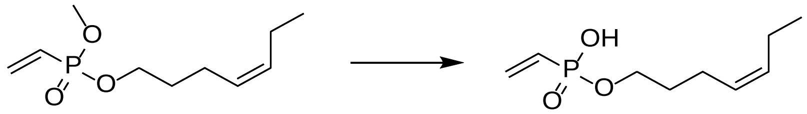

The title compound was prepared according to general method **A** from (*Z*)-hept-4-en-1-yl methyl vinylphosphonate **S9** (2.29 g, 10.49 mmol) in 95% yield (2.04 g, 10.0 mmol) as a colourless oil.

^{1}H NMR (401 MHz, $CDCl_3$): 8.85 (s, 1H, PO*H*), 6.34–6.00 (m, 3H, $C\mathit{H}{=}C\mathit{H}_2$), 5.44–5.37 (m, 1H, $CH_3CH_2C\mathit{H}CHCH_2CH_2CH_2O$), 5.32–5.25 (m, 1H, $CH_3CH_2CHC\mathit{H}CH_2CH_2CH_2O$), 4.03–3.98 (m, 2H, $CH_3CH_2CHCHCH_2CH_2C\mathit{H}_2O$), 2.15–2.09 (m, 2H, $CH_3CH_2CHCHC\mathit{H}_2CH_2CH_2O$), 2.07–1.99 (m, 2H, $CH_3C\mathit{H}_2CHCHCH_2CH_2CH_2O$), 1.76–1.69 (m, 2H, $CH_3CH_2CHCHCH_2C\mathit{H}_2CH_2O$), 0.97–0.93 (m, 3H, $C\mathit{H}_3CH_2CHCHCH_2CH_2CH_2O$).

^{13}C NMR (101 MHz, $CDCl_3$): 134.87 (d, J = 1.9 Hz, $CH{=}\mathit{C}H_2$), 133.03 ($CH_3CH_2\mathit{C}HCHCH_2CH_2CH_2O$), 127.51 ($CH_3CH_2CH\mathit{C}HCH_2CH_2CH_2O$), 126.26 (d, J = 189.9 Hz, $\mathit{C}H{=}CH_2$), 65.28 (d, J = 6.1 Hz, $CH_3CH_2CHCHCH_2CH_2\mathit{C}H_2O$), 30.50 (d, J = 6.8 Hz, $CH_3CH_2CHCHCH_2\mathit{C}H_2CH_2O$), 23.19 ($CH_3CH_2CHCH\mathit{C}H_2CH_2CH_2O$), 20.63 ($CH_3\mathit{C}H_2CHCHCH_2CH_2CH_2O$), 14.43 ($\mathit{C}H_3CH_2CHCHCH_2CH_2CH_2O$).

^{31}P{^{1}H} NMR (162 MHz, $CDCl_3$): 22.17.

IR ν_{max} (KBr) 3090 (w), 2965 (s), 2935 (m), 2875 (m), 2670 (w), 2339 (m), 1700 (m, br), 1654(m), 1615 (m), 1464 (m), 1456 (m), 1403 (m), 1375 (w), 1277 (m), 1198 (s), 1069 (m), 1038 (s), 1024 (s), 986 (vs), 862 (m).

HR-MS(ESI^{+}): For $C_9H_{17}O_3NaP$ $(M+Na)^{+}$ *m/z* calculated 227.08075, found 227.08081.

^{1}H NMR spectrum of compound **S27**

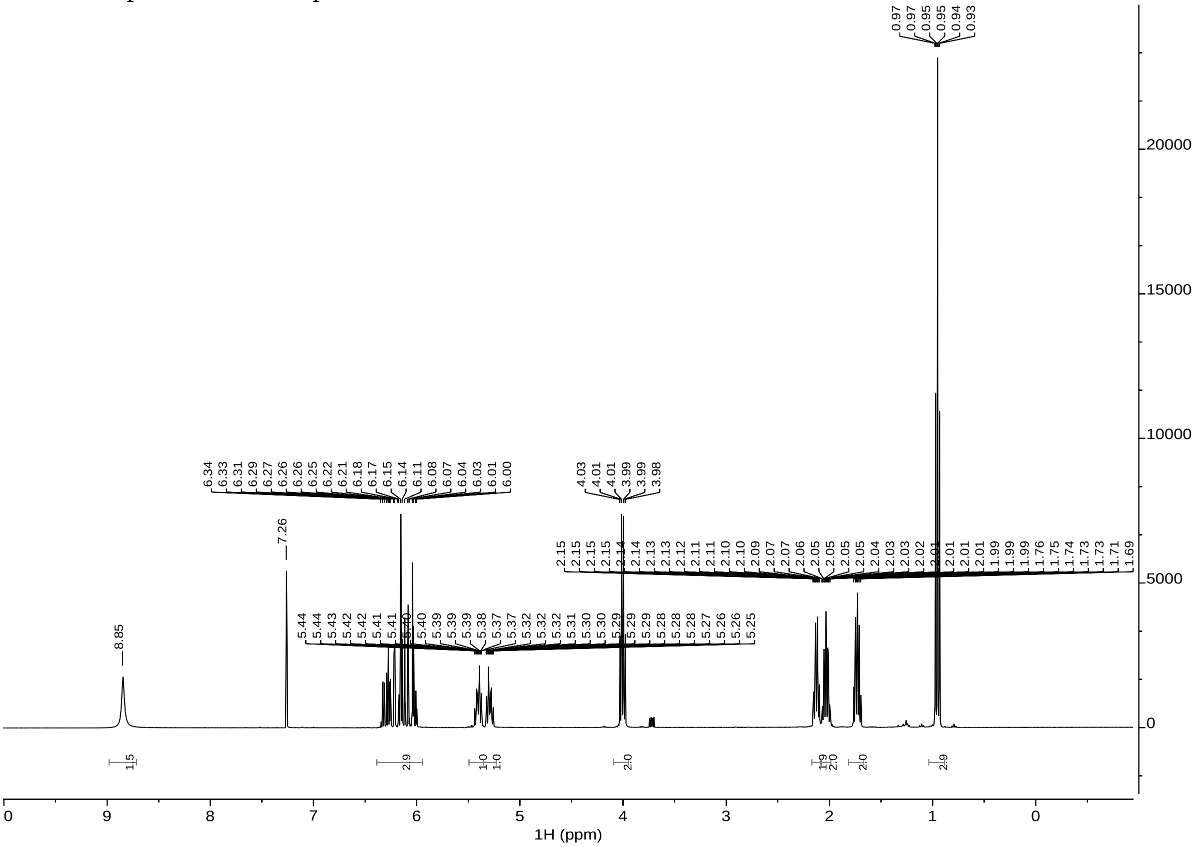

^{13}C NMR spectrum of compound **S27**

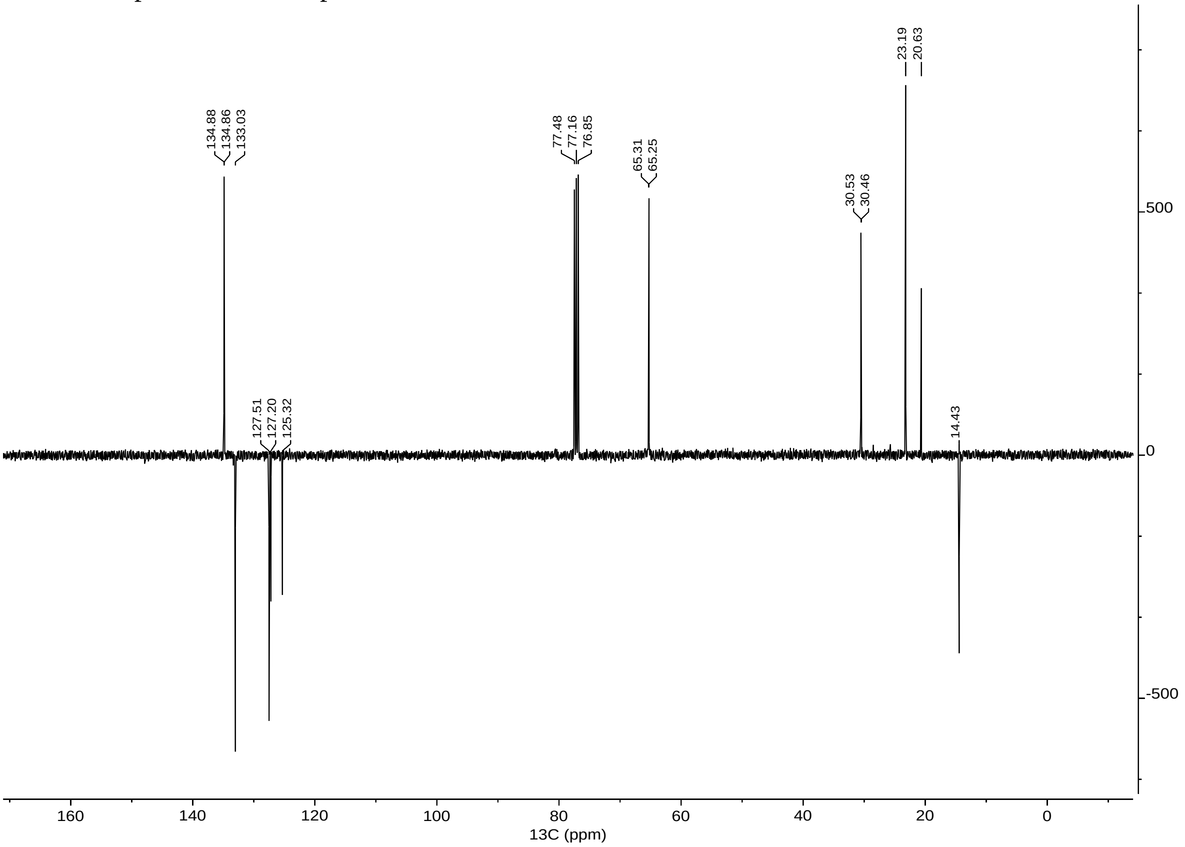

$^{31}P\{^{1}H\}$ NMR spectrum of compound **S27**

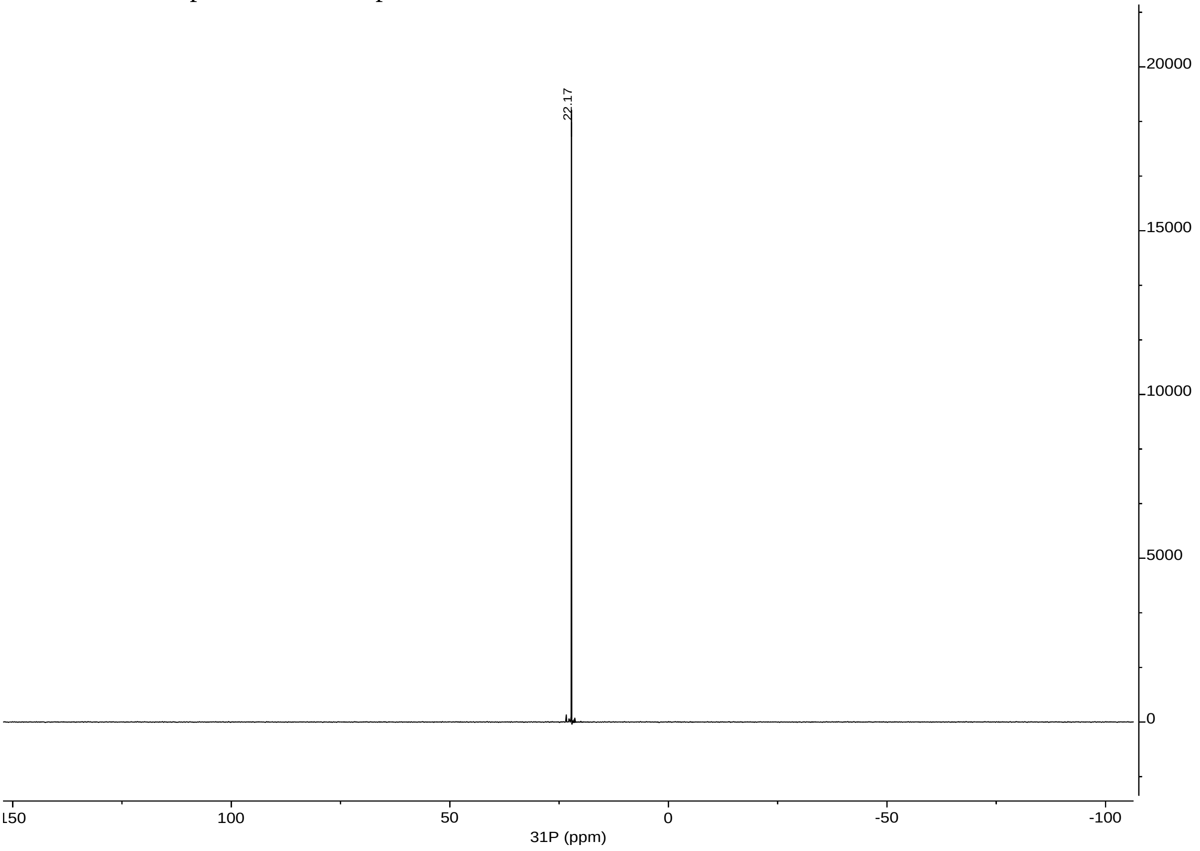

Mono octyl vinylphosphonate (S28)

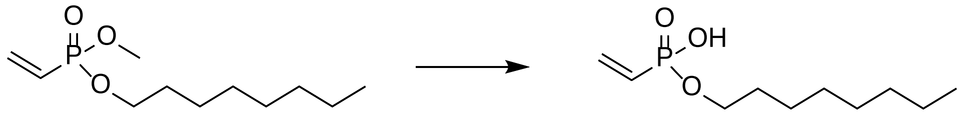

The title compound was prepared according to general method **A** from methyl octyl vinylphosphonate **S10** (6.10 g, 26.0 mmol) in 81% yield (4.64 g, 21.06 mmol) as a colourless oil.

^{1}H NMR (400 MHz, $CDCl_3$): 10.06 (s, 1H, PO*H*), 6.34–5.99 (m, 3H, C*H*=C*H*$_2$), 4.02–3.97 (m, 2H, $CH_3(CH_2)_3CH_2C$*H*$_2$O), 1.70–1.63 (m, 2H, $CH_3(CH_2)_5C$*H*$_2CH_2$O), 1.37–1.25 (m, 10H, $CH_3(C$*H*$_2)_5CH_2CH_2$O), 0.89–0.86 (m, 3H, C*H*$_3(CH_2)_6CH_2$O).

^{13}C NMR (101 MHz, $CDCl_3$): 134.76 (d, J = 1.8 Hz, CH=*C*H$_2$), 126.35 (d, J = 189.6 Hz, *C*H=CH$_2$), 65.94 (d, J = 6.1 Hz, $CH_3(CH_2)_5CH_2$*C*H$_2$O), 30.51 (d, J = 6.7 Hz, $CH_3(CH_2)_5$*C*H$_2CH_2$O), 31.92, 29.32, 29.27, 25.64, 22.78 (CH_3(*C*H$_2$)$_5CH_2CH_2$O), 14.22 (*C*H$_3(CH_2)_6CH_2$O).

^{31}P{^{1}H} NMR (162 MHz, $CDCl_3$): 21.79.

IR ν_{max} (KBr) 3089 (vw), 2958 (s), 2929 (s), 2872 (s), 2857 (s), 2672 (w, vbr), 2337 (m,vbr), 2183 (w, vbr), 1700 (w, vbr), 1615 (w), 1468 (m), 1458 (w), 1379 (w), 1277 (w), 1197 (s), 1070 (m, sh), 1025 (s), 986 (vs), 925 (w), 854 (w).

HR-MS(ESI$^+$): For $C_{10}H_{21}O_3NaP$ (M+Na)$^+$ m/z calculated 243.11205, found 243.11187.

^{1}H NMR spectrum of compound **S28**

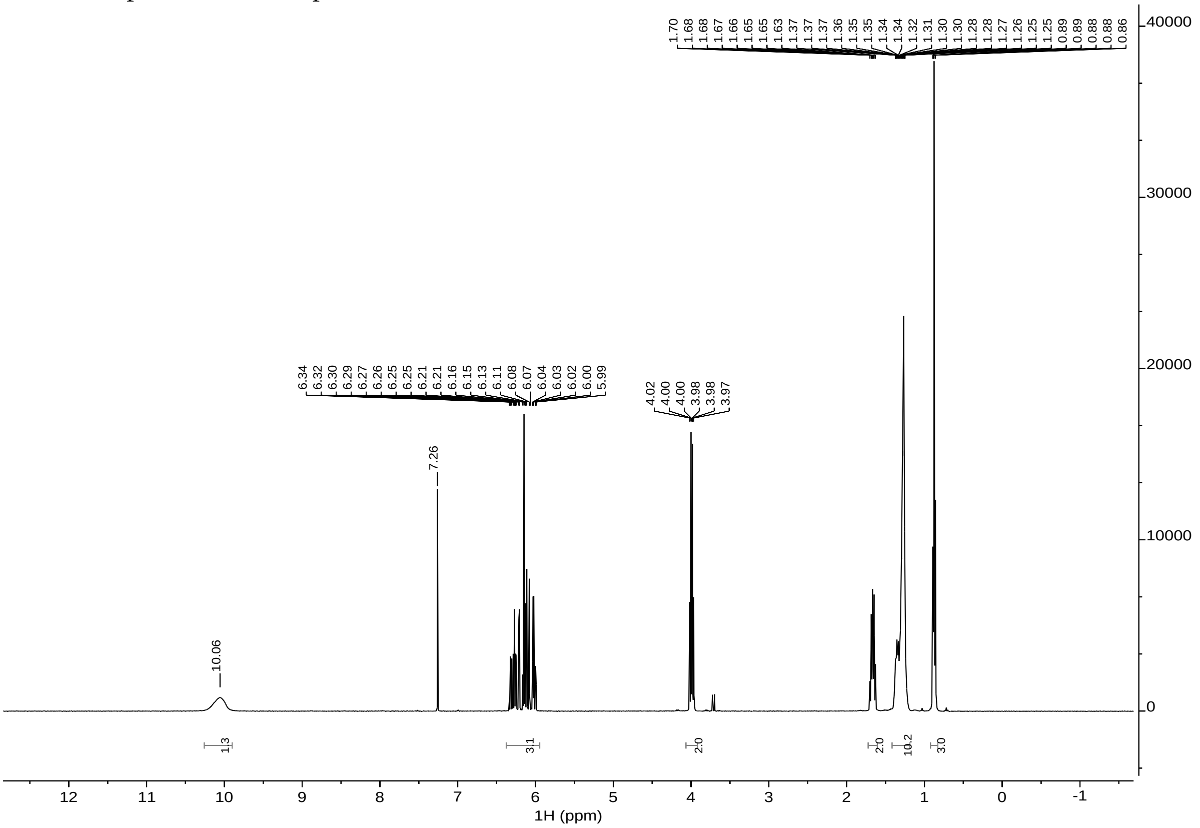

^{13}C NMR spectrum of compound **S28**

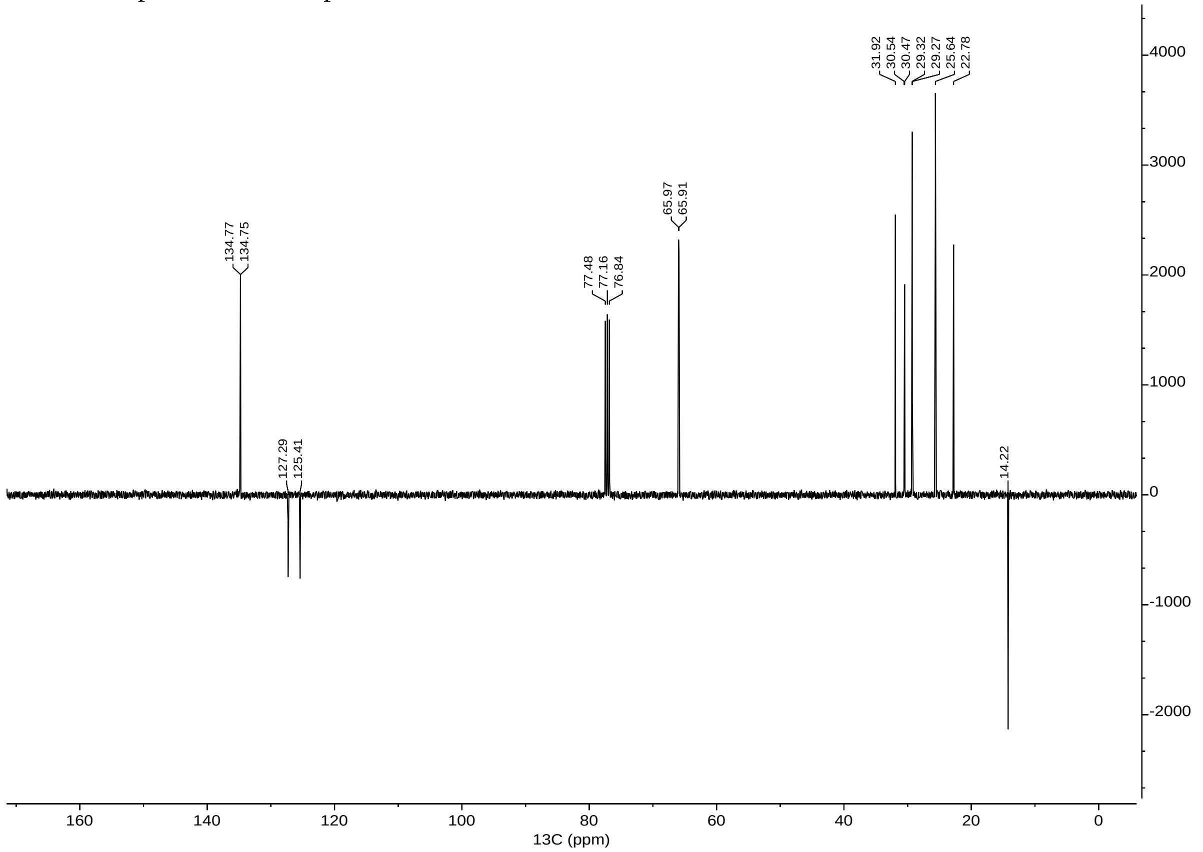

^{31}P{^{1}H} NMR spectrum of compound **S28**

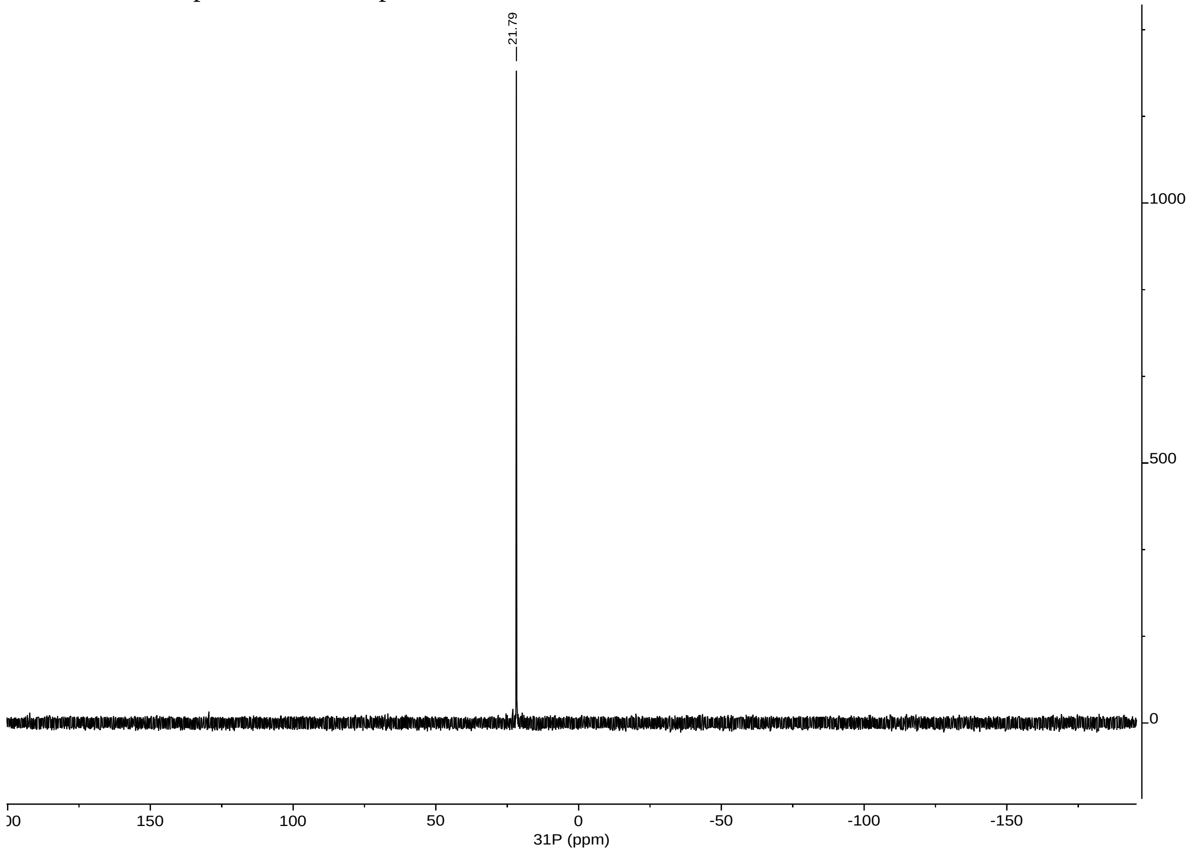

Mono 3-cyclohexylpropyl vinylphosphonate (S29)

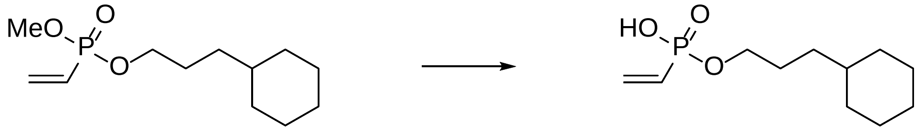

The title compound was prepared according to general method **A** from 3-cyclohexylmethyl methyl vinylphosphonate **S11** (3.46 g, 14.1 mmol) in 92% yield (3.01 g, 13.0 mmol) as a colourless oil.

^{1}H NMR (401 MHz, Chloroform-*d*) δ 9.98 (s, 1H, PO*H*), 6.35–5.99 (m, 3H, C*H*=C*H*$_2$), 3.98 (q, *J* = 6.9 Hz, 2H, OC*H*$_2$), 1.76–1.58 (m, 7H, 2a,3a,4a,5a,6a-Cy*H*, OCH$_2$C*H*$_2$), 1.28–1.06 (m, 6H, 1,3b,4b,5b-Cy*H*, O(CH$_2$)C*H*$_2$Cy), 0.95–0.79 (m, 2H, 2b,6b-Cy*H*).

^{13}C NMR (101 MHz, Chloroform-*d*) δ 134.81 (d, *J* = 2.0 Hz, =*C*H$_2$), 126.33 (d, *J* = 189.2 Hz, P*C*H), 66.29 (d, *J* = 6.2 Hz, O*C*H$_2$), 37.42 (1-*C*H$_{Cy}$), 33.40 (2,6-(*C*H$_2$)$_{Cy}$), 33.23 (*C*H$_2$Cy), 27.91 (d, *J* = 6.7 Hz, *C*H$_2$CH$_2$Cy), 26.76 (4-(*C*H$_2$)$_{Cy}$), 26.47 (3,5-(*C*H$_2$)$_{Cy}$).

^{31}P{^{1}H} NMR (162 MHz, Chloroform-*d*) δ 22.18.

IR ν_{max} (CHCl$_3$) 3090 (vw), 2926 (vs), 2853 (s), 2710 (w, vbr), 2330 (m, vbr), 1669 (m, vbr), 1615 (m), 1474 (w), 1463 (w), 1449 (m), 1403 (m), 1276 (w), 1198 (s), 1068 (s), 1053 (s), 1041 (s), 1013 (vs), 986 (vs).

HR-MS(CI$^+$/TOF): For $C_{11}H_{22}O_3P$ (M+H)$^+$ *m/z* calculated 233.1307, found 233.1310.

^{1}H NMR spectrum of compound **S29**

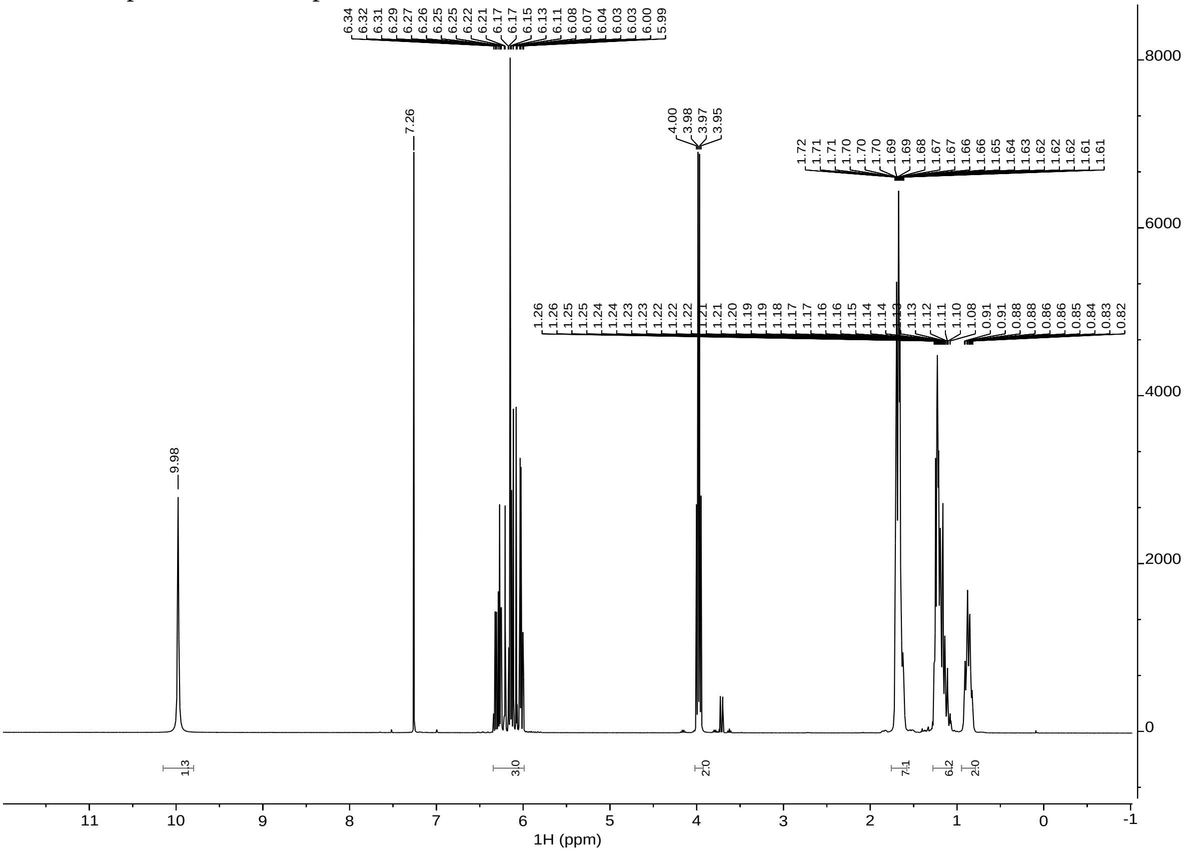

^{13}C NMR spectrum of compound **S29**

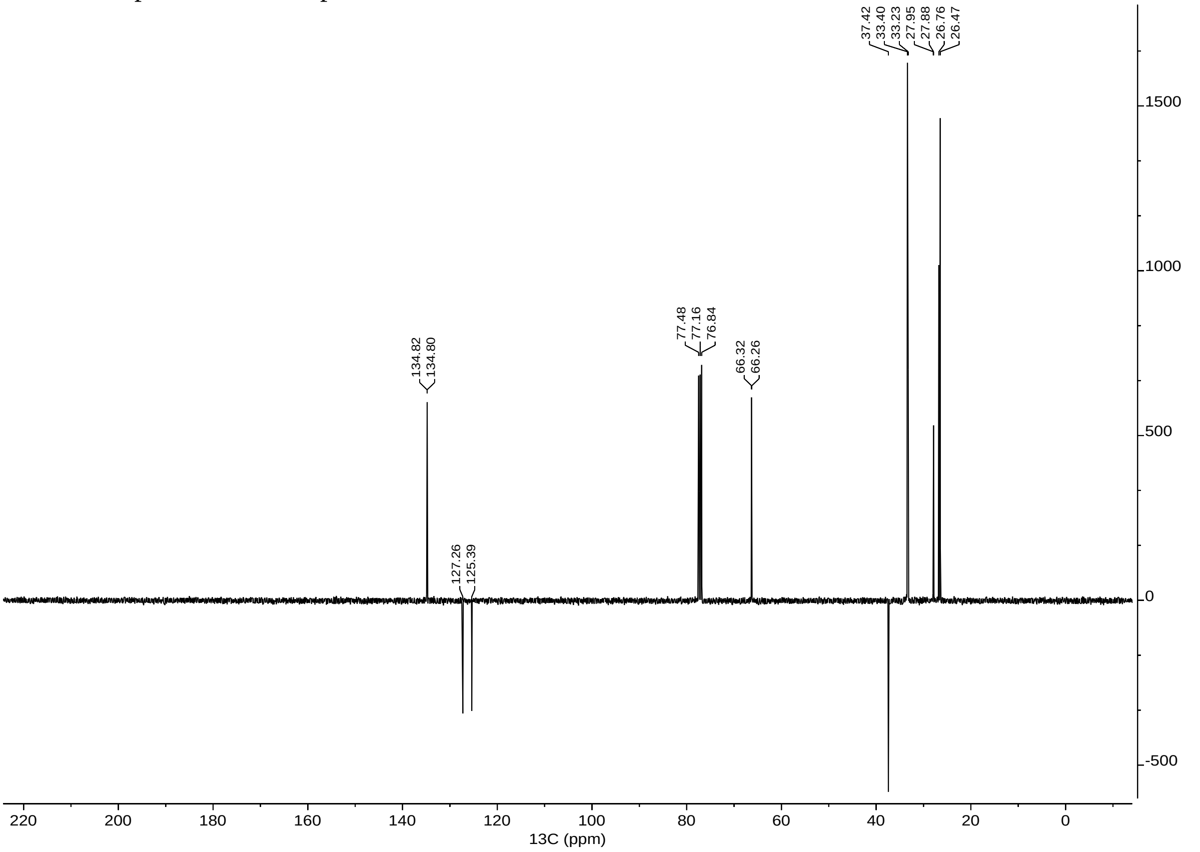

$^{31}P\{^{1}H\}$ NMR spectrum of compound **S29**

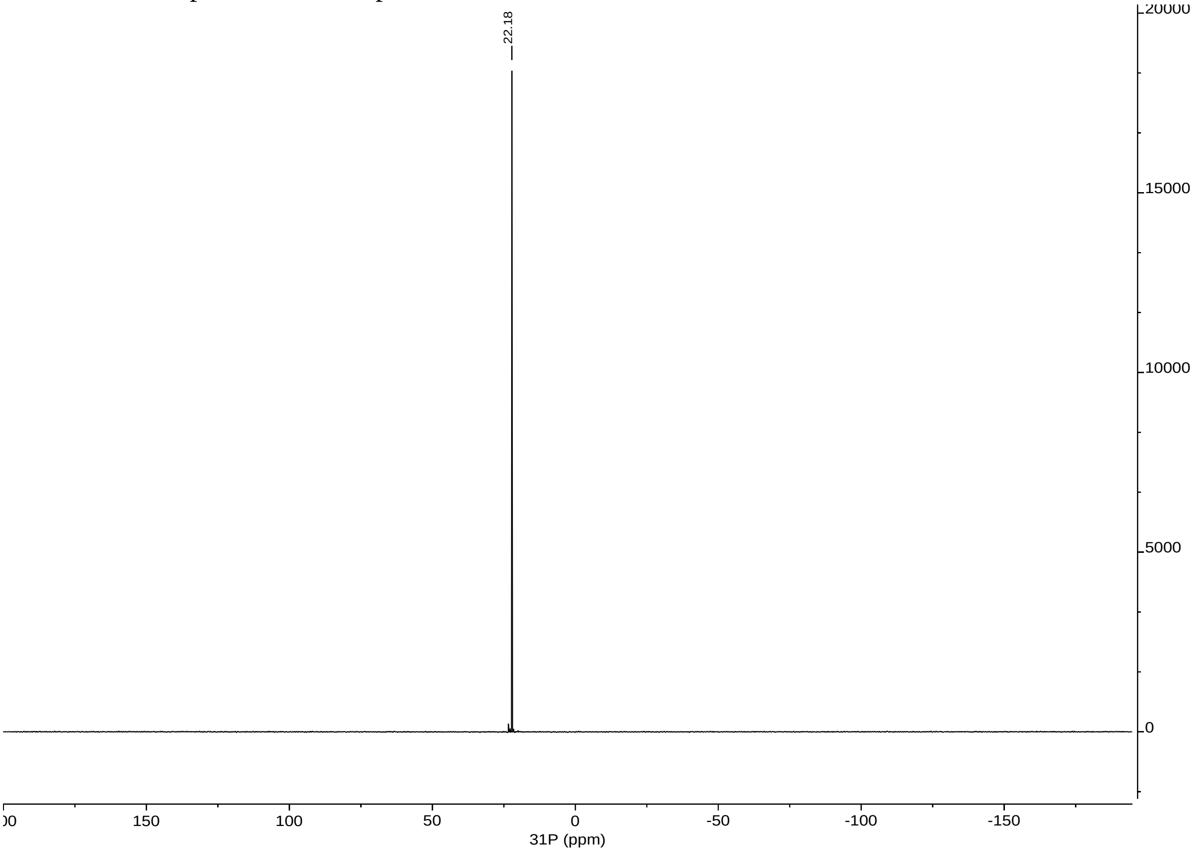

Mono (*Z*)-oct-3-en-1-yl vinylphosphonate (S30)

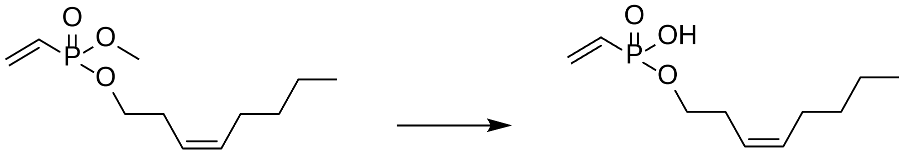

The title compound was prepared according to general method **A** from methyl (*Z*)-oct-3-en-1-yl vinylphosphonate **S12** (4.55 g, 19.6 mmol) in 80% yield (3.42 g, 15.7 mmol) as a colourless oil.

^{1}H NMR (400 MHz, $CDCl_3$): 10.79 (s, 1H, PO*H*), 6.34–5.99 (m, 3H, C*H*=C*H*$_2$), 5.5–5.47 (m, 1H, $CH_3(CH_2)_3C\mathit{H}CHCH_2CH_2O$), 5.37–5.30 (m, 1H, $CH_3(CH_2)_3CHC\mathit{H}CH_2CH_2O$), 3.98 (q, 2H, *J* = 7.2 Hz, $CH_3(CH_2)_2CH_2CHCHCH_2C\mathit{H_2}O$), 2.46–2.35 (m, 2H, $CH_3(CH_2)_2CH_2CHCHC\mathit{H_2}CH_2O$), 2.06–2.00 (m, 2H, $CH_3(CH_2)_2C\mathit{H_2}CHCHCH_2CH_2O$), 1.37–1.25 (m, 4H, $CH_3(C\mathit{H_2})_2CH_2CHCHCH_2CH_2O$), 0.92–0.86 (m, 3H, $C\mathit{H_3}(CH_2)_3CHCHCH_2CH_2O$).

^{13}C NMR (101 MHz, $CDCl_3$): 134.87 (d, *J* = 2.0 Hz, CH=*C*H$_2$), 133.41 ($CH_3(CH_2)_3\mathit{C}HCHCH_2CH_2O$), 126.26 (d, *J* = 189.8 Hz, *C*H=CH$_2$), 123.73 ($CH_3(CH_2)_3CH\mathit{C}HCH_2CH_2O$), 65.20 (d, *J* = 6.4 Hz, $CH_3(CH_2)_3CHCHCH_2\mathit{C}H_2O$), 31.88 ($CH_3CH_2\mathit{C}H_2CH_2CHCHCH_2CH_2O$), 28.71 (d, *J* = 6.7 Hz, $CH_3(CH_2)_3CHCH\mathit{C}H_2CH_2O$), 27.19 ($CH_3(CH_2)_2\mathit{C}H_2CHCHCH_2CH_2O$), 22.44 ($CH_3\mathit{C}H_2CH_2CH_2CHCHCH_2CH_2O$), 14.10 ($\mathit{C}H_3(CH_2)_3CHCHCH_2CH_2O$).

^{31}P{^{1}H} NMR (162 MHz, $CDCl_3$): 21.66.

IR ν_{max} (KBr) 3090 (w), 2960 (s), 2930 (s), 2873 (s), 2860 (s), 2734 (m, br), 2672 (m, vbr), 2330 (m, vbr), 2169 (m, vbr), 1665 (m, vbr), 1654 (m), 1615 (m), 1467 (m), 1458 (m), 1403 (s), 1379 (m), 1277 (m), 1198 (s), 1066 (s), 1053 (s), 1023 (vs), 986 (vs), 894 (w).

HR-MS(ESI^{+}): For $C_{10}H_{19}O_3NaP$ $(M+Na)^{+}$ *m/z* calculated 241.09640, found 241.09653.

^{1}H NMR spectrum of compound **S30**

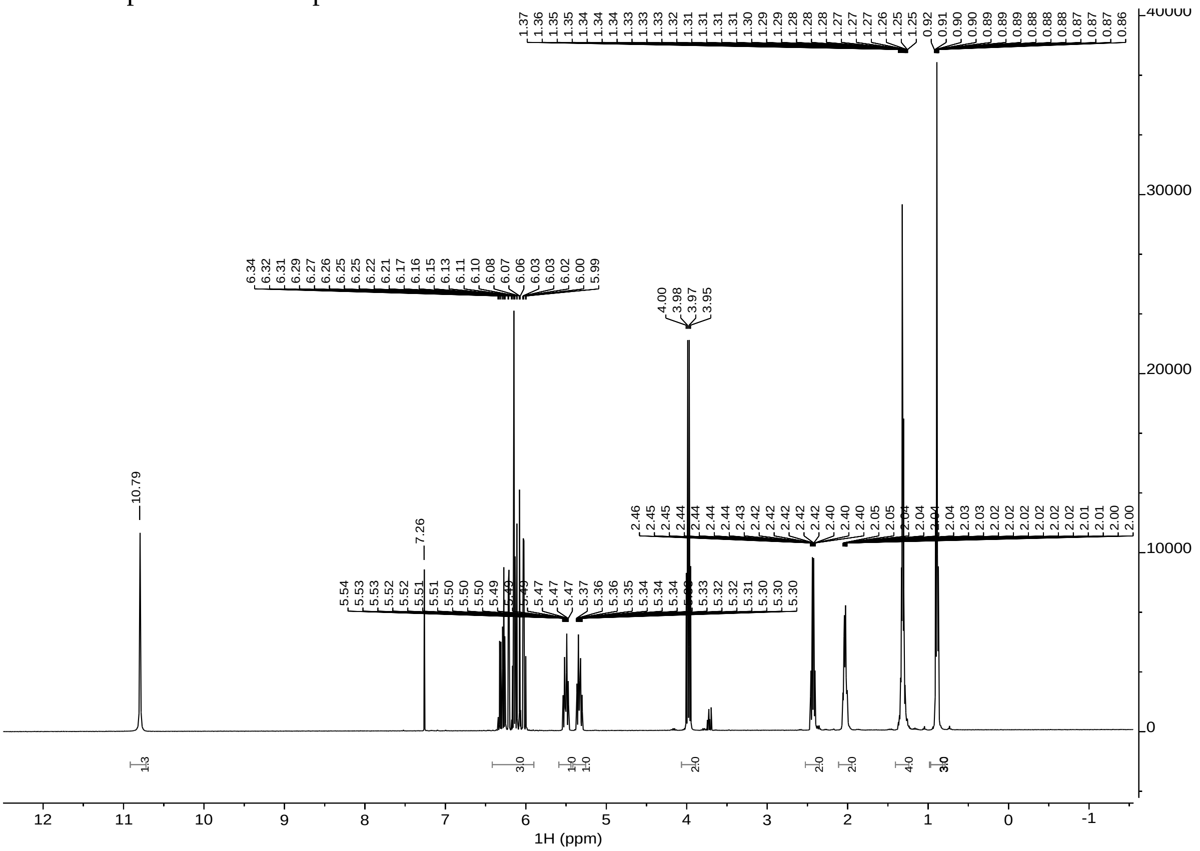

^{13}C NMR spectrum of compound **S30**

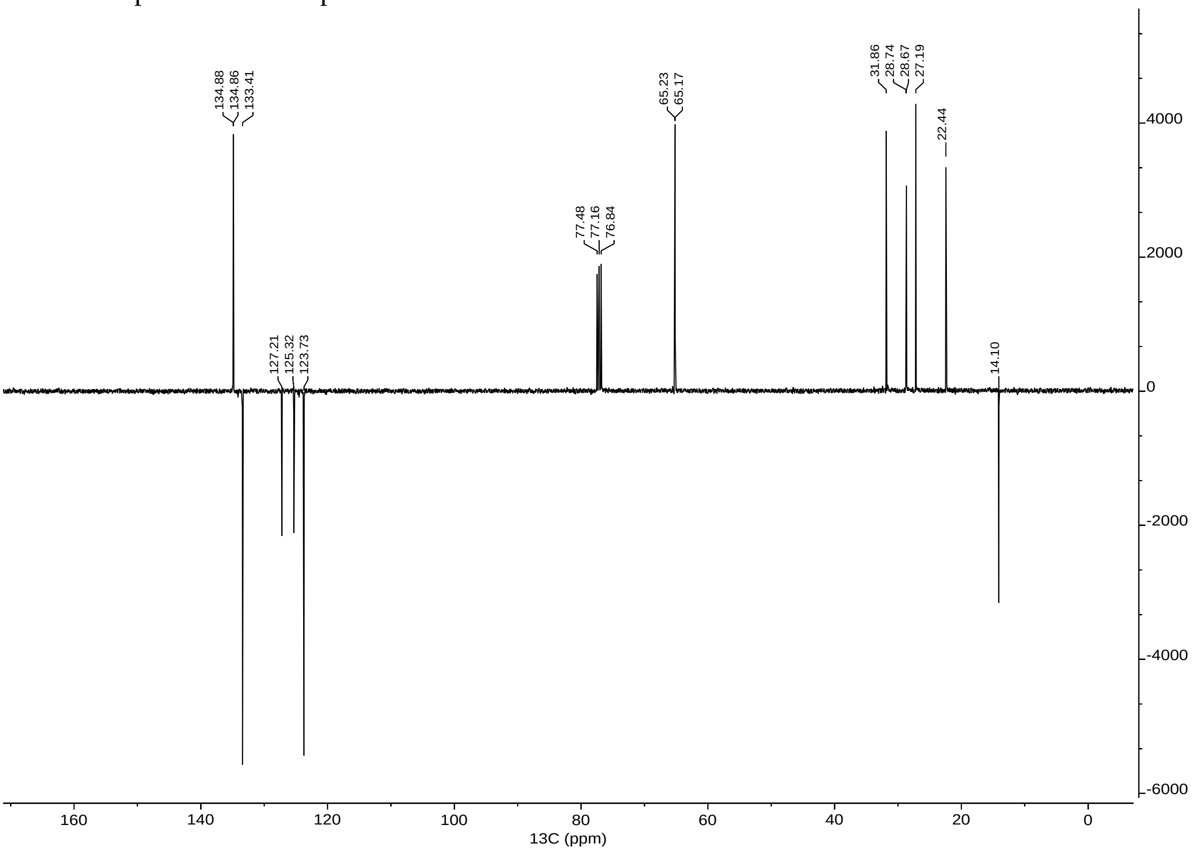

^{31}P{^{1}H} NMR spectrum of compound **S30**

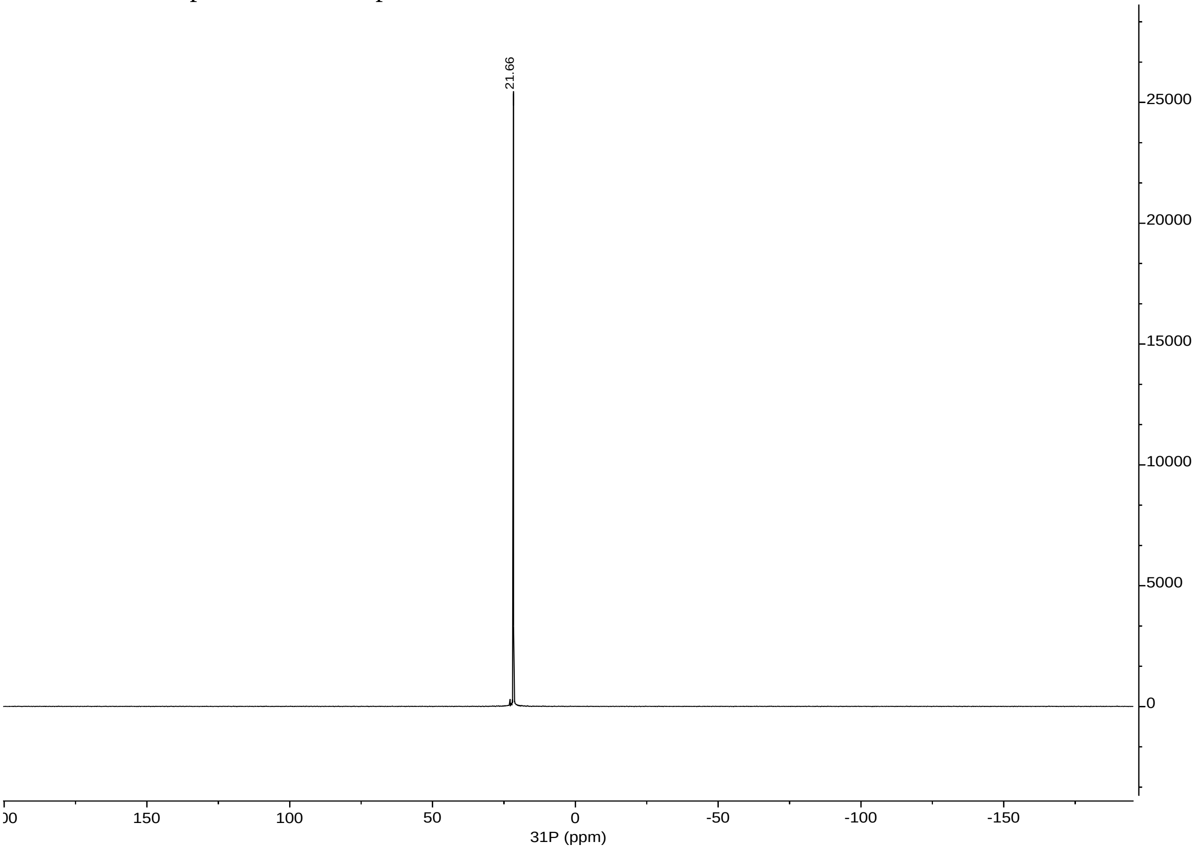

Mono phenethyl vinylphosphonate (S31)

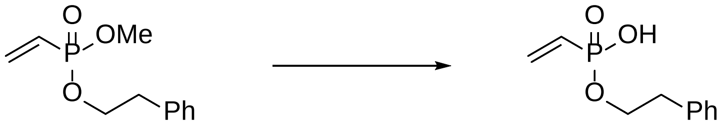

The title compound was prepared according to general method **A** from methyl phenethyl vinylphosphonate **S13** (6.70 g, 29.6 mmol) in 70% yield (4.42 g, 20.7 mmol) as a colourless oil.

^{1}H NMR (401 MHz, $CDCl_3$) δ 8.49 (brs, 1H, PO*H*), 7.39–7.12 (m, 5H, Ph*H*), 6.43–5.73 (m, 3H, C*H*=C*H*$_2$), 4.18 (q, *J* = 7.4 Hz, 2H, OC*H*$_2$), 2.98 (t, *J* = 7.2 Hz, 2H, C*H*$_2$Ph).

^{13}C NMR (101 MHz, $CDCl_3$) δ 137.28 (CH_2C_{quat}), 135.12 (=CH_2), 129.17, 128.64, 126.81 (C_{Ph}), 125.94 (d, *J* = 189.4 Hz, P*C*H), 66.23 (d, *J* = 6.0 Hz, O*C*H$_2$), 37.02 (d, *J* = 6.6 Hz, *C*H$_2$Ph).

^{31}P{^{1}H} NMR (162 MHz, $CDCl_3$) δ 22.26.

IR ν_{max}(film) 3086 (w), 3063 (w), 3029 (w), 2867 (w), 1614 (w), 1605 (m), 1497 (m), 1473 (w), 1454 (m), 1402 (m), 1276 (m), 1216 (s), 1179 (m), 1154 (m), 1061 (s), 1019 (vs), 1002(s), 988 (vs), 748 (m), 699 (s), 496 (m).

HR-MS(ESI$^+$): For $C_{10}H_{13}O_3NaP$ (M+Na)$^+$ *m*/*z* calculated 235.04945, found 235.04909. For $C_{10}H_{14}O_3P$ (M+H)$^+$ *m*/*z* calculated 213.06751, found 213.06721.

^{1}H NMR spectrum of compound **S31**

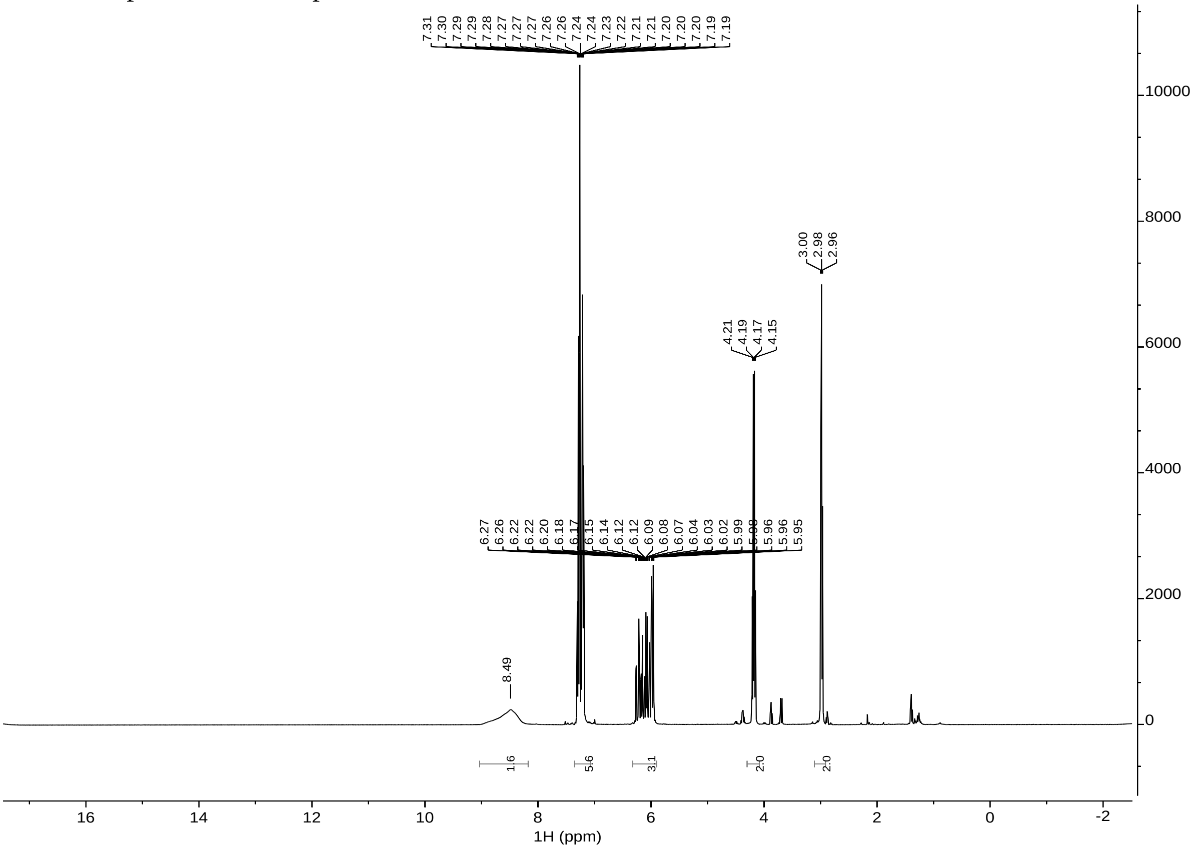

^{13}C NMR spectrum of compound **S31**

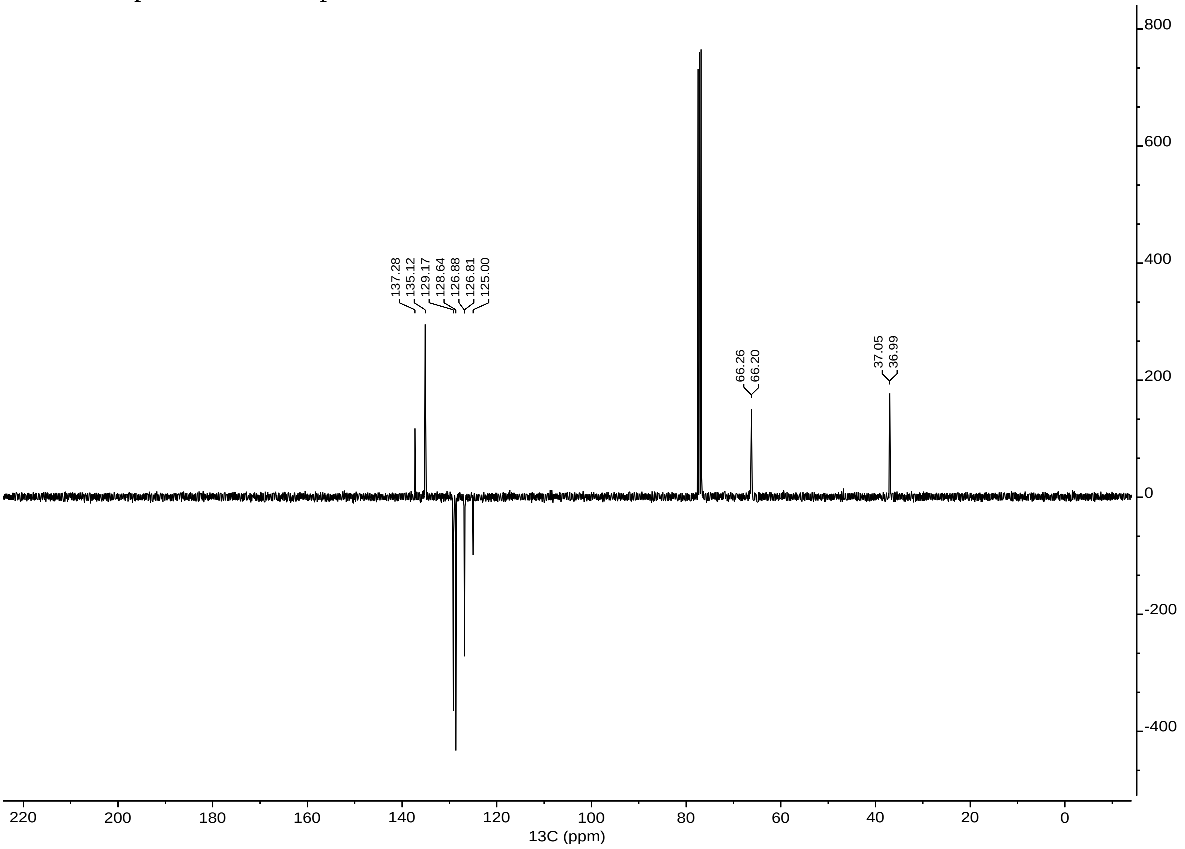

^{31}P{^{1}H} NMR spectrum of compound **S31**

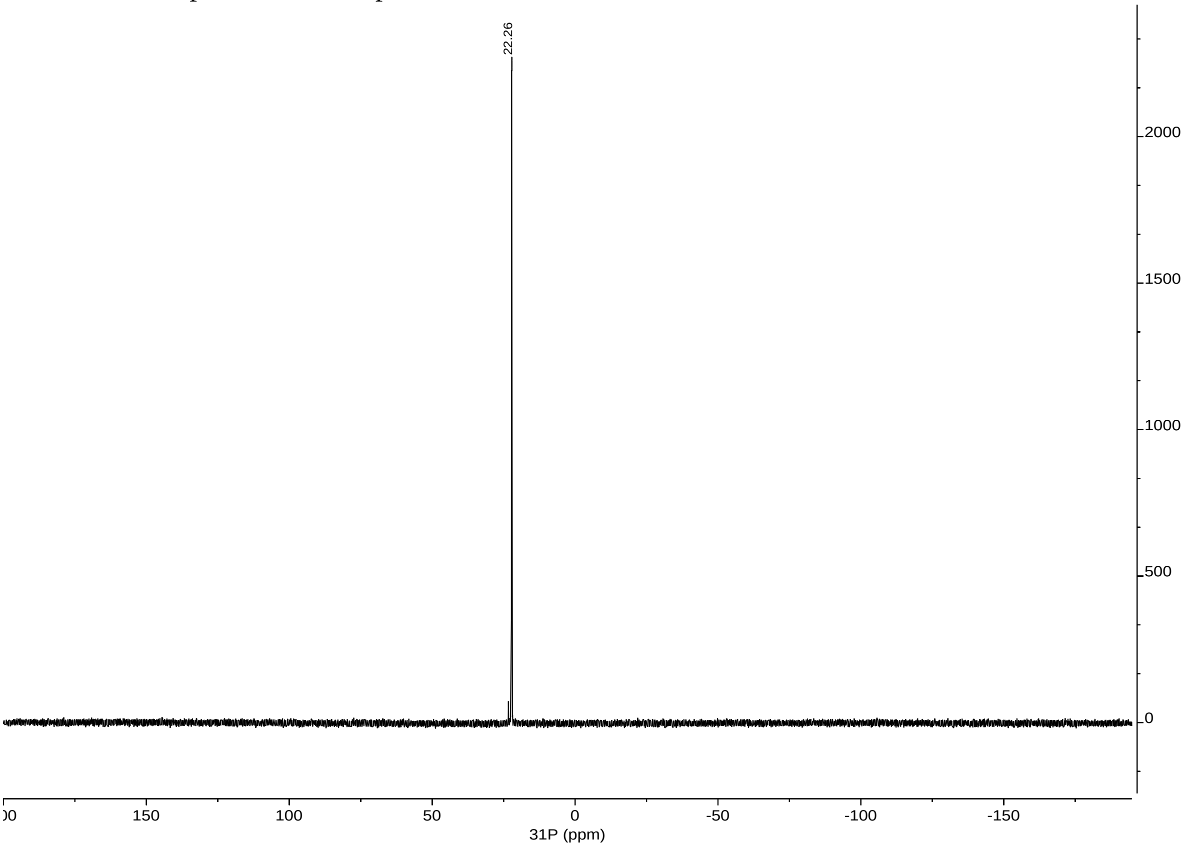

Mono (*Z*)-non-3-en-1-yl vinylphosphonate (S32)

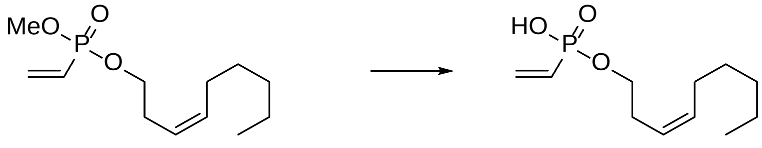

The title compound was prepared according to general method **A** from methyl (*Z*)-non-3-en-1-yl vinylphosphonate **S14** (4.25 g, 17.3 mmol) in 50% yield (2.00 g, 8.6 mmol) as a colourless oil.

^{1}H NMR (401 MHz, CD_3OD) δ 6.24–5.98 (m, 3H, C*H*=C*H*$_2$), 5.55–5.46 (m, 1H, C*H*(CH_2)$_4$$CH_3$), 5.43–5.34 (m, 1H, O($CH_2$)$_2$C*H*), 3.92 (q, *J* = 7.0 Hz, 2H, OC*H*$_2$), 2.41 (qd, *J* = 7.0, 1.5 Hz, 2H, OCH_2C*H*$_2$), 2.06 (qd, *J* = 7.1, 1.5 Hz, 2H, C*H*$_2$(CH_2)$_3$$CH_3$), 1.43–1.23 (m, 6H, (C*H*$_2$)$_3$$CH_3$), 0.91 (t, *J* = 6.7 Hz, 3H, C*H*$_3$).

^{13}C NMR (101 MHz, CD_3OD) δ 134.31 (d, *J* = 1.5 Hz, =*C*H_2), 133.87 (*C*H(CH_2)$_4$$CH_3$), 128.92 (d, *J* = 181.5 Hz, P*C*H), 125.37 (O(CH_2)$_2$*C*H), 66.04 (d, *J* = 5.7 Hz, O*C*H_2), 32.62 (*C*$H_2$$CH_2$$CH_3$), 30.40 (*C*$H_2$($CH_2$)$_2$$CH_3$), 29.72 (d, *J* = 6.6 Hz, OCH_2*C*H_2), 28.25 (*C*H_2(CH_2)$_3$$CH_3$), 23.61 (*C*$H_2$$CH_3$), 14.41 (*C*$H_3$).

^{31}P{^{1}H} NMR (162 MHz, CD_3OD) δ 18.44.

IR ν_{max} (KBr) 3088 (vw), 2959 (s), 2930 (s), 2873 (m), 2858 (s), 2733 (w), 2669 (w, vbr), 2337 (m, br), 1660 (m, vbr), 1655 (w), 1615 (m), 1468 (m), 1460 (m), 1403 (m), 1380 (w), 1277 (m), 1198 (s), 1165 (s, sh), 1155 (s), 1023 (vs), 986 (vs).

HR-MS(ESI^{+}): For $C_{11}H_{21}O_3NaP$ $(M+Na)^+$ *m*/*z* calculated 255.11205, found 255.11186.

^{1}H NMR spectrum of compound **S32**

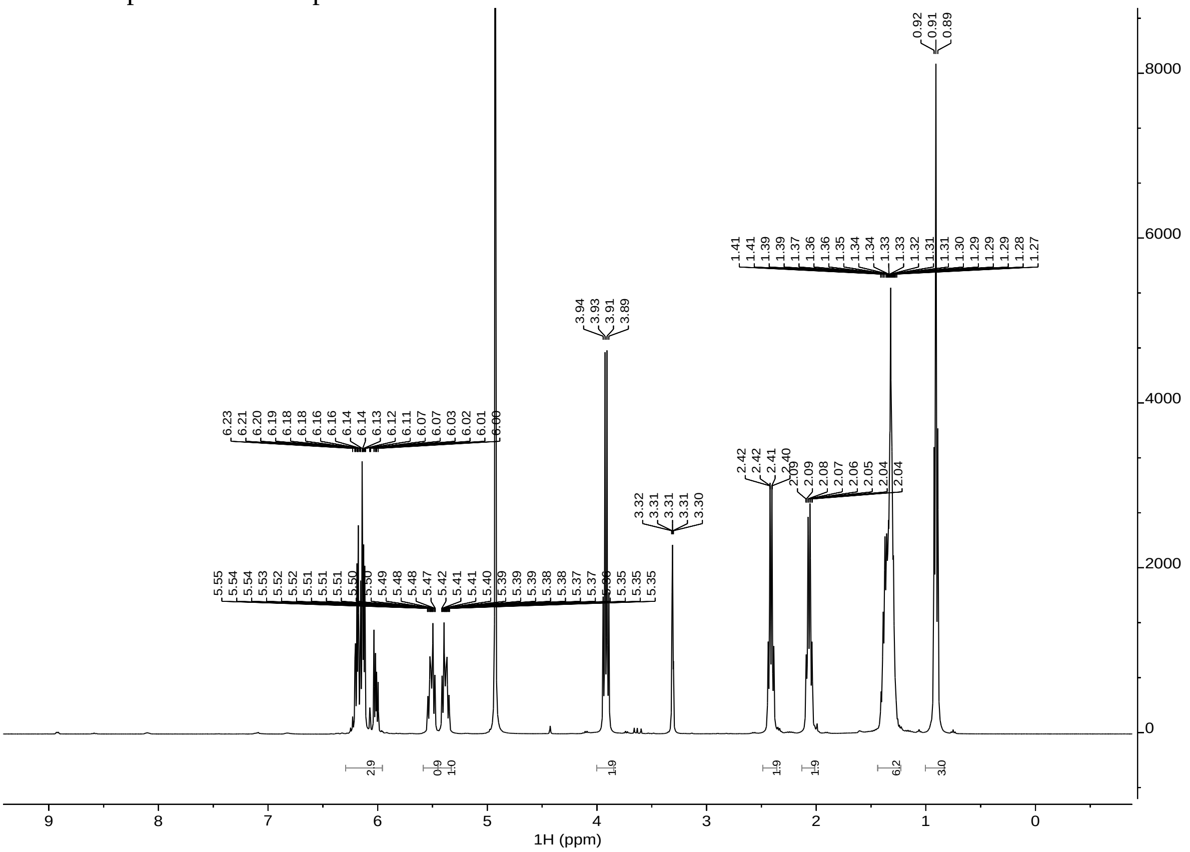

^{13}C NMR spectrum of compound **S32**

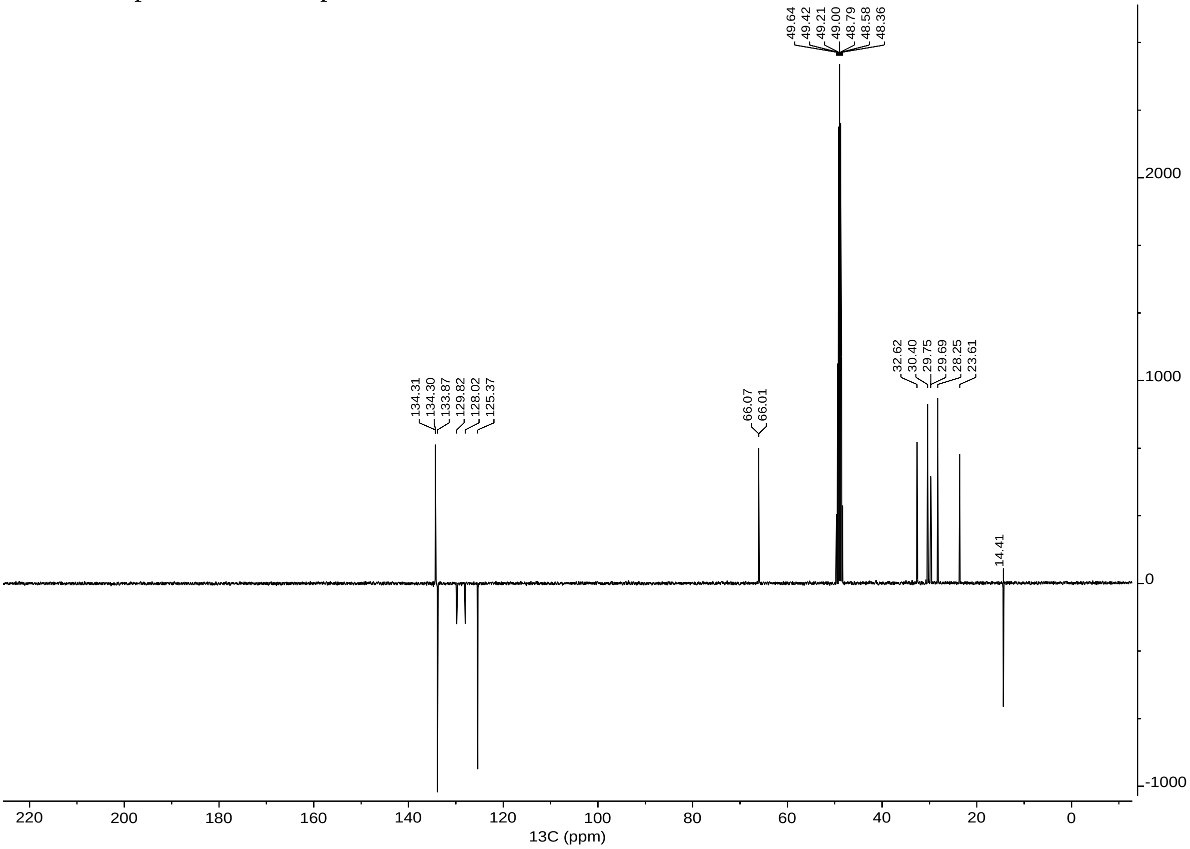

^{31}P{^{1}H} NMR spectrum of compound **S32**

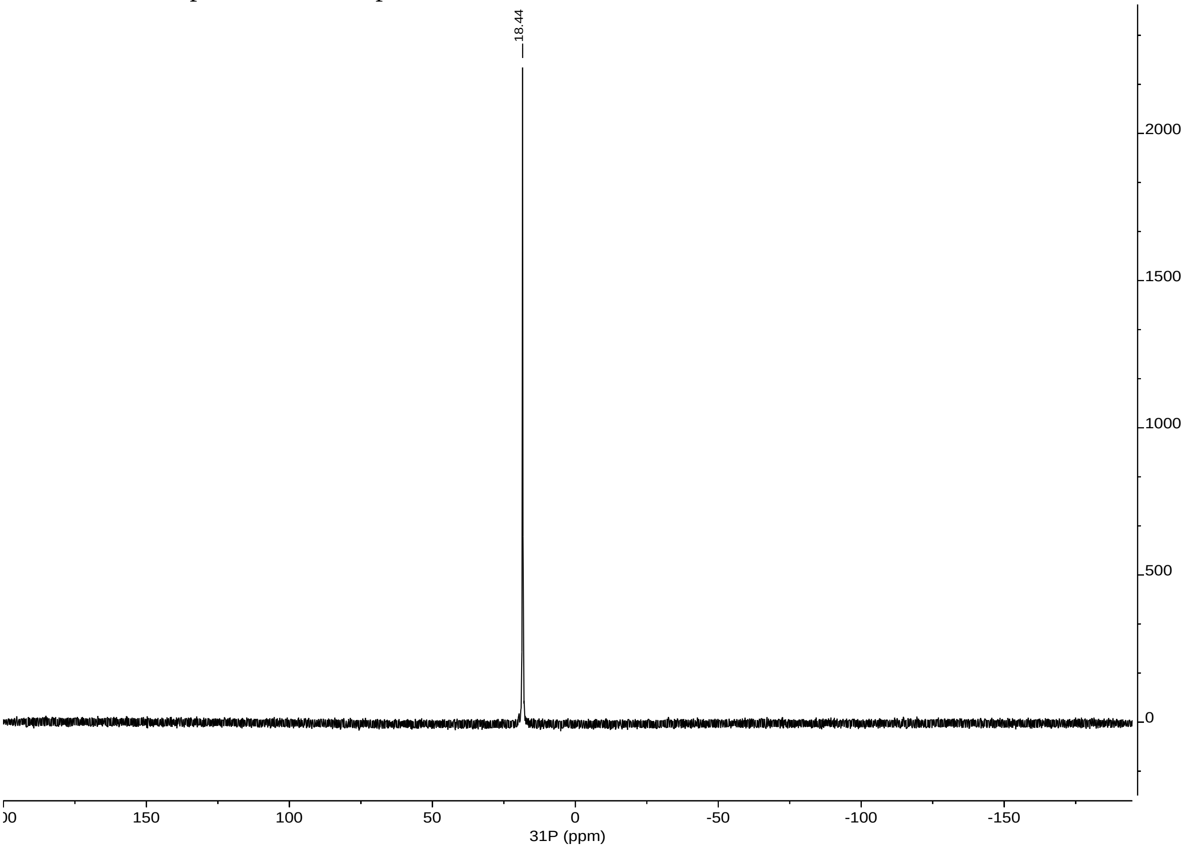

Mono decyl vinylphosphonate (S33)

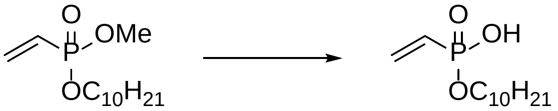

The title compound was prepared according to general method **A** from methyl decyl vinylphosphonate **S15** (11.6 g, 44.2 mmol) in 76% yield (8.52 g, 34.3 mmol) as a colourless oil.

^{1}H NMR (401 MHz, $CDCl_3$) δ 10.98 (brs, 1H, PO*H*), 6.35–5.99 (m, 3H, C*H*=C*H*$_2$), 3.99 (q, *J* = 6.8 Hz, 2H, OC*H*$_2$), 1.71–1.62 (m, 2H, OCH$_2$C*H*$_2$), 1.40–1.19 (m, 14H, (C*H*$_2$)$_7$CH$_3$), 0.91–0.84 (m, 3H, C*H*$_3$).

^{31}P{^{1}H} NMR (162 MHz, $CDCl_3$) δ 22.13.

^{13}C NMR (101 MHz, $CDCl_3$) δ 134.86 (=*C*H$_2$), 126.27 (d, *J* = 189.3 Hz, P*C*H), 65.97 (d, *J* = 6.1 Hz, O*C*H$_2$), 32.03 (*C*H$_2$CH$_2$CH$_3$), 30.50 (d, *J* = 6.6 Hz, OCH$_2$*C*H$_2$), 29.68, 29.66, 29.45, 29.31, 25.63, 22.82 (*C*H$_2$CH$_3$), 14.26 (*C*H$_3$).

IR ν_{max}($CHCl_3$) 3090 (vw), 2957 (vs), 2928 (vs), 2872 (s), 2856 (vs), 2731 (m, br), 2675 (m, vbr), 2327 (m, br), 2174 (m, br, sh), 1667 (m, vbr), 1615 (s), 1467 (s), 1458 (m), 1403 (s), 1379 (m), 1277 (m), 1197 (vs), 1024 (vs, br), 986 (vs, br), 901 (m).

HR-MS(ESI$^+$): For $C_{12}H_{25}O_3NaP$ (M+Na)$^+$ *m*/*z* calculated 271.14335, found 271.14340. For $C_{12}H_{26}O_3P$ (M+H)$^+$ *m*/*z* calculated 249.16141, found 249.16152.

^{1}H NMR spectrum of compound **S33**

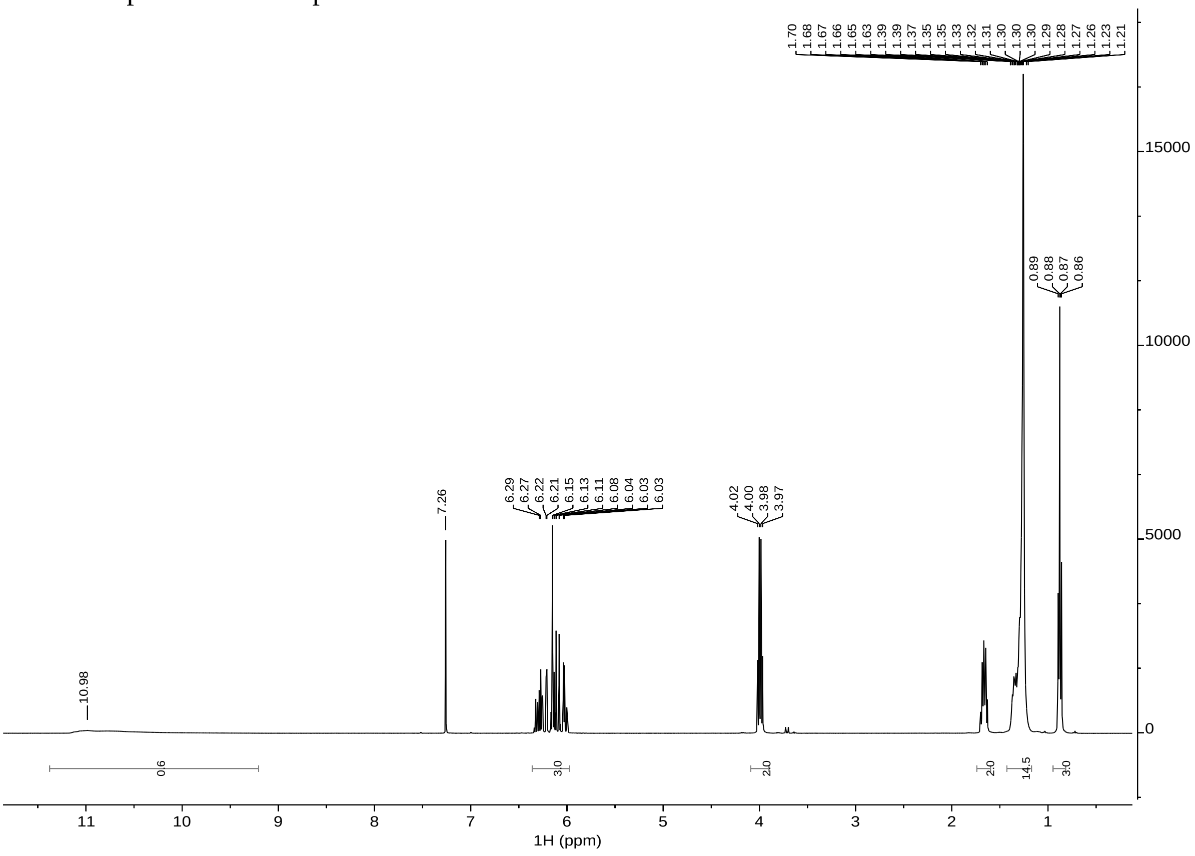

^{13}C NMR spectrum of compound **S33**

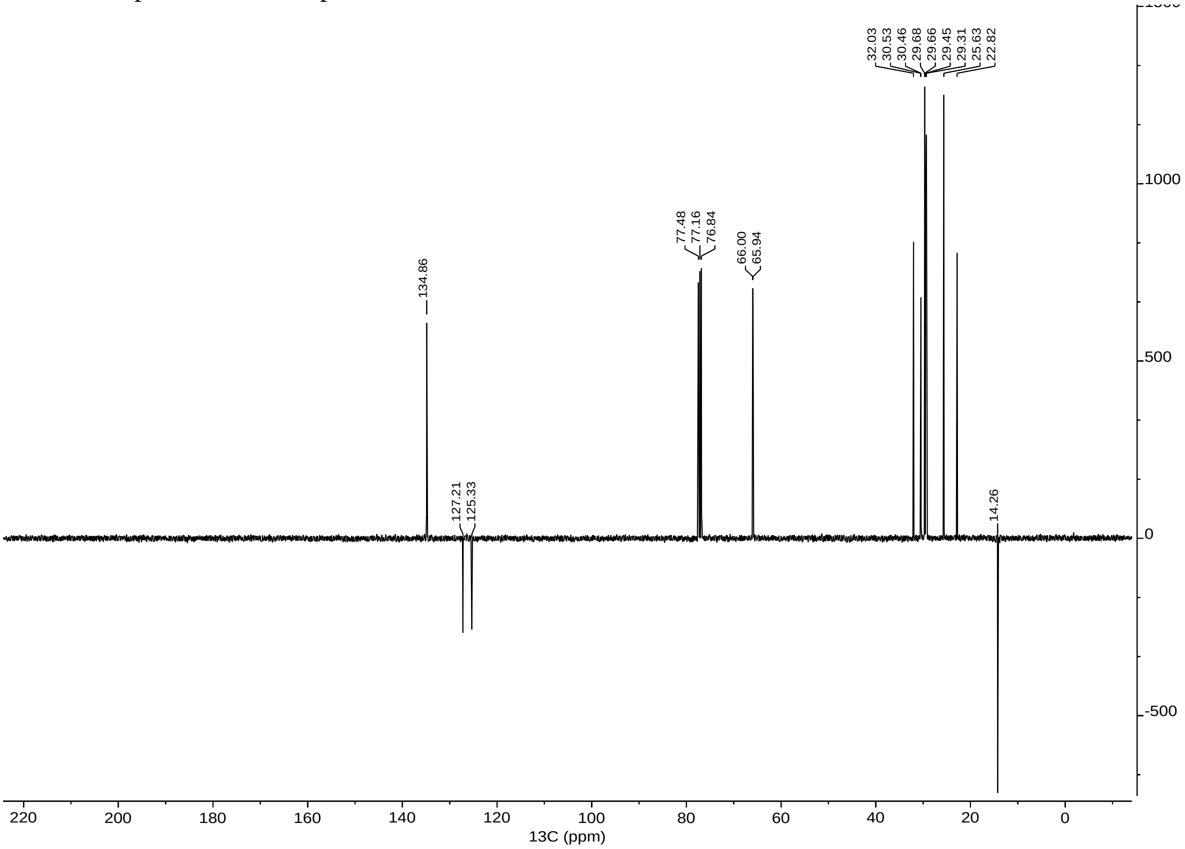

$^{31}P\{^{1}H\}$ NMR spectrum of compound **S33**

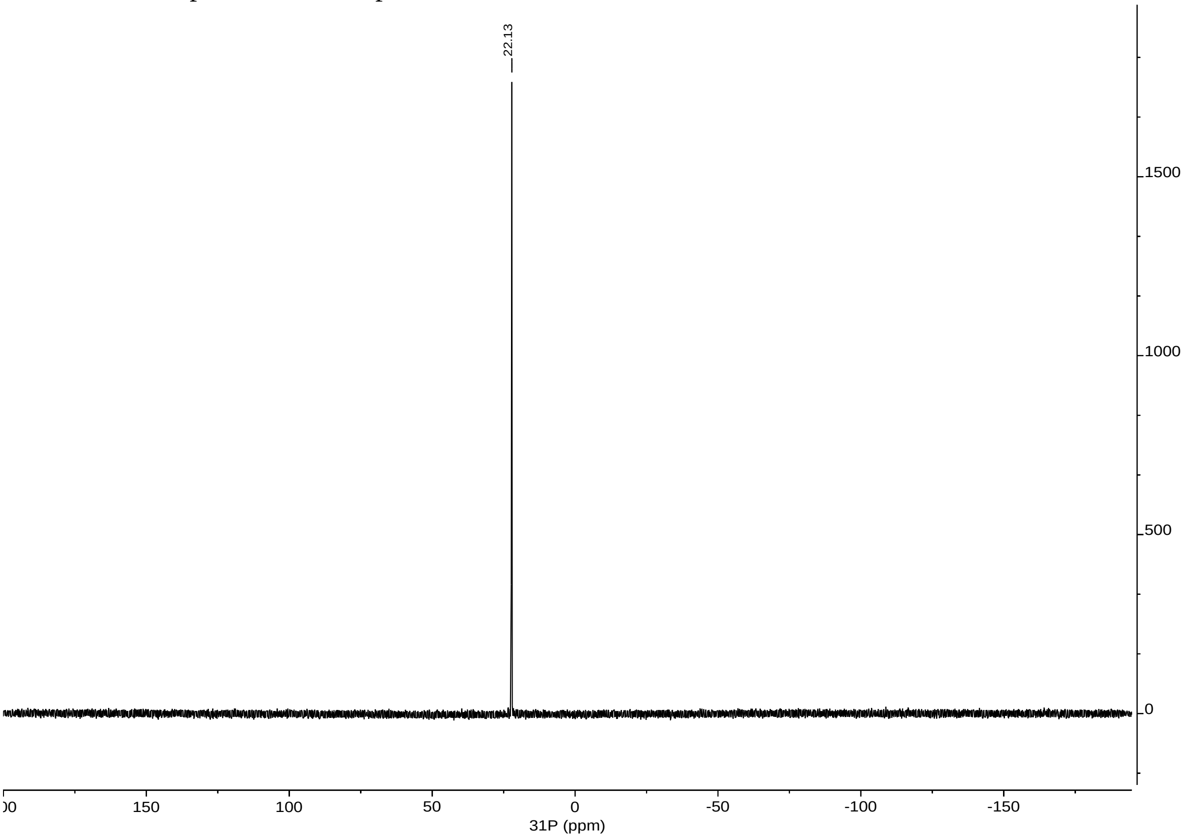

Mono (*Z*)-dec-4-en-1-yl vinylphosphonate (S34)

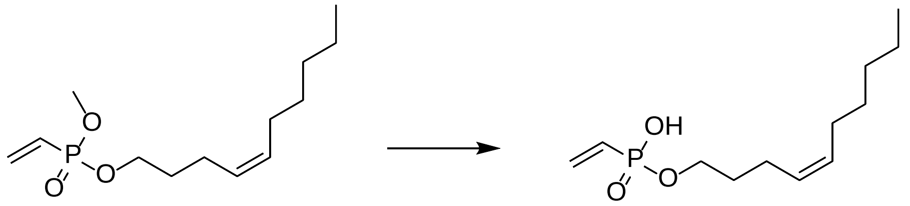

The title compound was prepared according to general method **A** from (*Z*)-dec-4-en-1-yl methyl vinylphosphonate **S16** (1.47 g, 5.67 mmol) in 86% yield (1.20 g, 4.87 mmol) as a colourless oil.

^{1}H NMR (401 MHz, $CDCl_3$): 7.34 (s, 1H, PO*H*), 6.36–6.00 (m, 3H, C*H*=C*H*$_2$), 5.44–5.37 (m, 1H, $CH_3(CH_2)_4$C*H*), 5.35–5.28 (m, 1H, C*H*$(CH_2)_3O$), 4.00 (q, 2H, *J* = 6.7 Hz, $CH(CH_2)_2$C*H*$_2$O), 2.15–1.95 (m, 2H, CHC*H*$_2$$(CH_2)_2O$), 2.01 (td, 2H, *J* = 7.2, 1.3 Hz, $CH_3(CH_2)_3$C*H*$_2$), 1.73 (dq, 2H, *J* = 8.6, 6.6 Hz, C*H*$_2$$CH_2O$), 1.37–1.22 (m, 6H, CH_3(C*H*$_2$)$_3$), 0.88 (t, 3H, *J* = 8.3, C*H*$_3$).

^{13}C NMR (101 MHz, $CDCl_3$): 134.84 (d, *J* = 2.1 Hz, CH=*C*H$_2$), 131.43 ($CH_3(CH_2)_4$*C*H), 128.06 (*C*H$(CH_2)_3O$), 126.29 (d, *J* = 189.6 Hz, *C*H=CH$_2$), 65.31 (d, *J* = 6.0 Hz, *C*H$_2$O), 31.65 (CH_3CH_2*C*H$_2$), 30.53 (d, *J* = 6.9 Hz, *C*H$_2$$CH_2O$), 29.48 ($CH_3(CH_2)_2$*C*H$_2$), 27.31 $CH_3(CH_2)_3$*C*H$_2$), 23.31 (*C*H$_2$$(CH_2)_2O$), 22.71 ($CH_3$*C*H$_2$), 14.22 (*C*H$_3$).

^{31}P{^{1}H} NMR (162 MHz, $CDCl_3$): 22.28.

IR ν_{max} (KBr) 3097 (w), 2959 (s), 2929 (s), 2873 (m), 2858 (m), 2731(w), 2668 (w), 2335 (m), 1654 (w), 1615 (m), 1467 (m), 1458 (m), 1403 (m), 1379 (m), 1277 (m), 1197 (s), 1085–1024 (vs), 986 (vs).

HR-MS(ESI^{+}): For $C_{12}H_{23}O_3NaP$ $(M+Na)^{+}$ *m/z* calculated 269.12770, found 269.12751.

^{1}H NMR spectrum of compound **S34**

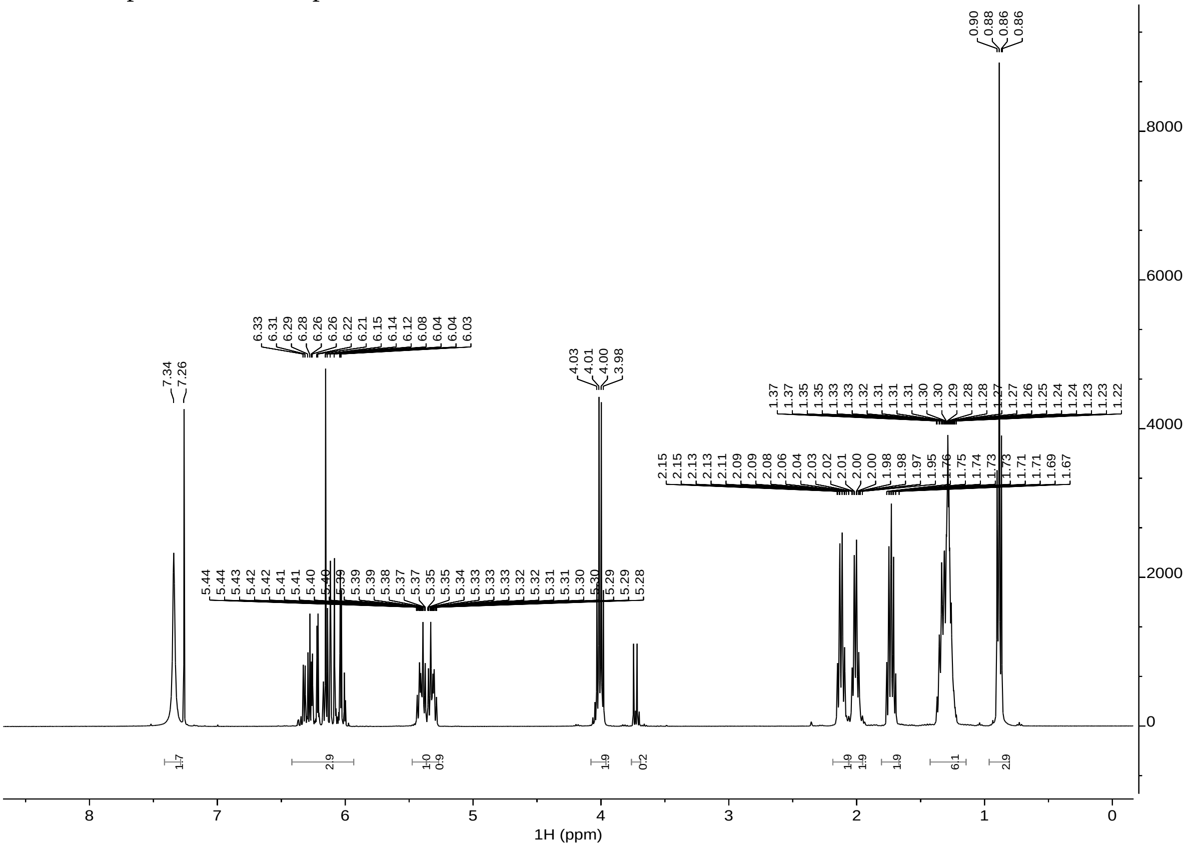

^{13}C NMR spectrum of compound **S34**

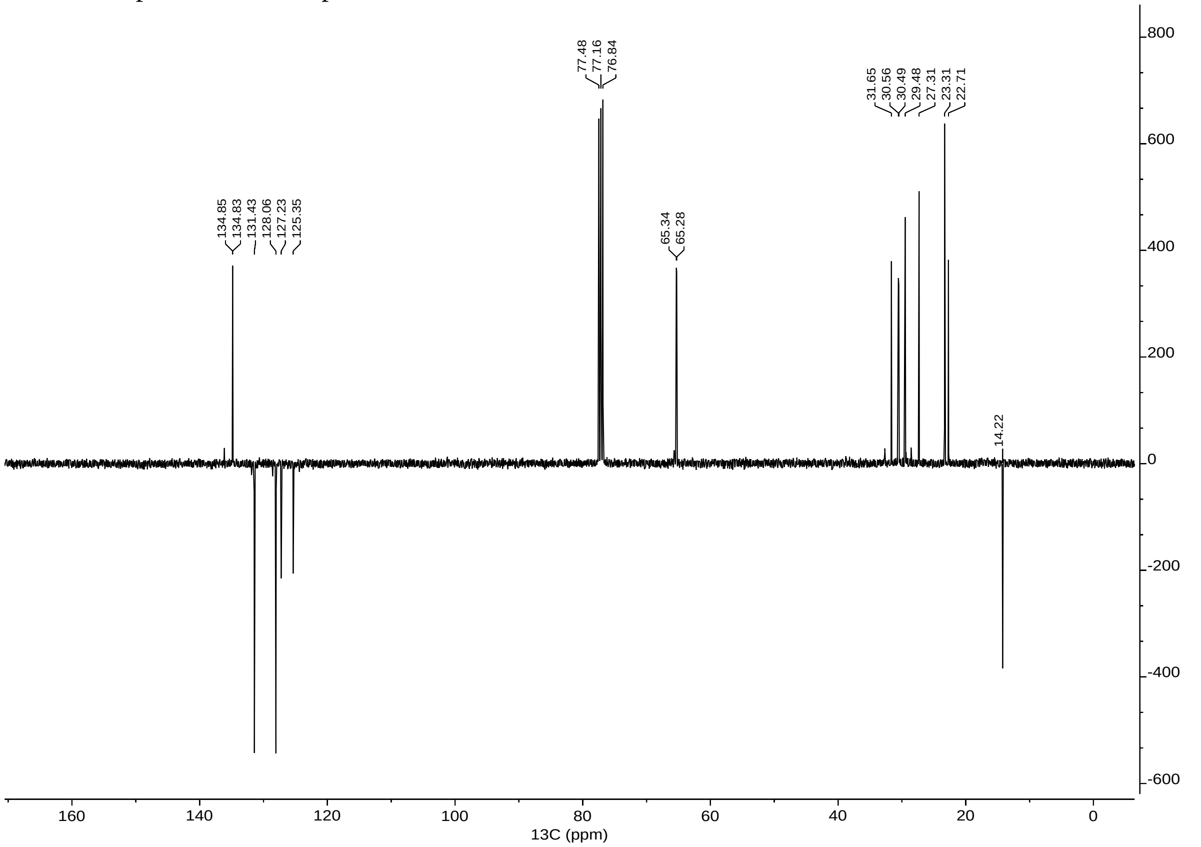

$^{31}P\{^{1}H\}$ NMR spectrum of compound **S34**

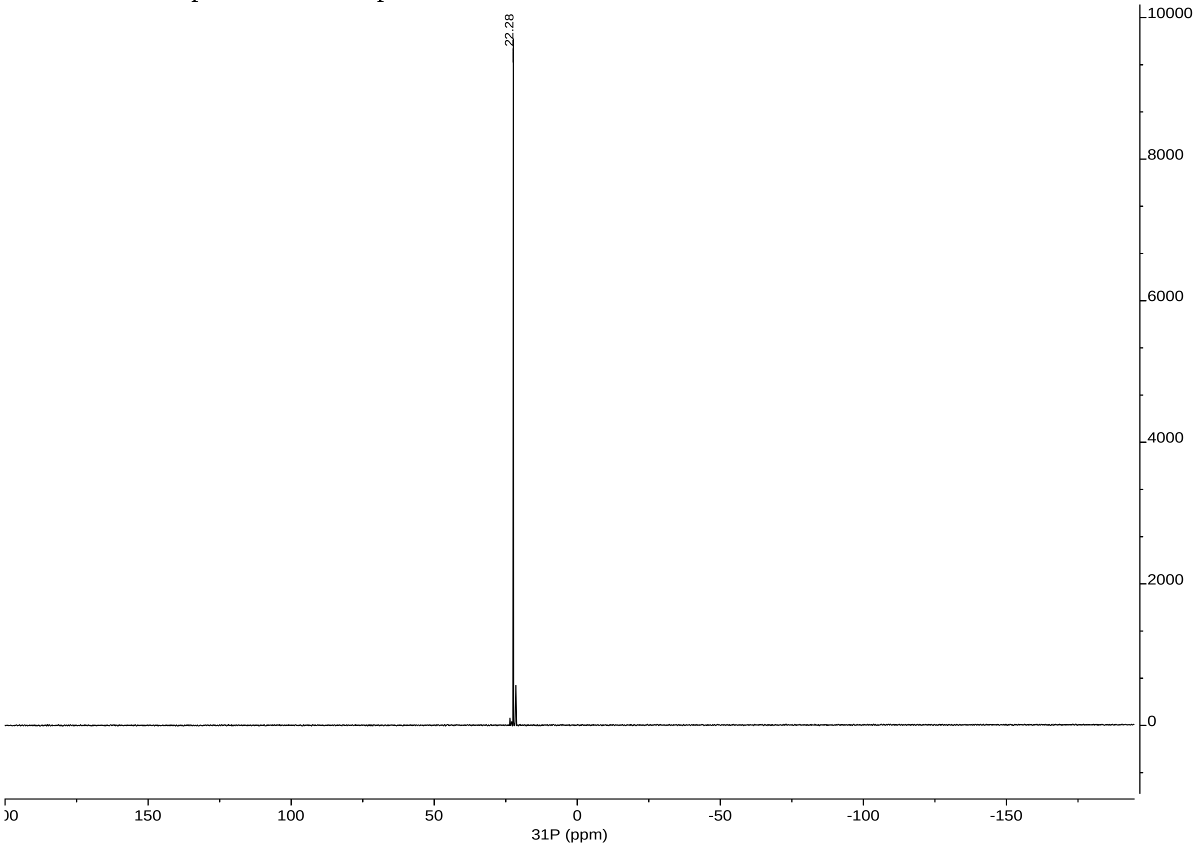

Mono (adamantan-1-yl)methyl vinylphosphonate (S35)

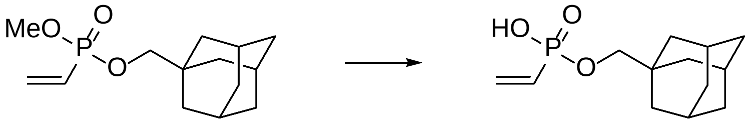

The title compound was prepared according to general method **A** from (adamantan-1-yl)methyl methyl vinylphosphonate **S17** (9.07 g, 33.6 mmol) in 90% yield (7.74 g, 30.2 mmol) as a colourless oil.

^{1}H NMR (401 MHz, CDCl$_3$) δ 10.61 (s, 1H, PO*H*), 6.34–5.98 (m, 3H, C*H*=C*H*$_2$), 3.55 (d, *J* = 5.9 Hz, 2H, OC*H*$_2$), 1.98 (p, *J* = 3.1 Hz, 3H, C$_{quat}$CH$_2$C*H*), 1.76–1.59 (m, 6H, C$_{quat}$C*H*$_2$CH), 1.53 (d, *J* = 2.9 Hz, 6H, *c*-(CHC*H*$_2$)$_3$).

^{13}C NMR (101 MHz, CDCl$_3$) δ 134.73 (d, *J* = 1.8 Hz, =*C*H$_2$), 126.23 (d, *J* = 189.8 Hz, P*C*H), 75.34 (d, *J* = 6.6 Hz, O*C*H$_2$), 38.96 (*c*-(CH*C*H$_2$)$_3$), 37.07 (C$_{quat}$*C*H$_2$CH), 33.86 (d, *J* = 7.1 Hz, *C*$_{quat}$), 28.14 (*c*-(*C*HCH$_2$)$_3$).

^{31}P{^{1}H} NMR (162 MHz, CDCl$_3$) δ 22.39.

IR ν_{max} (KBr) 3088 (vw), 2959 (s), 2930 (s), 2873 (m), 2858 (s), 2733 (w), 2669 (w, vbr), 2337 (m, br), 1660 (m, vbr), 1655 (w), 1615 (m), 1468 (m), 1460 (m), 1403 (m), 1380 (w), 1277 (m), 1198 (s), 1165 (s, sh), 1155 (s), 1023 (vs), 986 (vs).

HR-MS(ESI$^+$) For $C_{11}H_{21}O_3NaP$ (M+Na)$^+$ *m/z* calculated 255.11205, found 255.11186.

^{1}H NMR spectrum of compound **S35**

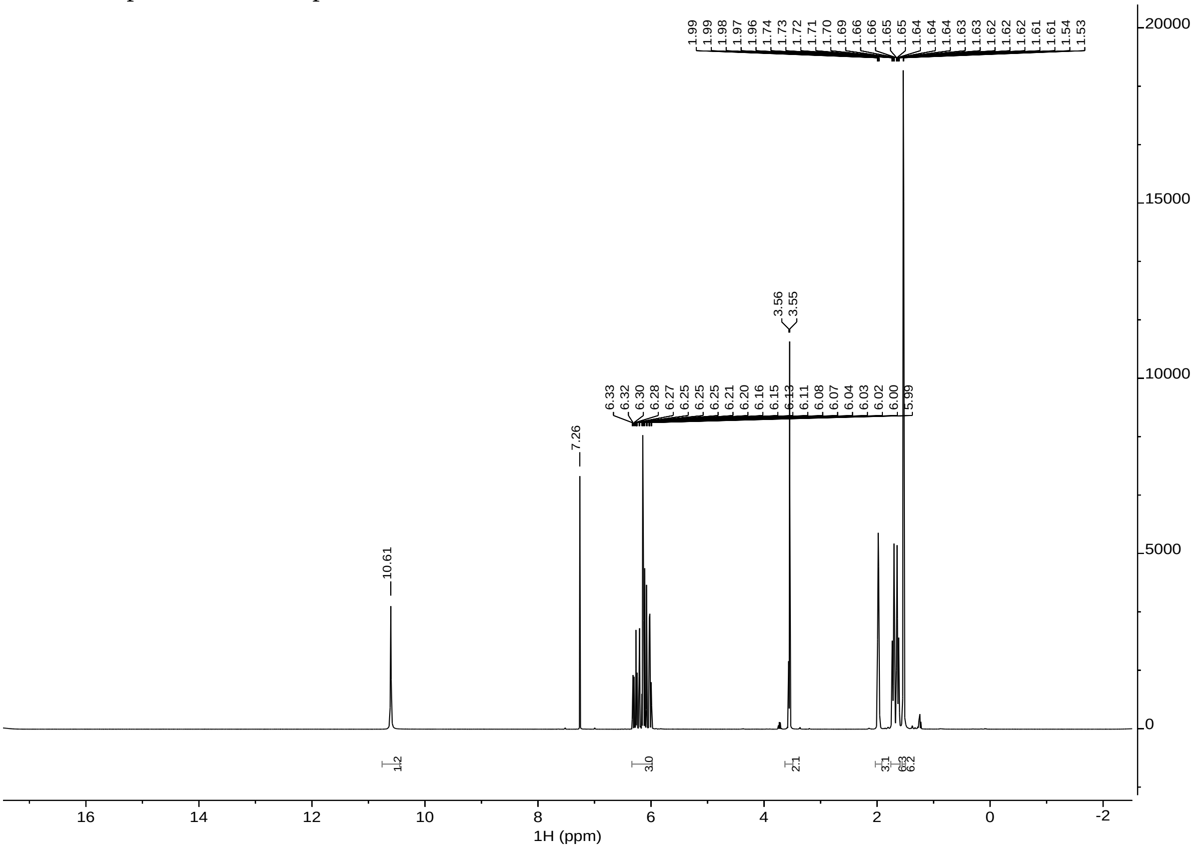

^{13}C NMR spectrum of compound **S35**

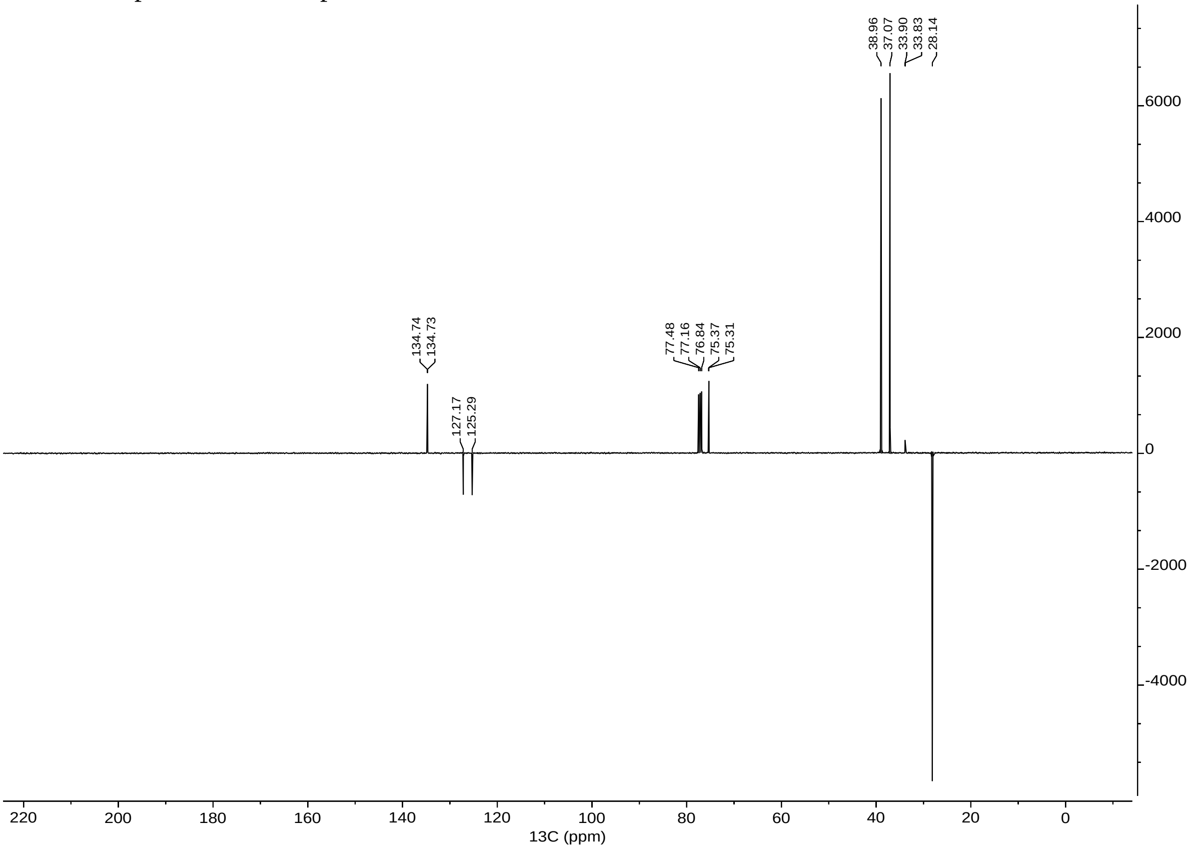

$^{31}P\{^1H\}$ NMR spectrum of compound **S35**

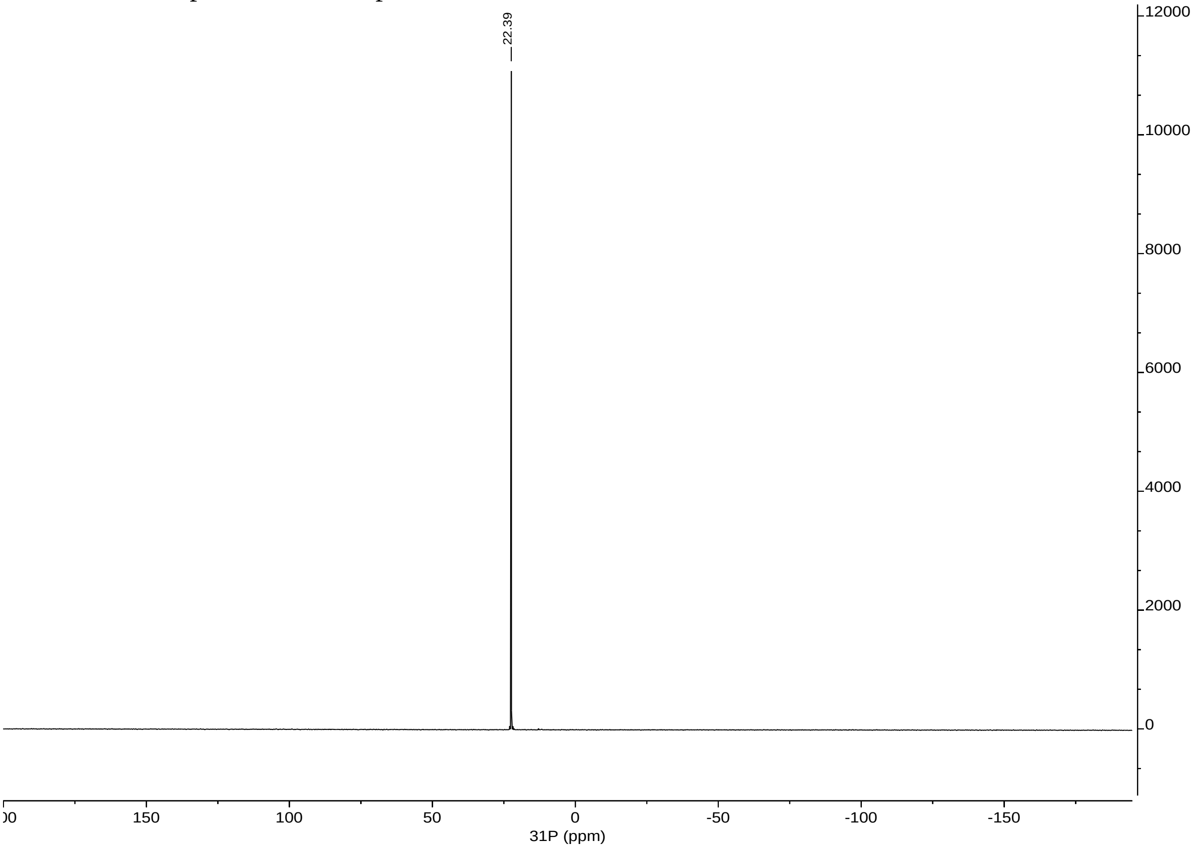

Mono 2-(adamantan-1-yl)ethyl vinylphosphonate (S36)

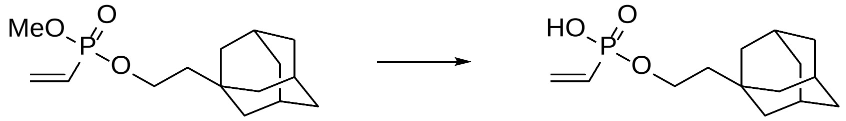

The title compound was prepared according to general method **A** from 2-(adamantan-1-yl)ethyl methyl vinylphosphonate **S18** (4.85 g, 17.0 mmol) in 71% yield (3.24 g, 12.0 mmol) as a colourless oil.

^{1}H NMR (401 MHz, $CDCl_3$) δ 8.44 (s, 1H, PO*H*), 6.35–5.99 (m, 3H, C*H*=C*H*$_2$), 4.08 (q, *J* = 7.4 Hz, 2H, *OCH*$_2$), 1.94 (p, *J* = 3.1 Hz, 3H, C*H*), 1.73–1.58 (m, 6H, C_{quat}C*H*$_2$CH), 1.54–1.46 (m, 8H, *c*-(CHC*H*$_2$)$_3$, OCH$_2$C*H*$_2$).

^{13}C NMR (101 MHz, $CDCl_3$) δ 134.83 (d, *J* = 2.0 Hz, =*C*H$_2$), 126.36 (d, *J* = 189.2 Hz, P*C*H), 62.30 (d, *J* = 6.1 Hz, O*C*H$_2$), 44.31 (d, *J* = 6.4 Hz, OCH$_2$*C*H$_2$), 42.61 (*c*-(CH*C*H$_2$)$_3$), 37.12 (C_{quat}*C*H$_2$CH), 31.92 (*C*$_{quat}$), 28.69 (*C*H).

^{31}P{^{1}H} NMR (162 MHz, $CDCl_3$) δ 22.26.

IR ν_{max}($CHCl_3$) 3083 (vw), ~2960 (m, sh), 2926 (s), 2904 (vs), 2849 (s), 2753–2637 (vw), ≈1660 (w, vbr), 1614 (w), 1451 (m), 1400 (m), 1362 (vw), 1346 (m), 1276 (m), 1201 (s), 1106 (m), 1072 (s), 1046 (s), ~1029 (s, sh), 1018 (s), 989 (s), 972 (s), 811 (m), 437 (w).

HR-MS(ESI$^-$) For $C_{14}H_{22}O_3P$ (M–H)$^-$ *m*/*z* calculated 269.13120, found 269.13132.

^{1}H NMR spectrum of compound **S36**

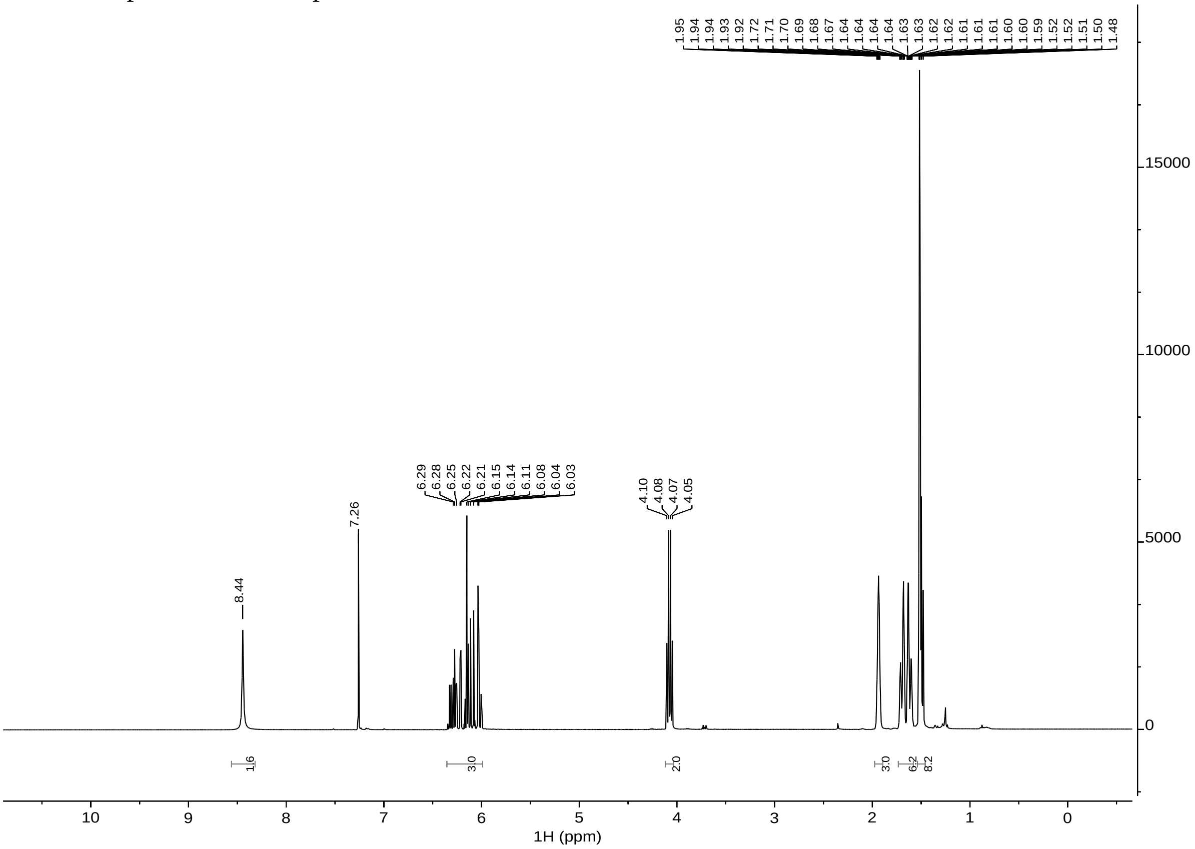

^{13}C NMR spectrum of compound **S36**

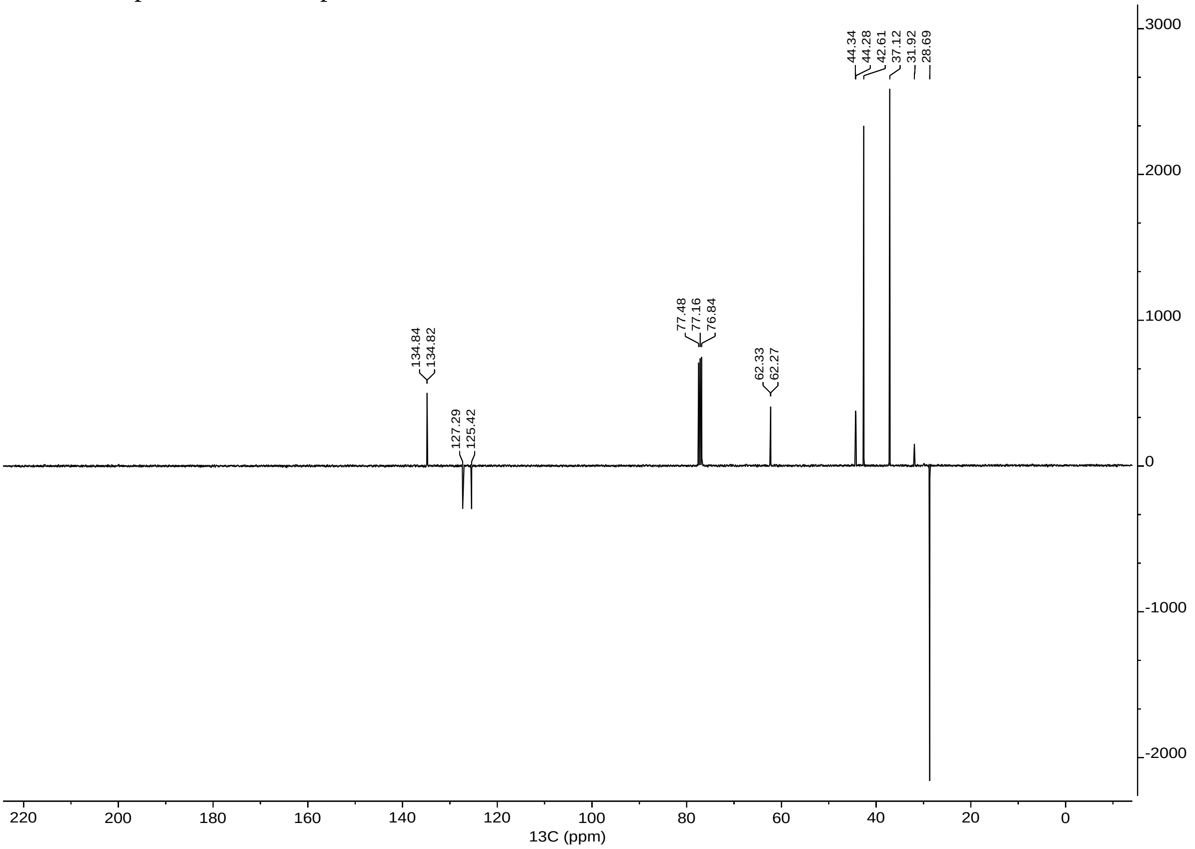

$^{31}P\{^1H\}$ NMR spectrum of compound **S36**

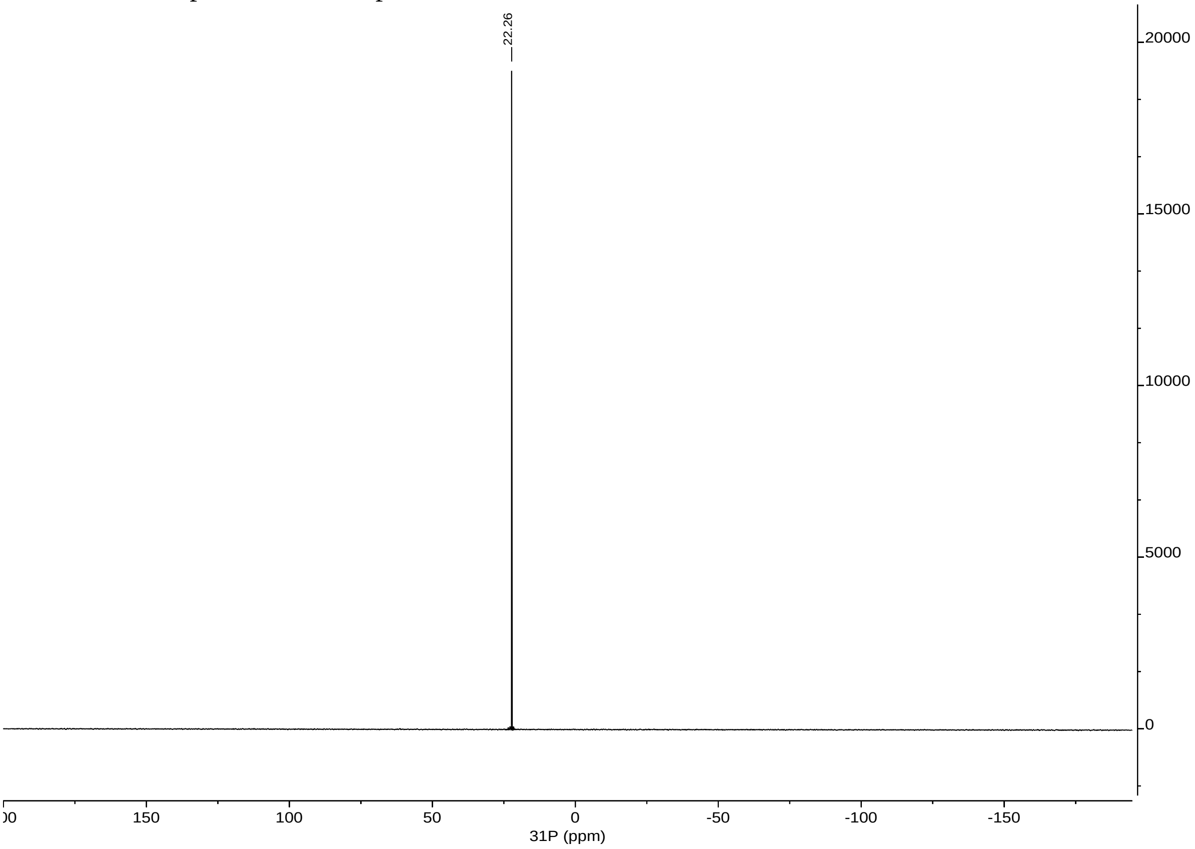

Bis(cyclopentylmethyl) propane-1,3-diyl bis(vinylphosphonate) (S37)

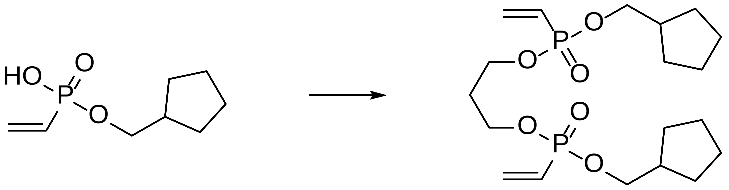

The title compound was prepared according to general method **C** from mono alkyl vinylphosphonate **S24** (5.00 g, 26.3 mmol) and 1,3-dibromopropane (0.93 mL, 9.20 mmol) in 77% yield (2.97 g, 7.06 mmol) as a colourless oil.

^{1}H NMR (401 MHz, $CDCl_3$) δ 6.33–5.93 (m, 6H, C*H*=C*H*$_2$), 4.09 (dt, *J* = 7.3, 6.1 Hz, 4H, OC*H*$_2$CH$_2$), 3.85 (t, *J* = 6.9 Hz, 4H, OC*H*$_2$-cyclopent), 2.19 (hept, *J* = 7.5 Hz, 2H, *H*-1-cyclopent), 2.00 (p, *J* = 6.1 Hz, 2H, OCH$_2$C*H*$_2$), 1.77–1.64 (m, 4H, *H*-2a,5a-cyclopent), 1.61–1.45 (m, 8H, *H*-3,4-cyclopent), 1.30–1.16 (m, 4H, *H*-2b,5b-cyclopent).

^{13}C NMR (101 MHz, $CDCl_3$) δ 135.89 (d, *J* = 2.0 Hz, =*C*H$_2$), 125.59 (d, *J* = 184.1 Hz, P*C*H), 69.81 (d, *J* = 6.0 Hz, O*C*H$_2$-cyclopent), 61.81 (d, *J* = 5.5 Hz, O*C*H$_2$CH$_2$), 40.03 (d, *J* = 6.5 Hz, *C*H-1-cyclopent), 31.50 (t, *J* = 6.6 Hz, OCH$_2$*C*H$_2$), 29.05 (d, *J* = 3.9 Hz, *C*H$_2$-2,5-cyclopent), 25.41 (*C*H$_2$-3,4-cyclopent).

^{31}P{^{1}H} NMR (162 MHz, $CDCl_3$) δ 20.14.

IR ν_{max} ($CHCl_3$) 3091 (vw), 2960 (s), 2871 (m), 1614 (w), 1467 (w), 1453 (w), 1400 (m), 1279 (w), 1241 (s), 1053 (s), 1023 (vs), 1009 (vs), 986 (vs).

HR-MS(ESI$^+$): For $C_{19}H_{35}O_6P_2$ $(M+H)^+$ *m*/*z* calculated 421.19034, found 421.18970. For $C_{19}H_{34}O_6NaP_2$ $(M+Na)^+$ *m*/*z* calculated 443.17228, found 443.17160.

^{1}H NMR spectrum of compound **S37**

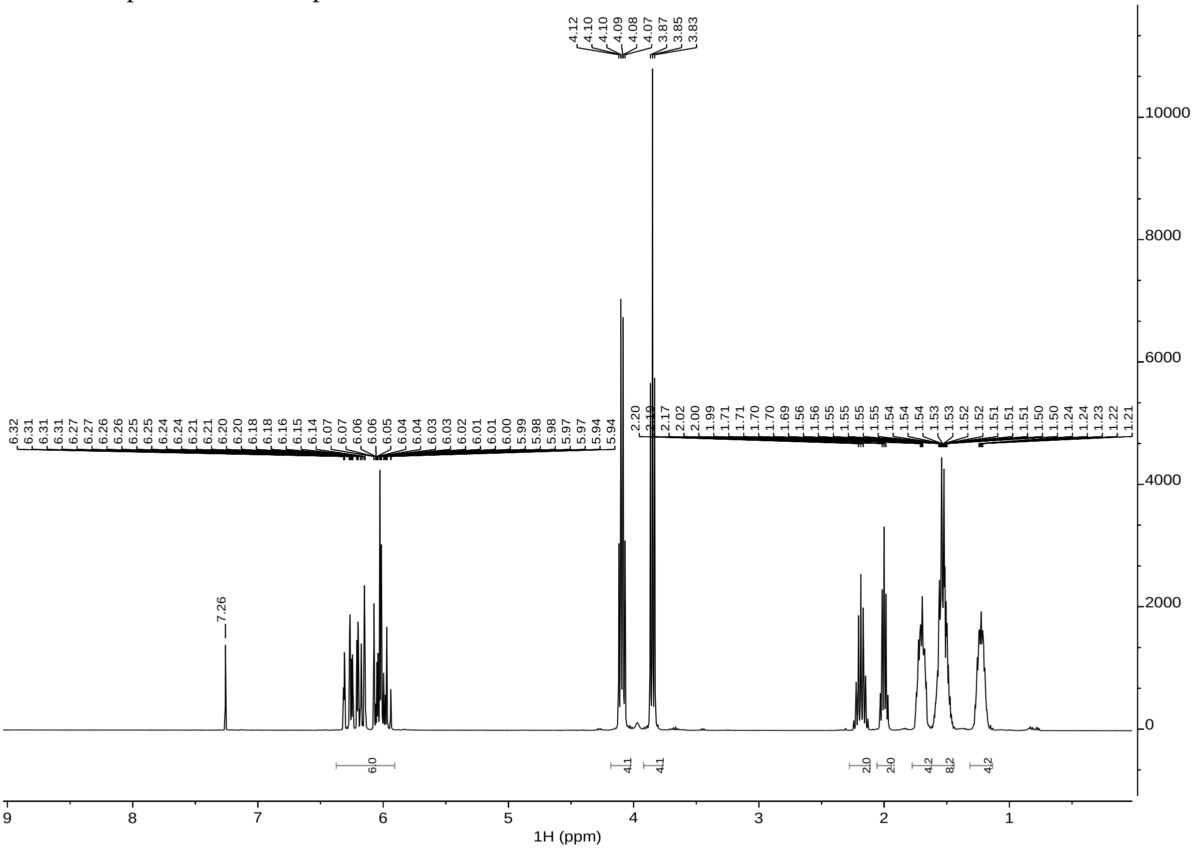

^{13}C NMR spectrum of compound **S37**

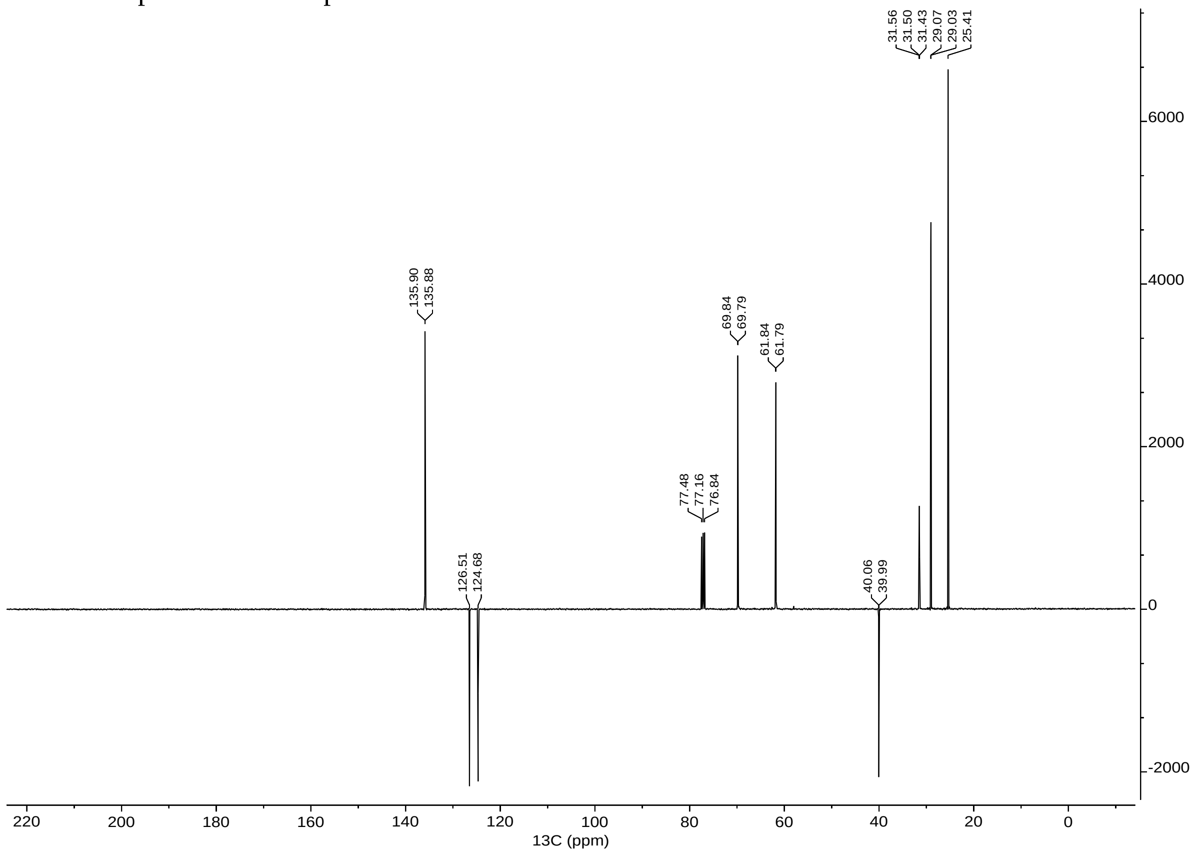

^{31}P{^{1}H} NMR spectrum of compound **S37**

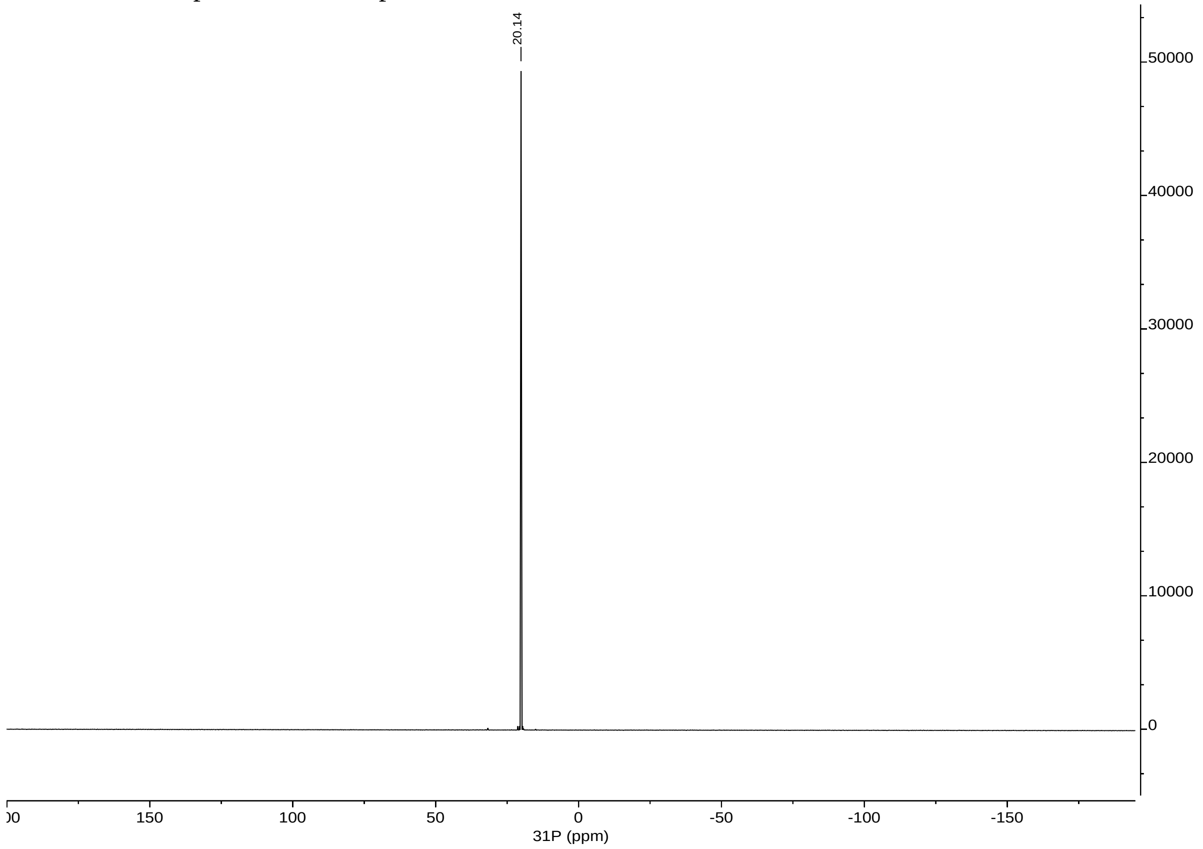

Bis((*Z*)-hept-3-en-1-yl) propane-1,3-diyl bis(vinylphosphonate) (S38)

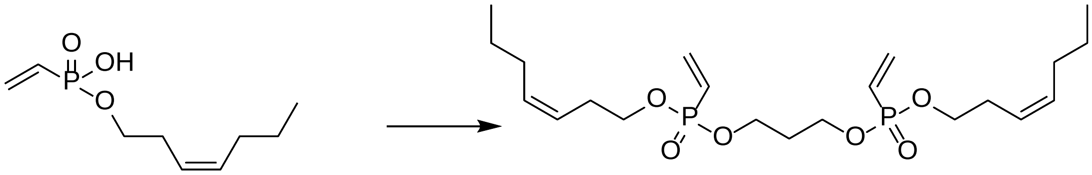

The title compound was prepared according to general method **C** from mono alkyl vinylphosphonate **S26** (1.10 g, 5.39 mmol) and 1,3-dibromopropane (0.36 g, 1.80 mmol) in 72% yield (0.61 g, 1.36 mmol) as a colourless oil.

^{1}H NMR (400 MHz, $CDCl_3$): 6.35–5.97 (m, 6H, C*H*=C*H*$_2$), 5.54–5.47 (m, 2H, $CH_3CH_2CH_2C\mathit{H}CHCH_2CH_2$), 5.38–5.31 (m, 2H, $CH_3CH_2CH_2CHC\mathit{H}CH_2CH_2O$), 4.12 (dt, 4H, *J* = 7.4, 6.0 Hz, $OC\mathit{H}_2CH_2C\mathit{H}_2O$), 4.00 (q, 4H, *J* = 7.1 Hz, $CH_3CH_2CH_2CHCHCH_2C\mathit{H}_2O$), 2.42 (qd, 4H, *J* = 7.1, 1.4 Hz, $CH_3CH_2CH_2CHCHC\mathit{H}_2CH_2O$), 2.06–1.98 (m, 6H, $CH_3CH_2C\mathit{H}_2CHCHCH_2CH_2O$, $OCH_2C\mathit{H}_2CH_2O$), 1.37 (h, 4H, *J* = 7.3 Hz, $CH_3C\mathit{H}_2CH_2CHCHCH_2CH_2O$), 0.98 (t, 6H, *J* = 7.4 Hz, $C\mathit{H}_3CH_2CH_2CHCHCH_2CH_2O$).

^{13}C NMR (101 MHz, $CDCl_3$): 136.07 (d, *J* = 2.1 Hz, CH=*C*H$_2$), 133.25 ($CH_3CH_2CH_2\mathit{C}HCHCH_2CH_2O$), 125.64 (d, *J* = 183.9 Hz, *C*H=CH$_2$), 123.96 ($CH_3CH_2CH_2CH\mathit{C}HCH_2CH_2O$), 65.52 (d, *J* = 6.3 Hz, $CH_3CH_2CH_2CHCHCH_2\mathit{C}H_2O$), 61.94 (d, *J* = 5.5 Hz, $O\mathit{C}H_2CH_2\mathit{C}H_2O$), 31.58 (t, *J* = 6.8 Hz, $OCH_2C\mathit{H}_2CH_2O$), 29.53 ($CH_3CH_2\mathit{C}H_2CHCHCH_2CH_2O$), 28.80 (d, *J* = 6.8 Hz, $CH_3CH_2CH_2CHCH\mathit{C}H_2CH_2O$), 22.79 ($CH_3C\mathit{H}_2CH_2CHCHCH_2CH_2O$), 13.88 ($C\mathit{H}_3CH_2CH_2CHCHCH_2CH_2O$).

^{31}P{^{1}H} NMR (162 MHz, $CDCl_3$): 20.06.

IR ν_{max} (KBr) 3086 (w), 3011 (m), 2958 (vs), 2931 (m), 2872 (m), 1656 (vw), 1613 (w), 1465 (m), 1398 (m), 1278 (m), 1251 (vs), 1060–1007 (vs), 984 (vs), 722 (m).

HR-MS(ESI$^+$): For $C_{21}H_{38}O_6NaP_2$ $(M+Na)^+$ *m*/*z* calculated 471.20358, found 471.20389.

^{1}H NMR spectrum of compound **S38**

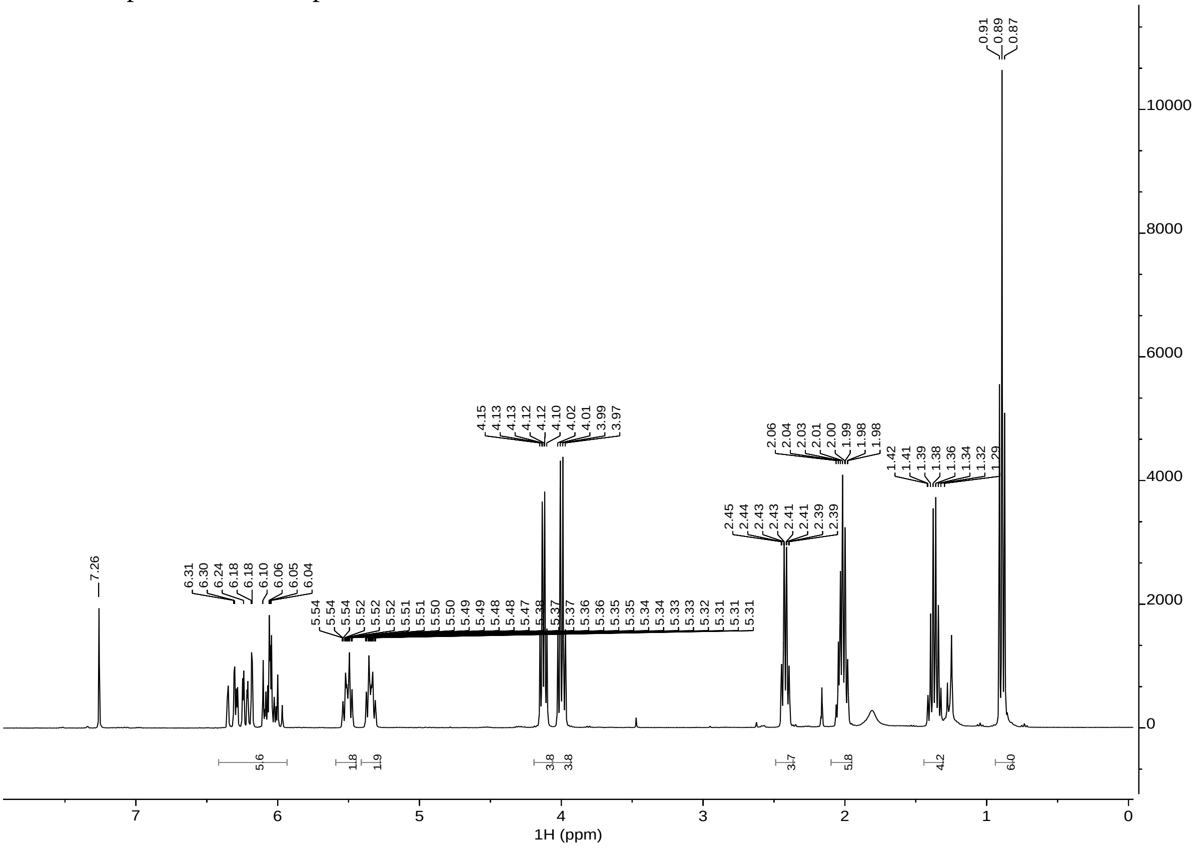

^{13}C NMR spectrum of compound **S38**

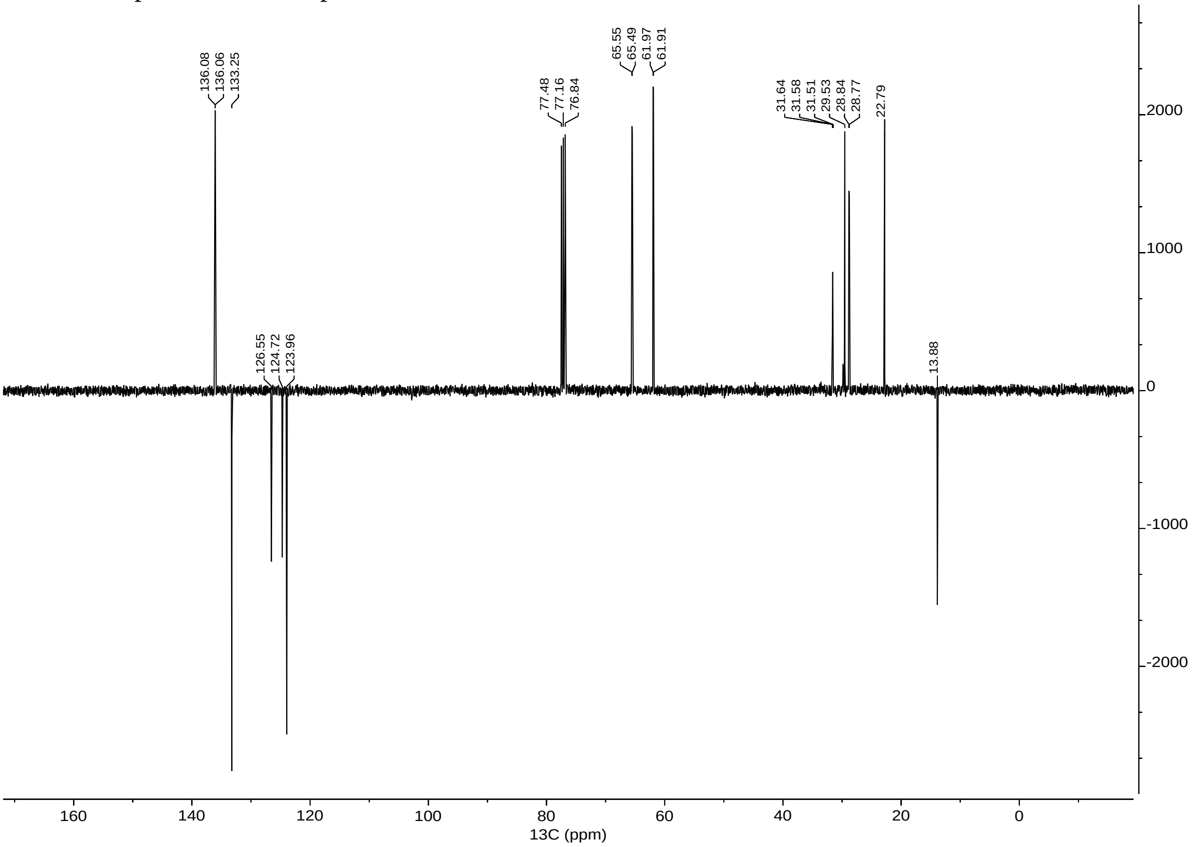

^{31}P{^{1}H} NMR spectrum of compound **S38**

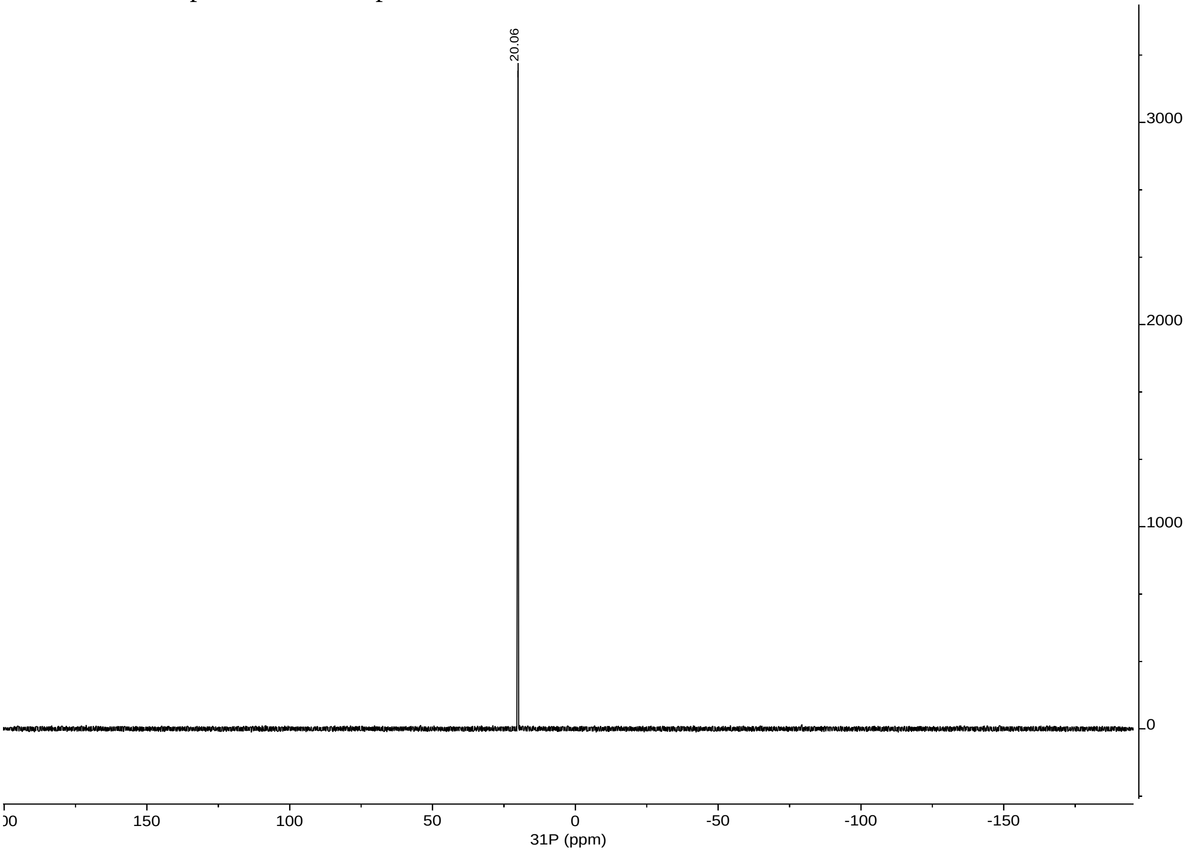

Bis((*Z*)-hept-4-en-1-yl) propane-1,3-diyl bis(vinylphosphonate) (S39)

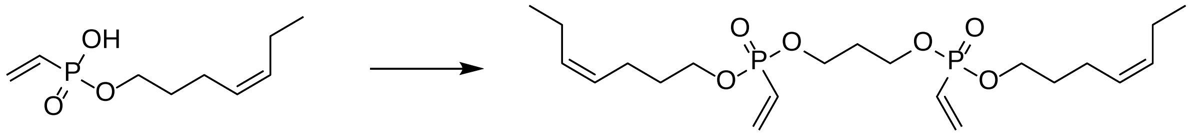

The title compound was prepared according to general method **C** from mono alkyl vinylphosphonate **S27** (0.70 g, 3.42 mmol) and 1,3-dibromopropane (0.23 g, 1.14 mmol) in 72% yield (0.37 g, 0.82 mmol) as a colourless oil.

^{1}H NMR (401 MHz, $CDCl_3$): 6.36–5.97 (m, 6H, $C\mathit{H}{=}C\mathit{H}_2$), 5.44–5.37 (m, 2H, $CH_3CH_2C\mathit{H}CHCH_2CH_2CH_2O$), 5.25–5.32 (m, 2H, $CH_3CH_2CHC\mathit{H}CH_2CH_2CH_2O$), 4.15–4.11 (m, 4H, $OC\mathit{H}_2CH_2C\mathit{H}_2O$), 4.04–3.99 (m, 4H, $CH_3CH_2CHCHCH_2CH_2C\mathit{H}_2O$), 2.08–2.14–2.08 (m, 4H, $CH_3CH_2CHCHC\mathit{H}_2CH_2CH_2O$), 2.07–1.98 (m, 6H, $CH_3C\mathit{H}_2CHCHCH_2CH_2CH_2O$, $OCH_2C\mathit{H}_2CH_2O$), 1.76–1.69 (m, 4H, $CH_3CH_2CHCHCH_2C\mathit{H}_2CH_2O$), 0.95 (t, 6H, J = 7.6 Hz, $C\mathit{H}_3CH_2CHCHCH_2CH_2CH_2O$).

^{13}C NMR (101 MHz, $CDCl_3$): 136.06 (d, J = 2.1 Hz, $CH{=}\mathit{C}H_2$), 133.05 ($CH_3CH_2\mathit{C}HCHCH_2CH_2CH_2O$), 127.42 ($CH_3CH_2CH\mathit{C}HCH_2CH_2CH_2O$), 125.65 (d, J = 183.1 Hz, $\mathit{C}H{=}CH_2$), 65.60 (d, J = 5.7 Hz, $CH_3CH_2CHCHCH_2CH_2\mathit{C}H_2O$), 61.91 (d, J = 5.5 Hz, $O\mathit{C}H_2CH_2\mathit{C}H_2O$), 31.60 (t, J = 6.6 Hz, $OCH_2\mathit{C}H_2CH_2O$), 30.62 (d, J = 6.7 Hz, $CH_3CH_2CHCHCH_2\mathit{C}H_2CH_2O$), 23.19 ($CH_3CH_2CHCH\mathit{C}H_2CH_2CH_2O$), 20.64 ($CH_3\mathit{C}H_2CHCHCH_2CH_2CH_2O$), 14.43 ($\mathit{C}H_3CH_2CHCHCH_2CH_2CH_2O$).

^{31}P{^{1}H} NMR (162 MHz, $CDCl_3$): 20.35.

IR ν_{max} (KBr) 3091 (w), 2966 (s), 2935 (m), 2875 (m), 1655 (vw), 1464 (m), 1456 (m), 1405 (sh), 1279 (m), 1243 (s), 1052 (s), 1033 (s), 1020 (vs), 986 (vs).

HR-MS(ESI$^+$): For $C_{21}H_{39}O_6P_2$ $(M+H)^+$ m/z calculated 449.22164, found 449.22140.

^{1}H NMR spectrum of compound **S39**

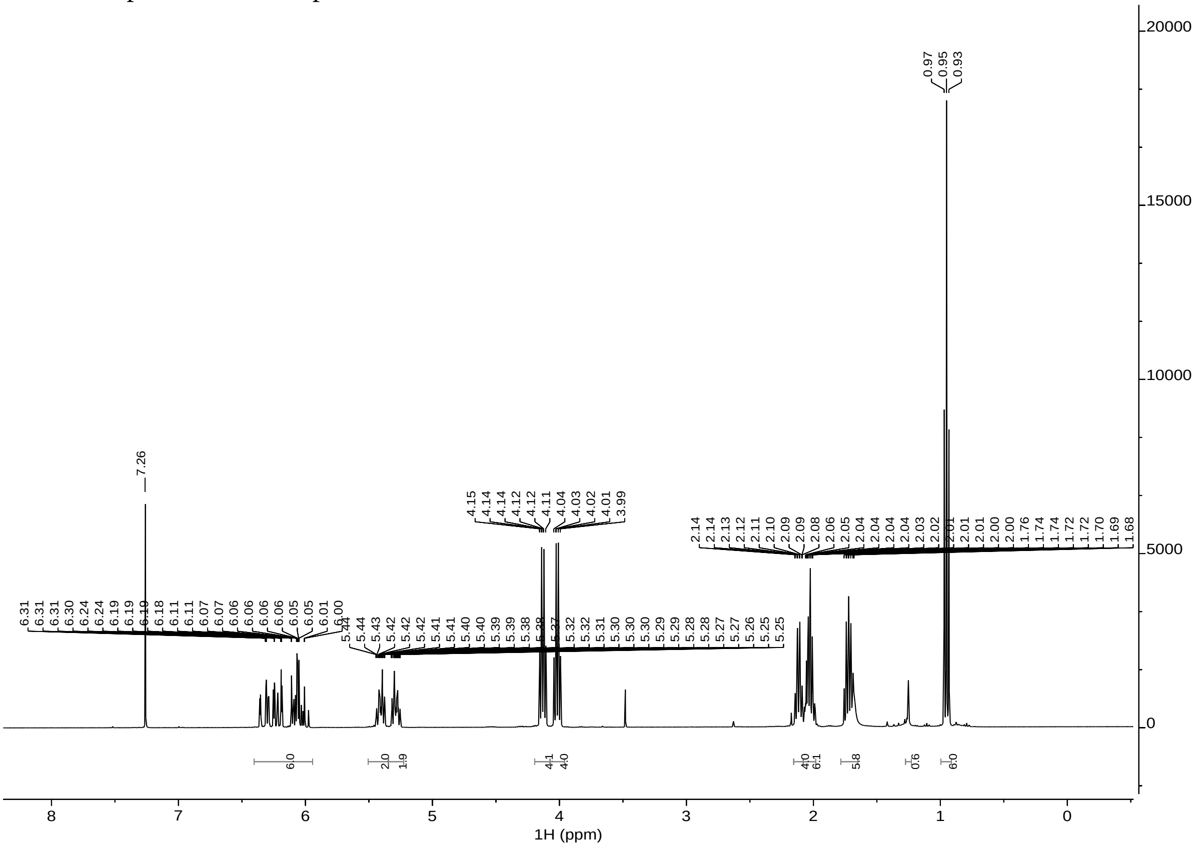

^{13}C NMR spectrum of compound **S39**

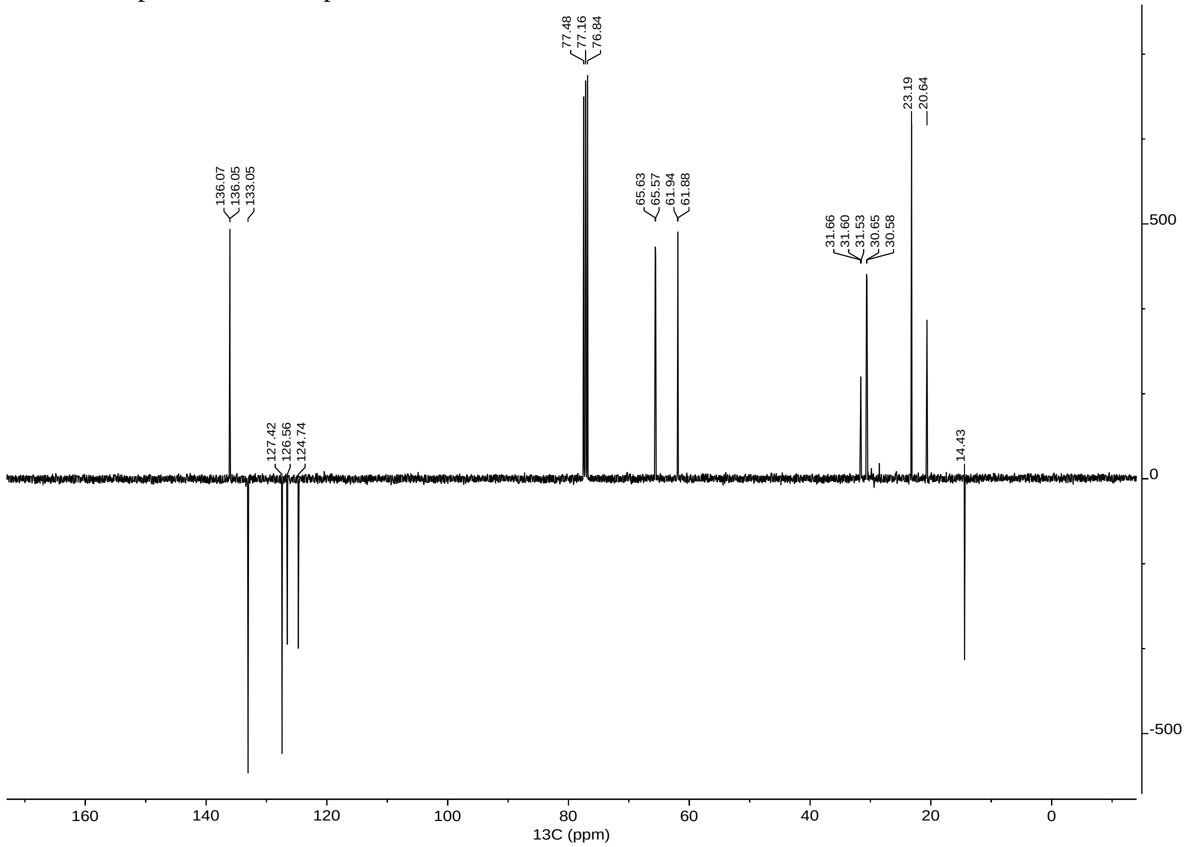

^{31}P{^{1}H} NMR spectrum of compound **S39**

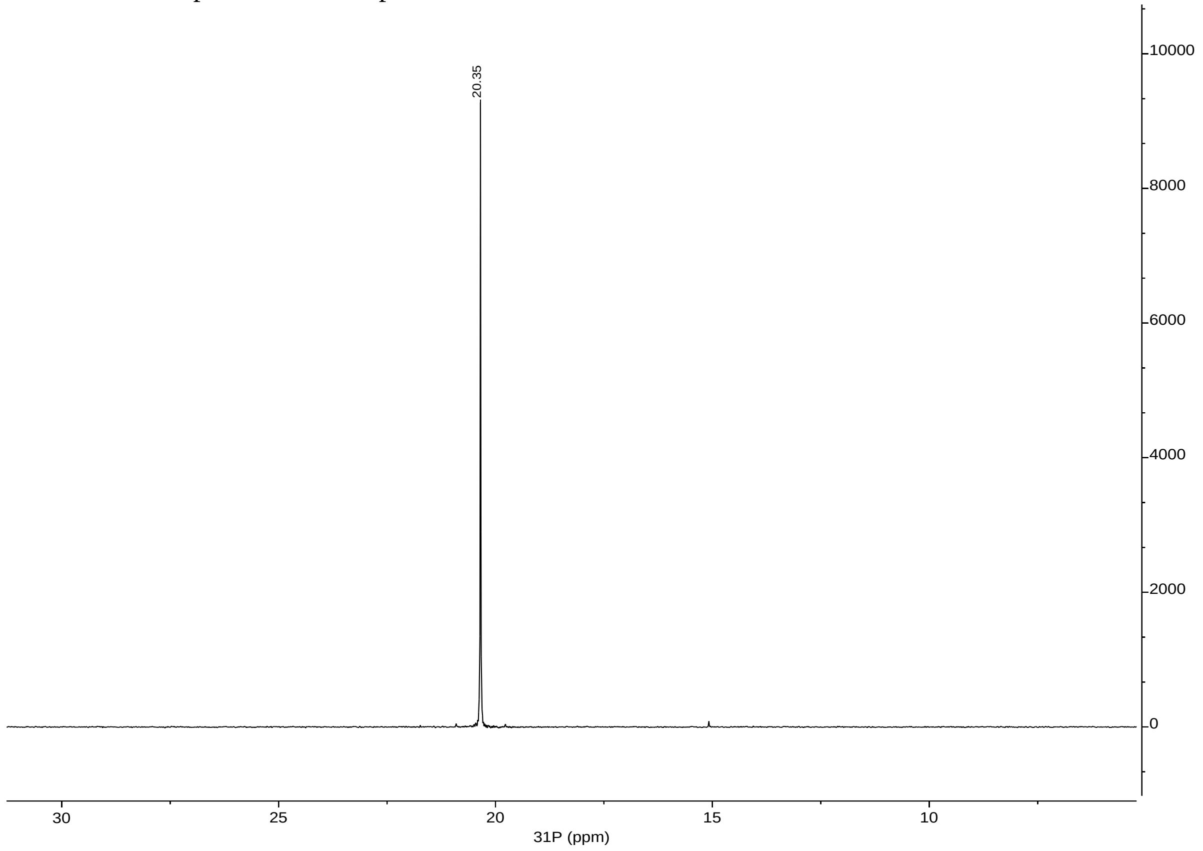

Dioctyl propane-1,3-diyl bis(vinylphosphonate) (S40)

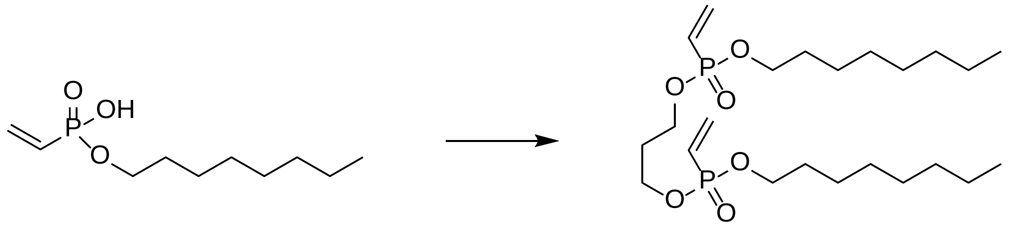

The title compound was prepared according to general method **C** from mono alkyl vinylphosphonate **S28** (1.10 g, 5.01 mmol) and 1,3-dibromopropane (0.34 g, 1.67 mmol) in 74% yield (0.59 g, 1.23 mmol) as a colourless oil.

^{1}H NMR (401 MHz, $CDCl_3$): 6.35–5.97 (m, 6H, C*H*=C*H*$_2$), 4.13 (dt, *J* = 7.2, 6.1 Hz, 4H, OC*H*$_2$CH$_2$C*H*$_2$O), 4.00 (q, *J* = 6.8 Hz, 4H, CH$_3$(CH$_2$)$_5$CH$_2$C*H*$_2$O), 2.07–2.00 (m, 2H, OCH$_2$C*H*$_2$CH$_2$O), 1.68–1.62 (m, 4H, CH$_3$(CH$_2$)$_5$C*H*$_2$CH$_2$O), 1.38–1.24 (m, 20H, CH$_3$(C*H*$_2$)$_5$CH$_2$CH$_2$O), 0.89–0.86 (m, 6H, C*H*$_3$(CH$_2$)$_6$CH$_2$O).

^{13}C NMR (101 MHz, $CDCl_3$): 135.85 (d, *J* = 2.1 Hz, CH=*C*H$_2$), 125.70 (d, *J* = 183.7 Hz, *C*H=CH$_2$), 66.25 (d, *J* = 5.8 Hz, CH$_3$(CH$_2$)$_5$CH$_2$*C*H$_2$O), 61.90 (d, *J* = 5.6 Hz, O*C*H$_2$CH$_2$*C*H$_2$O), 31.59 (t, *J* = 6.4 Hz, OCH$_2$*C*H$_2$CH$_2$O), 30.60 (d, *J* = 6.4 Hz, CH$_3$(CH$_2$)$_5$*C*H$_2$CH$_2$O), 31.91, 29.31, 29.26, 25.64, 22.77 (CH$_3$(*C*H$_2$)$_5$CH$_2$CH$_2$O), 14.23 (*C*H$_3$(CH$_2$)$_6$CH$_2$O).

^{31}P{^{1}H} NMR (162 MHz, $CDCl_3$): 20.30.

IR ν_{max} (KBr) 3091 (vw), 2959 (s), 2929 (s), 2872 (m), 2858 (m), 1468 (w), 1400 (w), 1279 (w), 1242 (s), 1049 (s), 1019 (vs), 986 (vs), 606 (w).

HR-MS(ESI$^+$): For $C_{23}H_{46}O_6NaP_2$ (M+Na)$^+$ *m/z* calculated 503.26618, found 503.26597.

^{1}H NMR spectrum of compound **S40**

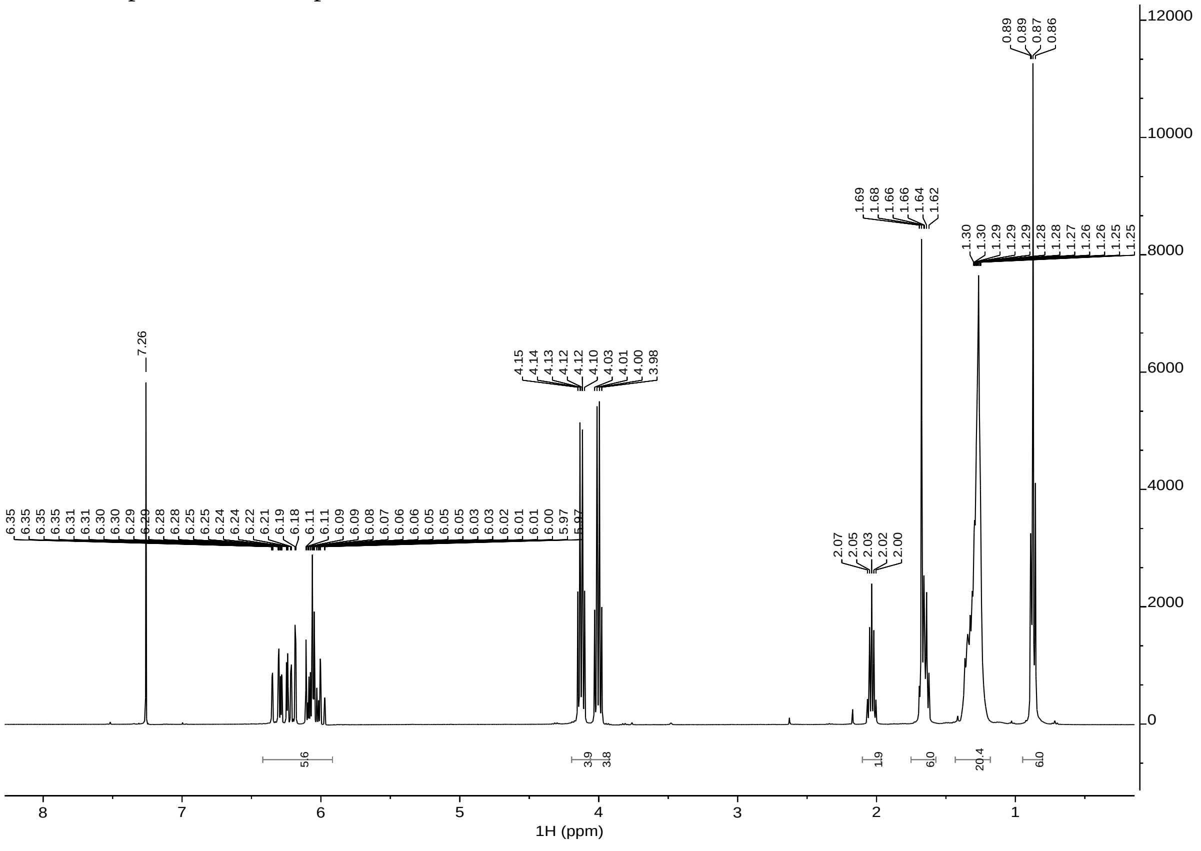

^{13}C NMR spectrum of compound **S40**

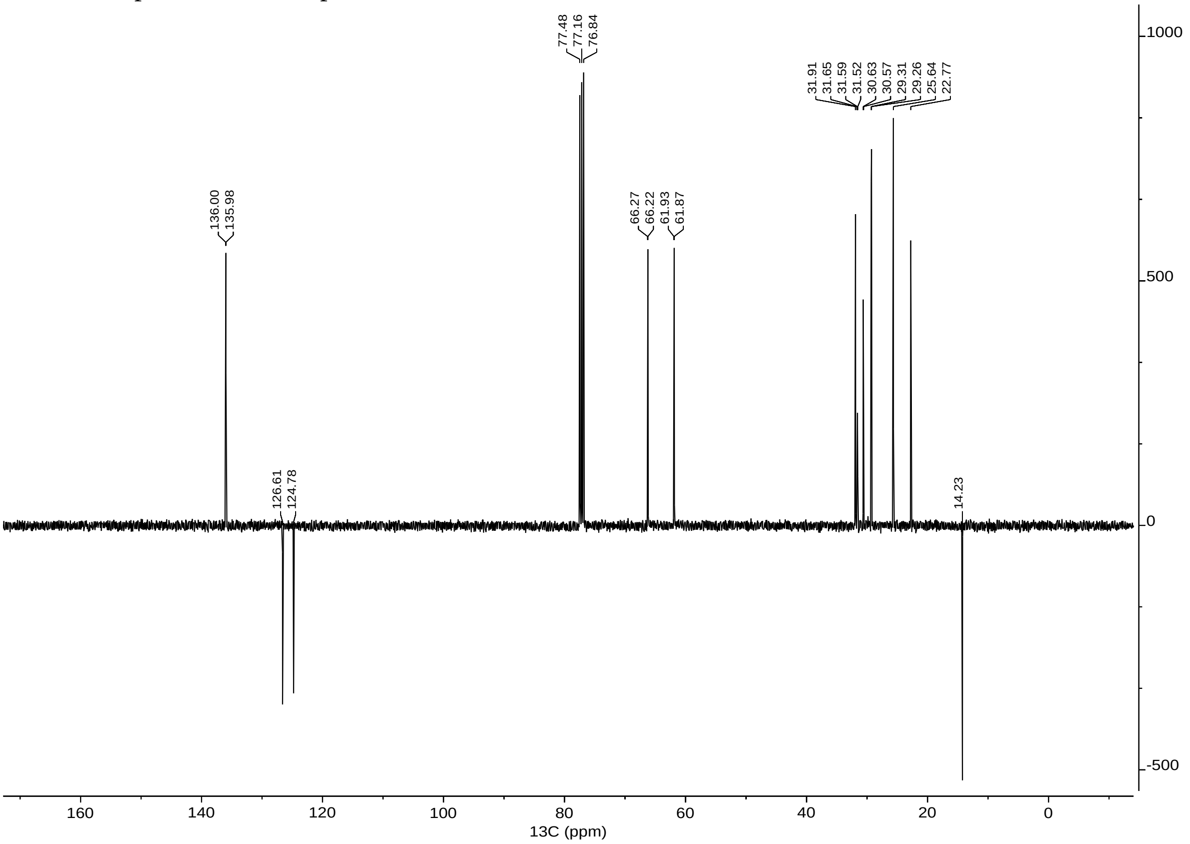

$^{31}P\{^{1}H\}$ NMR spectrum of compound **S40**

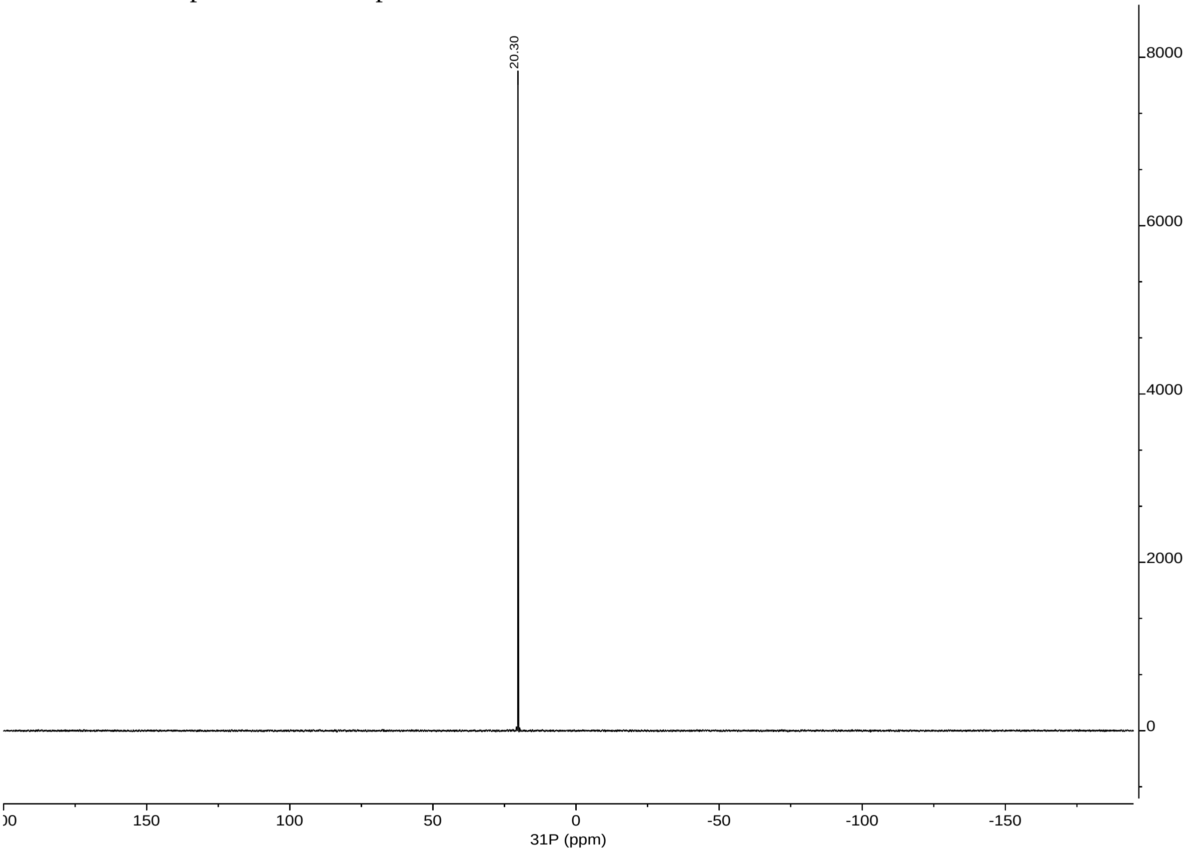

Bis((*Z*)-oct-3-en-1-yl) propane-1,3-diyl bis(vinylphosphonate) (S41)

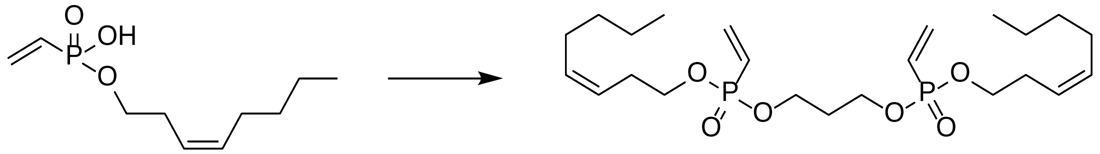

The title compound was prepared according to general method **C** from mono alkyl vinylphosphonate **S30** (0.9 g, 4.12 mmol) and 1,3-dibromopropane (0.28 g, 1.37 mmol) in 73% yield (0.48 g, 1.01 mmol) as a colourless oil.

^{1}H NMR (400 MHz, $CDCl_3$): 6.35–5.97 (m, 6H, $C\mathit{H}{=}C\mathit{H}_2$), 5.54–5.47 (m, 2H, $CH_3(CH_2)_3C\mathit{H}CHCH_2CH_2O$), 5.36–5.29 (m, 2H, $CH_3(CH_2)_3CHC\mathit{H}CH_2CH_2O$), 4.15–4.10 (m, 4H, $OC\mathit{H}_2CH_2C\mathit{H}_2O$), 3.99 (q, 4H, J = 7.1 Hz, $CH_3(CH_2)_2CH_2CHCHCH_2C\mathit{H}_2O$), 2.44–2.34 (m, 4H, $CH_3(CH_2)_2CH_2CHCHC\mathit{H}_2CH_2O$), 2.06–2.00 (m, 6H, $CH_3(CH_2)_2C\mathit{H}_2CHCHCH_2CH_2O$, $OCH_2C\mathit{H}_2CH_2O$), 1.36–1.24 (m, 8H, $CH_3(C\mathit{H}_2)_2CH_2CHCHCH_2CH_2O$), 0.92–0.85 (m, 6H, $C\mathit{H}_3(CH_2)_3CHCHCH_2CH_2O$).

^{13}C NMR (101 MHz, $CDCl_3$): 136.06 (d, J = 1.8 Hz, $CH{=}\mathit{C}H_2$), 133.46 ($CH_3(CH_2)_3\mathit{C}HCHCH_2CH_2O$), 125.64 (d, J = 184.1 Hz, $\mathit{C}H{=}CH_2$), 123.74 ($CH_3(CH_2)_3CH\mathit{C}HCH_2CH_2O$), 65.52 (d, J = 5.8 Hz, $CH_3(CH_2)_3CHCHCH_2\mathit{C}H_2O$), 61.94 (d, J = 5.6 Hz, $O\mathit{C}H_2CH_2\mathit{C}H_2O$), 31.83 ($CH_3CH_2\mathit{C}H_2CH_2CHCHCH_2CH_2O$), 31.57 (t, J = 6.8 Hz, $OCH_2\mathit{C}H_2CH_2O$), 28.78 (d, J = 6.7 Hz, $CH_3(CH_2)_2CH_2CHCH\mathit{C}H_2CH_2O$), 27.20 $CH_3(CH_2)_2\mathit{C}H_2CHCHCH_2CH_2O$), 22.44 ($CH_3\mathit{C}H_2CH_2CH_2CHCHCH_2CH_2O$), 14.09 ($\mathit{C}H_3(CH_2)_3CHCHCH_2CH_2O$).

^{31}P{^{1}H} NMR (162 MHz, $CDCl_3$): 20.07.

IR ν_{max} (KBr) 3086 (w), 3011 (w), 2958 (m), 2928 (m), 2872 (w), 2859 (w), 1653 (w), 1613 (w), 1467 (w), 1398 (w), 1278 (m), 1252 (s), 1051 (s), 1011 (vs), 977 (s), 857 (m), 608 (m).

HR-MS(APCI$^+$): For $C_{23}H_{43}O_6P_2$ $(M+H)^+$ m/z calculated 477.25294, found 477.25303.

^{1}H NMR spectrum of compound **S41**

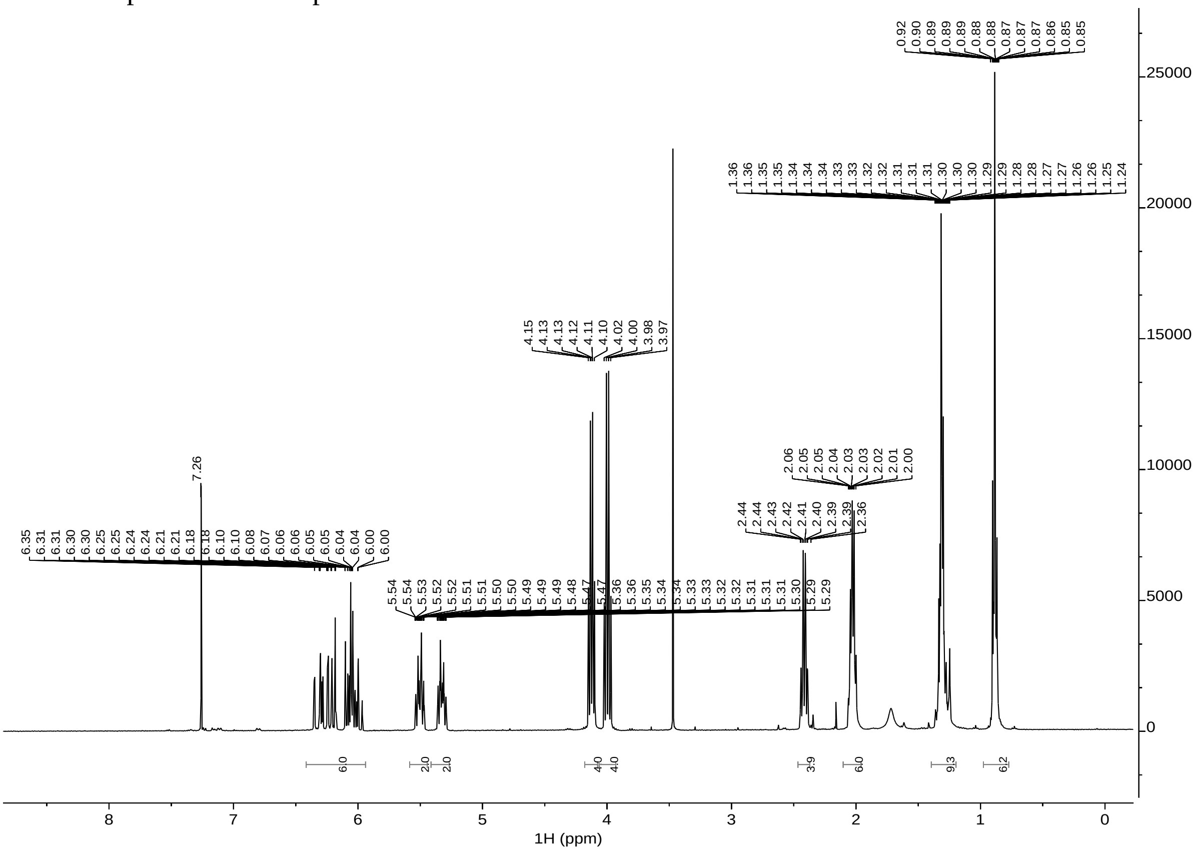

^{13}C NMR spectrum of compound **S41**

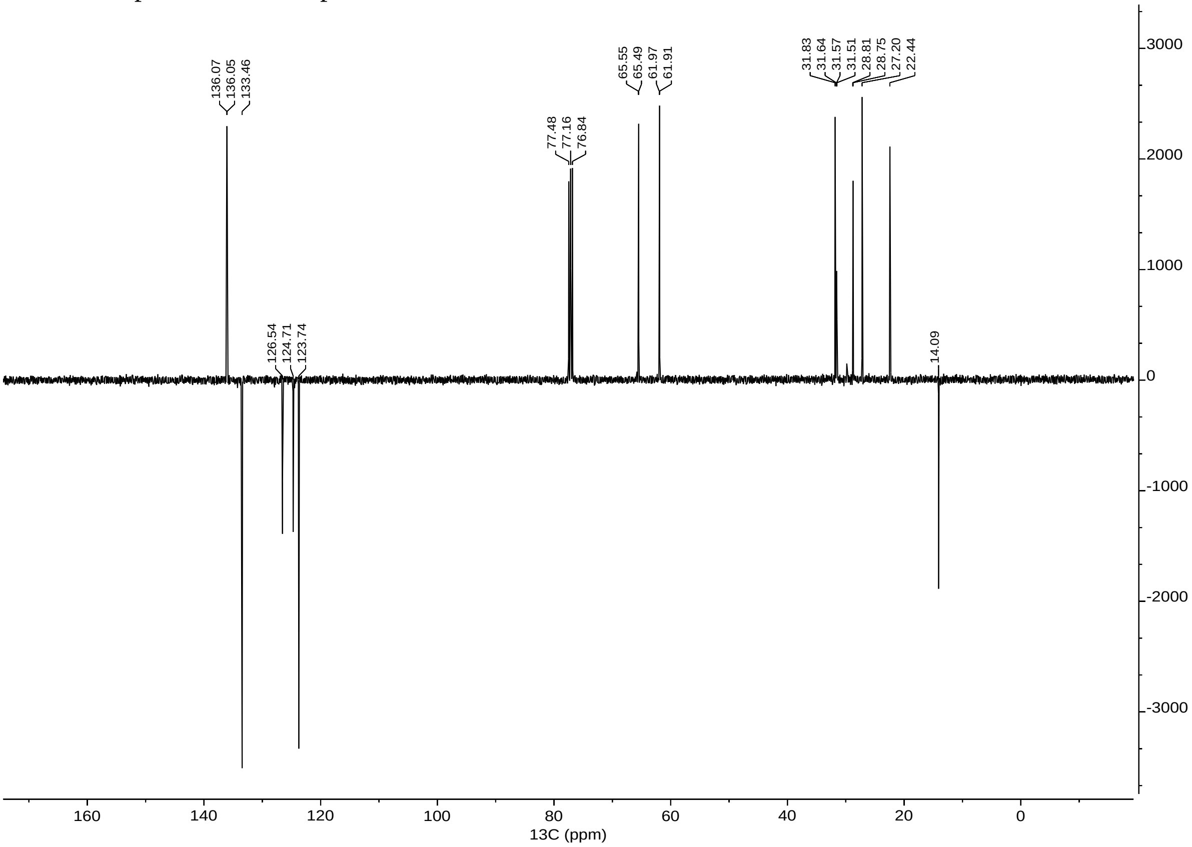

$^{31}P\{^1H\}$ NMR spectrum of compound **S41**

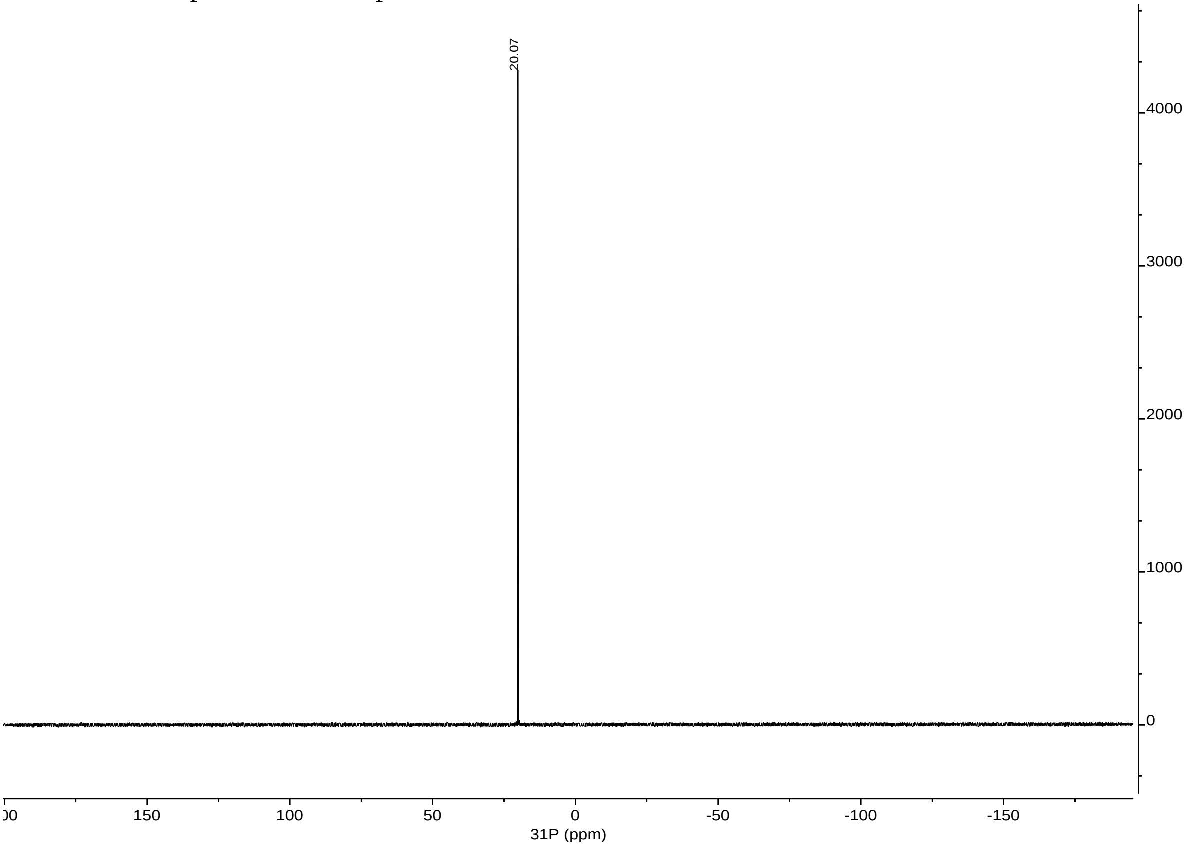

Bis((*Z*)-non-3-en-1-yl) propane-1,3-diyl bis(vinylphosphonate) (S42)

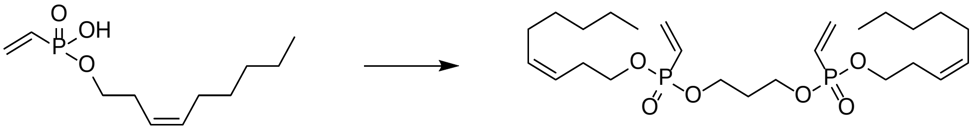

The title compound was prepared according to general method **C** from mono alkyl vinylphosphonate **S32** (1.50 g, 6.45 mmol) and 1,3-dibromopropane (0.43 g, 2.15 mmol) in 70% yield (0.76 g, 1.51 mmol) as a colourless oil.

^{1}H NMR (401 MHz, $CDCl_3$): 6.38–5.97 (m, 6H, C*H*=C*H*$_2$), 5.55–5.47 (m, 1H, $CH_3(CH_2)_4$C*H*$CHCH_2CH_2O$), 5.36–5.29 (m, 2H, $CH_3(CH_2)_4$CHC*H*CH_2CH_2O), 4.13 (dt, 4H, *J* = 7.3, 6.1 Hz, OC*H*$_2$$CH_2$C*H*$_2$O), 4.00 (q, 4H, *J* = 7.1 Hz, $CH_3(CH_2)_4CHCHCH_2$C*H*$_2$O), 2.42 (qd, 4H, *J* = 7.0, 1.5 Hz, $CH_3(CH_2)_4$CHCHC*H*$_2$$CH_2O$), 2.06–1.99 (m, 6H, $CH_3(CH_2)_3$C*H*$_2$$CHCHCH_2CH_2O$, OCH_2C*H*$_2$$CH_2O$), 1.21–1.38 (m, 12H, CH_3(C*H*$_2$)$_3$$CH_2CHCHCH_2CH_2O$), 0.86–0.90–0.86 (m, 6H, C*H*$_3$**(**$CH_2)_3CHCHCH_2CH_2CH_2O$).

^{13}C NMR (101 MHz, $CDCl_3$): 136.10 (d, *J* = 1.9 Hz, CH=*C*H$_2$), 133.53 ($CH_3(CH_2)_4$*C*$HCHCH_2CH_2O$), 125.62 (d, *J* = 184.1 Hz, *C*H=CH$_2$), 123.72 ($CH_3(CH_2)_3$CH*C*HCH_2CH_2O), 65.53 (d, *J* = 6.0 Hz, $CH_3(CH_2)_4CHCHCH_2$*C*H$_2$O), 61.94 (d, *J* = 5.6 Hz, O*C*H$_2$$CH_2$*C*H$_2$O), 31.61 ($CH_3CH_2$*C*H$_2$$CH_2CH_2CHCHCH_2CH_2O$), 31.57 (d, *J* = 6.4 Hz, OCH_2*C*H$_2$$CH_2O$), 29.35 ($CH_3CH_2CH_2$*C*H$_2$$CH_2CHCHCH_2CH_2O$), 28.79 (d, *J* = 6.4 Hz, $CH_3(CH_2)_4$CHCH*C*H$_2$$CH_2O$), 27.48 ($CH_3(CH_2)_3$*C*H$_2$$CHCHCH_2CH_2O$), 22.69 ($CH_3$*C*H$_2$$CH_2CH_2CH_2CHCHCH_2CH_2O$), 14.20 (*C*H$_3$$(CH_2)_4CHCHCH_2CH_2O$).

^{31}P{^{1}H} NMR (162 MHz, $CDCl_3$): 20.32.

IR ν_{max} (KBr) 3091 (vw), 2960 (s), 2930 (s), 2873 (m), 2858 (m), 1654 (vw), 1614 (w), 1468 (m), 1460 (w, sh), 1441 (vw), 1407 (m, sh), 1380 (w), 1279 (m), 1242 (s), 1050 (s), 1012 (vs), 986 (vs).

HR-MS(ESI^{+}): For $C_{25}H_{47}O_6P_2$ $(M+H)^{+}$ *m/z* calculated 505.28424, found 505.28430.

^{1}H NMR spectrum of compound **S42**

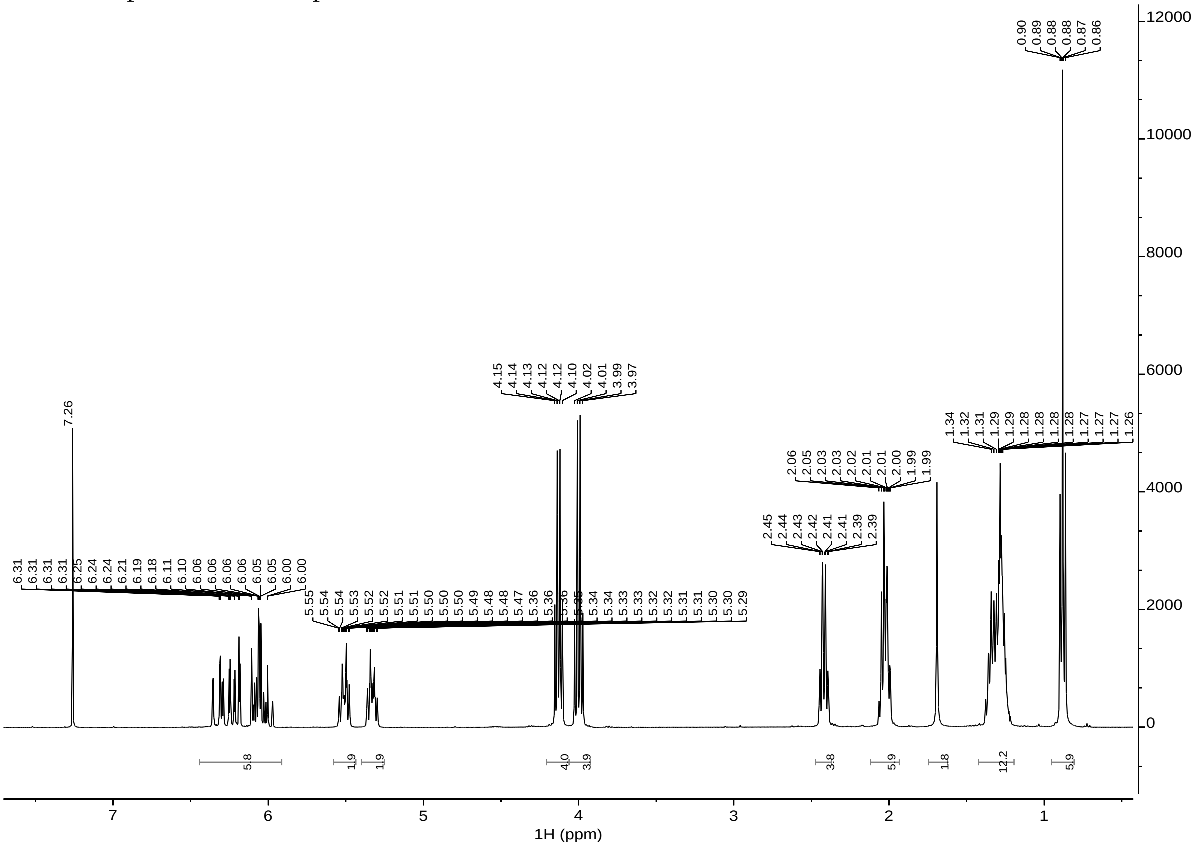

^{13}C NMR spectrum of compound **S42**

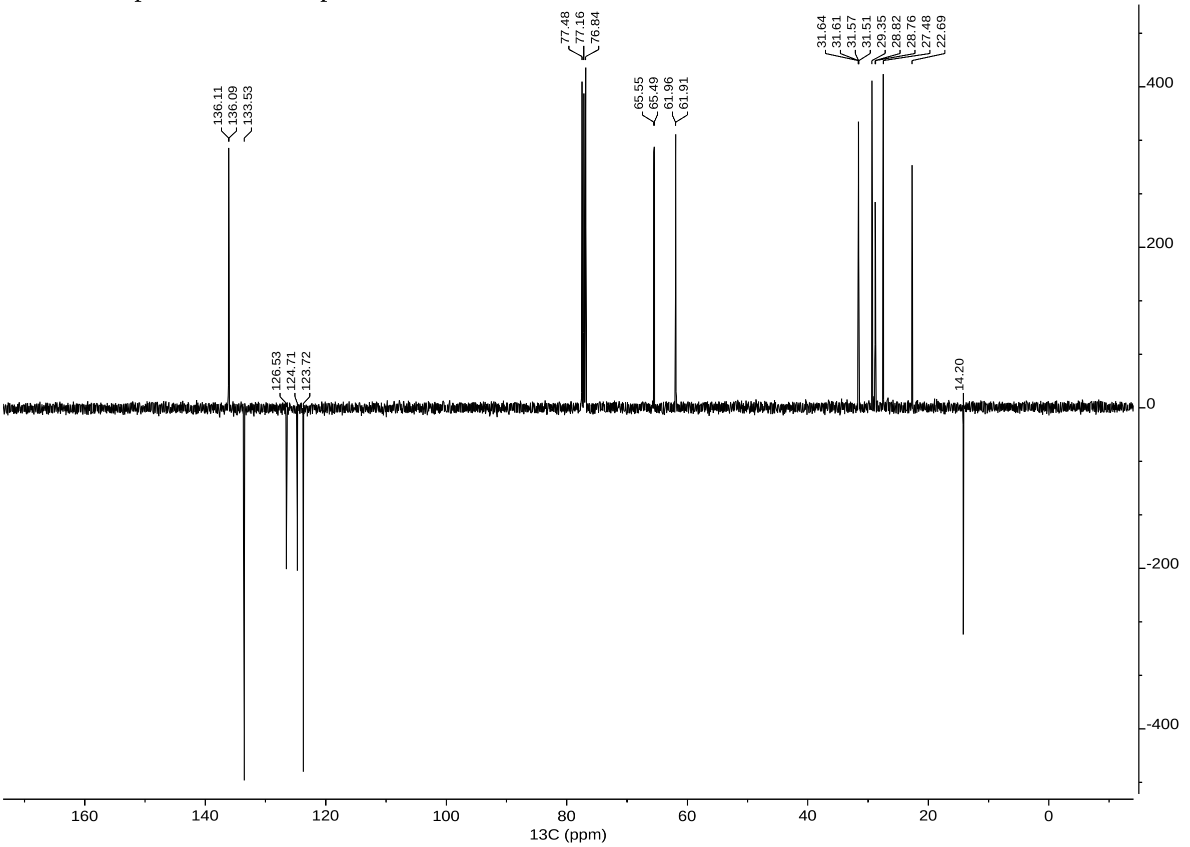

^{31}P{^{1}H} NMR spectrum of compound **S42**

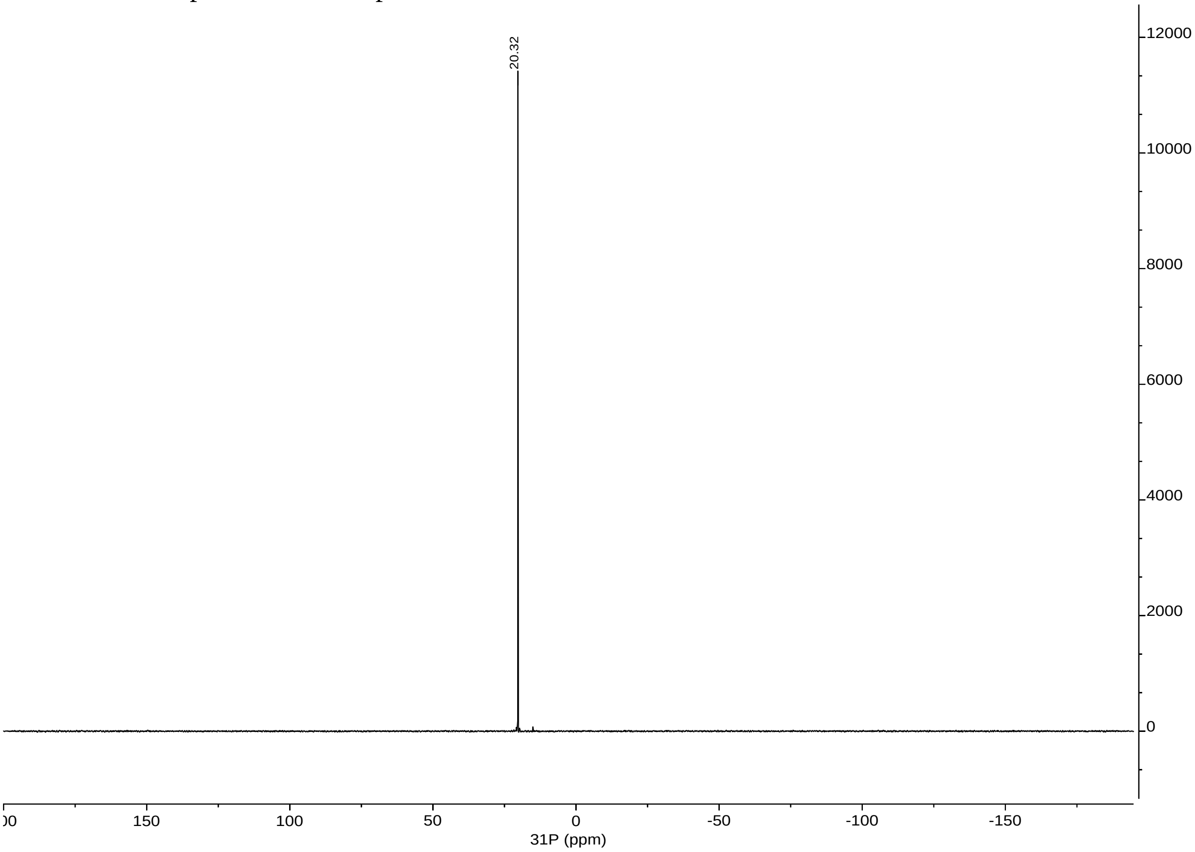

Didecyl propane-1,3-diyl bis(vinylphosphonate) (S43)

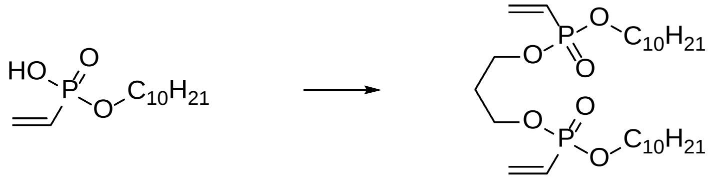

The title compound was prepared according to general method **C** from mono alkyl vinylphosphonate **S33** (3.7 g, 15.0 mmol) and 1,3-dibromopropane (0.51 mL, 5.0 mmol) in 41% yield (1.10 g, 2.05 mmol) as a colourless oil.

^{1}H NMR (401 MHz, $CDCl_3$) δ 6.35–5.95 (m, 6H, C*H*=C*H*$_2$), 4.11 (dt, *J* = 7.3, 6.1 Hz, 4H, OC*H*$_2$CH$_2$C*H*$_2$O), 3.99 (q, *J* = 6.8 Hz, 4H, OC*H*$_2$(CH$_2$)$_8$CH$_3$), 2.02 (p, *J* = 6.1 Hz, 2H, OCH$_2$C*H*$_2$CH$_2$O), 1.69–1.60 (m, 4H, C*H*$_2$(CH$_2$)$_7$CH$_3$), 1.38–1.16 (m, 28H, (C*H*$_2$)$_7$CH$_3$), 0.90–0.82 (m, 6H, C*H*$_3$).

^{13}C NMR (101 MHz, $CDCl_3$) δ 135.98 (d, *J* = 2.1 Hz, =*C*H$_2$), 125.66 (d, *J* = 184.0 Hz, P*C*H), 66.22 (d, *J* = 5.8 Hz, O*C*H$_2$(CH$_2$)$_8$CH$_3$), 61.87 (d, *J* = 5.5 Hz, O*C*H$_2$CH$_2$*C*H$_2$O), 31.99 (*C*H$_2$CH$_2$CH$_3$), 31.55 (t, *J* = 6.6 Hz, OCH$_2$*C*H$_2$CH$_2$O), 30.57 (d, *J* = 6.4 Hz, *C*H$_2$(CH$_2$)$_7$CH$_3$), 29.63, 29.62, 29.40, 29.27, 25.61 (O(CH$_2$)$_2$(*C*H$_2$)$_5$), 22.78 (*C*H$_2$CH$_3$), 14.22 (*C*H$_3$).

^{31}P{^{1}H} NMR (162 MHz, $CDCl_3$) δ 20.26.

IR ν_{max} ($CHCl_3$) 3091 (w), 2958 (s), 2928 (vs), 2871 (m), 2856 (s), 1614 (w), 1468 (w), 1400 (w), 1379 (w), 1279 (w), 1243 (s), 1052 (s), 1019 (s), 987 (vs).

HR-MS(ESI$^+$): For $C_{27}H_{55}O_6P_2$ $(M+H)^+$ *m/z* calculated 537.34684, found 537.34631. For $C_{27}H_{54}O_6NaP_2$ $(M+Na)^+$ *m/z* calculated 559.32878, found 559.32841.

^{1}H NMR spectrum of compound **S43**

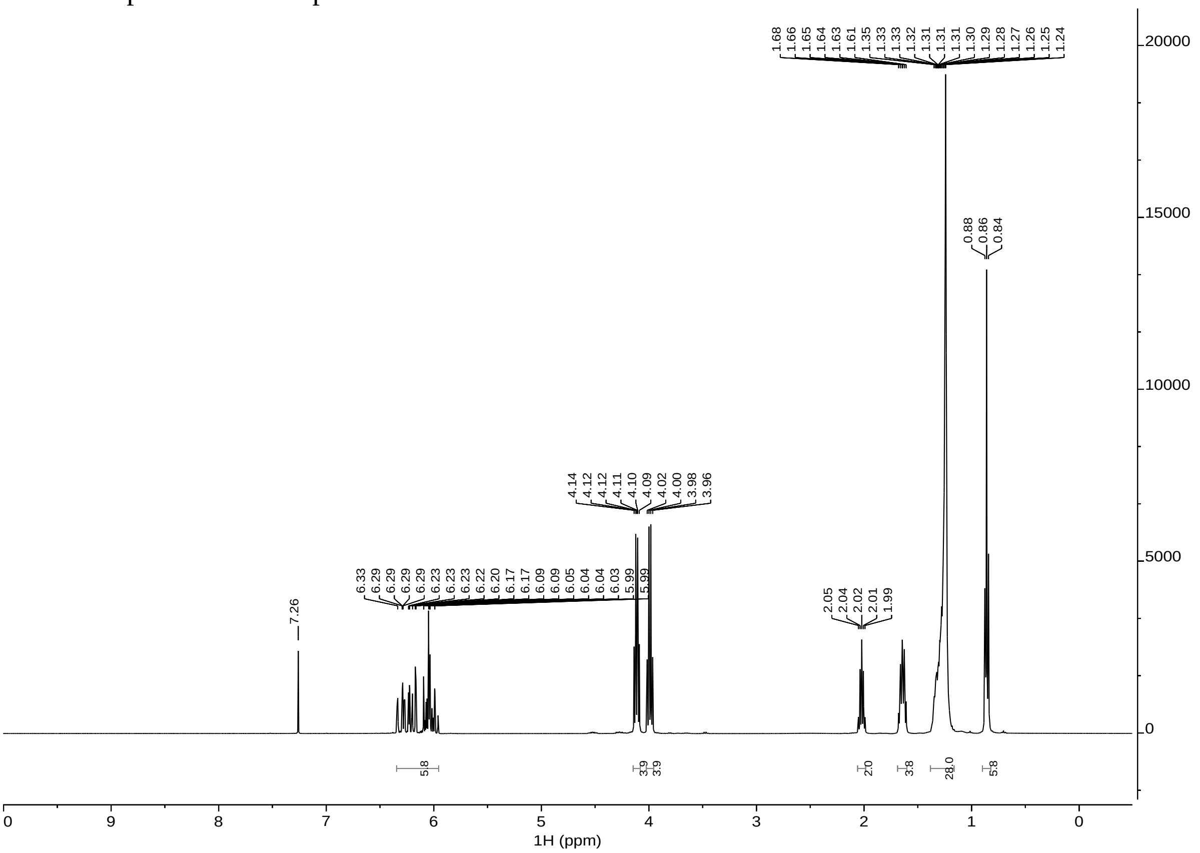

^{13}C NMR spectrum of compound **S43**

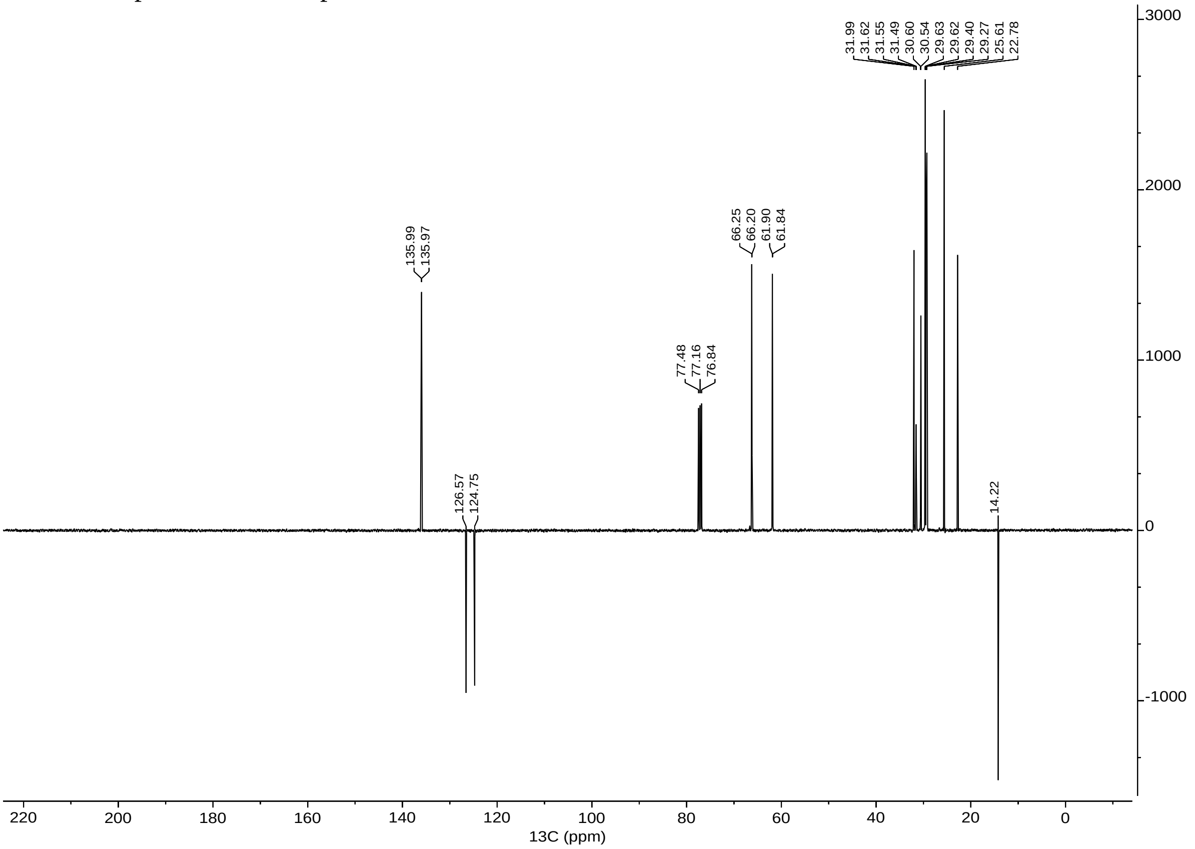

$^{31}P\{^{1}H\}$ NMR spectrum of compound **S43**

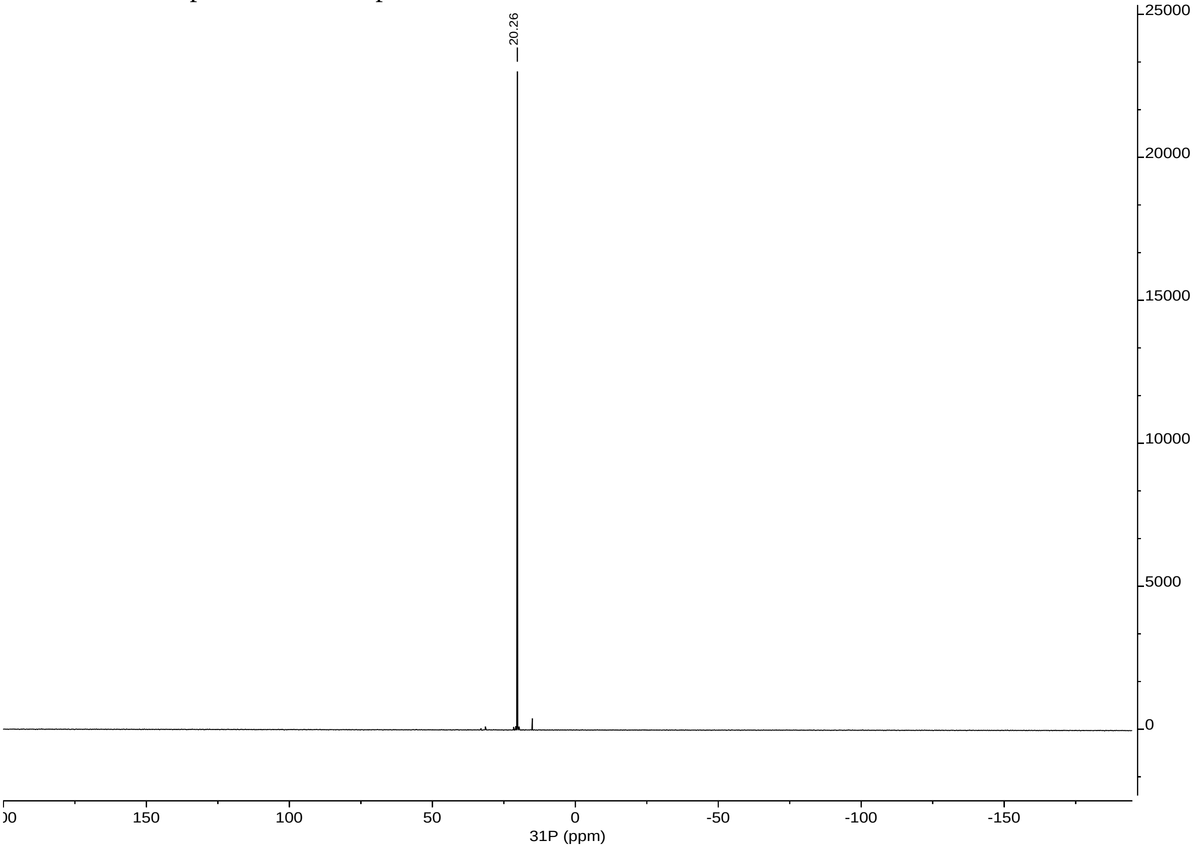

Bis((*Z*)-dec-4-en-1-yl) propane-1,3-diyl bis(vinylphosphonate) (S44)

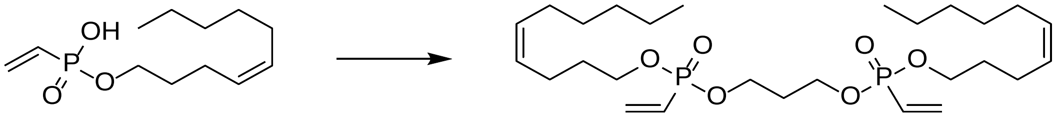

The title compound was prepared according to general method **C** from mono alkyl vinylphosphonate **S34** (1.25 g, 5.07 mmol) and 1,3-dibromopropane (0.34 g, 1.69 mmol) in 52% yield (0.47 g, 0.88 mmol) as a colourless oil.

^{1}H NMR (401 MHz, $CDCl_3$): 6.36–5.97 (m, 6H, C*H*=C*H*$_2$), 5.44–5.37 (m, 1H, CHC*H*(CH_2)$_3$O), 5.35–5.28 (m, 2H, CH_3(CH_2)$_4$C*H*), 4.13 (dt, 4H, *J* = 7.3, 6.1 Hz, OC*H*$_2$CH$_2$C*H*$_2$O), 4.02 (q, 4H, *J* = 6.8 Hz, CH_3(CH_2)$_4$CHCHCH$_2$CH$_2$C*H*$_2$O), 2.14–1.95 (m, 10H, CH_3(CH_2)$_3$C*H*$_2$, CHC*H*$_2$(CH_2)$_2$O, OCH$_2$C*H*$_2$CH$_2$O), 1.75–1.67 (m, 4H, CH_3(CH_2)$_4$CHCHCH$_2$C*H*$_2$CH$_2$O), 1.37–1.21 (m, 12H, CH_3(C*H*$_2$)$_3$CH$_2$CHCHCH$_2$CH$_2$CH$_2$O), 0.90–0.86 (m, 6H, C*H*$_3$(CH_2)$_4$CHCHCH$_2$CH$_2$CH$_2$O).

^{13}C NMR (101 MHz, $CDCl_3$): 1356.05 (d, *J* = 2.2 Hz, CH=*C*H$_2$), 131.46 (CH_3(CH_2)$_4$*C*HCHCH$_2$CH$_2$CH$_2$O), 127.96 (CH_3(CH_2)$_4$CH*C*HCH$_2$CH$_2$CH$_2$O), 127.96 (CH_3(CH_2)$_4$CH*C*HCH$_2$CH$_2$CH$_2$O), 125.65 (d, *J* = 183.1 Hz, *C*H=CH$_2$), 65.65 (d, *J* = 6.1 Hz, CH_3(CH_2)$_4$CHCHCH$_2$CH$_2$*C*H$_2$O), 61.92 (d, *J* = 5.2 Hz, O*C*H$_2$CH$_2$*C*H$_2$O), 31.67 (CH_3CH$_2$*C*H$_2$CH$_2$CH$_2$CHCHCH$_2$CH$_2$CH$_2$O), 31.60 (d, *J* = 6.5 Hz, OCH$_2$*C*H$_2$CH$_2$O), 30.64 (d, *J* = 6.7 Hz, CH_3(CH_2)$_4$CHCHCH$_2$*C*H$_2$CH$_2$O), 29.46 (CH_3CH$_2$CH$_2$*C*H$_2$CH$_2$CHCHCH$_2$CH$_2$O), 27.32 CH_3(CH_2)$_3$*C*H$_2$CHCHCH$_2$CH$_2$CH$_2$O), 23.31 (CH_3(CH_2)$_4$CHCH*C*H$_2$CH$_2$CH$_2$O), 22.71 (CH_3*C*H$_2$CH$_2$CH$_2$CH$_2$CHCHCH$_2$CH$_2$CH$_2$O), 14.22 (*C*H$_3$(CH_2)$_4$CHCHCH$_2$CH$_2$CH$_2$O).

^{31}P{^{1}H} NMR (162 MHz, $CDCl_3$): 20.34.

IR ν_{max} (KBr) 3091 (vw), 2960 (s), 2929 (s), 2873 (m), 2858 (m), 1654 (vw), 1614 (m), 1468 (m), 1457 (m), 1405 (m, sh), 1379 (w), 1279 (m), 1242 (s), 1057 (s), 1019 (vs), 986 (vs).

HR-MS(ESI$^+$): For $C_{27}H_{51}O_6P_2$ (M+H)$^+$ *m*/*z* calculated 533.31551, found 533.31541.

^{1}H NMR spectrum of compound **S44**

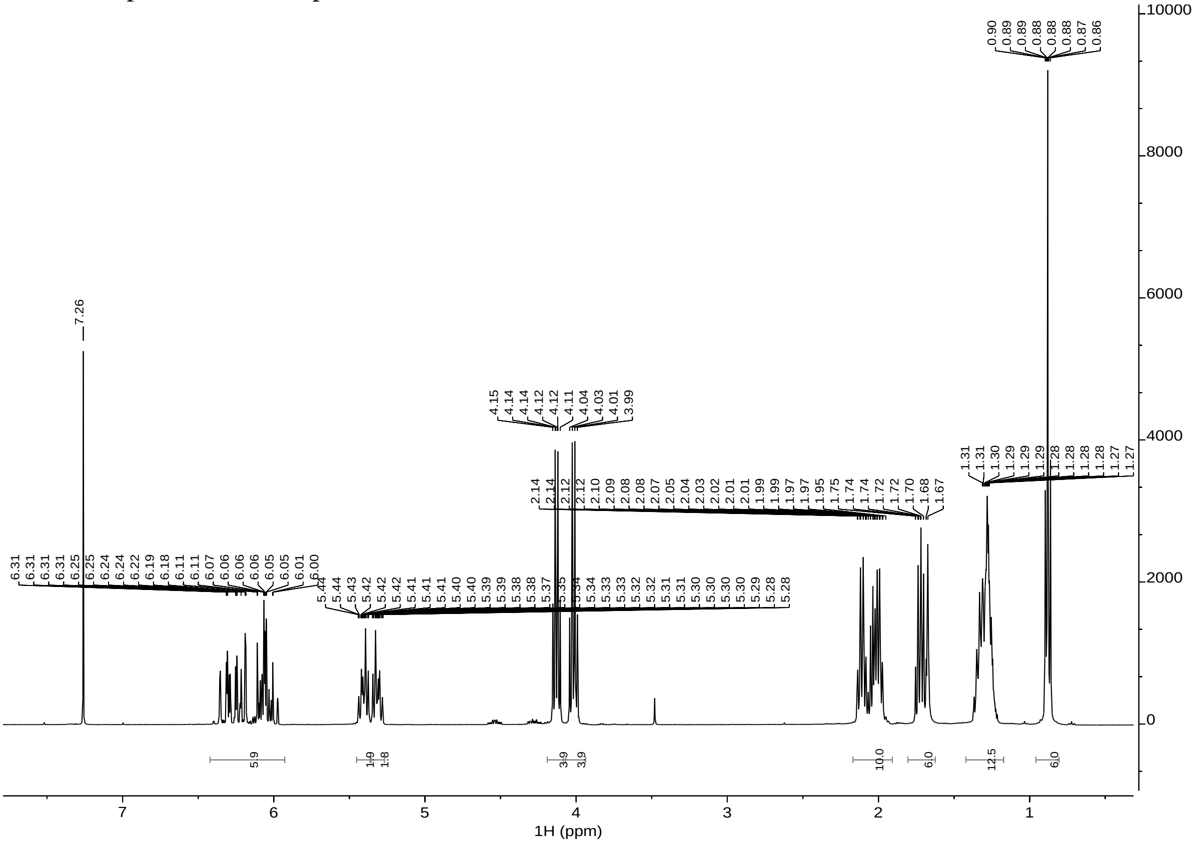

^{13}C NMR spectrum of compound **S44**

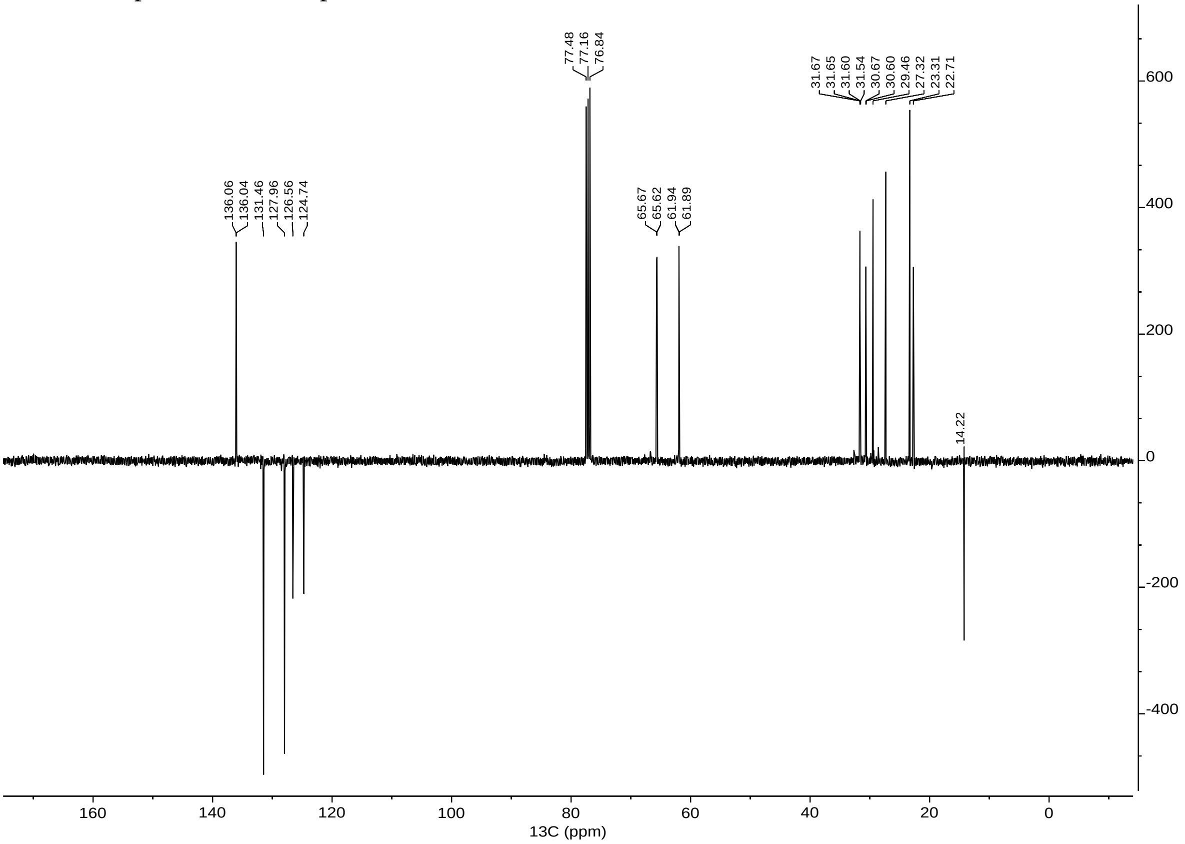

^{31}P{^{1}H} NMR spectrum of compound **S44**

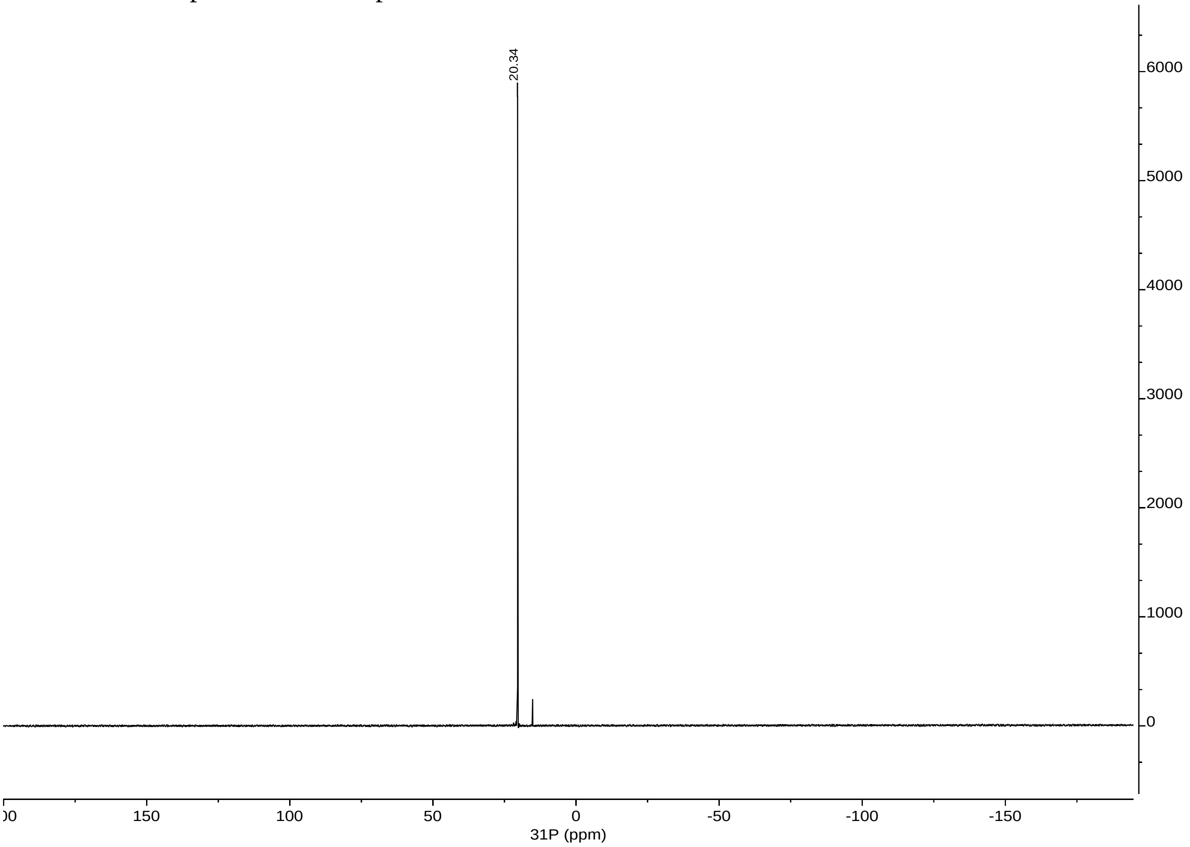

Bis((adamantan-1-yl)methyl) propane-1,3-diyl bis(vinylphosphonate) (S45)

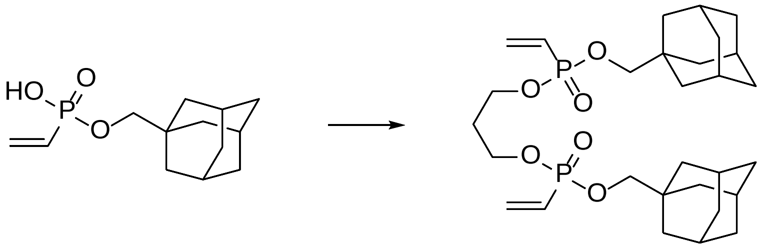

The title compound was prepared according to general method **C** from mono alkyl vinylphosphonate **S35** (2.50 g, 2.86 mmol) and 1,3-dibromopropane (0.35 mL, 3.41 mmol) in 96% yield (1.82 g, 3.29 mmol) as a colourless oil.

^{1}H NMR (401 MHz, $CDCl_3$) δ 6.37–5.95 (m, 6H, C*H*=C*H*$_2$), 4.14 (dt, *J* = 7.4, 6.1 Hz, 4H, OC*H*$_2$CH$_2$), 3.56 (d, *J* = 5.4 Hz, 4H, OC*H*$_2$C$_{quat}$), 2.04 (p, *J* = 6.1 Hz, 2H, OCH$_2$C*H*$_2$), 1.98 (p, *J* = 3.1 Hz, 6H, C$_{quat}$CH$_2$C*H*), 1.76–1.58 (m, 12H, C$_{quat}$C*H*$_2$CH), 1.52 (d, *J* = 2.6 Hz, 12H, *c*-(CHC*H*$_2$)$_3$).

^{13}C NMR (101 MHz, $CDCl_3$) δ 136.02 (d, *J* = 2.0 Hz, =*C*H$_2$), 125.49 (d, *J* = 184.4 Hz, P*C*H), 75.65 (d, *J* = 6.3 Hz, O*C*H$_2$CH$_2$), 61.94 (dd, *J* = 5.5, 1.4 Hz, O*C*H$_2$C$_{quat}$), 39.00 (*c*-(CH*C*H$_2$)$_3$), 37.03 (C$_{quat}$*C*H$_2$CH), 33.95 (d, *J* = 7.1 Hz, *C*$_{quat}$), 31.63 (t, *J* = 6.5 Hz, OCH$_2$*C*H$_2$), 28.09 (C$_{quat}$CH$_2$*C*H).

^{31}P{^{1}H} NMR (162 MHz, $CDCl_3$) δ 20.49.

IR ν_{max} ($CHCl_3$) 3090 (vw), ~2936 (s, sh), 2907 (vs), 2851 (s), 2755 (vw), 2677 (vw), 2660 (vw), 1614 (w), 1462 (w), 1453 (w), 1400 (w), 1386 (w), 1365 (w), 1279 (w), 1243 (s), 1155 (w), 1106 (w), 1059 (s), 1053 (s), 1045 (s), 1018 (vs), 1004 (s), 986 (s), 943 (w), 924 (w), 809 (w), 435 (w).

HR-MS(ESI$^+$): For $C_{29}H_{47}O_6P_2$ $(M+H)^+$ *m/z* calculated 553.28424, found 553.28326. For $C_{29}H_{46}O_6NaP_2$ $(M+Na)^+$ *m/z* calculated 575.26618, found 575.26514.

^{1}H NMR spectrum of compound **S45**

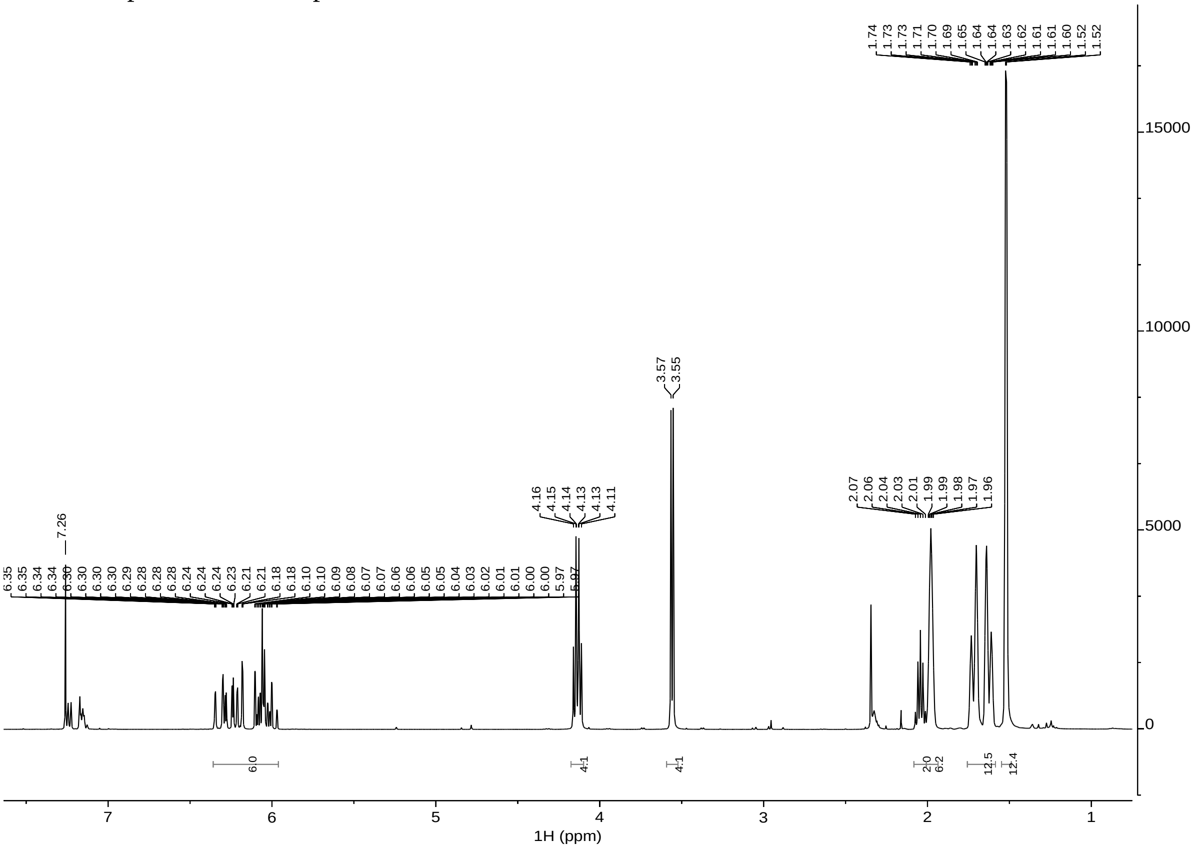

^{13}C NMR spectrum of compound **S45**

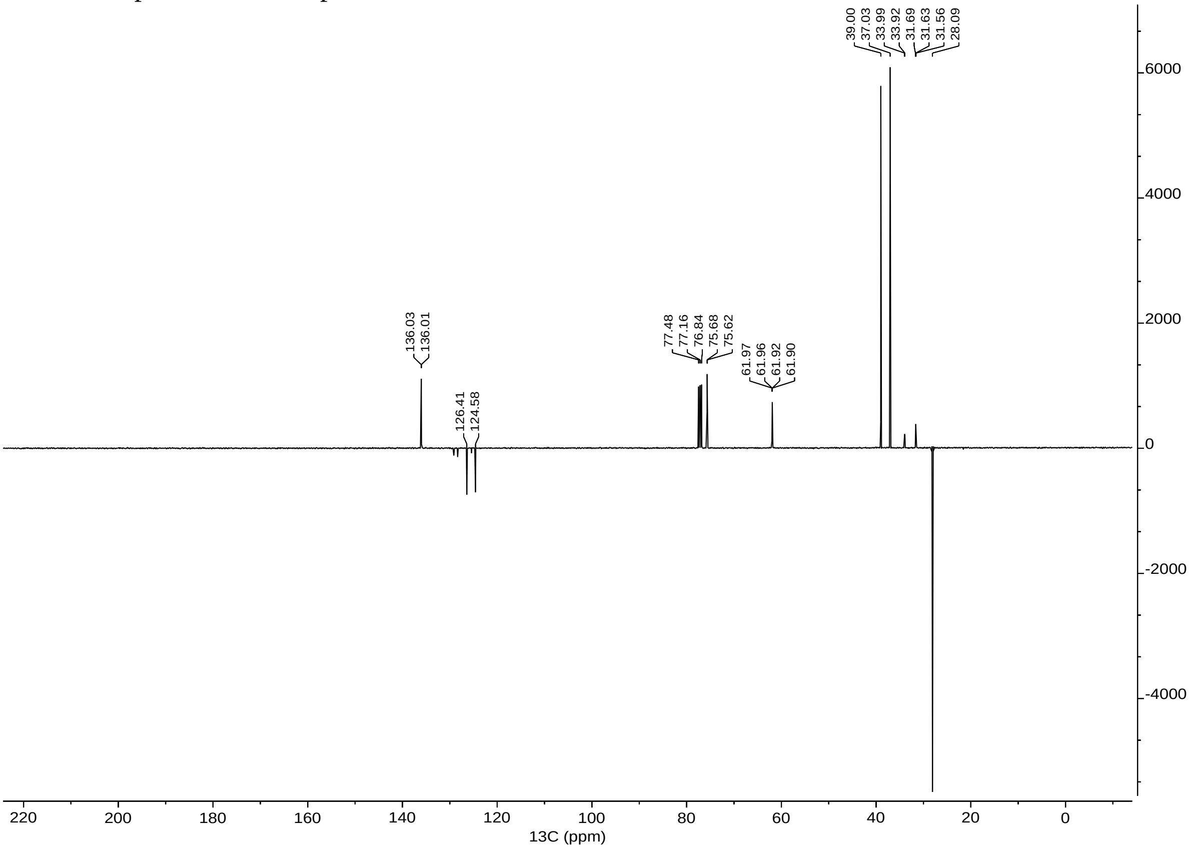

^{31}P{^{1}H} NMR spectrum of compound **S45**

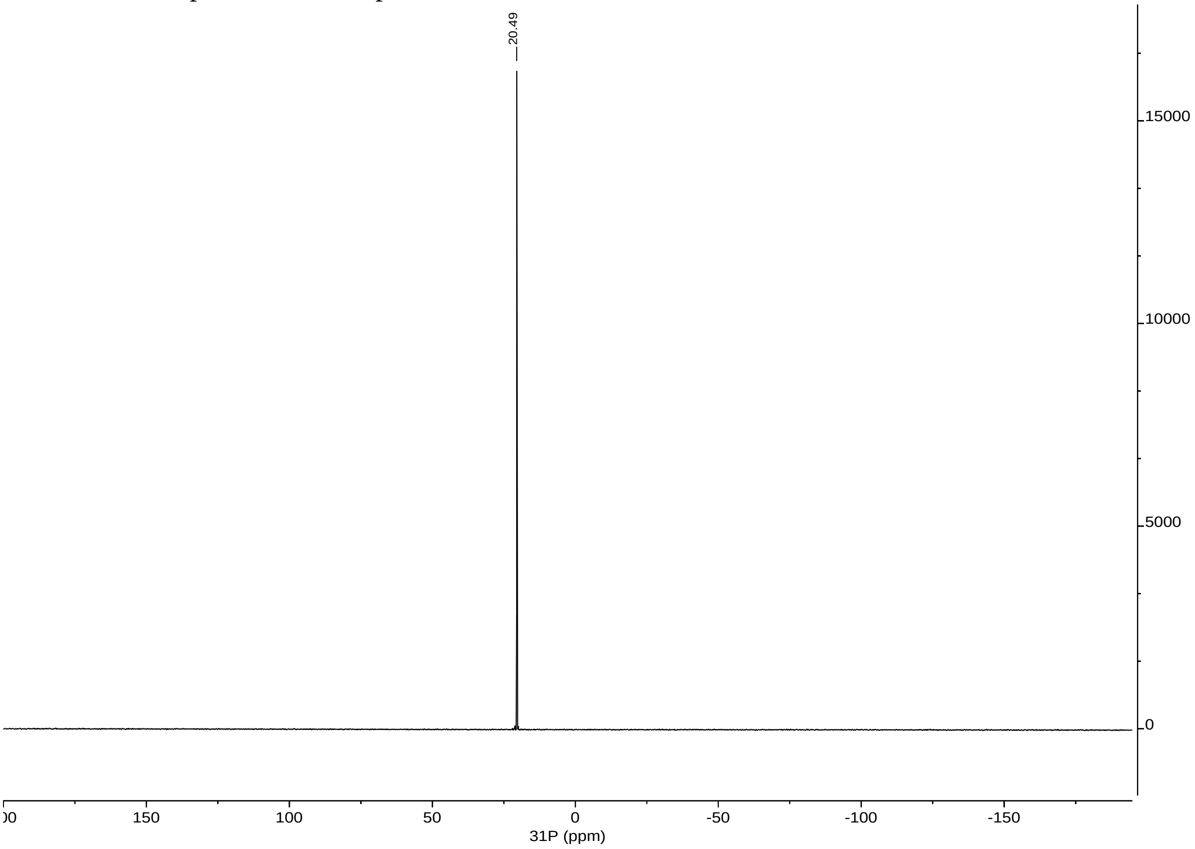

Bis(2-(adamantan-1-yl)ethyl) propane-1,3-diyl bis(vinylphosphonate) (S46)

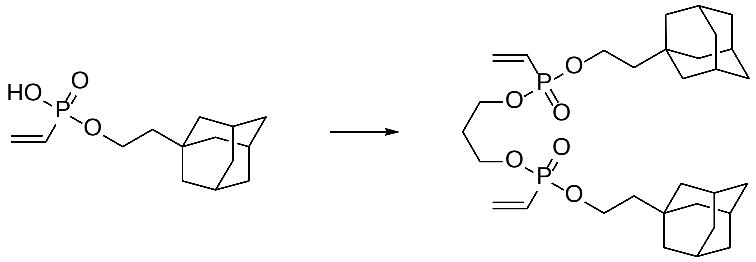

The title compound was prepared according to general method **C** from mono alkyl vinylphosphonate **S36** (3.18 g, 11.8 mmol) and 1,3-dibromopropane (0.40 mL, 3.92 mmol) in 63% yield (1.42 g, 2.45 mmol) as a colourless oil.

^{1}H NMR (401 MHz, $CDCl_3$) δ 6.37–5.96 (m, 6H, C*H*=C*H*$_2$), 4.13 (dt, *J* = 7.3, 6.1 Hz, 5H, OC*H*$_2$CH$_2$C*H*$_2$O), 4.08 (q, *J* = 7.4 Hz, 4H, OC*H*$_2$CH$_2$C$_{quat}$), 2.04 (p, *J* = 6.1 Hz, 2H, OCH$_2$C*H*$_2$CH$_2$O), 1.94 (p, *J* = 3.0 Hz, 6H, C$_{quat}$CH$_2$C*H*), 1.74–1.57 (m, 12H, C$_{quat}$C*H*$_2$CH), 1.54–1.45 (m, 16H, *c*-(CHC*H*$_2$)$_3$, OCH$_2$C*H*$_2$C$_{quat}$).

^{13}C NMR (101 MHz, $CDCl_3$) δ 136.13 (d, *J* = 2.1 Hz, =*C*H$_2$), 125.65 (d, *J* = 183.7 Hz, P*C*H), 62.70 (d, *J* = 5.8 Hz, O*C*H$_2$CH$_2$C$_{quat}$), 61.98 (d, *J* = 5.5 Hz, O*C*H$_2$CH$_2$*C*H$_2$O), 44.44 (d, *J* = 6.2 Hz, OCH$_2$*C*H$_2$C$_{quat}$), 42.64 (*c*-(CH*C*H$_2$)$_3$), 37.10 (C$_{quat}$*C*H$_2$CH), 31.92 (*C*$_{quat}$), 31.60 (t, *J* = 6.5 Hz, OCH$_2$*C*H$_2$CH$_2$O), 28.66 (C$_{quat}$CH$_2$*C*H).

^{31}P{^{1}H} NMR (162 MHz, $CDCl_3$) δ 20.35.

IR ν_{max} ($CHCl_3$) 3091 (vw), ~2936 (s, sh), 2907 (vs), 2851 (s), 2755 (vw), 2676 (vw), 2660 (vw), 1614(w), 1462 (w), 1453 (m), 1400 (m), 1387 (w), 1365 (w), 1347 (w), 1279 (m), 1242 (s), 1155 (w), 1106 (w), 1059 (s), 1013 (vs), 1001 (vs), 988 (vs), 943 (w), 924 (w), 809 (m), 434 (w).

HR-MS(ESI^{+}): For $C_{31}H_{51}O_6P_2$ $(M+H)^{+}$ *m/z* calculated 581.31554, found 581.31440. For $C_{31}H_{50}O_6NaP_2$ $(M+Na)^{+}$ *m/z* calculated 603.29748, found 603.29613.

^{1}H NMR spectrum of compound **S46**

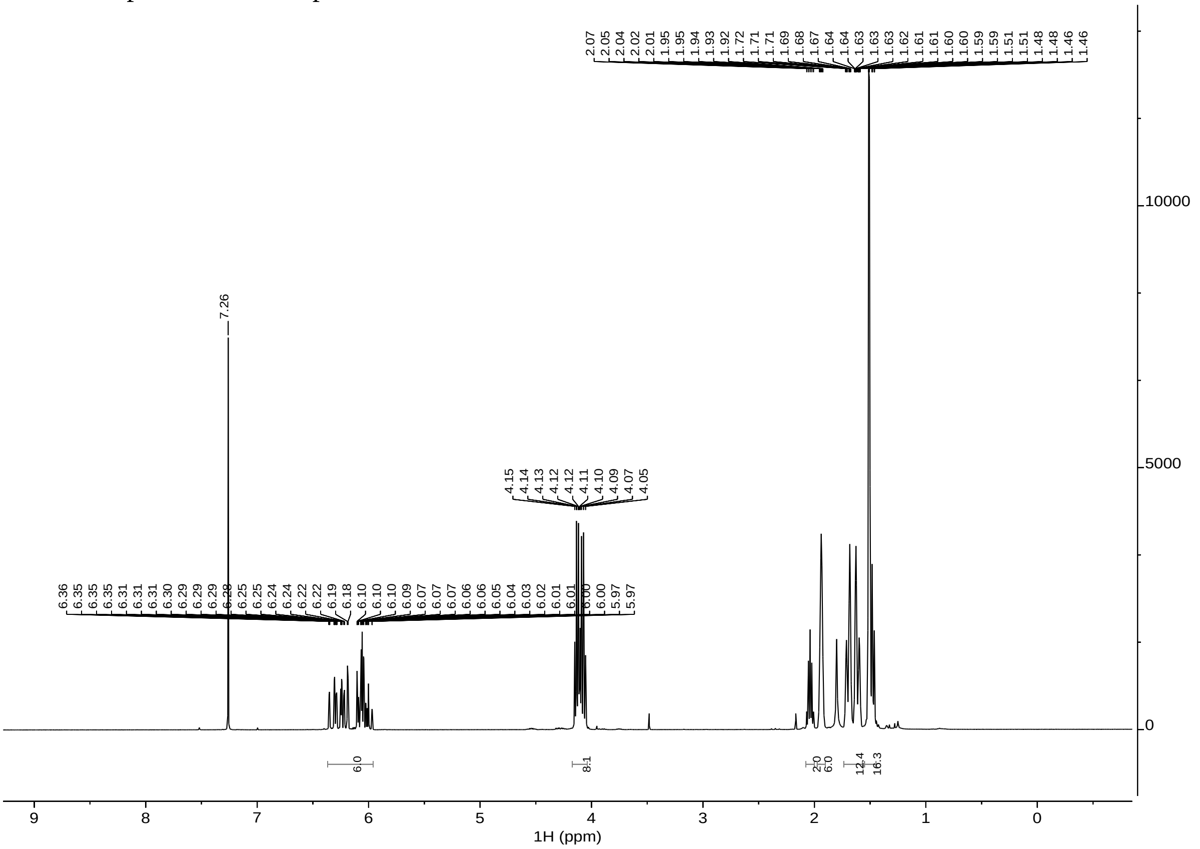

^{13}C NMR spectrum of compound **S46**

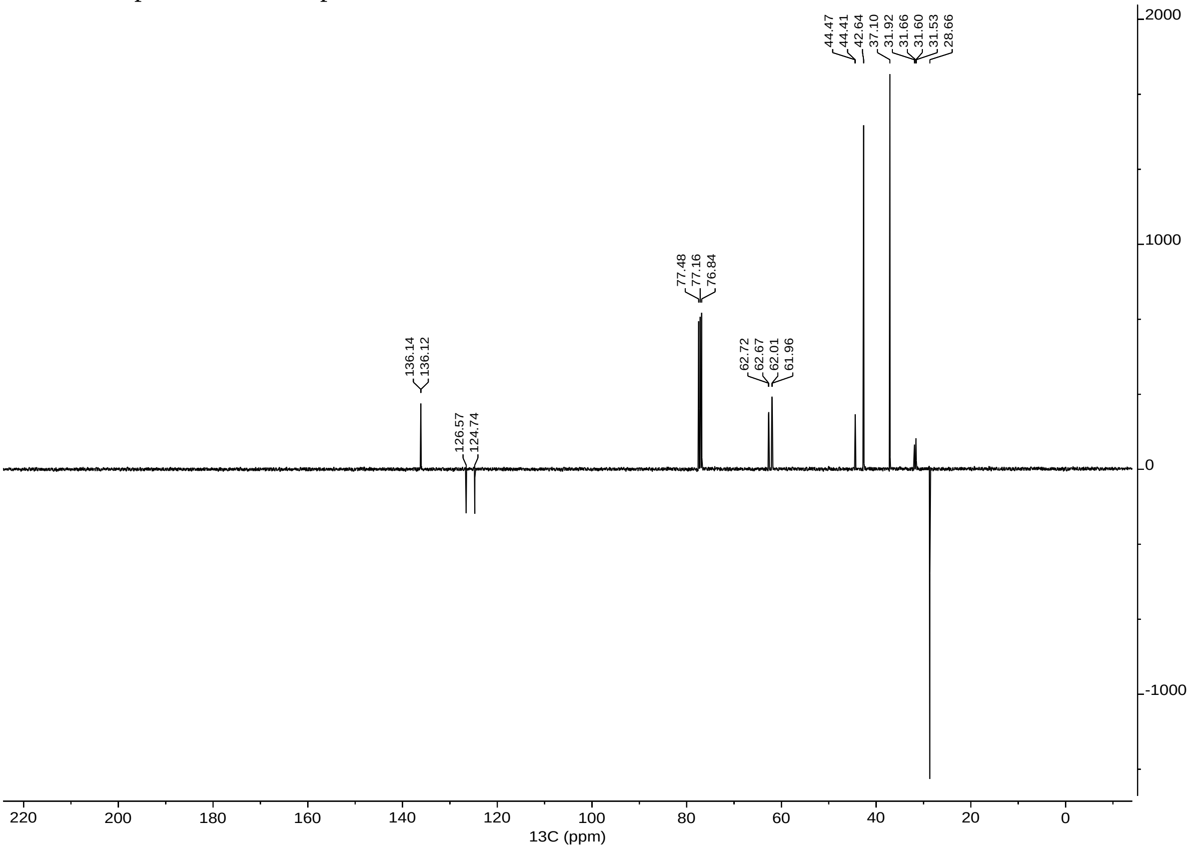

^{31}P{^{1}H} NMR spectrum of compound **S46**

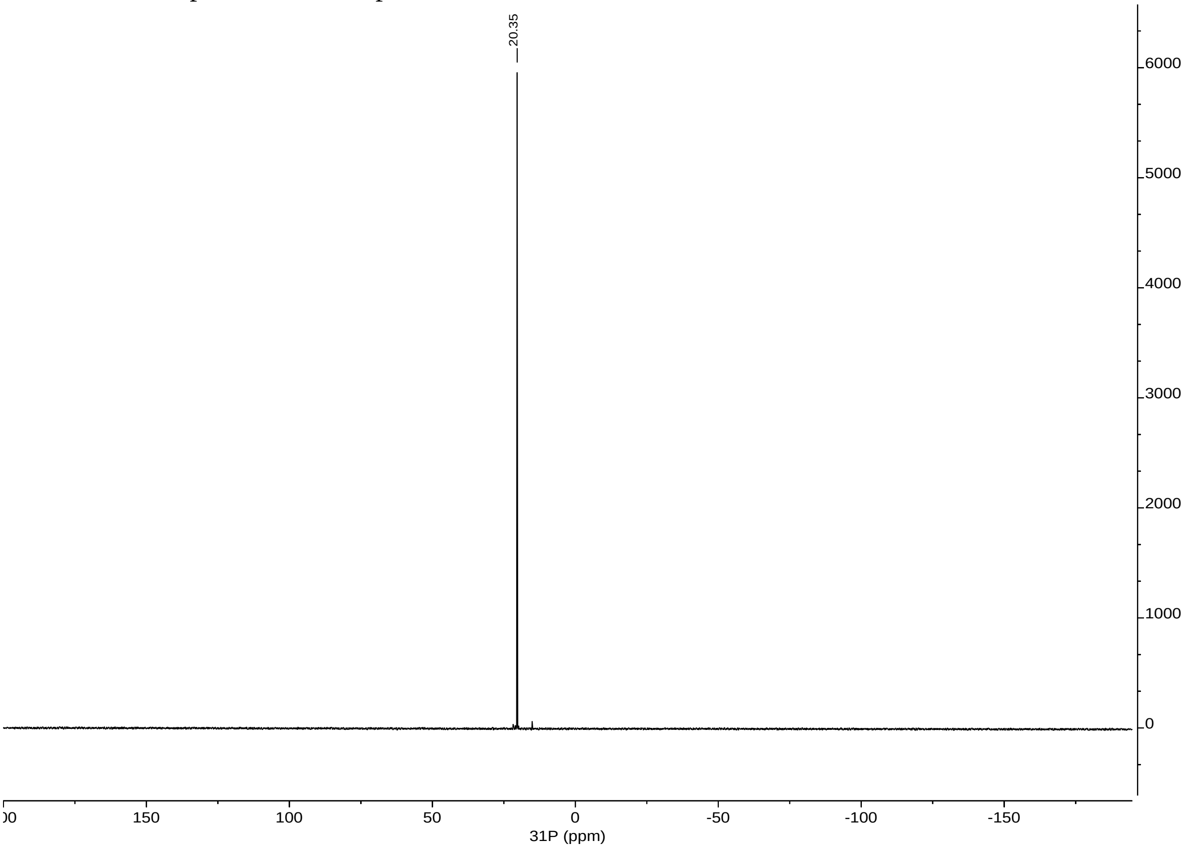

Butane-1,4-diyl diheptyl bis(vinylphosphonate) (S47)

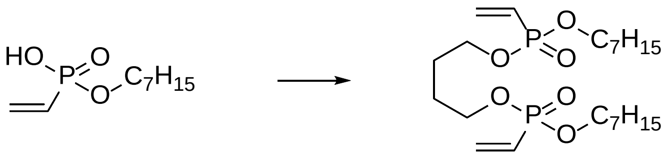

The title compound was prepared according to general method **C** from mono alkyl vinylphosphonate **S25** (1.92 g, 9.33 mmol) and 1,4-dibromobutane (99%, 0.38 mL, 3.11 mmol) in 17% yield (0.25 g, 0.53 mmol) as a colourless oil.

^{1}H NMR (401 MHz, CD_3OD) δ 6.34–6.09 (m, 6H, C*H*=C*H*$_2$), 4.11–3.99 (m, 8H, OC*H*$_2$), 1.80 (h, *J* = 3.0 Hz, 4H), 1.68 (dt, *J* = 8.1, 6.3 Hz, 4H, OCH_2CH_2), 1.45–1.26 (m, 16H, $(CH_2)_4CH_3$), 0.95–0.88 (m, 6H, C*H*$_3$).

^{13}C NMR (101 MHz, CD_3OD) δ 137.18 (d, *J* = 1.8 Hz, =*C*H$_2$), 126.32 (d, *J* = 184.1 Hz, P*C*H), 67.55 (d, *J* = 6.0 Hz), 66.83 (d, *J* = 6.0 Hz, O*C*H$_2$), 32.91 (*C*$H_2CH_2CH_3$), 31.51 (d, *J* = 6.3 Hz, *C*$H_2(CH_2)_4CH_3$), 29.93, 27.81 (d, *J* = 6.4 Hz, $OCH_2(CH_2)_2CH_2O$), 26.61, 23.65 (*C*H_2CH_3), 14.41 (*C*H$_3$).

^{31}P{^{1}H} NMR (162 MHz, CD_3OD) δ 20.80.

IR ν_{max} ($CHCl_3$) 3090 (w), 2960 (m), 2930 (m), 2872 (m), 2858 (m), 1615 (w), 1469 (m), 1458 (sh), 1401 (m), 1380 (m), 1278 (m), 1246 (s), 1080–1000 (m), 985 (m).

HR-MS(APCI$^+$): For $C_{22}H_{45}O_6P_2$ $(M+H)^+$ *m/z* calculated 467.26859, found 467.26867.

^{1}H NMR spectrum of compound **S47**

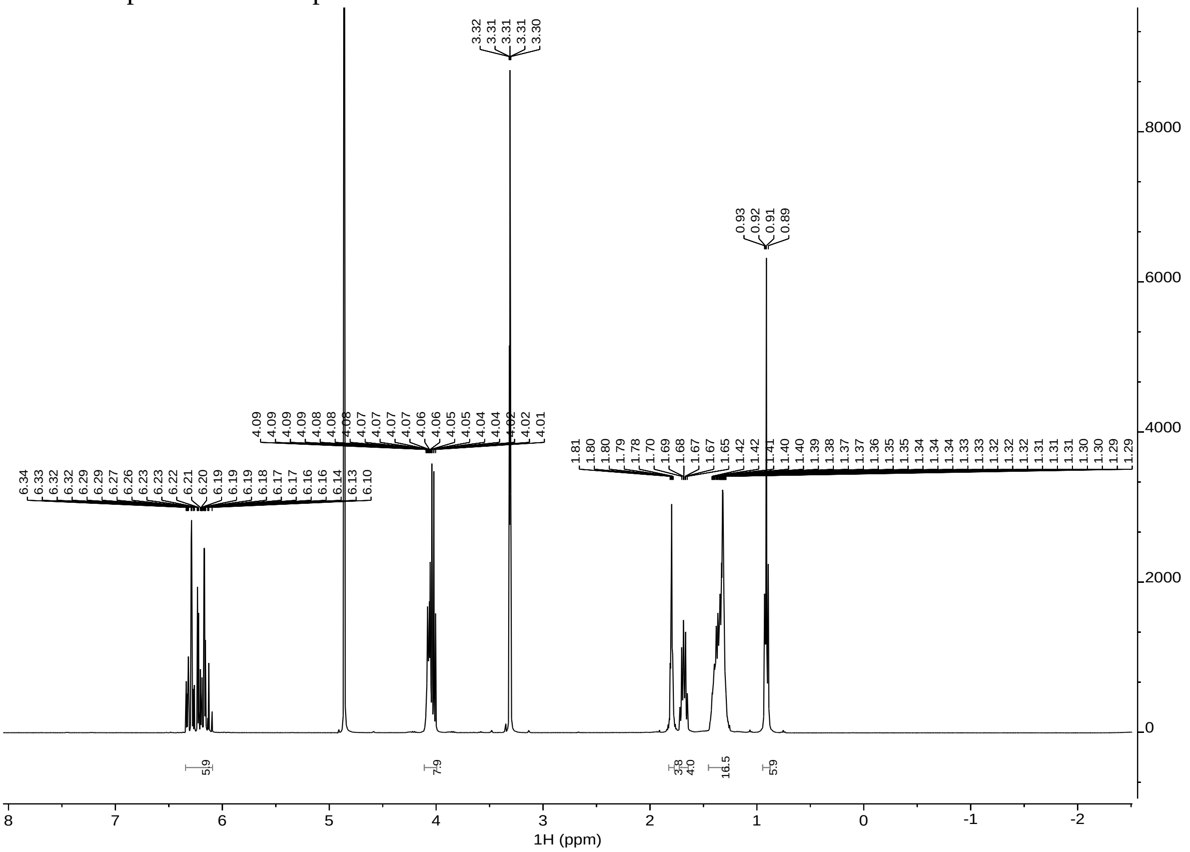

^{13}C NMR spectrum of compound **S47**

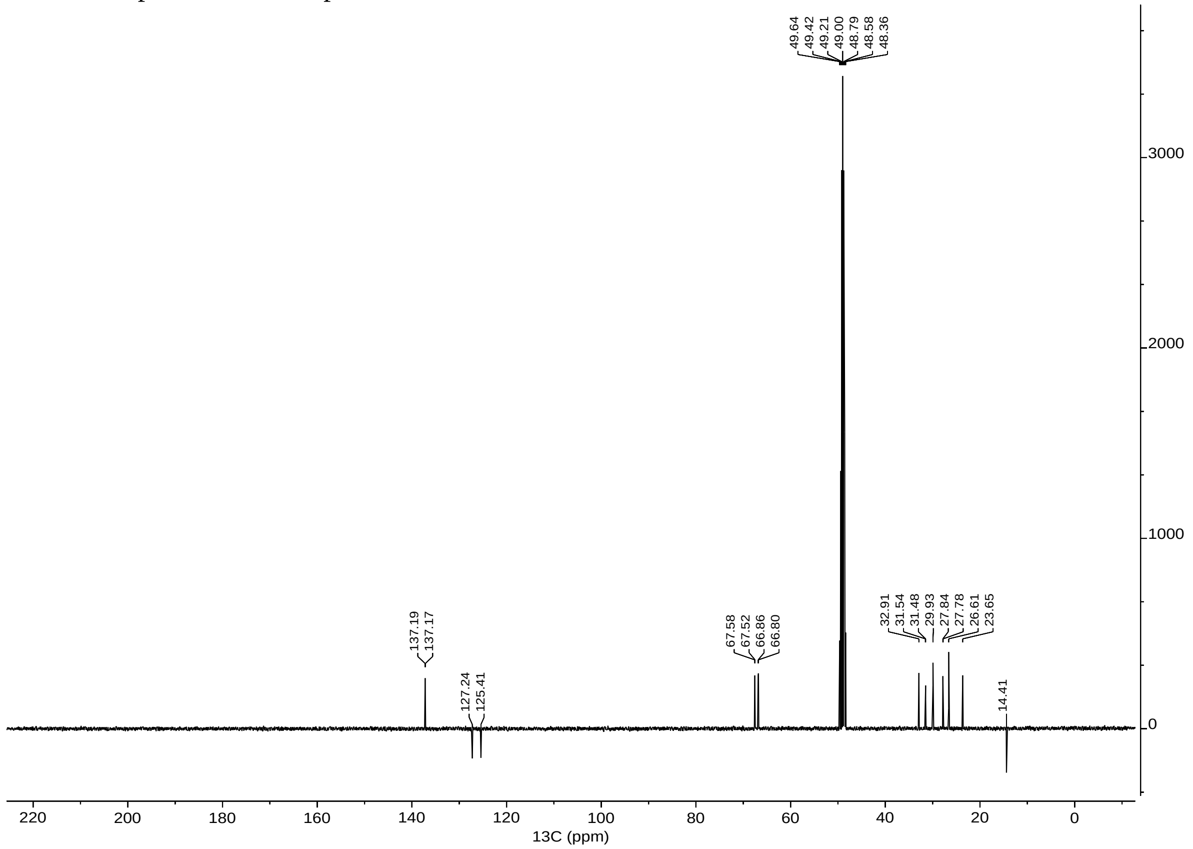

$^{31}P\{^{1}H\}$ NMR spectrum of compound **S47**

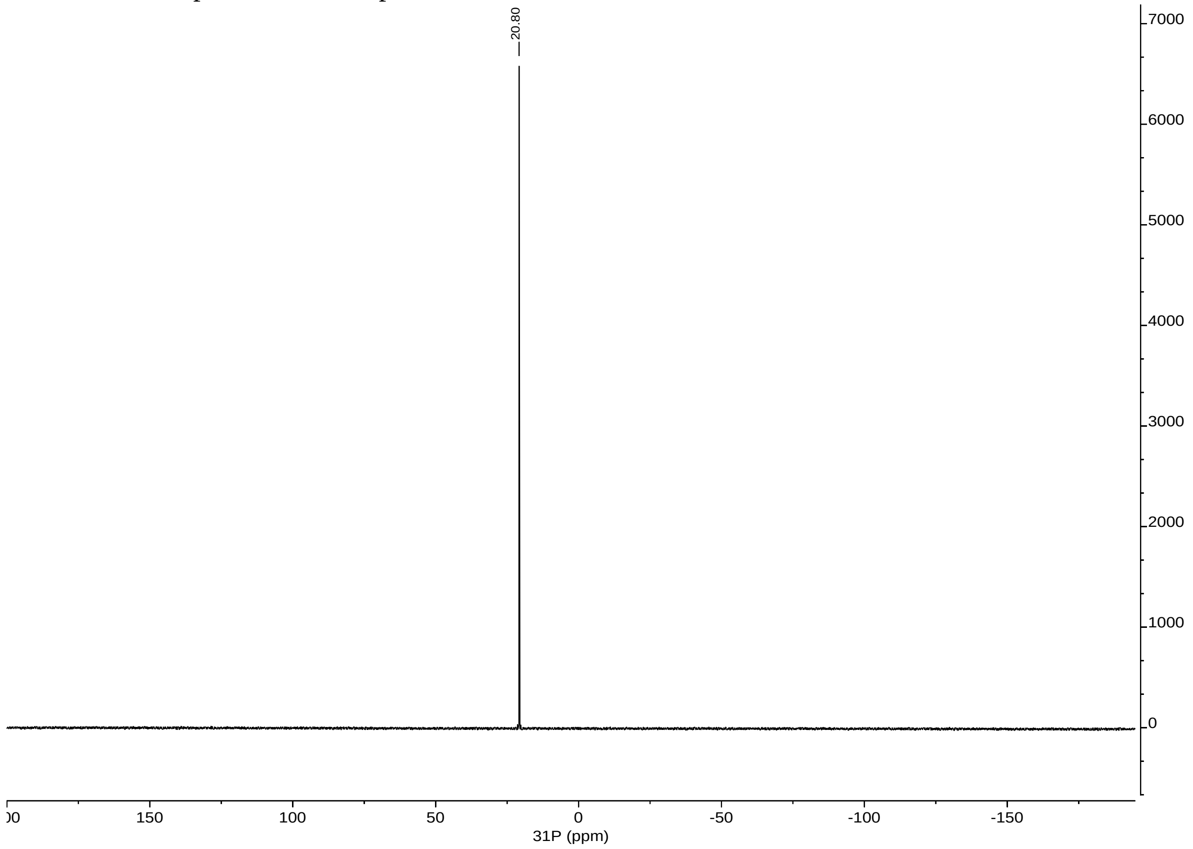

Butane-1,4-diyl dioctyl bis(vinylphosphonate) (S48)

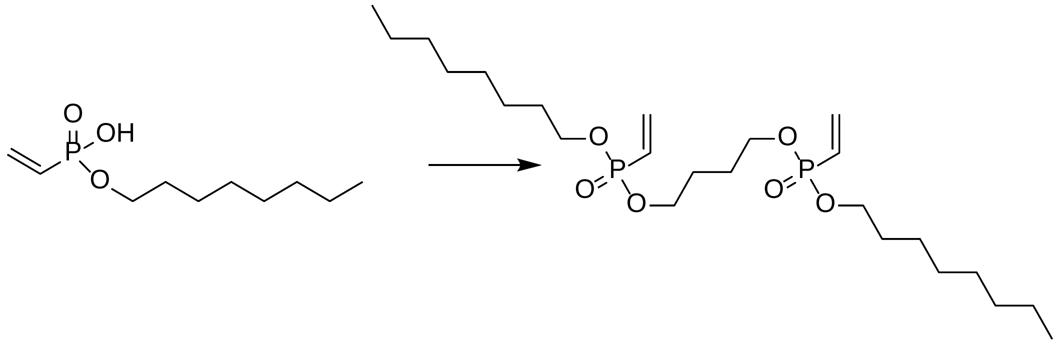

The title compound was prepared according to general method **C** from mono alkyl vinylphosphonate **S28** (1.0 g, 4.27 mmol) and 1,4-dibromobutane (0.31 g, 1.43 mmol) in 75% yield (0.53 g, 1.07 mmol) as a colourless oil.

^{1}H NMR (400 MHz, $CDCl_3$): 6.34–5.97 (m, 6H, C*H*=C*H*$_2$), 4.06–3.97 (m, 8H, $CH_3(CH_2)_5CH_2C\mathit{H}_2O$, $OC\mathit{H}_2CH_2CH_2C\mathit{H}_2O$), 1.74–1.80–1.74 (m, 4H, $OCH_2C\mathit{H}_2C\mathit{H}_2CH_2O$), 1.68–1.62 (m, 4H, $CH_3(CH_2)_5C\mathit{H}_2CH_2O$), 1.36–1.24 (m, 20H, $CH_3(C\mathit{H}_2)_5CH_2CH_2O$), 0.89–0.85 (m, 6H, $C\mathit{H}_3(CH_2)_6CH_2O$).

^{13}C NMR (101 MHz, $CDCl_3$): 135.76 (d, J = 2.1 Hz, CH=*C*H$_2$), 125.88 (d, J = 183.2 Hz, *C*H=CH$_2$), 66.17, 65.20 (d, J = 5.6 Hz, O*C*H$_2$CH$_2$CH$_2$*C*H$_2$O, $CH_3(CH_2)_5CH_2\mathit{C}H_2O$), 30.60 (d, J = 6.2 Hz, $CH_3(CH_2)_5\mathit{C}H_2CH_2O$), 26.88 (d, J = 6.5 Hz, $OCH_2\mathit{C}H_2\mathit{C}H_2CH_2O$), 31.90, 29.29, 29.24, 25.64, 22.76 ($CH_3(\mathit{C}H_2)_5CH_2CH_2O$), 14.21 ($\mathit{C}H_3(CH_2)_6CH_2O$).

^{31}P{^{1}H} NMR (162 MHz, $CDCl_3$): 19.88.

IR ν_{max} (KBr) 3091 (vw), 2959 (s), 2930 (s), 2872 (m), 2858 (m), 1468 (w), 1458 (w), 1446 (w), 1436 (w), 1400 (w), 1242 (s), 1070 (m), 1049 (s), 1013 (vs), 987 (vs), 856 (w).

HR-MS(ESI$^+$): For $C_{24}H_{49}O_6P_2$ $(M+H)^+$ m/z calculated 495.29989, found 495.29956.

^{1}H NMR spectrum of compound **S48**

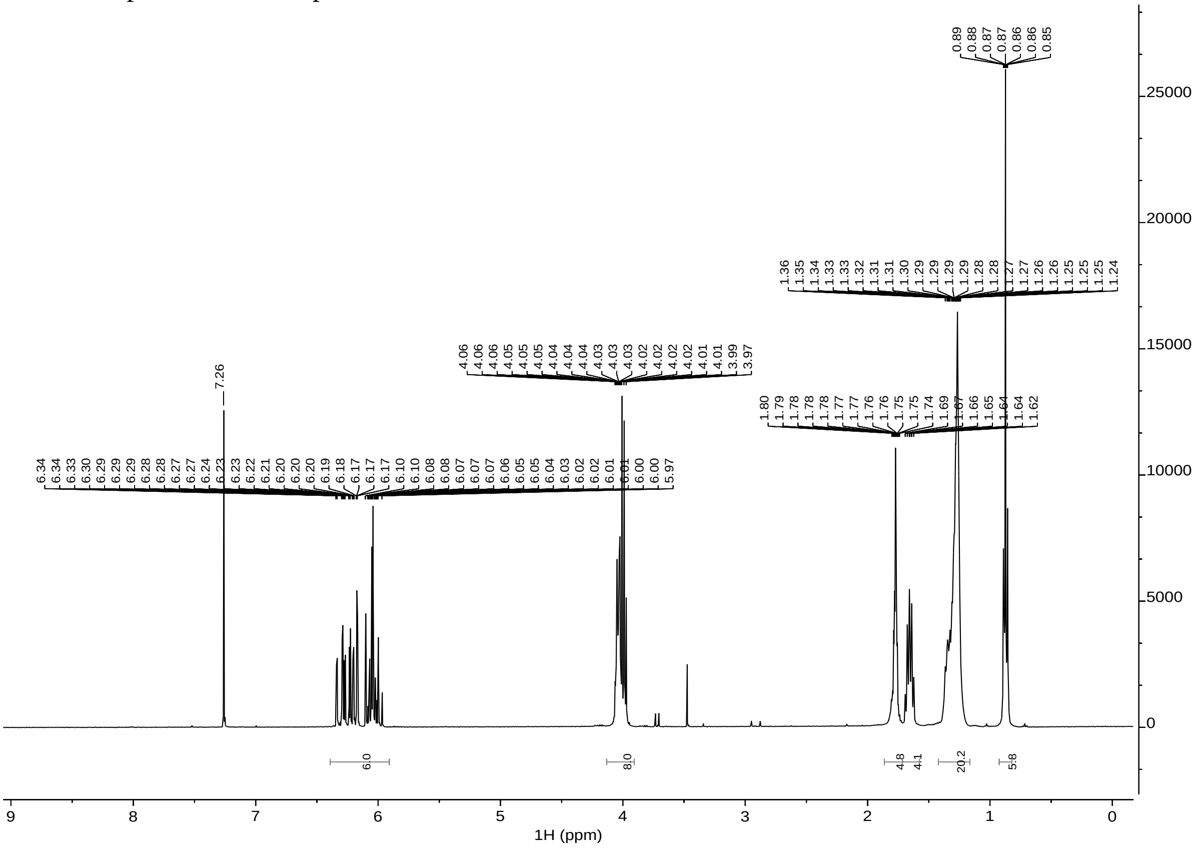

^{13}C NMR spectrum of compound **S48**

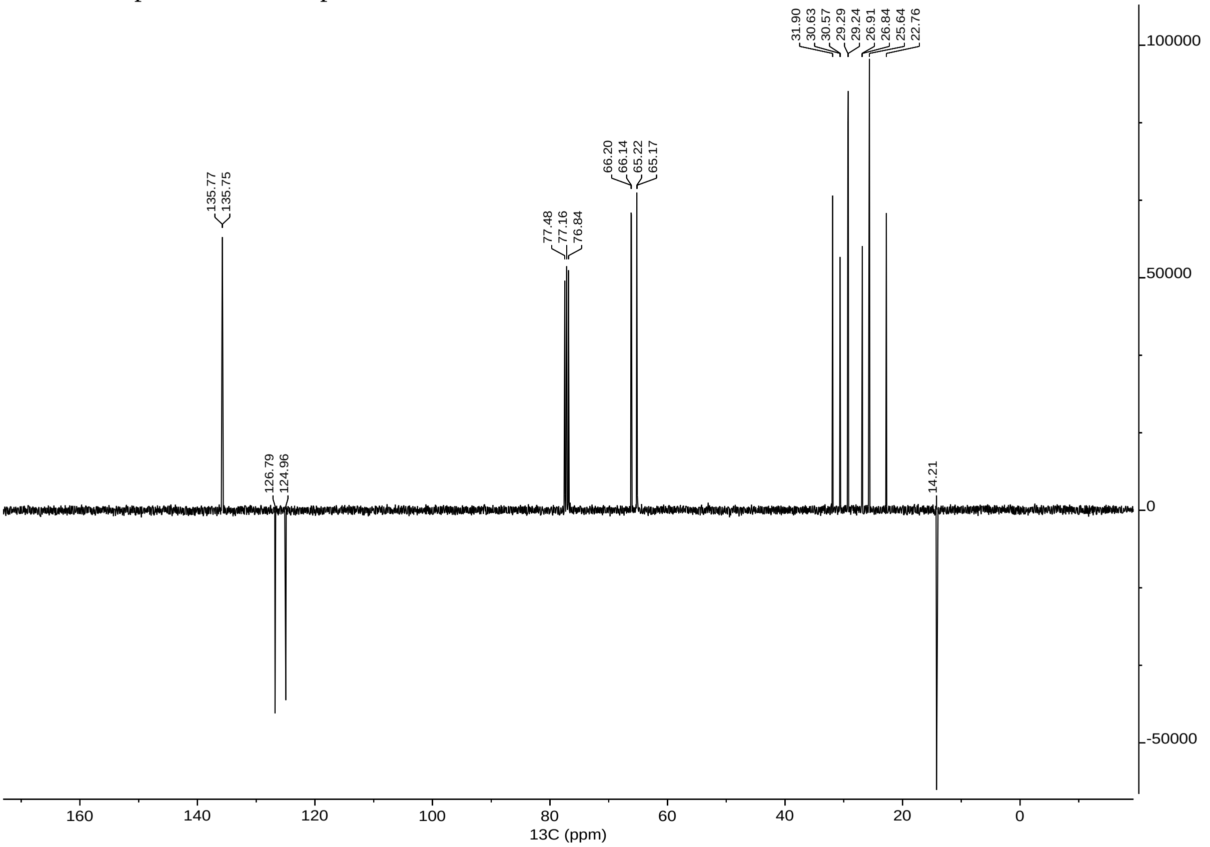

$^{31}P\{^{1}H\}$ NMR spectrum of compound **S48**

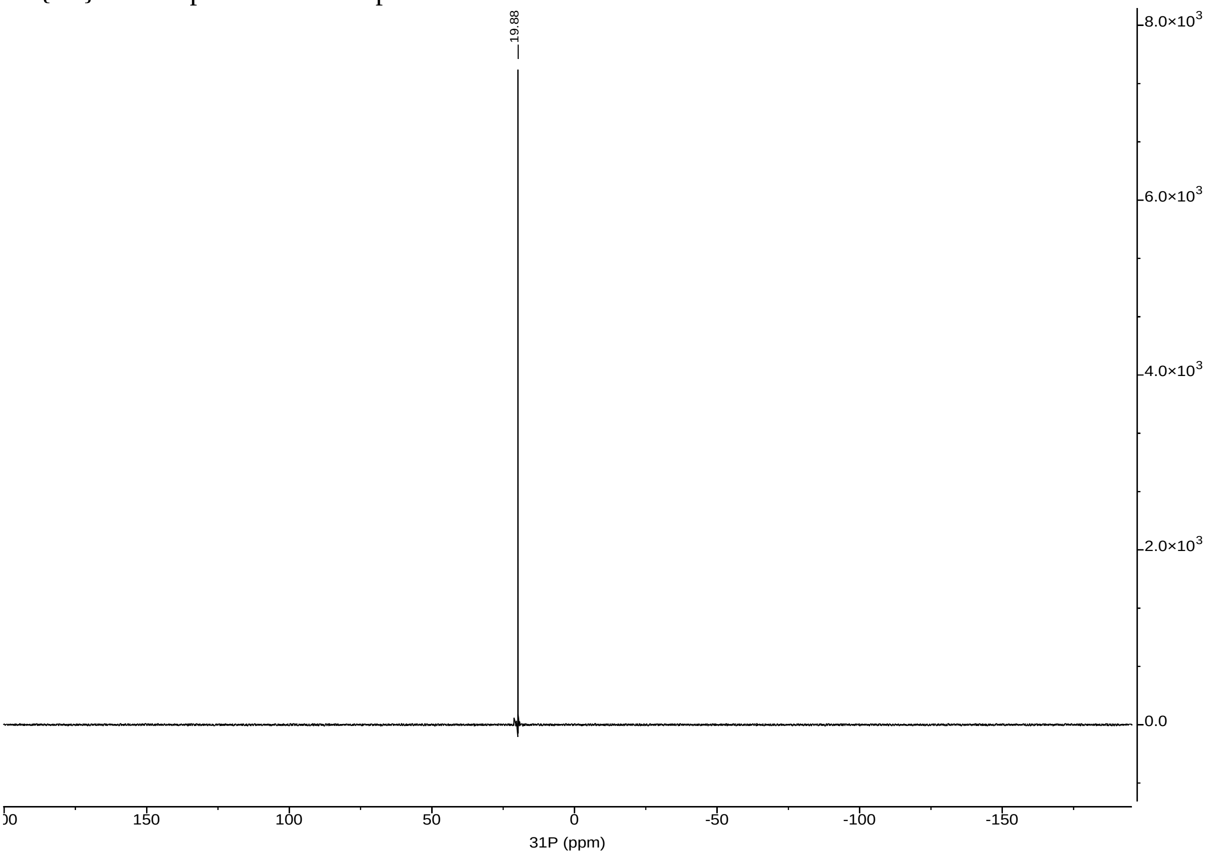

Bis((*Z*)-hept-3-en-1-yl) pentane-1,5-diyl bis(vinylphosphonate) (S49)

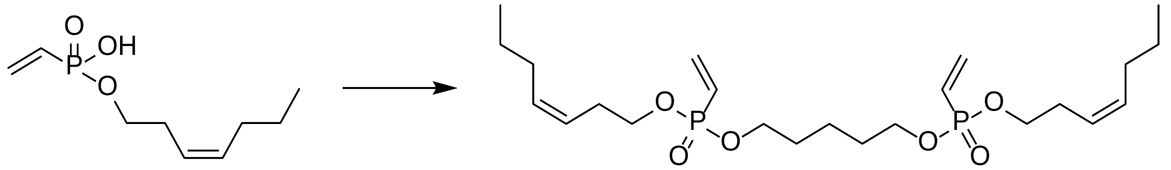

The title compound was prepared according to general method **C** from mono alkyl vinylphosphonate **S26** (1.10 g, 5.39 mmol) and 1,5-dibromopentane (0.42 g, 1.83 mmol) in 82% yield (0.72 g, 1.51 mmol) as a colourless oil.

^{1}H NMR (400 MHz, $CDCl_3$): 6.33–5.96 (m, 6H, C*H*=C*H*$_2$), 5.53–5.46 (m, 2H, C*H*=$CHCH_2CH_2O$), 5.38–5.30 (m, 2H, CH=C*H*CH_2CH_2O), 2.38–2.44 (m, 4H, $CH_3CH_2CH_2CHCHC$*H*$_2CH_2O$), 2.03–1.97 (m, 4H, CH_3CH_2C*H*$_2CHCHCH_2CH_2O$), 1.69 (dq, 4H, *J* = 8.1, 6.7 Hz, OCH_2C*H*$_2CH_2C$*H*$_2CH_2O$), 1.49–1.43 (m, 2H, OCH_2CH_2C*H*$_2CH_2CH_2O$), 1.35 (dt, 4H, *J* = 14.6, 7.3 Hz, CH_3C*H*$_2$), 0.88 (t, 6H, *J* = 7.4 Hz, C*H*$_3$).

^{13}C NMR (101 MHz, $CDCl_3$): 135.75 (d, *J* = 2.0 Hz, CH=*C*H_2), 133.17 (*C*H=$CHCH_2CH_2O$), 125.86 (d, *J* = 184.8 Hz, *C*H=CH_2), 124.01 (CH=*C*HCH_2CH_2O), 65.59, 65.39 (d, *J* = 5.9 Hz, O*C*$H_2(CH_2)_3$*C*H_2O, $CH=CHCH_2$*C*H_2O), 30.10 (d, *J* = 6.3 Hz, OCH_2*C*H_2CH_2*C*H_2CH_2O), 29.51 (CH_3CH_2*C*H_2), 28.81 (d, *J* = 6.5 Hz, CH=*C*H_2CH_2O), 22.78 (CH_3*C*H_2), 21.80 ($O(CH_2)_2$*C*$H_2(CH_2)_2O$), 13.86 (*C*H_3).

^{31}P{^{1}H} NMR (162 MHz, $CDCl_3$): 19.86.

IR ν_{max} (KBr) 3086 (w), 3011 (m), 2960 (s), 2930 (m), 2872 (m), 1656 (w), 1613 (w), 1466 (m), 1457 (m), 1380 (w), 1281 (m), 1252 (vs), 1066–1011 (vs), 981 (vs), 722 (m).

HR-MS(ESI^{+}): For $C_{23}H_{42}O_6NaP_2$ $(M+Na)^{+}$ *m*/*z* calculated 499.23488, found 499.23491.

^{1}H NMR spectrum of compound **S49**

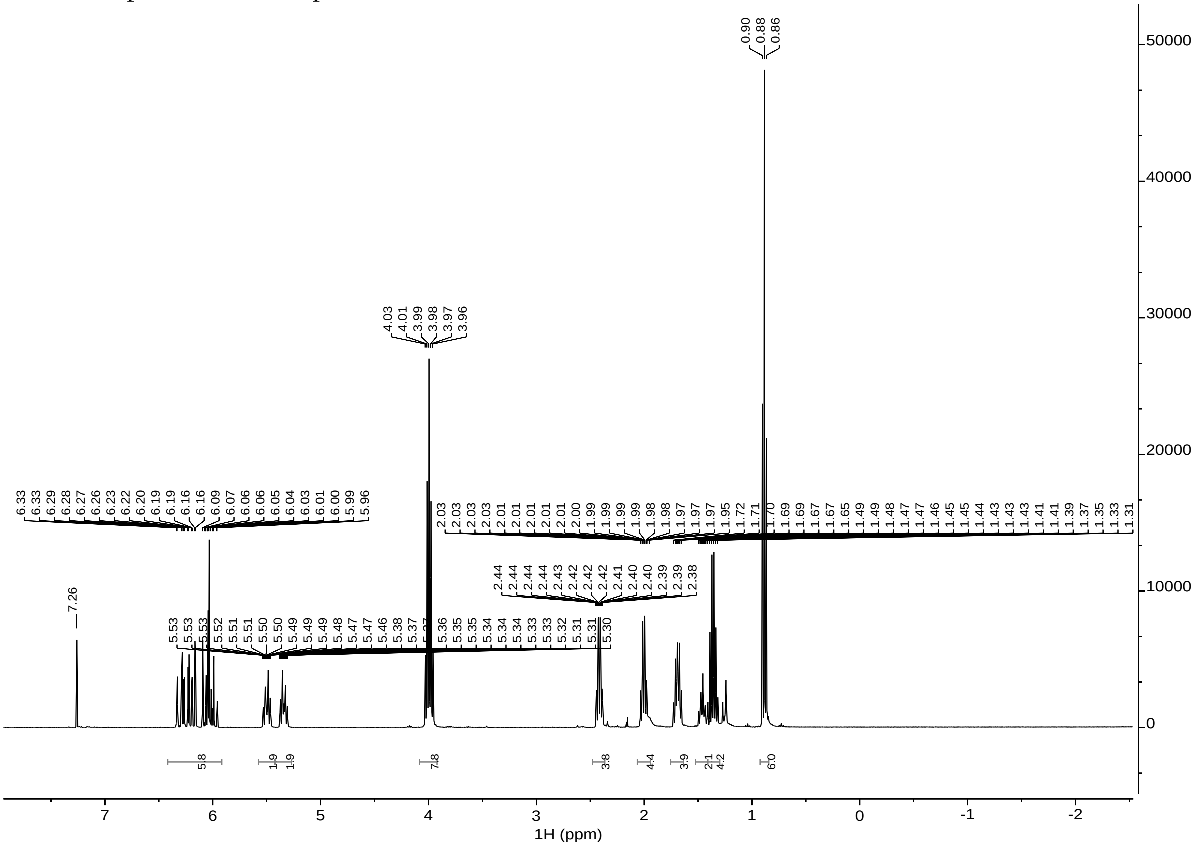

^{13}C NMR spectrum of compound **S49**

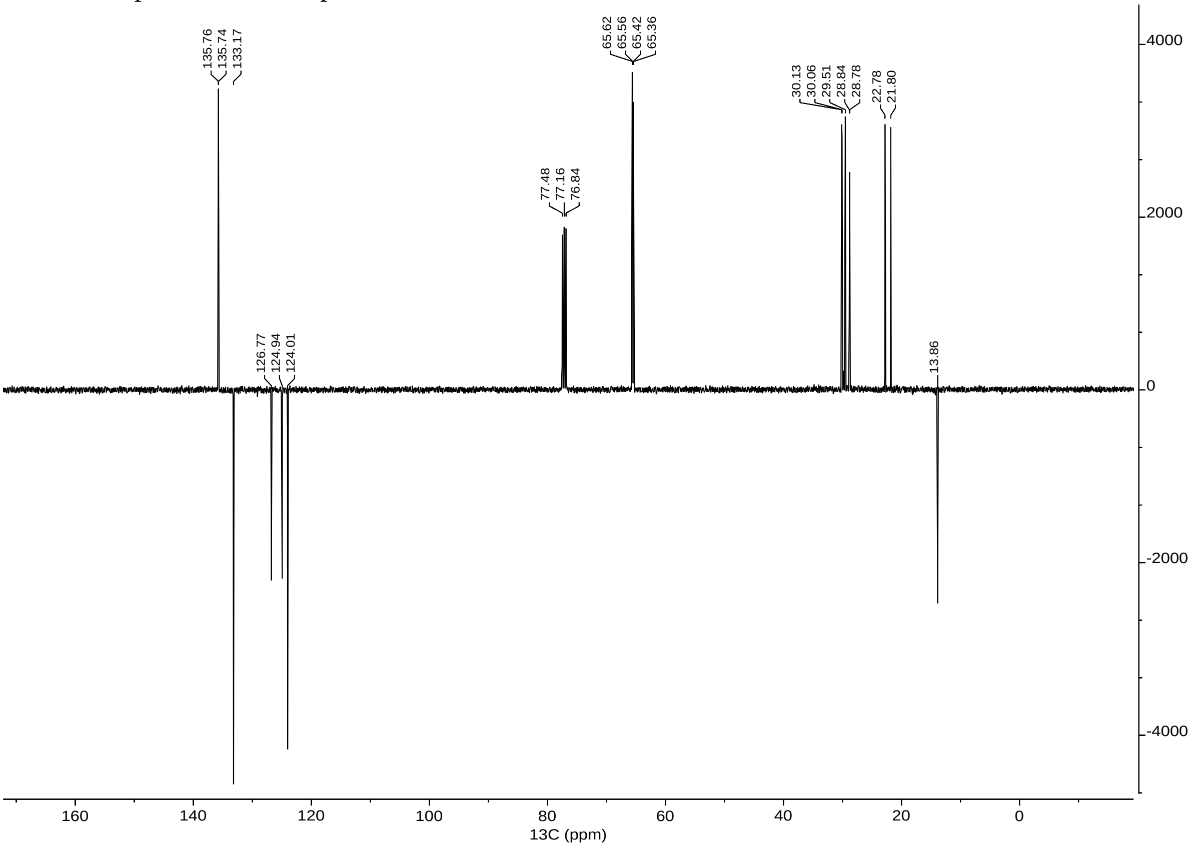

^{31}P{^{1}H} NMR spectrum of compound **S49**

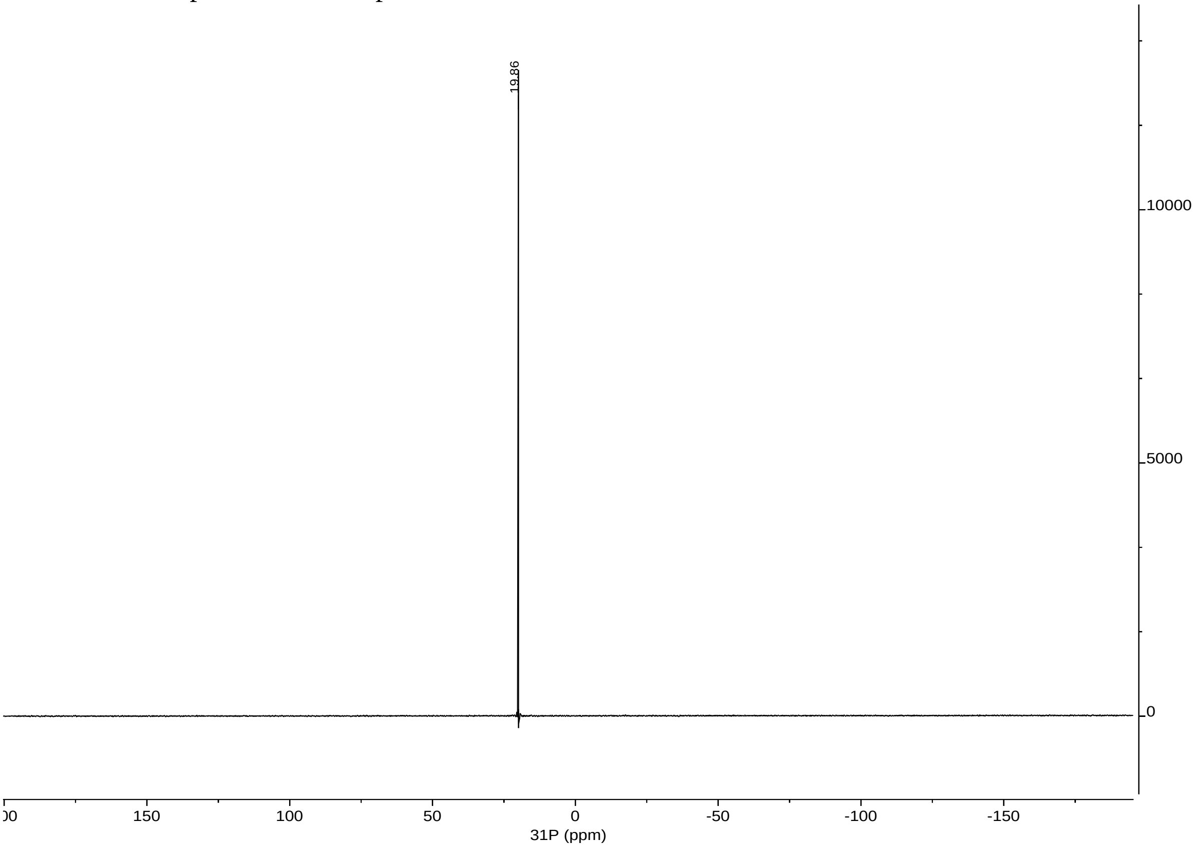

Bis((*Z*)-hept-4-en-1-yl) pentane-1,5-diyl bis(vinylphosphonate) (S50)

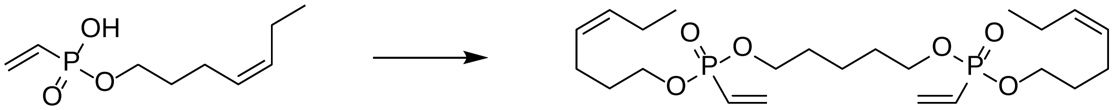

The title compound was prepared according to general method **C** from mono alkyl vinylphosphonate **S27** (0.60 g, 2.94 mmol) and 1,5-dibromopentane (0.23 g, 0.98 mmol) in 64% yield (0.30 g, 0.63 mmol) as a colourless oil.

^{1}H NMR (401 MHz, $CDCl_3$): 6.34–5.97 (m, 6H, C*H*=C*H*$_2$), 5.44–5.37 (m, 2H, CH_3CH_2C*H*$CHCH_2CH_2CH_2O$), 5.32–5.25 (m, 2H, CH_3CH_2CHC*H*$CH_2CH_2CH_2O$), 4.04–3.98 (m, 4H, $CH_3CH_2CHCHCH_2CH_2$C*H*$_2$O, OC*H*$_2CH_2CH_2CH_2$C*H*$_2$O), 2.15–2.09 (m, 4H, CH_3CH_2CHCHC*H*$_2CH_2CH_2O$), 2.06–1.98 (m, 4H, CH_3C*H*$_2CHCHCH_2CH_2CH_2O$), 1.76–1.67 (m, 8H, $CH_3CH_2CHCHCH_2$C*H*$_2CH_2O$, OCH_2C*H*$_2CH_2$C*H*$_2CH_2O$), 1.50–1.41 (m, 2H, OCH_2CH_2C*H*$_2CH_2CH_2O$), 0.95 (t, 6H, *J* = 7.6 Hz, C*H*$_3CH_2CHCHCH_2CH_2CH_2O$).

^{13}C NMR (101 MHz, $CDCl_3$): 135.77 (d, *J* = 2.0 Hz, CH=*C*H$_2$), 133.02 (CH_3CH_2*C*$HCHCH_2CH_2CH_2O$), 127.49 (CH_3CH_2CH*C*$HCH_2CH_2CH_2O$), 125.86 (d, *J* = 184.5 Hz, *C*H=CH$_2$), 65.60, 65.50 (d, *J* = 6.1 Hz, $CH_3CH_2CHCHCH_2CH_2$*C*H$_2$O, OCH_2CH_2*C*$H_2CH_2CH_2O$), 30.63, 30.14 (d, *J* = 6.3 Hz, $CH_3CH_2CHCHCH_2$*C*H_2CH_2O, OCH_2*C*H_2CH_2*C*H_2CH_2O), 23.22 (CH_3CH_2CHCH*C*$H_2CH_2CH_2O$), 21.84 (OCH_2CH_2*C*$H_2CH_2CH_2O$), 20.65 (CH_3*C*$H_2CHCHCH_2CH_2CH_2O$), 14.43 (*C*$H_3CH_2CHCHCH_2CH_2CH_2O$).

^{31}P{^{1}H} NMR (162 MHz, $CDCl_3$): 20.15.

IR ν_{max} (KBr) 3090 (vw), 2964 (s), 2935 (m), 2874 (m), 1653 (vw), 1614 (w), 1463 (m), 1457 (m), 1405 (sh), 1279 (m), 1242 (s), 1072–1012 (s), 989 (vs).

HR-MS(ESI$^+$): For $C_{23}H_{43}O_6P_2$ $(M+H)^+$ *m*/*z* calculated 477.25294, found 477.25250.

^{1}H NMR spectrum of compound **S50**

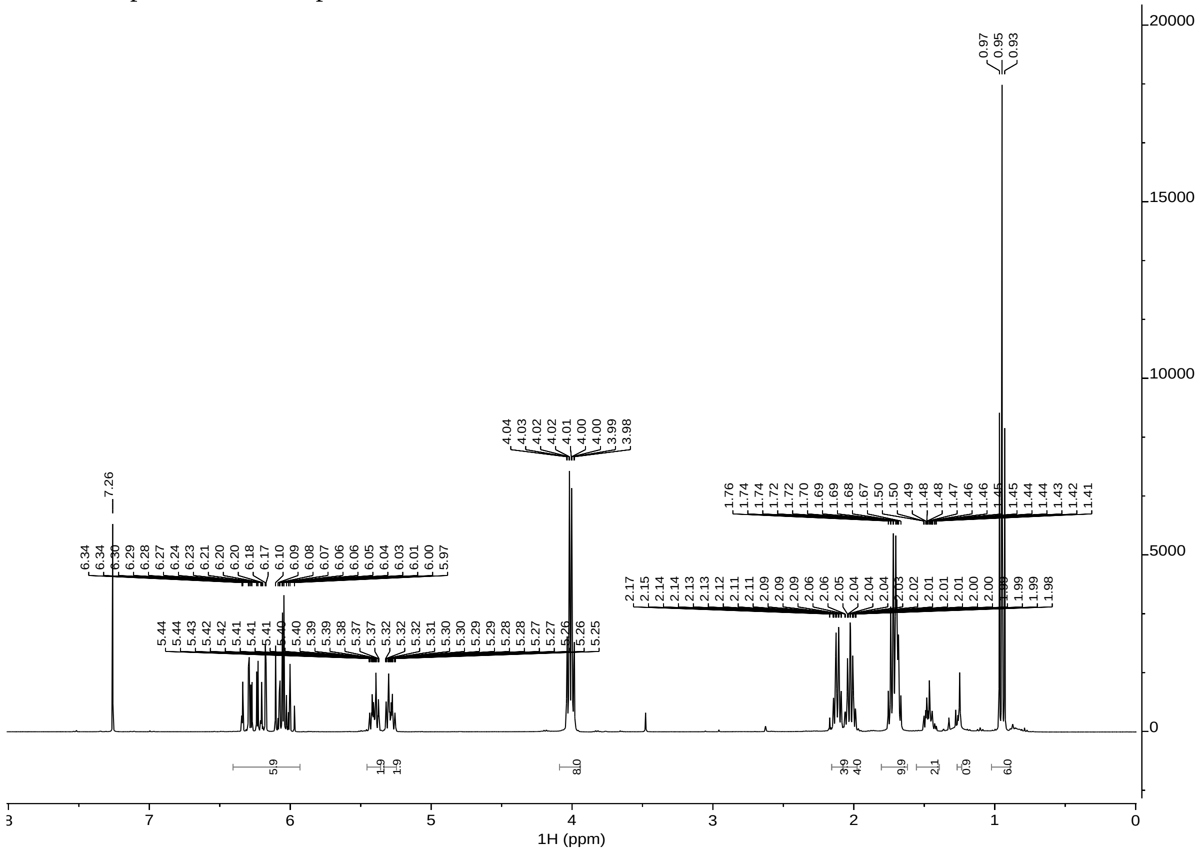

^{13}C NMR spectrum of compound **S50**

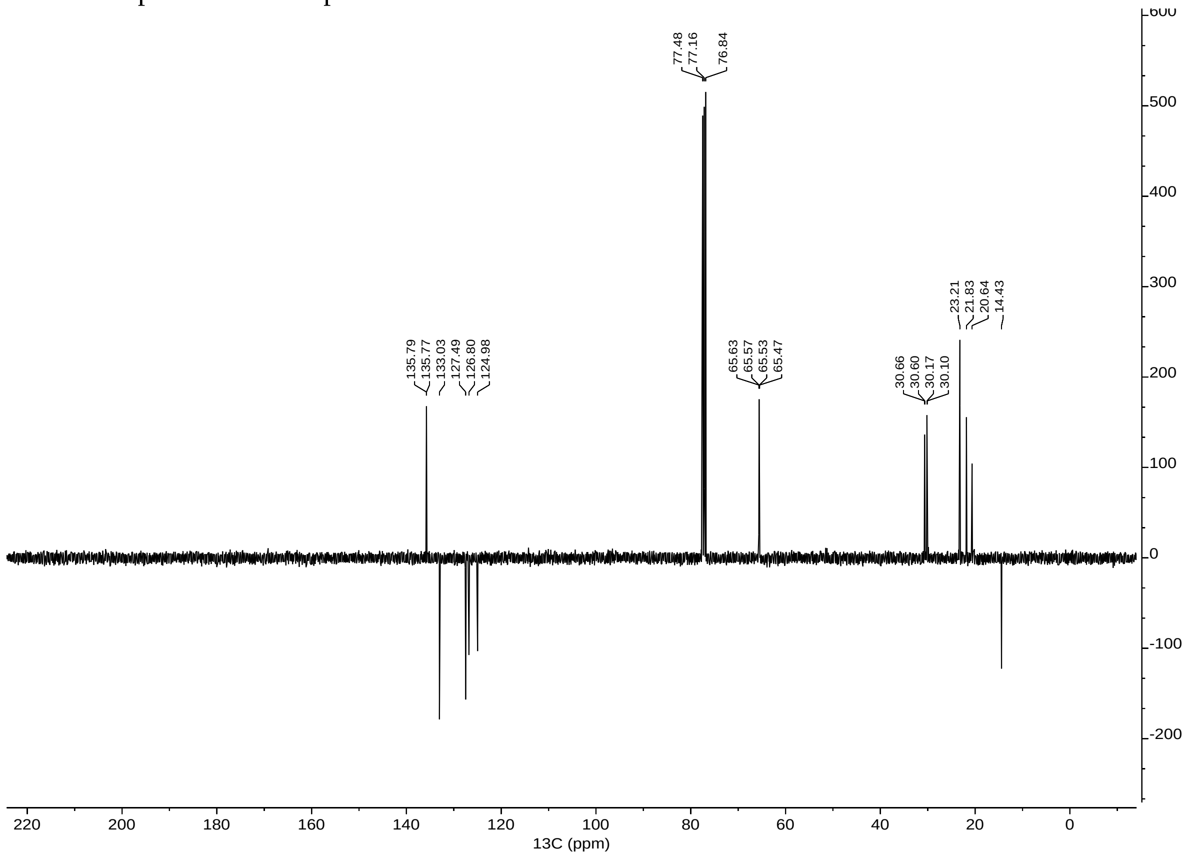

^{31}P{^{1}H} NMR spectrum of compound **S50**

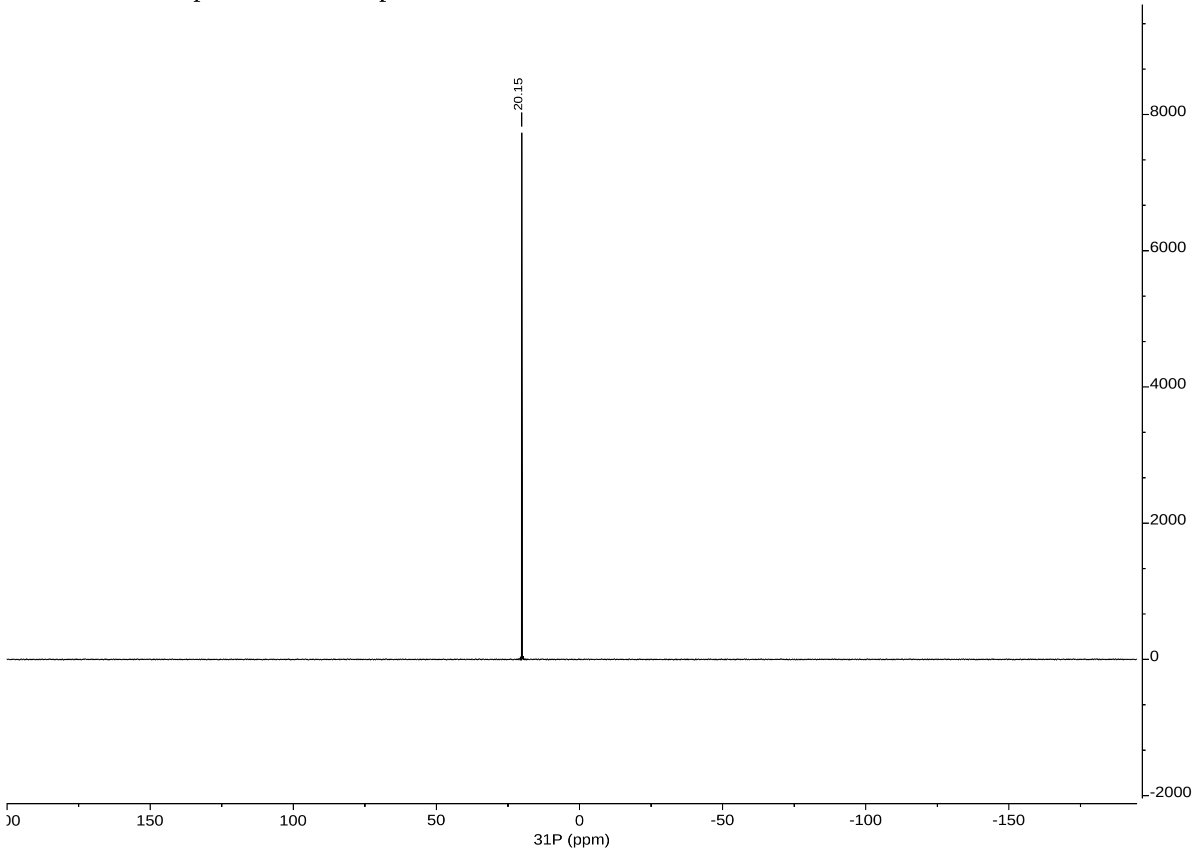

Bis((*Z*)-oct-3-en-1-yl) pentane-1,5-diyl bis(vinylphosphonate) (S51)

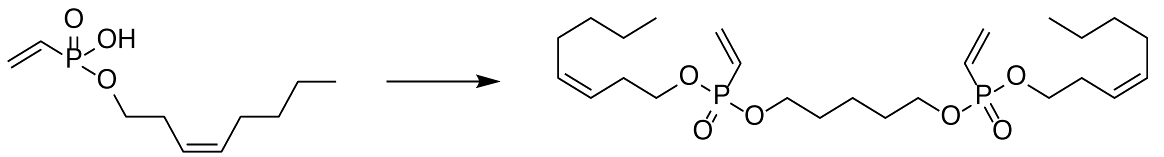

The title compound was prepared according to general method **C** from mono alkyl vinylphosphonate **S30** (0.9 g, 4.12 mmol) and 1,5-dibromopentane (0.32 g, 1.37 mmol) in 79% yield (0.56 g, 1.11 mmol) as a colourless oil.

^{1}H NMR (400 MHz, $CDCl_3$): 6.35–5.97 (m, 6H, $C\mathit{H}{=}C\mathit{H}_2$), 5.54–5.47 (m, 2H, $CH_3(CH_2)_3C\mathit{H}CHCH_2CH_2O$), 5.37–5.30 (m, 2H, $CH_3(CH_2)_3CHC\mathit{H}CH_2CH_2O$), 4.04–3.97 (m, 8H, $OC\mathit{H}_2CH_2CH_2CH_2C\mathit{H}_2O$, $CH_3(CH_2)_2CH_2CHCHCH_2C\mathit{H}_2O$), 2.45–2.35 (m, 4H, $CH_3(CH_2)_2CH_2CHCHC\mathit{H}_2CH_2O$), 2.06–2.00 (m, 4H, $CH_3(CH_2)_2C\mathit{H}_2CHCHCH_2CH_2O$), 1.65–1.73–1.65 (m, 4H, $OCH_2C\mathit{H}_2CH_2C\mathit{H}_2CH_2O$), 1.50–1.42 (m, 2H, $OCH_2CH_2C\mathit{H}_2CH_2CH_2O$), 1.36–1.25 (m, 8H, $CH_3(C\mathit{H}_2)_2CH_2CHCHCH_2CH_2O$), 0.94–0.86 (m, 6H, $C\mathit{H}_3(CH_2)_3CHCHCH_2CH_2O$).

^{13}C NMR (101 MHz, $CDCl_3$): 135.77 (d, J = 2.0 Hz, $CH{=}\mathit{C}H_2$), 133.42 ($CH_3(CH_2)_3\mathit{C}HCHCH_2CH_2O$), 125.89 (d, J = 184.1 Hz, $\mathit{C}H{=}CH_2$), 123.82 ($CH_3(CH_2)_3CH\mathit{C}HCH_2CH_2O$), 65.48 (d, J = 6.0 Hz, $CH_3(CH_2)_2CH_2CHCHCH_2\mathit{C}H_2O$), 65.28 (d, J = 5.9 Hz, $O\mathit{C}H_2CH_2CH_2CH_2\mathit{C}H_2O$), 31.85 ($CH_3CH_2\mathit{C}H_2CH_2CHCHCH_2CH_2O$), 30.13 (d, J = 6.7 Hz, $OCH_2\mathit{C}H_2CH_2\mathit{C}H_2CH_2O$), 28.82 (d, J = 6.6 Hz, $CH_3(CH_2)_2CH_2CHCHC\mathit{H}_2CH_2O$), 27.22 $CH_3(CH_2)_2\mathit{C}H_2CHCHCH_2CH_2O$), 22.46 $CH_3\mathit{C}H_2(CH_2)_2CHCHCH_2CH_2O$, 21.83 ($OCH_2CH_2\mathit{C}H_2CH_2CH_2O$), 14.11 ($\mathit{C}H_3(CH_2)_3CHCHCH_2CH_2O$).

^{31}P{^{1}H} NMR (162 MHz, $CDCl_3$): 19.87.

IR ν_{max} (KBr) 3086 (w), 3011 (m), 2957 (s), 2929 (s), 2872 (m), 2860 (m), 1613 (w), 1467 (m), 1363 (w), 1278 (m, sh), 1251 (vs), 1184 (w), 1054 (s), 1007 (vs), 970 (s, sh), 726 (m), 608 (m).

HR-MS(APCI$^+$): For $C_{25}H_{47}O_6P_2$ $(M+H)^+$ m/z calculated 505.28424, found 505.28375.

^{1}H NMR spectrum of compound **S51**

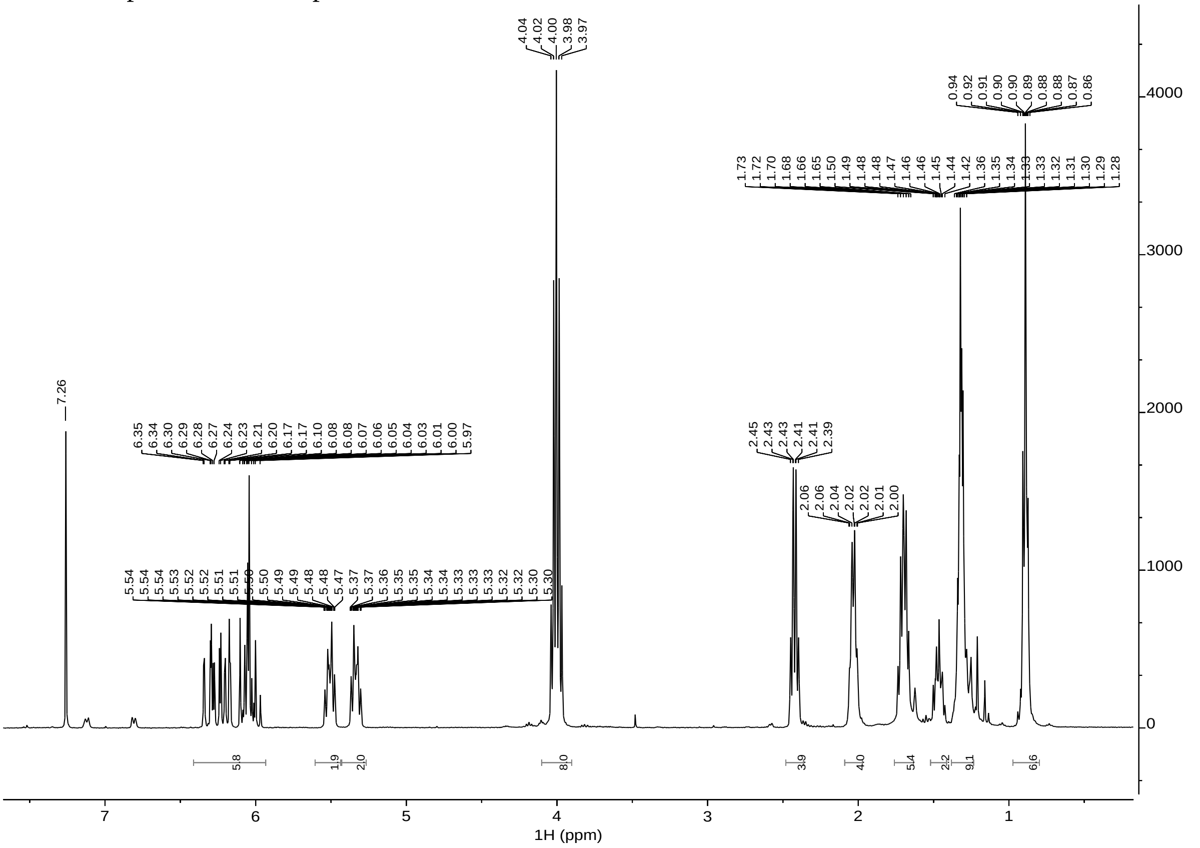

^{13}C NMR spectrum of compound **S51**

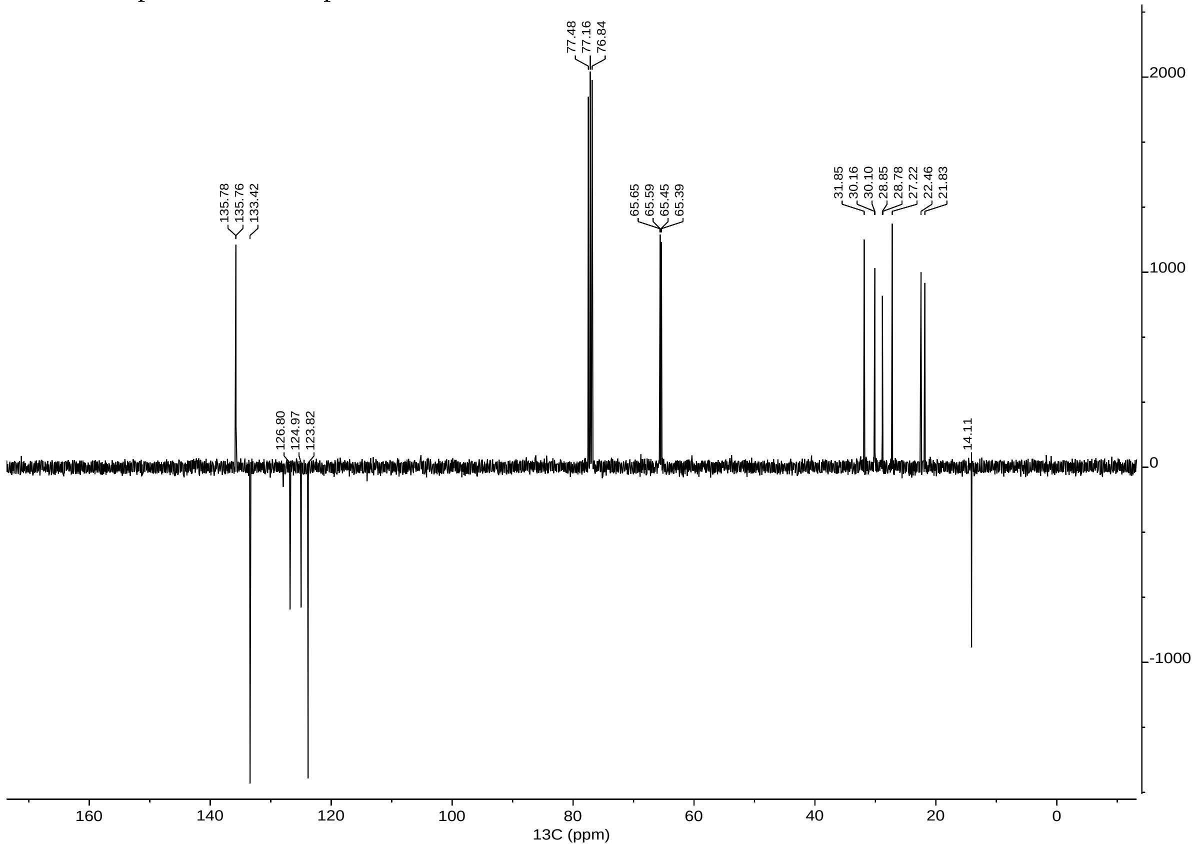

$^{31}P\{^{1}H\}$ NMR spectrum of compound **S51**

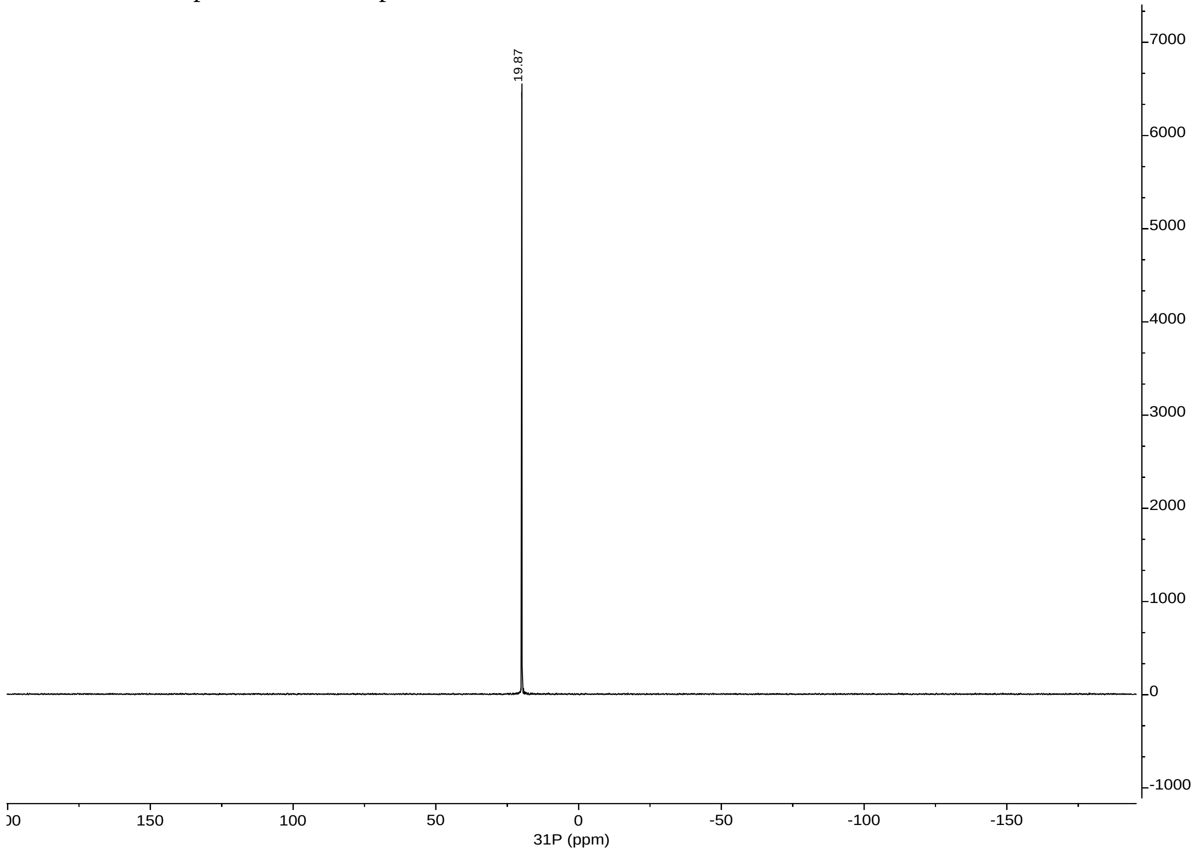

Bis((*Z*)-non-3-en-1-yl) pentane-1,5-diyl bis(vinylphosphonate) (S52)

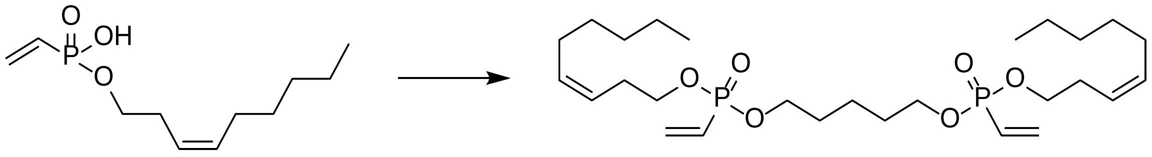

The title compound was prepared according to general method **C** from mono alkylvinylphosphonate **S32** (1.50 g, 6.45 mmol) and 1,5-dibromopentane (0.43 g, 2.15 mmol) in 70% yield (0.76 g, 1.51 mmol) as a colourless oil.

^{1}H NMR (401 MHz, $CDCl_3$): 6.34–5.96 (m, 6H, C*H*=C*H*$_2$), 5.54–5.47 (m, 1H, $CH_3(CH_2)_4$C*H*CHCH_2CH_2O), 5.36–5.29 (m, 2H, $CH_3(CH_2)_4$CHC*H*CH_2CH_2O), 4.03–3.96 (m, 8H, $CH_3(CH_2)_4$CHCHCH_2C*H*$_2$O, OC*H*$_2$CH_2CH_2CH_2C*H*$_2$O), 2.42–2.39 (m, 4H, $CH_3(CH_2)_4$CHCHC*H*$_2$CH_2O), 2.05–1.99 (m, 4H, $CH_3(CH_2)_3$C*H*$_2$CHCHCH_2CH_2O), 1.69 (dt, 4H, *J* = 14.7, 6.7 Hz, OCH_2C*H*$_2$CH_2C*H*$_2$CH_2O), 1.42–1.50 (m, 2H, OCH_2CH_2C*H*$_2$CH_2CH_2O), 1.21–1.38 (m, 12H, CH_3(C*H*$_2$)$_3$CH_2CHCHCH_2CH_2O), 0.90–0.86 (m, 6H, C*H*$_3$(CH_2)$_3$CHCHCH_2CH_2CH_2O).

^{13}C NMR (101 MHz, $CDCl_3$): 135.79 (d, *J* = 2.2 Hz, CH=*C*H_2), 133.47 ($CH_3(CH_2)_4$*C*HCHCH_2CH_2O), 125.87 (d, *J* = 184.3 Hz, *C*H=CH_2), 123.79 ($CH_3(CH_2)_3$CH*C*HCH_2CH_2O), 65.61, 65.41 (d, *J* = 5.9 Hz, O*C*H_2CH_2CH_2CH_2*C*H_2O, $CH_3(CH_2)_4$CHCHCH_2*C*H_2O), 31.61 (CH_3CH_2*C*H_2CH_2CH_2CHCHCH_2CH_2O), 30.12 (d, *J* = 6.3 Hz, OCH_2*C*H_2CH_2*C*H_2CH_2O), 29.35 ($CH_3CH_2CH_2$*C*H_2CH_2CHCHCH_2CH_2O), 28.81 (d, *J* = 6.3 Hz, $CH_3(CH_2)_4$CHCH*C*H_2CH_2O), 27.47 ($CH_3(CH_2)_3$*C*H_2CHCHCH_2CH_2O), 22.69 (CH_3*C*H_2CH_2CH_2CH_2CHCHCH_2CH_2O), 21.82 (OCH_2CH_2*C*H_2CH_2CH_2O), 14.19 (*C*$H_3(CH_2)_4$CHCHCH_2CH_2O).

^{31}P{^{1}H} NMR (162 MHz, $CDCl_3$): 20.12.

IR ν_{max} (KBr) 3090 (vw), 2960 (s), 2930 (s), 2873 (m), 2859 (m), 1654 (vw), 1614 (w), 1468 (m), 1460 (w, sh), 1436 (w), 1400 (m), 1380 (w), 1279 (m), 1241 (s), 1070 (s, sh), 1055 (s), 1009 (vs), 990 (vs).

HR-MS(ESI$^+$): For $C_{27}H_{50}O_6NaP_2$ $(M+Na)^+$ *m*/*z* calculated 555.29748, found 555.29742.

^{1}H NMR spectrum of compound **S52**

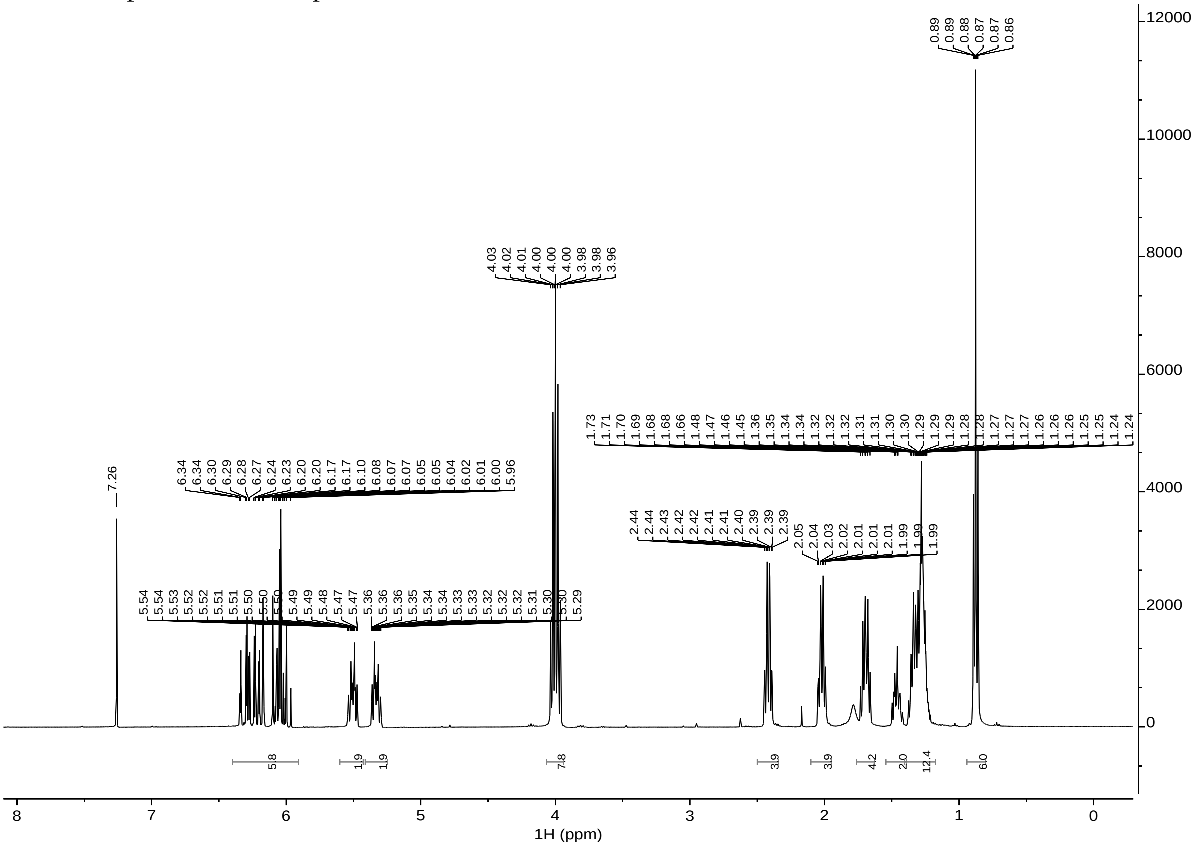

^{13}C NMR spectrum of compound **S52**

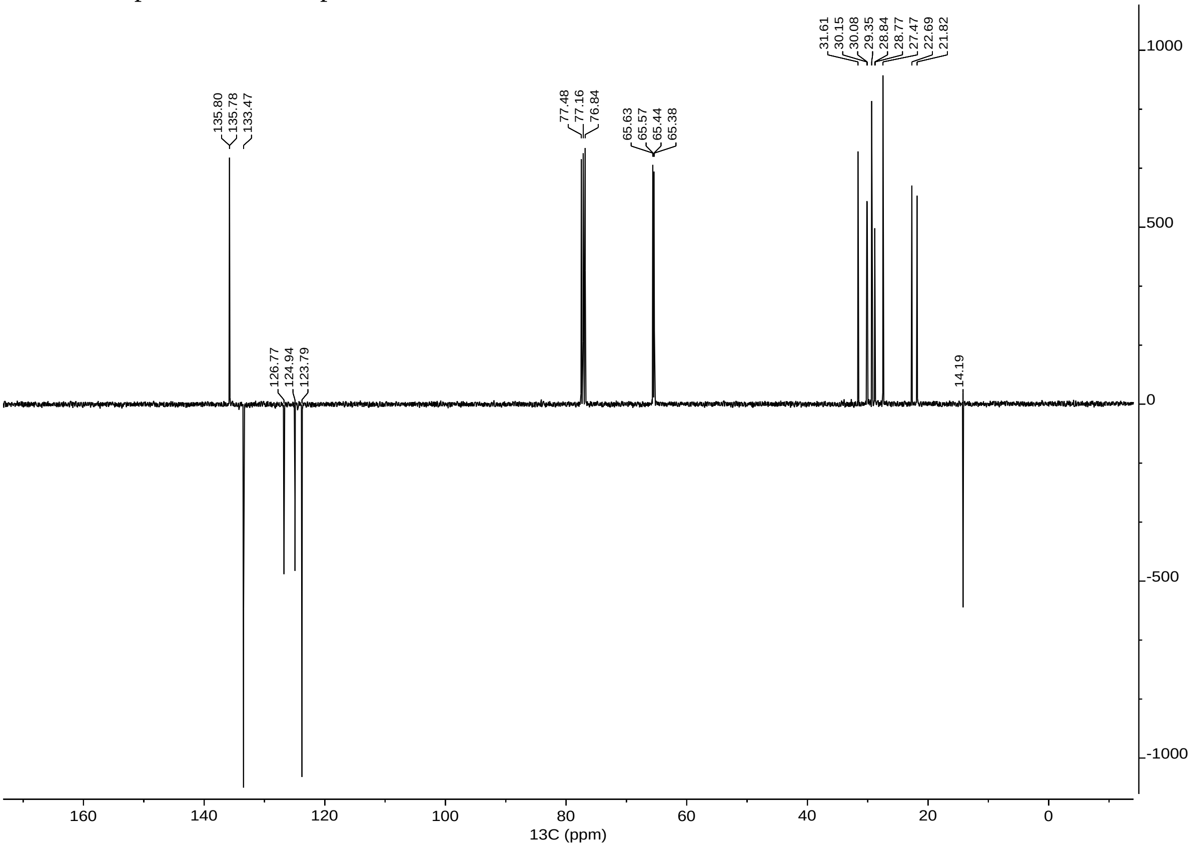

^{31}P{^{1}H} NMR spectrum of compound **S52**

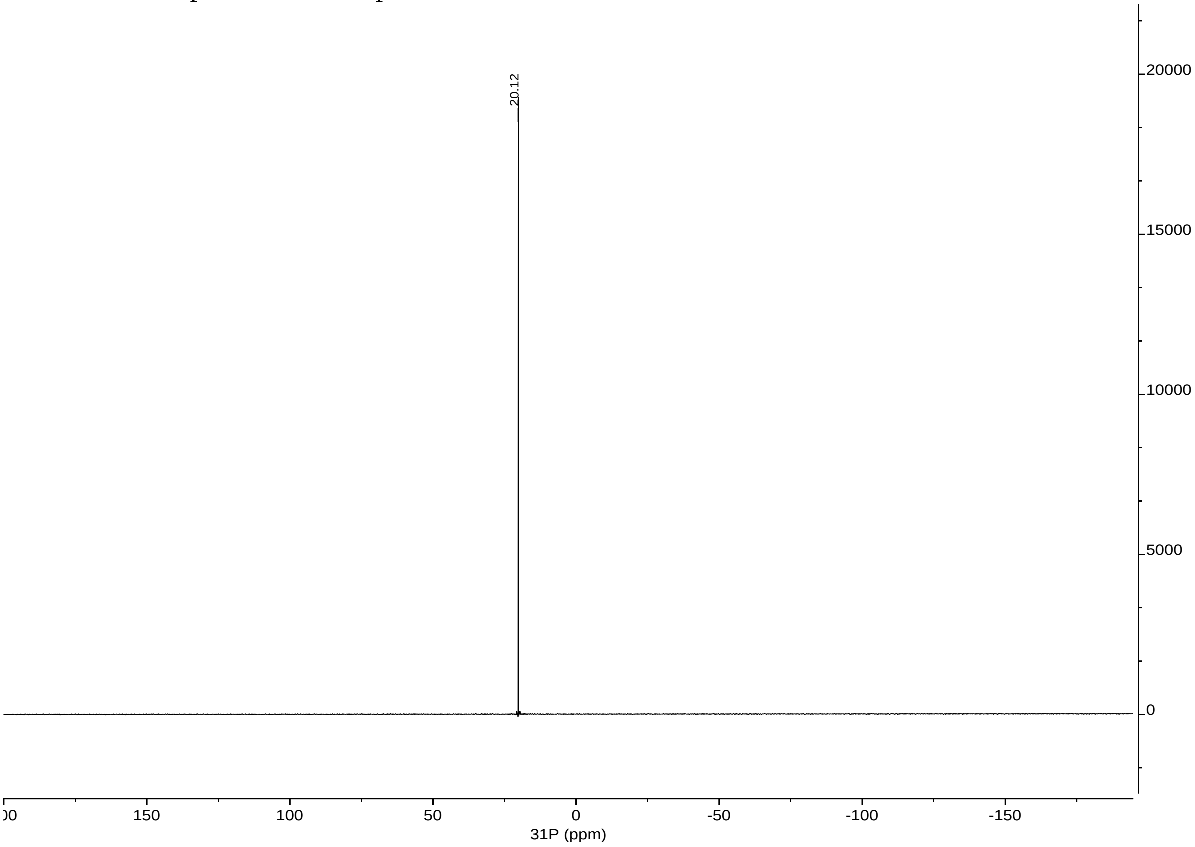

Bis((adamantan-1-yl)methyl) pentane-1,5-diyl bis(vinylphosphonate) (S53)

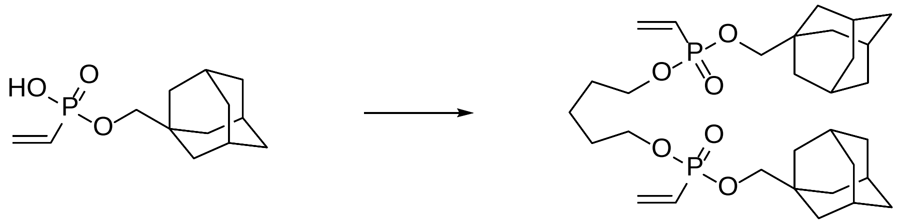

The title compound was prepared according to general method **C** from mono alkyl vinylphosphonate **S35** (1.91 g, 9.95 mmol) and 1,5-dibromopentane (0.42 mL, 3.07 mmol) in 81% yield (1.44 g, 2.48 mmol) as a colourless oil.

^{1}H NMR (401 MHz, $CDCl_3$) δ 6.34–5.95 (m, 6H, C*H*=C*H*$_2$), 4.01 (q, *J* = 6.7 Hz, 4H, OC*H*$_2$CH$_2$), 3.55 (d, *J* = 5.4 Hz, 4H, OC*H*$_2$C$_{quat}$), 1.97 (p, *J* = 3.1 Hz, 6H, C$_{quat}$CH$_2$C*H*), 1.75–1.58 (m, 16H, C$_{quat}$C*H*$_2$CH, OCH$_2$C*H*$_2$), 1.52 (d, *J* = 2.9 Hz, 12H, *c*-(CHC*H*$_2$)$_3$), 1.50–1.40 (m, 2H, O(CH$_2$)$_2$C*H*$_2$).

^{13}C NMR (101 MHz, $CDCl_3$) δ 135.71 (d, *J* = 2.0 Hz, =*C*H$_2$), 125.71 (d, *J* = 184.5 Hz, P*C*H), 75.56 (d, *J* = 6.3 Hz, O*C*H$_2$CH$_2$), 65.62 (d, *J* = 5.9 Hz, O*C*H$_2$C$_{quat}$), 39.00 (*c*-(CH*C*H$_2$)$_3$), 37.04 (C$_{quat}$*C*H$_2$CH), 33.94 (d, *J* = 7.0 Hz, *C*$_{quat}$), 30.13 (d, *J* = 6.3 Hz, OCH$_2$*C*H$_2$), 28.09 (C$_{quat}$CH$_2$*C*H), 21.84 (O(CH$_2$)$_2$*C*H$_2$).

^{31}P{^{1}H} NMR (162 MHz, $CDCl_3$) δ 20.27.

IR ν_{max} ($CHCl_3$) 3091 (vw), 2927 (s), 2905 (vs), 2849 (s), 2753 (vw), 2675 (vw), 2660 (vw), 2637 (vw), 1614 (w), 1451 (m), 1400 (m), 1363 (vw), 1347 (w), 1278 (w), 1107 (w), 1071 (m), ~1052 (s, sh), 1043 (s), 1011 (s), 986 (s), 974 (s), 815 (w).

HR-MS(ESI$^+$): For $C_{31}H_{51}O_6P_2$ (M+H)$^+$ *m/z* calculated 581.31554, found 581.31451. For $C_{31}H_{50}O_6NaP_2$ (M+Na)$^+$ *m/z* calculated 603.29748, found 603.29610.

^{1}H NMR spectrum of compound **S53**

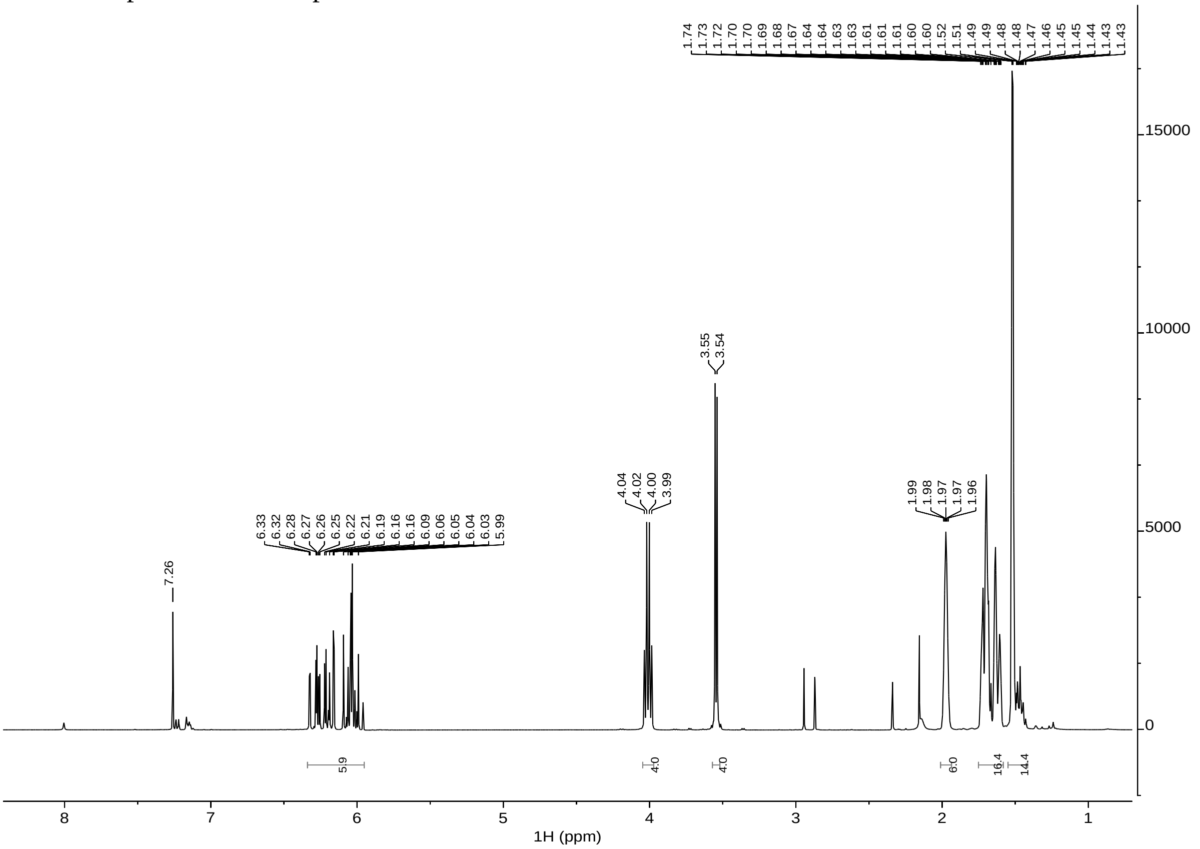

^{13}C NMR spectrum of compound **S53**

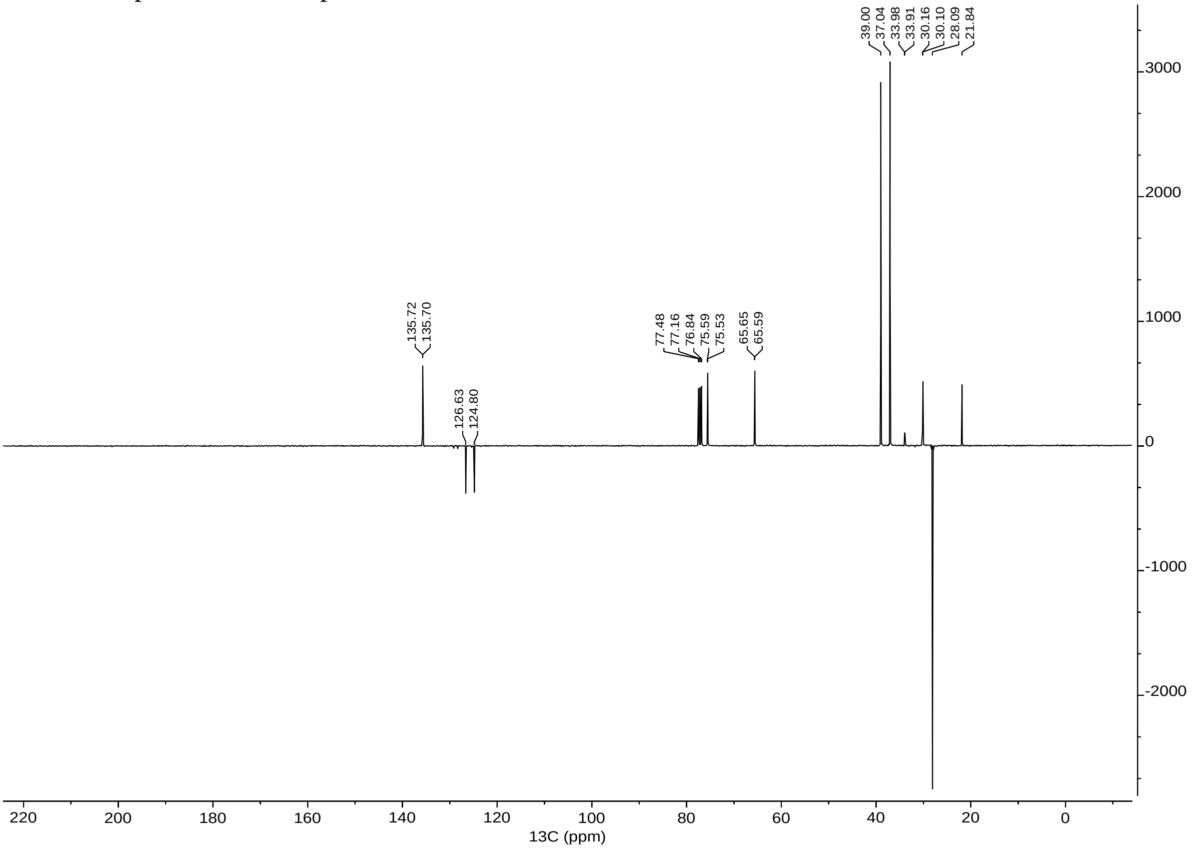

^{31}P{^{1}H} NMR spectrum of compound **S53**

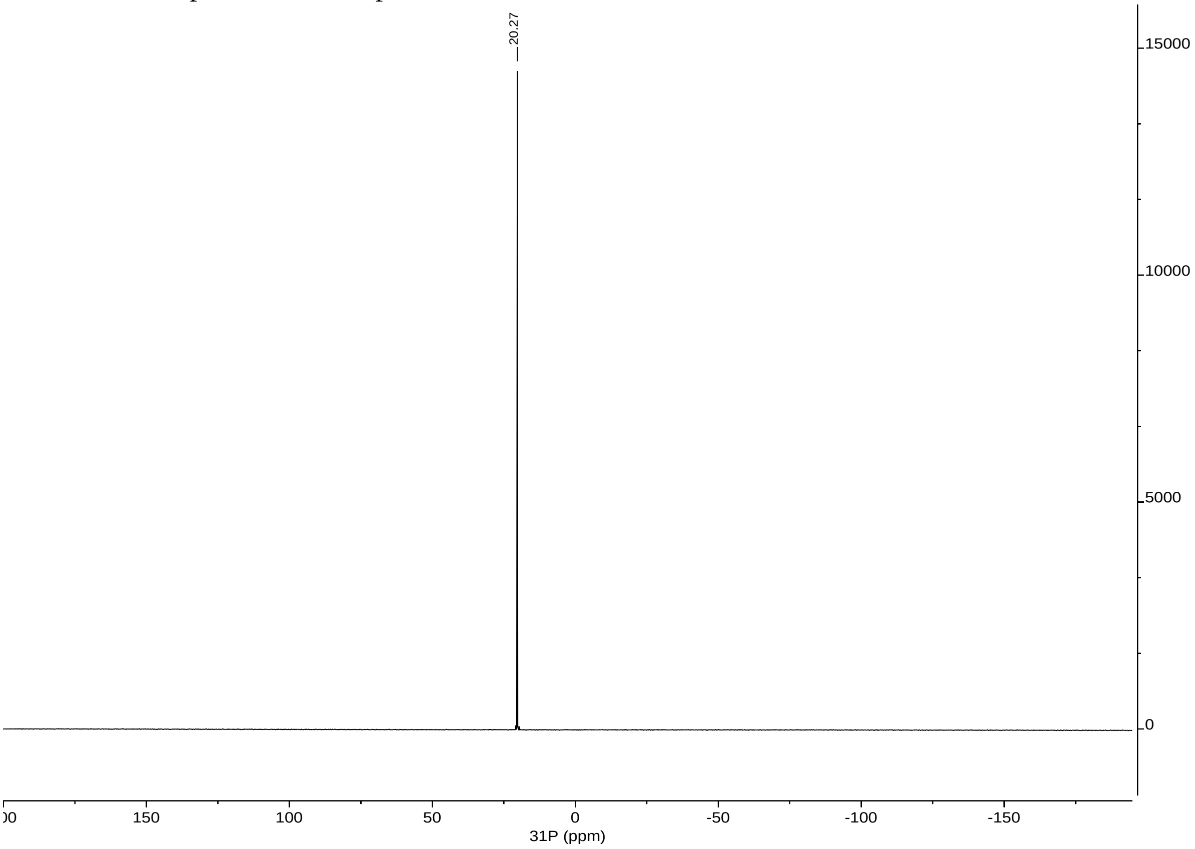

Hexane-1,6-diyl dihexyl bis(vinylphosphonate) (S54)

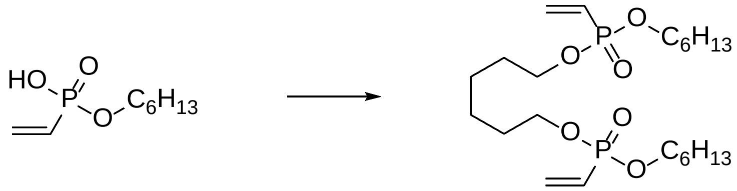

The title compound was prepared according to general method **C** from mono alkyl vinylphosphonate **S23** (1.91 g, 9.95 mmol) and 1,6-dibromohexane (96%, 0.53 mL, 3.32 mmol) in 55% yield (0.85 g, 1.83 mmol) as a colourless oil.

^{1}H NMR (400 MHz, $CDCl_3$) δ 6.37–5.93 (m, 6H, C*H*=CH_2), 4.00 (q, *J* = 6.8 Hz, 8H, OC*H*$_2$), 1.66 (dq, *J* = 8.0, 6.5 Hz, 8H, OCH_2C*H*$_2$), 1.42–1.23 (m, 16H, O(CH_2)$_2$C*H*$_2$, (C*H*$_2$)$_2$$CH_3$), 0.91–0.85 (m, 6H, C*H*$_3$).

^{13}C NMR (101 MHz, $CDCl_3$) δ 135.43 (=*C*H_2), 125.89 (d, *J* = 184.0 Hz, P*C*H), 65.96 (d, *J* = 5.9 Hz), 65.62 (d, *J* = 5.8 Hz, O*C*H_2), 31.32 (*C*$H_2CH_2CH_3$), 30.43 (d, *J* = 6.7 Hz), 30.36 (d, *J* = 6.6 Hz, OCH_2*C*H_2), 25.16 (O(CH_2)$_2$*C*H_2), 22.51 (*C*H_2CH_3), 13.97 (*C*H_3).

^{31}P{^{1}H} NMR (162 MHz, $CDCl_3$) δ 19.80.

IR ν_{max} ($CHCl_3$) 3090 (w), 2959 (s), 2934 (s), 2872 (m), 2861 (m), 1614 (w), 1468 (m), 1400 (m), 1380 (w), 1279 (m), 1241 (s), 1058 (s), 1039 (s), 998 (vs), 861 (m).

HR-MS(ESI^{+}): For $C_{22}H_{45}O_6P_2$ $(M+H)^+$ *m/z* calculated 467.26859, found 467.26808. For $C_{22}H_{44}O_6NaP_2$ $(M+Na)^+$ *m/z* calculated 489.25053, found 489.24980.

^{1}H NMR spectrum of compound **S54**

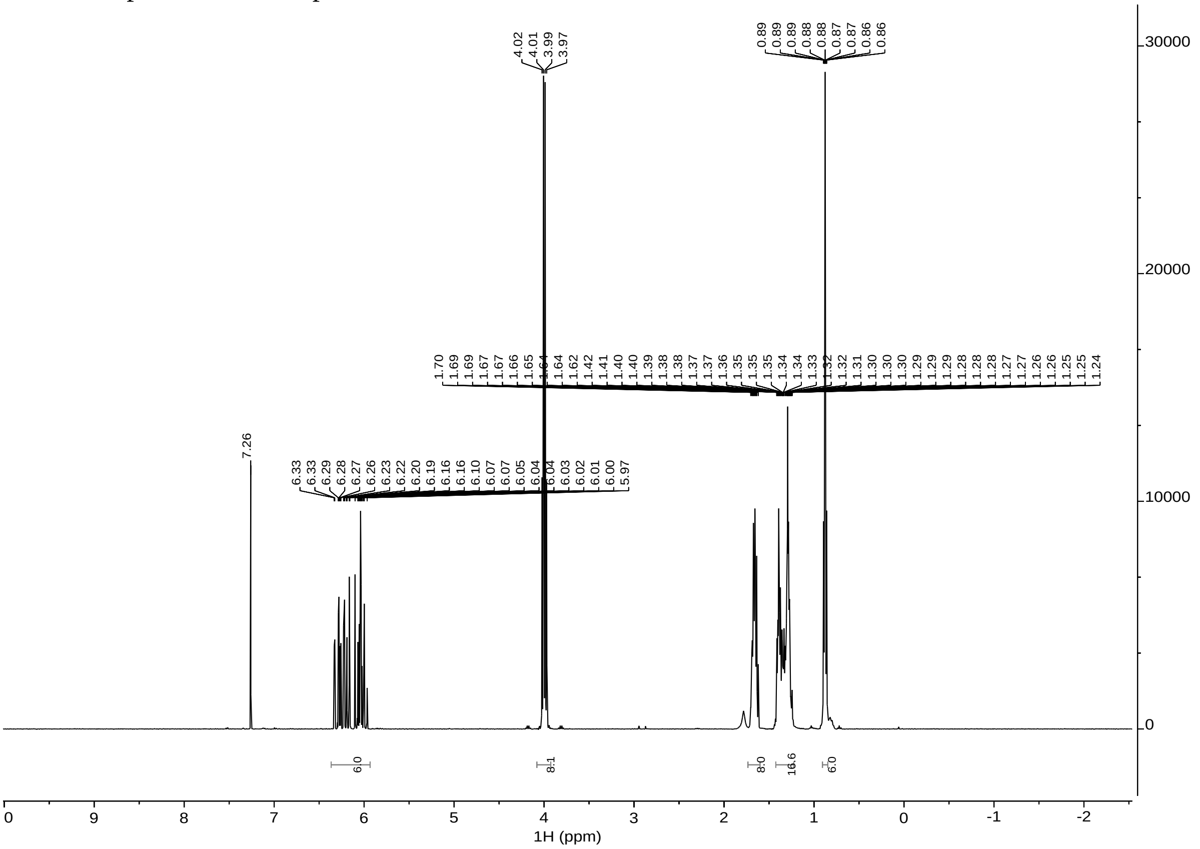

^{13}C NMR spectrum of compound **S54**

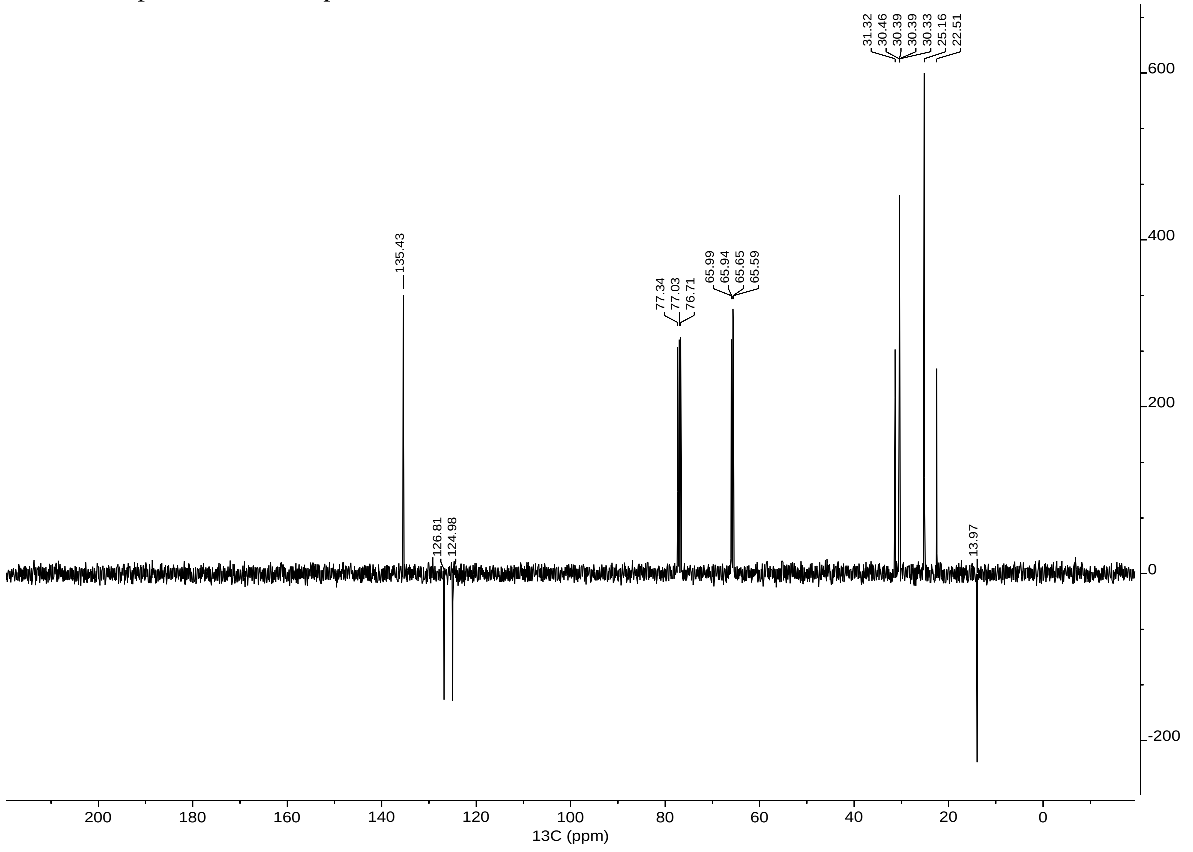

^{31}P{^{1}H} NMR spectrum of compound **S54**

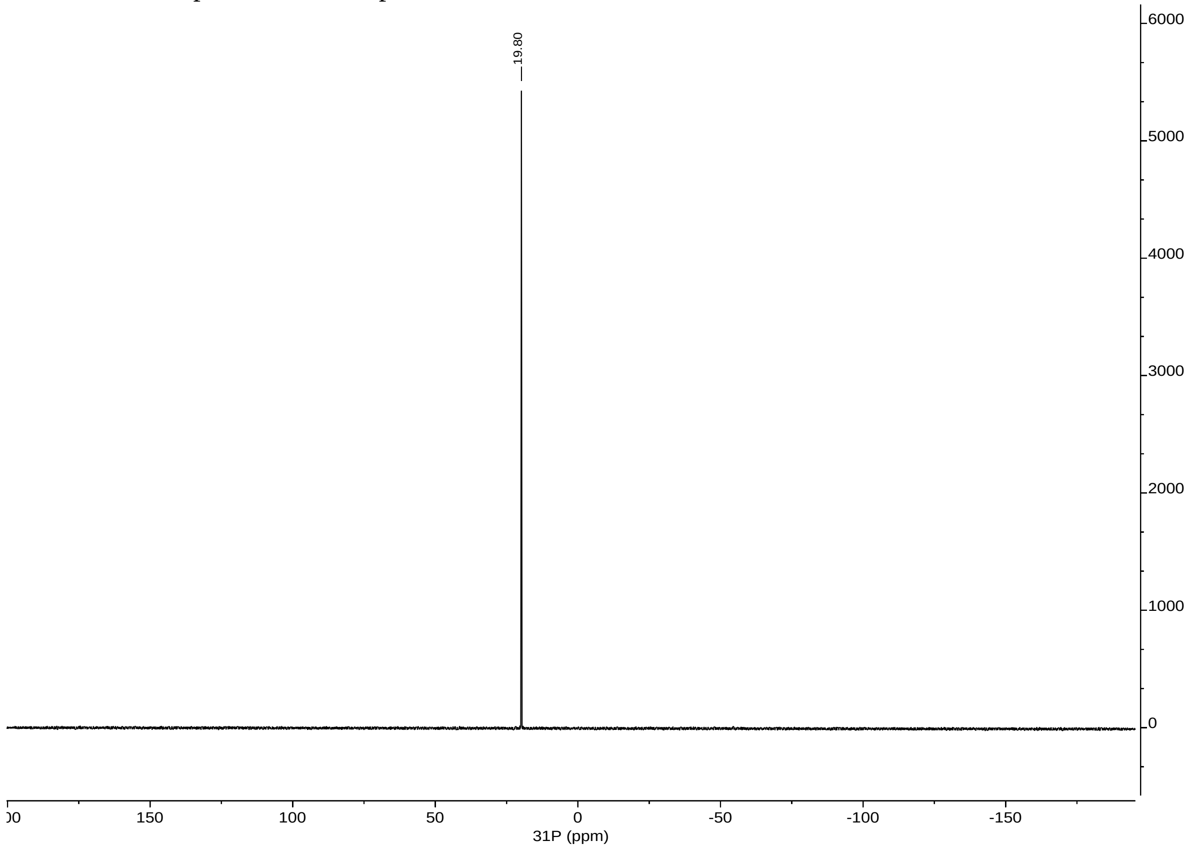

Bis((*Z*)-hept-3-en-1-yl) hexane-1,6-diyl bis(vinylphosphonate) (S55)

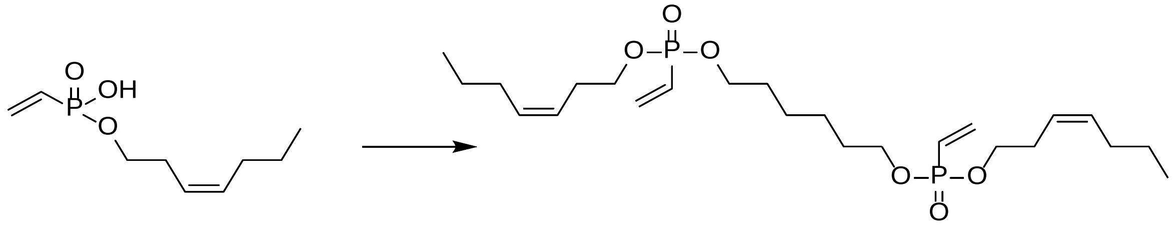

The title compound was prepared according to general method **C** from mono alkyl vinylphosphonate **S26** (1.10 g, 5.39 mmol) and 1,6-dibromohexane (0.44 g, 1.80 mmol) in 79% yield (0.68 g, 1.42 mmol) as a colourless oil.

^{1}H NMR (400 MHz, $CDCl_3$): 6.34–5.97 (m, 6H, C*H*=C*H*$_2$), 5.54–5.47 (m, 2H, C*H*=CH(CH_2)$_2$O), 5.38–5.31 (m, 2H, CH=C*H*CH_2CH_2O), 4.03–3.96 (m, 8H, C*H*$_2$O), 2.45–2.39 (m, 4H, =CHC*H*$_2$$CH_2$O), 2.04–1.98 (m, 4H, CH_3CH_2C*H*$_2$), 1.70–1.62 (m, 4H, OCH_2C*H*$_2$(CH_2)$_2$C*H*$_2$$CH_2$O), 1.43–1.32 (m, 8H, CH_3C*H*$_2$, O(CH_2)$_2$ (C*H*$_2$)$_2$(CH_2)$_2$O), 0.89 (t, 6H, *J* = 7.4 Hz, C*H*$_3$).

^{13}C NMR (101 MHz, $CDCl_3$): 135.70 (d, *J* = 2.1 Hz, CH=*C*H$_2$), 133.18 (CH_3(CH_2)$_2$*C*H), 125.94 (d, *J* = 184.1 Hz, *C*H=CH_2), 124.05 (CH=*C*HCH_2CH_2O), 65.80 (d, *J* = 5.5 Hz, CH=CHCH_2*C*H$_2$O), 65.38 (d, *J* = 6.4 Hz, O*C*H$_2$(CH_2)$_4$*C*H$_2$O), 30.49 (d, *J* = 6.8 Hz, OCH_2*C*H$_2$(CH_2)$_2$*C*H$_2$$CH_2$O), 29.53 ($CH_3CH_2$*C*H$_2$), 28.83 (d, *J* = 6.6 Hz, CH=CH*C*H$_2$$CH_2$O), 25.29 (O($CH_2$)$_2$ (*C*H$_2$)$_2$(CH_2)$_2$O), 22.80 (CH_3*C*H$_2$), 13.88 (*C*H$_3$).

^{31}P{^{1}H} NMR (162 MHz, $CDCl_3$): 19.84.

IR ν_{max} (KBr) 3085 (vw), 3011 (m), 2958 (s), 2931 (m), 2871 (m), 1654 (w), 1613 (w), 1465 (m), 1398 (m), 1280 (m, sh), 1251 (vs), 1066 (vs), 1053 (vs), 1003 (vs), 976 (s, sh), 851 (m), 727 (m).

HR-MS(ESI$^+$): For $C_{24}H_{45}O_6P_2$ (M+H)$^+$ *m/z* calculated 491.26859, found 491.26863.

^{1}H NMR spectrum of compound **S55**

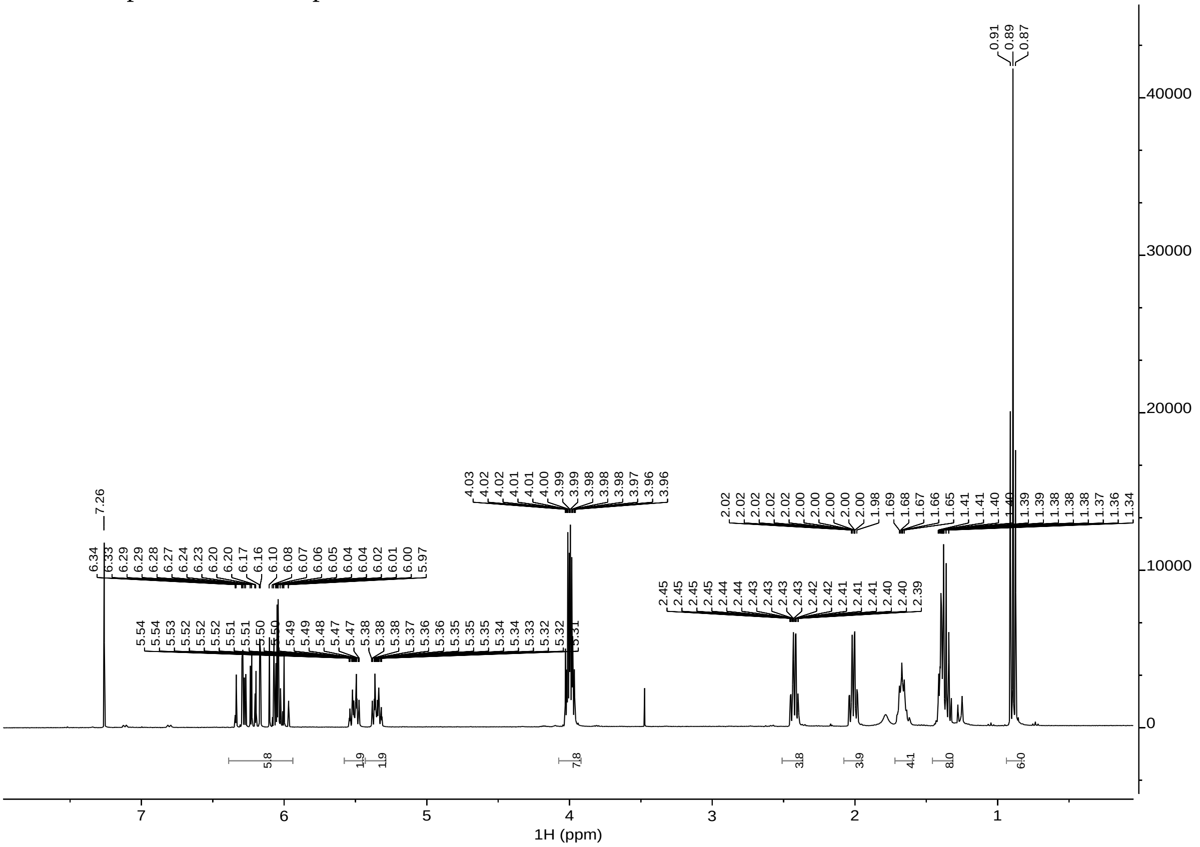

^{13}C NMR spectrum of compound **S55**

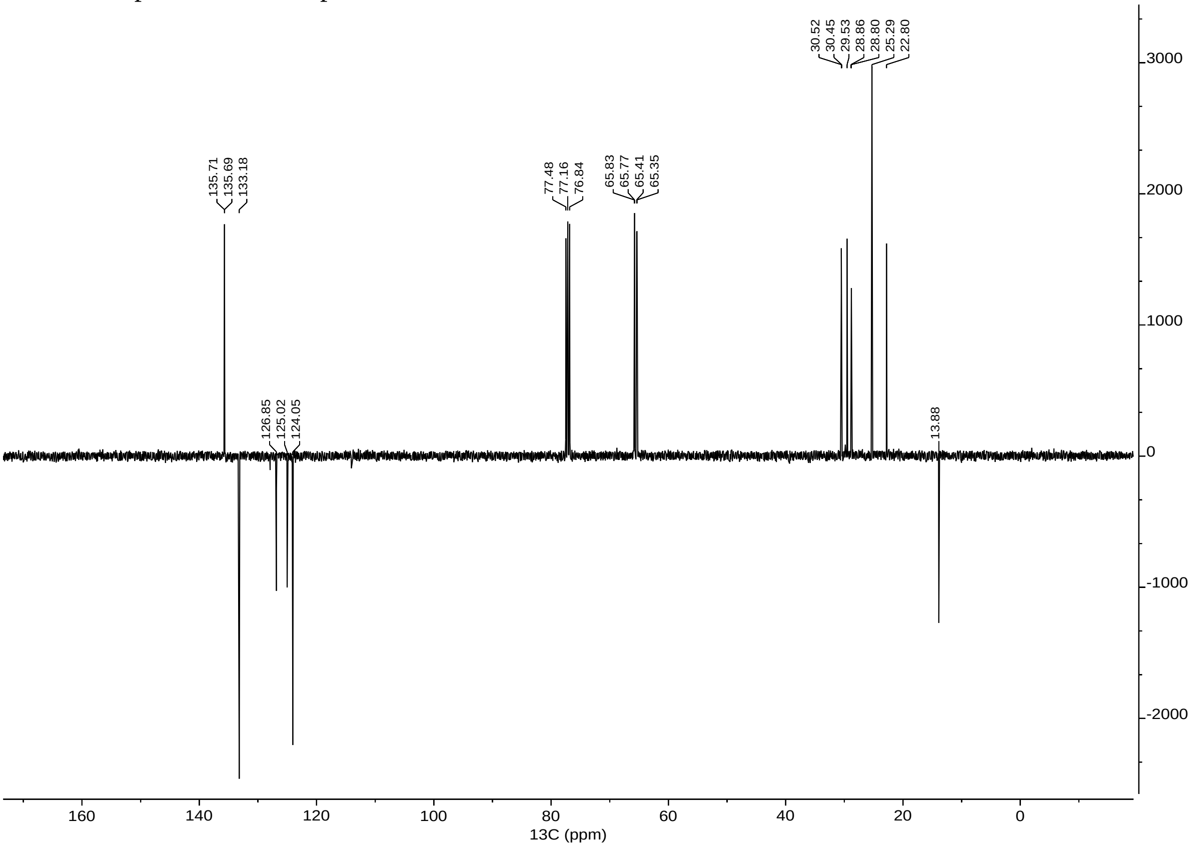

^{31}P{^{1}H} NMR spectrum of compound **S55**

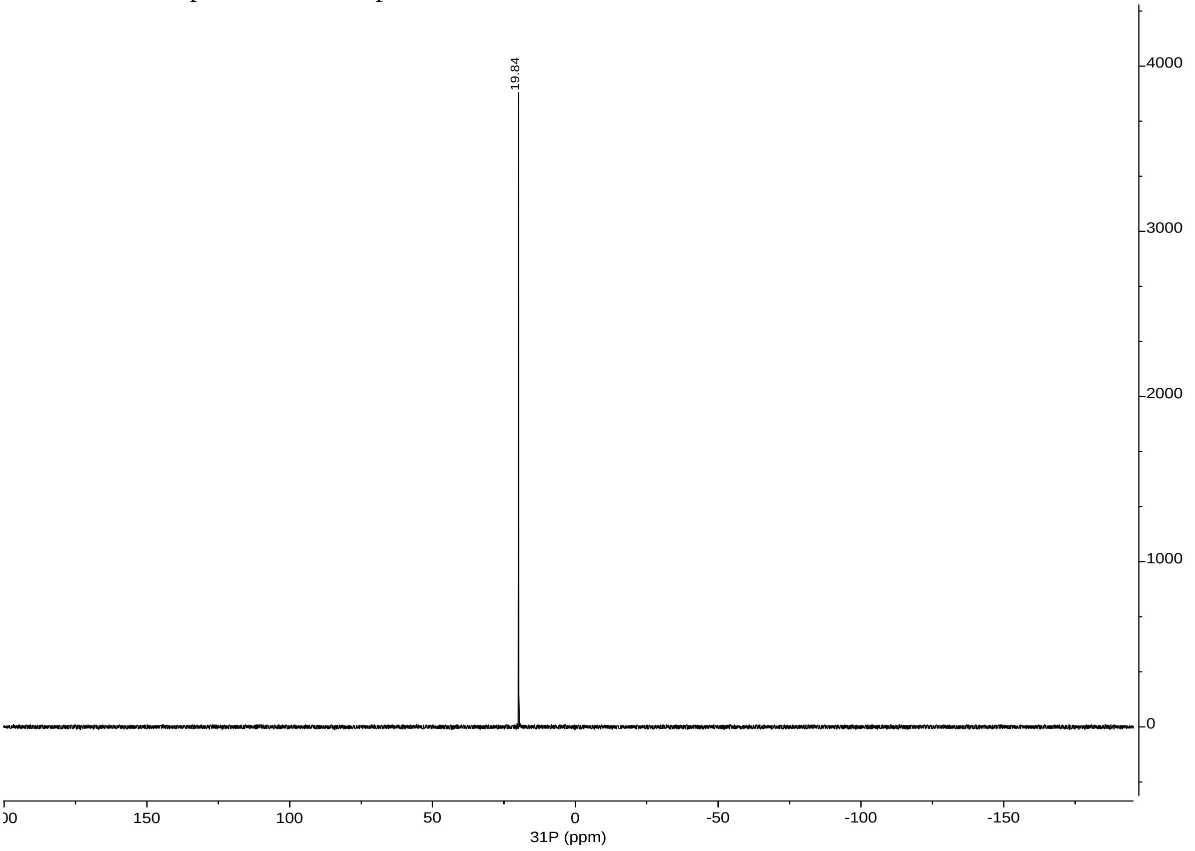

Bis((*Z*)-hept-4-en-1-yl) hexane-1,6-diyl bis(vinylphosphonate) (S56)

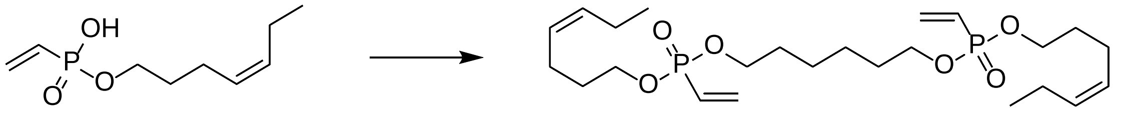

The title compound was prepared according to general method **C** from mono alkyl vinylphosphonate **S27** (0.60 g, 2.94 mmol) and 1,5-dibromohexane (0.24 g, 0.98 mmol) in 67% yield (0.32 g, 0.65 mmol) as a colourless oil.

^{1}H NMR (401 MHz, $CDCl_3$): 6.34–5.97 (m, 6H, $C\mathit{H}{=}C\mathit{H}_2$), 5.44–5.37 (m, 2H, $CH_3CH_2C\mathit{H}CHCH_2CH_2CH_2O$), 5.33–5.26 (m, 2H, $CH_3CH_2CHC\mathit{H}CH_2CH_2CH_2O$), 4.04–3.98 (m, 4H, $CH_3CH_2CHCHCH_2CH_2C\mathit{H}_2O$, $OC\mathit{H}_2CH_2CH_2CH_2CH_2C\mathit{H}_2O$), 2.15–2.09 (m, 4H, $CH_3CH_2CHCHC\mathit{H}_2CH_2CH_2O$), 2.07–1.99 (m, 4H, $CH_3C\mathit{H}_2CHCHCH_2CH_2CH_2O$), 1.76–1.64 (m, 8H, $CH_3CH_2CHCHCH_2C\mathit{H}_2CH_2O$, $OCH_2C\mathit{H}_2CH_2CH_2C\mathit{H}_2CH_2O$), 1.43–1.36 (m, 4H, $OCH_2CH_2C\mathit{H}_2C\mathit{H}_2CH_2CH_2O$), 0.95 (t, 6H, *J* = 7.6 Hz, $C\mathit{H}_3CH_2CHCHCH_2CH_2CH_2O$).

^{13}C NMR (101 MHz, $CDCl_3$): 135.69 (d, *J* = 2.0 Hz, $CH{=}\mathit{C}H_2$), 133.01 ($CH_3CH_2\mathit{C}HCHCH_2CH_2CH_2O$), 127.50 ($CH_3CH_2CH\mathit{C}HCH_2CH_2CH_2O$), 125.93 (d, *J* = 182.9 Hz, $\mathit{C}H{=}CH_2$), 65.79, 65.47 (d, *J* = 5.7 Hz, $O\mathit{C}H_2CH_2CH_2CH_2CH_2\mathit{C}H_2O$, $CH_3CH_2CHCHCH_2CH_2\mathit{C}H_2O$), 30.63, 30.50 (d, *J* = 6.4 Hz $CH_3CH_2CHCHCH_2\mathit{C}H_2CH_2O$, $OCH_2\mathit{C}H_2CH_2CH_2\mathit{C}H_2CH_2O$), 25.30 ($OCH_2CH_2\mathit{C}H_2\mathit{C}H_2CH_2CH_2O$), 23.22 ($CH_3CH_2CHCH\mathit{C}H_2CH_2CH_2O$), 20.64 ($CH_3\mathit{C}H_2CHCHCH_2CH_2CH_2O$), 14.43 ($\mathit{C}H_3CH_2CHCHCH_2CH_2CH_2O$).

^{31}P{^{1}H} NMR (162 MHz, $CDCl_3$): 20.11.

IR ν_{max} (KBr) 3090 (w), 2965 (s), 2936 (s), 2874 (m), 2860 (sh), 1655 (vw), 1614 (w), 1464 (m), 1456 (m), 1405 (m, sh), 1279 (m), 1240 (s), 1069–1002 (s), 990 (vs, sh).

HR-MS(ESI^{+}): For $C_{24}H_{45}O_6P_2$ $(M+H)^+$ *m/z* calculated 491.26859, found 491.26825.

^{1}H NMR spectrum of compound **S56**

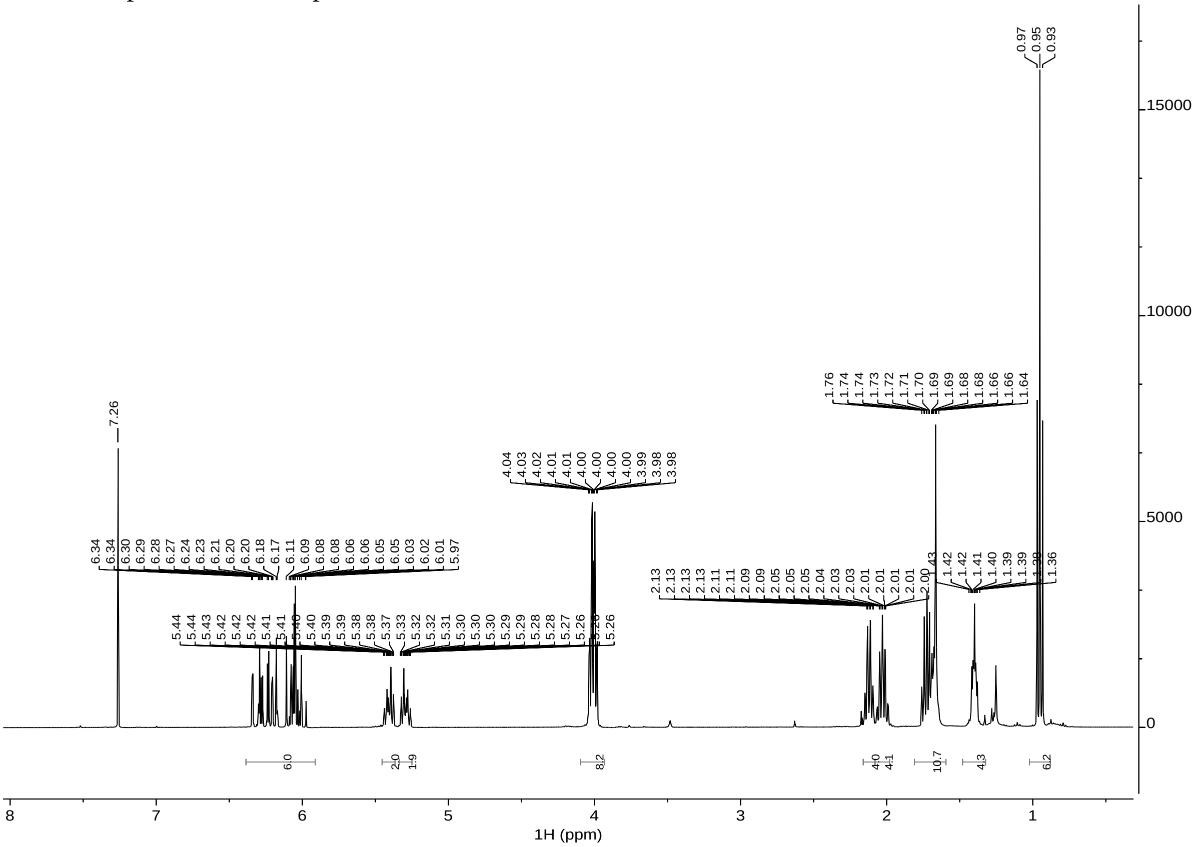

^{13}C NMR spectrum of compound **S56**

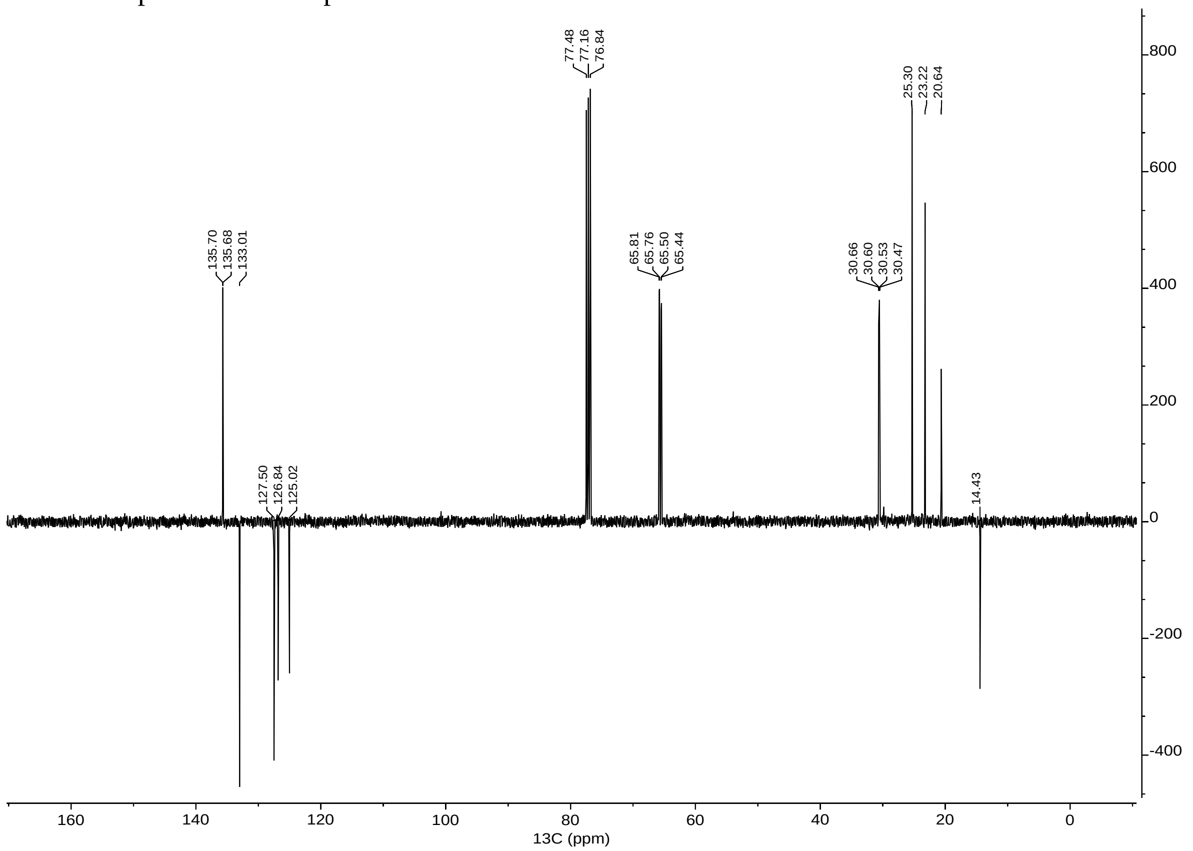

$^{31}P\{^1H\}$ NMR spectrum of compound **S56**

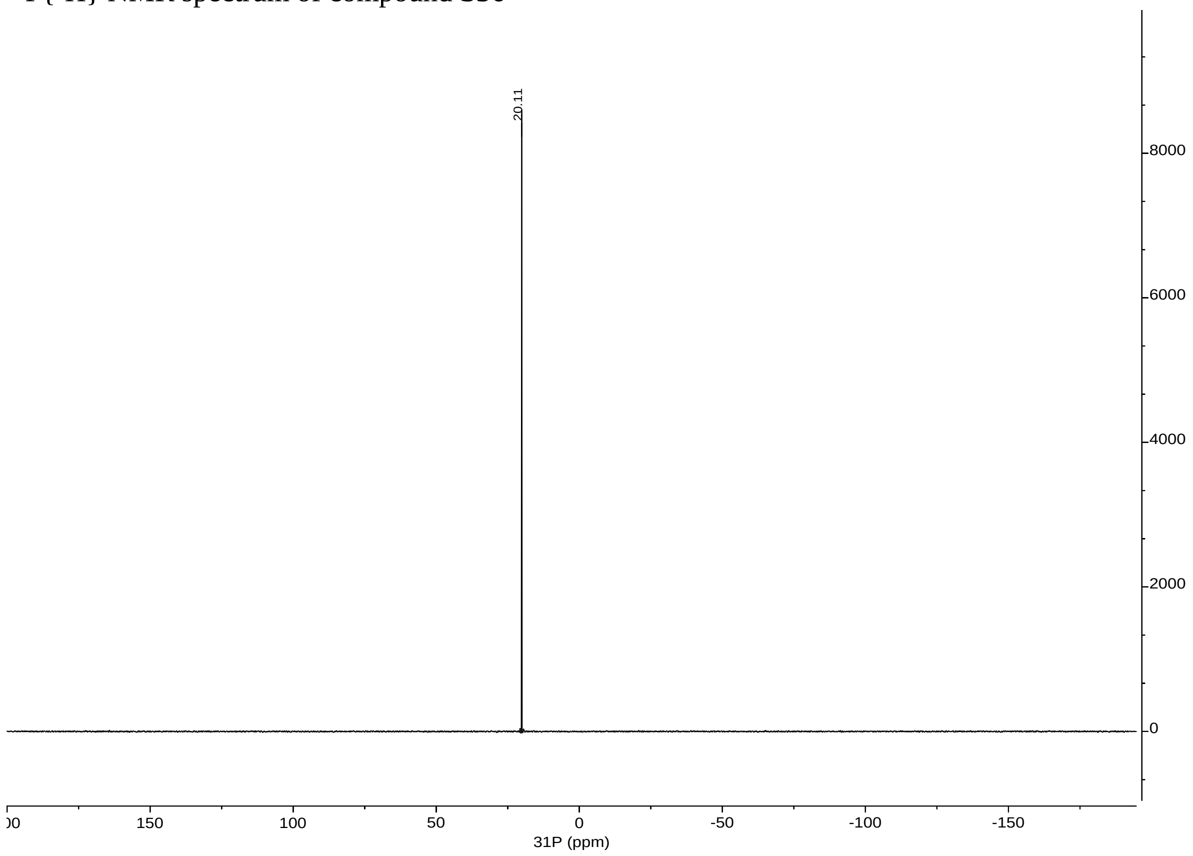

Hexane-1,6-diyl dioctyl bis(vinylphosphonate) (S57)

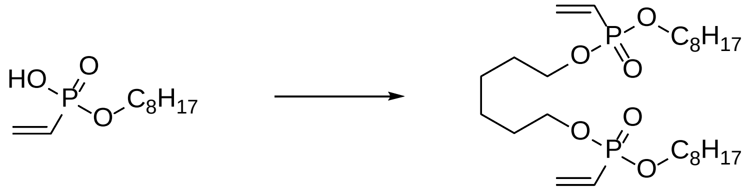

The title compound was prepared according to general method **C** from mono alkyl vinylphosphonate **S28** (1.92 g, 8.72 mmol) and 1,6-dibromohexane (96%, 466 µL, 2.91 mmol) in 82% yield (1.25 g, 2.39 mmol) as a colourless oil.

^{1}H NMR (401 MHz, $CDCl_3$) δ 6.45–5.85 (m, 6H, C*H*=C*H*$_2$), 4.09–3.93 (m, 8H, OC*H*$_2$), 1.86–1.61 (m, 8H, OCH_2C*H*$_2$), 1.49–1.19 (m, 24H, $O(CH_2)_2C$*H*$_2$, (C*H*$_2$)$_4CH_3$), 0.97–0.79 (m, 6H, C*H*$_3$).

^{13}C NMR (101 MHz, $CDCl_3$) δ 135.68 (=*C*H$_2$), 125.96 (d, *J* = 184.2 Hz, P*C*H), 66.14 (d, *J* = 5.9 Hz), 65.78 (d, *J* = 5.7 Hz, O*C*H$_2$), 31.91 (*C*$H_2CH_2CH_3$), 30.61 (d, *J* = 6.4 Hz), 30.50 (d, *J* = 6.6 Hz, OCH_2*C*H$_2$), 29.32, 29.26, 25.65, 25.30 ($O(CH_2)_2$*C*H$_2$, (*C*H$_2$)$_2(CH_2)_2CH_3$), 22.78 (*C*H_2CH_3), 14.22 (*C*H$_3$).

^{31}P{^{1}H} NMR (162 MHz, $CDCl_3$) δ 20.09.

IR ν_{max}($CHCl_3$) 3090 (vw), 2958 (s), 2930 (vs), 2871 (m), 2858 (m), 1614 (w), 1468 (w), 1458 (w, sh), 1434 (vw), 1400 (w), 1379 (w), 1279 (w), 1239 (s), ~1071 (m, sh), ~1050 (s, sh), ~1020 (s, sh), 1001 (vs), 986 (vs, sh).

HR-MS(ESI$^+$): For $C_{26}H_{53}O_6P_2$ $(M+H)^+$ *m*/*z* calculated 523.33119, found 523.33047. For $C_{26}H_{52}O_6NaP_2$ $(M+Na)^+$ *m*/*z* calculated 545.31313, found 545.31250.

^{1}H NMR spectrum of compound **S57**

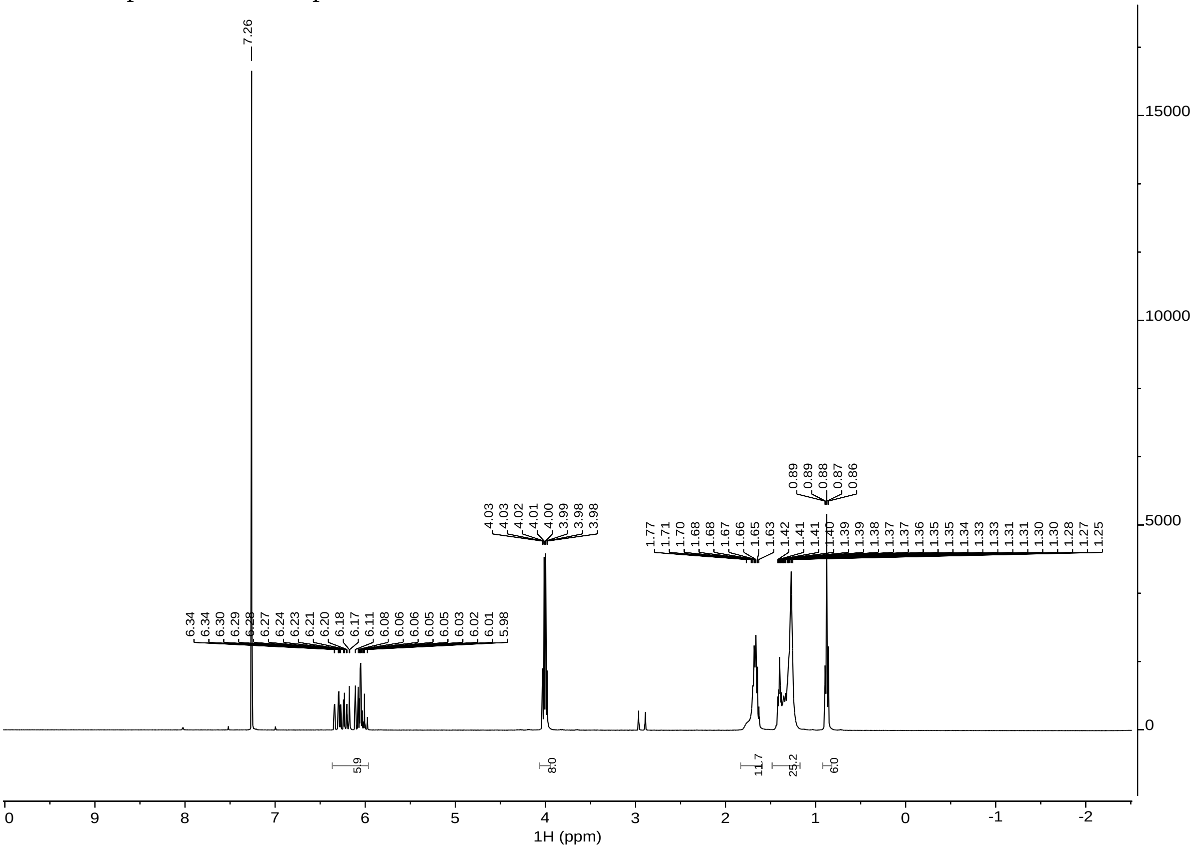

^{13}C NMR spectrum of compound **S57**

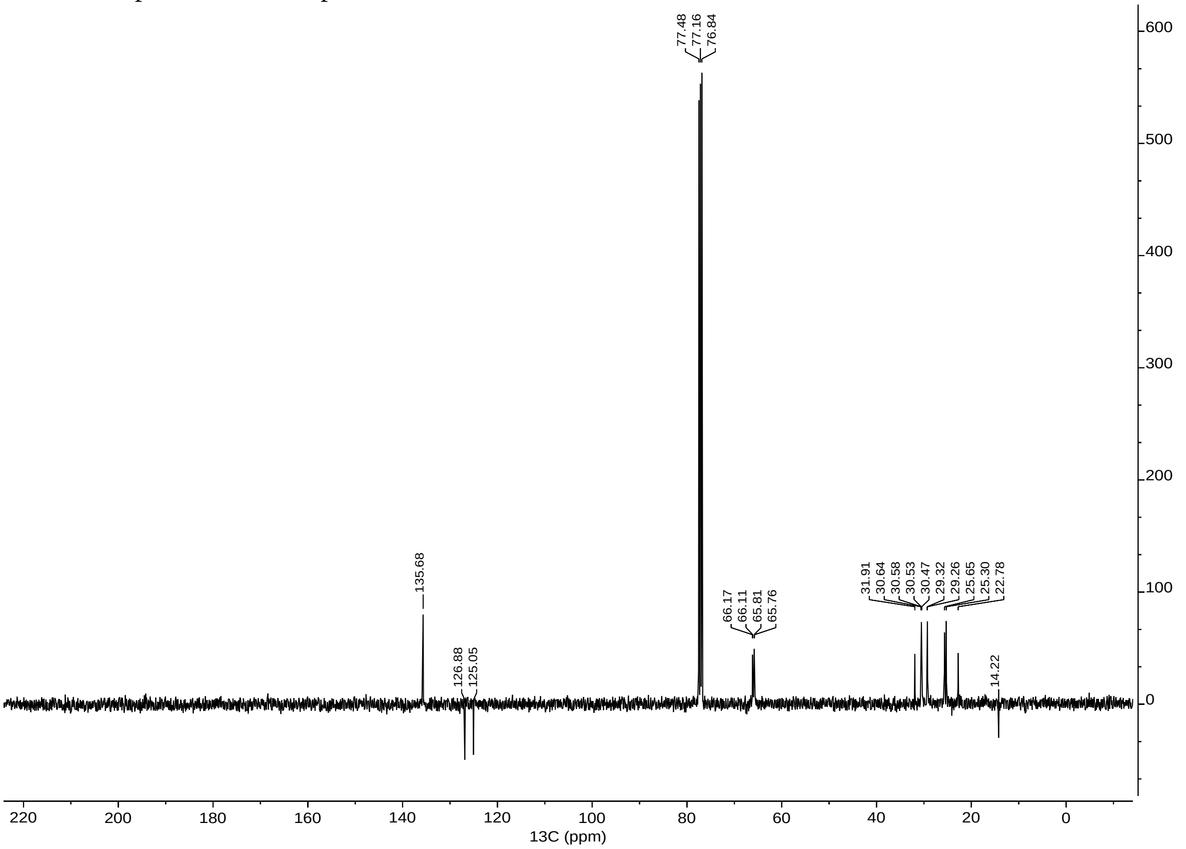

$^{31}P\{^1H\}$ NMR spectrum of compound **S57**

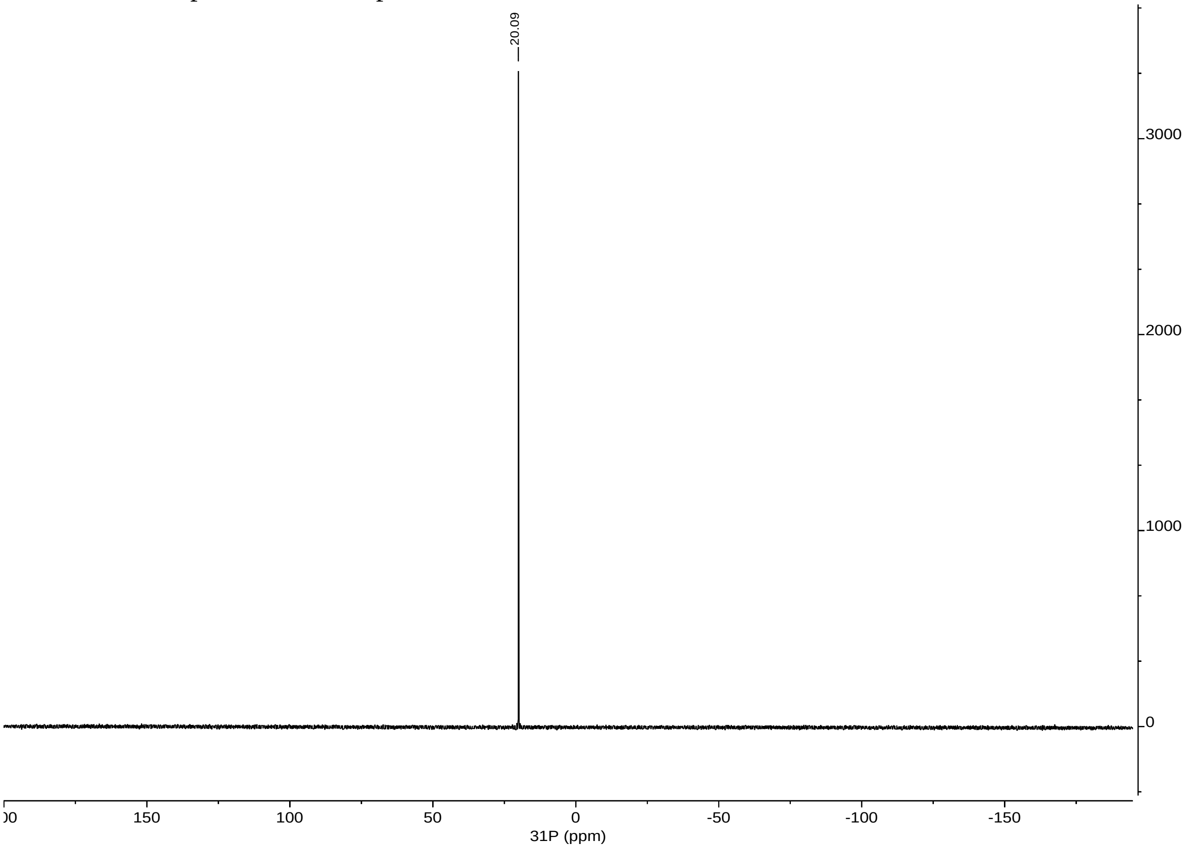

Bis(3-cyclohexylpropyl) hexane-1,6-diyl bis(vinylphosphonate) (S58)

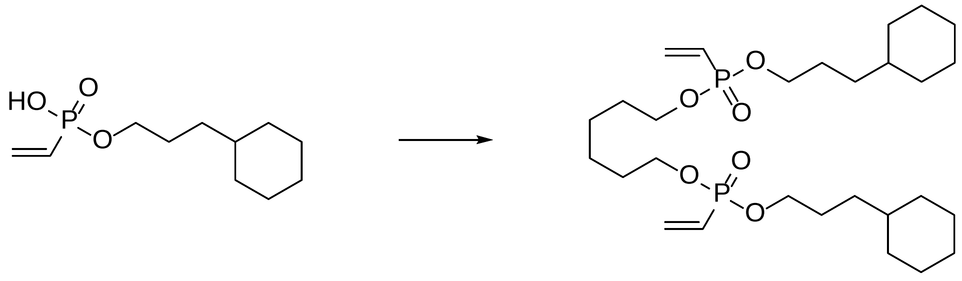

The title compound was prepared according to general method **C** from mono alkyl vinylphosphonate **S29** (1.50 g, 6.46 mmol) and 1,6-dibromohexane (0.35 mL, 2.26 mmol) in 84% yield (1.04 g, 1.90 mmol) as a colourless oil.

^{1}H NMR (401 MHz, Chloroform-*d*) δ 6.34–5.95 (m, 6H, C*H*=C*H*$_2$), 3.99 (p, *J* = 6.9 Hz, 8H, OC*H*$_2$), 1.75–1.58 (m, 18H, 2a,3a,4a,5a,6a-Cy*H*, OCH$_2$C*H*$_2$), 1.45–1.34 (m, 4H, O(CH$_2$)$_2$(C*H*$_2$)$_2$(CH$_2$)$_2$O), 1.27–1.05 (m, 12H, 1,3b,4b,5b-Cy*H*, O(CH$_2$)C*H*$_2$Cy), 0.93–0.78 (m, 4H, 2b,6b-Cy*H*).

^{13}C NMR (101 MHz, Chloroform-*d*) δ 135.66 (d, *J* = 2.1 Hz, =*C*H$_2$), 125.92 (d, *J* = 184.0 Hz, P*C*H), 66.47 (d, *J* = 5.8 Hz, O*C*H$_2$(CH$_2$)$_2$Cy), 65.77 (d, *J* = 5.8 Hz, O*C*H$_2$(CH$_2$)$_4$*C*H$_2$O), 37.38 (1-*C*H$_{Cy}$), 33.38 (2,6-(*C*H$_2$)$_{Cy}$), 33.23 (*C*H$_2$Cy), 30.47 (d, *J* = 6.5 Hz, OCH$_2$*C*H$_2$(CH$_2$)$_2$*C*H$_2$CH$_2$O), 28.00 (d, *J* = 6.4 Hz, *C*H$_2$CH$_2$Cy), 26.72 (4-(*C*H$_2$)$_{Cy}$), 26.43 (3,5-(*C*H$_2$)$_{Cy}$), 25.27 (O(CH$_2$)$_2$(*C*H$_2$)$_2$(CH$_2$)$_2$O).

^{31}P{^{1}H} NMR (162 MHz, Chloroform-*d*) δ 20.08.

IR ν_{max} (CHCl$_3$) 3090 (w), 2926 (vs), 2853 (s), 1614 (w), 1473 (w), 1463 (w), 1449 (m), 1400 (m), 1278 (m), 1240 (s), 1069 (s), 1052 (s), 1041 (s), 1004 (vs), ~987 (s, sh), ~965 (m, sh).

HR-MS(ESI$^+$): For $C_{28}H_{53}O_6P_2$ (M+H)$^+$ *m/z* calculated 547.33119, found 547.33026. For $C_{28}H_{52}O_6NaP_2$ (M+Na)$^+$ *m/z* calculated 569.31313, found 569.31219.

^{1}H NMR spectrum of compound **S58**

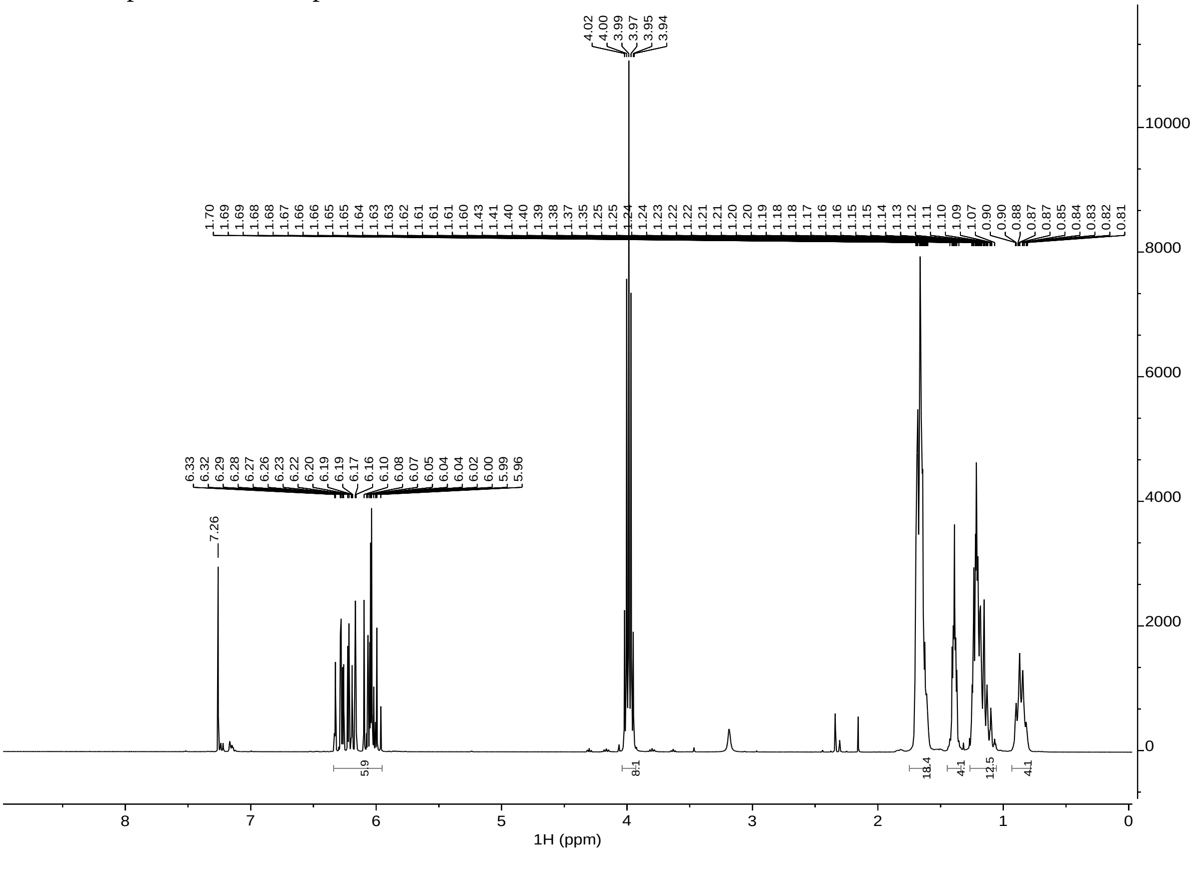

^{13}C NMR spectrum of compound **S58**

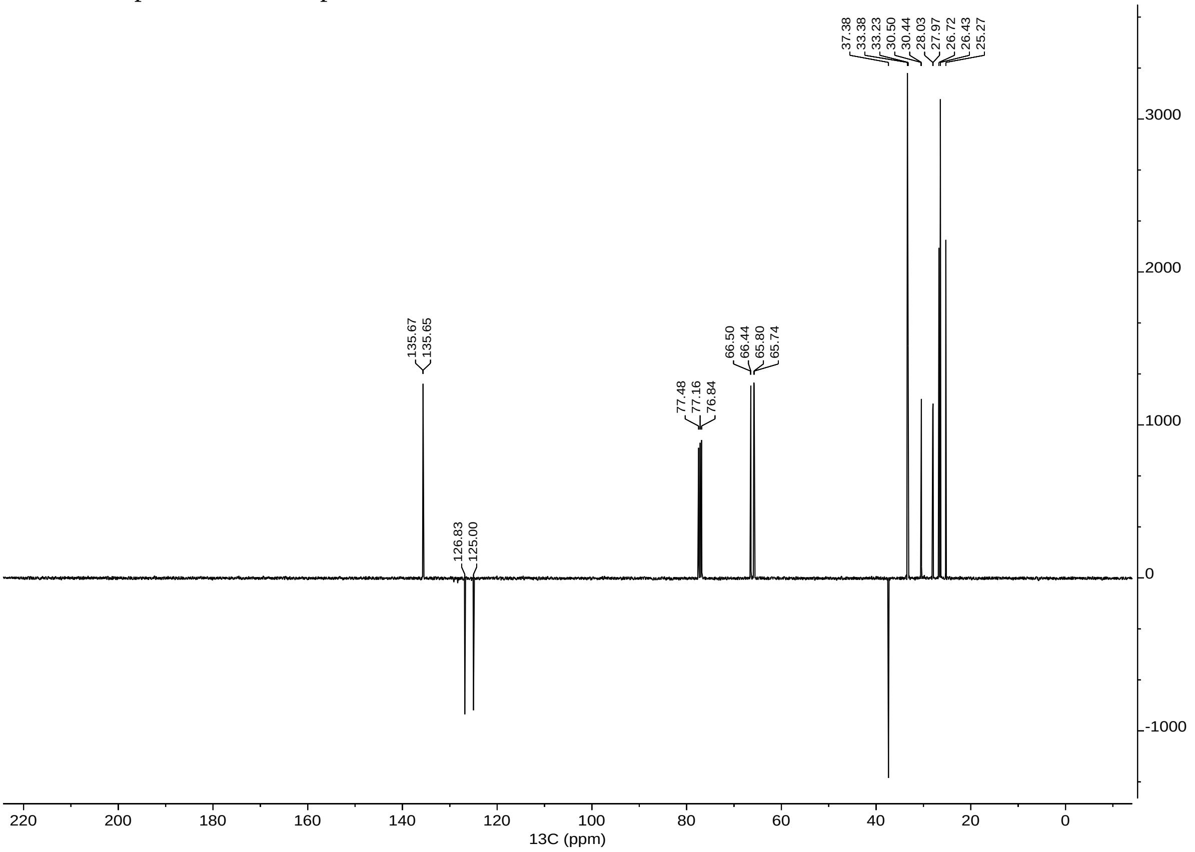

^{31}P{^{1}H} NMR spectrum of compound **S58**

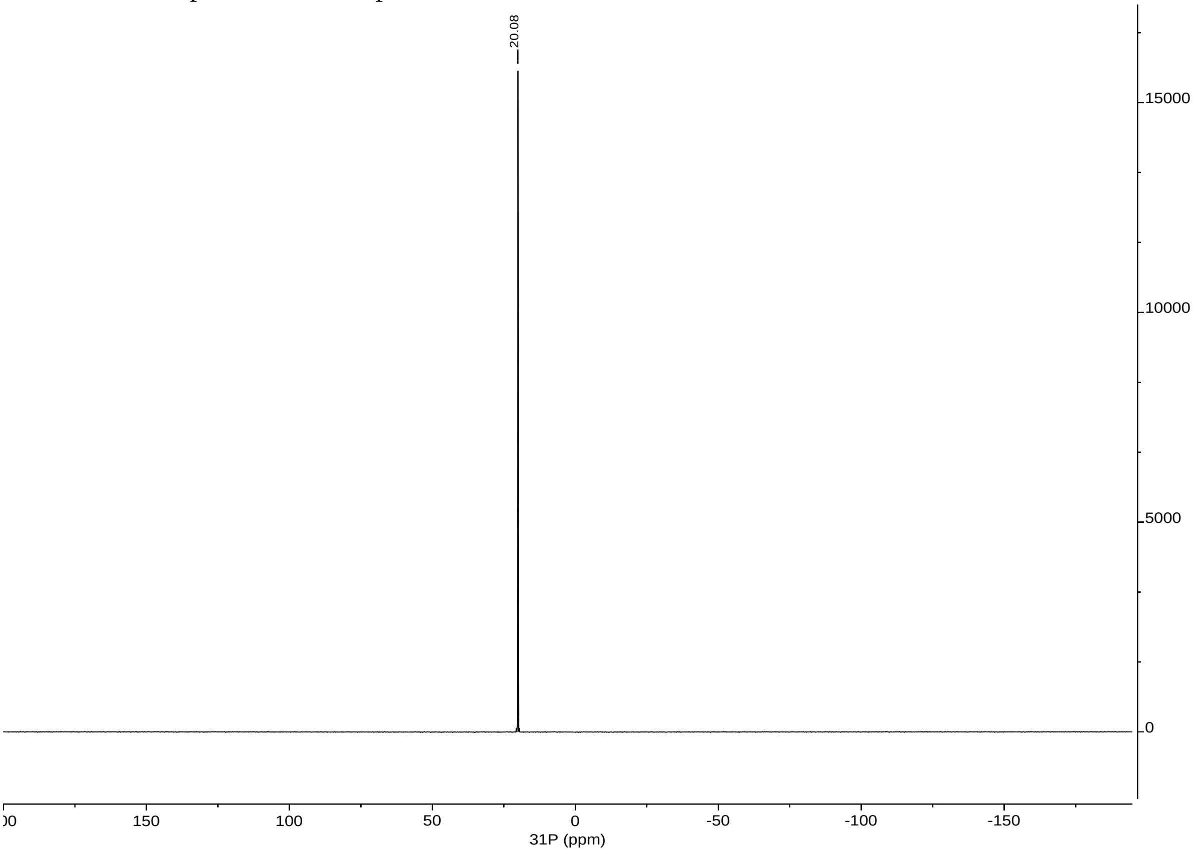

Hexane-1,6-diyl bis((*Z*)-oct-3-en-1-yl) bis(vinylphosphonate) (S59)

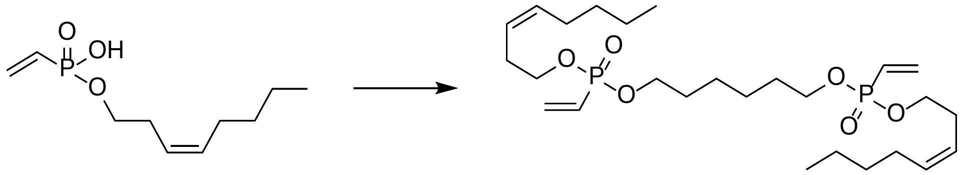

The title compound was prepared according to general method **C** from mono alkyl vinylphosphonate **S30** (0.9 g, 4.12 mmol) and 1,6-dibromohexane (0.34 g, 1.37 mmol) in 83% yield (0.59 g, 1.14 mmol) as a colourless oil.

^{1}H NMR (400 MHz, $CDCl_3$): 6.34–5.97 (m, 6H, C*H*=C*H*$_2$), 5.54–5.47 (m, 2H, $CH_3(CH_2)_3C\textit{H}CHCH_2CH_2O$), 5.37–5.30 (m, 2H, $CH_3(CH_2)_3CHC\textit{H}CH_2CH_2O$), 4.03–3.97 (m, 8H, $OC\textit{H}_2CH_2(CH_2)_2CH_2C\textit{H}_2O$, $CH_3(CH_2)_2CH_2CHCHCH_2C\textit{H}_2O$), 2.45–2.39 (m, 4H, $CH_3(CH_2)_2CH_2CHCHC\textit{H}_2CH_2O$), 2.06–2.00 (m, 4H, $CH_3(CH_2)_2C\textit{H}_2CHCHCH_2CH_2O$), 1.70–1.62 (m, 4H, $OCH_2C\textit{H}_2(CH_2)_2C\textit{H}_2CH_2O$), 1.41–1.36 (m, 4H, $OCH_2CH_2(C\textit{H}_2)_2CH_2CH_2O$), 1.35–1.25 (m, 8H, $CH_3(C\textit{H}_2)_2CH_2CHCHCH_2CH_2O$), 0.92–0.86 (m, 6H, $CH_3(CH_2)_3CHCHCH_2CH_2O$).

^{13}C NMR (101 MHz, $CDCl_3$): 135.71 (d, *J* = 1.8 Hz, CH=*C*H$_2$), 133.40 ($CH_3(CH_2)_3\textit{C}HCHCH_2CH_2O$), 125.92 (d, *J* = 184.1 Hz, *C*H=CH$_2$), 123.83 ($CH_3(CH_2)_3CH\textit{C}HCH_2CH_2O$), 65.81 (d, *J* = 6.0 Hz, $CH_3(CH_2)_2CH_2CHCHCH_2\textit{C}H_2O$), 65.40 (d, *J* = 6.1 Hz, $O\textit{C}H_2CH_2(CH_2)_2CH_2\textit{C}H_2O$), 31.85 ($CH_3CH_2\textit{C}H_2CH_2CHCHCH_2CH_2O$), 30.49 (d, *J* = 6.6 Hz, $OCH_2\textit{C}H_2(CH_2)_2\textit{C}H_2CH_2O$), 28.81 (d, *J* = 6.4 Hz, $CH_3(CH_2)_2CH_2CHCH\textit{C}H_2CH_2O$), 27.21 $CH_3(CH_2)_2\textit{C}H_2CHCHCH_2CH_2O$), 25.29 ($OCH_2CH_2(\textit{C}H_2)_2CH_2CH_2O$), 22.45 ($CH_3\textit{C}H_2(CH_2)_2CHCHCH_2CH_2O$), 14.10 ($\textit{C}H_3(CH_2)_3CHCHCH_2CH_2O$).

^{31}P{^{1}H} NMR (162 MHz, $CDCl_3$): 19.83.

IR ν_{max} (KBr) 3085 (w), 3011 (m), 2956 (s), 2929 (s), 2871 (m), 2859 (m), 1654 (w), 1613 (w), 1467 (m), 1398 (m), 1278 (m, sh), 1251 (s), 1053 (s), 1003 (s), 970 (s, sh), 854 (m), 729 (m), 698 (m).

HR-MS(APCI$^+$): For $C_{26}H_{49}O_6P_2$ $(M+H)^+$ *m/z* calculated 519.29989, found 519.29947.

^{1}H NMR spectrum of compound **S59**

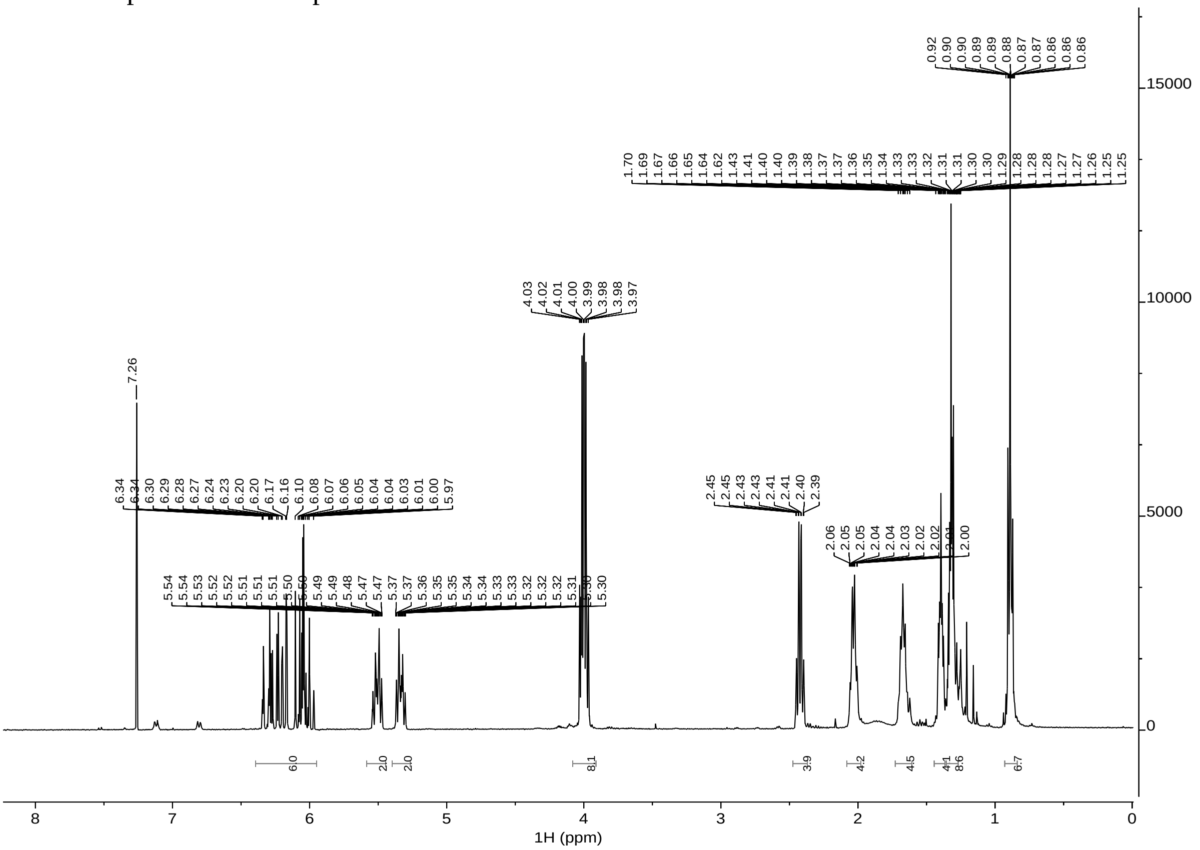

^{13}C NMR spectrum of compound **S59**

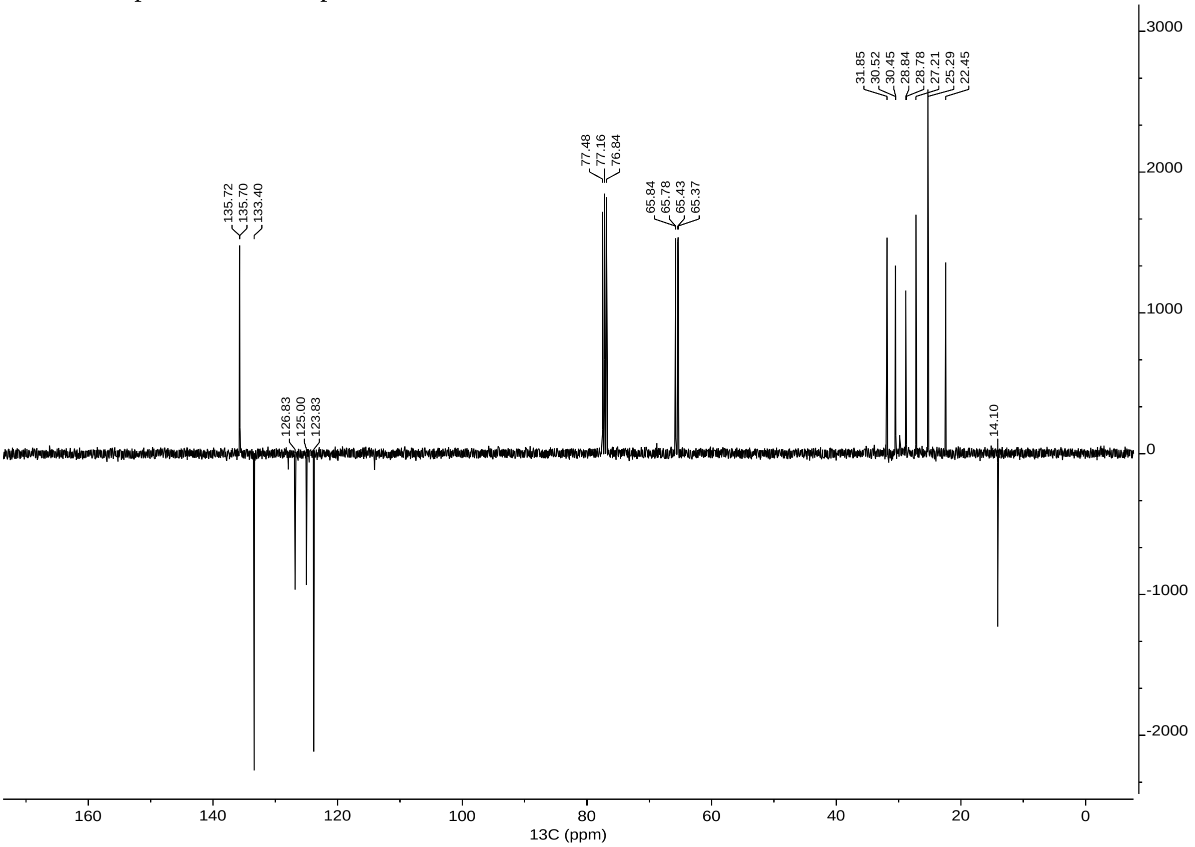

^{31}P{^{1}H} NMR spectrum of compound **S59**

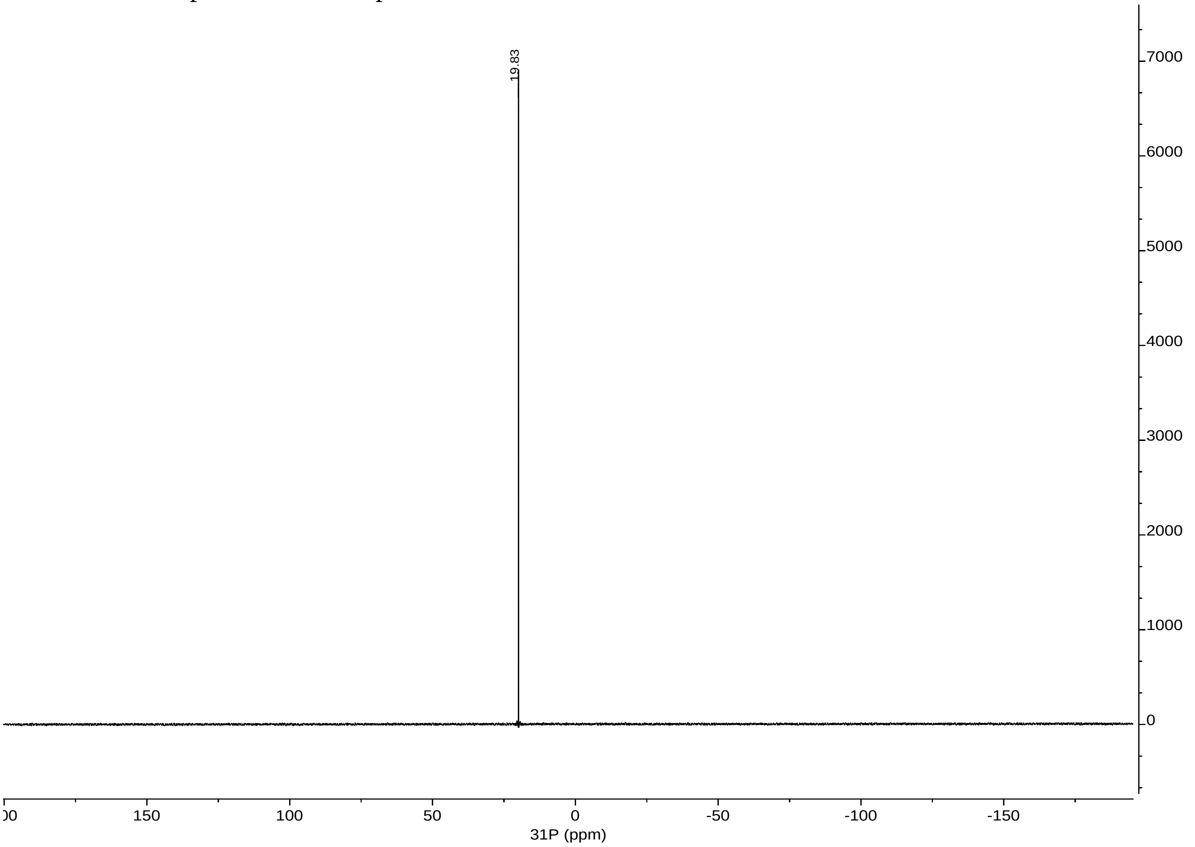

Hexane-1,6-diyl diphenethyl bis(vinylphosphonate) (S60)

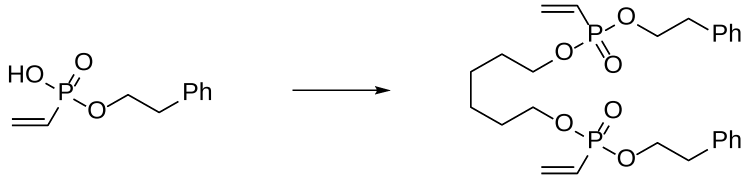

The title compound was prepared according to general method **B1** from mono alkyl vinylphosphonate **S31** (4.40 g, 20.7 mmol) and hexane-1,6-diol (817 mg, 6.91 mmol) in 64% yield (2.23 g, 4.40 mmol) as a colourless oil.

^{1}H NMR (401 MHz, $CDCl_3$) δ 7.35–7.15 (m, 10H, Ph), 6.53–5.78 (m, 6H, C*H*=C*H*$_2$), 4.38–4.10 (m, 4H, OC*H*$_2$(CH_2)$_2$), 4.02–3.83 (m, 4H, OC*H*$_2$$CH_2$Ph), 2.98 (t, *J* = 7.0 Hz, 4H, C*H*$_2$Ph), 1.67–1.55 (m, 4H, OCH_2C*H*$_2$$CH_2$), 1.40–1.28 (m, 4H, OCH_2CH_2C*H*$_2$).

^{13}C NMR (101 MHz, $CDCl_3$) δ 137.42 (*C*$_{quat}$), 135.77 (d, *J* = 2.1 Hz, =*C*H_2), 129.17, 128.63, 126.81 (*C*$_{Ph}$), 125.69 (d, *J* = 184.1 Hz, P*C*H), 66.34 (d, *J* = 5.8 Hz), 65.77 (d, *J* = 5.9 Hz, O*C*H_2), 37.11 (d, *J* = 6.5 Hz), 30.43 (d, *J* = 6.4 Hz, OCH_2*C*H_2), 25.23 (O(CH_2)$_2$*C*H_2).

^{31}P{^{1}H} NMR (162 MHz, $CDCl_3$) δ 20.10.

IR ν_{max}(film) 3086 (w), 3062 (w), 3029 (w), 2934 (m), 2863 (w), 1610 (w), 1605 (w), 1584 (vw), 1497 (vw), 1472 (w), 1454 (m), 1398 (m), 1281 (m, sh), 1248 (s), 1180 (w), 1153 (w), 1087 (m), 1059 (s), 1008 (vs), 1000 (vs), 900 (w), 700 (m), 551 (m), 493 (m).

HR-MS(APCI^{+}): For $C_{26}H_{37}O_6P_2$ $(M+H)^{+}$ *m*/*z* calculated 507.20599, found 507.20615.

^{1}H NMR spectrum of compound **S60**

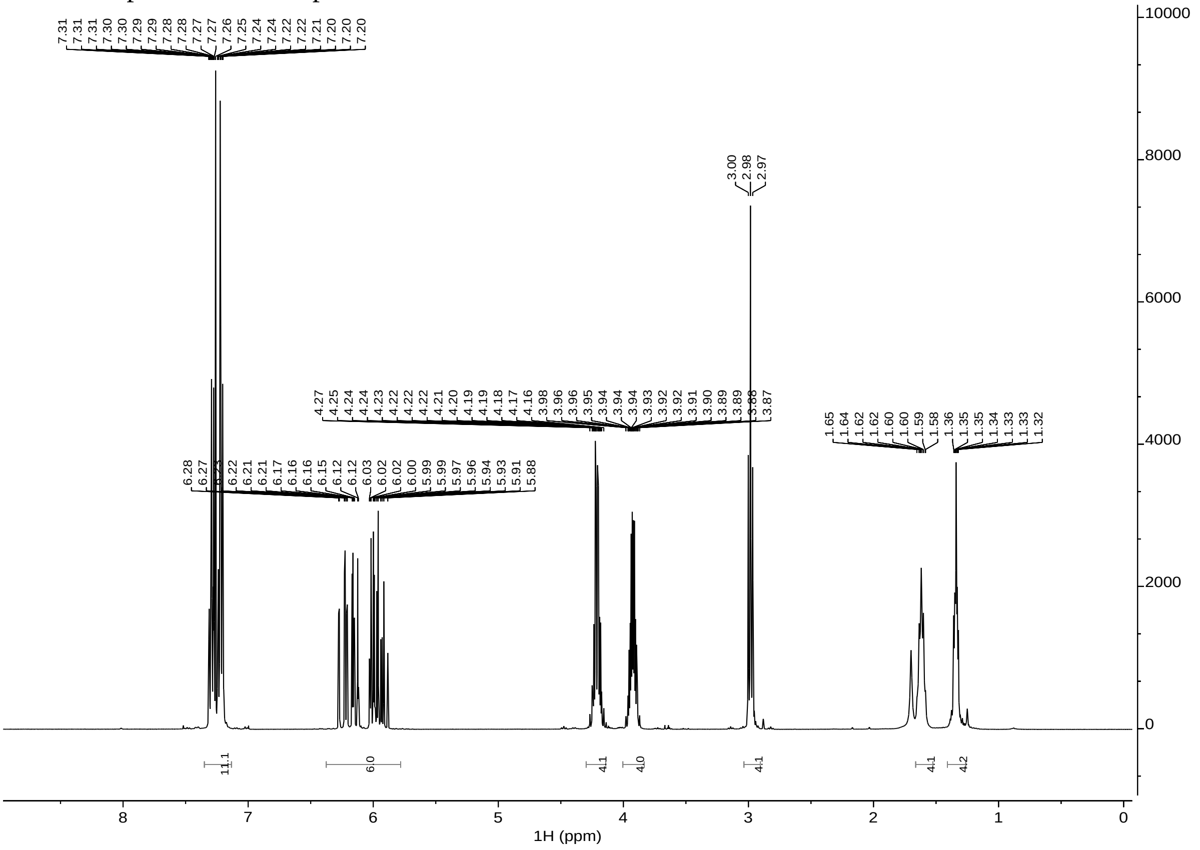

^{13}C NMR spectrum of compound **S60**

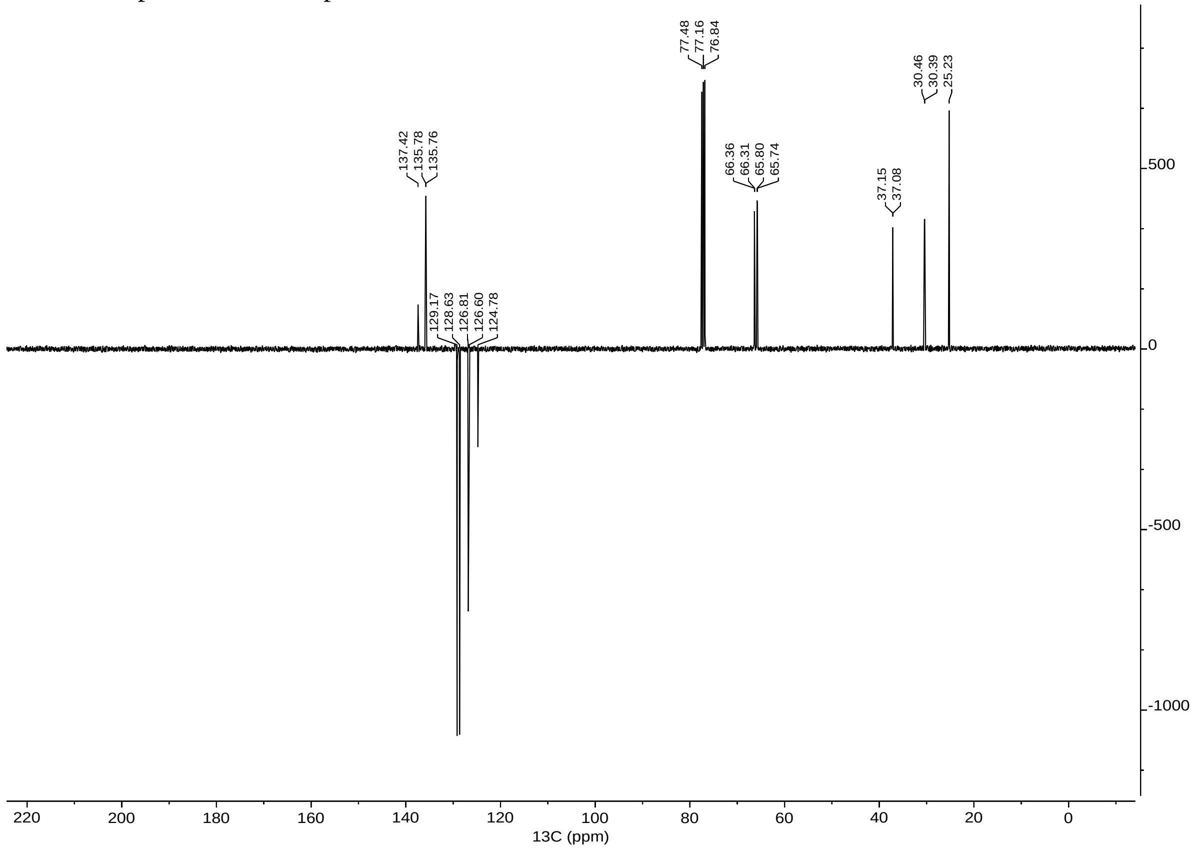

$^{31}P\{^1H\}$ NMR spectrum of compound **S60**

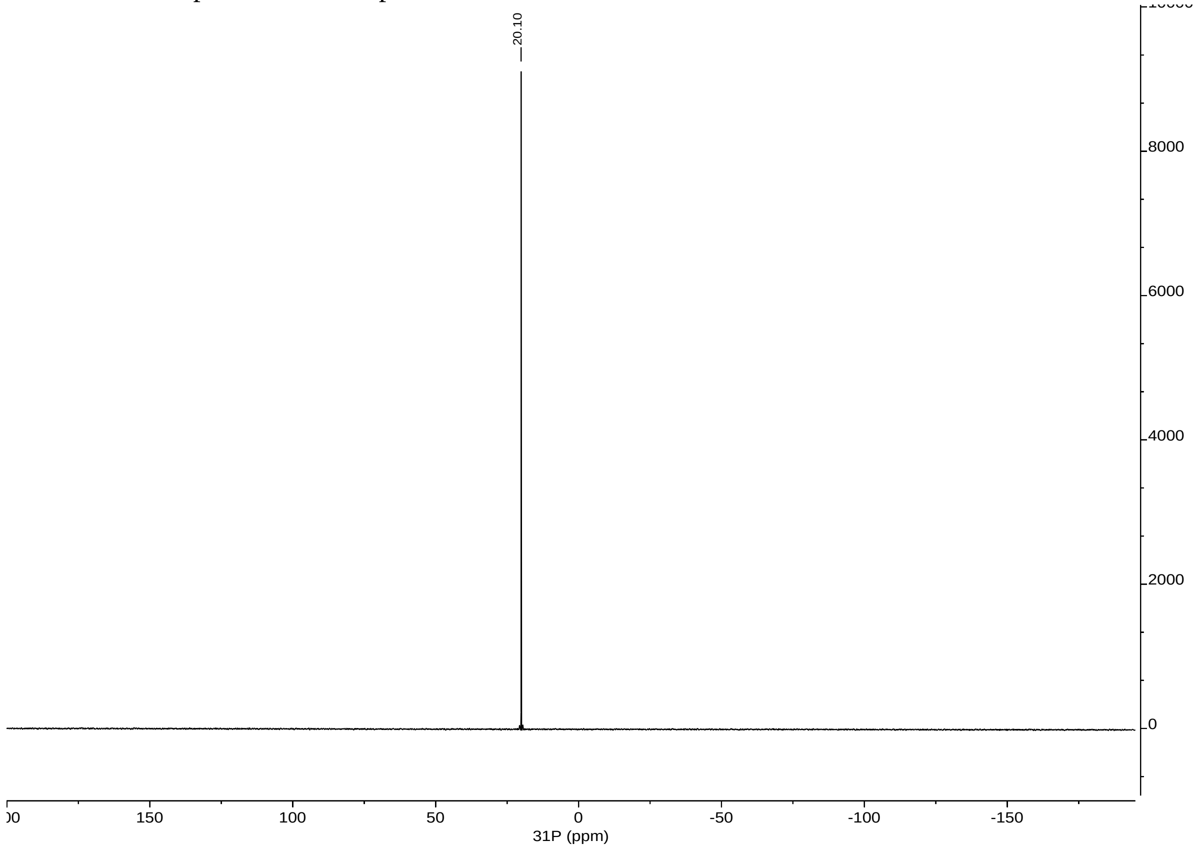

Hexane-1,6-diyl bis((*Z*)-non-3-en-1-yl) bis(vinylphosphonate) (S61)

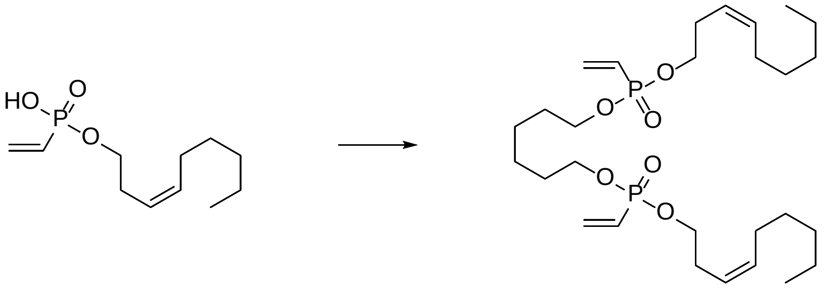

The title compound was prepared according to general method **C** from mono alkyl vinylphosphonate **S32** (2.00 g, 8.61 mmol) and 1,6-dibromohexane (464 µL, 3.02 mmol) in 66% yield (1.09 mg, 1.99 mmol) as a colourless oil.

^{1}H NMR (401 MHz, $CDCl_3$) δ 6.36–5.96 (m, 6H, C*H*=C*H*$_2$), 5.56–5.46 (m, 2H, C*H*$(CH_2)_4CH_3$), 5.38–5.29 (m, 2H, $O(CH_2)_2$C*H*), 4.05–3.96 (m, 8H, OC*H*$_2$), 2.42 (qd, *J* = 7.1, 1.5 Hz, 4H, OCH_2C*H*$_2$CH), 2.02 (qd, *J* = 7.2, 1.6 Hz, 4H, C*H*$_2(CH_2)_3CH_3$), 1.73–1.62 (m, 4H, OCH_2C*H*$_2CH_2$), 1.45–1.21 (m, 16H, $O(CH_2)_2$C*H*$_2$, (C*H*$_2)_3CH_3$), 0.93–0.83 (m, 6H, C*H*$_3$).

^{13}C NMR (101 MHz, $CDCl_3$) δ 135.73 (d, *J* = 2.1 Hz, =*C*H$_2$), 133.47 (*C*H$(CH_2)_4CH_3$), 125.93 (d, *J* = 184.1 Hz, P*C*H), 123.83 ($O(CH_2)_2$*C*H), 65.81 (d, *J* = 5.8 Hz, O*C*H$_2(CH_2)_2$), 65.39 (d, *J* = 5.8 Hz, O*C*H$_2CH_2CH$), 31.62 (*C*H$_2CH_2CH_3$), 30.50 (d, *J* = 6.4 Hz, OCH_2*C*H$_2CH_2$), 29.36 (*C*H$_2(CH_2)_2CH_3$), 28.82 (d, *J* = 6.3 Hz, OCH_2*C*H$_2$CH), 27.48 (*C*H$_2(CH_2)_3CH_3$), 25.30 ($O(CH_2)_2$*C*H$_2$), 22.70 (*C*H$_2CH_3$), 14.20 (*C*H$_3$).

^{31}P{^{1}H} NMR (162 MHz, $CDCl_3$) δ 20.09.

IR ν_{max} ($CHCl_3$) 3090 (vw), 2959 (s), 2931 (s), 2872 (m), 2859 (m), 1653 (vw), 1614 (w), 1468 (w), ~1455 (w, sh), 1434 (vw), 1400 (w), 1380 (w), 1279 (w), 1240 (s), ~1070 (m, sh), 1054 (s), 1004 (vs), ~986 (s, sh).

HR-MS(ESI^+) For $C_{28}H_{53}O_6P_2$ $(M+H)^+$ *m*/*z* calculated 547.33119, found 547.33133.

^{1}H NMR spectrum of compound **S61**

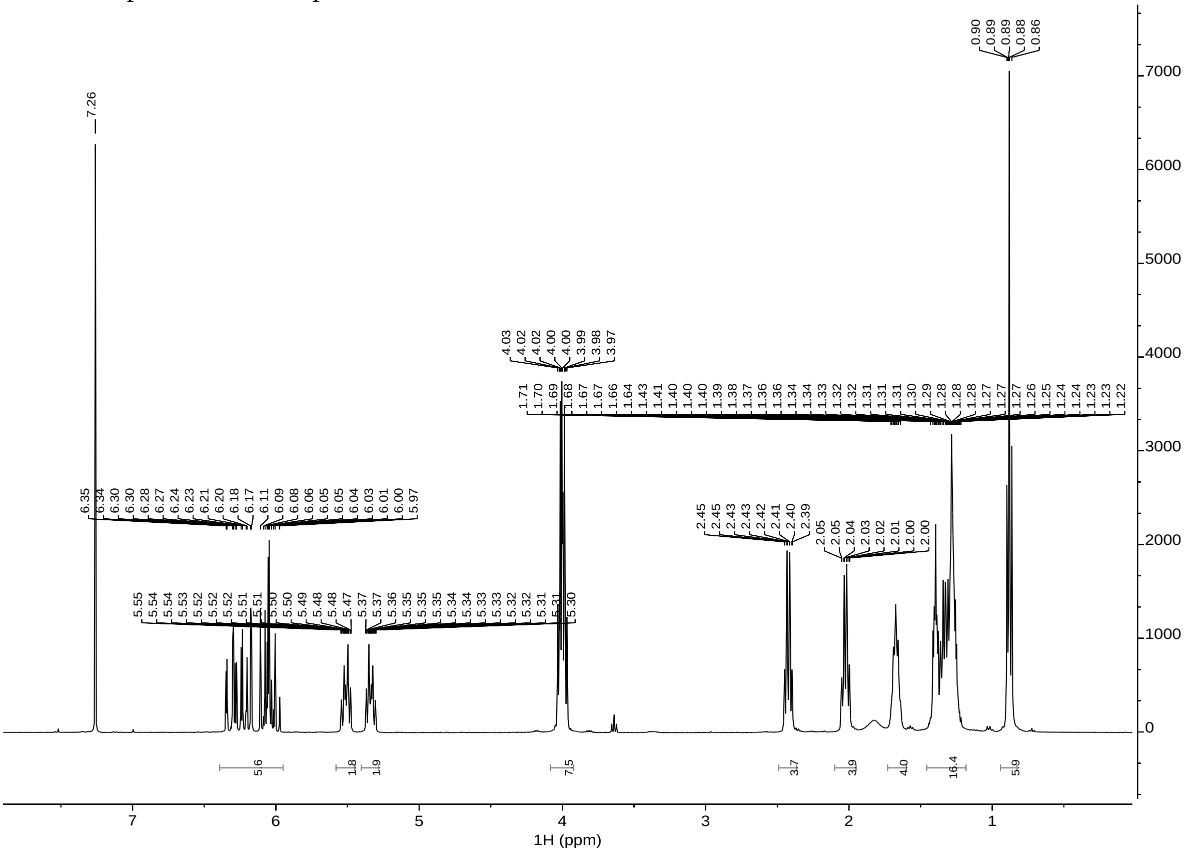

^{13}C NMR spectrum of compound **S61**

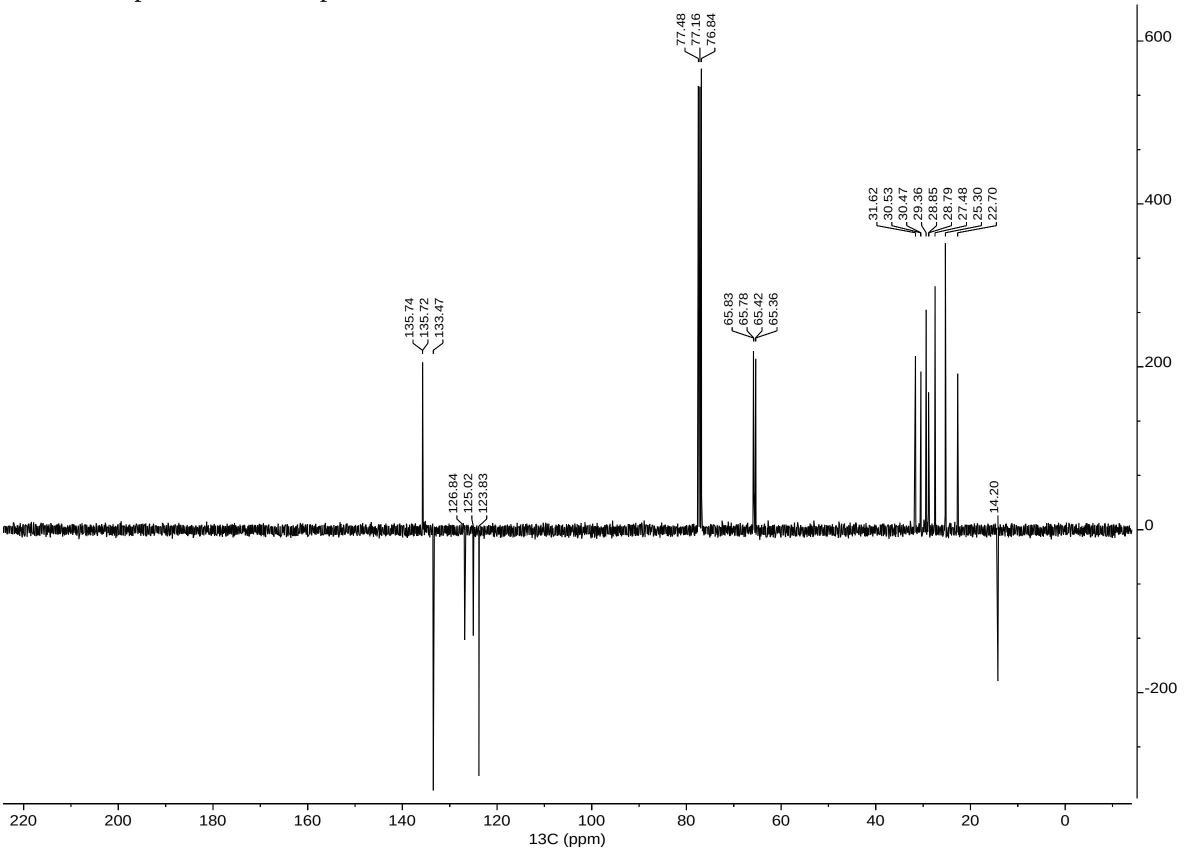

^{31}P{^{1}H} NMR spectrum of compound **S61**

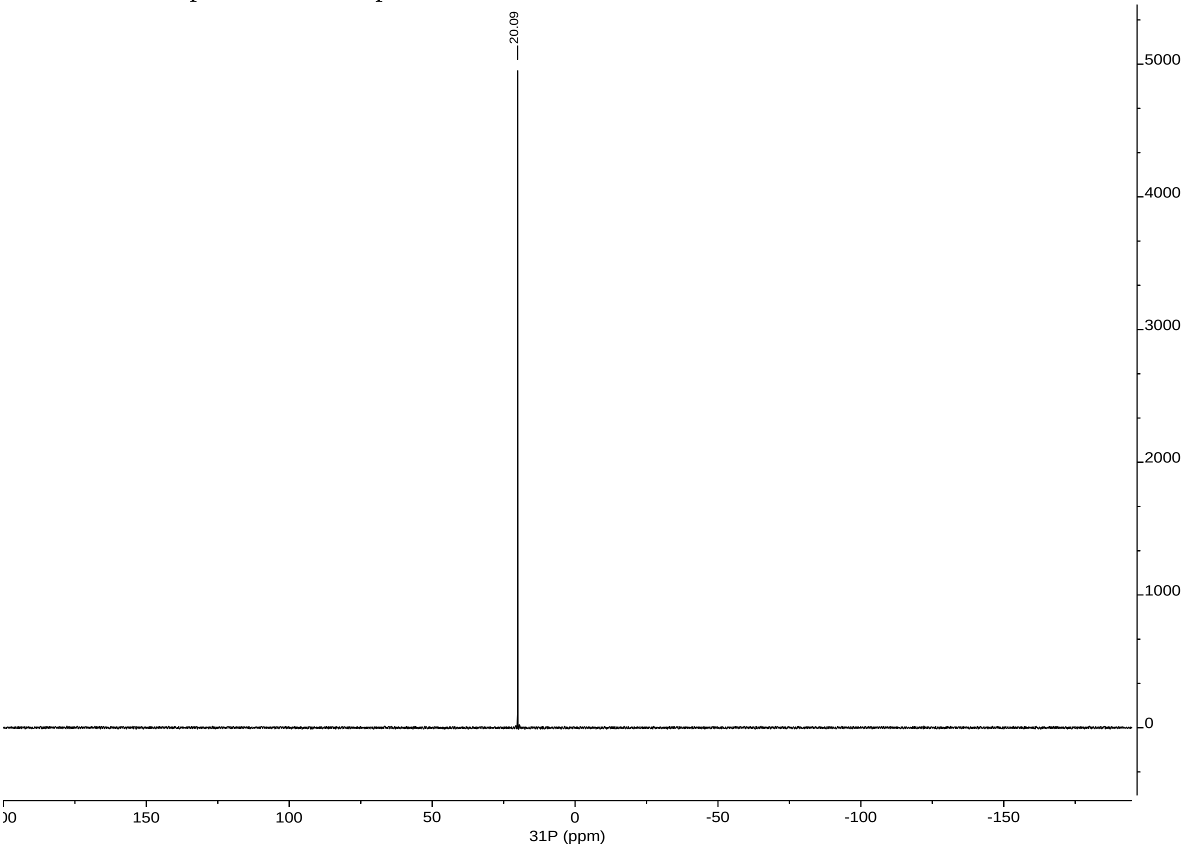

Didecyl hexane-1,6-diyl bis(vinylphosphonate) (S62)

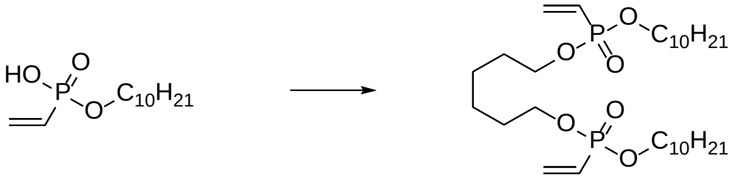

The title compound was prepared according to general method **C** from mono alkyl vinylphosphonate **S33** (4.20 g, 16.9 mmol) and 1,6-dibromohexane (96%, 903 µL, 5.64 mmol) in 80% yield (2.60 g, 4.49 mmol) as a colourless oil.

^{1}H NMR (401 MHz, $CDCl_3$) δ 6.36–5.94 (m, 6H, C*H*=C*H*$_2$), 4.07–3.91 (m, 8H, OC*H*$_2$), 1.75–1.59 (m, 8H, OCH_2C*H*$_2$), 1.46–1.15 (m, 32H, $O(CH_2)_2$C*H*$_2$, (C*H*$_2$)$_6$$CH_3$), 0.95–0.77 (m, 6H, C*H*$_3$).

^{13}C NMR (101 MHz, $CDCl_3$) δ 135.57 (=*C*H_2), 125.80 (d, J = 184.0 Hz, P*C*H), 66.02 (d, J = 5.9 Hz), 65.66 (d, J = 5.9 Hz, O*C*H_2), 31.89 (*C*$H_2CH_2CH_3$), 30.47 (d, J = 6.5 Hz), 30.36 (d, J = 6.2 Hz, OCH_2*C*H_2), 29.52, 29.30, 29.17, 25.52, 25.16, 22.68 (*C*H_2CH_3), 14.12 (*C*H_3).

^{31}P{^{1}H} NMR (162 MHz, $CDCl_3$) δ 20.08.

IR ν_{max} ($CHCl_3$) 3094 (w), 2957 (s), 2929 (vs), 2874 (sh), 2857 (s), 1614 (w), 1467 (m), 1458 (m), 1400 (m), 1379 (w), 1280 (m), 1240 (s), 1070–1000 (s), 998 (vs), 861 (m).

HR-MS(ESI^{+}): For $C_{30}H_{61}O_6P_2$ $(M+H)^+$ *m/z* calculated 579.39379, found 579.39373. For $C_{30}H_{60}O_6NaP_2$ $(M+Na)^+$ *m/z* calculated 601.37573, found 601.37569.

^{1}H NMR spectrum of compound S62

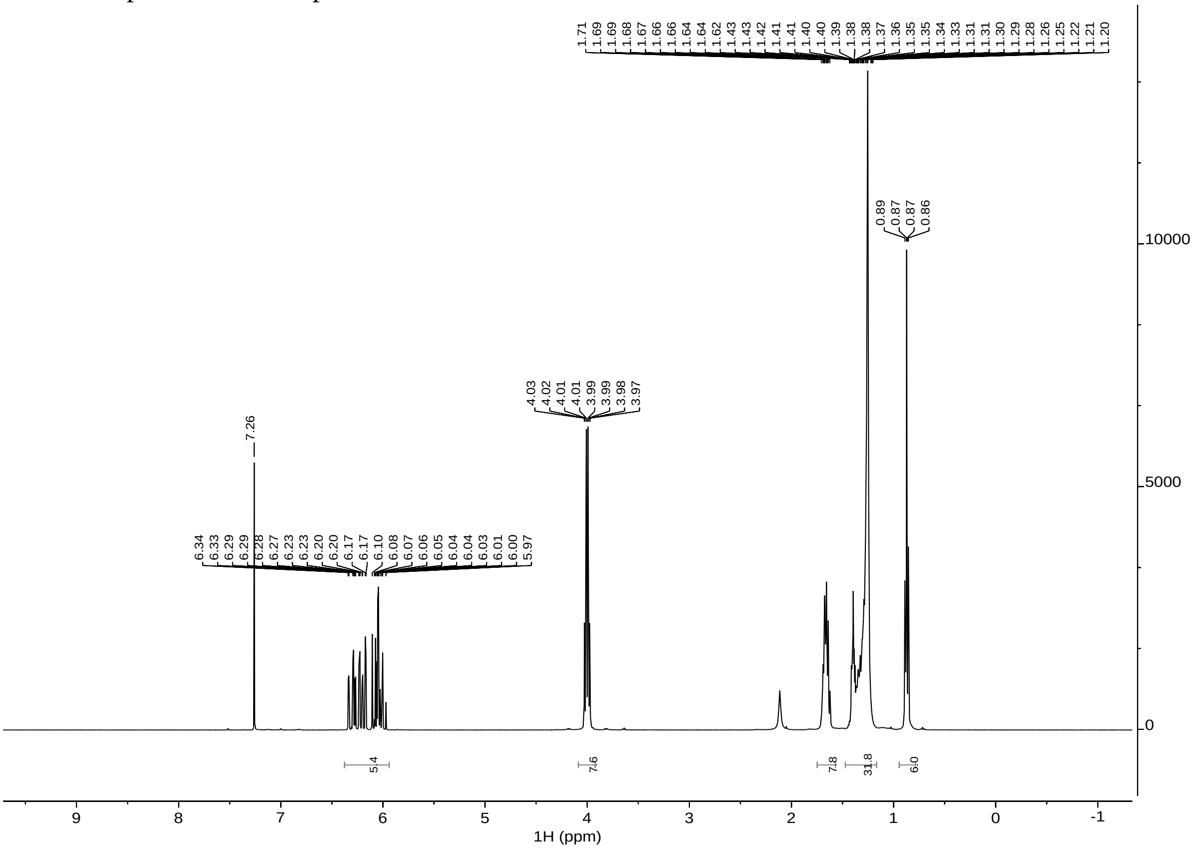

^{13}C NMR spectrum of compound S62

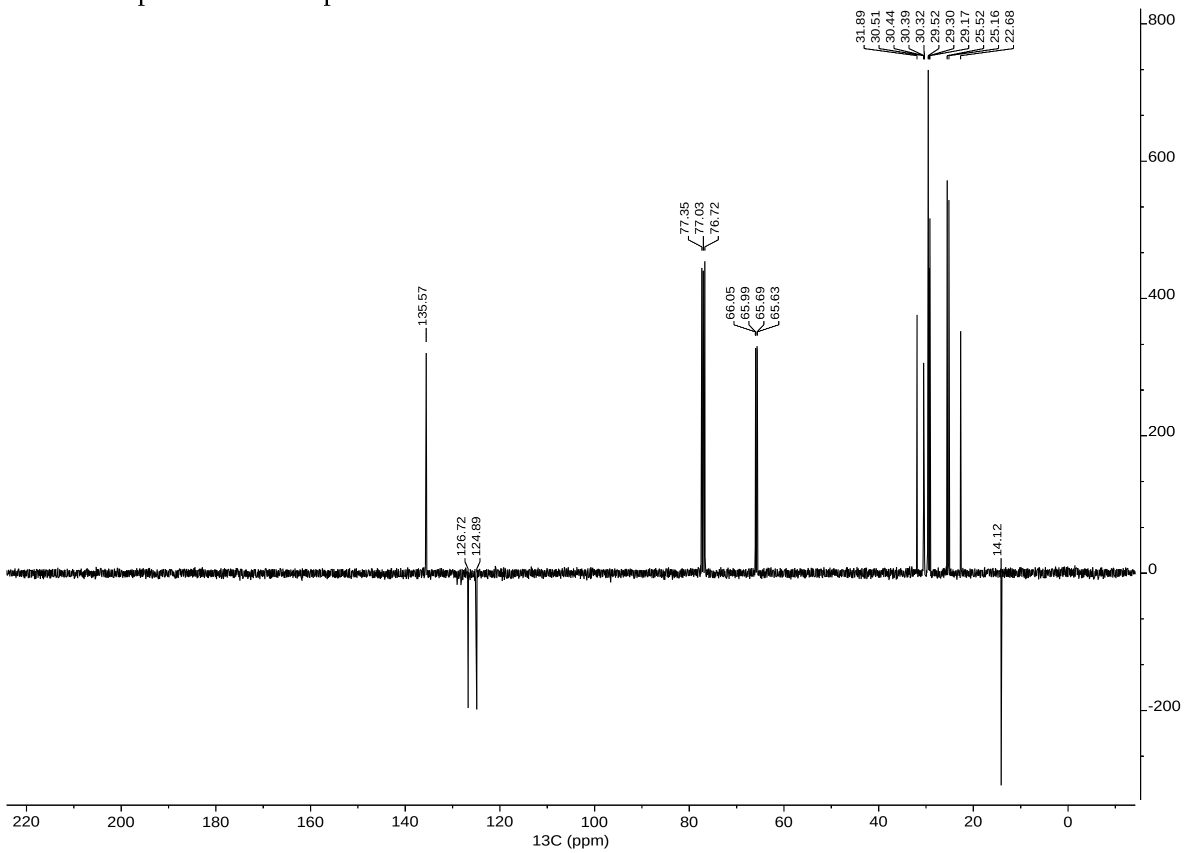

^{31}P{^{1}H} NMR spectrum of compound S62

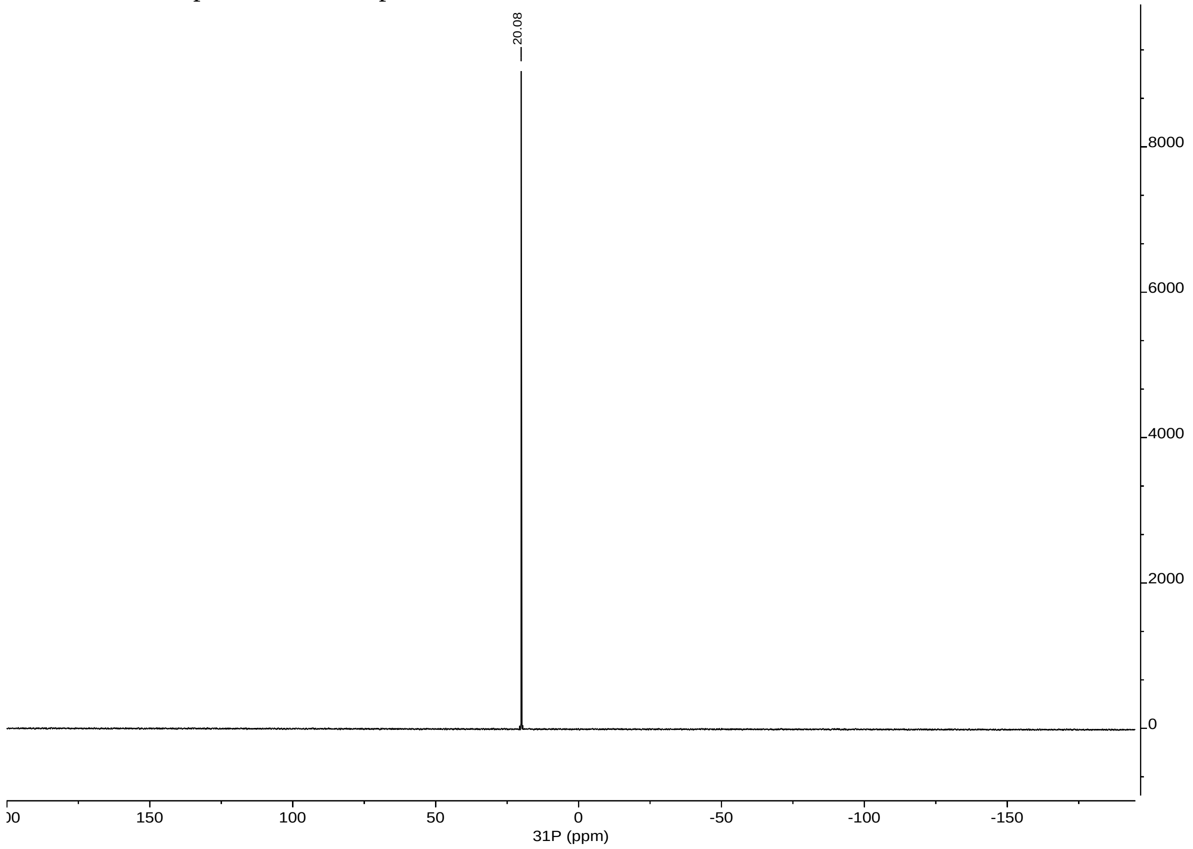

Bis((*Z*)-dec-4-en-1-yl) hexane-1,6-diyl bis(vinylphosphonate) (S63)

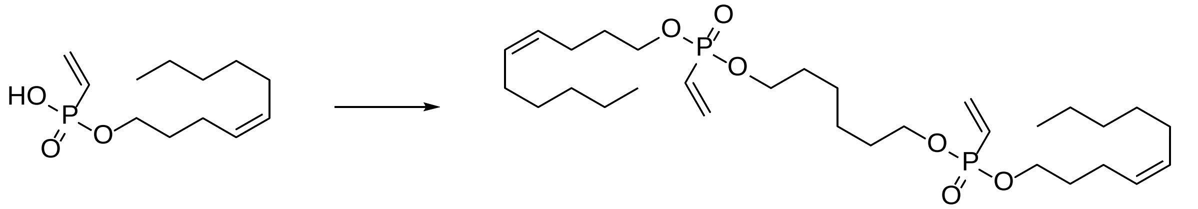

The title compound was prepared according to general method **C** from mono alkyl vinylphosphonate **S34** (0.95 g, 3.86 mmol) and 1,6-dibromohexane (96%, 206 μL, 1.29 mmol) in 16% yield (118 mg, 205 μmol) as a colourless oil.

^{1}H NMR (401 MHz, $CDCl_3$) δ 6.37–5.95 (m, 6H, C*H*=C*H*$_2$), 5.47–5.26 (m, 4H, C*H*=C*H*), 4.05–3.97 (m, 8H, OC*H*$_2$), 2.12 (q, *J* = 7.4 Hz, 4H, O(CH_2)$_2$C*H*$_2$CH), 2.01 (q, *J* = 6.9 Hz, 4H, C*H*$_2$(CH_2)$_3$$CH_3$), 1.78–1.61 (m, 8H, OCH$_2$C*H*$_2$), 1.45–1.20 (m, 16H, O($CH_2$)$_2$(C*H*$_2$)$_2$, (C*H*$_2$)$_3$$CH_3$), 0.92–0.84 (m, 6H, C*H*$_3$).

^{13}C NMR (101 MHz, $CDCl_3$) δ 135.69 (=*C*H$_2$), 131.43 (*C*H(CH_2)$_4$), 128.05 (O(CH_2)$_3$*C*H), 125.96 (d, *J* = 184.0 Hz, P*C*H), 65.78 (d, *J* = 5.8 Hz), 65.50 (d, *J* = 5.8 Hz, O*C*H$_2$), 31.66 (*C*H$_2$CH$_2$CH$_3$), 30.65 (d, *J* = 6.4 Hz), 30.51 (d, *J* = 6.4 Hz, OCH$_2$*C*H$_2$), 29.47 (*C*H$_2$(CH_2)$_2$$CH_3$), 27.32 (*C*H$_2$($CH_2$)$_3$$CH_3$), 25.31 (O($CH_2$)$_2$(*C*H$_2$)$_2$), 23.34 (O($CH_2$)$_2$*C*H$_2$CH), 22.71 (*C*H$_2CH_3$), 14.22 (*C*H$_3$).

^{31}P{^{1}H} NMR (162 MHz, $CDCl_3$) δ 20.10.

IR ν_{max} ($CHCl_3$) 3088 (vw), 2960 (s), 2933 (s), 2873 (m), 2862 (m), 1615 (w), 1467 (m), ~1457 (m, sh), 1434 (w), 1401 (m), 1380 (w), ~1279 (m, sh), 1234 (s), 1198 (m), 1008 (vs, br), ~989 (vs, sh), 606 (w).

HR-MS(ESI$^+$): For $C_{30}H_{57}O_6P_2$ $(M+H)^+$ *m*/*z* calculated 575.36249, found 575.36281. For $C_{30}H_{56}O_6NaP_2$ $(M+Na)^+$ *m*/*z* calculated 597.34443, found 597.34454.

^{1}H NMR spectrum of compound **S63**

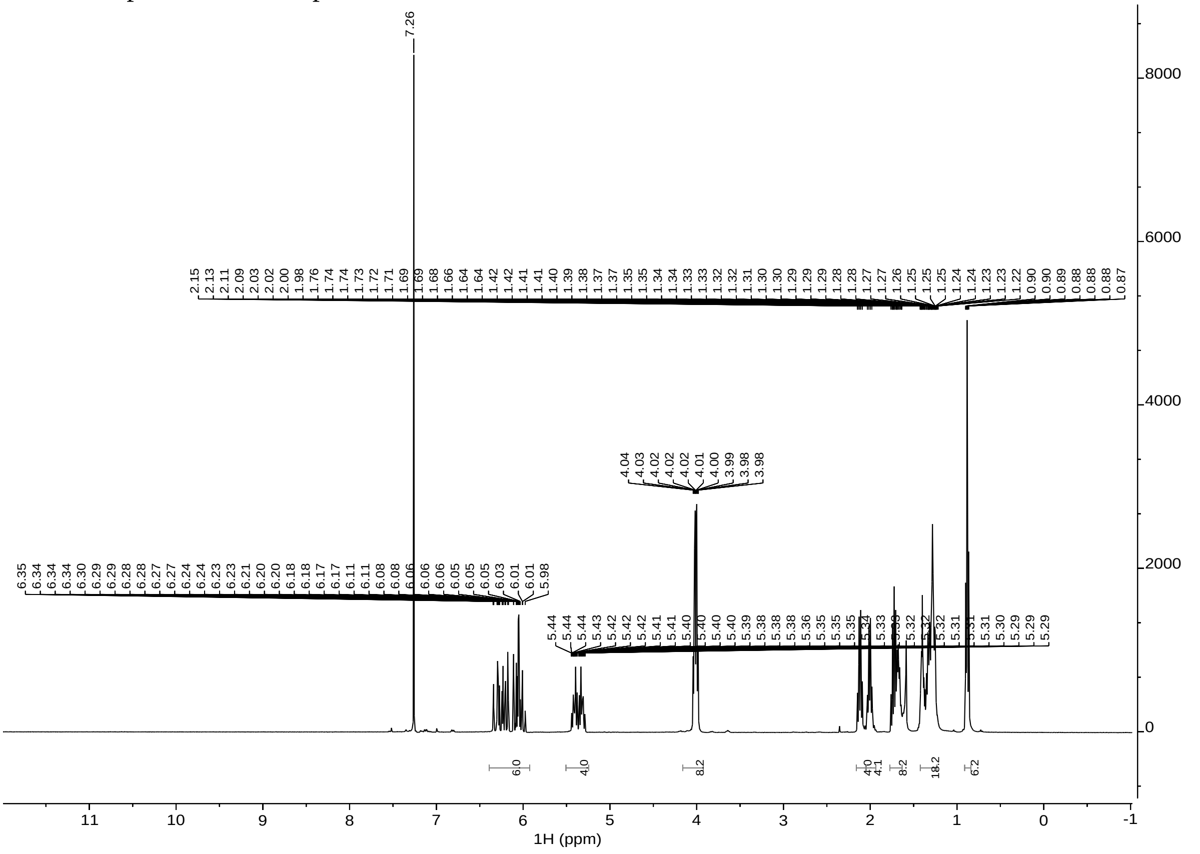

^{13}C NMR spectrum of compound **S63**

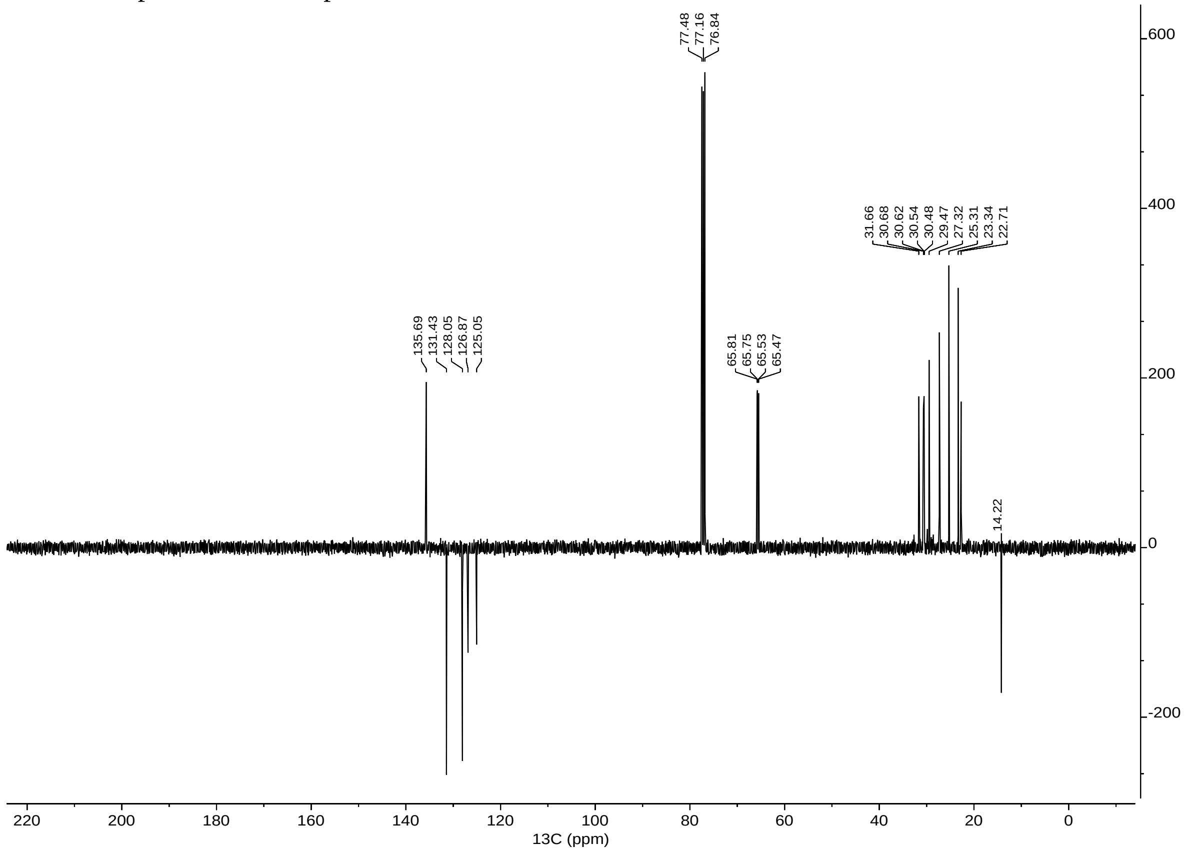

$^{31}P\{^1H\}$ NMR spectrum of compound **S63**

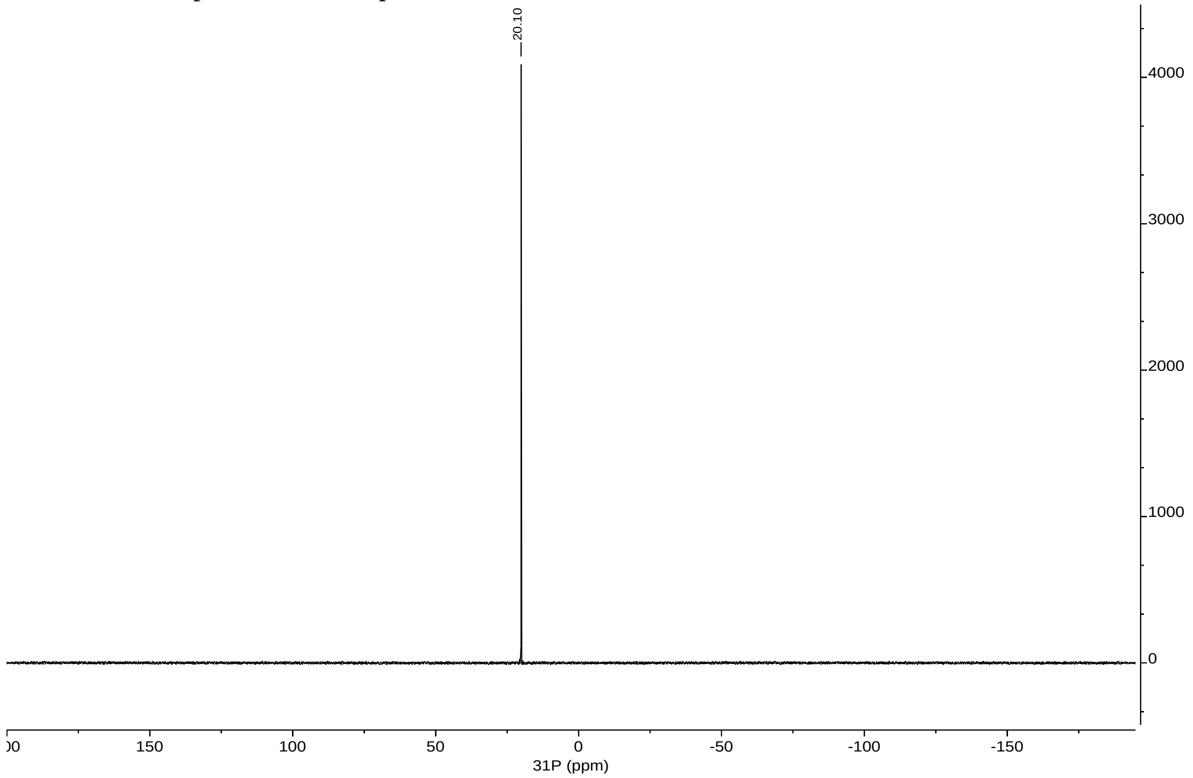

Bis(2-(adamantan-1-yl)ethyl) hexane-1,6-diyl bis(vinylphosphonate) (S64)

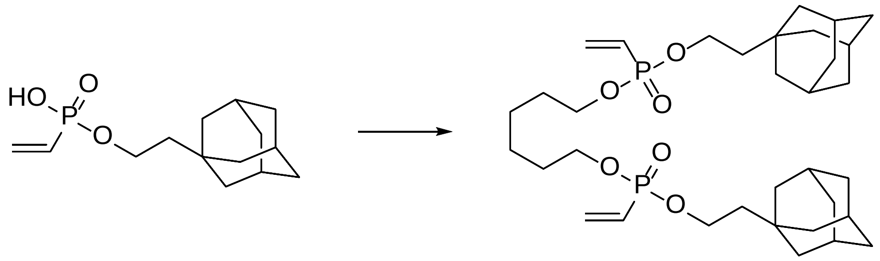

The title compound was prepared according to general method **C** from mono alkyl vinylphosphonate **S36** (2.18 g, 8.05 mmol) and 1,6-dibromohexane (96%, 0.43 mL, 2.68 mmol) in 44% yield (0.79 g, 1.26 mmol) as a colourless oil.

^{1}H NMR (401 MHz, $CDCl_3$) δ 6.38–5.94 (m, 6H, C*H*=C*H*$_2$), 4.08 (dtd, *J* = 8.9, 7.1, 2.1 Hz, 4H, OC*H*$_2$CH$_2$C$_{quat}$), 4.00 (q, *J* = 6.7 Hz, 4H, OC*H*$_2$(CH$_2$)$_2$), 1.94 (p, *J* = 3.1 Hz, 6H, C*H*), 1.74–1.57 (m, 16H, C$_{quat}$CH$_2$CH, OCH$_2$C*H*$_2$CH$_2$), 1.51 (d, *J* = 2.9 Hz, 12H, *c*-(CHC*H*$_2$)$_3$), 1.48 (dtd, *J* = 12.7, 7.5, 1.9 Hz, 4H, OCH$_2$C*H*$_2$C$_{quat}$), 1.43–1.37 (m, 4H, O(CH$_2$)$_2$C*H*$_2$).

^{13}C NMR (101 MHz, $CDCl_3$) δ 135.68 (d, *J* = 2.0 Hz, =*C*H$_2$), 126.00 (d, *J* = 183.7 Hz, P*C*H), 65.81 (d, *J* = 5.9 Hz, O*C*H$_2$(CH$_2$)$_2$), 62.49 (d, *J* = 5.8 Hz, O*C*H$_2$CH$_2$C$_{quat}$), 44.45 (d, *J* = 6.3 Hz, OCH$_2$*C*H$_2$C$_{quat}$), 42.65 (*c*-(CH*C*H$_2$)$_3$), 37.11 (C$_{quat}$*C*H$_2$CH), 31.92 (*C*$_{quat}$), 30.51 (d, *J* = 6.4 Hz, OCH$_2$*C*H$_2$CH$_2$), 28.68 (*C*H), 25.32 (O(CH$_2$)$_2$*C*H$_2$).

^{31}P{^{1}H} NMR (162 MHz, $CDCl_3$) δ 20.13.

IR ν_{max} ($CHCl_3$) 3091 (vw), 2927 (vs), 2905 (vs), 2849 (s), 2797 (vw), 2753 (vw), 2675 (w), 2660 (w), 2637 (vw), 1614 (w), 1451 (m), 1400 (m), 1347 (w), 1241 (s), 1099 (m), ~1025 (s, sh), 1006 (vs), 998 (vs), 988 (vs), 973 (s), 883 (vw).

HR-MS(ESI^{+}): For $C_{34}H_{57}O_6P_2$ $(M+H)^+$ *m*/*z* calculated 623.36249, found 623.36220. For $C_{34}H_{56}O_6NaP_2$ $(M+Na)^+$ *m*/*z* calculated 645.34443, found 645.34397.

^{1}H NMR spectrum of compound **S64**

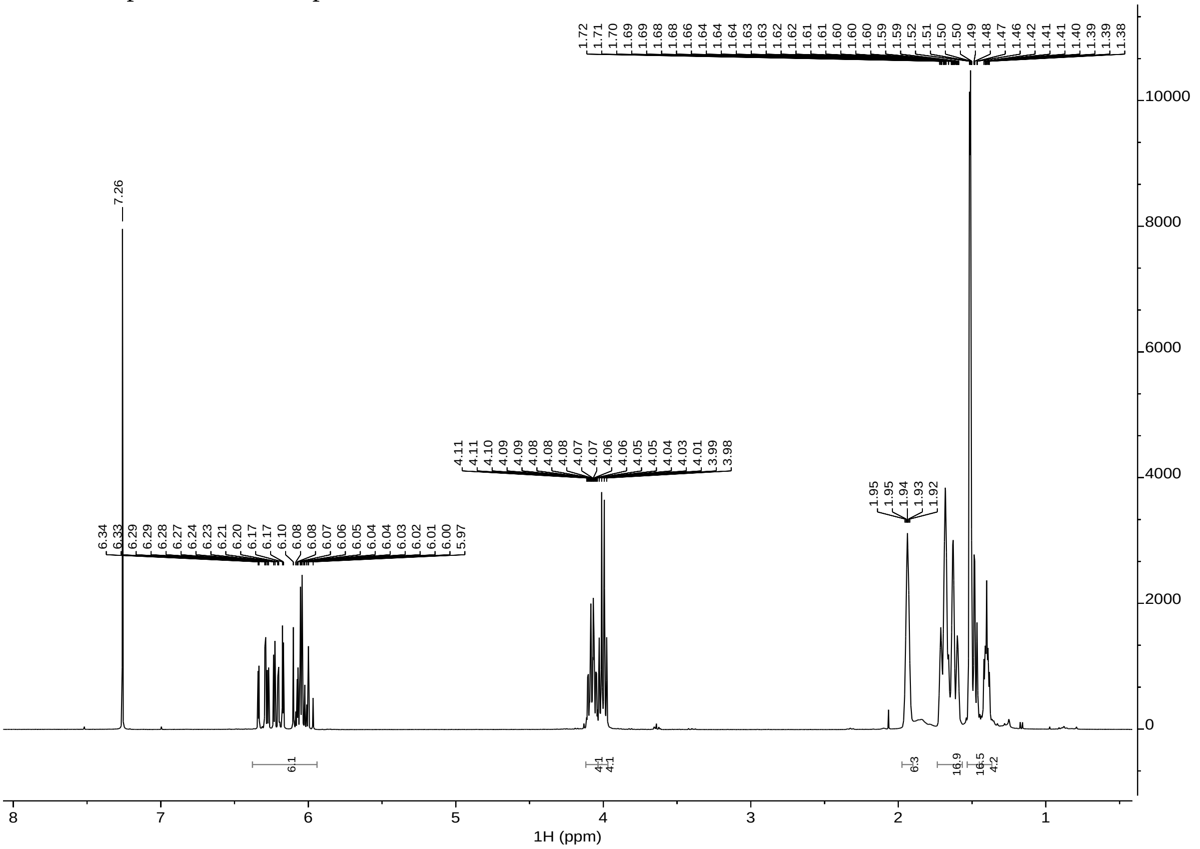

^{13}C NMR spectrum of compound **S64**

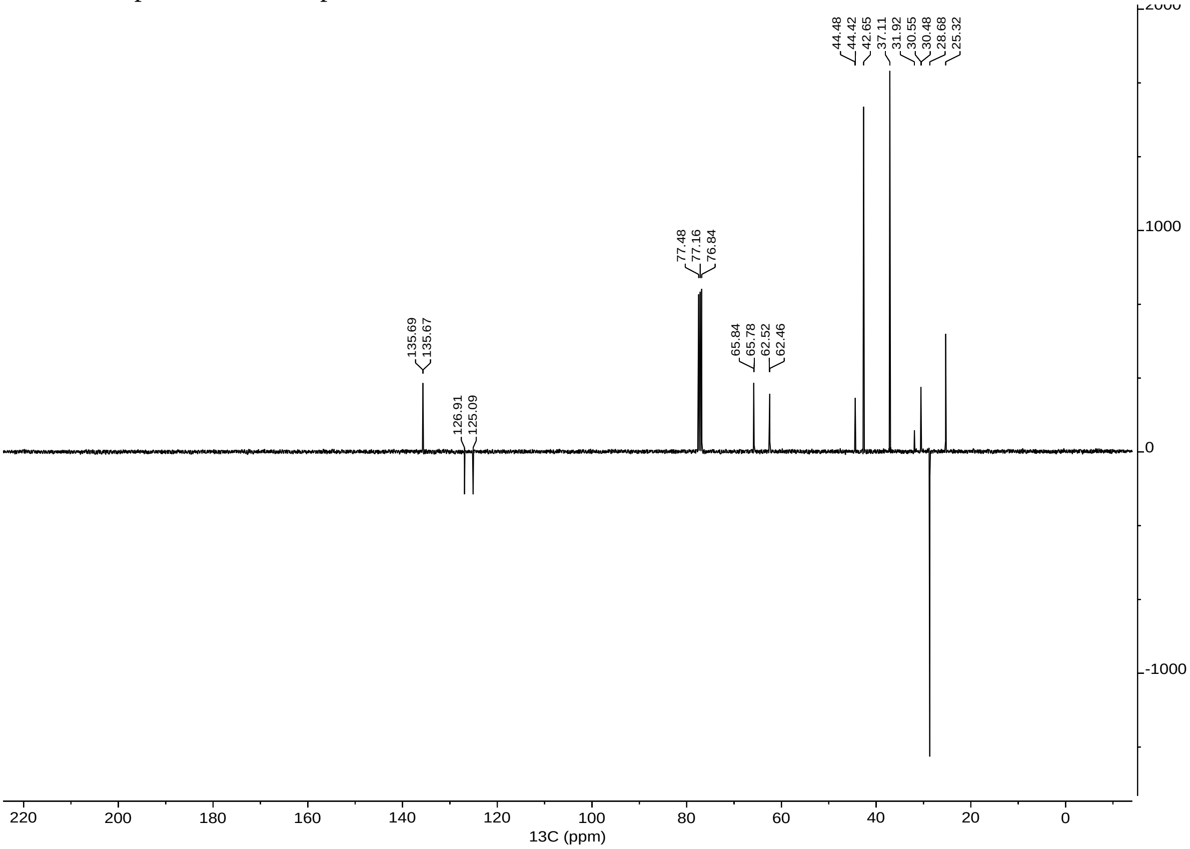

^{31}P{^{1}H} NMR spectrum of compound **S64**

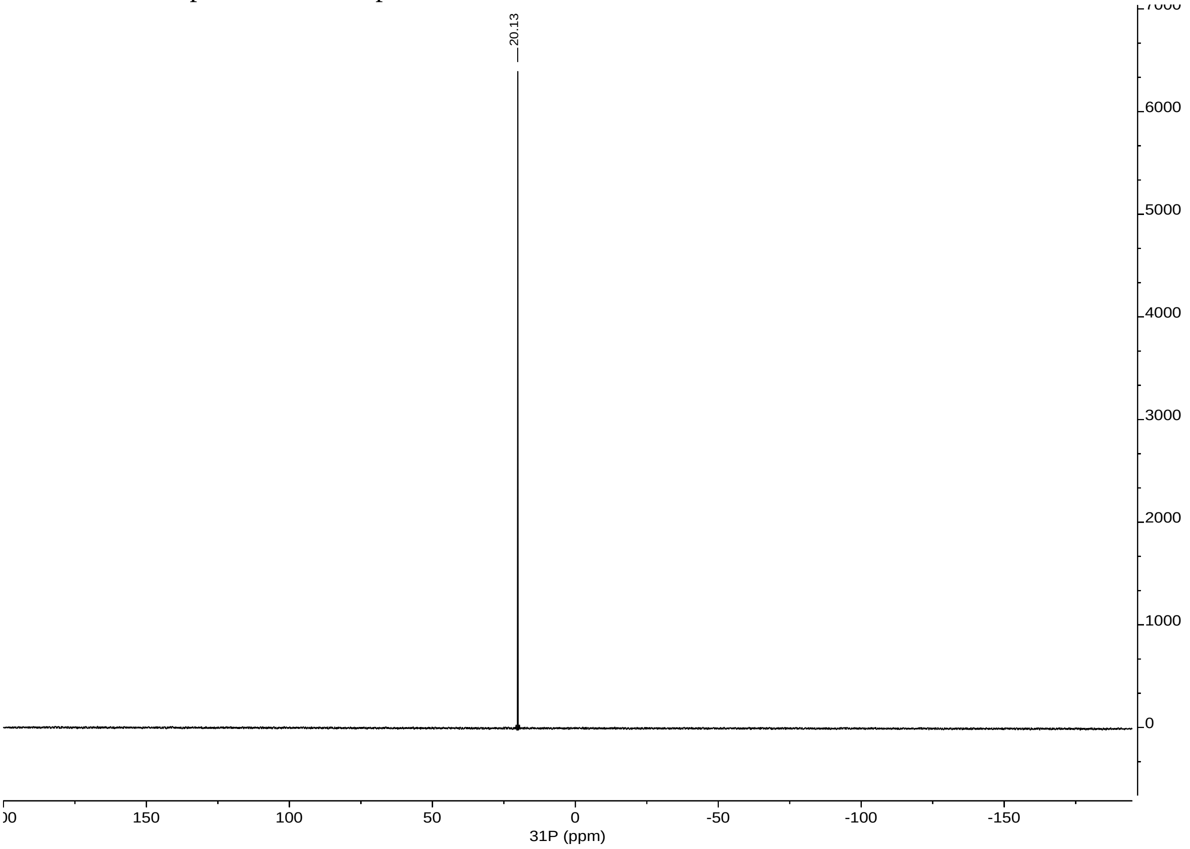

Heptane-1,7-diyl bis(4,4,4-trifluorobutyl) bis(vinylphosphonate) (S65)

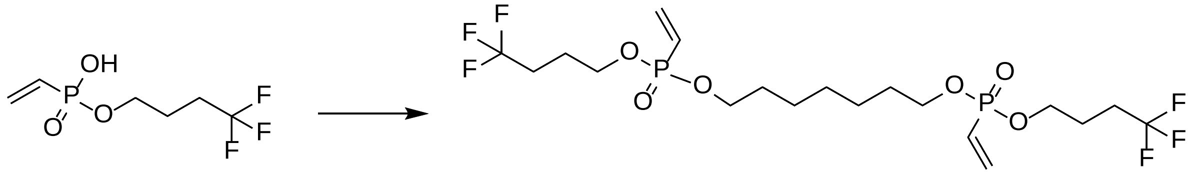

The title compound was prepared according to general method **C** from mono alkyl vinylphosphonate **S21** (1.10 g, 5.04 mmol) and 1,7-dibromoheptane (0.43 g, 1.67 mmol) in 70% yield (0.62 g, 1.17 mmol) as a colourless oil.

^{1}H NMR (401 MHz, $CDCl_3$): 6.37–5.97 (m, 6H, CH=CH_2), 4.13–4.05 (m, 4H, $CF_3(CH_2)_2CH_2O$), 4.04–3.98 (m, 4H, $OCH_2(CH_2)_5CH_2O$), 2.28–2.16 (m, 4H, $CF_3CH_2(CH_2)_2O$), 1.97–1.90 (m, 4H, $CF_3CH_2CH_2CH_2O$), 1.70–1.65 (m, 4H, $OCH_2CH_2(CH_2)_3CH_2CH_2O$), 1.41–1.25 (m, 6H, $OCH_2CH_2(CH_2)_3CH_2CH_2O$).

^{13}C NMR (101 MHz, $CDCl_3$): 136.28 (d, J = 2.4 Hz, CH=CH$_2$), 127.04 (q, J = 276.2 Hz, CF_3), 125.51 (d, J = 183.9 Hz, CH=CH$_2$), 66.04 (d, J = 5.4 Hz, $OCH_2(CH_2)_5CH_2O$), 64.01 (d, J = 5.6 Hz, $CF_3(CH_2)_2CH_2O$), 30.39 (d, J = 6.4 Hz, $OCH_2CH_2(CH_2)_3CH_2CH_2O$), 30.48 (q, J = 29.3 Hz, CF_3CH_2), 28.82 ($O(CH_2)_3CH_2(CH_2)_3O$), 25.54 ($O(CH_2)_2CH_2CH_2CH_2(CH_2)_2O$), 23.46 (dq, J = 6.3, 3.0 Hz, $CF_3CH_2CH_2$).

^{31}P{^{1}H} NMR (162 MHz, $CDCl_3$): 20.16.

^{19}F NMR (377 MHz, $CDCl_3$) δ -66.33 (t, J = 10.8 Hz, CF_3).

IR ν_{max} 3091 (vw), 2962 (m), 2861 (m), 2180 (m, sh), 1451 (m), 1340 (s), 1256 (vs), 1156 (vs), 1135 (s), 1086–1014 (vs), 987 (s), 825 (m), 855 (m).

HR-MS(ESI$^+$): For $C_{19}H_{32}O_6F_6NaP_2$ (M+Na)$^+$ m/z calculated 555.14705, found 555.14685.

^{1}H NMR spectrum of compound **S65**

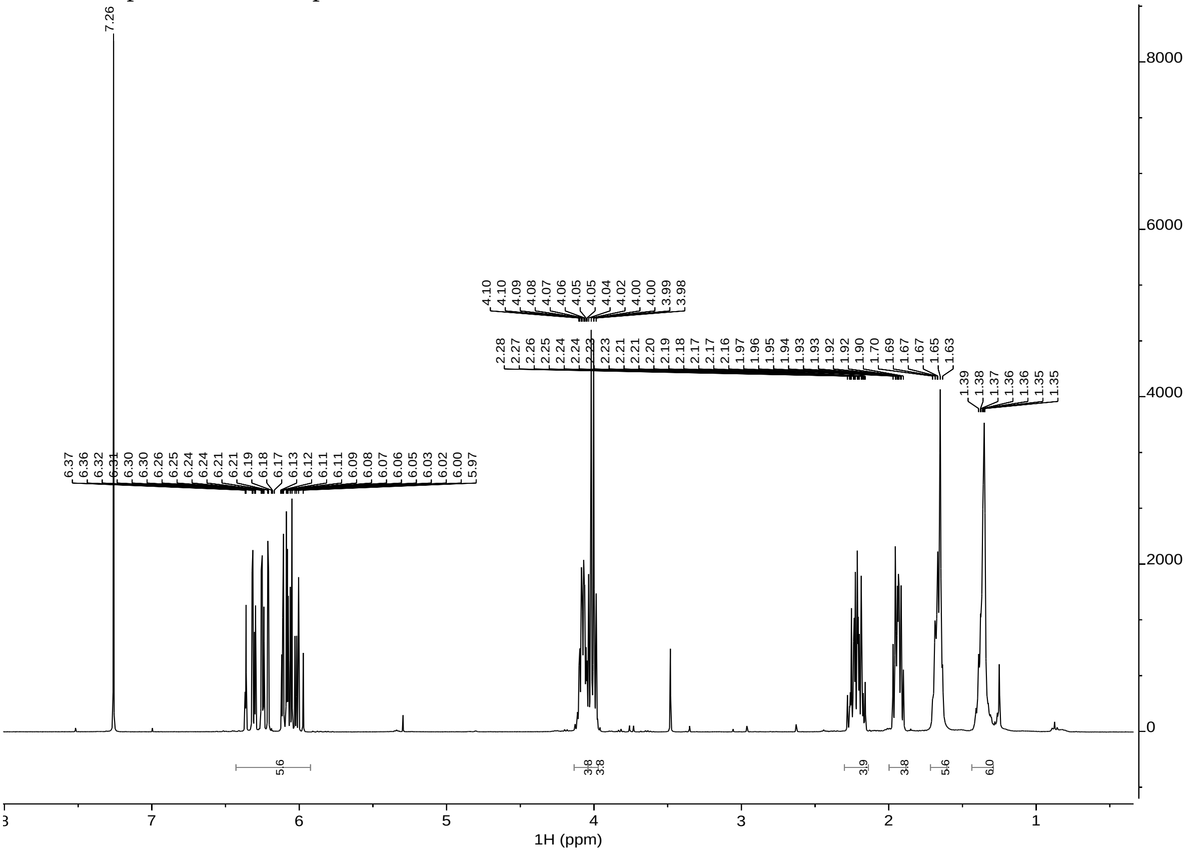

^{13}C NMR spectrum of compound **S65**

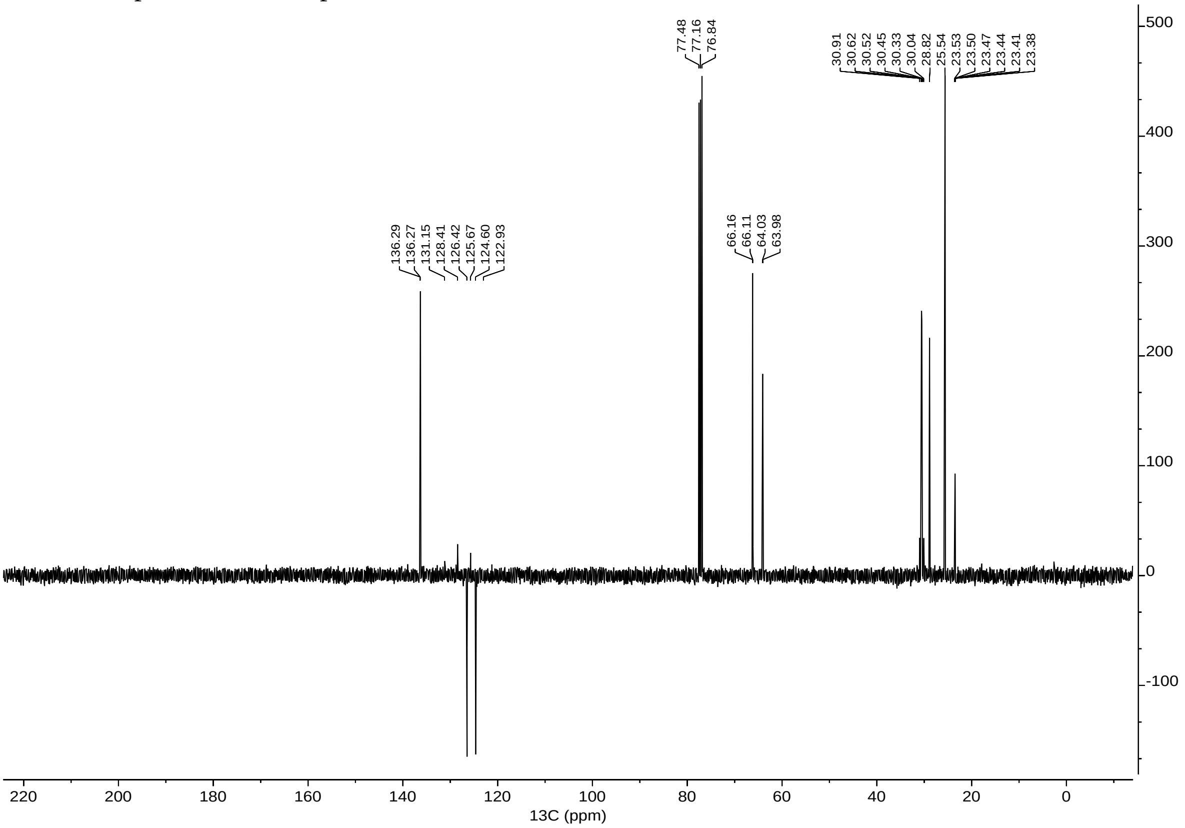

$^{31}P\{^1H\}$ NMR spectrum of compound **S65**

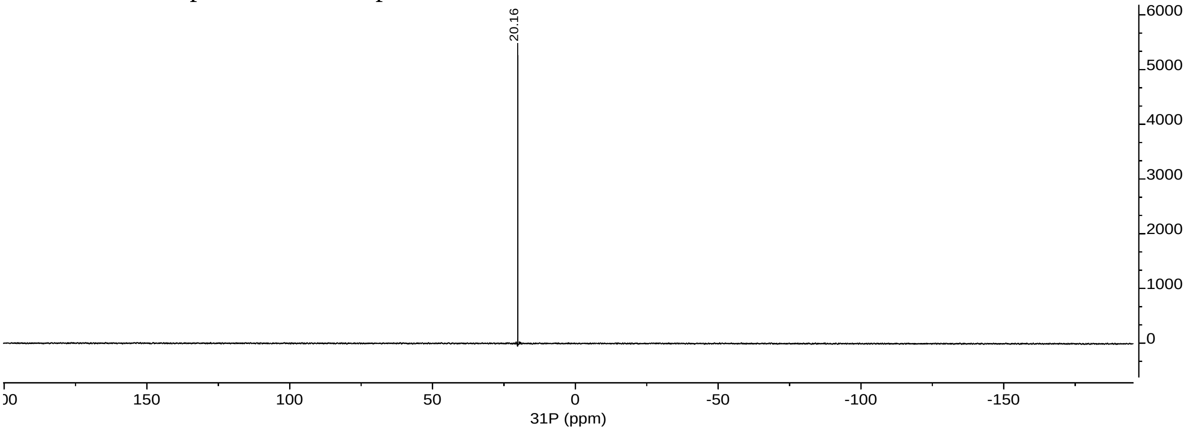

^{19}F NMR spectrum of compound **S65**

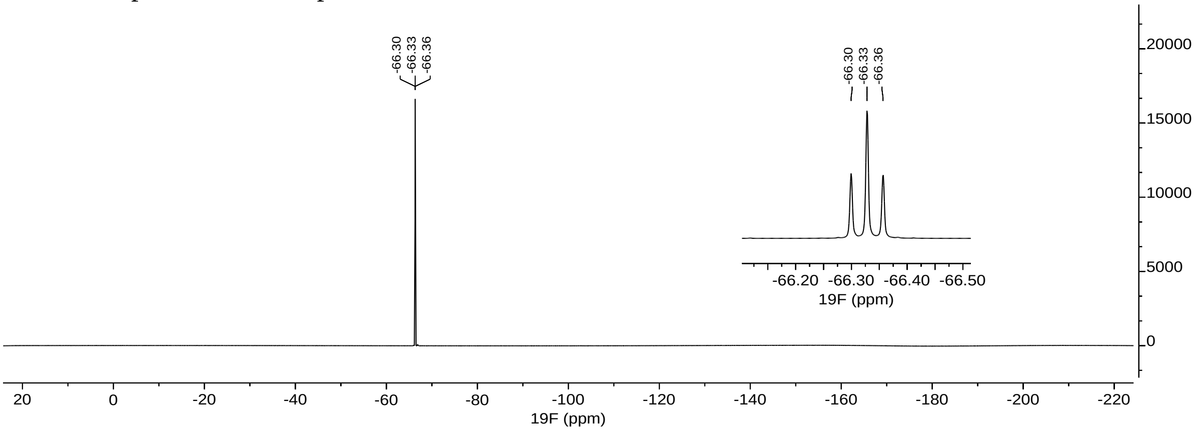

Heptane-1,7-diyl diheptyl bis(vinylphosphonate) (S66)

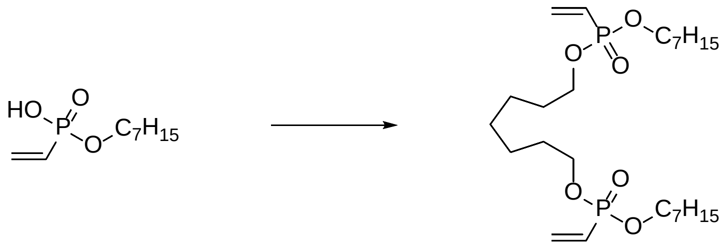

The title compound was prepared according to general method **C** from mono alkyl vinylphosphonate **S25** (2.63 g, 12.8 mmol) and 1,7-dibromoheptane (99%, 725 µL, 4.26 mmol) in 37% yield (800 mg, 1.57 mmol) as a colourless oil.

^{1}H NMR (401 MHz, $CDCl_3$) δ 6.36–5.95 (m, 6H, C*H*=C*H*$_2$), 4.00 (q, *J* = 6.9 Hz, 8H, OC*H*$_2$), 1.66 (p, *J* = 6.6 Hz, 8H, OCH_2CH_2), 1.43–1.20 (m, 22H, $O(CH_2)_2(CH_2)_2$, $(CH_2)_2CH_3$), 0.91–0.83 (m, 6H, C*H*$_3$).

^{13}C NMR (101 MHz, $CDCl_3$) δ 135.69 (=*C*H$_2$), 125.94 (d, *J* = 184.1 Hz, P*C*H), 66.14 (d, *J* = 5.9 Hz), 65.92 (d, *J* = 5.9 Hz, O*C*H$_2$), 31.84 (*C*H$_2$CH$_2$CH$_3$), 30.60 (d, *J* = 6.5 Hz), 30.50 (d, *J* = 6.2 Hz, OCH$_2$*C*H$_2$), 28.95, 28.83, 25.60, 25.55 (O(CH$_2$)$_2$(*C*H$_2$)$_2$), 22.70 (*C*H$_2$CH$_3$), 14.19 (*C*H$_3$).

^{31}P{^{1}H} NMR (162 MHz, $CDCl_3$) δ 20.06.

IR ν_{max} ($CHCl_3$) 3090 (vw), 2958 (s), 2932 (s), 2782 (m), 2859 (s), 1614 (w), ~1475 (w, sh), 1467 (m), ~1455 (m, sh), 1434 (w), 1400 (m), 1380 (w), 1279 (m), 1240 (s), ~1065 (s, sh), 1051 (s), 1007 (vs), 988 (vs), 606 (w),

HR-MS(ESI$^+$): For $C_{25}H_{51}O_6P_2$ $(M+H)^+$ *m/z* calculated 509.31554, found 509.31559. For $C_{25}H_{50}O_6NaP_2$ $(M+Na)^+$ *m/z* calculated 531.29748, found 531.29751.

^{1}H NMR spectrum of compound **S66**

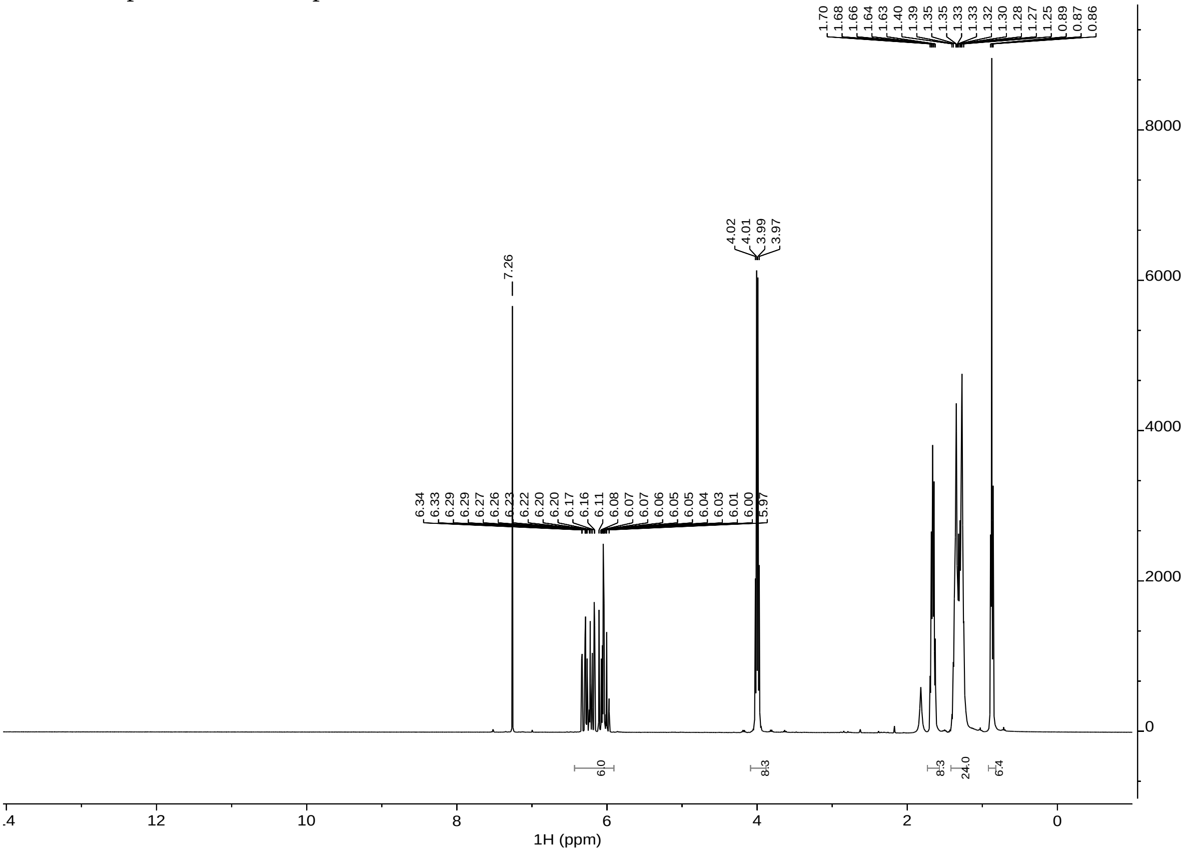

^{13}C NMR spectrum of compound **S66**

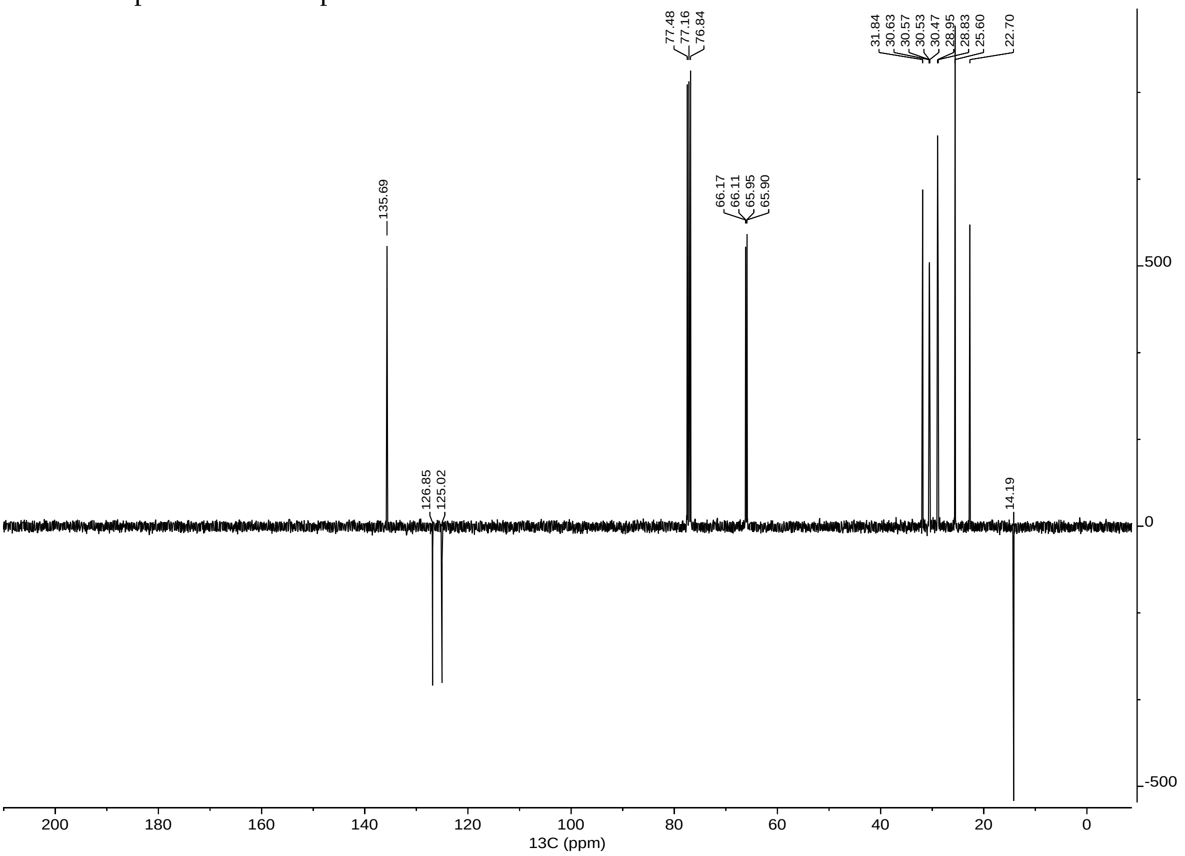

$^{31}P\{^{1}H\}$ NMR spectrum of compound **S66**

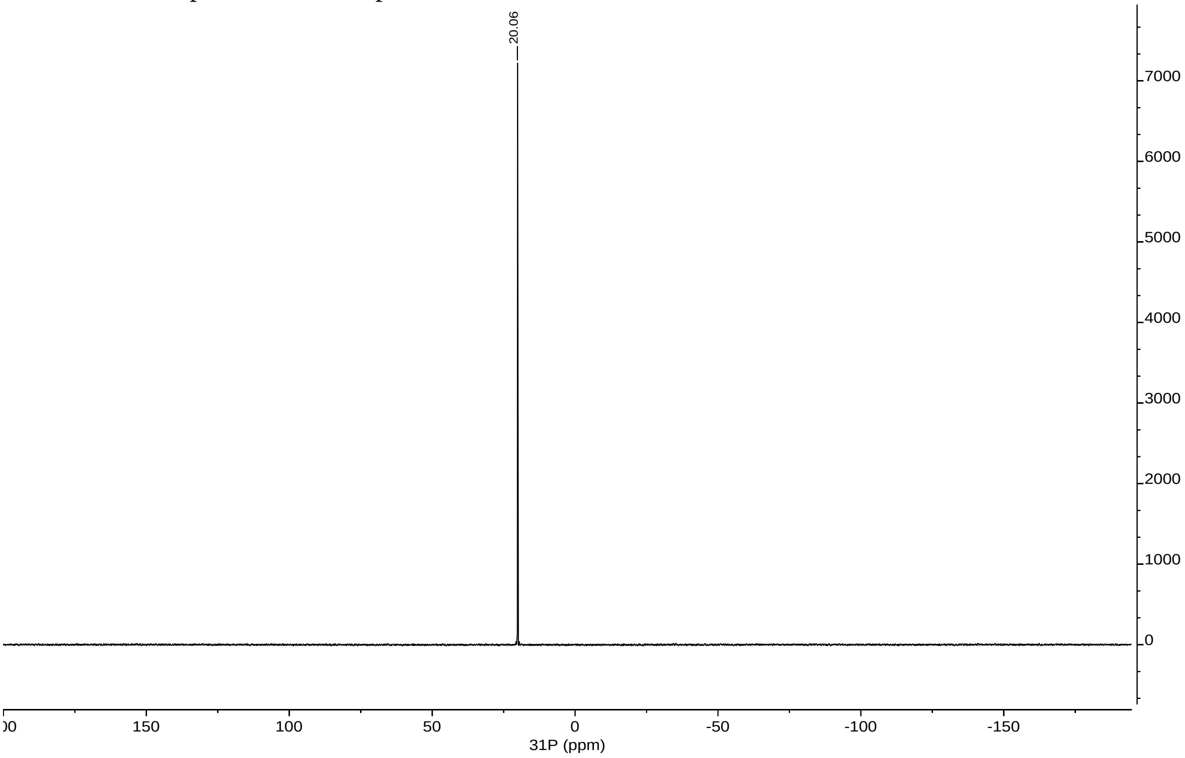

Bis((*Z*)-hept-3-en-1-yl) heptane-1,7-diyl bis(vinylphosphonate) (S67)

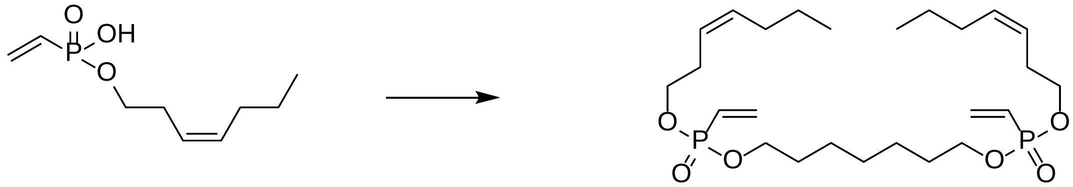

The title compound was prepared according to general method **C** from mono alkyl vinylphosphonate **S26** (2.0 g, 9.78 mmol) and 1,7-dibromoheptane (0.84 g, 3.26 mmol) in 76% yield (1.25 g, 2.48 mmol) as a colourless oil.

^{1}H NMR (401 MHz, $CDCl_3$): 6.34–5.97 (m, 6H, C*H*=C*H*$_2$), 5.54–5.32 (m, 2H, $CH_3(CH_2)_2$CHC*H*CH_2CH_2O), 4.02–3.96 (m, 8H, C*H*$_2$O), 2.45–2.40 (m, 4H, $CH_3CH_2CH_2$CHCHC*H*$_2$$CH_2O$), 2.04–1.98 (m, 4H, CH_3CH_2C*H*$_2$$CHCHCH_2CH_2O$), 1.69–1.62 (m, 4H, OCH_2C*H*$_2$$(CH_2)_3$C*H*$_2$$CH_2O$), 1.41–1.24 (m, 10H, CH_3C*H*$_2$$CH_2CHCHCH_2CH_2O$, OCH_2CH_2(C*H*$_2$)$_3$$CH_2CH_2O$), 0.89 (t, 6H, *J* = 7.4 Hz, C*H*$_3$).

^{13}C NMR (101 MHz, $CDCl_3$): 135.67 (d, *J* = 1.9 Hz, CH=*C*H$_2$), 133.17 ($CH_3(CH_2)_2$*C*$HCHCH_2CH_2O$), 125.94 (d, *J* = 183.4 Hz, *C*H=CH$_2$), 124.05 ($CH_3(CH_2)_2$CH*C*HCH_2CH_2O), 65.91 (d, *J* = 6.1 Hz, $CH_3CH_2CH_2CHCHCH_2$*C*H$_2$O), 65.35 (d, *J* = 5.3 Hz, O*C*H$_2$$CH_2(CH_2)_3CH_2$*C*H$_2$O), 30.52 (d, *J* = 6.6 Hz, OCH_2CH_2*C*H$_2$$CH_2$*C*H$_2$$CH_2CH_2O$), 29.53 ($CH_3CH_2$*C*H$_2$$CHCHCH_2CH_2O$), 28.86 ($OCH_2CH_2$*C*H$_2$$CH_2$*C*H$_2$$CH_2CH_2O$), 28.83 (d, *J* = 5.9 Hz, $CH_3CH_2CH_2CHCH$*C*H$_2$$CH_2O$), 25.56 ($OCH_2CH_2CH_2$*C*H$_2$$CH_2CH_2CH_2O$), 22.81 ($CH_3$*C*H$_2$$CH_2CHCHCH_2CH_2O$), 13.89 (*C*H$_3$$(CH_2)_2CHCHCH_2CH_2O$).

^{31}P{^{1}H} NMR (162 MHz, $CDCl_3$): 20.06.

IR ν_{max} (KBr) 3090 (w), 2961 (s), 2932 (s), 2863 (s), 1655 (w), 1614 (w), 1466 (m), 1458 (m), 1279 (m), 1241 (s), 1070–950 (vs, br), 987 (s, sh), 857 (m).

HR-MS(ESI$^+$): For $C_{25}H_{47}O_6P_2$ $(M+H)^+$ *m*/*z* calculated 505.28424, found 505.28427.

^{1}H NMR spectrum of compound **S67**

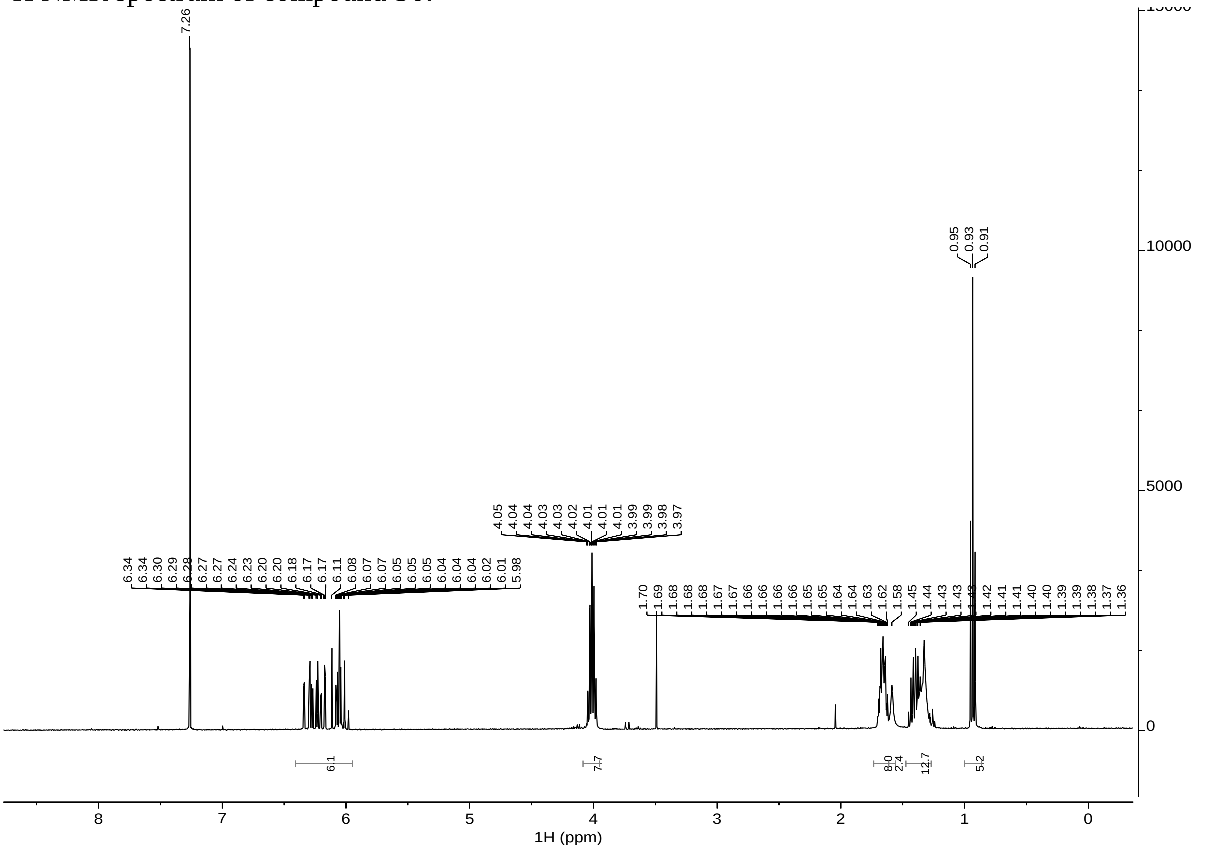

^{13}C NMR spectrum of compound **S67**

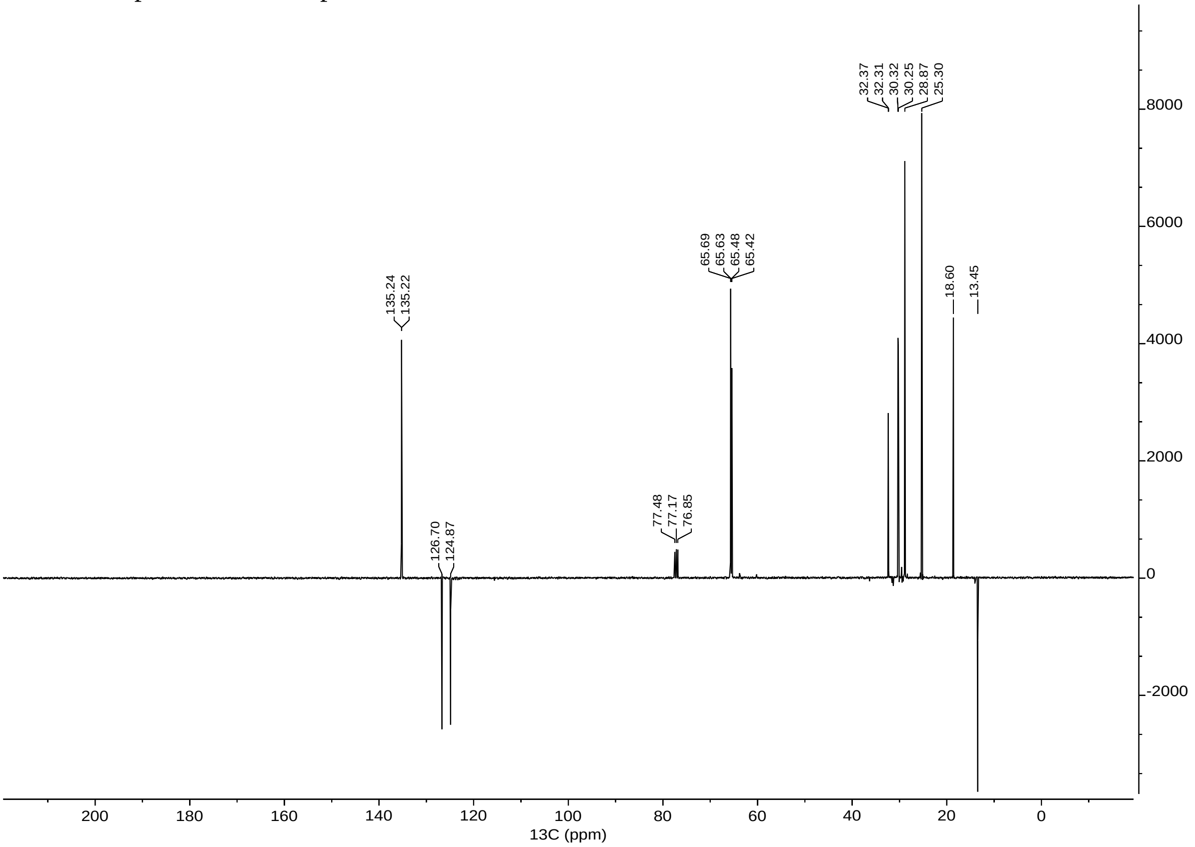

^{31}P{^{1}H} NMR spectrum of compound **S67**

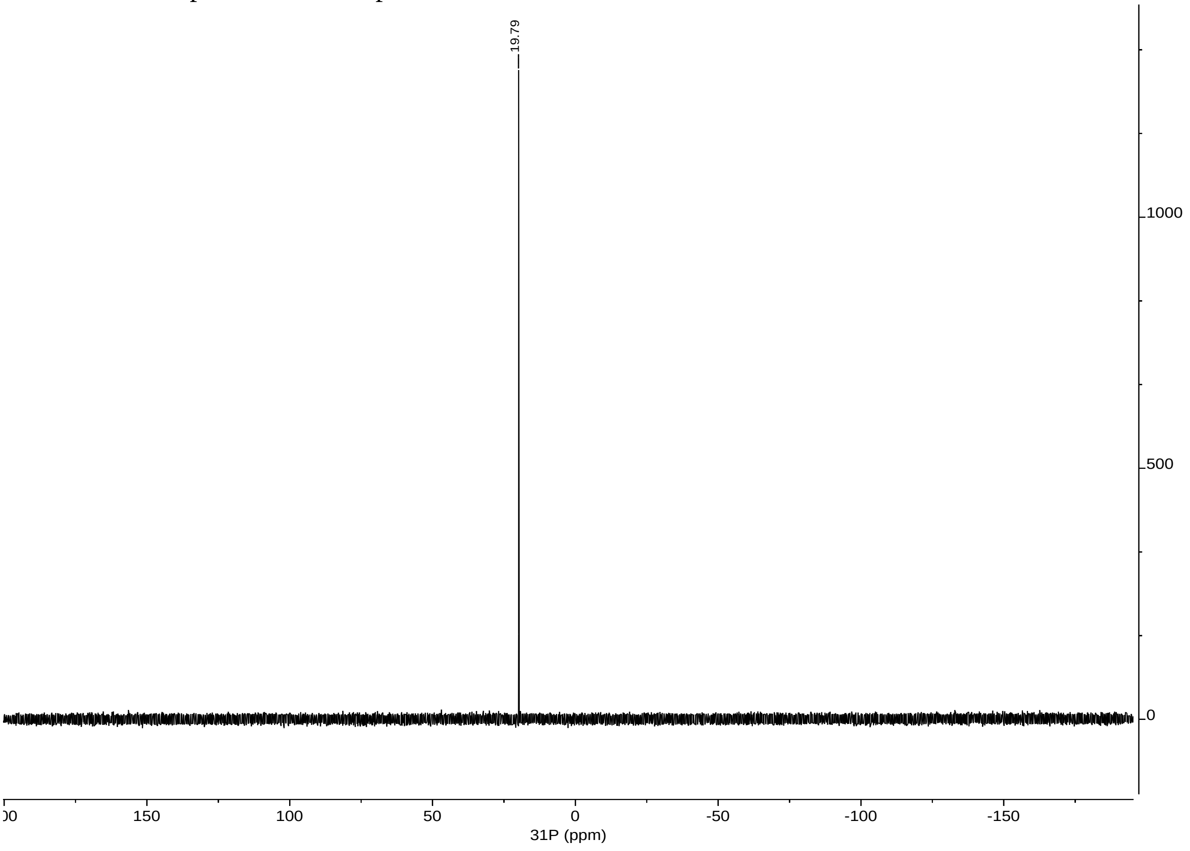

Diisobutyl octane-1,8-diyl bis(vinylphosphonate) (S68)

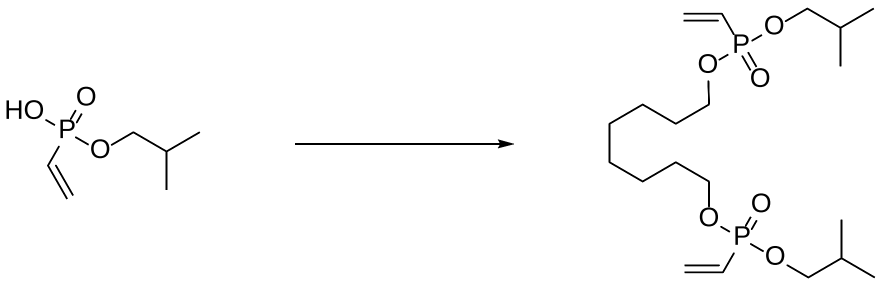

The title compound was prepared according to general method **C** from mono alkyl vinylphosphonate **S19** (500 mg, 3.05 mmol) and 1,8-dibromooctane (95%, 196 μL, 1.01 mmol) in 96% yield (425 mg, 0.97 mmol) as a colourless oil.

^{1}H NMR (401 MHz, $CDCl_3$) δ 6.46–5.95 (m, 6H, C*H*=C*H*$_2$), 4.00 (q, *J* = 6.8 Hz, 4H, OC*H*$_2$CH$_2$), 3.77 (td, *J* = 6.6, 1.7 Hz, 4H, OC*H*$_2$CH), 1.93 (dp, *J* = 13.3, 6.7 Hz, 2H, OCH$_2$C*H*), 1.74–1.61 (m, 4H, OCH$_2$C*H*$_2$), 1.44–1.22 (m, 8H, O(CH$_2$)$_2$(C*H*$_2$)$_2$), 0.93 (dd, *J* = 6.7, 0.7 Hz, 12H, C*H*$_3$).

^{13}C NMR (101 MHz, $CDCl_3$) δ 135.59 (d, *J* = 2.0 Hz, =*C*H$_2$), 125.93 (d, *J* = 184.4 Hz, P*C*H), 71.93 (d, *J* = 6.0 Hz, O*C*H$_2$CH), 65.97 (d, *J* = 5.9 Hz, O*C*H$_2$CH$_2$), 30.56 (d, *J* = 6.4 Hz, OCH$_2$*C*H$_2$), 29.30 (d, *J* = 6.7 Hz, OCH$_2$*C*H), 29.16, 25.57 (O(CH$_2$)$_2$(*C*H$_2$)$_2$), 18.86 (d, *J* = 1.9 Hz, *C*H$_3$).

^{31}P{^{1}H} NMR (162 MHz, $CDCl_3$) δ 19.98.

IR ν_{max} ($CHCl_3$) 2964 (m), 2936 (m), 2875 (m), 2860 (w), 1614 (vw), 1471 (w), 1467 (w), 1370 (w), 914 (vw).

HR-MS(ESI$^+$): For $C_{20}H_{40}O_6NaP_2$ $(M+Na)^+$ *m*/*z* calculated 461.21923, found 461.21892.

^{1}H NMR spectrum of compound **S68**

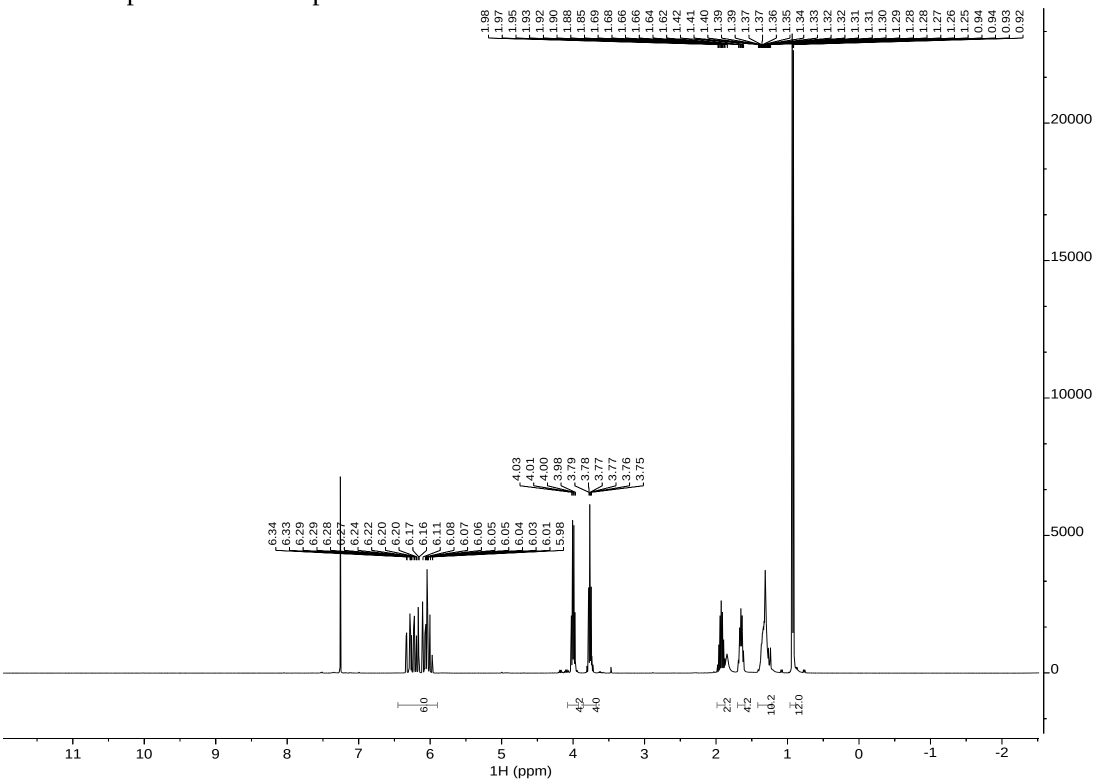

^{13}C NMR spectrum of compound **S68**

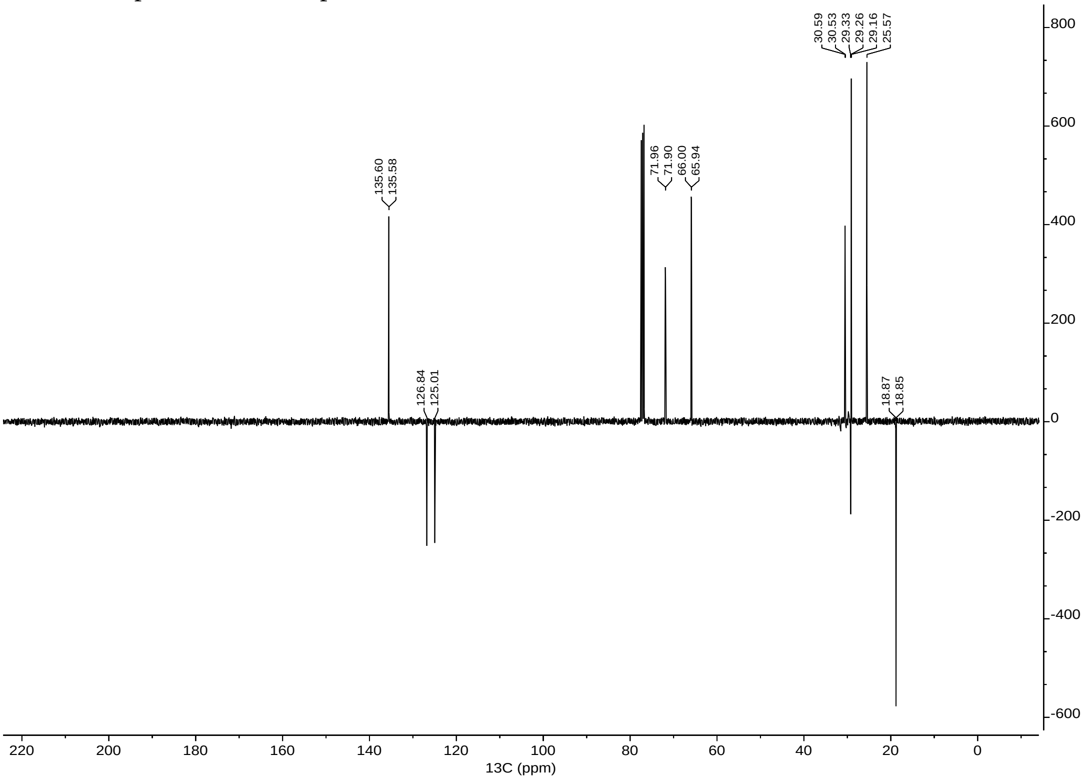

^{31}P{^{1}H} NMR spectrum of compound **S68**

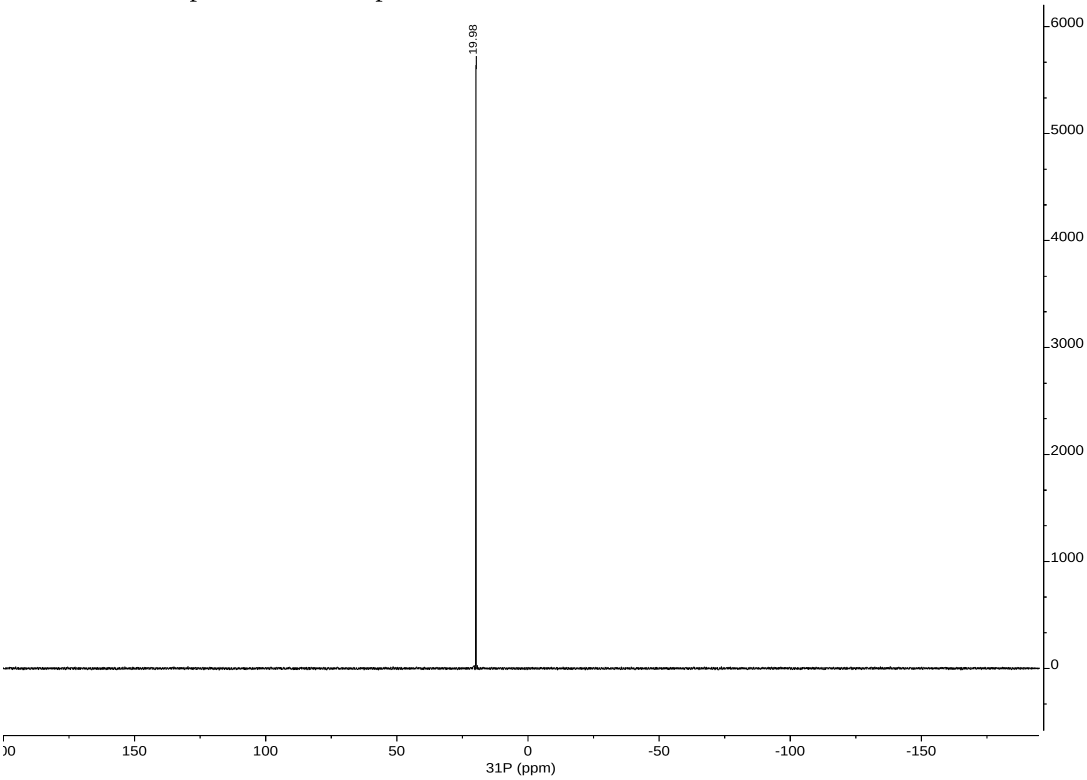

Dibutyl octane-1,8-diyl bis(vinylphosphonate) (S69)

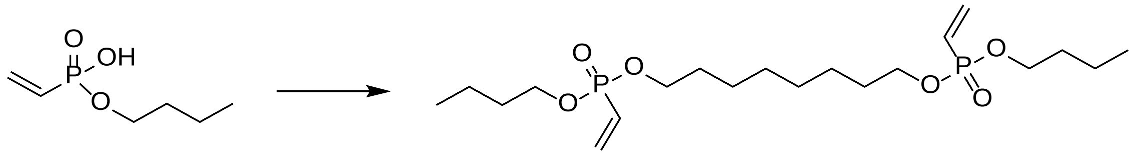

The title compound was prepared according to general method **C** from mono alkyl vinylphosphonate **S20** (0.63 g, 3.84 mmol) and 1,8-dibromooctane (0.35 g, 1.28 mmol) in 71% yield (0.40 g, 0.91 mmol) as a colourless oil.

^{1}H NMR (400 MHz, $CDCl_3$): 6.34–5.98 (m, 6H, C*H*=C*H*$_2$), 4.05–3.97 (m, 8H, $CH_3(CH_2)_3C$*H*$_2$O, OC*H*$_2$$CH_2(CH_2)_4CH_2$C*H*$_2$O), 1.70–1.62 (m, 8H, CH_3CH_2C*H*$_2$$CH_2O$, OCH_2C*H*$_2$$(CH_2)_4C$*H*$_2$$CH_2O$), 1.45–1.36 (m, 12H, CH_3C*H*$_2$$CH_2CH_2O$, $OCH_2CH_2(C$*H*$_2)_4CH_2CH_2O$), 0.93 (t, 6H, *J* = 8.3 Hz, C*H*$_3$$(CH_2)_2CH_2O$).

^{13}C NMR (101 MHz, $CDCl_3$): 135.23 (d, *J* = 2.0 Hz, CH=*C*H$_2$), 125.79 (d, *J* = 184.0 Hz, *C*H=CH$_2$), 65.66, 65.45 (d, *J* = 6.3 Hz, $CH_3CH_2CH_2$*C*H$_2$O, O*C*H$_2$$CH_2(CH_2)_4CH_2$*C*H$_2$O), 30.36, 32.29 (d, *J* = 6.3 Hz, CH_3CH_2*C*H$_2$$CH_2O$, $OCH_2CH_2(CH_2)_4CH_2CH_2O$), 28.87, 25.30, 18.60 ($CH_3$*C*H$_2$$CH_2CH_2O$, OCH_2CH_2(*C*H$_2$)$_4$$CH_2CH_2O$), 13.45 (*C*H$_3$$(CH_2)_2CH_2O$).

^{31}P{^{1}H} NMR (162 MHz, $CDCl_3$): 19.79.

IR ν_{max} (KBr) 3091 (vw), 2963 (s), 2936 (s), 2875 (m), 2860 (m), 1614 (w), 1466 (m), 1459 (m), 1433 (w), 1400 (m), 1380 (w, sh), 1279 (m), 1241 (s), 1063 (s), 1021 9vs, br), 988 (vs), 852 (m), 606 (m).

HR-MS(ESI$^+$): For $C_{20}H_{40}O_6NaP_2$ (M+Na)$^+$ *m*/*z* calculated 461.21923, found 461.21878.

^{1}H NMR spectrum of compound **S69**

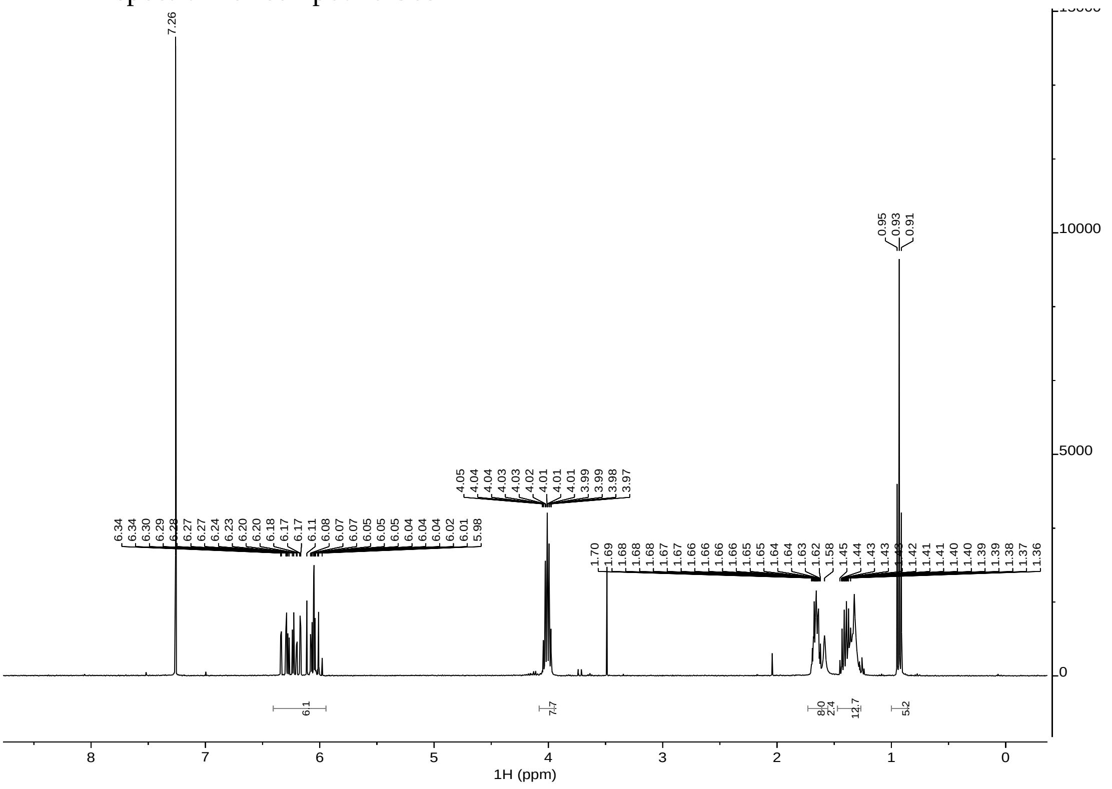

^{13}C NMR spectrum of compound **S69**

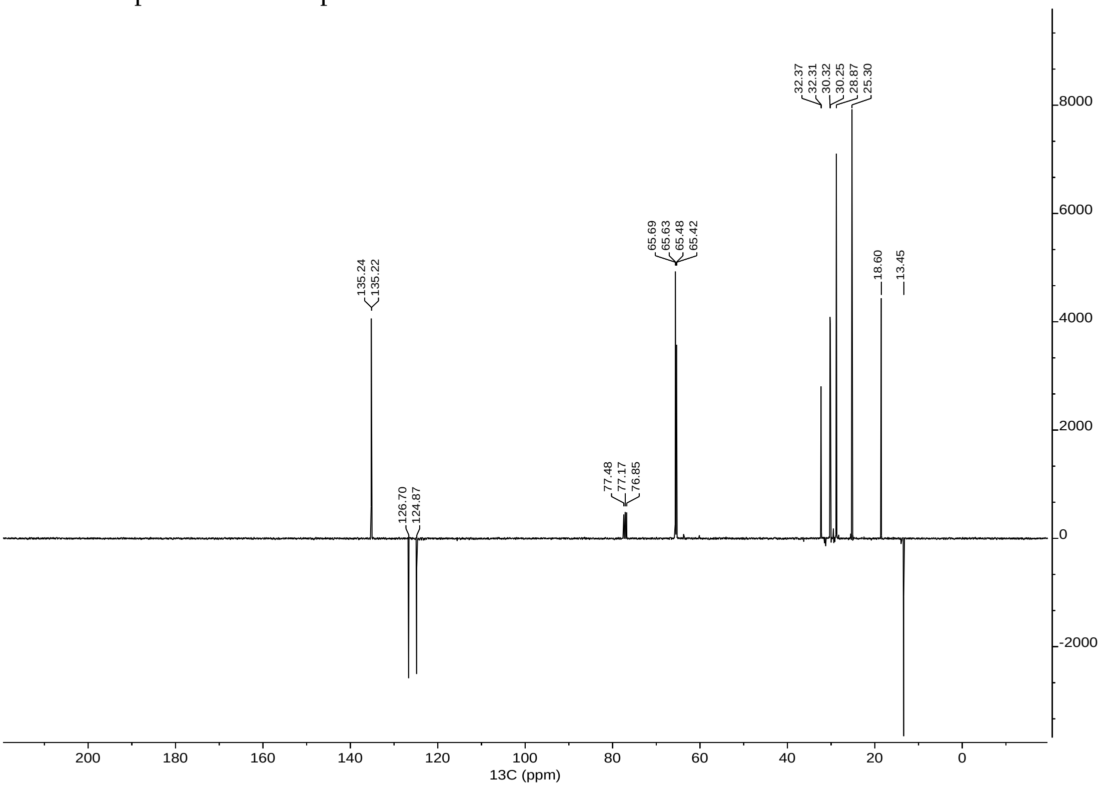

^{31}P{^{1}H} NMR spectrum of compound **S69**

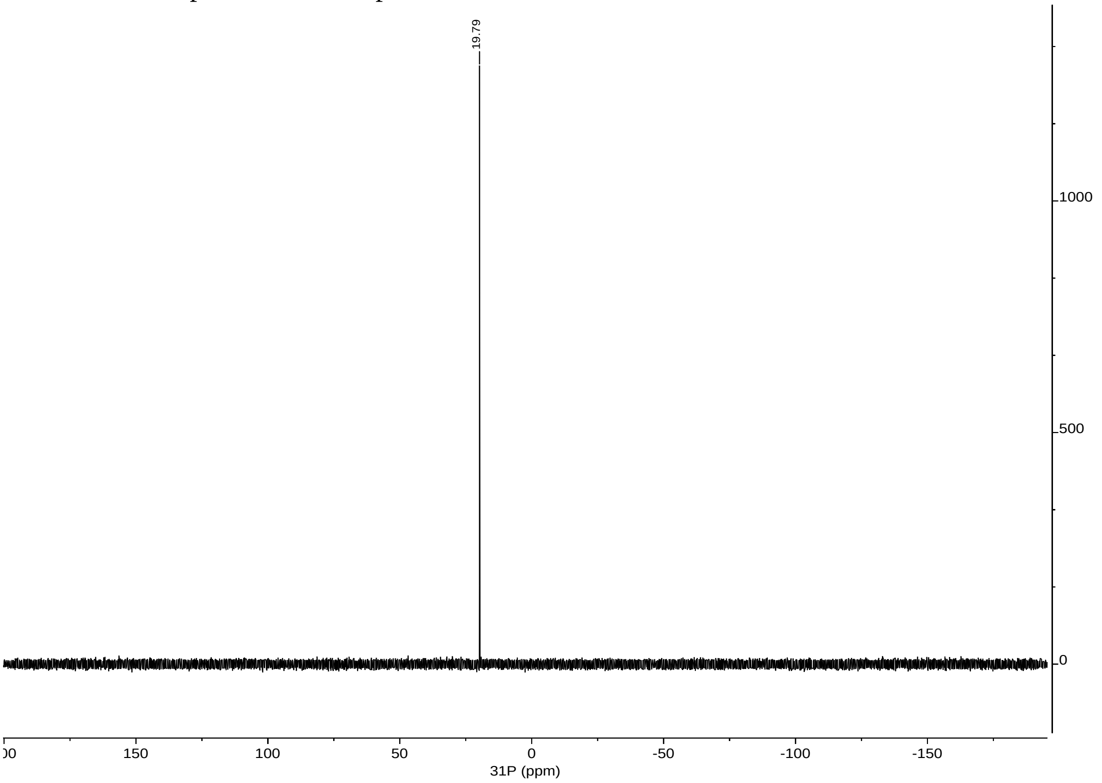

Octane-1,8-diyl dipentyl bis(vinylphosphonate) (S70)

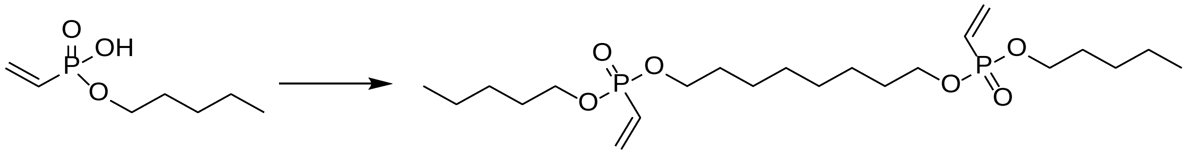

The title compound was prepared according to general method **C** from mono alkyl vinylphosphonate **S22** (1.20 g, 6.73 mmol) and 1,8-dibromooctane (0.61 g, 2.24 mmol) in 71% yield (0.75 g, 1.61 mmol) as a colourless oil.

^{1}H NMR (401 MHz, $CDCl_3$): 6.33–5.97 (m, 6H, C*H*=C*H*$_2$), 4.02–3.95 (m, 8H, $CH_3(CH_2)_3C\mathit{H}_2O$, $OC\mathit{H}_2CH_2(CH_2)_4CH_2C\mathit{H}_2O$), 1.68–1.60 (m, 8H, $CH_3(CH_2)_2C\mathit{H}_2CH_2O$, $OCH_2C\mathit{H}_2(CH_2)_4C\mathit{H}_2CH_2O$), 1.37–1.26 (m, 16H, $CH_3(C\mathit{H}_2)_2CH_2CH_2O$, $OCH_2CH_2(C\mathit{H}_2)_4CH_2CH_2O$), 0.89–0.84 (m, 6H, $C\mathit{H}_3(CH_2)_3CH_2O$).

^{13}C NMR (101 MHz, $CDCl_3$): 135.51 (d, J = 2.1 Hz, CH=*C*H$_2$), 125.97 (d, J = 184.1 Hz, *C*H=CH$_2$), 66.00, 65.90 (d, J = 5.9 Hz, $CH_3(CH_2)_2CH_2\mathit{C}H_2O$, $O\mathit{C}H_2CH_2(CH_2)_4CH_2\mathit{C}H_2O$), 30.52, 30.25 (d, J = 6.5 Hz, $CH_3(CH_2)_2\mathit{C}H_2CH_2O$, $OCH_2CH_2(CH_2)_4\mathit{C}H_2CH_2O$), 29.13, 27.74, 25.54, 22.32 ($CH_3(\mathit{C}H_2)_2CH_2CH_2O$, $OCH_2CH_2(\mathit{C}H_2)_4CH_2CH_2O$), 14.05 ($\mathit{C}H_3(CH_2)_3CH_2O$).

^{31}P{^{1}H} NMR (162 MHz, $CDCl_3$): 20.01.

IR ν_{max} (KBr) 3090 (vw), 2960 (s), 2935 (s), 2873 (m), 2860 (m), 1614 (w), 1467 (m), 1400 (m), 1381 (w), 1279 (w), 1240 (s), 1381 (w), 1075 (m, sh), 1051 (s), 1017 (vs), 992 (vs), 855 (w).

HR-MS(ESI$^+$): For $C_{22}H_{44}O_6NaP_2$ $(M+Na)^+$ *m*/*z* calculated 489.25053, found 489.25015.

^{1}H NMR spectrum of compound **S70**

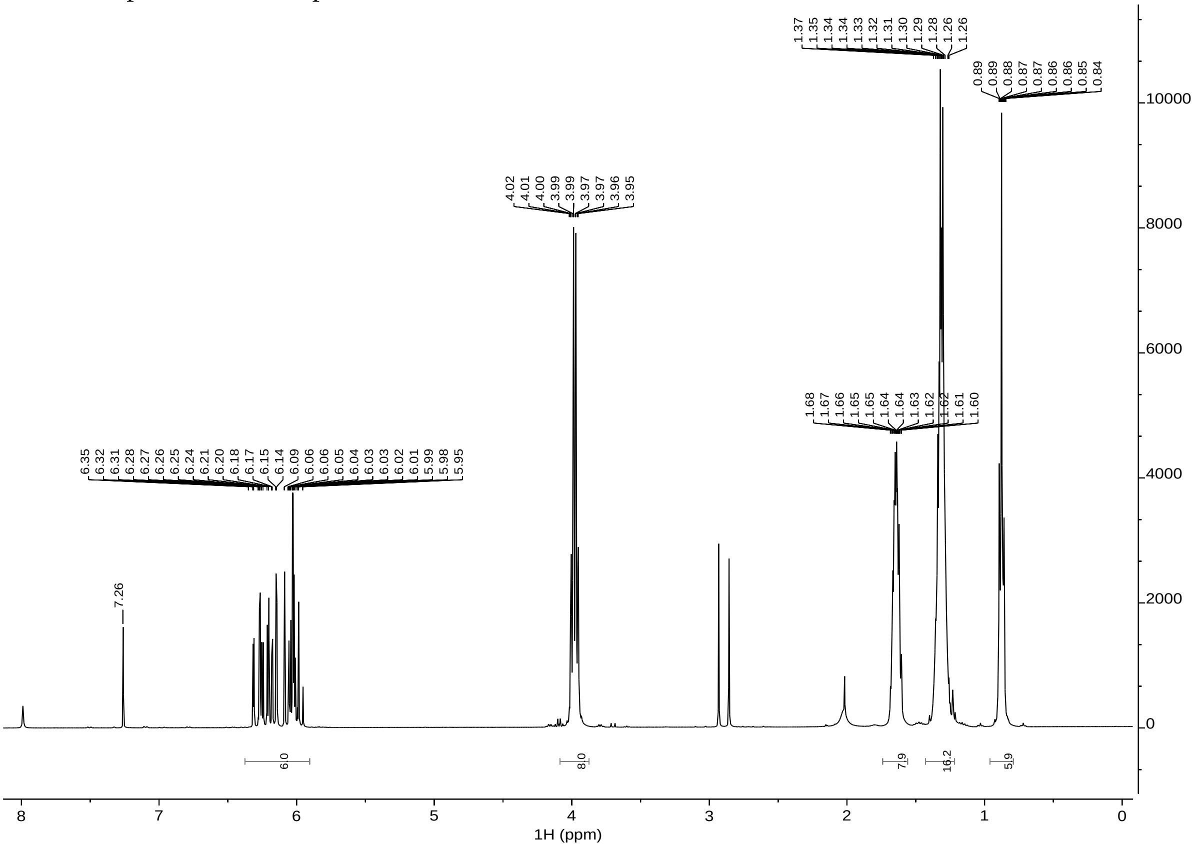

^{13}C NMR spectrum of compound **S70**

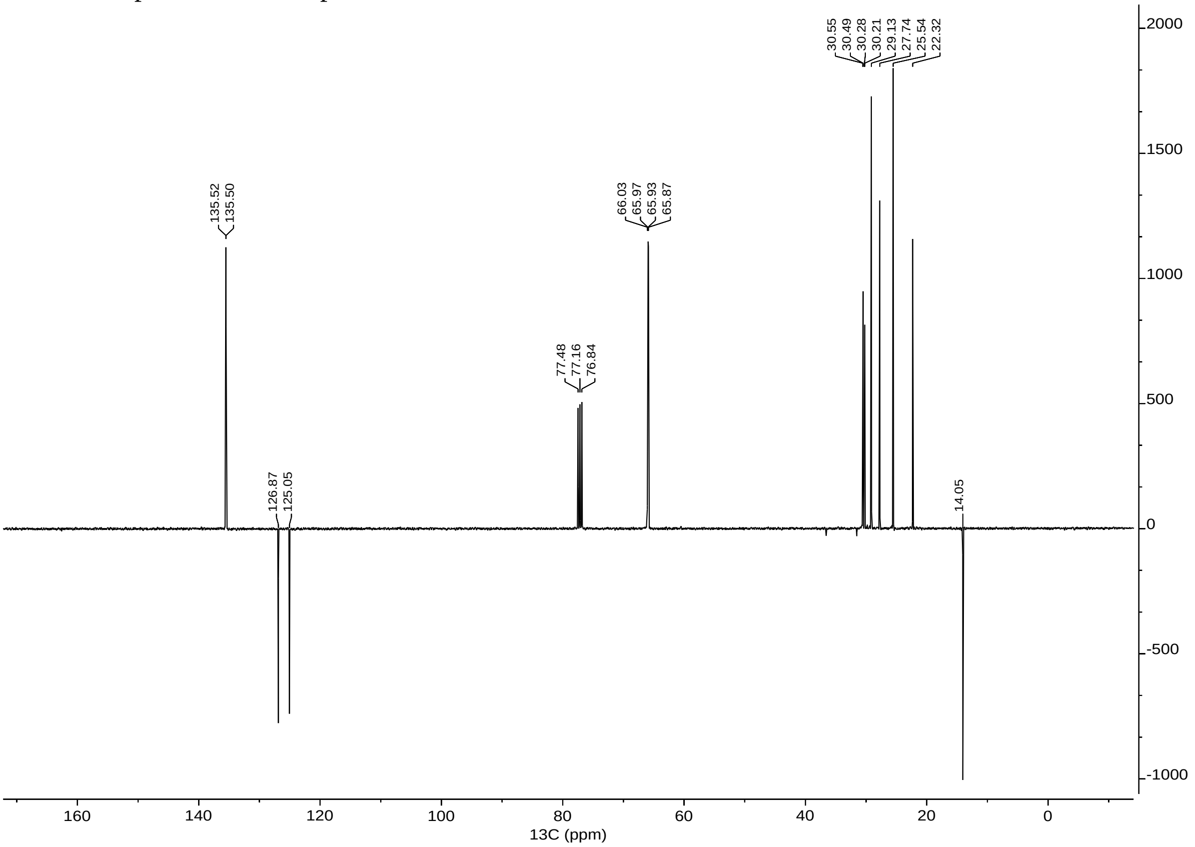

^{31}P{^{1}H} NMR spectrum of compound **S70**

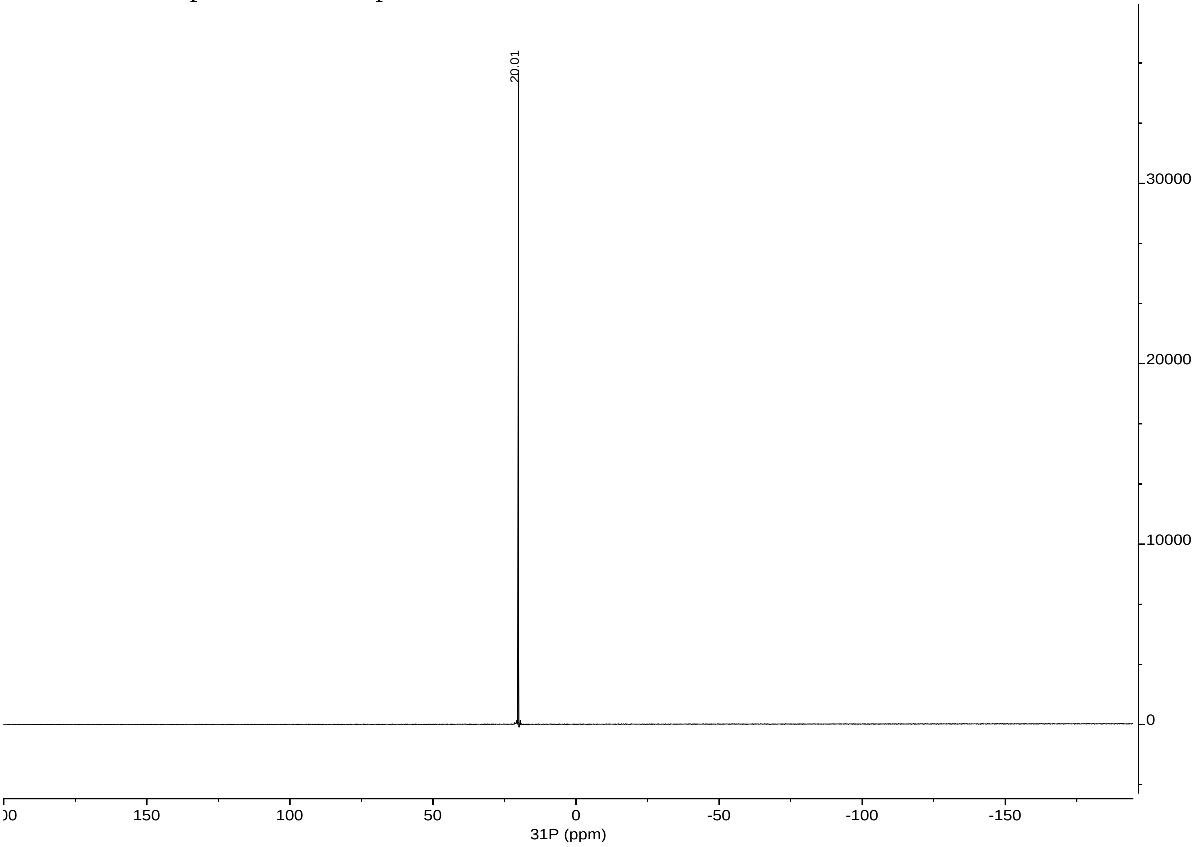

Dihexyl octane-1,8-diyl bis(vinylphosphonate) (S71)

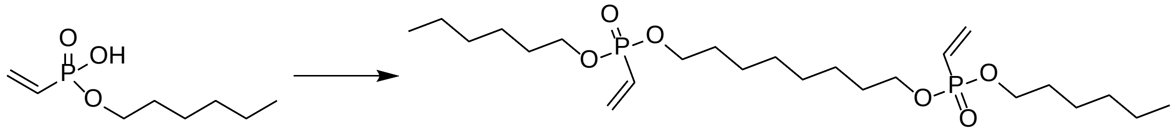

The title compound was prepared according to general method **C** from mono alkyl vinylphosphonate **S23** (1.0 g, 5.20 mmol) and 1,8-dibromooctane (0.47 g, 1.74 mmol) in 74% yield (0.64 g, 1.29 mmol) as a colourless oil.

^{1}H NMR (400 MHz, $CDCl_3$): 6.34–5.97 (m, 6H, $C\mathit{H}{=}C\mathit{H}_2$), 4.11–3.97 (m, 8H, $CH_3(CH_2)_4C\mathit{H}_2O$, $OC\mathit{H}_2CH_2(CH_2)_4CH_2C\mathit{H}_2O$), 1.70–1.62 (m, 8H, $CH_3(CH_2)_3C\mathit{H}_2CH_2O$, $OCH_2C\mathit{H}_2(CH_2)_4C\mathit{H}_2CH_2O$), 1.40–1.24 (m, 20H, $CH_3(C\mathit{H}_2)_3CH_2CH_2O$, $OCH_2CH_2(C\mathit{H}_2)_4CH_2CH_2O$), 0.90–0.83 (m, 6H, $C\mathit{H}_3(CH_2)_4CH_2O$).

^{13}C NMR (101 MHz, $CDCl_3$): 135.51 (d, J = 1.9 Hz, $CH{=}\mathit{C}H_2$), 126.05 (d, J = 184.1 Hz, $\mathit{C}H{=}CH_2$), 66.08, 65.95 (d, J = 6.1 Hz, $CH_3(CH_2)_3CH_2\mathit{C}H_2O$, $O\mathit{C}H_2CH_2(CH_2)_4CH_2\mathit{C}H_2O$), 30.43 (d, J = 6.2 Hz, $CH_3(CH_2)_3\mathit{C}H_2CH_2O$, $OCH_2\mathit{C}H_2(CH_2)_4\mathit{C}H_2CH_2O$), 31.47, 29.18, 25.59, 25.32, 22.66 ($CH_3(\mathit{C}H_2)_3CH_2CH_2O$, $OCH_2CH_2(\mathit{C}H_2)_4CH_2CH_2O$), 14.12 ($\mathit{C}H_3(CH_2)_4CH_2O$).

^{31}P{^{1}H} NMR (162 MHz, $CDCl_3$): 19.77.

IR ν_{max} (KBr) 3091 (vw), 2959 (s), 2934 (s), 2872 (m), 2860 (w), 1614 (w), 1468 (w), 1456 (w, sh), 1434 (w), 1400 (m), 1279 (m), 1241 (s), 1059 (s), 1040 (s, sh), 1009 (vs), 998 (vs), 606 (w).

HR-MS(ESI^{+}): For $C_{24}H_{49}O_6P_2$ $(M+H)^{+}$ m/z calculated 495.29989, found 495.29906.

^{1}H NMR spectrum of compound **S71**

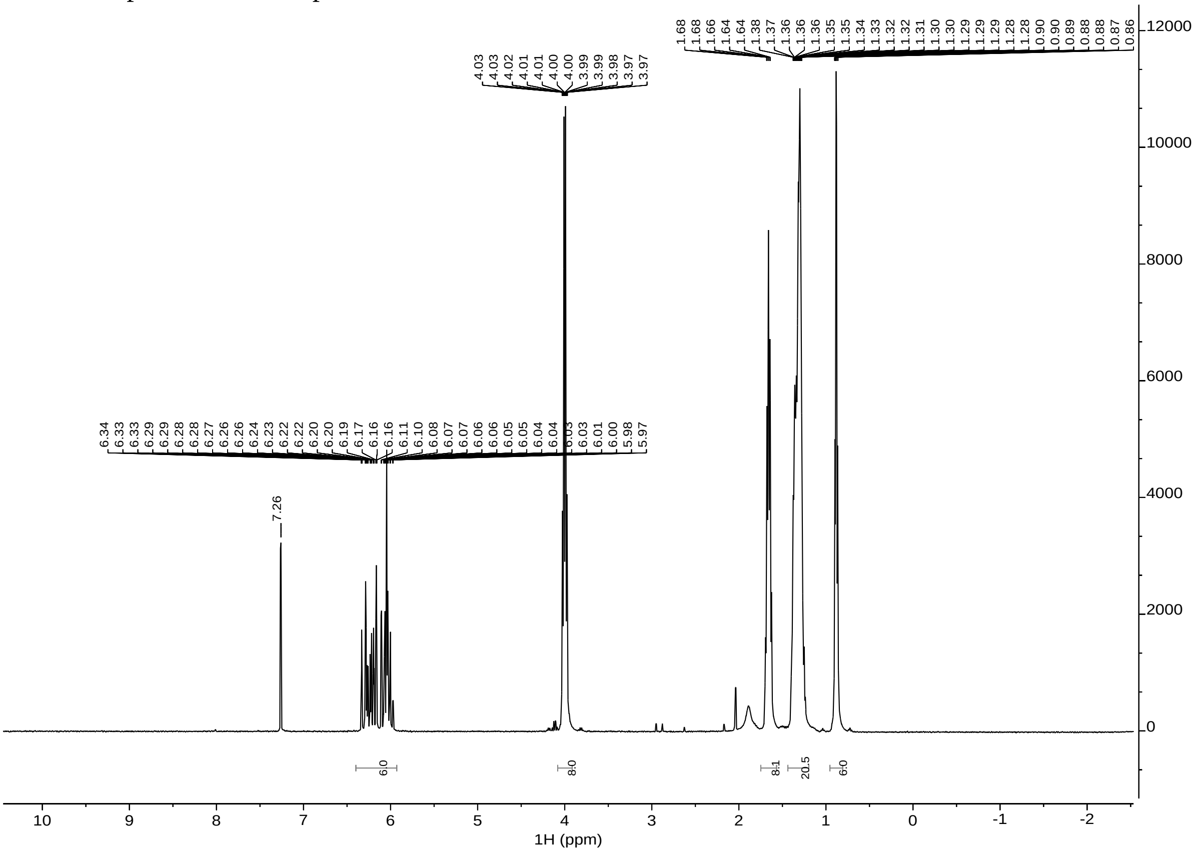

^{13}C NMR spectrum of compound **S71**

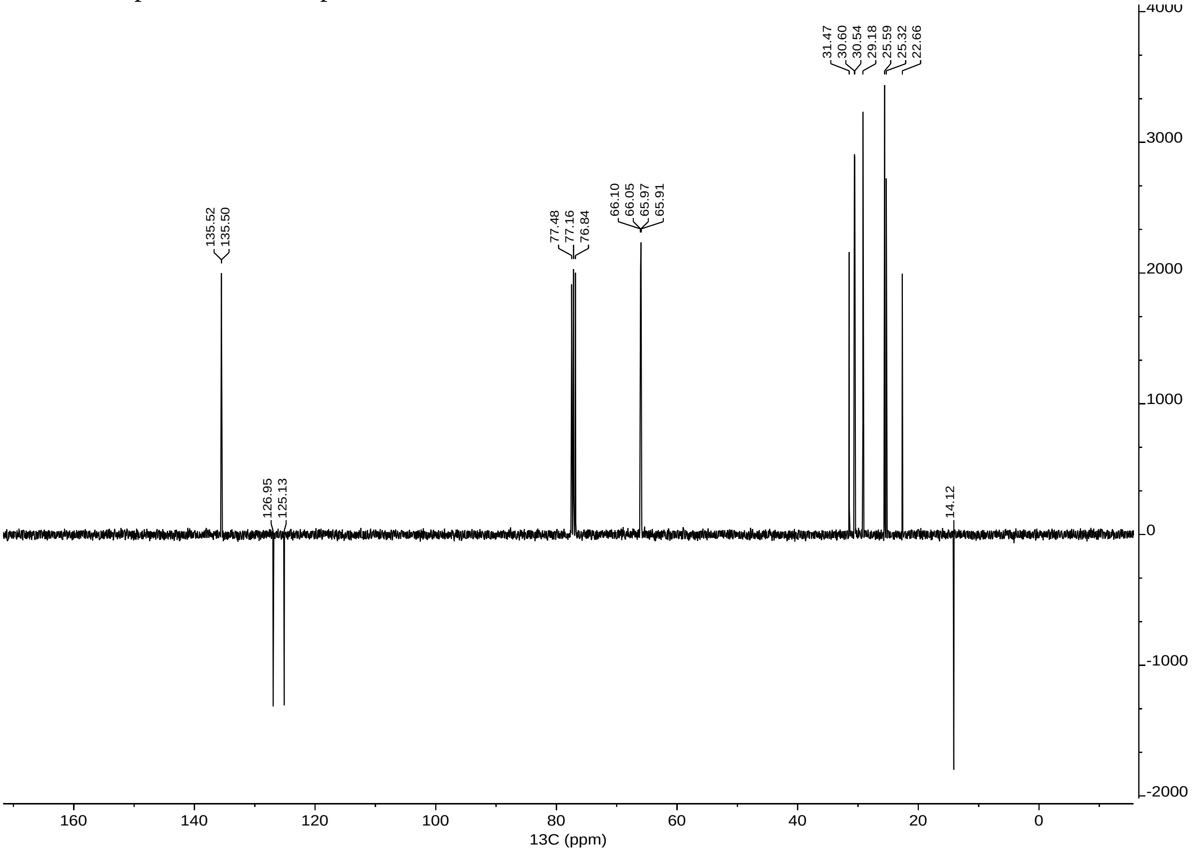

^{31}P{^{1}H} NMR spectrum of compound **S71**

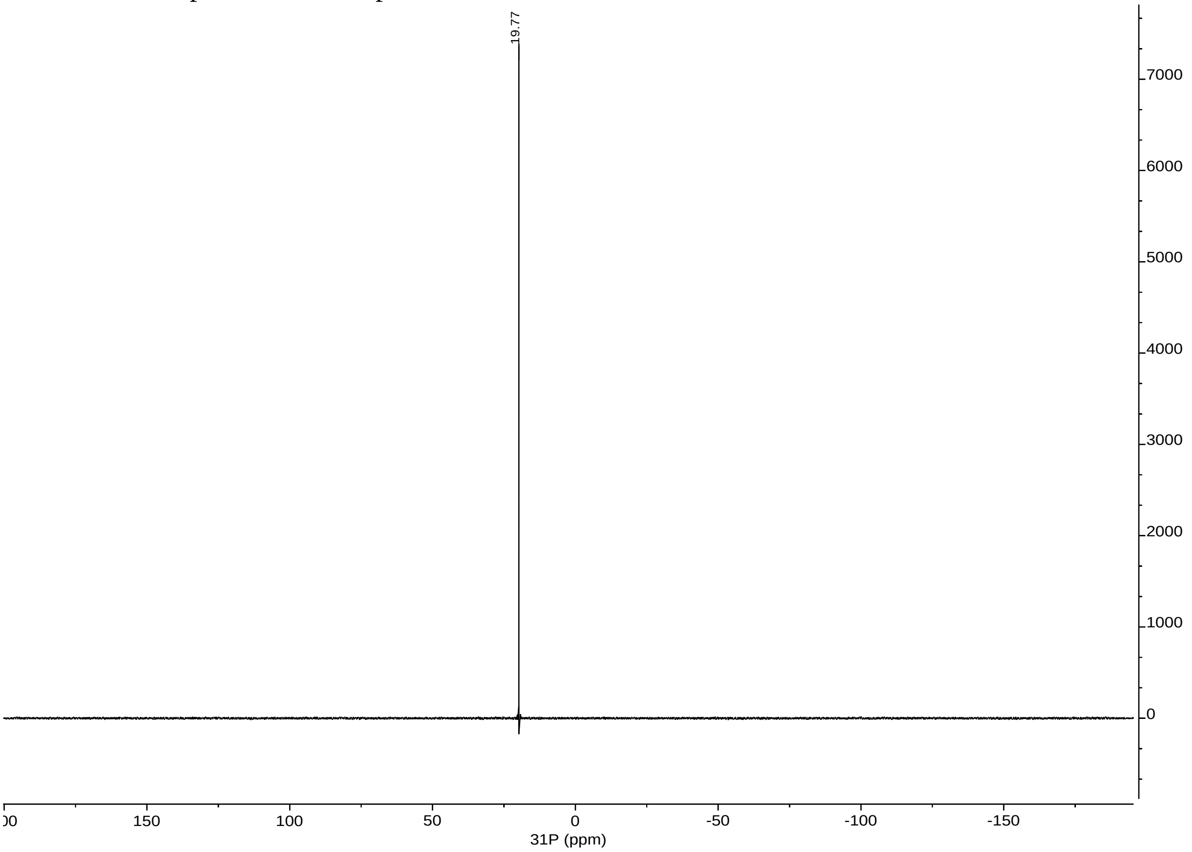

Bis((*Z*)-hept-3-en-1-yl) octane-1,8-diyl bis(vinylphosphonate) (S72)

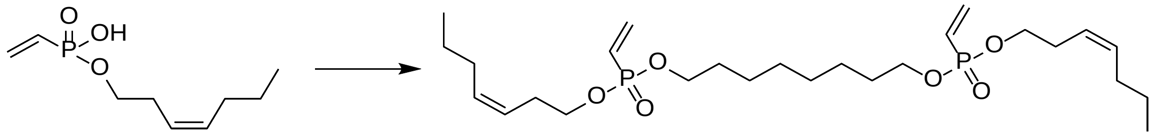

The title compound was prepared according to general method **C** from mono alkyl vinylphosphonate **S26** (1.0 g, 4.89 mmol) and 1,8-dibromooctane (0.45 g, 1.63 mmol) in 83% yield (0.70 g, 1.35 mmol) as a colourless oil.

^{1}H NMR (401 MHz, $CDCl_3$): 6.34–5.97 (m, 6H, C*H*=C*H*$_2$), 5.54–5.47 (m, 2H, $CH_3(CH_2)_2C\mathit{H}CHCH_2CH_2O$), 5.39–5.32 (m, 2H, $CH_3(CH_2)_2CHC\mathit{H}CH_2CH_2O$), 4.03–3.96 (m, 8H, $OC\mathit{H}_2CH_2(CH_2)_2CH_2C\mathit{H}_2O$, $CH_3CH_2CH_2CHCHCH_2C\mathit{H}_2O$), 2.45–2.39 (m, 4H, $CH_3CH_2CH_2CHCHC\mathit{H}_2CH_2O$), 2.04–1.98 (m, 4H, $CH_3CH_2C\mathit{H}_2CHCHCH_2CH_2O$), 1.69–1.62 (m, 4H, $OCH_2C\mathit{H}_2(CH_2)_2C\mathit{H}_2CH_2O$), 1.41–1.24 (m, 12H, $CH_3C\mathit{H}_2CH_2CHCHCH_2CH_2O$, $OCH_2CH_2(C\mathit{H}_2)_4CH_2CH_2O$), 0.89 (t, 6H, *J* = 7.4 Hz, $C\mathit{H}_3(CH_2)_2CHCHCH_2CH_2O$).

^{13}C NMR (101 MHz, $CDCl_3$): 135.63 (d, *J* = 2.1 Hz, CH=*C*H$_2$), 133.16 ($CH_3(CH_2)_2\mathit{C}HCHCH_2CH_2O$), 125.97 (d, *J* = 183.1 Hz, *C*H=CH$_2$), 124.06 ($CH_3(CH_2)_2CH\mathit{C}HCH_2CH_2O$), 65.98 (d, *J* = 5.8 Hz, $CH_3CH_2CH_2CHCHCH_2\mathit{C}H_2O$), 65.34 (d, *J* = 5.9 Hz, $O\mathit{C}H_2CH_2(CH_2)_2CH_2\mathit{C}H_2O$), 30.57 (d, *J* = 6.1 Hz, $OCH_2\mathit{C}H_2(CH_2)_2\mathit{C}H_2CH_2O$), 29.53 ($CH_3CH_2\mathit{C}H_2CHCHCH_2CH_2O$), 28.84 (d, *J* = 6.4 Hz, $CH_3CH_2CH_2CHCH\mathit{C}H_2CH_2O$), 28.87, 25.58 ($OCH_2CH_2(\mathit{C}H_2)_4CH_2CH_2O$), 22.81 ($CH_3\mathit{C}H_2CH_2CHCHCH_2CH_2O$), 13.89 ($\mathit{C}H_3(CH_2)_2CHCHCH_2CH_2O$).

^{31}P{^{1}H} NMR (162 MHz, $CDCl_3$): 20.04.

IR ν_{max} (KBr) 3090 (w), 2961 (s), 2932 (s), 2860 (m), 1655 (w), 1614 (w), 1466 (m), 1458 (m), 1379 (m), 1241 (vs), 1070–950 (vs, br), 987 (s, sh), 857 (m).

HR-MS(ESI$^+$): For $C_{26}H_{49}O_6P_2$ $(M+H)^+$ *m*/*z* calculated 519.29989, found 519.29975.

^{1}H NMR spectrum of compound **S72**

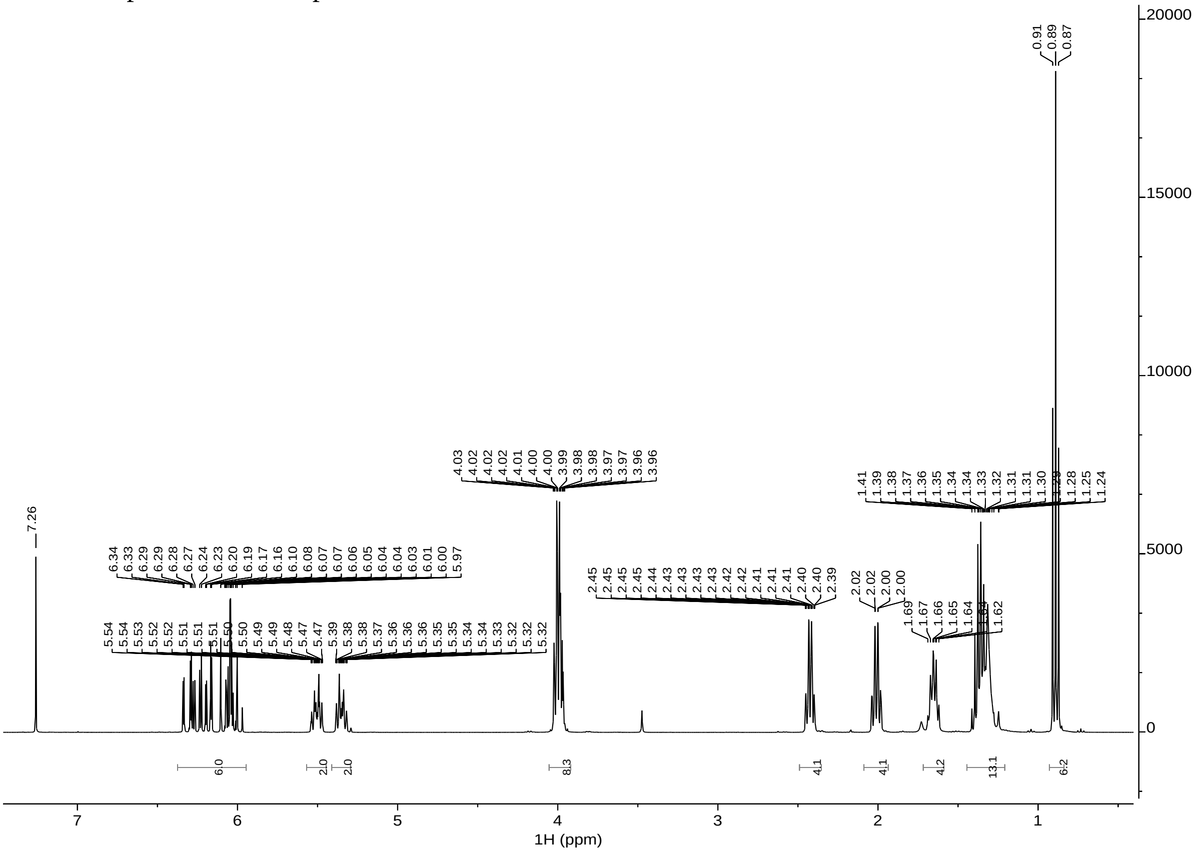

^{13}C NMR spectrum of compound **S72**

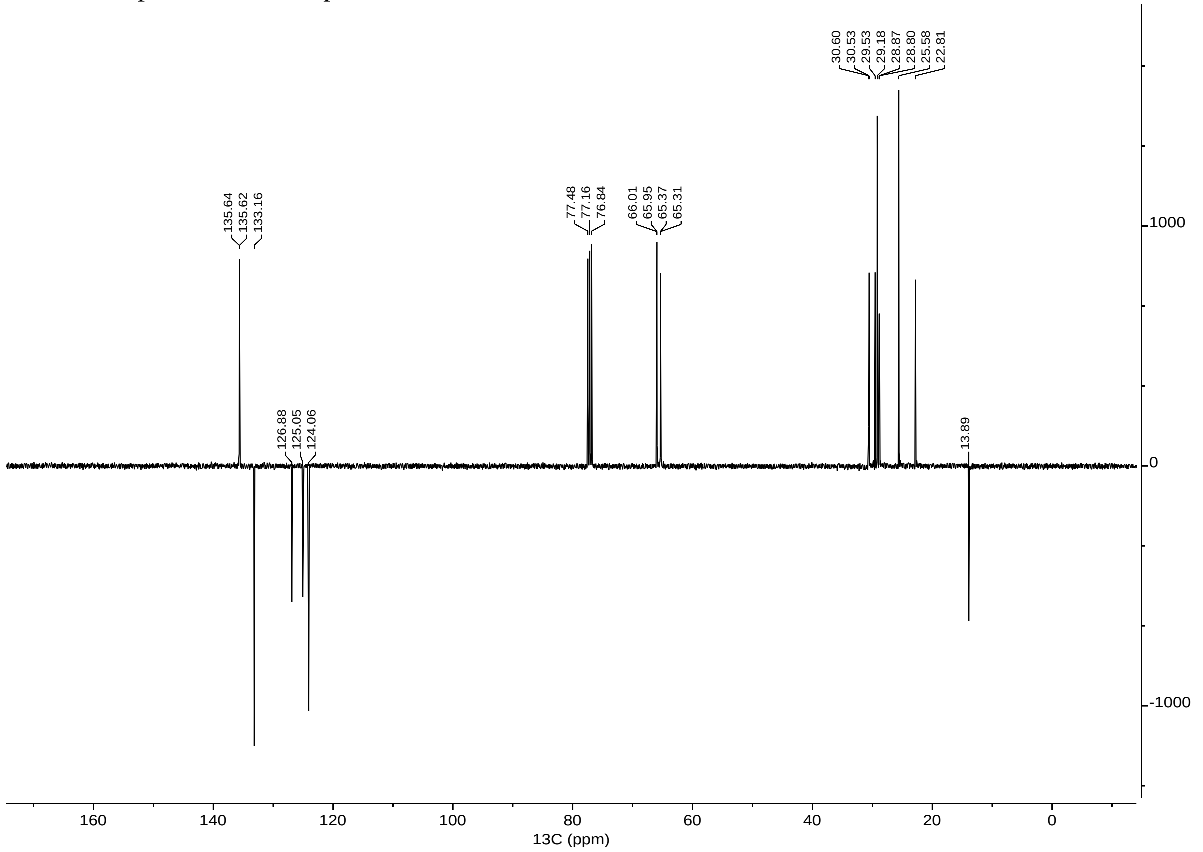

$^{31}P\{^{1}H\}$ NMR spectrum of compound **S72**

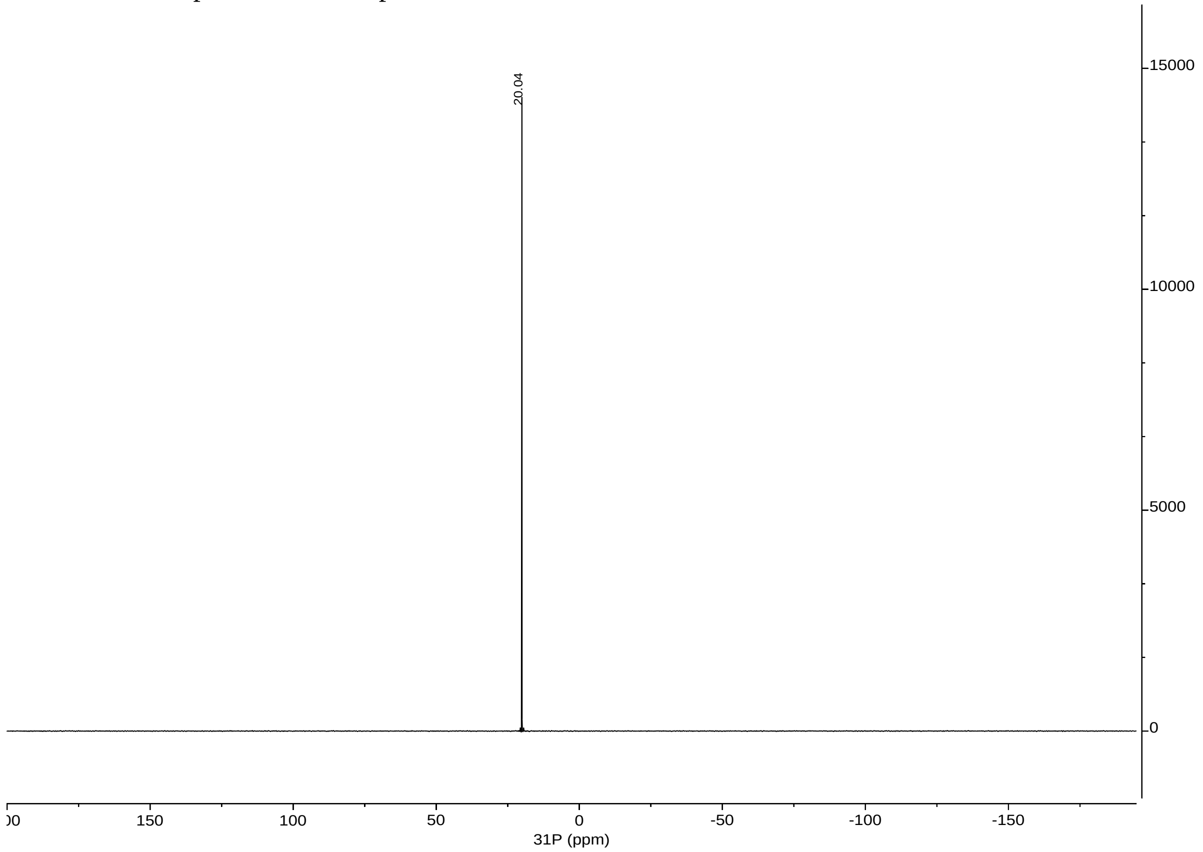

Octane-1,8-diyl dioctyl bis(vinylphosphonate) (S73)

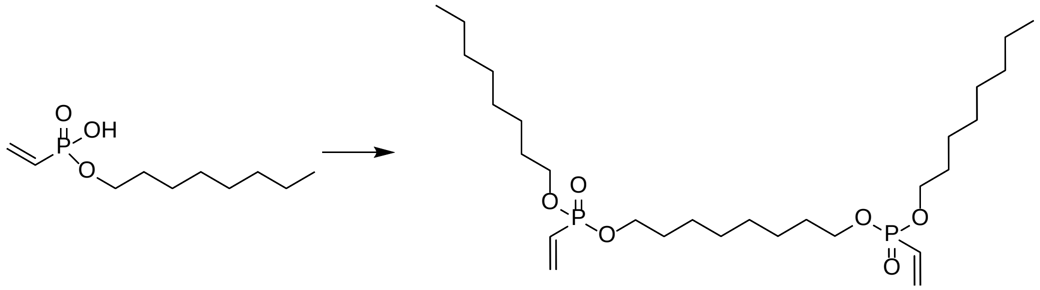

The title compound was prepared according to general method **C** from mono alkyl vinylphosphonate **S28** (1.0 g, 4.56 mmol) and 1,8-dibromooctane (0.42 g, 1.52 mmol) in 80% yield (0.67 g, 1.22 mmol) as a colourless oil.

^{1}H NMR (401 MHz, $CDCl_3$): 6.34–5.97 (m, 6H, $C\mathit{H}{=}C\mathit{H}_2$), 4.03–3.97 (m, 8H, $CH_3(CH_2)_5CH_2C\mathit{H}_2O$, $OC\mathit{H}_2CH_2(CH_2)_4CH_2C\mathit{H}_2O$), 1.70–1.63 (m, 8H, $CH_3(CH_2)_5C\mathit{H}_2CH_2O$, $OCH_2C\mathit{H}_2(CH_2)_4C\mathit{H}_2CH_2O$), 1.38–1.25 (m, 28H, $CH_3(C\mathit{H}_2)_5CH_2CH_2O$, $OCH_2CH_2(C\mathit{H}_2)_4CH_2CH_2O$), 0.89–0.86 (m, 6H, $C\mathit{H}_3(CH_2)_6CH_2O$).

^{13}C NMR (101 MHz, $CDCl_3$): 135.53 (d, J = 1.8 Hz, $CH{=}\mathit{C}H_2$), 126.05 (d, J = 184.9 Hz, $\mathit{C}H{=}CH_2$), 66.04, 65.94 (d, J = 6.1 Hz, $CH_3(CH_2)_5CH_2\mathit{C}H_2O$, $O\mathit{C}H_2CH_2(CH_2)_4CH_2\mathit{C}H_2O$), 30.62, 30.59 (d, J = 6.7 Hz, $CH_3(CH_2)_5\mathit{C}H_2CH_2O$, $OCH_2\mathit{C}H_2(CH_2)_4\mathit{C}H_2CH_2O$), 31.91, 29.32, 29.26, 29.20, 25.66, 25.60, 22.78 ($CH_3(\mathit{C}H_2)_5CH_2CH_2O$, $OCH_2CH_2(\mathit{C}H_2)_4CH_2CH_2O$), 14.23 ($\mathit{C}H_3(CH_2)_6CH_2O$).

^{31}P{^{1}H} NMR (162 MHz, $CDCl_3$): 20.03.

IR ν_{max} (KBr) 3093 (vw), 2957 (s), 2931 (s), 2876 (m), 2860 (m), 1468 (w), 1458 (w), 1446 (w), 1436 (w), 1400 (w), 1242 (s), 1070 (m), 1049 (s), 1015 (vs), 987 (vs), 856 (w).

HR-MS(ESI^{+}): For $C_{28}H_{57}O_6P_2$ $(M+H)^{+}$ m/z calculated 551.36249, found 551.36214.

^{1}H NMR spectrum of compound **S73**

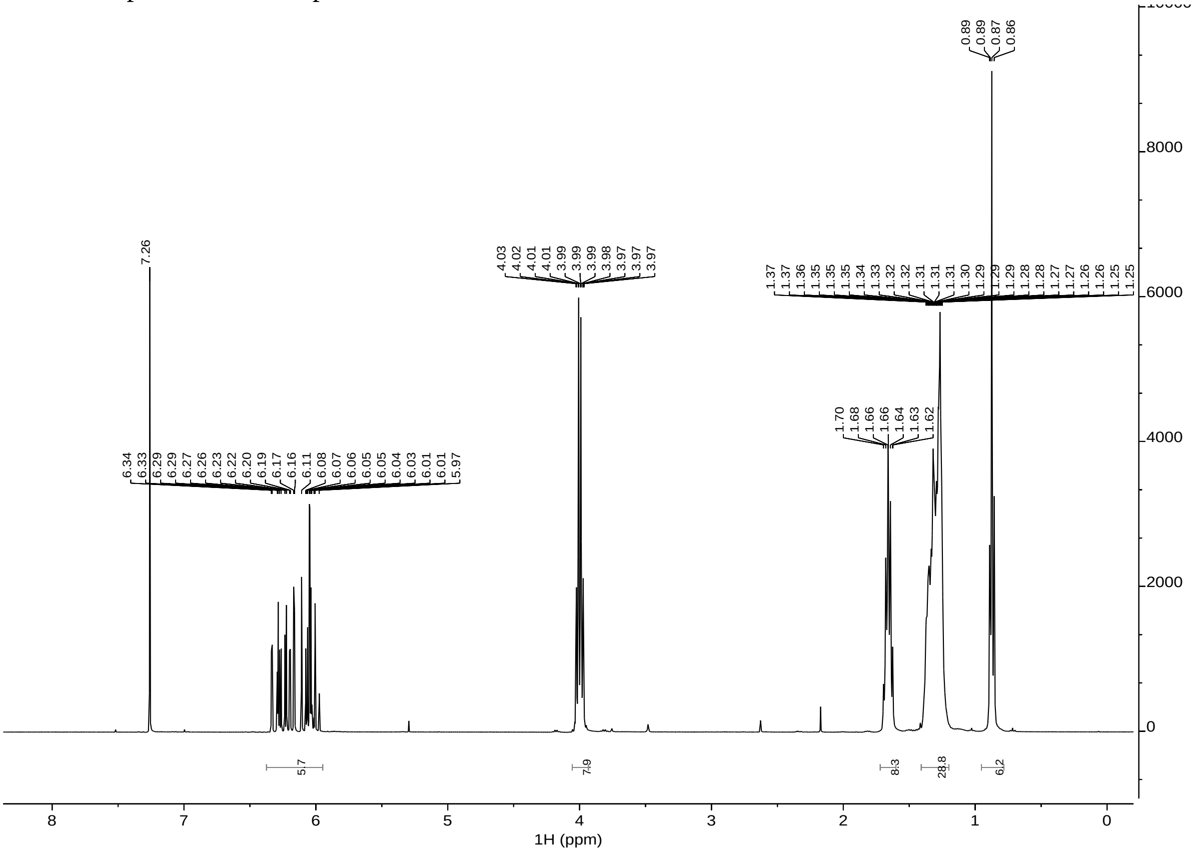

^{13}C NMR spectrum of compound **S73**

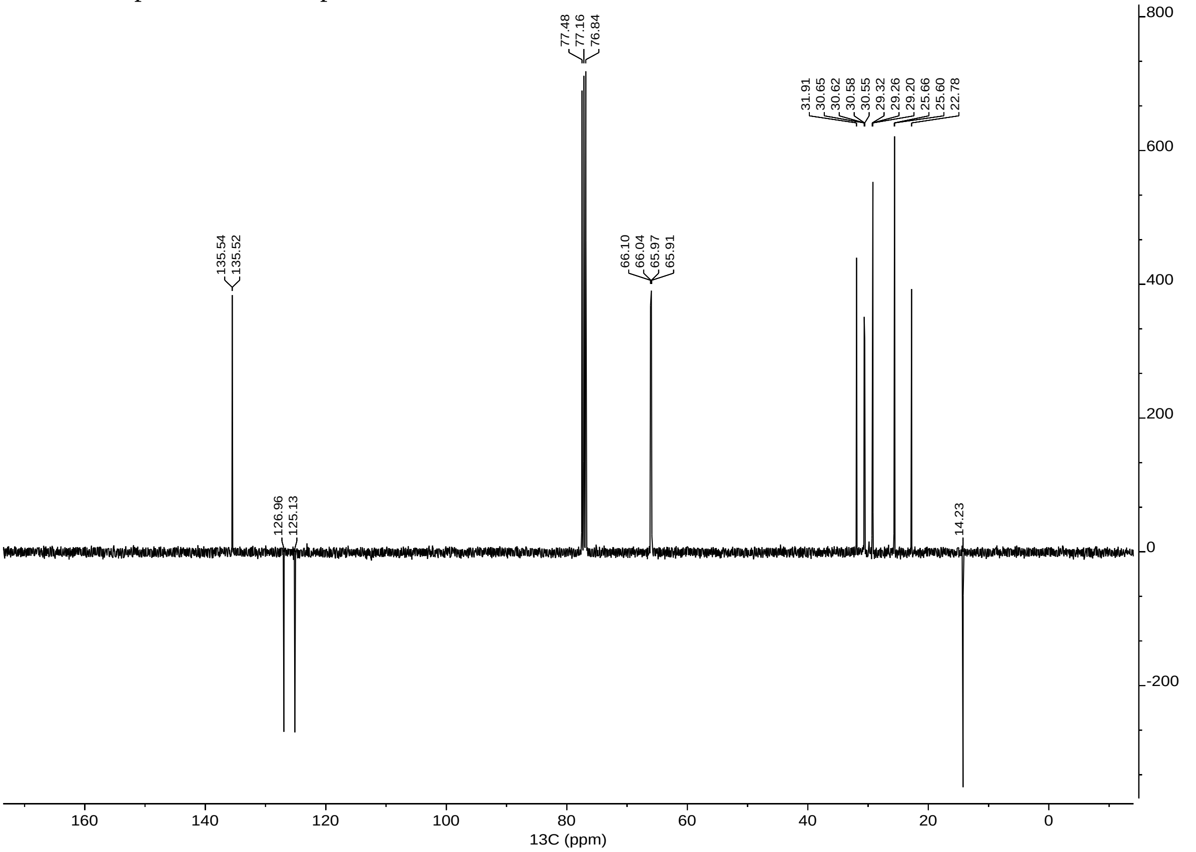

^{31}P{^{1}H} NMR spectrum of compound **S73**

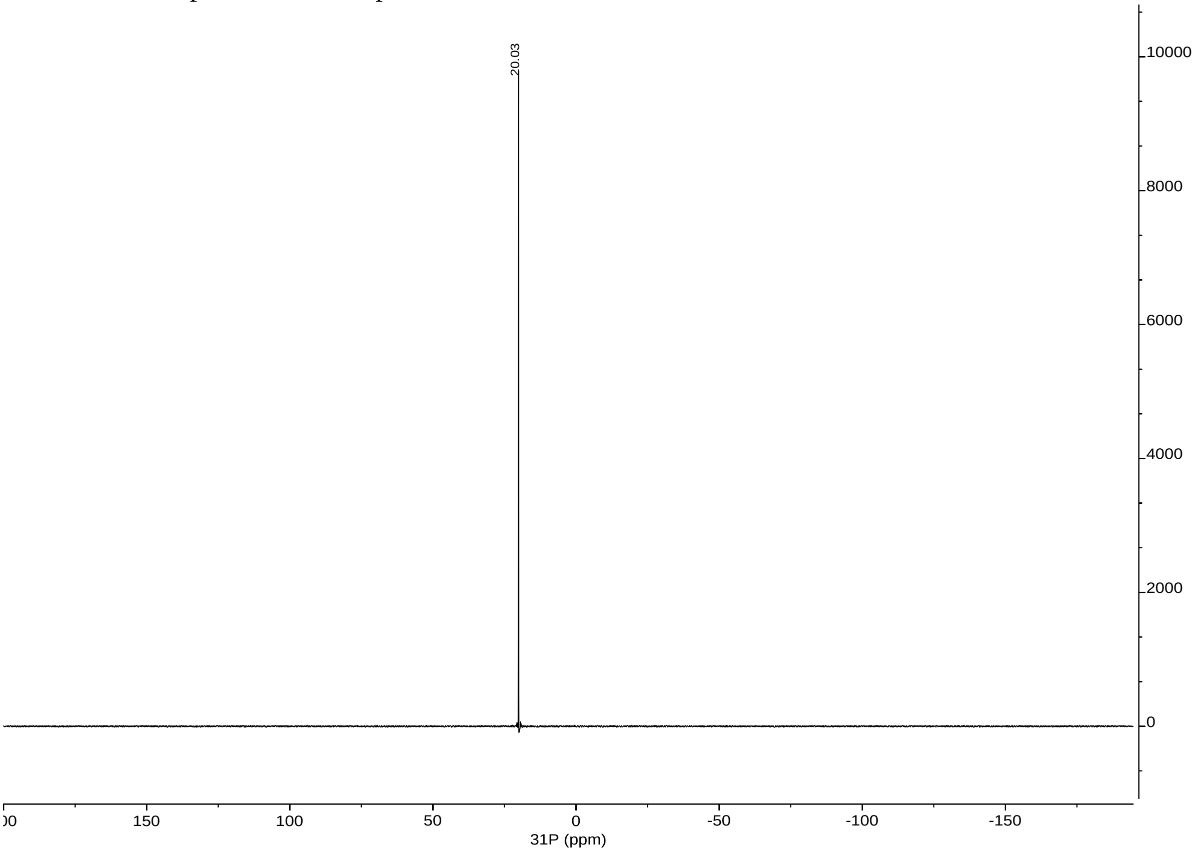

Octane-1,8-diyl diphenethyl bis(vinylphosphonate) (S74)

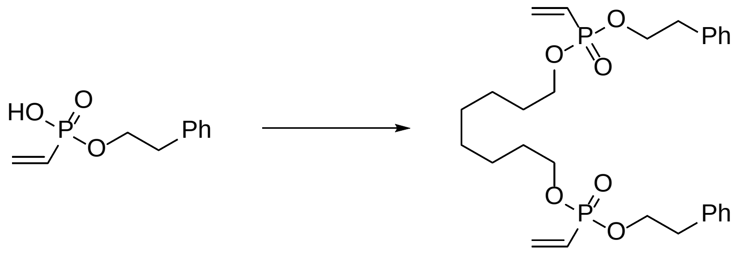

The title compound was prepared according to general method **C** from mono alkyl vinylphosphonate **S31** (1.44 g, 6.79 mmol) and 1,8-dibromooctane (95%, 438 μL, 2.26 mmol) in 13% yield (158 mg, 0.296 mmol) as a colourless oil.

^{1}H NMR (401 MHz, $CDCl_3$) δ 7.43–7.20 (m, 10H, Ph*H*), 6.34–5.89 (m, 6H, C*H*=C*H*$_2$), 4.34–4.17 (m, 4H, OC*H*$_2$(CH_2)$_3$), 4.04–3.89 (m, 4H, OC*H*$_2$$CH_2$Ph), 3.01 (t, *J* = 7.0 Hz, 4H, C*H*$_2$Ph), 1.83–1.57 (m, 4H, OCH_2C*H*$_2$$CH_2$), 1.43–1.26 (m, 8H, O($CH_2$)$_2$(C*H*$_2$)$_2$).

^{13}C NMR (101 MHz, $CDCl_3$) δ 137.44 (CH_2*C*$_{quat}$), 135.71 (d, *J* = 2.1 Hz, =CH_2), 129.18, 128.64, 126.81 (*C*$_{Ph}$), 125.74 (d, *J* = 184.2 Hz, P*C*H), 66.32 (d, *J* = 5.8 Hz), 65.97 (d, *J* = 5.9 Hz, O*C*H_2), 37.13 (d, *J* = 6.5 Hz), 30.52 (d, *J* = 6.5 Hz, OCH_2*C*H_2), 29.16, 25.55 (O(CH_2)$_2$(*C*H_2)$_2$).

^{31}P{^{1}H} NMR (162 MHz, $CDCl_3$) δ 20.06.

IR ν_{max}($CHCl_3$) 3089 (w), 3066 (w), 2936 (m), 2859 (m), 1614 (w), 1604 (w), 1497 (m), 1468 (m), 1454 (m), 1400 (m), 1278 (m), 1241 (s), 1070–1000 (vs), 1030 (sh), 987 (sh), 946 (w), 815 (w, sh), 700 (m), 571 (w), 495 (w).

HR-MS(ESI$^+$): For $C_{28}H_{40}O_6NaP_2$ (M+Na)$^+$ *m*/*z* calculated 557.21923, found 557.21907. For $C_{28}H_{41}O_6P_2$ (M+H)$^+$ *m*/*z* calculated 535.23729, found 535.23730.

^{1}H NMR spectrum of compound **S74**

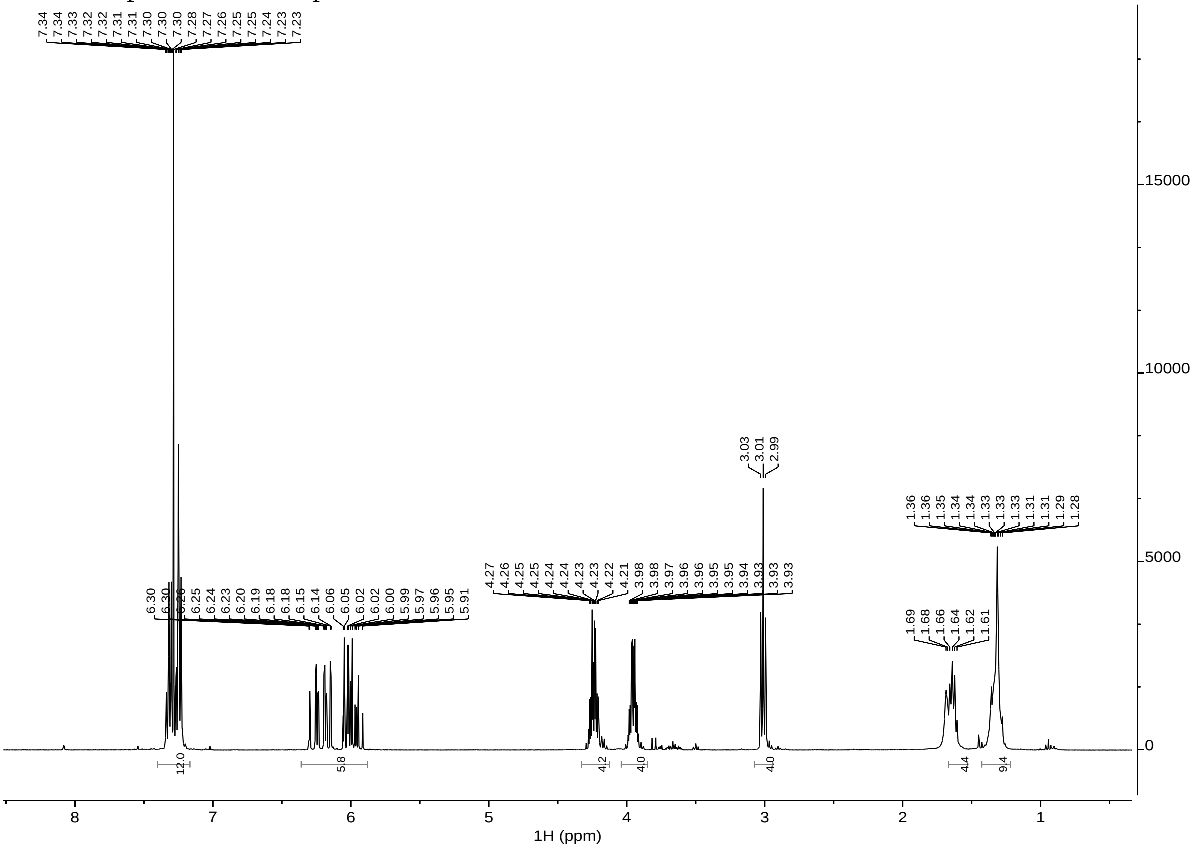

^{13}C NMR spectrum of compound **S74**

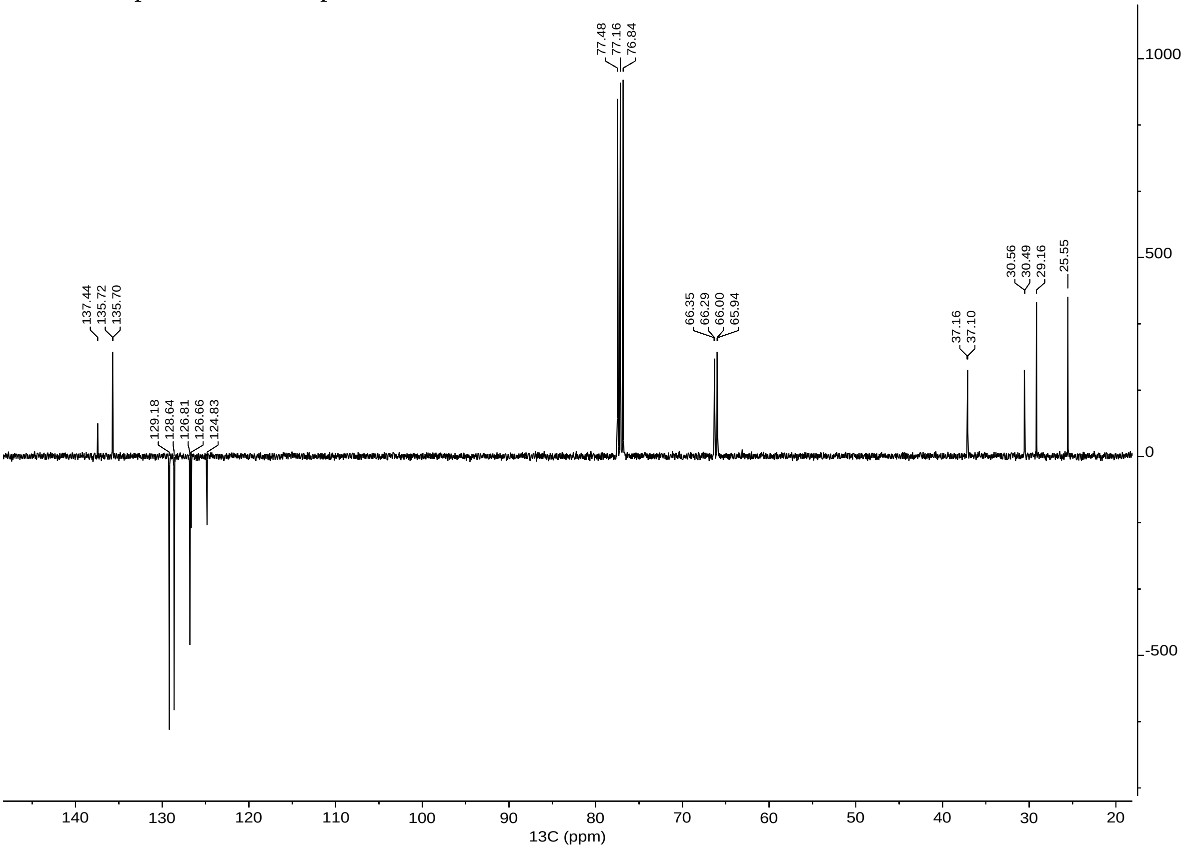

^{31}P{^{1}H} NMR spectrum of compound **S74**

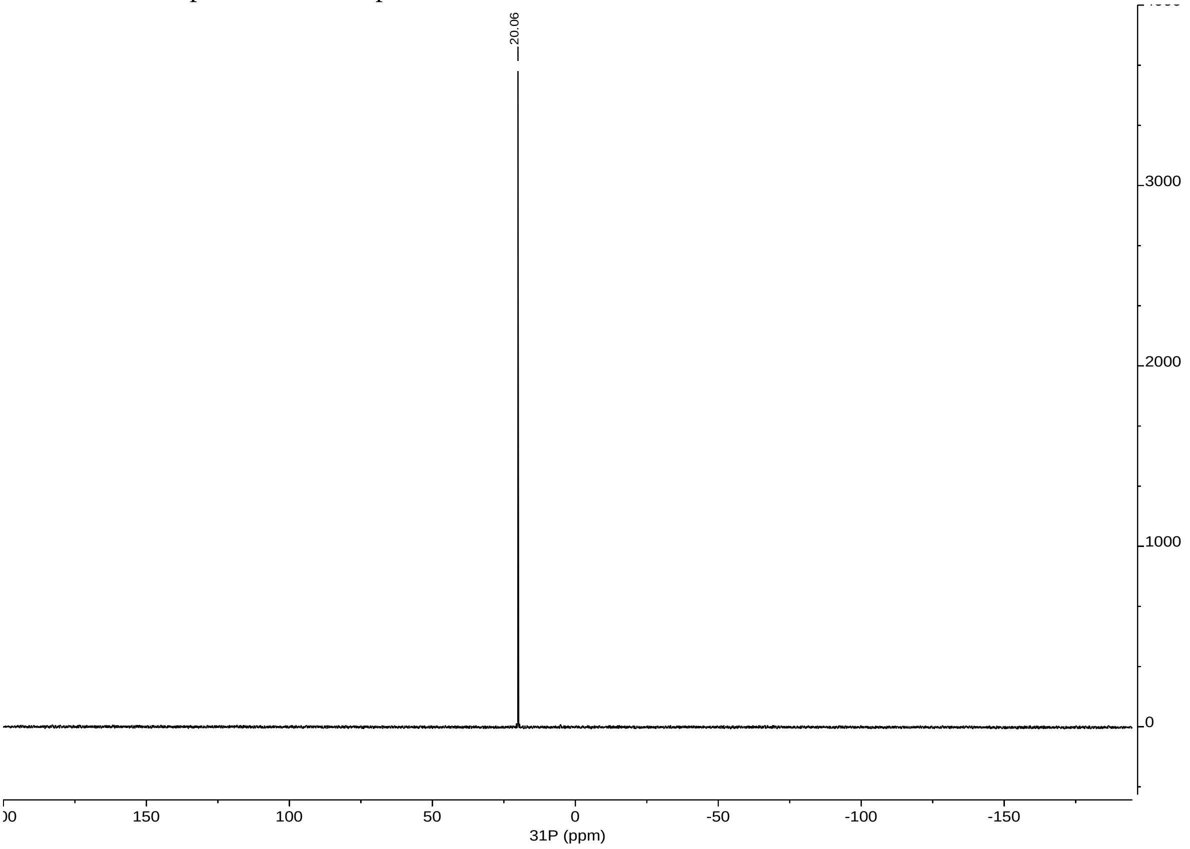

Decane-1,10-diyl diisobutyl bis(vinylphosphonate) (S75)

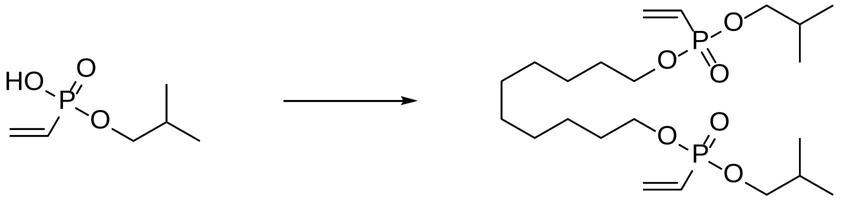

The title compound was prepared according to general method **C** from mono alkyl vinylphosphonate **S19** (500 mg, 3.05 mmol) and 1,10-dibromodecane (97%, 314 µL, 1.01 mmol) in 88% yield (416 mg, 0.89 mmol) as a colourless oil.

^{1}H NMR (401 MHz, CDCl$_3$) δ 6.37–5.94 (m, 6H, C*H*=C*H*$_2$), 4.01 (q, *J* = 6.8 Hz, 4H, OC*H*$_2$CH$_2$), 3.77 (td, *J* = 6.6, 1.6 Hz, 4H, OC*H*$_2$CH), 1.94 (dp, *J* = 13.4, 6.7 Hz, 2H, OCH$_2$C*H*), 1.72–1.61 (m, 4H, OCH$_2$C*H*$_2$), 1.42–1.21 (m, 12H, O(CH$_2$)$_2$(C*H*$_2$)$_3$), 0.93 (d, *J* = 6.7 Hz, 12H, C*H*$_3$).

^{13}C NMR (101 MHz, CDCl$_3$) δ 135.56 (d, *J* = 2.0 Hz, =*C*H$_2$), 125.94 (d, *J* = 184.4 Hz, P*C*H), 71.92 (d, *J* = 6.0 Hz, O*C*H$_2$CH), 66.04 (d, *J* = 5.9 Hz, O*C*H$_2$CH$_2$), 30.60 (d, *J* = 6.3 Hz, OCH$_2$*C*H$_2$), 29.54, 29.30 (d, *J* = 6.4 Hz, OCH$_2$*C*H), 29.25, 25.63 (O(CH$_2$)$_2$(*C*H$_2$)$_3$), 18.86 (d, *J* = 1.9 Hz, *C*H$_3$).

^{31}P{^{1}H} NMR (162 MHz, CDCl$_3$) δ 19.92.

IR ν_{max}(CHCl$_3$) 3091 (vw), 2964 (s), 2932 (vs), 2875 (m), 2857 (s), 1614 (w), 1471 (s), 1399 (s), 1370 (m), 1278 (s), 1240 (vs), 1167 (w), ~1047 (s, sh), 1013 (vs, br), ~990 (vs, sh), ~965 (s, sh), 862 (m).

HR-MS(ESI$^+$): For $C_{22}H_{44}O_6NaP_2$ (M+Na)$^+$ *m/z* calculated 489.25053, found 489.25012.

^{1}H NMR spectrum of compound **S75**

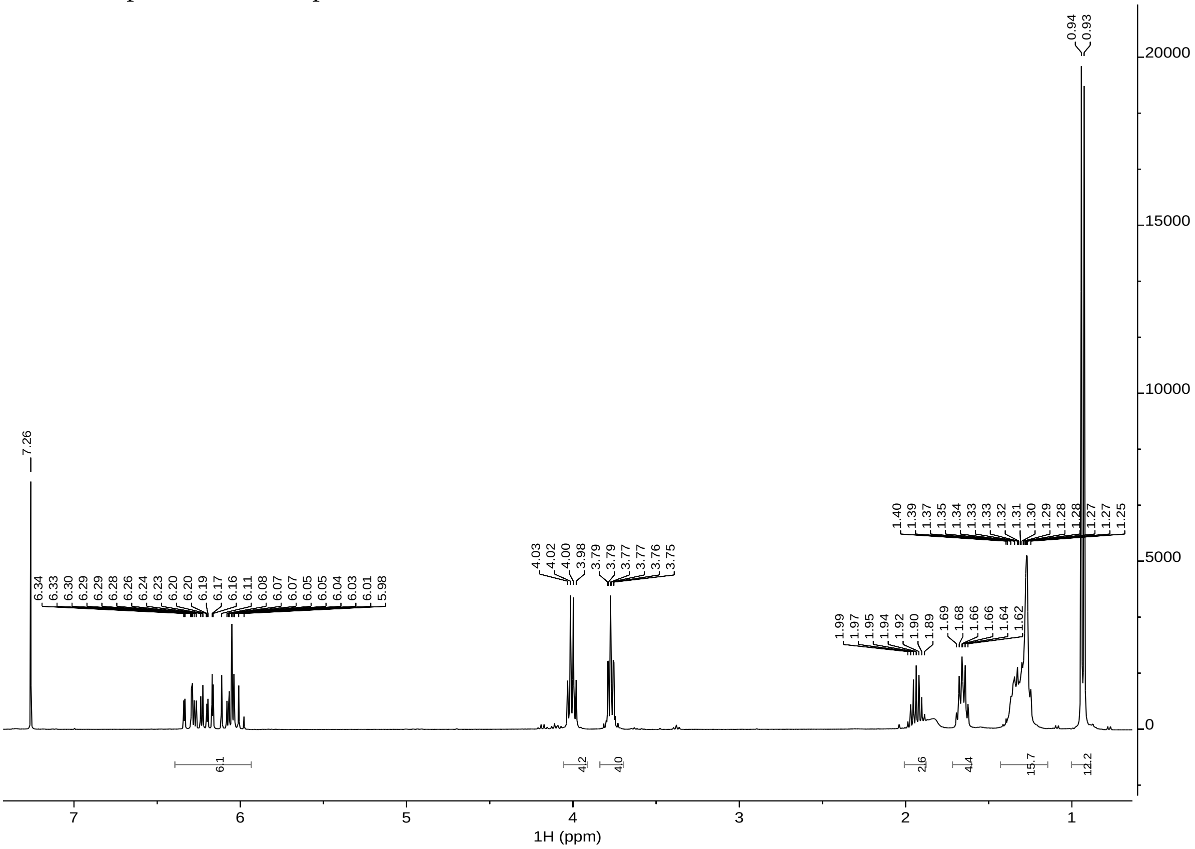

^{13}C NMR spectrum of compound **S75**

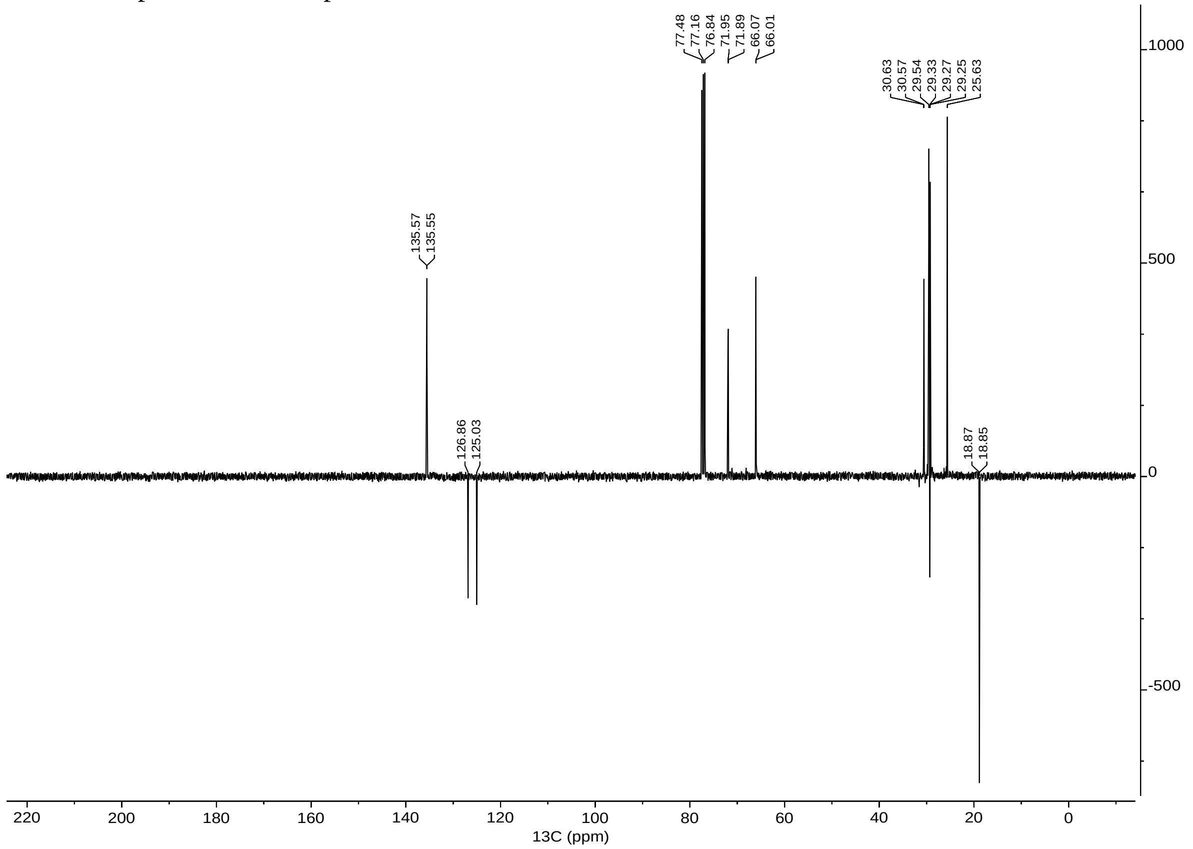

$^{31}P\{^{1}H\}$ NMR spectrum of compound **S75**

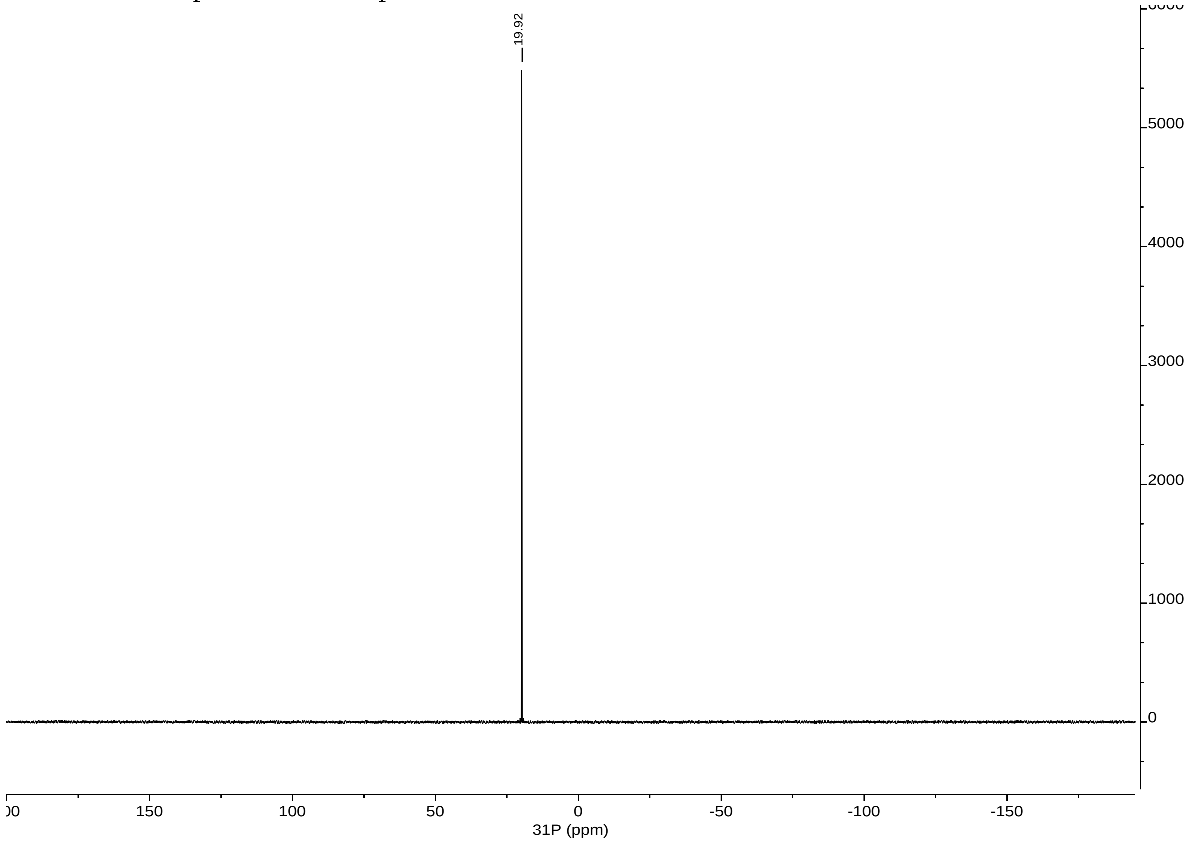

Dibutyl decane-1,10-diyl bis(vinylphosphonate) (S76)

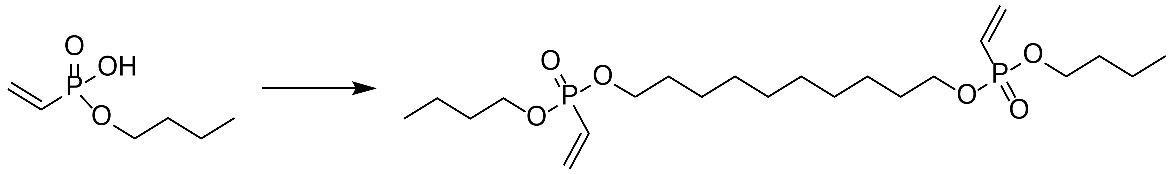

The title compound was prepared according to general method **C** from mono alkyl vinylphosphonate **S20** (1.50 g, 9.14 mmol) and 1,10-dibromodecane (0.91 g, 3.05 mmol) in 62% yield (0.88g, 1.89 mmol) as a colourless oil.

^{1}H NMR (400 MHz, CDCl$_3$): 6.33–5.97 (m, 6H, C*H*=C*H*$_2$), 4.04–3.97 (m, 8H, CH$_3$(CH$_2$)$_3$C*H*$_2$O, OC*H*$_2$CH$_2$(CH$_2$)$_6$CH$_2$C*H*$_2$O), 1.69–1.61 (m, 8H, CH$_3$CH$_2$C*H*$_2$CH$_2$O, OCH$_2$C*H*$_2$(CH$_2$)$_6$C*H*$_2$CH$_2$O), 1.44–1.24 (m, 16H, CH$_3$C*H*$_2$CH$_2$CH$_2$O, OCH$_2$CH$_2$(C*H*$_2$)$_6$CH$_2$CH$_2$O), 0.92 (t, 6H, *J* = 7.4 Hz, C*H*$_3$(CH$_2$)$_2$CH$_2$O).

^{13}C NMR (101 MHz, CDCl$_3$): 135.49 (d, *J* = 1.8 Hz, CH=*C*H$_2$), 126.04 (d, *J* = 184.5 Hz, *C*H=CH$_2$), 66.01, 65.73 (d, *J* = 5.9 Hz, CH$_3$CH$_2$CH$_2$*C*H$_2$O, O*C*H$_2$CH$_2$(CH$_2$)$_6$CH$_2$*C*H$_2$O), 32.61, 30.59 (d, *J* = 6.4 Hz, CH$_3$CH$_2$*C*H$_2$CH$_2$O, OCH$_2$*C*H$_2$(CH$_2$)$_6$*C*H$_2$CH$_2$O), 29.53, 29.25, 25.63, 18.87 (CH$_3$*C*H$_2$CH$_2$CH$_2$O, OCH$_2$CH$_2$(*C*H$_2$)$_6$CH$_2$CH$_2$O), 13.73 (*C*H$_3$(CH$_2$)$_2$CH$_2$O).

^{31}P{^{1}H} NMR (162 MHz, CDCl$_3$): 19.77.

IR ν_{max} (KBr) 3090 (vw), 3001 (vw), 2962 (s), 2935 (vs), 2850 (s), 1612 (w), 1463 (m), 1395 (m), 1271 (m, sh), 1250 (vs), 1068 (vs), 1025 (vs), 985 (vs), 980 (m, sh), 726 (m).

HR-MS(ESI$^+$): For $C_{22}H_{44}O_6NaP_2$ (M+Na)$^+$ *m/z* calculated 489.25053, found 489.24997.

^{1}H NMR spectrum of compound **S76**

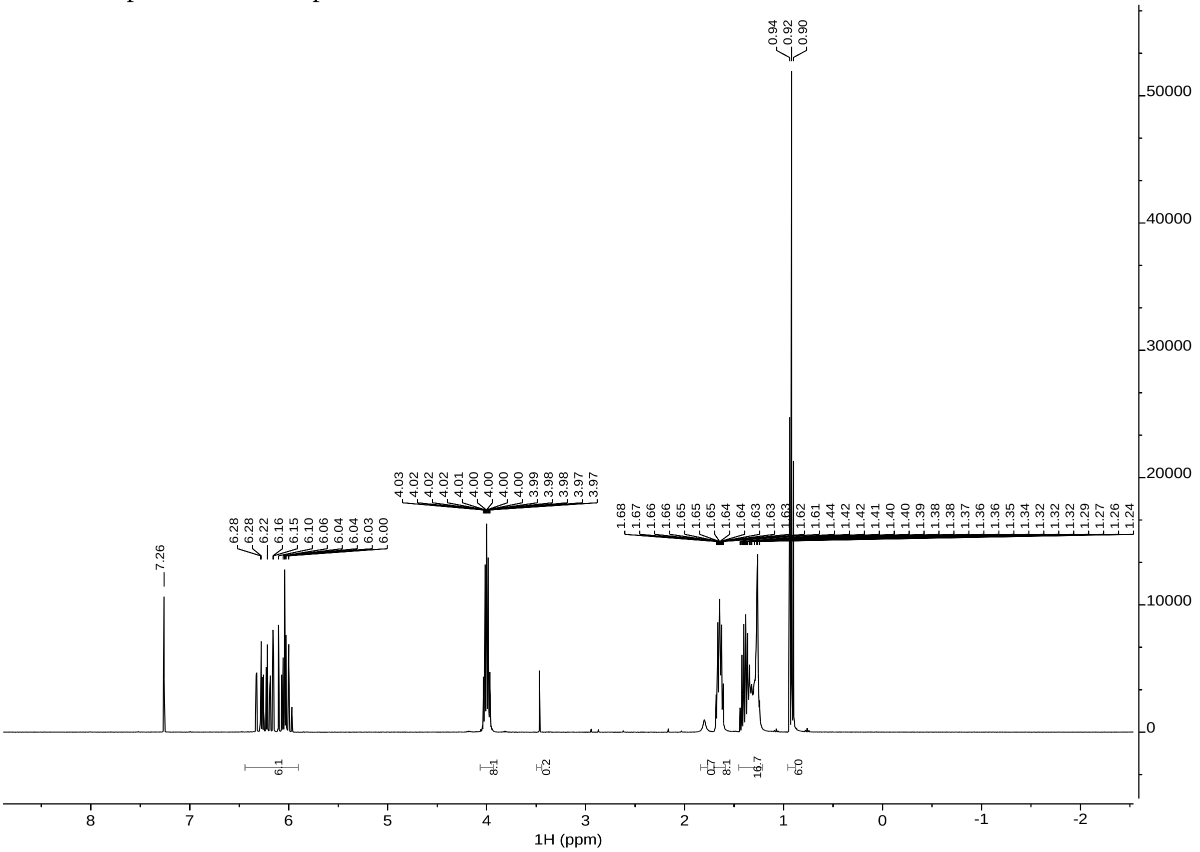

^{13}C NMR spectrum of compound **S76**

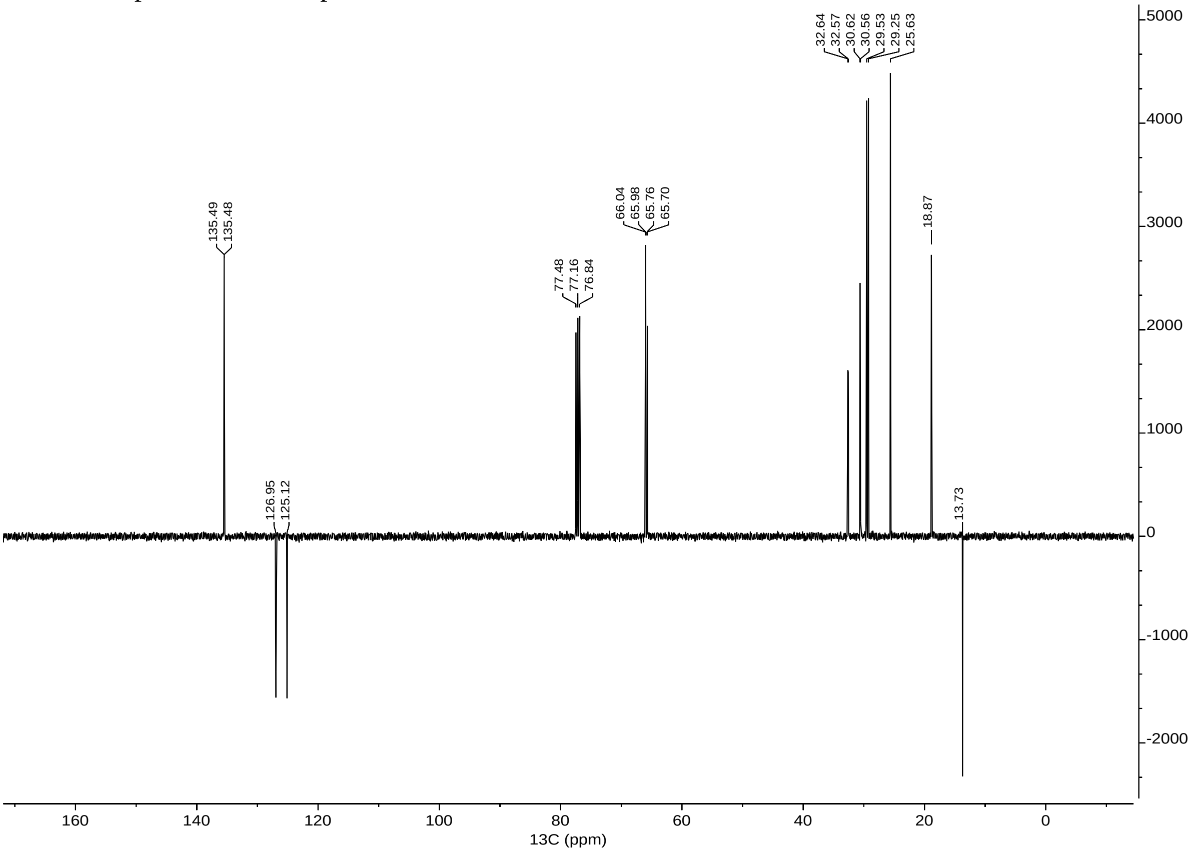

^{31}P{^{1}H} NMR spectrum of compound **S76**

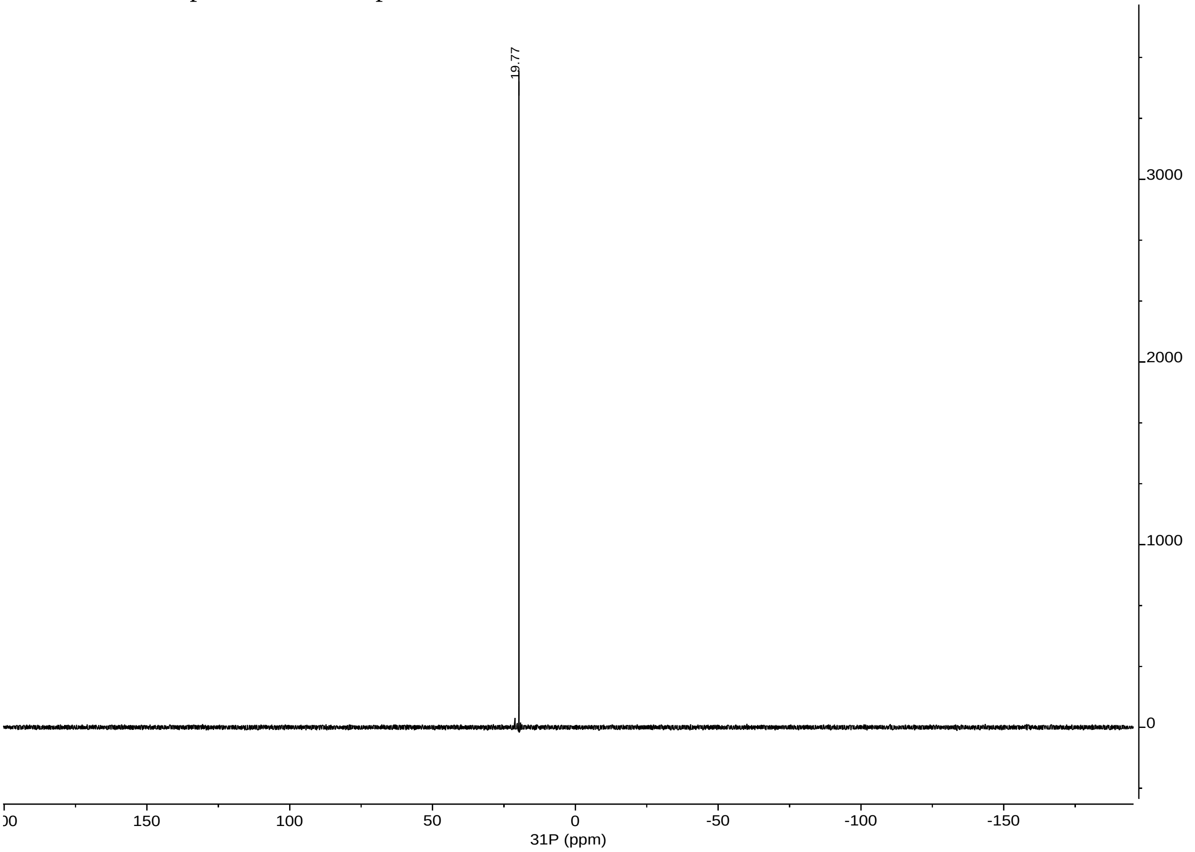

Decane-1,10-diyl dipentyl bis(vinylphosphonate) (S77)

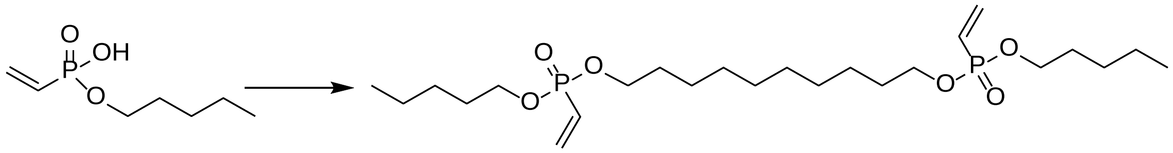

The title compound was prepared according to general method **C** from mono alkyl vinylphosphonate **S22** (1.20 g, 6.73 mmol) and 1,10-dibromodecane (0.67 g, 2.24 mmol) in 76% yield (0.84 g, 1.70 mmol) as a colourless oil.

^{1}H NMR (400 MHz, CDCl$_3$): 6.33–5.97 (m, 6H, C*H*=C*H*$_2$), 4.04–3.97 (m, 8H, CH$_3$(CH$_2$)$_3$C*H*$_2$O, OC*H*$_2$CH$_2$(CH$_2$)$_6$CH$_2$C*H*$_2$O), 1.70–1.62 (m, 8H, CH$_3$(CH$_2$)$_2$C*H*$_2$CH$_2$O, OCH$_2$C*H*$_2$(CH$_2$)$_6$C*H*$_2$CH$_2$O), 1.39–1.25 (m, 20H, CH$_3$(C*H*$_2$)$_2$CH$_2$CH$_2$O, OCH$_2$CH$_2$(C*H*$_2$)$_6$CH$_2$CH$_2$O), 0.92–0.85 (m, 6H, C*H*$_3$(CH$_2$)$_3$CH$_2$O).

^{13}C NMR (101 MHz, CDCl$_3$): 135.49 (d, J = 2.0 Hz, CH=*C*H$_2$), 126.06 (d, J = 184.0 Hz, *C*H=CH$_2$), 66.05, 66.02 (d, J = 5.9 Hz, CH$_3$(CH$_2$)$_2$CH$_2$*C*H$_2$O, O*C*H$_2$CH$_2$(CH$_2$)$_4$CH$_2$*C*H$_2$O), 30.52, 30.30 (d, J = 6.4 Hz, CH$_3$(CH$_2$)$_2$*C*H$_2$CH$_2$O, OCH$_2$CH$_2$(CH$_2$)$_6$*C*H$_2$CH$_2$O), 29.55, 29.27, 27.79, 25.65, 22.37 (CH$_3$(*C*H$_2$)$_2$CH$_2$CH$_2$O, OCH$_2$CH$_2$(*C*H$_2$)$_6$CH$_2$CH$_2$O), 14.09 (*C*H$_3$(CH$_2$)$_3$CH$_2$O).

^{31}P{^{1}H} NMR (162 MHz, CDCl$_3$): 19.76.

IR ν_{max} (KBr) 3085 (vw), 2956 (s), 2930 (vs), 2857 (s), 1613 (w), 1467 (m), 1458 (m), 1397 (m), 1377 (w,sh), 1280 (m), 1253 (vs), 1018 (vs), 1051 (s), 1074 (m), 990 (vs).

HR-MS(ESI$^+$): For $C_{24}H_{48}O_6NaP_2$ (M+Na)$^+$ *m/z* calculated 517.28183, found 517.28171.

^{1}H NMR spectrum of compound **S77**

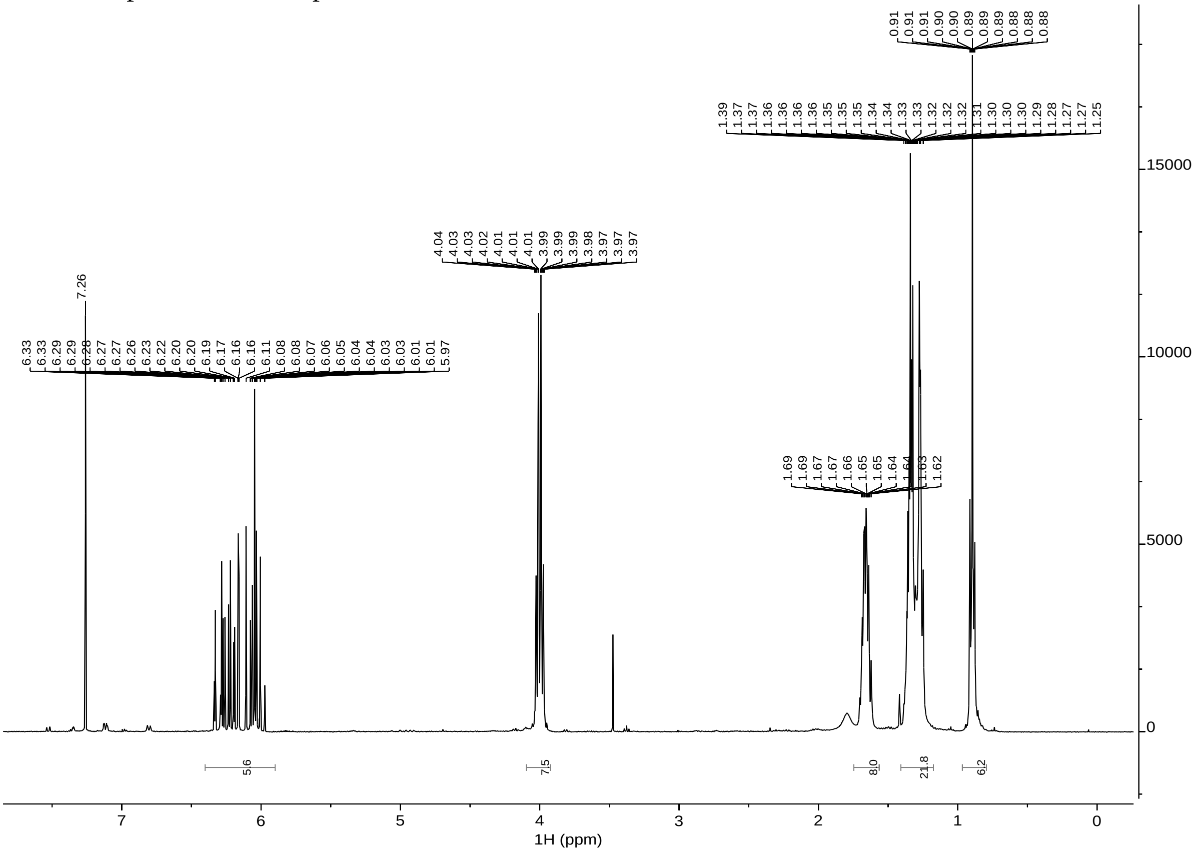

^{13}C NMR spectrum of compound **S77**

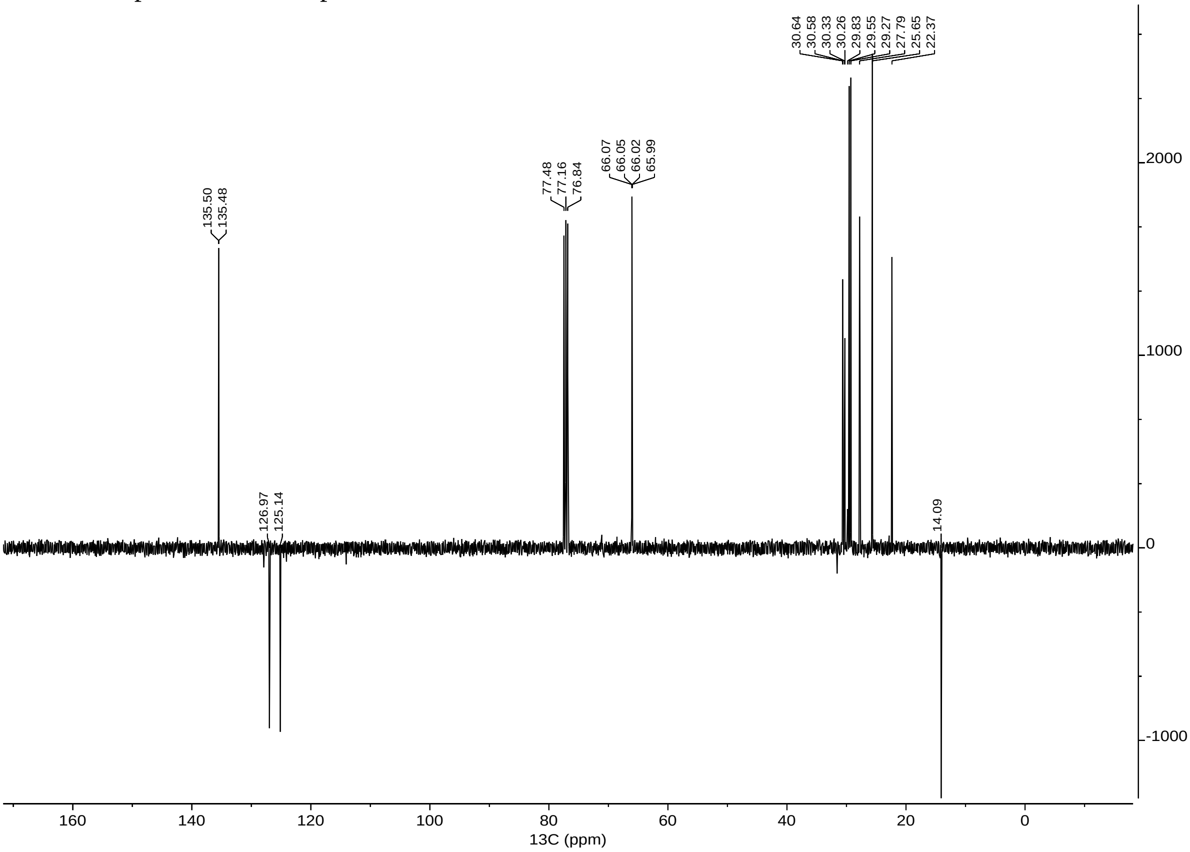

^{31}P{^{1}H} NMR spectrum of compound **S77**

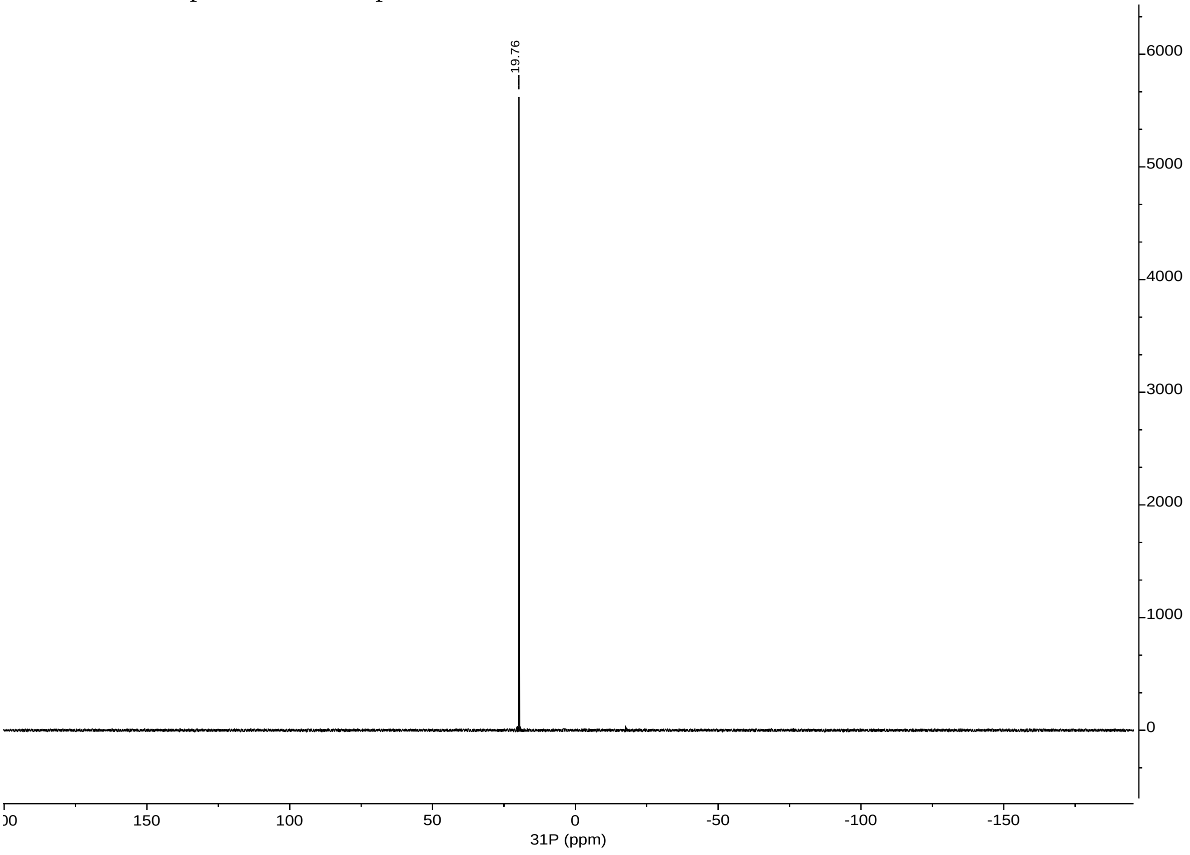

Decane-1,10-diyl dihexyl bis(vinylphosphonate) (S78)

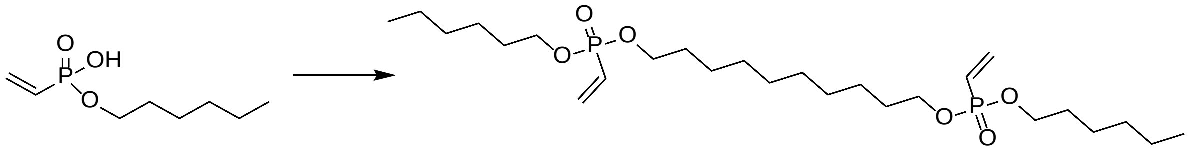

The title compound was prepared according to general method **C** from mono alkyl vinylphosphonate **S23** (1.0 g, 5.20 mmol) and 1,10-dibromodecane (0.62 g, 1.74 mmol) in 75% yield (0.68 g, 1.31 mmol) as a colourless oil.

^{1}H NMR (400 MHz, $CDCl_3$): 6.33–5.97 (m, 6H, C*H*=C*H*$_2$), 4.04–3.96 (m, 8H, $CH_3(CH_2)_4C$*H*$_2$O, OC*H*$_2CH_2(CH_2)_6CH_2C$*H*$_2$O), 1.69–1.62 (m, 8H, $CH_3(CH_2)_3C$*H*$_2CH_2O$, OCH_2C*H*$_2(CH_2)_6C$*H*$_2CH_2O$), 1.40–1.25 (m, 24H, $CH_3(C$*H*$_2)_3CH_2CH_2O$, $OCH_2CH_2(C$*H*$_2)_6CH_2CH_2O$), 0.90–0.85 (m, 6H, C*H*$_3(CH_2)_4CH_2O$).

^{13}C NMR (101 MHz, $CDCl_3$): 135.49 (d, *J* = 1.9 Hz, CH=*C*H$_2$), 126.07 (d, *J* = 184.1 Hz, *C*H=CH$_2$), 66.08, 66.02 (d, *J* = 6.1 Hz, $CH_3(CH_2)_3CH_2$*C*H$_2$O, O*C*H$_2CH_2(CH_2)_4CH_2$*C*H$_2$O), 30.63, 30.56 (d, *J* = 6.2 Hz, $CH_3(CH_2)_3$*C*H$_2CH_2O$, OCH_2*C*H$_2(CH_2)_6$*C*H$_2CH_2O$), 31.48, 29.56, 29.28, 25.65, 25.33, 22.67 (CH_3(*C*H$_2)_3CH_2CH_2O$, OCH_2CH_2(*C*H$_2)_6CH_2CH_2O$), 14.13 (*C*H$_3(CH_2)_4CH_2O$).

^{31}P{^{1}H} NMR (162 MHz, $CDCl_3$): 19.76.

IR ν_{max} (KBr) 3091 (vw), 2959 (s), 2932 (vs), 2872 (m), 2858 (m), 1614 (w), 1468 (m), 1458 (w), 1434 (w), 1400 (m), 1380 (w), 1279 (w), 1241 (s), 1058 (s), 1038 (s, sh), 1007 (vs), 997 (vs), 606 (w).

HR-MS(ESI$^+$): For $C_{26}H_{53}O_6P_2$ $(M+H)^+$ *m/z* calculated 523.33119, found 523.33031.

^{1}H NMR spectrum of compound **S78**

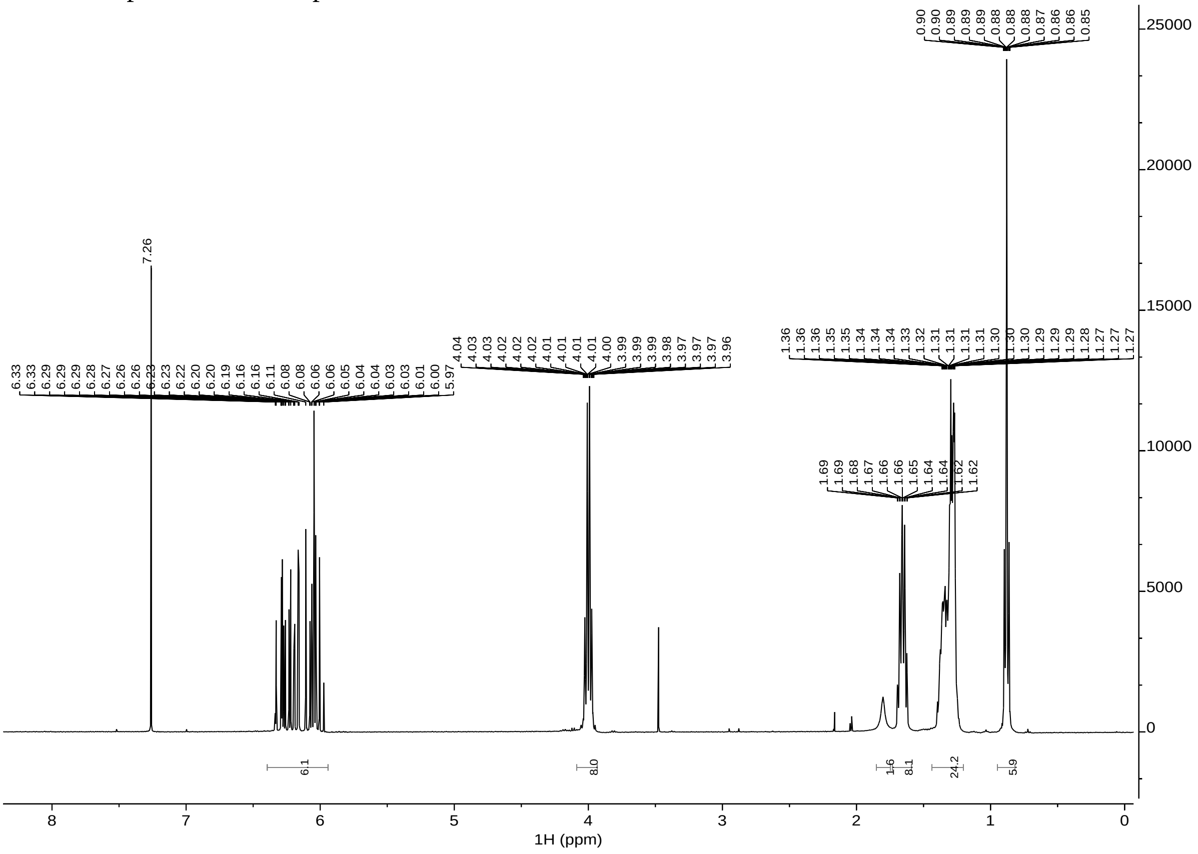

^{13}C NMR spectrum of compound **S78**

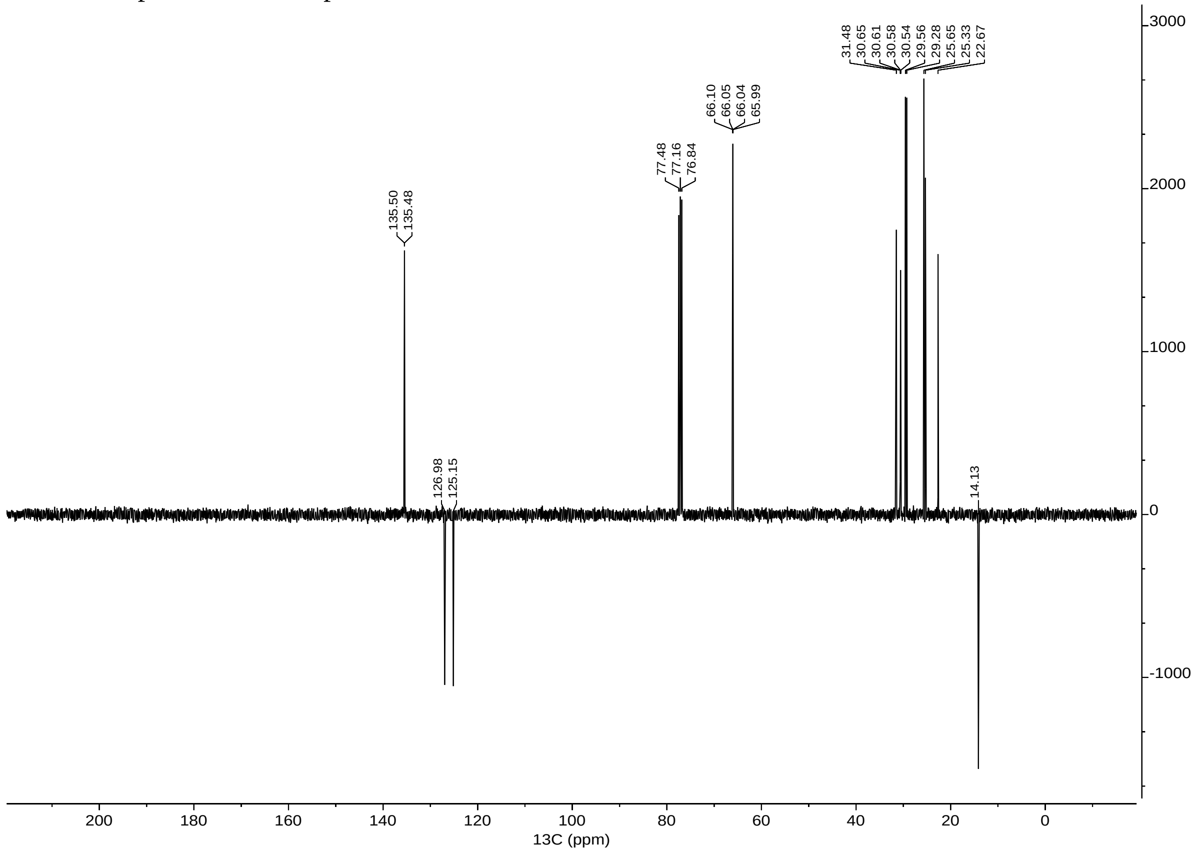

$^{31}P\{^{1}H\}$ NMR spectrum of compound **S78**

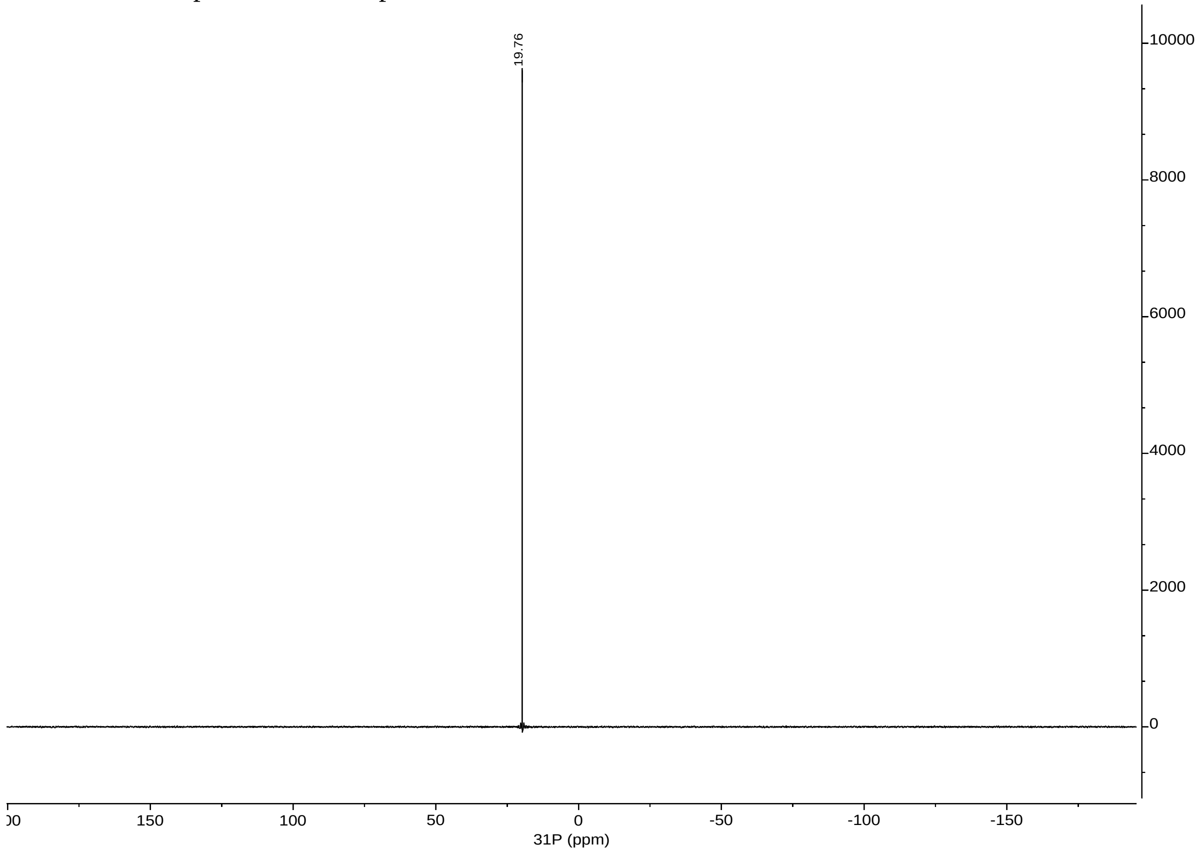

Decane-1,10-diyl dioctyl bis(vinylphosphonate) (S79)

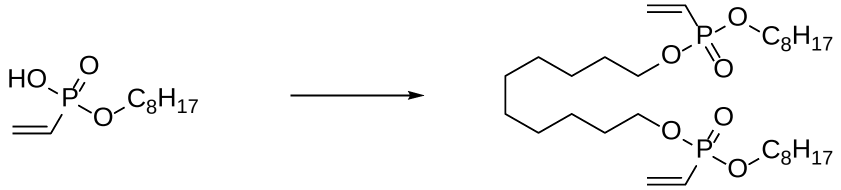

The title compound was prepared according to general method **C** from mono alkyl vinylphosphonate **S28** (2.00 g, 9.08 mmol) and 1,10-dibromodecane (1.00 g, 3.33 mmol) in 47% yield (0.91 g, 1.57 mmol) as a colourless oil.

^{1}H NMR (400 MHz, $CDCl_3$) δ 6.37–5.92 (m, 6H, C*H*=C*H*$_2$), 4.05–3.91 (m, 8H, OC*H*$_2$), 1.74–1.57 (m, 8H, OCH_2C*H*$_2$), 1.44–1.17 (m, 32H, O$(CH_2)_2$(C*H*$_2$)$_3$, (C*H*$_2$)$_2$$CH_3$), 0.94–0.76 (m, 6H, C*H*$_3$).

^{13}C NMR (101 MHz, $CDCl_3$) δ 135.42 (d, *J* = 2.1 Hz, =*C*H$_2$), 126.07 (d, *J* = 184.0 Hz, P*C*H), 66.02 (d, *J* = 5.4 Hz), 65.97 (d, *J* = 5.4 Hz, O*C*H$_2$), 31.87 (*C*H$_2$$CH_2CH_3$), 30.58 (d, *J* = 6.4 Hz, OCH_2*C*H$_2$), 29.53, 29.27, 29.24, 29.22, 25.62, 22.73 (*C*H$_2$$CH_3$), 14.18 (*C*H$_3$).

^{31}P{^{1}H} NMR (162 MHz, $CDCl_3$) δ 19.72.

IR ν_{max}($CHCl_3$) 3091 (vw), 2958 (s), 2930 (vs), 2857 (s), 1614 (w), 1468 (m), 1458 (w), 1400 (m), 1379 (w), ~1303 (w, sh), 1240 (s), ≈1050 (s, sh), 1010 (vs), 989 (vs), 857 (w), 535 (w, br).

HR-MS(ESI$^+$): For $C_{30}H_{60}O_6NaP_2$ $(M+Na)^+$ *m/z* calculated 601.37573, found 601.37549.

^{1}H NMR spectrum of compound **S79**

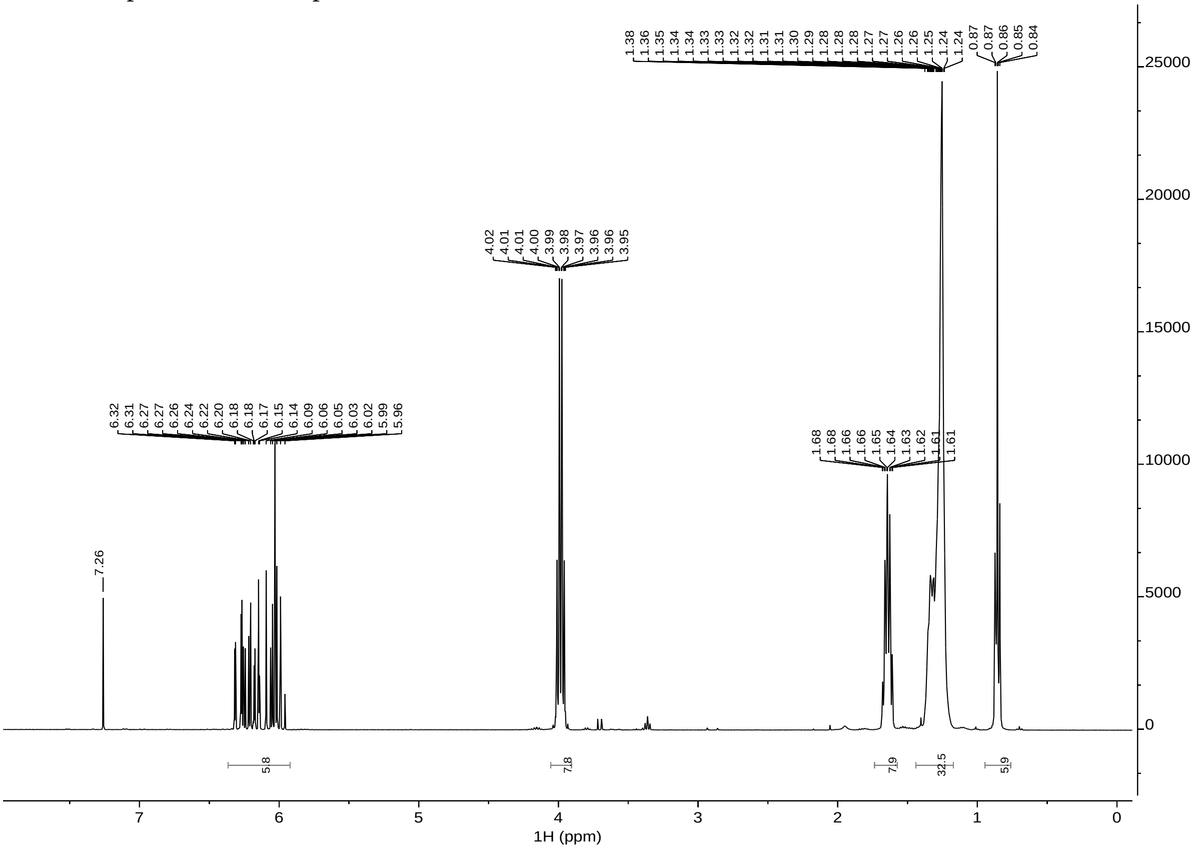

^{13}C NMR spectrum of compound **S79**

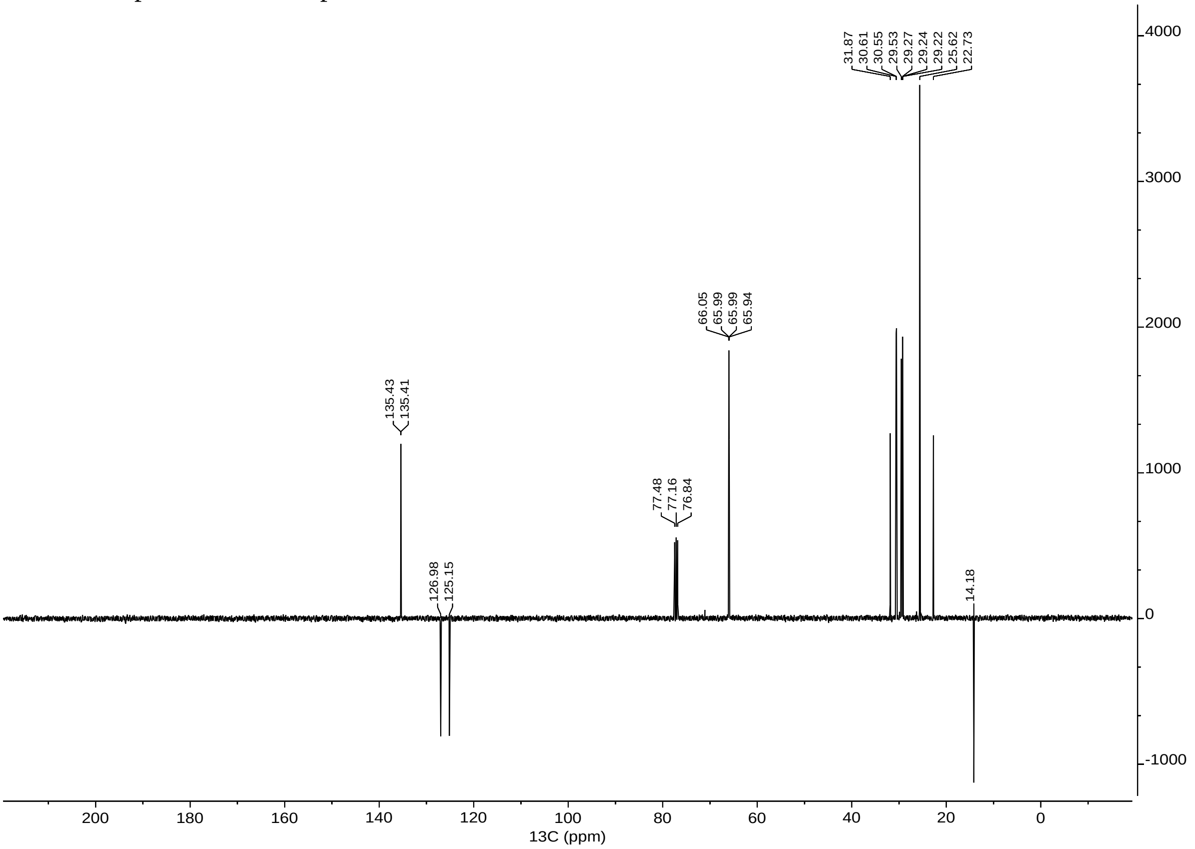

^{31}P{^{1}H} NMR spectrum of compound **S79**

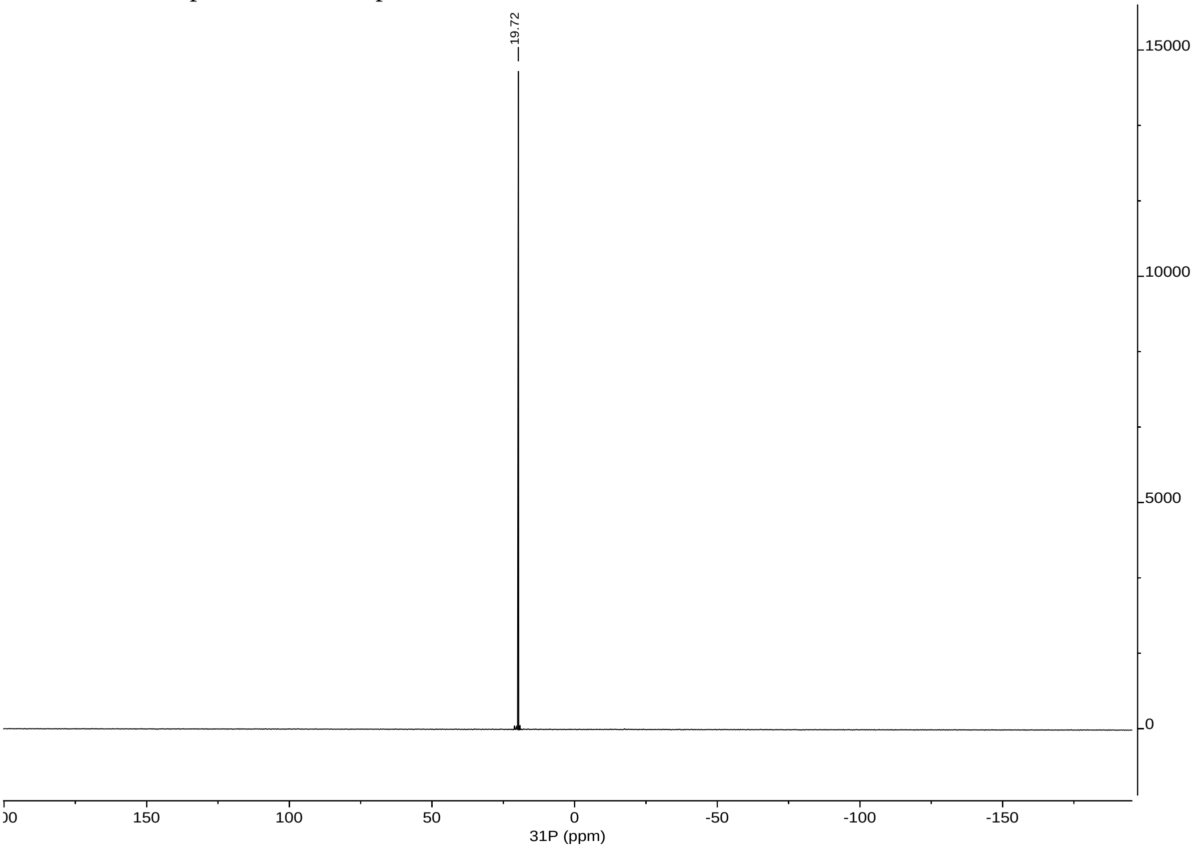

Decane-1,10-diyl diphenethyl bis(vinylphosphonate) (S80)

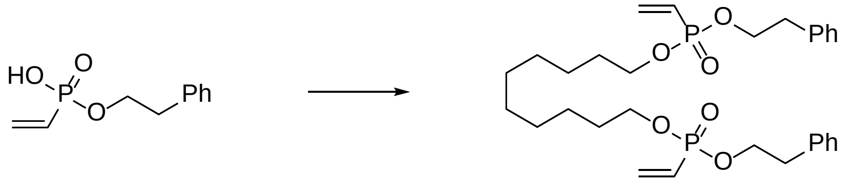

The title compound was prepared according to general method **C** from mono alkyl vinylphosphonate **S31** (0.86 g, 4.05 mmol) and 1,10-dibromodecane (0.30 mL, 1.33 mmol) in 44% yield (0.33 g, 0.59 mmol) as a colourless oil.

^{1}H NMR (401 MHz, CD_3OD) δ 7.32–7.19 (m, 10H, Ph*H*), 6.26–5.96 (m, 6H, C*H*=C*H*$_2$), 4.25–4.17 (m, 4H, OC*H*$_2$(CH_2)$_4$), 3.90 (dtd, *J* = 7.3, 6.5, 2.5 Hz, 4H, OC*H*$_2$$CH_2$Ph), 2.98 (t, *J* = 6.7 Hz, 4H, C*H*$_2$Ph), 1.60 (h, *J* = 6.6 Hz, 4H, OCH_2C*H*$_2$$CH_2$), 1.36–1.27 (m, 12H, O($CH_2$)$_2$(C*H*$_2$)$_3$).

^{13}C NMR (101 MHz, CD_3OD) δ 138.89 (C_{quat}), 137.00 (d, *J* = 1.8 Hz, =*C*H$_2$), 130.17, 129.53, 127.72 (C_{Ph}), 126.16 (d, *J* = 184.4 Hz, P*C*H), 67.97 (d, *J* = 6.1 Hz), 67.39 (d, *J* = 6.3 Hz, O*C*H$_2$), 37.80 (d, *J* = 6.4 Hz), 31.41 (d, *J* = 6.4 Hz, OCH_2*C*H$_2$), 30.51, 30.16, 26.56 (O(CH_2)$_2$(*C*H$_2$)$_3$).

^{31}P{^{1}H} NMR (162 MHz, CD_3OD) δ 20.61.

IR ν_{max}($CHCl_3$) 3089 (vw), 3066 (vw), 3030 (w), 2931 (m), 2857 (m), 1614 (w), 1604 (w), ~1588 (vw, sh), 1467 (w), 1455 (w), 1400 (w), 1279 (w), 1241 (s), ~1155 (vw, sh), 1061 (s), 1010 (vs), 1002 (vs), 989 (s), ~970 (m, sh), 912 (vw), 700 (m), 570 (vw), 496 (vw).

HR-MS(ESI^{+}): For $C_{30}H_{45}O_6P_2$ $(M+H)^{+}$ *m/z* calculated 563.26859, found 563.26810. For $C_{30}H_{44}O_6NaP_2$ $(M+Na)^{+}$ *m/z* calculated 585.25053, found 585.24983.

^{1}H NMR spectrum of compound **S80**

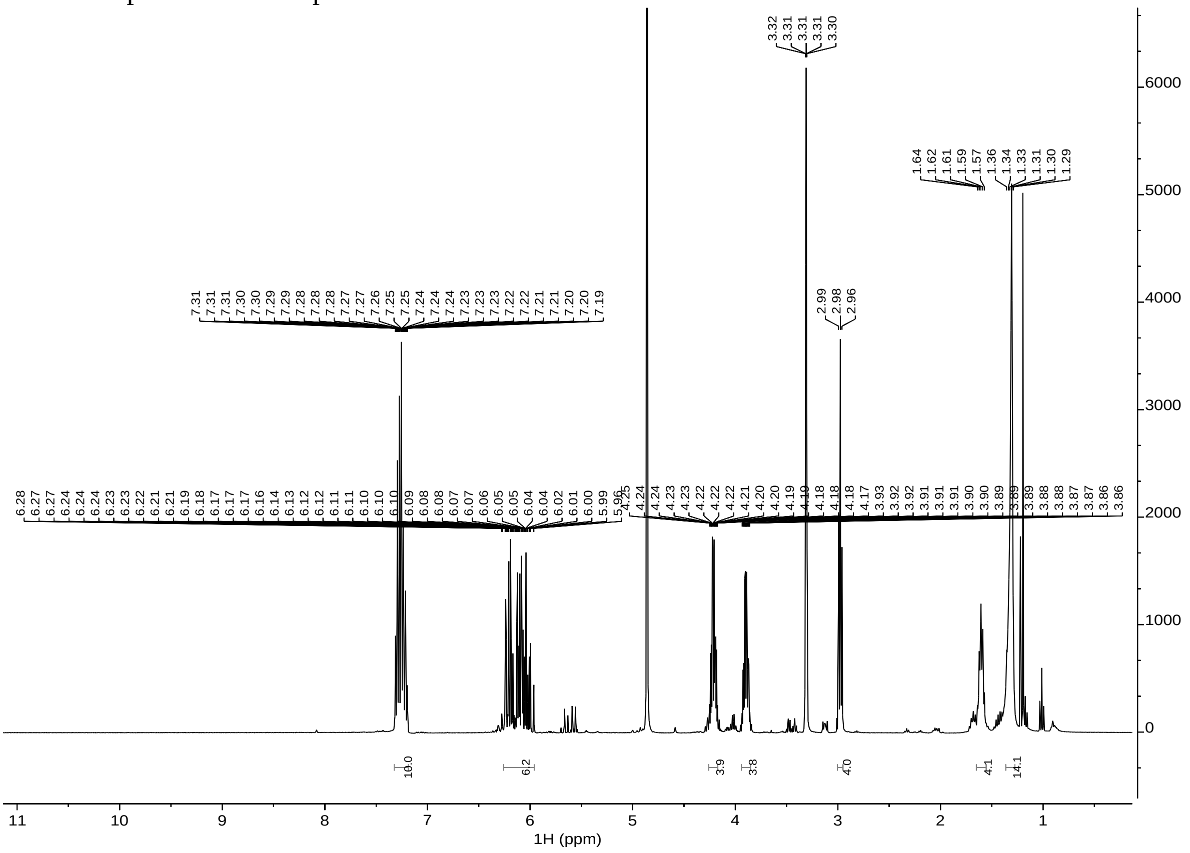

^{13}C NMR spectrum of compound **S80**

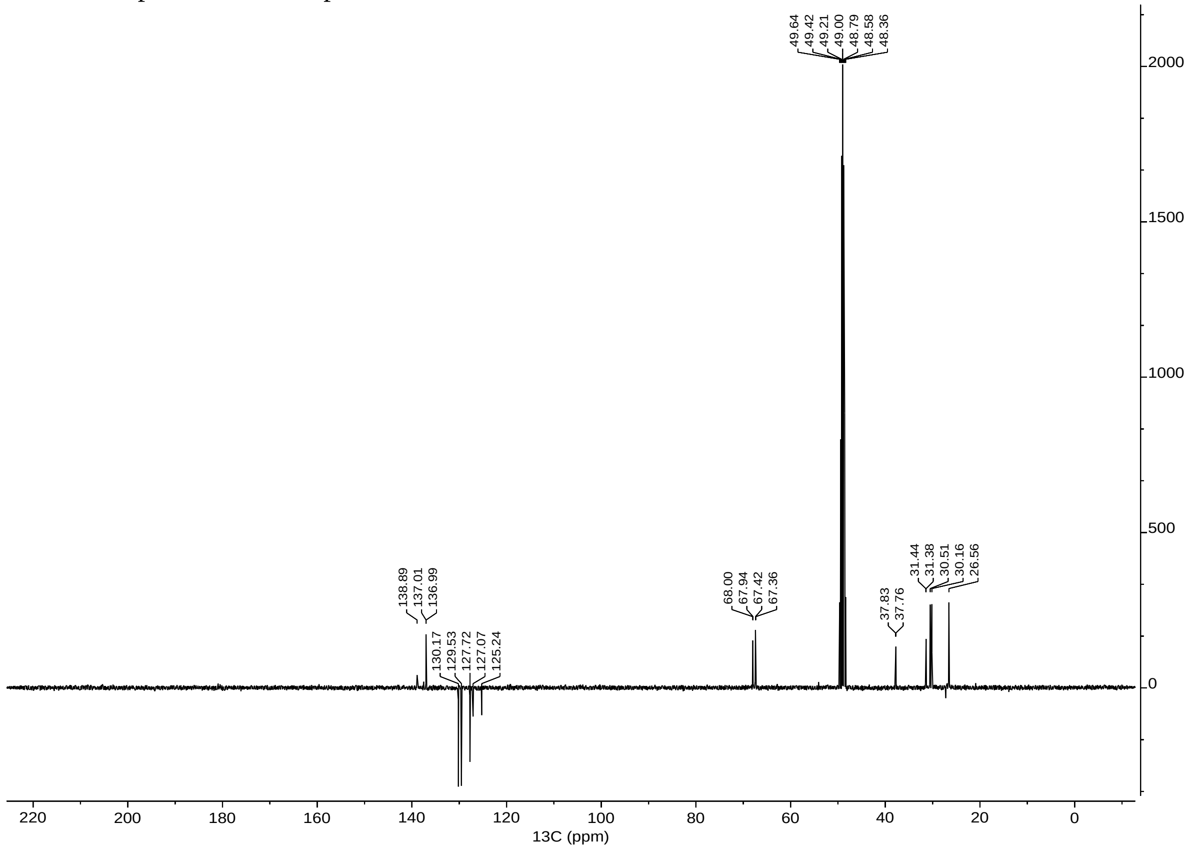

^{31}P{^{1}H} NMR spectrum of compound **S80**

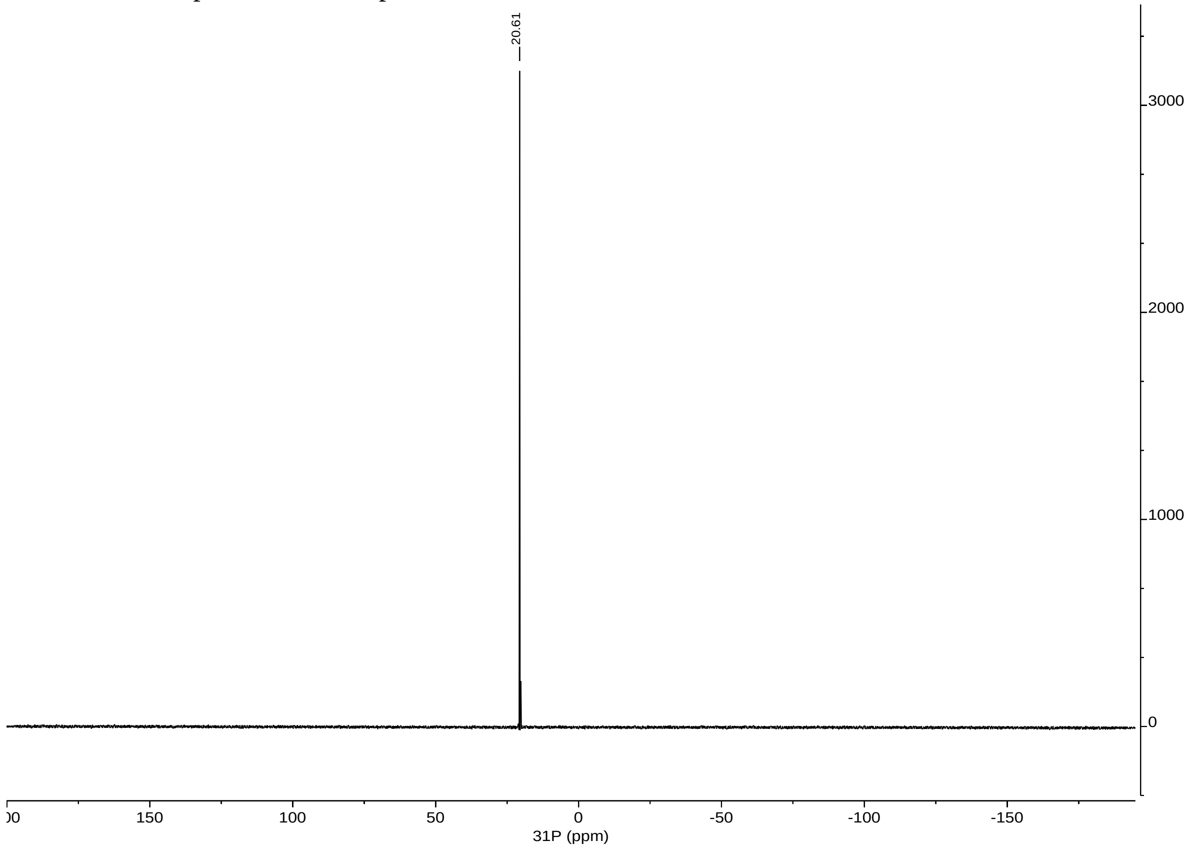

Decane-1,10-diyl didecyl bis(vinylphosphonate) (S81)

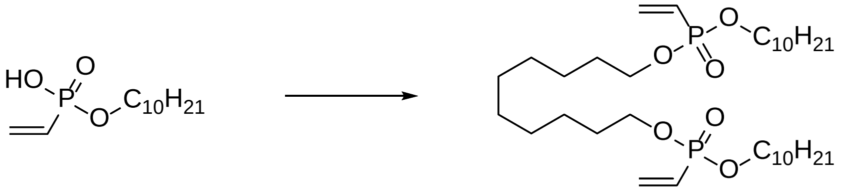

The title compound was prepared according to general method **C** from mono alkyl vinylphosphonate **S33** (1.00 g, 4.03 mmol) and 1,10-dibromodecane (0.30 mL, 1.33 mmol) in 71% yield (0.65 g, 0.94 mmol) as a colourless oil.

^{1}H NMR (400 MHz, $CDCl_3$) δ 6.34–5.96 (m, 6H, C*H*=C*H*$_2$), 4.04–3.95 (m, 8H, OC*H*$_2$), 1.71–1.59 (m, 8H, OCH_2C*H*$_2$), 1.42–1.17 (m, 40H, $O(CH_2)_2$(C*H*$_2$)$_3$, (C*H*$_2$)$_4CH_3$), 0.91–0.82 (m, 6H, C*H*$_3$).

^{13}C NMR (101 MHz, $CDCl_3$) δ 135.43 (d, *J* = 2.0 Hz, =*C*H_2), 126.09 (d, *J* = 184.0 Hz, P*C*H), 66.04 (d, *J* = 5.6 Hz), 65.99 (d, *J* = 5.7 Hz, OCH_2), 32.00 (*C*$H_2CH_2CH_3$), 30.60 (d, *J* = 6.3 Hz, OCH_2*C*H_2), 29.63, 29.55, 29.41, 29.28, 29.26, 25.64, 22.79 (*C*H_2CH_3), 14.22 (*C*H_3).

^{31}P{^{1}H} NMR (162 MHz, $CDCl_3$) δ 19.73.

IR ν_{max}($CHCl_3$) 3090 (vw), 2957 (s), 2929 (vs), 2857 (s), 1614 (w), 1467 (m), ~1457 (m, sh), 1400 (m), 1379 (w), 1279 (w), 1241 (s), ≈1050 (s, sh), ~1011 (s, sh), 991 (vs), 859 (w).

HR-MS(ESI$^+$): For $C_{34}H_{69}O_6P_2$ $(M+H)^+$ *m*/*z* calculated 635.45639, found 635.45633. For $C_{34}H_{68}O_6NaP_2$ $(M+Na)^+$ *m*/*z* calculated 657.43833, found 657.43772.

^{1}H NMR spectrum of compound **S81**

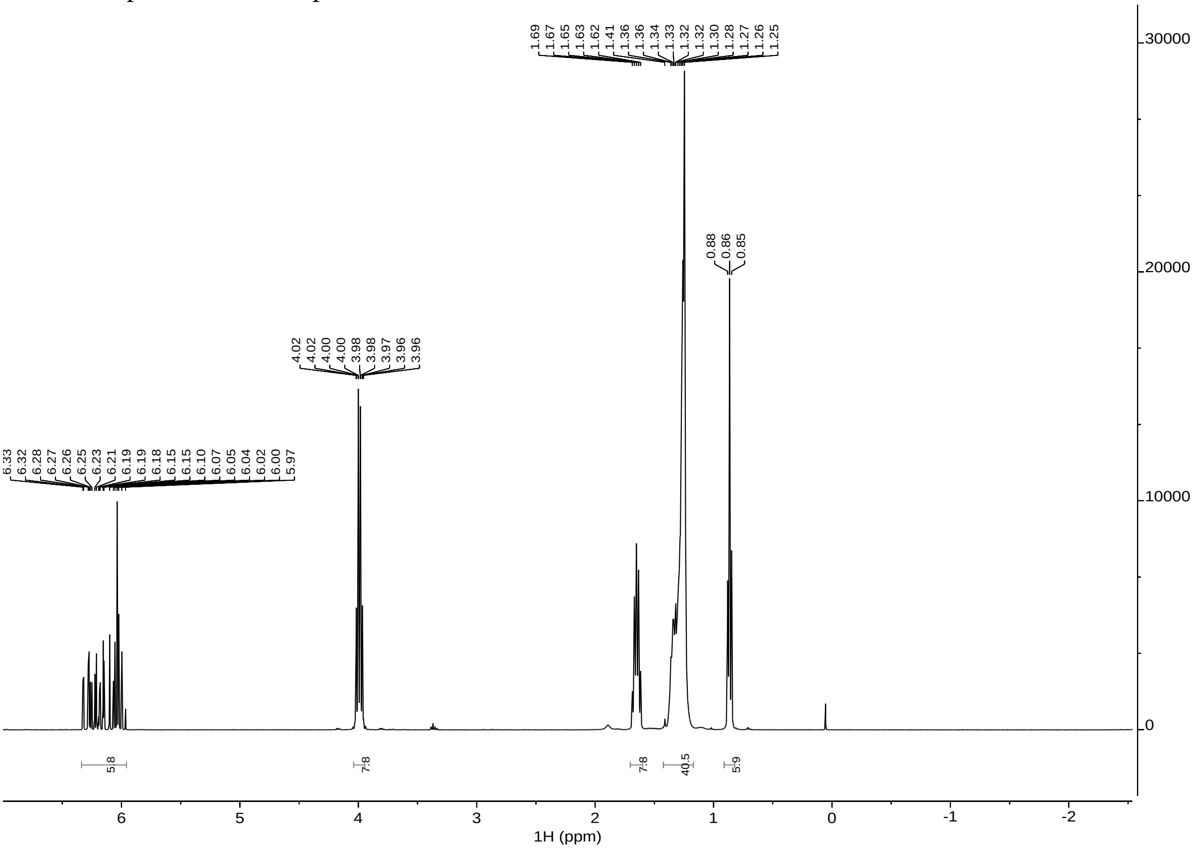

^{13}C NMR spectrum of compound **S81**

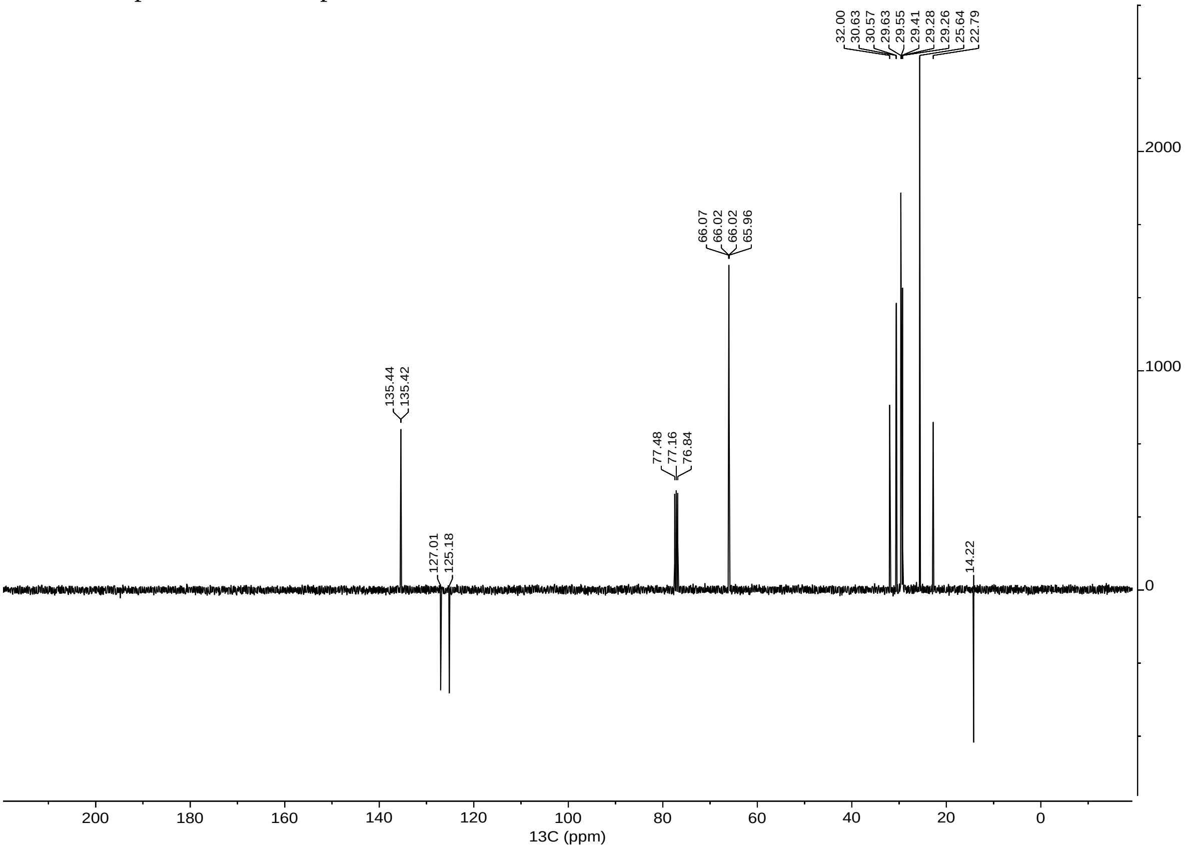

^{31}P{^{1}H} NMR spectrum of compound **S81**

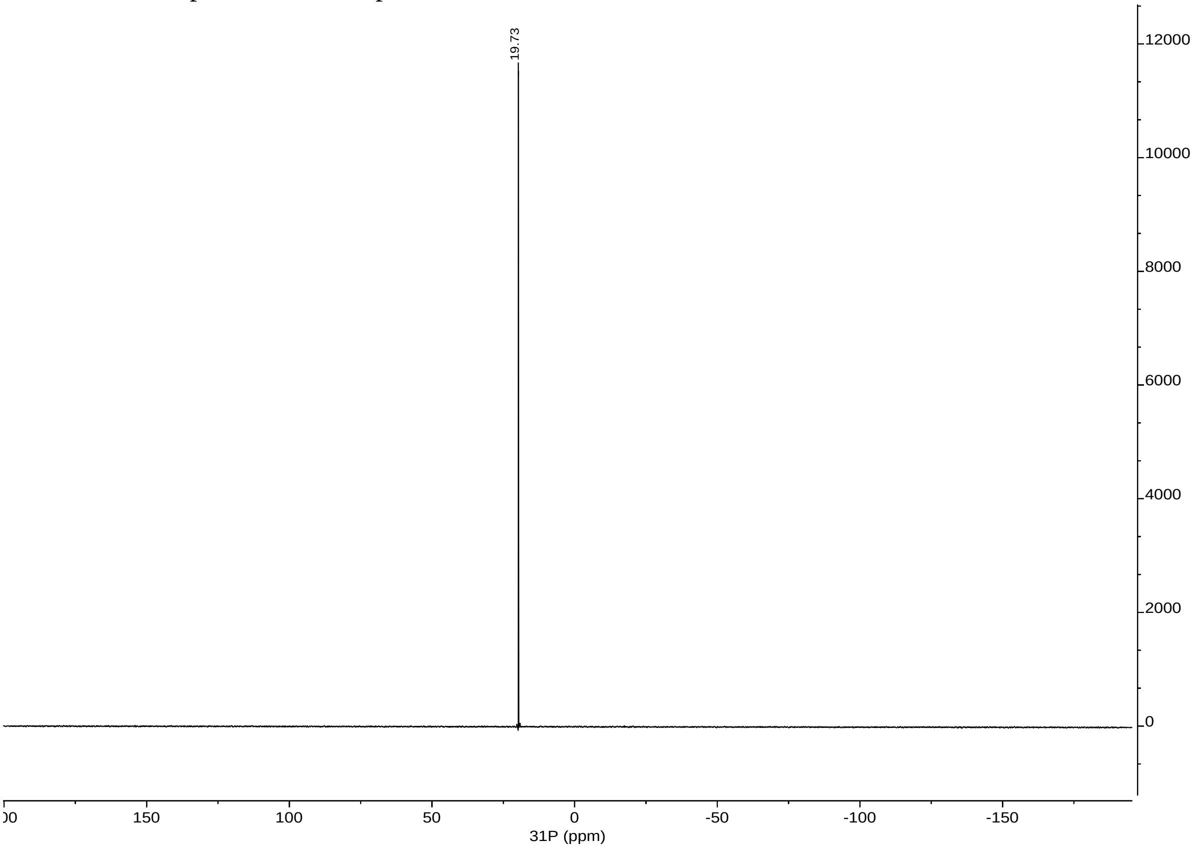

Dodecane-1,12-diyl diisobutyl bis(vinylphosphonate) (S82)

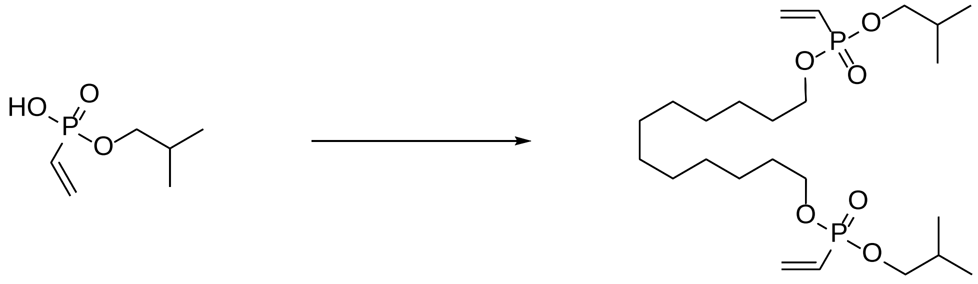

The title compound was prepared according to general method **C** from mono alkyl vinylphosphonate **S19** (523 mg, 3.19 mmol) and 1,12-dibromododecane (98%, 356 µL, 1.06 mmol) in 69% yield (360 mg, 0.73 mmol) as a colourless oil.

^{1}H NMR (401 MHz, $CDCl_3$) δ 6.60–5.95 (m, 6H, C*H*=C*H*$_2$), 4.01 (q, *J* = 6.9 Hz, 4H, OC*H*$_2$CH$_2$), 3.77 (td, *J* = 6.6, 1.6 Hz, 4H, OC*H*$_2$CH), 1.94 (dt, *J* = 13.3, 6.7 Hz, 2H, OCH$_2$C*H*), 1.65 (dt, *J* = 8.1, 6.6 Hz, 4H, OCH$_2$C*H*$_2$), 1.47–1.21 (m, 16H, O(CH$_2$)$_2$(C*H*$_2$)$_4$), 0.94 (d, *J* = 6.7 Hz, 12H, C*H*$_3$).

^{13}C NMR (101 MHz, $CDCl_3$) δ 135.54 (=*C*H$_2$), 125.96 (d, *J* = 184.7 Hz, P*C*H), 71.92 (d, *J* = 6.2 Hz, O*C*H$_2$CH), 66.08 (d, *J* = 6.0 Hz, O*C*H$_2$CH$_2$), 30.61 (d, *J* = 6.4 Hz, OCH$_2$*C*H$_2$), 29.66, 29.63, 29.30 (d, *J* = 7.6 Hz, OCH$_2$*C*H), 29.30, 25.65 (O(CH$_2$)$_2$(*C*H$_2$)$_4$), 18.88 (*C*H$_3$).

^{31}P{^{1}H} NMR (162 MHz, $CDCl_3$) δ 19.95.

IR ν_{max}($CHCl_3$) 3090 (vw), 2964 (s), 2930 (vs), 2875 (m), 2856 (s), 1614 (w), 1471 (s), 1399 (s), 1370 (m), 1279 (m), 1240 (vs), 1167 (w), 1047 (vs, sh), 1012 (vs, vbr), ~988 (vs, sh), ~965 (s, sh), 862 (m).

HR-MS(ESI^{+}): For $C_{24}H_{48}O_6NaP_2$ (M+Na)$^{+}$ *m*/*z* calculated 517.28183, found 517.28131.

^{1}H NMR spectrum of compound **S82**

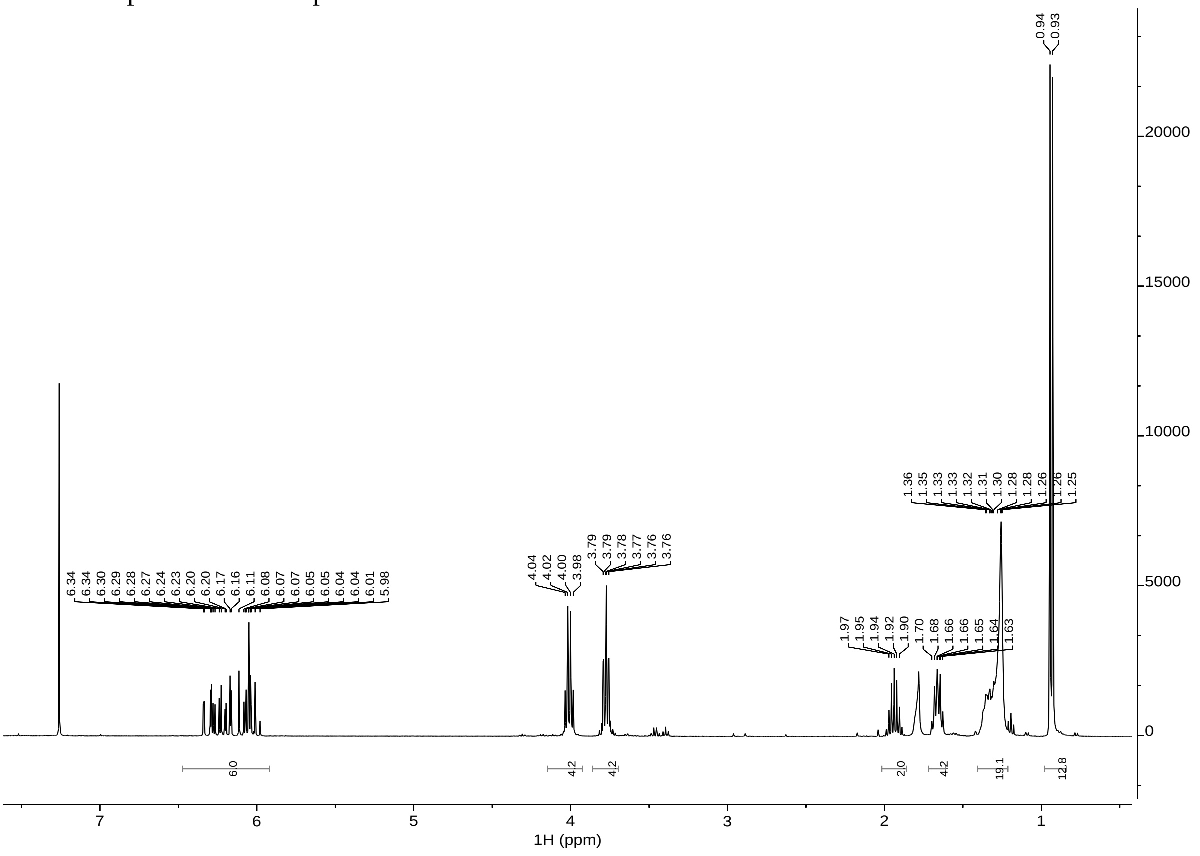

^{13}C NMR spectrum of compound **S82**

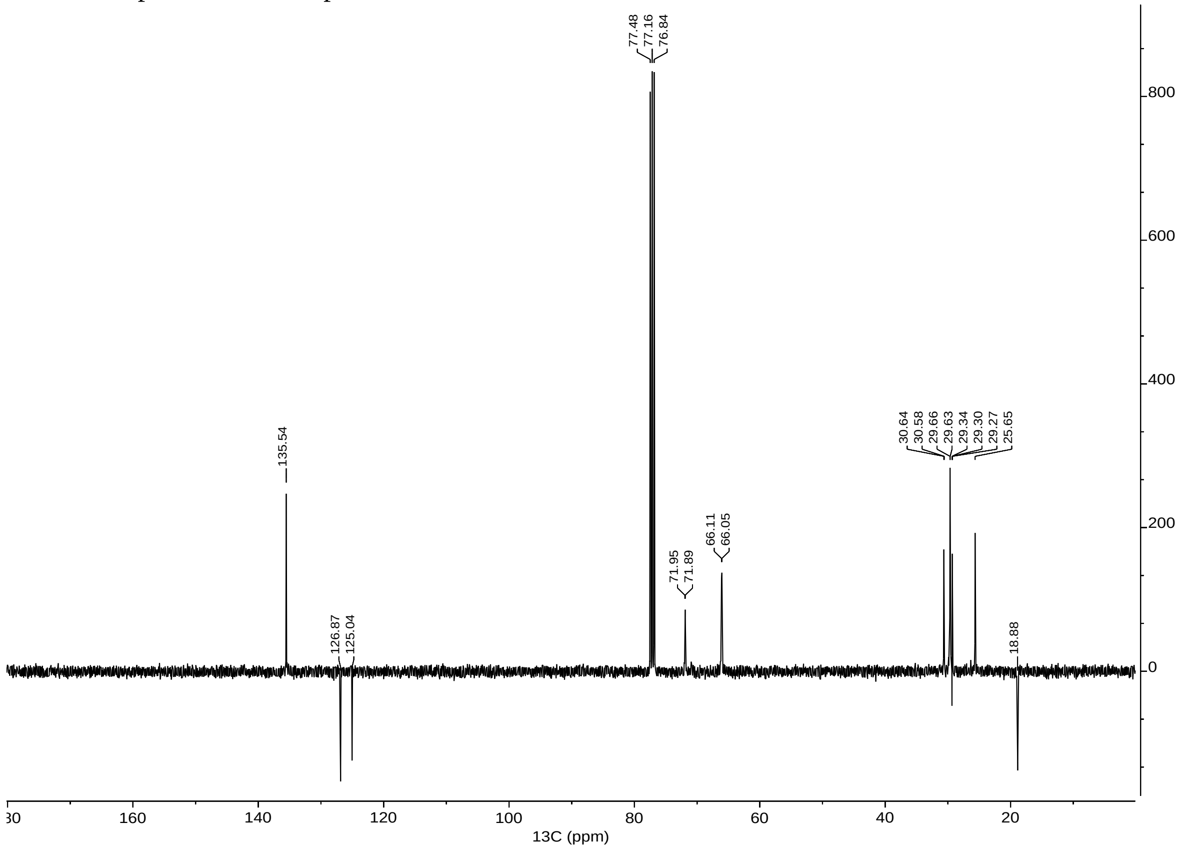

$^{31}P\{^1H\}$ NMR spectrum of compound **S82**

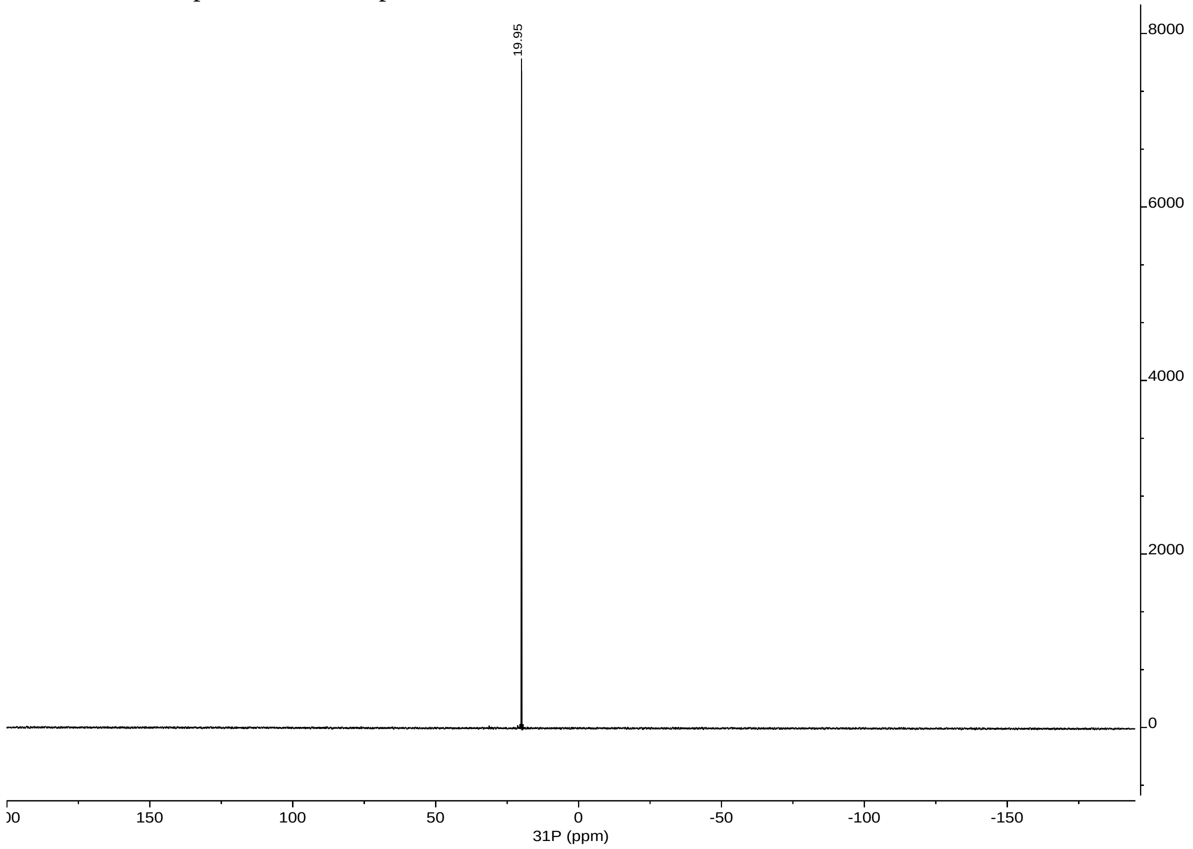

Dibutyl dodecane-1,12-diyl bis(vinylphosphonate) (S83)

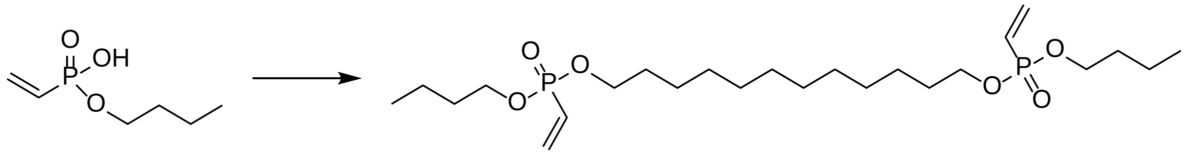

The title compound was prepared according to general method **C** from mono alkyl vinylphosphonate **S20** (1.50 g, 9.14 mmol) and 1,12-dibromododecane (1.0 g, 3.05 mmol) in 65% yield (0.98g, 1.98 mmol) as a colourless oil.

^{1}H NMR (400 MHz, CDCl$_3$): 6.33–5.97 (m, 6H, C*H*=C*H*$_2$), 4.04–3.96 (m, 8H, CH$_3$(CH$_2$)$_3$C*H*$_2$O, OC*H*$_2$CH$_2$(CH$_2$)$_8$CH$_2$C*H*$_2$O), 1.69–1.61 (m, 8H, CH$_3$CH$_2$C*H*$_2$CH$_2$O, OCH$_2$C*H*$_2$(CH$_2$)$_8$C*H*$_2$CH$_2$O), 1.44–1.24 (m, 20H, CH$_3$C*H*$_2$CH$_2$CH$_2$O, OCH$_2$CH$_2$(C*H*$_2$)$_8$CH$_2$CH$_2$O), 0.92 (t, 6H, J = 8.1 Hz, C*H*$_3$(CH$_2$)$_2$CH$_2$O).

^{13}C NMR (101 MHz, CDCl$_3$): 135.45 (d, J = 1.9 Hz, CH=*C*H$_2$), 126.05 (d, J = 183.1 Hz, *C*H=CH$_2$), 66.03, 65.71 (d, J = 6.0 Hz, CH$_3$CH$_2$CH$_2$*C*H$_2$O, O*C*H$_2$CH$_2$(CH$_2$)$_8$CH$_2$*C*H$_2$O), 32.61, 30.60 (d, J = 7.2 Hz, CH$_3$CH$_2$*C*H$_2$CH$_2$O, OCH$_2$CH$_2$(CH$_2$)$_8$*C*H$_2$CH$_2$O), 29.64, 29.61, 29.28, 25.64, 18.87 (CH$_3$*C*H$_2$CH$_2$CH$_2$O, OCH$_2$CH$_2$(*C*H$_2$)$_8$CH$_2$CH$_2$O), 13.72 (*C*H$_3$(CH$_2$)$_2$CH$_2$O).

^{31}P{^{1}H} NMR (162 MHz, CDCl$_3$): 19.74.

IR ν_{max} (KBr) 3086 (vw), 3007 (vw), 2959 (s), 2929 (vs), 2855 (s), 1613 (w), 1466 (m), 1397 (m), 1275 (m, sh), 1252 (vs), 1065 (vs), 1022 (vs), 985 (vs), 980 (m, sh), 721 (m).

HR-MS(ESI$^+$): For $C_{24}H_{49}O_6P_2$ (M+H)$^+$ *m/z* calculated 495.29989, found 495.29965.

^{1}H NMR spectrum of compound **S83**

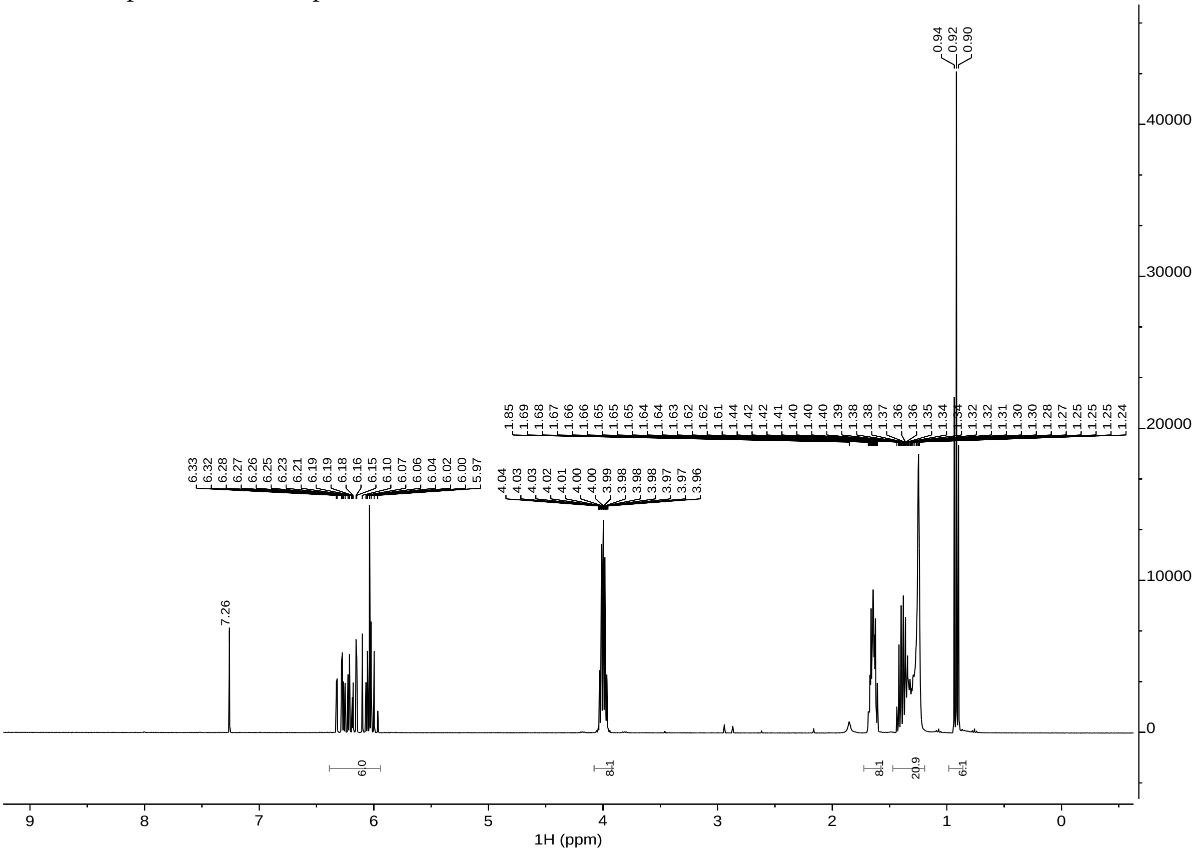

^{13}C NMR spectrum of compound **S83**

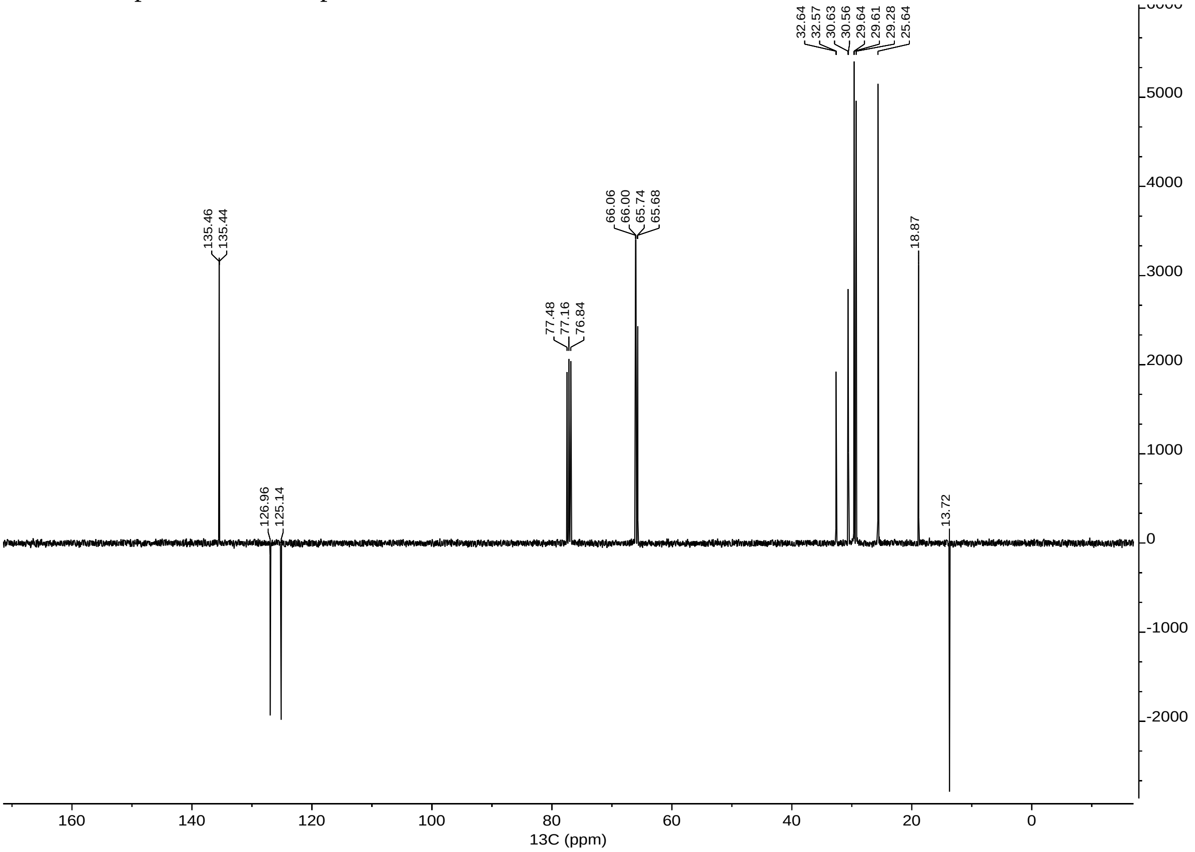

^{31}P{^{1}H} NMR spectrum of compound **S83**

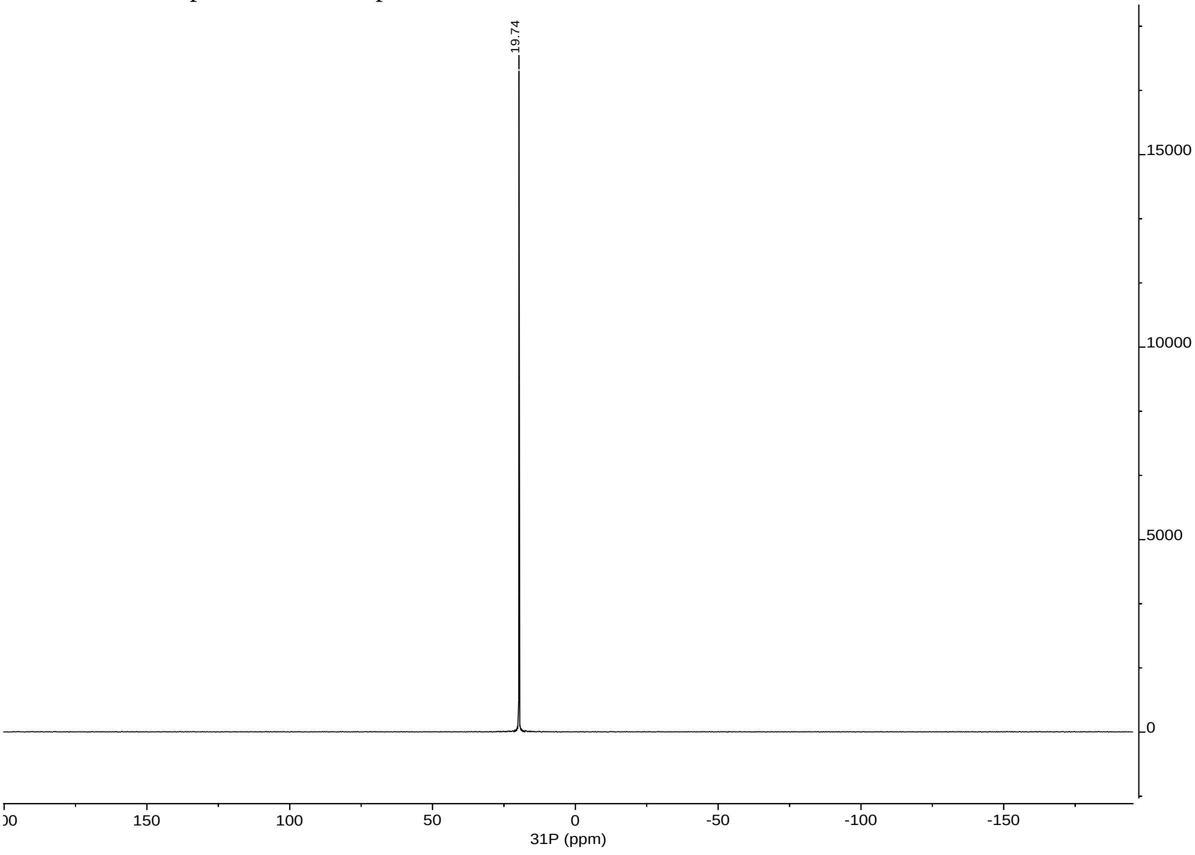

Dodecane-1,12-diyl dipentyl bis(vinylphosphonate) (S84)

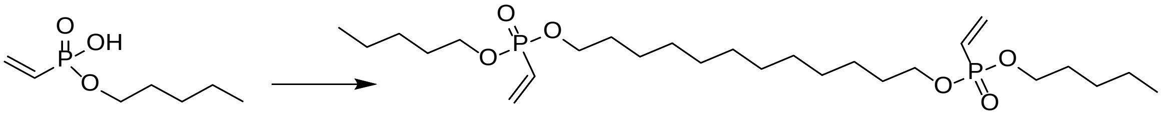

The title compound was prepared according to general method **C** from mono alkyl vinylphosphonate **S22** (1.50 g, 8.42 mmol) and 1,12-dibromododecane (0.92 g, 2.81 mmol) in 78% yield (1.15 g, 2.19 mmol) as a colourless oil.

^{1}H NMR (400 MHz, CD_3OD): 6.32–6.08 (m, 6H, C*H*=C*H*$_2$), 4.05–3.99 (m, 8H, $CH_3(CH_2)_3CH_2O$, $OCH_2CH_2(CH_2)_8CH_2CH_2O$), 1.73–1.65 (m, 8H, $CH_3(CH_2)_2CH_2CH_2O$, $OCH_2CH_2(CH_2)_8CH_2CH_2O$), 1.43–1.29 (m, 24H, $CH_3(CH_2)_2CH_2CH_2O$, $OCH_2CH_2(CH_2)_8CH_2CH_2O$), 0.96–0.91 (m, 6H, $CH_3(CH_2)_3CH_2O$).

^{13}C NMR (101 MHz, CD_3OD): 136.92 (d, J = 1.8 Hz, CH=*C*H$_2$), 126.44 (d, J = 185.0 Hz, *C*H=CH$_2$), 67.44 (d, J = 6.1 Hz, $CH_3(CH_2)_2CH_2CH_2O$, $OCH_2CH_2(CH_2)_8CH_2CH_2O$), 31.49, 31.21 (d, J = 6.3 Hz, $CH_3(CH_2)_2CH_2CH_2O$, $OCH_2CH_2(CH_2)_8CH_2CH_2O$), 30.65, 30.64, 30.22, 28.84, 26.64, 23.27 ($CH_3(CH_2)_2CH_2CH_2O$, $OCH_2CH_2(CH_2)_8CH_2CH_2O$), 14.32 ($CH_3(CH_2)_3CH_2O$).

^{31}P{^{1}H} NMR (162 MHz, CD_3OD): 20.44.

IR ν_{max} (KBr) 3089 (vw), 3013 (w), 2968 (s), 2930 (vs), 2873 (s), 2857 (s), 1614 (w), 1467 (m), 1458 (m), 1434 (w), 1398 (m), 1381 (w), 1279 (m), 1253 (vs), 1052 (vs), 1020 (vs, sh), 989 (vs), 854 (m).

HR-MS(ESI$^+$): For $C_{26}H_{52}O_6NaP_2$ (M+Na)$^+$ m/z calculated 545.31313, found 545.31275.

^{1}H NMR spectrum of compound **S84**

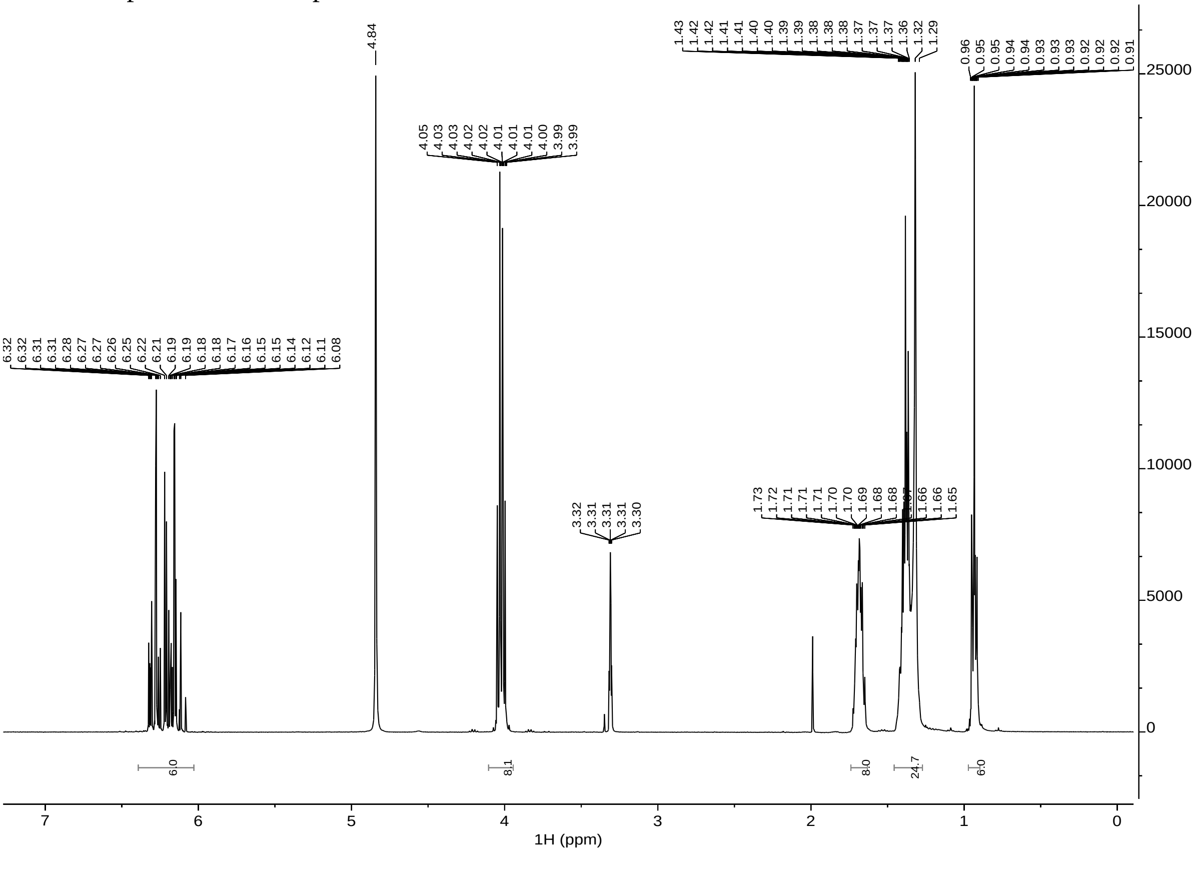

^{13}C NMR spectrum of compound **S84**

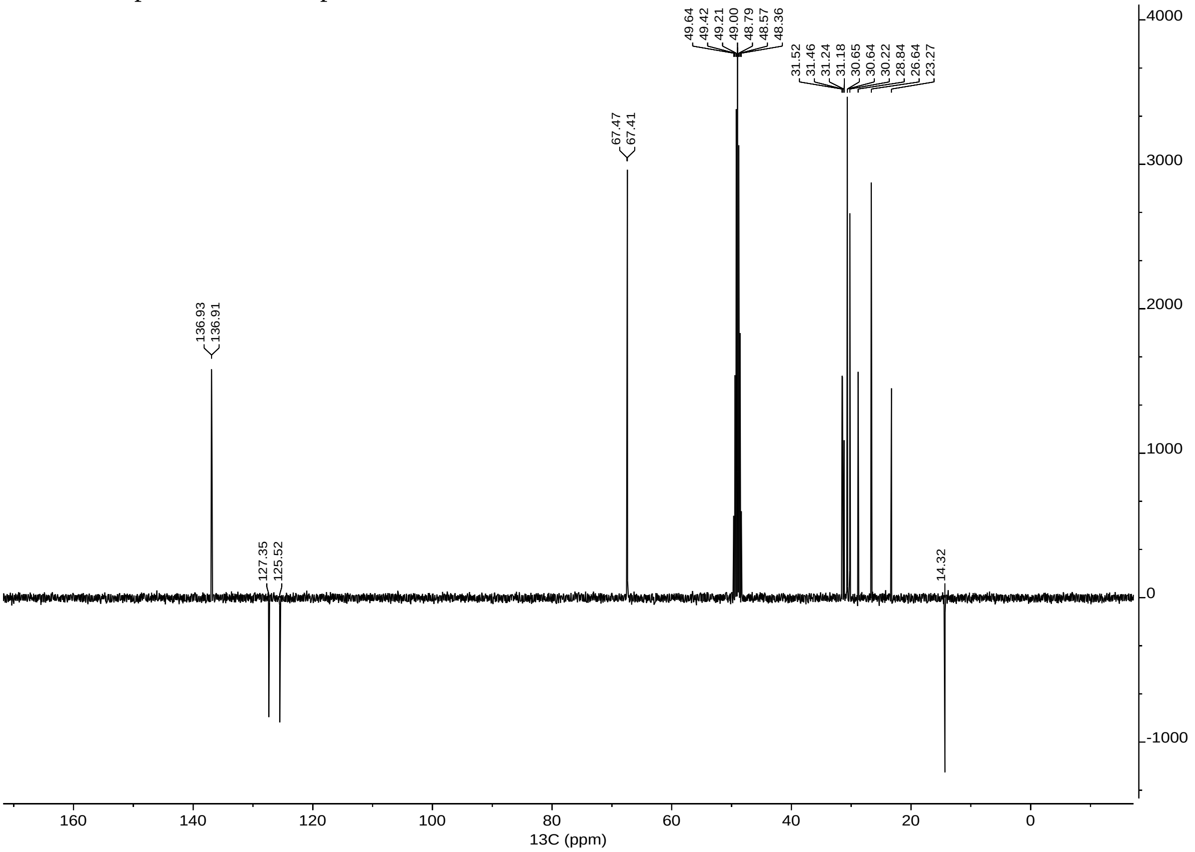

^{31}P{^{1}H} NMR spectrum of compound **S84**

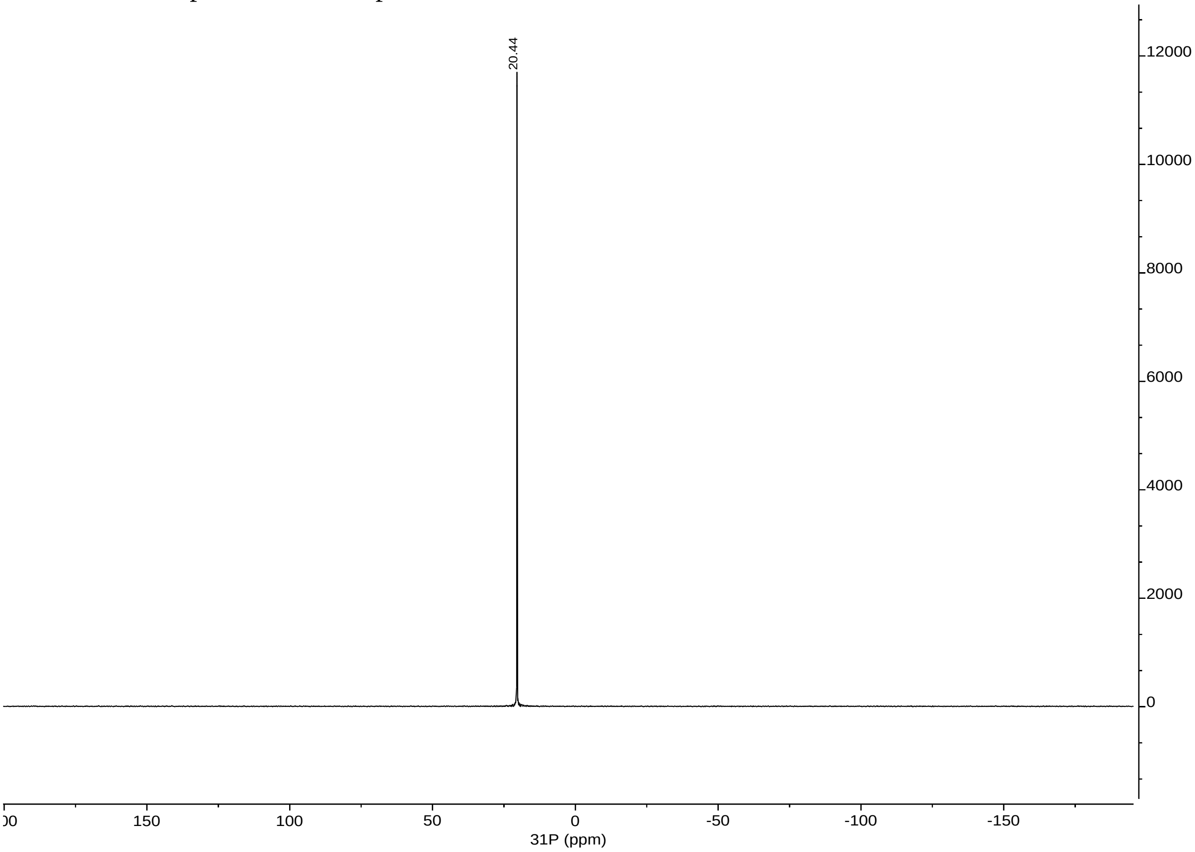

Dodecane-1,12-diyl dioctyl bis(vinylphosphonate) (S85)

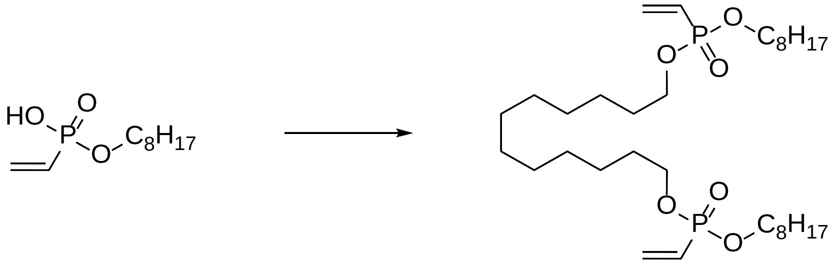

The title compound was prepared according to general method **C** from mono octyl vinylphosphonate **S28** (1.2 g, 5.45 mmol) and 1,12-dibromododecane (0.59 g, 1.8 mmol) in 53% yield (0.58 g, 0.96 mmol) as a colourless oil.

^{1}H NMR (400 MHz, $CDCl_3$) δ 6.35–5.90 (m, 6H, C*H*=C*H*$_2$), 4.07–3.89 (m, 8H, OC*H*$_2$), 1.72–1.55 (m, 8H, OCH_2C*H*$_2$), 1.39–1.16 (m, 36H, $O(CH_2)_2$(C*H*$_2$)$_4$, C*H*$_2$$CH_3$), 0.93–0.79 (m, 6H, C*H*$_3$).

^{13}C NMR (101 MHz, $CDCl_3$) δ 135.38 (d, *J* = 2.1 Hz, =*C*H_2), 126.05 (d, *J* = 184.0 Hz, P*C*H), 65.99 (d, *J* = 5.9 Hz), 65.97 (d, *J* = 6.0 Hz, O*C*H_2), 31.85 (*C*$H_2CH_2CH_3$), 30.56 (d, *J* = 6.3 Hz, OCH_2*C*H_2), 29.61, 29.59, 29.25, 29.20, 25.60, 22.70 (*C*H_2CH_3), 14.15 (*C*H_3).

^{31}P{^{1}H} NMR (162 MHz, $CDCl_3$) δ 19.70.

IR ν_{max}($CHCl_3$) 3091 (vw), 2957 (s), 2929 (vs), 2857 (s), 1614 (w), 1468 (m), 1458 (m), 1400 (m), 1379 (w), ~1303 (w, sh), 1240 (s), ~1050 (s, sh), 1008 (vs), 989 (vs), 857 (w), 535 (w, br).

HR-MS(ESI^+): For $C_{32}H_{64}O_6NaP_2$ $(M+Na)^+$ *m*/*z* calculated 629.40703, found 629.40594.

^{1}H NMR spectrum of compound **S85**

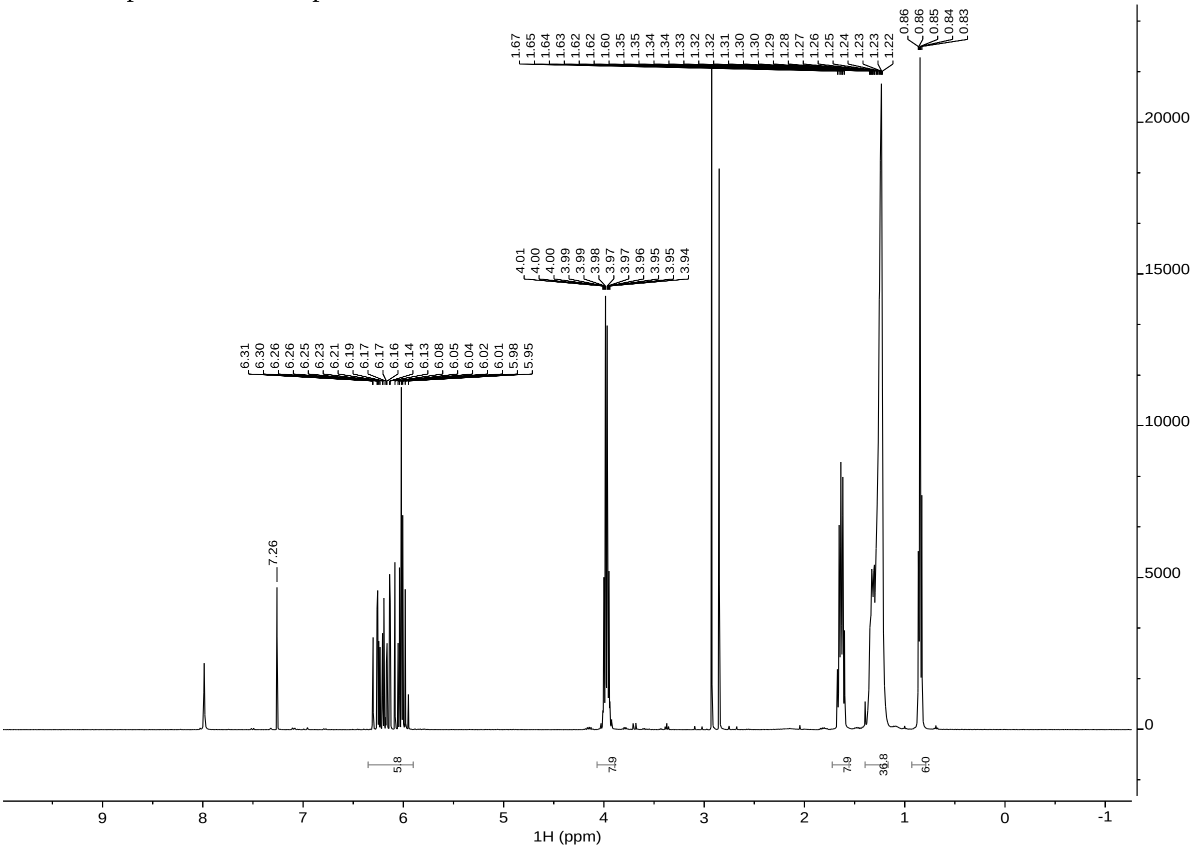

^{13}C NMR spectrum of compound **S85**

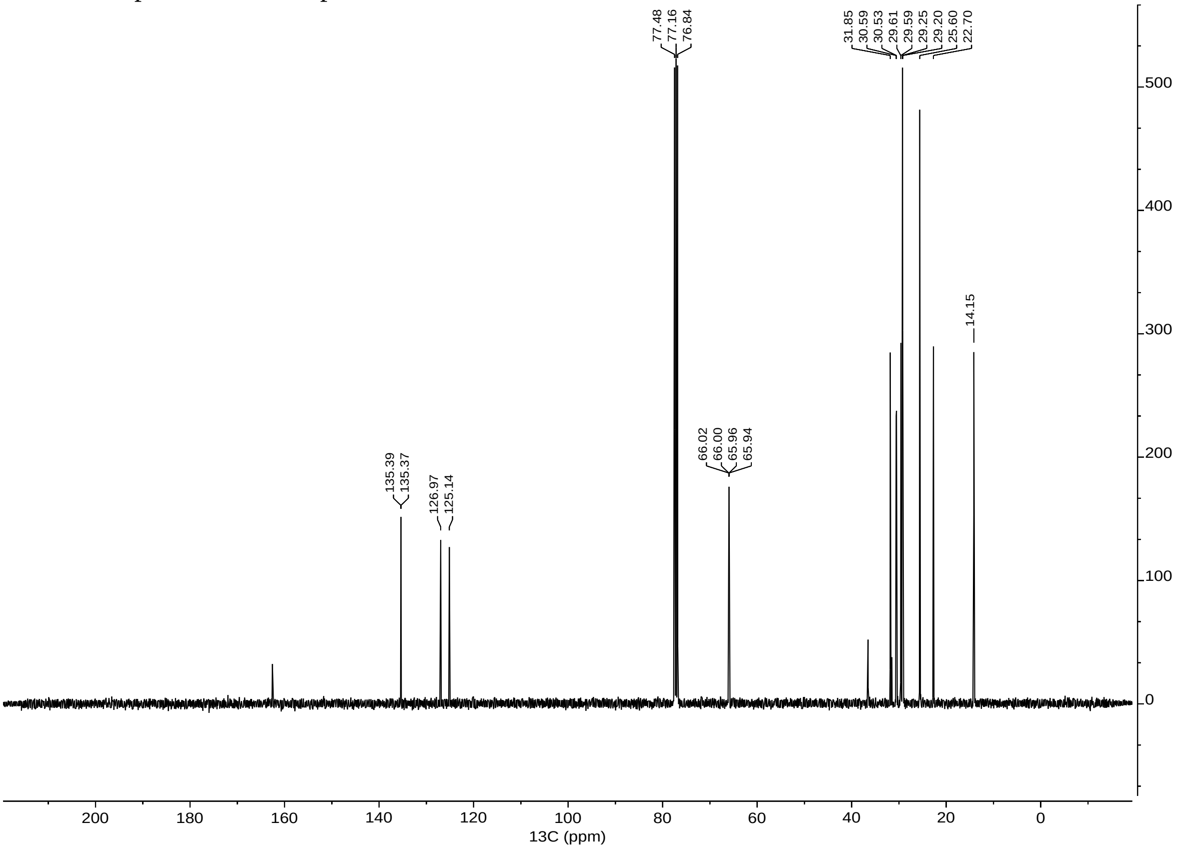

^{31}P{^{1}H} NMR spectrum of compound **S85**

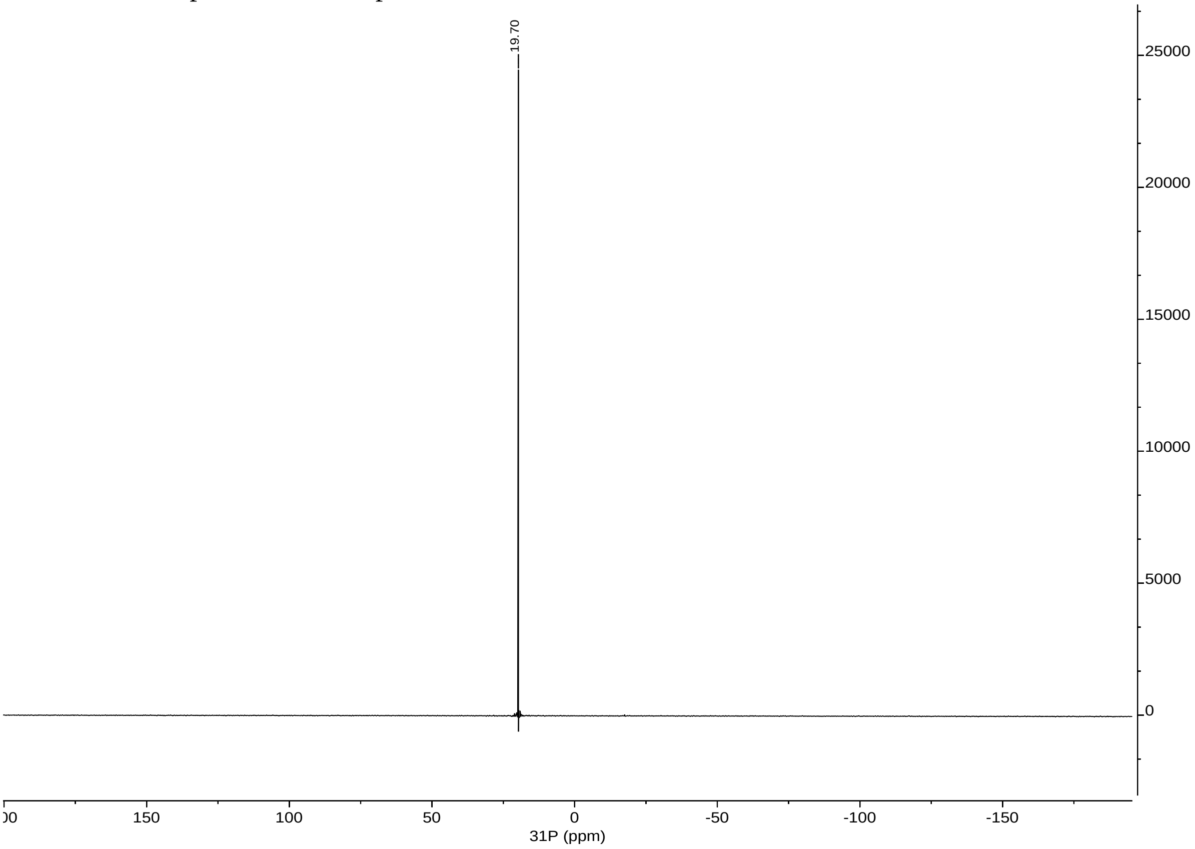

Dodecane-1,12-diyl diphenethyl bis(vinylphosphonate) (S86)

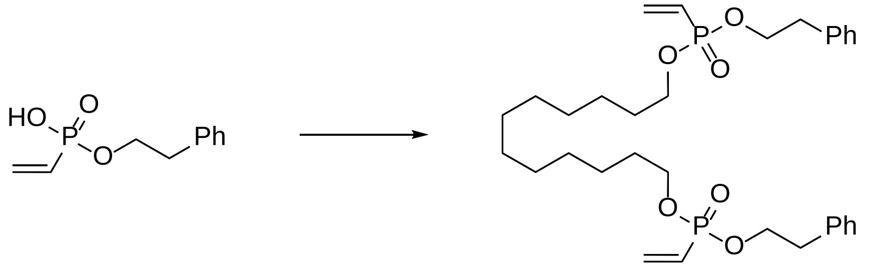

The title compound was prepared according to general method **C** from mono alkyl vinylphosphonate **S31** (1.0 g, 4.7 mmol) and 1,12-dibromododecane (0.56 g, 1.7 mmol) in 81% yield (816 mg, 1.38 mmol) as a colourless oil.

^{1}H NMR (400 MHz, $CDCl_3$) δ 7.33–7.18 (m, 10H, Ph*H*), 6.29–5.87 (m, 6H, C*H*=C*H*$_2$), 4.28–4.15 (m, 4H, OC*H*$_2$(CH_2)$_5$), 3.93 (qd, *J* = 6.8, 1.7 Hz, 4H, OC*H*$_2$$CH_2$Ph), 2.99 (t, *J* = 7.1 Hz, 4H, C*H*$_2$Ph), 1.61 (dt, *J* = 7.9, 6.5 Hz, 4H, OCH_2C*H*$_2$$CH_2$), 1.37–1.19 (m, 16H, O($CH_2$)$_2$(C*H*$_2$)$_4$).

^{13}C NMR (101 MHz, $CDCl_3$) δ 137.45 (CH_2*C*$_{quat}$), 135.58 (d, *J* = 2.1 Hz, =*C*H$_2$), 129.17, 128.62, 126.79 (*C*$_{Ph}$), 125.82 (d, *J* = 184.3 Hz, P*C*H), 66.29 (d, *J* = 5.8 Hz), 66.07 (d, *J* = 5.9 Hz, O*C*H2), 37.14 (d, *J* = 6.3 Hz), 30.57 (d, *J* = 6.3 Hz, OCH_2*C*H$_2$), 29.66, 29.63, 29.28, 25.62 (O(CH_2)$_2$(*C*H$_2$)$_4$).

^{31}P{^{1}H} NMR (162 MHz, $CDCl_3$) δ 19.77.

IR ν_{max}($CHCl_3$) 3089 (vw), 3066 (vw), 3031 (w), 1614 (w), 1605 (w), 1497 (w), 1454 (m), 1400 (m), 1279 (m), 1241 (s), 1087 (m), 1061 (s), ~1028 (s, sh), 1010 (vs), 1002 (vs), 860 (w), ~988 (s, sh), 912 (vw), 700 (m).

HR-MS(ESI$^+$): For $C_{32}H_{49}O_6P_2$ $(M+H)^+$ *m/z* calculated 591.29989, found 591.29918. For $C_{32}H_{48}O_6NaP_2$ $(M+Na)^+$ *m/z* calculated 613.28183, found 613.28082.

^{1}H NMR spectrum of compound **S86**

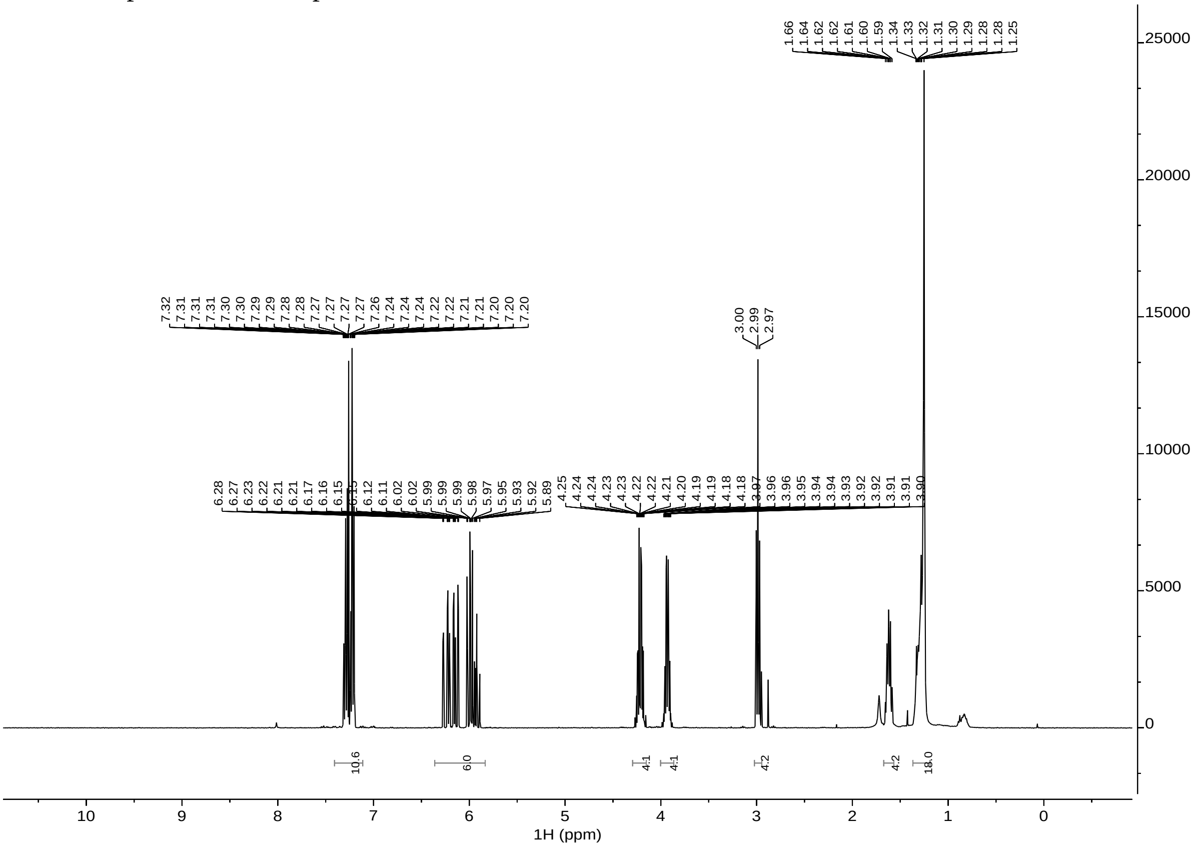

^{13}C NMR spectrum of compound **S86**

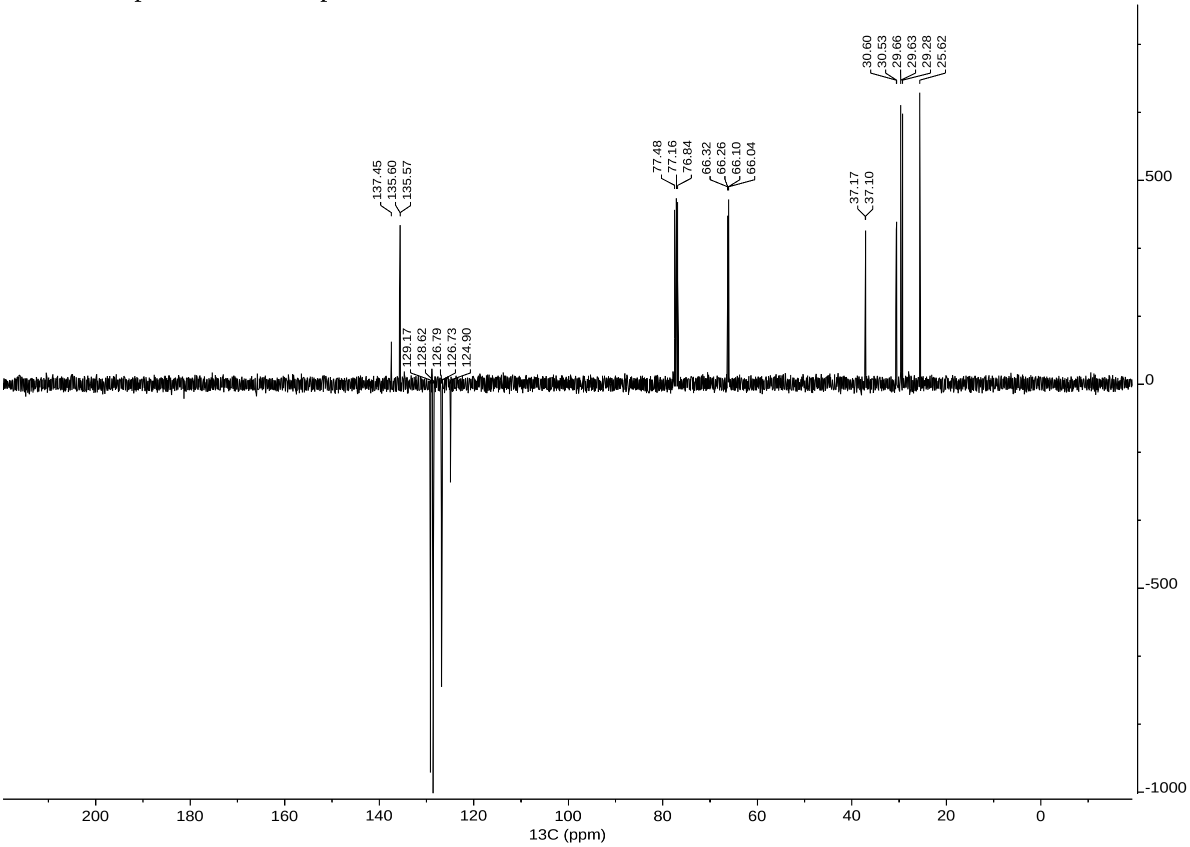

$^{31}P\{^{1}H\}$ NMR spectrum of compound **S86**

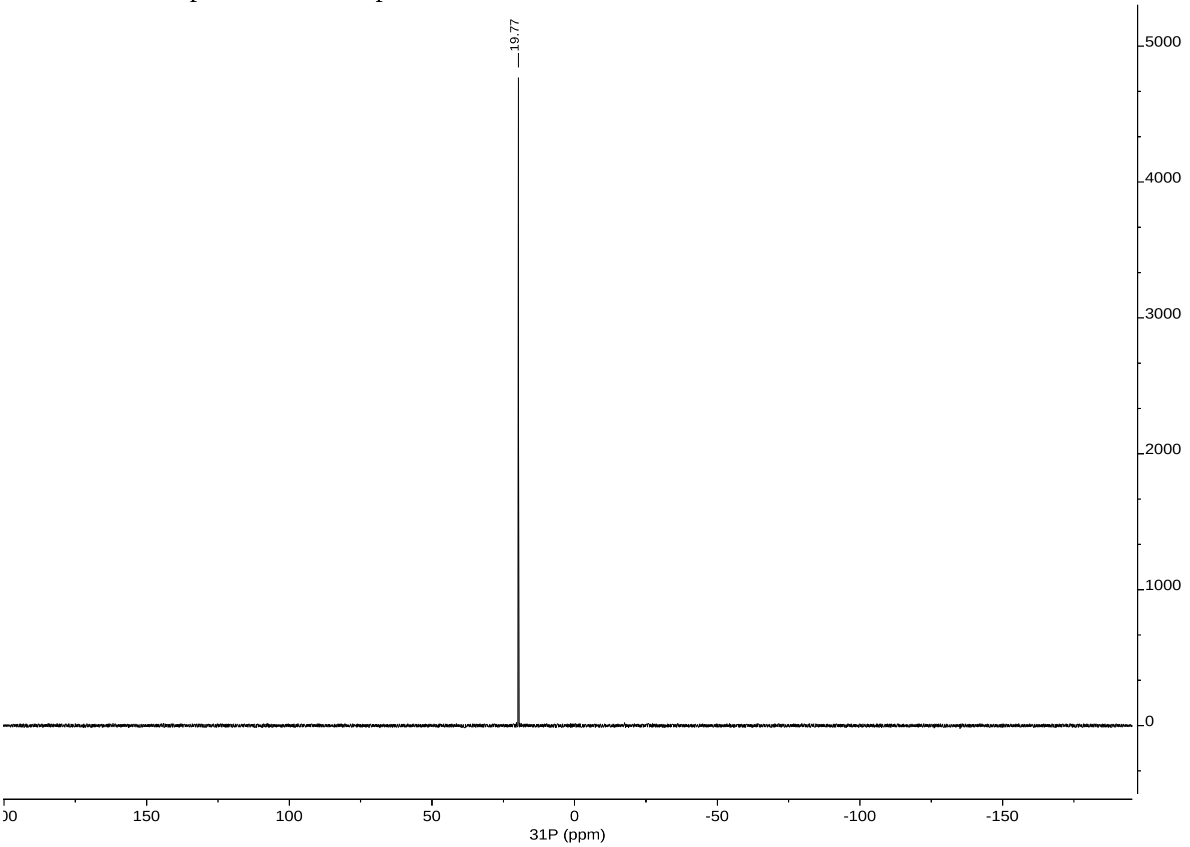

Diisobutyl tetradecane-1,14-diyl bis(vinylphosphonate) (S87)

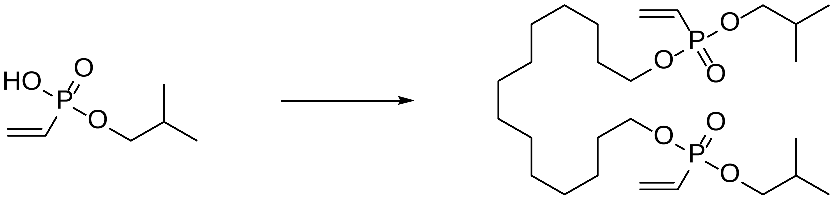

The title compound was prepared according to general method **C** from mono alkyl vinylphosphonate **S19** (527 mg, 3.21 mmol) and 1,14-dibromotetradecane (381 mg, 1.07 mmol) in 89% yield (496 mg, 0.95 mmol) as a colourless oil.

^{1}H NMR (401 MHz, $CDCl_3$) δ 6.38–5.96 (m, 6H, C*H*=C*H*$_2$), 4.08–3.96 (m, 4H, OC*H*$_2$CH$_2$), 3.77 (tt, *J* = 6.7, 1.0 Hz, 4H, OC*H*$_2$CH), 1.94 (dp, *J* = 13.3, 6.7 Hz, 2H, OCH$_2$C*H*), 1.66 (dt, *J* = 8.2, 6.5 Hz, 4H, OCH$_2$C*H*$_2$), 1.44–1.20 (m, 20H, O(CH$_2$)$_2$(C*H*$_2$)$_5$), 0.94 (d, *J* = 6.7 Hz, 12H, C*H*$_3$).

^{13}C NMR (101 MHz, $CDCl_3$) δ 135.53 (=*C*H$_2$), 125.97 (d, *J* = 184.6 Hz, P*C*H), 71.92 (d, *J* = 6.1 Hz, O*C*H$_2$CH), 66.09 (d, *J* = 6.0 Hz, O*C*H$_2$CH$_2$), 30.62 (d, *J* = 6.3 Hz, OCH$_2$*C*H$_2$), 29.76, 29.70, 29.65, 29.31 (d, *J* = 7.3 Hz, OCH$_2$*C*H), 29.30, 25.66 (O(CH$_2$)$_2$(*C*H$_2$)$_5$), 18.86 (*C*H$_3$).

^{31}P{^{1}H} NMR (162 MHz, $CDCl_3$) δ 19.95.

IR ν_{max}($CHCl_3$) 3091 (vw), 2964 (s), 2929 (vs), 2875 (m), 2856 (s), 1614 (w), 1471 (m), ~1458 (w, sh), 1400 (m), 1370 (w), 1279 (m), 1239 (s), ~1048 (s, sh), 1012 (vs), ~988 (s, sh).

HR-MS(ESI$^+$): For $C_{26}H_{53}O_6P_2$ $(M+H)^+$ *m/z* calculated 523.33119, found 523.33167. For $C_{26}H_{52}O_6NaP_2$ $(M+Na)^+$ *m/z* calculated 545.31313, found 545.31342.

^{1}H NMR spectrum of compound **S87**

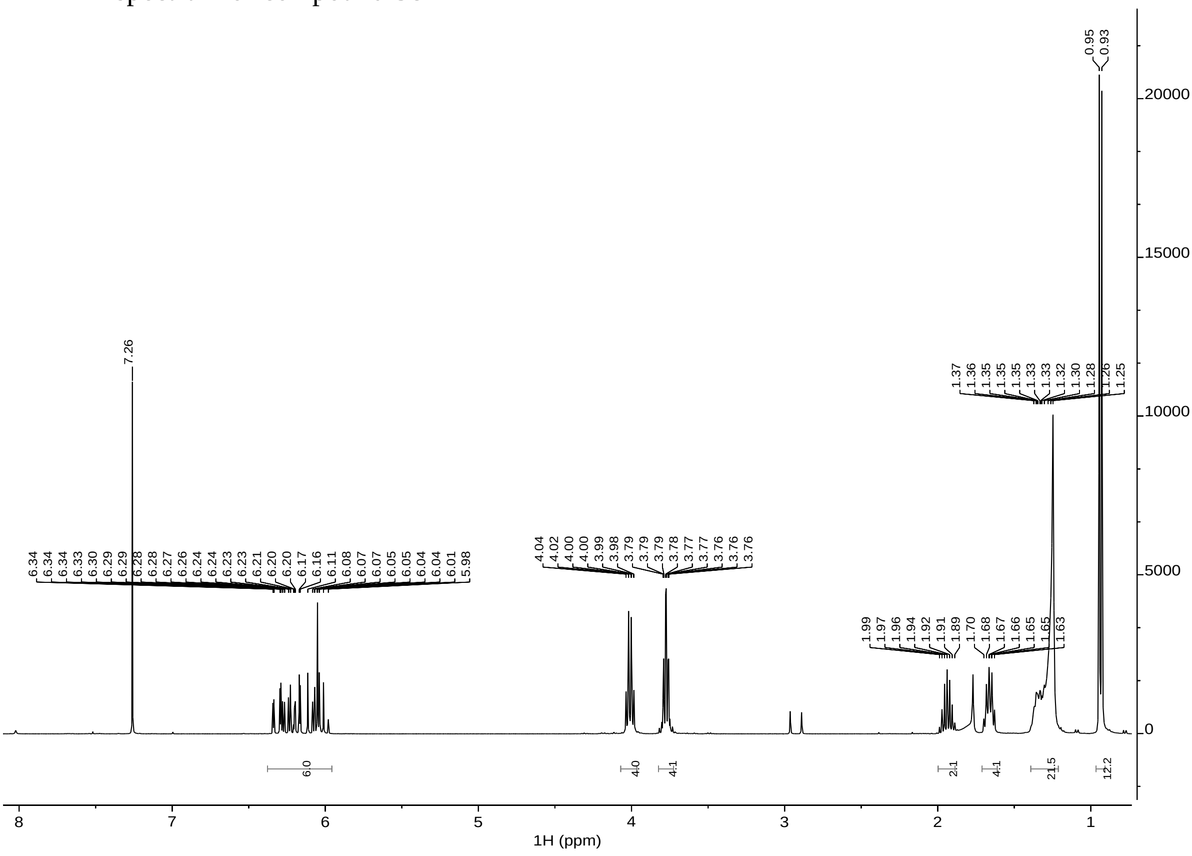

^{13}C NMR spectrum of compound **S87**

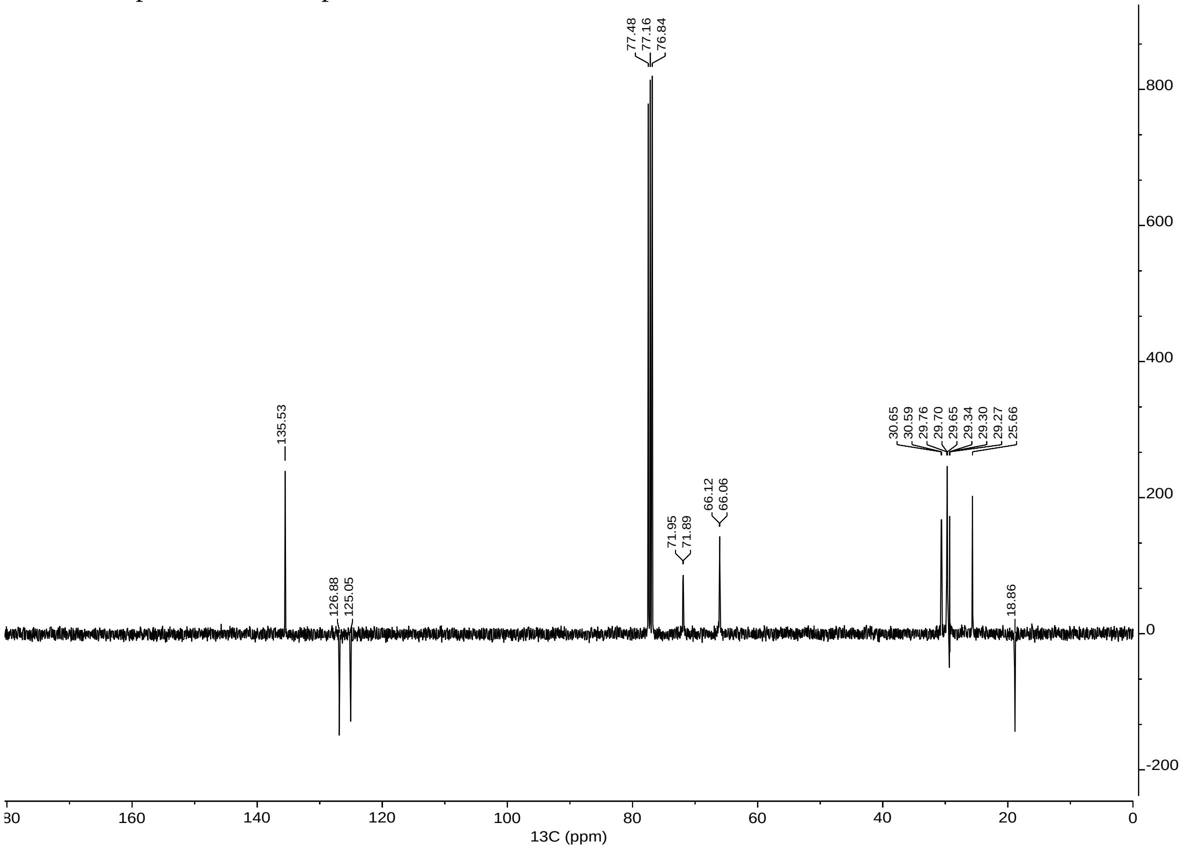

^{31}P{^{1}H} NMR spectrum of compound **S87**

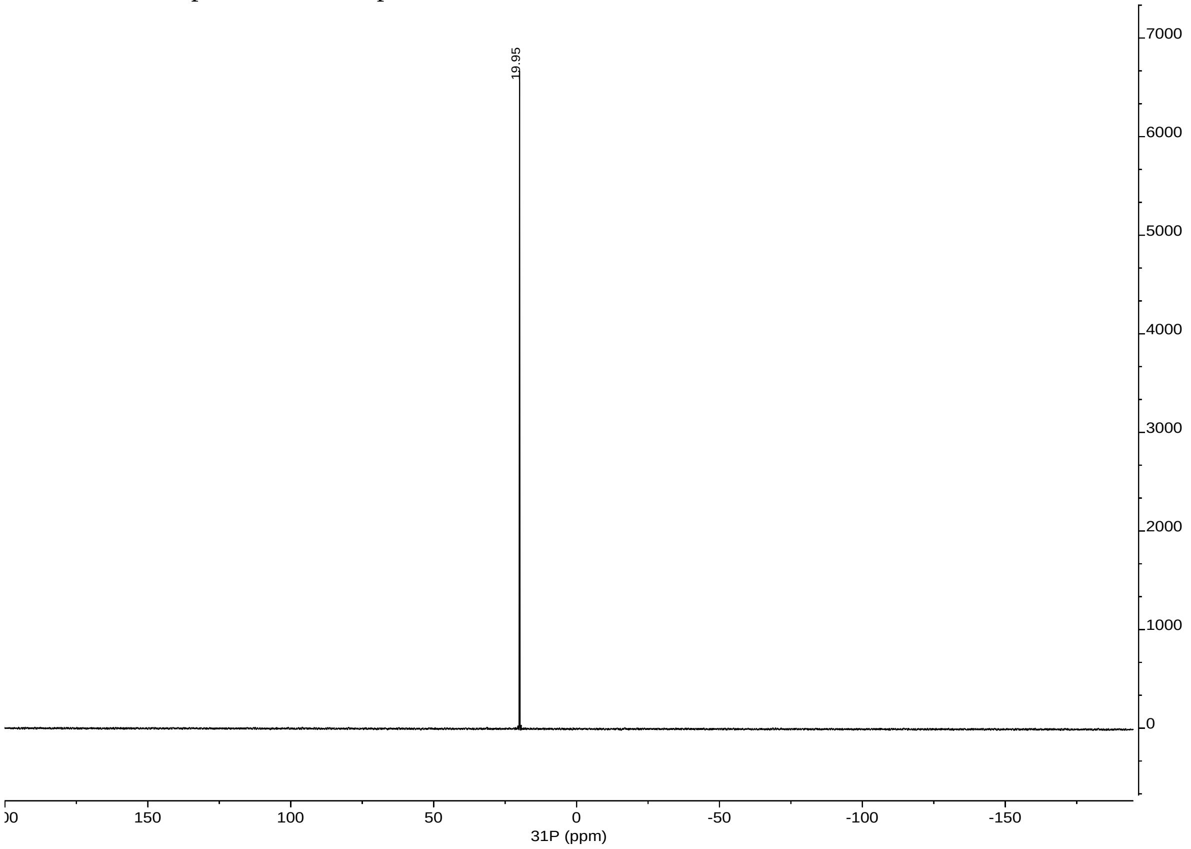

Dibutyl tetradecane-1,14-diyl bis(vinylphosphonate) (S88)

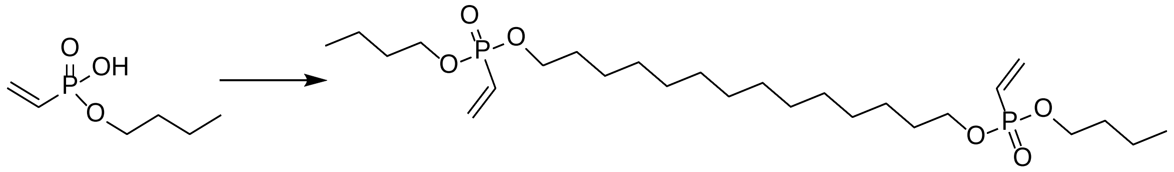

The title compound was prepared according to general method **C** from mono alkyl vinylphosphonate **S20** (1.50 g, 9.14 mmol) and 1,14-dibromotetradecane (1.09 g, 3.05 mmol) in 81% yield (1.29g, 2.47 mmol) as a colourless oil.

^{1}H NMR (401 MHz, $CDCl_3$): 6.33–5.96 (m, 6H, C*H*=C*H*$_2$), 4.04–3.96 (m, 8H, $CH_3(CH_2)_3$C*H*$_2$O, OC*H*$_2$$CH_2(CH_2)_{10}CH_2$C*H*$_2$O), 1.68–1.60 (m, 8H, CH_3CH_2C*H*$_2$$CH_2O, OCH_2C*H*_2$$(CH_2)_{10}$C*H*$_2$$CH_2$O), 1.43–1.23 (m, 24H, CH_3C*H*$_2$$CH_2CH_2$O, OCH_2CH_2(C*H*$_2$)$_{10}$$CH_2CH_2$O), 0.91 (t, H, *J* = 8.0 Hz, C*H*$_3$$(CH_2)_2CH_2$O).

^{13}C NMR (101 MHz, $CDCl_3$): 135.52 (d, *J* = 2.0 Hz, CH=*C*H$_2$), 125.97 (d, *J* = 184.1 Hz, *C*H=CH_2), 66.07, 65.75 (d, *J* = 6.1 Hz, $CH_3CH_2CH_2$*C*H$_2$O, O*C*H$_2$$CH_2(CH_2)_{10}CH_2$*C*H$_2$O), 32.58, 30.58 (d, *J* = 6.3 Hz, CH_3CH_2*C*H$_2$$CH_2$O, $OCH_2CH_2(CH_2)_{10}$*C*H$_2$$CH_2$O), 29.73, 29.67, 29.62, 29.27, 25.63, 18.85 (CH_3*C*H$_2$$CH_2CH_2$O, OCH_2CH_2(*C*H$_2$)$_{10}$$CH_2CH_2$O), 13.71 (*C*H$_3$$(CH_2)_2CH_2$O).

^{31}P{^{1}H} NMR (162 MHz, $CDCl_3$): 20.04.

IR ν_{max} (KBr) 3091 (w), 3015 (m), 2963 (m), 2931 (s), 2872 (m), 2856 (m), 1614 (w), 1467 (w, sh), 1466 (w), 1400 (m), 1277 (m, sh), 1240 (m), 1020 (s), 988 (s), 980 (m, sh), 857 (w), 606 (w).

HR-MS(ESI$^+$): For $C_{26}H_{52}O_6NaP_2$ $(M+Na)^+$ *m*/*z* calculated 545.31313, found 545.31335.

^{1}H NMR spectrum of compound **S88**

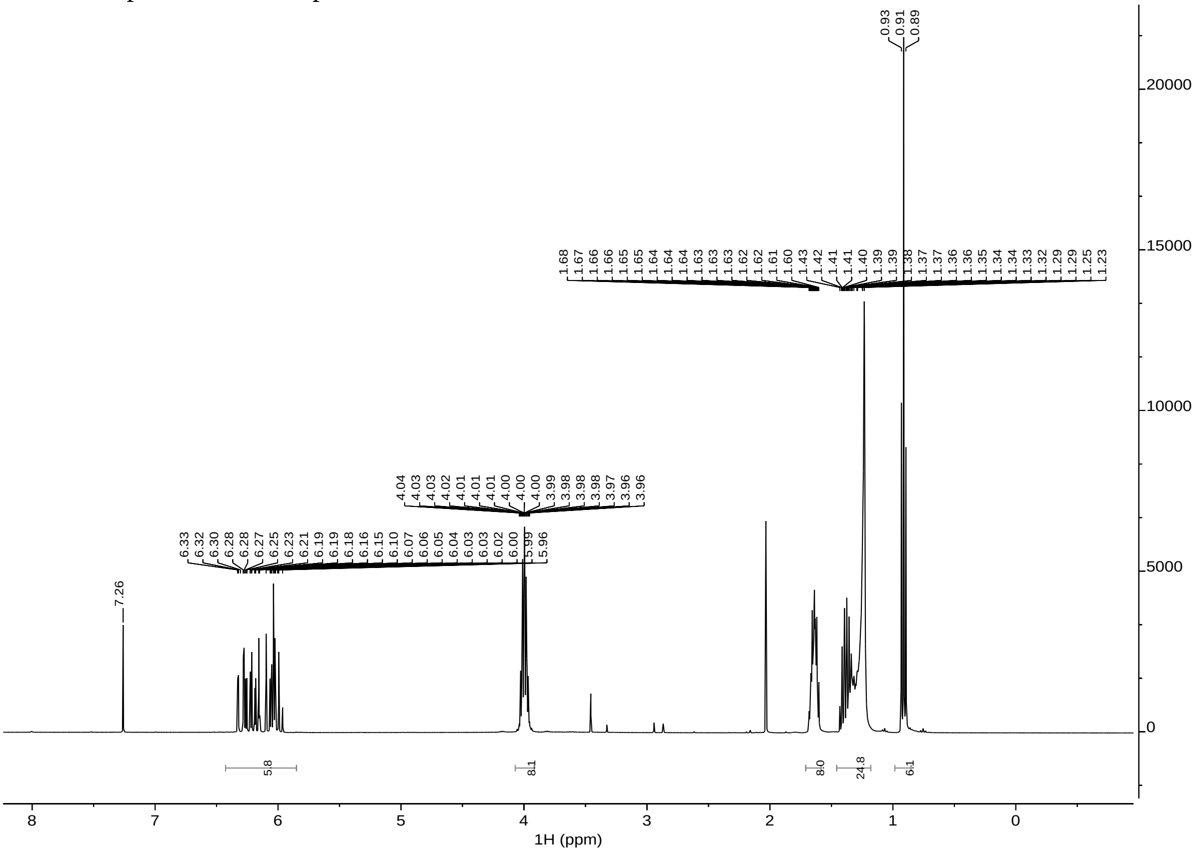

^{13}C NMR spectrum of compound **S88**

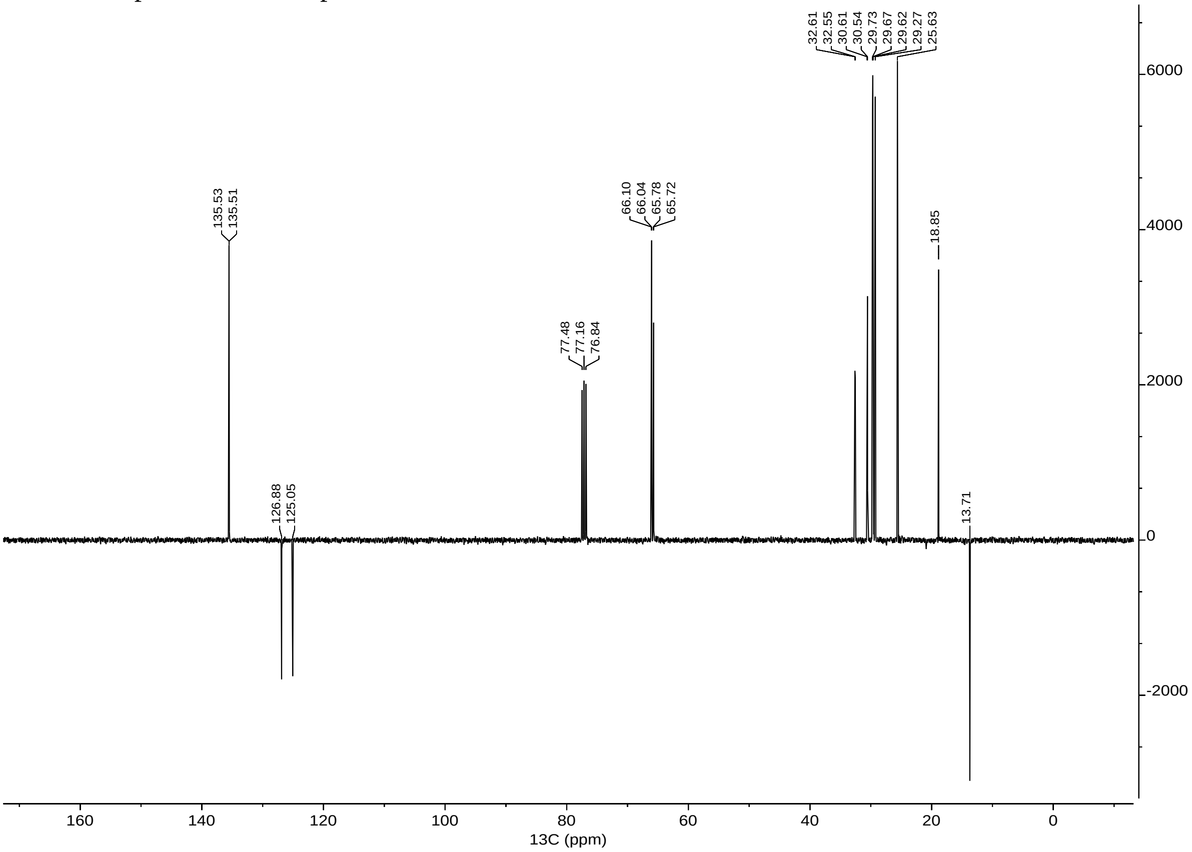

$^{31}P\{^{1}H\}$ NMR spectrum of compound **S88**

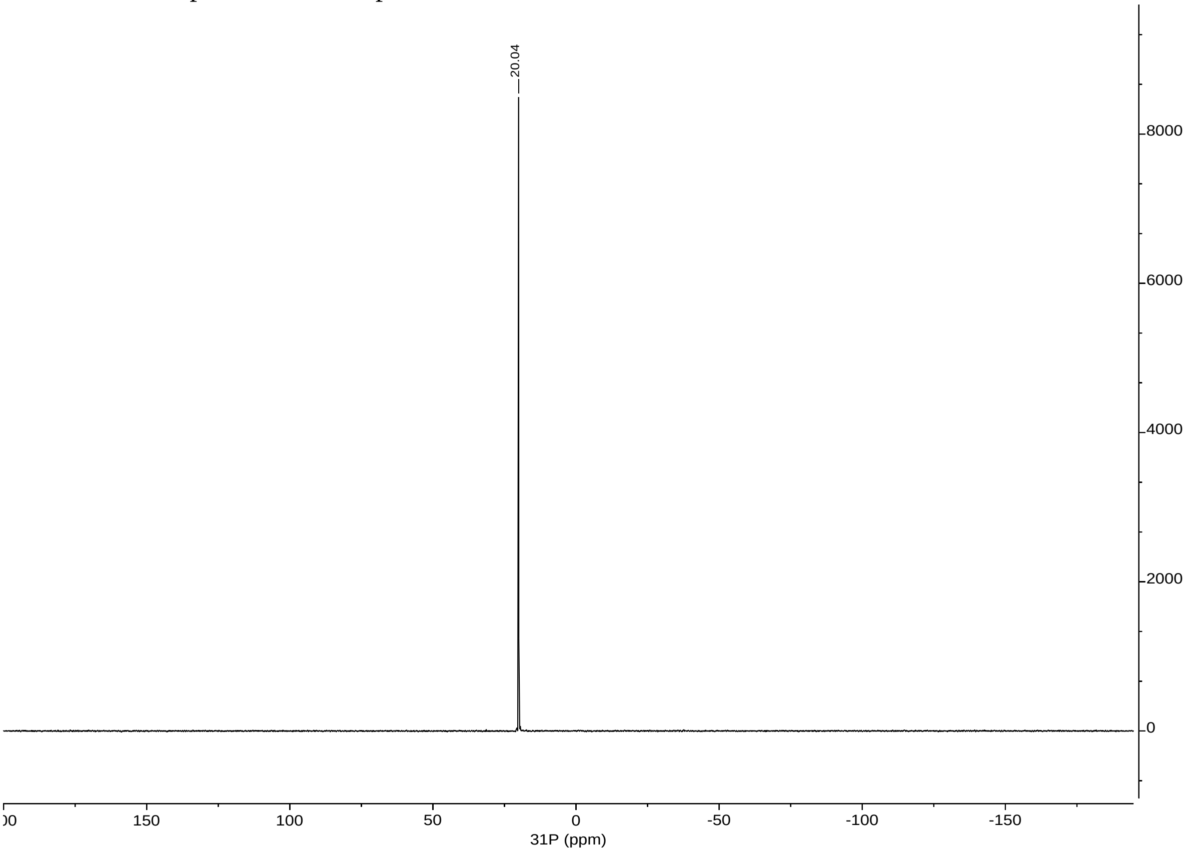

Dipentyl tetradecane-1,14-diyl bis(vinylphosphonate) (S89)

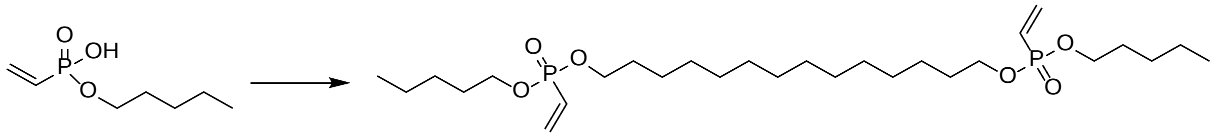

The title compound was prepared according to general method **C** from mono alkyl vinylphosphonate **S22** (1.50 g, 8.42 mmol) and 1,14-dibromotetradecane (1.0 g, 2.81 mmol) in 80% yield (1.24 g, 2.25 mmol) as a colourless oil.

^{1}H NMR (400 MHz, CD_3OD): 6.32–6.08 (m, 6H, C*H*=C*H*$_2$), 4.05–3.99 (m, 8H, $CH_3(CH_2)_3C\mathit{H}_2O$, $OC\mathit{H}_2CH_2(CH_2)_{10}CH_2C\mathit{H}_2O$), 1.73–1.65 (m, 8H, $CH_3(CH_2)_2C\mathit{H}_2CH_2O$, $OCH_2C\mathit{H}_2(CH_2)_{10}C\mathit{H}_2CH_2O$), 1.42–1.31 (m, 28H, $CH_3(C\mathit{H}_2)_2CH_2CH_2O$, $OCH_2CH_2(C\mathit{H}_2)_{10}CH_2CH_2O$), 0.96–0.91 (m, 6H, $C\mathit{H}_3(CH_2)_3CH_2O$).

^{13}C NMR (101 MHz, CD_3OD): 136.92 (d, J = 1.9 Hz, CH=*C*H$_2$), 126.44 (d, J = 185.1 Hz, *C*H=CH$_2$), 67.44 (d, J = 6.4 Hz, $CH_3(CH_2)_2CH_2\mathit{C}H_2O$, $O\mathit{C}H_2CH_2(CH_2)_{10}CH_2\mathit{C}H_2O$), 31.22, 31.49 (d, J = 6.4 Hz, $CH_3(CH_2)_2\mathit{C}H_2CH_2O$, $OCH_2\mathit{C}H_2(CH_2)_{10}\mathit{C}H_2CH_2O$), 30.75, 30.68, 30.64, 30.22, 28.84, 26.63, 23.27 ($CH_3(\mathit{C}H_2)_2CH_2CH_2O$, $OCH_2CH_2(\mathit{C}H_2)_{10}CH_2CH_2O$), 14.33 ($\mathit{C}H_3(CH_2)_3CH_2O$).

^{31}P{^{1}H} NMR (162 MHz, CD_3OD): 20.44.

IR ν_{max} (KBr) 3090 (vw), 2960 (s), 2930 (vs), 2873 (m), 2857 (s), 1467 (m), 1455 (w, sh), 1381 (w), 1279 (m), 1240 (s), 1050 (s), 1021 (vs, sh), 993 (vs).

HR-MS(ESI$^+$): For $C_{28}H_{56}O_6NaP_2$ (M+Na)$^+$ *m/z* calculated 573.34443, found 545.34422.

^{1}H NMR spectrum of compound **S89**

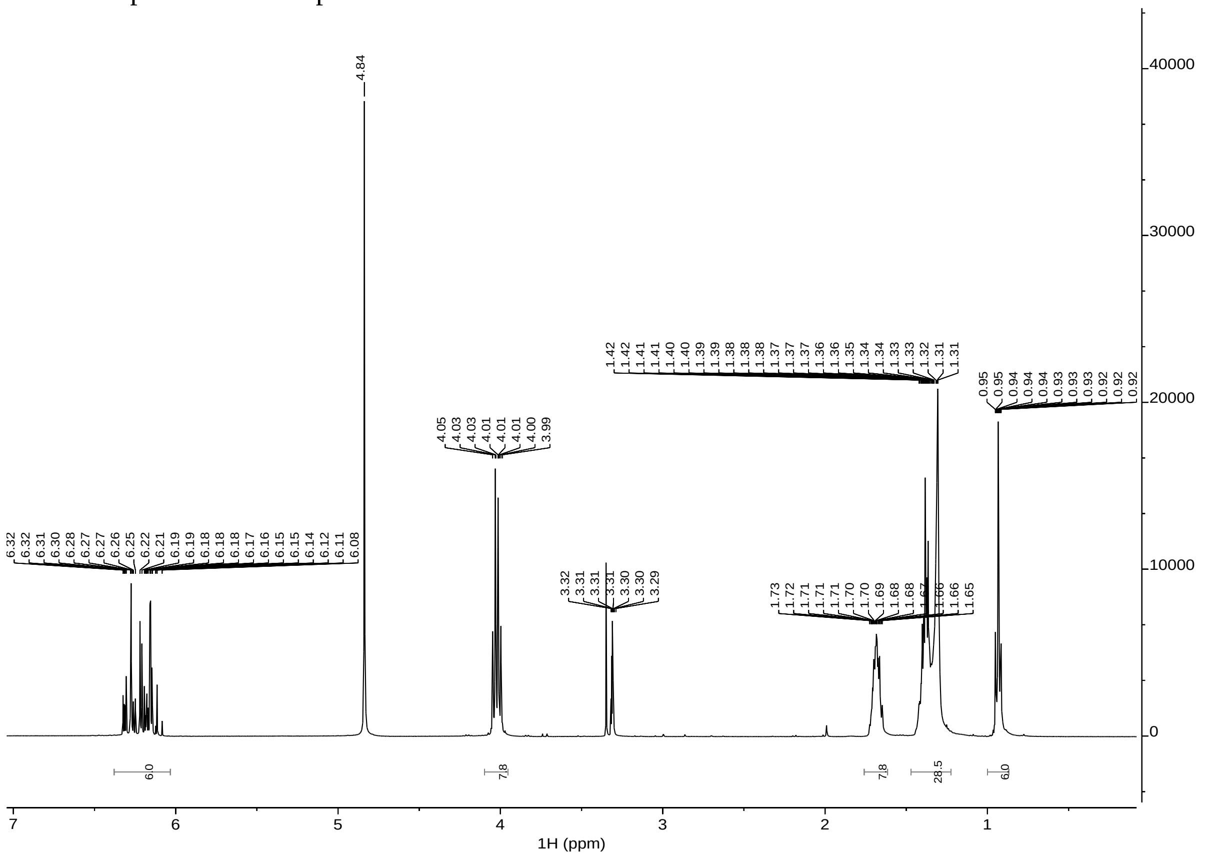

^{13}C NMR spectrum of compound **S89**

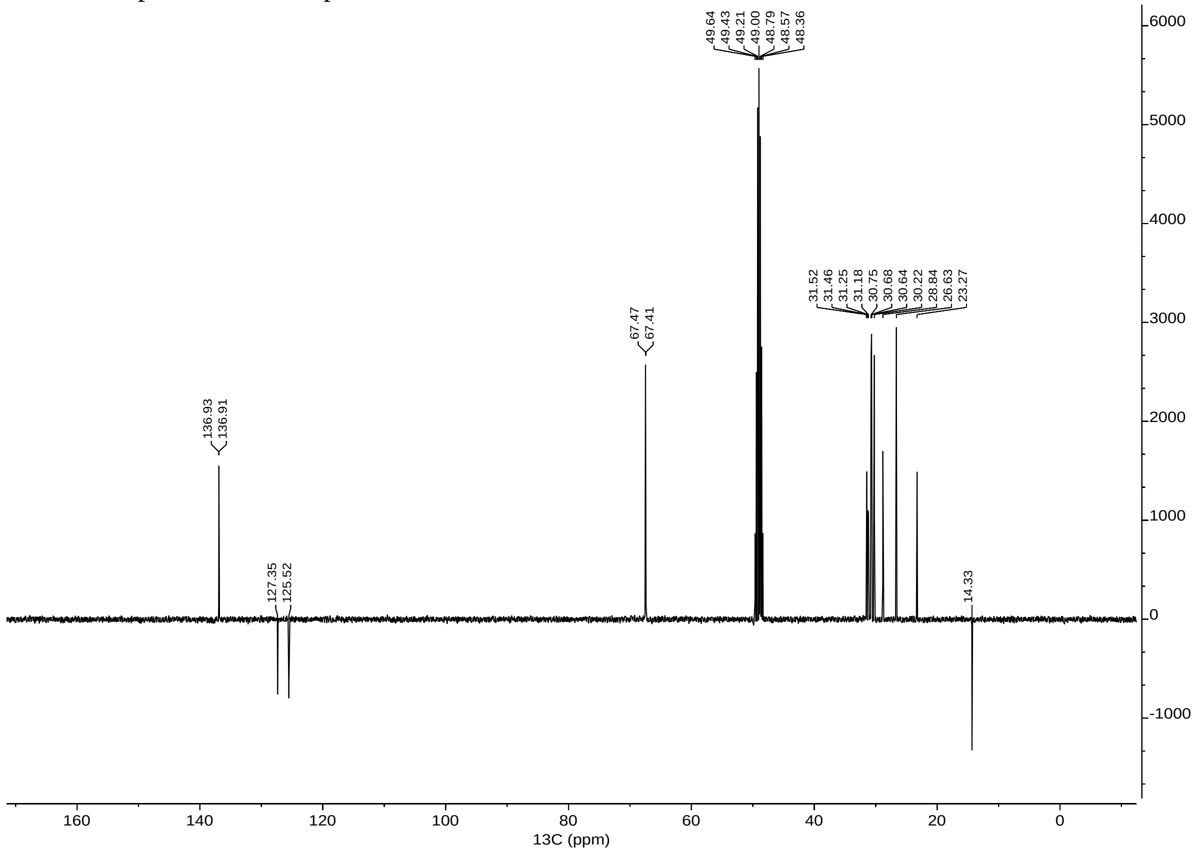

$^{31}P\{^{1}H\}$ NMR spectrum of compound **S89**

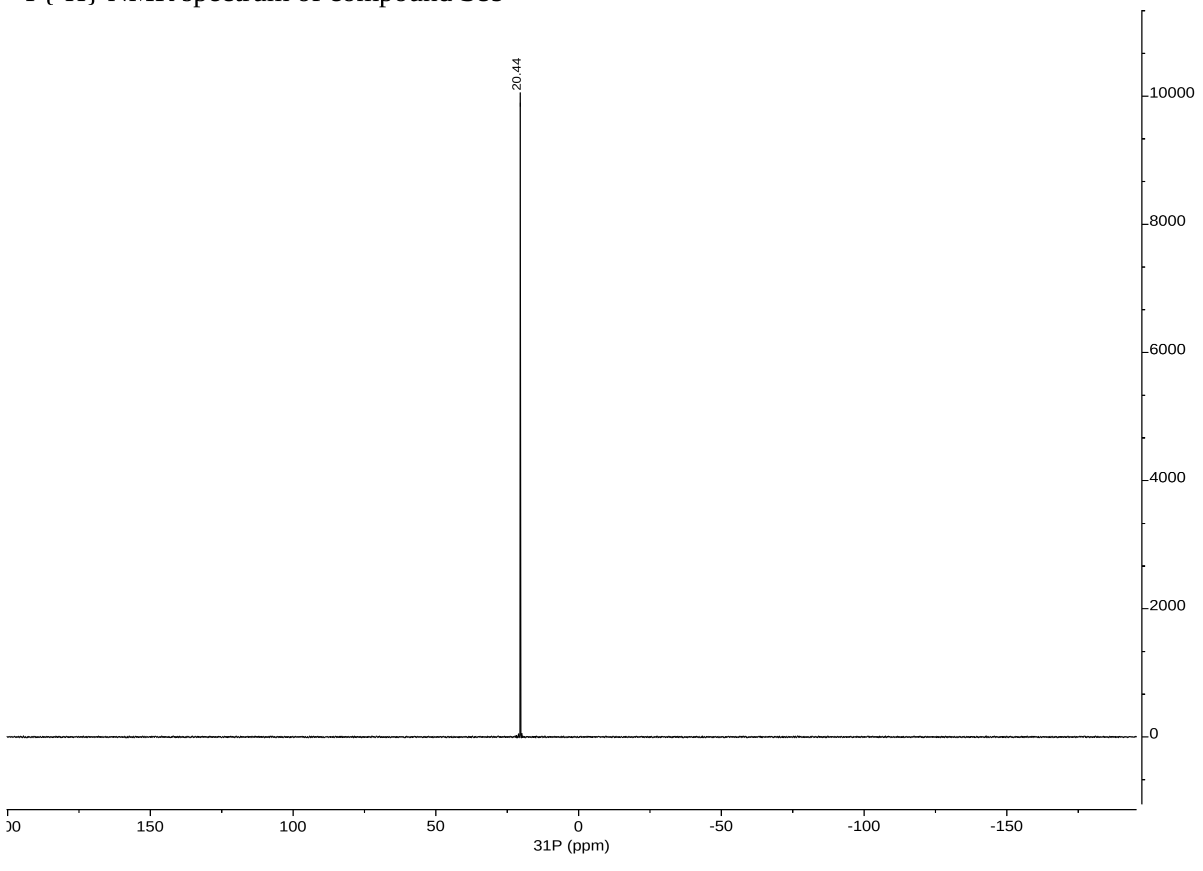

Dihexyl tetradecane-1,14-diyl bis(vinylphosphonate) (S90)

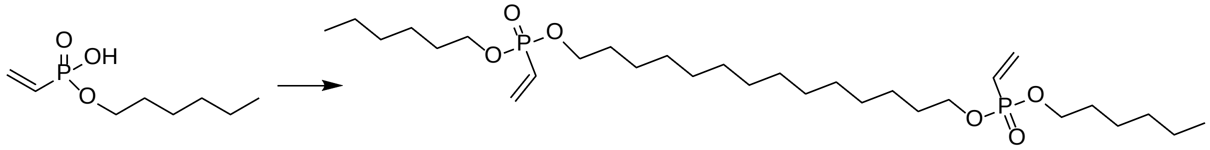

The title compound was prepared according to general method **C** from mono alkyl vinylphosphonate **S23** (1.0 g, 5.20 mmol) and 1,14-dibromotetradecane (0.62 g, 1.74 mmol) in 85% yield (0.86 g, 1.47 mmol) as a colourless oil.

^{1}H NMR (400 MHz, $CDCl_3$): 6.33–5.97 (m, 6H, $C\mathit{H}{=}C\mathit{H}_2$), 4.04–3.95 (m, 8H, $CH_3(CH_2)_4C\mathit{H}_2O$, $OC\mathit{H}_2CH_2(CH_2)_{10}CH_2C\mathit{H}_2O$), 1.69–1.62 (m, 8H, $CH_3(CH_2)_3C\mathit{H}_2CH_2O$, $OCH_2C\mathit{H}_2(CH_2)_{10}C\mathit{H}_2CH_2O$), 1.39–1.24 (m, 32H, $CH_3(C\mathit{H}_2)_3CH_2CH_2O$, $OCH_2CH_2(C\mathit{H}_2)_{10}CH_2CH_2O$), 0.90–0.86 (m, 6H, $C\mathit{H}_3(CH_2)_4CH_2O$).

^{13}C NMR (101 MHz, $CDCl_3$): 135.45 (d, J = 1.9 Hz, $CH{=}\mathit{C}H_2$), 126.09 (d, J = 184.1 Hz, $\mathit{C}H{=}CH_2$), 66.05 (d, J = 6.0 Hz, $CH_3(CH_2)_3CH_2\mathit{C}H_2O$, $O\mathit{C}H_2CH_2(CH_2)_{10}CH_2\mathit{C}H_2O$), 30.68, 30.62 (d, J = 6.4 Hz $CH_3(CH_2)_3\mathit{C}H_2CH_2O$, $OCH_2\mathit{C}H_2(CH_2)_{10}\mathit{C}H_2CH_2O$), 31.48, 29.76, 29.71, 29.66, 29.31, 25.67, 25.33, 22.66 ($CH_3(\mathit{C}H_2)_3CH_2CH_2O$, $OCH_2CH_2(\mathit{C}H_2)_{10}CH_2CH_2O$), 14.12 ($\mathit{C}H_3(CH_2)_4CH_2O$).

^{31}P{^{1}H} NMR (162 MHz, $CDCl_3$): 19.74.

IR ν_{max} (KBr) 3090 (vw), 2958 (s), 2930 (vs), 2871 (m), 2857 (s), 1614 (w), 1468 (m), 1458 (w), 1434 (w), 1400 (m), 1380 (w), 1279 (w), 1241 (s), 1058 (s), 1038 (s), 1010 (vs), 998 (vs), 861 (w), 606 (w).

HR-MS(ESI^{+}): For $C_{30}H_{61}O_6P_2$ $(M+H)^+$ m/z calculated 579.39379, found 579.39286.

^{1}H NMR spectrum of compound **S90**

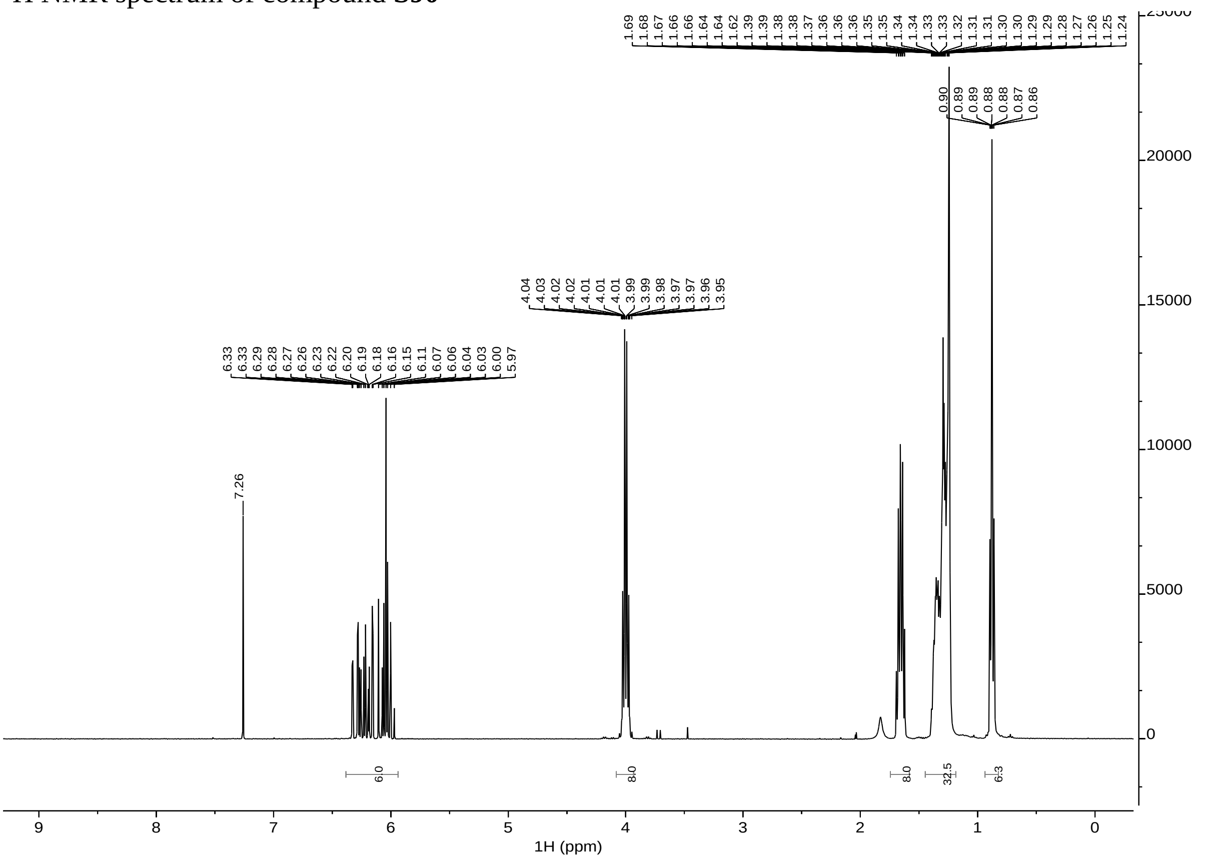

^{13}C NMR spectrum of compound **S90**

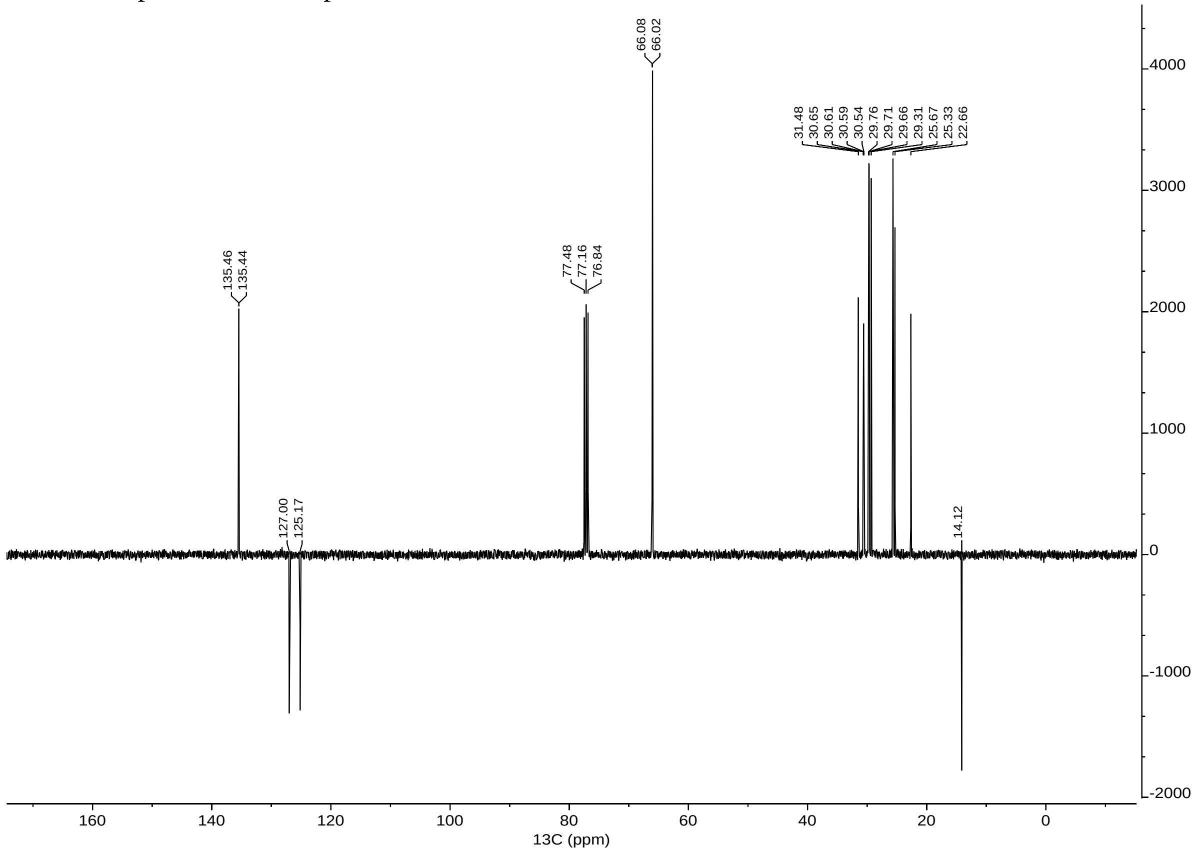

^{31}P{^{1}H} NMR spectrum of compound **S90**

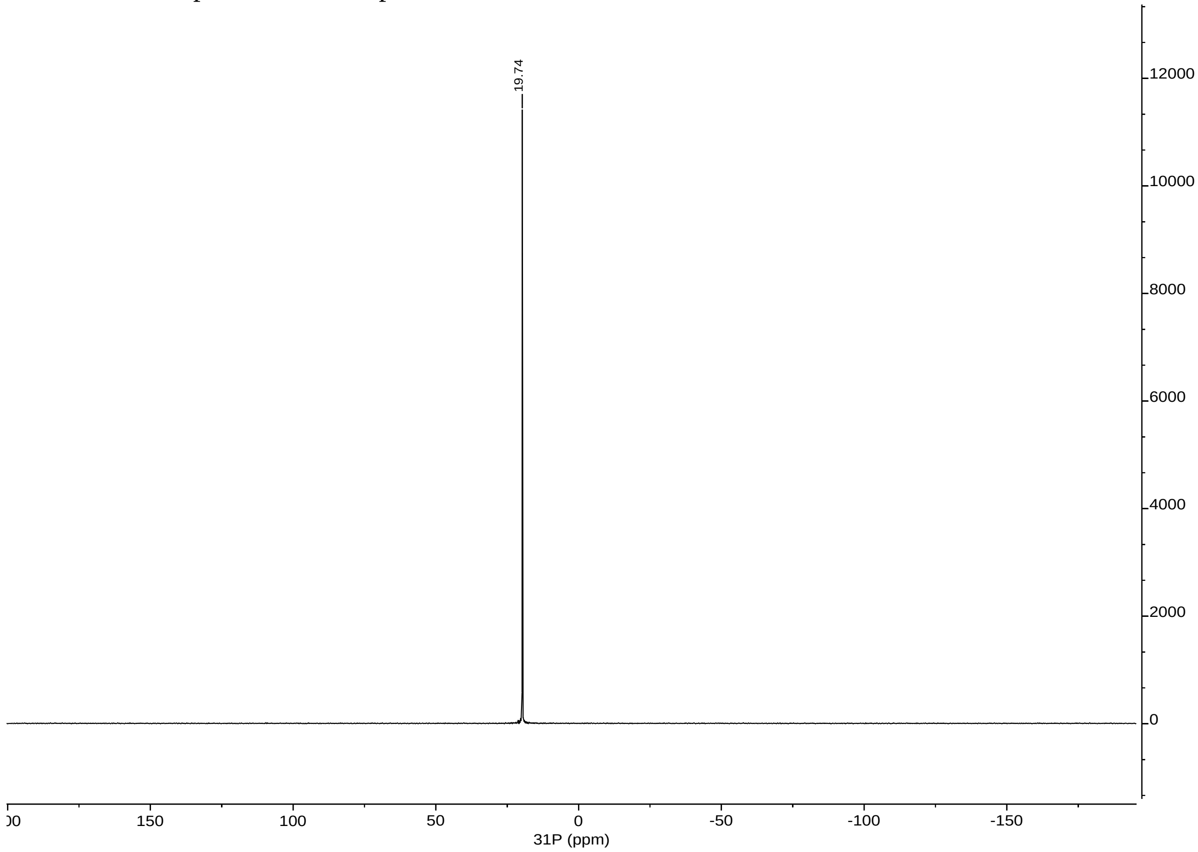

***N,N',N'',N'''*-Tetrakis(*tert*-butyloxycarbonyl) bis((*Z*)-hept-3-en-1-yl) propane-1,3-diyl bis((2-(bis(3-aminopropyl)amino)ethyl)phosphonate) (S91)**

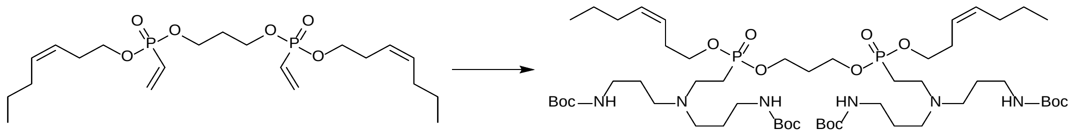

The title compound was prepared according to general method **D** from vinylphosphonate dimer **S38** (0.58 g, 1.29 mmol) and bis(3-*tert*-butyloxycarbonylaminopropyl)amine (1.70 g, 5.16 mmol) in 57% yield (0.82 g, 0.74 mmol) as a colourless oil.

^{1}H NMR (400 MHz, CD_3OD): 5.59–5.52 (m, 2H, $CH_3(CH_2)_2C\mathit{H}CHCH_2CH_2O$), 5.45–5.39 (m, 2H, $CH_3(CH_2)_2CHC\mathit{H}CH_2CH_2O$), 4.17 (p, 4H, J = 5.9 Hz, $OC\mathit{H}_2CH_2C\mathit{H}_2O$), 4.06 (p, 4H, J = 6.7 Hz, $CH_3(CH_2)_2CH_2CHCHCH_2C\mathit{H}_2O$), 3.08 (t, 8H, J = 6.7 Hz, $NCH_2CH_2C\mathit{H}_2NH$), 2.78 (q, 4H, J = 8.3 Hz, $PCH_2C\mathit{H}_2N$), 2.49–2.43 (m, 12H, $NC\mathit{H}_2CH_2CH_2NH$, $CH_3(CH_2)_2CH_2CHCHC\mathit{H}_2CH_2O$), 2.10–1.98 (m, 10H, $CH_3CH_2C\mathit{H}_2CHCHCH_2CH_2O$, $PC\mathit{H}_2CH_2N$, $OCH_2C\mathit{H}_2CH_2O$), 1.63 (p, 8H, J = 6.8 Hz, $NCH_2C\mathit{H}_2CH_2NH$), 1.44–1.38 (m, 40H, $C(C\mathit{H}_3)_3$, $CH_3C\mathit{H}_2CH_2CHCHCH_2CH_2O$), 0.93 (t, 6H, J = 7.4 Hz, $C\mathit{H}_3(CH_2)_2CHCHCH_2CH_2O$).

^{13}C NMR (101 MHz, CD_3OD): 158.46 (N*C*OO), 133.99 ($CH_3(CH_2)_3\mathit{C}HCHCH_2CH_2O$), 125.53 ($CH_3(CH_2)_2CH\mathit{C}HCH_2CH_2O$), 79.86 ($\mathit{C}(CH_3)_3$, 66.86 (d, J = 7.8 Hz, $CH_3CH_2CH_2CHCHCH_2\mathit{C}H_2O$), 63.24 (d, J = 6.1 Hz, $O\mathit{C}H_2CH_2\mathit{C}H_2O$), 51.86 ($N\mathit{C}H_2CH_2CH_2NH$), 47.69 ($PCH_2\mathit{C}H_2N$), 39.64 ($NCH_2CH_2\mathit{C}H_2NH$), 32.59 (t, J = 7.4 Hz, $OCH_2\mathit{C}H_2CH_2O$), 30.47 ($CH_3CH_2\mathit{C}H_2CHCHCH_2CH_2O$), 29.74 (d, J = 6.0 Hz, $CH_3CH_2CH_2CHCH\mathit{C}H_2CH_2O$), 28.86 ($C(\mathit{C}H_3)_3$), 28.17 ($NCH_2\mathit{C}H_2CH_2NH$), 23.81 ($CH_3\mathit{C}H_2CH_2CHCHCH_2CH_2O$), 22.82 (d, J = 138.2 Hz, $P\mathit{C}H_2CH_2N$), 14.18 ($\mathit{C}H_3CH_2$).

^{31}P{^{1}H} NMR (162 MHz, CD_3OD): 34.74.

IR ν_{max} (KBr) 3328 (m), 3005 (w, sh), 2964 (m), 2931 (m), 2871 (m), 2815 (w), 1712 (vs), 1693 (s), 1524 (s), 1456 (m), 1391 (m), 1365 (s), 1250 (s), 1068–1012 (m), 721 (w), 464 (w).

HR-MS(ESI^{+}): For $C_{53}H_{106}O_{14}N_6P_2$ $(M+H)^{+}$ m/z calculated 556.36156, found 556.36212.

^{1}H NMR spectrum of compound **S91**

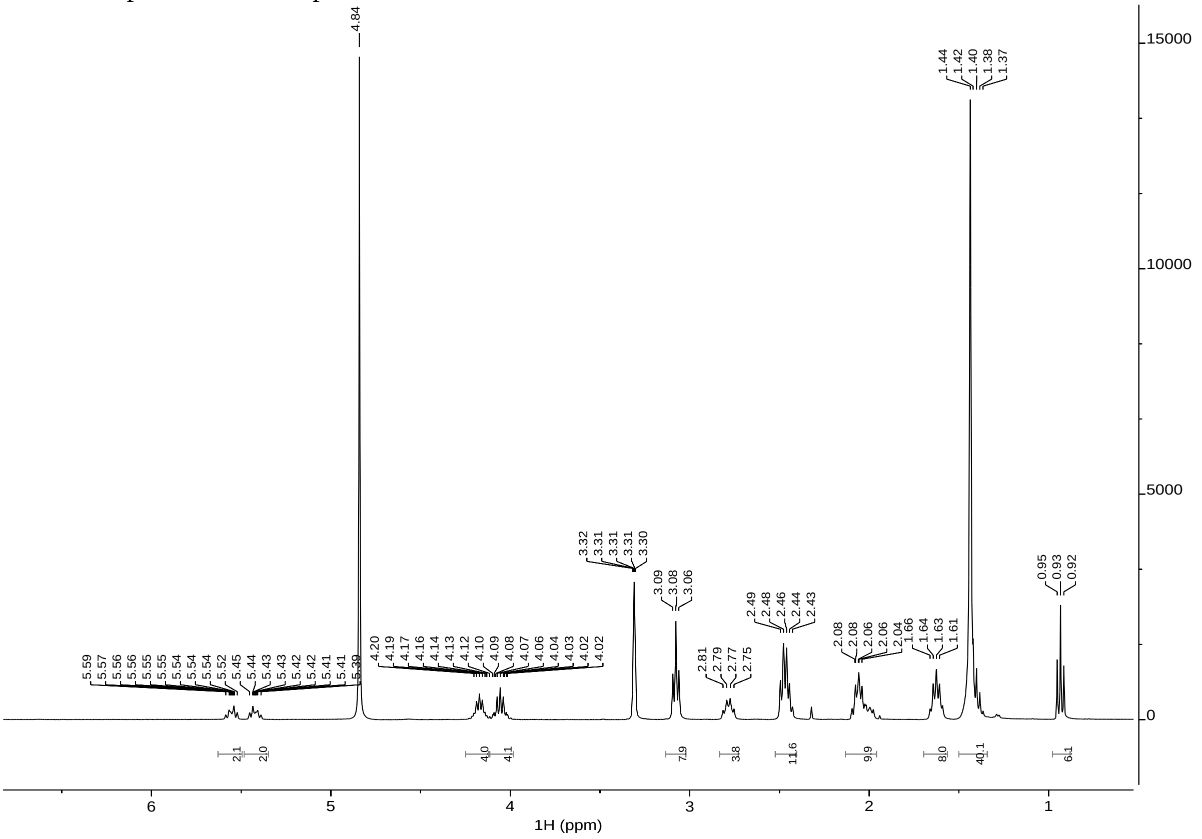

^{13}C NMR spectrum of compound **S91**

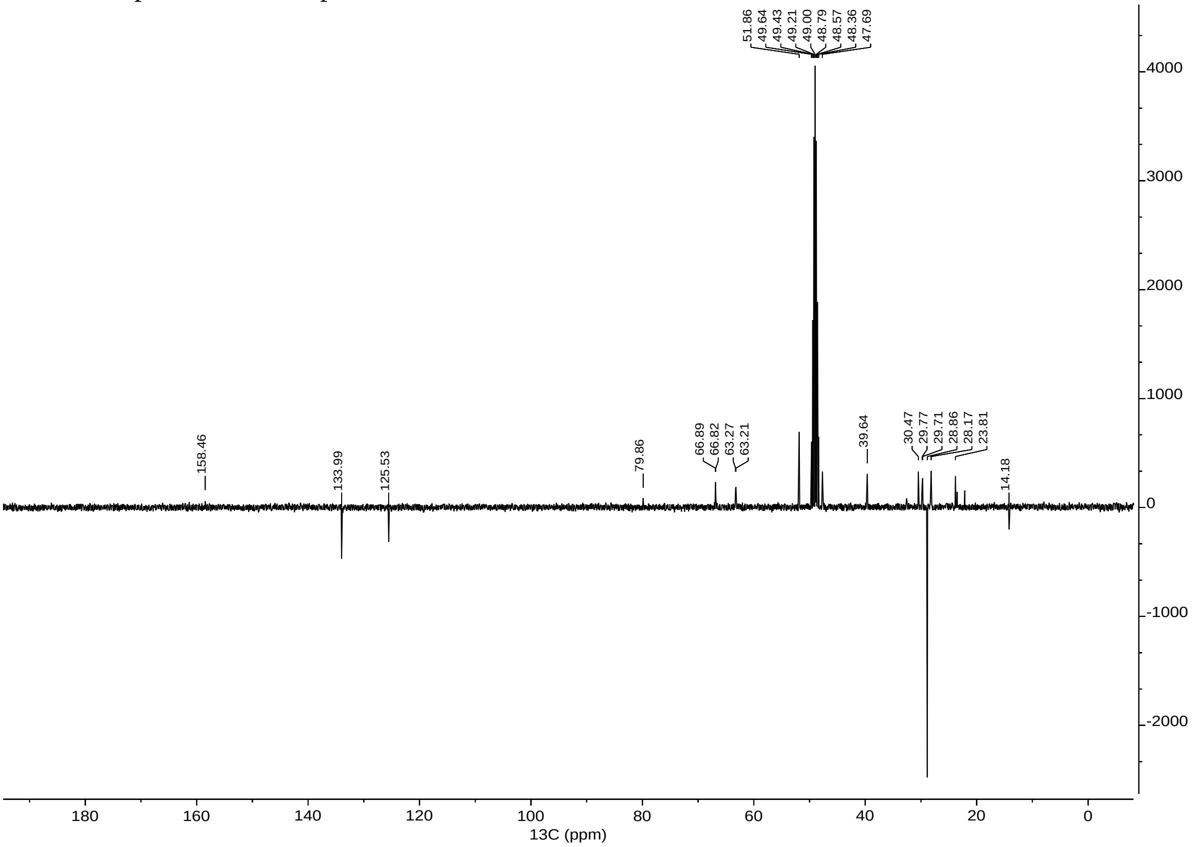

$^{31}P\{^{1}H\}$ NMR spectrum of compound **S91**

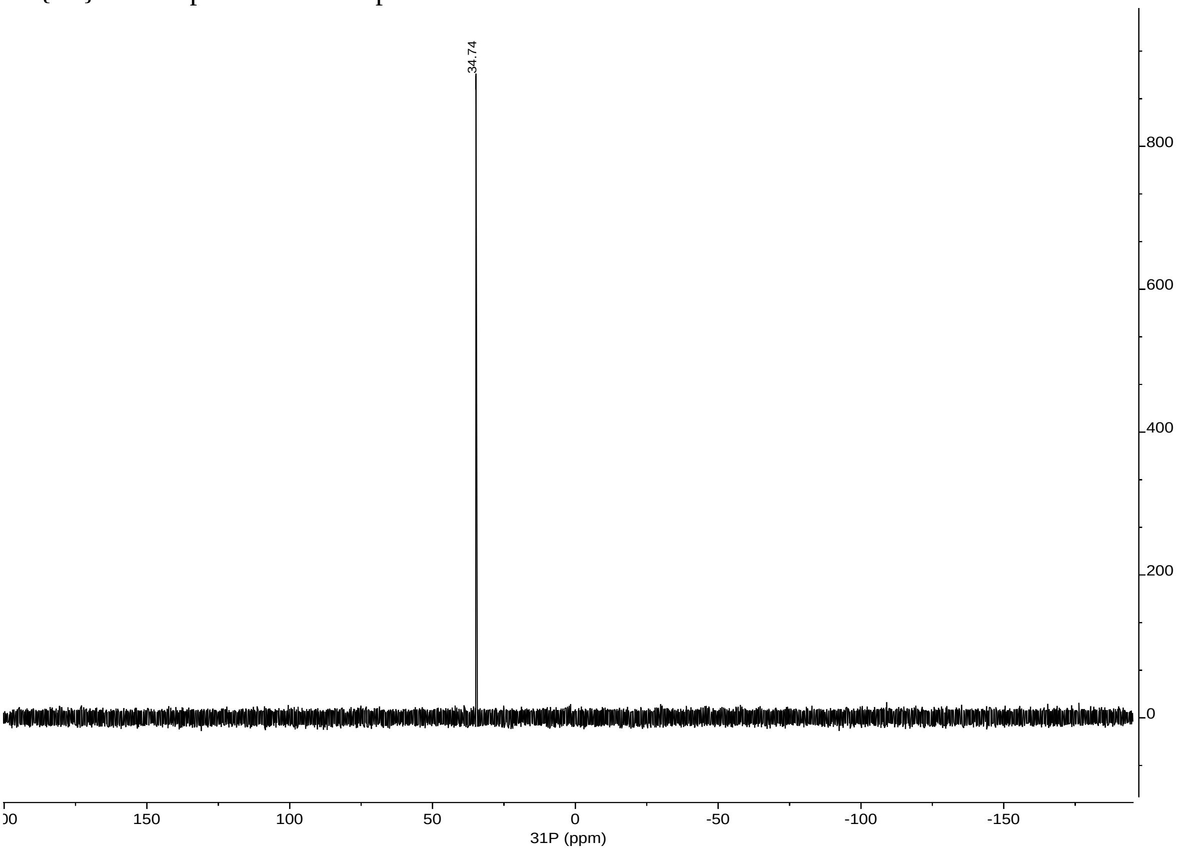

***N,N',N'',N'''*-Tetrakis(*tert*-butyloxycarbonyl) bis((*Z*)-hept-4-en-1-yl) propane-1,3-diyl bis((2-(bis(3-aminopropyl)amino)ethyl)phosphonate) (S92)**

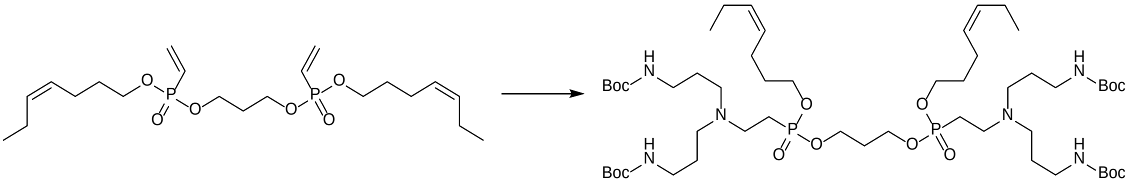

The title compound was prepared according to general method **D** from vinylphosphonate dimer **S39** (0.91 g, 2.75 mmol) and bis(3-*tert*-butyloxycarbonylaminopropyl)amine (0.35 g, 0.79 mmol) in 52% yield (0.46 g, 0.41 mmol) as a colourless oil.

^{1}H NMR (401 MHz, CD_3OD): 5.48–5.41 (m, 2H, $CH_3CH_2C\mathit{H}CH$), 5.38–5.31 (m, 2H, $CH_3CH_2CHC\mathit{H}$), 4.22–4.14 (m, 4H, $OC\mathit{H_2}CH_2C\mathit{H_2}O$), 4.11–4.03 (m, 4H, $CHCH(CH_2)_2C\mathit{H_2}O$), 3.08 (t, 8H, J = 6.7 Hz, $NCH_2CH_2C\mathit{H_2}NH$), 2.82–2.76 (m, 4H, $PCH_2C\mathit{H_2}N$), 2.48 (dd, J = 8.2, 6.3 Hz, 8H, $NC\mathit{H_2}(CH_2)_2NH$), 2.20–2.14 (m, 4H, $CHCHC\mathit{H_2}(CH_2)_2O$), 2.11–1.99 (m, 10H, $PC\mathit{H_2}$, $OCH_2C\mathit{H_2}CH_2O$, $CH_3C\mathit{H_2}$), 1.78–1.71 (m, 4H, $CHCHCH_2C\mathit{H_2}CH_2O$), 1.66–1.61 (m, 8H, $NCH_2C\mathit{H_2}CH_2NH$), 1.44 (s, 36H, $C(C\mathit{H_3})_3$), 0.98 (t, 6H, J = 7.4 Hz, $C\mathit{H_3}CH_2$).

^{13}C NMR (101 MHz, CD_3OD): 158.48 (N*C*OO), 133.83 ($CH_3CH_2\mathit{C}HH$), 128.57 ($CH_3CH_2CH\mathit{C}H$), 79.87 ($\mathit{C}(CH_3)_3$), 66.77 (d, J = 7.0 Hz, $CH(CH_2)_2\mathit{C}H_2O$), 63.26 (d, J = 6.6 Hz, $O\mathit{C}H_2CH_2\mathit{C}H_2O$), 51.84 ($N\mathit{C}H_2CH_2CH_2NH$), 47.71 ($PCH_2\mathit{C}H_2$), 39.62 ($\mathit{C}H_2NH$), 32.60 (t, J = 6.1 Hz, $OCH_2\mathit{C}H_2CH_2O$), 31.70 (d, J = 6.3 Hz, $CHCH_2\mathit{C}H_2CH_2O$), 28.15 ($\mathit{C}H_2CH_2NH$), 28.86 ($C(\mathit{C}H_3)_3$, 24.11 ($CH\mathit{C}H_2(CH_2)_2O$), 22.76 (d, J = 136.8 Hz, $P\mathit{C}H_2$), 21.52 ($CH_3\mathit{C}H_2$), 14.77 ($\mathit{C}H_3CH_2$).

^{31}P{^{1}H} NMR (162 MHz, CD_3OD): 34.98.

IR ν_{max} (KBr) 3455 (m), 3363 (w), 2979 (s), 2935 (s), 2874 (m), 2823 (m), 1706 (vs), 1507 (s), 1467 (m), 1456 (m), 1393 (m), 1367 (s), 1250 (s), 1080–970 (m), 462 (w).

HR-MS(ESI^{+}): For $C_{53}H_{104}O_{14}N_6NaP_2$ $(M+Na)^{+}$ *m/z* calculated 1133.69780, found 1133.69702.

^{1}H NMR spectrum of compound **S92**

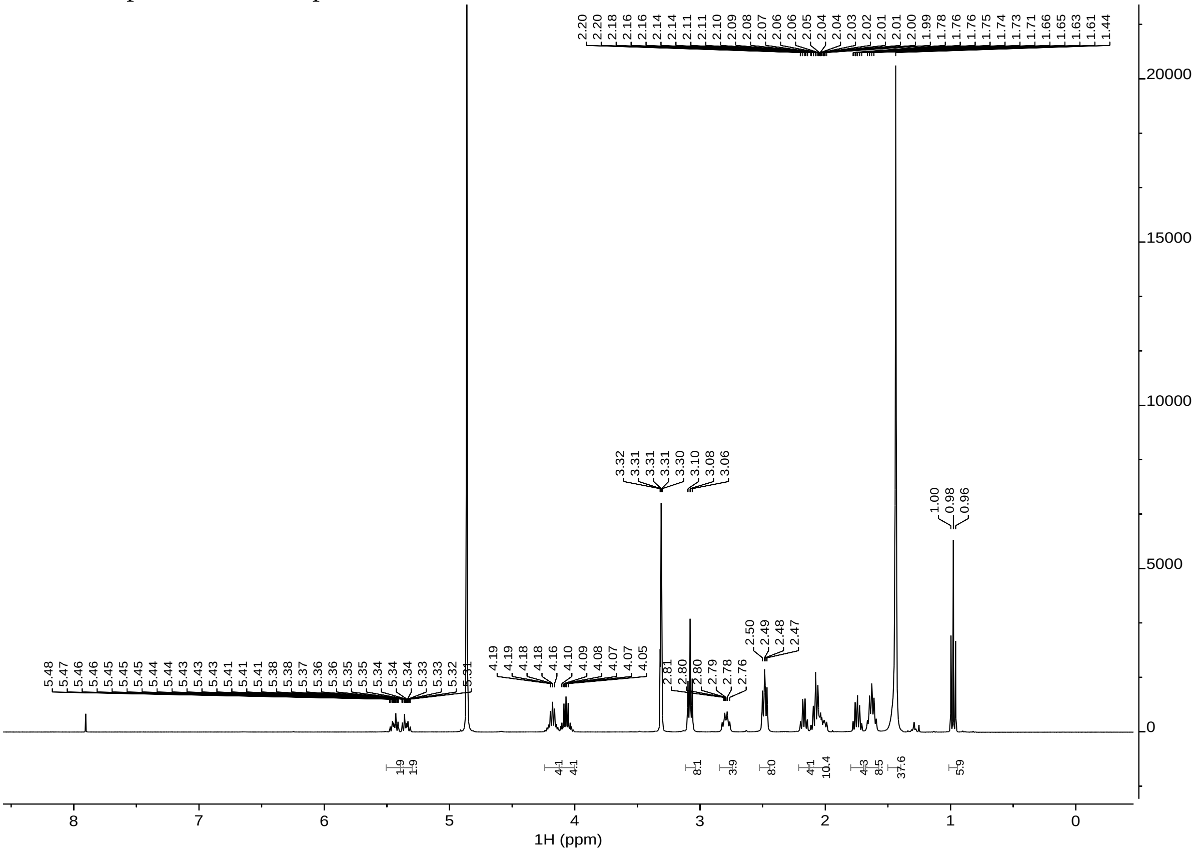

^{13}C NMR spectrum of compound **S92**

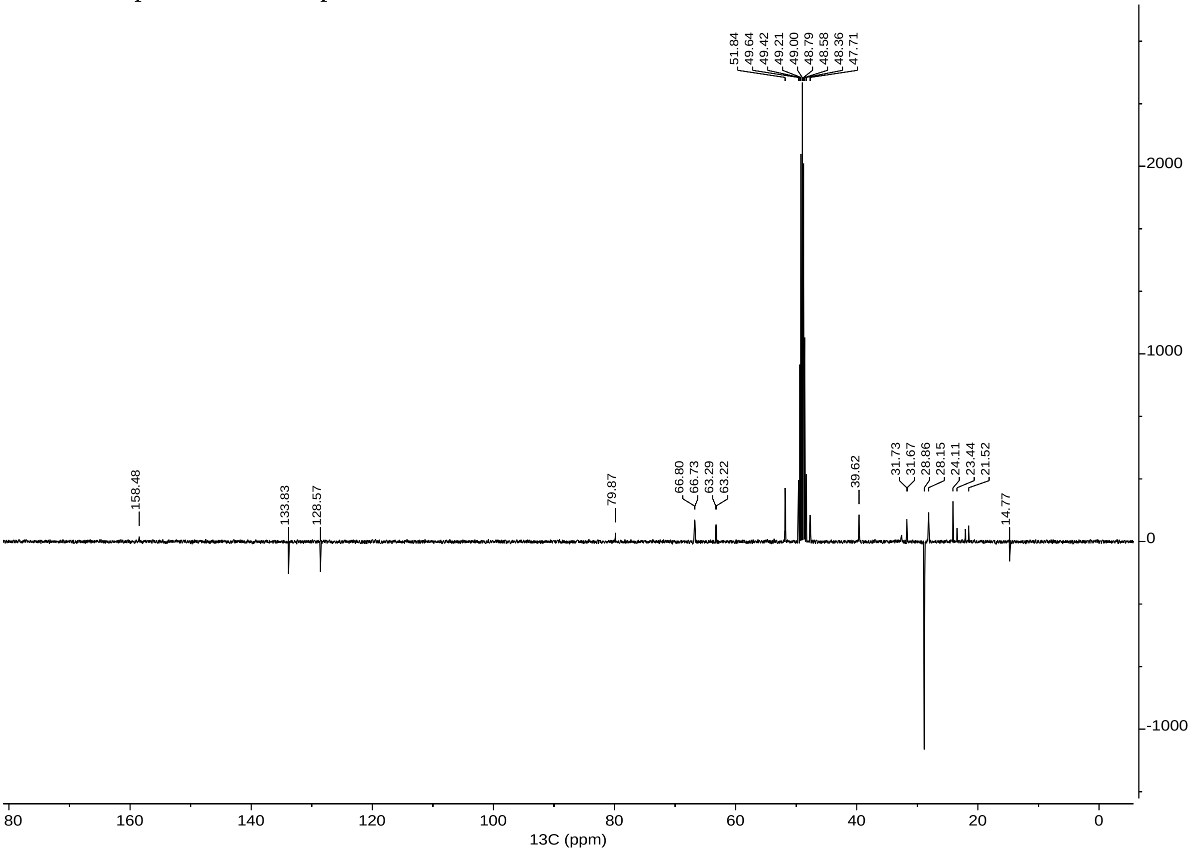

^{31}P{^{1}H} NMR spectrum of compound **S92**

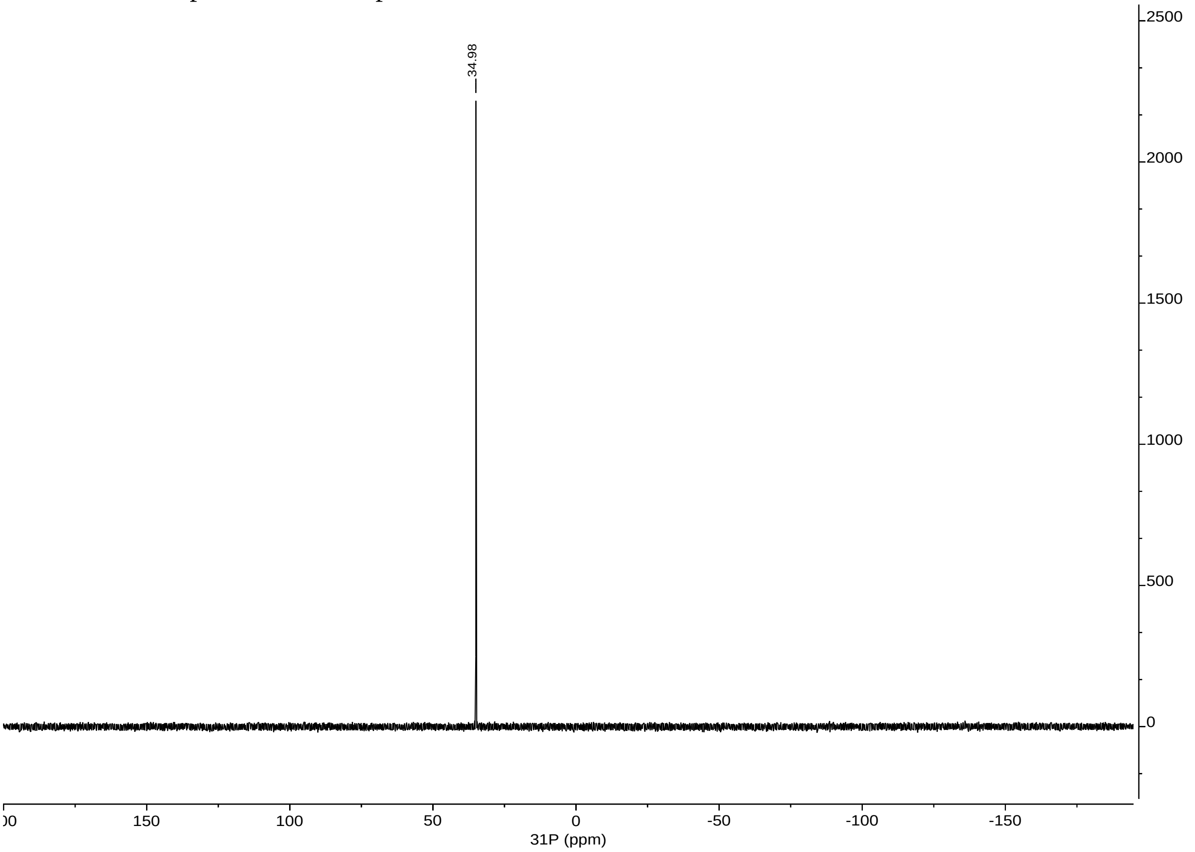

***N,N',N'',N'''*-Tetrakis(*tert*-butyloxycarbonyl) dioctyl propane-1,3-diyl bis((2-(bis(3-aminopropyl)amino)ethyl)phosphonate) (S93)**

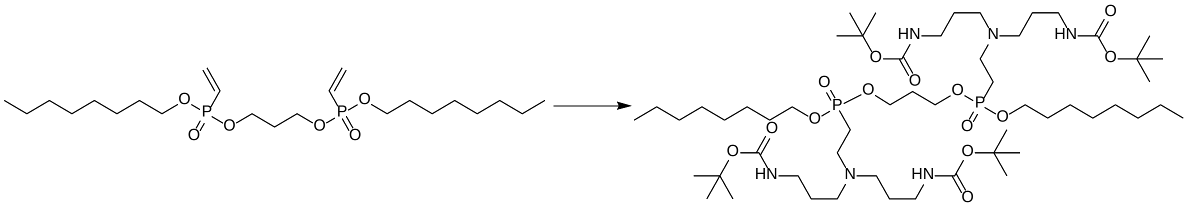

The title compound was prepared according to general method **D** from vinylphosphonate dimer **S40** (0.58 g, 1.21 mmol) and bis(3-*tert*-butyloxycarbonylaminopropyl)amine (1.80 g, 5.43 mmol) in 52% yield (0.72 g, 0.63 mmol) as a colourless oil.

^{1}H NMR (401 MHz, CD_3OD): 4.21–4.13 (m, 4H, $OCH_2CH_2CH_2O$), 4.11–4.00 (m, 4H, $CH_3(CH_2)_5CH_2CH_2O$), 3.08 (t, 8H, J = 6.7 Hz $NCH_2CH_2CH_2NH$), 2.81–2.75 (m, 4H, PCH_2CH_2N), 2.48 (t, 8H, J = 8.0 Hz, $NCH_2CH_2CH_2NH$), 2.09–1.98 (m, 6H, PCH_2CH_2N, $OCH_2CH_2CH_2O$), 1.73–1.61 (m, 12H, $CH_3(CH_2)_5CH_2CH_2O$, $NCH_2CH_2CH_2NH_2$), 1.44–1.29 (m, 56H, $CH_3(CH_2)_5CH_2CH_2O$, $C(CH_3)_3$), 0.93–0.89 (m, 6H, CH_3CH_2).

^{13}C NMR (101 MHz, CD_3OD): 158.47 (NCOO), 79.85 ($C(CH_3)_3$), 67.38 (d, J = 6.0 Hz, $CH_3(CH_2)_6CH_2O$), 63.20 (d, J = 7.3 Hz, $OCH_2CH_2CH_2O$), 51.84 ($NCH_2(CH_2)_2NH$), 47.69 (PCH_2CH_2), 39.63 (CH_2NH), 33.01 ($CH_3CH_2CH_2$), 32.58 (t, J = 6.0 Hz, $OCH_2CH_2CH_2O$), 31.67 (d, J = 6.3 Hz, $CH_3CH_2(CH_2)_4CH_2CH_2O$), 28.86 ($C(CH_3)_3$), 28.16 ($CH_2CH_2NH$), 30.41, 30.30, 26.76 ($(CH_2)_3(CH_2)_2O$), 23.74 ($CH_3CH_2$), 22.76 (d, J = 136.8 Hz, PCH_2CH_2N), 14.49 (CH_3CH_2).

^{31}P{^{1}H} NMR (162 MHz, CD_3OD): 34.90.

IR ν_{max} (KBr) 3455 (w), 3362 (w, br), 2980 (s), 2960 (s), 2931 (s), 2871 (m), 2858 (m), 2825 (w), 1707 (vs), 1507 (s), 1475 (m, sh), 1467 (m), 1456 (m), 1440 (w, sh), 1393 (m), 1379 (w), 1367 (s), 1249 (s), 1052 (s), 1023 (s), 979 (s).

HR-MS(ESI^{+}): For $C_{55}H_{112}O_{14}N_6NaP_2$ $(M+Na)^+$ m/z calculated 1165.76040, found 1165.75925.

^{1}H NMR spectrum of compound **S93**

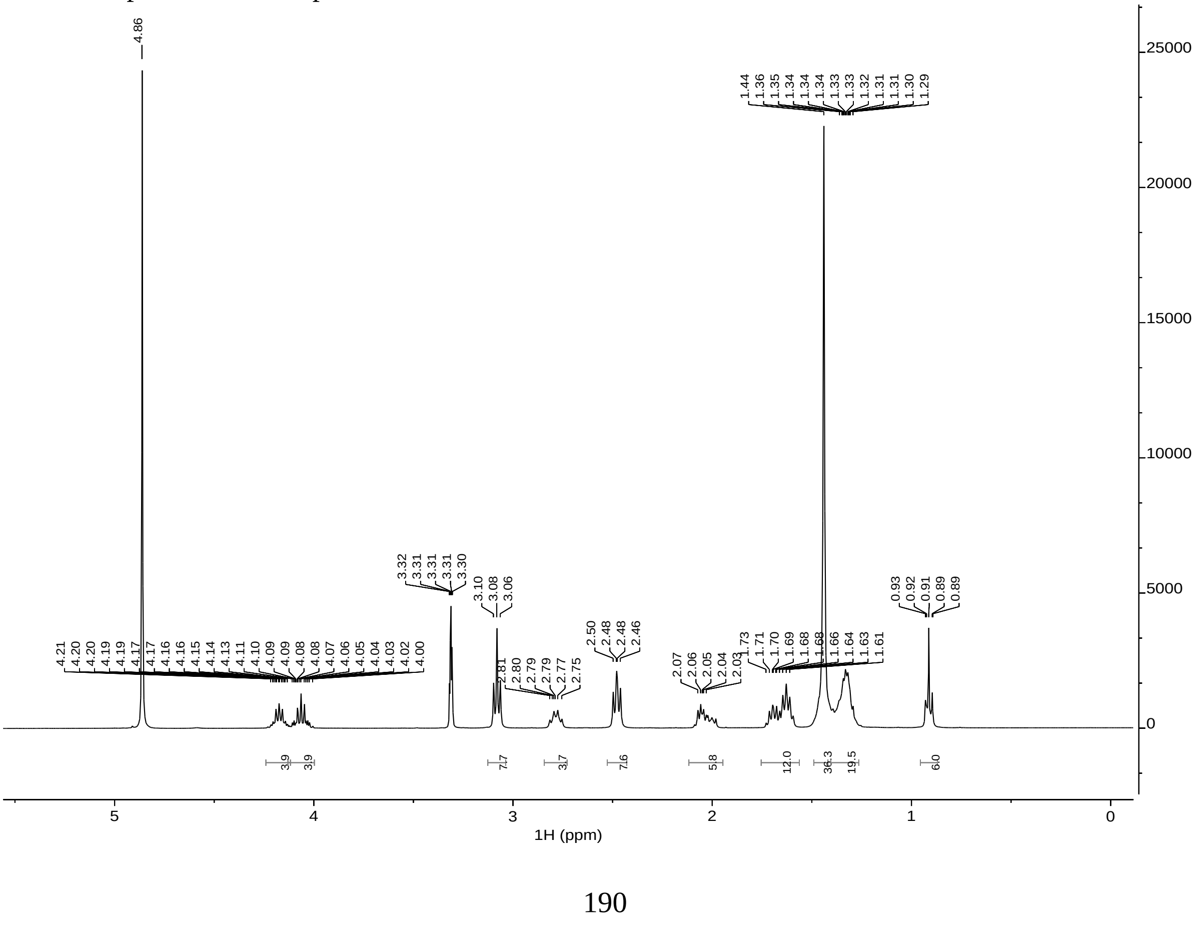

^{13}C NMR spectrum of compound **S93**

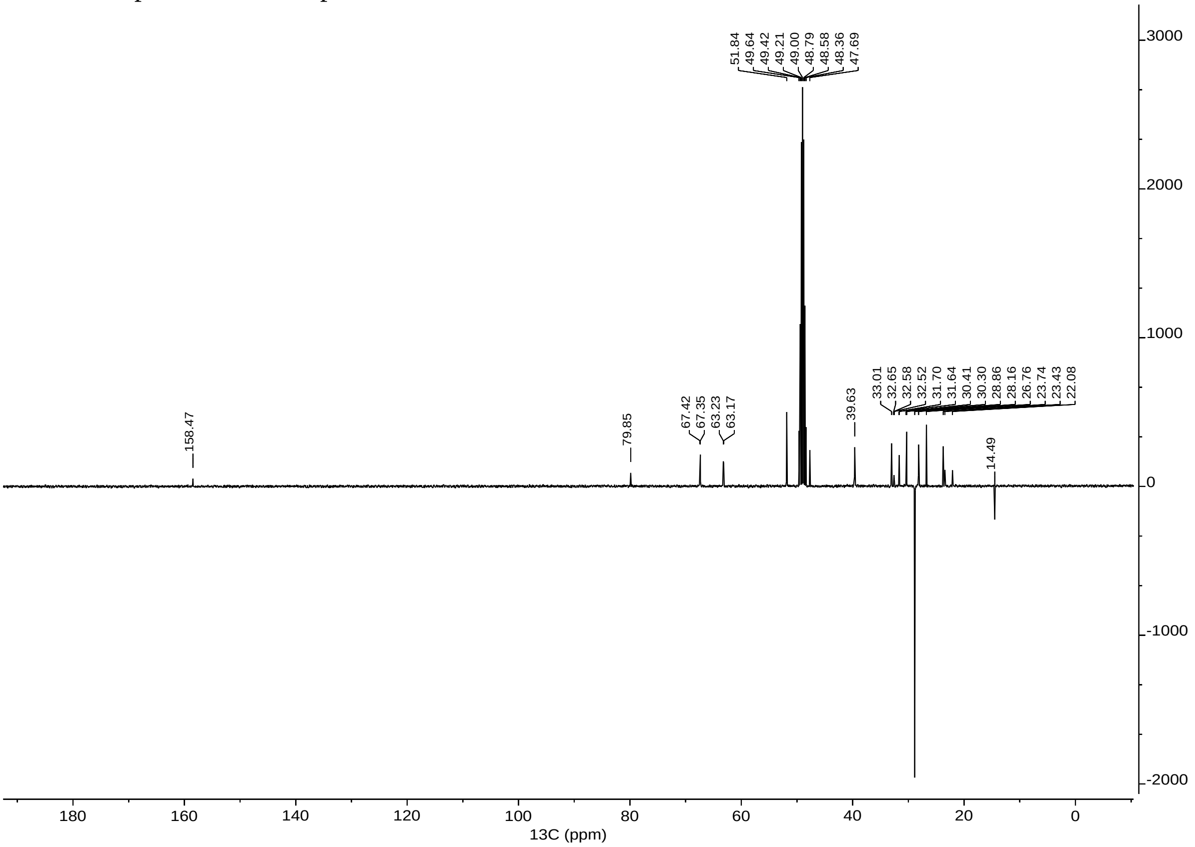

^{31}P{^{1}H} NMR spectrum of compound **S93**

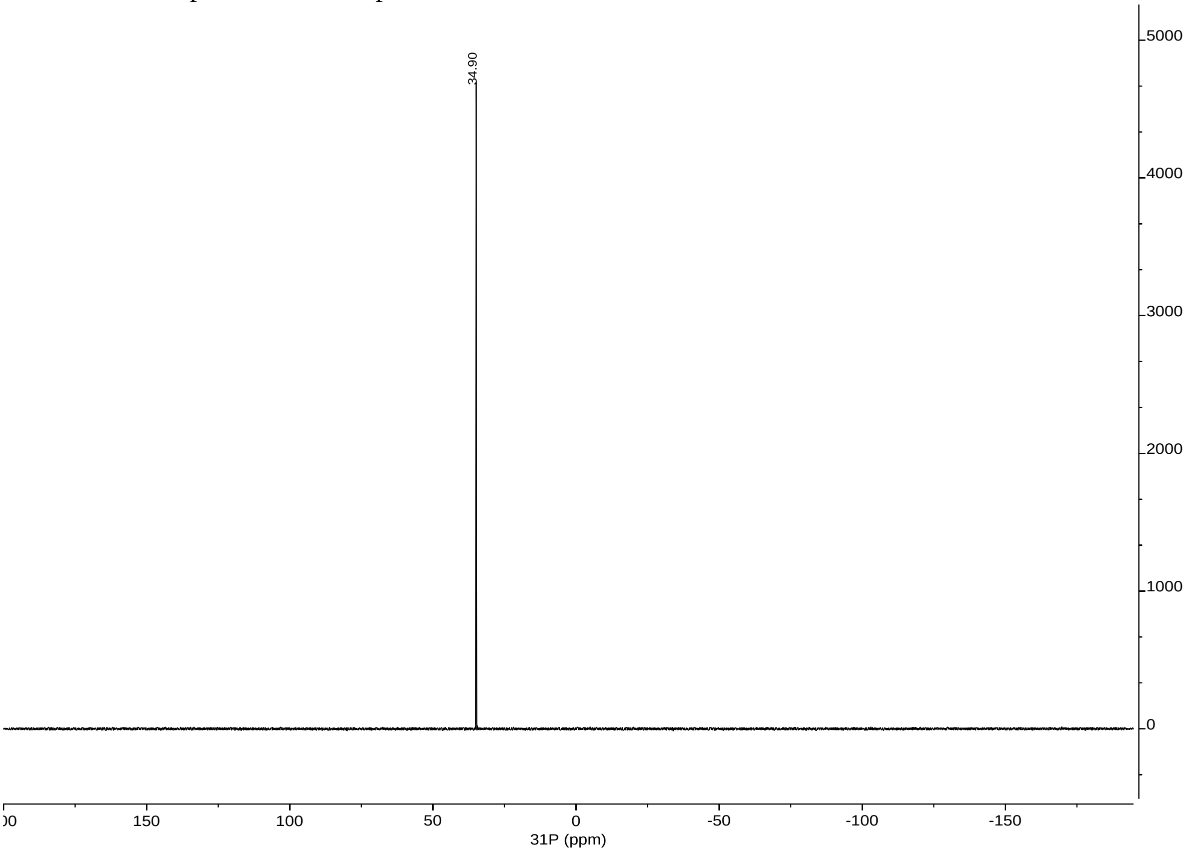

***N,N',N'',N'''*-Tetrakis(*tert*-butyloxycarbonyl) bis((*Z*)-oct-3-en-1-yl) propane-1,3-diyl bis((2-(bis(3-aminopropyl)amino)ethyl)phosphonate) (S94)**

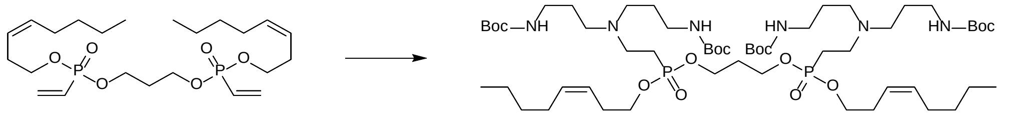

The title compound was prepared according to general method **D** from vinylphosphonate dimer **S41** (0.45 g, 0.95 mmol) and bis(3-*tert*-butyloxycarbonylaminopropyl)amine (1.25, 3.78 mmol) in 55% yield (0.59 g, 0.52 mmol) as a colourless oil.

^{1}H NMR (401 MHz, CD_3OD): 5.59–5.52 (m, 2H, $CH_3(CH_2)_3CH$), 5.44–5.37 (m, 2H, $CH(CH_2)_2O$), 4.22–4.13 (m, 4H, $OCH_2CH_2CH_2O$), 4.10–4.00 (m, 4H, $CHCH_2CH_2O$), 3.08 (t, 8H, J = 6.7 Hz, CH_2NH), 2.82–2.75 (m, 4H, PCH_2CH_2), 2.50–2.42 (m, 12H, $CH_2(CH_2)_2NH$, $CHCH_2CH_2O$), 2.12–1.94 (m, 10H, PCH_2, $CH_3(CH_2)_2CH_2$, $OCH_2CH_2CH_2O$), 1.63 (p, 8H, J = 6.8 Hz, CH_2CH_2NH), 1.55–1.52 (m, 2H, $O(CH_2)_2CH_2(CH_2)_2O$), 1.44 (s, 36H, $C(CH_3)_3$), 1.38–1.33 (m, 8H, $CH_3(CH_2)_2$), 0.95–0.900 (m, 6H, CH_3CH_2).

^{13}C NMR (101 MHz, CD_3OD): 158.47 (NCOO), 134.19 ($CH_3(CH_2)_3CH$), 125.33 ($CHCH_2CH_2O$), 79.87 ($C(CH_3)_3$, 66.86 (d, J = 7.1 Hz, $CHCH_2CH_2O$), 63.24 (d, J = 7.3 Hz, $OCH_2CH_2CH_2O$), 51.85 ($CH_2(CH_2)_2NH$), 47.69 (PCH_2CH_2), 39.64 (CH_2NH), 32.96 ($CH_3CH_2CH_2$), 32.59 (t, J = 6.0 Hz, $OCH_2CH_2CH_2O$), 29.42 (d, J = 6.3 Hz, $CHCH_2CH_2O$), 28.86 ($C(CH_3)_3$), 28.16 ($CH_3(CH_2)_2CH_2$), 28.12 (CH_2CH_2NH), 23.40 (CH_3CH_2), 22.82 (d, J = 136.8 Hz, PCH_2), 14.40 (CH_3CH_2).

^{31}P{^{1}H} NMR (162 MHz, CD_3OD): 34.73.

IR ν_{max} (KBr) 3328 (w), 3005 (w, sh), 2962 (m), 2930 (m), 2871 (m), 2815 (w), 1712 (vs), 1696 (s, sh), 1525 (m), 1457 (m), 1390 (m), 1365 (s), 1251 (s), 1174 (s), 1052 (m), 1014 (m), 974 (m), 936 (m, sh).

HR-MS(ESI^{+}): For $C_{55}H_{108}O_{14}N_6NaP_2$ $(M+Na)^{+}$ m/z calculated 1161.72910, found 1161.72953

^{1}H NMR spectrum of compound **S94**

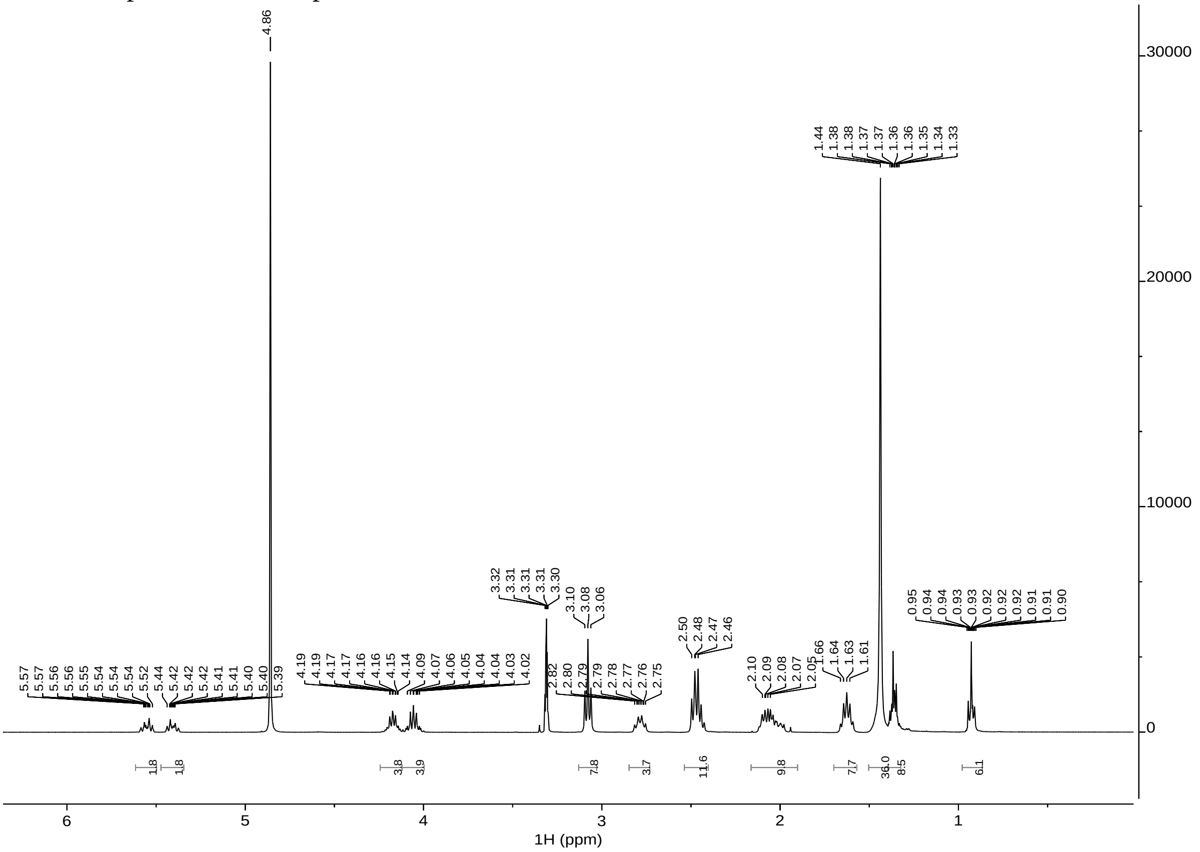

^{13}C NMR spectrum of compound **S94**

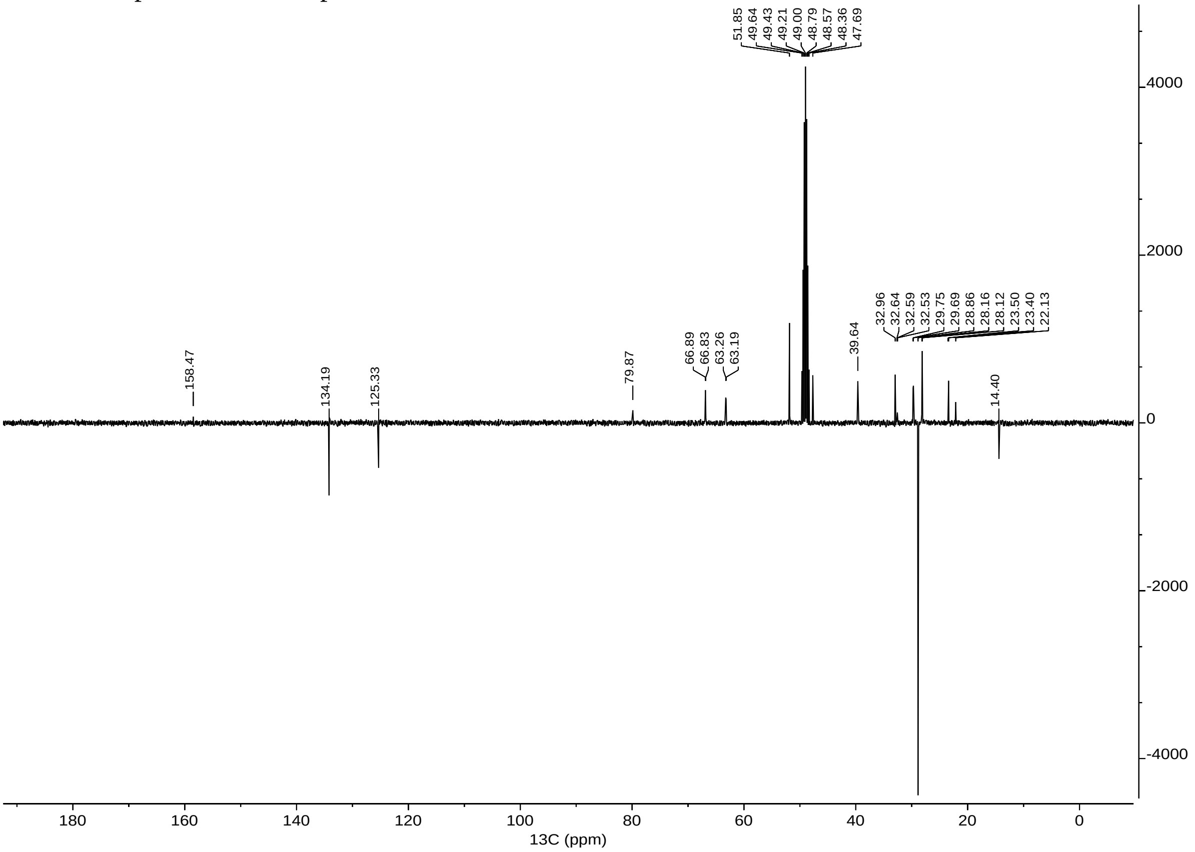

^{31}P{^{1}H} NMR spectrum of compound **S94**

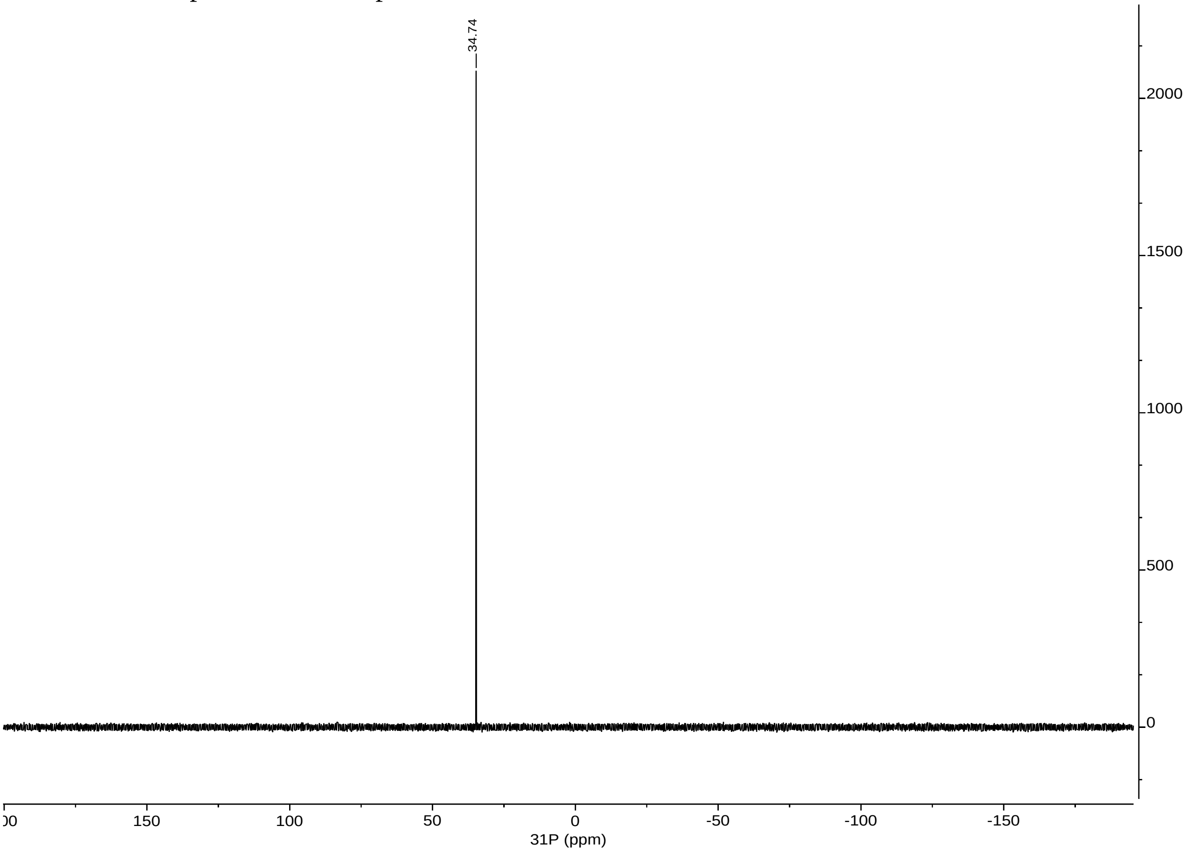

_N,N’,N’’,N’’’_-Tetrakis(_tert_-butyloxycarbonyl) bis((_Z_)-non-3-en-1-yl) propane-1,3-diyl bis((2-(bis(3-aminopropyl)amino)ethyl)phosphonate) (S95)

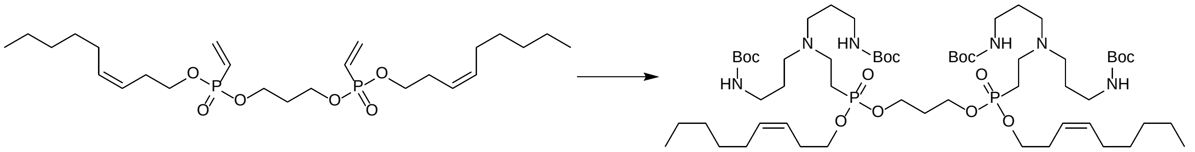

The title compound was prepared according to general method **D** from vinylphosphonate dimer **S42** (0.70 g, 1.39 mmol) and bis(3-*tert*-butyloxycarbonylaminopropyl)amine (1.38 g, 4.16 mmol) in 81% yield (1.31 g, 1.12 mmol) as a colourless oil.

^{1}H NMR (401 MHz, CD_3OD): 5.59–5.52 (m, 2H, $CH_3(CH_2)_4CH$), 5.44–5.37 (m, 2H, $CH(CH_2)_2O$), 4.21–4.13 (m, 4H, $OCH_2CH_2CH_2O$), 4.10–4.02 (m, 4H, $CHCH_2CH_2O$), 3.08 (t, 8H, J = 6.8 Hz, CH_2NH), 2.78 (q, 4H, J = 8.6 Hz, PCH_2CH_2), 2.49–2.42 (m, 12H, $CH_2(CH_2)_2NH$, $CHCH_2CH_2O$), 2.11–1.98 (m, 10H, PCH_2, $OCH_2CH_2CH_2O$, $CH_3(CH_2)_3CH_2$), 1.62 (p, 8H, J = 6.7 Hz, $CH_2CH_2NH_2$), 1.44 (s, 36H, $C(CH_3)_3$), 1.39–1.29 (m, 12H, $CH_3(CH_2)_3$), 0.93–0.90 (m, 6H, CH_3CH_2).

^{13}C NMR (101 MHz, CD_3OD): 158.47 (NCOO), 134.24 ($CH_3(CH_2)_3CHCHCH_2CH_2CH_2O$), 125.32 ($CH_3(CH_2)_3CHCHCH_2CH_2CH_2O$), 79.86 ($C(CH_3)_3$), 66.96 (d, J = 7.3 Hz, $CH_3(CH_2)_3CHCHCH_2CH_2CH_2O$), 63.23 (d, J = 6.0 Hz, $OCH_2CH_2CH_2O$), 47.67 (PCH_2CH_2N), 51.84 ($NCH_2CH_2CH_2NH$), 39.64 ($NCH_2CH_2CH_2NH$), 32.68 ($CH_3CH_2CH_2CH_2CH_2CHCHCH_2CH_2O$), 32.58 (t, J = 6.1 Hz, $OCH_2CH_2CH_2O$), 30.44 ($CH_3CH_2CH_2CH_2CH_2CHCHCH_2CH_2O$), 29.86 (d, J = 6.4 Hz, $CH_3(CH_2)_4CHCHCH_2CH_2O$), 28.87 ($C(CH_3)_3$, 28.39 ($CH_3(CH_2)_3CH_2CHCHCH_2CH_2O$), 28.18 ($NCH_2CH_2CH_2NH$), 23.67 ($CH_3CH_2$), 22.81 (d, J = 136.8 Hz, PCH_2CH_2N), 14.49 (CH_3CH_2).

^{31}P{^{1}H} NMR (162 MHz, CD_3OD): 35.05.

IR ν_{max} (KBr) 3455 (m), 3363 (w), 2980 (s), 2962 (s), 2931 (s), 2859 (m), 2825 (m), 1707 (vs), 1507 (s), 1467 (m), 1456 (m), 1440 (m), 1380 (s), 1249 (s), 1053 (m), 1016 (s), 978 (m).

HR-MS(ESI^{+}): For $C_{57}H_{113}O_{14}N_6P_2$ $(M+H)^{+}$ m/z calculated 1167.77845, found 1167.77811.

^{1}H NMR spectrum of compound **S95**

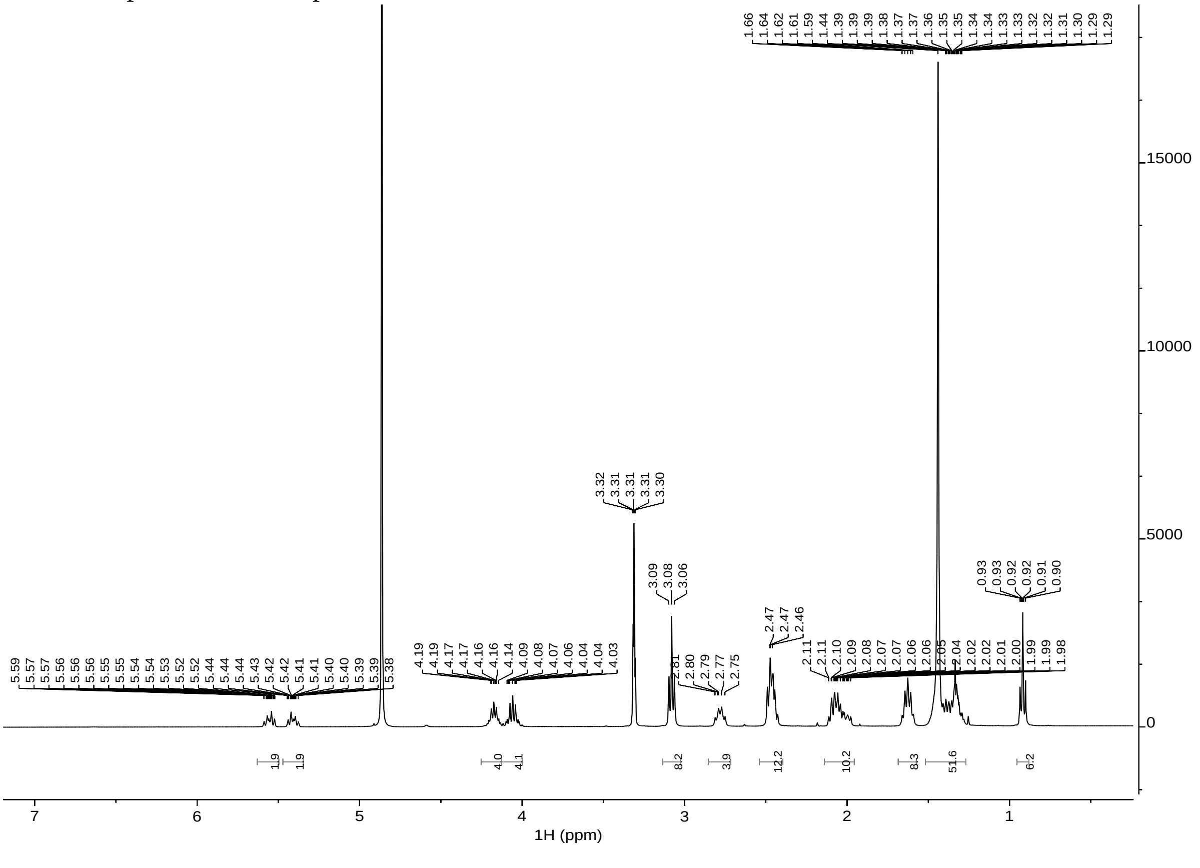

^{13}C NMR spectrum of compound **S95**

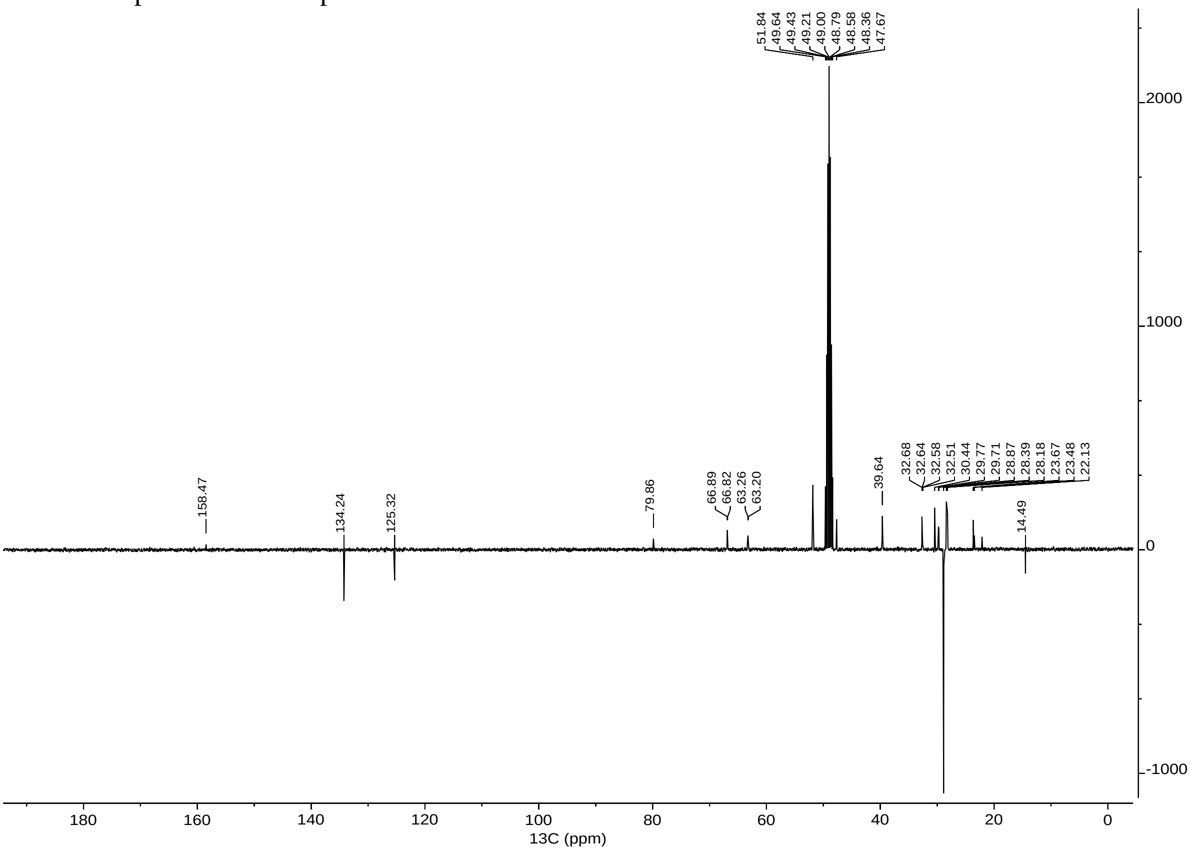

^{31}P{^{1}H} NMR spectrum of compound **S95**

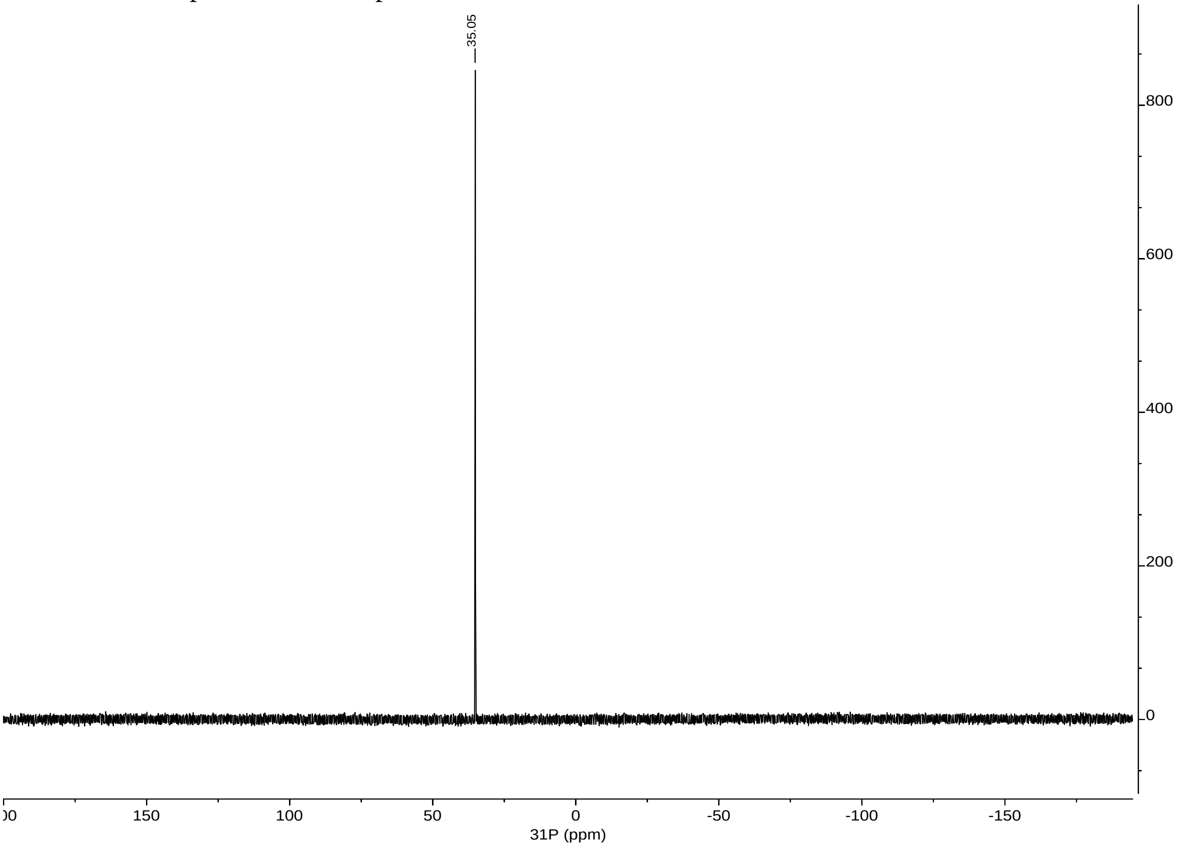

_N,N',N'',N'''_-Tetrakis(_tert_-butyloxycarbonyl) bis((_Z_)-dec-4-en-1-yl) propane-1,3-diyl bis((2-(bis(3-aminopropyl)amino)ethyl)phosphonate) (S96)

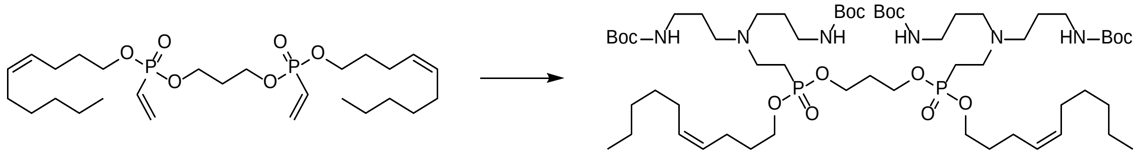

The title compound was prepared according to general method **D** from vinylphosphonate dimer **S44** (0.42 g, 0.79 mmol) and bis(3-*tert*-butyloxycarbonylaminopropyl)amine (1.05 g, 3.16 mmol) in 74.0% yield (0.70 g, 0.58 mmol) as a colourless oil.

^{1}H NMR (401 MHz, CD_3OD): 5.48–5.35 (m, 4H, C*H*=C*H*), 4.22–4.14 (m, 4H, OC*H*$_2$CH$_2$C*H*$_2$O), 4.11–4.03 (m, 4H, CH(CH$_2$)$_2$C*H*$_2$O), 3.08 (t, 8H, *J* = 6.8 Hz, C*H*$_2$NH), 2.80 (q, 4H, *J* = 8.5 Hz, PCH$_2$C*H*$_2$), 2.48 (dd, 8H, *J* = 8.1, 6.3 Hz, C*H*$_2$(CH$_2$)$_2$NH), 2.20–2.14 (PC*H*$_2$), 2.09–1.98 (m, 10H, CH$_3$(CH$_2$)$_3$C*H*$_2$, CHC*H*$_2$(CH$_2$)$_2$O, OCH$_2$C*H*$_2$CH$_2$O), 1.75 (dt, 4H, *J* = 8.0, 6.5 Hz, CHCH$_2$C*H*$_2$CH$_2$O), 1.63 (p, 8H, *J* = 6.8 Hz, C*H*$_2$CH$_2$NH$_2$), 1.44 (s, 36H, , C(C*H*$_3$)$_3$), 1.40–1.29 (m, 12H, CH$_3$(C*H*$_2$)$_3$), 0.93–0.89 (m, 6H, C*H*$_3$CH$_2$).

^{13}C NMR (101 MHz, CD_3OD): 158.46 (N*C*OO), 132.21 (CH$_3$(CH$_2$)$_4$*C*H), 129.18 (*C*H(CH$_2$)$_3$O), 79.86 (*C*(CH$_3$)$_3$), 66.79 (d, *J* = 7.0 Hz, CH(CH$_2$)$_2$*C*H$_2$O), 63.29 (d, *J* = 6.4 Hz, O*C*H$_2$CH$_2$*C*H$_2$O), 51.85 (*C*H$_2$(CH$_2$)$_2$NH), 47.71 (PCH$_2$*C*H$_2$), 39.64 (*C*H$_2$NH), 32.70 (CH$_3$CH$_2$*C*H$_2$), 32.63 (OCH$_2$*C*H$_2$CH$_2$O), 31.72 (d, *J* = 6.3 Hz, CHCH$_2$*C*H$_2$CH$_2$O), 30.52 (CH$_3$(CH$_2$)$_2$*C*H$_2$), 28.87 (C(*C*H$_3$)$_3$, 28.23 (CH$_3$(CH$_2$)$_3$*C*H$_2$), 28.18 (*C*H$_2$CH$_2$NH), 24.26 (CH*C*H$_2$(CH$_2$)$_2$O), 23.68 (CH$_3$*C*H$_2$), 22.80 (d, *J* = 136.8 Hz, P*C*H$_2$), 14.51 (*C*H$_3$CH$_2$).

^{31}P{^{1}H} NMR (162 MHz, CD_3OD): 35.04.

IR ν_{max} (KBr) 3455 (m), 3365 (w), 2980 (s), 2960 (s), 2932 (s), 2872 (m), 2859 (m), 2825 (m), 1707 (vs), 1507 (s), 1467 (m), 1456 (m), 1440 (m), 1379 (vw), 1352 (w), 1249 (s), 1169 (s), 1028 (s), 1016 (s), 939 (w, sh), 978 (m), 464 (vw).

HR-MS(ESI$^+$): For $C_{59}H_{116}O_{14}N_6NaP_2$ (M+Na)$^+$ *m/z* calculated 1217.79170, found 1217.79224.

^{1}H NMR spectrum of compound **S96**

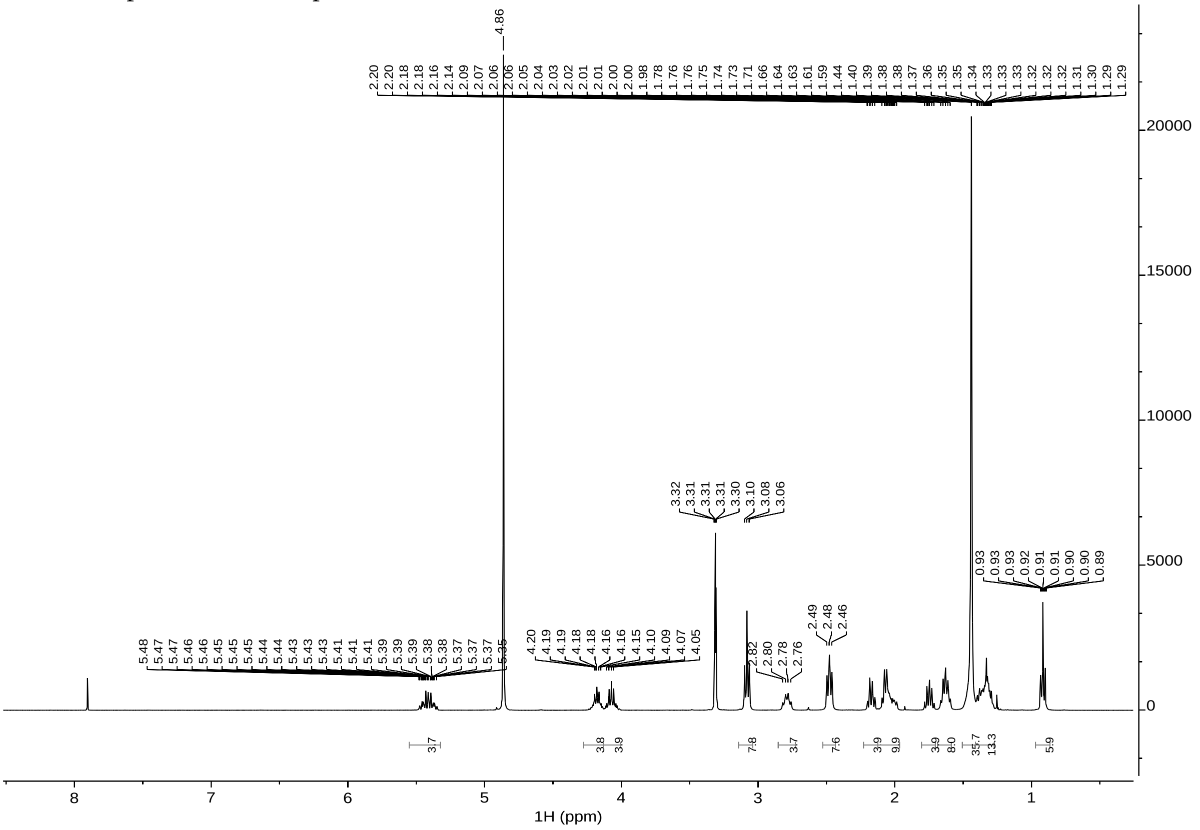

^{13}C NMR spectrum of compound **S96**

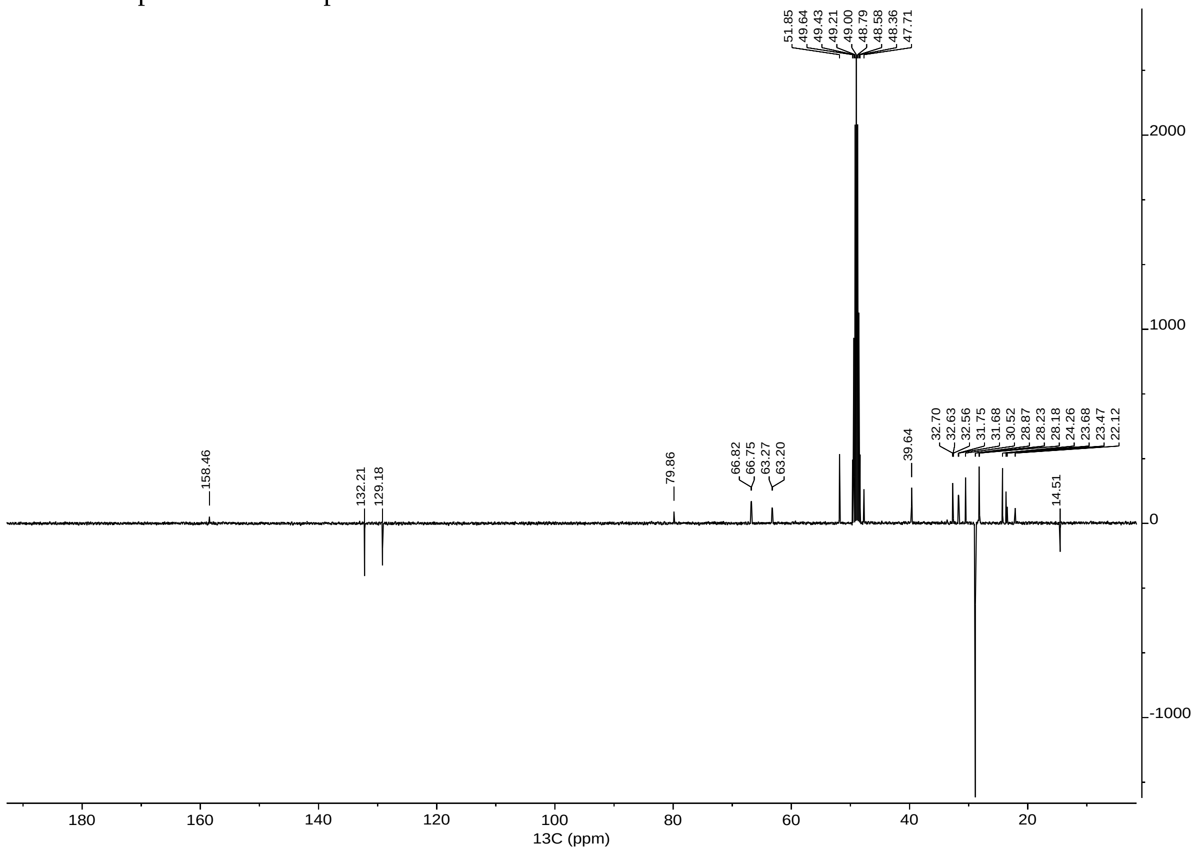

$^{31}P\{^{1}H\}$ NMR spectrum of compound **S96**

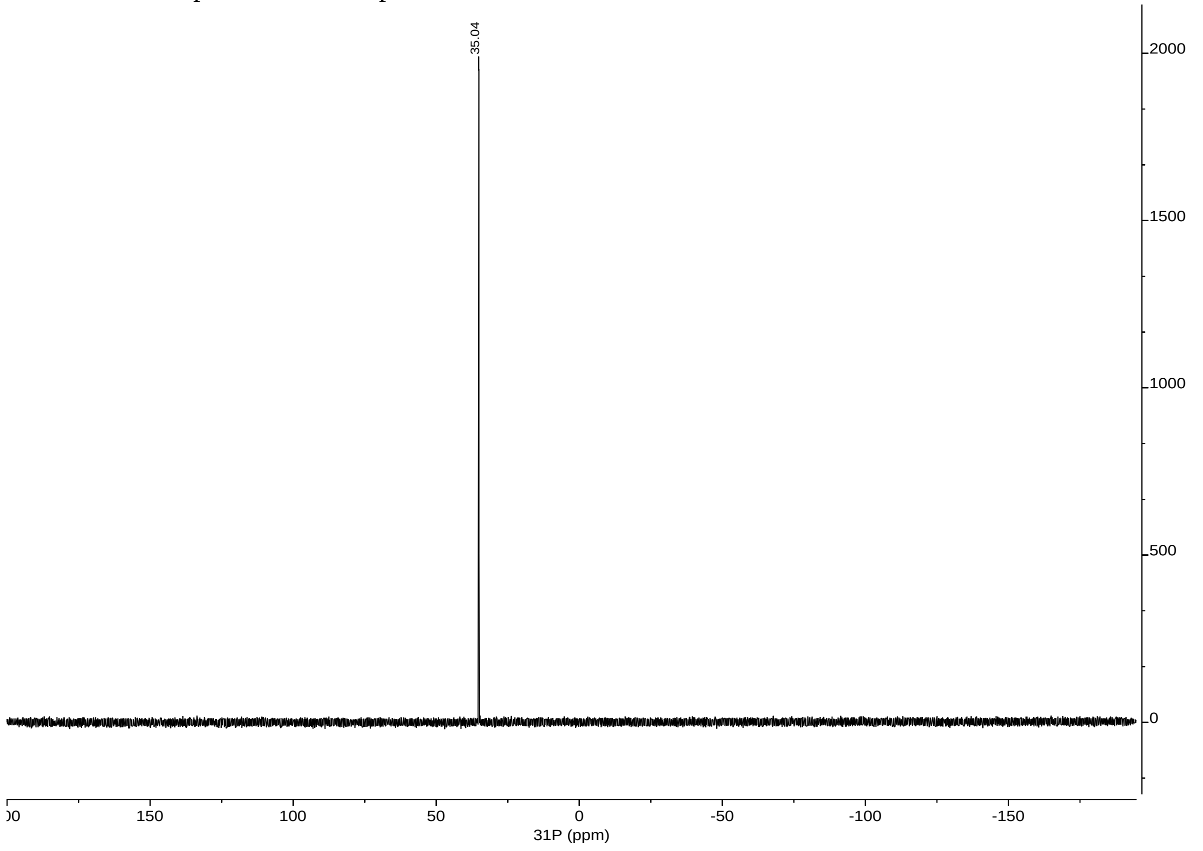

***N,N',N'',N'''*-Tetrakis(*tert*-butyloxycarbonyl) butane-1,4-diyl dioctyl bis((2-(bis(3-aminopropyl)amino)ethyl)phosphonate) (S97)**

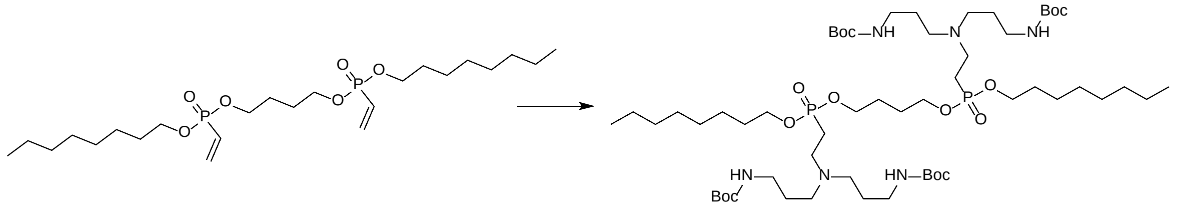

The title compound was prepared according to general method **D** from vinylphosphonate dimer **S48** (0.45 g, 0.91 mmol) and bis(3-*tert*-butyloxycarbonylaminopropyl)amine (1.21 g, 3.64 mmol) in 59% yield (0.62 g, 0.54 mmol) as a colourless oil.

^{1}H NMR (400 MHz, CD_3OD): 4.12–4.01 (m, 8H, $CH_3(CH_2)_5CH_2CH_2O$, $OCH_2CH_2CH_2CH_2O$), 3.08 (t, 8H, J = 6.8 Hz, $NCH_2CH_2CH_2NH$), 2.49–2.46 (m, 8H, $NCH_2CH_2CH_2NH$), 2.08–1.96 (m, 4H, PCH_2), 1.80 (p, 4H, J = 3.1 Hz, $OCH_2CH_2CH_2CH_2O$), 1.72–1.61 (m, 12H, $CH_3(CH_2)_5CH_2$, CH_2CH_2NH), 1.44 (s, 36H, $C(CH_3)_3$), 1.36–1.28 (m, 20H, $CH_3(CH_2)_5$), 0.92–0.89 (m, 6H, CH_3CH_2).

^{13}C NMR (101 MHz, CD_3OD): 158.49 (NCOO), 79.94 ($C(CH_3)_3$), 66.64, 67.38 (d, J = 6.9 Hz, $OCH_2CH_2CH_2CH_2O$, $CH_3(CH_2)_5CH_2CH_2O$), 51.83 ($NCH_2CH_2CH_2NH$), 47.66 (PCH_2CH_2), 39.63 (CH_2NH), 32.96 ($CH_3CH_2CH_2$), 31.61 (d, J = 5.8 Hz, $CH_3(CH_2)_5CH_2$), 28.09 (CH_2CH_2NH), 28.86 ($C(CH_3)_3$), 27.95 (d, J = 6.2 Hz, $OCH_2(CH_2)_2CH_2O$), 30.35, 30.23, 26.72 ($(CH_2)_3CH_2CH_2O$), 23.70 (CH_3CH_2), 22.68 (d, J = 136.8 Hz, PCH_2), 14.49 (CH_3CH_2).

^{31}P{^{1}H} NMR (162 MHz, CD_3OD): 34.54.

IR ν_{max} (KBr) 3455 (w), 3363 (w, br), 2980 (s), 2959 (s), 2931 (s), 2872 (m), 2858 (m), 2825 (w), 1707 (vs), 1507 (s), 1476 (m, sh), 1467 (m), 1456 (m), 1393 (m), 1367 (s), 1249 (s), 1046 (m), 1022 (m), 984 (m).

HR-MS(ESI$^+$): For $C_{56}H_{115}O_{14}N_6P_2$ $(M+H)^+$ m/z calculated 1157.79410, found 1157.79320.

^{1}H NMR spectrum of compound **S97**

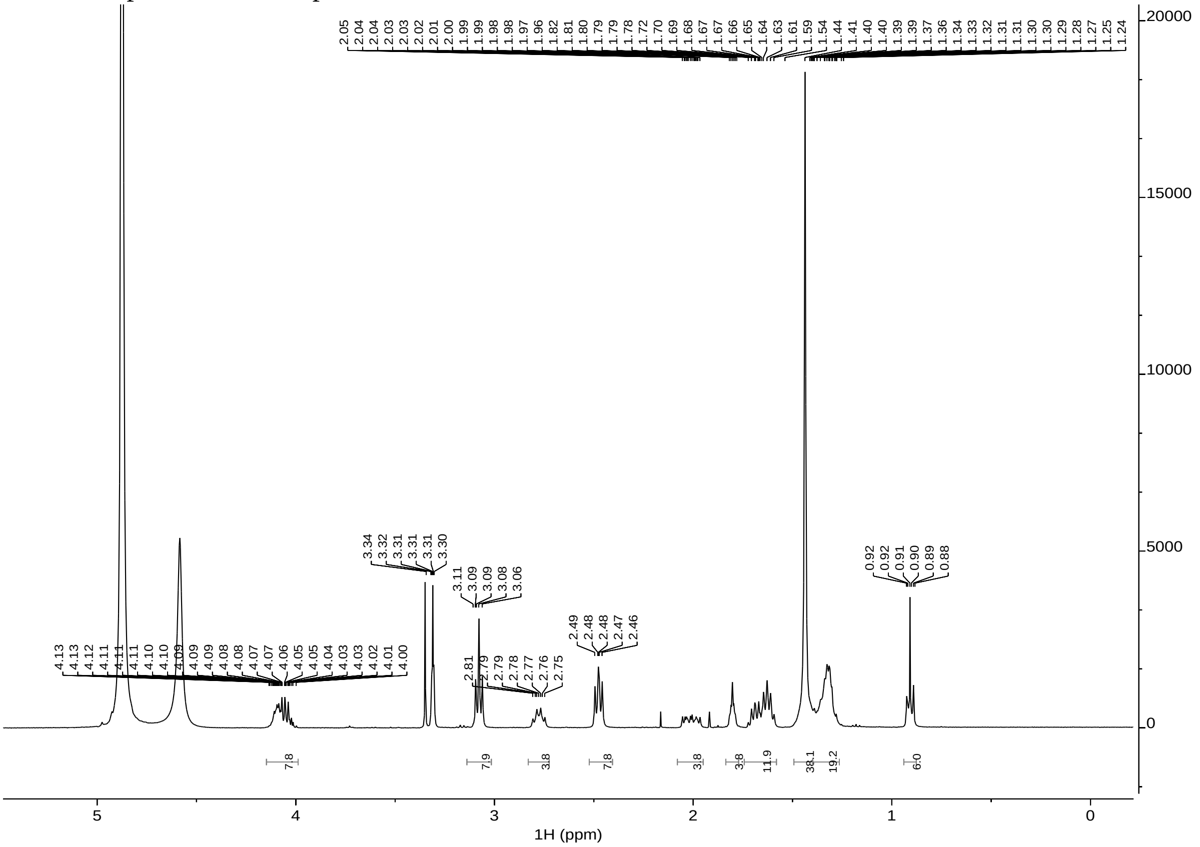

^{13}C NMR spectrum of compound **S97**

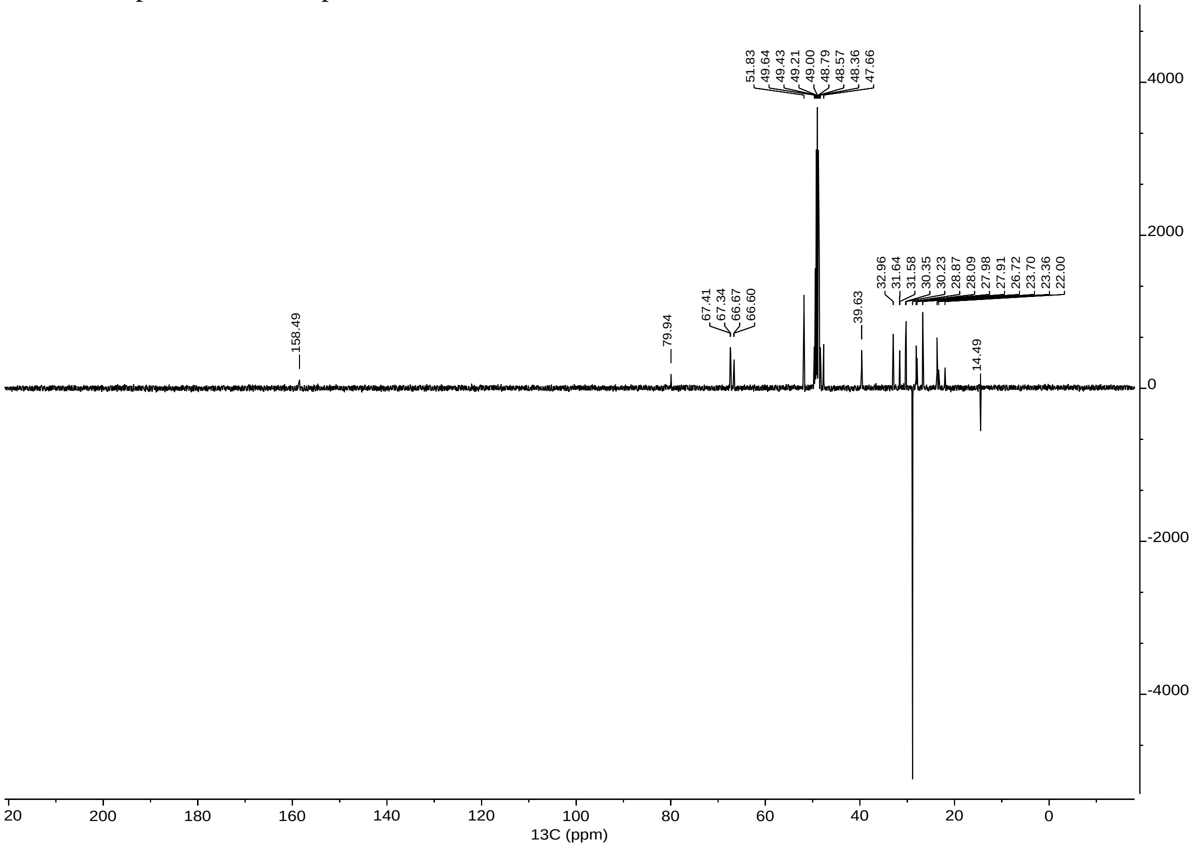

^{31}P{^{1}H} NMR spectrum of compound **S97**

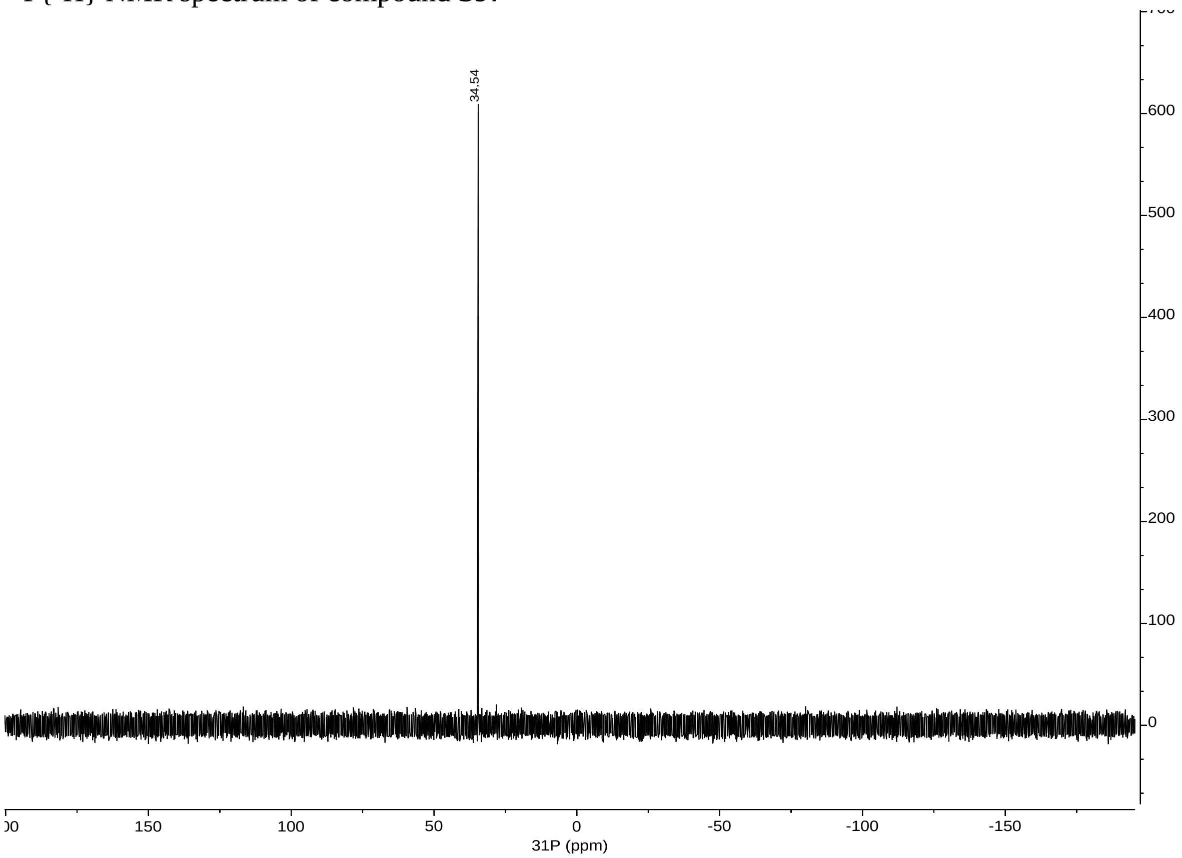

***N,N',N'',N'''*-Tetrakis(*tert*-butyloxycarbonyl) bis((*Z*)-hept-3-en-1-yl) pentane-1,5-diyl bis((2-(bis(3-aminopropyl)amino)ethyl)phosphonate) (S98)**

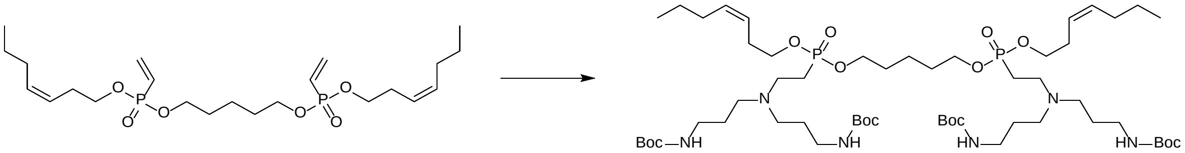

The title compound was prepared according to general method **D** from vinylphosphonate dimer **S49** (0.68 g, 1.43 mmol) and bis(3-*tert*-butyloxycarbonylaminopropyl)amine (1.89 g, 5.16 mmol) in 57% yield (0.98 g, 0.86 mmol) as a colourless oil.

^{1}H NMR (400 MHz, CD_3OD): 5.59–5.52 (m, 2H, $CH_3(CH_2)_2CH$), 5.45–5.38 (m, 2H, $CH(CH_2)_2O$), 4.10–3.98 (m, 8H, CH_2O), 3.08 (t, 8H, J = 6.7 Hz, CH_2NH), 2.82–2.75 (m, 4H, PCH_2CH_2), 2.50–2.42 (m, 12H, $NCH_2(CH_2)_2NH$, $CHCH_2CH_2O$), 2.10–1.90 (m, 8H, $CH_3CH_2CH_2$, PCH_2), 1.77–1.70 (m, 4H, $OCH_2CH_2CH_2CH_2CH_2O$), 1.63 (p, 8H, J = 6.9 Hz, CH_2CH_2NH), 1.55–1.52 (m, 2H, $O(CH_2)_2CH_2(CH_2)_2O$), 1.44–1.37 (m, 40H, $C(CH_3)_3$, CH_3CH_2), 0.93 (t, 6H, J = 7.4 Hz, CH_3CH_2).

^{13}C NMR (101 MHz, CD_3OD): 158.45 (NCOO), 133.93 ($CH_3(CH_2)_3CHCH(CH_2)_2O$), 125.57 ($CH_3(CH_2)_2CHCH(CH_2)_2O$), 79.85 ($C(CH_3)_3$, 66.95 (d, J = 6.7 Hz, OCH_2), 66.73 (d, J = 6.9 Hz, OCH_2), 51.87 ($NCH_2(CH_2)_2NH$), 47.69 (PCH_2CH_2), 39.64 ($N(CH_2)_2CH_2NH$), 31.14 (d, J = 7.1 Hz, $OCH_2CH_2CH_2CH_2CH_2O$), 30.46 ($CH_3CH_2CH_2$), 29.73 (d, J = 6.2 Hz, $CHCH_2CH_2O$), 28.86 ($C(CH_3)_3$), 28.18 (CH_2CH_2NH), 23.81 (CH_3CH_2), 22.90 ($O(CH_2)_2CH_2(CH_2)_2O$), 22.81 (d, J = 136.7 Hz, PCH_2), 14.19 (CH_3CH_2).

^{31}P{^{1}H} NMR (162 MHz, CD_3OD): 34.48.

IR ν_{max} (KBr) 3328 (m), 3005 (w, sh), 2963 (s), 2932 (s), 2871 (m), 2814 (m), 1712 (vs), 1693 (s), 1524 (s), 1456 (m), 1390 (m), 1365 (s), 1251 (s), 1174 (vs), 1054 (s), 1007 (s), 722 (w), 462 (w).

HR-MS(ESI$^+$): For $C_{55}H_{108}O_{14}N_6NaP_2$ $(M+Na)^+$ m/z calculated 1161.72910, found 1161.73010.

NMR spectrum of compound **S98**

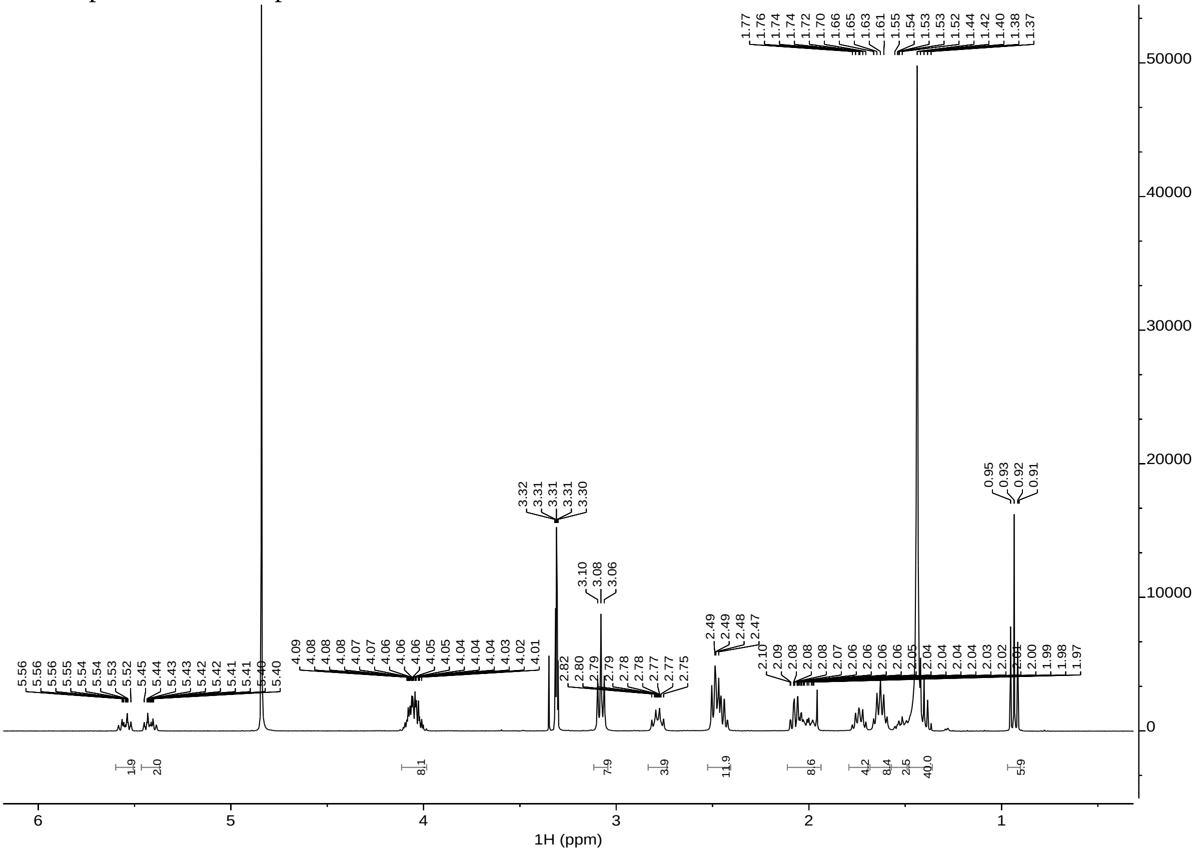

^{13}C NMR spectrum of compound **S98**

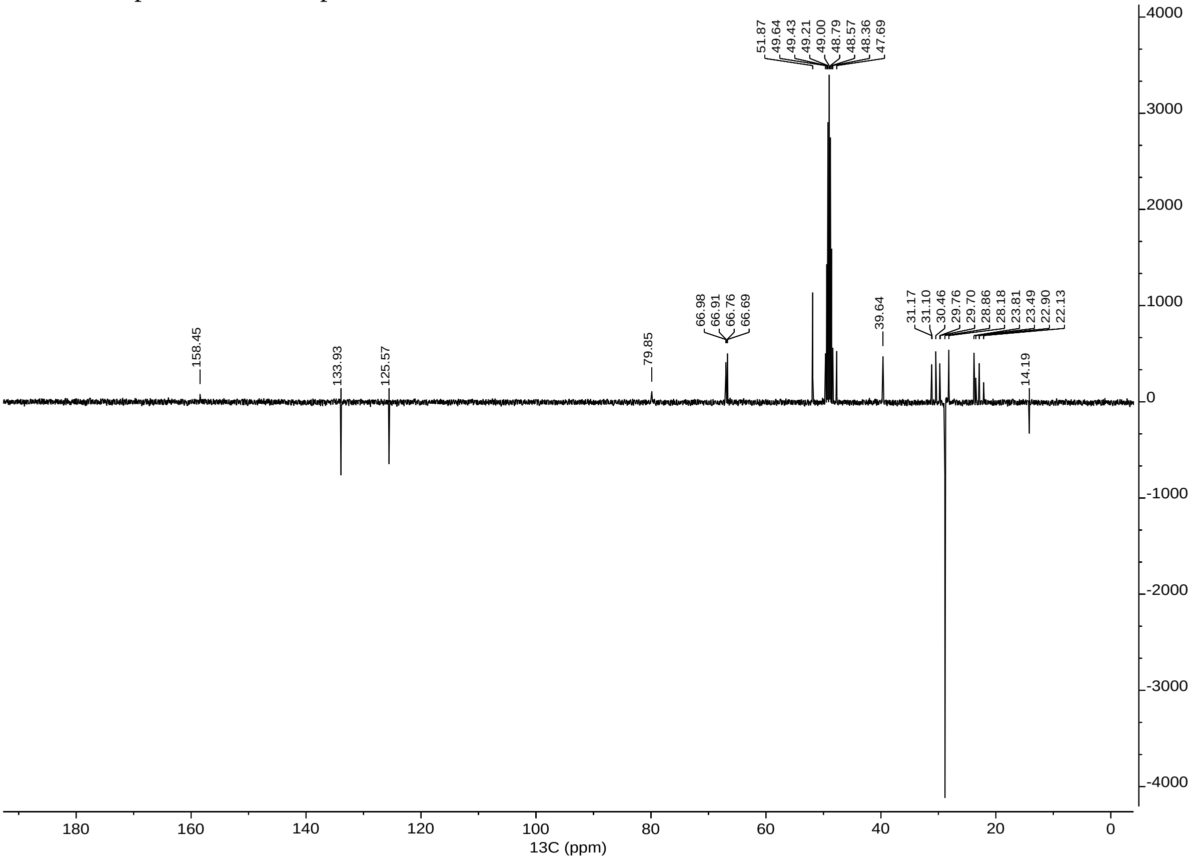

^{31}P{^{1}H} NMR spectrum of compound **S98**

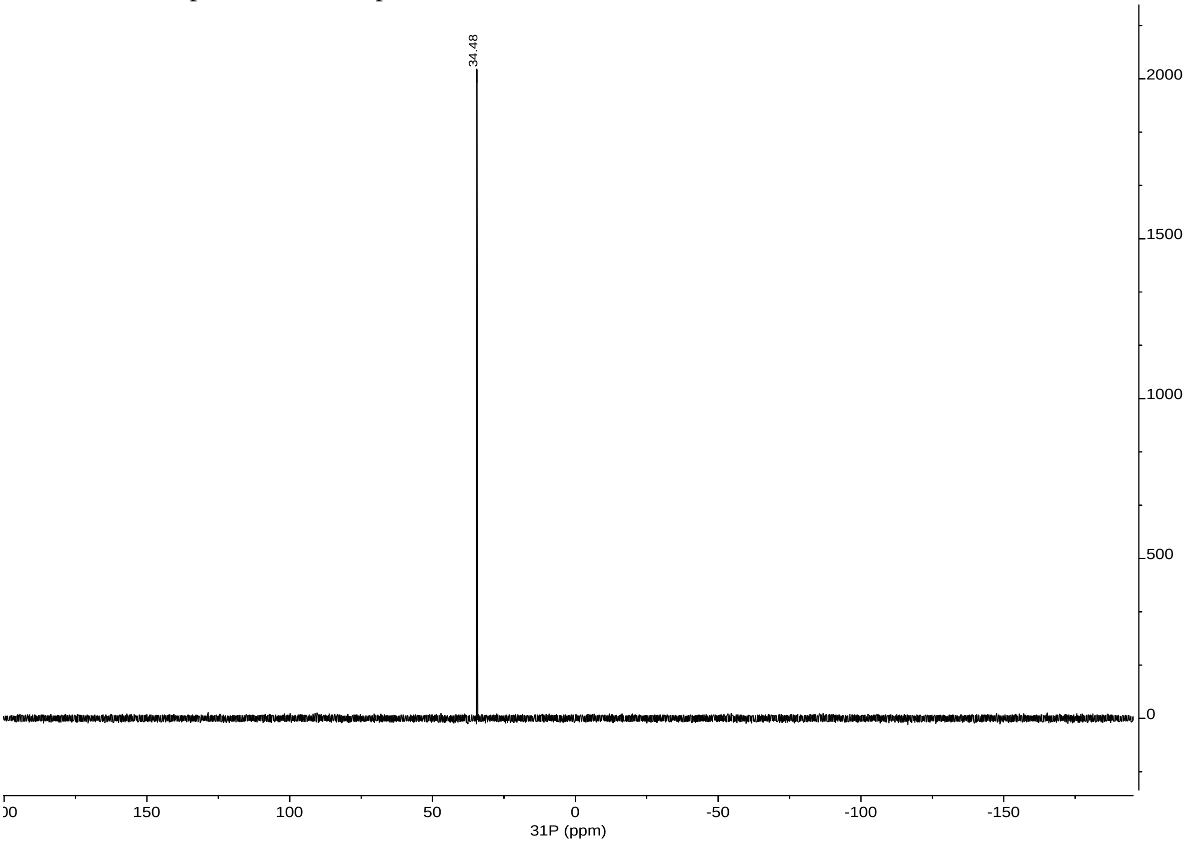

***N,N',N'',N'''*-Tetrakis(*tert*-butyloxycarbonyl) bis((*Z*)-hept-4-en-1-yl) pentane-1,5-diyl bis((2-(bis(3-aminopropyl)amino)ethyl)phosphonate) (S99)**

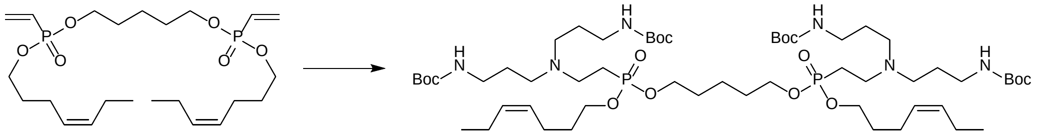

The title compound was prepared according to general method **D** from vinylphosphonate dimer **S50** (0.27 g, 0.57 mmol) and bis(3-*tert*-butyloxycarbonylaminopropyl)amine (0.76 g, 2.3 mmol) in 78% yield (0.51 g, 0.45 mmol) as a colourless oil.

^{1}H NMR (401 MHz, CD_3OD): 5.48–5.41 (m, 2H, CH_3CH_2CH), 5.38–5.31 (m, 2H, $CH(CH_2)_3O$), 4.11–4.00 (m, 8H, CH_2O), 3.08 (t, 8H, J = 6.7 Hz, CH_2NH), 2.81–2.75 (m, 4H, PCH_2CH_2), 2.47 (t, 8H, J = 7.6 Hz, $CH_2(CH_2)_2NH$), 2.20–2.14 (m, 4H, PCH_2), 2.11–1.96 (m, 8H, CH_3CH_2, $CHCH_2(CH_2)_2O$), 1.78–1.70 (m, 8H, $CHCH_2CH_2CH_2O$, $OCH_2CH_2CH_2CH_2CH_2O$), 1.66–1.61 (m, 8H, CH_2CH_2NH), 1.56–1.52 ($O(CH_2)_2CH_2(CH_2)_2O$), 1.44 (s, 36H, $C(CH_3)_3$), 0.98 (t, 6H, J = 7.4 Hz, CH_3CH_2).

^{13}C NMR (101 MHz, CD_3OD): 158.47 (NCOO), 133.81 ($CH_3CH_2CHCHCH_2CH_2CH_2O$), 128.59 ($CH_3CH_2CHCHCH_2CH_2CH_2O$), 79.86 ($C(CH_3)_3$), 66.99, 66.63 (d, J = 6.1 Hz, $CH_3CH_2CHCHCH_2CH_2CH_2O$, $OCH_2CH_2CH_2CH_2CH_2O$), 51.85 ($NCH_2CH_2CH_2NH$), 47.71 (PCH_2CH_2N), 39.63 ($NCH_2CH_2CH_2NH$), 31.68, 31.16 (d, J = 6.4 Hz, $CH_3CH_2CHCHCH_2CH_2CH_2O$, $OCH_2CH_2CH_2CH_2CH_2O$), 28.86 ($C(CH_3)_3$, 28.17 ($NCH_2CH_2CH_2NH$), 24.10 ($CH_3CH_2CHCHCH_2CH_2CH_2O$), 22.91 ($OCH_2CH_2CH_2CH_2CH_2O$), 22.76 (d, J = 136.8 Hz, PCH_2CH_2N), 21.51 ($CH_3CH_2CHCHCH_2CH_2CH_2O$), 14.77 ($CH_3CH_2CHCHCH_2CH_2CH_2O$).

^{31}P{^{1}H} NMR (162 MHz, CD_3OD): 34.48.

IR ν_{max} (KBr) 3455 (m), 3369 (w), 2979 (s), 2935 (s), 2873 (m), 2823 (m), 1707 (vs), 1507 (s), 1477 (m), 1467 (m), 1456 (m), 1393 (m), 1367 (s), 1249 (s), 1080–990 (m), 462 (w).

HR-MS(ESI$^+$): For $C_{55}H_{109}O_{14}N_6P_2$ $(M+H)^+$ *m/z* calculated 1139.74715, found 1139.74671.

^{1}H NMR spectrum of compound **S99**

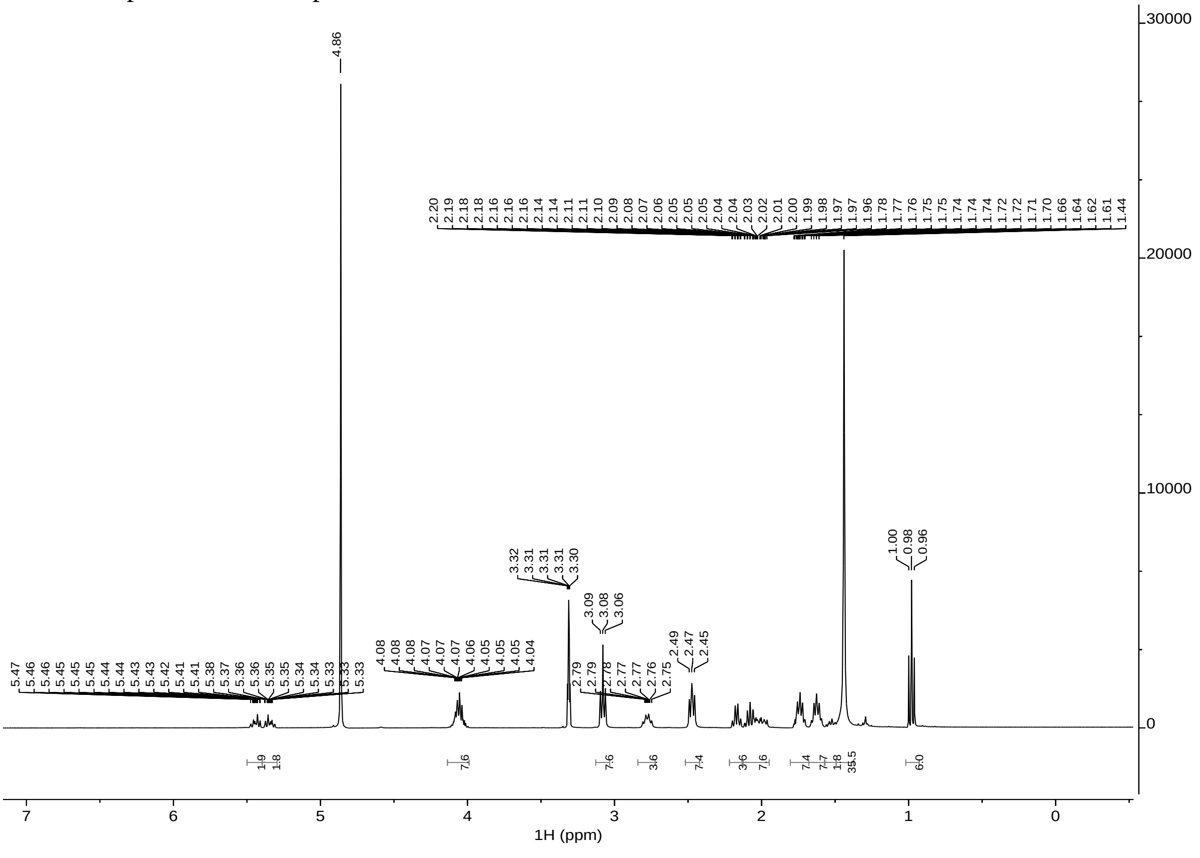

^{13}C NMR spectrum of compound **S99**

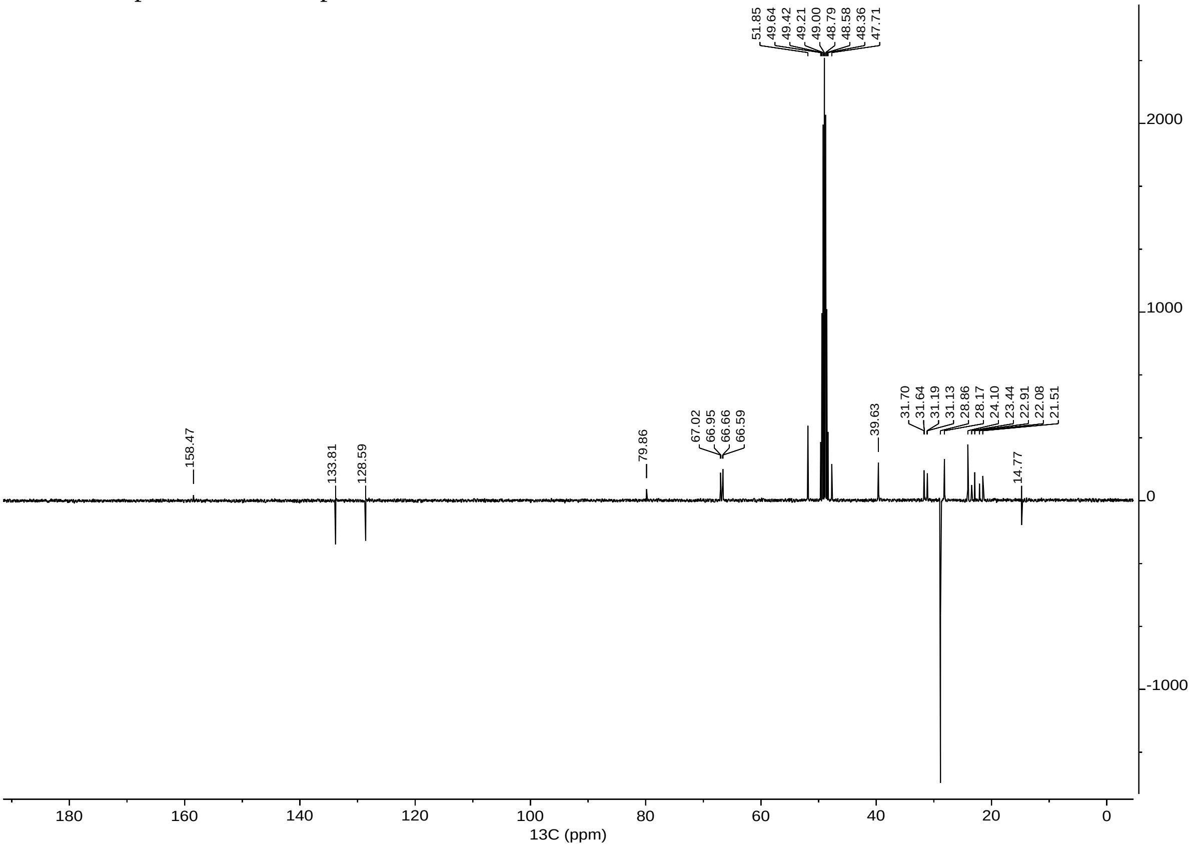

^{31}P{^{1}H} NMR spectrum of compound **S99**

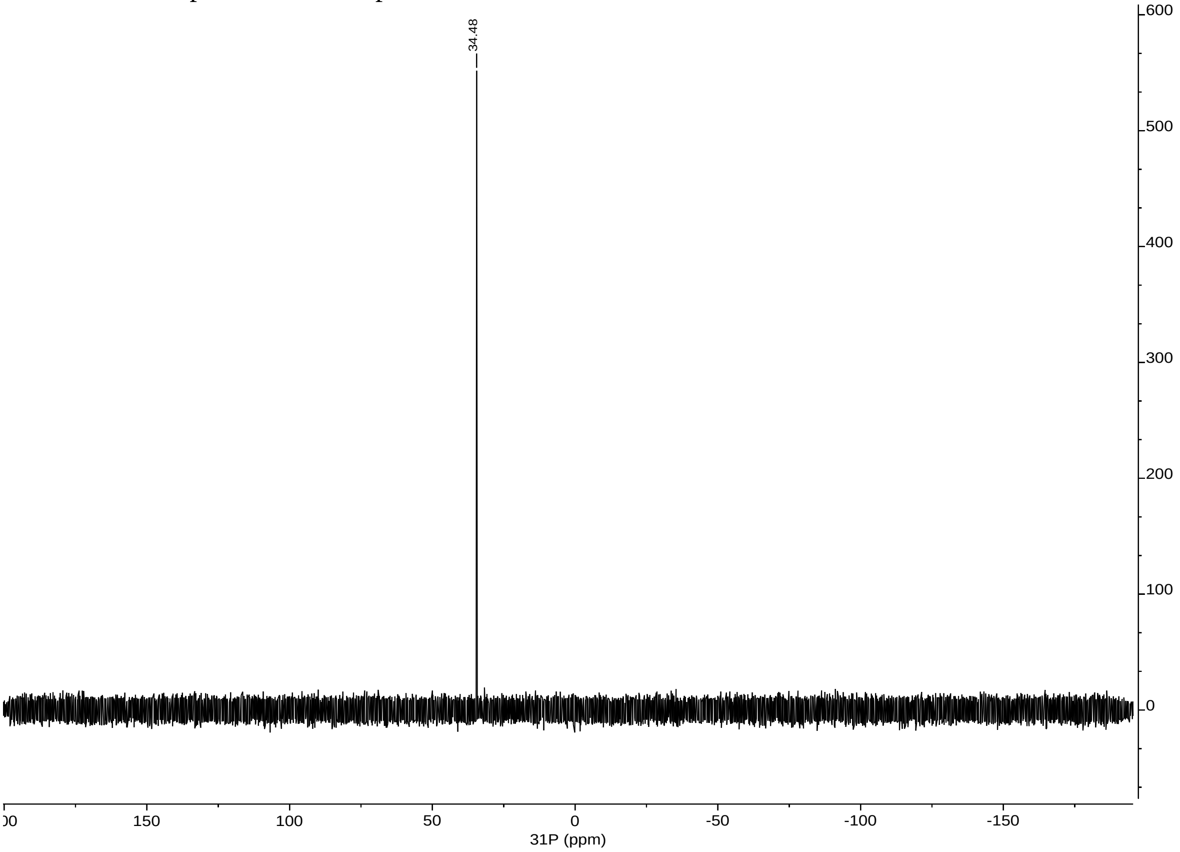

***N,N',N'',N'''*-Tetrakis(*tert*-butyloxycarbonyl) bis((*Z*)-oct-3-en-1-yl) pentane-1,5-diyl bis((2-(bis(3-aminopropyl)amino)ethyl)phosphonate) (S100)**

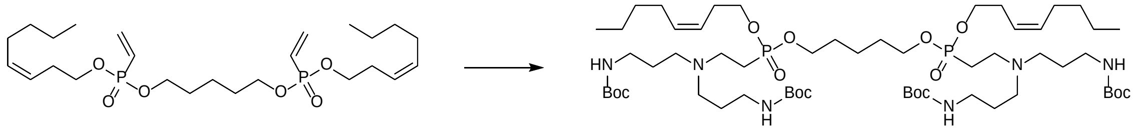

The title compound was prepared according to general method **D** from vinylphosphonate dimer **S51** (0.54 g, 1.07 mmol) and bis(3-*tert*-butyloxycarbonylaminopropyl)amine (1.42, 4.28 mmol) in 54% yield (0.68 g, 0.58 mmol) as a colourless oil.

^{1}H NMR (400 MHz, CD_3OD): 5.58–5.51 (m, 2H, $CH_3(CH_2)_3CH$), 5.45–5.37 (m, 2H, $CHCH_2CH_2O$), 4.10–3.98 (m, 8H, CH_2O), 3.08 (t, 8H, J = 6.8 Hz, CH_2NH), 2.81–2.74 (m, 4H, PCH_2CH_2), 2.49–2.42 (m, 12H, $CH_2(CH_2)_2NH$, $CHCH_2CH_2O$), 2.12–2.06 (m, 4H, $CH_3(CH_2)_2CH_2$), 2.04–1.95 (m, 4H, PCH_2), 1.77–1.70 (m, 4H, $OCH_2CH_2CH_2CH_2CH_2O$), 1.66–1.61 (m, 8H, CH_2CH_2NH), 1.55–1.52 (m, 2H, $O(CH_2)_2CH_2(CH_2)_2O$), 1.44 (s, 36H, $C(CH_3)_3$), 1.38–1.33 (m, 8H, $CH_3(CH_2)_2$), 0.95–0.90 (m, 6H, CH_3CH_2).

^{13}C NMR (101 MHz, CD_3OD): 158.46 (NCOO), 134.15 ($CH_3(CH_2)_3CH$), 125.37 ($CHCH_2CH_2O$), 79.87 ($C(CH_3)_3$, 66.95 (d, J = 7.1 Hz, $CHCH_2CH_2O$), 66.75 (d, J = 7.2 Hz, $OCH_2(CH_2)_3CH_2O$), 47.70 (PCH_2CH_2), 51.87 ($CH_2(CH_2)_2NH$), 39.63 (CH_2NH), 32.97 ($CH_3CH_2CH_2$), 31.14 (d, J = 6.5 Hz, $OCH_2CH_2CH_2CH_2CH_2O$), 29.72 (d, J = 6.0 Hz, $CHCH_2CH_2O$), 28.86 ($C(CH_3)_3$), 28.16 ($CH_3(CH_2)_2CH_2CH$), 28.11 (CH_2CH_2NH), 23.40 (CH_3CH_2), 22.90 ($O(CH_2)_2CH_2(CH_2)_2O$), 22.80 (d, J = 136.8 Hz, PCH_2), 14.40 (CH_3CH_2).

^{31}P{^{1}H} NMR (162 MHz, CD_3OD): 34.43.

IR ν_{max} (KBr) 3328 (m), 3005 (m), 2960 (s), 2930 (s), 2871 (m), 2815 (w), 1712 (vs), 1695 (s, sh), 1525 (s), 1456 (m), 1390 (m), 1365 (s), 1251 (vs), 1175 (vs), 1054 (s), 1007 (s), 970 (m, sh), 938 (m, sh).

HR-MS(ESI^{+}): For $C_{57}H_{114}O_{14}N_6P_2$ $(M+2H)^{2+}$ $m/2z$ calculated 584.39286, found 584.39368.

^{1}H NMR spectrum of compound **S100**

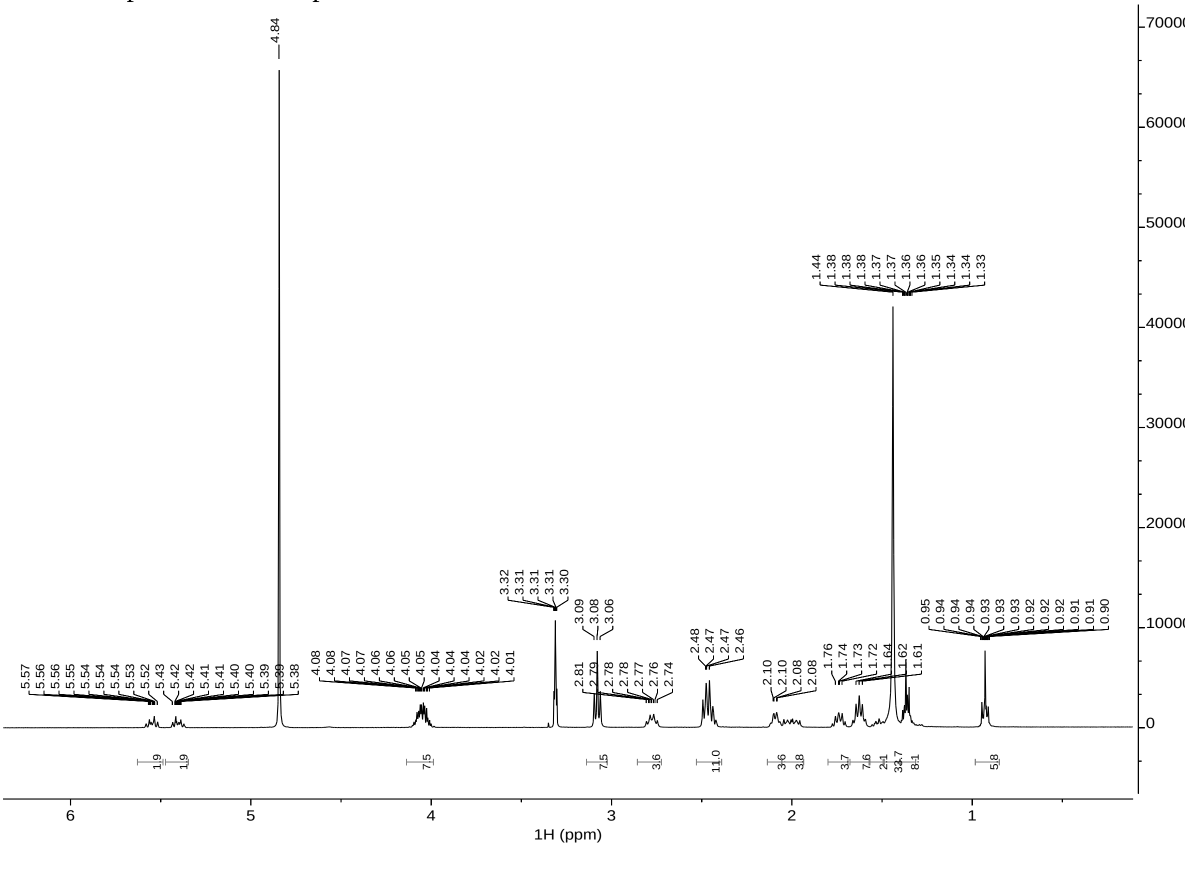

^{13}C NMR spectrum of compound **S100**

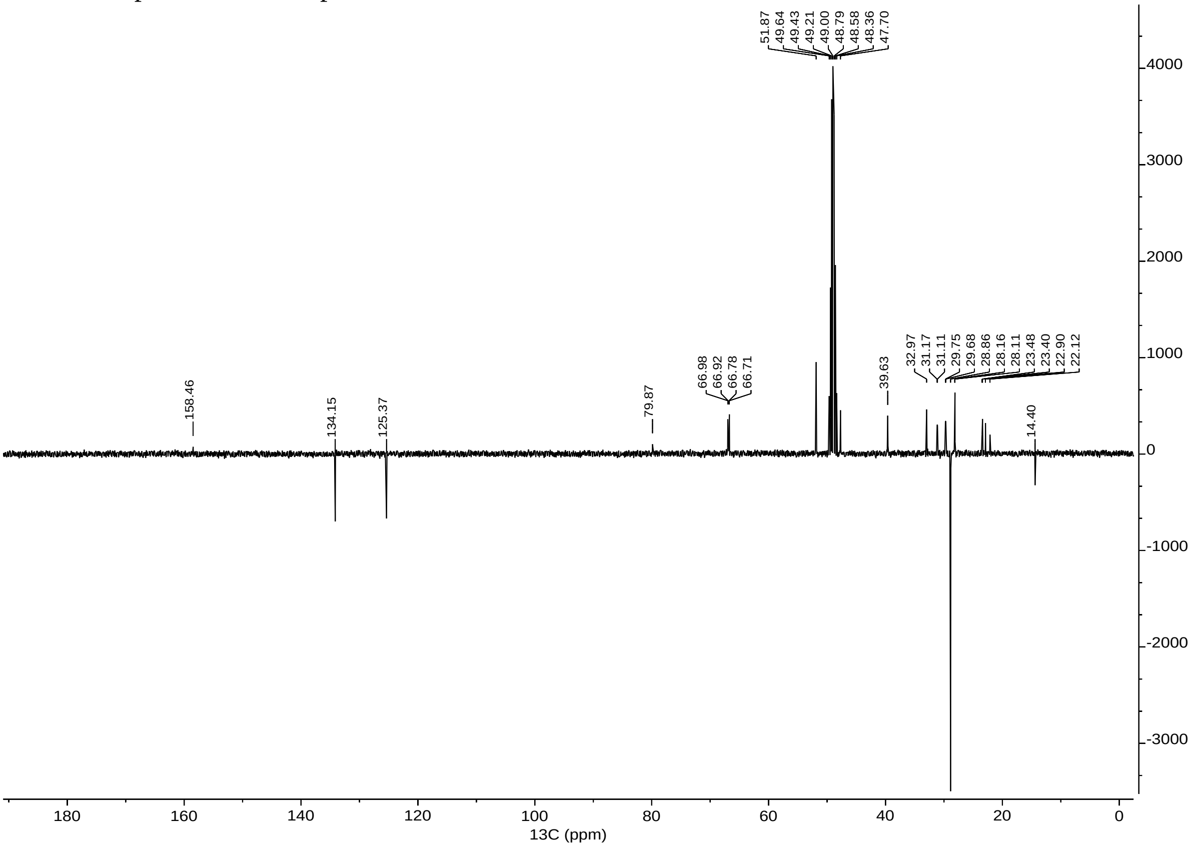

^{31}P{^{1}H} NMR spectrum of compound **S100**

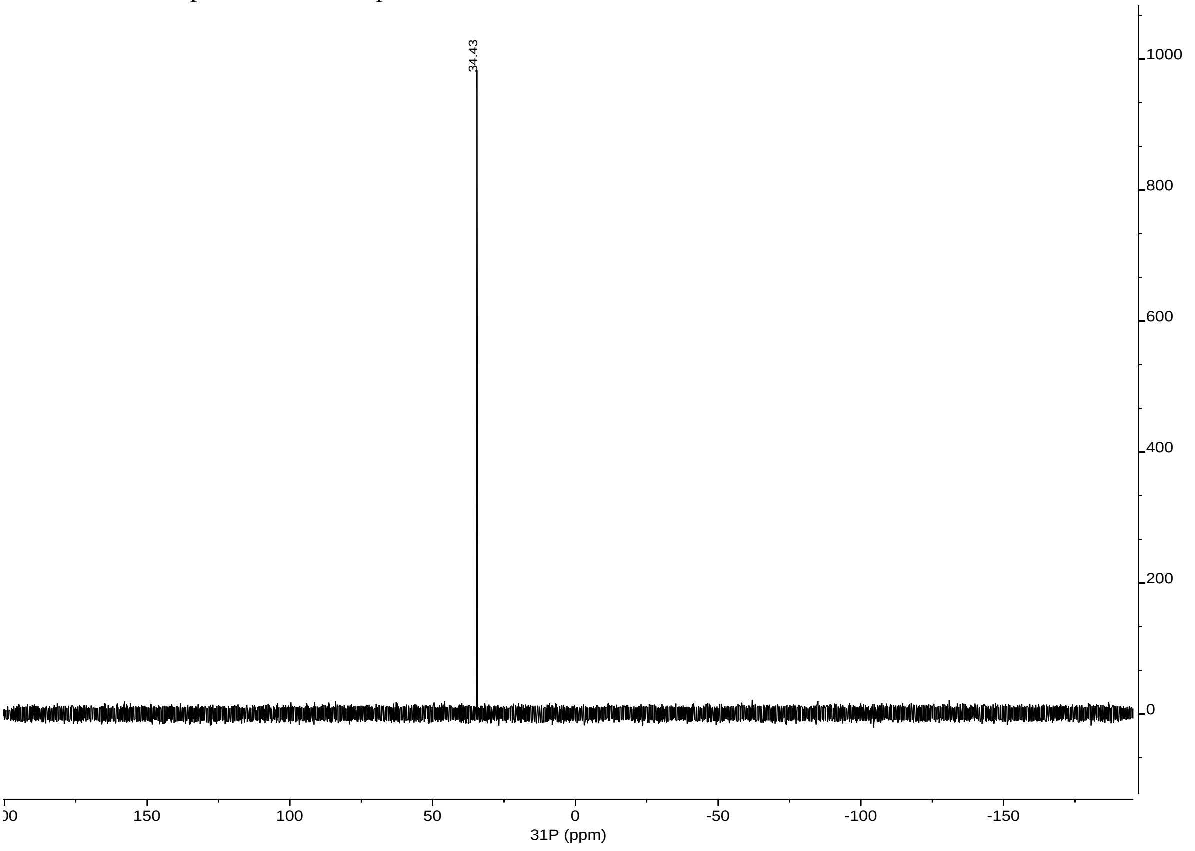

***N,N',N'',N'''*-Tetrakis(*tert*-butyloxycarbonyl) bis((*Z*)-non-3-en-1-yl) pentane-1,5-diyl bis((2-(bis(3-aminopropyl)amino)ethyl)phosphonate) (S101)**

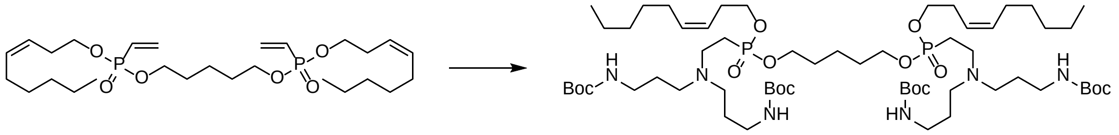

The title compound was prepared according to general method **D** from vinylphosphonate dimer **S52** (0.75 g, 1.41 mmol) and bis(3-*tert*-butyloxycarbonylaminopropyl)amine (1.40 g, 4.21 mmol) in 66% yield (1.10 g, 0.92 mmol) as a colourless oil.

^{1}H NMR (401 MHz, CD_3OD): 5.59–5.52 (m, 2H, $CH_3(CH_2)_4CH$), 4.10–3.98(m, 8H, CH_2O), 3.08 (t, 8H, J = 6.8 Hz, CH_2NH), 2.80–2.74 (m, 4H, PCH_2CH_2), 2.49–2.42 (m, 12H, $CH_2(CH_2)_2NH$, $CHCH_2CH_2O$), 2.11–1.95 (m, 8H, PCH_2, $CH_3(CH_2)_3CH_2$), 1.77–1.70 (m, 4H, $CH_2CH_2CH_2O$), 1.62 (p, 8H, J = 6.9 Hz, CH_2CH_2NH), 1.55–1.52 (m, 2H, $CH_2(CH_2)_2O$), 1.44 (s, 36H, $C(CH_3)_3$, 1.40–1.29 (m, 12H, $CH_3(CH_2)_3$), 0.94–0.90 (m, 6H, CH_3CH_2).

^{13}C NMR (101 MHz, CD_3OD): 158.47 (NCOO), 134.20 ($CH_3(CH_2)_4CH$), 125.37 ($CH(CH_2)_2O$), 79.86 ($C(CH_3)_3$), 66.94, 66.75 (d, J = 6.0 Hz, CH_2O), 51.86 ($CH_2(CH_2)_2NH$), 47.68 (PCH_2CH_2), 39.64 (CH_2NH), 32.68 ($CH_3CH_2CH_2$), 31.15 (d, J = 6.2 Hz, $OCH_2CH_2CH_2CH_2CH_2O$), 30.45 ($CH_3(CH_2)_2CH_2$), 29.73 (d, J = 6.4 Hz, $CHCH_2CH_2O$), 28.86 ($C(CH_3)_3$, 28.38 ($CH_3(CH_2)_3CH_2$), 28.18 (CH_2CH_2NH), 23.67 (CH_3CH_2), 22.91 ($O(CH_2)_2CH_2(CH_2)_2O$), 22.79 (d, J = 136.8 Hz, PCH_2), 14.49 (CH_3CH_2O).

^{31}P{^{1}H} NMR (162 MHz, CD_3OD): 34.74.

IR ν_{max} (KBr) 3455 (m), 3369 (w), 2980 (s), 2960 (s), 2932 (s), 2872 (m), 2860 (m), 2825 (w), 1707 (vs), 1507 (s), 1467 (m), 1456 (m), 1439 (w), 1380 (s), 1249 (s), 1071 (m), 1055 (s), 1039 (m), 1010 (s), 998 (s).

HR-MS(ESI^{+}): For $C_{59}H_{116}O_{14}N_6NaP_2$ $(M+Na)^{+}$ m/z calculated 1217.79170, found 1217.79198.

^{1}H NMR spectrum of compound **S101**

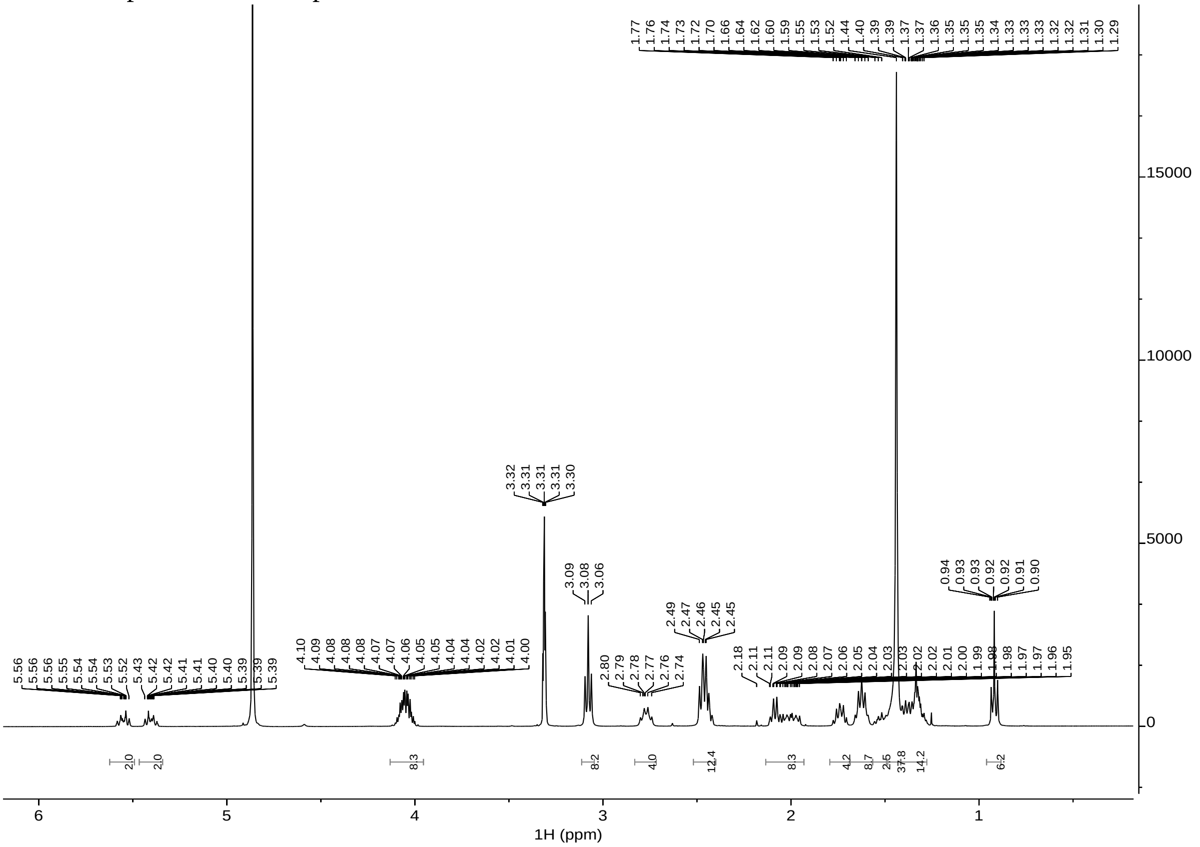

^{13}C NMR spectrum of compound **S101**

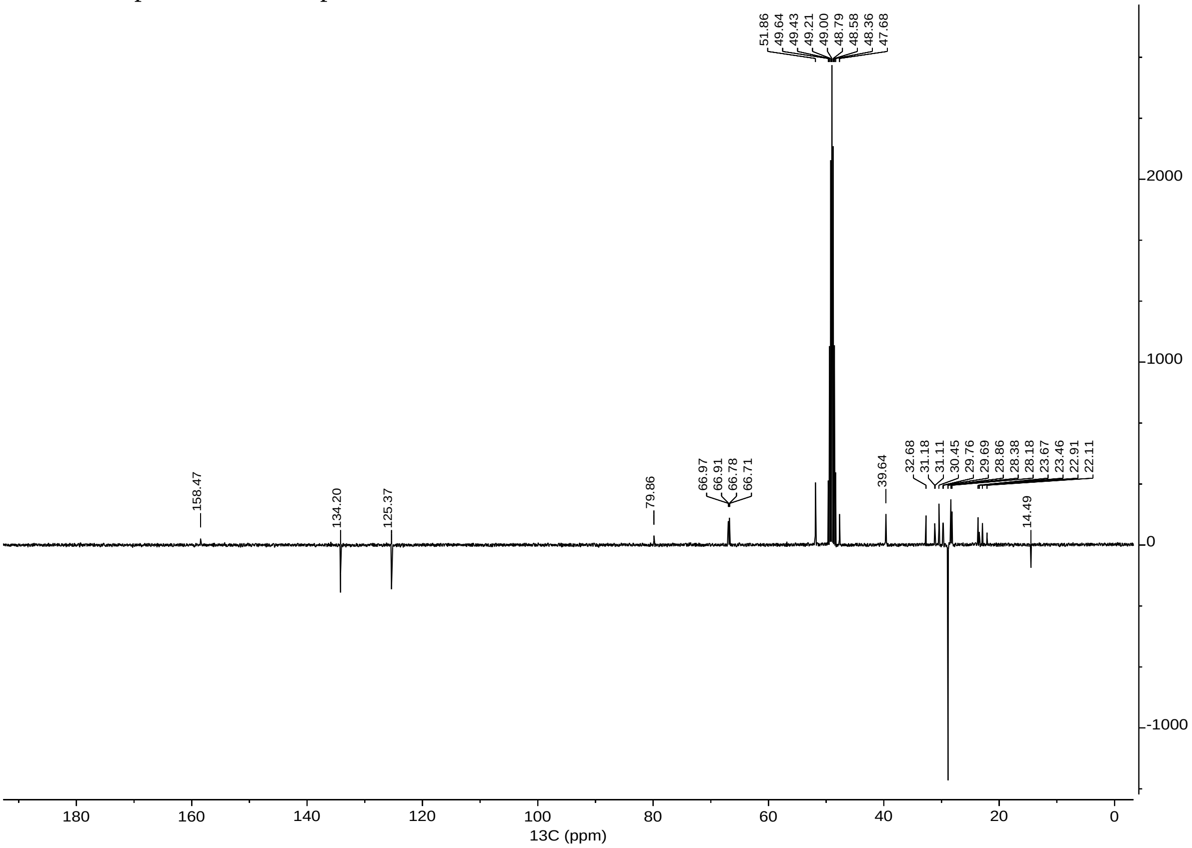

$^{31}P\{^1H\}$ NMR spectrum of compound **S101**

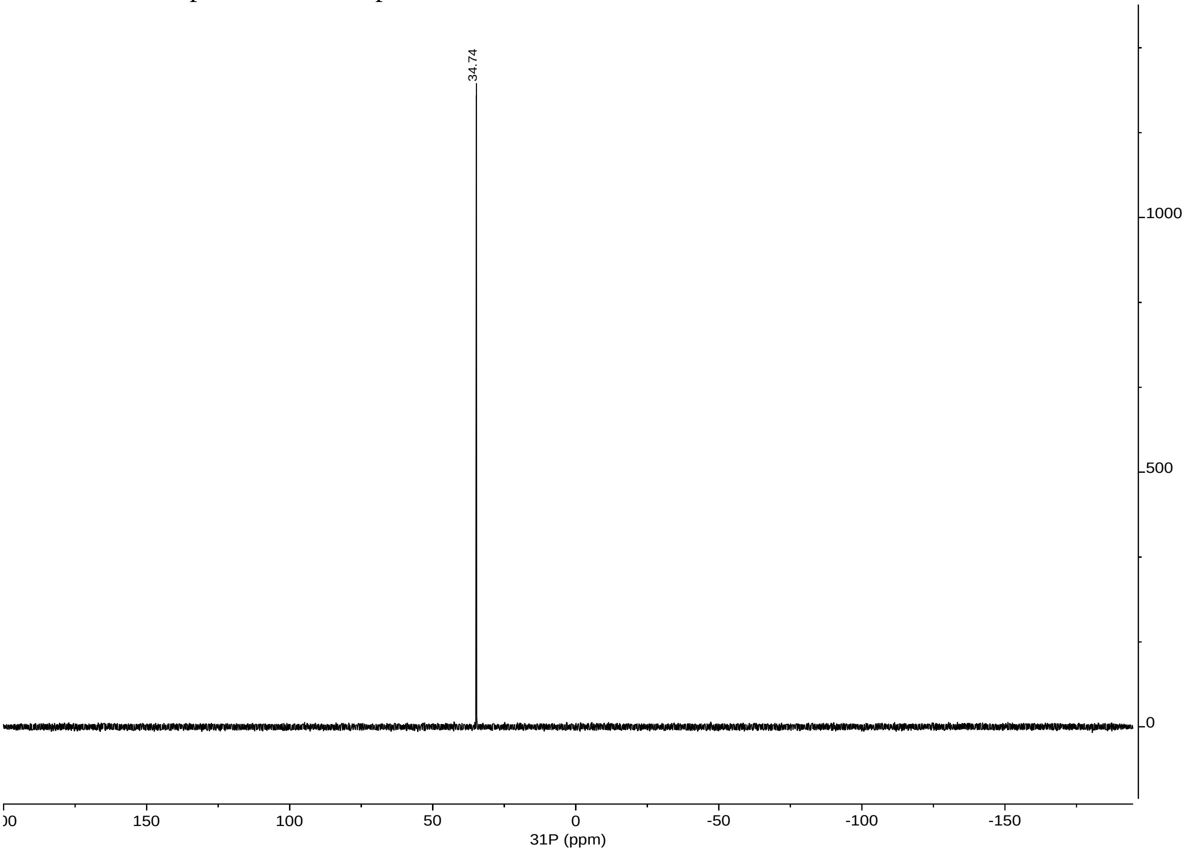

***N,N',N'',N'''*-Tetrakis(*tert*-butyloxycarbonyl) bis((*Z*)-hept-3-en-1-yl) hexane-1,6-diyl bis((2-(bis(3-aminopropyl)amino)ethyl)phosphonate) (S102)**

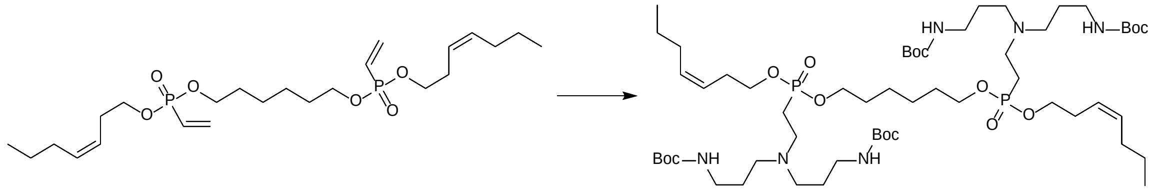

The title compound was prepared according to general method **D** from vinylphosphonate dimer **S55** (0.65 g, 1.33 mmol) and bis(3-*tert*-butyloxycarbonylaminopropyl)amine (1.81 g, 5.46 mmol) in 51% yield (0.77 g, 0.68 mmol) as a colourless oil.

^{1}H NMR (400 MHz, CD_3OD): 5.58–5.51 (m, 2H, C*H*CHCH$_2$CH$_2$O), 5.45–5.38 (m, 2H, CHC*H*CH$_2$CH$_2$O), 4.22–4.00 (m, 8H, C*H*$_2$O), 3.08 (t, 8H, *J* = 6.7 Hz, C*H*$_2$NH), 2.80–2.74 (m, 4H, PCH$_2$C*H*$_2$), 2.49–2.42 (m, 12H, NC*H*$_2$(CH$_2$)$_2$NH, CHCHC*H*$_2$CH$_2$O), 2.10–2.06 (m, 4H, CH$_3$CH$_2$C*H*$_2$), 1.95–2.04 (m, 4H, PC*H*$_2$), 1.73–1.68 (m, 4H, OCH$_2$C*H*$_2$(CH$_2$)$_2$C*H*$_2$CH$_2$O), 1.66–1.60 (m, 8H, C*H*$_2$CH$_2$NH), 1.47–1.37 (m, 44H, CH$_3$C*H*$_2$, O(CH$_2$)$_2$(C*H*$_2$)$_2$(CH$_2$)$_2$O, C(C*H*$_3$)$_3$), 0.95–0.92 (m, 6H, C*H*$_3$CH$_2$).

^{13}C NMR (101 MHz, CD_3OD): 158.46 (N*C*OO), 133.93 (CH$_3$(CH$_2$)$_3$*C*H), 125.57 (*C*HCH$_2$CH$_2$O), 79.85 (*C*(CH$_3$)$_3$, 67.08 (d, *J* = 7.2 Hz, CHCH$_2$*C*H$_2$O), 66.72 (d, *J* = 7.0 Hz, O*C*H$_2$(CH$_2$)$_6$*C*H$_2$O), 47.69 (PCH$_2$*C*H$_2$), 51.88 (*C*H$_2$(CH$_2$)$_2$NH), 39.64 (*C*H$_2$NH), 31.25 (d, *J* = 6.3 Hz, OCH$_2$*C*H$_2$(CH$_2$)$_2$*C*H$_2$CH$_2$O), 30.46 (CH$_3$CH$_2$*C*H$_2$), 29.72 (d, *J* = 6.3 Hz, CH*C*H$_2$CH$_2$O), 28.86 (C(*C*H$_3$)$_3$), 28.17 (*C*H$_2$CH$_2$NH), 26.32 (O(CH$_2$)$_2$(*C*H$_2$)$_2$(CH$_2$)$_2$O), 23.82 (CH$_3$*C*H$_2$), 22.80 (d, *J* = 137.8 Hz, P*C*H$_2$), 14.18 (*C*H$_3$CH$_2$).

^{31}P{^{1}H} NMR (162 MHz, CD_3OD): 34.42.

IR ν_{max} (KBr) 3325 (m), 3005 (w), 2962 (m), 2932 (m), 2870 (m), 2814 (w), 1711 (vs), 1693 (vs, sh), 1524 (m), 1456 (m), 1390 (m), 1365 (s), 1251 (s), 1174 (vs), 1066 (s), 1053 (s), 1004 (s), 970 (m, sh), 722 (w), 469 (w).

HR-MS(ESI$^+$): For $C_{56}H_{112}O_{14}N_6P_2$ $(M+2H)^{2+}$ *m*/2*z* calculated 557.38504, found 557.38529.

^{1}H NMR spectrum of compound **S102**

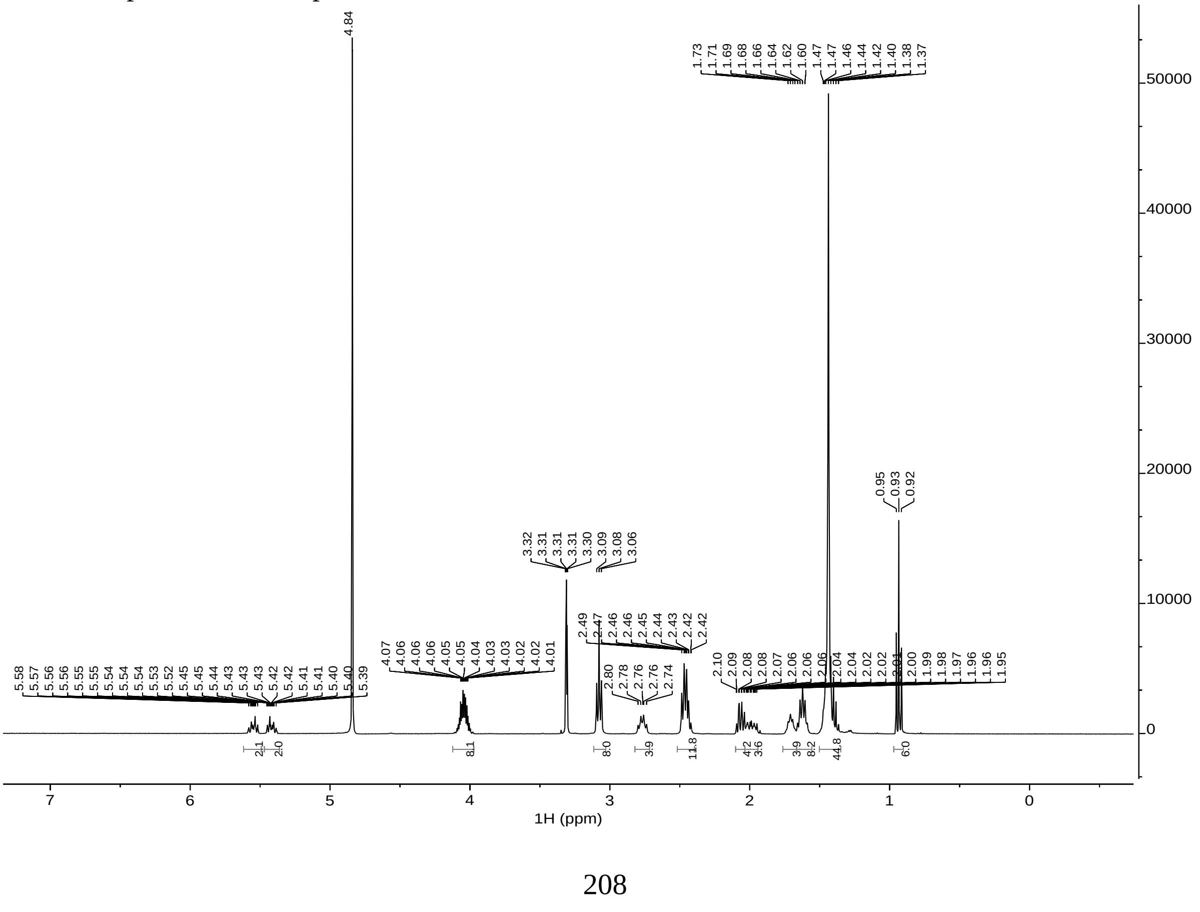

^{13}C NMR spectrum of compound **S102**

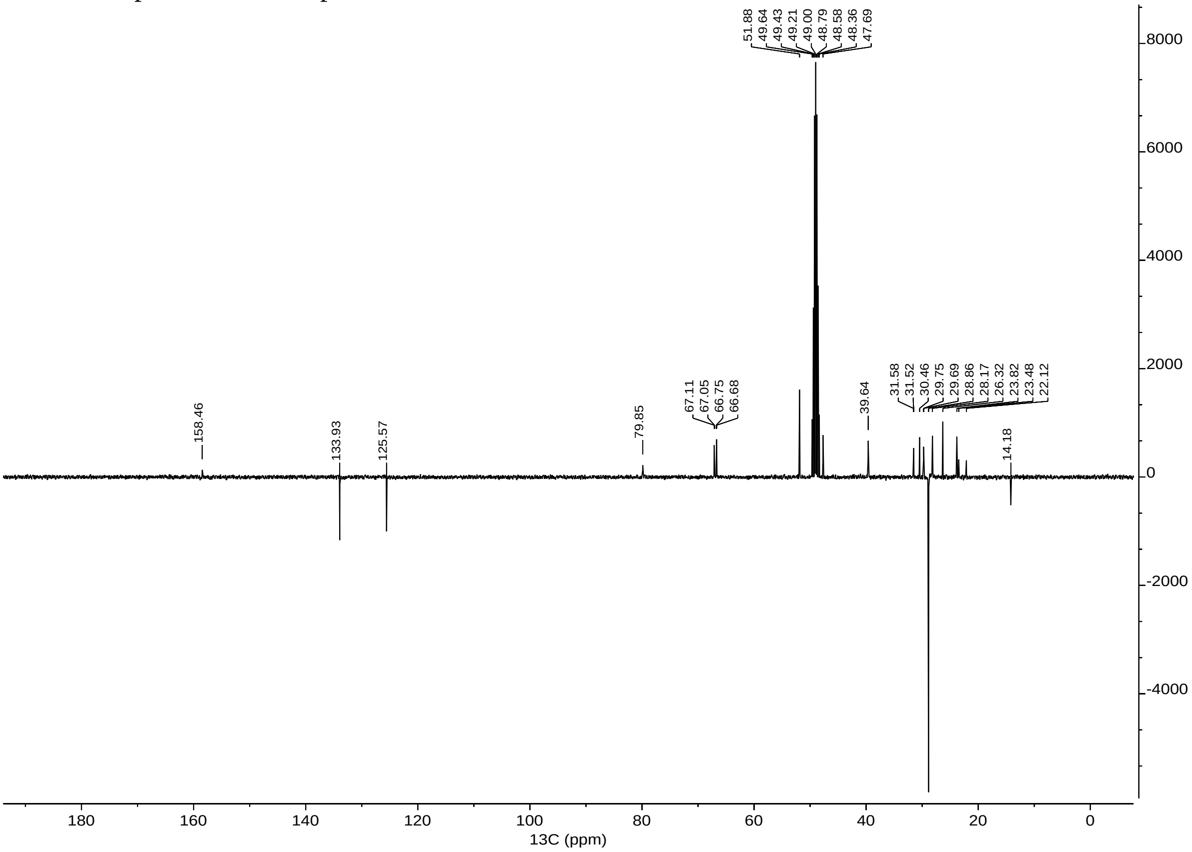

^{31}P{^{1}H} NMR spectrum of compound **S102**

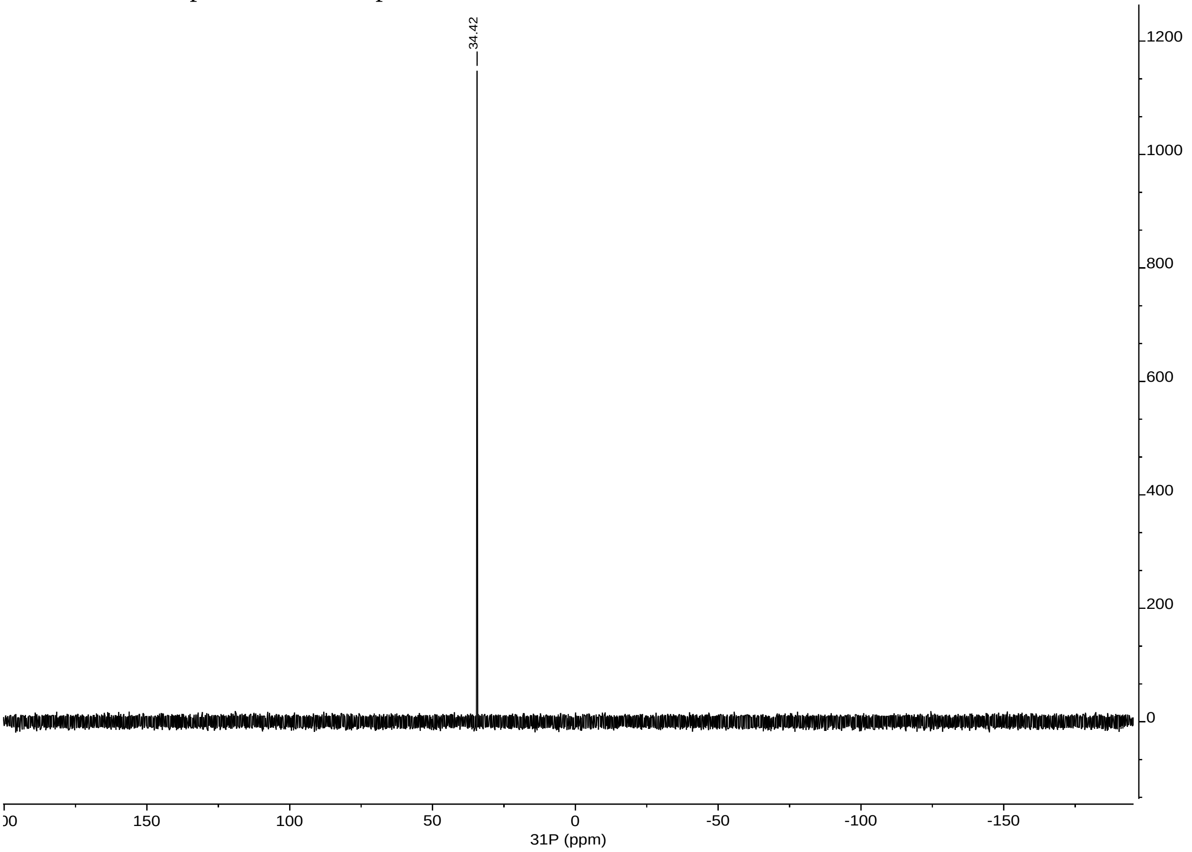

***N,N',N'',N'''*-Tetrakis(*tert*-butyloxycarbonyl) bis((*Z*)-hept-4-en-1-yl) hexane-1,6-diyl bis((2-(bis(3-aminopropyl)amino)ethyl)phosphonate) (S103)**

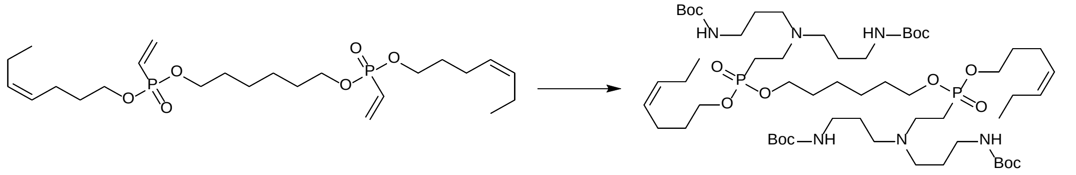

The title compound was prepared according to general method **D** from vinylphosphonate dimer **S56** (0.31 g, 0.62 mmol) and bis(3-*tert*-butyloxycarbonylaminopropyl)amine (0.82 g, 2.49 mmol) in 80% yield (0.57 g, 0.49 mmol) as a colourless oil.

^{1}H NMR (401 MHz, CD_3OD): 5.48–5.41 (m, 2H, $CH_3CH_2C\mathit{H}$), 5.38–5.31 (m, 2H, $C\mathit{H}(CH_2)_3O$), 4.10–4.01 (m, 8H, $C\mathit{H}_2O$), 3.08 (t, 8H, *J* = 6.7 Hz, $C\mathit{H}_2NH$), 2.81–2.75 (m, 4H, $PCH_2C\mathit{H}_2$), 2.47 (t, 8H, *J* = 7.6 Hz, $C\mathit{H}_2(CH_2)_2NH$), 2.20–1.96 (m, 12H, $PC\mathit{H}_2$, $CH_3C\mathit{H}_2$, $CHC\mathit{H}_2(CH_2)_2O$), 1.77–1.70 (m, 8H, $CHCH_2C\mathit{H}_2CH_2O$, $OCH_2C\mathit{H}_2(CH_2)_2C\mathit{H}_2CH_2O$), 1.66–1.59 (m, 8H, $C\mathit{H}_2CH_2NH_2$), 1.48–1.44 (m, 40H, $C(C\mathit{H}_3)_3$, $O(CH_2)_2(C\mathit{H}_2)_2(CH_2)_2O$), 0.98 (t, 6H, *J* = 7.4 Hz, $C\mathit{H}_3CH_2$).

^{13}C NMR (101 MHz, CD_3OD): 158.48 (N*C*OO), 133.80 ($CH_3CH_2\mathit{C}HCHCH_2CH_2CH_2O$), 128.59 ($CH_3CH_2CH\mathit{C}HCH_2CH_2CH_2O$), 79.86 ($\mathit{C}(CH_3)_3$), 67.13, 66.62 (d, *J* = 7.2 Hz, $CH_3CH_2CHCHCH_2CH_2\mathit{C}H_2O$, $O\mathit{C}H_2CH_2CH_2CH_2CH_2\mathit{C}H_2O$), 51.86 ($N\mathit{C}H_2CH_2CH_2NH$), 47.71 ($PCH_2\mathit{C}H_2N$), 39.62 ($NCH_2CH_2\mathit{C}H_2NH$), 31.66, 31.57 (d, *J* = 6.0 Hz, $CH_3CH_2CHCHCH_2\mathit{C}H_2CH_2O$, $OCH_2\mathit{C}H_2CH_2CH_2\mathit{C}H_2CH_2O$), 28.85 ($C(\mathit{C}H_3)_3$, 28.16 ($NCH_2\mathit{C}H_2CH_2NH$), 26.34 ($OCH_2CH_2\mathit{C}H_2\mathit{C}H_2CH_2CH_2O$), 24.10 ($CH_3CH_2CHCH\mathit{C}H_2CH_2CH_2O$), 22.75 (d, *J* = 136.8 Hz, $P\mathit{C}H_2CH_2N$), 21.51 ($CH_3\mathit{C}H_2CHCHCH_2CH_2CH_2O$), 14.47 ($\mathit{C}H_3CH_2CHCHCH_2CH_2CH_2O$).

^{31}P{^{1}H} NMR (162 MHz, CD_3OD): 34.67.

IR ν_{max} (KBr) 3455 (m), 3369 (w), 2979 (s), 2935 (m), 2872 (m), 2824 (m), 1707 (vs), 1507 (s), 1467 (m), 1456 (m), 1393 (m), 1367 (s), 1249 (s), 1080–1000 (m), 464 (w).

HR-MS(ESI^{+}): For $C_{56}H_{111}O_{14}N_6P_2$ $(M+H)^{+}$ *m*/*z* calculated 356.25671, found 356.25693.

^{1}H NMR spectrum of compound **S103**

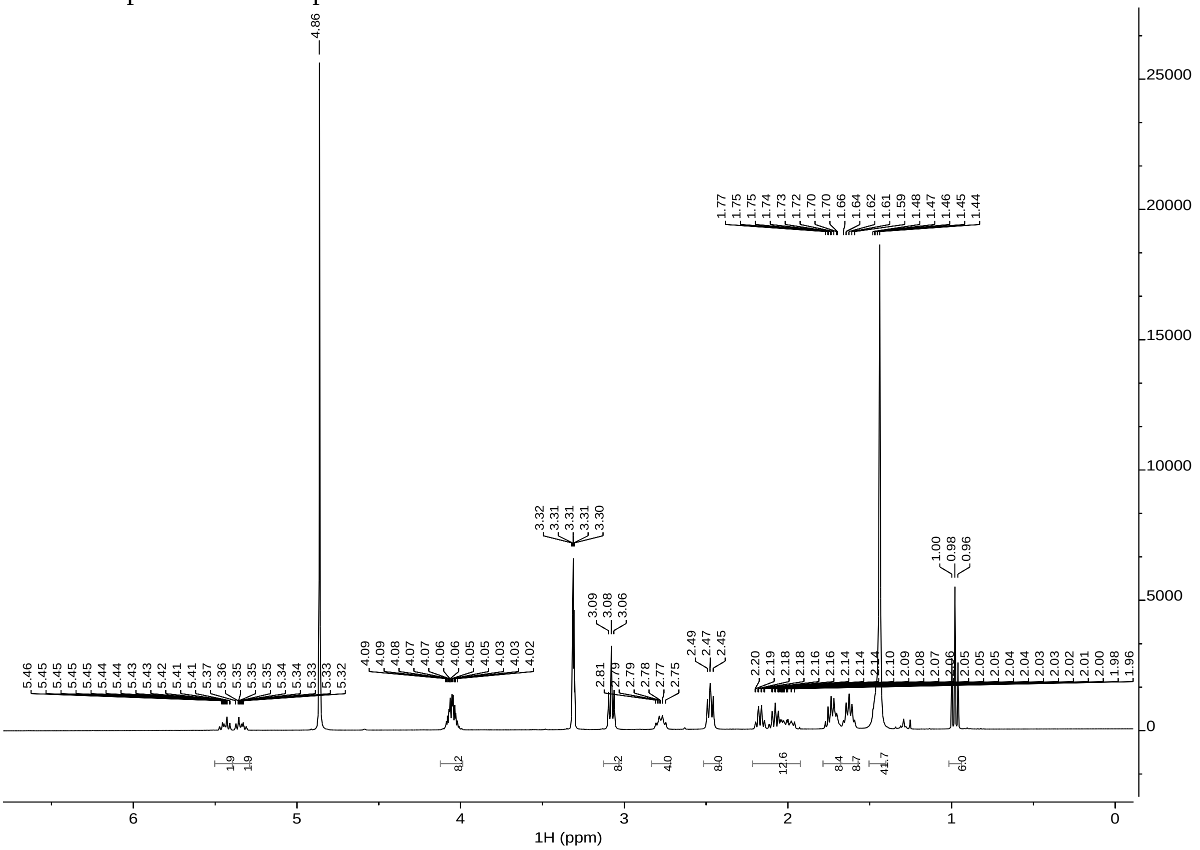

^{13}C NMR spectrum of compound **S103**

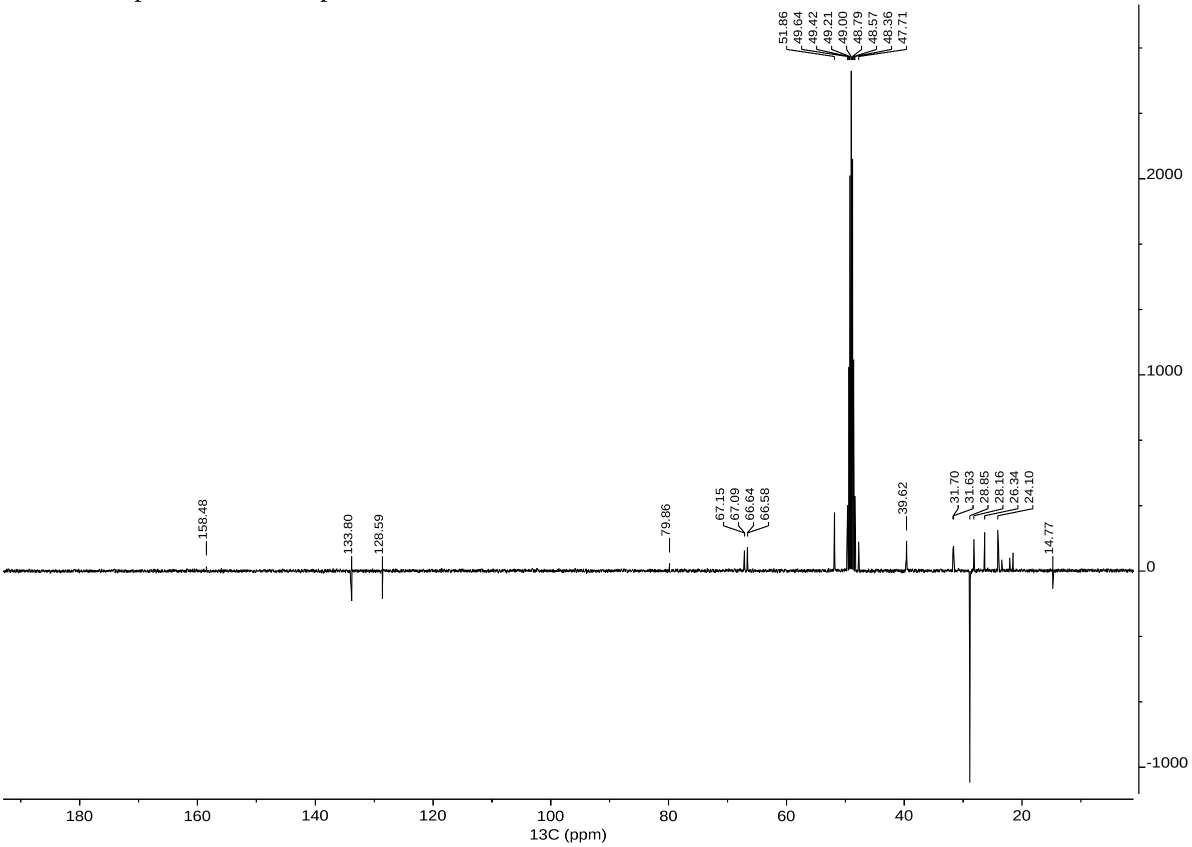

$^{31}P\{^{1}H\}$ NMR spectrum of compound **S103**

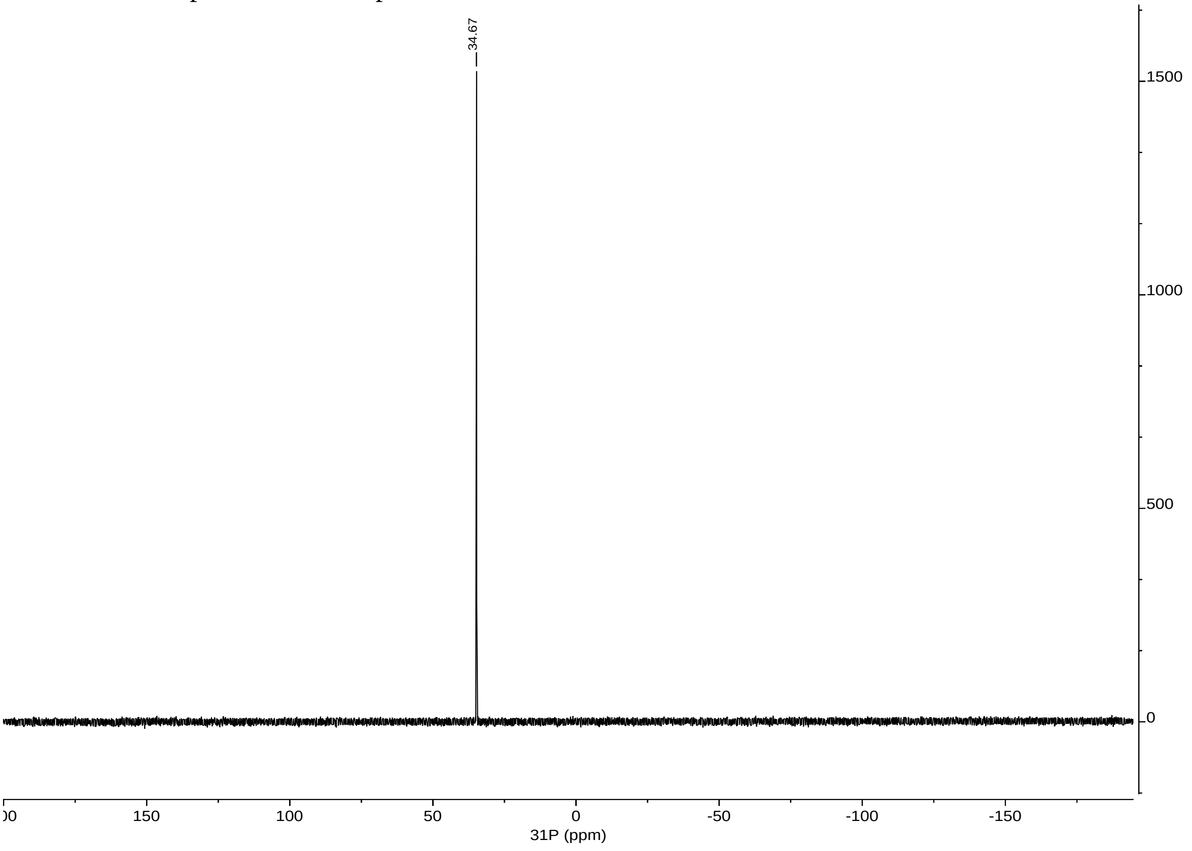

***N,N',N'',N'''*-Tetrakis(*tert*-butyloxycarbonyl) hexane-1,6-diyl dioctyl bis((2-(bis(2-aminoethyl)-amino)ethyl)phosphonate) (S104)**

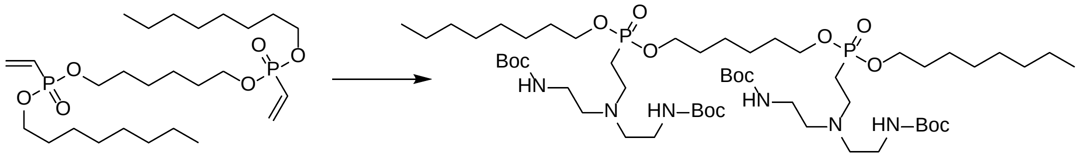

The title compound was prepared according to general method **D** from vinylphosphonate dimer **S57** (1.06 g, 2.03 mmol) and bis(2-*tert*-butyloxycarbonylaminoethyl)amine (2.46 g, 8.11 mmol) in 40% yield (0.92 g, 0.82 mmol) as a colourless oil.

^{1}H NMR (401 MHz, CD_3OD): 4.09–4.01 (m, 8H, $CH_3(CH_2)_5CH_2CH_2O$, $OCH_2CH_2CH_2CH_2CH_2CH_2O$), 3.11 (t, 8H, J = 6.3 Hz, NCH_2CH_2NH), 2.80 (dt, 4H, J = 11.0, 7.5 Hz, PCH_2CH_2N), 2.55 (t, J = 6.3 Hz, 8H, NCH_2CH_2NH), 2.05–1.98 (m, 4H, PCH_2CH_2N), 1.75–1.67 (m, 8H, $CH_3(CH_2)_5CH_2CH_2O$, $OCH_2CH_2CH_2CH_2CH_2CH_2O$), 1.58–1.45 (m, 40H, $C(CH_3)_3$, $OCH_2CH_2CH_2CH_2CH_2CH_2O$), 1.34–1.29 (m, 20H, $CH_3(CH_2)_5CH_2CH_2O$), 0.93–0.89 (m, 6H, $CH_3(CH_2)_6CH_2O$).

^{13}C NMR (101 MHz, CD_3OD): 158.49 (NCOO), 79.91 ($C(CH_3)_3$), 67.23, 67.04 (d, J = 6.8 Hz, $CH_3(CH_2)_5CH_2CH_2O$, $OCH_2CH_2CH_2CH_2CH_2O$), 54.39 (NCH_2CH_2NH), 48.45 (PCH_2CH_2N), 39.46 (NCH_2CH_2NH), 32.99 ($CH_3CH_2CH_2(CH_2)_3CH_2CH_2O$), 31.65, 31.57 (d, J = 6.5 Hz, $OCH_2CH_2CH_2CH_2CH_2CH_2O$), 28.87 ($C(CH_3)_3$), 30.39, 30.27, 26.75 ($CH_3CH_2CH_2(CH_2)_3CH_2CH_2O$), 26.35 ($OCH_2CH_2CH_2CH_2CH_2CH_2O$), 23.96 (d, J = 136.8 Hz, PCH_2CH_2N), 23.74 ($CH_3CH_2(CH_2)_4CH_2CH_2O$), 14.49 ($CH_3(CH_2)_6CH_2O$).

^{31}P{^{1}H} NMR (162 MHz, CD_3OD): 34.78.

IR ν_{max} (KBr) 3452 (w), 3326 (w, br), 2980 (s), 2959 (s), 2871 (m), 2858 (m), 2827 (w), 1704 (vs), 1506 (s), 1468 (m), 1456 (m), 1393 (m), 1367 (s), 1248 (s), 1038 (m), 1000 (s), 984 (m).

HR-MS(ESI$^+$): For $C_{54}H_{111}O_{14}N_6P_2$ $(M+H)^+$ m/z calculated 1129.76280, found 1129.76343.

^{1}H NMR spectrum of compound **S104**

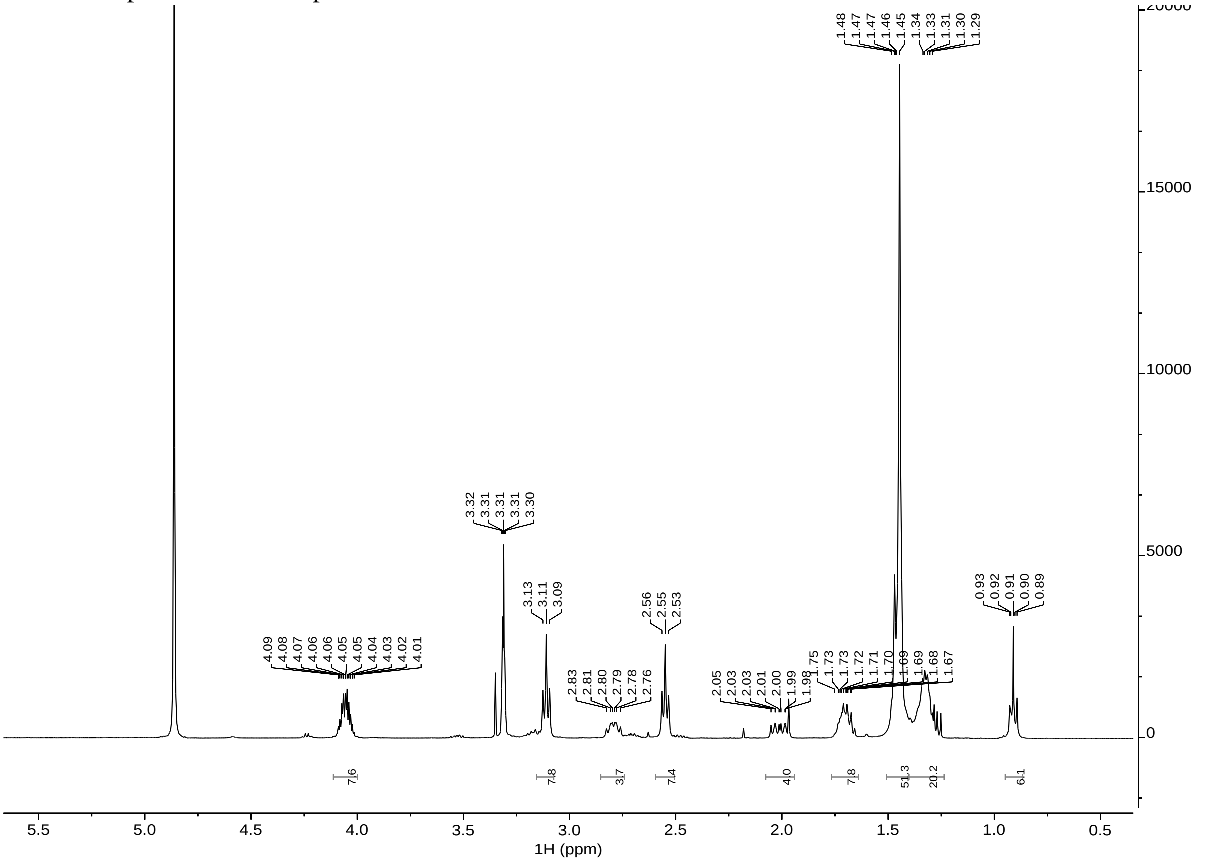

^{13}C NMR spectrum of compound **S104**

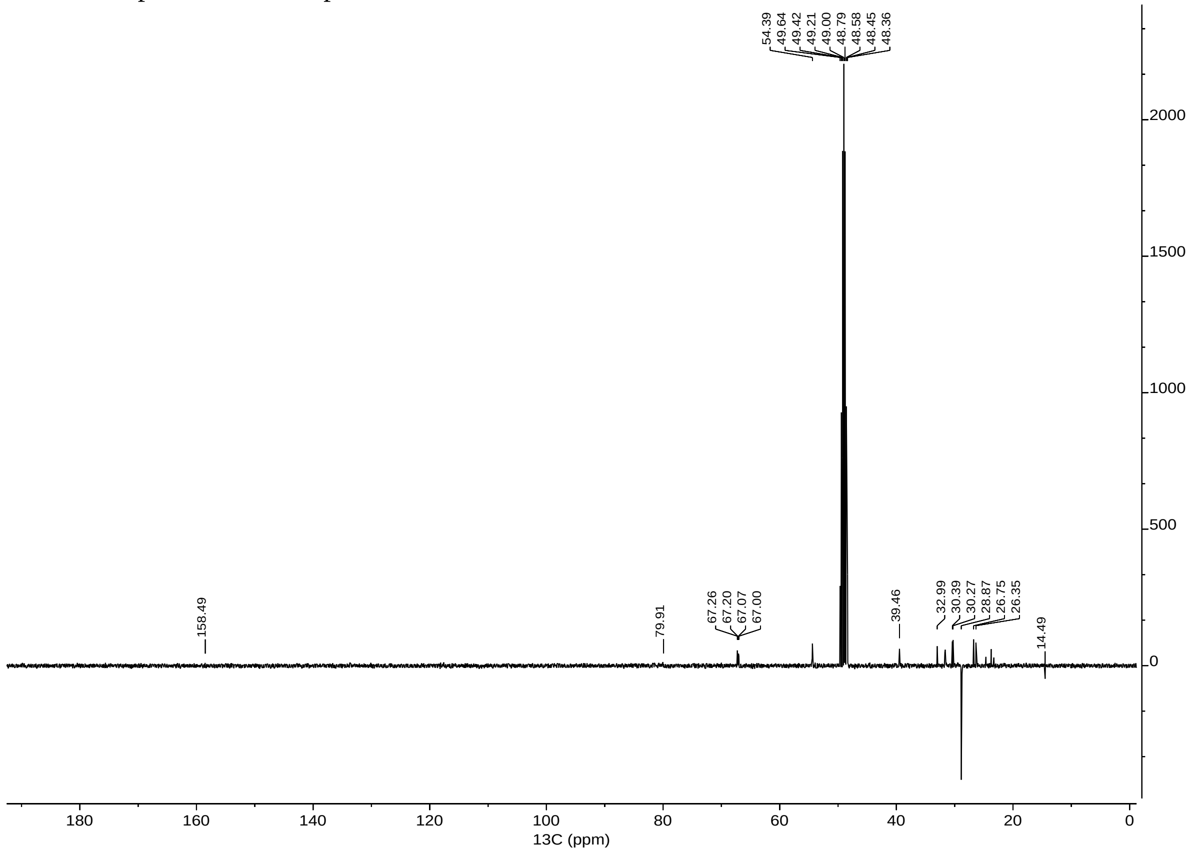

^{31}P{^{1}H} NMR spectrum of compound **S104**

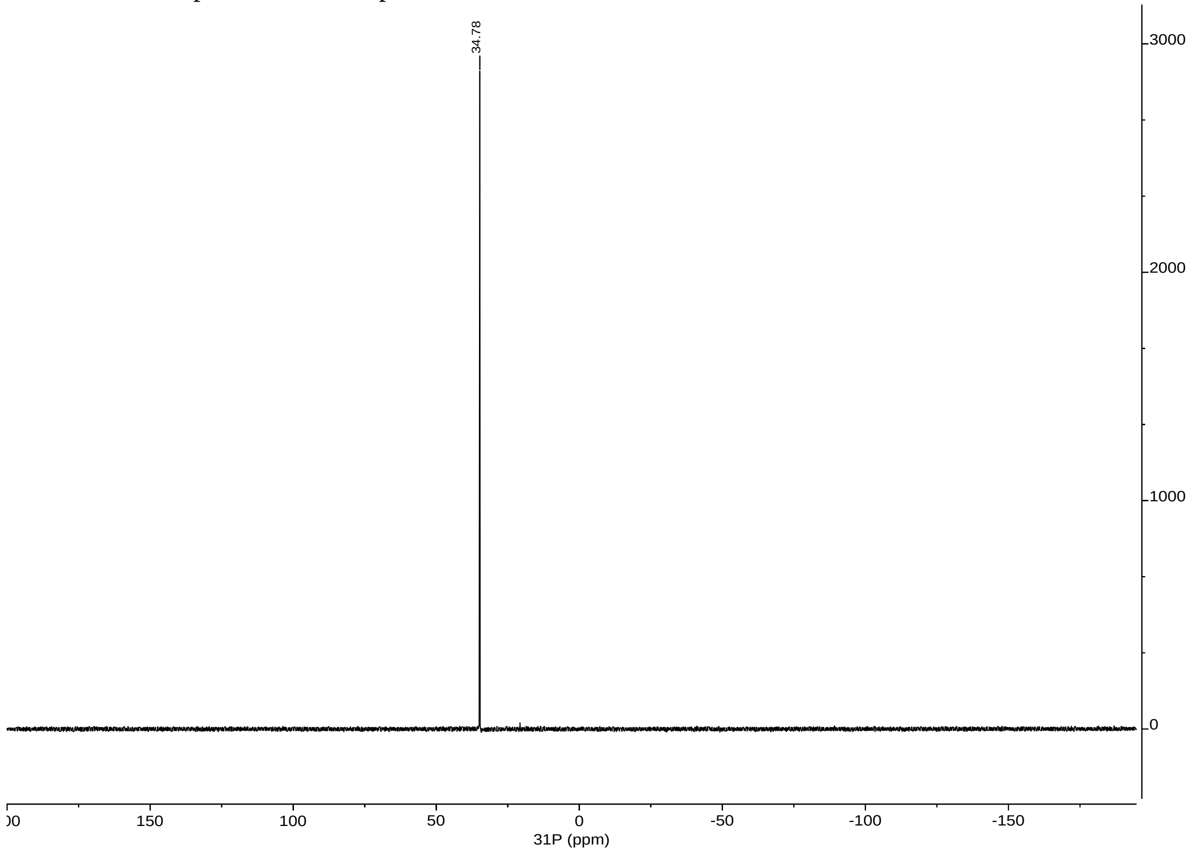

***N,N',N'',N'''*-Tetrakis(*tert*-butyloxycarbonyl) hexane-1,6-diyl bis((*Z*)-oct-3-en-1-yl) bis((2-(bis(3-aminopropyl)amino)ethyl)phosphonate) (S105)**

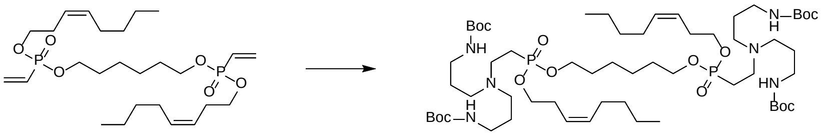

The title compound was prepared according to general method **D** from vinylphosphonate dimer **S59** (0.59 g, 1.14 mmol) and bis(3-*tert*-butyloxycarbonylaminopropyl)amine (1.50 g, 4.55 mmol) in 54% yield (0.87 g, 0.73 mmol) as a colourless oil.

^{1}H NMR (400 MHz, CD_3OD): 5.58–5.51 (m, 2H, $CH_3(CH_2)_3CH$), 5.43–5.36 (m, 2H, $CH(CH_2)_2O$), 4.09–4.00 (m, 8H, CH_2O), 3.08 (t, 8H, J = 6.7 Hz, CH_2NH), 2.80–2.74 (m, 4H, PCH_2CH_2), 2.49–2.42 (m, 12H, $CH_2(CH_2)_2NH$, $CHCH_2CH_2O$), 2.11–2.06 (m, 4H, $CH_3(CH_2)_2CH_2$), 2.04–1.94 (m, 4H, PCH_2), 1.72–1.68 (m, 4H, $OCH_2CH_2CH_2CH_2CH_2CH_2O$), 1.66–1.60 (m, 8H, CH_2CH_2NH), 1.47–1.6 (m, 4H, $O(CH_2)_2(CH_2)_2(CH_2)_2O$), 1.44 (s, 36H, $C(CH_3)_3$), 1.38–1.33 (m, 8H, $CH_3(CH_2)_2$), 0.94–0.91 (m, 6H, CH_3CH_2).

^{13}C NMR (101 MHz, CD_3OD): 158.47 (NCOO), 134.14 ($CH_3(CH_2)_3CH$), 125.37 ($CHCH_2CH_2O$), 79.86 ($C(CH_3)_3$, 67.09 (d, J = 7.0 Hz, $CHCH_2CH_2O$), 66.73 (d, J = 7.3 Hz, $OCH_2(CH_2)_4CH_2O$), 51.88 ($CH_2(CH_2)_2NH$), 47.69 (PCH_2CH_2), 39.63 (CH_2NH), 32.96 ($CH_3CH_2CH_2$), 31.55 (d, J = 7.0 Hz, $OCH_2CH_2(CH_2)_2CH_2CH_2O$), 29.71 (d, J = 6.4 Hz, $CHCH_2CH_2O$), 28.86 ($C(CH_3)_3$), 28.16 ($CH_3(CH_2)_2CH_2$), 28.11 (CH_2CH_2NH), 26.32 ($O(CH_2)_2(CH_2)_2(CH_2)_2O$), 23.40 ($CH_3CH_2$), 22.79 (d, J = 136.8 Hz, PCH_2), 14.40 (CH_3CH_2).

^{31}P{^{1}H} NMR (162 MHz, CD_3OD): 34.29.

IR ν_{max} (KBr) 3328 (m), 3005 (w), 2975 (m, sh), 2960 (m), 2931 (m), 2870 (m), 2815 (w), 1712 (vs), 1695 (vs), 1522 (s), 1457 (m), 1365 (s), 1251 (s), 1174 (vs), 1054 (m), 1004 (s), 970 (m, sh), 940 (m), 728 (m).

HR-MS(ESI$^+$): For $C_{58}H_{115}O_{14}N_6P_2$ $(M+H)^+$ m/z calculated 1181.79410, found 1181.79321.

^{1}H NMR spectrum of compound **S105**

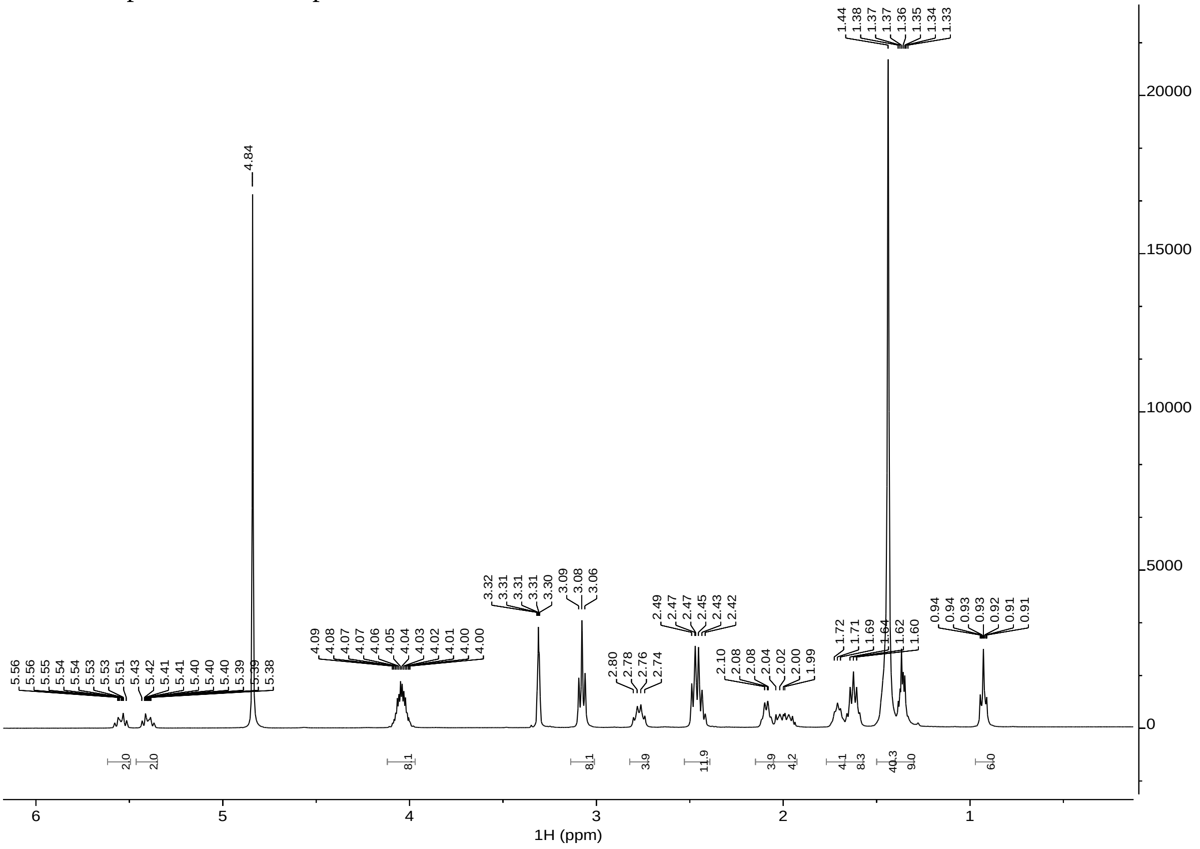

^{13}C NMR spectrum of compound **S105**

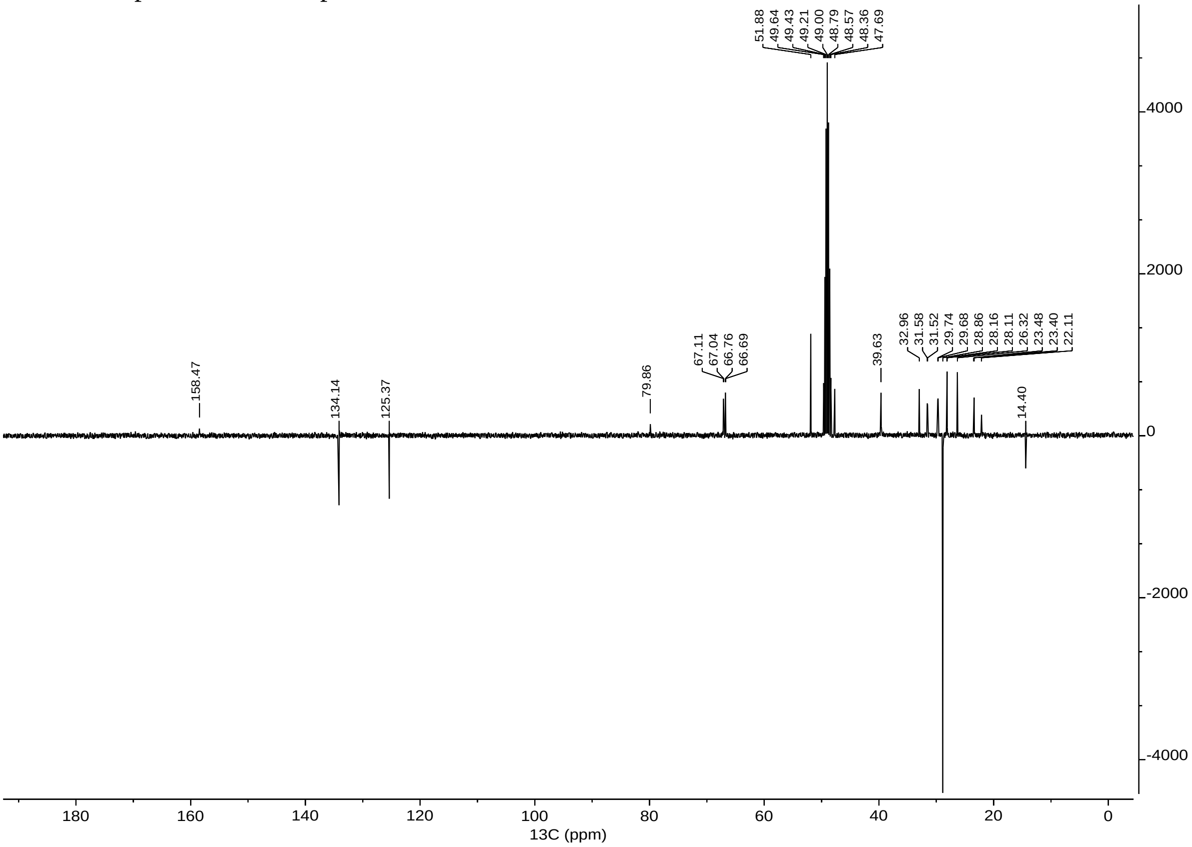

^{31}P{^{1}H} NMR spectrum of compound **S105**

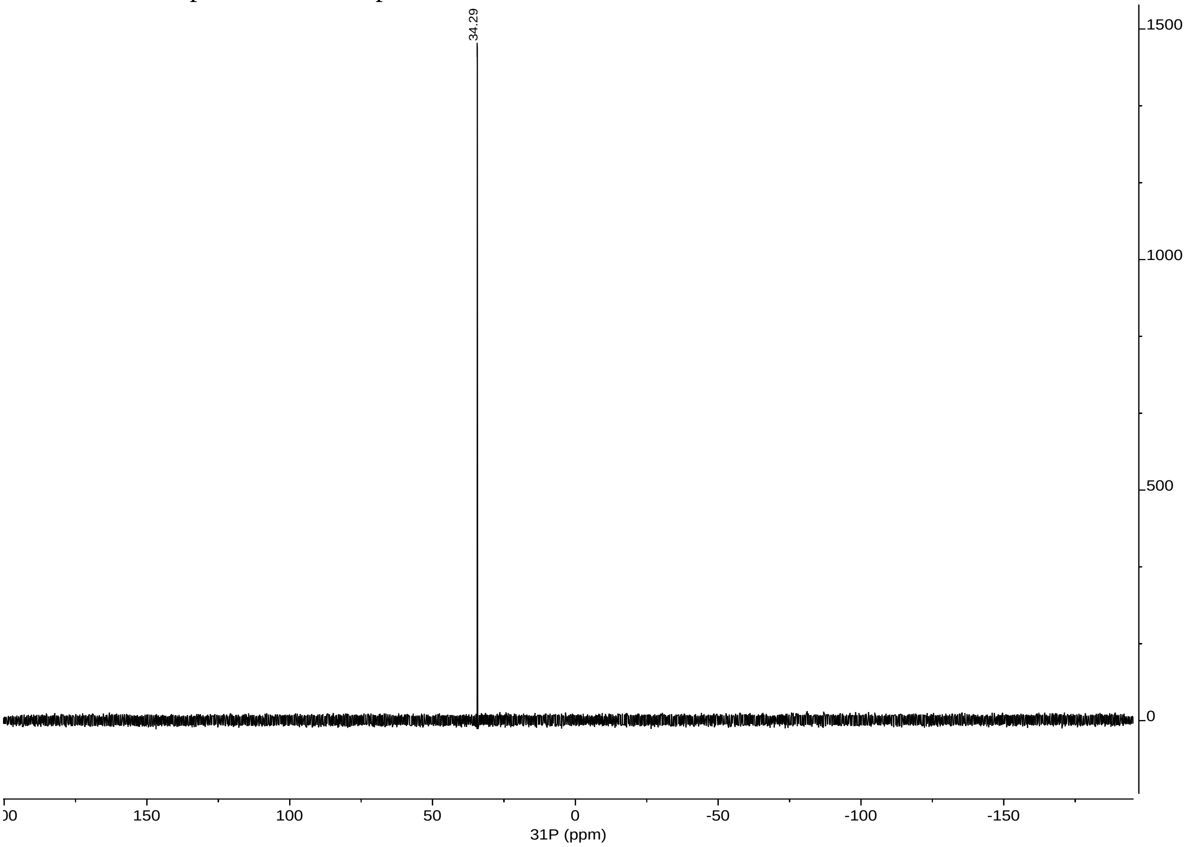

_N,N',N'',N'''_-Tetrakis(_tert_-butyloxycarbonyl) hexane-1,6-diyl bis((_Z_)-oct-3-en-1-yl) bis((2-(bis(2-aminoethyl)amino)ethyl)phosphonate) (S106)

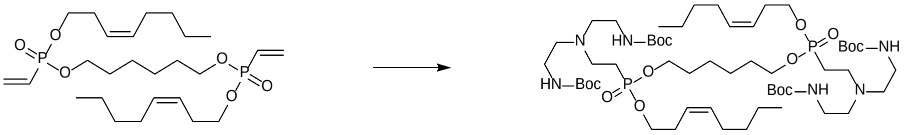

The title compound was prepared according to general method **D** from vinylphosphonate dimer **S59** (0.71 g, 1.37 mmol) and bis(2-*tert*-butyloxycarbonylaminoethyl)amine (1.67, 5.48 mmol) in 41% yield (0.63 g, 0.56 mmol) as a colourless oil.

^{1}H NMR (400 MHz, CD_3OD): 5.58–5.51 (m, 2H, $CH_3(CH_2)_3CH$), 5.44–5.37 (m, 2H, $CHCH_2CH_2O$), 4.10–4.01 (m, 8H, CH_2O), 3.11 (t, 8H, J = 6.3 Hz, CH_2NH), 2.83–2.76 (m, 4H, PCH_2CH_2), 2.55 (t, 8H, J = 6.8 Hz, CH_2CH_2NH), 2.45 (qd, 4H, J = 6.8, 1.4 Hz, $CHCH_2CH_2O$), 2.12–1.97 (m, 8H, PCH_2, $CH_3(CH_2)_2CH_2$), 1.75–1.68 (m, 4H, $OCH_2CH_2(CH_2)_2CH_2CH_2O$), 1.48–1.45 (m, 40H, $C(CH_3)_3$, $O(CH_2)_2(CH_2)_2(CH_2)_2O$), 1.38–1.33 (m, 8H, $CH_3(CH_2)_2$), 0.95–0.90 (m, 6H, CH_3CH_2).

^{13}C NMR (101 MHz, CD_3OD): 158.46 (NCOO), 134.14 ($CH_3(CH_2)_3CH$), 125.36 ($CHCH_2CH_2O$), 80.03 ($C(CH_3)_3$, 67.04 (d, J = 7.2 Hz, $CHCH_2CH_2O$), 66.68 (d, J = 7.5 Hz, $OCH_2(CH_2)_4CH_2O$), 54.37 (CH_2CH_2NH), 48.43 (PCH_2CH_2), 39.43 (CH_2NH), 32.96 ($CH_3CH_2CH_2$), 31.54 (d, J = 6.1 Hz, $OCH_2CH_2(CH_2)_2CH_2CH_2O$), 29.72 (d, J = 6.9 Hz, $CHCH_2CH_2O$), 28.87 ($C(CH_3)_3$), 28.12 ($CH_3(CH_2)_2CH_2$), 26.34 ($O(CH_2)_2(CH_2)_2(CH_2)_2O$), 23.95 (d, J = 137.5 Hz, PCH_2), 23.40 (CH_3CH_2), 14.41 (CH_3CH_2).

^{31}P{^{1}H} NMR (162 MHz, CD_3OD): 34.79.

IR ν_{max} (KBr) 3451 (w), 3325 (w, br), 2980 (s), 2962 (s), 2933 (m), 2873 (m), 2826 (w), 1704 (vs), 1650 (w), 1506 (s), 1467 (m), 1456 (m), 1393 (s), 1367 (s), 1248 (s), 1057 (m), 1003 (s).

HR-MS(ESI$^+$): For $C_{54}H_{107}O_{14}N_6P_2$ $(M+H)^+$ *m/z* calculated 1125.73150, found 1125.73133.

^{1}H NMR spectrum of compound **S106**

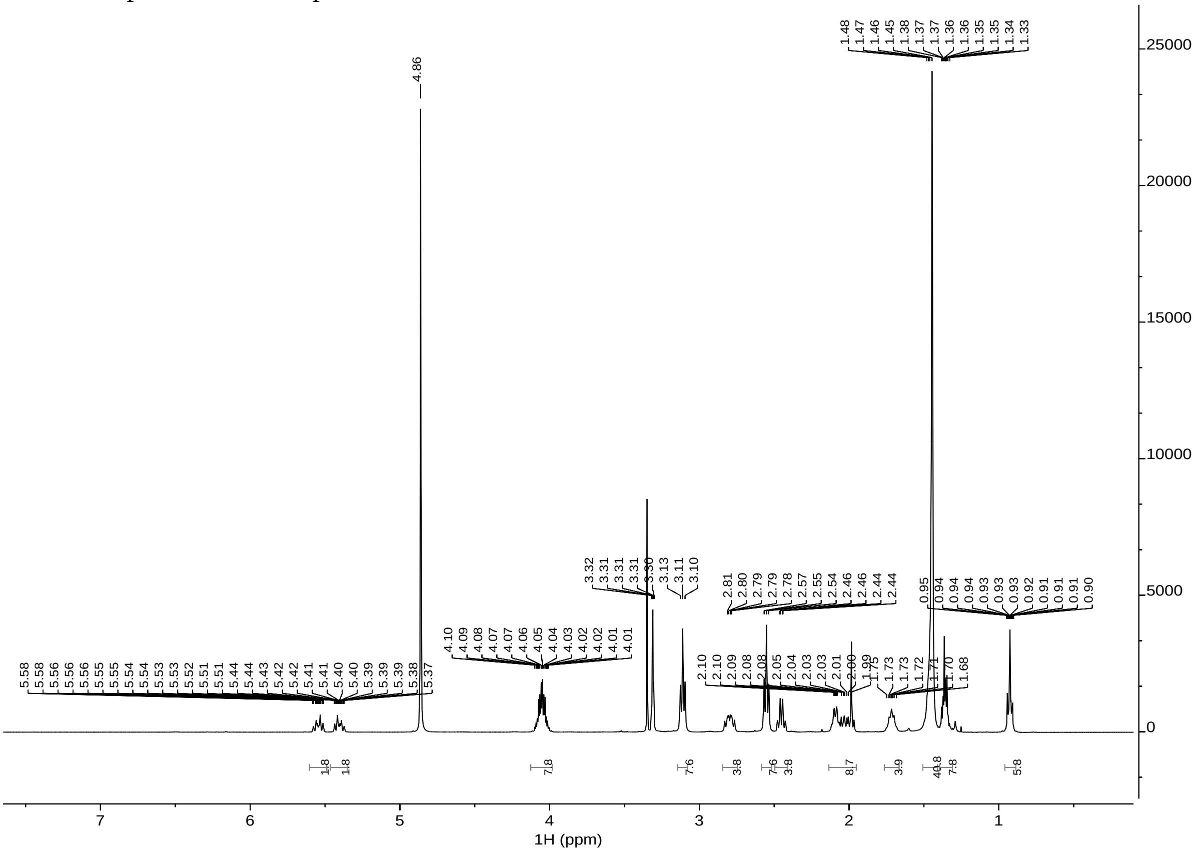

^{13}C NMR spectrum of compound **S106**

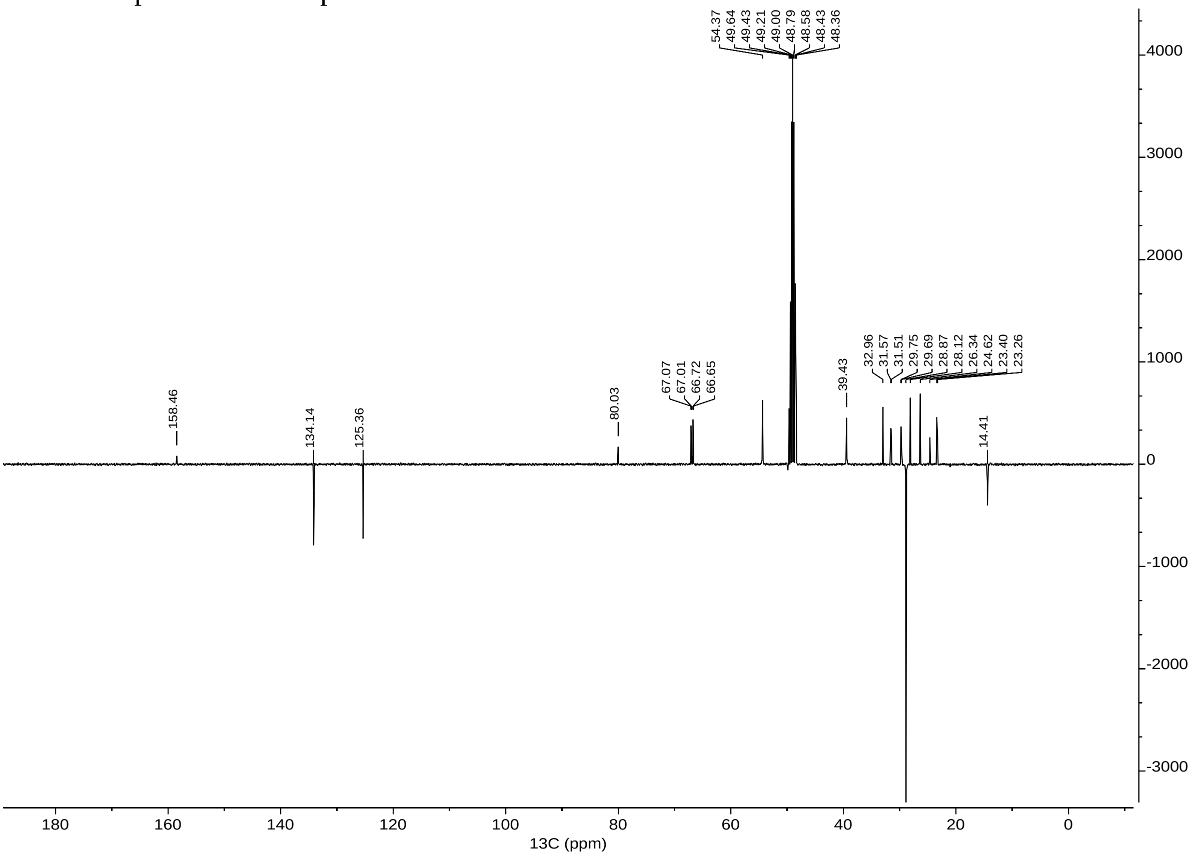

^{31}P{^{1}H} NMR spectrum of compound **S106**

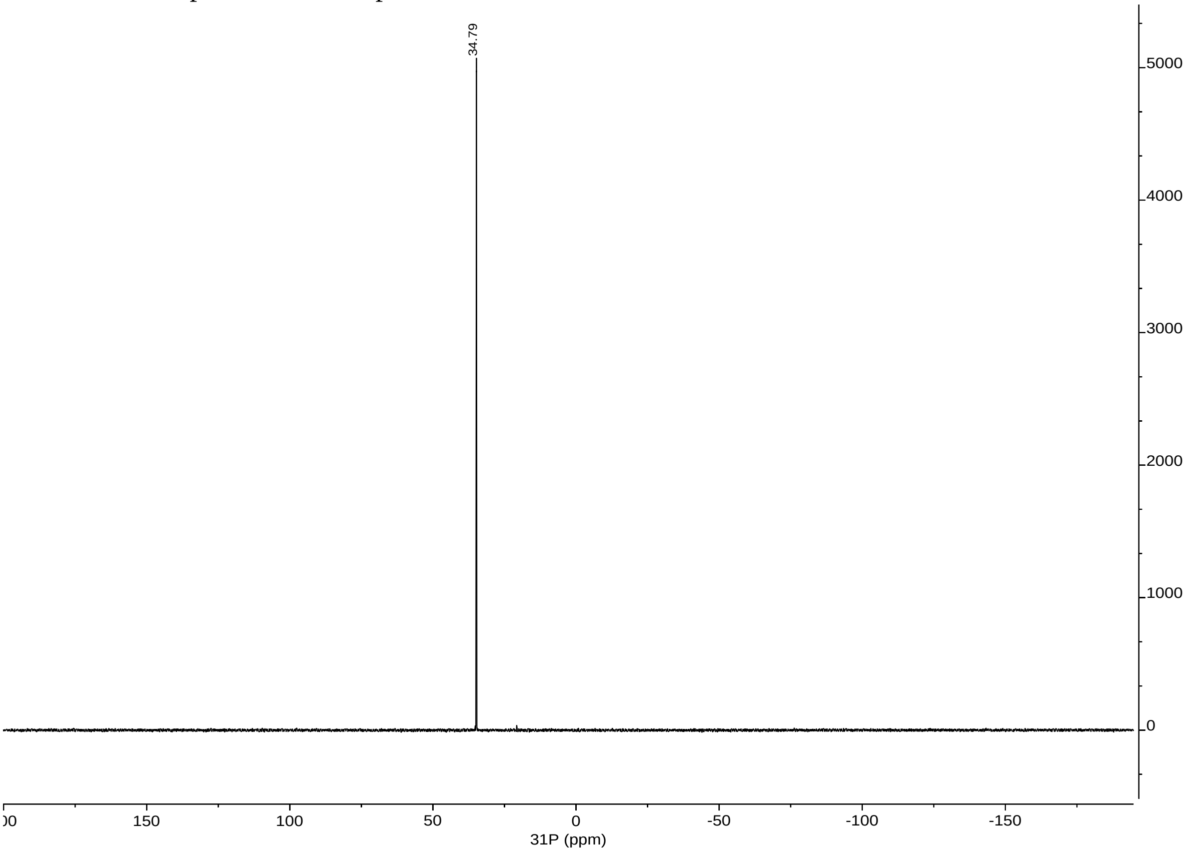

***N,N',N'',N'''*-Tetrakis(*tert*-butyloxycarbonyl) heptane-1,7-diyl bis(4,4,4-trifluorobutyl) bis((2-(bis(3-aminopropyl)amino)ethyl)phosphonate) (S107)**

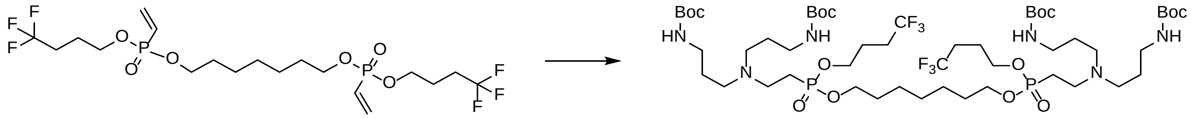

The title compound was prepared according to general method **D** from vinylphosphonate dimer **S65** (0.60 g, 1.13 mmol) and bis(3-*tert*-butyloxycarbonylaminopropyl)amine (1.45 g, 4.51 mmol) in 53% yield (0.72 g, 0.60 mmol) as a colourless oil.

^{1}H NMR (401 MHz, CD_3OD): 4.14–4.03 (m, 8H, CH_2O), 3.08 (t, 8H, J = 6.8 Hz, CH_2NH), 2.78 (q, 4H, J = 8.7 Hz, PCH_2CH_2), 2.49–2.46 (m, 8H, C$H_2(CH_2)_2$NH), 2.37–2.25 (m, 4H, CF_3CH_2), 2.07–1.90 (m, 8H, $CF_3CH_2CH_2$, PCH_2), 1.72–1.61 (m, 12H, $OCH_2CH_2(CH_2)_3CH_2CH_2O$, $NCH_2CH_2CH_2NH_2$), 1.47–1.42 (m, 42H, $C(CH_3)_3$, $OCH_2CH_2(CH_2)_3CH_2CH_2O$).

^{13}C NMR (101 MHz, CD_3OD): 158.50 (NCOO), 128.65 (q, J = 275.3 Hz, $CF_3CH_2CH_2CH_2O$), 79.87 ($C(CH_3)_3$), 67.43 (d, J = 6.8 Hz, $CF_3CH_2CH_2CH_2O$), 65.46 (d, J = 7.1 Hz, $OCH_2CH_2(CH_2)_3CH_2CH_2O$), 51.82 ($NCH_2CH_2CH_2NH$), 47.65 ($PCH_2$), 39.60 ($CH_2NH$), 31.56 (d, J = 6.1 Hz, $OCH_2CH_2(CH_2)_3CH_2CH_2O$), 31.01 (q, J = 29.1 Hz, $CF_3CH_2CH_2CH_2O$), 29.83 ($OCH_2CH_2CH_2CH_2CH_2CH_2CH_2O$), 28.84 ($C(CH_3)_3$, 28.12 ($NCH_2CH_2CH_2NH_2$), 26.63 ($OCH_2CH_2CH_2CH_2CH_2CH_2CH_2O$), 24.51 (dq, J = 6.2, 3.2 Hz, $CF_3CH_2CH_2$), 22.71 (d, J = 136.5 Hz, PCH_2).

^{31}P{^{1}H} NMR (162 MHz, CD_3OD): 35.00.

^{19}F NMR (377.3 MHz, CD_3OD): δ -67.79 (t, J = 11.1 Hz, CF_3).

IR ν_{max} 3456 (w), 3366 (w, br), 2981 (m), 2937 (m), 2867 (w), 2822 (w), 1707 (vs), 1507 (s), 1478 (w), 1393 (m), 1368 (s), 1340 (m), 1271 (s, sh), 1255 (vs), 1240 (vs), 1161 (vs), 1137 (m), 1085 (m, sh), 1050 (m), 1028 (s).

HR-MS(ESI^{+}): For $C_{51}H_{98}O_{14}N_6F_6NaP_2$ $(M+Na)^{+}$ m/z calculated 1217.64126, found 1217.64038.

^{1}H NMR spectrum of compound **S107**

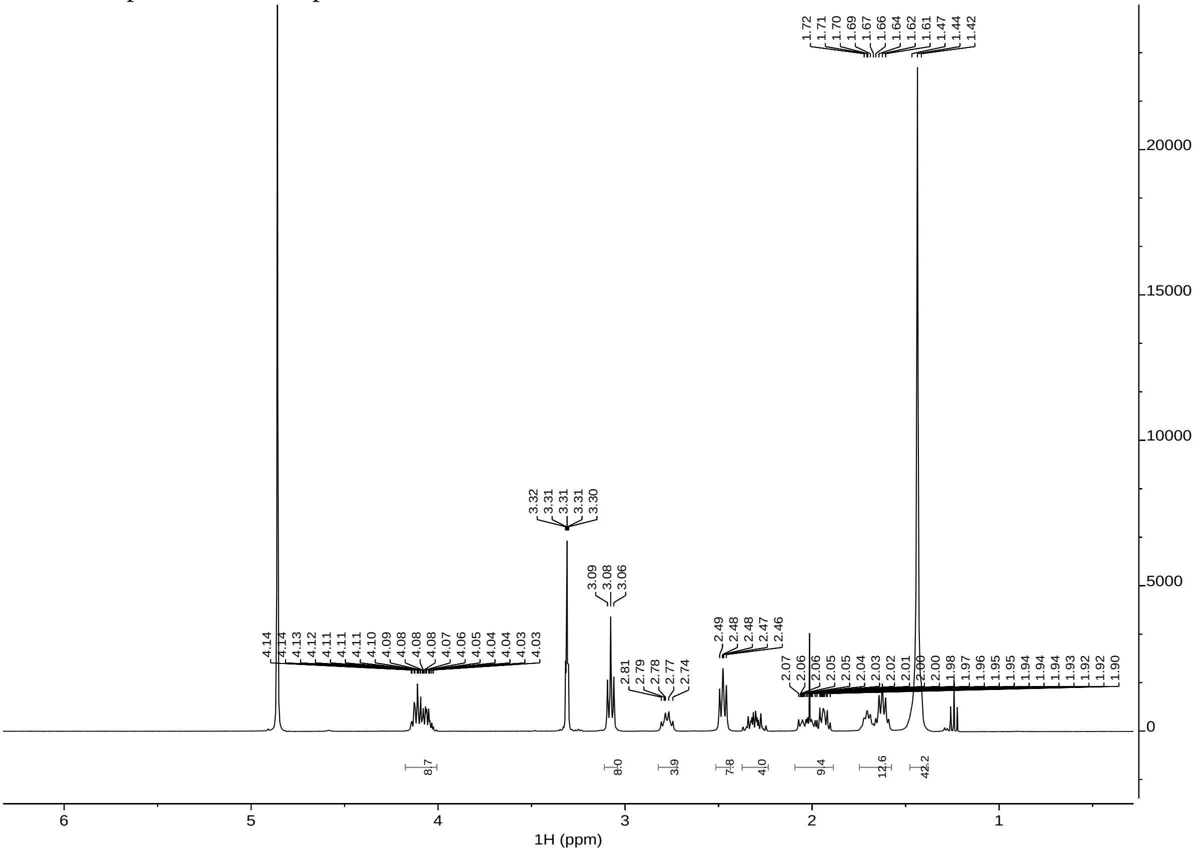

^{13}C NMR spectrum of compound **S107**

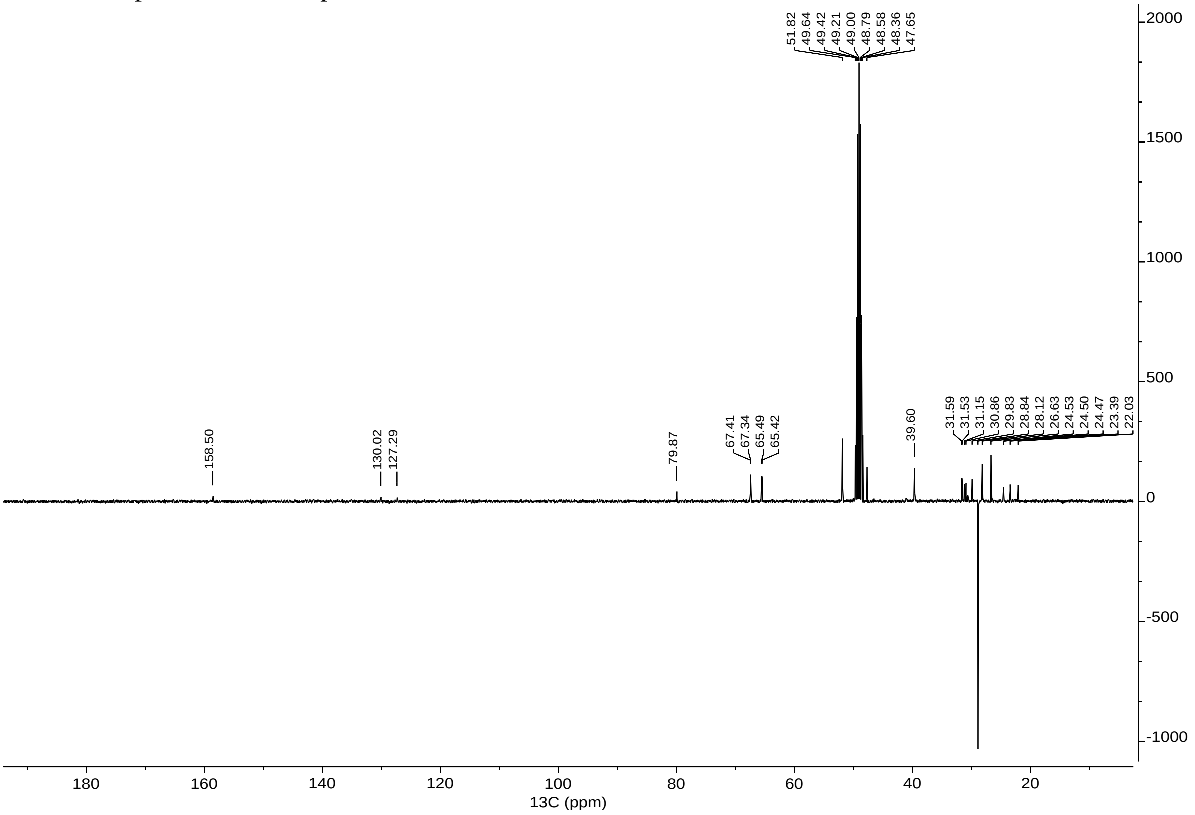

$^{31}P\{^{1}H\}$ NMR spectrum of compound **S107**

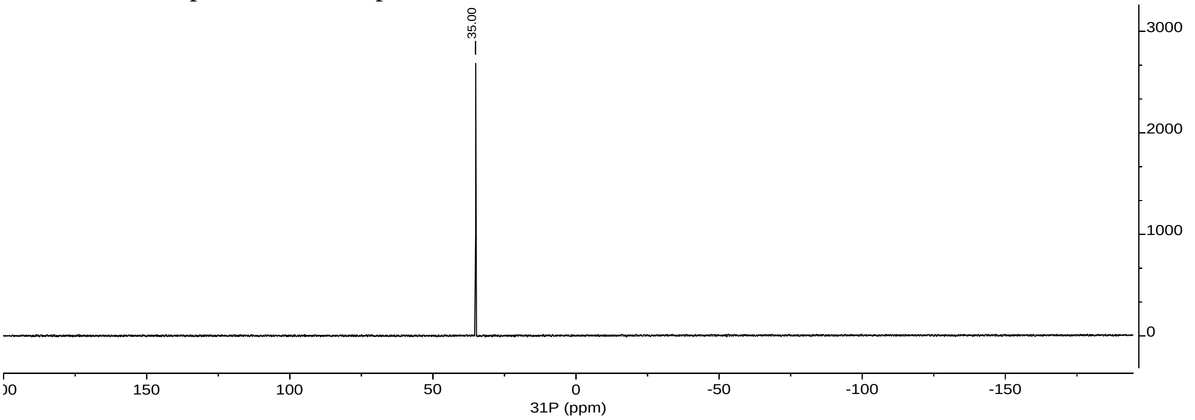

^{19}F NMR spectrum of compound **S107**

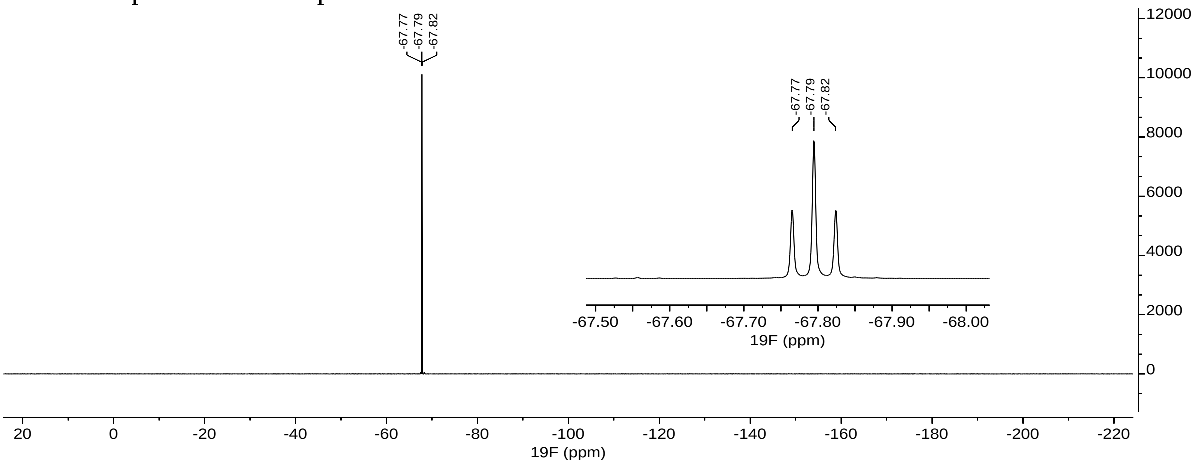

***N,N',N'',N'''*-Tetrakis(*tert*-butyloxycarbonyl) bis((*Z*)-hept-3-en-1-yl) heptane-1,7-diyl bis((2-(bis(3-aminopropyl)amino)ethyl)phosphonate) (S108)**

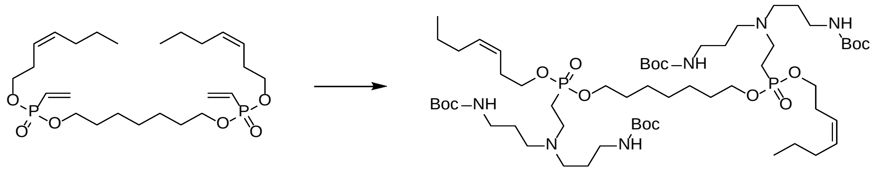

The title compound was prepared according to general method **D** from vinylphosphonate dimer **S67** (0.61 g, 1.21 mmol) and bis(3-*tert*-butyloxycarbonylaminopropyl)amine (1.61 g, 4.84 mmol) in 51% yield (0.77 g, 0.68 mmol) as a colourless oil.

^{1}H NMR (401 MHz, CD_3OD): 5.58–5.51 (m, 2H, C*H*CH(CH_2)$_2$O), 5.45–5.38 (m, 2H, C*H*(CH_2)$_2$O), 4.22–4.00 (m, 8H, C*H*$_2$O), 3.08 (t, 8H, J = 6.7 Hz, C*H*$_2$NH), 2.80–2.74 (m, 4H, PCH_2C*H*$_2$), 2.49–2.42 (m, 12H, C*H*$_2$(CH_2)$_2$NH, CHC*H*$_2$$CH_2$O), 2.10–2.06 (m, 4H, CH_3CH_2C*H*$_2$), 2.04–1.95 (m, 4H, PC*H*$_2$), 1.73–1.68 (m, 4H, OCH_2C*H*$_2$(CH_2)$_3$C*H*$_2$$CH_2$O), 1.66–1.60 (m, 8H, C*H*$_2$$CH_2$NH), 1.47–1.37 (m, 46H, CH_3C*H*$_2$, OCH_2CH_2(C*H*$_2$)$_3$$CH_2CH_2$O, C(C*H*$_3$)$_3$), 0.93 (t, 6H, J = 7.4 Hz, C*H*$_3$$CH_2$).

^{13}C NMR (101 MHz, CD_3OD): 158.48 (N*C*OO), 133.93 (CH_3(CH_2)$_2$*C*H), 125.57 (*C*HCH_2CH_2O), 79.87 (*C*(CH_3)$_3$, 67.17 (d, J = 7.1 Hz, $CHCH_2$*C*H$_2$O), 66.71 (d, J = 7.0 Hz, O*C*H$_2$(CH_2)$_5$*C*H$_2$O), 51.86 (*C*H$_2$(CH_2)$_2$NH), 47.67 (PCH_2*C*H$_2$), 39.61 (*C*H$_2$NH), 31.56 (d, J = 6.1 Hz, OCH_2*C*H$_2$(CH_2)$_3$*C*H$_2$$CH_2$O), 30.46 ($CH_3CH_2$*C*H$_2$), 29.84 (O($CH_2$)$_2$*C*H$_2$$CH_2$*C*H$_2$($CH_2$)$_2$O), 29.71 (d, J = 5.8 Hz, CH*C*H$_2$$CH_2$O), 28.86 (C(*C*H$_3$)$_3$), 28.13 (*C*H$_2$$CH_2$NH), 26.65 (O($CH_2$)$_3$*C*H$_2$($CH_2$)$_3$O), 23.82 ($CH_3$*C*H$_2$), 22.73 (d, J = 136.9 Hz, P*C*H$_2$), 14.19 (*C*H$_3$$CH_2$).

^{31}P{^{1}H} NMR (162 MHz, CD_3OD): 34.58.

IR ν_{max} (KBr) 3455 (m), 3367 (w), 2980 (s), 2963 (s), 2934 (s), 22871 (m), 2824 (m), 1707 (vs), 1656 (w, sh), 1507 (s), 1393 (m), 1367 (s), 1249 (vs), 1169 (vs), 1065–1007 (s).

HR-MS(ESI$^+$): For $C_{57}H_{113}O_{14}N_6P_2$ (M+H)$^+$ *m/z* calculated 1167.77845, found 1167.77825.

^{1}H NMR spectrum of compound **S108**

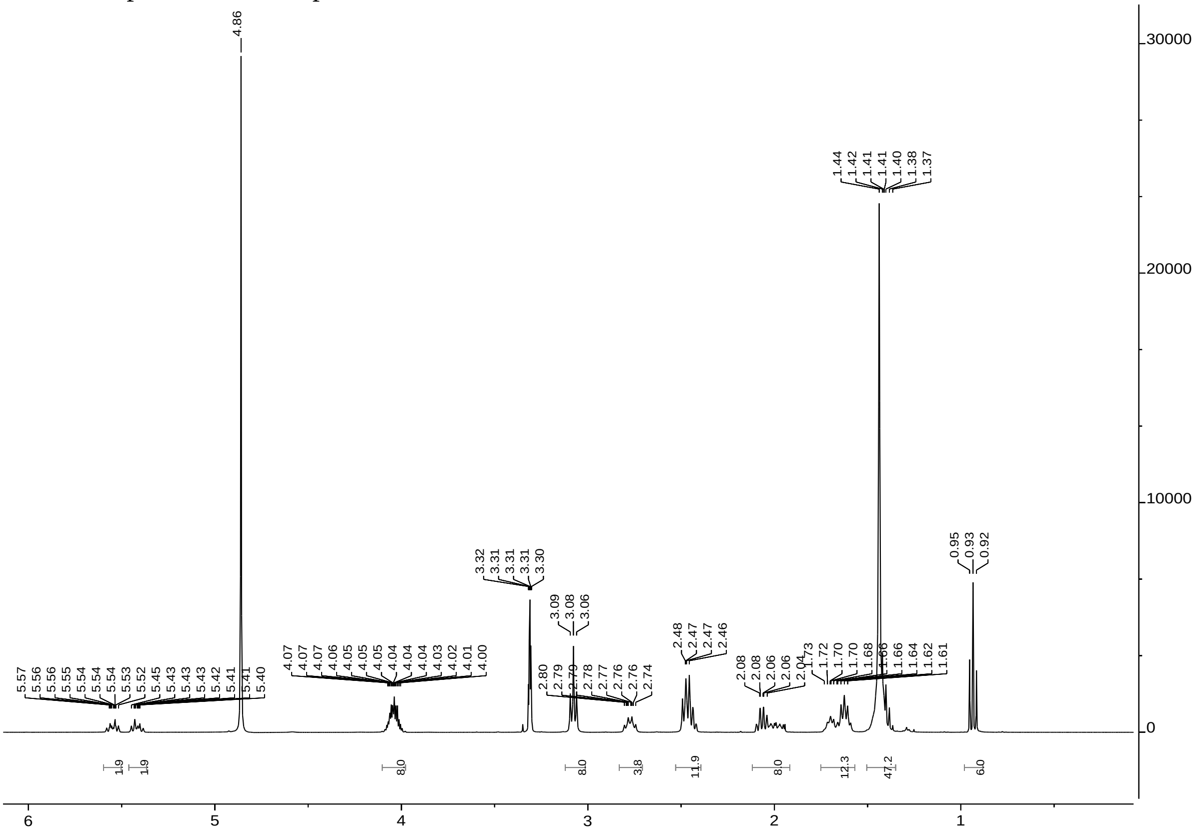

^{13}C NMR spectrum of compound **S108**

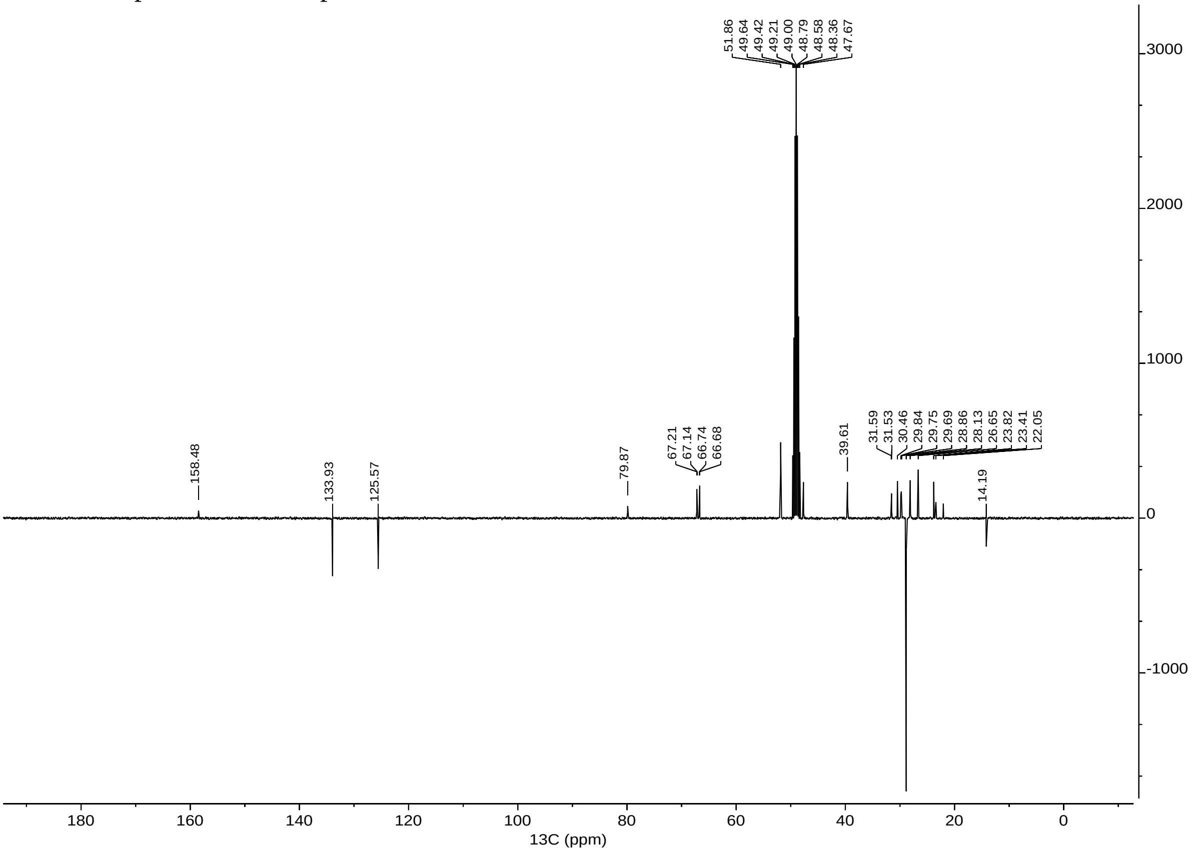

^{31}P{^{1}H} NMR spectrum of compound **S108**

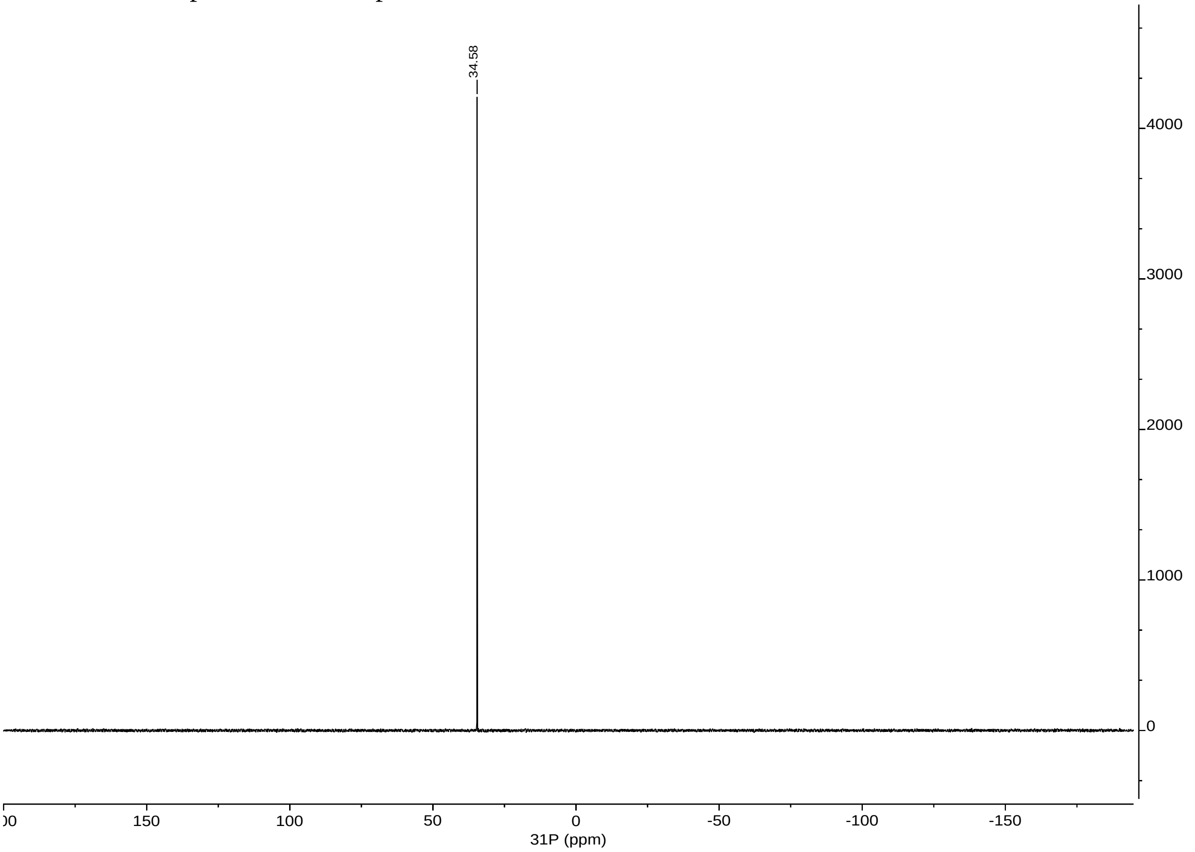

***N,N',N'',N'''*-Tetrakis(*tert*-butyloxycarbonyl) bis((*Z*)-hept-3-en-1-yl) heptane-1,7-diyl bis((2-(bis(2-aminoethyl)amino)ethyl)phosphonate) (S109)**

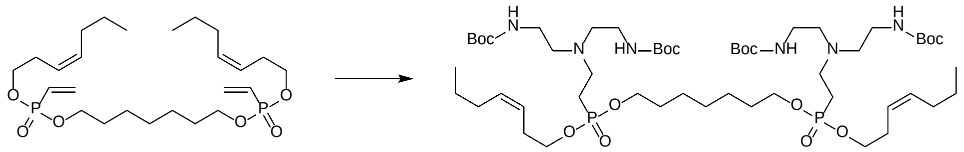

The title compound was prepared according to general method **D** from vinylphosphonate dimer **S67** (0.65 g, 1.29 mmol) and bis(2-*tert*-butyloxycarbonylaminoethyl)amine (1.56 g, 5.15 mmol) in 31% yield (0.45 g, 0.41 mmol) as a colourless oil.

^{1}H NMR (401 MHz, CD_3OD): 5.58–5.51 (m, 2H, $CH_3(CH_2)_2CH$), 5.45–5.38 (m, 2H, $CHCH_2CH_2O$), 4.09–4.00 (m, 8H, CH_2O), 3.11 (t, 8H, J = 6.2 Hz, CH_2NH), 2.79 (dt, 4H, J = 11.1, 7.4 Hz, PCH_2CH_2), 2.54 (t, 8H, J = 6.3 Hz, CH_2CH_2NH), 2.45 (qd, 4H, J = 6.7, 1.4 Hz, $CHCH_2CH_2O$), 2.10–1.96 (m, 8H, PCH_2, $CH_3CH_2CH_2$), 1.74–1.67 (m, 4H, $OCH_2CH_2(CH_2)_3CH_2CH_2O$), 1.45–1.36 (m, 46H, $C(CH_3)_3$, CH_3CH_2, $O(CH_2)_2(CH_2)_3(CH_2)_2O$), 0.93 (t, 6H, J = 7.4 Hz, CH_3CH_2).

^{13}C NMR (101 MHz, CD_3OD): 158.47 (NCOO), 133.93 ($CH_3(CH_2)_3CHCHCH_2CH_2O$), 125.56 ($CH_3(CH_2)_2CHCHCH_2CH_2O$), 80.03 ($C(CH_3)_3$, 67.13 (d, J = 6.7 Hz, $CH_3(CH_2)_2CHCHCH_2CH_2O$), 66.72 (d, J = 6.7 Hz, $OCH_2CH_2CH_2CH_2CH_2CH_2CH_2O$), 54.37 ($NCH_2CH_2NH$), 48.42 ($PCH_2CH_2N$), 39.45 ($NCH_2CH_2NH$), 31.57 (d, J = 5.9 Hz, $OCH_2CH_2CH_2CH_2CH_2CH_2CH_2O$), 30.46 ($CH_3CH_2CH_2CHCHCH_2CH_2O$), 29.86 ($OCH_2CH_2CH_2CH_2CH_2CH_2CH_2O$), 29.73 ($J$ = 7.5 Hz, $CH_3CH_2CH_2CHCHCH_2CH_2O$), 28.87 ($C(CH_3)_3$), 26.65 ($OCH_2CH_2CH_2CH_2CH_2CH_2CH_2O$), 23.97 (d, J = 136.8 Hz, PCH_2CH_2N), 23.81 ($CH_3CH_2CH_2CHCHCH_2CH_2O$), 14.18 ($CH_3(CH_2)_2CHCHCH_2CH_2O$).

^{31}P{^{1}H} NMR (162 MHz, CD_3OD): 34.83.

IR ν_{max} (KBr) 3451 (w), 3329 (w, br), 2980 (s), 2964 (s), 2933 (s), 2871 (m), 2825 (m), 1707 (vs), 1507 (s), 1394 (m), 1368 (s), 1249 (vs), 1065–1011 (m), 463 (vw).

HR-MS(ESI$^+$): For $C_{53}H_{104}O_{14}N_6NaP_2$ $(M+Na)^+$ m/z calculated 1133.69780, found 1133.69801.

^{1}H NMR spectrum of compound **S109**

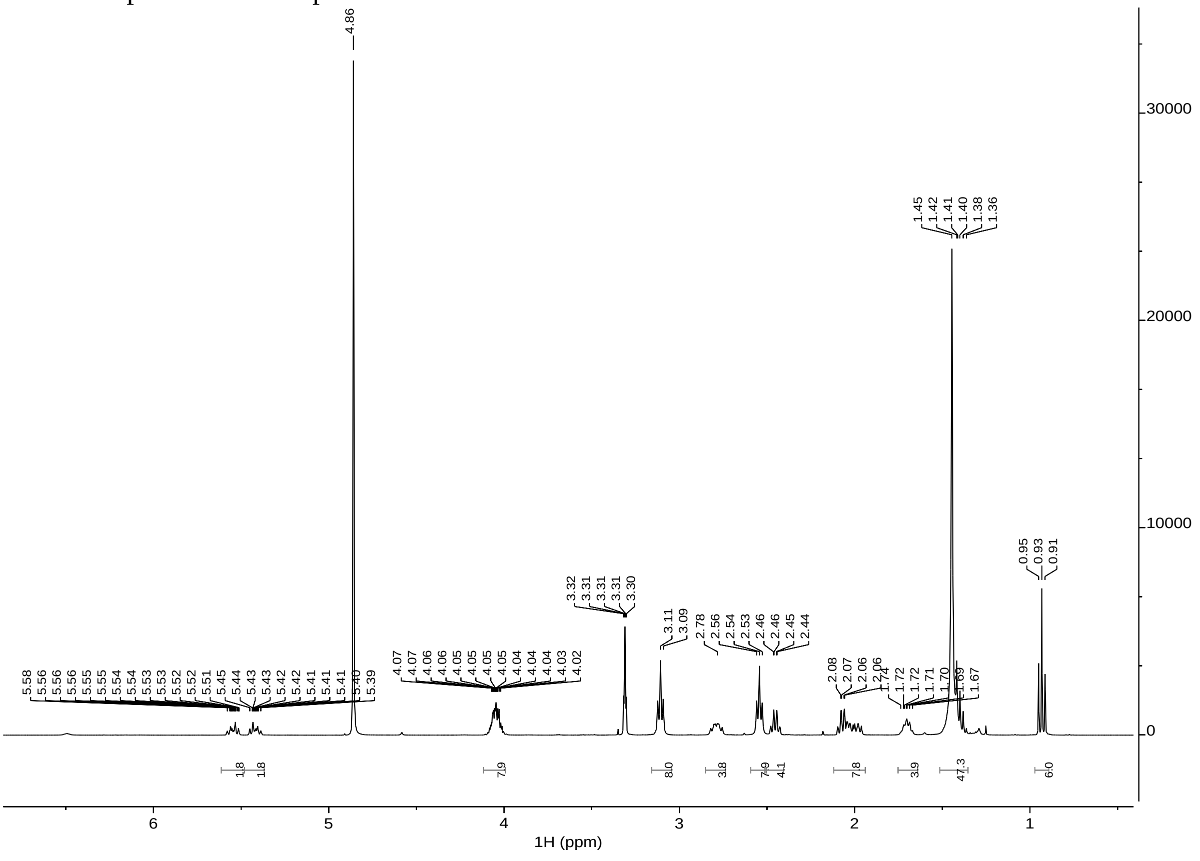

^{13}C NMR spectrum of compound **S109**

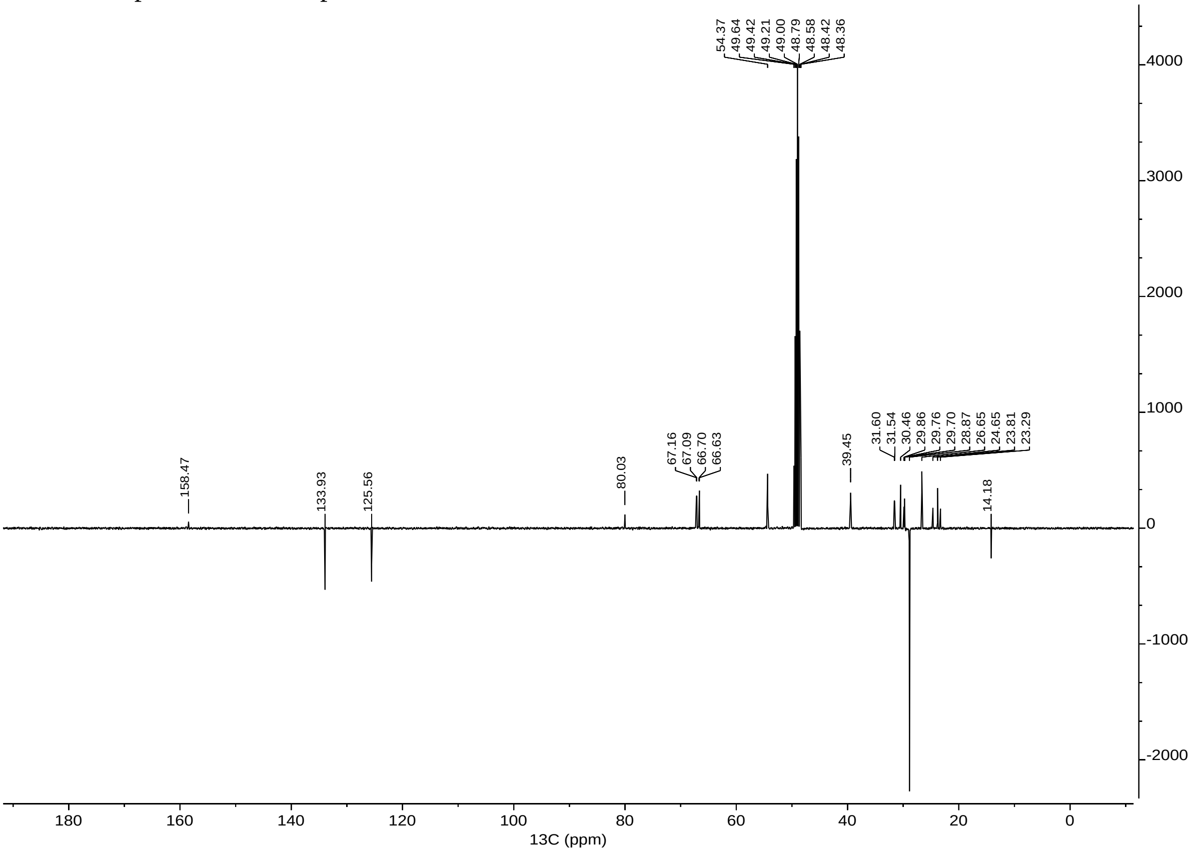

^{31}P{^{1}H} NMR spectrum of compound **S109**

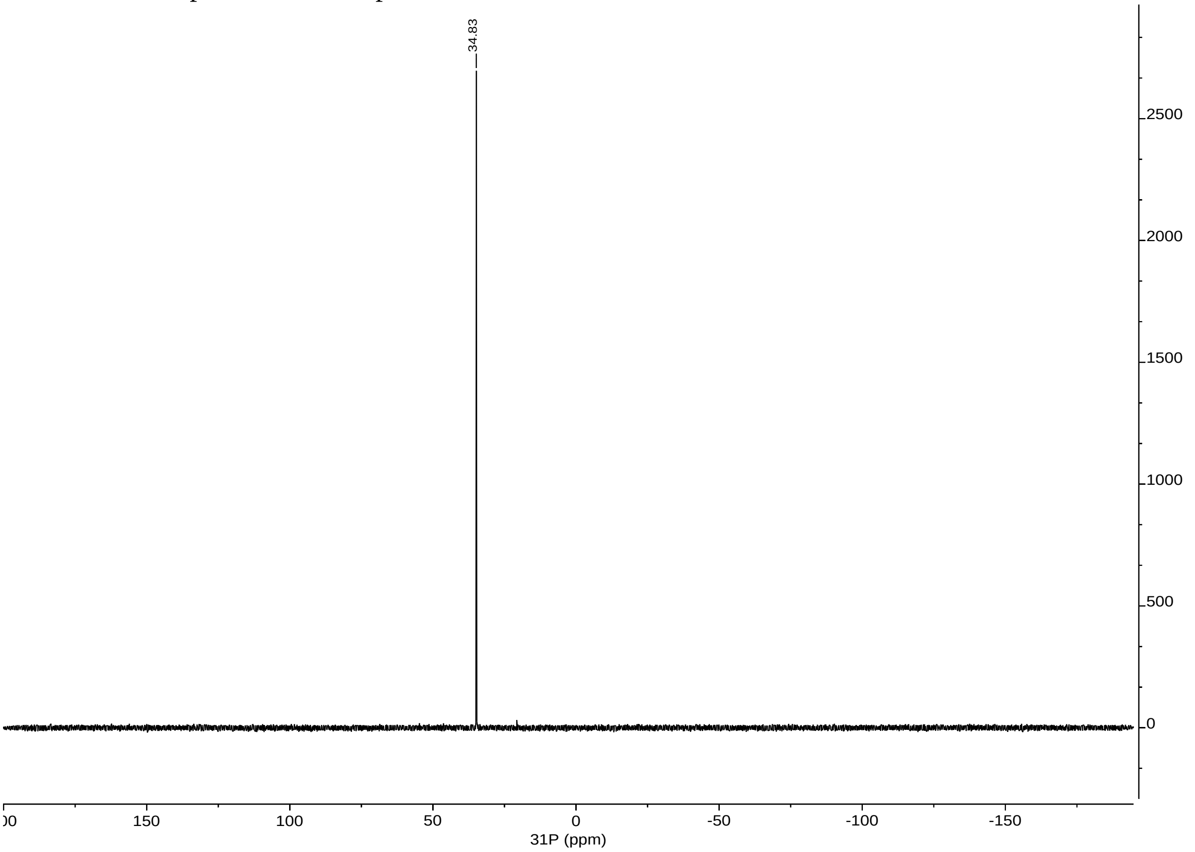

***N,N',N'',N'''*-Tetrakis(*tert*-butyloxycarbonyl) dibutyl octane-1,8-diyl bis((2-(bis(3-aminopropyl)amino)ethyl)phosphonate) (S110)**

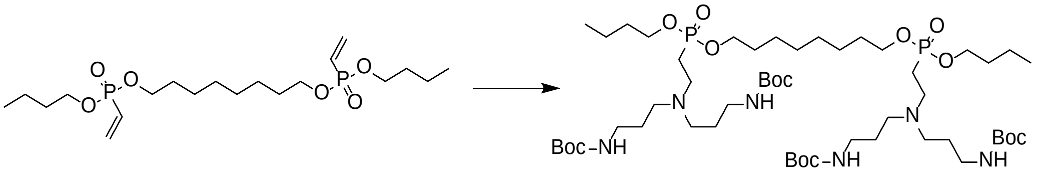

The title compound was prepared according to general method **D** from vinylphosphonate dimer **S69** (0.40 g, 0.91 mmol) and bis(3-*tert*-butyloxycarbonylaminopropyl)amine (1.21 g, 3.65 mmol) in 59% yield (0.65 g, 0.54 mmol) as a colourless oil.

^{1}H NMR (400 MHz, CD_3OD): 4.11–3.99 (m, 8H, $CH_3CH_2CH_2CH_2O$, $OCH_2CH_2(CH_2)_4CH_2CH_2O$), 3.10–3.07 (m, 8H, $NCH_2CH_2CH_2NH$), 2.86–2.80 (m, 4H, PCH_2CH_2N), 2.57–2.53 (m, 8H, $NCH_2CH_2CH_2NH$), 2.07–2.00 (m, 4H, PCH_2CH_2N), 1.71–1.59 (m, 16H, $CH_3CH_2CH_2CH_2O$, $OCH_2CH_2(CH_2)_4CH_2CH_2O$, $NCH_2CH_2CH_2NH$), 1.47–1.36 (m, 48H, $CH_3CH_2CH_2CH_2O$, $OCH_2CH_2(CH_2)_4CH_2CH_2O$, $C(CH_3)_3$), 0.99–0.94 (m, 6H, $CH_3(CH_2)_2CH_2O$).

^{13}C NMR (101 MHz, CD_3OD): 158.62 (NHCOO), 79.94 ($C(CH_3)_3$), 67.29, 67.03 (d, J = 7.0 Hz, $CH_3CH_2CH_2CH_2O$, $OCH_2CH_2(CH_2)_4CH_2CH_2O$), 51.83 ($NCH_2CH_2CH_2NH$), 47.73 ($PCH_2CH_2N$), 39.45 ($NCH_2CH_2CH_2NH$), 33.66, 31.60 (d, J = 6.0 Hz, $CH_3CH_2CH_2CH_2O$, $OCH_2CH_2(CH_2)_4CH_2CH_2O$), 28.84 ($C(CH_3)_3$, 27.88 ($NCH_2CH_2CH_2NH$), 30.21, 26.66 ($OCH_2CH_2(CH_2)_4CH_2CH_2O$), 22.56 (d, J = 137.8 Hz, PCH_2CH_2N), 19.86 ($CH_3CH_2CH_2CH_2O$), 13.98 ($(CH_3CH_2)_2CH_2O$).

^{31}P{^{1}H} NMR (162 MHz, CD_3OD): 33.68.

IR ν_{max} (KBr) 3455 (m), 3367 (w, vbr), 2982 (s), 2968 (s), 2932 (s), 2868 (m), 2821 (m), 1705 (vs), 1507 (vs), 1477 (m), 1466 (m), 1455 (s), 1395 (s), 1381 (w, sh), 1369 (vs), 1350 (m), 1247 (vs), 1169 (vs), 1062 (s), 1048 (s, sh), 1029 (s), 1013 (s, sh), 989 (s), 463 (w).

HR-MS(ESI^{+}): For $C_{52}H_{106}N_6O_{14}NaP_2$ $(M+Na)^+$ m/z calculated 1123.71345, found 1123.71407.

^{1}H NMR spectrum of compound **S110**

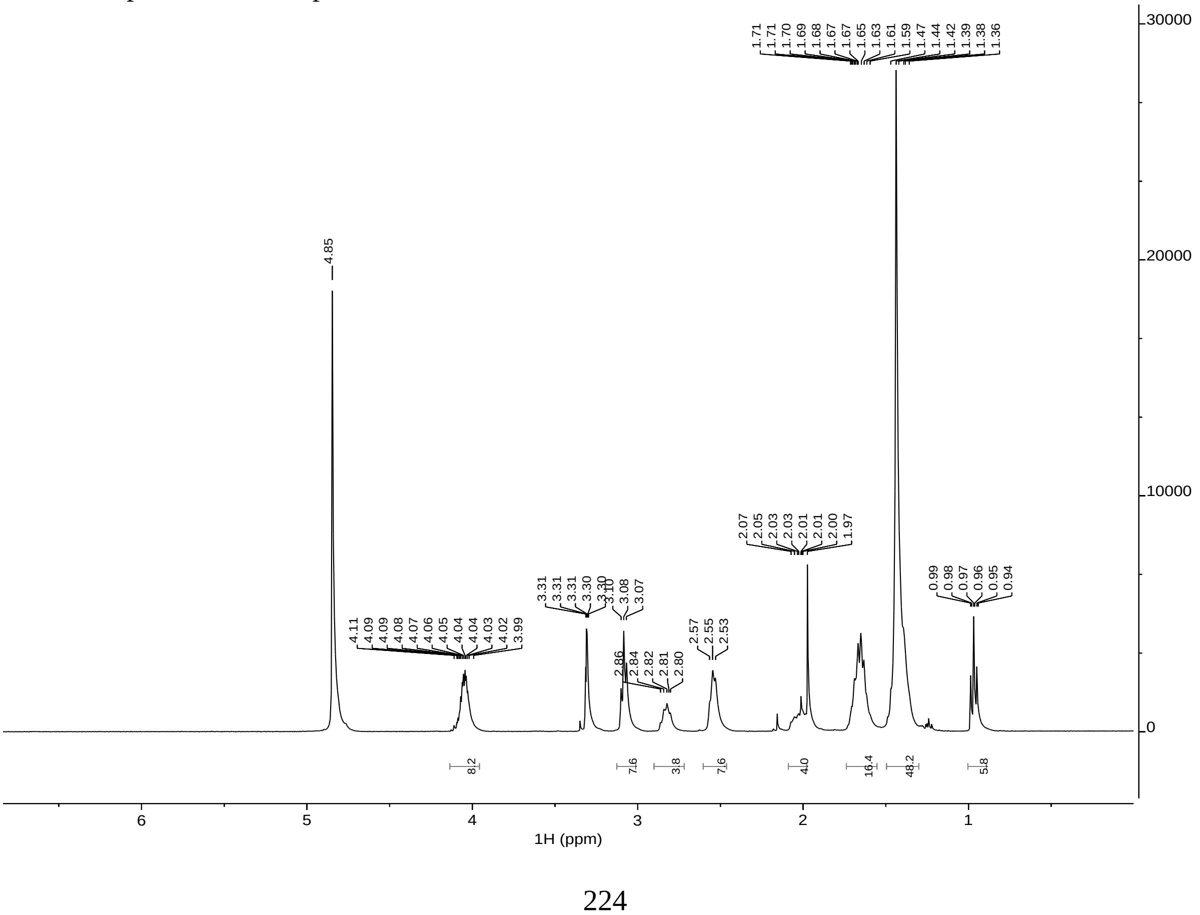

^{13}C NMR spectrum of compound **S110**

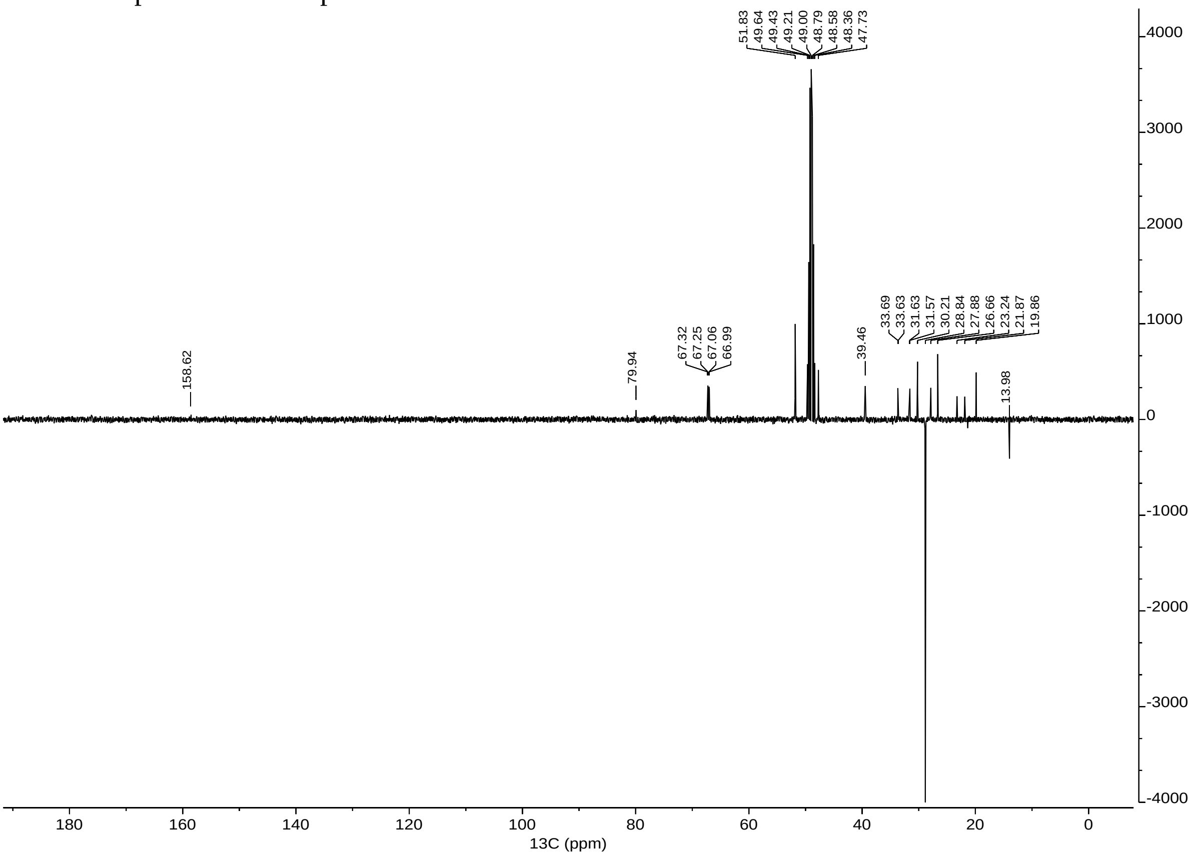

$^{31}P\{^{1}H\}$ NMR spectrum of compound **S110**

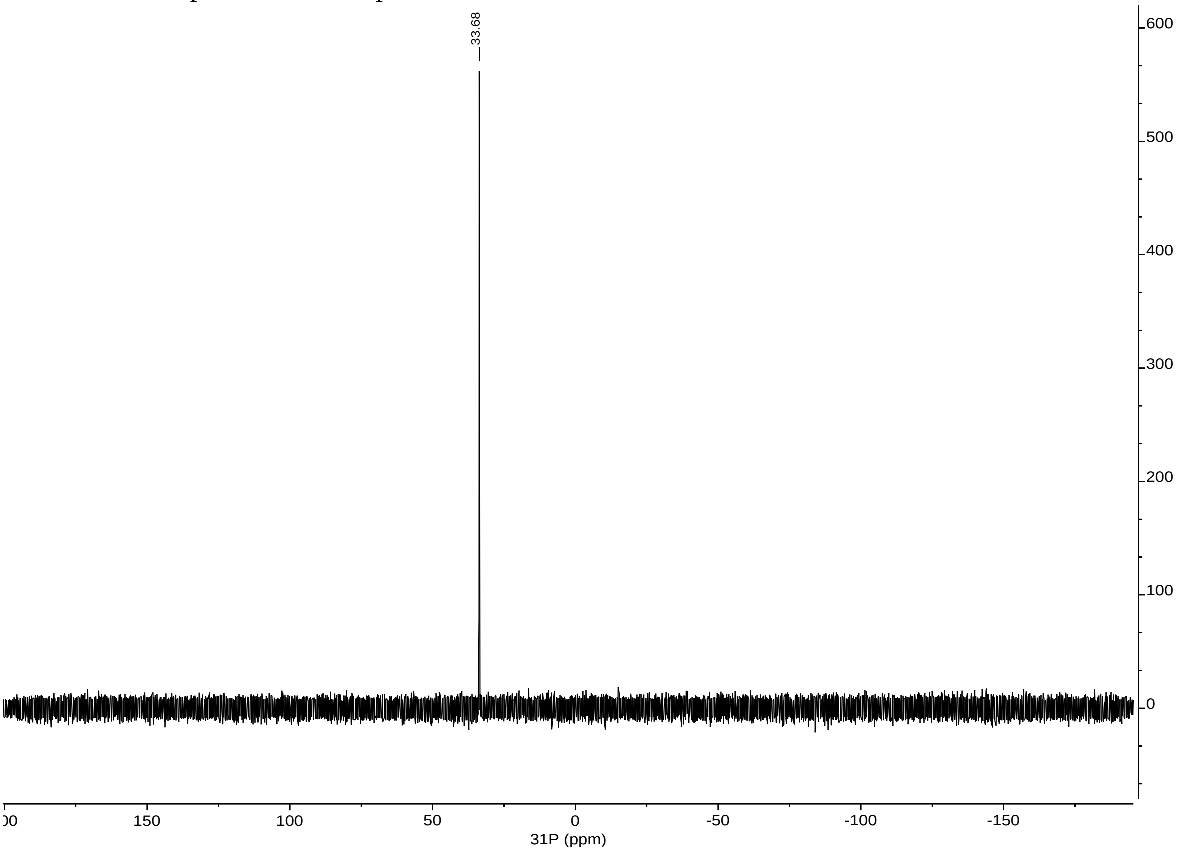

_N,N’,N’’,N’’’_-Tetrakis(_tert_-butyloxycarbonyl) octane-1,8-diyl dipentyl bis((2-(bis(3-aminopropyl)amino)ethyl)phosphonate) (S111)

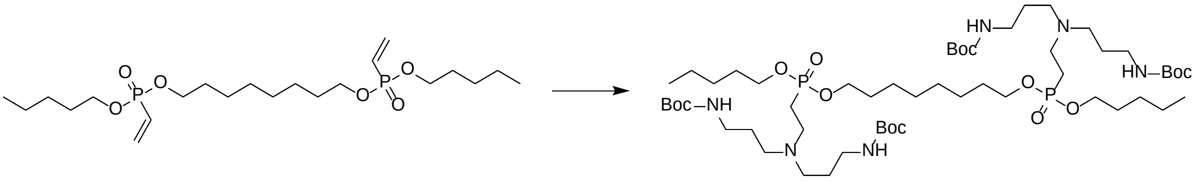

The title compound was prepared according to general method **D** from vinylphosphonate dimer **S70** (0.70 g, 1.50 mmol) and bis(3-*tert*-butyloxycarbonylaminopropyl)amine (2.0 g, 6.0 mmol) in 58% yield (0.98 g, 0.87 mmol) as a colourless oil.

^{1}H NMR (400 MHz, CD_3OD): 4.10–4.00 (m, 8H, $CH_3(CH_2)_2CH_2CH_2O$, $OCH_2CH_2(CH_2)_4CH_2CH_2O$), 3.08 (t, 8H, J = 6.8 Hz, $NCH_2CH_2CH_2NH$), 2.80–2.74 (m, 4H, PCH_2CH_2N), 2.49–2.46 (m, 8H, $NCH_2CH_2CH_2NH$), 2.04–1.95 (m, 4H, PCH_2CH_2N), 1.73–1.61 (m, 16H, $CH_3(CH_2)_2CH_2CH_2O$, $OCH_2CH_2(CH_2)_4CH_2CH_2O$, $NCH_2CH_2CH_2NH$), 1.44–1.25 (m, 52H, $CH_3(CH_2)_2CH_2CH_2O$, $OCH_2CH_2(CH_2)_4CH_2CH_2O$, $C(CH_3)_3$), 0.96–0.92 (m, 6H, $CH_3(CH_2)_3CH_2O$).

^{13}C NMR (101 MHz, CD_3OD): 158.48 (NHCOO), 79.87 ($C(CH_3)_3$), 67.26 (d, J = 6.7 Hz, $CH_3(CH_2)_2CH_2CH_2O$), 67.19 (d, J = 6.9 Hz, $OCH_2CH_2(CH_2)_4CH_2CH_2O$), 51.85 ($NCH_2CH_2CH_2NH$), 47.68 ($PCH_2CH_2N$), 39.60 ($NCH_2CH_2CH_2NH$), 31.61, 31.33 (d, J = 6.1 Hz, $CH_3(CH_2)_2CH_2CH_2O$, $OCH_2CH_2(CH_2)_4CH_2CH_2O$), 28.85 ($C(CH_3)_3$, 28.12 ($NCH_2CH_2CH_2NH$), 30.23, 28.92, 26.69, 23.31 ($CH_3(CH_2)_2CH_2CH_2O$, $OCH_2CH_2(CH_2)_4CH_2CH_2O$), 22.69 (d, J = 136.8 Hz, PCH_2CH_2N), 14.40 ($CH_3(CH_2)_3CH_2O$).

^{31}P{^{1}H} NMR (162 MHz, CD_3OD): 34.29.

IR ν_{max} (KBr) 3000–2600 (vs, vbr), 2957 (vs), 2929 (vs), 2856 (s), 1475 (m, sh), 1467 (m), 1457 (m), 1250 (s), 1174 (s), 1019 (s), 994 (s).

HR-MS(ESI^{+}): For $C_{54}H_{110}N_6O_{14}NaP_2$ $(M+Na)^{+}$ m/z calculated 1151.74475, found 1151.74538.

^{1}H NMR spectrum of compound **S111**

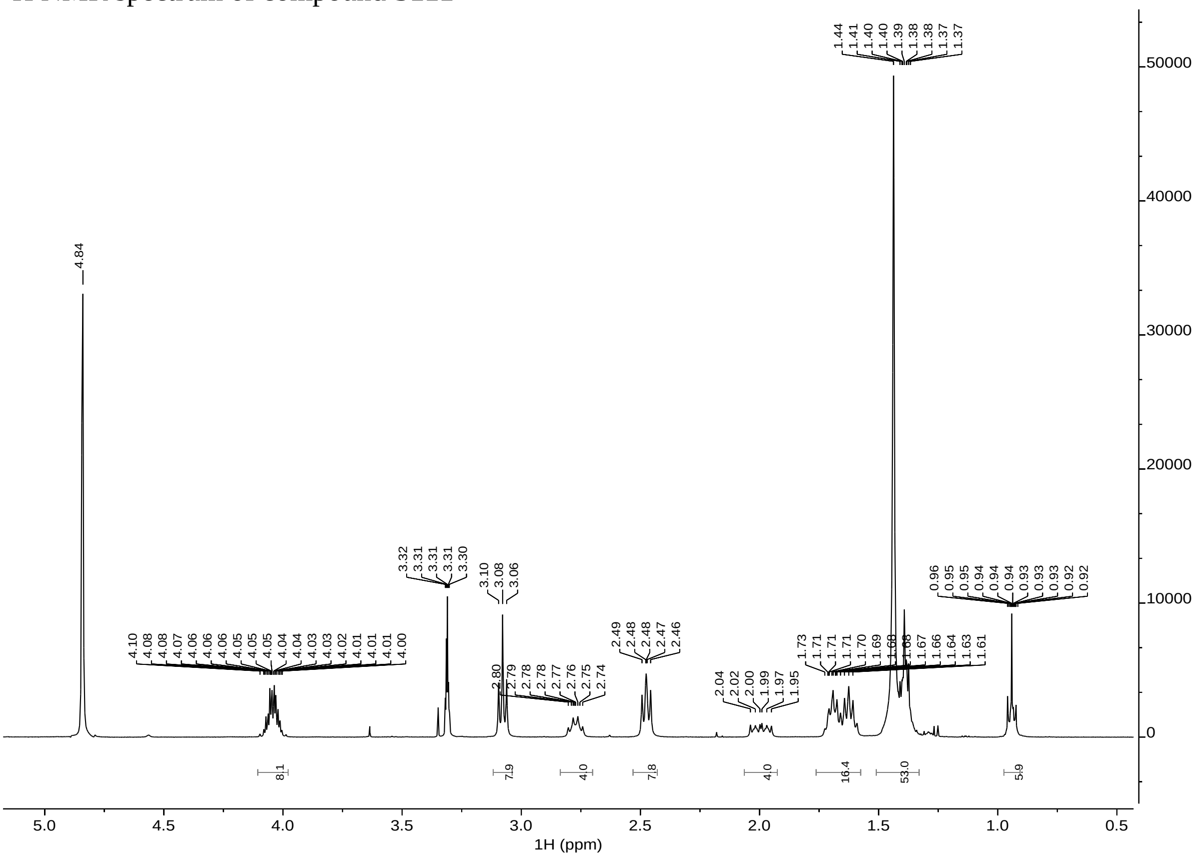

^{13}C NMR spectrum of compound **S111**

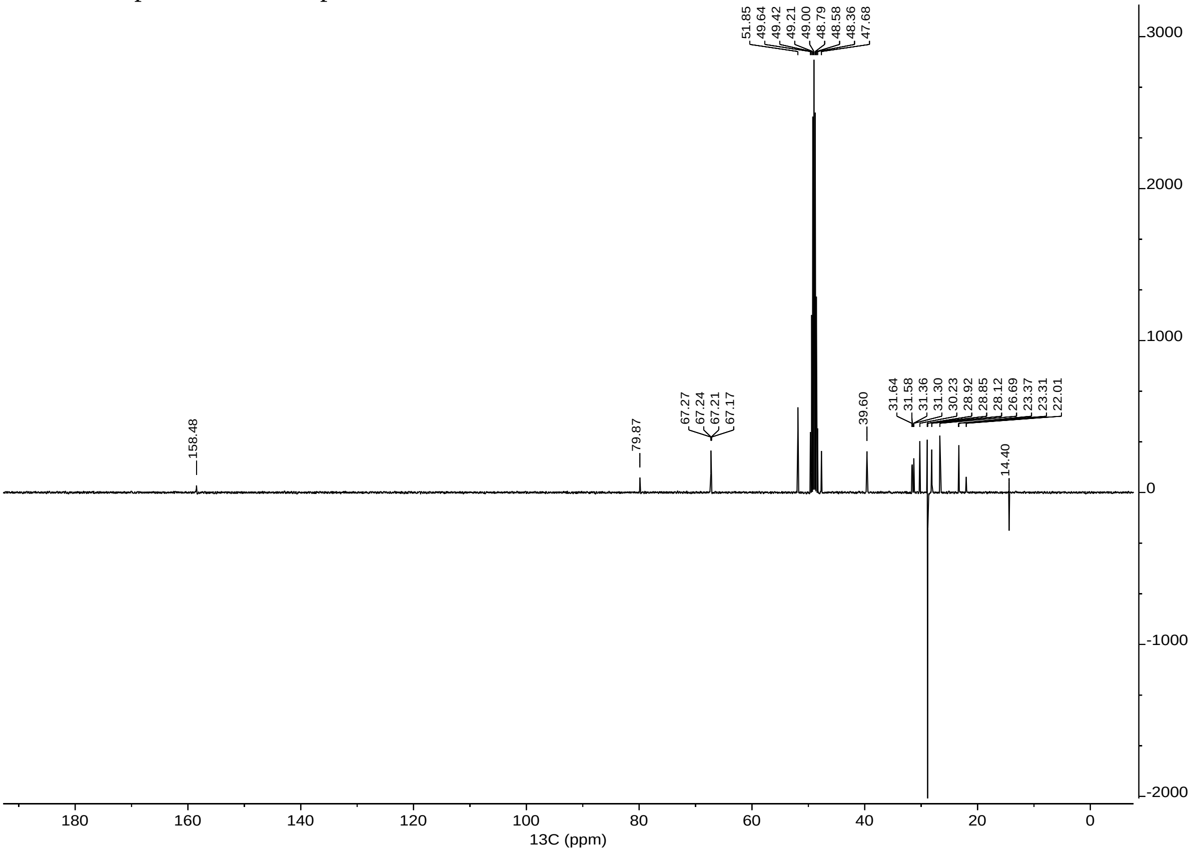

$^{31}P\{^1H\}$ NMR spectrum of compound **S111**

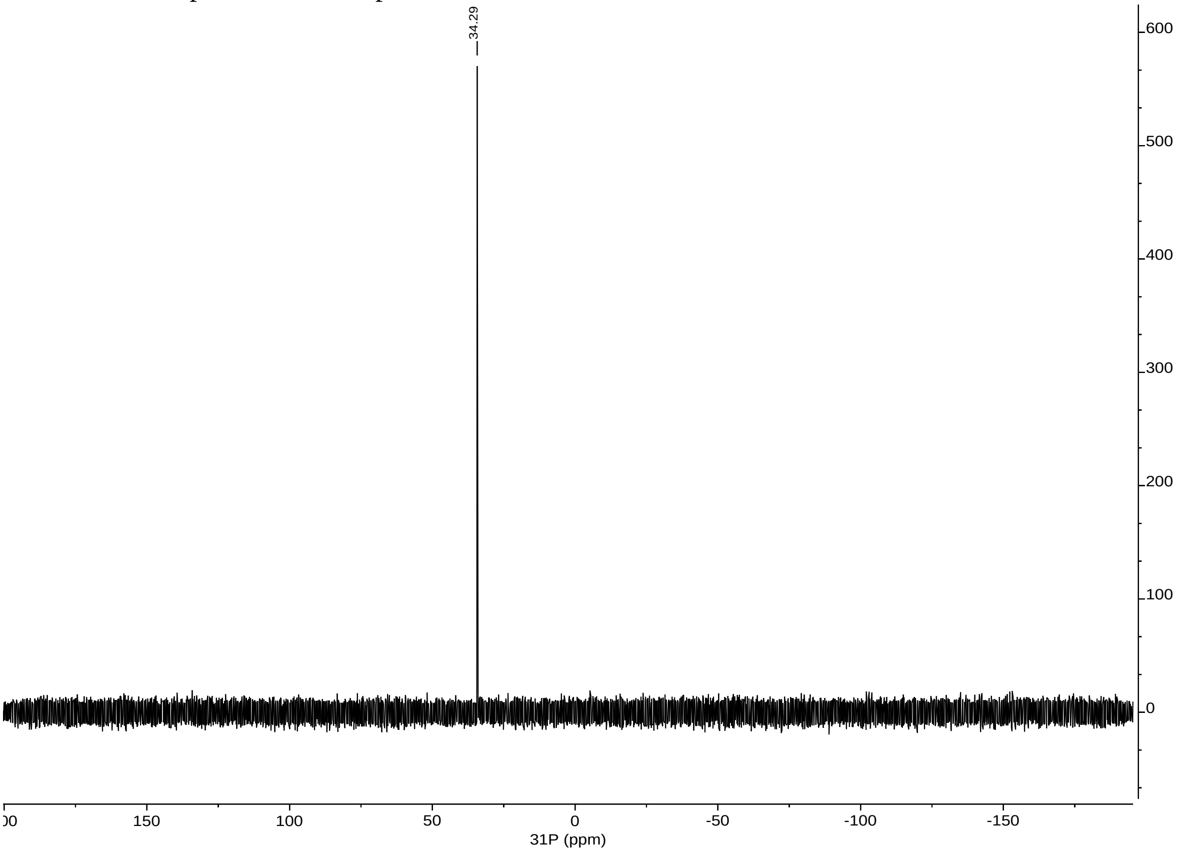

_N,N',N'',N'''_-Tetrakis(_tert_-butyloxycarbonyl) octane-1,8-diyl dipentyl bis((2-(bis(2-aminoethyl)-amino)ethyl)phosphonate) (S112)

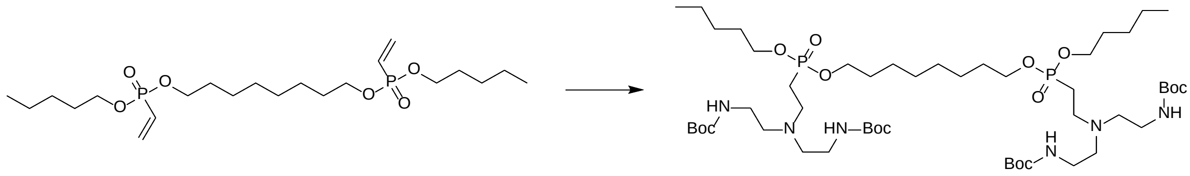

The title compound was prepared according to general method **D** from vinylphosphonate dimer **S70** (0.70 g, 1.50 mmol) and bis(2-*tert*-butyloxycarbonylaminoethyl)amine (1.43 g, 4.69 mmol) in 37% yield (0.62 g, 0.58 mmol) as a colourless oil.

^{1}H NMR (401 MHz, CD_3OD): 4.10–4.00 (m, 8H, $CH_3(CH_2)_2CH_2CH_2O$, $OCH_2CH_2(CH_2)_4CH_2CH_2O$), 3.11 (t, 8H, J = 6.3 Hz, NCH_2CH_2NH), 2.79 (dt, 4H, J = 10.8, 7.4 Hz, PCH_2CH_2N), 2.55 (t, 8H, J = 6.3 Hz, NCH_2CH_2NH), 2.05–1.95 (m, 4H, PCH_2CH_2N), 1.73–1.66 (m, 8H, $CH_3(CH_2)_2CH_2CH_2O$, $OCH_2CH_2(CH_2)_4CH_2CH_2O$), 1.45–1.35 (m, 52H, $CH_3(CH_2)_2CH_2CH_2O$, $OCH_2CH_2(CH_2)_4CH_2CH_2O$, $C(CH_3)_3$), 0.96–0.91 (m, 6H, $CH_3(CH_2)_3CH_2O$).

^{13}C NMR (101 MHz, CD_3OD): 158.47 (NHCOO), 80.02 ($C(CH_3)_3$), 67.17, 67.20 (d, J = 7.2 Hz $OCH_2CH_2(CH_2)_4CH_2CH_2O$, $CH_3(CH_2)_2CH_2CH_2O$), 54.39 (NCH_2CH_2NH), 48.44 (PCH_2CH_2N), 39.45 (NCH_2CH_2NH), 31.63, 31.34 (d, J = 6.3 Hz, $CH_3(CH_2)_2CH_2CH_2O$, $OCH_2CH_2(CH_2)_4CH_2CH_2O$), 28.86 ($C(CH_3)_3$, 30.24, 28.93, 26.70, 23.33 ($CH_3(CH_2)_2CH_2CH_2O$, $OCH_2CH_2(CH_2)_4CH_2CH_2O$), 23.95 (d, J = 136.6 Hz, PCH_2CH_2N), 14.39 ($CH_3(CH_2)_3CH_2O$).

^{31}P{^{1}H} NMR (162 MHz, CD_3OD): 34.77.

IR ν_{max} (KBr) 3451 (w), 3330 (w, br), 2980 (s), 2963 (vs), 2934 (vs), 2873 (m), 2861 (m), 2825 (w), 1704 (vs), 1507 (s), 1467 (m), 1456 (m), 1435 (w, sh), 1393 (m), 1367 (s), 1248 (s), 1174 (s), 1071 (m, sh), 1052 (m), 1038 (m), 1018 (m), 994 (s).

HR-MS(ESI^{+}): For $C_{50}H_{102}N_6O_{14}NaP_2$ $(M+Na)^{+}$ m/z calculated 1095.68215, found 1095.68237.

^{1}H NMR spectrum of compound **S112**

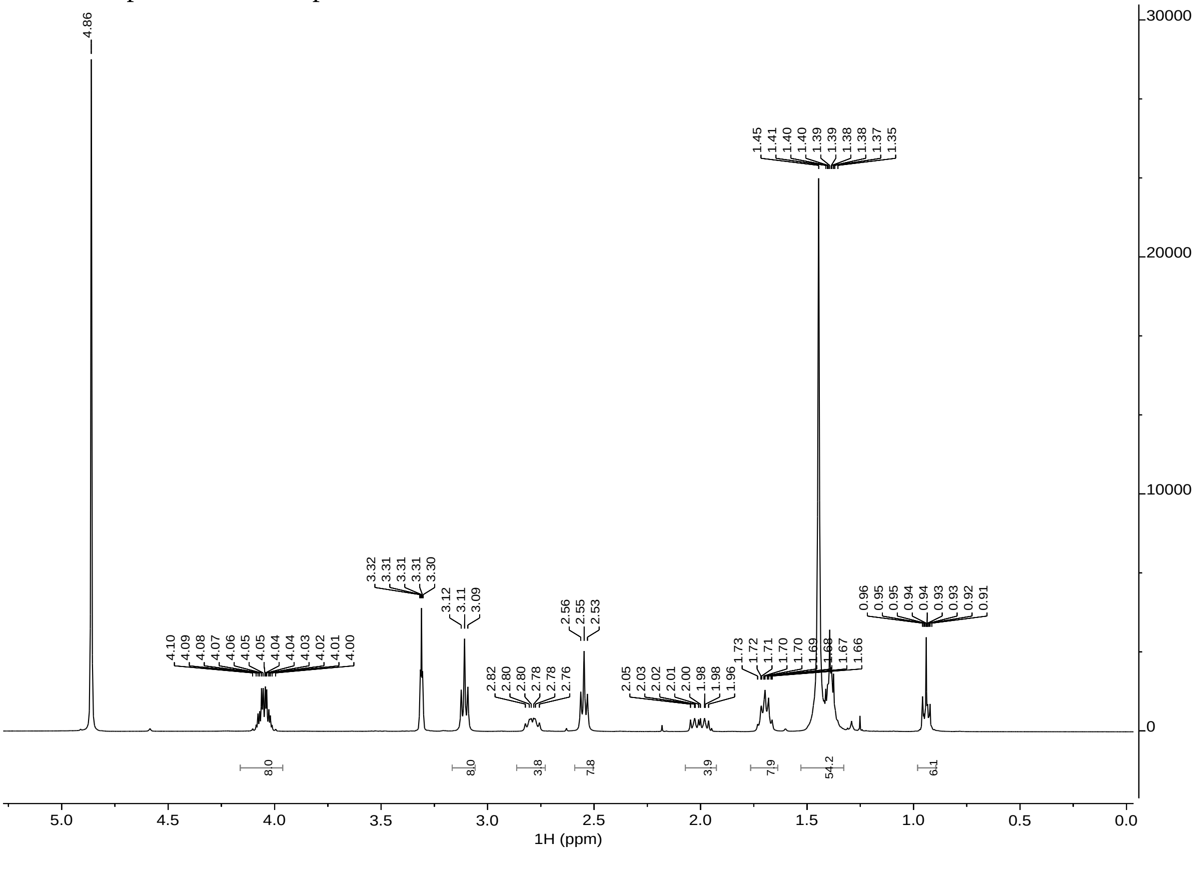

^{13}C NMR spectrum of compound **S112**

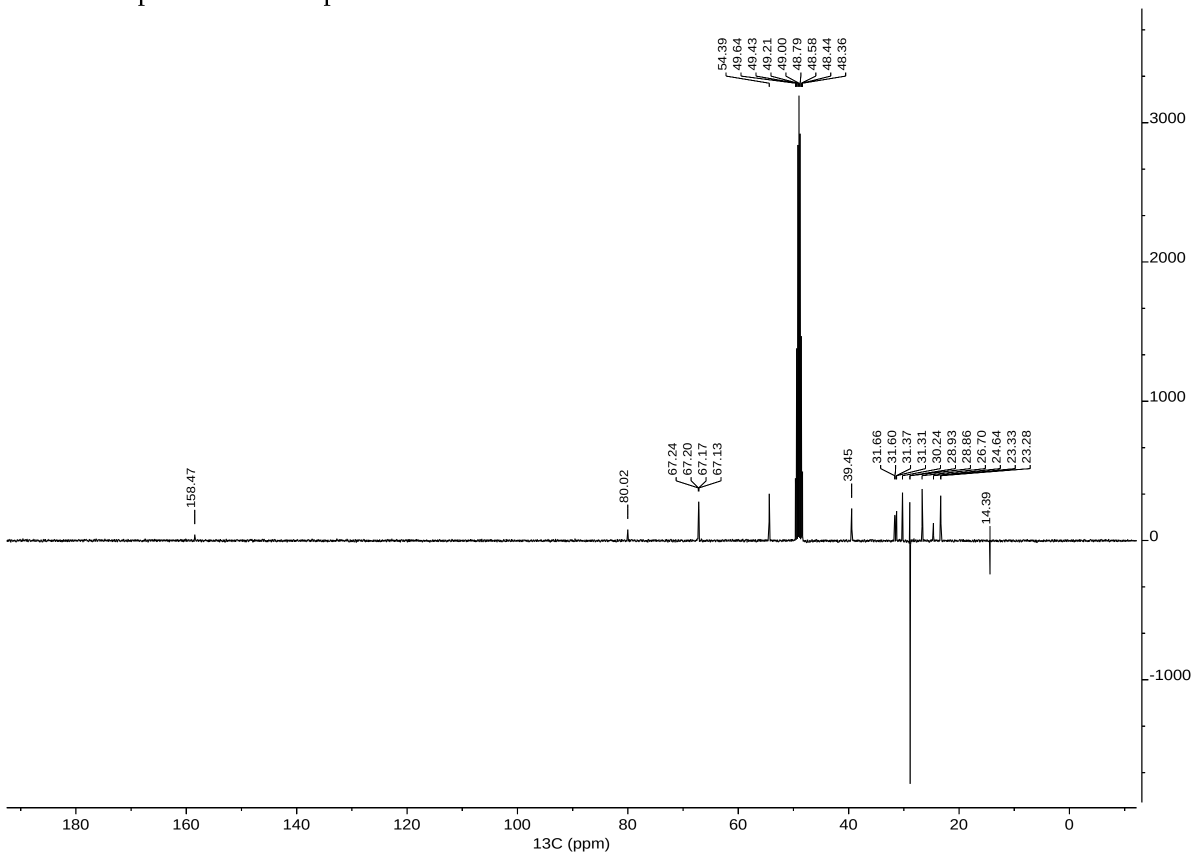

$^{31}P\{^1H\}$ NMR spectrum of compound **S112**

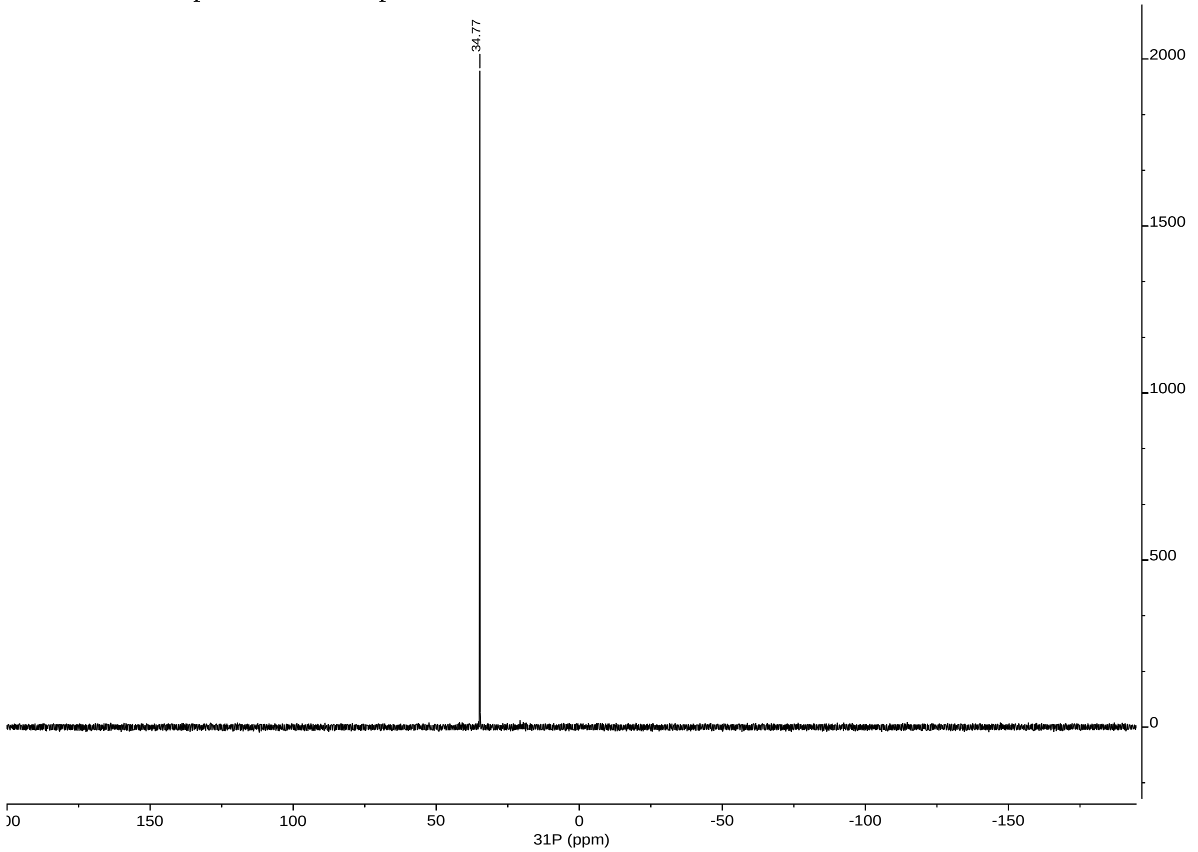

***N,N',N'',N'''*-Tetrakis(*tert*-butyloxycarbonyl) dihexyl octane-1,8-diyl bis((2-(bis(3-aminopropyl)amino)ethyl)phosphonate) (S113)**

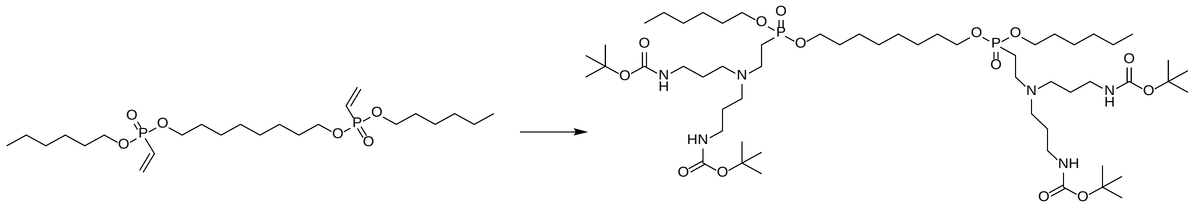

The title compound was prepared according to general method **D** from vinylphosphonate dimer **S71** (0.60 g, 1.21 mmol) and bis(3-*tert*-butyloxycarbonylaminopropyl)amine (1.61 g, 4.85 mmol) in 60% yield (0.85 g, 0.73 mmol) as a colourless oil.

^{1}H NMR (400 MHz, CD_3OD): 4.09–4.02 (m, 8H, C*H*$_2$O), 3.09 (t, 8H, *J* = 6.7 Hz, C*H*$_2$NH), 2.88–2.82 (m, 4H, PCH_2C*H*$_2$), 2.59–2.56 (m, 8H, C*H*$_2$(CH_2)$_2$$NH_2$), 2.09–2.02 (m, 4H, PC*H*$_2$), 1.72–1.62 (m, 16H, C*H*$_2$$CH_2$O, C*H*$_2$$CH_2$$NH_2$), 1.40–1.33 (m. 56H, CH_3C*H*$_2$, (C*H*$_2$)$_2$(CH_2)$_2$O, C(C*H*$_3$)$_3$), 0.94–0.91 (m, 6H, C*H*$_3$$CH_2$).

^{13}C NMR (101 MHz, CD_3OD): 155.42 (N*C*OO), 78.55 (*C*(CH_3)$_3$), 67.36 (d, *J* = 6.4 Hz), 67.31 (d, *J* = 6.4 Hz, *C*H_2O), 51.83 (*C*H_2(CH_2)$_2$NH), 47.75 (PCH_2*C*H_2), 39.42 (*C*H_2NH), 32.51 (CH_3CH_2*C*H_2), 31.61 (d, *J* = 6.3 Hz), 31.58 (d, *J* = 6.3 Hz, *C*H_2CH_2O), 28.84 (C(*C*H_3)$_3$), 27.80 (*C*H_2CH_2NH), 30.23, 26.68, 26.40 (CH_3(CH_2)$_2$*C*H_2, OC(CH_2)$_2$(*C*H_2)$_4$(CH_2)$_2$O), 23.65 (CH_3*C*H_2), 22.52 (d, *J* = 137.8 Hz, P*C*H_2), 14.40 (*C*H_3CH_2).

^{31}P{^{1}H} NMR (162 MHz, CD_3OD): 33.48.

IR ν_{max} (KBr) 3445 (w), 3361 (w, br), 2980 (m), 2959 (m), 2933 (s), 2871 (m), 2860 (m), 2820 (w, sh), 1710 (vs), 1507 (s), 1476 (w), 1467 (m), 1456 (m), 1393 (m), 1368 (s), 1249 (s), 1169 (s), 1057 (m), 1043 (m), 1010 (m), 1000 (m, sh), 463 (vw).

HR-MS(ESI$^+$): For $C_{56}H_{115}O_{14}N_6P_2$ (M+H)$^+$ *m*/*z* calculated 1157.79410, found 1157.79319.

^{1}H NMR spectrum of compound **S113**

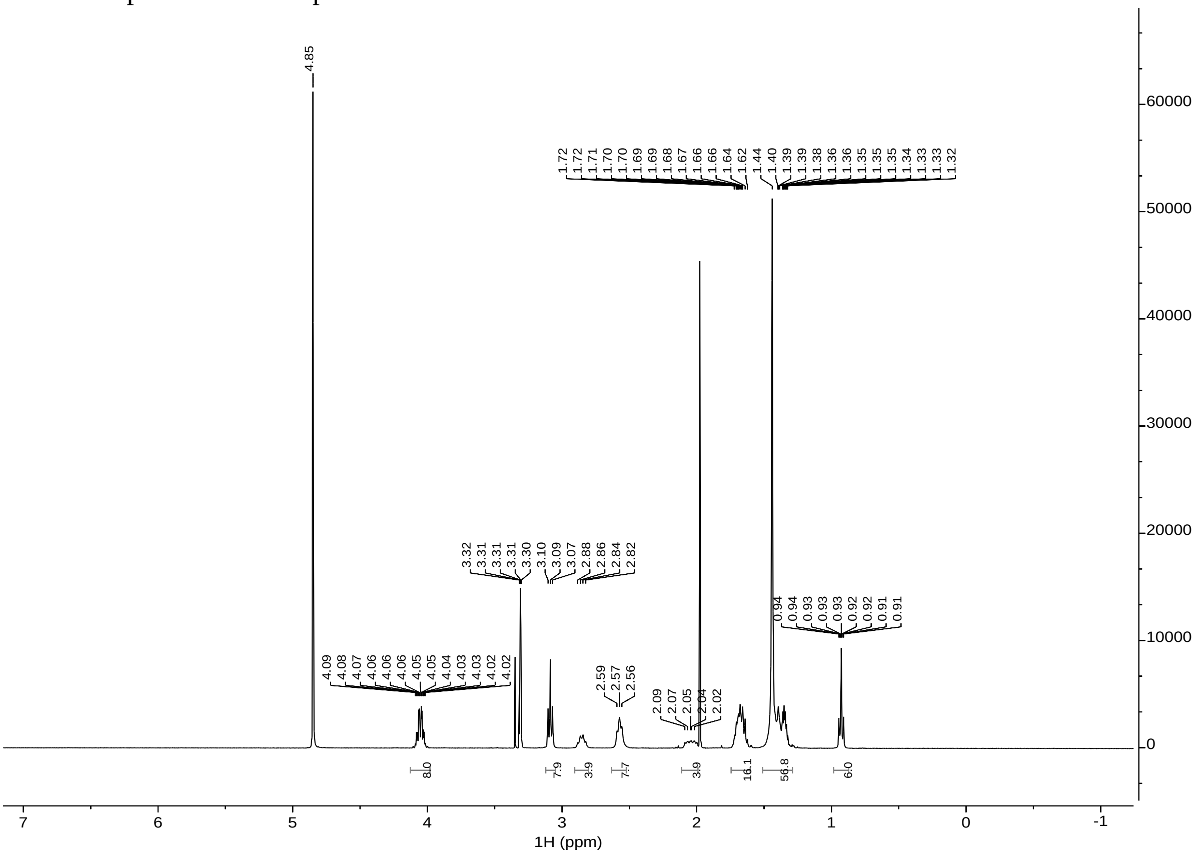

^{13}C NMR spectrum of compound **S113**

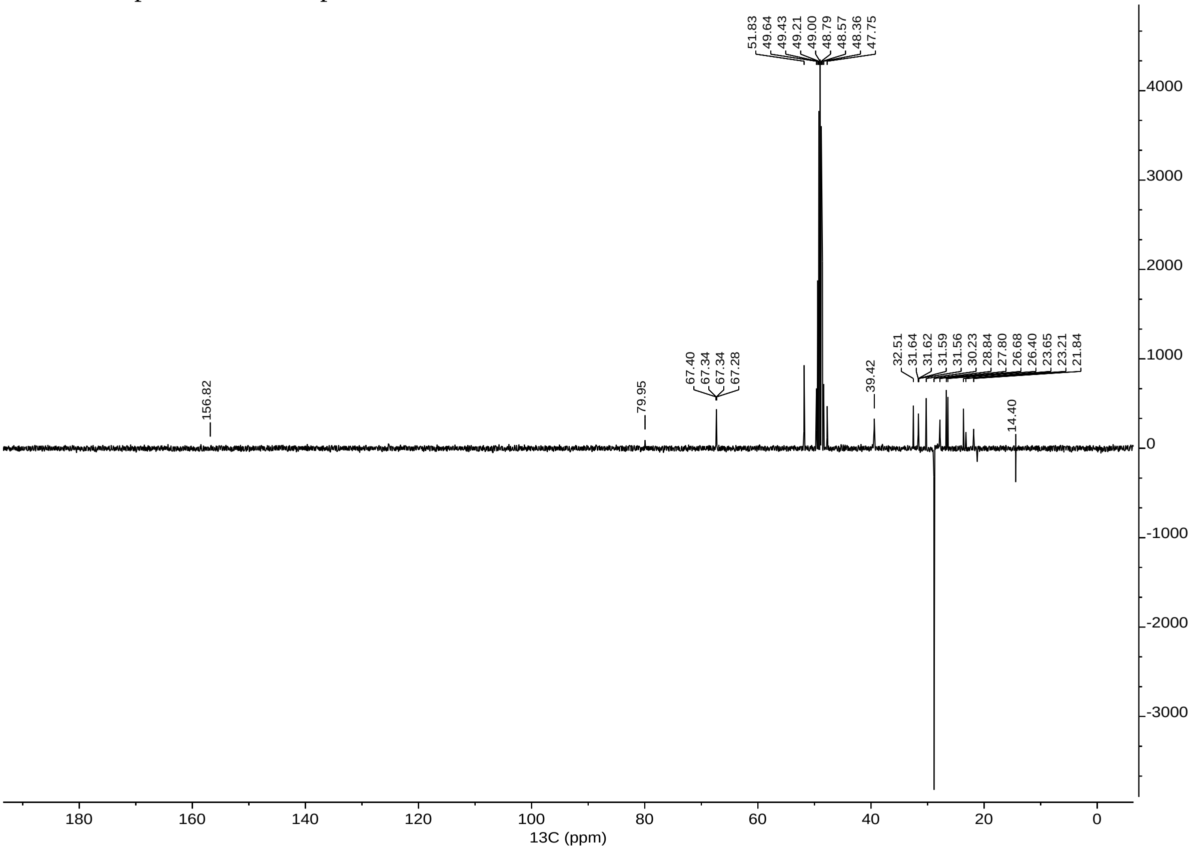

^{31}P{^{1}H} NMR spectrum of compound **S113**

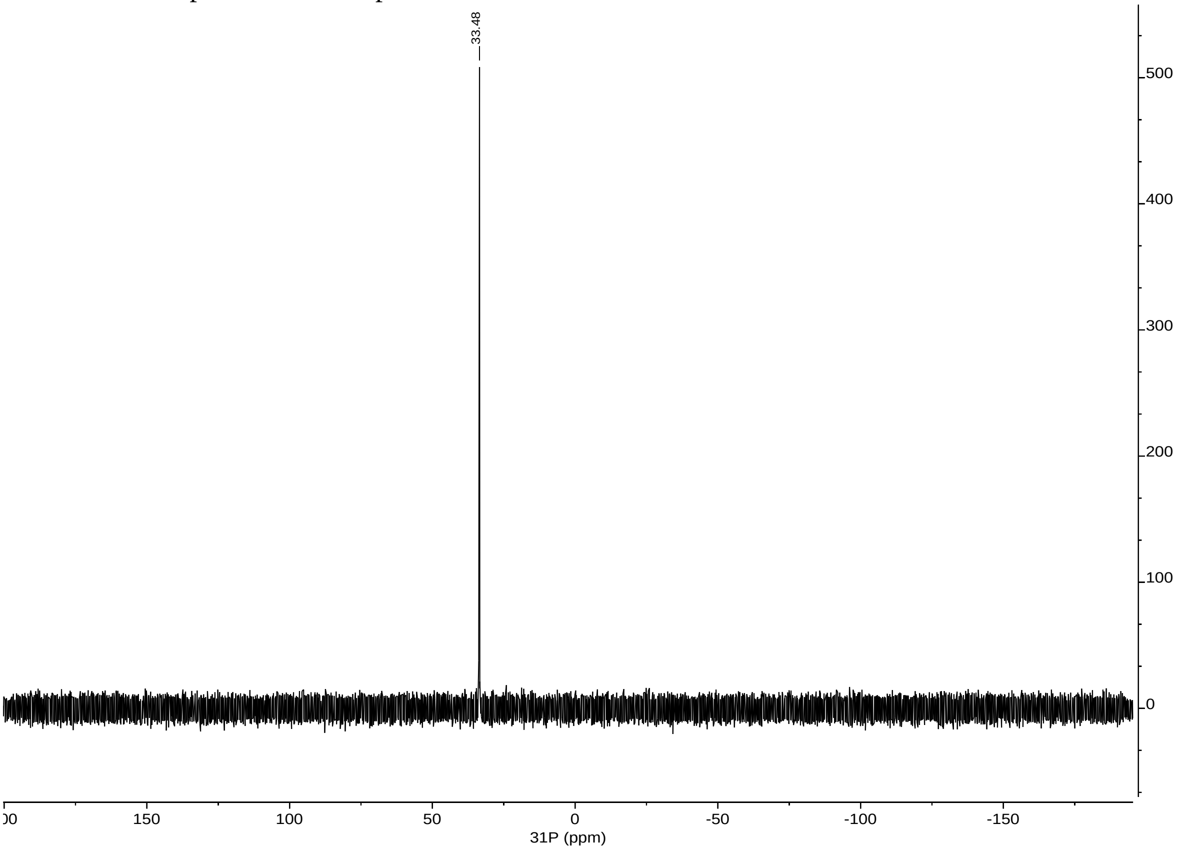

***N,N’,N’’,N’’’*-Tetrakis(*tert*-butyloxycarbonyl) dihexyl octane-1,8-diyl bis((2-(bis(2-aminoethyl)-amino)ethyl)phosphonate) (S114)**

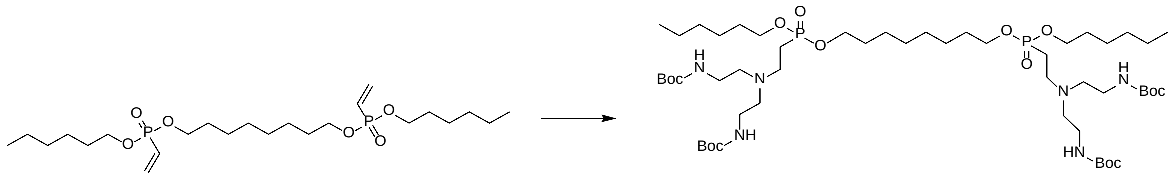

The title compound was prepared according to general method **D** from vinylphosphonate dimer **S71** (1.40 g, 2.83 mmol) and bis(2-*tert*-butyloxycarbonylaminoethyl)amine (3.44 g, 11.32 mmol) in 35% yield (1.20 g, 0.98 mmol) as a colourless oil.

^{1}H NMR (400 MHz, CD_3OD): 4.10–4.00 (m, 8H, $CH_3(CH_2)_3CH_2CH_2O$, $OCH_2CH_2(CH_2)_4CH_2CH_2O$), 3.11 (t, 8H, J = 6.2 Hz, NCH_2CH_2NH), 2.84–2.77 (m, 4H, PCH_2CH_2N), 2.56 (t, 8H, J = 6.3 Hz, $NCH_2CH_2NH_2$), 2.05–1.97 (m, 4H, PCH_2CH_2N), 1.73–1.66 (m, 8H, $CH_3(CH_2)_3CH_2CH_2O$, $OCH_2CH_2(CH_2)_4CH_2CH_2O$), 1.45–1.29 (m. 56H, $CH_3(CH_2)_3CH_2CH_2O$, $OCH_2CH_2(CH_2)_4CH_2CH_2O$, $C(CH_3)_3$), 0.94–0.91 (m, 6H, $CH_3(CH_2)_4CH_2O$).

^{13}C NMR (101 MHz, CD_3OD): 158.47 (NCOO), 80.04 ($C(CH_3)_3$), 67.24, 67.17 (d, J = 6.5 Hz, $OCH_2CH_2(CH_2)_4CH_2CH_2O$, $CH_3(CH_2)_3CH_2CH_2O$), 54.41 (NCH_2CH_2NH), 48.46 (PCH_2CH_2N), 39.41 (NCH_2CH_2NH), 32.52 ($CH_3CH_2CH_2CH_2CH_2CH_2O$), 31.71, 31.60 (d, J = 6.4 Hz, $CH_3(CH_2)_3CH_2CH_2O$, $OCH_2CH_2(CH_2)_4CH_2CH_2O$), 28.86 ($C(CH_3)_3$), 30.24, 26.69, 26.41 ($CH_3CH_2CH_2CH_2CH_2CH_2O$, $OCH_2CH_2(CH_2)_4CH_2CH_2O$), 23.94 (d, J = 136.5 Hz, PCH_2CH_2N), 23.65 ($CH_3CH_2CH_2CH_2CH_2CH_2O$), 14.40 ($CH_3(CH_2)_4CH_2O$).

^{31}P{^{1}H} NMR (162 MHz, CD_3OD): 34.41.

IR ν_{max} (KBr) 3451 (w), 3328 (w, br), 2980 (s), 2961 (m), 2933 (s), 2872 (m), 2860 (m), 2826 (m), 1704 (vs), 1507 (s), 1476 (w), 1467 (m), 1456 (m), 1393 (m), 1380 (w), 1367 (s), 1248 (s), 1170 (s), 1058 (s), 1039 (s), 1008 (s), 998 (s), 464 (vw).

HR-MS(ESI^{+}): For $C_{52}H_{106}O_{14}N_6NaP_2$ $(M+Na)^{+}$ m/z calculated 1123.71345, found 1123.71367.

^{1}H NMR spectrum of compound **S114**

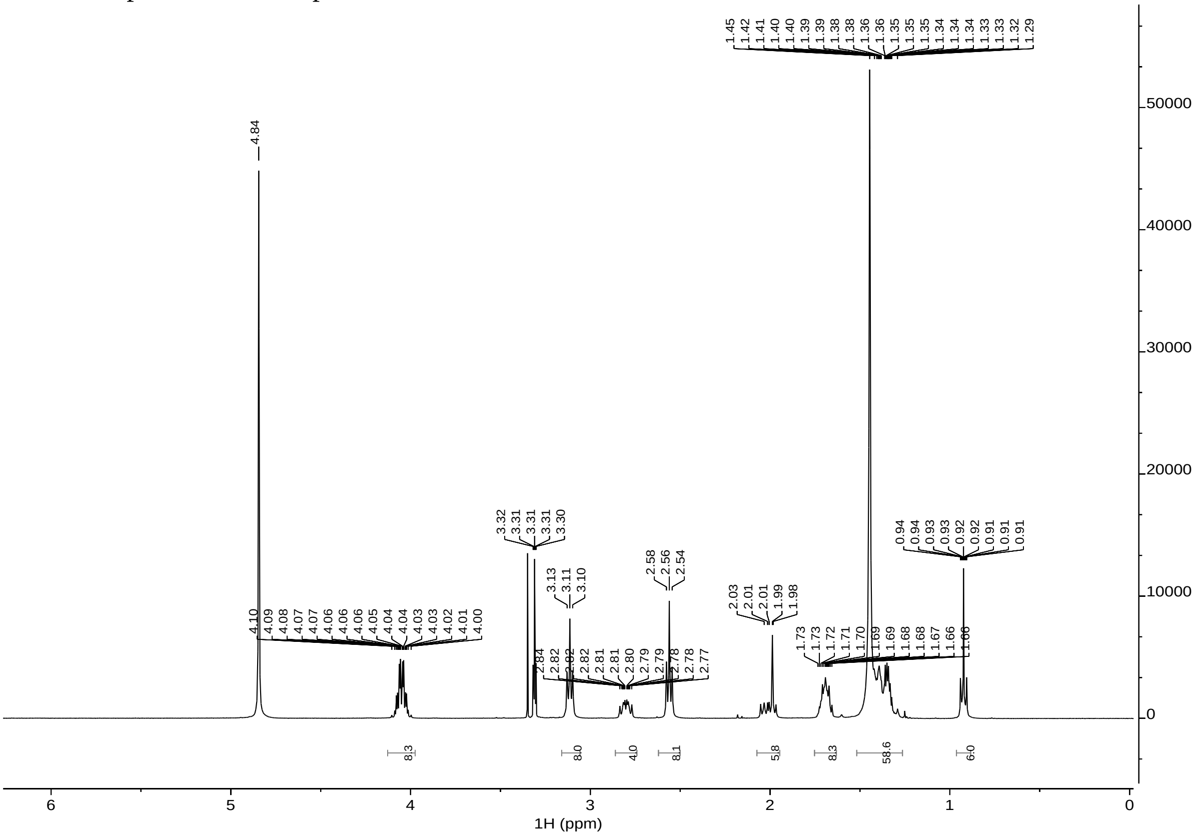

^{13}C NMR spectrum of compound **S114**

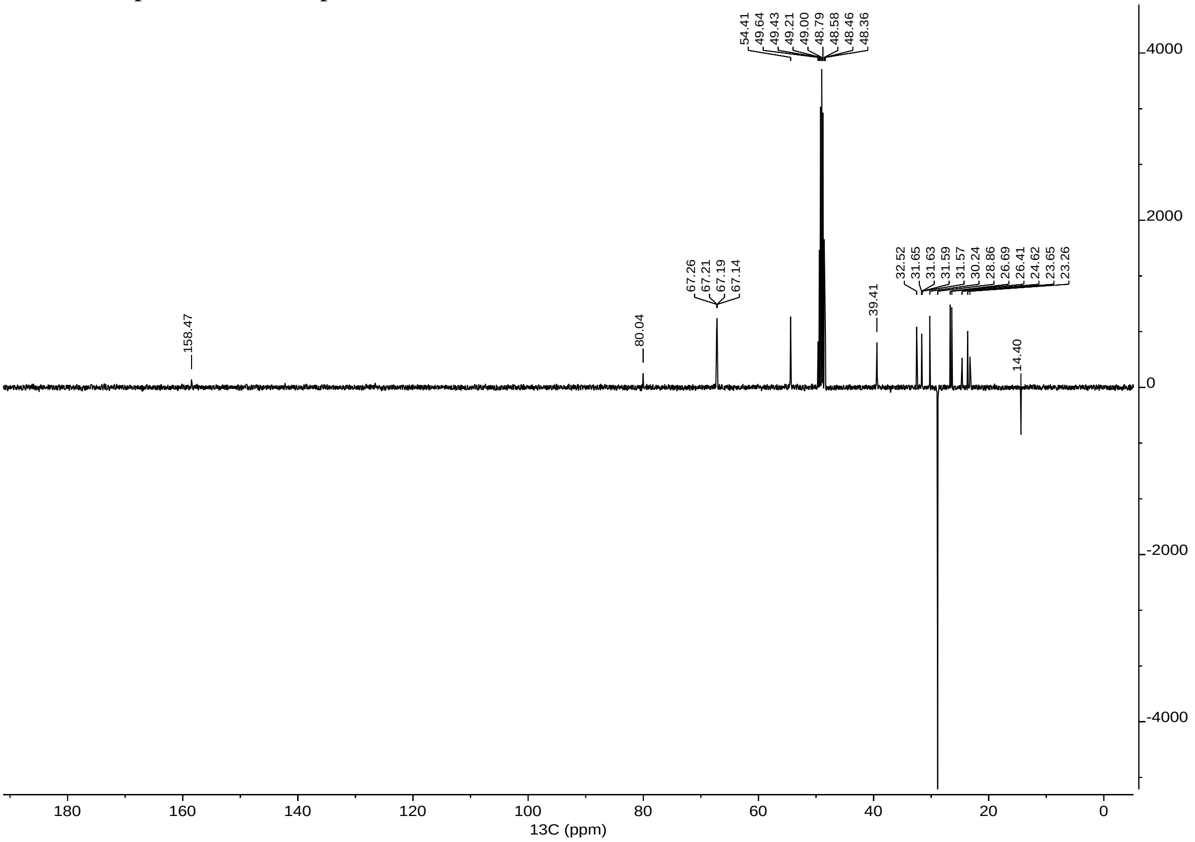

$^{31}P\{^1H\}$ NMR spectrum of compound **S114**

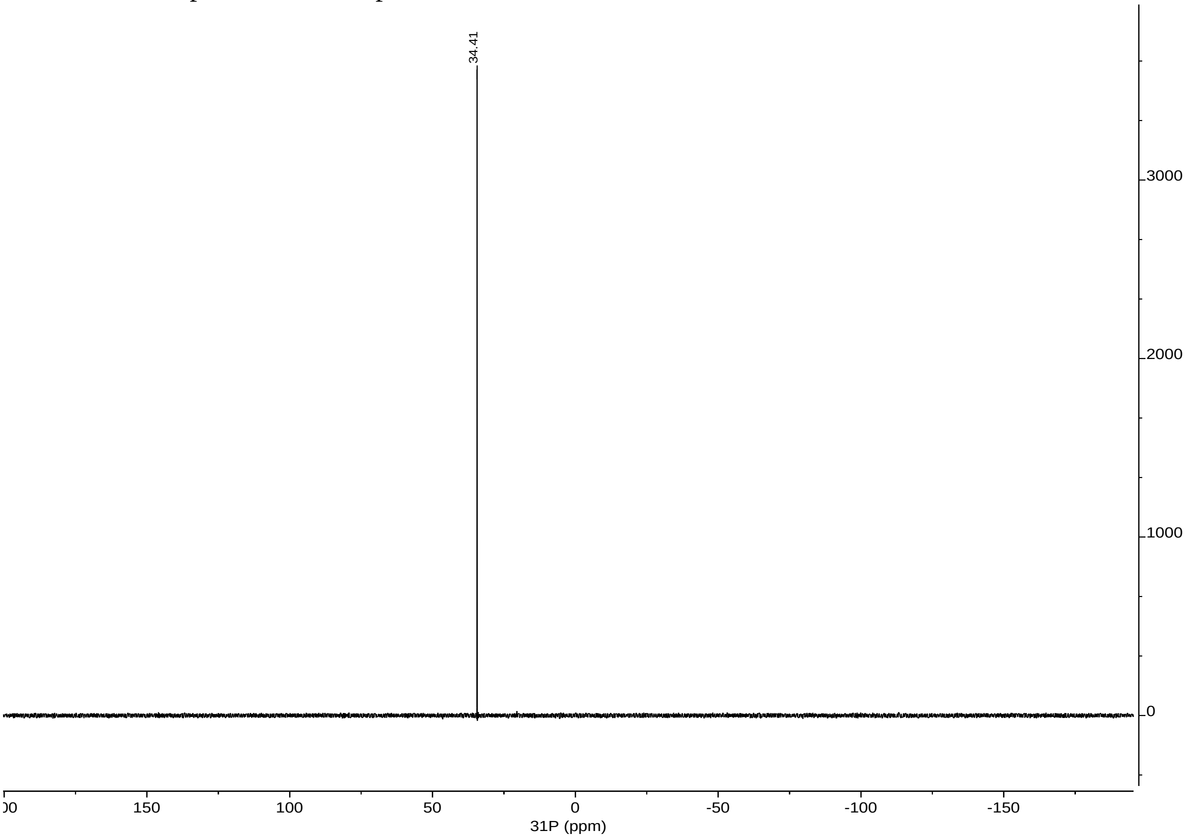

N,N',N'',N'''-Tetrakis(tert-butyloxycarbonyl) bis((Z)-hept-3-en-1-yl) octane-1,8-diyl bis((2-(bis(3-aminopropyl)amino)ethyl)phosphonate) (S115)

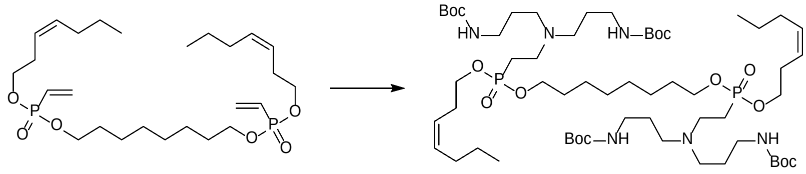

The title compound was prepared according to general method **D** from vinylphosphonate dimer **S72** (0.68 g, 1.31 mmol) and bis(3-*tert*-butyloxycarbonylaminopropyl)amine (1.74 g, 5.25 mmol) in 59% yield (0.92 g, 0.78 mmol) as a colourless oil.

^{1}H NMR (401 MHz, CD_3OD): 5.58–5.51 (m, 2H, $CH_3(CH_2)_2CH$), 5.45–5.38 (m, 2H, $CH(CH_2)_2O$), 4.08–4.00 (m, 8H, CH_2O), 3.08 (t, 8H, J = 6.7 Hz, CH_2NH), 2.80–2.74 (m, 4H, PCH_2CH_2), 2.49–2.42 (m, 12H, $CH_2(CH_2)_2NH$, $CHCH_2CH_2O$), 2.10–1.94 (m, 8H, PCH_2, $CH_3CH_2CH_2$), 1.73–1.61 (m, 12H, CH_2CH_2NH, $OCH_2CH_2(CH_2)_4CH_2CH_2O$), 1.46–1.37 (m, 48H, CH_3CH_2, $O(CH_2)_2(CH_2)_4(CH_2)_2O$, $C(CH_3)_3$), 0.93 (t, 6H, J = 7.4 Hz, CH_3CH_2).

^{13}C NMR (101 MHz, CD_3OD): 158.50 (NCOO), 133.93 ($CH_3(CH_2)_2CH$), 125.57 ($CH(CH_2)_2O$), 79.87 ($C(CH_3)_3$), 67.22 (d, J = 7.2 Hz, $CHCH_2CH_2O$), 66.71 (d, J = 7.0 Hz, $OCH_2(CH_2)_6CH_2O$), 51.86 ($CH_2(CH_2)_2NH$), 47.68 (PCH_2CH_2), 39.61 (CH_2NH), 31.54 (d, J = 6.3 Hz, $OCH_2CH_2(CH_2)_4CH_2CH_2O$), 30.46, 30.24 ($O(CH_2)_2(CH_2)_4(CH_2)_2O$), 29.71 (d, J = 5.8 Hz, $CHCH_2CH_2O$), 28.85 ($C(CH_3)_3$), 28.13 (CH_2CH_2NH), 23.82 (CH_3CH_2), 22.74 (d, J = 138.1 Hz, PCH_2), 14.19 (CH_3CH_2).

^{31}P{^{1}H} NMR (162 MHz, CD_3OD): 34.57.

IR ν_{max} (KBr) 3455 (m), 3369 (w), 2979 (s), 2964 (s), 2933 (s), 2871 (m), 2825 (m), 1707 (vs), 1507 (s), 1393 (m), 1367 (s), 1249 (vs), 1066–1010 (m), 463 (vw).

HR-MS(ESI$^+$): For $C_{58}H_{116}O_{14}N_6P_2$ $(M+2H)^{2+}$ $m/2z$ calculated 591.40069, found 591.40044.

^{1}H NMR spectrum of compound **S115**

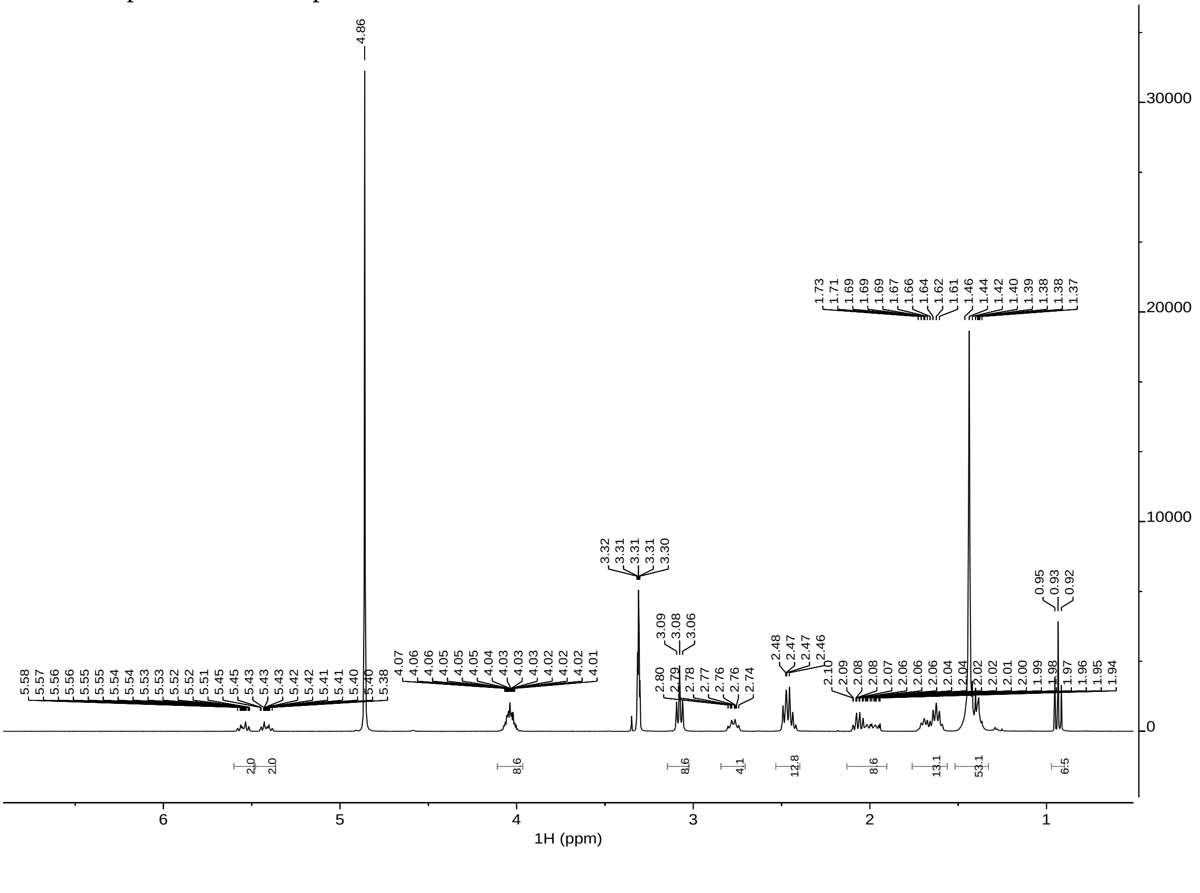

^{13}C NMR spectrum of compound **S115**

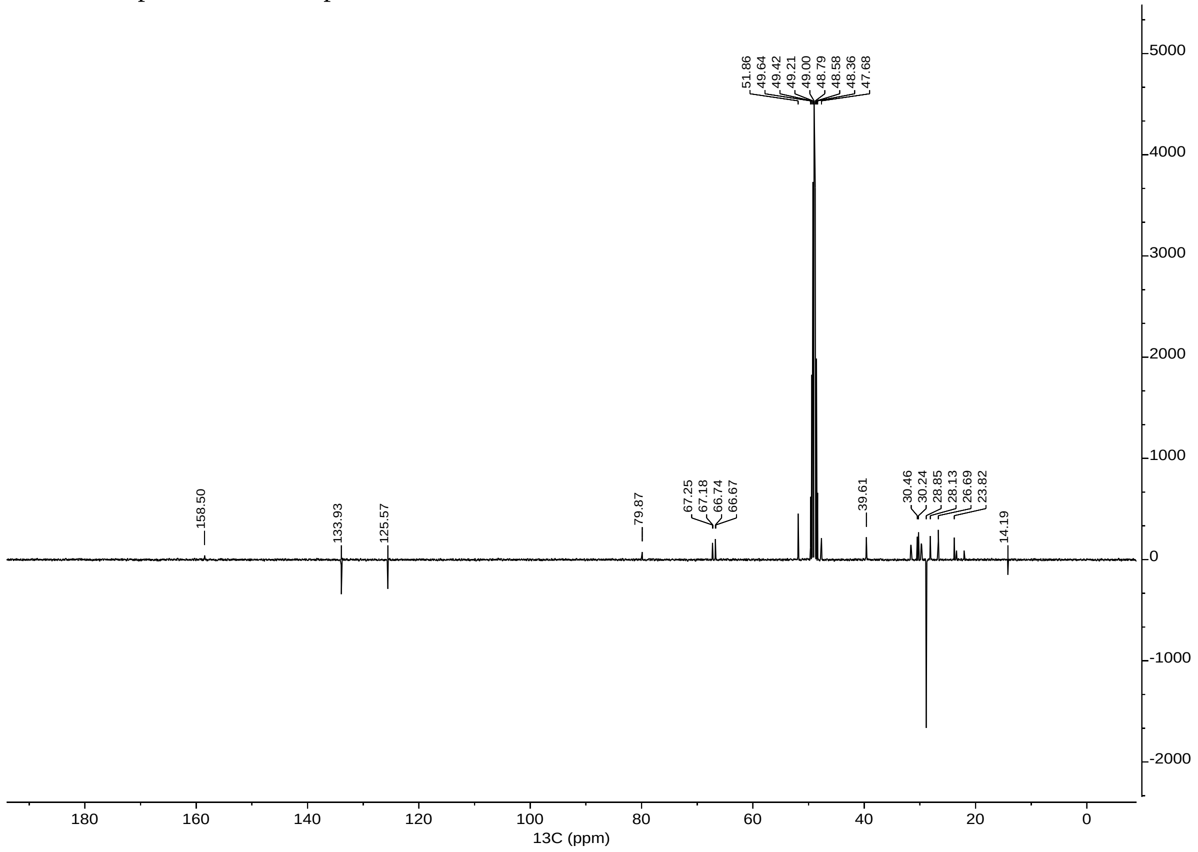

$^{31}P\{^{1}H\}$ NMR spectrum of compound **S115**

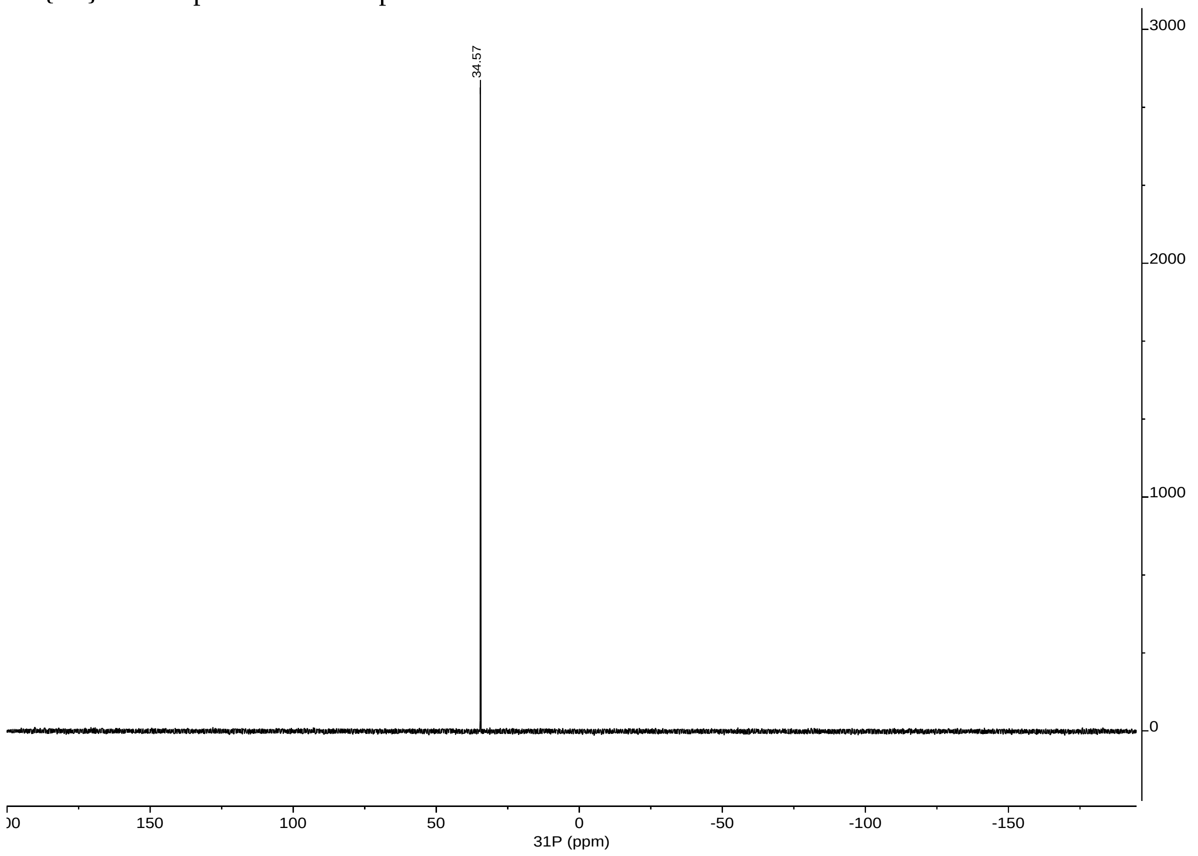

_N,N’,N’’,N’’’_-Tetrakis(_tert_-butyloxycarbonyl) octane-1,8-diyl dioctyl bis((2-(bis(3-aminopropyl)-amino)ethyl)phosphonate) (S116)

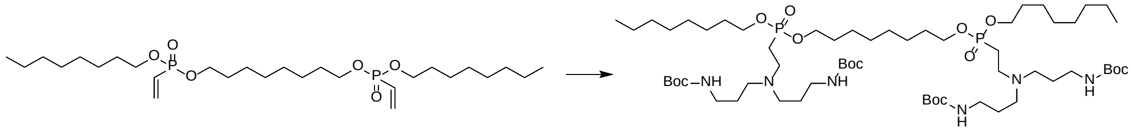

The title compound was prepared according to general method **D** from vinylphosphonate dimer **S73** (0.60 g, 1.09 mmol) and bis(3-*tert*-butyloxycarbonylaminopropyl)amine (1.45 g, 4.36 mmol) in 69% yield (0.91 g, 0.75 mmol) as a colourless oil.

^{1}H NMR (400 MHz, CD_3OD): 4.08–3.99 (m, 8H, $CH_3(CH_2)_5CH_2CH_2O$, $OCH_2CH_2(CH_2)_4CH_2CH_2O$), 3.08 (t, 8H, J = 6.7 Hz, $NCH_2CH_2CH_2NH$), 2.80–2.72 (m, 4H, PCH_2CH_2N), 2.49–2.45 (m, 8H, $NCH_2CH_2CH_2NH$), 2.03–1.93 (m, 4H, PCH_2CH_2N), 1.73–1.59 (m, 16H, $CH_3(CH_2)_5CH_2CH_2O$, $OCH_2CH_2(CH_2)_4CH_2CH_2O$, $NCH_2CH_2CH_2NH$), 1.44–1.28 (m, 64H, $CH_3(CH_2)_5CH_2CH_2O$, $C(CH_3)_3$, $OCH_2CH_2(CH_2)_4CH_2CH_2O$), 0.93–0.89 (m, 6H, $CH_3(CH_2)_6CH_2O$).

^{13}C NMR (101 MHz, CD_3OD): 158.46 (NCOO), 79.84 ($C(CH_3)_3$), 67.71, 67.23 (d, J = 7.0 Hz, $OCH_2CH_2(CH_2)_4CH_2CH_2O$, $CH_3(CH_2)_5CH_2CH_2O$), 51.88 ($NCH_2CH_2CH_2NH$), 47.70 (PCH_2CH_2N), 39.64 ($NCH_2CH_2CH_2NH$), 32.98 ($CH_3CH_2CH_2(CH_2)_3CH_2CH_2O$), 31.62 (d, J = 7.0 Hz, $OCH_2CH_2(CH_2)_4CH_2CH_2O$, $CH_3CH_2CH_2(CH_2)_3CH_2CH_2O$), 28.86 ($C(CH_3)_3$), 28.17 ($NCH_2CH_2CH_2NH$), 30.38, 30.24, 26.73, 26.70, 23.73 ($CH_3CH_2CH_2(CH_2)_3CH_2CH_2O$, $OCH_2CH_2(CH_2)_4CH_2CH_2O$), 22.78 (d, J = 138.2 Hz, PCH_2CH_2N), 14.49 ($CH_3(CH_2)_6CH_2O$).

^{31}P{^{1}H} NMR (162 MHz, CD_3OD): 34.37.

IR ν_{max} (KBr) 3455 (m), 3369 (w, br), 2980 (s), 2958 (m), 2932 (s), 2858 (m), 2826 (w), 1708 (vs), 1507 (s), 1475 (m), 1467 (m), 1457 (m), 1393 (m), 1367 (s), 1249 (s), 1071 (m, sh), 1040 (s), 1014 (s), 995 (s).

HR-MS(ESI$^+$): For $C_{60}H_{123}O_{14}N_6P_2$ $(M+H)^+$ m/z calculated 1213.86570, found 1213.85642.

^{1}H NMR spectrum of compound **S116**

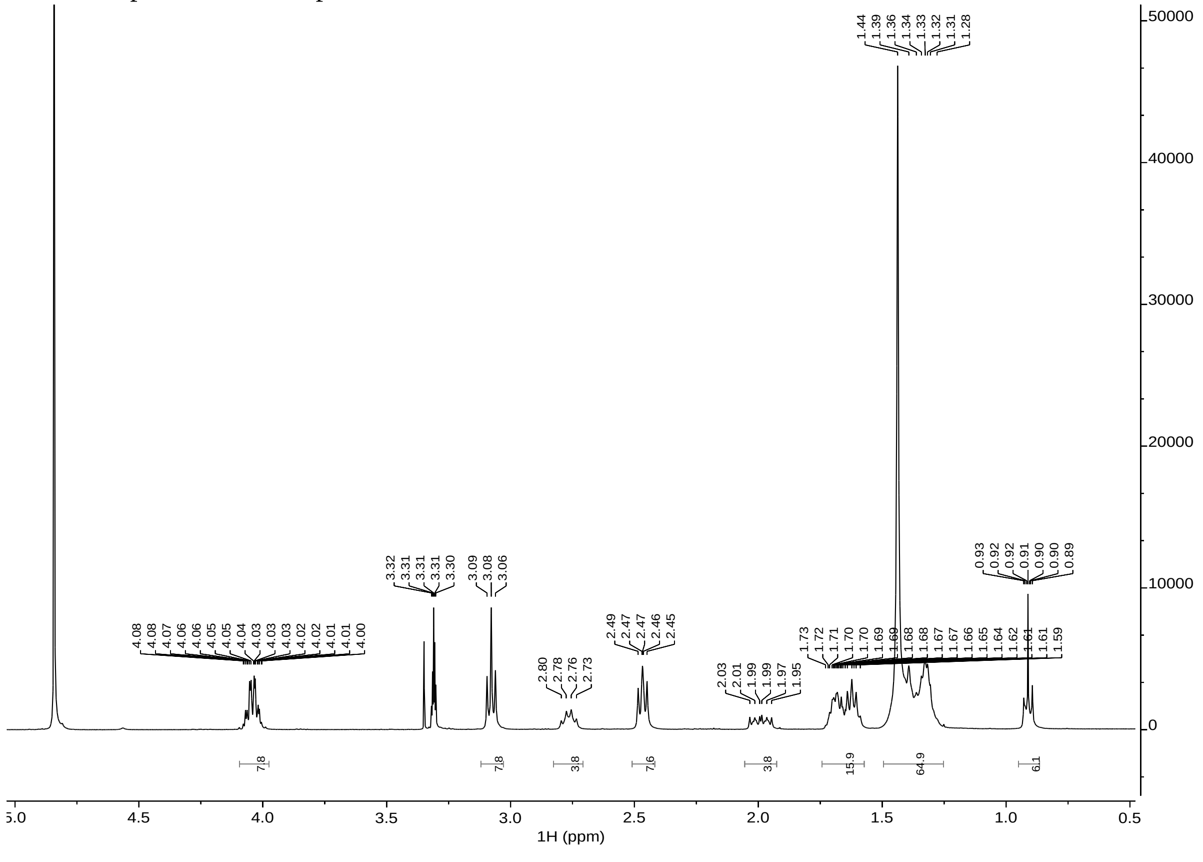

^{13}C NMR spectrum of compound **S116**

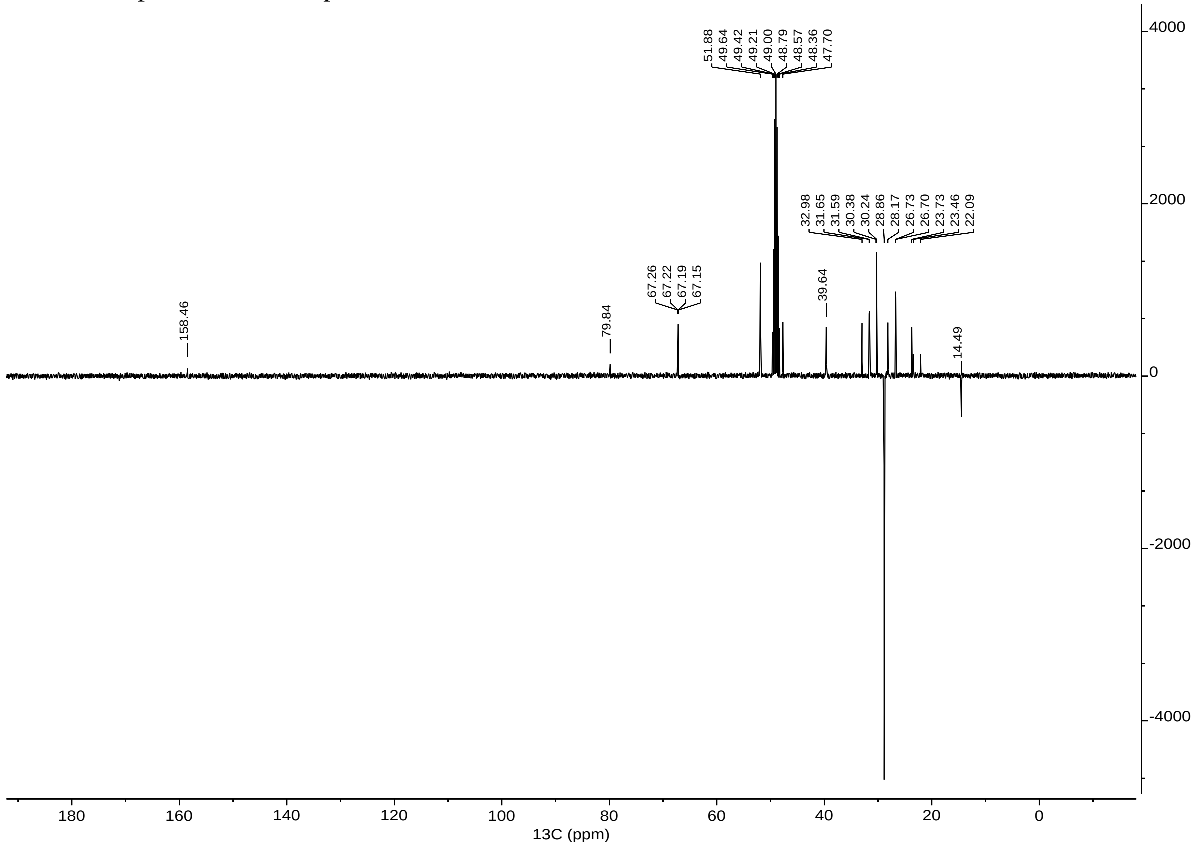

^{31}P{^{1}H} NMR spectrum of compound **S116**

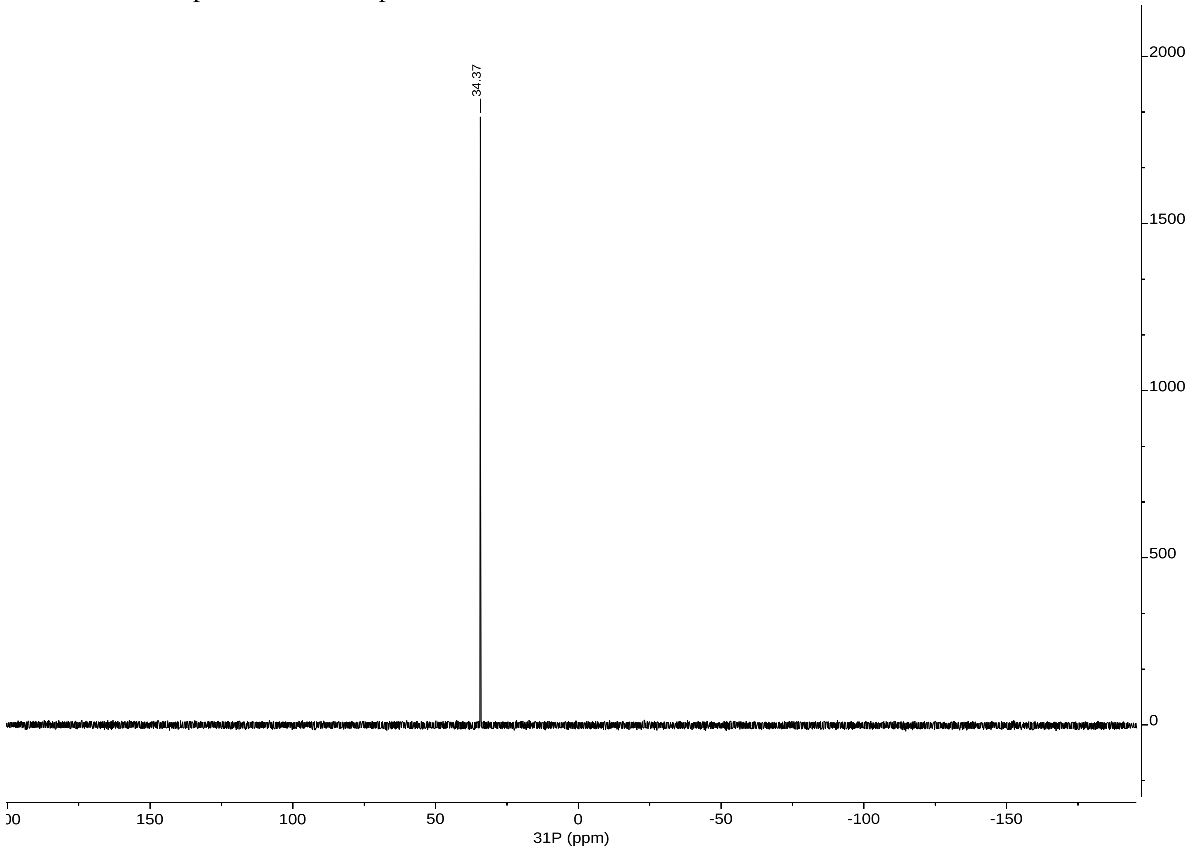

***N,N',N'',N'''*-Tetrakis(*tert*-butyloxycarbonyl) octane-1,8-diyl dioctyl bis((2-(bis(2-aminoethyl)-amino)ethyl)phosphonate) (S117)**

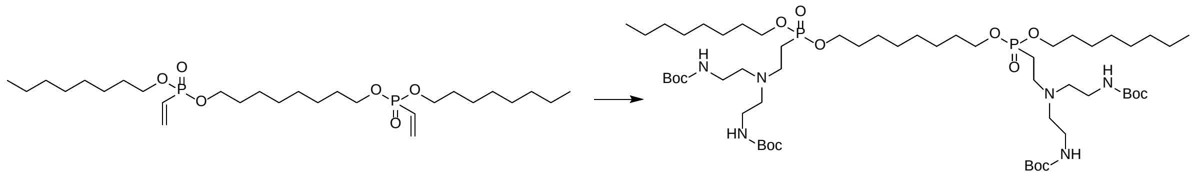

The title compound was prepared according to general method **D** from vinylphosphonate dimer **S73** (0.65 g, 1.18 mmol) and bis(2-*tert*-butyloxycarbonylaminoethyl)amine (1.43g, 4.72 mmol) in 43% yield (0.59 g, 0.51 mmol) as a colourless oil.

^{1}H NMR (400 MHz, CD_3OD): 4.25–4.01 (m, 8H, $CH_3(CH_2)_5CH_2C\textit{H}_2O$, $OC\textit{H}_2CH_2(CH_2)_4CH_2C\textit{H}_2O$), 3.11 (t, 8H, J = 6.3 Hz, $NCH_2C\textit{H}_2NH$), 2.79 (dt, 4H, J = 11.0, 7.5 Hz, $PCH_2C\textit{H}_2N$), 2.55 (t, J = 6.3 Hz, 8H, $NC\textit{H}_2CH_2NH$), 2.05–1.96 (m, 4H, $PC\textit{H}_2CH_2N$), 1.73–1.66 (m, 8H, $CH_3(CH_2)_5C\textit{H}_2CH_2O$, $OCH_2C\textit{H}_2(CH_2)_4C\textit{H}_2CH_2O$), 1.47–1.25 (m, 64H, $CH_3(C\textit{H}_2)_5CH_2CH_2O$, $C(C\textit{H}_3)_3$, $OCH_2CH_2(C\textit{H}_2)_4CH_2CH_2O$), 0.92–0.89 (m, 6H, $C\textit{H}_3(CH_2)_6CH_2O$).

^{13}C NMR (101 MHz, CD_3OD): 157.78 (N*C*OO), 79.99 ($\textit{C}(CH_3)_3$), 67.21, 67.17 (d, J = 6.8 Hz, $CH_3(CH_2)_5CH_2\textit{C}H_2O$, $O\textit{C}H_2CH_2(CH_2)_4CH_2\textit{C}H_2O$), 54.41 ($N\textit{C}H_2CH_2NH$), 48.46 ($PCH_2\textit{C}H_2N$), 39.46 ($NCH_2\textit{C}H_2NH$), 32.98 ($CH_3CH_2\textit{C}H_2(CH_2)_3CH_2CH_2O$), 31.59 (d, J = 6.7 Hz, $CH_3(CH_2)_5\textit{C}H_2CH_2O$, $OCH_2\textit{C}H_2(CH_2)_4\textit{C}H_2CH_2O$), 28.87 ($C(\textit{C}H_3)_3$), 30.38, 30.26, 26.75, 26.71 ($CH_3CH_2CH_2(\textit{C}H_2)_3CH_2CH_2O$, $OCH_2CH_2(\textit{C}H_2)_4CH_2CH_2O$), 23.99 (d, J = 136.4 Hz, $P\textit{C}H_2CH_2N$), 23.73 ($CH_3\textit{C}H_2(CH_2)_4CH_2CH_2O$), 14.48 ($\textit{C}H_3(CH_2)_6CH_2O$).

^{31}P{^{1}H} NMR (162 MHz, CD_3OD): 34.49.

IR ν_{max} (KBr) 3452 (w), 3328 (w, br), 2979 (s), 2959 (s), 2873 (m), 2858 (m), 2827 (w), 1707 (vs), 1504 (s), 1468 (m), 1456 (m), 1393 (m), 1368 (s), 1248 (s), 1038 (m), 1000 (s), 984 (m).

HR-MS(ESI$^+$): For $C_{56}H_{115}O_{14}N_6P_2$ $(M+H)^+$ m/z calculated 1157.79410, found 1157.79462.

^{1}H NMR spectrum of compound **S117**

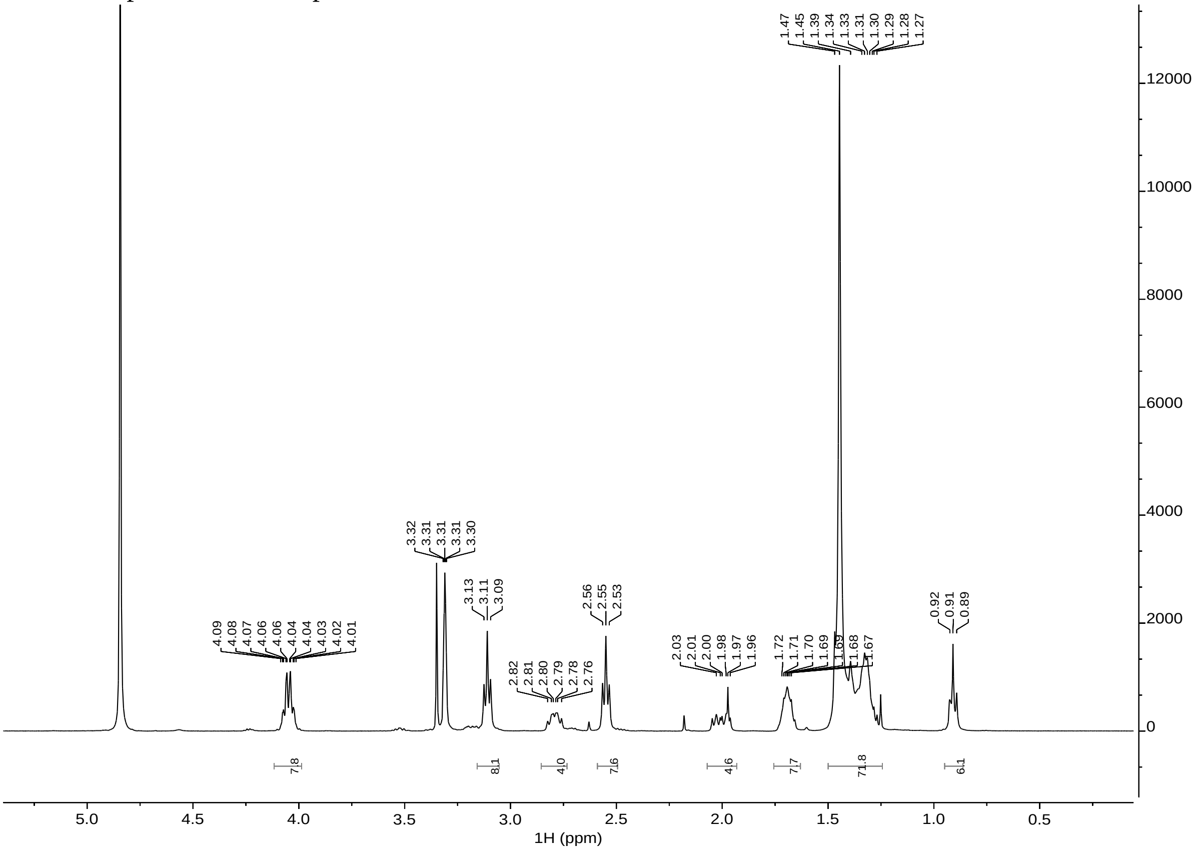

^{13}C NMR spectrum of compound **S117**

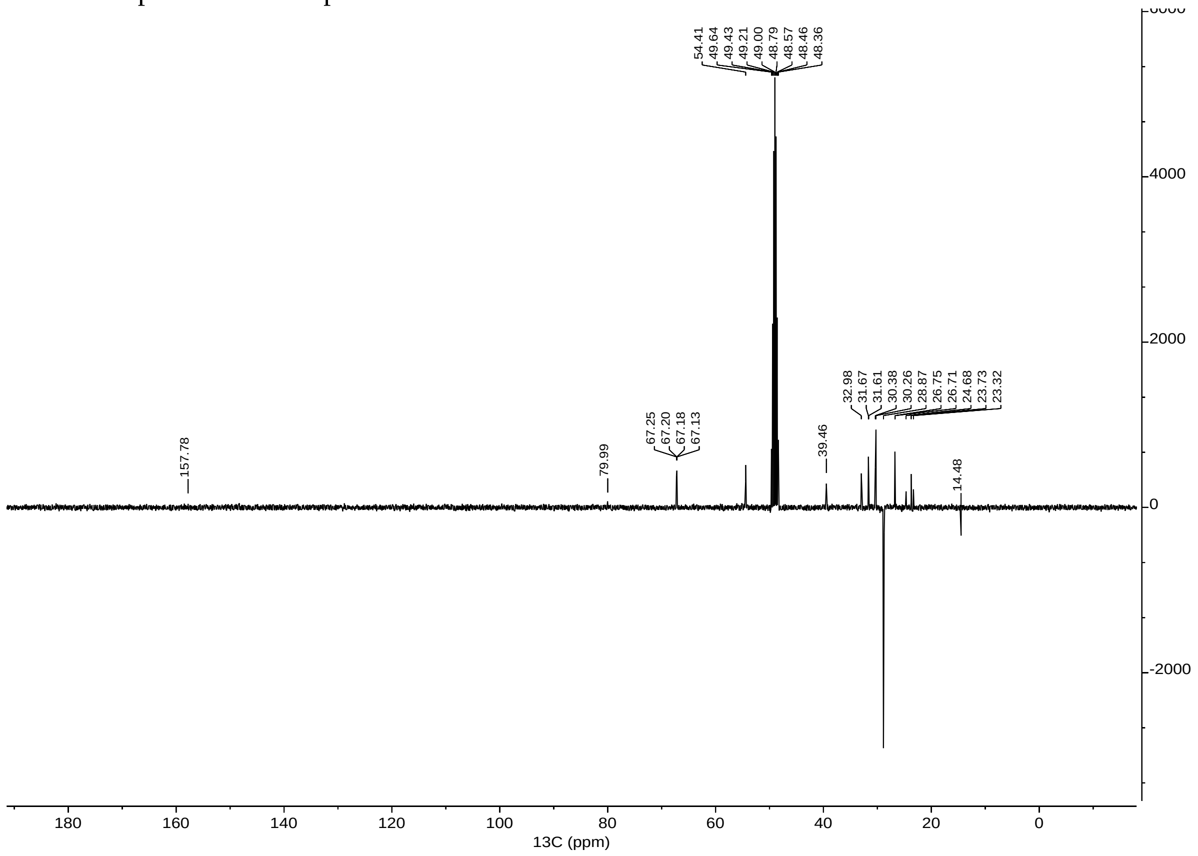

^{31}P{^{1}H} NMR spectrum of compound **S117**

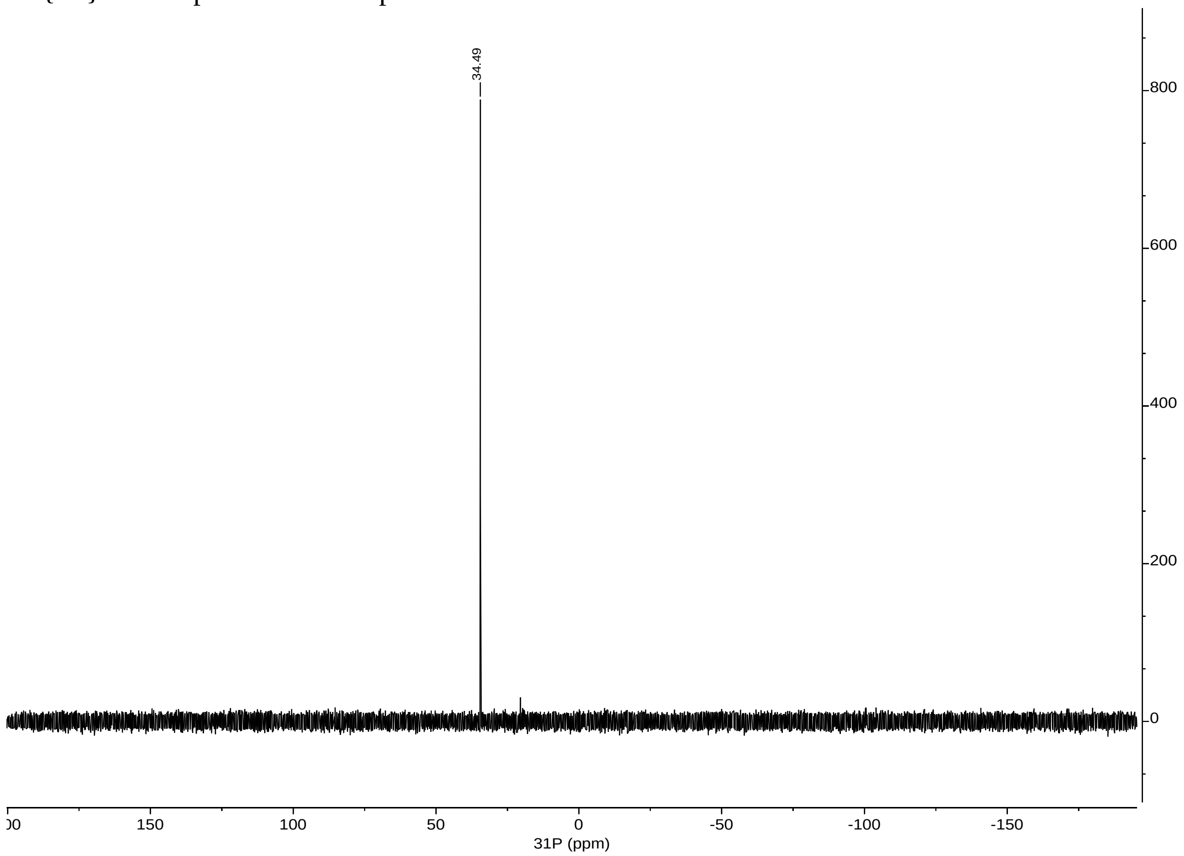

***N,N',N'',N'''*-Tetrakis(*tert*-butyloxycarbonyl) dibutyl decane-1,10-diyl bis((2-(bis(3-aminopropyl)amino)ethyl)phosphonate) (S118)**

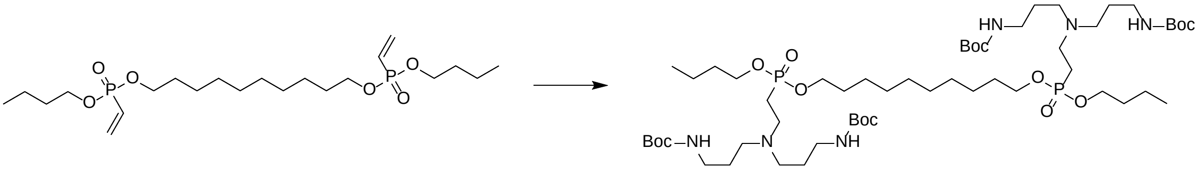

The title compound was prepared according to general method **D** from vinylphosphonate dimer **S76** (1.0 g, 2.14 mmol) and bis(3-*tert*-butyloxycarbonylaminopropyl)amine (2.84 g, 8.57 mmol) in 52% yield (1.26 g, 1.11 mmol) as a colourless oil.

^{1}H NMR (401 MHz, CD_3OD): 4.09–4.00 (m, 8H, $CH_3CH_2CH_2CH_2O$, $OCH_2CH_2(CH_2)_6CH_2CH_2O$), 3.08 (t, 8H, J = 6.7 Hz, $NCH_2CH_2CH_2NH$), 2.81–2.75 (m, 4H, PCH_2CH_2N), 2.50–2.47 (m, 8H, $NCH_2CH_2CH_2NH$), 2.04–1.95 (m, 4H, PCH_2CH_2N), 1.72–1.61 (m, 16H, $CH_3CH_2CH_2CH_2O$, $OCH_2CH_2(CH_2)_6CH_2CH_2O$, $NCH_2CH_2CH_2NH$), 1.47–1.34 (m, 52H, $CH_3CH_2CH_2CH_2O$, $OCH_2CH_2(CH_2)_6CH_2CH_2O$, $C(CH_3)_3$), 0.97 (t, 6H, J = 7.6 Hz, $CH_3(CH_2)_2CH_2O$).

^{13}C NMR (101 MHz, CD_3OD): 158.50(NHCOO), 79.87 ($C(CH_3)_3$), 67.24, 66.95 (d, J = 6.9 Hz, $CH_3CH_2CH_2CH_2O$, $OCH_2CH_2(CH_2)_6CH_2CH_2O$), 51.86 ($NCH_2CH_2CH_2NH$), 47.70 ($PCH_2CH_2N$), 39.59 ($NCH_2CH_2CH_2NH$), 33.67, 31.62 (d, J = 6.0 Hz, $CH_3CH_2CH_2CH_2O$, $OCH_2CH_2(CH_2)_6CH_2CH_2O$), 28.85 ($C(CH_3)_3$, 28.10 ($NCH_2CH_2CH_2NH$), 30.63, 30.27, 26.72 ($OCH_2CH_2(CH_2)_6CH_2CH_2O$), 22.71 (d, J = 138.3 Hz, PCH_2CH_2N), 19.86 ($CH_3CH_2CH_2CH_2O$), 13.98 ($CH_3CH_2)_2CH_2O$).

^{31}P{^{1}H} NMR (162 MHz, CD_3OD): 34.45.

IR ν_{max} (KBr) 3455 (m), 3369 (w, vbr), 2980 (s), 2966 (s), 2934 (s), 2868 (m), 2858 (m), 2824 (m), 1707 (vs), 1507 (vs), 1477 (m), 1466 (m), 1457 (s), 1393 (s), 1381 (w, sh), 1367 (vs), 1350 (m), 1249 (vs), 1169 (vs), 1064 (s), 1048 (s, sh), 1027 (s), 1013 (s, sh), 989 (s), 463 (w).

HR-MS(ESI$^+$): For $C_{54}H_{111}N_6O_{14}P_2$ $(M+H)^+$ m/z calculated 1129.76280, found 1129.76210.

^{1}H NMR spectrum of compound **S118**

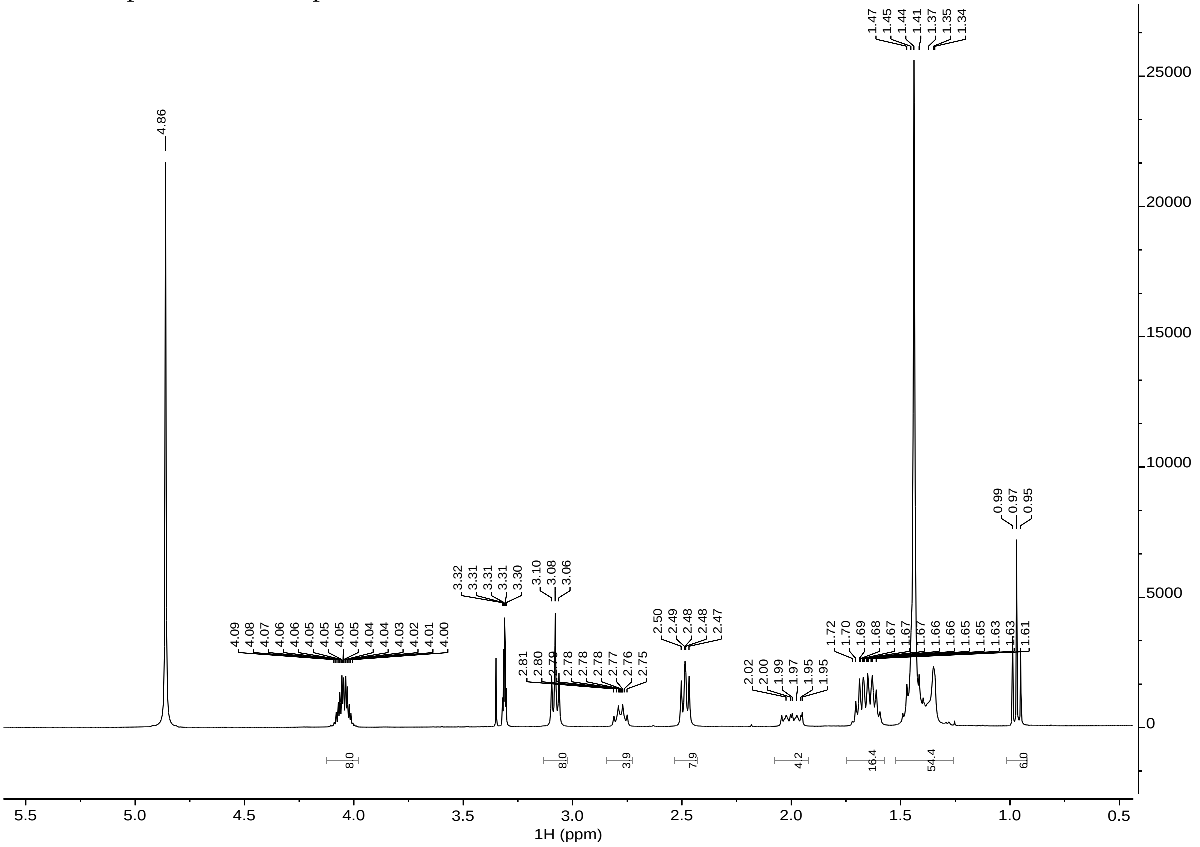

^{13}C NMR spectrum of compound **S118**

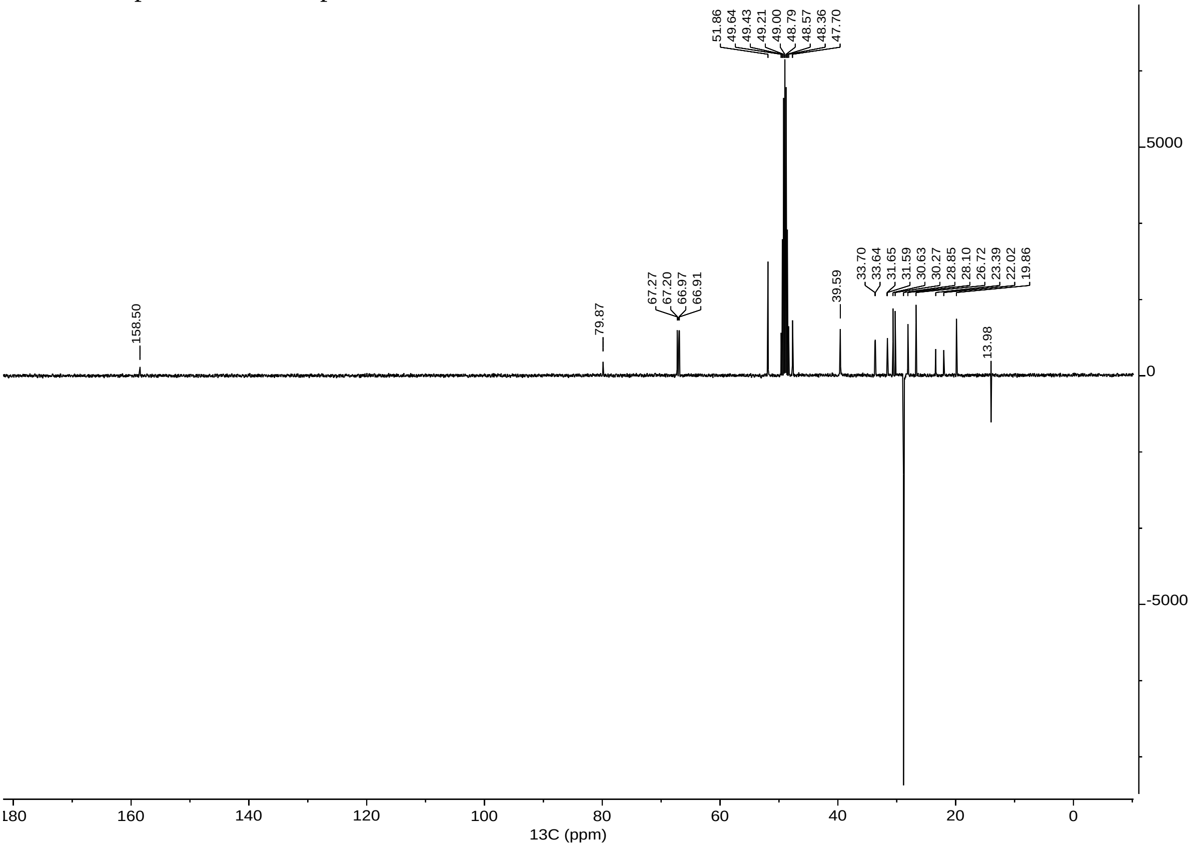

$^{31}P\{^1H\}$ NMR spectrum of compound **S118**

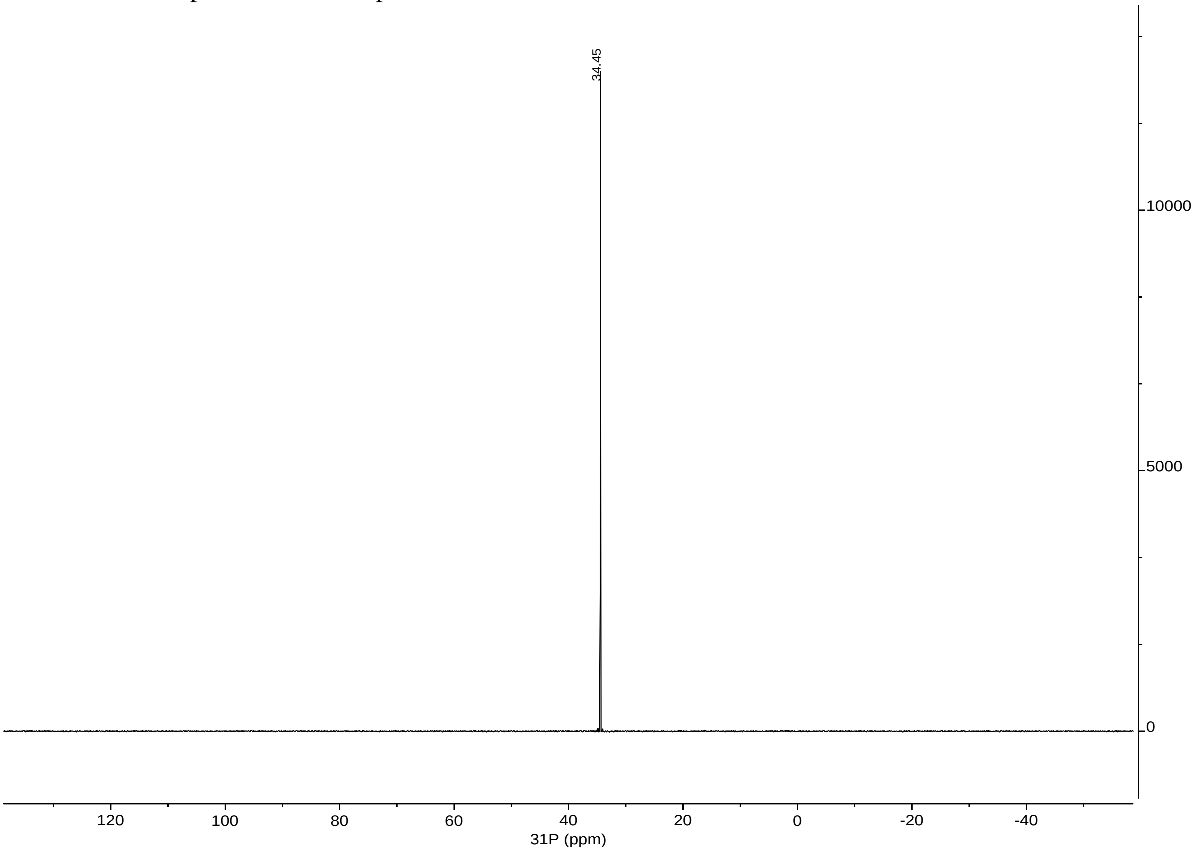

_N,N’,N’’,N’’’_-Tetrakis(_tert_-butyloxycarbonyl) dibutyl decane-1,10-diyl bis((2-(bis(2-aminoethyl)amino)ethyl)phosphonate) (S119)

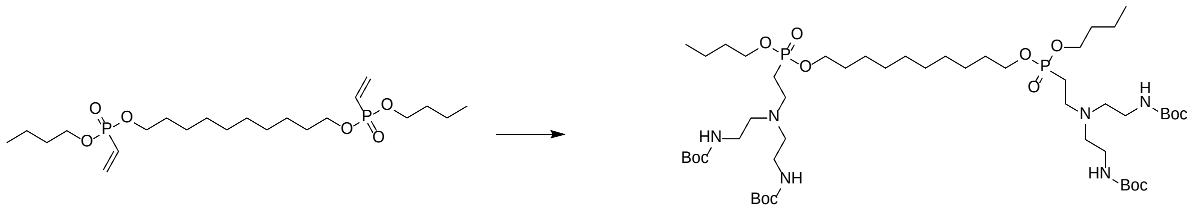

The title compound was prepared according to general method **D** from vinylphosphonate dimer **S76** (1.0 g, 2.14 mmol) and bis(2-*tert*-butyloxycarbonylaminoethyl)amine (2.60 g, 8.6 mmol) in 31% yield (0.64 g, 0.60 mmol) as a colourless oil.

^{1}H NMR (400 MHz, CD_3OD): 4.09–4.01 (m, 8H, $CH_3CH_2CH_2C\mathit{H}_2O$, $OC\mathit{H}_2CH_2(CH_2)_6CH_2C\mathit{H}_2O$), 3.11 (t, 8H, J = 6.3 Hz, $NCH_2C\mathit{H}_2NH$), 2.79 (dt, 4H, J = 11.1, 7.5 Hz, $PCH_2C\mathit{H}_2N$), 2.55 (t, 8H, J = 6.3 Hz, $NC\mathit{H}_2CH_2NH$), 2.04–1.96 (m, 4H, $PC\mathit{H}_2CH_2N$), 1.73–1.64 (m, 8H, $CH_3CH_2C\mathit{H}_2CH_2O$, $OCH_2C\mathit{H}_2(CH_2)_6C\mathit{H}_2CH_2O$), 1.49–1.34 (m, 52H, $CH_3C\mathit{H}_2CH_2CH_2O$, $OCH_2CH_2(C\mathit{H}_2)_6CH_2CH_2O$, $C(C\mathit{H}_3)_3$), 0.97 (t, 6H, J = 7.4 Hz, $C\mathit{H}_3(CH_2)_2CH_2O$).

^{13}C NMR (101 MHz, CD_3OD): 158.47 ($NH\mathit{C}OO$), 60.01 ($\mathit{C}(CH_3)_3$), 67.18, 66.90 (d, J = 6.8 Hz, $CH_3CH_2CH_2\mathit{C}H_2O$, $O\mathit{C}H_2CH_2(CH_2)_6CH_2\mathit{C}H_2O$), 54.39 ($N\mathit{C}H_2CH_2NH$), 48.45 ($PCH_2\mathit{C}H_2N$), 39.46 ($NCH_2\mathit{C}H_2NH$), 33.68, 31.6 (d, J = 6.3 Hz, $CH_3CH_2\mathit{C}H_2CH_2O$, $OCH_2\mathit{C}H_2(CH_2)_6\mathit{C}H_2CH_2O$), 28.86 ($C(\mathit{C}H_3)_3$, 30.63, 30.29, 26.73 ($OCH_2CH_2(\mathit{C}H_2)_6CH_2CH_2O$), 22.99 (d, J = 137.3 Hz, $P\mathit{C}H_2CH_2N$), 19.87 ($CH_3\mathit{C}H_2CH_2CH_2O$), 13.99 ($\mathit{C}H_3CH_2)_2CH_2O$).

^{31}P{^{1}H} NMR (162 MHz, CD_3OD): 34.51.

IR ν_{max} (KBr) 3451 (m), 3322 (w), 2894 (m), 2821 (w), 1704 (vs), 1506 (s), 1475 (m), 1393 (m), 1367 (m), 1247 (s), 1170 (s), 1063 (m), 1025 (s), 987 (m), 946 (m, sh).

HR-MS(ESI^{+}): For $C_{50}H_{102}N_6O_{14}NaP_2$ $(M+H)^{+}$ *m/z* calculated 1095.68215, found 1095.68218.

^{1}H NMR spectrum of compound **S119**

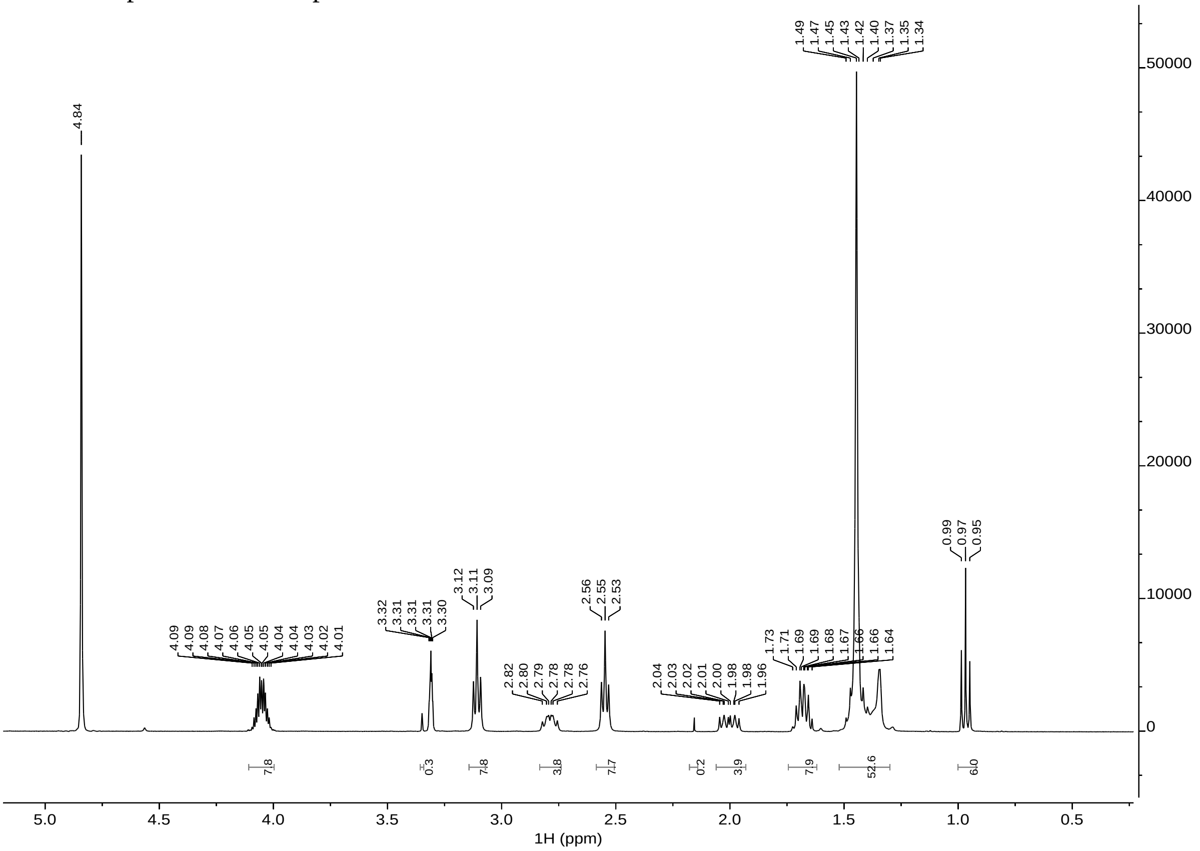

^{13}C NMR spectrum of compound **S119**

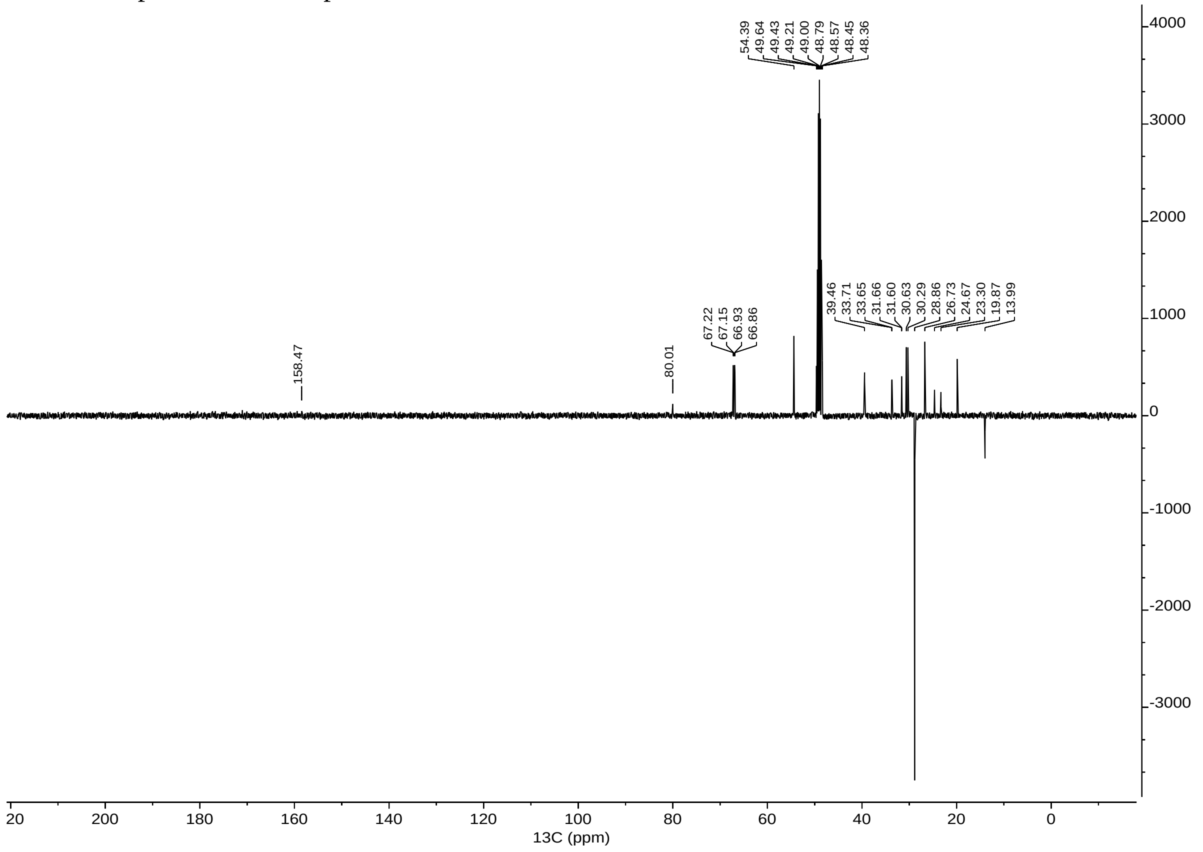

^{31}P{^{1}H} NMR spectrum of compound **S119**

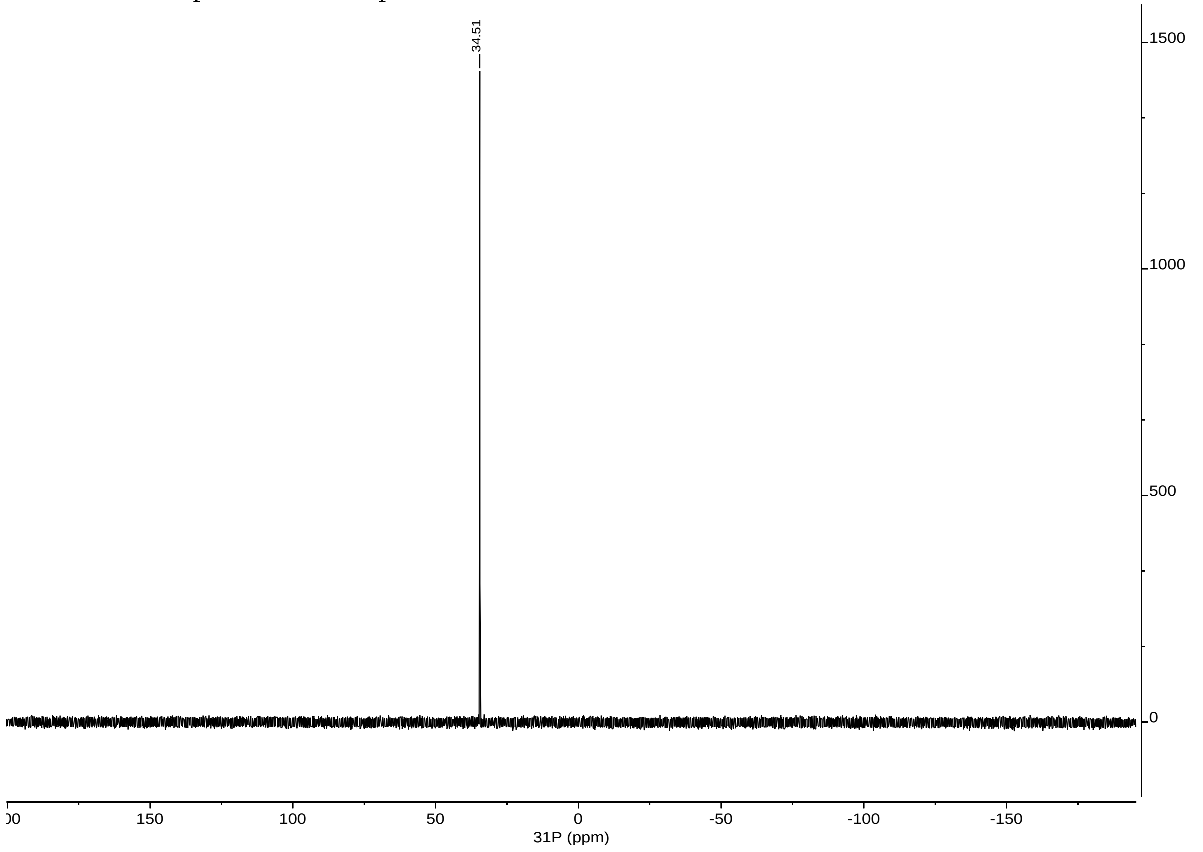

***N,N',N'',N'''*-Tetrakis(*tert*-butyloxycarbonyl) decane-1,10-diyl dipentyl bis((2-(bis(3-aminopropyl)amino)ethyl)phosphonate) (S120)**

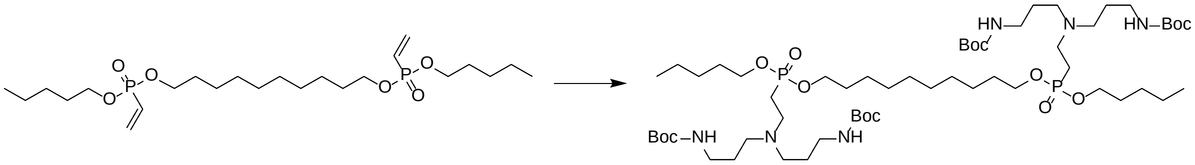

The title compound was prepared according to general method **D** from vinylphosphonate dimer **S77** (0.73 g, 1.57 mmol) and bis(2-*tert*-butyloxycarbonylaminopropyl)amine (3.08 g, 9.30 mmol) in 49% yield (1.85 g, 2.28 mmol) as a colourless oil.

^{1}H NMR (400 MHz, CD_3OD): 4.09–3.99 (m, 8H, $CH_3(CH_2)_2CH_2CH_2O$, $OCH_2CH_2(CH_2)_4CH_2CH_2O$), 3.08 (t, 8H, J = 6.7 Hz, $NCH_2CH_2CH_2NH$), 2.80–2.74 (m, 4H, PCH_2CH_2N), 2.49–2.45 (m, 8H, $NCH_2CH_2CH_2NH_2$), 2.03–1.95 (m, 4H, PCH_2CH_2N), 1.72–1.61 (m, 16H, $CH_3(CH_2)_2CH_2CH_2O$, $OCH_2CH_2(CH_2)_4CH_2CH_2O$, $NCH_2CH_2CH_2NH$), 1.44 (s, 36H, $C(CH_3)_3$), 1.41–1.34 (m, 20H, $CH_3(CH_2)_2CH_2CH_2O$, $OCH_2CH_2(CH_2)_6CH_2CH_2O$), 0.96–0.92 (m, 6H, $CH_3(CH_2)_3CH_2O$).

^{13}C NMR (101 MHz, CD_3OD): 158.47 (NHCOO), 79.85 ($C(CH_3)_3$), 67.23 (d, J = 7.0 Hz, $CH_3(CH_2)_2CH_2CH_2O$, $OCH_2CH_2(CH_2)_6CH_2CH_2O$), 51.87 ($NCH_2CH_2CH_2NH$), 47.69 ($PCH_2CH_2N$), 39.63 ($NCH_2CH_2CH_2NH$), 31.63, 31.33 (d, J = 6.3 Hz, $CH_3(CH_2)_2CH_2CH_2O$, $OCH_2CH_2(CH_2)_6CH_2CH_2O$), 28.85 ($C(CH_3)_3$, 28.16 ($NCH_2CH_2CH_2NH$), 30.65, 30.29, 28.92, 26.73, 23.31 ($CH_3(CH_2)_2CH_2CH_2O$, $OCH_2CH_2(CH_2)_6CH_2CH_2O$), 22.75 (d, J = 136.8 Hz, PCH_2CH_2N), 14.39 ($CH_3(CH_2)_3CH_2O$).

^{31}P{^{1}H} NMR (162 MHz, CD_3OD): 34.34.

IR ν_{max} (KBr) 3455 (w), 3370 (w, vbr), 2960 (m), 2933 (s), 2980 (m), 2859 (m), 2825 (w), 1707 (vs), 1505 (s), 1476 (m), 1393 (m), 1381 (w), 1367 (s), 1350 (w), 1169 (s), 1249 (s), 1075 (m, sh), 1052 (m), 1016 (m), 996 (m).

HR-MS(ESI^{+}): For $C_{56}H_{114}N_6O_{14}NaP_2$ $(M+Na)^+$ m/z calculated 1179.77605, found 1179.77624.

^{1}H NMR spectrum of compound **S120**

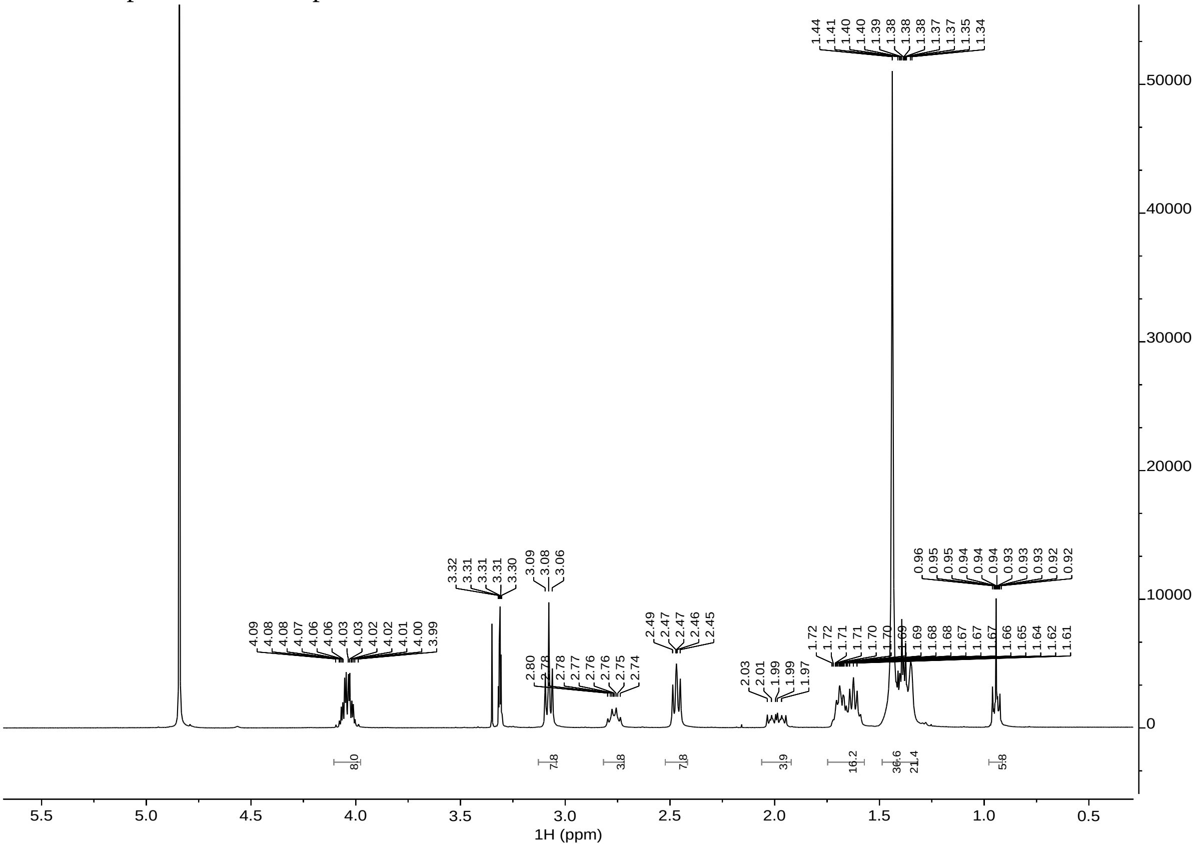

^{13}C NMR spectrum of compound **S120**

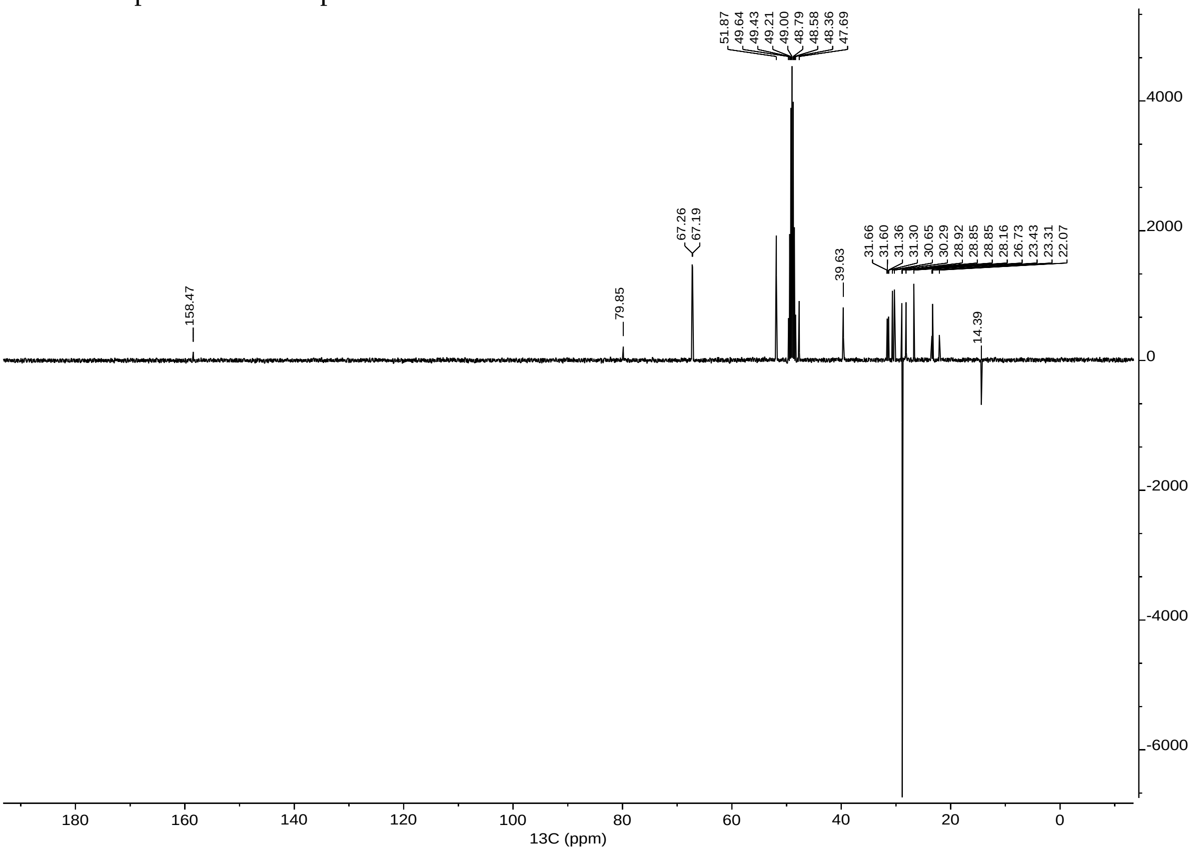

$^{31}P\{^{1}H\}$ NMR spectrum of compound **S120**

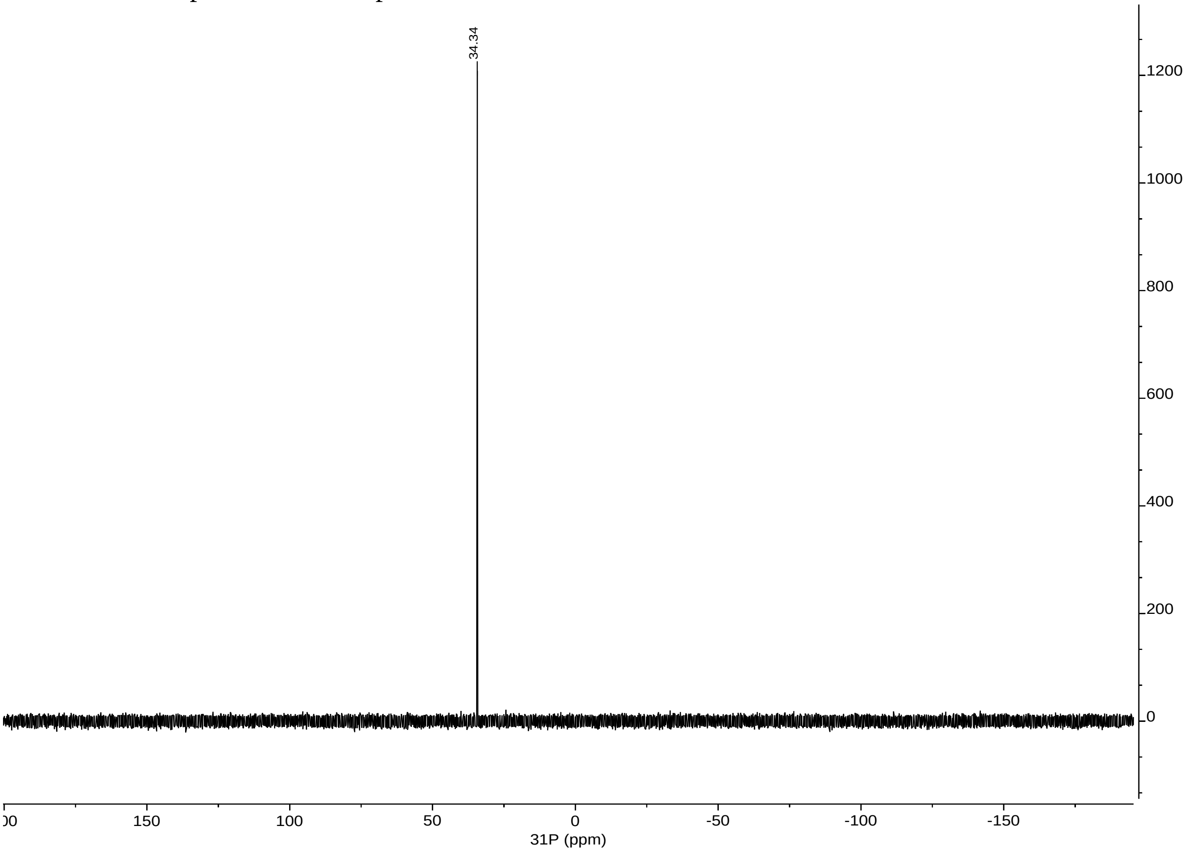

***N,N',N'',N'''*-Tetrakis(*tert*-butyloxycarbonyl) decane-1,10-diyl dihexyl bis((2-(bis(3-aminopropyl)amino)ethyl)phosphonate) (S121)**

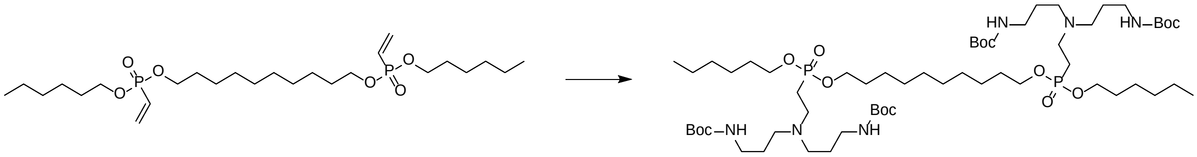

The title compound was prepared according to general method **D** from vinylphosphonate dimer **S78** (0.65 g, 1.24 mmol) and bis(3-*tert*-butyloxycarbonylaminopropyl)amine (1.65 g, 4.97 mmol) in 65% yield (0.96 g, 0.81 mmol) as a colourless oil.

^{1}H NMR (400 MHz, CD_3OD): 4.09–4.02 (m, 8H, $CH_3(CH_2)_3CH_2CH_2O$, $OCH_2CH_2(CH_2)_6CH_2CH_2O$), 3.09 (t, 8H, J = 6.7 Hz, $NCH_2CH_2CH_2NH$), 2.94–2.88 (m, 4H, PCH_2CH_2N), 2.67–2.63 (m, 8H, $NCH_2CH_2CH_2NH$), 2.16–2.01 (m, 4H, PCH_2CH_2N), 1.72–1.65 (m, 16H, $CH_3(CH_2)_3CH_2CH_2O$, $OCH_2CH_2(CH_2)_6CH_2CH_2O$, $NCH_2CH_2CH_2NH$), 1.44–1.32 (m. 60H, $CH_3(CH_2)_3CH_2CH_2O$, $OCH_2CH_2(CH_2)_6CH_2CH_2O$, $C(CH_3)_3$), 0.94–0.91 (m, 6H, $CH_3(CH_2)_4CH_2O$).

^{13}C NMR (101 MHz, CD_3OD): 158.57 (NCOO), 80.03 ($C(CH_3)_3$), 67.44 (d, J = 6.2 Hz, $CH_3(CH_2)_3CH_2CH_2O$, $OCH_2CH_2(CH_2)_6CH_2CH_2O$), 51.78 ($NCH_2CH_2CH_2NH$), 47.77 ($PCH_2CH_2N$), 39.27 ($NCH_2CH_2CH_2NH$), 32.51 ($CH_3CH_2CH_2CH_2CH_2CH_2O$), 31.61, 30.48 (d, J = 6.3 Hz, $CH_3(CH_2)_3CH_2CH_2O$, $OCH_2CH_2(CH_2)_6CH_2CH_2O$), 27.53 ($NCH_2CH_2CH_2NH$), 28.84 ($C(CH_3)_3$, 30.66, 30.30, 26.72, 26.39 ($CH_3CH_2CH_2CH_2CH_2CH_2O$, $OCH_2CH_2(CH_2)_6CH_2CH_2O$), 23.65 ($CH_3CH_2CH_2CH_2CH_2CH_2O$), 22.35 (d, J = 137.8 Hz, PCH_2CH_2N), 14.40 ($CH_3(CH_2)_4CH_2O$).

^{31}P{^{1}H} NMR (162 MHz, CD_3OD): 32.90.

IR ν_{max} (KBr) 3455 (w), 3355 (vw, br), 2980 (m), 2959 (m), 2933 (m), 2871 (w), 2859 (m), 2825 (w, sh), 1711 (s), 1507 (s), 1476 (w), 1467 (w), 1456 (m), 1393 (m), 1368 (m), 1249 (s), 1170 (s), 1060 (m, sh), 1044 (m), 1009 (m), 1000 (m), 458 (vw).

HR-MS(ESI$^+$): For $C_{58}H_{118}O_{14}N_6NaP_2$ $(M+Na)^+$ m/z calculated 1207.80735, found 1207.80755.

^{1}H NMR spectrum of compound **S121**

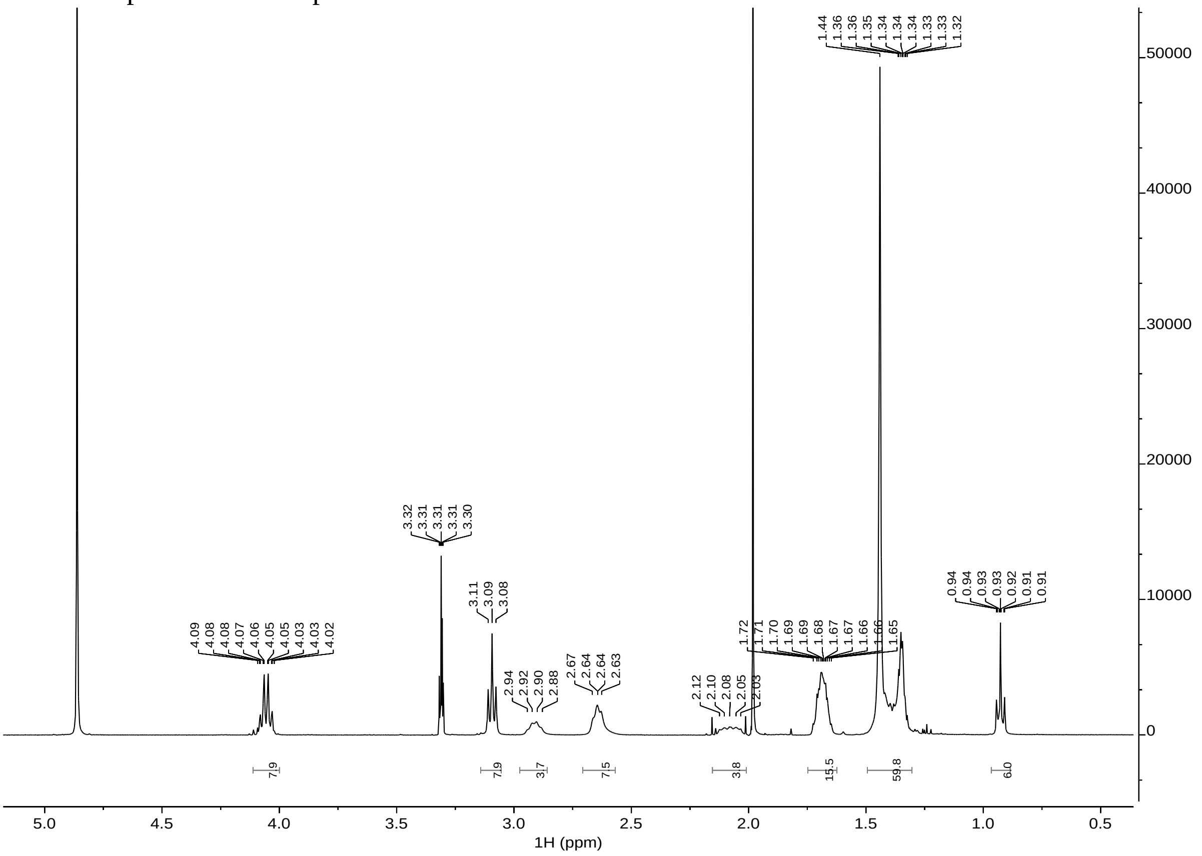

^{13}C NMR spectrum of compound **S121**

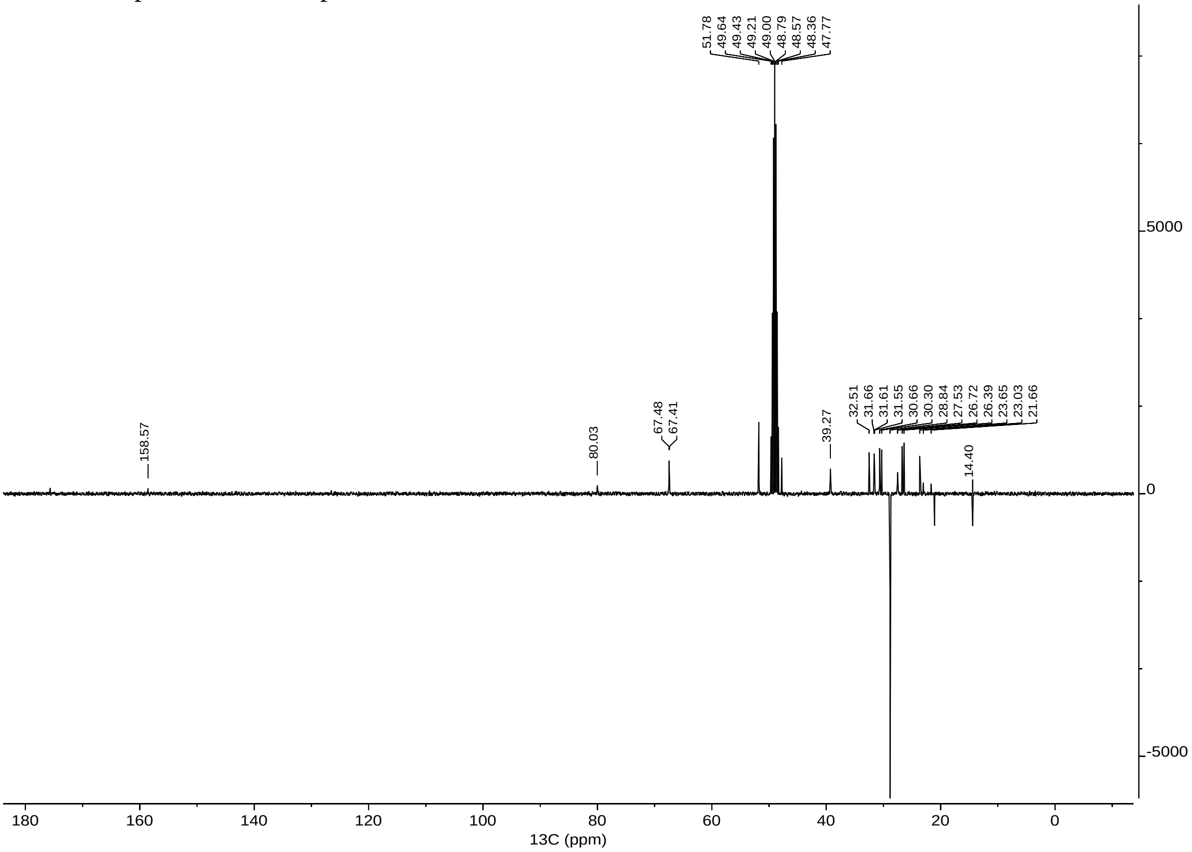

^{31}P{^{1}H} NMR spectrum of compound **S121**

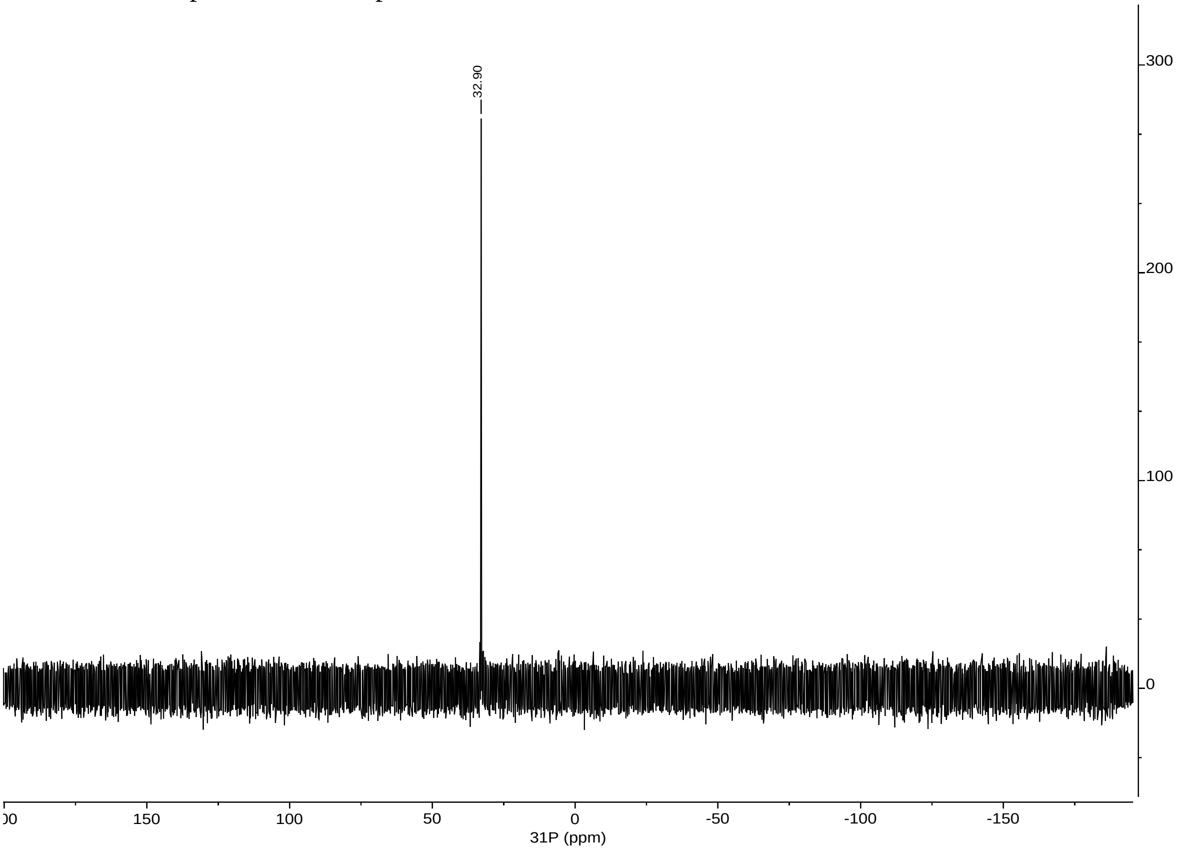

_N,N',N'',N'''_-Tetrakis(_tert_-butyloxycarbonyl) dibutyl dodecane-1,12-diyl bis((2-(bis(3-amino-propyl)amino)ethyl)phosphonate) (S122)

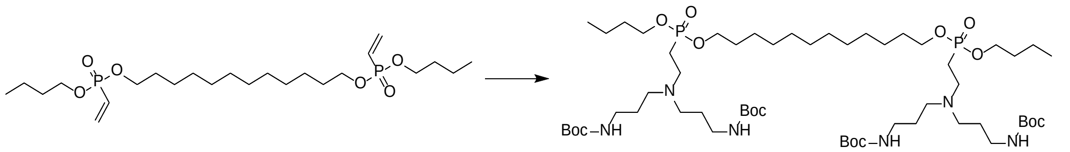

The title compound was prepared according to general method **D** from vinylphosphonate dimer **S83** (0.97 g, 1.96 mmol) and bis(3-*tert*-butyloxycarbonylaminopropyl)amine (2.60 g, 7.84 mmol) in 54% yield (1.18 g, 1.06 mmol) as a colourless oil.

^{1}H NMR (400 MHz, CD_3OD): 4.09–4.01 (m, 8H, $CH_3CH_2CH_2C\mathit{H}_2O$, $OC\mathit{H}_2CH_2(CH_2)_8CH_2C\mathit{H}_2O$), 3.08 (t, 8H, J = 6.7 Hz, $NCH_2CH_2C\mathit{H}_2NH$), 2.81–2.75 (m, 4H, $PCH_2C\mathit{H}_2N$), 2.50–2.47 (m, 8H, $NC\mathit{H}_2CH_2CH_2NH$), 2.04–1.95 (m, 4H, $PC\mathit{H}_2CH_2N$), 1.72–1.61 (m, 16H, $CH_3CH_2C\mathit{H}_2CH_2O$, $OCH_2C\mathit{H}_2(CH_2)_8C\mathit{H}_2CH_2O$, $NCH_2C\mathit{H}_2CH_2NH$), 1.47–1.33 (m, 56H, $CH_3C\mathit{H}_2CH_2CH_2O$, $OCH_2CH_2(C\mathit{H}_2)_8CH_2CH_2O$, $C(C\mathit{H}_3)_3$), 0.97 (t, 6H, J = 7.4 Hz, $C\mathit{H}_3(CH_2)_2CH_2O$).

^{13}C NMR (101 MHz, CD_3OD): 158.49 ($NH\mathit{C}OO$), 79.87 ($\mathit{C}(CH_3)_3$), 67.25–66.95 (d, J = 7.2 Hz, $CH_3CH_2CH_2\mathit{C}H_2O$, $O\mathit{C}H_2CH_2(CH_2)_8CH_2\mathit{C}H_2O$), 51.85 ($N\mathit{C}H_2CH_2CH_2NH$), 47.69 ($PCH_2\mathit{C}H_2N$), 39.58 ($NCH_2CH_2\mathit{C}H_2NH$), 31.66, 31.60 (d, J = 6.3 Hz, $CH_3CH_2\mathit{C}H_2CH_2O$, $OCH_2\mathit{C}H_2(CH_2)_8\mathit{C}H_2CH_2O$), 28.85 ($C(\mathit{C}H_3)_3$, 28.08 ($NCH_2\mathit{C}H_2CH_2NH$), 30.71, 30.28, 26.71 ($OCH_2CH_2(\mathit{C}H_2)_8CH_2CH_2O$), 22.69 (d, J = 136.8 Hz, $P\mathit{C}H_2CH_2N$), 19.86 ($CH_3\mathit{C}H_2CH_2CH_2O$), 13.98 ($\mathit{C}H_3CH_2)_2CH_2O$).

^{31}P{^{1}H} NMR (162 MHz, CD_3OD): 34.19.

IR ν_{max} (KBr) 3455 (m), 3370 (w, vbr), 2980 (s), 2966 (s), 2933 (s), 2857 (m), 2824 (w), 1707 (vs), 1507 (vs), 1477 (m), 1466 (m), 1457 (m), 1393 (s), 1381 (w, sh), 1367 (vs), 1350 (w), 1249 (s), 1169 (vs), 1064 (m), 1048 (m, sh), 1027 (s), 1006 (m, sh), 989 (m), 462 (vw).

HR-MS(ESI^{+}): For $C_{56}H_{115}N_6O_{14}P_2$ $(M+H)^{+}$ m/z calculated 1157.79410, found 1157.79443.

^{1}H NMR spectrum of compound **S122**

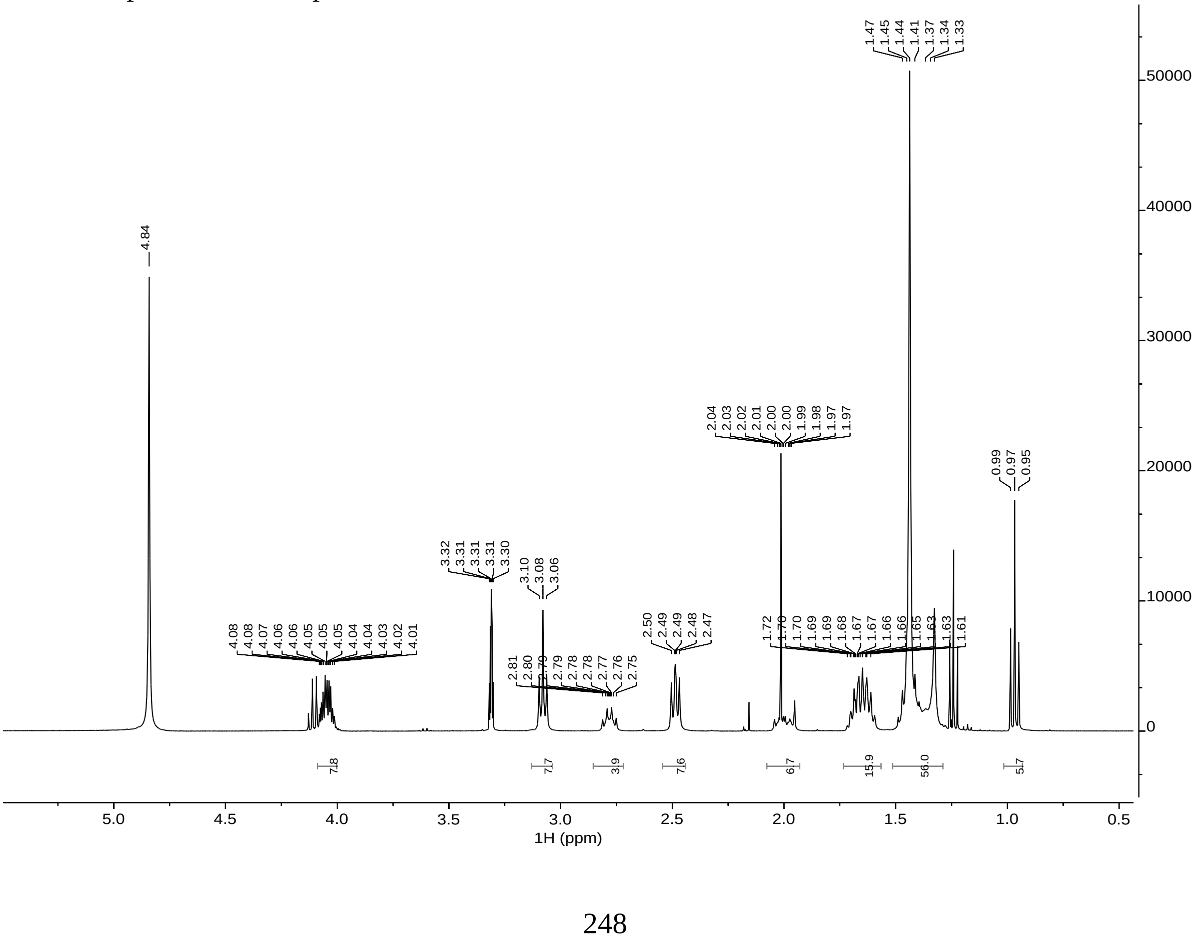

^{13}C NMR spectrum of compound **S122**

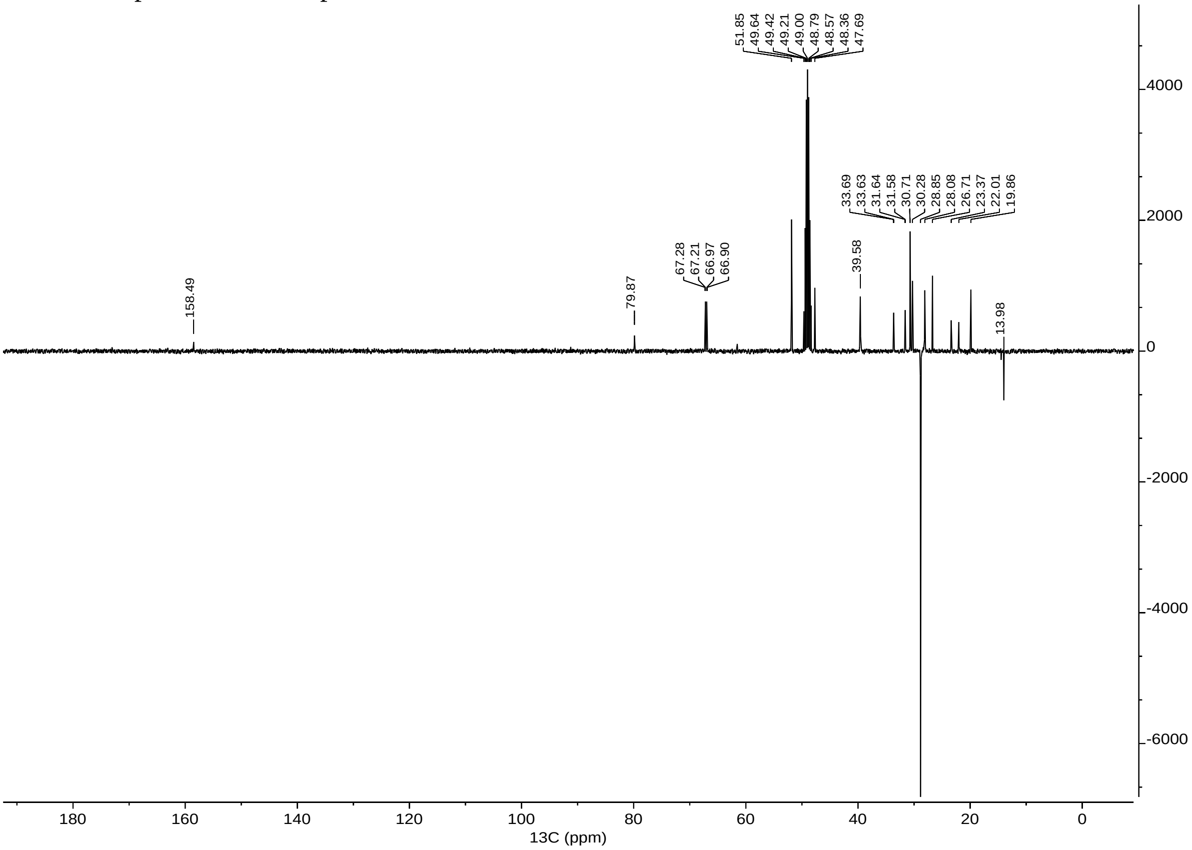

$^{31}P\{^{1}H\}$ NMR spectrum of compound **S122**

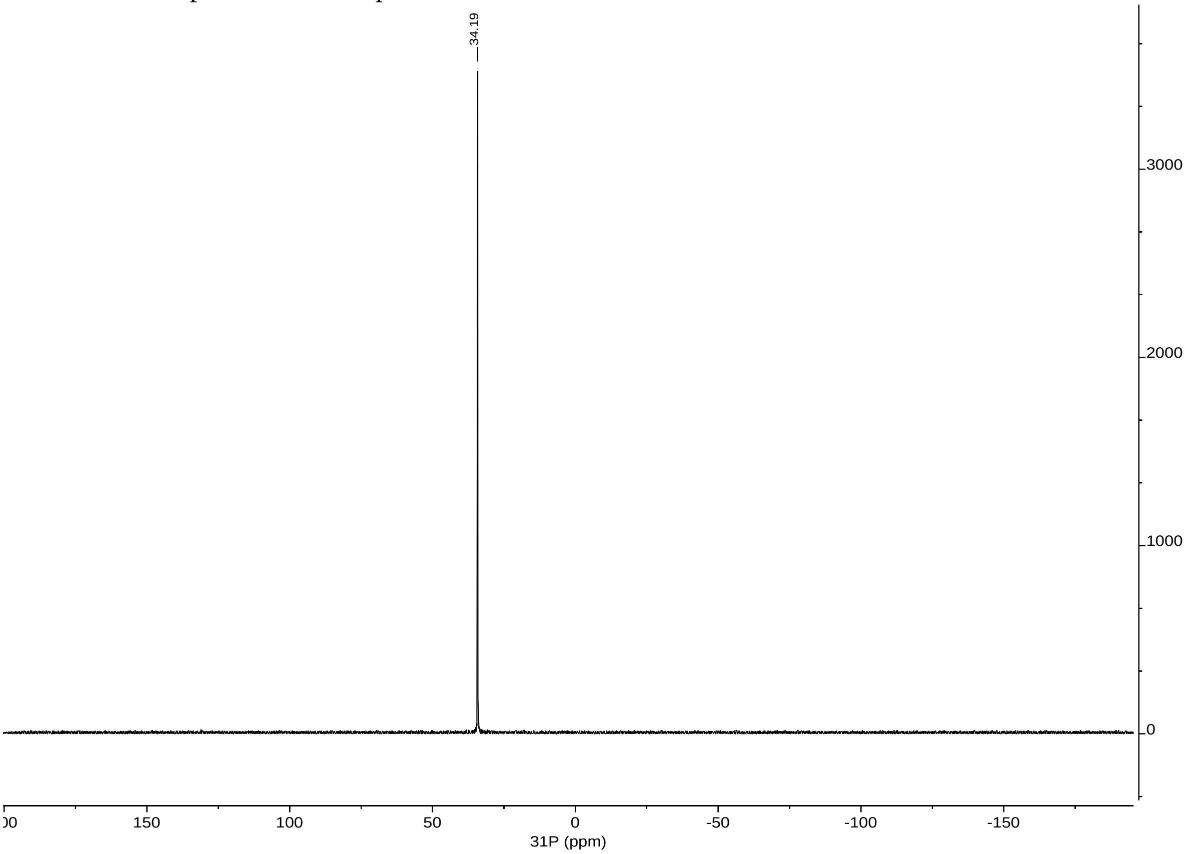

***N,N',N'',N'''*-Tetrakis(*tert*-butyloxycarbonyl) dodecane-1,12-diyl dipentyl bis((2-(bis(3-amino-propyl)amino)ethyl)phosphonate) (S123)**

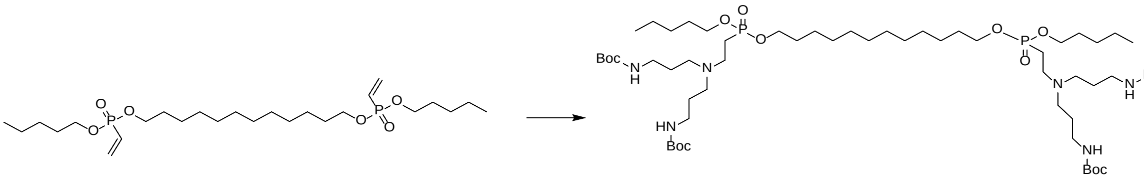

The title compound was prepared according to general method **D** from vinylphosphonate dimer **S84** (1.10 g, 2.10 mmol) and bis(3-*tert*-butyloxycarbonylaminopropyl)amine (2.79 g, 8.42 mmol) in 64% yield (1.59 g, 1.34 mmol) as a colourless oil.

^{1}H NMR (400 MHz, CD_3OD): 4.08–4.01 (m, 8H, $CH_3(CH_2)_2CH_2CH_2O$, $OCH_2CH_2(CH_2)_8CH_2CH_2O$), 3.08 (t, 8H, *J* = 6.7 Hz, $NCH_2CH_2CH_2NH$), 2.83–2.77 (m, 4H, PCH_2CH_2N), 2.53–2.49 (m, 8H, $NCH_2CH_2CH_2NH$), 2.06–1.96 (m, 4H, PCH_2CH_2N), 1.72–1.60 (m, 16H, $CH_3(CH_2)_2CH_2CH_2O$, $OCH_2CH_2(CH_2)_4CH_2CH_2O$, $NCH_2CH_2CH_2NH$), 1.44 (s, 36H, $C(CH_3)_3$), 1.41–1.33 (m, 24H, $CH_3(CH_2)_2CH_2CH_2O$, $OCH_2CH_2(CH_2)_8CH_2CH_2O$), 0.96–0.92 (m, 6H, $CH_3(CH_2)_3CH_2O$).

^{13}C NMR (101 MHz, CD_3OD): 158.64 (NHCOO), 79.89 ($C(CH_3)_3$), 67.27 (d, *J* = 6.8 Hz, $CH_3(CH_2)_2CH_2CH_2O$, $OCH_2CH_2(CH_2)_6CH_2CH_2O$), 51.85 ($NCH_2CH_2CH_2NH$), 47.71 ($PCH_2CH_2N$), 39.53 ($NCH_2CH_2CH_2NH$), 31.62, 31.32 (d, *J* = 6.3 Hz, $CH_3(CH_2)_2CH_2CH_2O$, $OCH_2CH_2(CH_2)_8CH_2CH_2O$), 28.85 ($C(CH_3)_3$, 28.0 ($NCH_2CH_2CH_2NH$), 30.72, 30.30, 28.91, 26.73, 23.33 ($CH_3(CH_2)_2CH_2CH_2O$, $OCH_2CH_2(CH_2)_8CH_2CH_2O$), 22.66 (d, *J* = 136.8 Hz, PCH_2CH_2N), 14.39 ($CH_3(CH_2)_3CH_2O$).

^{31}P{^{1}H} NMR (162 MHz, CD_3OD): 33.98.

IR ν_{max} (KBr) 3455 (w), 3367 (w, vbr), 2980 (m), 2961 (m), 2932 (s), 2858 (m), 2826 (w), 1708 (vs), 1507 (s), 1476 (w), 1467 (m), 1457 (m), 1393 (m), 1368 (s), 1249 (s), 1169 (s), 1051 (m), 1015 (m), 997 (m), 466 (w).

HR-MS(ESI^+): For $C_{58}H_{118}N_6O_{14}NaP_2$ $(M+Na)^+$ *m/z* calculated 1207.80735, found 1207.80748.

^{1}H NMR spectrum of compound **S123**

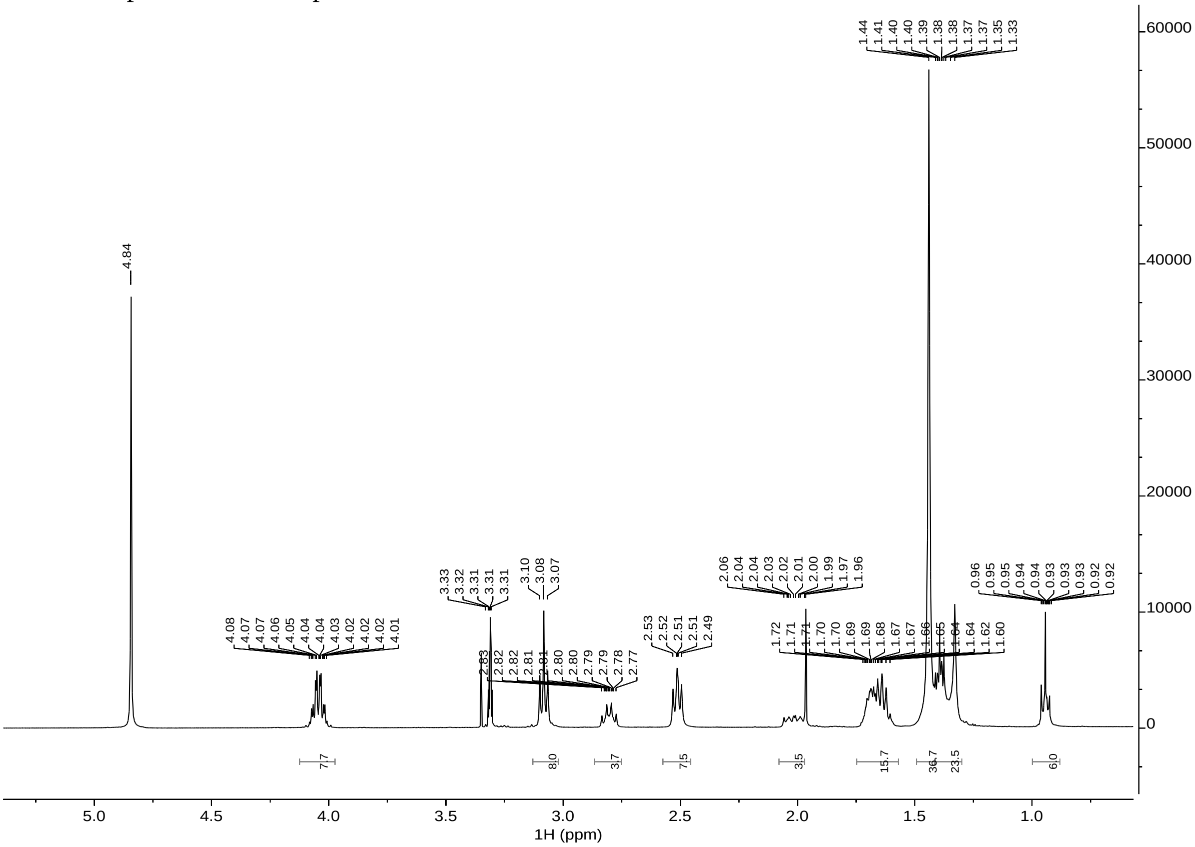

^{13}C NMR spectrum of compound **S123**

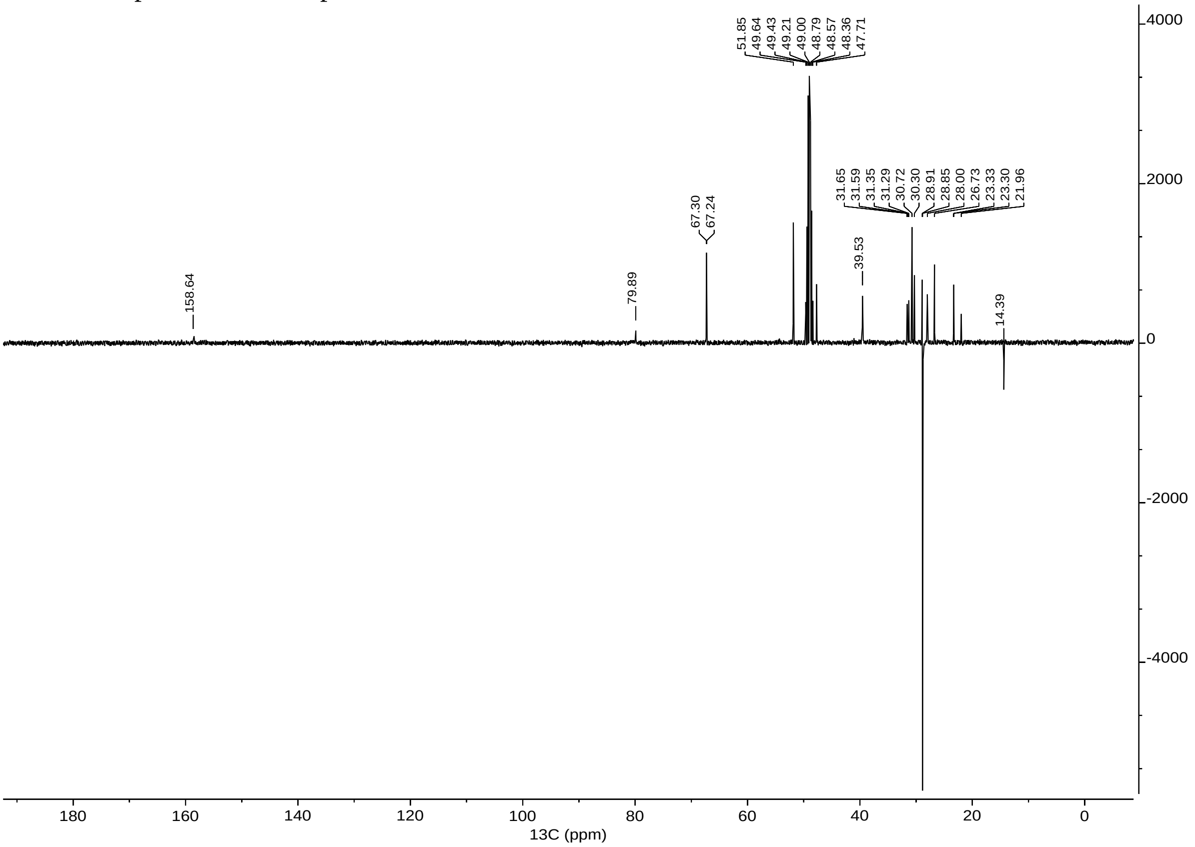

^{31}P{^{1}H} NMR spectrum of compound **S123**

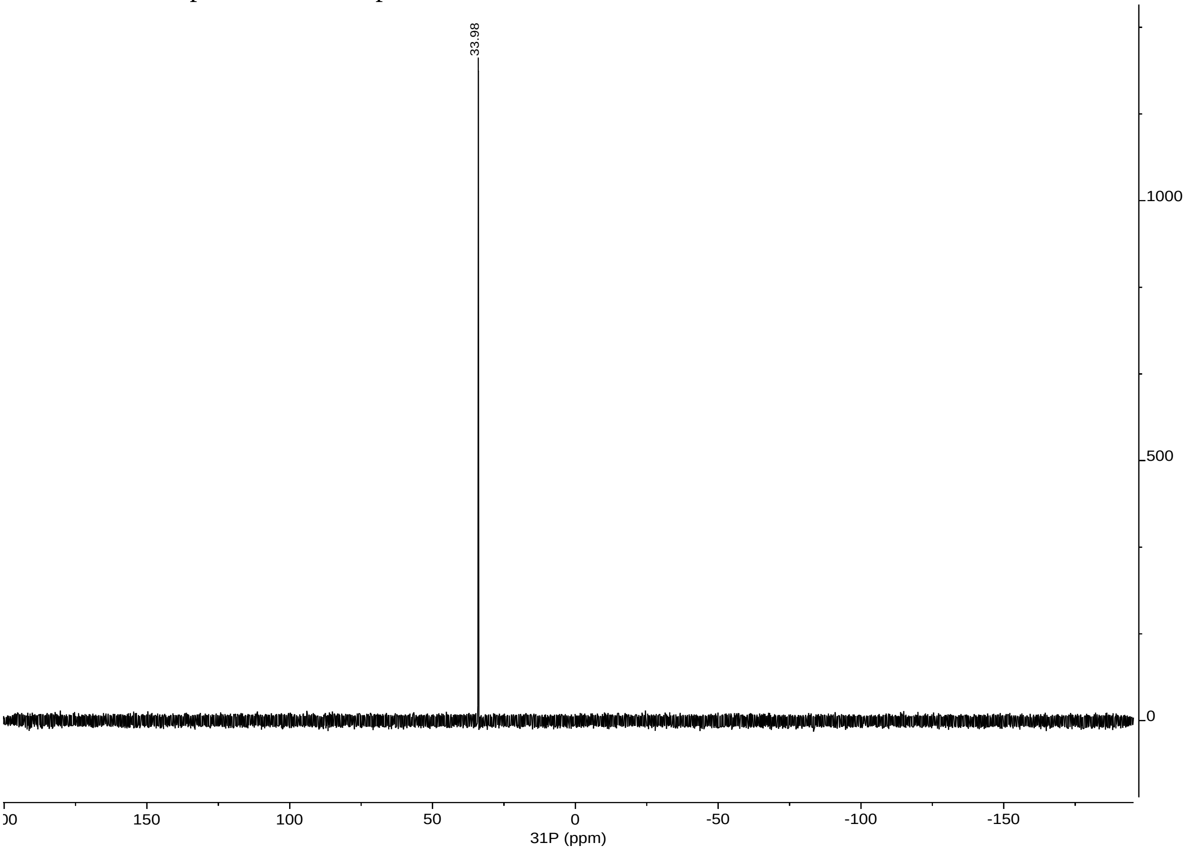

***N,N',N'',N'''*-Tetrakis(*tert*-butyloxycarbonyl) dibutyl tetradecane-1,14-diyl bis((2-(bis(3-amino-propyl)amino)ethyl)phosphonate) (S124)**

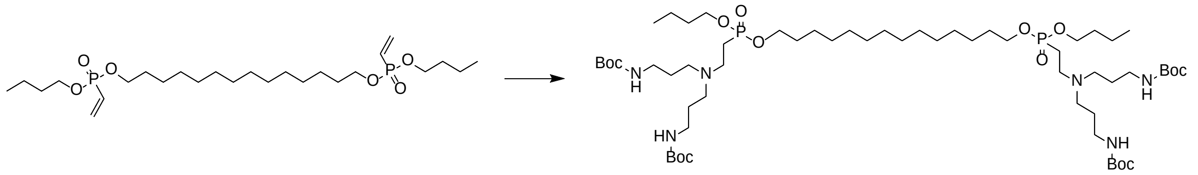

The title compound was prepared according to general method **D** from vinylphosphonate dimer **S88** (1.20 g, 2.30 mmol) and bis(3-*tert*-butyloxycarbonylaminopropyl)amine (3.04 g, 9.18 mmol) in 63% yield (1.72 g, 1.45 mmol) as a colourless oil.

^{1}H NMR (400 MHz, CD_3OD): 4.11–4.02 (m, 8H, $CH_3CH_2CH_2CH_2O$, $OCH_2CH_2(CH_2)_{10}CH_2CH_2O$), 3.08 (t, 8H, J = 6.7 Hz, $NCH_2CH_2CH_2NH$), 2.86–2.80 (m, 4H, PCH_2CH_2N), 2.56–2.52 (m, 8H, $NCH_2CH_2CH_2NH$), 2.07–2.00 (m, 4H, PCH_2CH_2N), 1.72–1.61 (m, 16H, $CH_3CH_2CH_2CH_2O$, $OCH_2CH_2(CH_2)_{10}CH_2CH_2O$, $NCH_2CH_2CH_2NH$), 1.47–1.31 (m, 60H, $CH_3CH_2CH_2CH_2O$, $OCH_2CH_2(CH_2)_{10}CH_2CH_2O$, $C(CH_3)_3$), 0.99–0.90 (m, 6H, $CH_3(CH_2)_2CH_2O$).

^{13}C NMR (101 MHz, CD_3OD): 151.51 (NHCOO), 79.94 ($C(CH_3)_3$), 67.33, 67.02 (d, J = 7.2 Hz, $CH_3CH_2CH_2CH_2O$, $OCH_2CH_2(CH_2)_{10}CH_2CH_2O$), 51.83 ($NCH_2CH_2CH_2NH$), 47.72 ($PCH_2CH_2N$), 39.46 ($NCH_2CH_2CH_2NH$), 31.66, 31.60 (d, J = 6.3 Hz, $CH_3CH_2CH_2CH_2O$, $OCH_2CH_2(CH_2)_{10}CH_2CH_2O$), 27.44 ($C(CH_3)_3$, 26.48 ($NCH_2CH_2CH_2NH$), 30.80, 30.73, 30.70, 30.27, 26.71 ($OCH_2CH_2(CH_2)_{10}CH_2CH_2O$), 22.57 (d, J = 137.8 Hz, PCH_2CH_2N), 19.86 ($CH_3CH_2CH_2CH_2O$), 13.98 ($CH_3CH_2)_2CH_2O$).

^{31}P{^{1}H} NMR (162 MHz, CD_3OD): 33.72.

IR ν_{max} (KBr) 3455 (w), 3373 (w), 2982 (s), 2967 (s), 2932 (s), 2878 (m, sh), 2821 (m), 2754 (w), 1708 (vs), 1507 (s), 1393 (m), 1368 (m), 1249 (s), 1169 (vs), 1067 (m), 988 (m).

HR-MS(ESI^+): For $C_{58}H_{118}N_6O_{14}NaP_2$ $(M+Na)^+$ *m/z* calculated 1207.80735, found 1207.80679.

^{1}H NMR spectrum of compound **S124**

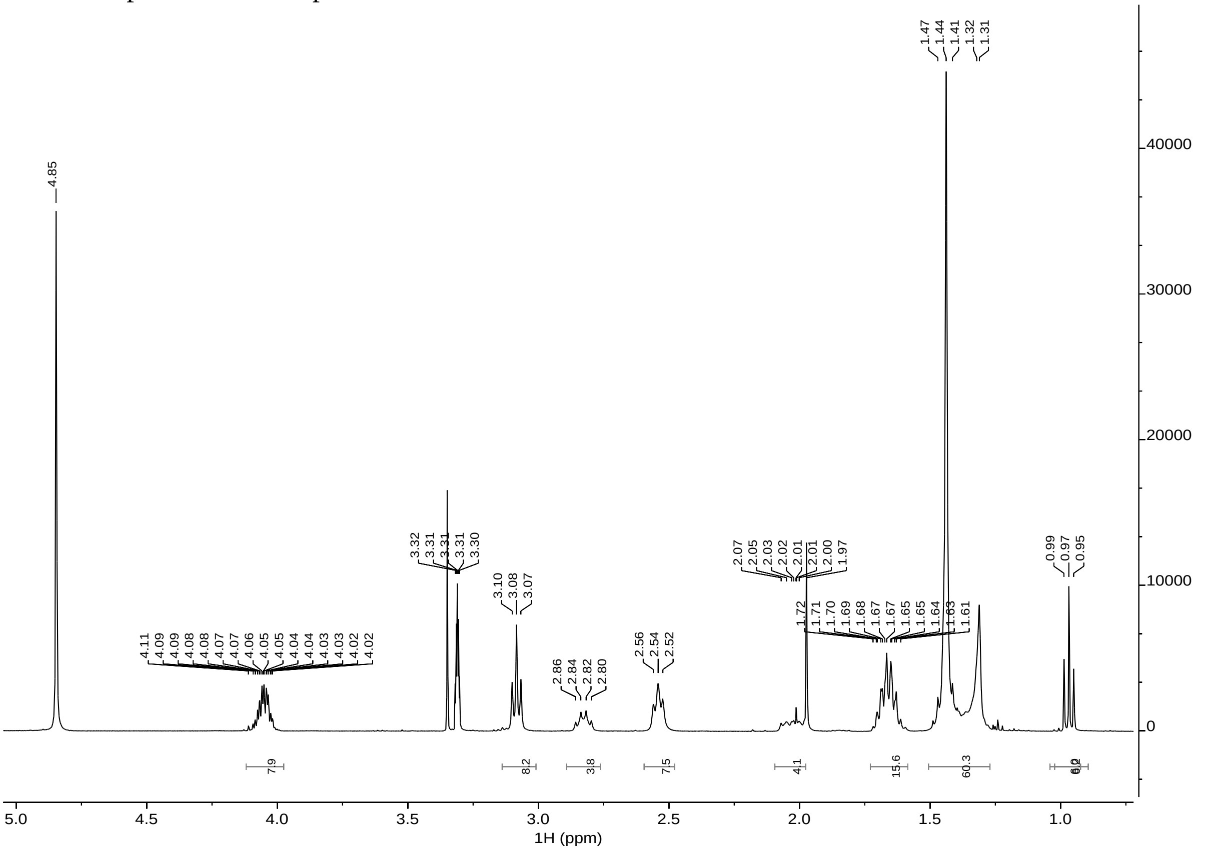

^{13}C NMR spectrum of compound **S124**

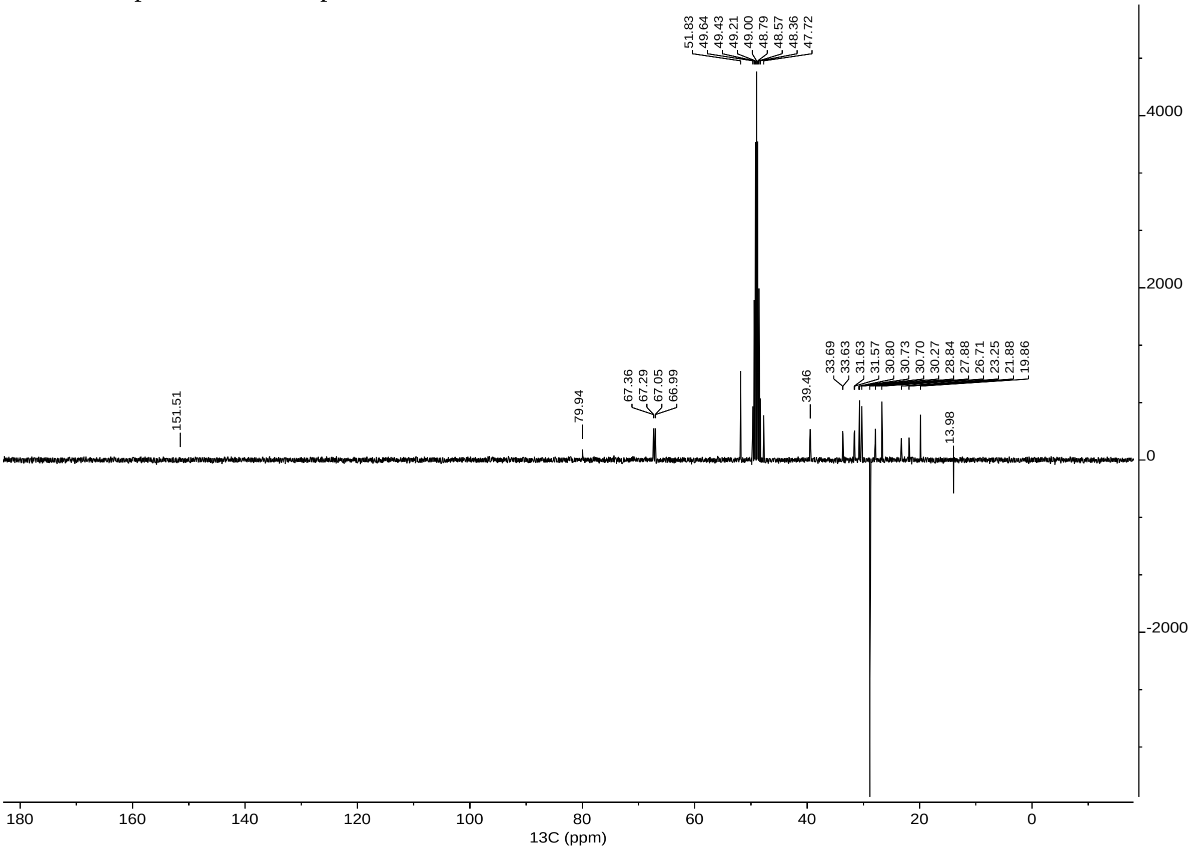

^{31}P{^{1}H} NMR spectrum of compound **S124**

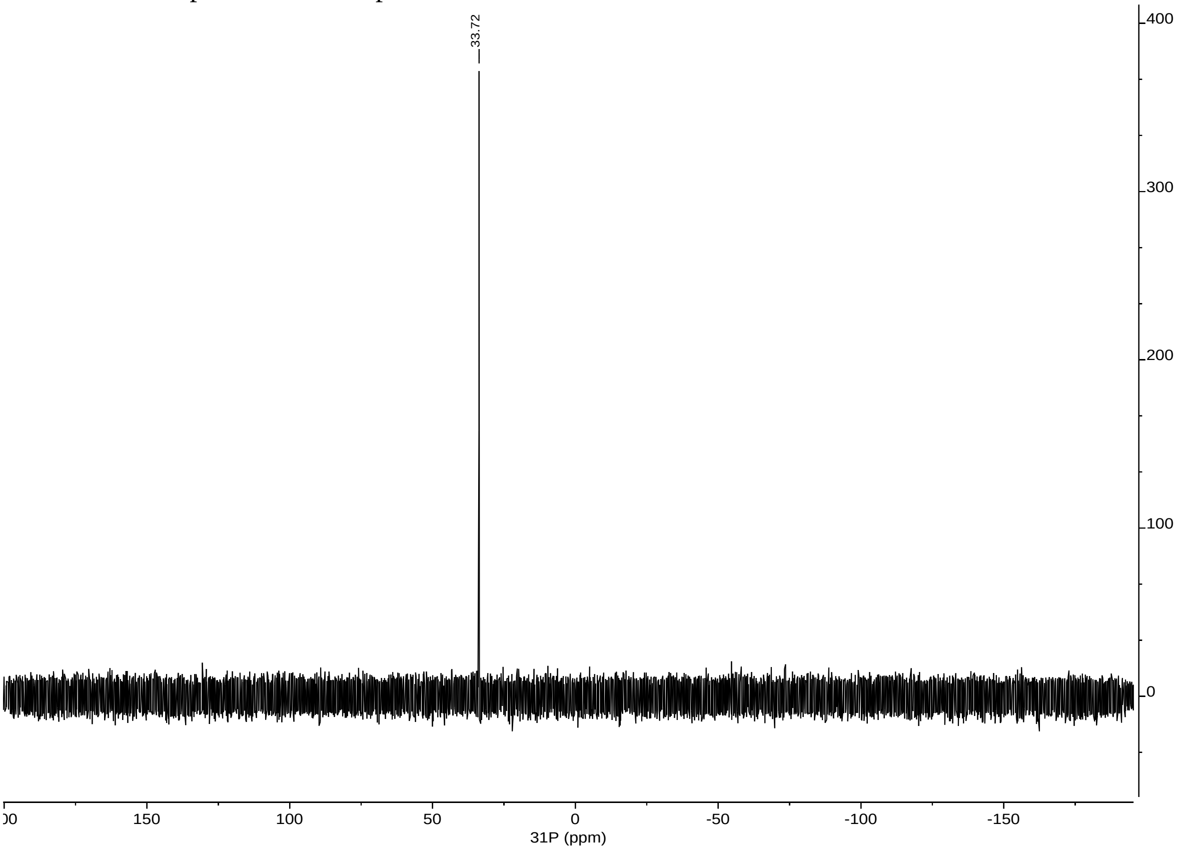

_N,N',N'',N'''_-Tetrakis(_tert_-butyloxycarbonyl) dipentyl tetradecane-1,14-diyl bis((2-(bis(3-aminopropyl)amino)ethyl)phosphonate) (S125)

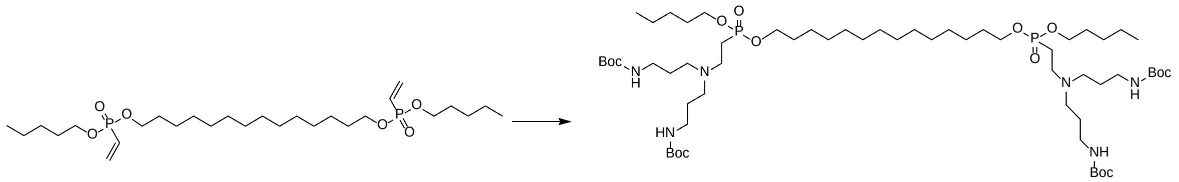

The title compound was prepared according to general method **D** from vinylphosphonate dimer **S89** (0.80 g, 1.45 mmol) and bis(3-_tert_-butyloxycarbonylaminopropyl)amine (1.76 g, 5.81 mmol) in 61% yield (1.12 g, 0.90 mmol) as a colourless oil.

^{1}H NMR (400 MHz, CD_3OD): 4.10–3.99 (m, 8H, CH_2O), 3.08 (td, J = 6.7, 1.3 Hz, 8H, CH_2NH), 2.89–2.77 (m, 4H, PCH_2CH_2), 2.54 (t, J = 7.4 Hz, 8H, $CH_2(CH_2)_2NH$), 2.08–1.98 (m, 4H, PCH_2), 1.73–1.58 (m, 16H, CH_2CH_2O, CH_2CH_2NH), 1.49–1.56 (m. 64H, $CH_3(CH_2)_2$, $OCH_2CH_2(CH_2)_{10}CH_2CH_2O$, $C(CH_3)_3$), 0.91–0.96 (m, 6H, CH_3CH_2).

^{13}C NMR (101 MHz, CD_3OD): 158.51 (NCOO), 79.92 ($C(CH_3)_3$), 67.31 (d, J = 6.8 Hz, $CH_3(CH_2)_2CH_2CH_2O$, $OCH_2CH_2(CH_2)_{10}CH_2CH_2O$), 51.84 ($NCH_2CH_2CH_2NH$), 47.73 ($PCH_2CH_2N$), 39.48 ($NCH_2CH_2CH_2NH$), 31.61, 31.33 (d, J = 6.3 Hz, $CH_3(CH_2)_2CH_2CH_2O$, $OCH_2CH_2(CH_2)_{10}CH_2CH_2O$), 28.85 ($C(CH_3)_3$, 27.91 ($NCH_2CH_2CH_2NH$), 30.80, 30.74, 30.71, 30.28, 28.91, 26.72, 23.31 ($CH_3(CH_2)_2CH_2CH_2O$, $OCH_2CH_2(CH_2)_{10}CH_2CH_2O$), 22.60 (d, J = 137.8 Hz, PCH_2CH_2N), 14.38 ($CH_3(CH_2)_3CH_2O$).

^{31}P{^{1}H} NMR (162 MHz, CD_3OD): 33.76.

IR ν_{max} (KBr) 3331 (s, br), 2962 (vs), 2929 (vs), 2857 (s), 2817 (m), 1713 (vs), 1524 (s, br), 1478 (m, sh), 1391 (s), 1365 (s), 1251 (vs), 1175 (vs), 1051 (s), 995 (s), 461 (w).

HR-MS(ESI$^+$): For $C_{60}H_{122}N_6O_{14}NaP_2$ (M+Na)$^+$ m/z calculated 1235.83865, found 1235.83921.

^{1}H NMR spectrum of compound **S125**

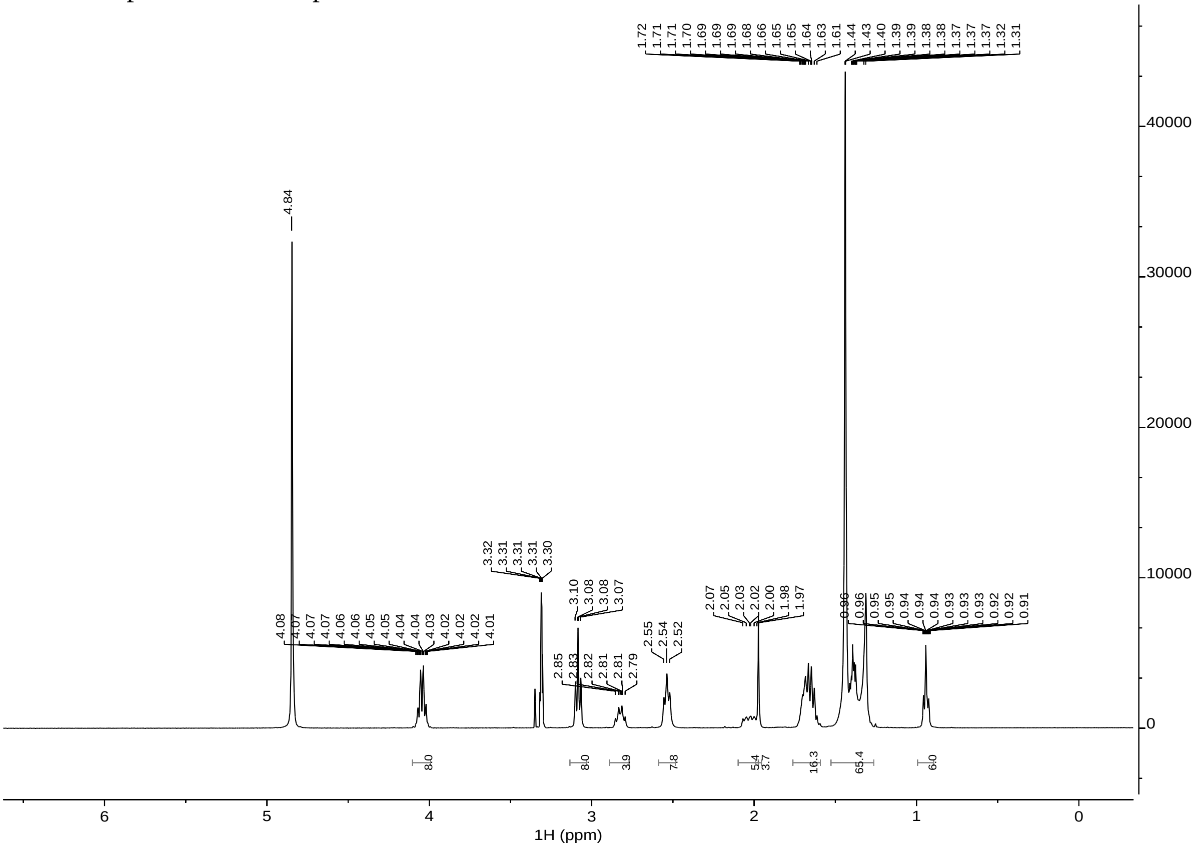

^{13}C NMR spectrum of compound **S125**

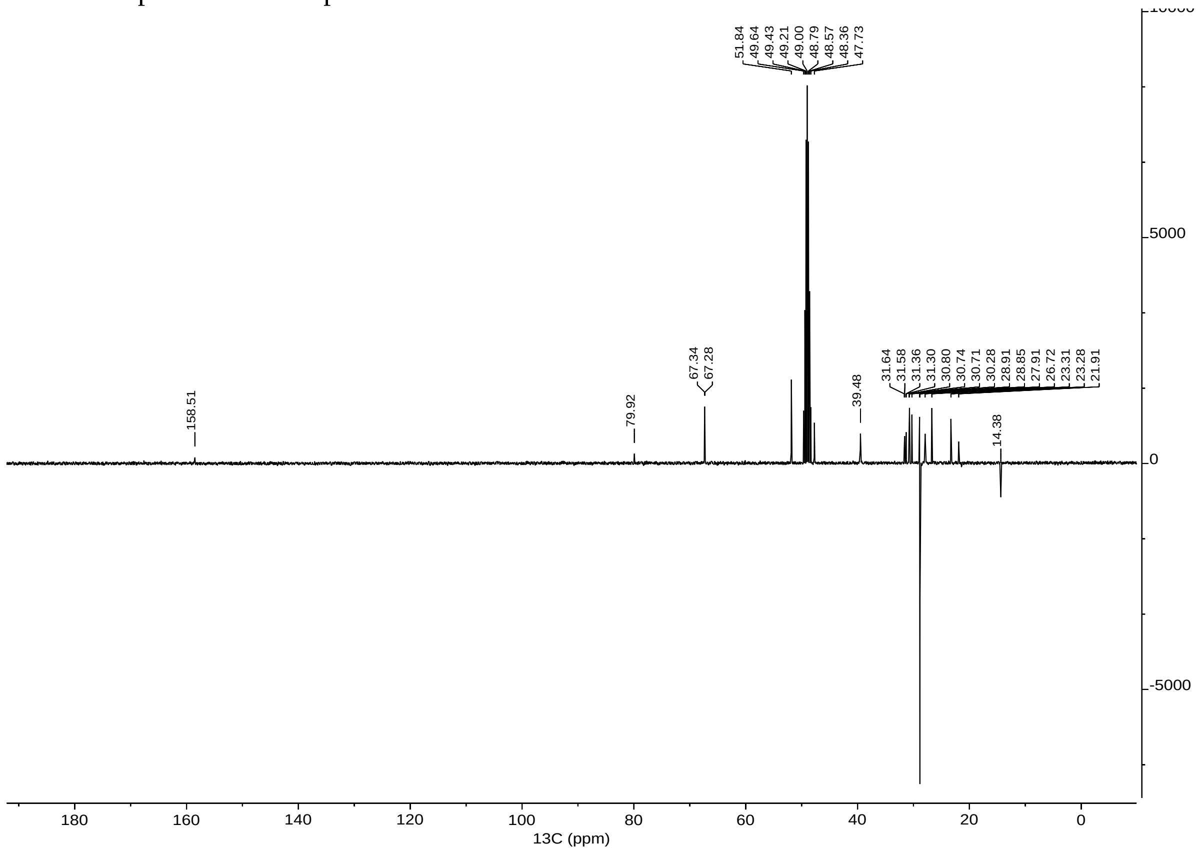

^{31}P{^{1}H} NMR spectrum of compound **S125**

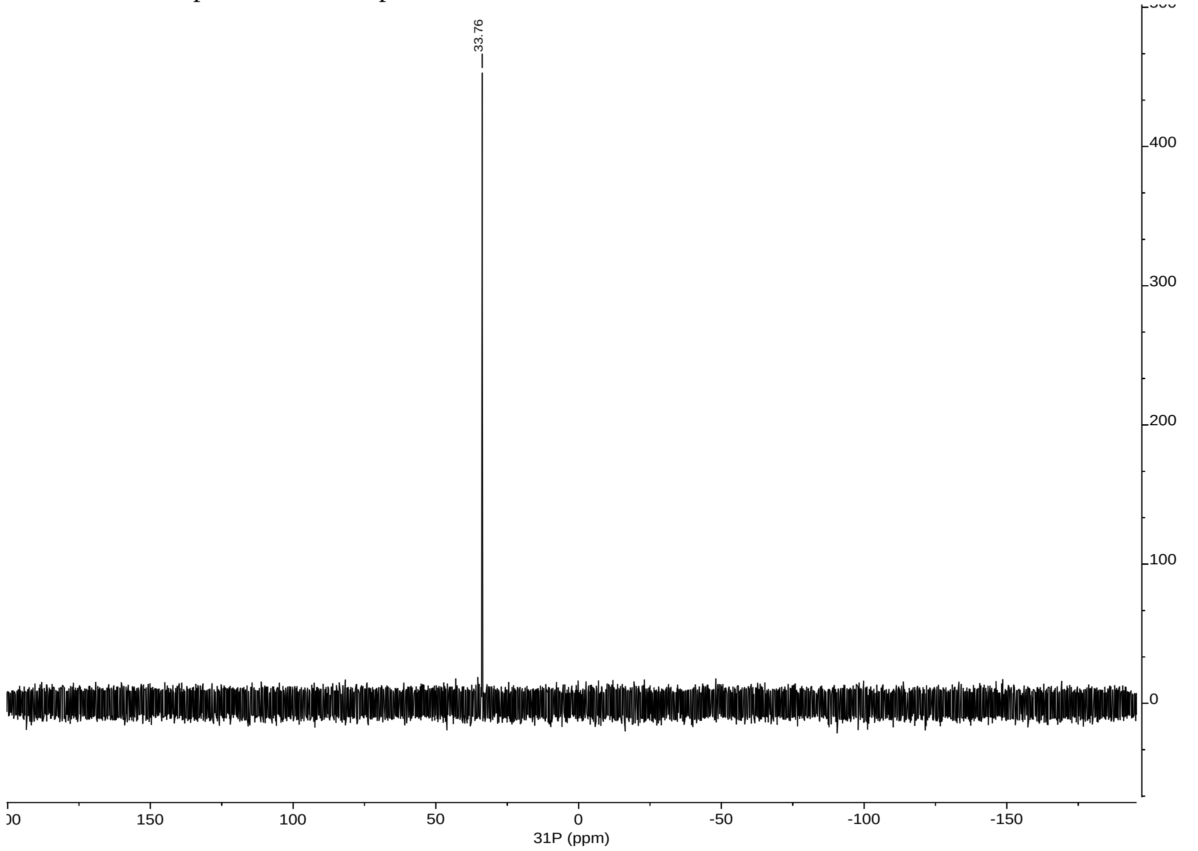

N,N',N'',N'''-Tetrakis(*tert*-butyloxycarbonyl) dihexyl tetradecane-1,14-diyl bis((2-(bis(3-amino-propyl)amino)ethyl)phosphonate) (S126)

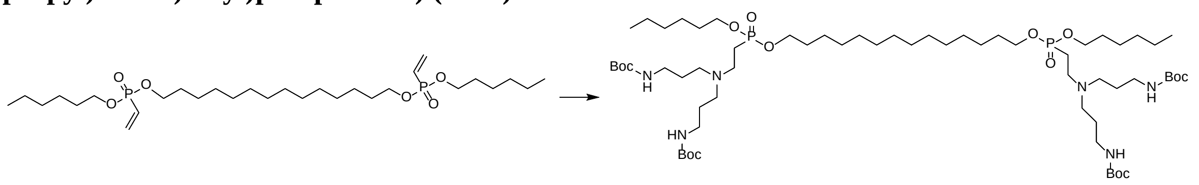

The title compound was prepared according to general method **D** from vinylphosphonate dimer **S90** (0.85 g, 1.47 mmol) and bis(3-*tert*-butyloxycarbonylaminopropyl)amine (1.95 g, 5.88 mmol) in 61% yield (1.12 g, 0.90 mmol) as a colourless oil.

^{1}H NMR (400 MHz, CD_3OD): 4.09–4.02 (m, 8H, $CH_3(CH_2)_3CH_2CH_2O$, $OCH_2CH_2(CH_2)_{10}CH_2CH_2O$), 3.09 (t, 8H, J = 6.7 Hz, $NCH_2CH_2CH_2NH$), 2.88–2.82 (m, 4H, PCH_2CH_2N), 2.59–2.52 (m, 8H, $NCH_2CH_2CH_2NH$), 2.08–2.01 (m, 4H, PCH_2CH_2N), 1.72–1.62 (m, 16H, $CH_3(CH_2)_3CH_2CH_2O$, $OCH_2CH_2(CH_2)_{10}CH_2CH_2O$, $NCH_2CH_2CH_2NH$), 1.44–1.31 (m. 68H, $CH_3(CH_2)_3CH_2CH_2O$, $OCH_2CH_2(CH_2)_{10}CH_2CH_2O$, $C(CH_3)_3$), 0.94–0.91 (m, 6H, $CH_3(CH_2)_4CH_2O$).

^{13}C NMR (101 MHz, CD_3OD): 158.54 (NCOO), 79.97 ($C(CH_3)_3$), 67.37 (d, J = 7.1 Hz, $CH_3(CH_2)_3CH_2CH_2O$, $OCH_2CH_2(CH_2)_{10}CH_2CH_2O$), 51.82 ($NCH_2CH_2CH_2NH$), 47.74 ($PCH_2CH_2N$), 39.40 ($NCH_2CH_2CH_2NH$), 32.51 ($CH_3CH_2CH_2CH_2CH_2CH_2O$), 31.63, 31.57 (d, J = 7.4 Hz, $CH_3(CH_2)_3CH_2CH_2O$, $OCH_2CH_2(CH_2)_{10}CH_2CH_2O$), 28.84 ($C(CH_3)_3$), 26.36 ($NCH_2CH_2CH_2NH$), 30.81, 30.75, 30.72, 30.29, 26.72, 26.40 ($CH_3CH_2CH_2CH_2CH_2CH_2O$, $OCH_2CH_2(CH_2)_{10}CH_2CH_2O$), 23.65 ($CH_3CH_2CH_2CH_2CH_2CH_2O$), 22.51 (d, J = 137.8 Hz, PCH_2CH_2N), 14.40 ($CH_3(CH_2)_4CH_2O$).

^{31}P{^{1}H} NMR (162 MHz, CD_3OD): 33.52.

IR ν_{max} (KBr) 3456 (w), 3366 (w, vbr), 2980 (m), 2958 (m), 2931 (s), 2857 (m), 2824 (w, sh), 1709 (vs), 1507 (s), 1475 (w), 1467 (w), 1457 (m), 1393 (m), 1368 (s), 1249 (s), 1169 (s), 1058 (m, sh), 1046 (m), 1007 (m), 1000 (m), 939 (vw, sh), 463 (vw).

HR-MS(ESI^{+}): For $C_{62}H_{126}O_{14}N_6NaP_2$ $(M+Na)^{+}$ m/z calculated 1263.86995, found 1263.87056.

^{1}H NMR spectrum of compound **S126**

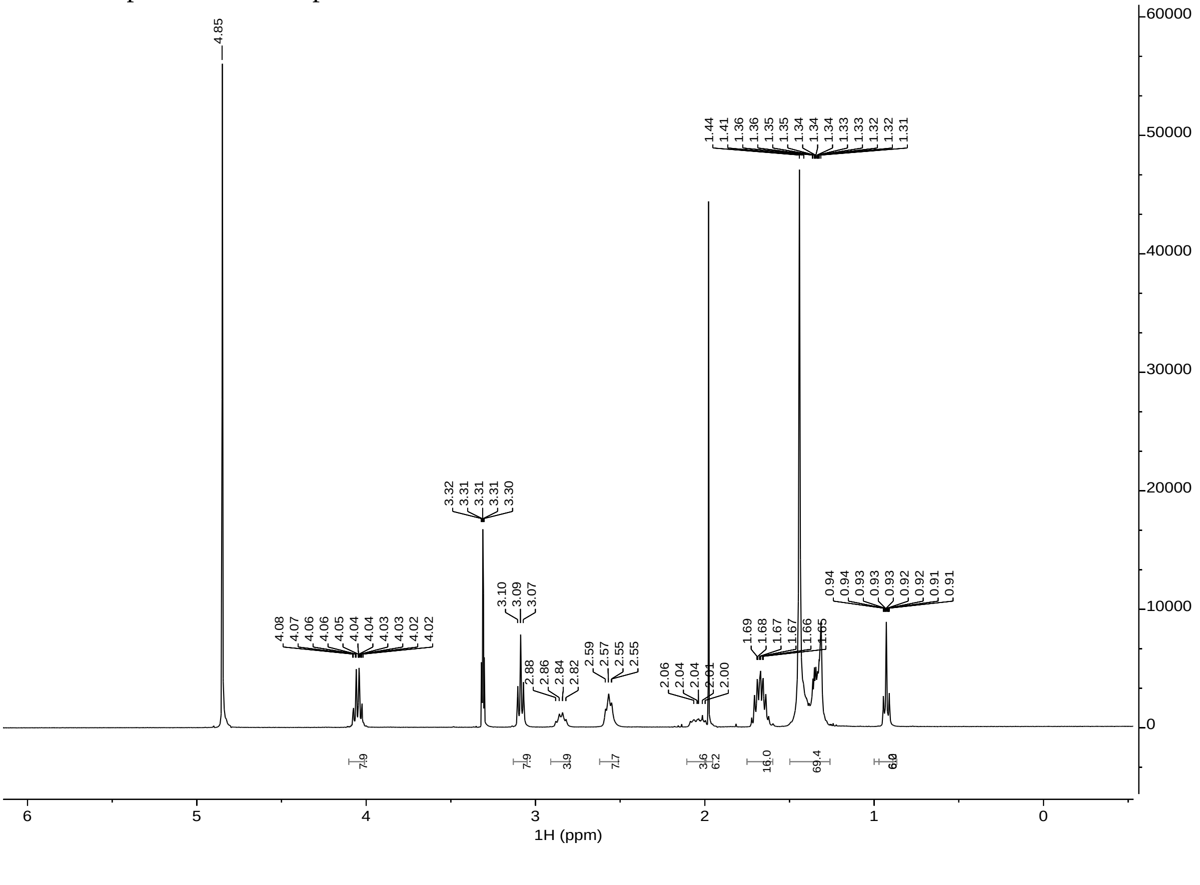

^{13}C NMR spectrum of compound **S126**

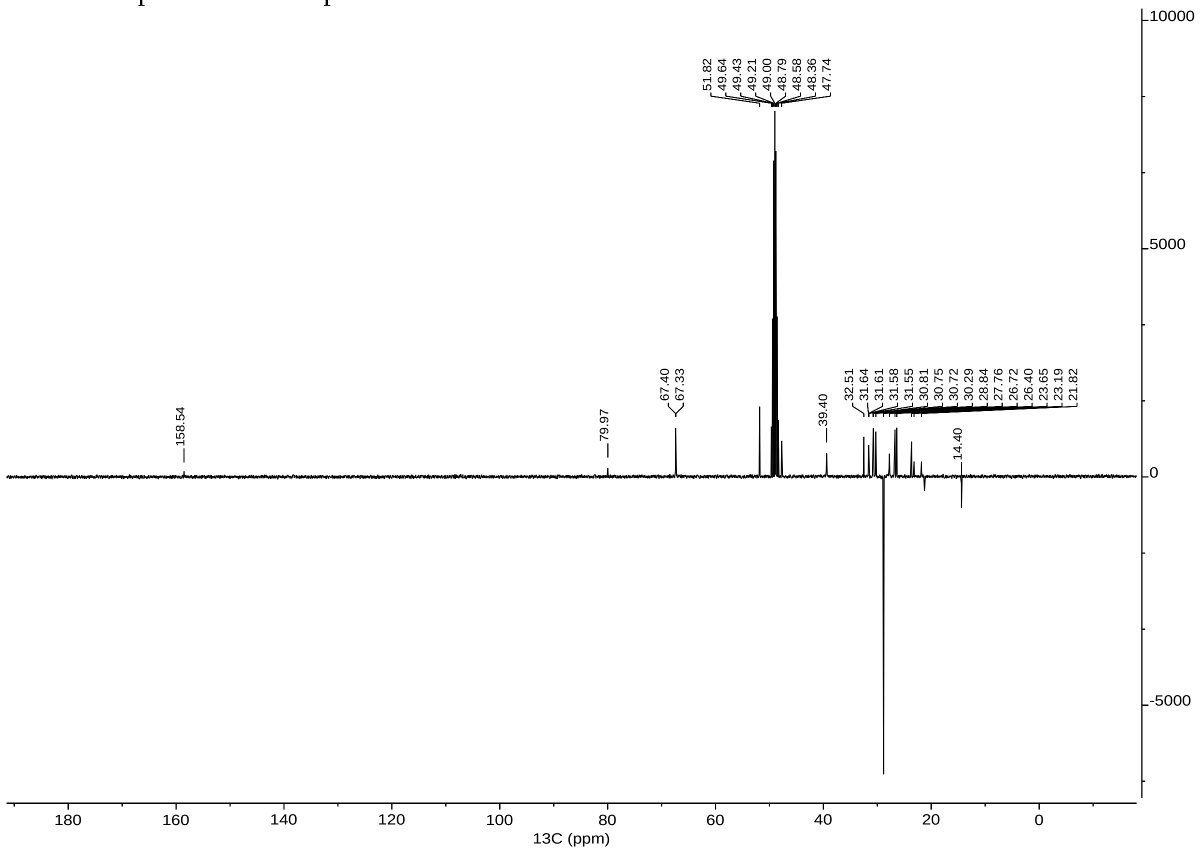

$^{31}P\{^{1}H\}$ NMR spectrum of compound **S126**

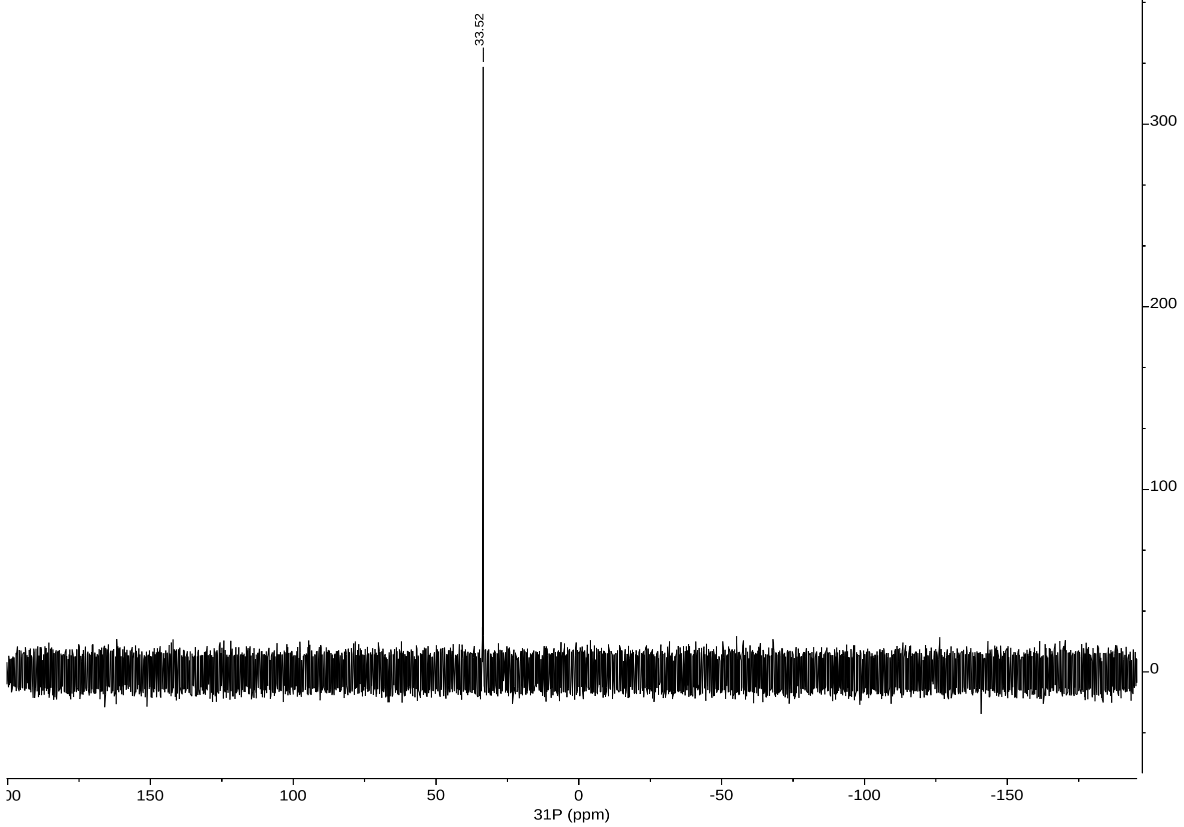

Ethyl tetradecyl (2-(bis(3-aminopropyl)amino)ethyl)phosphonate trihydrochloride (10a)

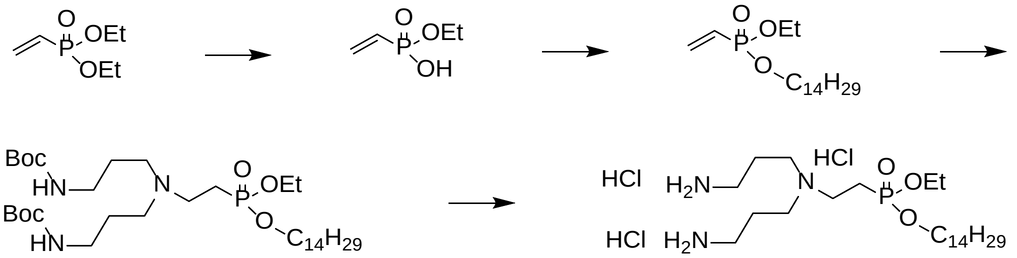

The title compound was prepared from diethyl vinylphosphonate (1.33 g, 8.1 mmol), according to general methods **A2**, **B2**, **D** and **E** in 40% overall yield (1.74 g, 3.24 mmol) as a white amorphous solid.

^{1}H NMR (500.0 MHz, CD_3OD): 4.26–4.09 (m, 4H, CH_2O), 3.50–3.38 (m, 6H, NCH_2), 3.12 (t, 4H, J = 7.4 Hz, CH_2NH$_2$), 2.61–2.51 (m, 2H, PCH_2), 2.27–2.18 (m, 4H, CH_2CH$_2$NH$_2$), 1.75–1.68 (m, 2H, CH$_3$(CH$_2$)$_{11}$CH_2), 1.45–1.22 (m, 25H, CH$_3$(CH_2)$_{11}$, CH_3CH$_2$O), 0.90 (m, 3H, CH_3(CH$_2$)$_{13}$).

^{13}C NMR (125.7 MHz, CD_3OD): 68.18 (d, J = 6.7 Hz, CH$_3$(CH$_2$)$_{12}$$CH_2$O), 64.32 (d, J = 6.5 Hz, CH$_3$$CH_2$O), 51.00 ($CH_2$(CH$_2$)$_2NH_2$), 48.93 ($CH_2CH_2$P), 37.89 ($CH_2NH_2$), 33.05 (CH$_3$($CH_2$)$_{11}$), 31.56 (d, J = 6.0 Hz, CH$_3$(CH$_2$)$_{11}$$CH_2CH_2$O), 30.77, 30.76, 30.74, 30.73, 30.70, 30.65, 30.45, 30.28 26.58, 23.71 (CH$_3$(CH$_2$)$_{11}$), 23.17 (CH$_2$CH$_2$NH$_2$), 21.38 (d, J = 140.2 Hz, PCH$_2$), 16.79 (d, J = 5.9 Hz, CH$_3$CH$_2$O), 14.45 (CH$_3$(CH$_2$)$_{13}$).

^{31}P{^{1}H} NMR (202.4 MHz, CD_3OD): 27.17.

IR ν_{max} (KBr) 3100–2500 (vs–s), 2960 (vs), 2922 (vs), 2852 (vs), 2750–2546 (s), 2025 (w, br), 1605 (m), 1593 (m), 1509 (m), 1484 (m), 1446 (s), 1227 (s), 1164 (m), 1095 (w), 1052 (s, sh), 1037 (s), 1025 (s), 1005 (s), 967 (m, sh), 808 (w).

HR-MS(ESI$^+$): For $C_{24}H_{55}N_3O_3P$ [M+H]$^+$ calculated 464.39756, found 464.39783.

^{1}H NMR spectrum of compound **10a**

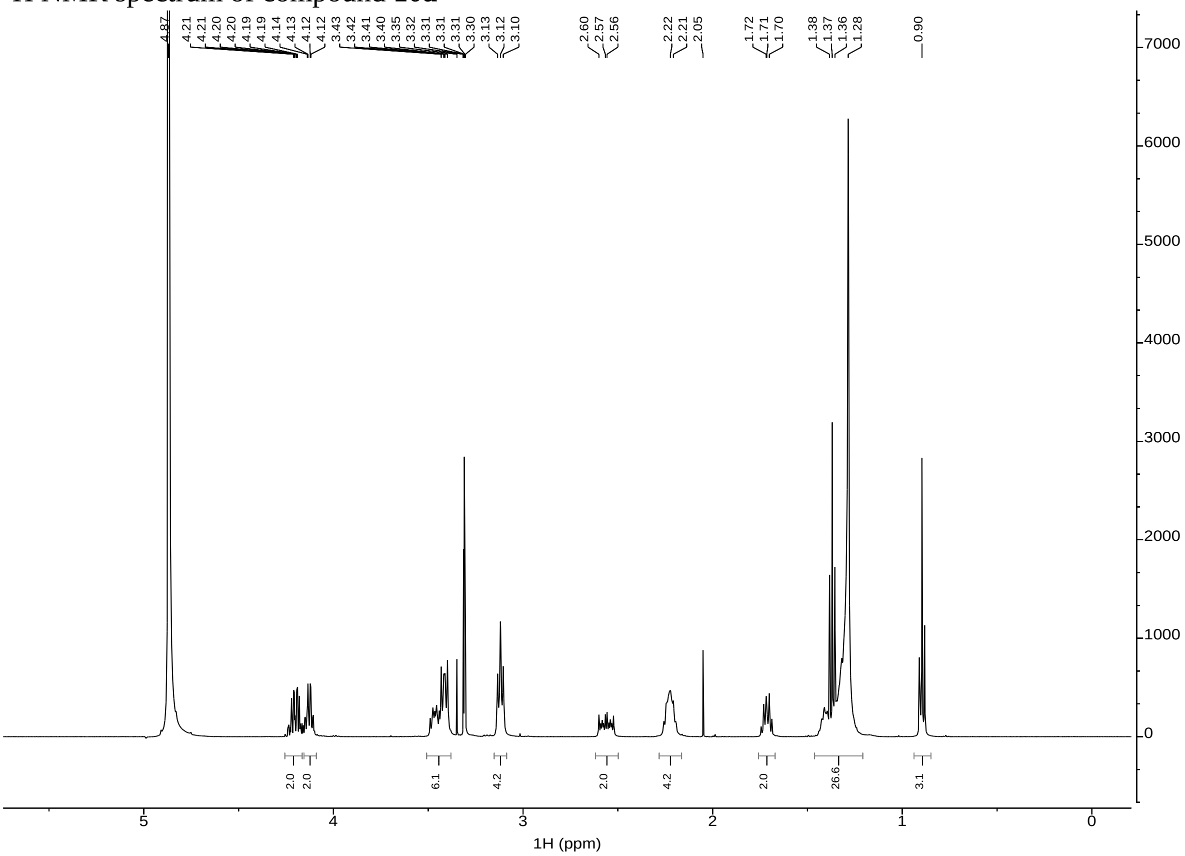

^{13}C NMR spectrum of compound **10a**

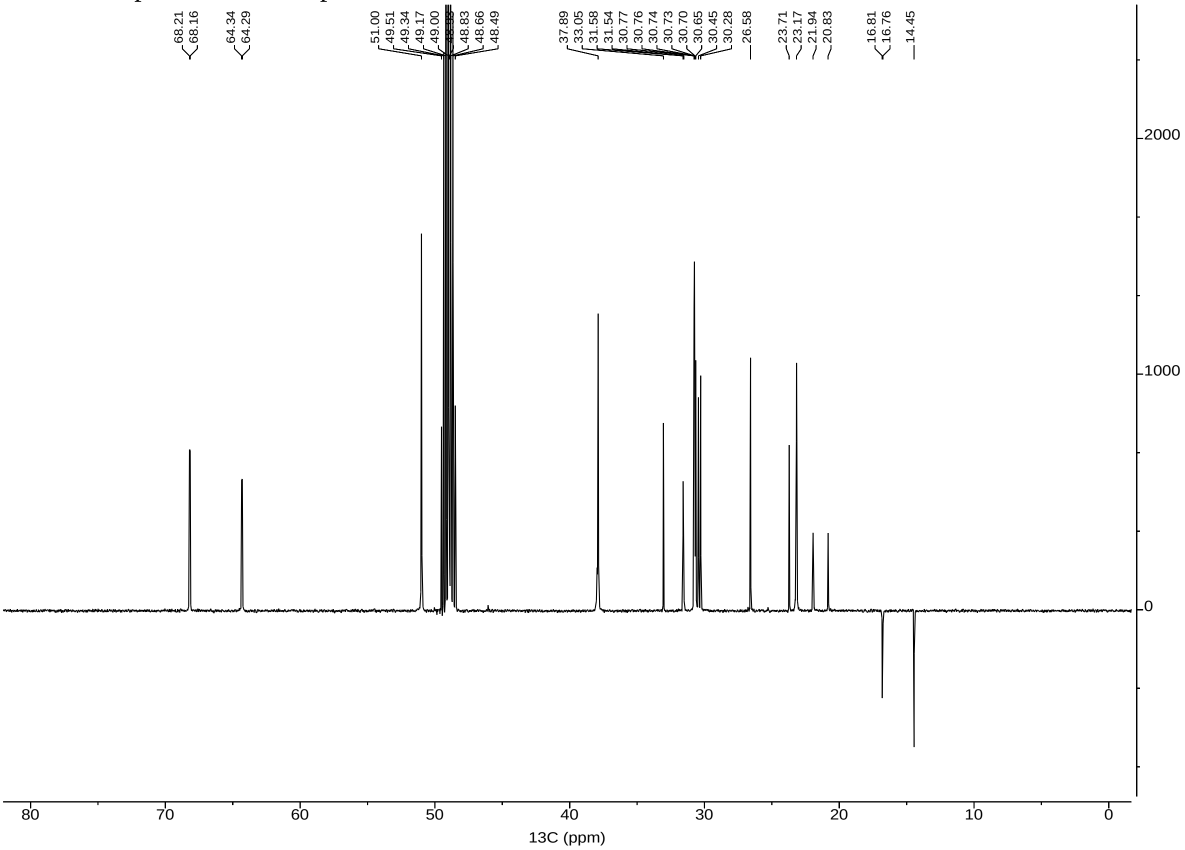

^{31}P{^{1}H} NMR spectrum of compound **10a**

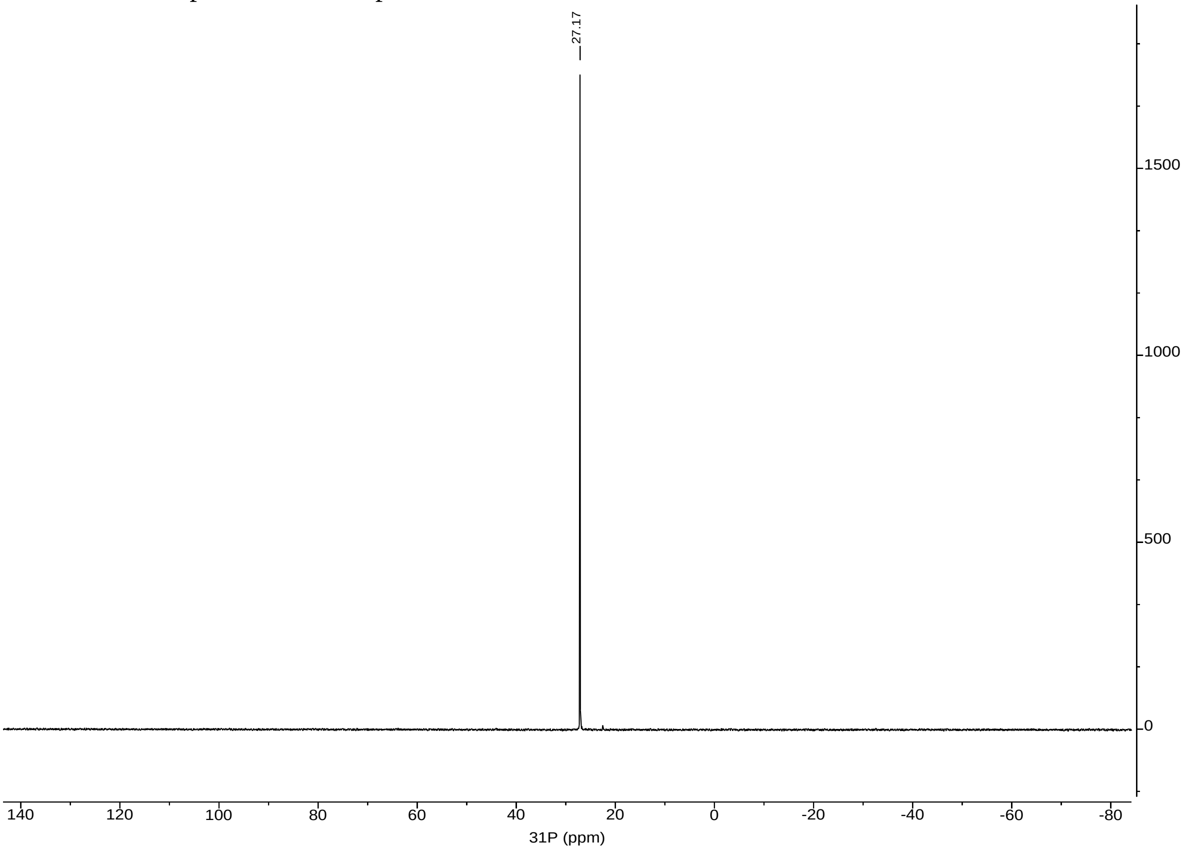

LC-MS chromatograms for compound **10a**

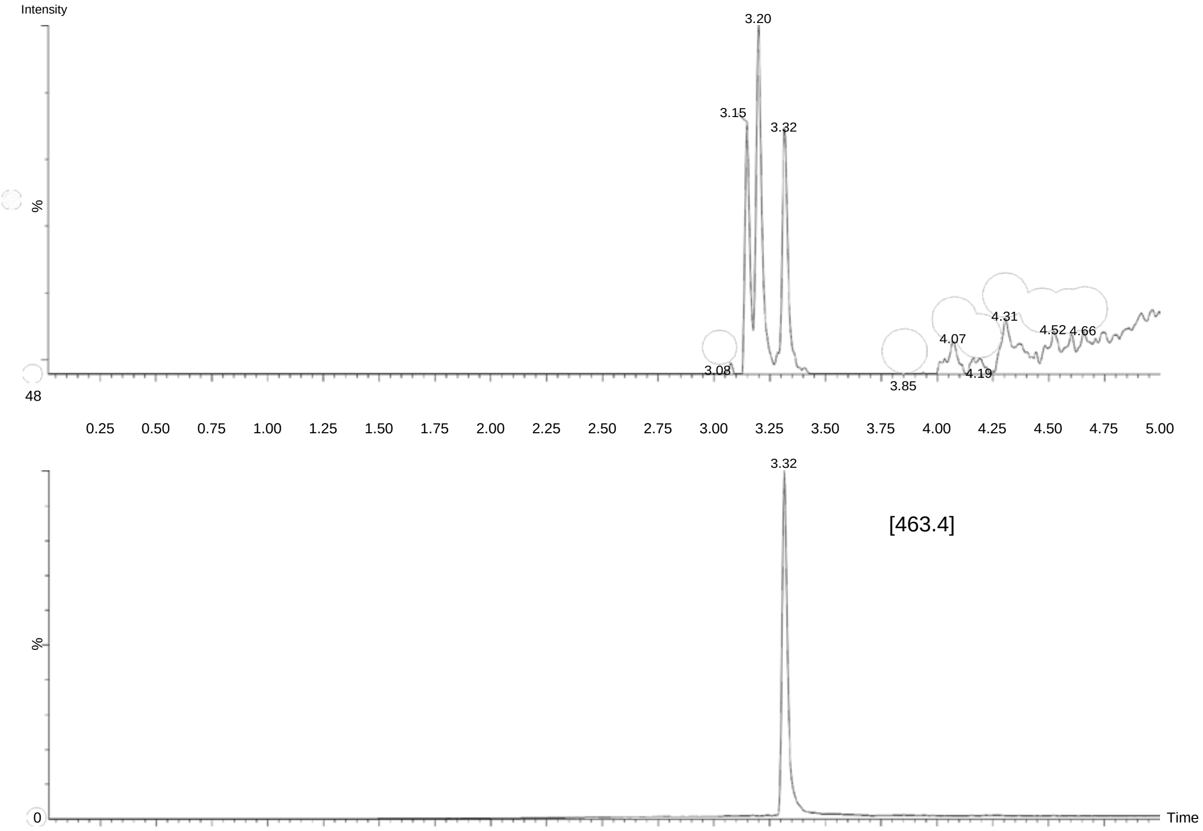

Ethyl pentadecyl (2-(bis(3-aminopropyl)amino)ethyl)phosphonate trihydrochloride (10b)

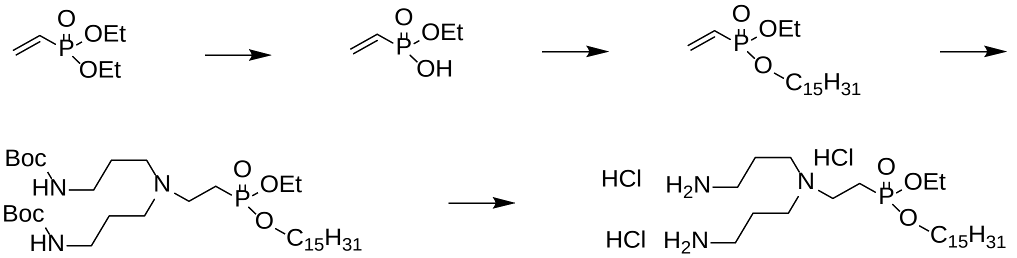

The title compound was prepared from diethyl vinylphosphonate (1.98 g, 12.05 mmol), according to general methods **A2**, **B2**, **D** and **E** in 43% overall yield (3.04 g, 5.18 mmol) as a white amorphous solid.
^{1}H NMR (500.0 MHz, CD_3OD): 4.26–4.16 (m, 2H, $CH_3C\mathit{H}_2O$), 4.16–4.09 (m, 2H, $CH_3(CH_2)_{13}C\mathit{H}_2O$), 3.49–3.43 (m, 2H, $PCH_2C\mathit{H}_2$), 3.42–3.36 (m, 4H, $C\mathit{H}_2(CH_2)_2NH_2$), 3.10 (t, 4H, J = 7.5 Hz, $C\mathit{H}_2NH_2$), 2.58–2.48 (m, 2H, $PC\mathit{H}_2$), 2.25–2.16 (m, 4H, $C\mathit{H}_2CH_2NH_2$), 1.76–1.68 (m, 2H, $CH_3(CH_2)_{12}C\mathit{H}_2CH_2O$), 1.45–1.22 (m, 27H, $CH_3(C\mathit{H}_2)_{12}(CH_2)_2O$, $C\mathit{H}_3CH_2O$), 0.90 (m, 3H, $C\mathit{H}_3(CH_2)_{14}$).
^{13}C NMR (125.7 MHz, CD_3OD): 68.19 (d, J = 6.8 Hz, $CH_3(CH_2)_{13}\mathit{C}H_2O$), 64.32 (d, J = 6.6 Hz, $CH_3\mathit{C}H_2O$), 51.02 ($\mathit{C}H_2(CH_2)_2NH_2$), 48.80 ($\mathit{C}H_2CH_2P$), 37.85 ($\mathit{C}H_2NH_2$), 33.06 ($CH_3(\mathit{C}H_2)_{12}$), 31.57 (d, J = 5.9 Hz, $CH_3(CH_2)_{12}\mathit{C}H_2CH_2O$), 30.79, 30.78, 30.76, 30.75, 30.72, 30.66, 30.46, 30.29, 26.59, 23.72 $CH_3(\mathit{C}H_2)_{12}$), 23.22 ($\mathit{C}H_2CH_2NH_2$), 21.29 (d, J = 140.5 Hz, $P\mathit{C}H_2$), 16.77 (d, J = 5.9 Hz, $\mathit{C}H_3CH_2O$), 14.44 ($\mathit{C}H_3(CH_2)_{14}$).
^{31}P{^{1}H} NMR (202.4 MHz, CD_3OD): 27.22.
IR ν_{max} (KBr) 2923 (vs), 2854 (vs), 2500–2800, 2022 (w), 1602 (m), 1468 (s), 1394 (m), 1379 (w), 1227 (s), 1060 (s, sh), 1018 (vs), 990 (s, sh), 722 (w).
HR-MS(ESI$^+$): For $C_{25}H_{57}N_3O_3P$ $[M+H]^+$ calculated 478.41321, found 478.41332.

^{1}H NMR spectrum of compound **10b**

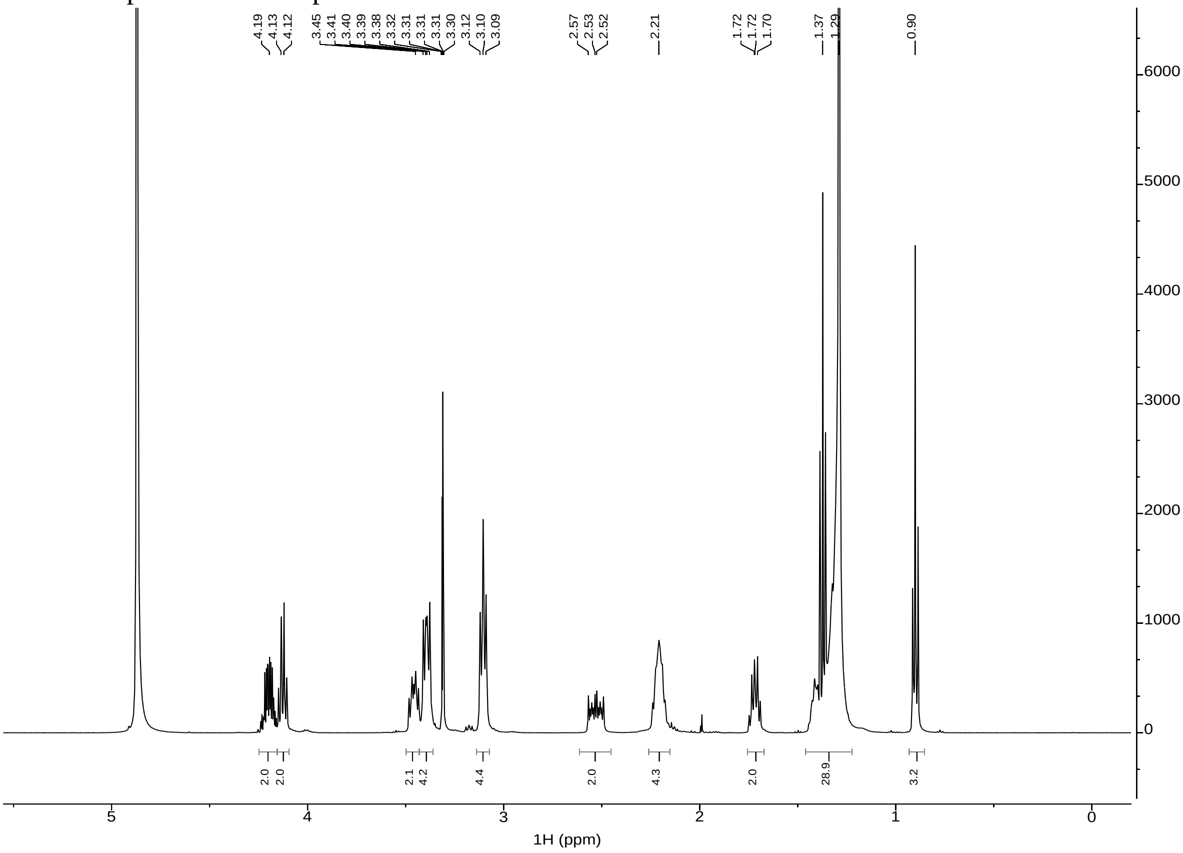

^{13}C NMR spectrum of compound **10b**

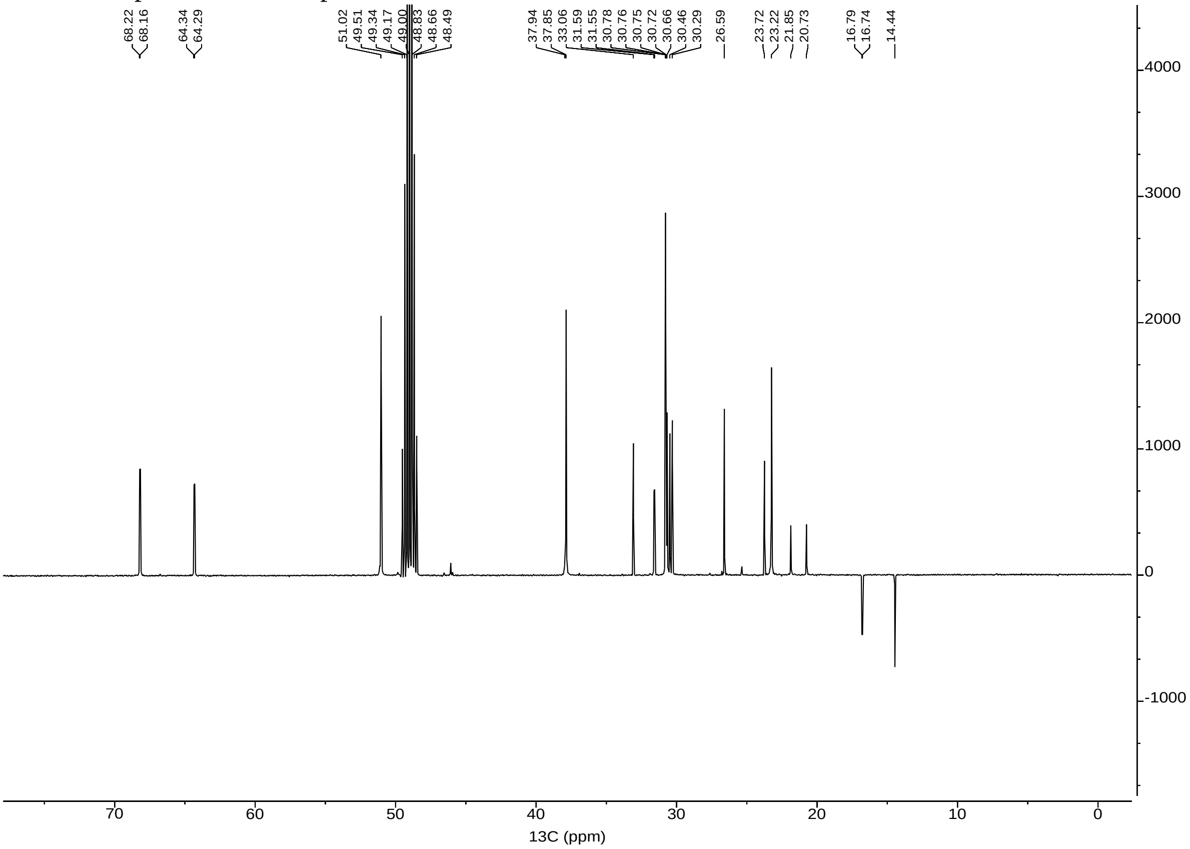

^{31}P{^{1}H} NMR spectrum of compound **10b**

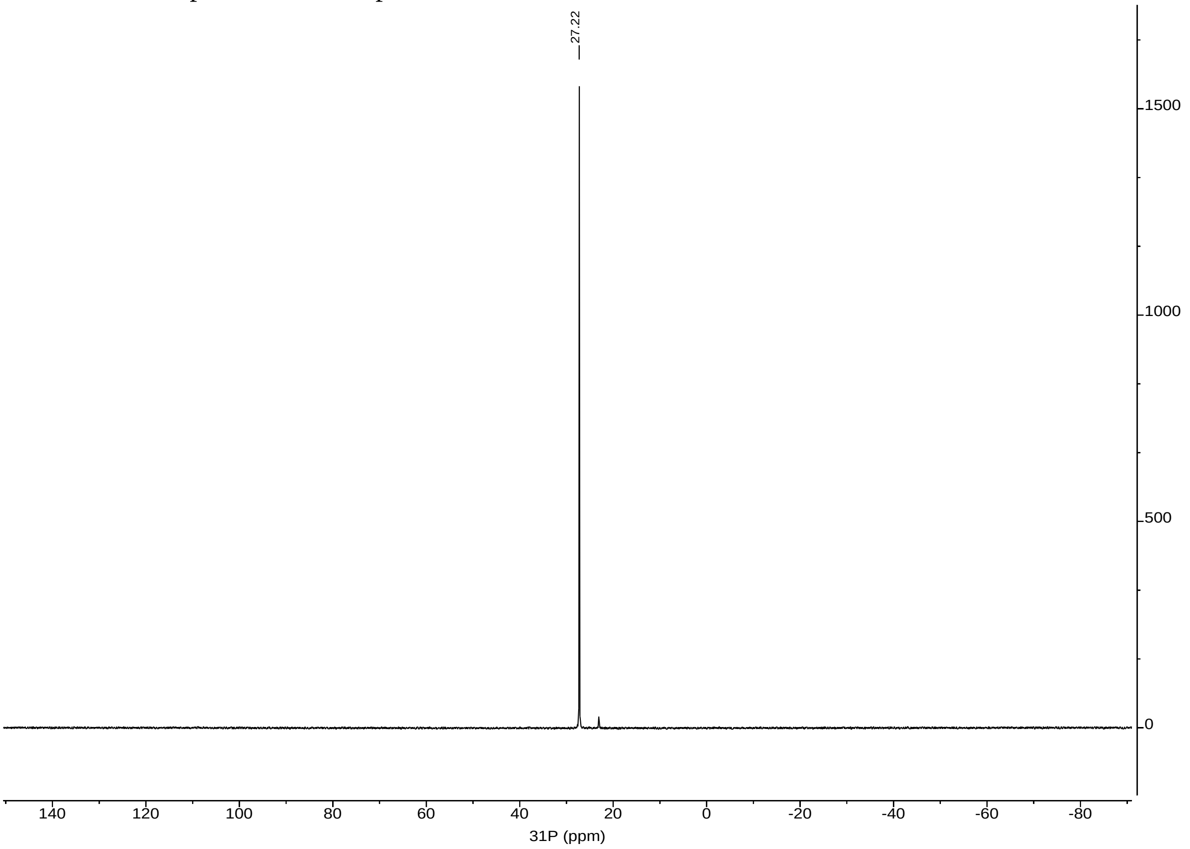

LC-MS chromatograms for compound **10b**

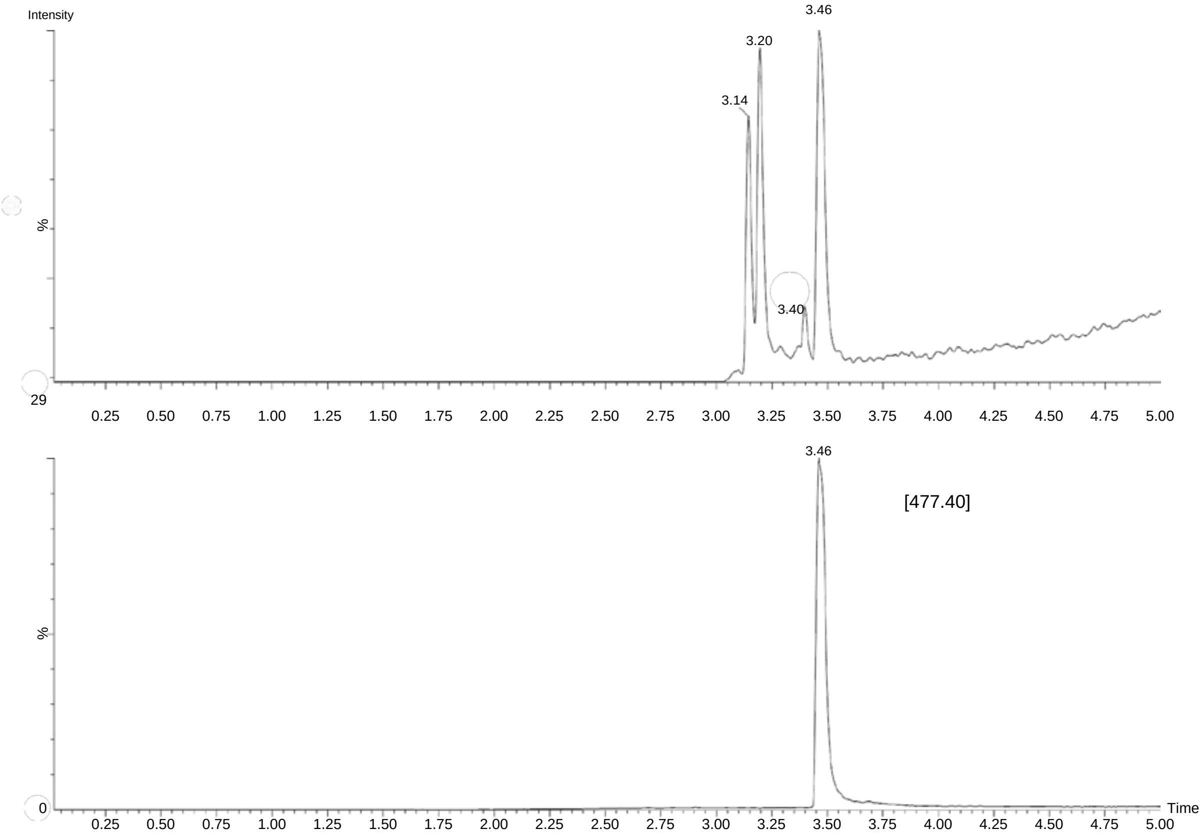

Ethyl hexadecyl (2-(bis(3-aminopropyl)amino)ethyl)phosphonate trihydrochloride (10c)

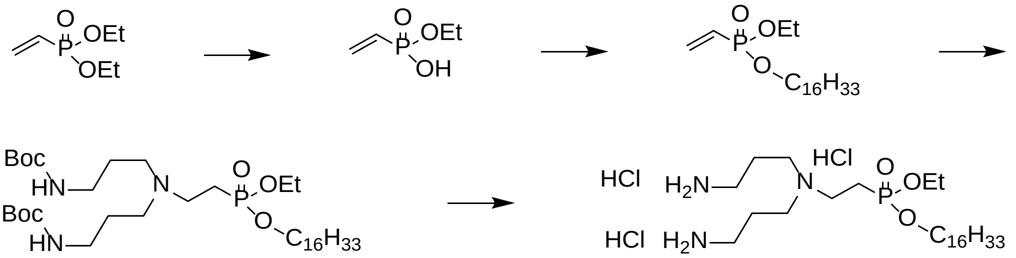

The title compound was prepared from diethyl vinylphosphonate (1.45 g, 8.81 mmol), according to general methods **A2**, **B2**, **D** and **E** in 27% overall yield (1.43 g, 2.38 mmol) as a white amorphous solid.

^{1}H NMR (500.0 MHz, CD_3OD): 4.26–4.16 (m, 2H, CH_3CH_2O), 4.16–4.08 (m, 2H, $CH_3(CH_2)_{14}CH_2O$), 3.49–3.43 (m, 2H, PCH_2CH_2), 3.43–3.37 (m, 4H, $CH_2(CH_2)_2NH_2$), 3.11 (t, 4H, J = 7.5 Hz, CH_2NH_2), 2.59–2.49 (m, 2H, PCH_2), 2.26–2.16 (m, 4H, $CH_2CH_2NH_2$), 1.75–1.68 (m, 2H, CH_2CH_2O), 1.45–1.22 (m, 29H, $CH_3(CH_2)_{13}(CH_2)_2O$, CH_3CH_2O), 0.90 (m, 3H, $CH_3(CH_2)_{15}$).

^{13}C NMR (125.7 MHz, CD_3OD): 68.17 (d, J = 6.7 Hz, $CH_3(CH_2)_{14}CH_2O$), 64.30 (d, J = 6.6 Hz, CH_3CH_2O), 51.00 ($CH_2(CH_2)_2NH_2$), 48.80 (PCH_2CH_2), 37.84 (CH_2NH_2), 33.05 ($CH_3(CH_2)_{13}$), 31.56 (d, J = 6.0 Hz, CH_2CH_2O), 30.77, 30.76, 30.74, 30.72, 30.66, 30.46, 30.29, 26.58, 23.72 ($CH_3(CH_2)_{13}$), 23.19 ($CH_2CH_2NH_2$), 21.30 (d, J = 140.4 Hz, PCH_2), 16.77 (d, J = 5.9 Hz, CH_3CH_2O), 14.45 ($CH_3(CH_2)_{15}$).

^{31}P{^{1}H} NMR (202.4 MHz, CD_3OD): 27.21.

IR ν_{max} (KBr) 3100–2500 (vs–s), 2990 (vs), 2960 (vs), 2916 (vs, br), 2852 (vs), 2725 (s), 2676 (s), 2617 (s), 2545 (s), 2488 (s), 2033 (w, br), 1604 (s), 1543 (m), 1508 (m), 1485 (s), 1468 (s), 1401 (m), 1390 (m), 1367 (m), 1227 (vs), 1164 (m), 1095 (m), 1052 (s, sh), 1036 (s, sh), 1025 (vs), 1010 (vs), 968 (s, sh), 807 (m), 721 (m).

HR-MS(ESI^+): For $C_{26}H_{59}N_3O_3P$ $(M+H)^+$ calculated 492.42886, found 492.42893.

^{1}H NMR spectrum of compound **10c**

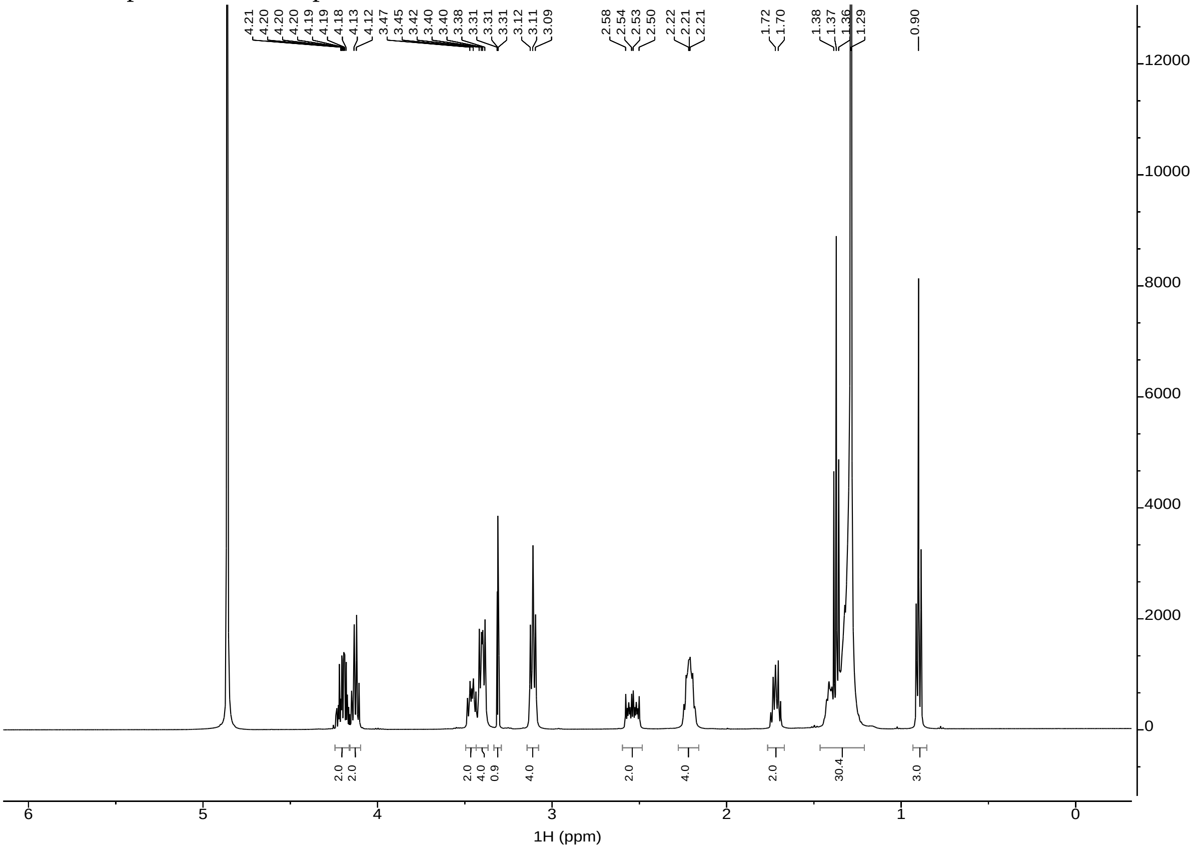

^{13}C NMR spectrum of compound **10c**

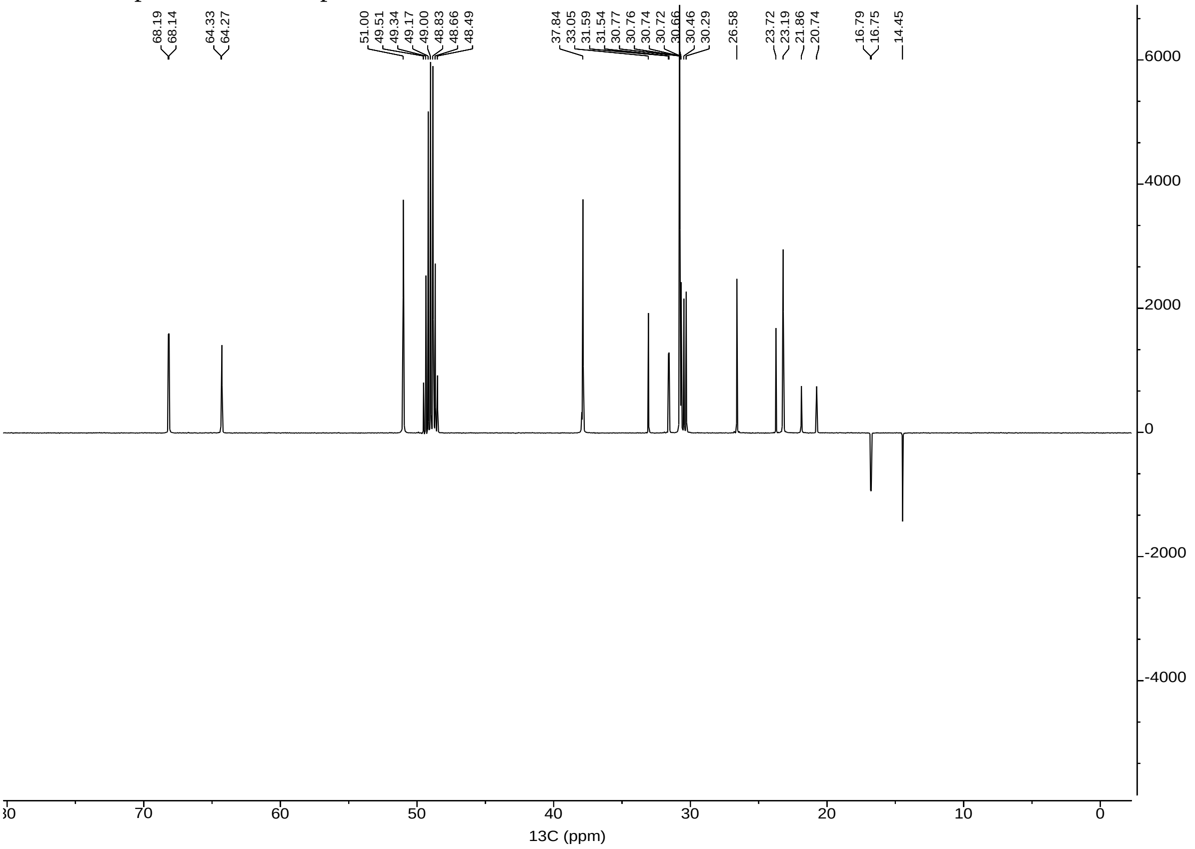

$^{31}P\{^{1}H\}$ NMR spectrum of compound **10c**

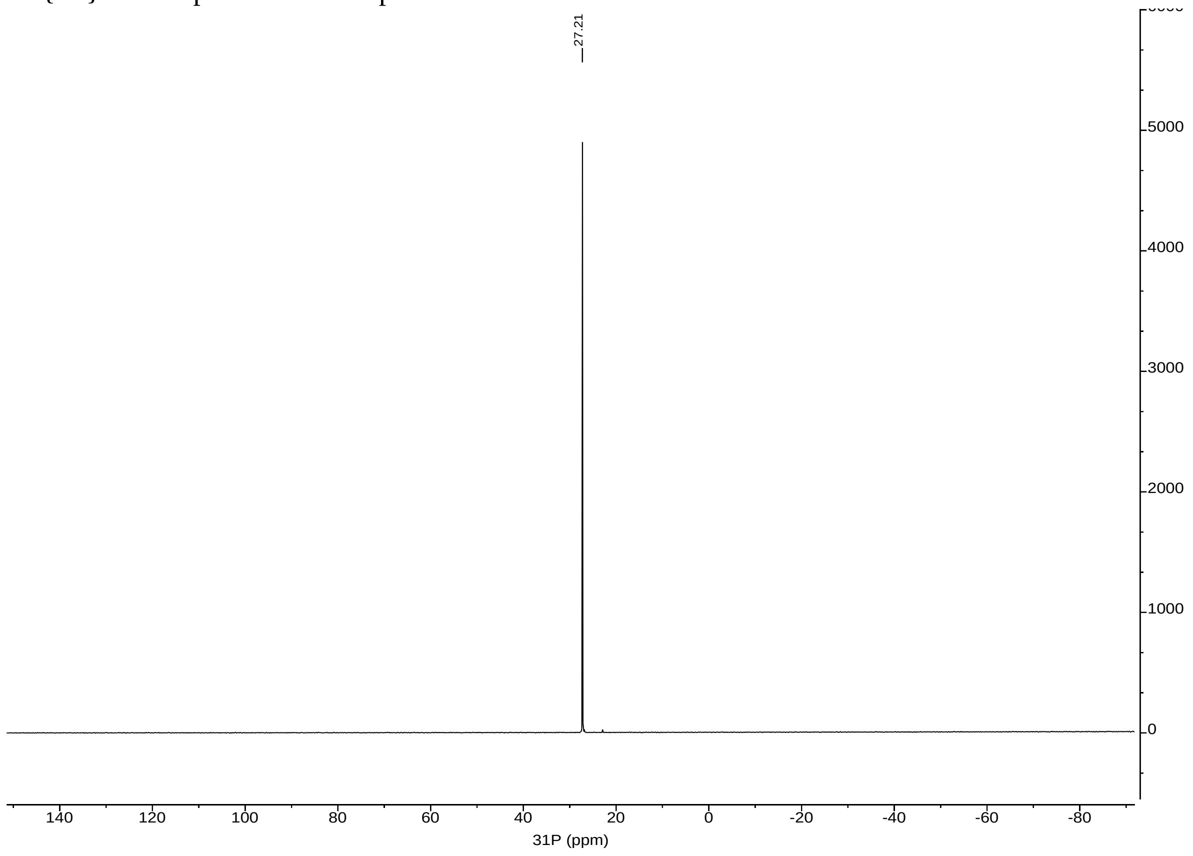

LC-MS chromatograms for compound **10c**

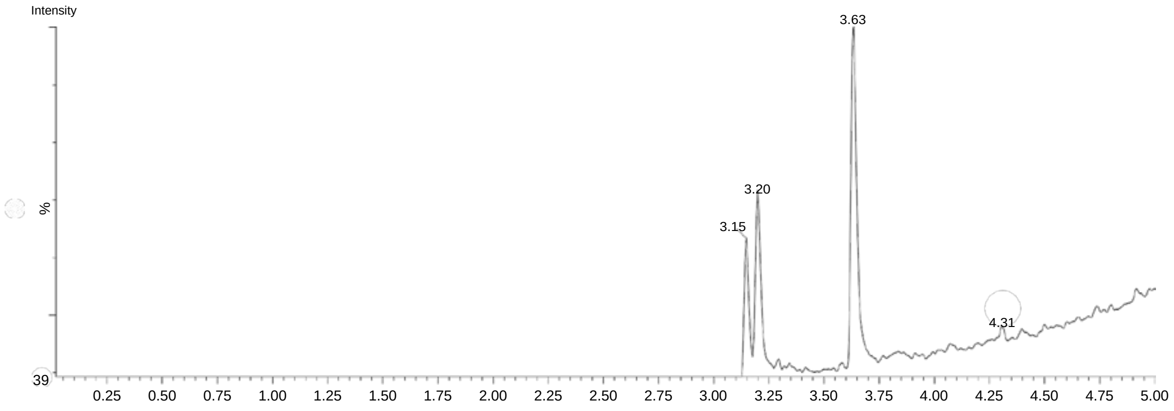

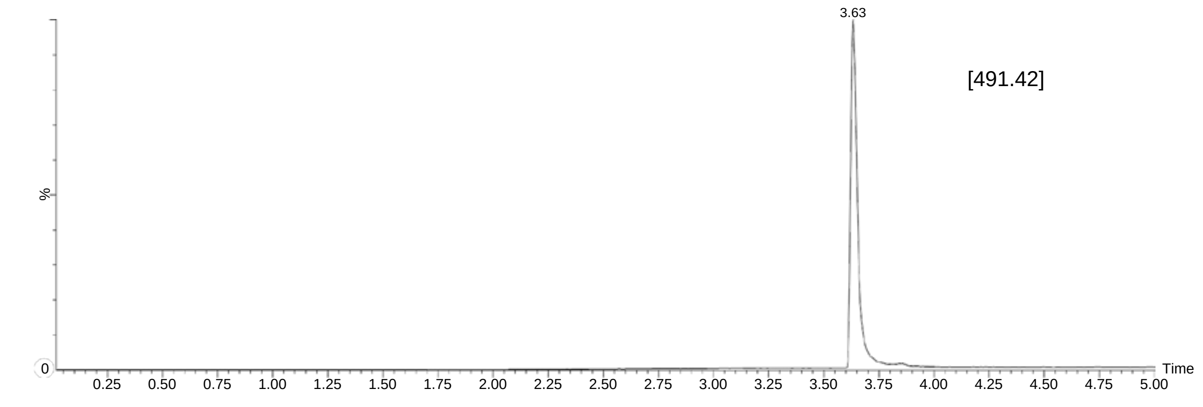

Hexyl 2-(naphthalen-1-yl)ethyl (2-(bis(3-aminopropyl)amino)ethyl)phosphonate trihydrochloride (10d)

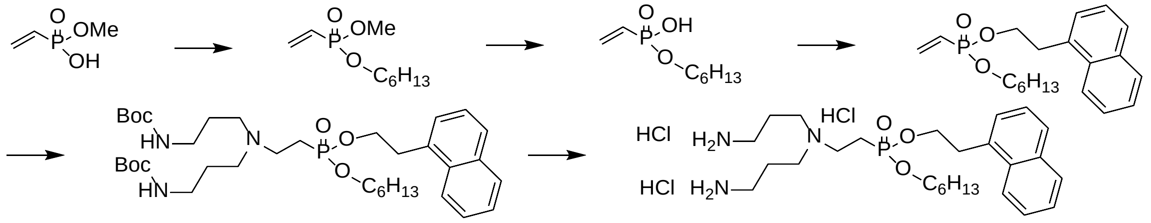

The title compound was prepared according to general methods **A**, **B2**, **D** and **E** from mono methyl vinylphosphonate (1.66 g, 12.2 mmol) in 7% overall yield (0.52 g, 0.88 mmol) as a white solid.

^{1}H NMR (401 MHz, CD_3OD): 8.17–8.11 (m, 1H, Naph*H*), 7.93–7.88 (m, 1H, Naph*H*), 7.84–7.78 (m, 1H, Naph*H*), 7.61–7.43 (m, 4H, Naph*H*), 4.53–4.42 (m 2H, OC*H*$_2$CH$_2$Naph), 3.94–3.77 (m, 2H, (CH$_2$)$_4$C*H*$_2$O), 3.53 (t, 2H, *J* = 6.6 Hz, C*H*$_2$Naph), 3.31–3.22 (m, 6H, C*H*$_2$N), 3.07 (t, 4H *J* = 7.5 Hz, C*H*$_2$NH$_2$), 2.49–2.36 (m, 2H, PC*H*$_2$), 2.22–2.07 (m, 4H, C*H*$_2$CH$_2$NH$_2$), 1.57–1.46 (m, 2H, CH$_2$C*H*$_2$CH$_2$O), 1.34–1.19 (m, 6H, CH$_3$(C*H*$_2$)$_3$), 0.96–0.83 (m, 3H, C*H*$_3$).

^{13}C NMR (101 MHz, CD_3OD): 135.44, 134.63, 133.36, 129.96, 128.71, 128.67, 126.84, 126.64, 124.63 (*C*$_{\text{Naph}}$), 68.07 (d, *J* = 6.8 Hz, *C*H$_2$O), 50.99 (*C*H$_2$(CH$_2$)$_2$NH$_2$), 48.64 (PCH$_2$*C*H$_2$), 37.84 (*C*H$_2$NH$_2$), 34.62 (d, *J* = 6.2 Hz, *C*H$_2$Naph), 32.43 (CH$_3$CH$_2$*C*H$_2$), 31.37 (d, *J* = 6.1 Hz, CH$_2$*C*H$_2$CH$_2$O), 26.13 (CH$_3$(CH$_2$)$_2$*C*H$_2$), 23.57 (CH$_3$*C*H$_2$), 23.21 (*C*H$_2$CH$_2$NH$_2$), 21.14 (d, *J* = 140.6 Hz, P*C*H$_2$), 14.34 (*C*H$_3$).

^{31}P{^{1}H} NMR (162 MHz, CD_3OD): 28.53.

IR ν_{max} (KBr) 3200–2600 (vs, vbr), 2600–2500 (m), 2956 (vs), 2930 (vs), 2859 (s), 1620 (w), 1598 (w), 1547 (w), 1470 (m), 1397 (w), 1239 (m), 1043 (m), 1050–1000 (m), 801 (w), 776 (w), 624 (w), 588 (w), 555 (w).

HR-MS(ESI$^+$): For $C_{26}H_{45}N_3O_3P$ (M+H)$^+$ *m/z* calculated 478.31931, found 478.31909.

^{1}H NMR spectrum of compound **10d**

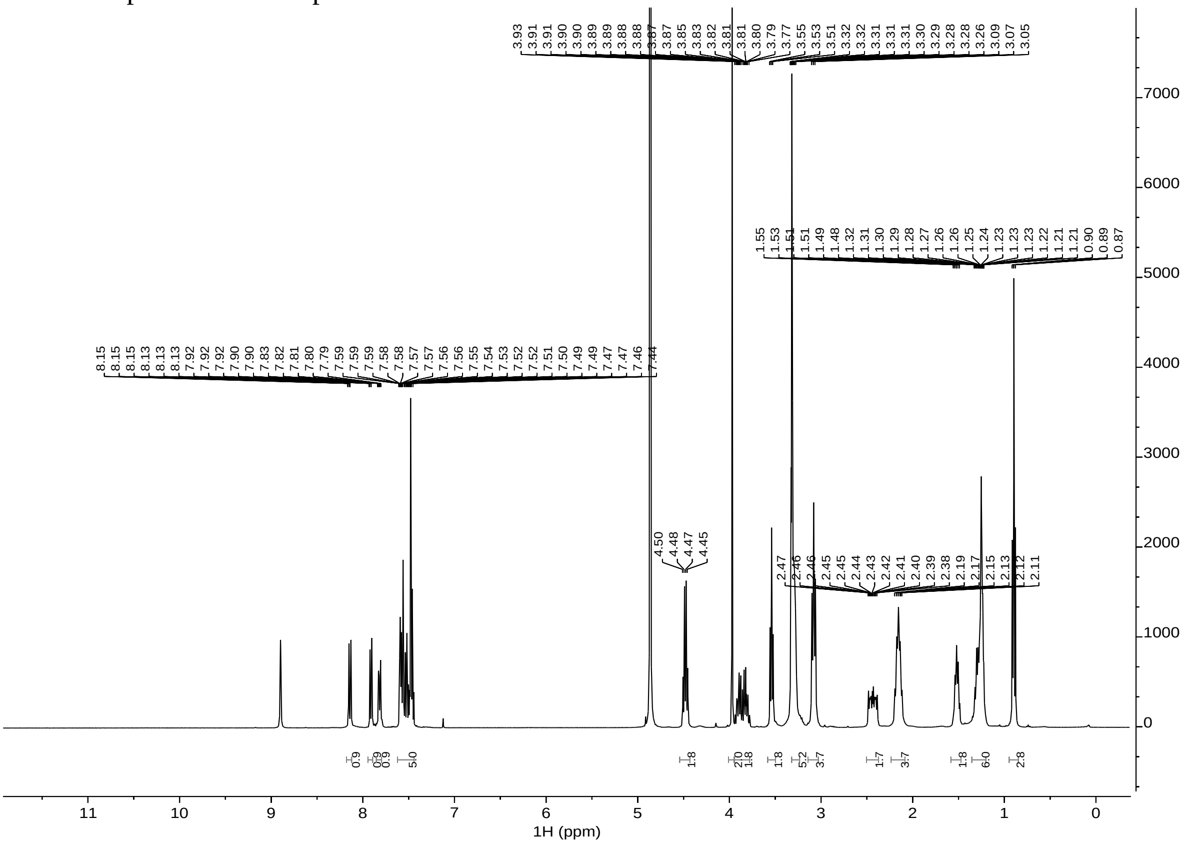

^{13}C NMR spectrum of compound **10d**

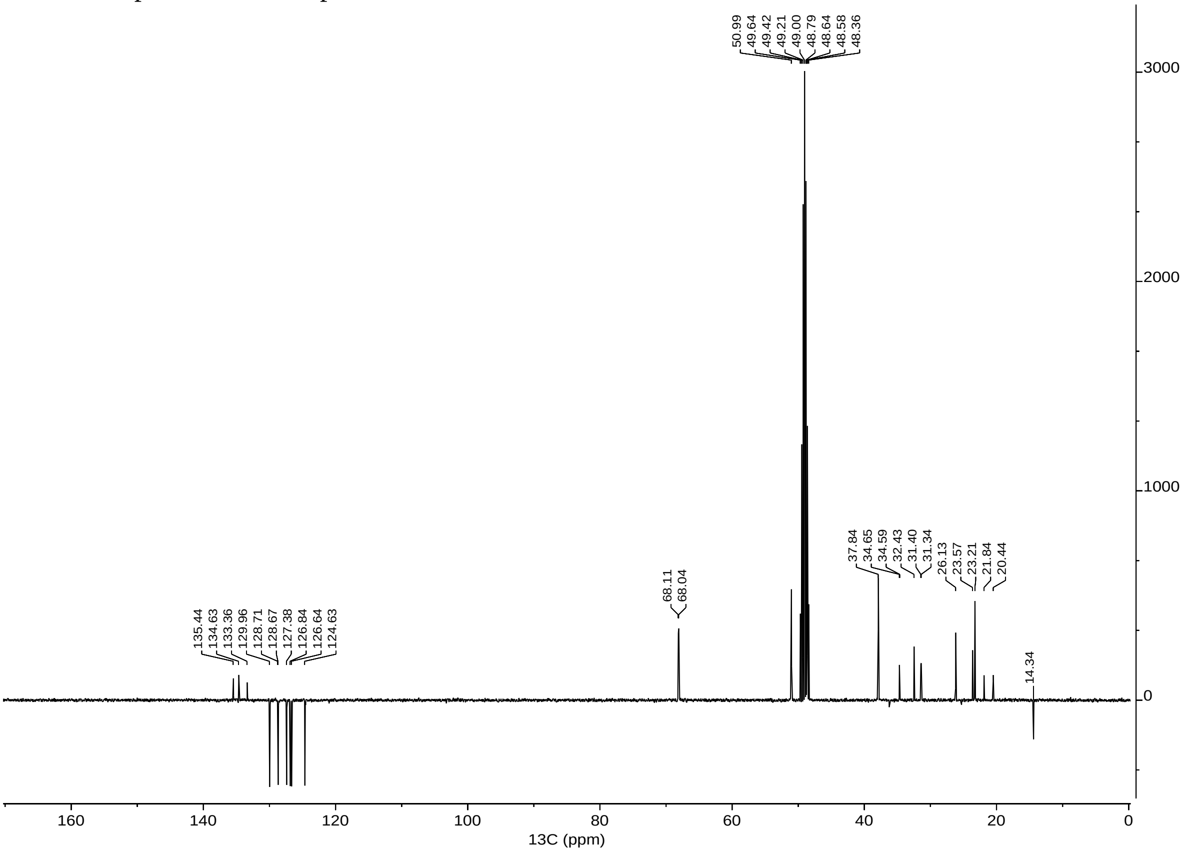

$^{31}P\{^{1}H\}$ NMR spectrum of compound **10d**

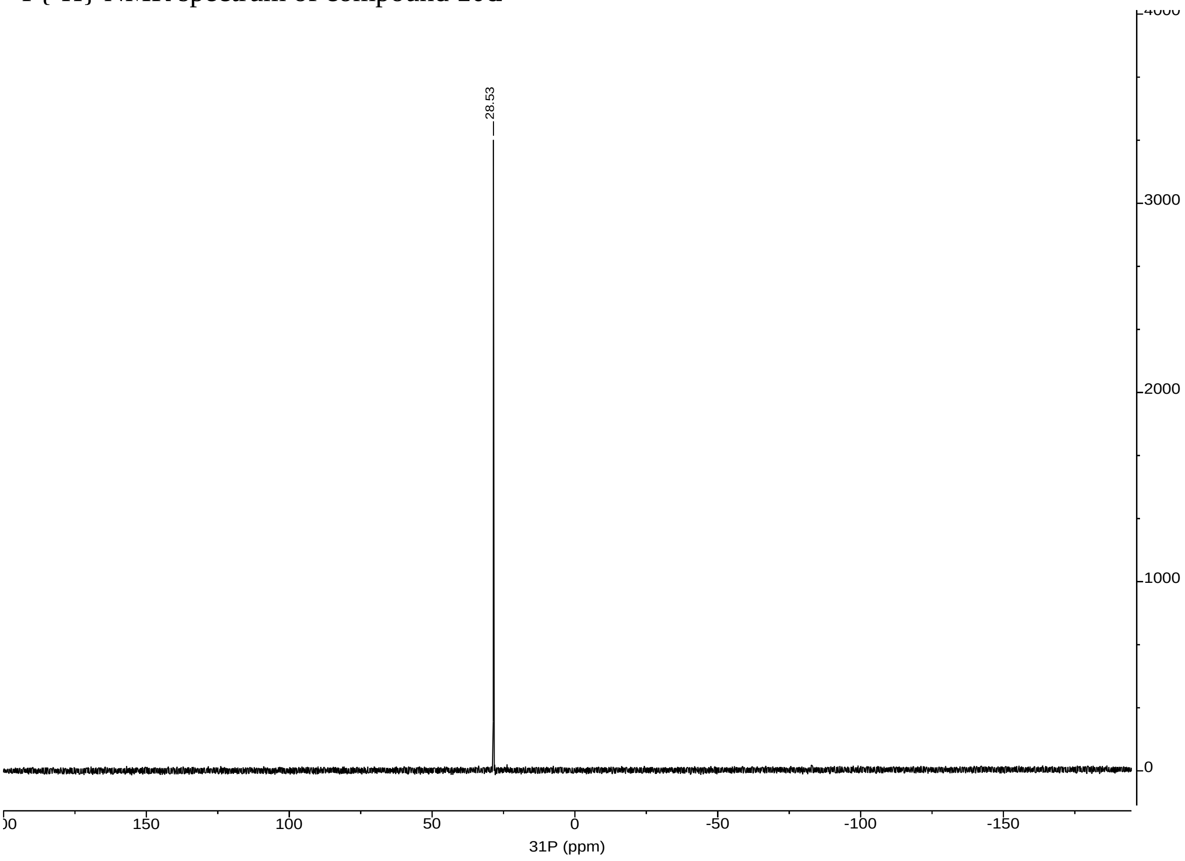

LC-MS chromatograms for compound **10d**

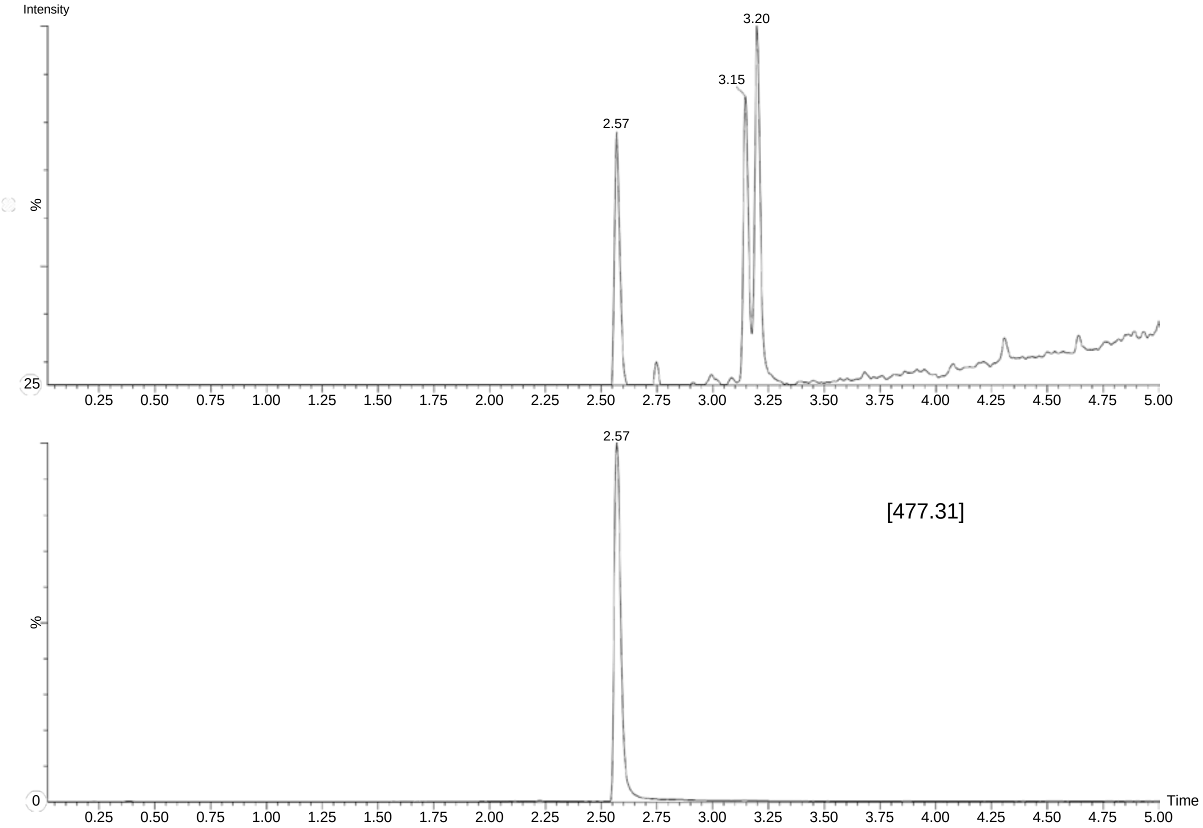

2-(Benzyloxy)ethyl octyl (2-(bis(3-aminopropyl)amino)ethyl)phosphonate trihydrochloride (10e)

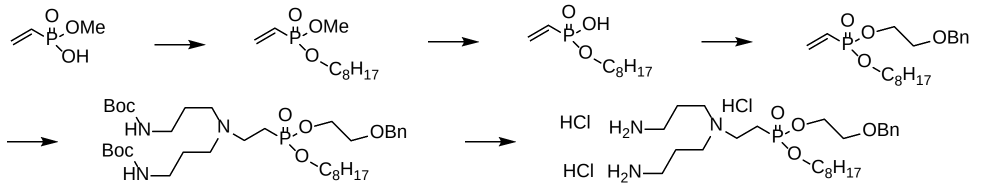

The title compound was prepared according to general methods **A**, **B2**, **D** and **E** from mono methyl vinylphosphonate (0.86 g, 6.30 mmol) in 8% overall yield (0.28 g, 0.47 mmol) as a white solid.

^{1}H NMR (400 MHz, CD_3OD): 7.44–7.27 (m, 5H, Ph*H*), 4.60 (d, 2H, *J* = 1.9 Hz, C*H*$_2$Ph), 4.37–4.24 (m, 2H, POC*H*$_2$CH$_2$O), 4.16–4.04 (m, 2H, (CH$_2$)$_6$C*H*$_2$O), 3.81–3.71 (m, 2H, POCH$_2$C*H*$_2$O), 3.51–3.38 (m, 2H, PCH$_2$C*H*$_2$), 3.30–3.22 (m, 4H, C*H*$_2$(CH$_2$)$_2$NH$_2$), 3.06 (t, 4H, *J* = 7.2 Hz, C*H*$_2$NH$_2$), 2.61–2.43 (m, 2H, PC*H*$_2$), 2.24–2.08 (m, 4H, C*H*$_2$CH$_2$NH$_2$), 1.74–1.61 (m, 2H, CH$_2$C*H*$_2$CH$_2$O), 1.42–1.21 (m, 10H, CH$_3$(C*H*$_2$)$_5$), 0.96–0.86 (m, 3H, C*H*$_3$).

^{13}C NMR (101 MHz, CD_3OD): 139.36 (*C*$_{quat}$), 129.55, 129.05, 128.94 (*C*$_{Ph}$), 74.05 (*C*H$_2$Ph), 70.35 (d, *J* = 5.6 Hz, POCH$_2$*C*H$_2$O), 68.12 (d, *J* = 6.9 Hz, CH$_3$(CH$_2$)$_6$*C*H$_2$O), 67.35 (d, *J* = 6.6 Hz, PO*C*H$_2$CH$_2$O), 51.06 (*C*H$_2$(CH$_2$)$_2$NH$_2$), 48.70 (PCH$_2$*C*H$_2$), 37.87 (*C*H$_2$NH$_2$), 32.96 (CH$_3$CH$_2$*C*H$_2$), 31.51 (d, *J* = 6.2 Hz, CH$_2$*C*H$_2$CH$_2$O), 30.32, 30.24, 26.54 ((*C*H$_2$)$_3$(CH$_2$)$_2$O), 23.69 (CH$_3$*C*H$_2$), 23.27 (*C*H$_2$CH$_2$NH$_2$), 21.33 (d, *J* = 141.5 Hz, P*C*H$_2$), 14.42 (*C*H$_3$).

^{31}P{^{1}H} NMR (162 MHz, CD_3OD): 28.96.

IR ν_{max} (KBr) 3700–3000 (s, br), 2952 (vs), 2935 (vs), 2855 (vs), 2700–2500 (m, br), 1602 (m), 1540 (w), 1492 (m), 1465 (m), 1456 (m), 1337 (w), 1226 (s), 1100–100 (vs), 1053 (vs), 1023 (vs), 1004 (s), 902 (w), 878 (w), 733 (m), 697 (m), 556 (w).

HR-MS(ESI^{+}): For $C_{30}H_{66}N_4O_6P_2$ $(M+H)^{+}$ *m*/*z* calculated 486.34552, found 486.34590.

^{1}H NMR spectrum of compound **10e**

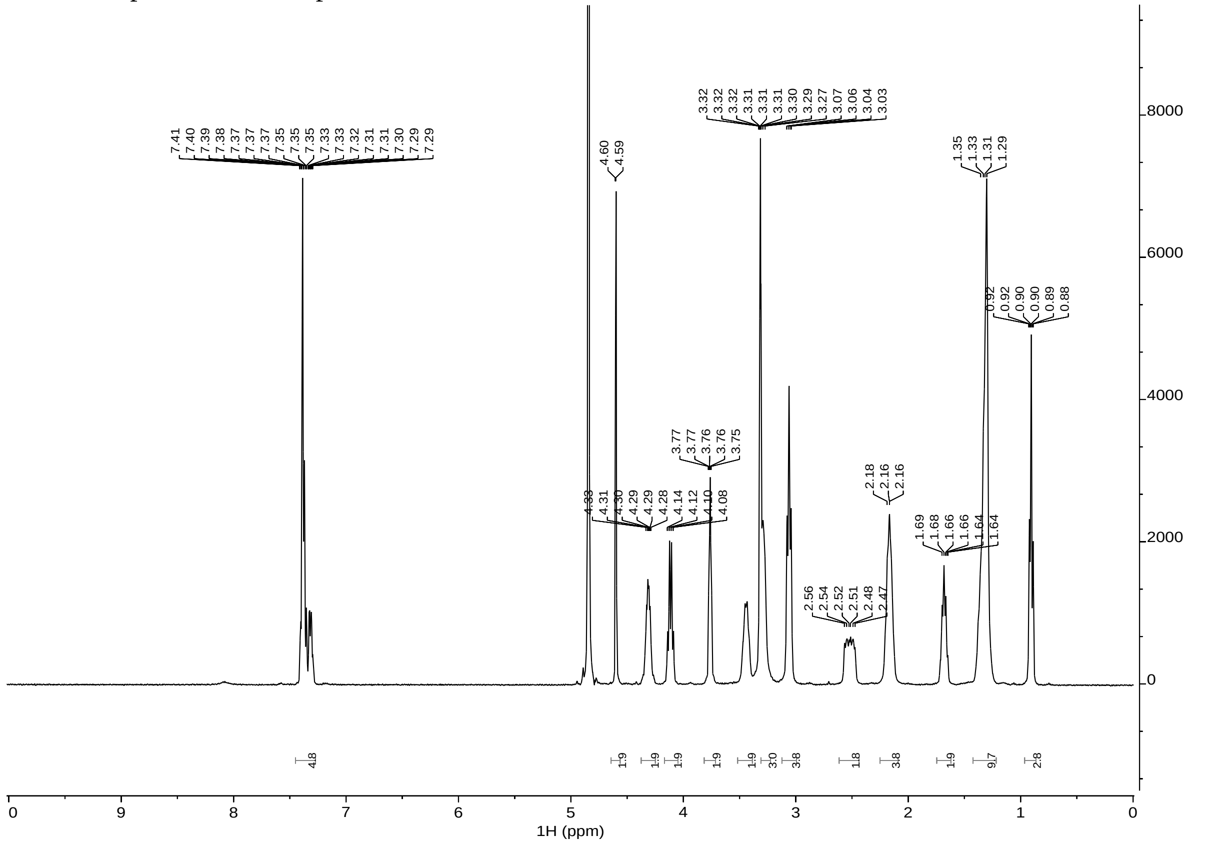

^{13}C NMR spectrum of compound **10e**

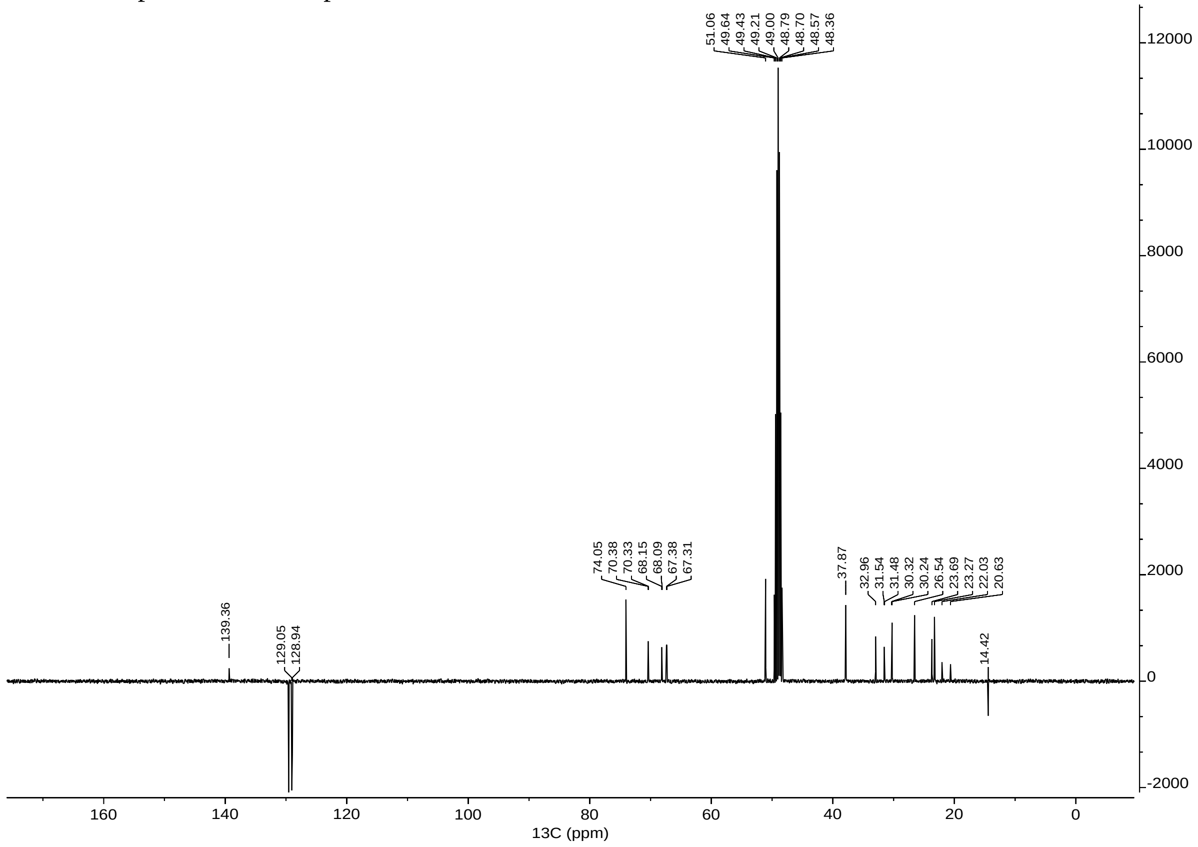

^{31}P{^{1}H} NMR spectrum of compound **10e**

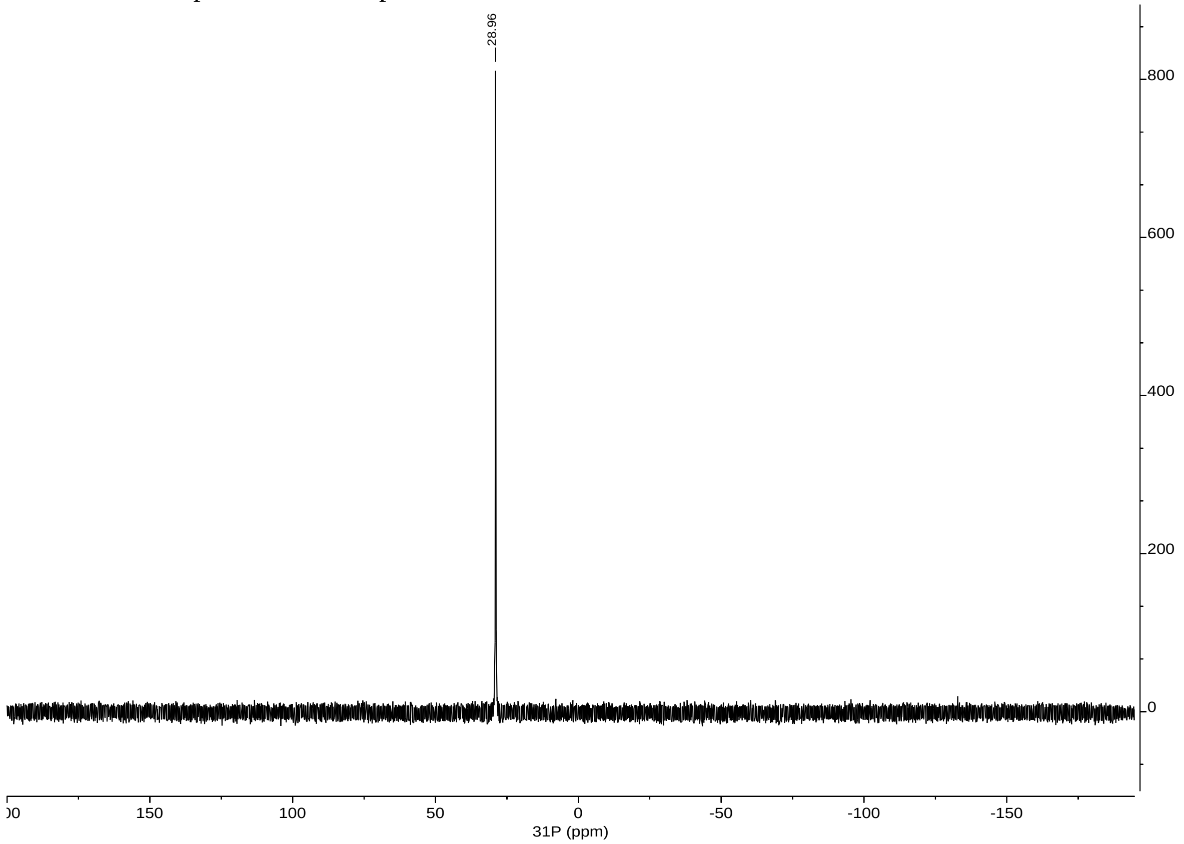

LC-MS chromatograms for compound **10e**

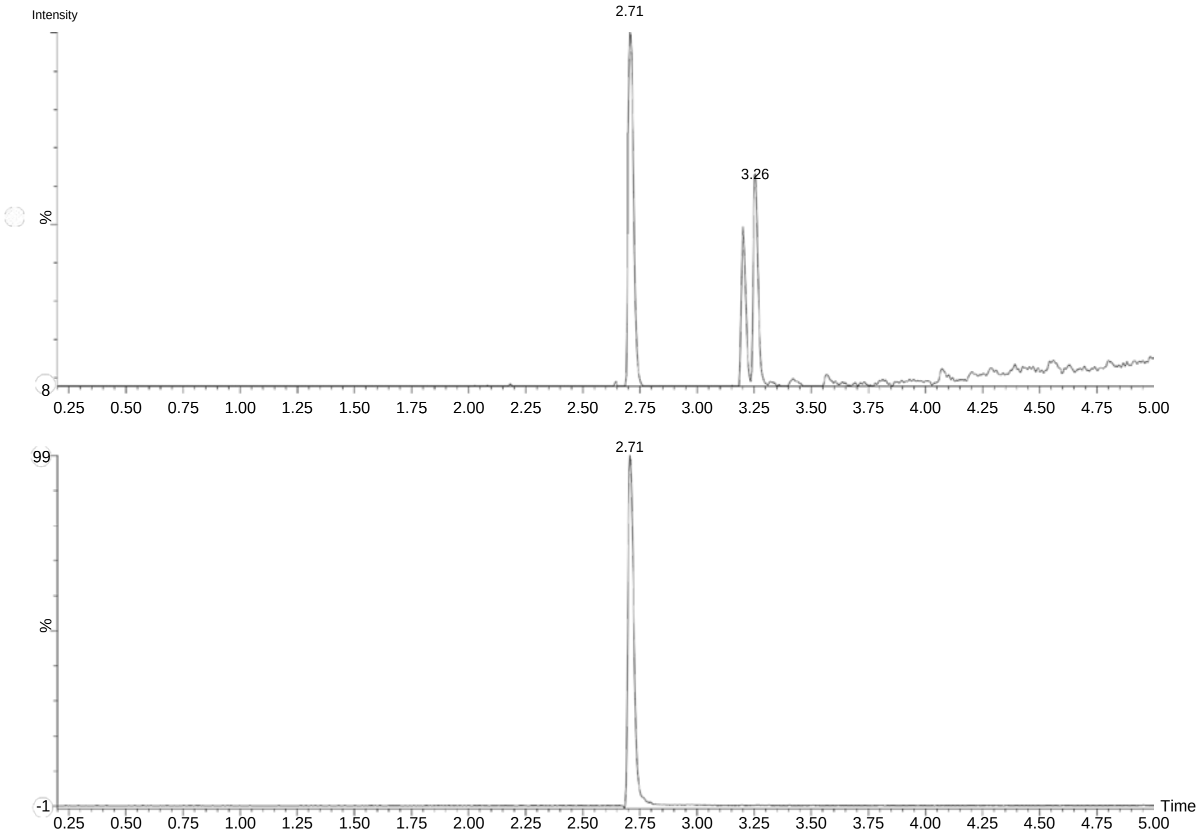

2-((4-Methoxybenzyl)oxy)ethyl nonyl (2-(bis(3-aminopropyl)amino)ethyl)phosphonate trihydrochloride (10f)

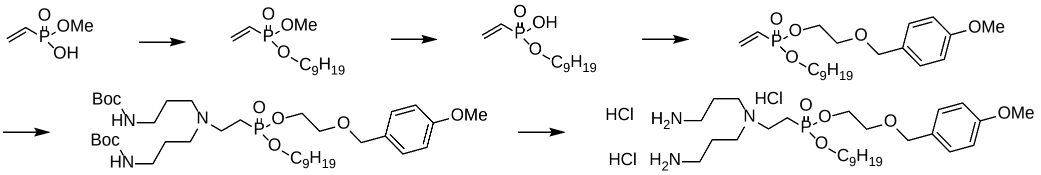

The title compound was prepared according to general methods **A**, **B2**, **D** and **E** from mono methyl vinylphosphonate (1.95 g, 14.3 mmol) in 10% overall yield (0.88 g, 1.37 mmol) as a white solid.

^{1}H NMR (400 MHz, CD_3OD): 7.30–7.14 (m, 2H, *o*-Ph*H*), 7.01–6.82 (m, 2H, *m*-Ph*H*), 4.36–4.25 (m, 2H, $POCH_2CH_2O$), 4.04–3.89 (m, 2H, $(CH_2)_7CH_2O$), 3.78 (s, 3H, CH_3O), 3.37–3.27 (10H, m, CH_2N, CH_3OPhCH_2O), 3.09 (t, 4H, J = 7.5 Hz, CH_2NH_2), 2.96 (t, 2H, J = 6.6 Hz, $POCH_2CH_2O$), 2.52–2.35 (m, 2H, PCH_2), 2.24–2.10 (m, 4H, $CH_2CH_2NH_2$), 1.69–1.58 (m, 2H, $CH_2CH_2CH_2O$), 1.39–1.25 (m, 12H, $CH_3(CH_2)_6$), 0.95–0.85 (m, 3H, CH_3).

^{13}C NMR (101 MHz, CD_3OD): 160.05 (CH_3OC), 131.23 (*o*-Ph*C*), 130.68 (OCH_2CqPh), 115.09 (*m*-Ph*C*), 68.83 (d, J = 6.9 Hz, $POCH_2CH_2O$), 68.08 (d, J = 6.8 Hz, $(CH_2)_6CH_2O$), 55.78 (CH_3O), 51.05 ($CH_2(CH_2)_2NH_2$), 48.65, 48.63 (PCH_2CH_2, CH_3OPhCH_2O), 37.87 (CH_2NH_2), 36.86 (d, J = 6.4 Hz, $POCH_2CH_2O$), 33.04 ($CH_3CH_2CH_2$), 31.53 (d, J = 6.3 Hz, $(CH_2)_6CH_2CH_2O$), 30.64, 30.39, 30.28, 26.53 ($(CH_2)_4(CH_2)_2O$), 23.72 (CH_3CH_2), 23.29 ($CH_2CH_2NH_2$), 21.15 (d, J = 140.9 Hz, PCH_2), 14.43 (CH_3).

^{31}P{^{1}H} NMR (162 MHz, CD_3OD): 28.25.

IR ν_{max} (KBr) 3100–2500 (vs, vbr), 2956 (s), 2926 (vs), 2856 (s), 1612 (m), 1584 (w), 1514 (m), 1444 (w, sh), 1247 (s), 1180 (w), 1068 (m), 1041 (m), 1007 (m), 827 (w), 811 (w), 758 (w), 703 (w), 562 (w), 522 (w).

HR-MS(ESI^{+}): For $C_{26}H_{51}N_3O_4P$ $(M+H)^{+}$ *m/z* calculated 500.36117, found 500.36069.

^{1}H NMR spectrum of compound **10f**

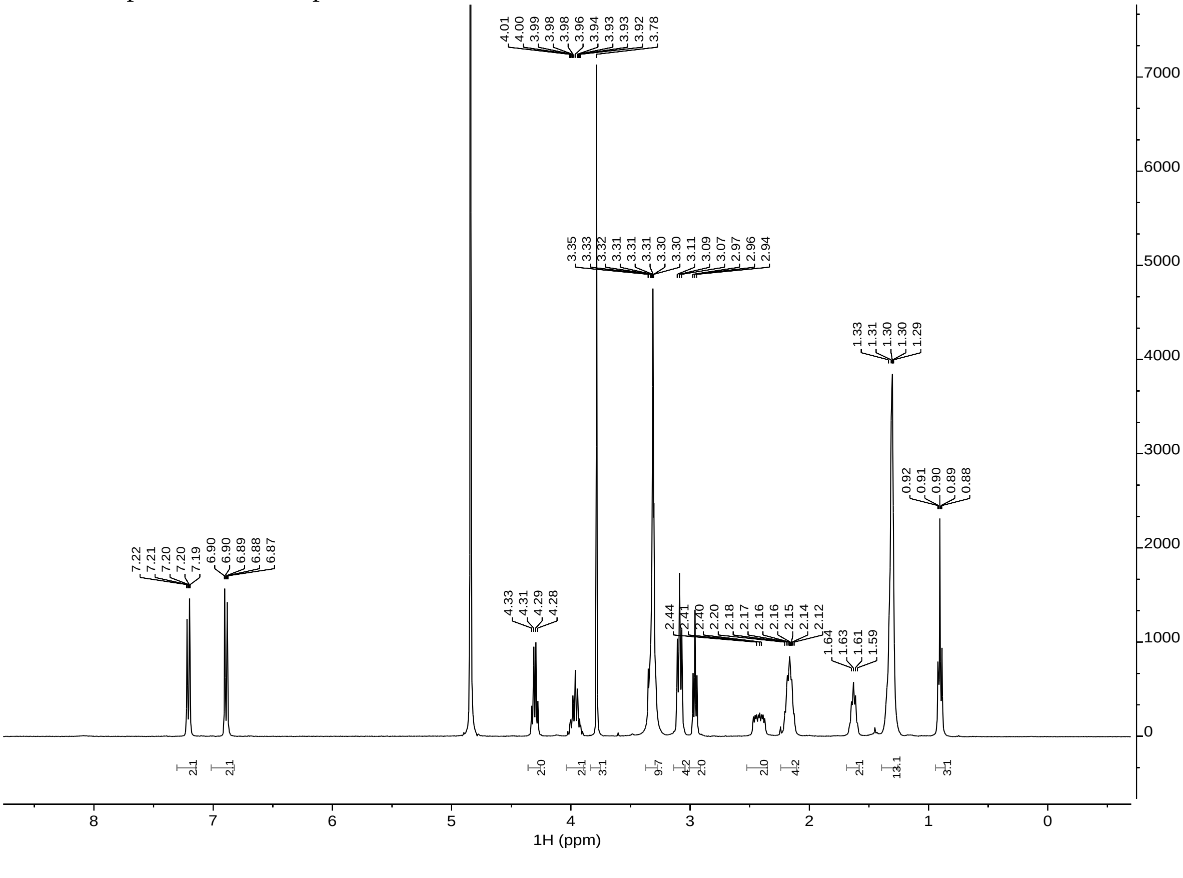

^{13}C NMR spectrum of compound **10f**

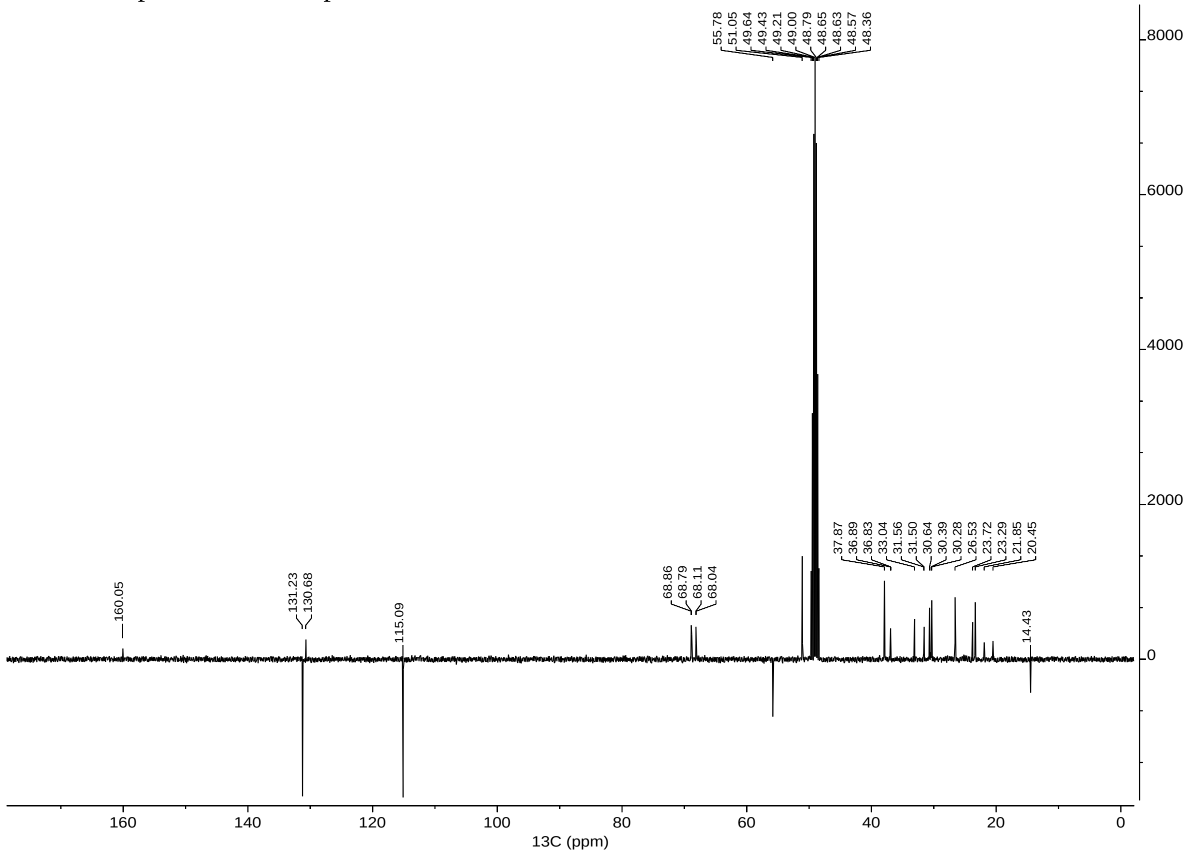

^{31}P{^{1}H} NMR spectrum of compound **10f**

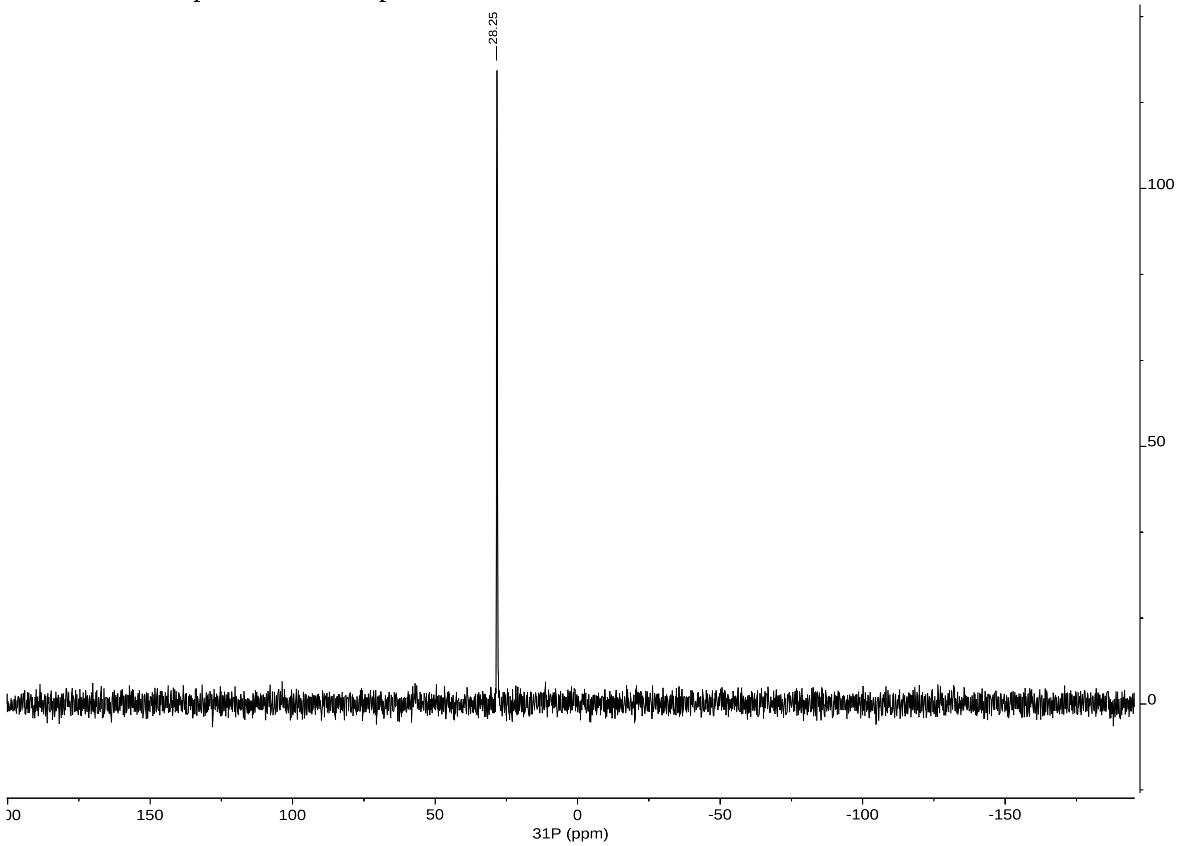

2-(Naphthalen-1-yl)ethyl nonyl (2-(bis(3-aminopropyl)amino)ethyl)phosphonate trihydrochloride (10g)

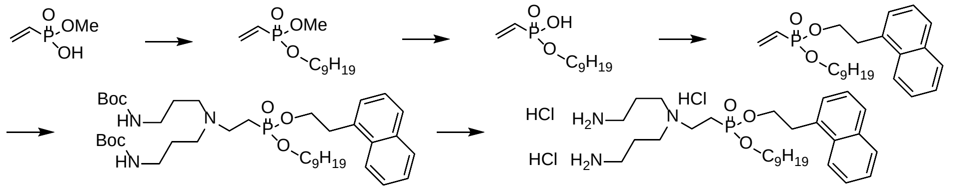

The title compound was prepared according to general methods **A**, **B2**, **D** and **E** from mono methyl vinylphosphonate (0.52 g, 3.82 mmol) in 9.0% overall yield (0.21 g, 0.34 mmol) as a white solid.

^{1}H NMR (400 MHz, CD_3OD): 8.18–8.10 (m, 1H, Naph*H*), 7.92–7.90 (m, 1H, Naph*H*), 7.86–7.78 (m, 1H, Naph*H*), 7.62–7.42 (m, 4H, Naph*H*), 4.54–4.40 (m, 2H, C$H_2$$CH_2$Naph), 3.96–3.77 (m, 2H, $(CH_2)_7$CH_2O), 3.53 (t, 2H, J = 6.6 Hz, CH_2Naph), 3.29–3.22 (m, 6H, CH_2N), 3.06 (t, 4H, J = 7.6 Hz, C$H_2$$NH_2$), 2.49–2.30 (m, 2H, PC$H_2$), 2.19–2.04 (m, 4H, C$H_2$$CH_2NH_2$), 1.57–1.46 (m, 2H, CH_2C$H_2$$CH_2$O), 1.36–1.17 (m, 12H, CH_3(CH_2)$_6$, 0.95–0.84 (m, 3H, CH_3).

^{13}C NMR (101 MHz, CD_3OD): 135.44, 134.61, 133.36, 129.97, 128.72, 128.65, 127.38, 126.84, 126.63, 124.62 (Naph*C*), 68.08 (d, J = 6.9 Hz), 68.05 (d, J = 6.8 Hz, *C*H_2O), 50.99 (*C*$H_2$$(CH_2)_2NH_2$), 48.60 ($PCH_2$*C*$H_2$), 37.84 (*C*$H_2$$NH_2$), 34.63 (d, J = 6.2 Hz, *C*H_2Naph), 33.02 (CH_3CH_2*C*H_2), 31.41 (d, J = 6.0 Hz, CH_2*C*$H_2$$CH_2$O), 30.58, 30.37, 30.22, 26.44 ((*C*H_2)$_4$$(CH_2)_2$O), 23.72 ($CH_3$*C*$H_2$), 23.24 (*C*$H_2$$CH_2NH_2$), 21.14 (d, J = 140.6 Hz, P*C*H_2), 14.44 (*C*H_3).

^{31}P{^{1}H} NMR (162 MHz, CD_3OD): 28.58.

IR ν_{max} (KBr) 3445 (m, br), 2957 (vs), 2925 (vs), 2855 (s), 2667 (m), 2627 (m), 2558 (m), 1611 (w), 1599 (w), 1511 (w), 1473 (m), 1396 (w), 1257 (m), 1238 (m), 1049 (m), 1020 (m), 1002 (m), 801 (m), 776 (m), 589 (w), 556 (w).

HR-MS(ESI$^+$): For $C_{29}H_{51}N_3O_3P$ (M+H)$^+$ *m/z* calculated 520.36626, found 520.36578.

^{1}H NMR spectrum of compound **10g**

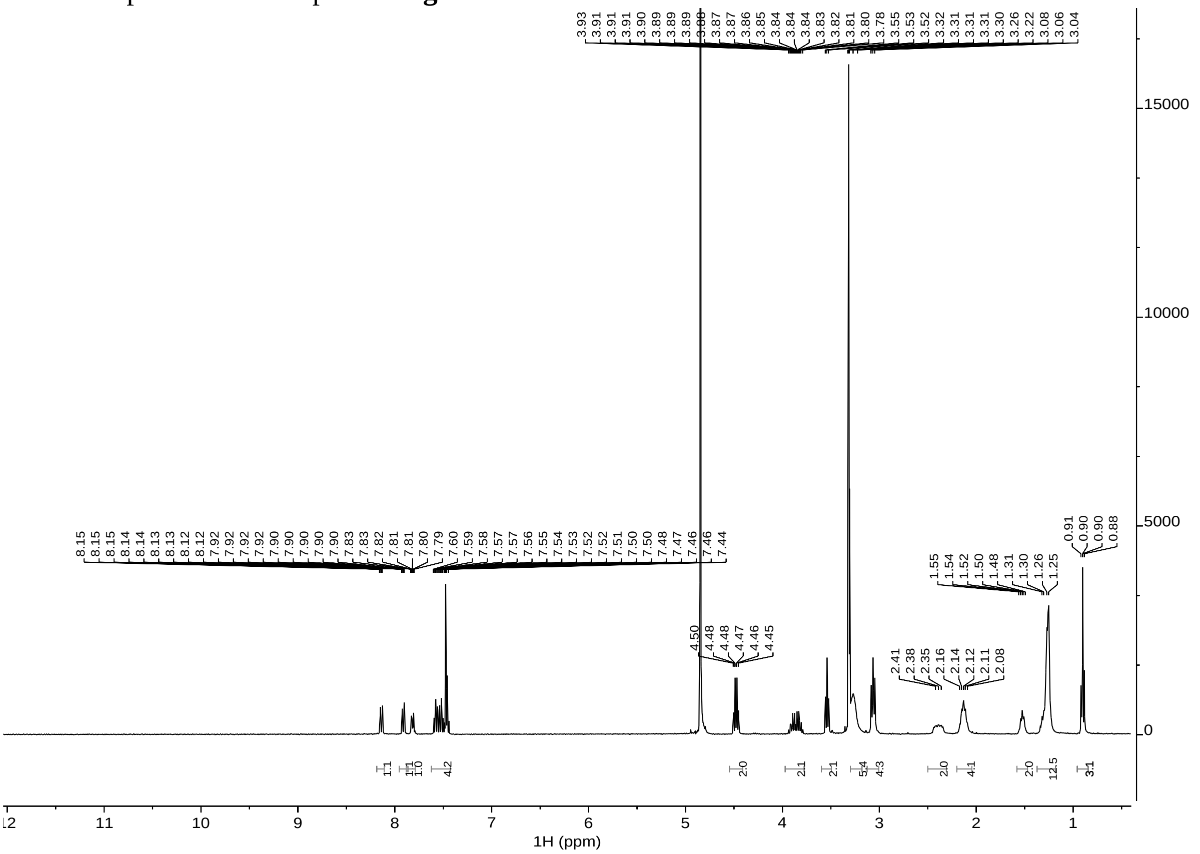

^{13}C NMR spectrum of compound **10g**

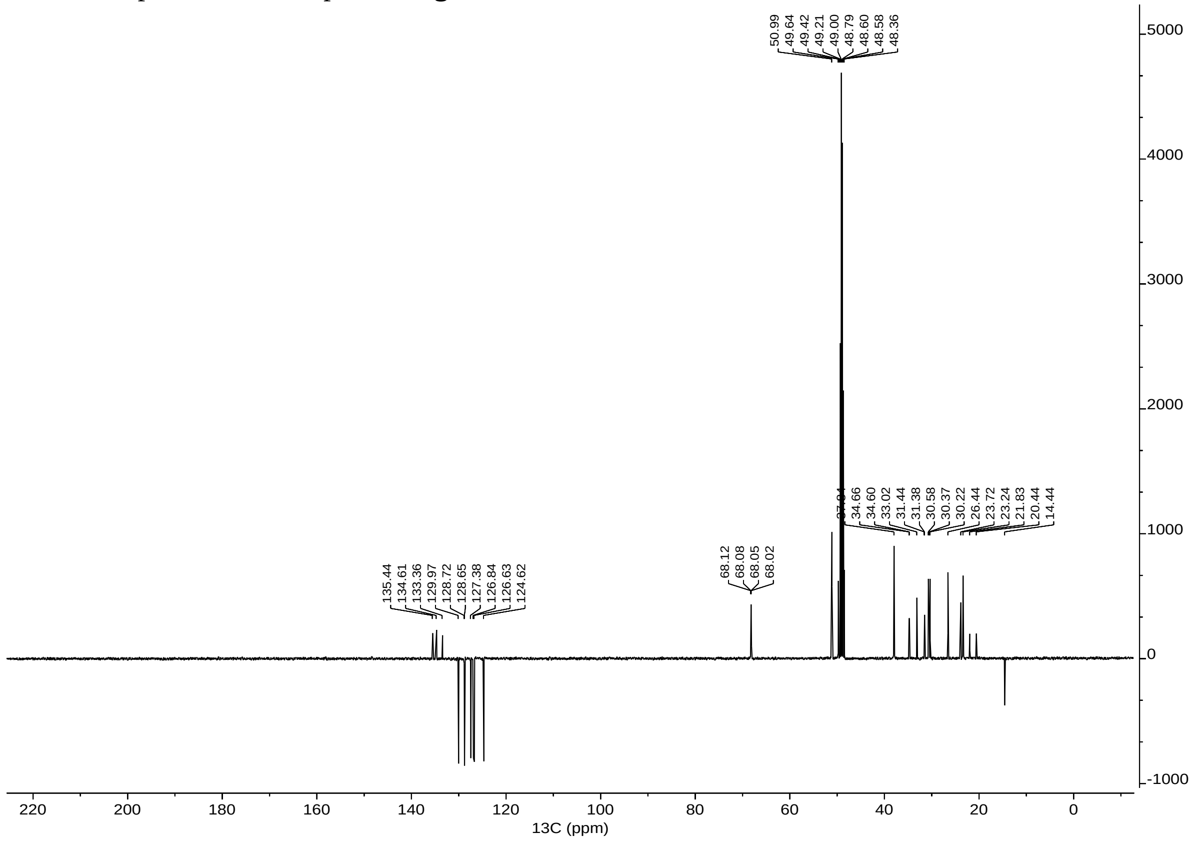

$^{31}P\{^1H\}$ NMR spectrum of compound **10g**

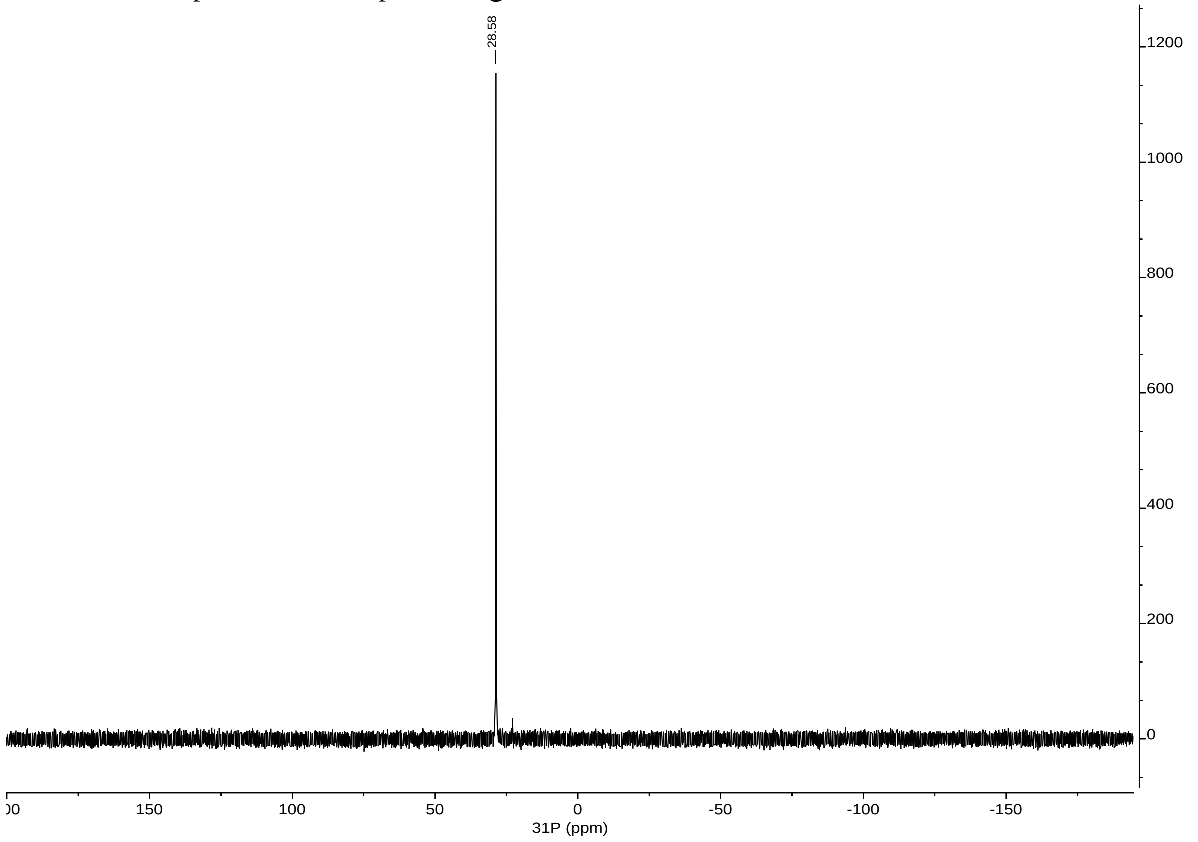

LC-MS chromatograms for compound **10g**

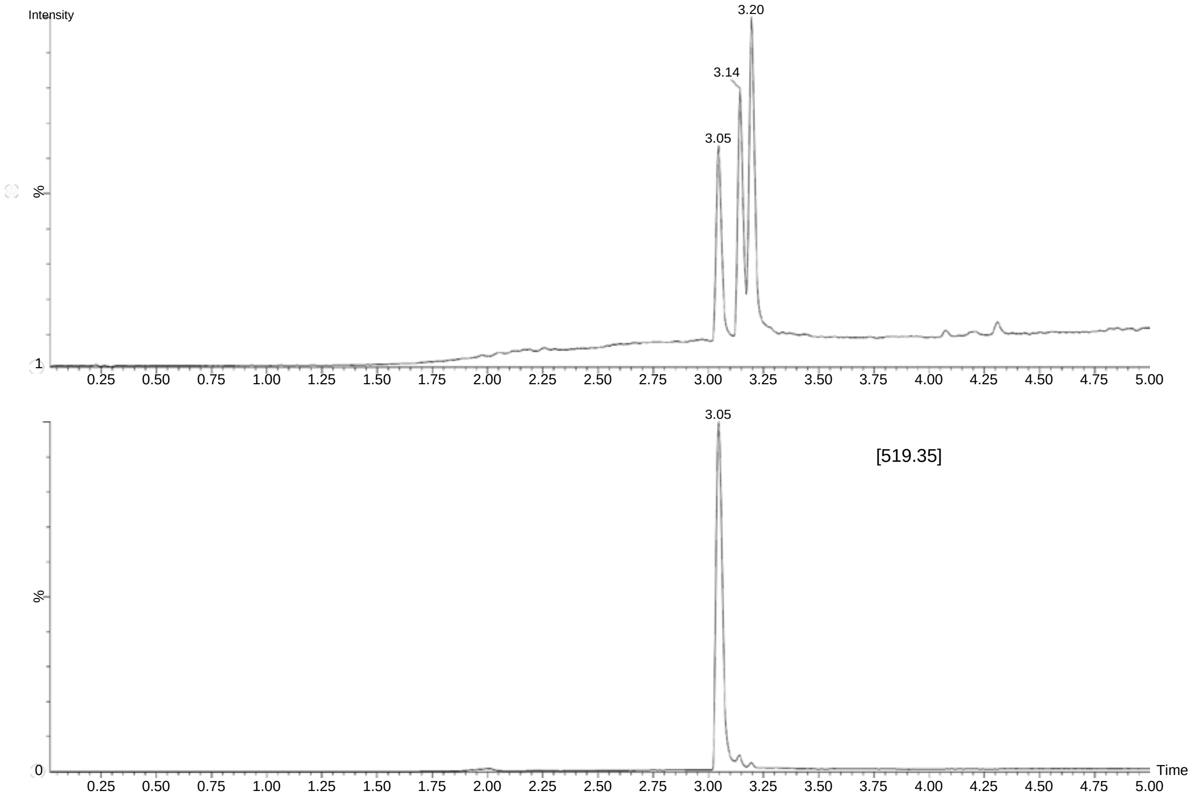

2-(Benzyloxy)ethyl nonyl (2-(bis(3-aminopropyl)amino)ethyl)phosphonate trihydrochloride (10h)

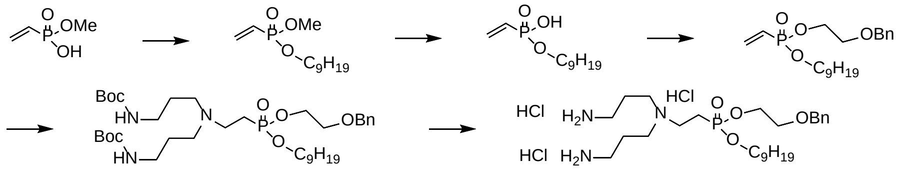

The title compound was prepared according to general methods **A**, **B2**, **D** and **E** from mono methyl vinylphosphonate (0.98 g, 7.20 mmol) in 9.0% overall yield (0.40 g, 0.66 mmol) as a white solid.

^{1}H NMR (400 MHz, CD_3OD) δ 7.46–7.27 (m, 5H, Ph*H*), 4.59 (s, 2H, C*H*$_2$Ph), 4.36–4.25 (m, 2H, POC*H*$_2$CH$_2$O), 4.19–4.05 (m, 2H, (CH$_2$)$_7$C*H*$_2$O), 3.76 (t, 2H, *J* = 4.4 Hz, POCH$_2$C*H*$_2$O), 3.51–3.38 (m, 2H, PCH$_2$C*H*$_2$), 3.30–3.22 (m, 4H, C*H*$_2$(CH$_2$)$_2$NH$_2$), 3.05 (t, 4H, *J* = 7.5 Hz, C*H*$_2$NH$_2$), 2.61–2.42 (m, 2H, PC*H*$_2$), 2.25–2.06 (m, 4H, C*H*$_2$CH$_2$NH$_2$), 1.74–1.61 (m, 2H, CH$_2$C*H*$_2$CH$_2$O), 1.29–1.33 (m, 12H, CH$_3$(C*H*$_2$)$_6$, 0.96–0.84 (m, 3H, C*H*$_3$).

^{13}C NMR (101 MHz, CD_3OD): 139.36, 129.56, 129.05, 128.96 (*C*$_{Ph}$), 74.06 (*C*H$_2$Ph), 70.36 (d, *J* = 5.4 Hz, POCH$_2$*C*H$_2$O), 68.13 (d, *J* = 7.0 Hz, (CH$_2$)$_6$*C*H$_2$O), 67.35 (d, *J* = 6.7 Hz, PO*C*H$_2$CH$_2$O), 51.07 (*C*H$_2$(CH$_2$)$_2$NH$_2$), 48.65 (PCH$_2$*C*H$_2$), 37.86 (*C*H$_2$NH$_2$), 33.03 (CH$_3$CH$_2$*C*H$_2$), 31.52 (d, *J* = 6.1 Hz, CH$_2$*C*H$_2$CH$_2$O), 30.62, 30.39, 30.29, 26.55 ((*C*H$_2$)$_4$(CH$_2$)$_2$O), 23.72 (CH$_3$*C*H$_2$), 23.29 (*C*H$_2$CH$_2$NH$_2$), 21.29 (*J* = 142 Hz, P*C*H$_2$), 14.43 (*C*H$_3$).

^{31}P{^{1}H} NMR (162 MHz, CD_3OD): 29.87.

IR ν_{max} (KBr) 3700–3000 (s, br), 2956 (vs), 2925 (vs), 2855 (vs), 2700–2500 (m, br), 1604 (m), 1544 (w), 1496 (m), 1467 (m), 1456 (m), 1337 (w), 1226 (s), 1100–100 (vs), 1050 (vs), 1026 (vs), 1004 (s), 902 (w), 878 (w), 733 (m), 697 (m), 556 (w).

HR-MS(ESI$^+$): For $C_{26}H_{51}N_3O_6P$ $(M+H)^+$ *m*/*z* calculated 500.36117, found 500.36134.

^{1}H NMR spectrum of compound **10h**

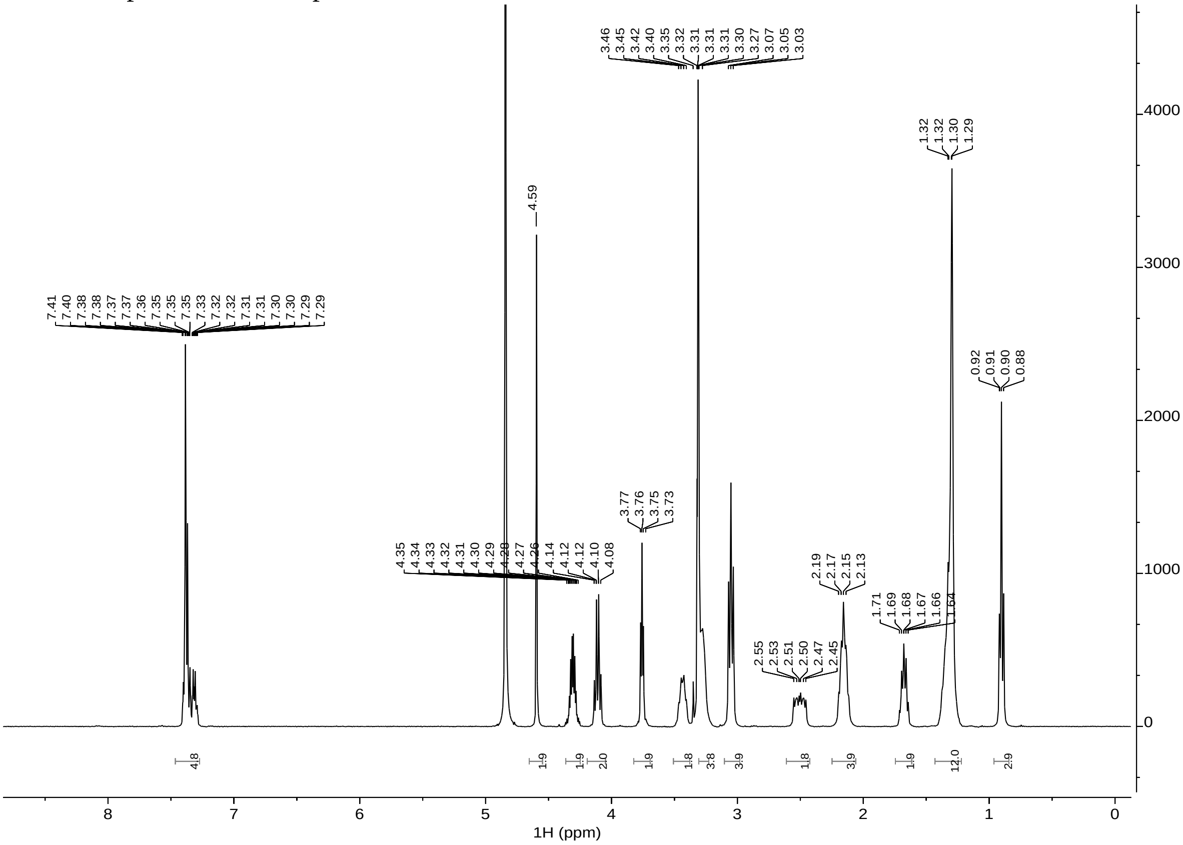

^{13}C NMR spectrum of compound **10h**

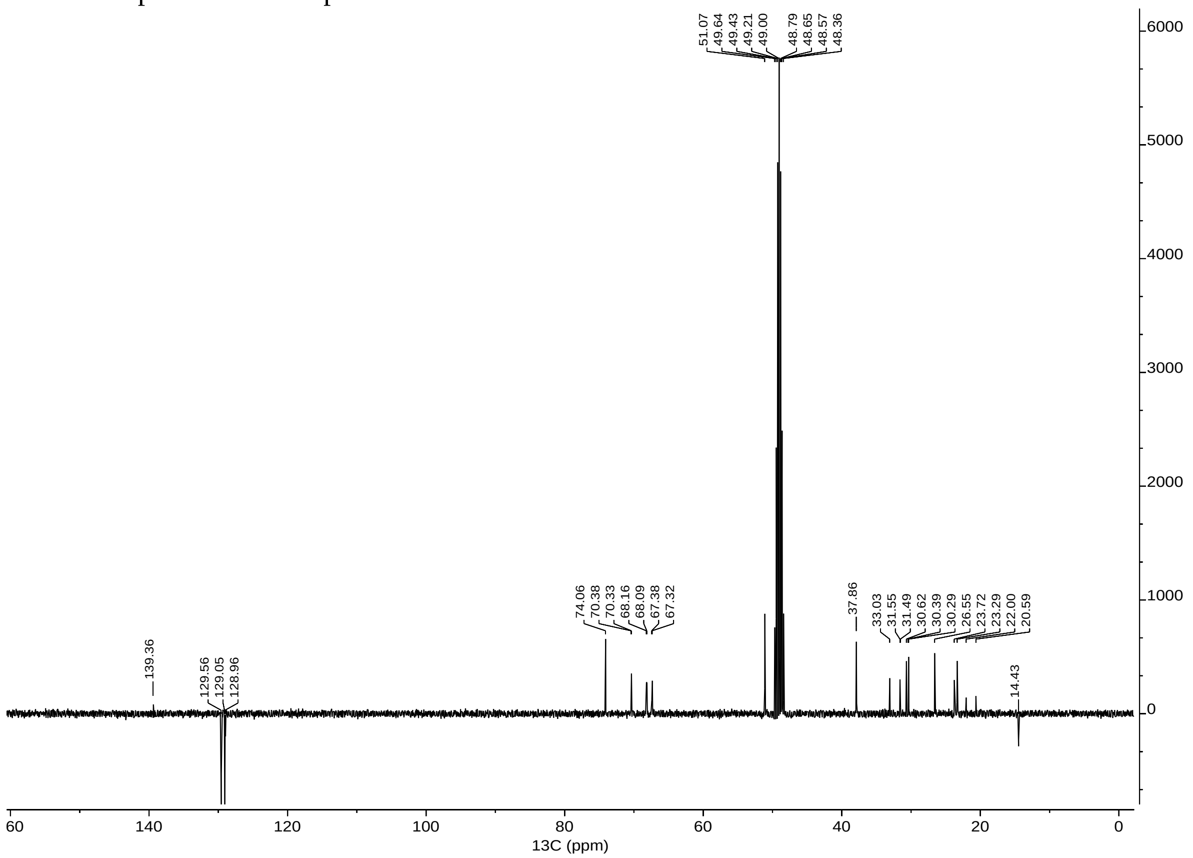

^{31}P{^{1}H} NMR spectrum of compound **10h**

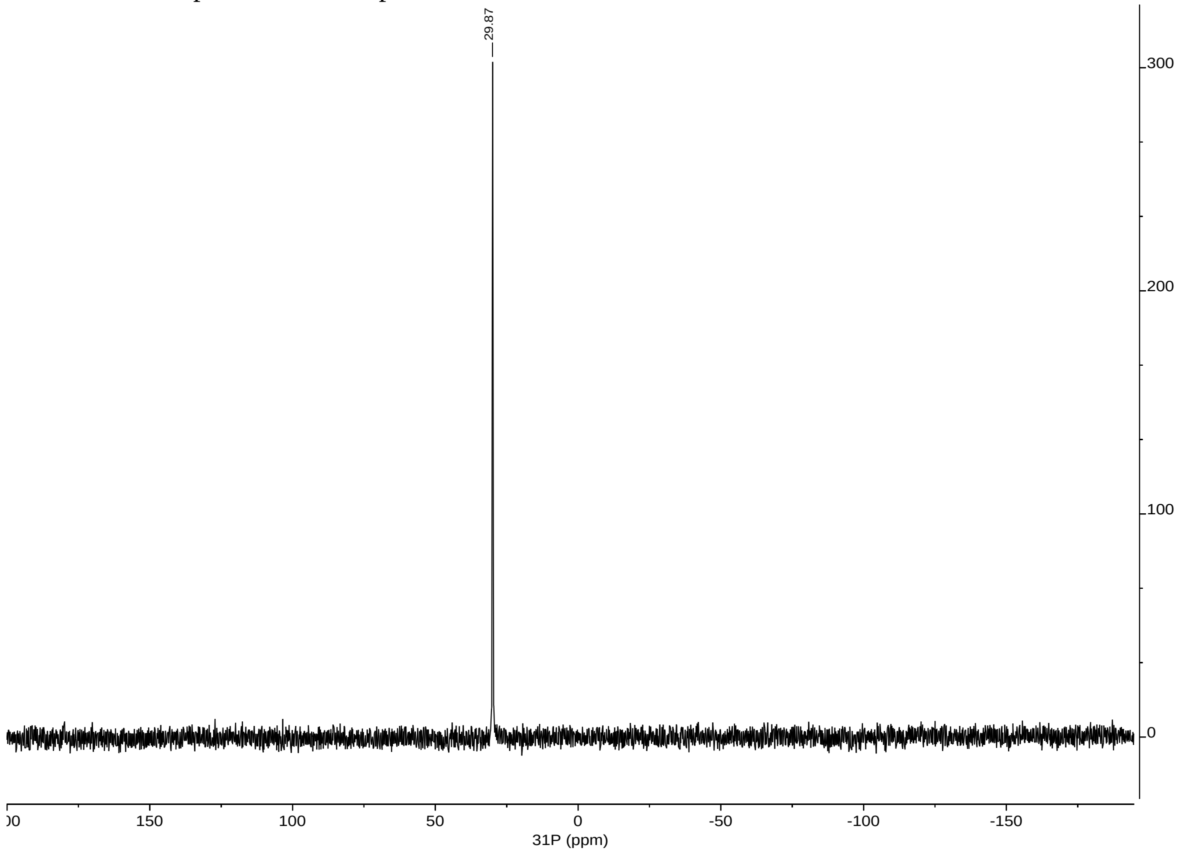

LC-MS chromatograms for compound **10h**

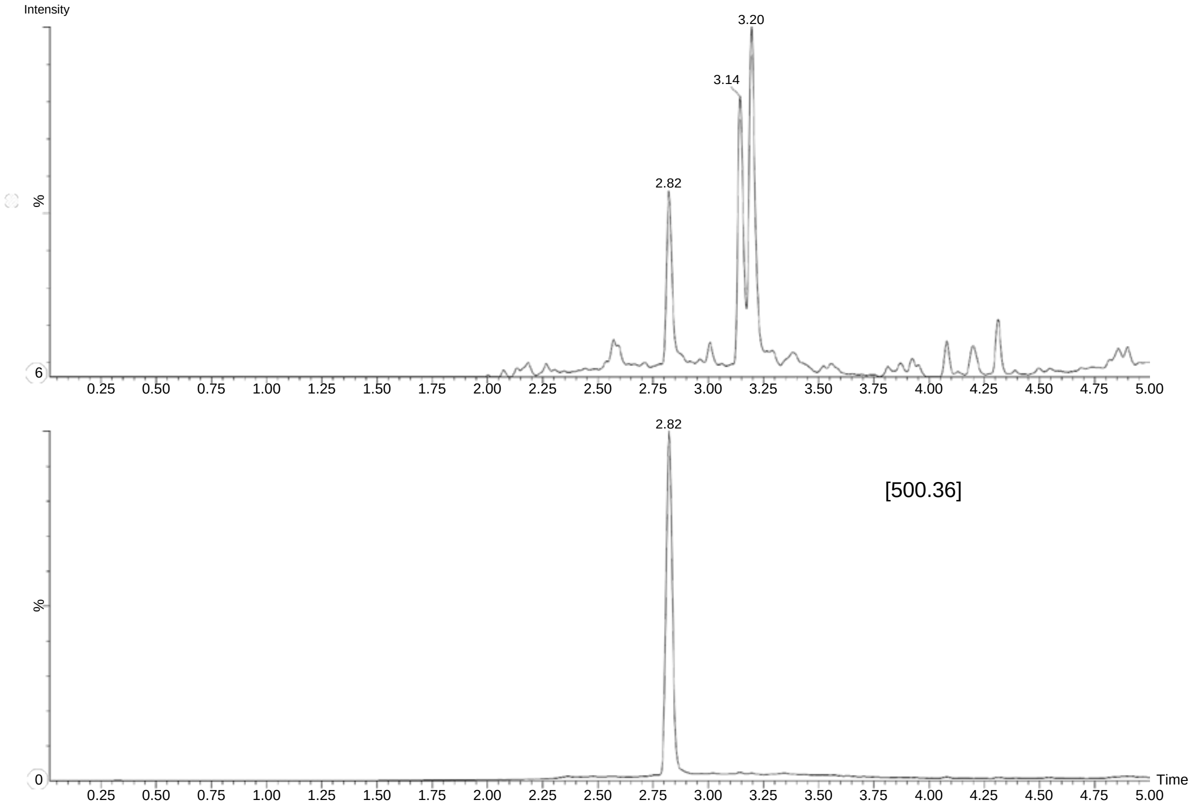

4-Chlorophenethyl nonyl (2-(bis(3-aminopropyl)amino)ethyl)phosphonate trihydrochloride (10i)

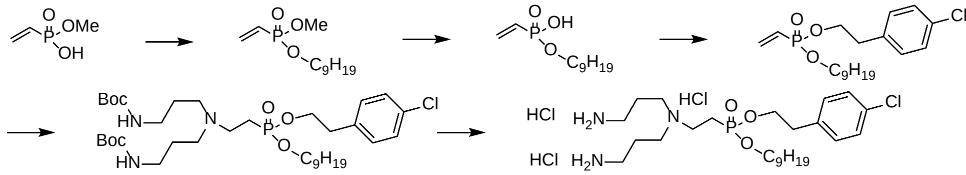

The title compound was prepared according to general methods **A**, **B2**, **D** and **E** from mono methyl vinylphosphonate (1.24 g, 9.14 mmol) in 11% overall yield (0.59 g, 0.96 mmol) as a white solid.

^{1}H NMR (400 MHz, CD_3OD): 7.36–7.32 (m, 2H, *m*-Ph*H*), 7.32–7.28 (m, 2H, *o*-Ph*H*), 4.39–4.29 (m, 2H, CH_2CH_2PhCl), 4.05–3.88 (m, 2H, $(CH_2)_7CH_2O$), 3.40–3.31 (m, 6H, CH_2N), 3.09 (t, 4H, J = 7.5 Hz, CH_2NH_2), 3.02 (t, 2H, J = 6.4 Hz, CH_2PhCl), 2.54–2.38 (m, 2H, PCH_2), 2.26–2.10 (m, 4H, $CH_2CH_2NH_2$), 1.69–1.56 (m, 2H, $CH_2CH_2CH_2O$), 1.39–1.24 (m, 12H, $CH_3(CH_2)_6$), 0.96–0.85 (m, 3H, CH_3).

^{13}C NMR (101 MHz, CD_3OD): 137.74 (CH_2C_{quat}), 133.61 ($C_{quat}Cl$), 131.90 (*o*-C_{Ph}), 129.60 (*m*-C_{Ph}), 68.28 (d, J = 6.6 Hz, CH_2CH_2PhCl), 68.15 (d, J = 6.7 Hz, $(CH_2)_7CH_2O$), 51.07 ($CH_2(CH_2)_2NH_2$), 48.66 (PCH_2CH_2), 37.87 (CH_2NH_2), 36.96 (d, J = 6.4 Hz, $ClPhCH_2$), 33.04 ($CH_3CH_2CH_2$), 31.52 (d, J = 6.0 Hz, $CH_2CH_2CH_2O$), 30.64, 30.39, 30.29, 26.53 ($(CH_2)_4(CH_2)_2O$), 23.72 (CH_3CH_2), 23.30 ($CH_2CH_2NH_2$), 21.18 (d, J = 140.5 Hz, PCH_2), 14.43 (CH_3).

^{31}P{^{1}H} NMR (162 MHz, CD_3OD): 28.29.

IR ν_{max} (KBr) 3200–2500 (vs, br), 2959 (vs), 2927 (vs), 2856 (s), 1598 (w), 1469 (m), 1493 (m), 1231 (w), 1190 (w, sh), 1107 (w), 1091 (w), 1051 (m), 1009 (m), 821 (w), 723 (w).

HR-MS(ESI$^+$): For $C_{25}H_{48}N_3O_3ClP$ $(M+H)^+$ m/z calculated 504.31163, found 504.31108.

^{1}H NMR spectrum of compound **10i**

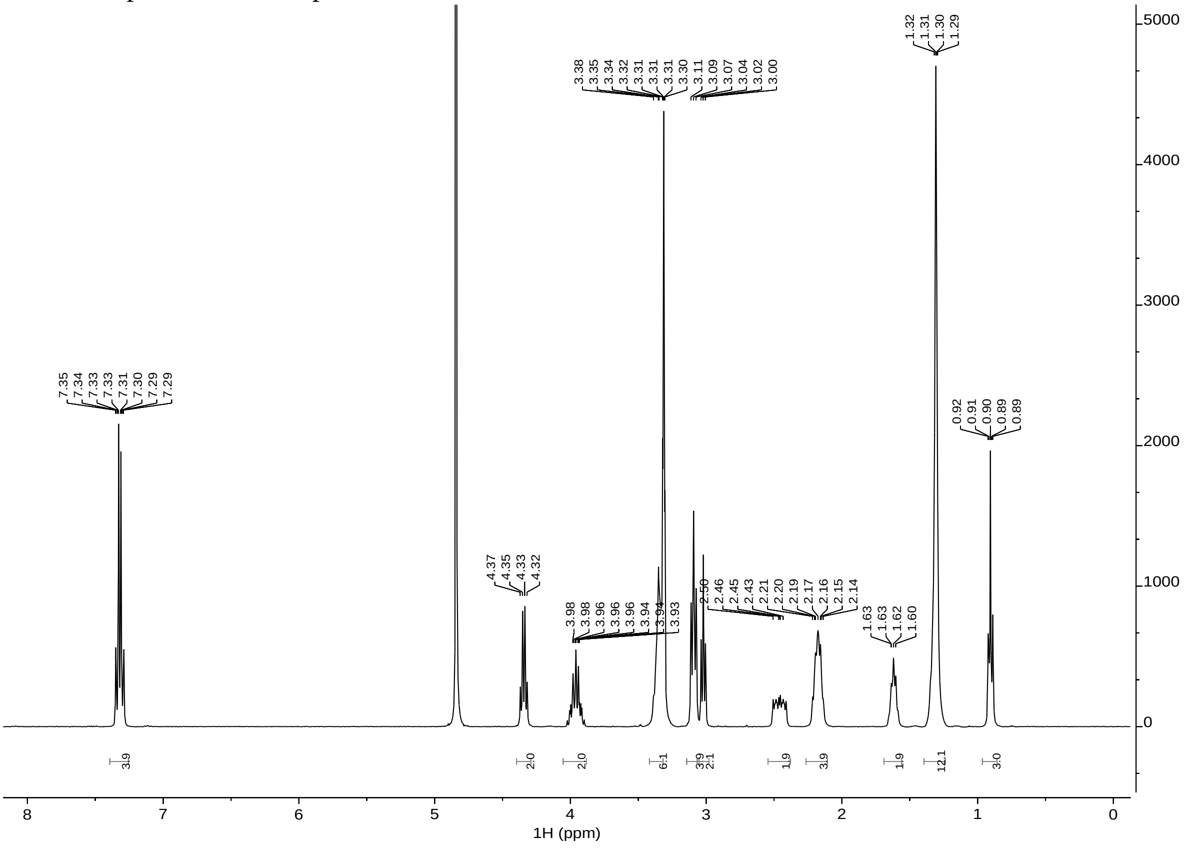

^{13}C NMR spectrum of compound **10i**

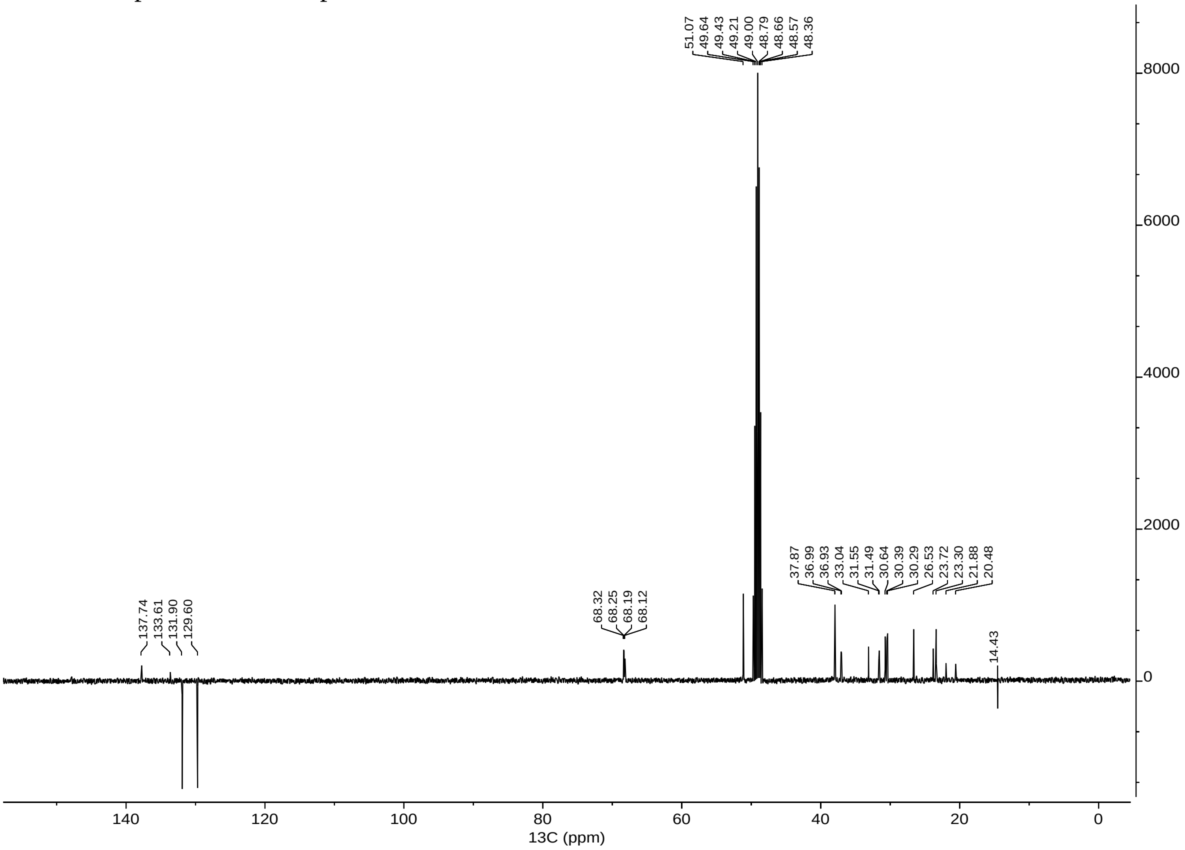

^{31}P{^{1}H} NMR spectrum of compound **10i**

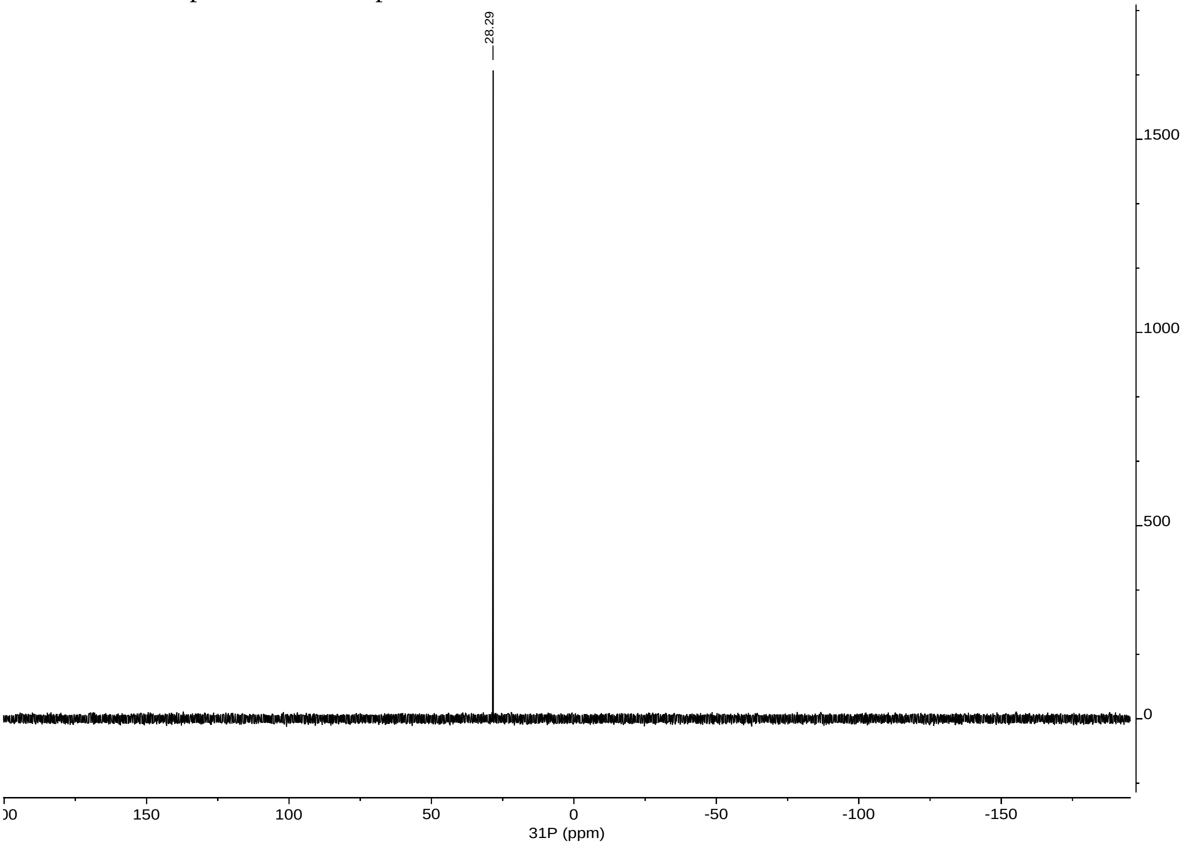

LC-MS chromatograms for compound **10i**

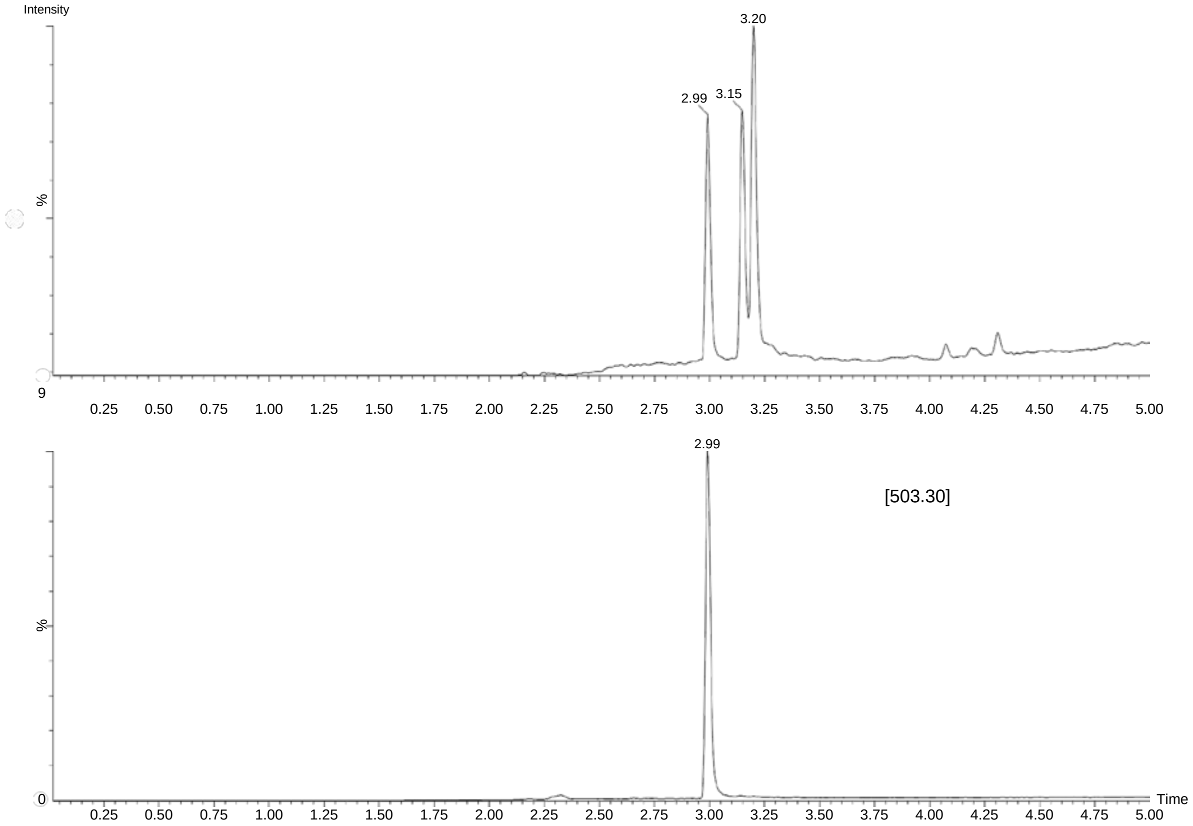

4-Aminophenethyl nonyl (2-(bis(3-aminopropyl)amino)ethyl)phosphonate tetrahydrochloride (10j)

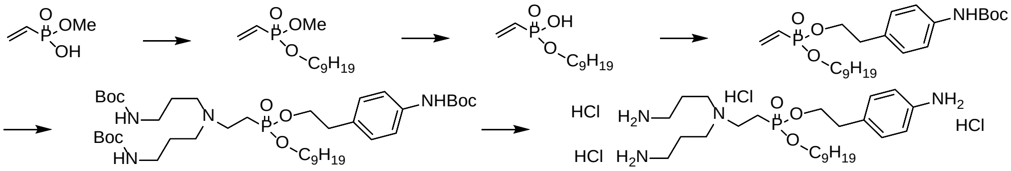

The title compound was prepared according to general methods **A**, **B2**, **D** and **E** from mono methyl vinylphosphonate (2.05 g, 15.1 mmol) in 9% overall yield (0.86 g, 1.36 mmol) as a white solid.

^{1}H NMR (401 MHz, CD_3OD): 7.54–7.47 (m, 2H, *o*-Ph*H*), 7.42–7.35 (m, 2H, *m*-Ph*H*), 4.45–4.29 (m, 2H, $H_2NPhCH_2CH_2O$), 4.11–3.97 (m, 2H, $(CH_2)_7CH_2O$), 3.40–3.32 (m, 6H, CH_2N), 3.15–3.05 (m, 6H, CH_2NH_2, H_2NPhCH_2), 2.57–2.42 (m, 2H, PCH_2), 2.25–2.13 (m, 4H, $CH_2CH_2NH_2$), 1.73–1.61 (m, 2H, $CH_2CH_2CH_2O$), 1.44–1.23 (m, 12H, $CH_3(CH_2)_6$), 0.94–0.85 (m, 3H, CH_3).

^{13}C NMR (101 MHz, CD_3OD): 140.07 ($C_{quat}NH_2$), 132.03 (*o*-C_{Ph}**)**, 130.85 (CH_2C_{quat}), 124.18 (*m*-C_{Ph}), 68.22 (d, J = 6.8 Hz, $(CH_2)_7CH_2O$), 68.02 (d, J = 6.6 Hz, $H_2NPhCH_2CH_2$), 51.00 ($CH_2(CH_2)_2NH_2$), 48.64 (PCH_2CH_2), 37.89 (CH_2NH_2), 37.07 (d, J = 6.5 Hz, H_2NPhCH_2), 33.03 ($CH_3CH_2CH_2$), 31.54 (d, J = 6.0 Hz, $CH_2CH_2CH_2O$), 30.64, 30.41, 30.30, 26.59 ($(CH_2)_4(CH_2)_2O$), 23.73 (CH_3CH_2), 23.28 ($CH_2CH_2NH_2$), 21.22 (d, J = 140.2 Hz, PCH_2), 14.44 (CH_3).

^{31}P{^{1}H} NMR (162 MHz, CD_3OD): 28.82.

IR ν_{max} (KBr) 3434 (m), 3100–2500 (m), 2956 (m), 2929 (m), 2857 (m), 1624 (w), 1513 (w), 1468 (w), 1226 (w), 1180 (w, sh), 1065 (w), 1007 (m), 824 (w), 558 (w).

HR-MS(ESI$^+$): For $C_{25}H_{50}N_4O_3P$ $(M+H)^+$ *m/z* calculated 485.36150, found 485.36085.

^{1}H NMR spectrum of compound **10j**

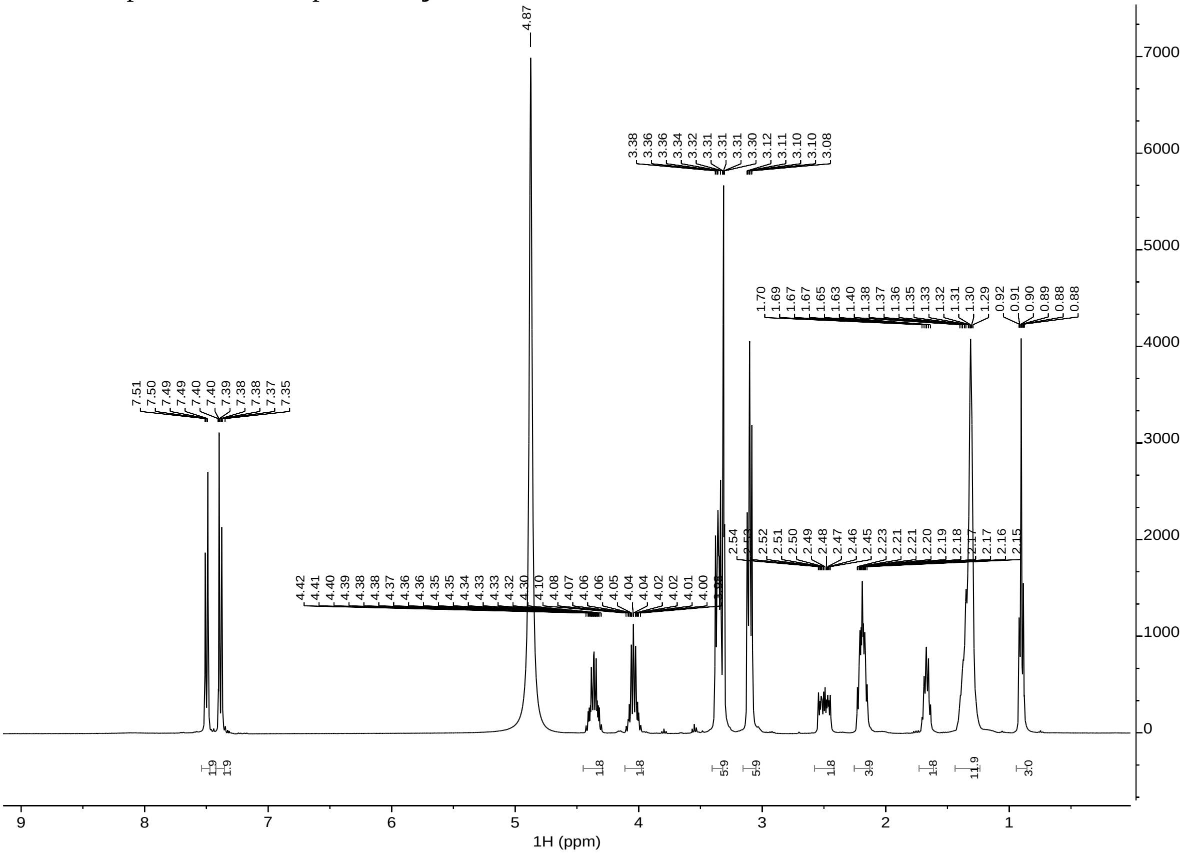

^{13}C NMR spectrum of compound **10j**

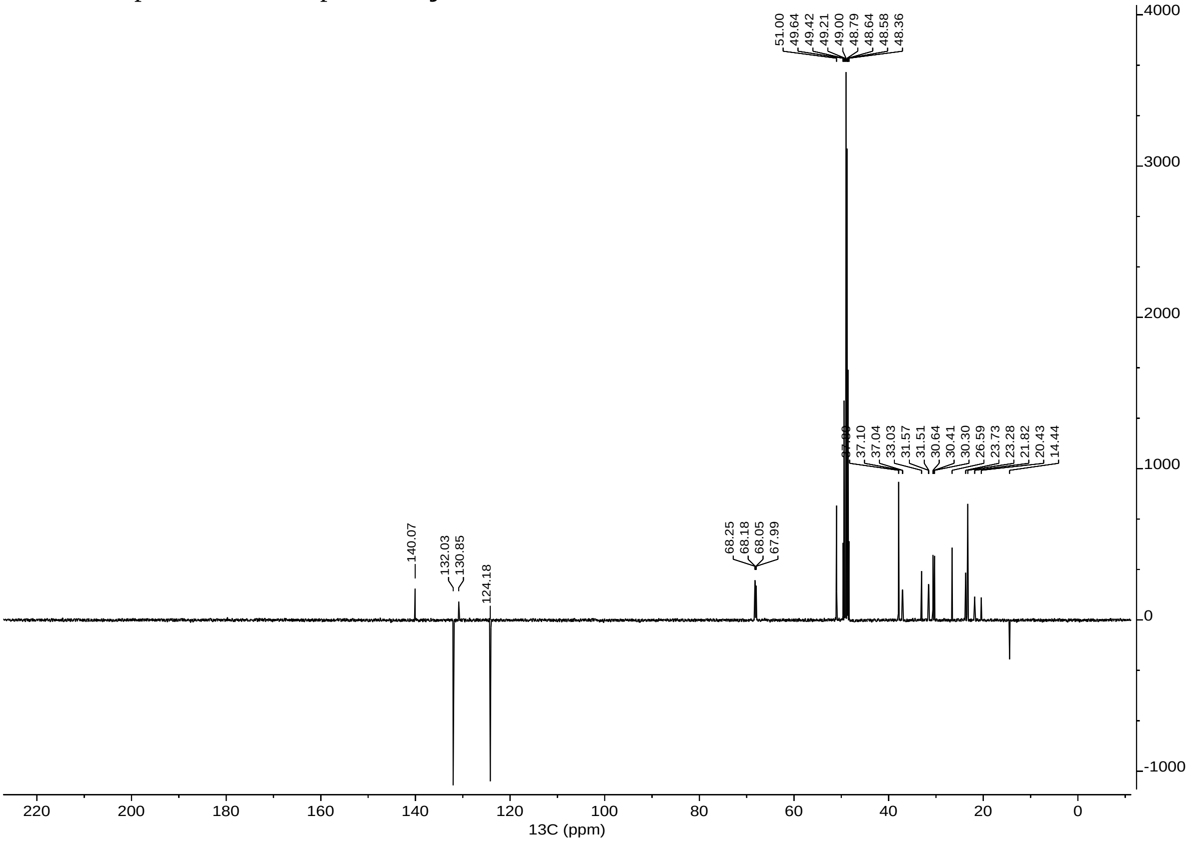

^{31}P{^{1}H} NMR spectrum of compound **10j**

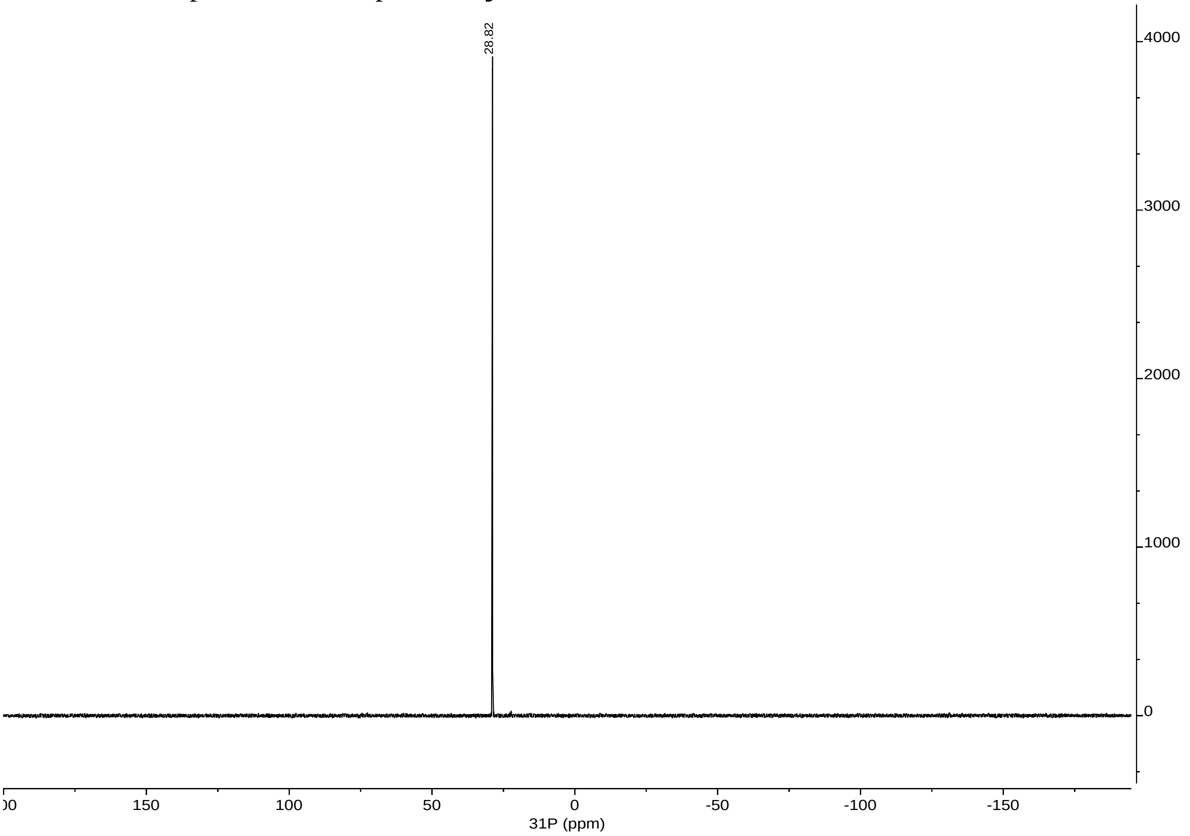

LC-MS chromatograms for compound **10j**

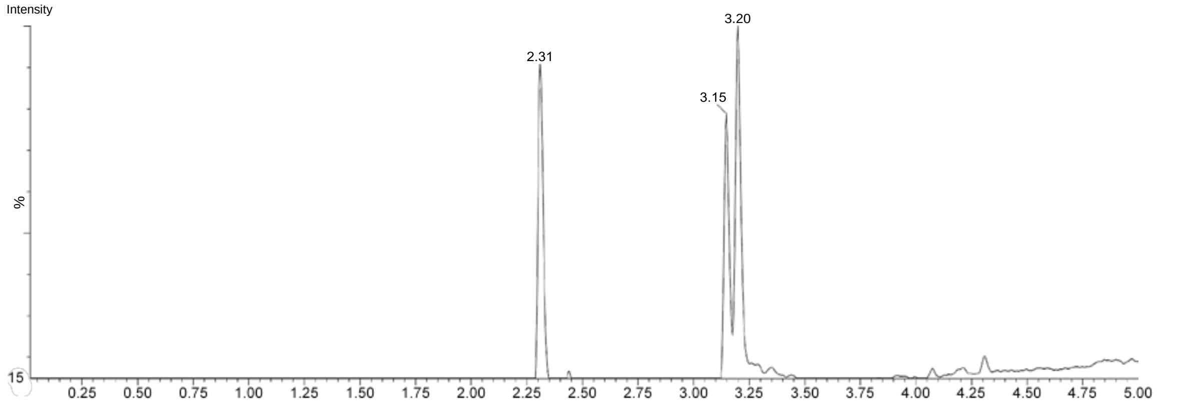

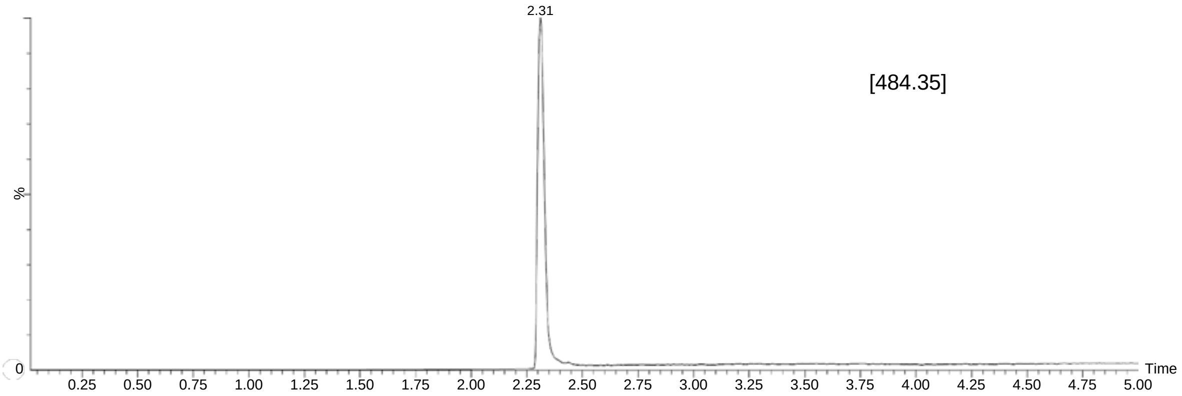

Nonyl phenethyl (2-(bis(3-aminopropyl)amino)ethyl)phosphonate trihydrochloride (10k)

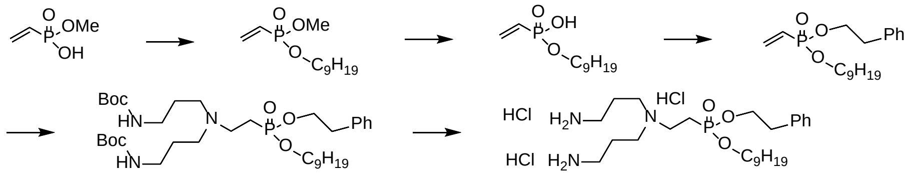

The title compound was prepared according to general methods **A**, **B2**, **D** and **E** from mono methyl vinylphosphonate (3.47 g, 25.5 mmol) in 11% yield (1.56 g, 2.73 mmol) as a white solid.

^{1}H NMR (400 MHz, CD_3OD): 7.46–7.16 (m, 5H, Ph*H*), 4.44–4.28 (m, 2H, CH_2CH_2Ph), 4.11–3.88 (m, 2H, $(CH_2)_7CH_2O$), 3.34–3.27 (m, 6H, CH_2N), 3.08 (t, 4H, J = 7.5 Hz, CH_2NH_2), 3.03 (t, 2H, J = 6.6 Hz, PhCH_2), 2.54–2.31 (m, 2H, PCH_2), 2.26–2.07 (m, 4H, $CH_2CH_2NH_2$), 1.71–1.53 (m, 2H, $CH_2CH_2CH_2O$), 1.44–1.23 (m, 12H, $CH_3(CH_2)_6$), 0.98–0.83 (m, 3H, CH_3).

^{13}C NMR (101 MHz, CD_3OD): 138.81, 130.22, 129.65, 127.84 (C_{Ph}), 68.54 (d, J = 6.7 Hz), 68.04 (d, J = 6.8 Hz, CH_2O), 51.01 ($CH_2(CH_2)_2NH_2$), 48.61 (PCH_2CH_2), 37.91 (CH_2NH_2), 37.71 (d, J = 6.3 Hz, PhCH_2), 33.03 ($CH_3CH_2CH_2$), 31.50 (d, J = 6.0 Hz, $CH_2CH_2CH_2O$), 30.62, 30.38, 30.26, 26.53 ($(CH_2)_4(CH_2)_2O$), 23.72 (CH_3CH_2), 23.31 ($CH_2CH_2NH_2$), 21.21 (d, J = 140.3 Hz, PCH_2), 14.44 (CH_3).

^{31}P{^{1}H} NMR (162 MHz, CD_3OD): 28.24.

IR ν_{max} (KBr) 3100–2600 (vs, br), 3026 (s, sh), 2958 (vs), 2926 (vs), 2856 (s), 1603 (w), 1587 (w, sh), 1500 (w, sh), 1468 (m), 1455 (m), 1230 (m), 1096 (w, sh), 1053 (m), 1010 (m), 881 (w), 815 (w), 751 (w), 724 (w), 700 (w), 573 (vw), 548 (vw), 489 (vw).

HR-MS(ESI$^+$): For $C_{25}H_{49}N_3O_3P$ $(M+H)^+$ m/z calculated 470.35061, found 470.35038.

^{1}H NMR spectrum of compound **10k**

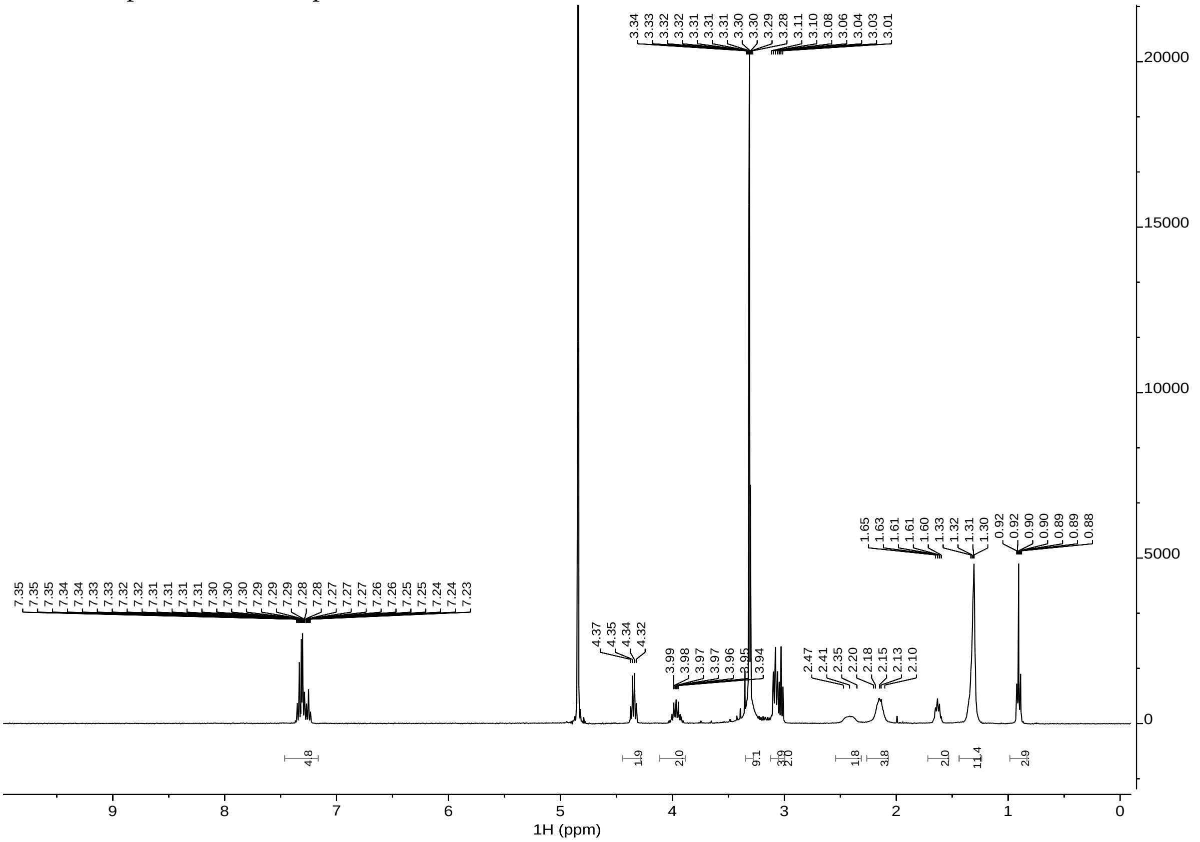

^{13}C NMR spectrum of compound **10k**

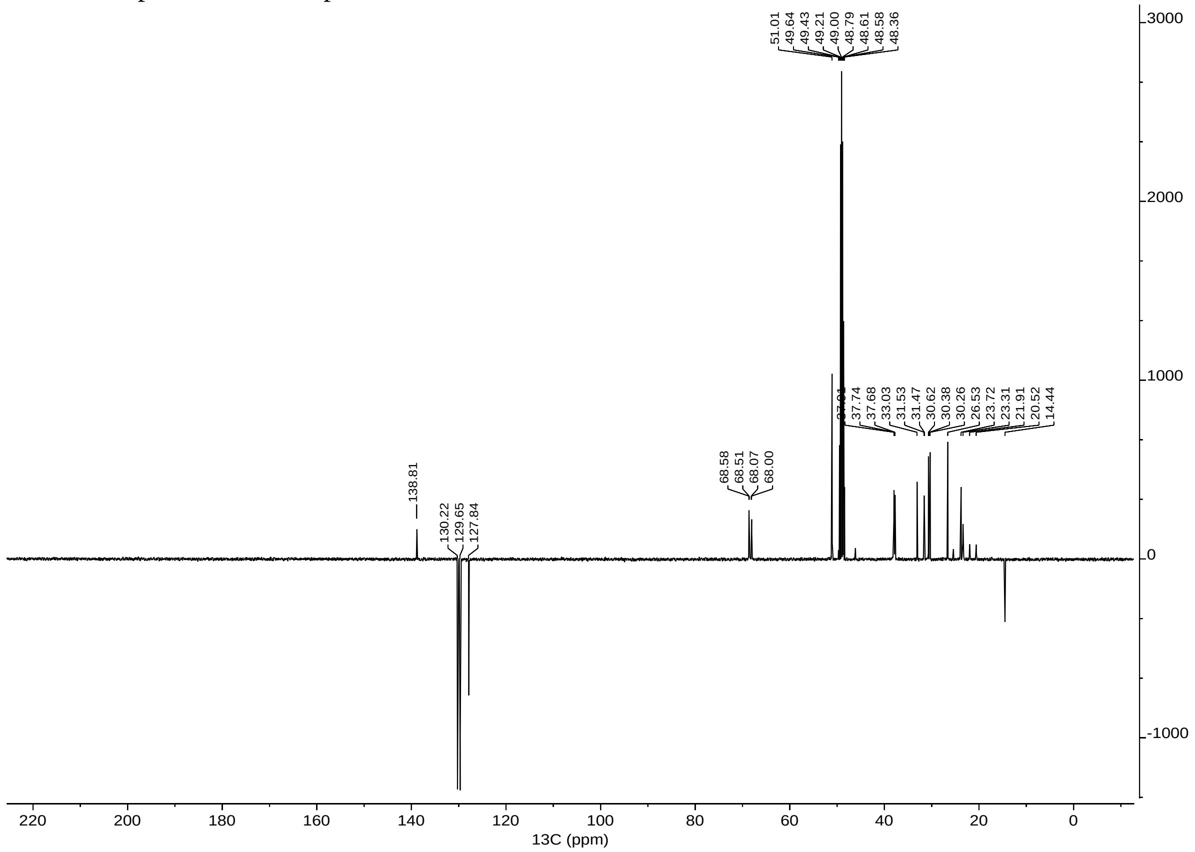

^{31}P{^{1}H} NMR spectrum of compound **10k**

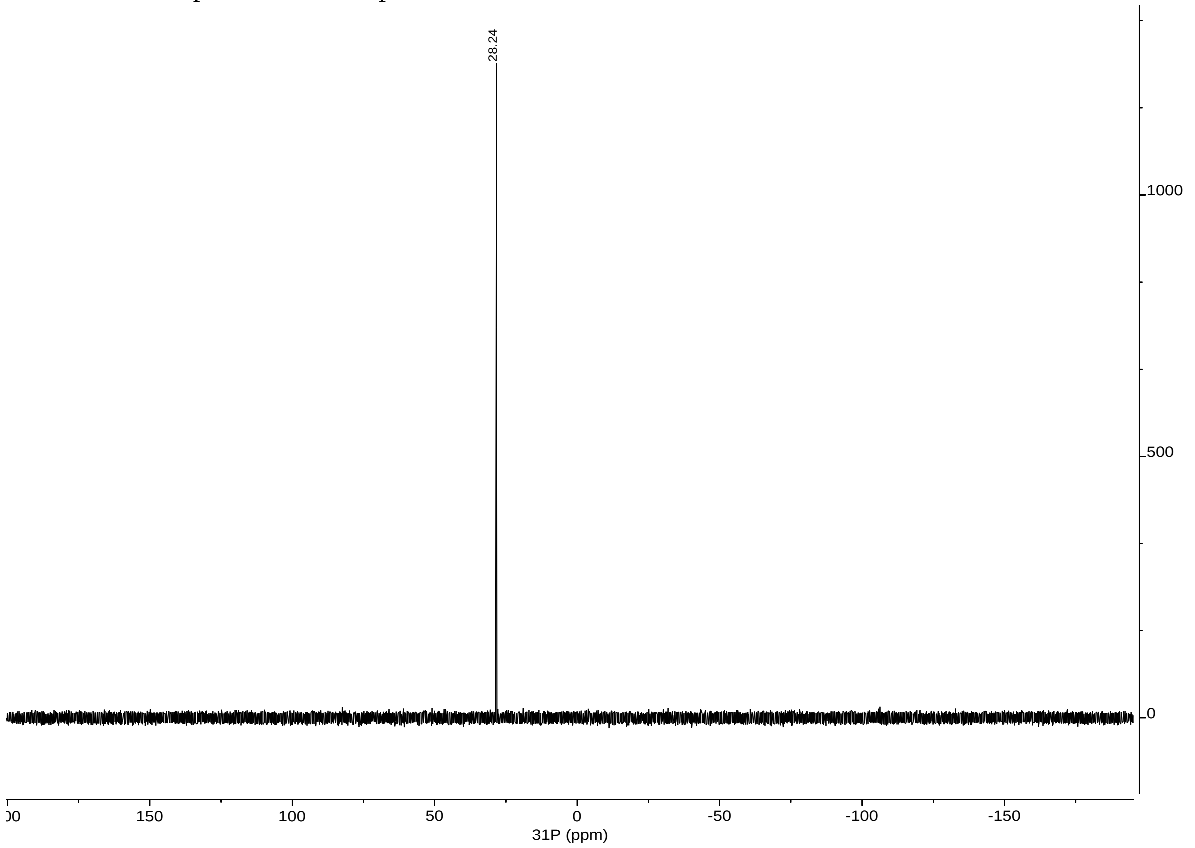

LC-MS chromatograms for compound **10k**

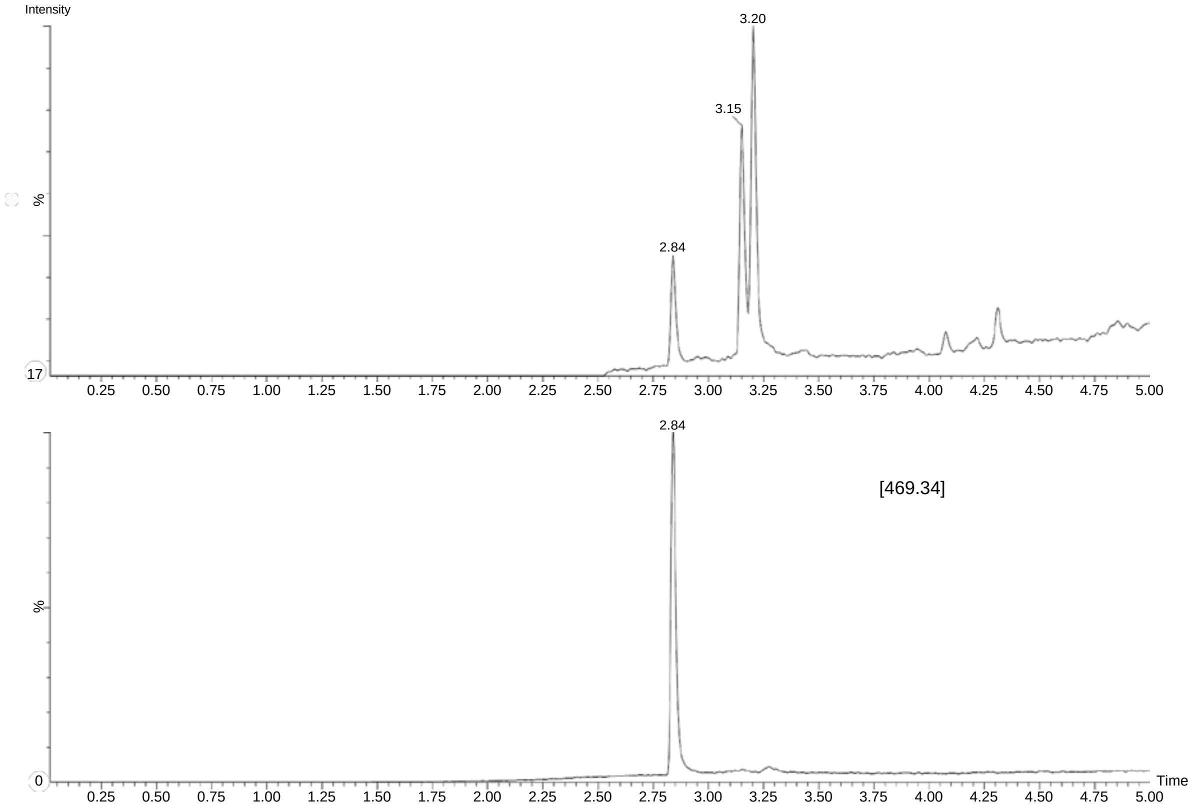

4-Nitrophenethyl nonyl (2-(bis(3-aminopropyl)amino)ethyl)phosphonate trihydrochloride (10l)

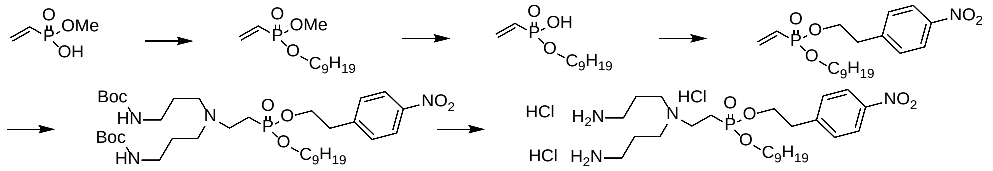

The title compound was prepared according to general methods **A**, **B2**, **D** and **E** from mono methyl vinylphosphonate (0.91 g, 6.68 mmol) in 9% overall yield (0.37 g, 0.59 mmol) as a white solid.

^{1}H NMR (400 MHz, CD_3OD): 8.32–8.15 (m, 2H, *m*-Ph*H*), 7.69–7.51 (m, 2H, *o*-Ph*H*), 4.54–4.34 (m, 2H, $O_2NPhCH_2CH_2$), 4.08–3.89 (m, 2H, $(CH_2)_7CH_2O$), 3.48–3.25 (m, 6H, CH_2N), 3.23–3.14 (m, 2H, O_2NPhCH_2), 3.14–3.01 (m, 4H, CH_2NH_2), 2.63–2.40 (m, 2H, PCH_2), 2.29–2.07 (m, 4H, $CH_2CH_2NH_2$), 1.70–1.51 (m, 2H, $CH_2CH_2CH_2O$), 1.43–1.17 (m, 12H, $CH_3(CH_2)_6$), 1.00–0.83 (m, 3H, CH_3).

^{13}C NMR (101 MHz, CD_3OD): 148.37 (O_2NC_{quat}), 147.08 (CH_2C_{quat}), 131.48 (*o*-C_{Ph}), 124.62 (*m*-C_{Ph}), 68.21 (d, J = 6.8 Hz, $(CH_2)_7CH_2O$), 67.67 (d, J = 6.5 Hz, $CH_2CH_2PhNO_2$), 51.03 ($CH_2(CH_2)_2NH_2$), 48.72 (PCH_2CH_2), 37.89 (CH_2NH_2), 37.33 (d, J = 6.5 Hz, CH_2PhNO_2), 33.01 ($CH_3CH_2CH_2$), 31.49 (d, J = 5.9 Hz, $CH_2CH_2CH_2O$), 30.61, 30.36, 30.26, 26.51 ($(CH_2)_4(CH_2)_2O$), 23.70 (CH_3CH_2), 23.28 ($CH_2CH_2NH_2$), 21.27 (d, J = 140.2 Hz, PCH_2), 14.42 (CH_3).

^{31}P{^{1}H} NMR (162 MHz, CD_3OD): 28.70.

IR ν_{max} (KBr) 3200–2700 (vs, br), 2958 (vs), 2927 (vs), 2856 (m), 1601 (m), 1521 (m), 1469 (m), 1347 (vs), 1230 (w), 1206 (w), 1180 (w, sh), 1109 (w), 1051 (w), 1010 (m), 857 (m).

HR-MS(ESI$^+$): For $C_{25}H_{49}N_4O_5P$ $(M+H)^+$ *m/z* calculated 515.33568, found 515.33523.

^{1}H NMR spectrum of compound **10l**

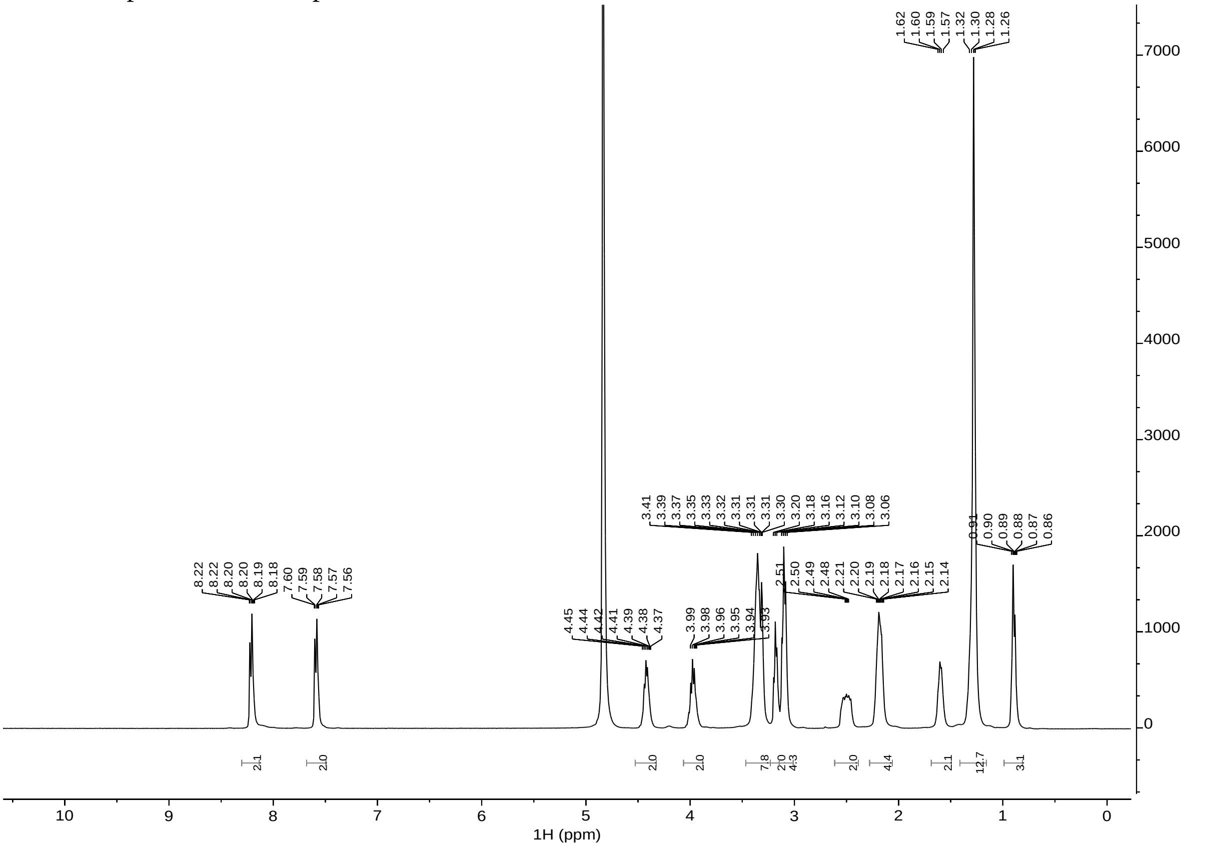

^{13}C NMR spectrum of compound **10l**

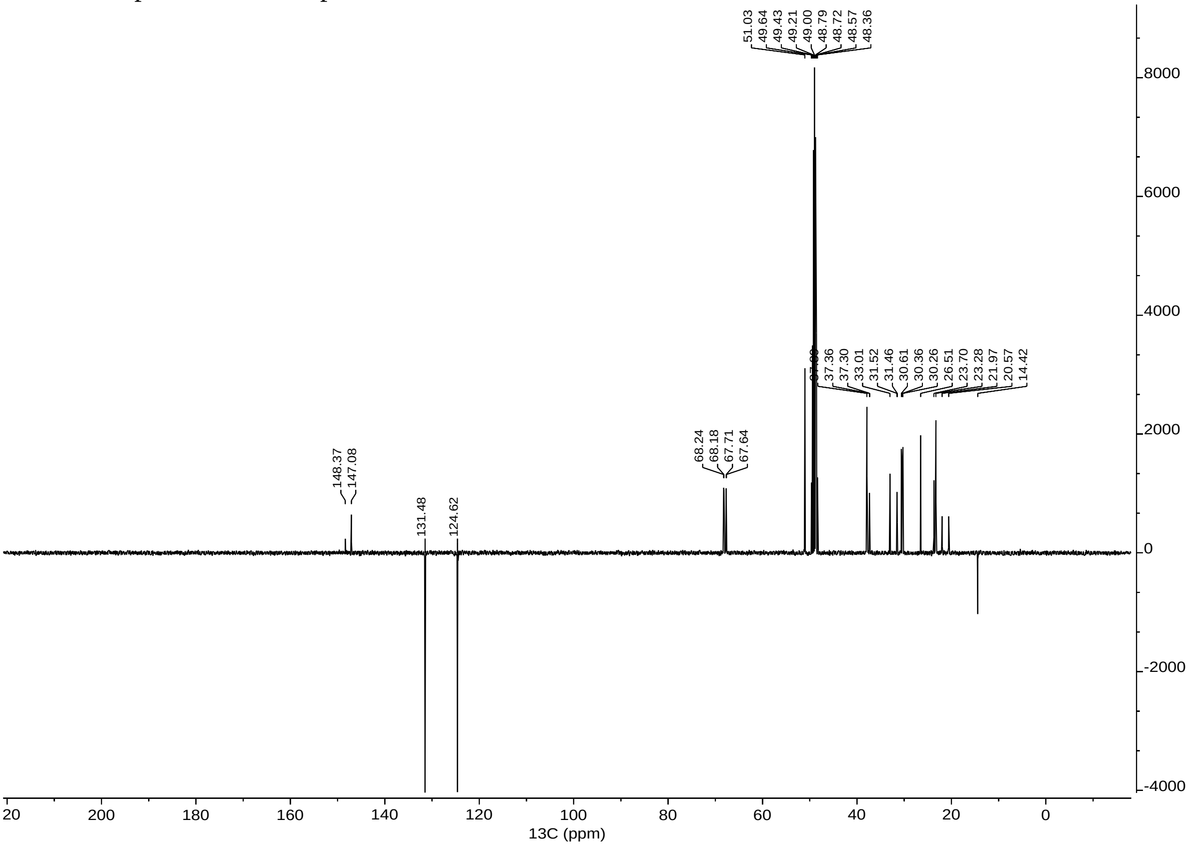

^{31}P{^{1}H} NMR spectrum of compound **10l**

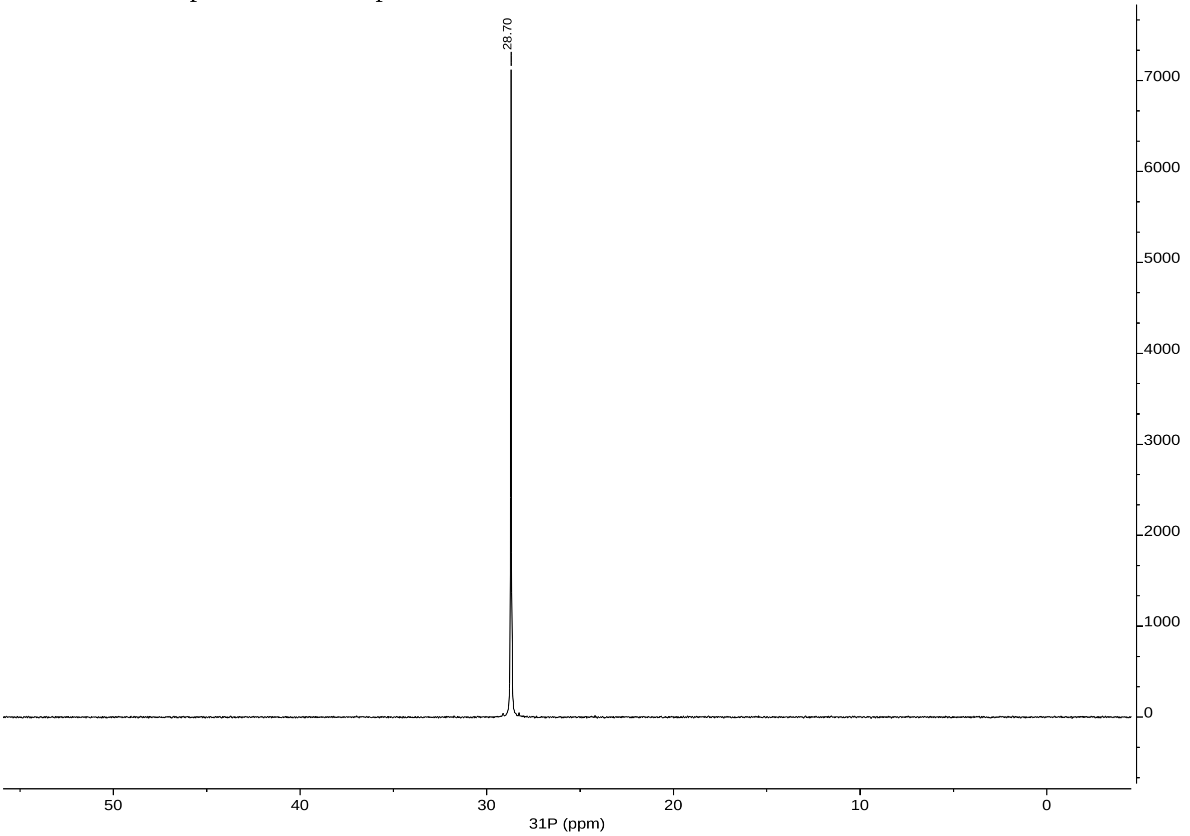

LC-MS chromatograms for compound **10l**

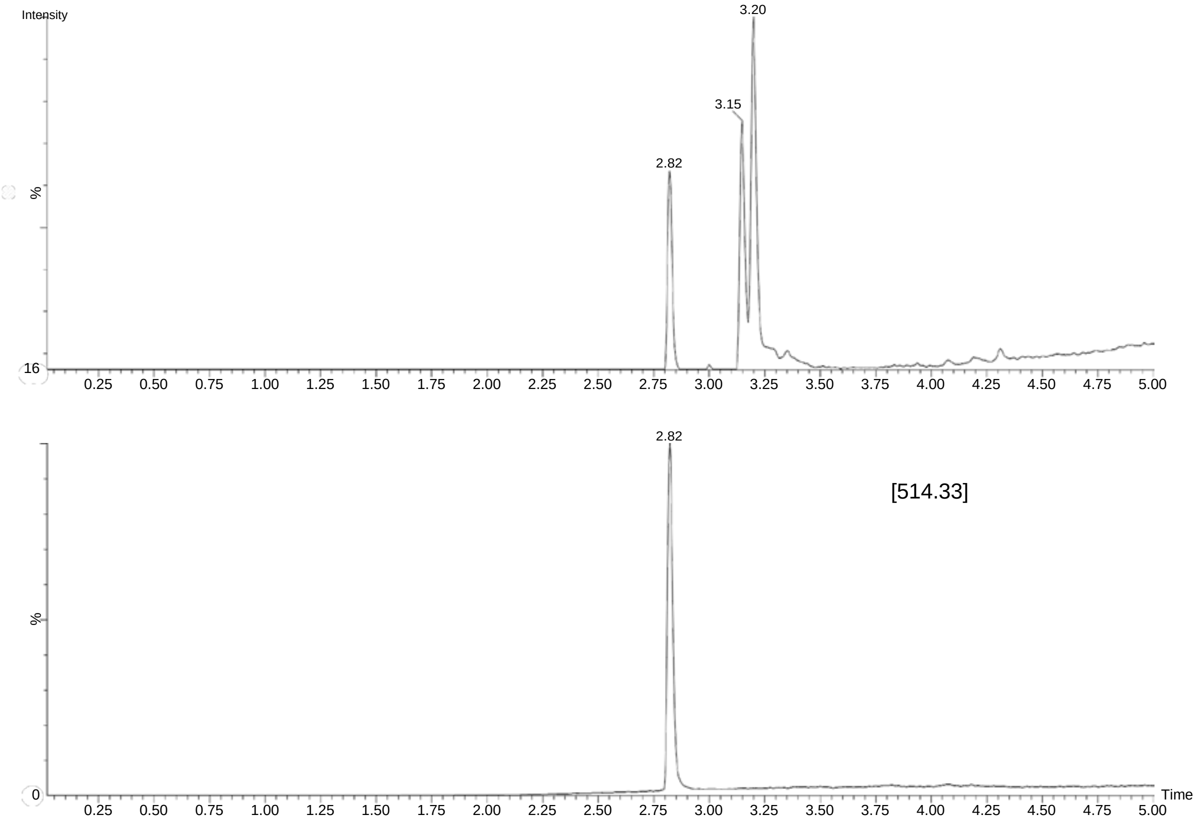

Didodecyl (2-(bis(3-aminopropyl)amino)ethyl)phosphonate trihydrochloride (10m)

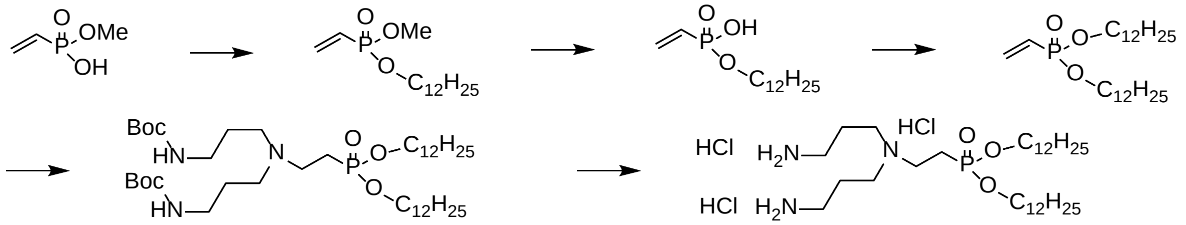

The title compound was prepared according to general methods **A**, **B1**, **D**, and **E** from mono methyl vinylphosphonate (0.97 g, 7.1 mmol) in 7% overall yield (0.35 g, 0.51 mmol) as a white solid.

^{1}H NMR (401 MHz, CD_3OD): 4.13 (dtd, *J* = 8.3, 6.6, 1.7 Hz, 4H, OC*H*$_2$), 3.50–3.42 (m, 2H, PCH_2CH_2), 3.38 (dd, *J* = 10.3, 6.3 Hz, 4H, C*H*$_2$(CH_2)$_2$$NH_2$), 3.10 (t, *J* = 7.5 Hz, 4H, C*H*$_2$$NH_2$), 2.58–2.46 (m, 2H, PC*H*$_2$), 2.26–2.13 (m, 4H, C*H*$_2$$CH_2NH_2$), 1.71 (dt, *J* = 8.3, 6.4 Hz, 4H, OCH_2C*H*$_2$), 1.48–1.22 (m, 36H, (C*H*$_2$)$_9$$CH_3$), 0.96–0.84 (m, 6H, C*H*$_3$).

^{13}C NMR (101 MHz, CD_3OD): 68.24 (d, *J* = 6.7 Hz, O*C*H$_2$), 51.03 (*C*H$_2$(CH_2)$_2$$NH_2$), 48.75 ($PCH_2$*C*H$_2$), 37.86 (*C*H$_2$$NH_2$), 33.09, 31.61 (d, *J* = 5.8 Hz, OCH_2*C*H$_2$), 30.82, 30.79, 30.76, 30.73, 30.50, 30.32, 26.65, 23.75, 23.26 (*C*H$_2$$CH_2NH_2$), 21.21 (d, *J* = 140.8 Hz, P*C*H$_2$), 14.46 (*C*H$_3$).

^{31}P{^{1}H} NMR (162 MHz, CD_3OD): 28.70.

IR ν_{max} (KBr) 3100–2500 (m, vbr), 2958 (s), 2922 (s), 2853 (s), 2740 (m, br), 2673 (m), 2615 (m, br), 2547 (m), 2030 (w, vbr), 1606 (m), 1543 (w), 1510 (w, sh), 1485 (s, sh), 1468 (s), 1400 (m, sh), 1379 (m), 1227 (s), 1071 (s, sh), 1030 (s, sh), 998 (vs), 721 (m).

HR-MS(ESI$^+$): For $C_{32}H_{71}N_3O_3P$ $(M+H)^+$ calculated 576.52276, found 576.52234.

^{1}H NMR spectrum of compound **10m**

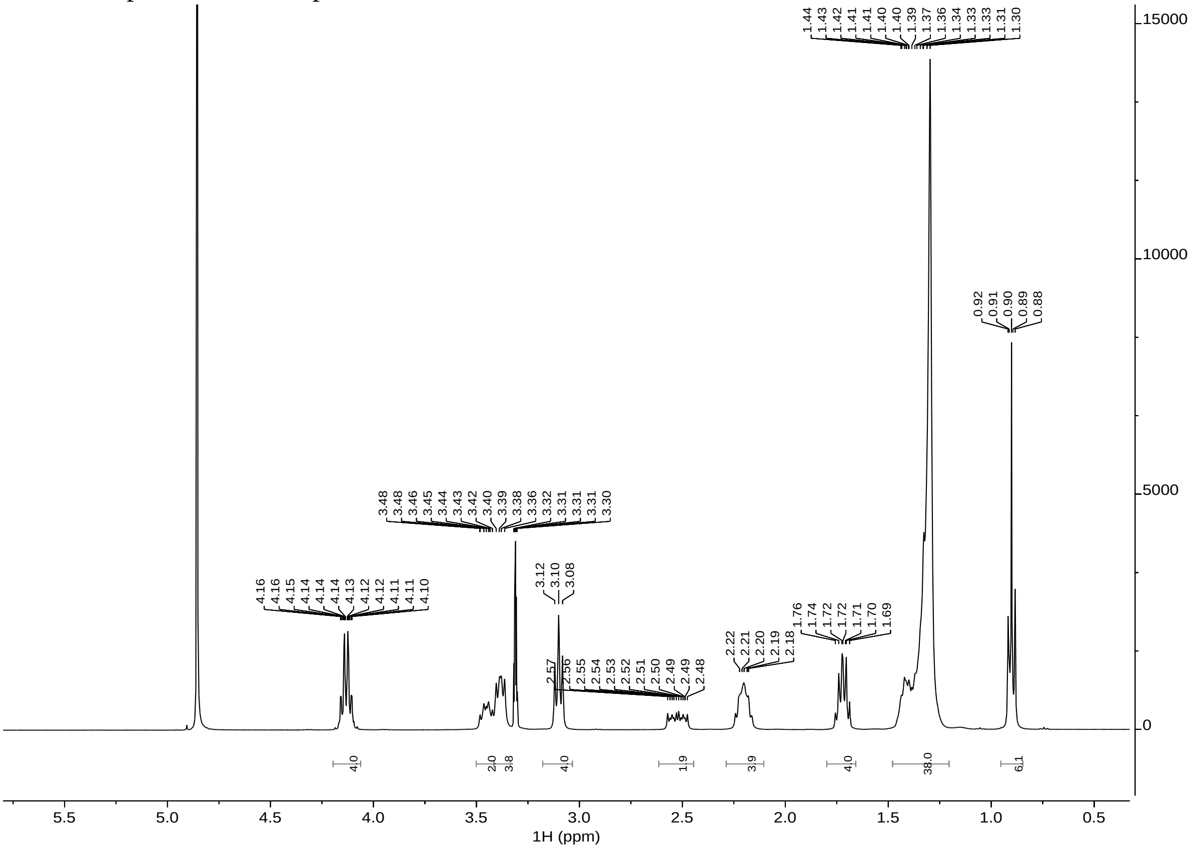

^{13}C NMR spectrum of compound **10m**

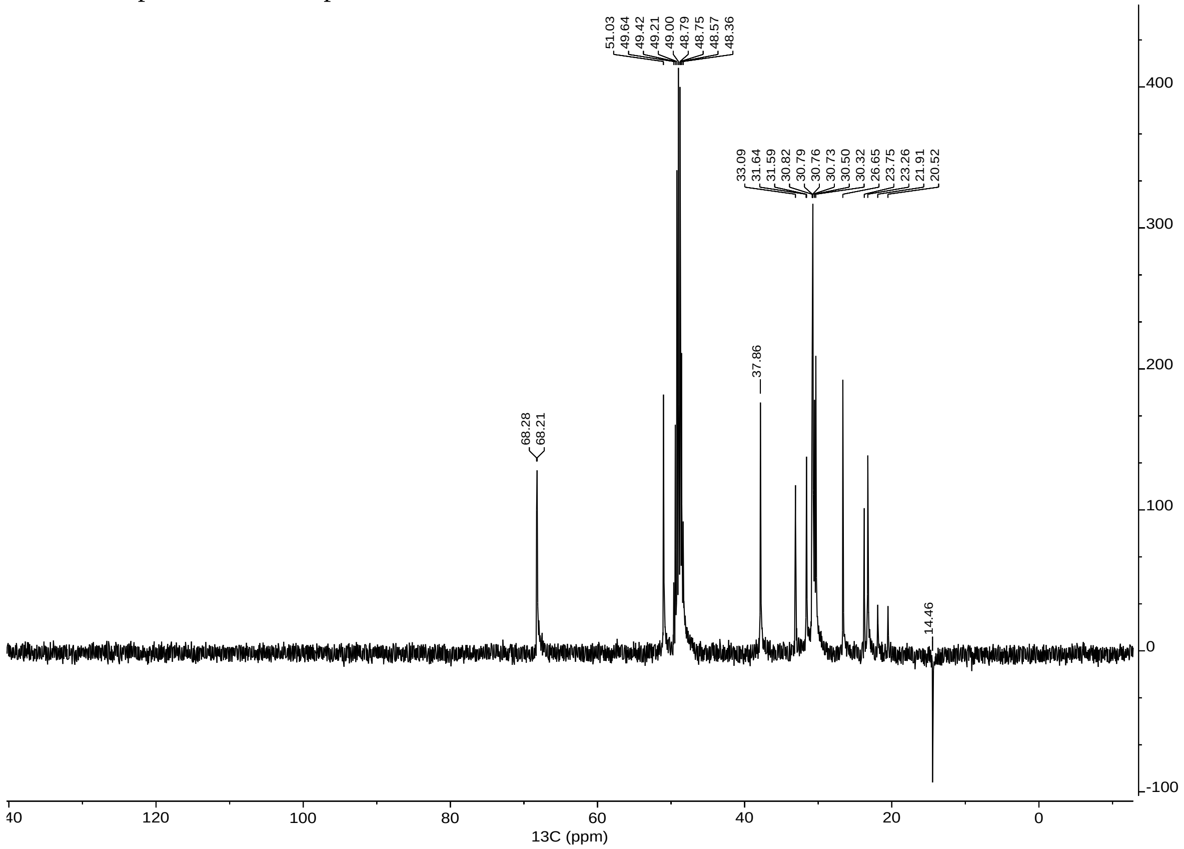

$^{31}P\{^1H\}$ NMR spectrum of compound **10m**

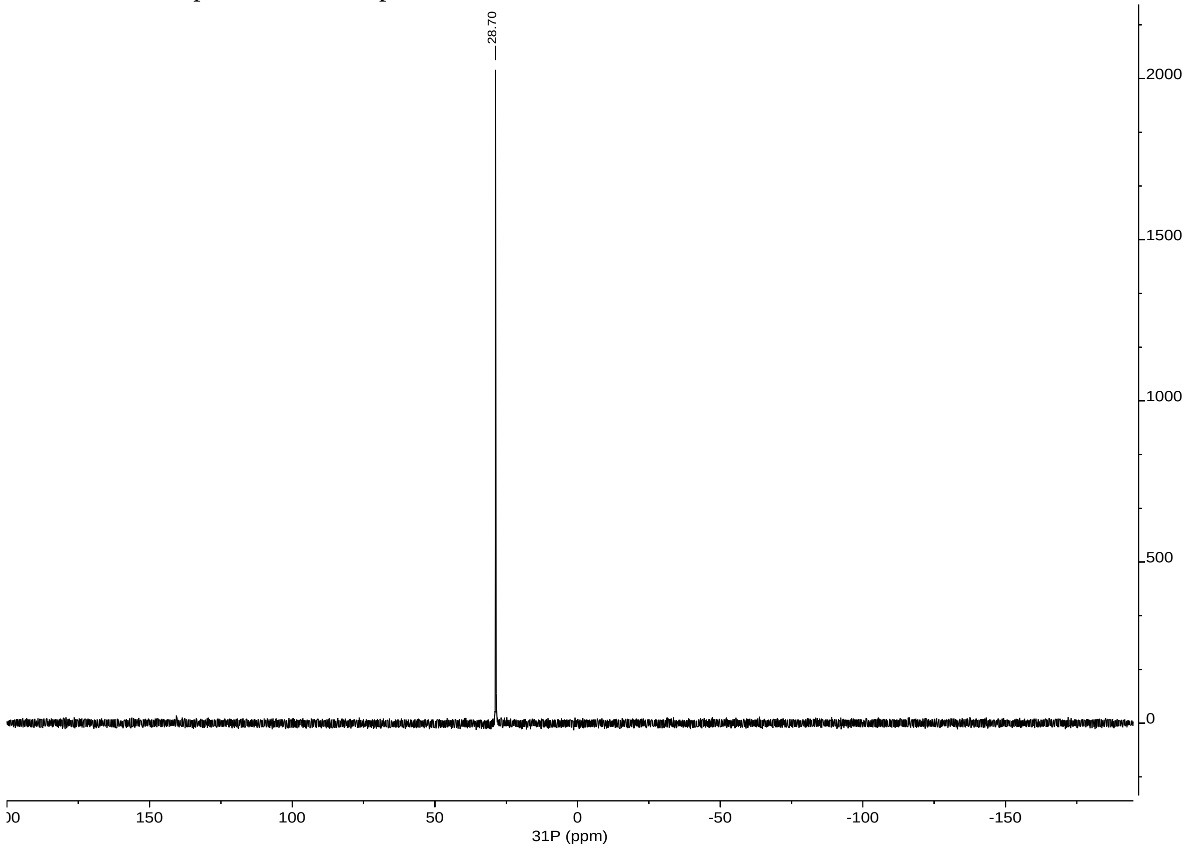

LC-MS chromatograms for compound **10m**

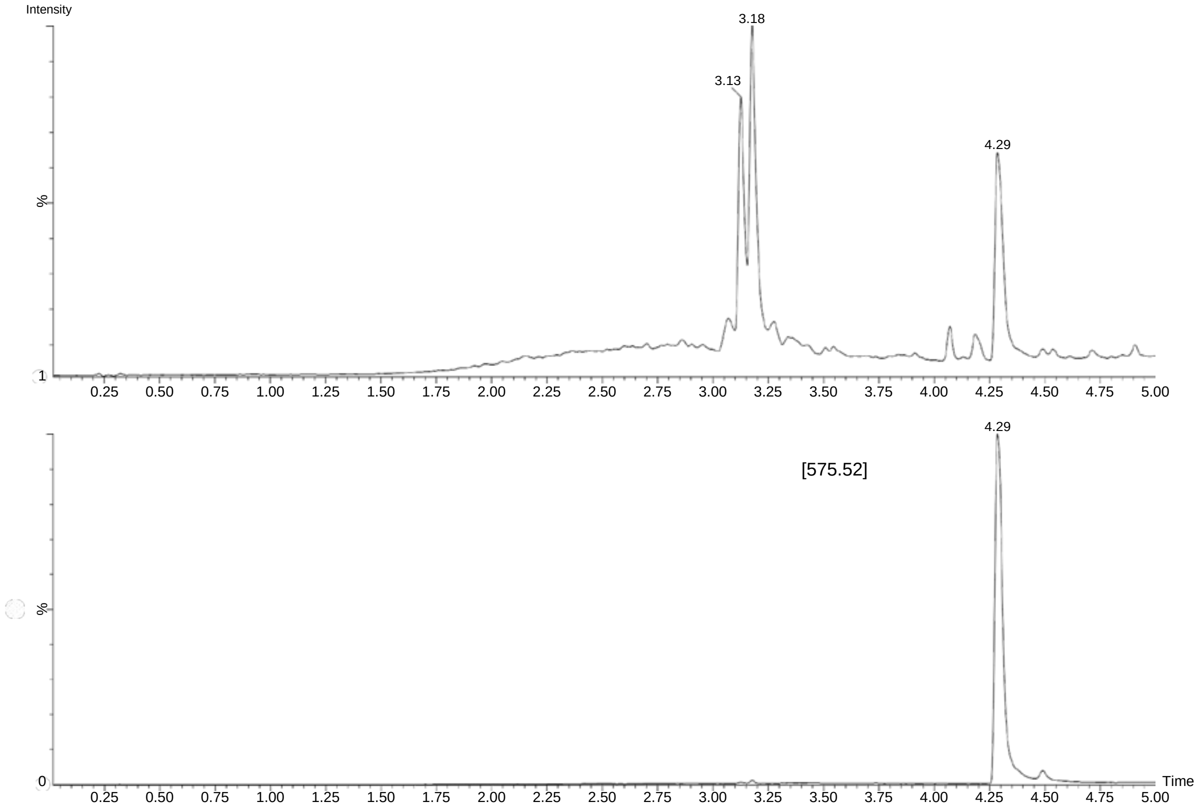

Dodecyl 2-(indol-3-yl)ethyl (2-(bis(3-aminopropyl)amino)ethyl)phosphonate tetrahydrochloride (10n)

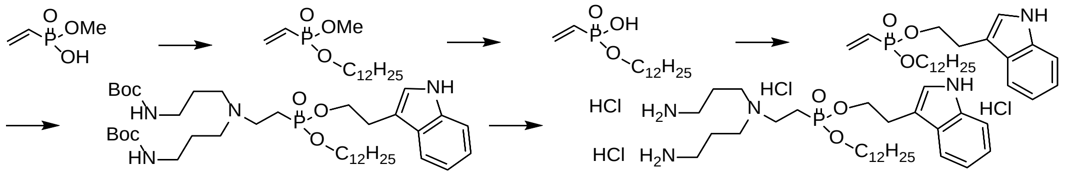

The title compound was prepared according to general methods **A**, **B2**, **D** and **E** from mono methyl vinylphosphonate (0.45 g, 3.32 mmol) in 22% overall yield (0.48 g, 0.73 mmol) as a white amorphous solid.

^{1}H NMR (401 MHz, CD_3OD): 7.60 (dt, *J* = 7.9, 1.1 Hz, 1H, C^4H), 7.40 (dt, *J* = 8.1, 0.9 Hz, 1H, C^7H), 7.19 (s, 1H, C^2H), 7.13 (ddd, *J* = 8.1, 7.0, 1.2 Hz, 1H, C^6H), 7.05 (ddd, *J* = 8.0, 7.0, 1.1 Hz, 1H, C^5H), 4.38 (qd, *J* = 6.6, 2.2 Hz, 2H, OCH_2CH_2Ar), 4.01–3.89 (m, 2H, $OCH_2C_{11}H_{23}$), 3.20–3.08 (m, 8H, OCH_2CH_2Ar, NCH_2), 3.03 (t, *J* = 7.5 Hz, 4H, CH_2NH_2), 2.38–2.26 (m, 2H, PCH_2), 2.13–2.03 (m, 4H, $CH_2CH_2NH_2$), 1.59 (p, *J* = 6.7 Hz, 2H, $CH_2C_{10}H_{21}$), 1.28 (s, 18H, $(CH_2)_9CH_3$), 0.92–0.86 (m, 3H, CH_3).

^{13}C NMR (101 MHz, CD_3OD): 138.02 (C^{7a}), 128.79 (C^{3a}), 124.44 (C^2), 122.58 (C^6), 119.96 (C^5), 119.42 (C^7), 112.57 (C^4), 111.53 (C^3), 68.49 (d, *J* = 7.0 Hz, OCH_2CH_2Ar), 68.03 (d, *J* = 6.8 Hz, $OCH_2C_{11}H_{23}$), 50.89 ($CH_2(CH_2)_2NH_2$), 48.53 (PCH_2CH_2), 37.81 (CH_2NH_2), 33.07, 31.48 (d, *J* = 5.9 Hz, $OCH_2CH_2C_{10}H_{21}$), 30.80, 30.78, 30.76, 30.71, 30.65, 30.47, 30.25, 27.57 (d, *J* = 6.5 Hz, OCH_2CH_2Ar), 26.50, 23.73, 23.16 ($CH_2CH_2NH_2$), 21.03 (d, *J* = 140.9 Hz, PCH_2), 14.44 (CH_3).

^{31}P{^{1}H} NMR (162 MHz, CD_3OD): 28.43.

IR ν_{max} (KBr) 3426 (m, br), 3256 (m, vbr), 3100–2500 (m-w, vbr), 2955 (s), 2924 (vs), 2854 (s), 2745 (w, br, sh), 2634 (w, br), 2558 (w, br), 2035 (vw, vbr), 1618 (w), 1610 (w, sh), 1491 (w, sh), 1467 (m), 1458 (m), 1433 (w, sh), 1379 (vw), 1352 (vw), 1302 (vw), 1252 (w), 1226 (m), 1180 (w), 1148 (vw), 1083 (w, sh), 1058 (m), 1007 (m), 964 (w, sh), 936 (w, sh), 876 (vw), 741 (w), 721 (w), 430 (w).

HR-MS(ESI$^+$): For $C_{30}H_{56}N_4O_3P$ $(M+H)^+$ calculated 551.40845, found 551.40785.

^{1}H NMR spectrum of compound **10n**

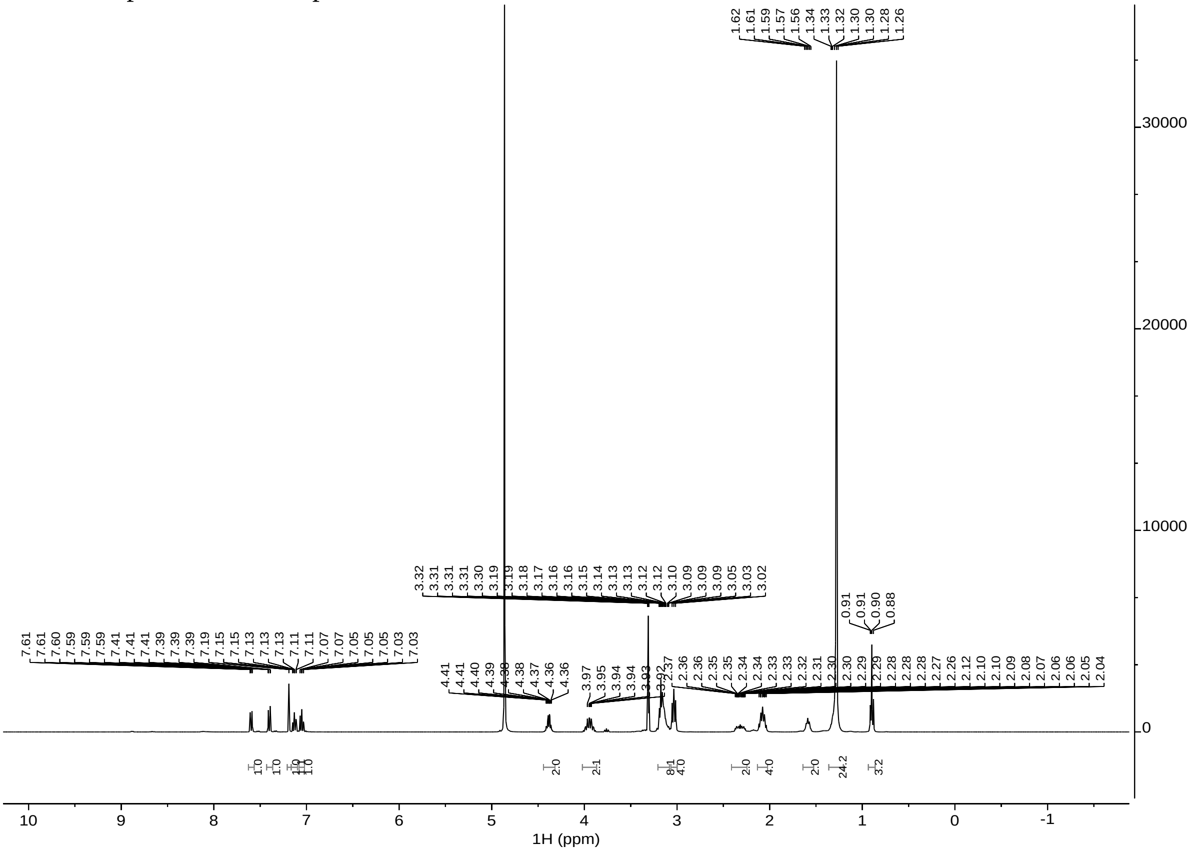

^{13}C NMR spectrum of compound **10n**

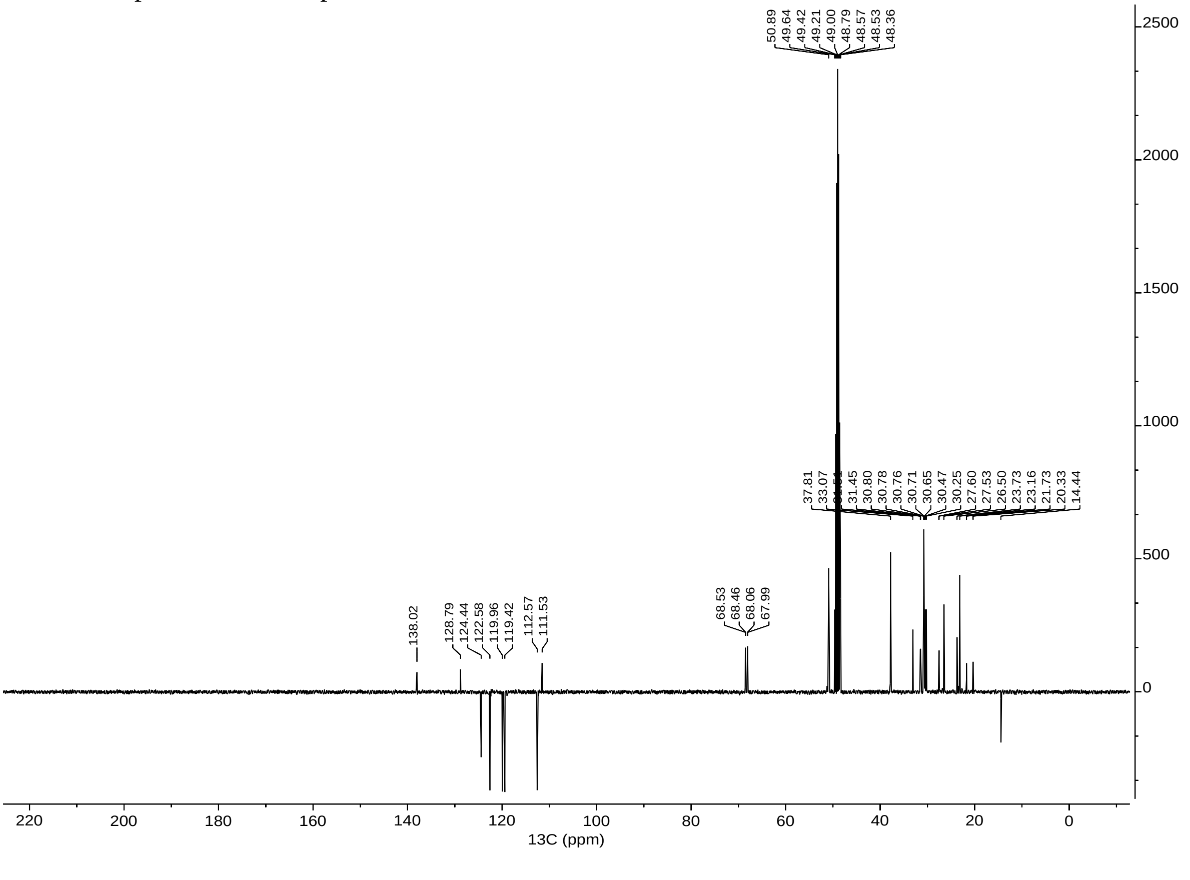

$^{31}P\{^{1}H\}$ NMR spectrum of compound **10n**

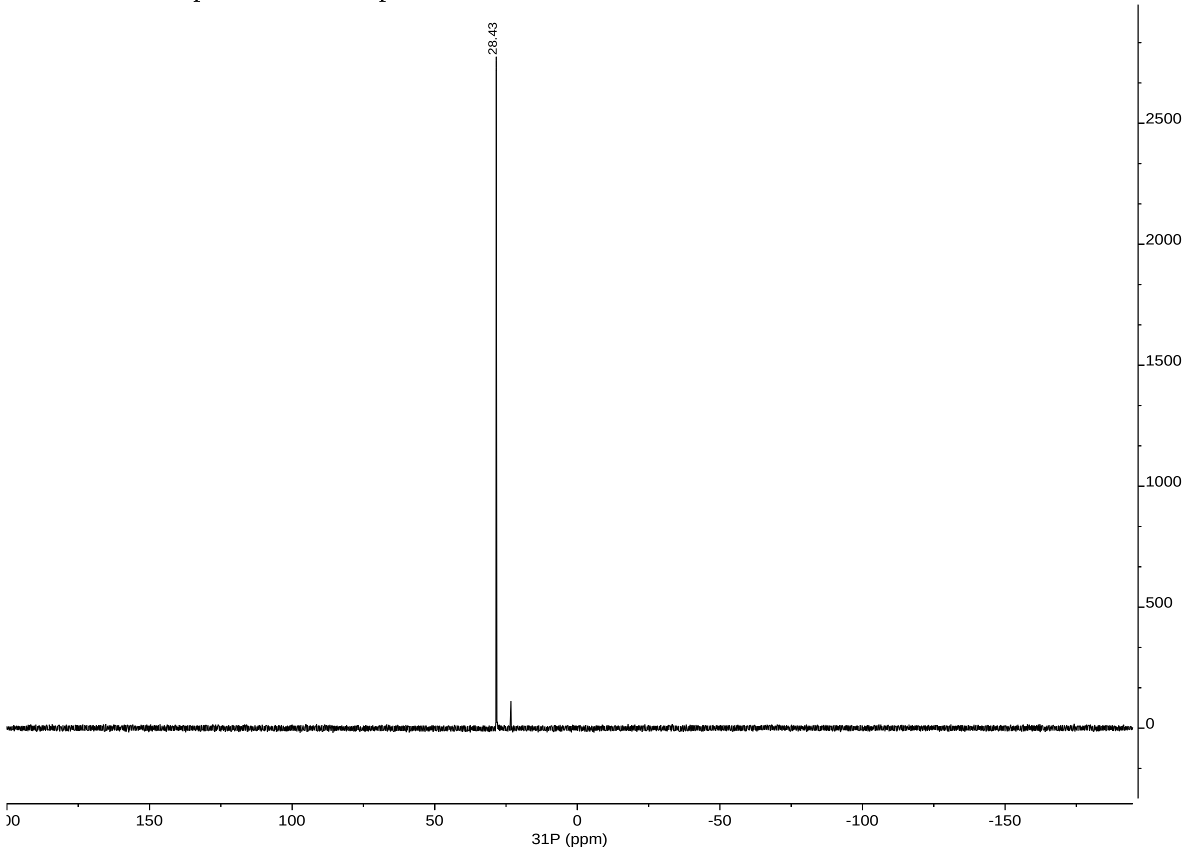

LC-MS chromatograms for compound **10n**

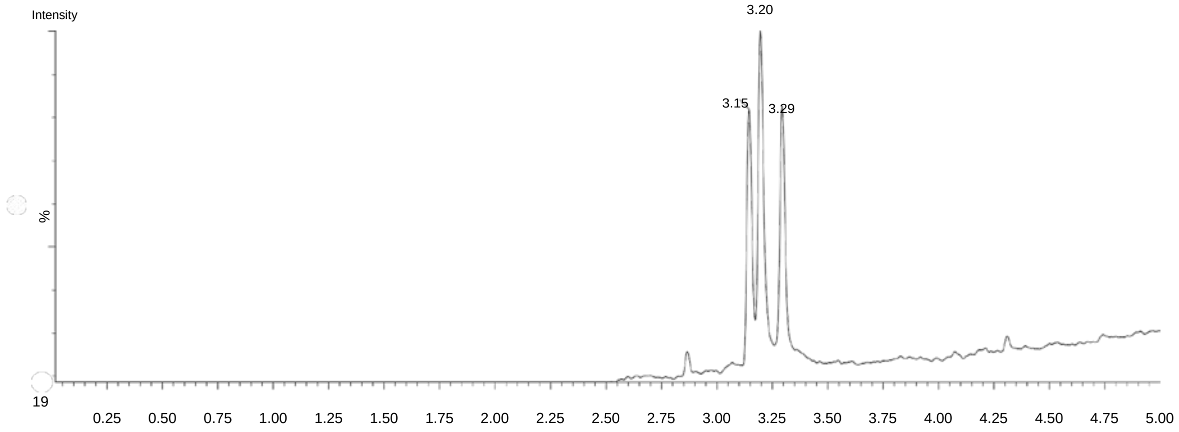

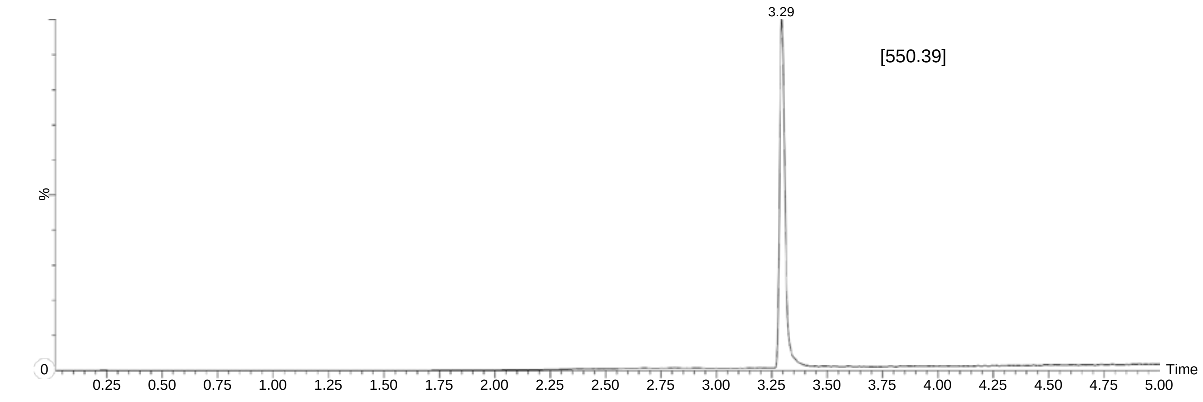

2-(Indol-3-yl)ethyl tetradecyl (2-(bis(3-aminopropyl)amino)ethyl)phosphonate tetrahydrochloride (10o)

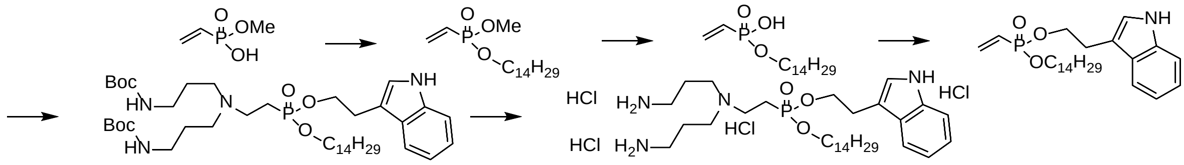

The title compound was prepared according to general methods **A**, **B2**, **D** and **E** from mono methyl vinylphosphonate (0.64 g, 4.7 mmol) in 21% overall yield (0.87 g, 0.99 mmol) as a white amorphous solid.

^{1}H NMR (401 MHz, CD_3OD): 7.60 (dt, J = 7.9, 1.1 Hz, 1H, C^{7}*H*), 7.40 (dt, J = 8.1, 1.0 Hz, 1H, C^{4}*H*), 7.19 (s, 1H, C^{2}*H*), 7.13 (ddd, J = 8.2, 7.0, 1.2 Hz, 1H, C^{6}*H*), 7.05 (ddd, J = 8.0, 7.0, 1.1 Hz, 1H, C^{5}*H*), 4.46–4.32 (m, 2H, OC*H*$_2$CH$_2$Ar), 4.03–3.87 (m, 2H, OC*H*$_2$C$_{11}$H$_{23}$), 3.24–3.09 (m, 8H, NC*H*$_2$, OCH$_2$C*H*$_2$Ar), 3.04 (t, J = 7.5 Hz, 4H, C*H*$_2$NH$_2$), 2.39–2.24 (m, 2H, PC*H*$_2$), 2.14–2.02 (m, 4H, C*H*$_2$CH$_2$NH$_2$), 1.64–1.53 (m, 2H, OCH$_2$C*H*$_2$C$_{10}$H$_{21}$), 1.28 (s, 22H, (C*H*$_2$)$_9$CH$_3$), 0.95–0.85 (m, 3H, C*H*$_3$).

^{13}C NMR (101 MHz, CD_3OD): 138.01 (*C*^{7a}), 128.79 (*C*^{3a}), 124.44 (*C*^{2}), 122.57 (*C*^{6}), 119.96 (*C*^{5}), 119.42 (*C*^{7}), 112.57 (*C*^{4}), 111.53 (*C*^{3}), 68.48 (d, J = 7.0 Hz, *C*H$_2$CH$_2$Ar), 68.02 (d, J = 6.9 Hz, O*C*H$_2$C$_{11}$H$_{23}$), 50.88 (*C*H$_2$(CH$_2$)$_2$NH$_2$), 48.55 (PCH$_2$*C*H$_2$), 37.80 (*C*H$_2$NH$_2$), 33.06, 31.48 (d, J = 6.0 Hz, OCH$_2$*C*H$_2$C$_{10}$H$_{21}$), 30.77, 30.74, 30.70, 30.64, 30.47, 30.25, 27.56 (d, J = 6.5 Hz, OCH$_2$*C*H$_2$Ar), 26.49, 23.73, 23.14 (*C*H$_2$CH$_2$NH$_2$), 21.04 (d, J = 140.9 Hz, P*C*H$_2$), 14.44 (*C*H$_3$).

^{31}P{^{1}H} NMR (162 MHz, CD_3OD): 28.41.

IR ν_{max} (KBr) 3422 (s, br), 3246 (m, br), 3000–2500 (m, vbr), 2956 (s), 2924 (vs), 2854 (s), 2745 (m, br, sh), 2460 (m, br), 2559 (m, br), 2040 (w, vbr), 1617 (m), 1610 (m, sh), 1548 (w, sh), 1520 (w, sh), 1490 (m, sh), 1467 (m), 1458 (s), 1435 (m, sh), 1379 (w), 1352 (w), 1300 (vw, sh), 1252 (m, sh), 1227 (m), 1181 (m), 1149 (w), 1084 (m, sh), 1058 (m), 1005 (s), 962 (m, sh), 934 (w, sh), 877 (vw), 741 (m), 721 (m), 427 (m).

HR-MS(ESI^{+}): For $C_{32}H_{60}N_4O_3P$ $(M+H)^{+}$ calculated 579.44030, found 579.44012.

^{1}H NMR spectrum of compound **10o**

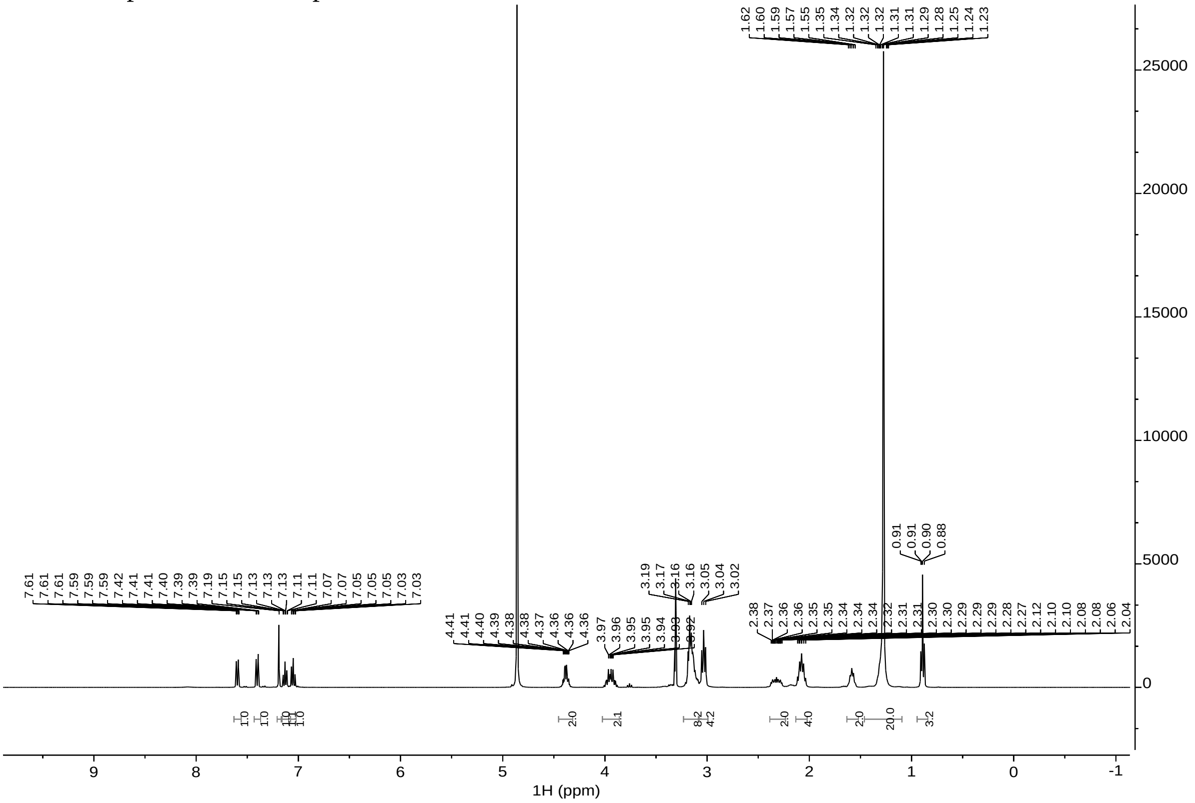

^{13}C NMR spectrum of compound **10o**

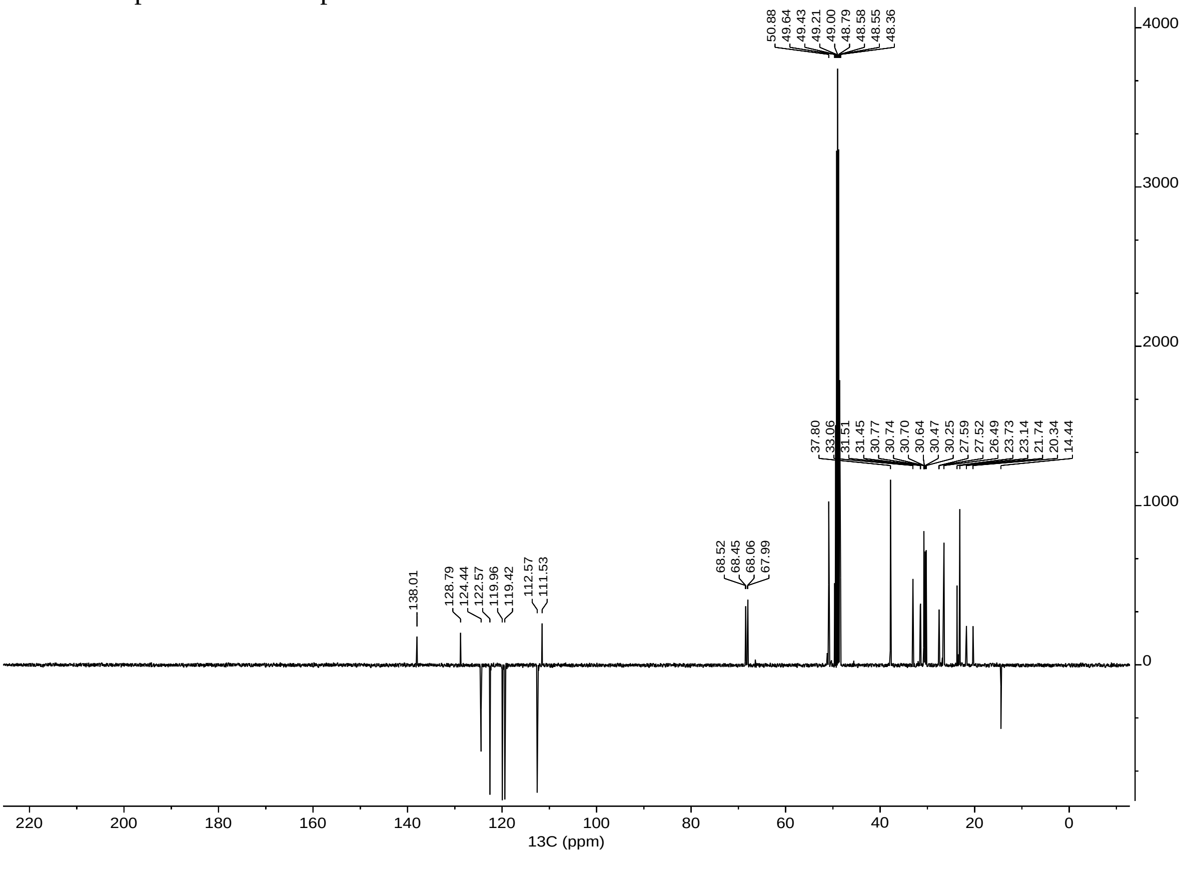

$^{31}P\{^1H\}$ NMR spectrum of compound **10o**

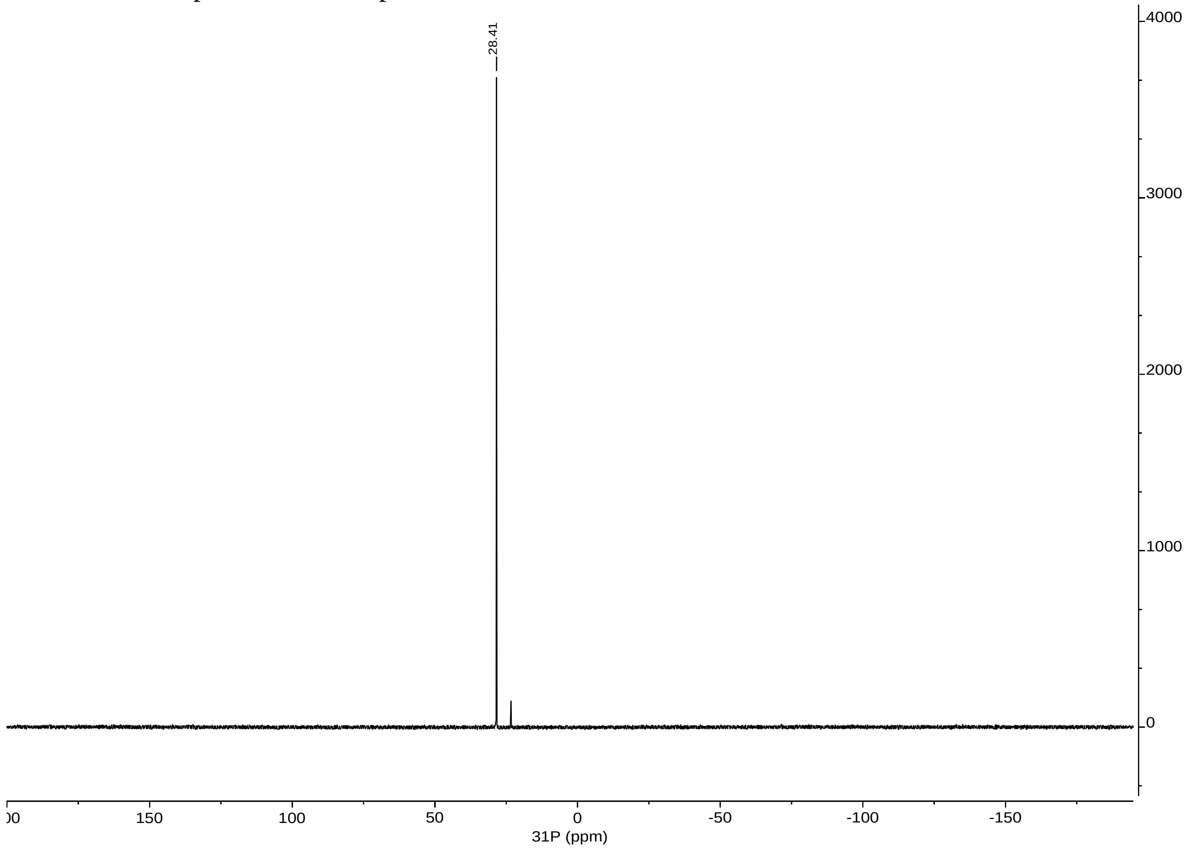

LC-MS chromatograms for compound **10o**

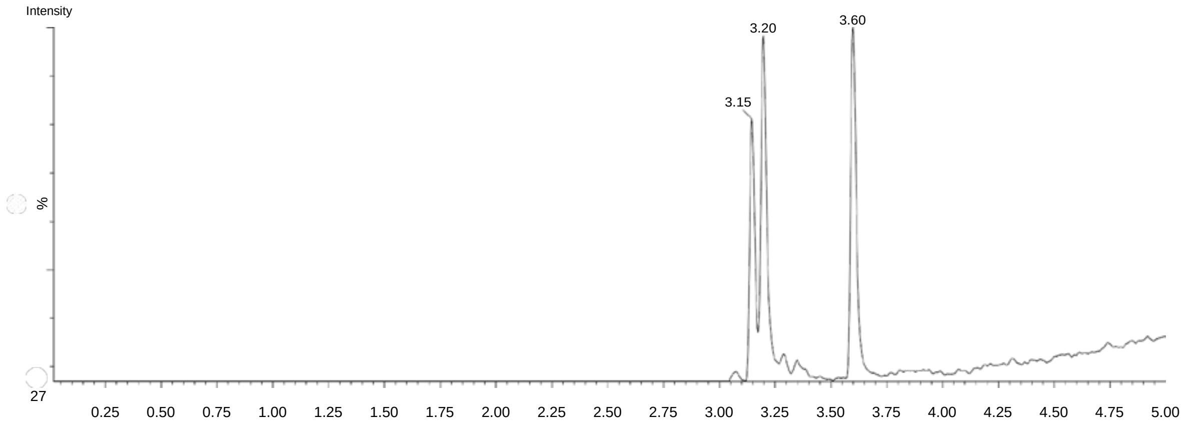

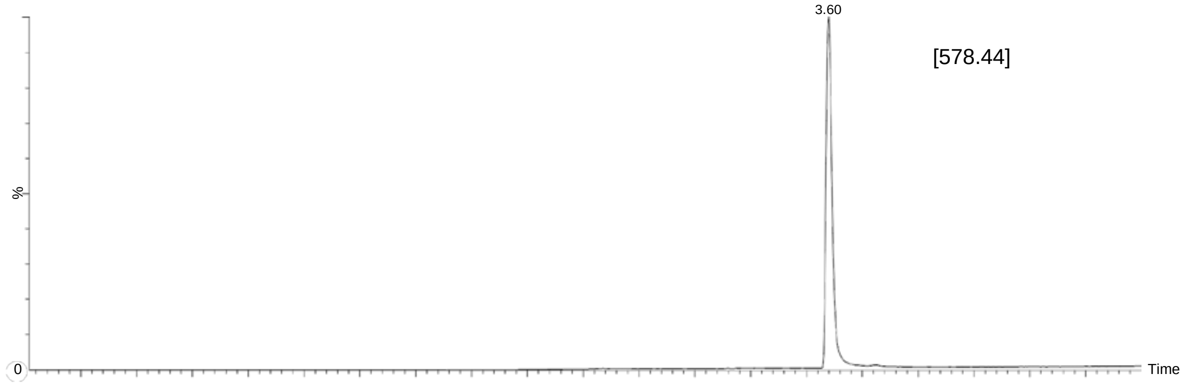

Bis(cyclopentylmethyl) propane-1,3-diyl bis((2-(bis(3-aminopropyl)amino)ethyl)phosphonate) hexahydrochloride (14)

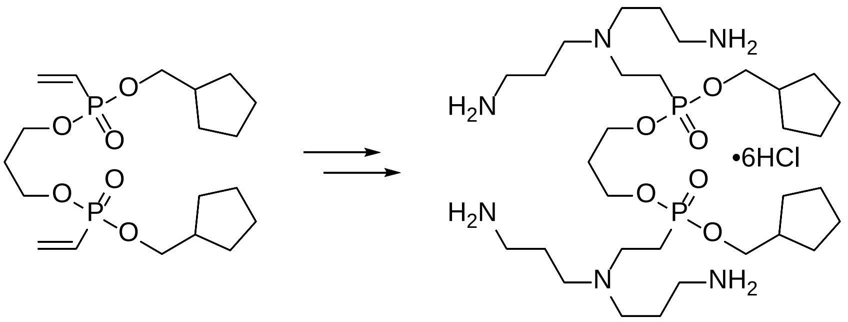

The title compound was prepared according to general methods **D** and **E** from vinylphosphonate dimer **S37** (1.50 g, 3.57 mmol) in 57% overall yield (1.82 g, 2.02 mmol) as a white solid.
Mixture of diastereoisomers (A, B).
^{1}H NMR (500.2 MHz, CD_3OD): 4.40–4.20 (m, 8H, OC*H*$_2$CH$_2$-A,B), 4.09–3.99 (m, 8H, OC*H*$_2$-cyclopent-A,B), 3.55–3.47 (m, 8H, PCH$_2$C*H*$_2$-A,B), 3.47–3.40 (m, 16H, C*H*$_2$(CH$_2$)$_2$NH$_2$-A,B), 3.13 (bt, 16H, *J* = 7.3 Hz, C*H*$_2$NH$_2$-A,B), 2.71–2.60 (m, 8H, PC*H*$_2$-A,B), 2.34–2.19 (m, 20H, *H*-1-cyclopent, C*H*$_2$CH$_2$NH$_2$-A,B), 2.16–2.10 (m, 4H, OCH$_2$C*H*$_2$-A,B), 1.85–1.77 (m, 8H, *H*-2a,5a-cyclopent-A,B), 1.70–1.56 (m, 16H, *H*-3,4-cyclopent-A,B), 1.38–1.30 (m, 8H, *H*-2b,5b-cyclopent-A,B).
^{13}C NMR (125.8 MHz, CD_3OD): 71.93 (d, *J* = 7.1 Hz, O-*C*H$_2$-cyclopent-A), 71.87 (d, *J* = 7.5 Hz, O-*C*H$_2$-cyclopent-B), 64.24 (d, *J* = 6.4 Hz, O*C*H$_2$CH$_2$-B), 63.89 (d, *J* = 6.3 Hz, O*C*H$_2$CH$_2$-A), 51.03 (*C*H$_2$(CH$_2$)$_2$NH$_2$-A,B), 48.96 (PCH$_2$*C*H$_2$-A,B, overlapped by CD_3OD), 41.33 (d, *J* = 6.1 Hz, *C*H-1-cyclopent-A,B), 38.00 (*C*H$_2$NH$_2$-B), 37.91 (*C*H$_2$NH$_2$-A), 32.24 (t, *J* = 6.5 Hz, OCH$_2$*C*H$_2$-B), 31.87 (t, *J* = 6.9 Hz, OCH$_2$*C*H$_2$-A), 29.98 (d, *J* = 2.4 Hz, *C*H$_2$-2,5-cyclopent-A,B), 26.34 (*C*H$_2$-3,4-cyclopent-A,B), 23.18 (*C*H$_2$CH$_2$NH$_2$-A,B), 21.54 (d, *J* = 140.1 Hz, P*C*H$_2$-A), 21.44 (d, *J* = 139.8 Hz, P*C*H$_2$-B).
^{31}P{^{1}H} NMR (202.5 MHz, CD_3OD): 26.92 (B), 26.94 (A).
IR ν_{max} (KBr) 2955 (vs), 2950 (s, vbr), 2906 (s, sh), 2870 (s), 2744 (m, br, sh), 2635 (m, vbr), 2559 (m, vbr), 2017 (w, vbr), 1602 (w, br), 1505 (w, br, sh), 1520 (w, br, sh), 1471 (m), 1405 (w, br), 1253 (m, br, sh), 1224 (m), 1076 (w, sh), 1023 (s), 1005 (s).
HR-MS(ESI$^+$): For $C_{31}H_{69}N_6O_6P_2$ $(M+H)^+$ *m/z* calculated 683.47483, found 683.47472.

^{1}H NMR spectrum of compound **14**

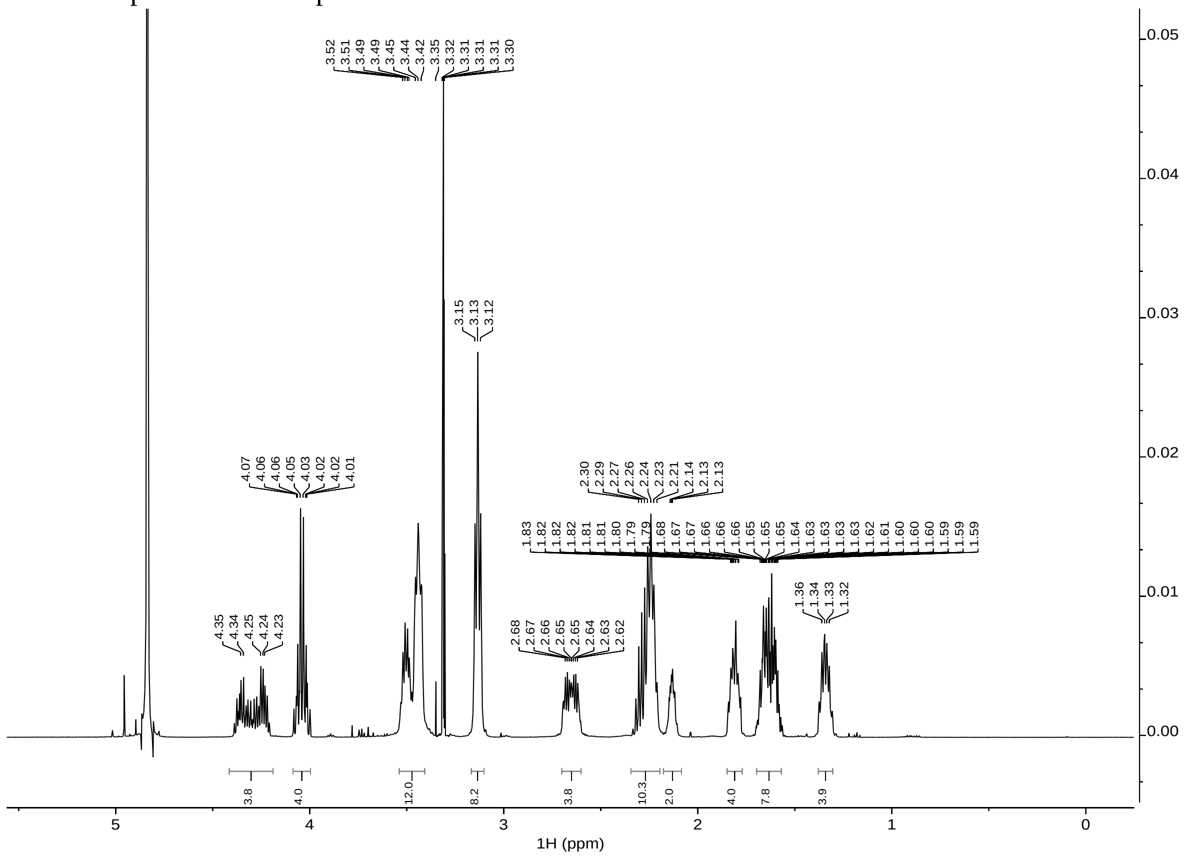

^{13}C NMR spectrum of compound **14**

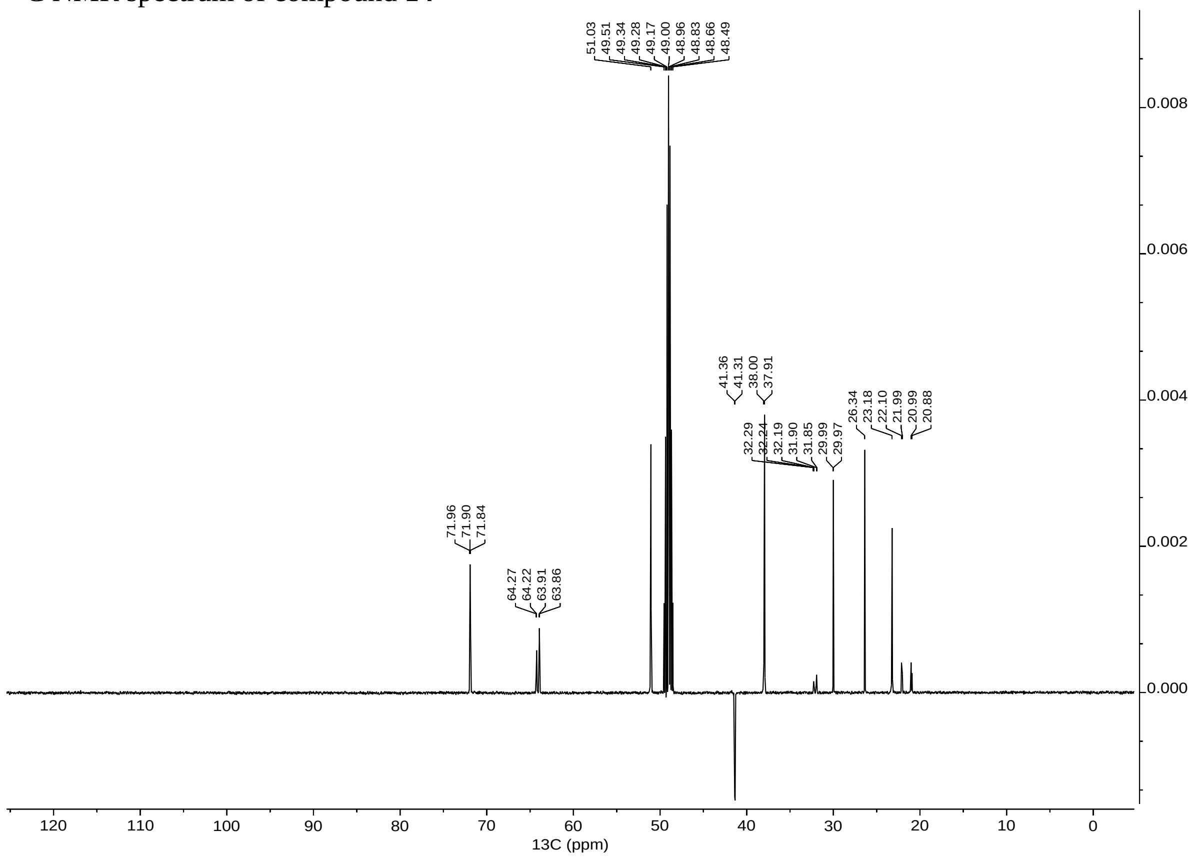

$^{31}P\{^{1}H\}$ NMR spectrum of compound **14**

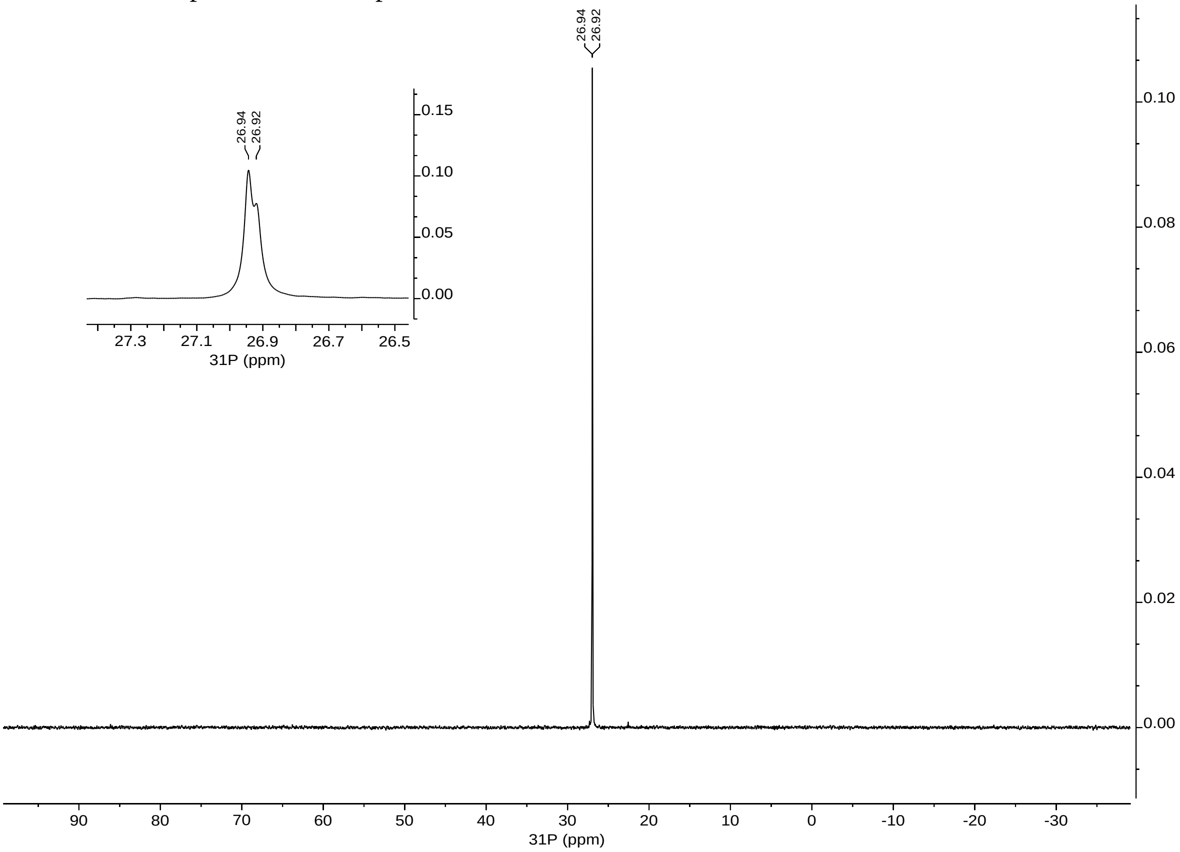

LC-MS chromatograms for compound **14**

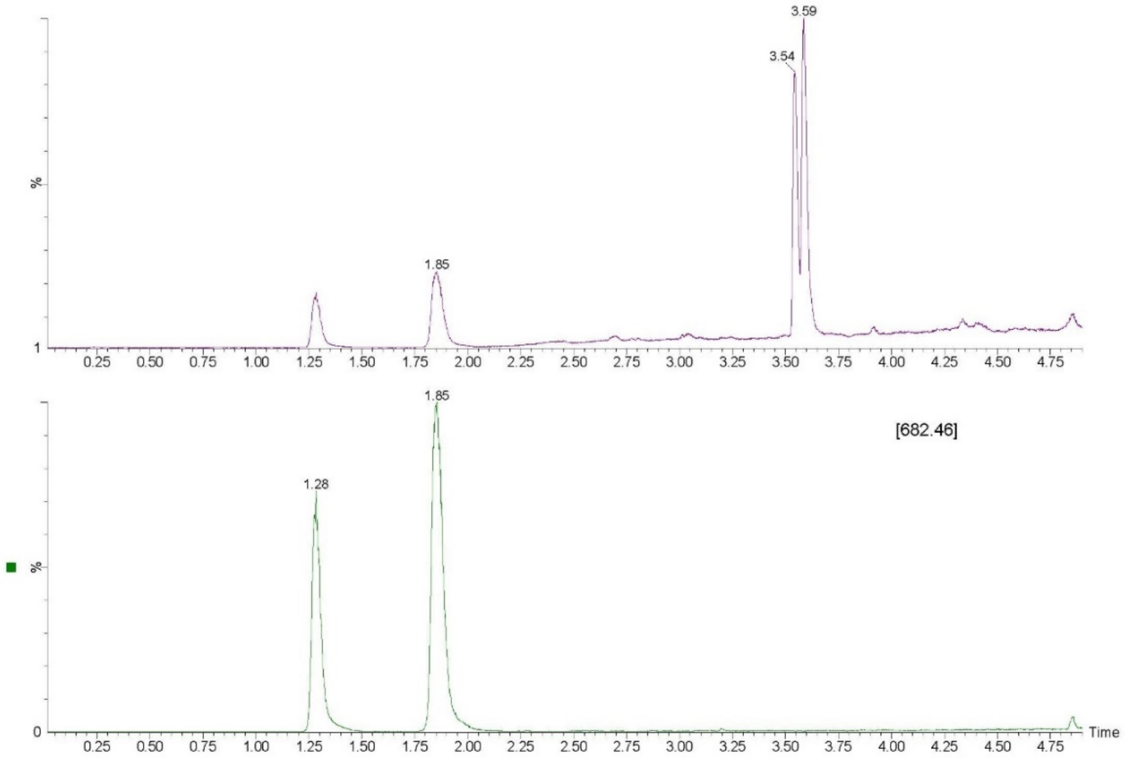

Bis((*Z*)-hept-3-en-1-yl) propane-1,3-diyl bis((2-(bis(3-aminopropyl)amino)ethyl)phosphonate) hexahydrochloride (15)

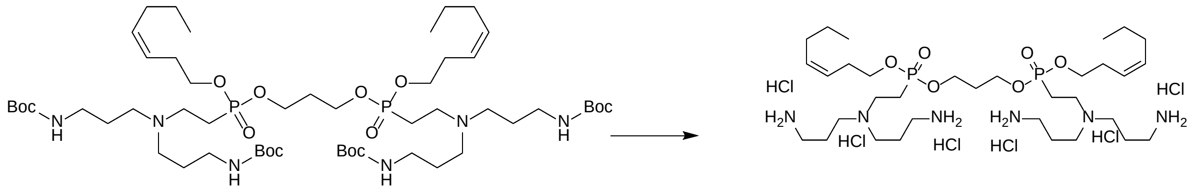

The title compound was prepared according to general method **E** from Boc derivative **S91** (0.67 g, 0.61 mmol) in 96% yield (0.54 g, 0.58 mmol) as a white solid.

Mixture of diastereoisomers.

^{1}H NMR (400 MHz, CD_3OD): 5.62–5.52 (m, 2H, $CH_3(CH_2)_2CH$), 5.49–5.39 (m, 2H, $CH(CH_2)_2O$), 4.40–4.19 (m, 8H, $OCH_2CH_2CH_2O$), 4.19–4.10 (m, 8H, $CHCH_2CH_2O$), 3.57–3.38 (m, 12H, $CH_2(CH_2)_2NH_2$, PCH_2CH_2), 3.13 (t, 8H, J = 7.4 Hz, CH_2NH_2), 2.73–2.58 (m, 4H, PCH_2), 2.54–2.45 (m, 4H, $CHCH_2CH_2O$), 2.24 (p, 8H, J = 7.6 Hz, $CH_2CH_2NH_2$), 2.17–2.03 (m, 6H, $OCH_2CH_2CH_2O$, $CH_3CH_2CH_2$), 1.41 (h, 4H, J = 7.4 Hz, CH_3CH_2), 0.93 (t, 6H, J = 7.4 Hz, CH_3).

^{13}C NMR (101 MHz, CD_3OD): 134.14 ($CH_3(CH_2)_2CH$), 125.19 ($CH(CH_2)_2O$), 67.78 (d, J = 6.9 Hz), 67.72 (d, J = 6.8 Hz, $CHCH_2CH_2O$), 64.23 (d, J = 6.4 Hz), 63.86 (d, J = 6.5 Hz, $OCH_2CH_2CH_2O$), 51.03 ($CH_2(CH_2)_2NH_2$), 48.91, 48.88 (PCH_2CH_2), 37.93 (CH_2NH_2), 32.21 (t, J = 6.7 Hz), 31.86 (t, J = 7.0 Hz, $OCH_2CH_2CH_2O$), 30.44 ($CH_3CH_2CH_2$), 29.64 (d, J = 6.1 Hz, $CHCH_2CH_2O$), 23.76 (CH_3CH_2), 23.21 ($CH_2CH_2NH_2$), 21.56 (d, J = 140.1, 11.40 Hz, PCH_2), 21.45 (d, J = 139.7 Hz, PCH_2), 14.13 (CH_3).

^{31}P{^{1}H} NMR (162 MHz, CD_3OD): 28.64.

IR ν_{max} 3010 (vs), 2959 (vs), 2925 (s), 2897 (s), 2869 (s), 2043 (w), 1700 (w), 1609 (m), 1467 (s), 1380 (m), 1230 (s), 720 (w).

HR-MS(ESI$^+$): For $C_{33}H_{74}O_6N_6P_2$ $(M+2H)^{2+}$ *m/2z* calculated 356.25671, found 356.25688.

^{1}H NMR spectrum of compound **15**

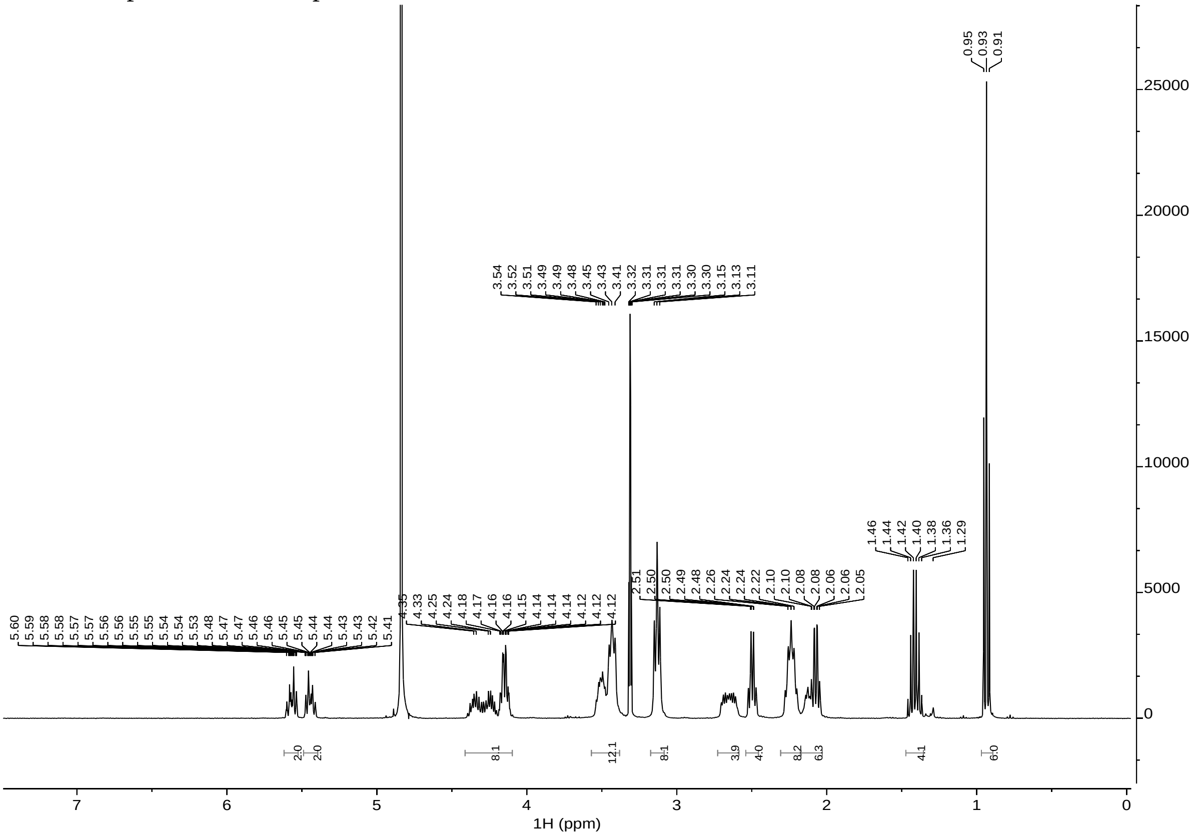

^{13}C NMR spectrum of compound **15**

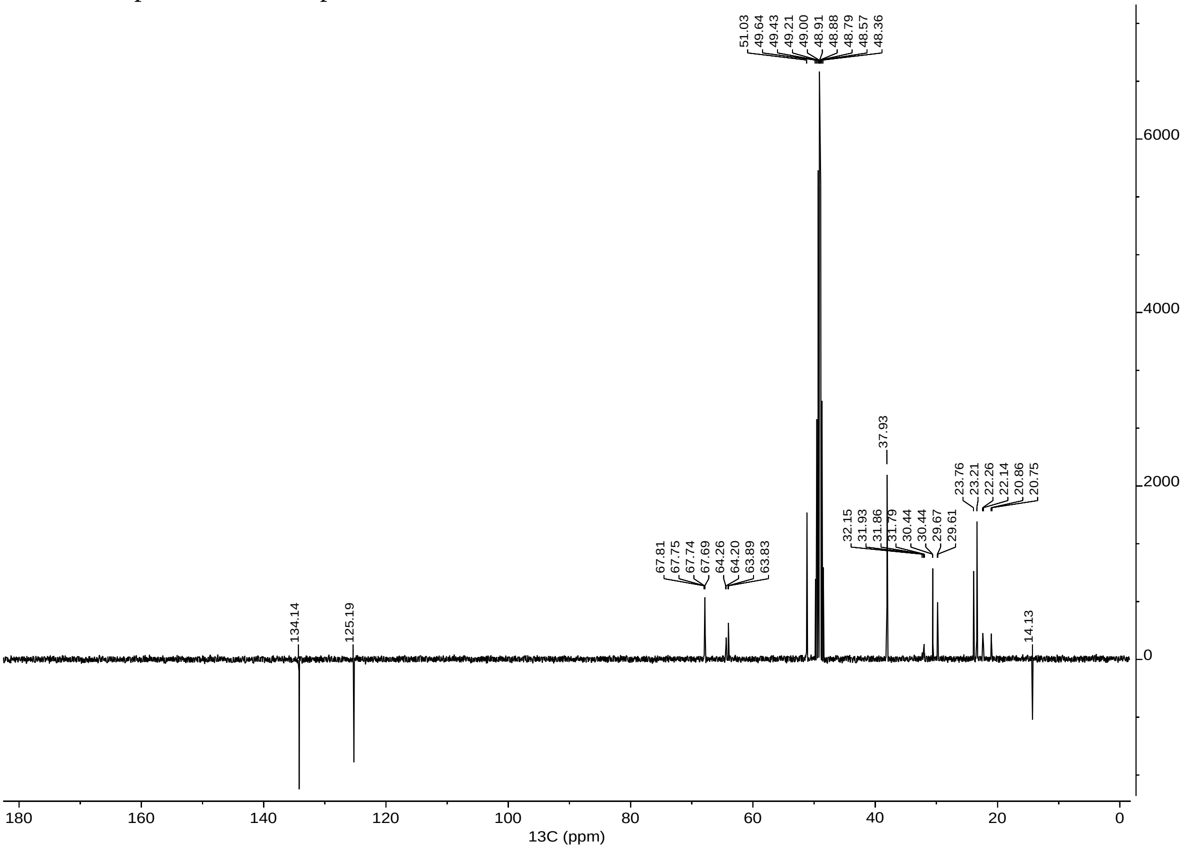

^{31}P{^{1}H} NMR spectrum of compound **15**

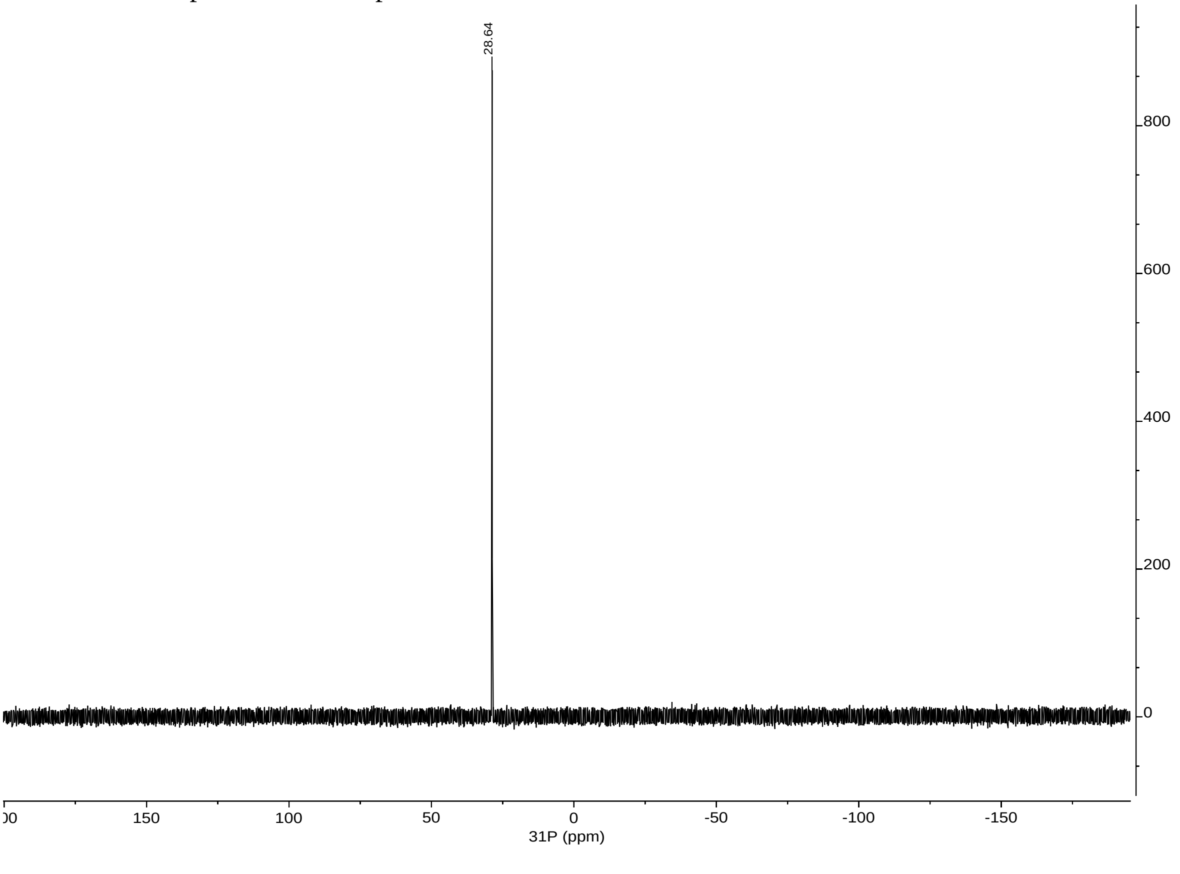

LC-MS chromatograms for compound **15**

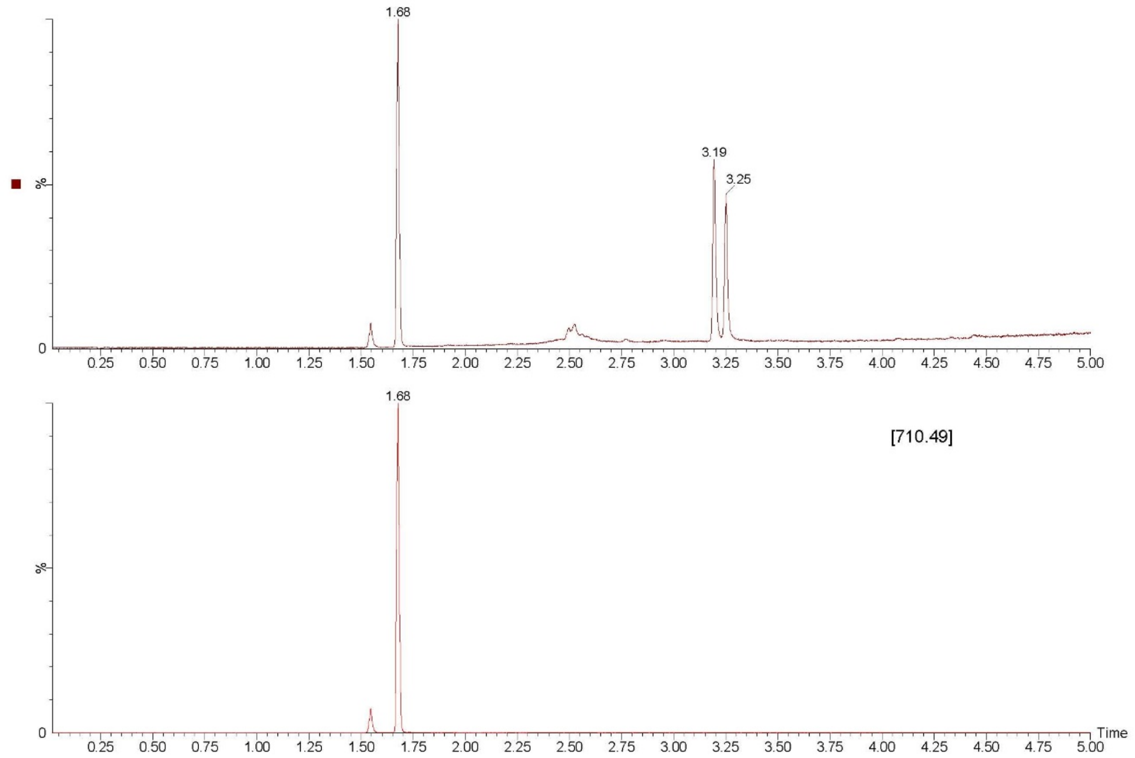

Bis((*Z*)-hept-4-en-1-yl) propane-1,3-diyl bis((2-(bis(3-aminopropyl)amino)ethyl)phosphonate) hexahydrochloride (16)

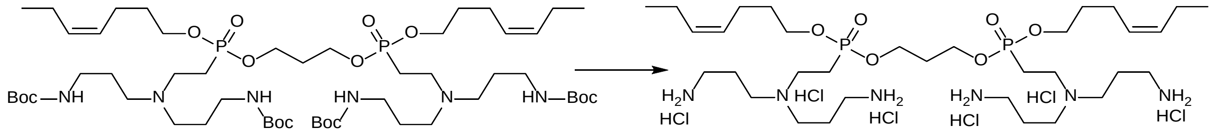

The title compound was prepared according to general method **E** from Boc derivative **S92** (0.20 g, 0.18 mmol) in 96% yield (0.16 g, 0.17 mmol) as a white solid.

^{1}H NMR (401 MHz, CD_3OD): 5.50–5.41 (m, 2H, $CH_3CH_2C\mathit{H}$), 5.40–5.31 (m, 2H, $C\mathit{H}(CH_2)_3O$), 4.42–4.10 (m, 8H, $C\mathit{H}_2O$), 3.52–3.47 (m, 4H, $PCH_2C\mathit{H}_2$), 3.46–3.38 (m, 8H, $C\mathit{H}_2(CH_2)_2NH_2$), 3.12 (t, 8H, *J* = 7.5 Hz, $C\mathit{H}_2NH_2$), 2.73–2.60 (m, 4H, $PC\mathit{H}_2$), 2.29–2.02 (m, 18H, $OCH_2C\mathit{H}_2CH_2O$, $CH_3C\mathit{H}_2(CH)_2C\mathit{H}_2$, $C\mathit{H}_2CH_2NH_2$), 1.85–1.73 (m, 4H, $CHCH_2C\mathit{H}_2$), 0.98 (t, 6H, *J* = 7.5 Hz, $C\mathit{H}_3$).

^{13}C NMR (101 MHz, CD_3OD): 133.89 ($CH_3CH_2\mathit{C}H$), 128.45 ($\mathit{C}H(CH_2)_3O$), 67.85 (d, *J* = 6.6 Hz, $CH(CH_2)_2\mathit{C}H_2O$), 63.86 (d, *J* = 6.2 Hz, $O\mathit{C}H_2CH_2\mathit{C}H_2O$), 51.03 ($\mathit{C}H_2(CH_2)_2NH_2$), 48.88 ($PCH_2\mathit{C}H_2$), 37.93 ($\mathit{C}H_2NH_2$), 31.91 (t, *J* = 6.9 Hz, $OCH_2\mathit{C}H_2CH_2O$), 31.68 (d, *J* = 6.0 Hz, $CHCH_2\mathit{C}H_2CH_2O$), 24.02 ($CH\mathit{C}H_2(CH_2)_2O$), 23.27 ($\mathit{C}H_2CH_2NH_2$), 21.50 ($CH_3\mathit{C}H_2$), 21.53 (d, *J* = 140.0 Hz, $P\mathit{C}H_2CH_2N$), 14.72 ($\mathit{C}H_3$).

^{31}P{^{1}H} NMR (162 MHz, CD_3OD): 28.97.

IR ν_{max} 3013 (s, sh), 2962 (vs), 2935 (s), 2875 (s, sh), 2010 (w), 1653 (w), 1405 (m), 1380 (w, sh), 1222 (m), 1016 (s), 987 (s), 841 (w), 756 (w).

HR-MS(ESI^{+}): For $C_{33}H_{74}O_6N_6P_2$ $(M+2H)^{2+}$ *m/2z* calculated 356.25671, found 356.25693.

^{1}H NMR spectrum of compound **16**

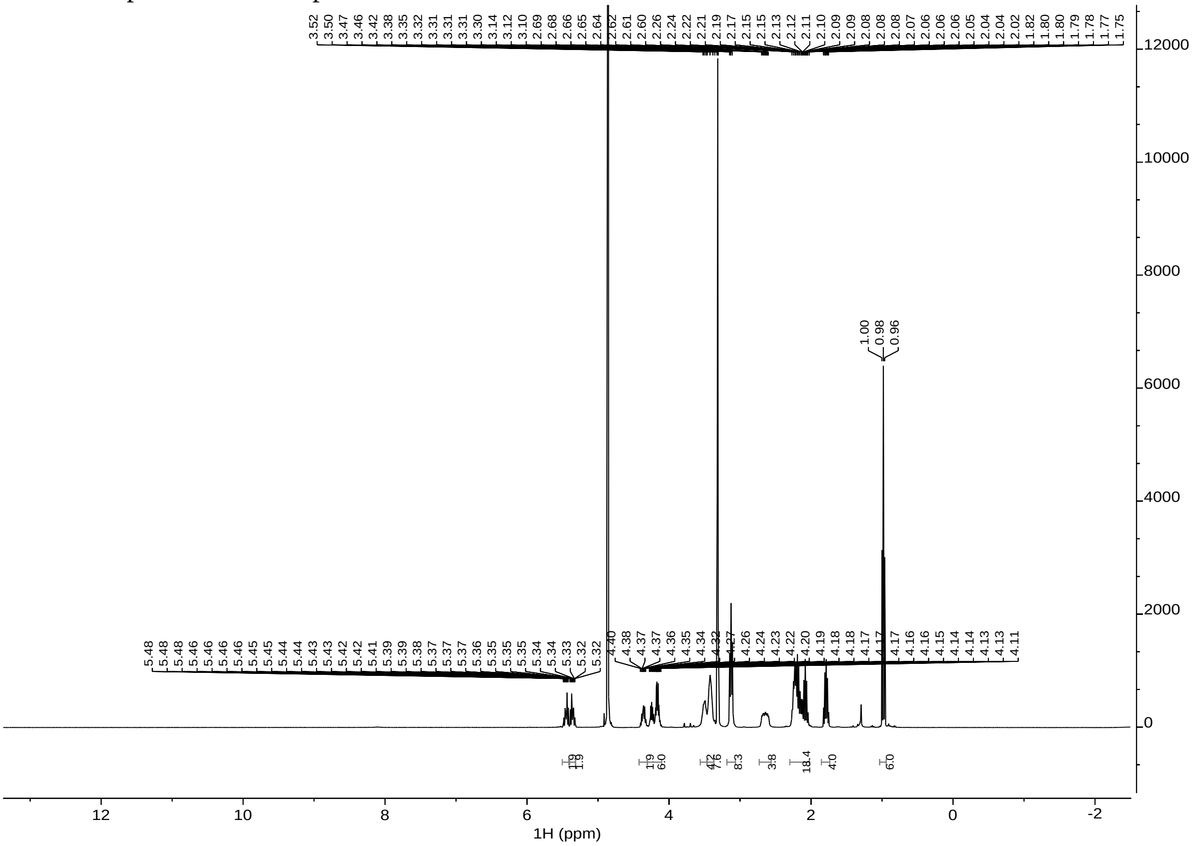

^{13}C NMR spectrum of compound **16**

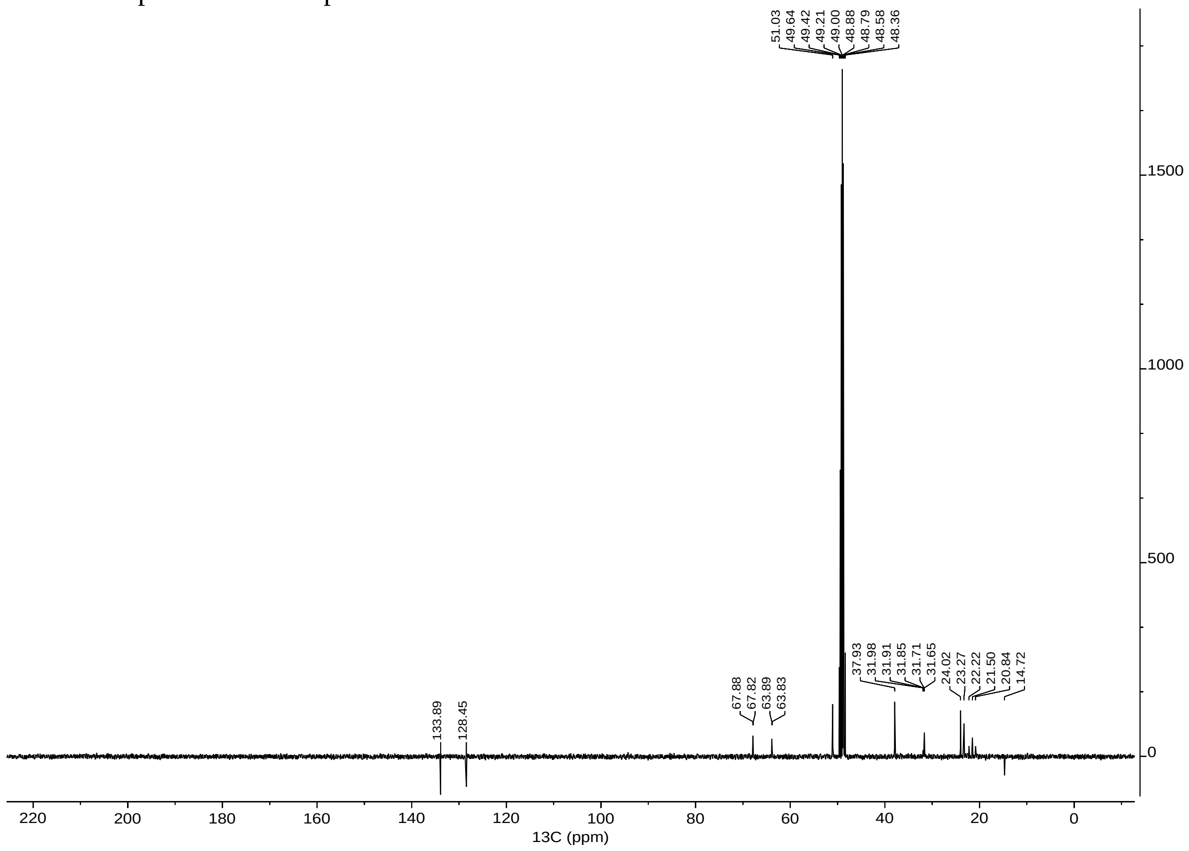

^{31}P{^{1}H} NMR spectrum of compound **16**

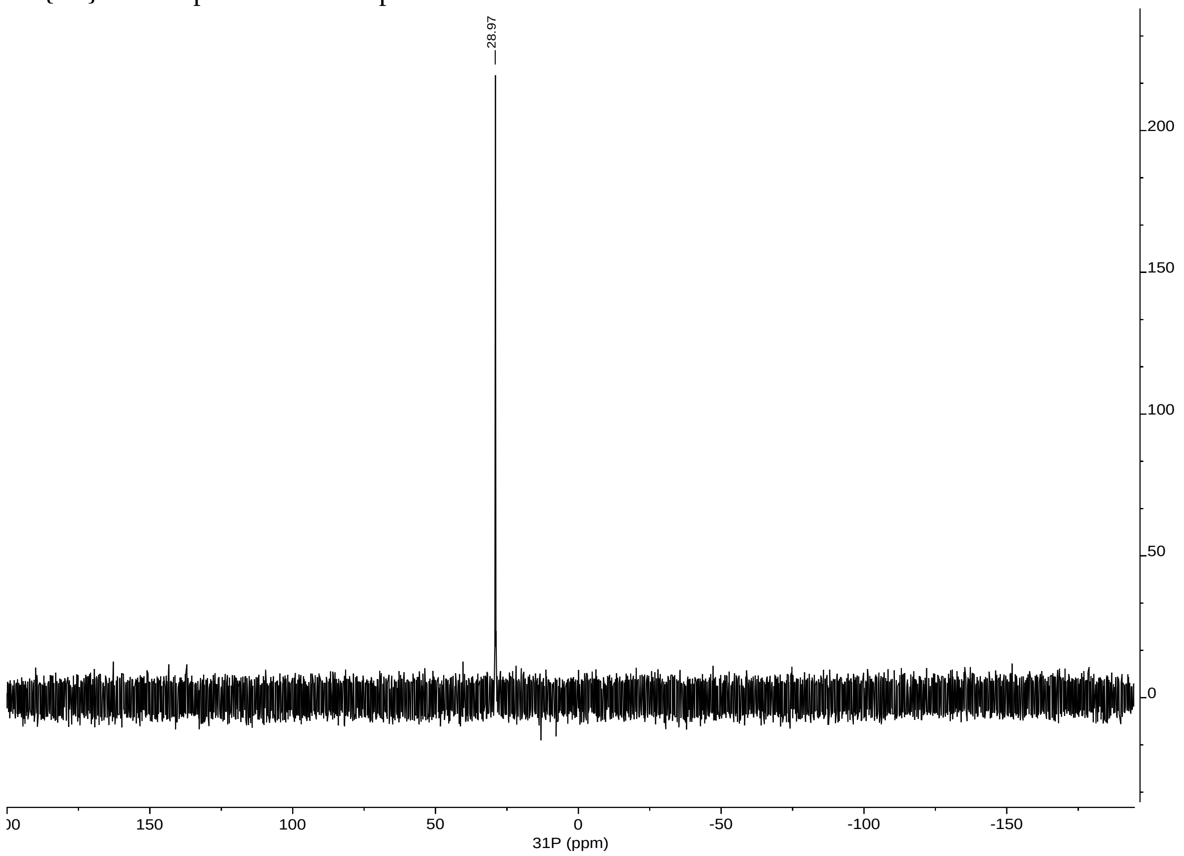

LC-MS chromatograms for compound **16**

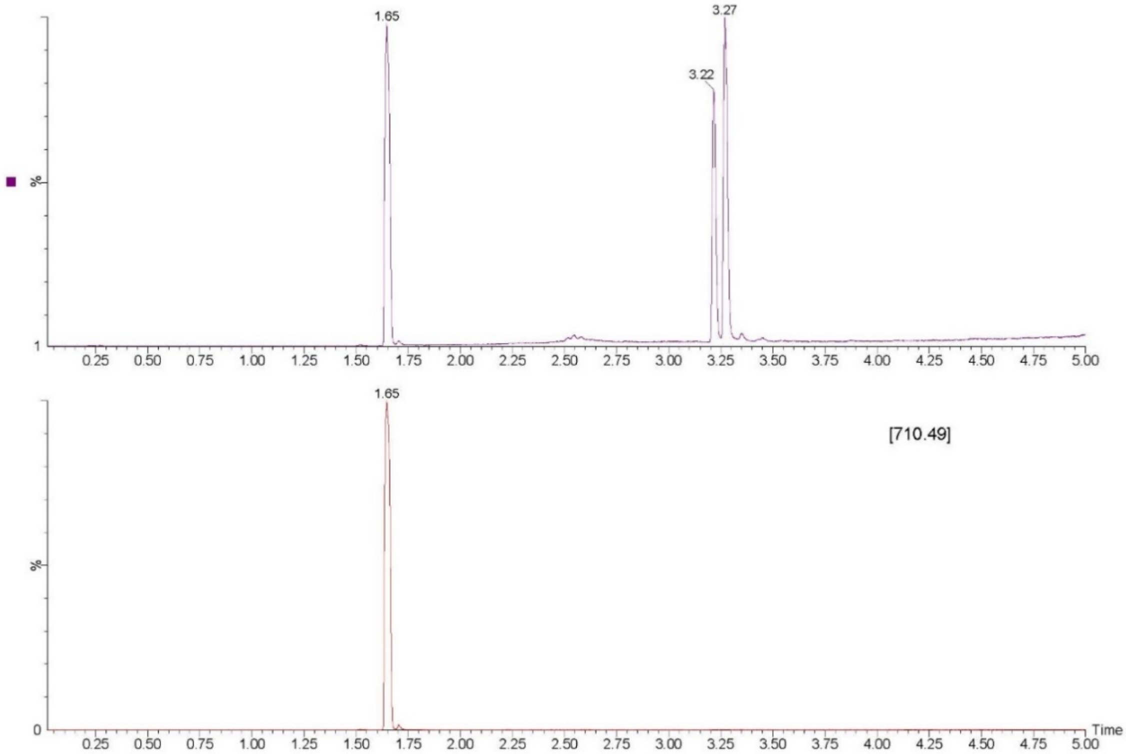

Dioctyl propane-1,3-diyl bis((2-(bis(3-aminopropyl)amino)ethyl)phosphonate) hexahydrochloride (17)

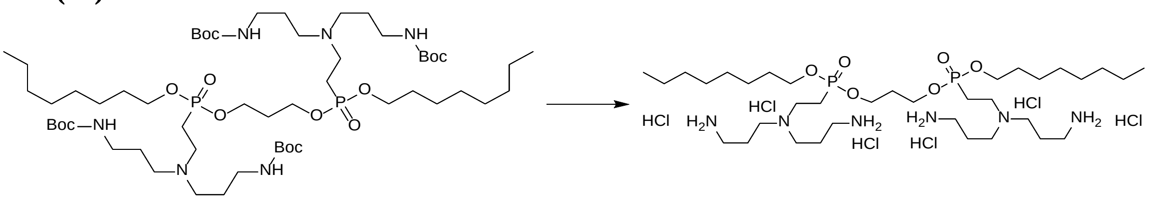

The title compound was prepared according to general method **E** from Boc derivative **S93** (0.34 g, 0.30 mmol) in 98% yield (0.28 g, 0.29 mmol) as a white solid.

^{1}H NMR (401 MHz, CD_3OD): 4.42–4.08 (m, 8H, CH_2O), 3.57–3.36 (m, 12H, CH_2N), 3.12 (t, 8H, J = 7.4 Hz, C$H_2$$NH_2$), 2.72–2.56 (m, 4H, PC$H_2$), 2.23 (p, 8H, J = 8.2 Hz, C$H_2$$CH_2NH_2$), 2.13 (p, 2H, J = 5.7 Hz OCH_2C$H_2$$CH_2O$), 1.79–1.68 (m, 4H, $CH_3(CH_2)_5$CH_2), 1.47–1.25 (m, 20H, CH_3(CH_2)$_5$), 0.95–0.87 (m, 6H, CH_3).

^{13}C NMR (101 MHz, CD_3OD): 68.43 (d, J = 6.6 Hz, O*C*H_2CH_2*C*H_2O), 63.83 (d, J = 6.3 Hz, $CH_3(CH_2)_6$*C*H_2O), 51.02 (*C*$H_2(CH_2)_2NH_2$), 48.92 (PCH_2*C*H_2), 37.91 (*C*H_2NH_2), 33.00 (CH_3CH_2*C*H_2), 31.88 (t, J = 7.3 Hz, OCH_2*C*H_2CH_2O), 31.64 (d, J = 6.0 Hz, $CH_3(CH_2)_5$*C*H_2), 30.38, 30.33, 26.65, 23.73 (CH_3*C*H_2), 23.23 (*C*$H_2CH_2NH_2$), 21.51 (d, J = 140.1 Hz, P*C*H_2), 14.44 (*C*H_3).

^{31}P{^{1}H} NMR (162 MHz, CD_3OD): 28.86.

IR ν_{max} 3000 (vs, vbr), 2957 (vs), 2927 (vs), 2856 (s), 2740 (s, sh), 2636 (s, br), 2559 (s, br), 2019 (w, vbr), 1602 (m), 1516 (m, sh), 1468 (s), 1379 (m), 1255 (s, sh), 1227 (s), 1073 (s, sh), 1014 (s, br), 987 (s).

HR-MS(ESI^{+}): For $C_{35}H_{82}O_6N_6P_2$ $(M+2H)^{2+}$ $m/2z$ calculated 372.28801, found 372.28777.

^{1}H NMR spectrum of compound **17**

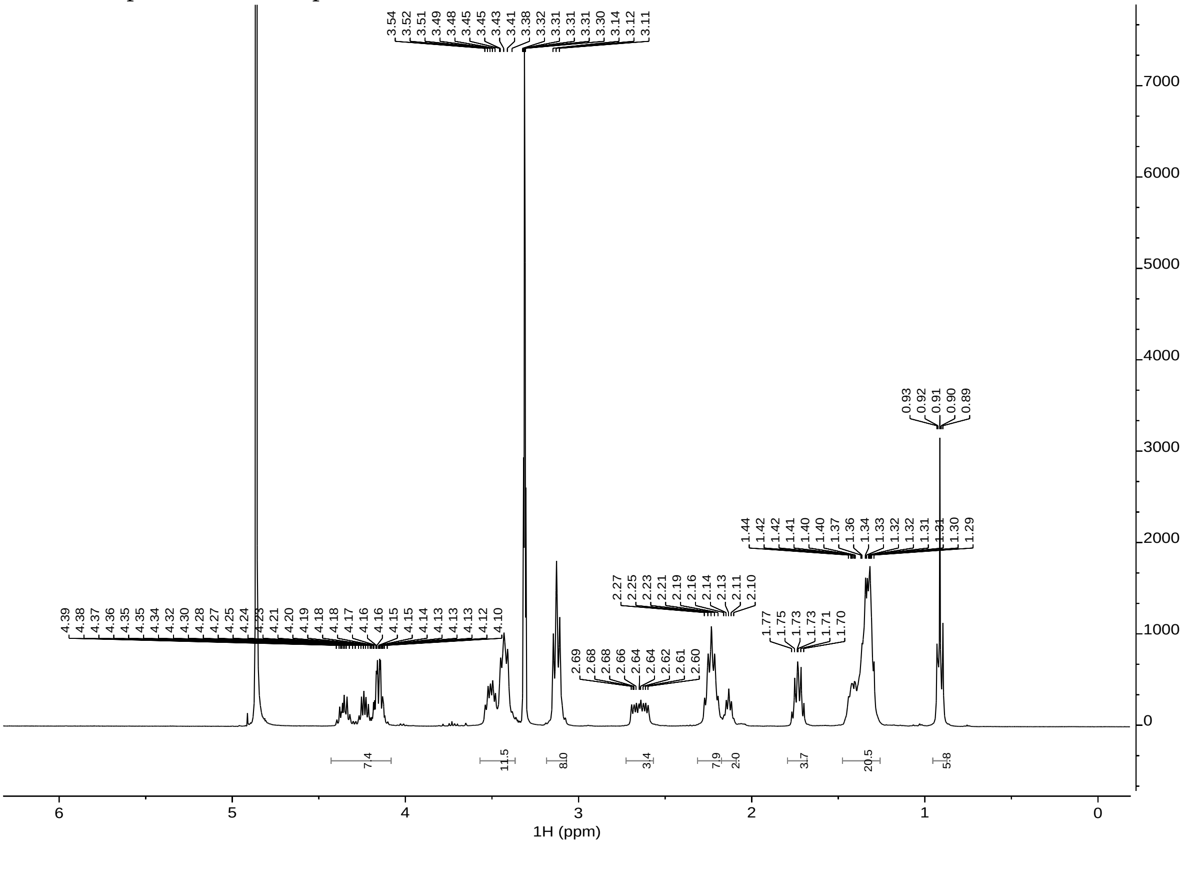

^{13}C NMR spectrum of compound **17**

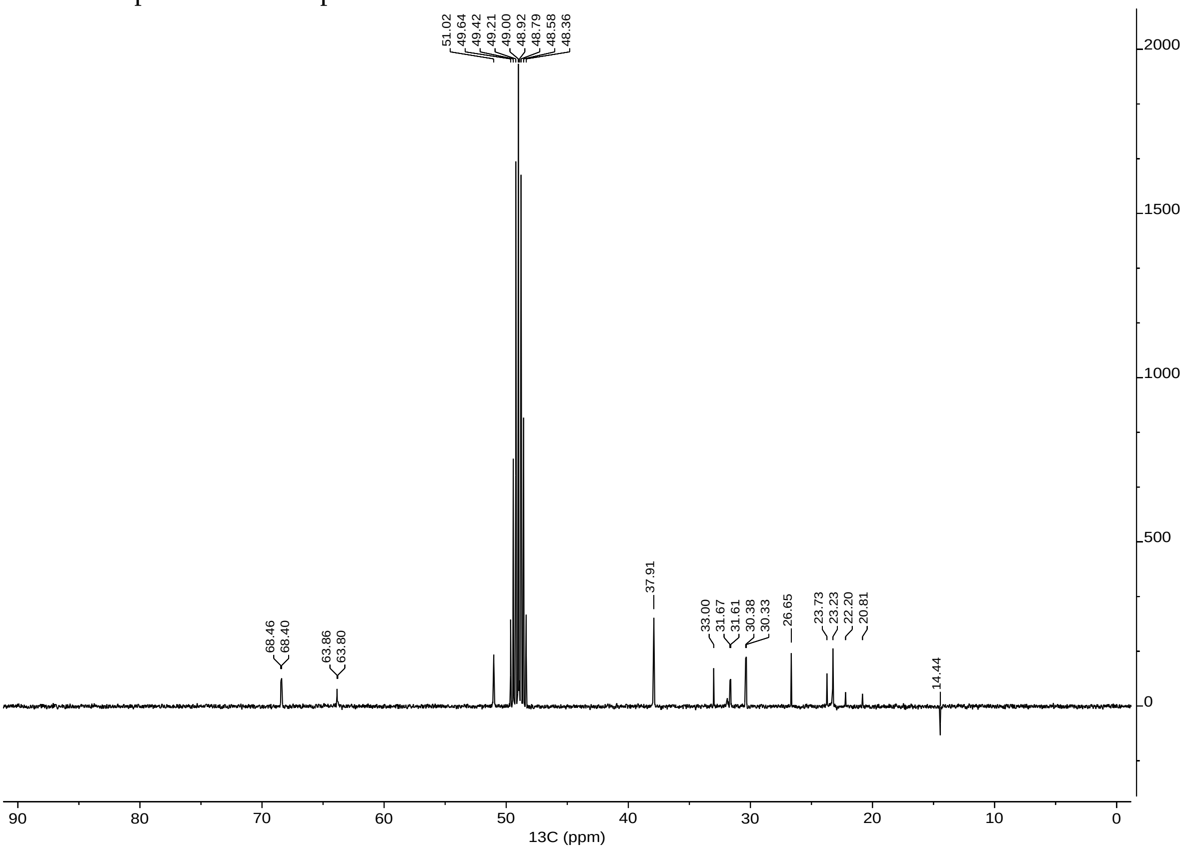

^{31}P{^{1}H} NMR spectrum of compound **17**

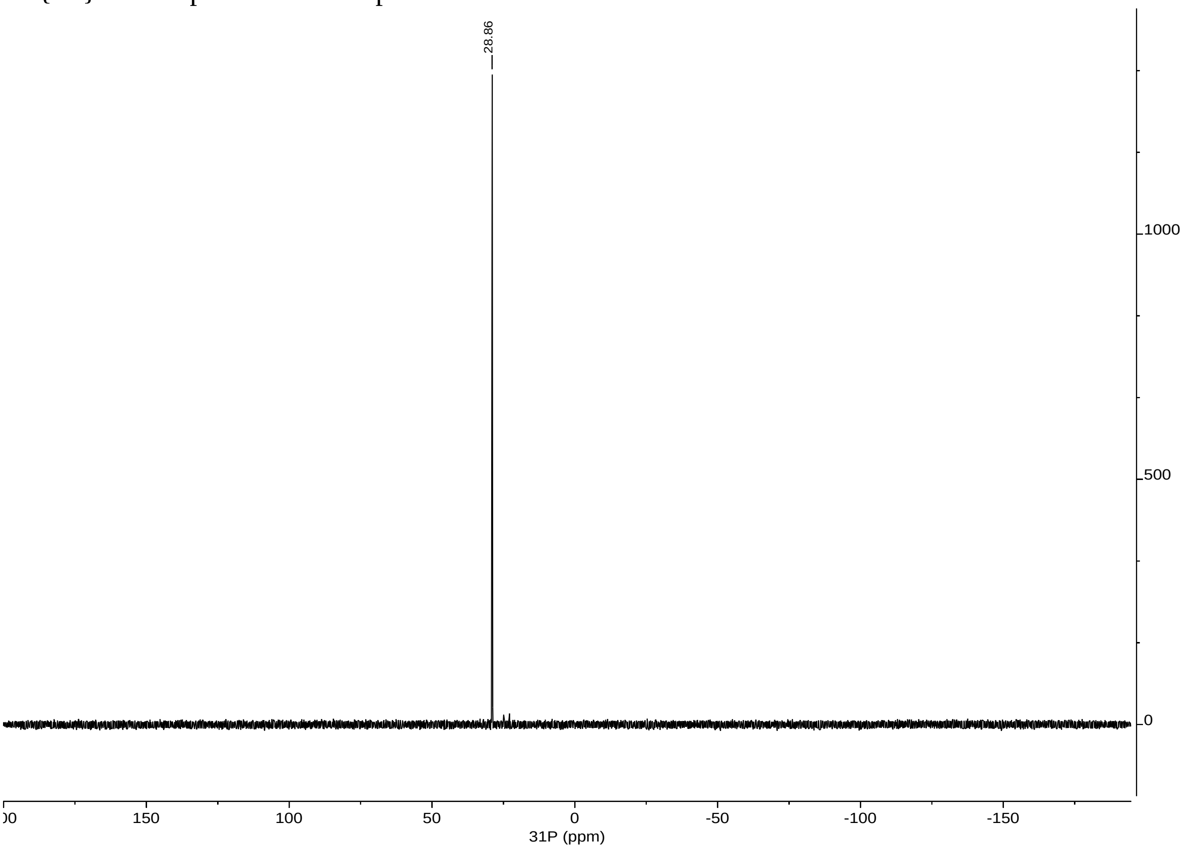

LC-MS chromatograms for compound **17**

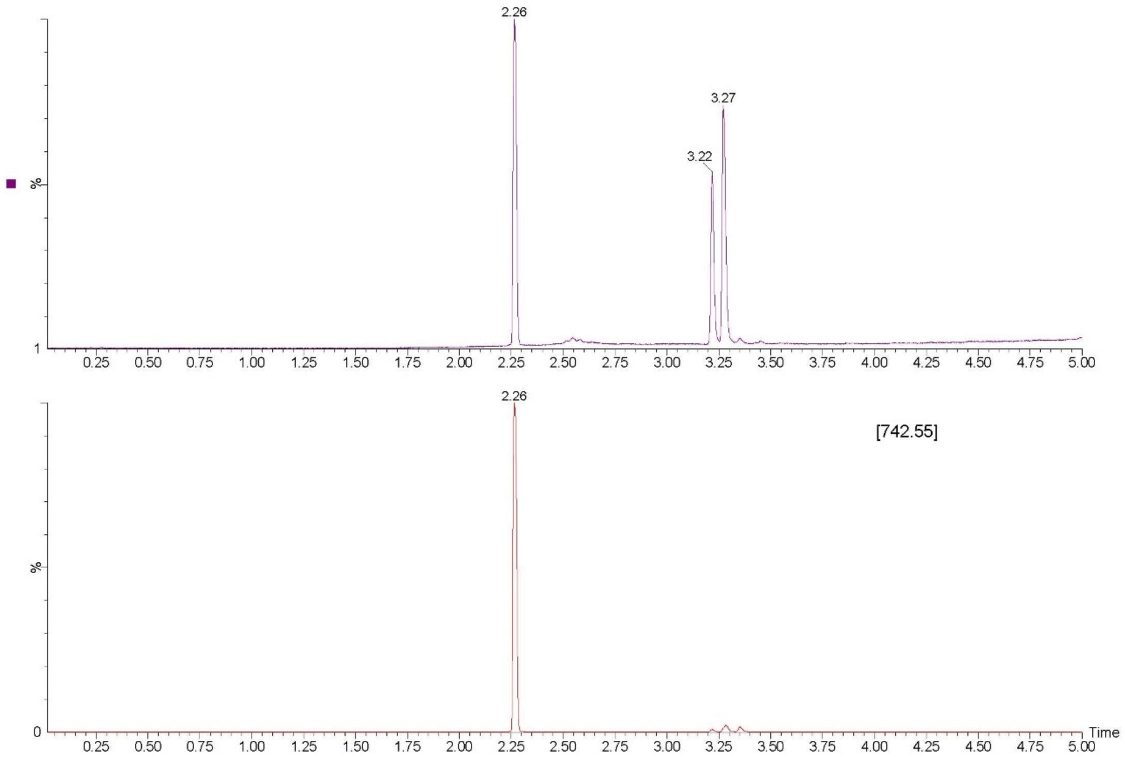

Bis((*Z*)-oct-3-en-1-yl) propane-1,3-diyl bis((2-(bis(3-aminopropyl)amino)ethyl)phosphonate) hexahydrochloride (18)

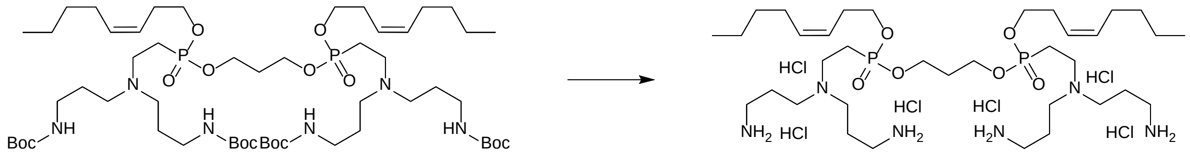

The title compound was prepared according to general method **E** from Boc derivative **S94** (0.57 g, 0.50 mmol) in 92% yield (0.44 g, 0.46 mmol) as a white solid.

Mixture of diastereoisomers.

^{1}H NMR (400 MHz, CD_3OD): 5.64–5.50 (m, 2H, $CH_3(CH_2)_3CH$), 5.50–5.36 (m, 2H, C*H*$(CH_2)_2O$), 4.43–4.19 (m, 4H, OC*H*$_2$$CH_2$C*H*$_2$O), 4.19–4.06 (m, 4H, $CHCH_2$C*H*$_2$O), 3.59–3.46 (m, 4H, PCH_2C*H*$_2$), 3.46–3.37 (m, 8H, C*H*$_2$$(CH_2)_2$NH), 3.12 (t, 8H, *J* = 7.5 Hz, C*H*$_2$$NH_2$), 2.75–2.56 (m, 4H, PC*H*$_2$), 2.49 (q, 4H, *J* = 6.9 Hz, CHC*H*$_2$$CH_2O$), 2.273 (p, 8H, *J* = 6.9 Hz, C*H*$_2$$CH_2NH_2$), 2.17–2.03 (m, 6H, $CH_3(CH_2)_2$C*H*$_2$, OCH_2C*H*$_2$$CH_2O$), 1.44–1.29 (m, 8H, CH_3(C*H*$_2$)$_2$), 1.00–0.88 (m, 6H, C*H*$_3$).

^{13}C NMR (101 MHz, CD_3OD): 134.37 ($CH_3(CH_2)_3$*C*H), 124.95 (*C*H$(CH_2)_2O$), 67.79 (d, *J* = 6.5 Hz), 67.73 (d, *J* = 6.3 Hz $CHCH_2$*C*H$_2$O), 64.23 (d, *J* = 6.6 Hz), 63.86 (d, *J* = 6.2 Hz, O*C*H$_2$$CH_2$*C*H$_2$O), 51.05 (*C*H$_2$$(CH_2)_2NH_2$), 48.89, 48.85 ($PCH_2$*C*H$_2$N), 37.93 (*C*H$_2$$NH_2$), 32.91 ($CH_3CH_2$*C*H$_2$), 32.23 (t, *J* = 5.8 Hz), 31.87 (t, *J* = 7.2 Hz, OCH_2*C*H$_2$$CH_2O$), 29.63 (d, *J* = 6.3 Hz, CH*C*H$_2$$CH_2O$), 28.12 ($CH_3(CH_2)_2$*C*H$_2$), 23.38 ($CH_3$*C*H$_2$), 23.24 (*C*H$_2$$CH_2NH$), 21.55 (d, *J* = 140.4 Hz), 21.44 (d, *J* = 140.3 Hz, P*C*H$_2$), 14.35 (*C*H$_3$).

^{31}P{^{1}H} NMR (162 MHz, CD_3OD): 28.66.

IR ν_{max} (KBr) 3428 (m, br), 3015 (vs, sh), 2958 (vs), 2929 (vs), 2873 (s), 2559 (s, br), 1607 (m), 1467 (m), 1380 (w, sh), 1229 (m), 1011 (s), 1070 (s).

HR-MS(ESI^{+}): For $C_{35}H_{78}O_6N_6P_2$ $(M+2H)^{2+}$ *m*/2*z* calculated 370.27236, found 370.27233.

^{1}H NMR spectrum of compound **18**

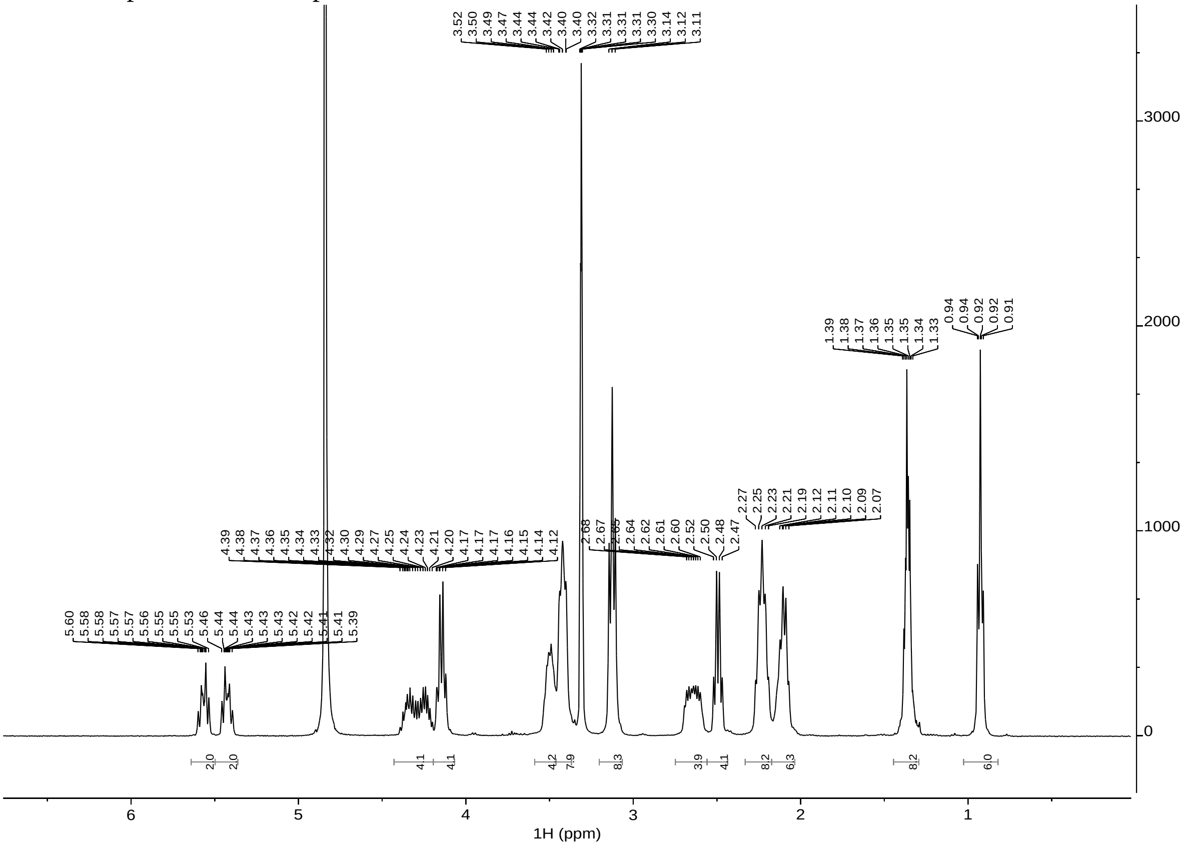

^{13}C NMR spectrum of compound **18**

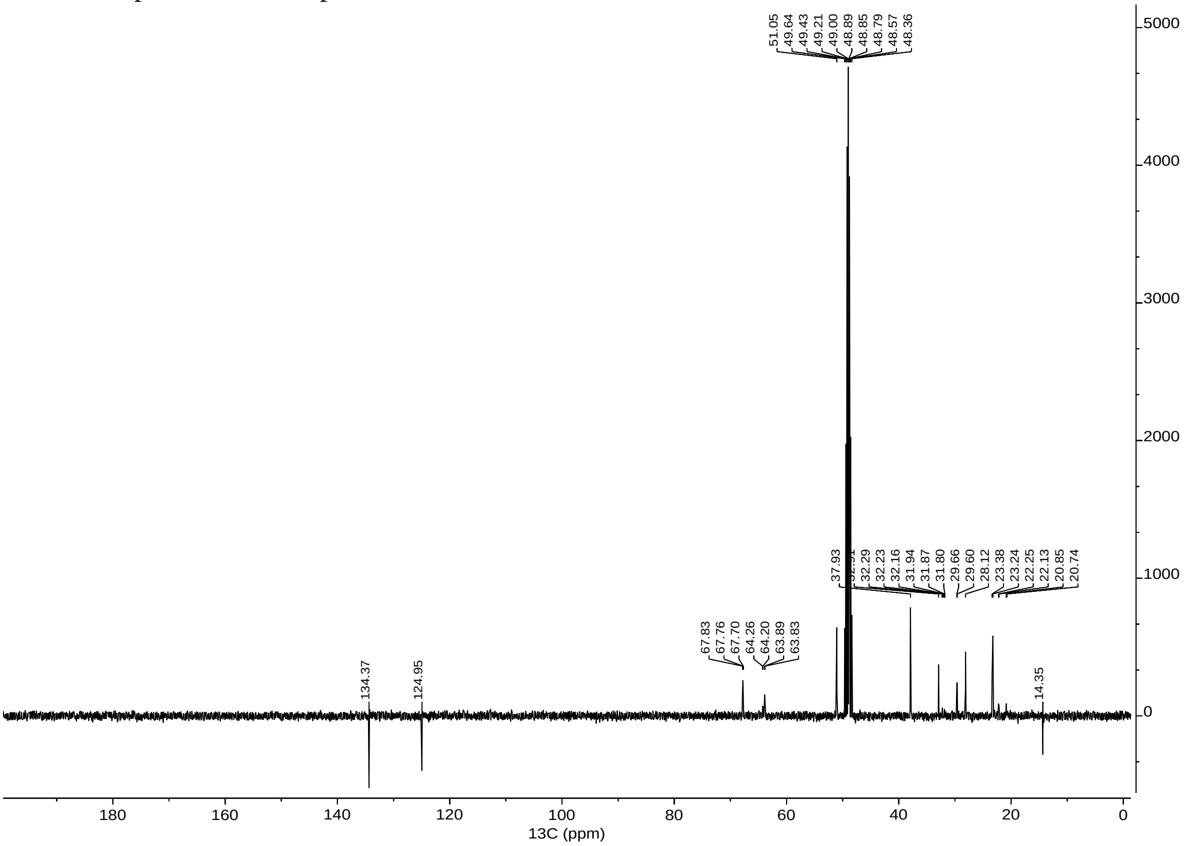

^{31}P{^{1}H} NMR spectrum of compound **18**

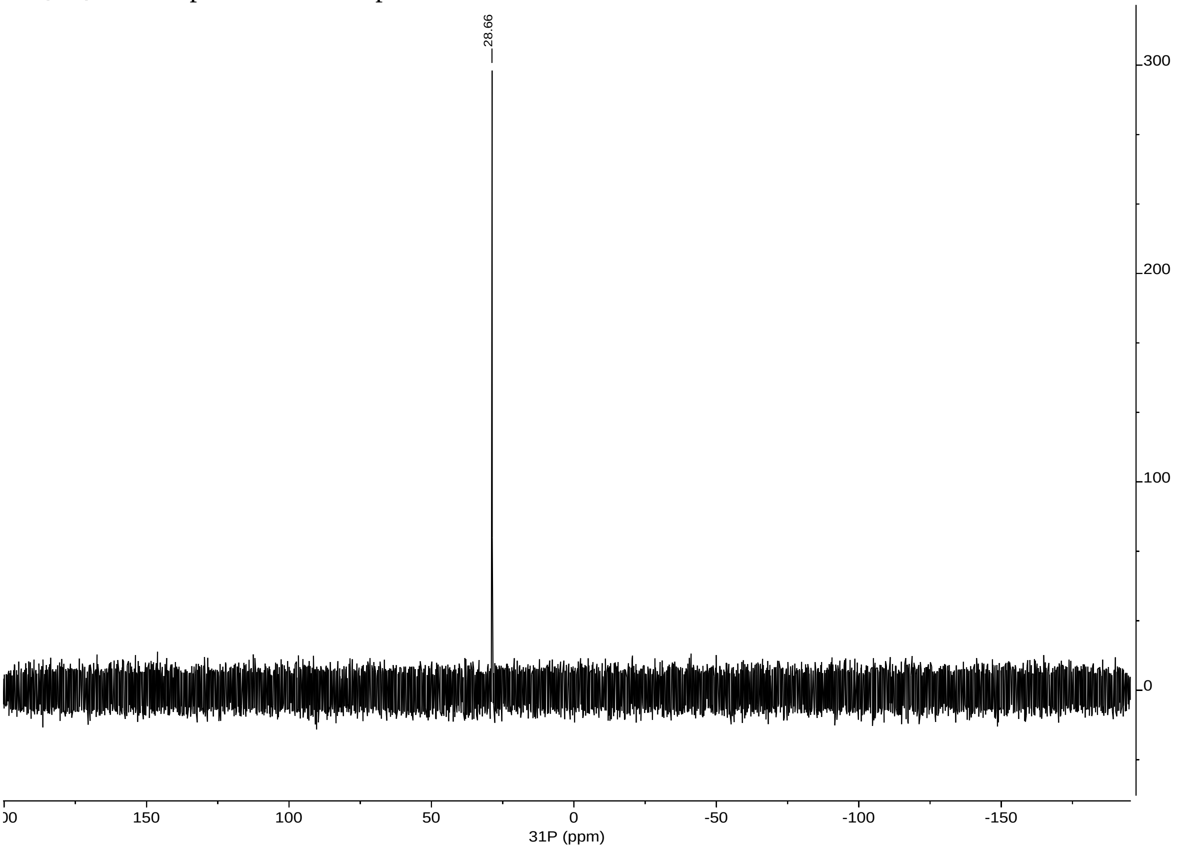

LC-MS chromatograms for compound **18**

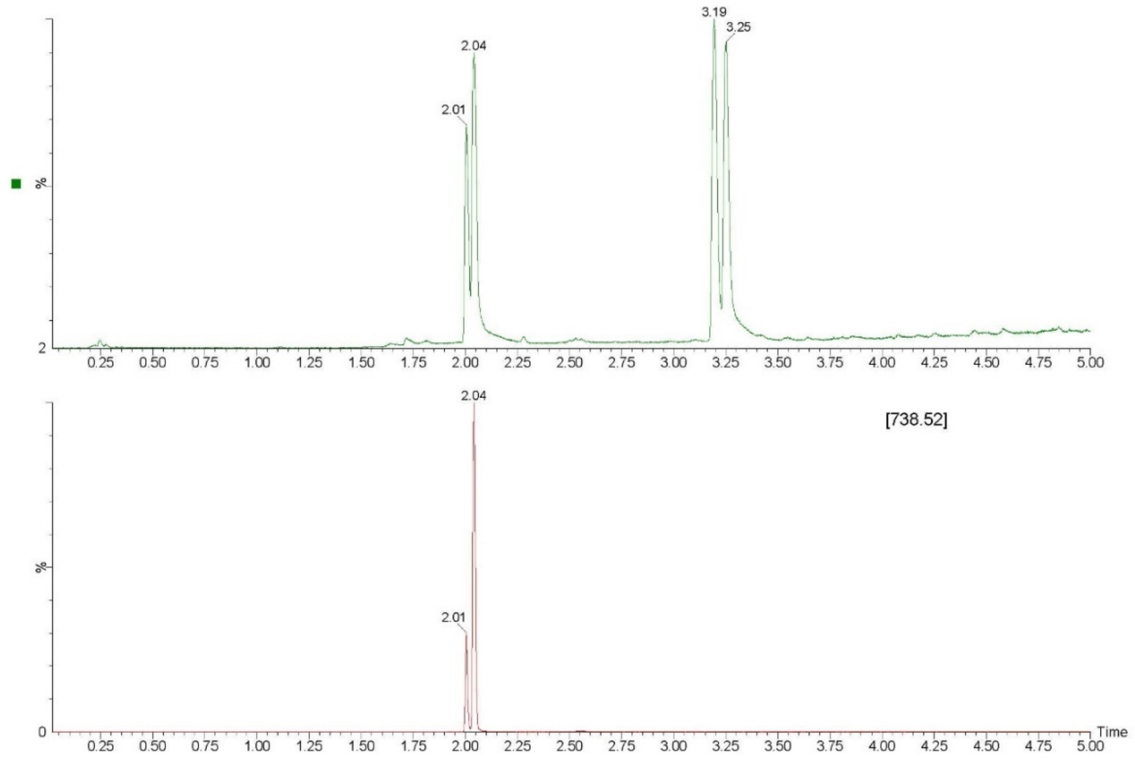

Bis((*Z*)-non-3-en-1-yl) propane-1,3-diyl bis((2-(bis(3-aminopropyl)amino)ethyl)phosphonate) hexahydrochloride (19)

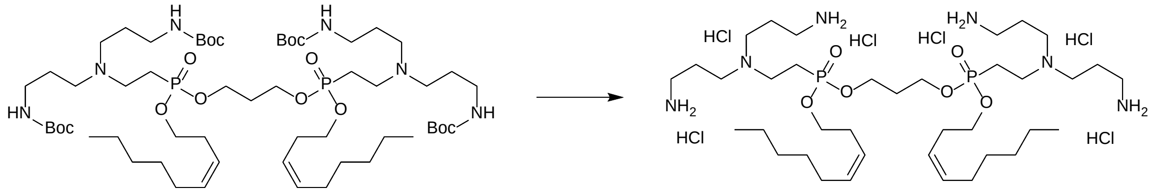

The title compound was prepared according to general method **E** from Boc derivative **S95** (1.25 g, 1.07 mmol) in 97% yield (1.03 g, 1.04 mmol) as a white solid.

Mixture of diastereoisomers.

^{1}H NMR (401 MHz, CD_3OD): 5.64–5.51 (m, 2H, $CH_2(CH_2)_4CH$), 5.48–5.36 (m, 2H, $CH(CH_2)_2O$), 4.41–4.19 (m, 4H, $OCH_2CH_2CH_2O$), 4.19–4.10 (m, 4H, $CHCH_2CH_2O$), 3.56–3.46 (m, 4H, PCH_2CH_2), 3.43 (t, 8H, J = 8.2 Hz, $CH_2(CH_2)_2NH_2$), 3.13 (t, 8H, J = 7.5 Hz, CH_2NH_2), 2.74–2.58 (m, 4H, PCH_2), 2.49 (q, 4H, J = 6.8 Hz, $CHCH_2CH_2O$), 2.27–2.19 (p, 8H, J = 7.9 Hz, $CH_2CH_2NH_2$), 2.17–2.04 (m, 6H, $OCH_2CH_2CH_2O$, $CH_2(CH_2)_3CH_2$), 1.47–1.23 (m, 12H, $CH_2(CH_2)_3$), 0.97–0.86 (m, 6H, CH_3).

^{13}C NMR (101 MHz, CD_3OD): 134.40 ($CH_3(CH_2)_4CH$), 124.93 ($CH(CH_2)_2O$), 67.78, (d, J = 6.4 Hz), 67.72 (d, J = 6.5 Hz, $CHCH_2CH_2O$), 64.21 (d, J = 6.3 Hz), 63.83 (d, J = 6.5 Hz, $OCH_2CH_2CH_2O$), 51.01 ($CH_2(CH_2)_2NH_2$), 48.90, 48.84 (PCH_2CH_2), 37.91 (CH_2NH_2), 32.66 ($CH_3CH_2CH_2$), 32.21 (t, J = 6.9 Hz), 31.85 (t, J = 6.7 Hz, $OCH_2CH_2CH_2O$), 30.40 ($CH_3(CH_2)_2CH_2$), 29.64 (d, J = 6.0 Hz, $CHCH_2CH_2O$), 28.38 ($CH_3(CH_2)_3CH_2$), 23.64 (CH_3CH_2), 23.21 ($CH_2CH_2NH_2$), 21.54 (d, J = 139.9 Hz, PCH_2), 21.42 (d, J = 139.7 Hz, PCH_2), 14.44 (CH_3).

^{31}P{^{1}H} NMR (162 MHz, CD_3OD): 28.88, 28.87.

IR ν_{max} 3200–2800 (s), 2958 (vs), 2928 (w), 2873 (s), 2858 (s), 2045 (w), 1650 (sh), 1612 (m), 1516 (sh), 1406 (m), 1380 (m), 1231 (s), 725 (sh).

HR-MS(ESI$^+$): For $C_{37}H_{82}O_6N_6P_2$ $(M+2H)^{2+}$ $m/2z$ calculated 384.28801, found 384.28792.

^{1}H NMR spectrum of compound **19**

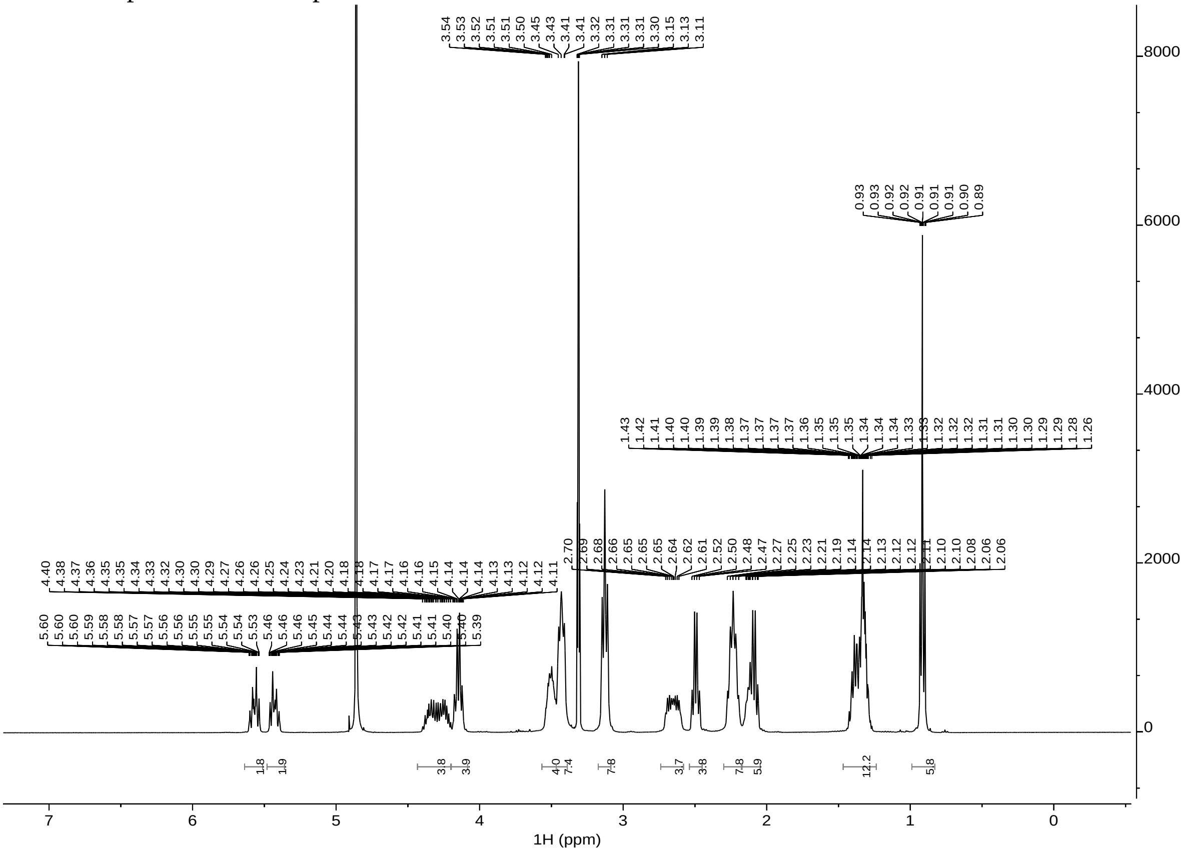

^{13}C NMR spectrum of compound **19**

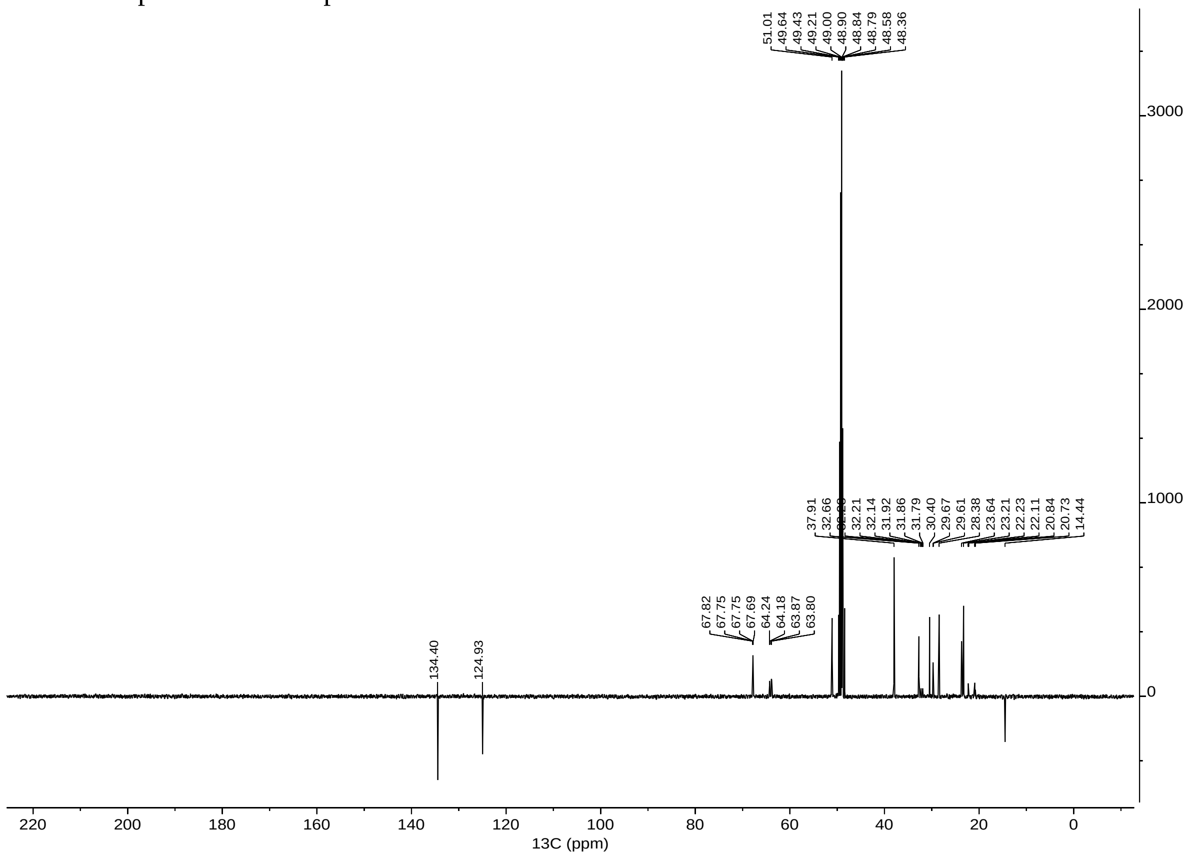

^{31}P{^{1}H} NMR spectrum of compound **19**

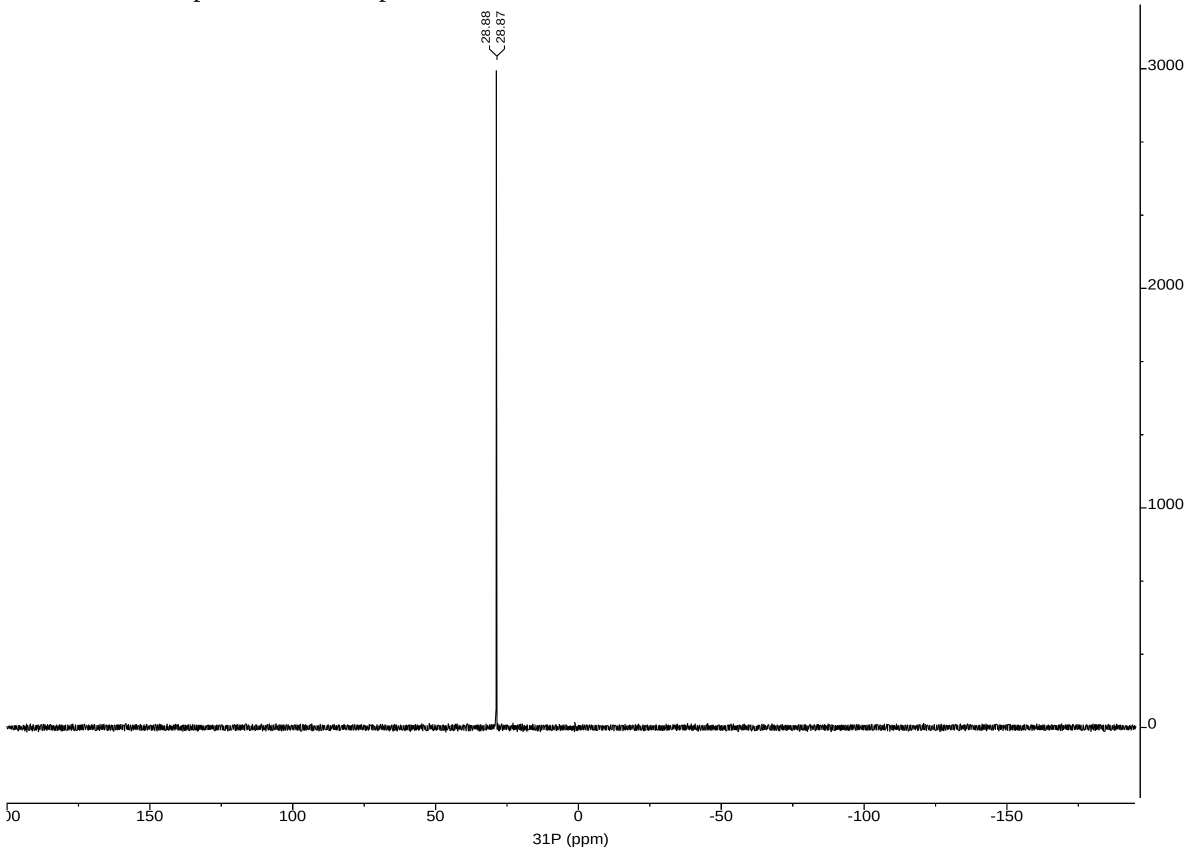

LC-MS chromatograms for compound **19**

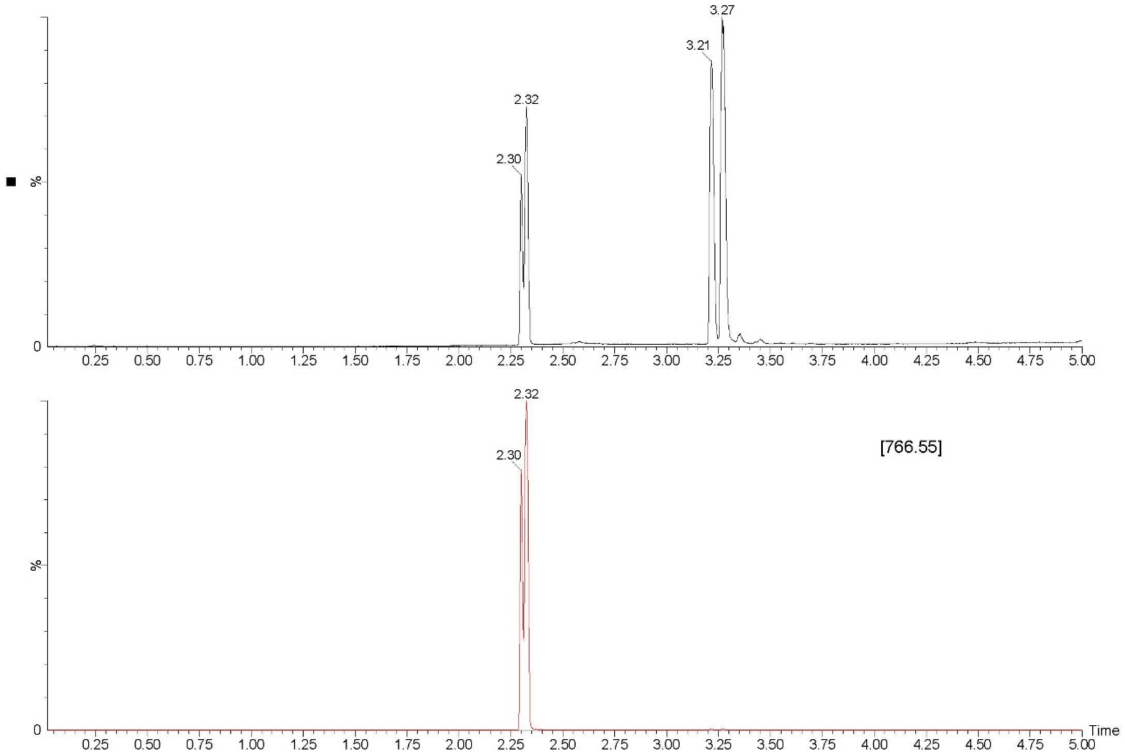

Didecyl propane-1,3-diyl bis((2-(bis(3-aminopropyl)amino)ethyl)phosphonate) hexahydrochloride (20)

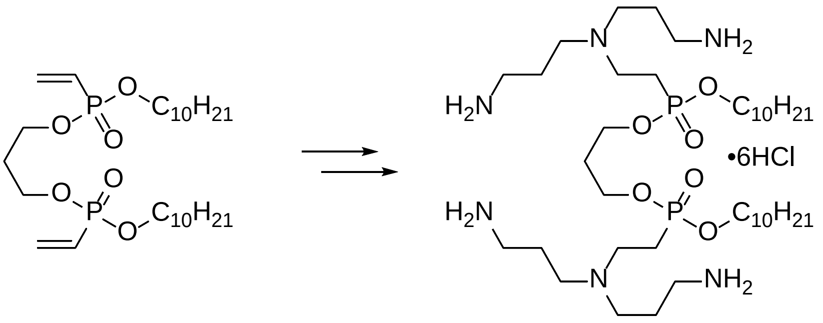

The title compound was prepared according to general methods **D** and **E** from vinylphosphonate dimer **S43** (1.10 g, 2.05 mmol) in 43% overall yield (0.91 g, 0.89 mmol) as a white solid.
Mixture of diastereoisomers.
^{1}H NMR (500.2 MHz, CD_3OD): 4.43–4.19 (m, 4H, OCH_2CH$_2$CH_2O), 4.19–4.11 (m, 4H, OCH_2(CH$_2$)$_8$CH$_3$), 3.51 (dq, 4H, J = 11.5, 6.3, 5.3 Hz, PCH$_2$CH_2), 3.46–3.40 (m, 8H, CH_2(CH$_2$)$_2$NH$_2$), 3.13 (t, 8H, J = 7.4 Hz, CH_2NH$_2$), 2.71–2.57 (m, 4H, PCH_2), 2.23 (p, 8H, J = 8.2, 7.6 Hz, CH_2CH$_2$NH$_2$), 2.13 (tt, 2H, J = 8.7, 4.4 Hz, OCH$_2$CH_2CH$_2$O), 1.77–1.69 (m, 4H, CH_2(CH$_2$)$_7$CH$_3$), 1.47–1.38 (m, 4H, CH_2(CH$_2$)$_6$CH$_3$), 1.38–1.24 (m, 24H, (CH_2)$_6$CH$_3$), 0.93–0.87 (m, 6H, CH_3).
^{13}C NMR (125.8 MHz, CD_3OD): 68.43 (d, J = 7.1 Hz), 68.37 (d, J = 6.9 Hz, OCH$_2$(CH$_2$)$_8$CH$_3$), 64.23 (d, J = 6.4 Hz), 63.85 (d, J = 6.4 Hz, OCH$_2$CH$_2$$CH_2$O), 51.04 ($CH_2$(CH$_2$)$_2NH_2$), 48.95, 48.90 (PCH$_2$$CH_2$), 37.92 ($CH_2NH_2$), 33.06 ($CH_2CH_2CH_3$), 32.24 (t, J = 7.0 Hz), 31.90 (t, J = 7.0 Hz, OCH$_2$$CH_2CH_2$O), 31.64 (d, J = 6.1 Hz, CH$_2$(CH$_2$)$_7$CH$_3$), 30.71, 30.46, 30.36, 26.64, 23.73 (CH$_2$)$_6$CH$_3$), 23.22 (CH$_2$CH$_2$NH$_2$), 21.53 (d, J = 140.1 Hz), 21.42 (d, J = 139.9 Hz, PCH$_2$), 14.43 (CH$_3$).
^{31}P{^{1}H} NMR (202.5 MHz, CD_3OD) δ 26.81.
IR ν_{max} (KBr) 2957 (vs), 2926 (vs), 2855 (vs), 2745 (s, sh), 2639 (s), 2559 (m), 2040 (w, br), 1609 (m), 1517 (m, br, sh), 1468 (s), 1402 (m), 1379 (m), 1257 (s, br, sh), 1230 (s), 1062 (s, sh), 1015 (vs), 988 (vs).
HR-MS(ESI$^+$): For $C_{39}H_{89}N_6O_6P_2$ (M+H)$^+$ m/z calculated 799.63133, found 799.63190.

^{1}H NMR spectrum of compound **20**

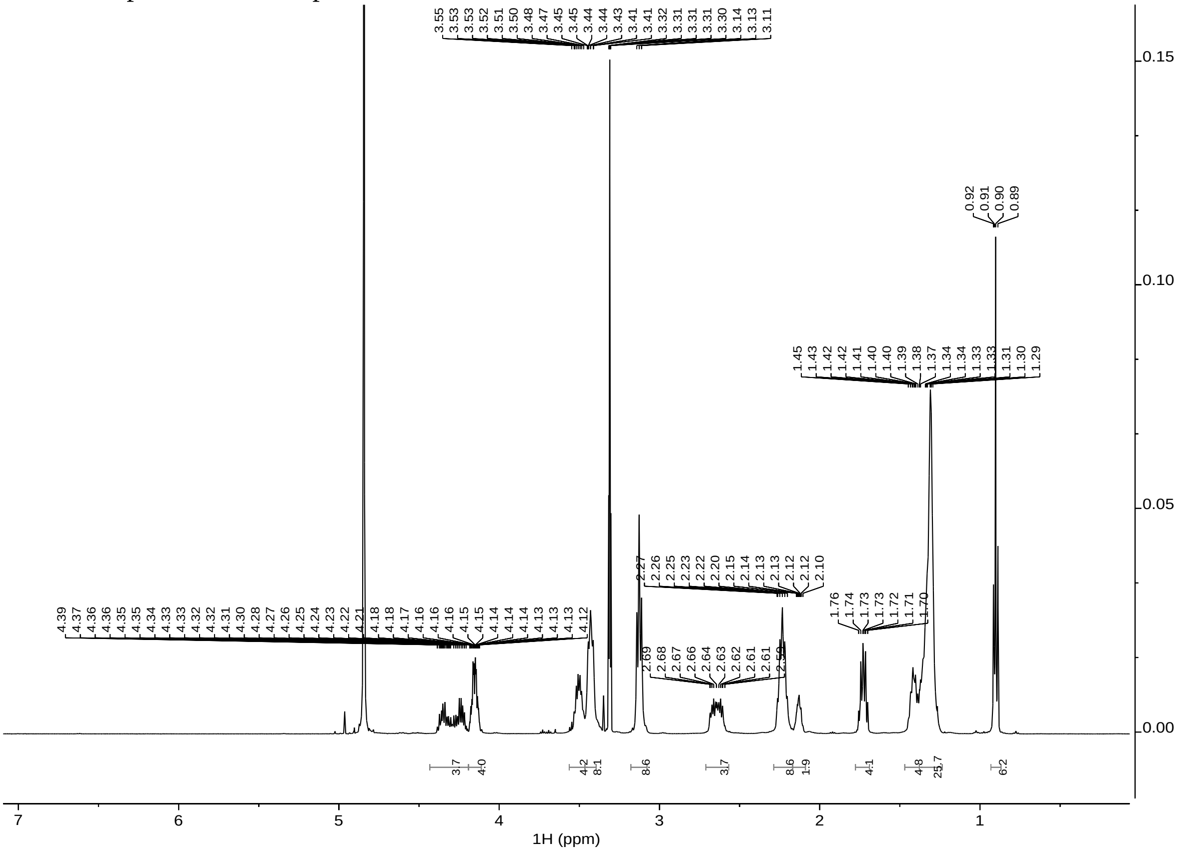

^{13}C NMR spectrum of compound **20**

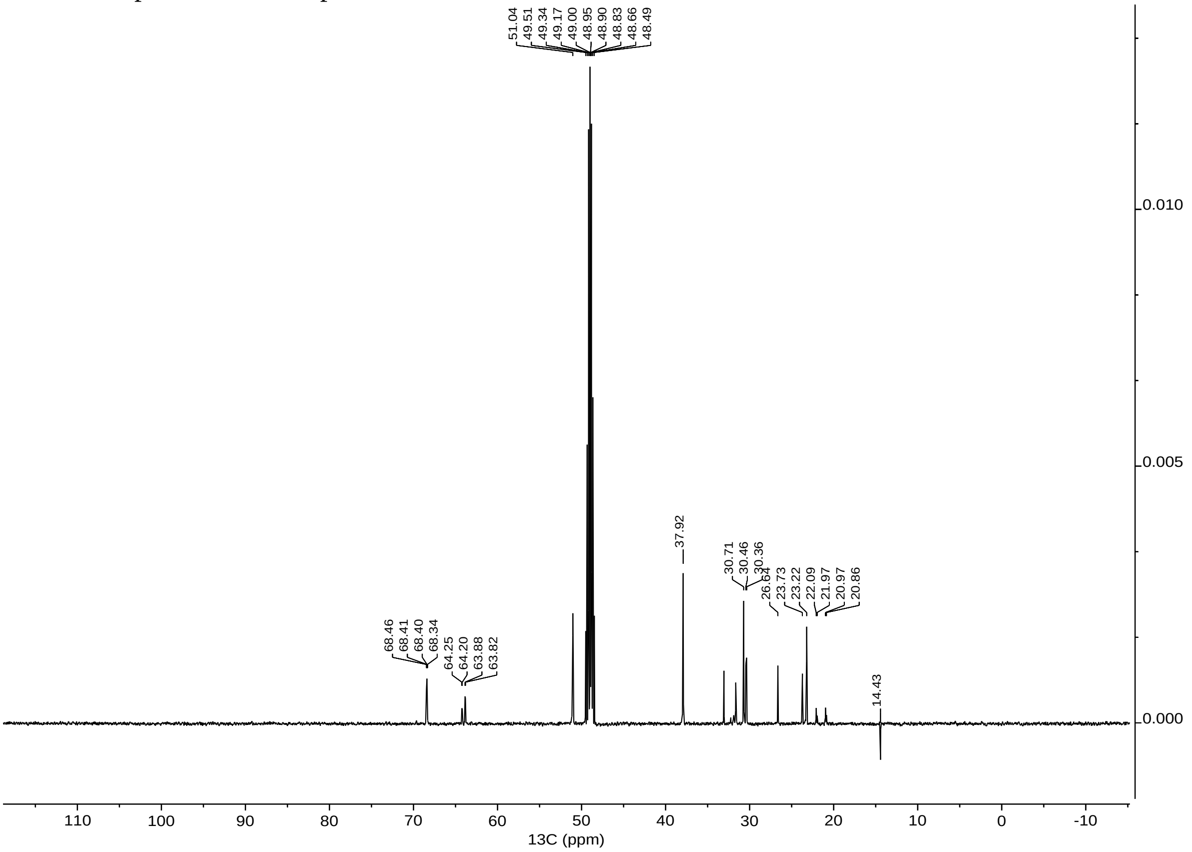

$^{31}P\{^1H\}$ NMR spectrum of compound **20**

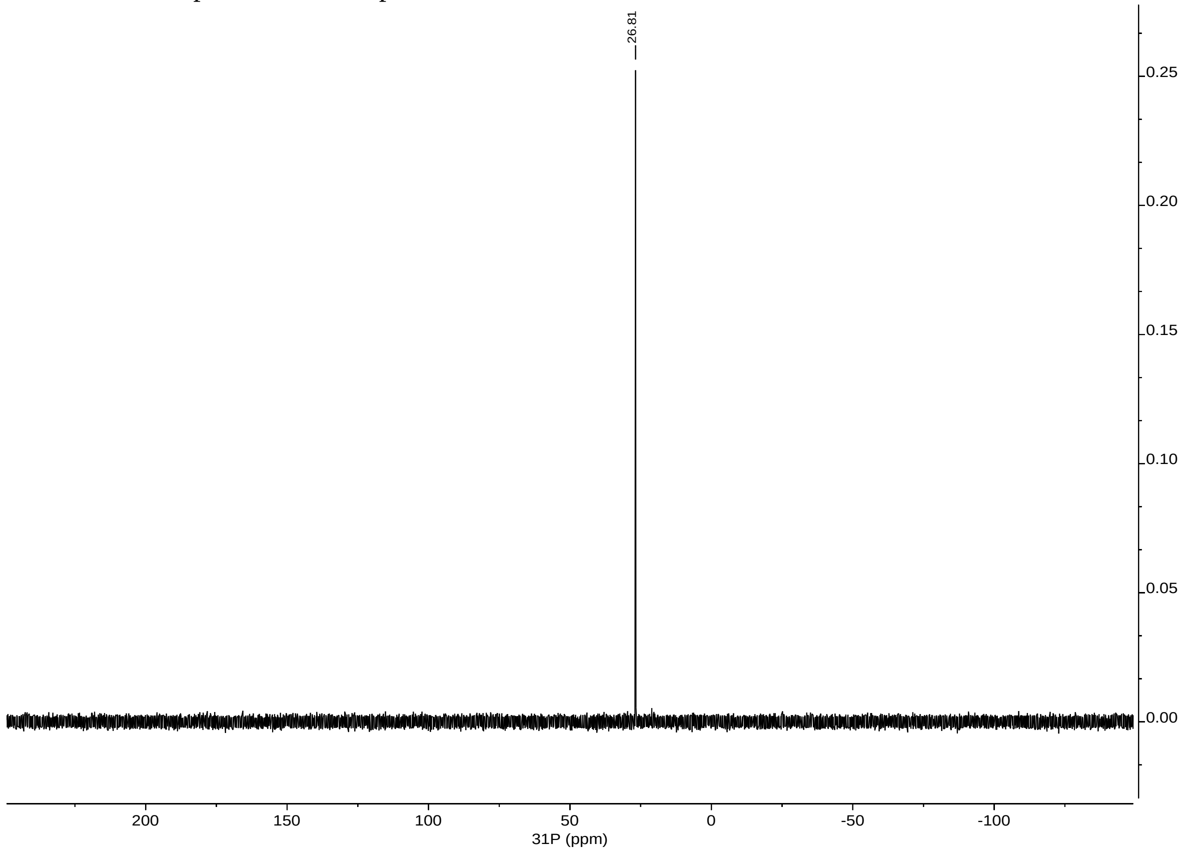

LC-MS chromatograms for compound **20**

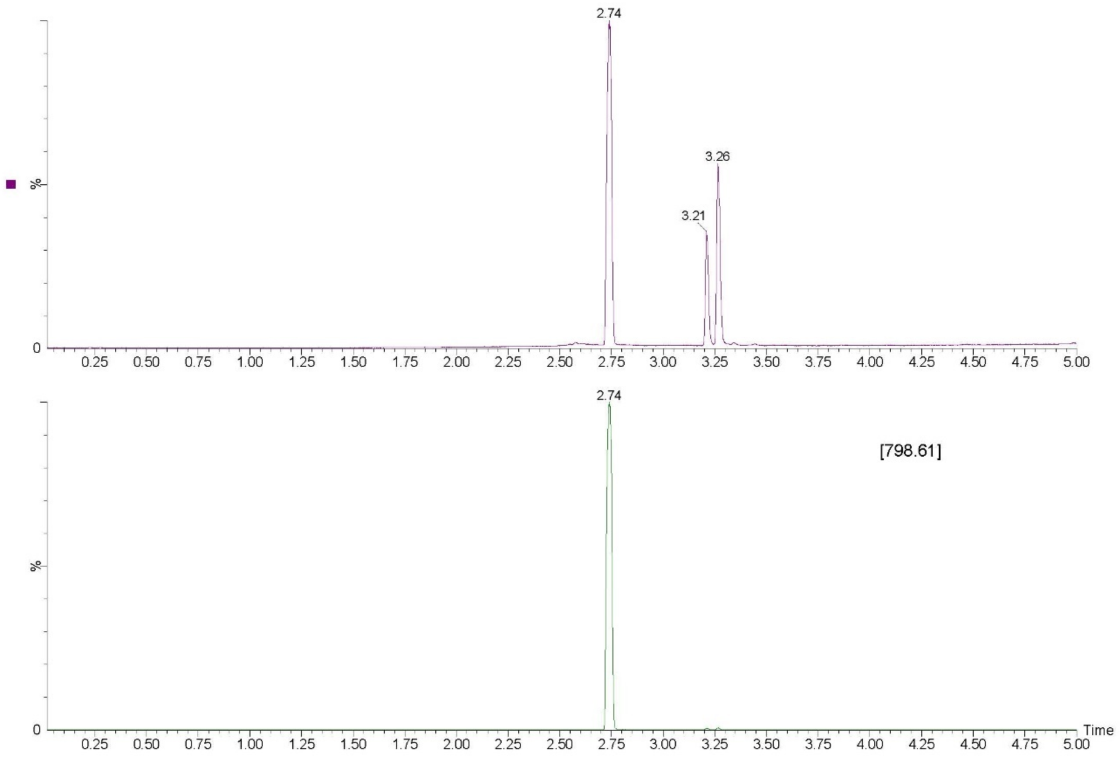

Bis((*Z*)-dec-4-en-1-yl) propane-1,3-diyl bis((2-(bis(3-aminopropyl)amino)ethyl)phosphonate) hexahydrochloride (21)

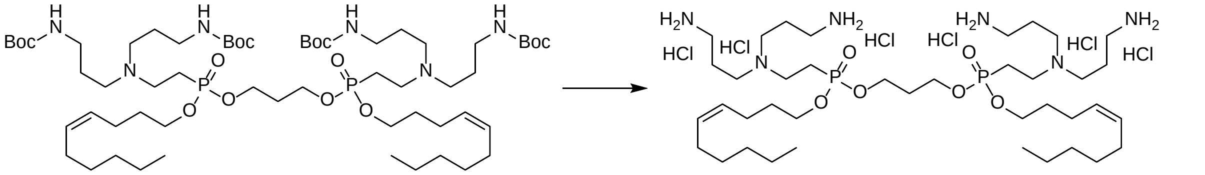

The title compound was prepared according to general method **E** from **S96** (0.61 g, 0.57 mmol) in 97% yield (0.51 g, 0.50 mmol) as a white solid.
Mixture of diastereoisomers.
^{1}H NMR (401 MHz, CD_3OD): 5.51–5.33 (m, 4H, C*H*C*H*$(CH_2)_3O$), 4.42–4.12 (m, 8H, C*H*$_2$O), 3.56–3.37 (m, 12H, C*H*$_2(CH_2)_2NH_2$, PCH_2C*H*$_2$), 3.13 (t, 8H, *J* = 7.4 Hz, C*H*$_2NH_2$), 2.74–2.56 (m, 4H, PC*H*$_2$), 2.30–2.10 (m, 14H, CHC*H*$_2(CH_2)_2O$, OCH_2C*H*$_2CH_2O$, C*H*$_2CH_2NH_2$), 2.06 (q, 4H, *J* = 6.8 Hz, $CH_3(CH_2)_3$C*H*$_2$), 1.78 (p, 4H, *J* = 6.8 Hz, $CHCH_2$C*H*$_2CH_2O$), 1.43–1.26 (m, 12H, CH_3(C*H*$_2)_3$), 0.97–0.84 (m, 6H, C*H*$_3$).
^{13}C NMR (101 MHz, CD_3OD): 132.22 ($CH_3(CH_2)_4$*C*H), 129.07 (*C*H$(CH_2)_3O$), 67.91 (d, *J* = 6.4 Hz), 67.85 (d, *J* = 6.3 Hz, $CH(CH_2)_2$*C*H$_2$O), 64.21 (d, *J* = 6.2 Hz), 63.81 (d, *J* = 6.5 Hz, O*C*H$_2CH_2$*C*H$_2$O), 48.24 (*C*H$_2(CH_2)_2NH_2$), 48.91, 48.89 (PCH_2*C*H$_2$), 37.92 (*C*H$_2NH_2$), 32.66 (CH_3CH_2*C*H$_2$), 32.26 (t, *J* = 5.1 Hz), 31.91 (t, *J* = 6.6 Hz, OCH_2*C*H$_2CH_2O$), 31.72 (d, *J* = 6.1 Hz, $CHCH_2$*C*H$_2CH_2O$), 30.49 ($CH_3(CH_2)_2$*C*H$_2$), 28.19 ($CH_3(CH_2)_3$*C*H$_2$), 24.17 (CH*C*H$_2(CH_2)_2O$), 23.65 (CH_3*C*H$_2$), 22.23 (*C*H$_2CH_2NH_2$), 21.54 (d, *J* = 140.4 Hz), 21.42 (d, *J* = 139.5 Hz, P*C*H$_2$), 14.45 (*C*H$_3$).
^{31}P{^{1}H} NMR (162 MHz, CD_3OD): 28.96, 28.93.
IR ν_{max} 3200–2400 (s), 3013 (s, sh), 2956 (s), 2926 (vs), 2873 (s), 2011 (w), 1657 (vw), 1600 (w), 1467 (m), 1379 (w), 1224 (m), 843 (m), 754 (m).
HR-MS(ESI^{+}): For $C_{39}H_{85}O_6N_6P_2$ $(M+H)^{+}$ *m*/*z* calculated 795.60003, found 795.60019.

^{1}H NMR spectrum of compound **21**

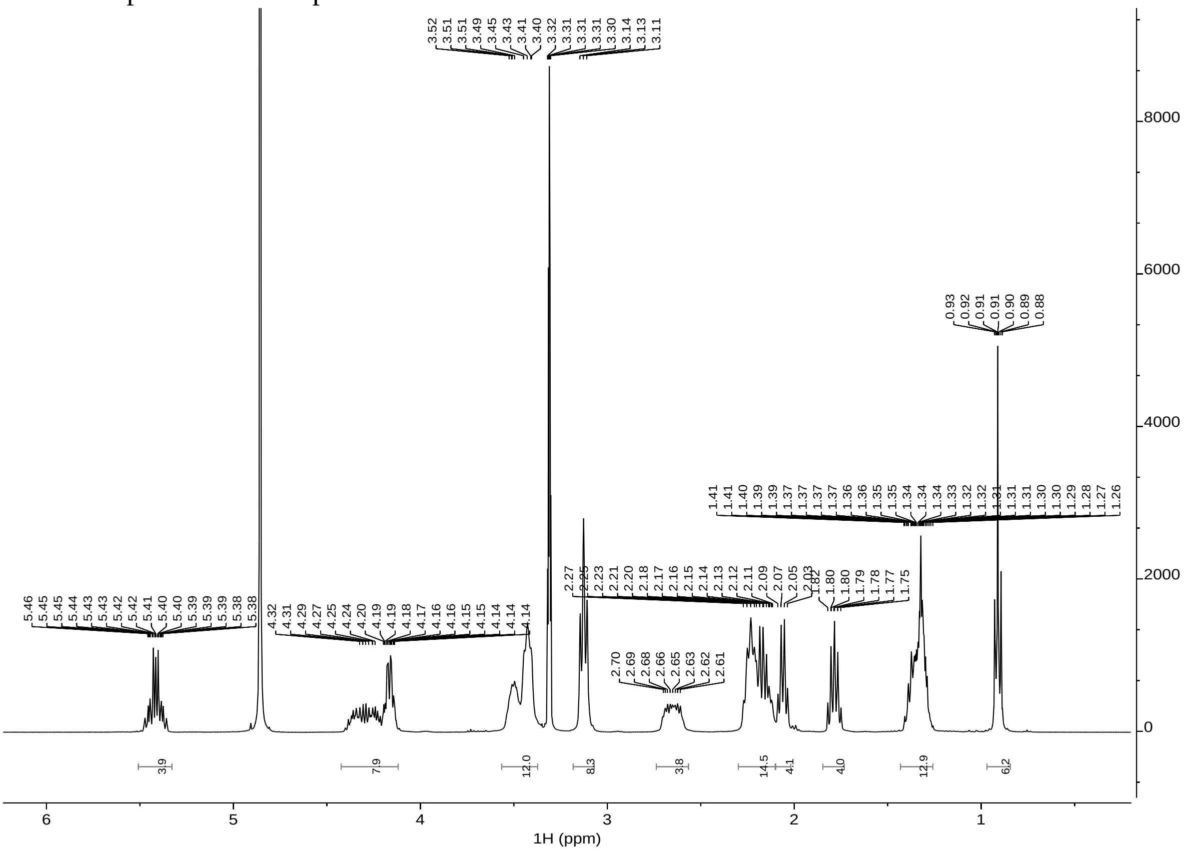

^{13}C NMR spectrum of compound **21**

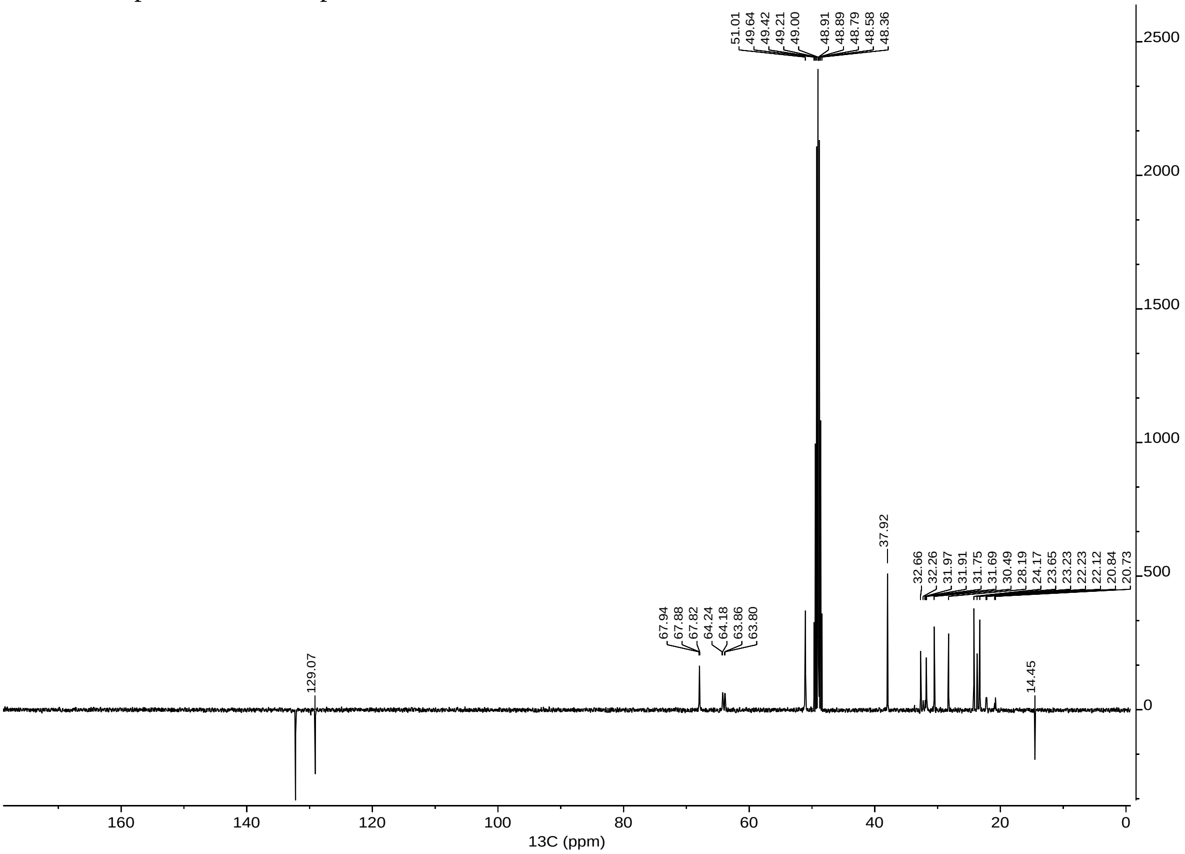

$^{31}P\{^{1}H\}$ NMR spectrum of compound **21**

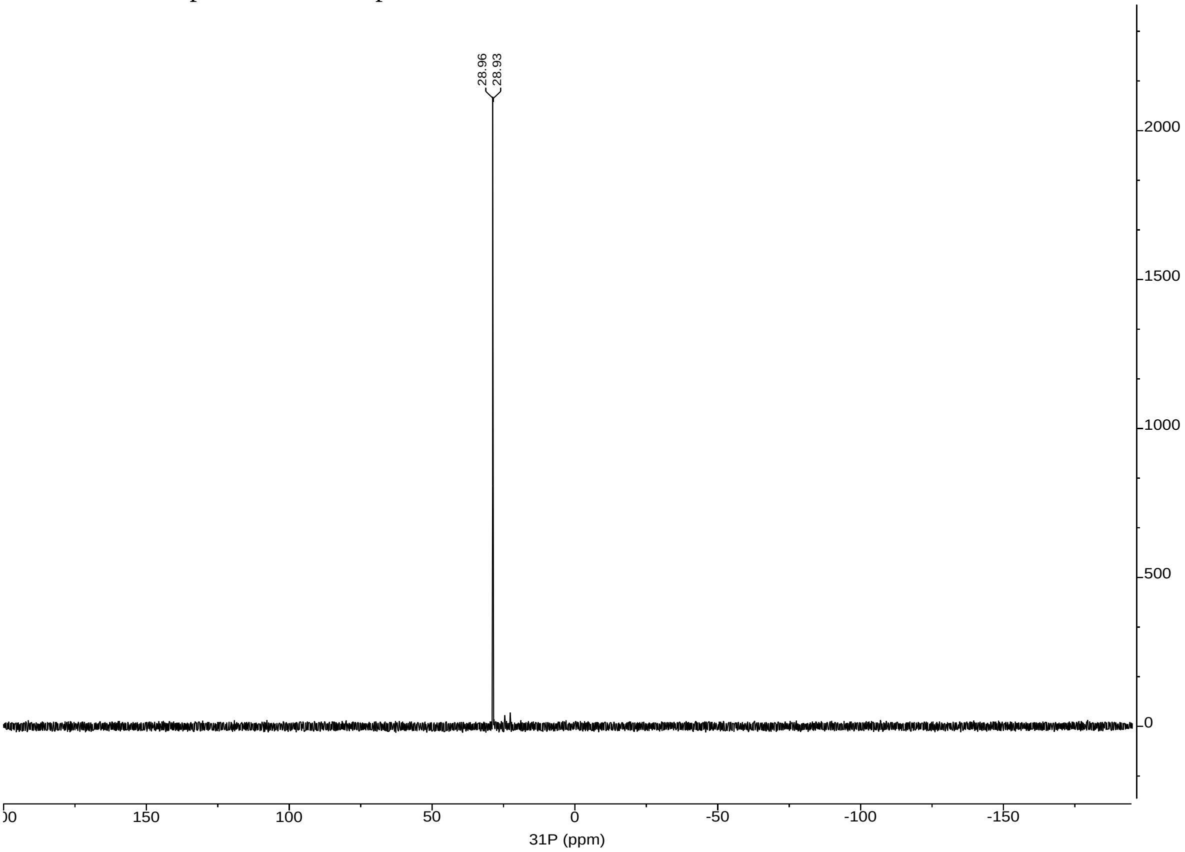

LC-MS chromatograms for compound **21**

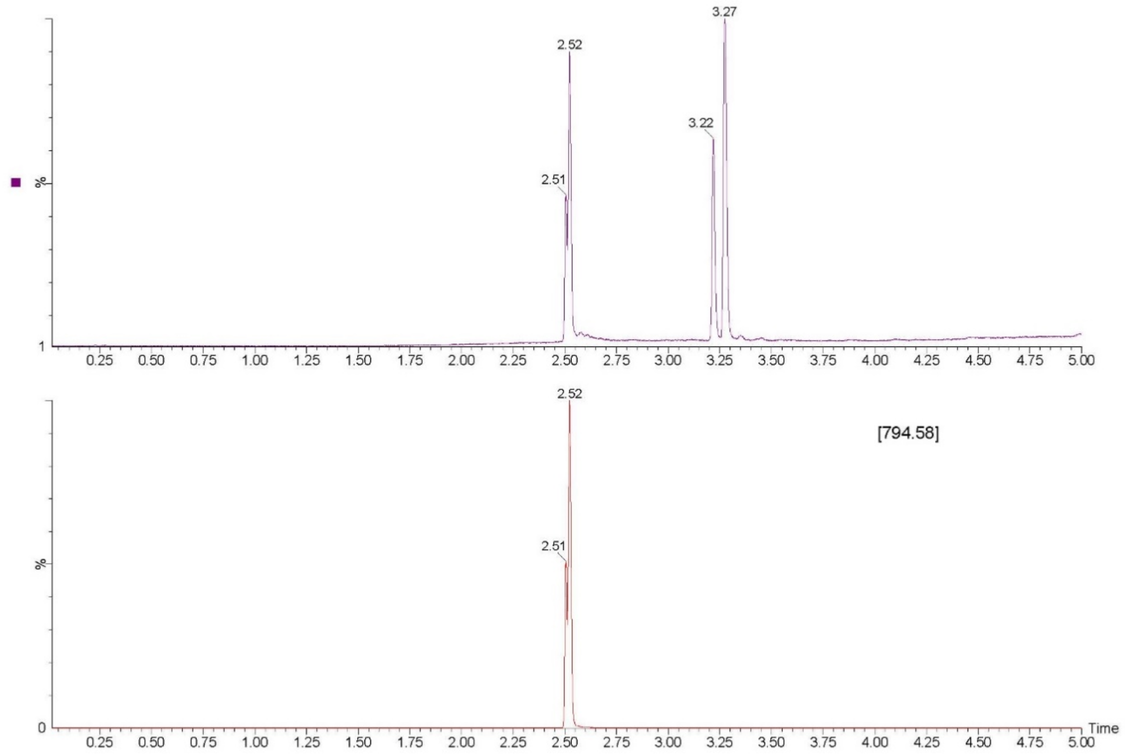

Bis((adamantan-1-yl)methyl) propane-1,3-diyl bis((2-(bis(3-aminopropyl)amino)ethyl)phosphonate) hexahydrochloride (22)

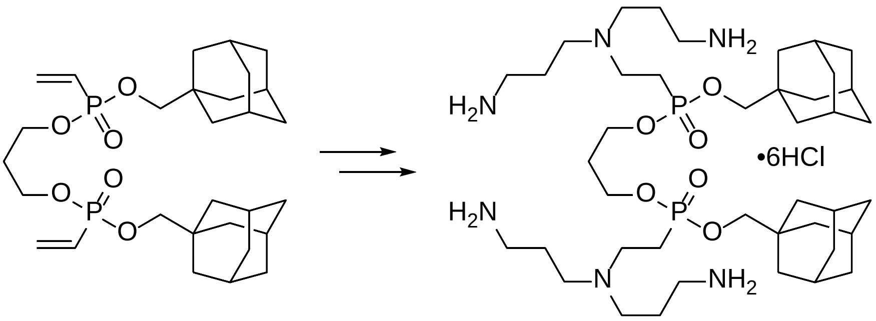

The title compound was prepared according to general methods **D** and **E** from vinylphosphonate dimer **S45** (1.80 g, 3.26 mmol) in 34% overall yield (1.16 g, 1.12 mmol) as a white solid.
Mixture of diastereoisomers A:B ~ 8:3.
^{1}H NMR (500.2 MHz, CD_3OD): 4.41–4.21 (m, 8H, OC*H*$_2$CH$_2$-A,B), 3.76–3.67 (m, 8H, O-CH$_2$-adamantane-A,B), 3.55–3.47 (m, 8H, PCH$_2$C*H*$_2$-A,B), 3.47–3.40 (m, 16H, C*H*$_2$(CH$_2$)$_2$NH$_2$-A,B), 3.13 (bt, 16H, J = 7.3 Hz, C*H*$_2$NH$_2$-A,B), 2.72–2.58 (m, 8H, PC*H*$_2$-A,B), 2.28–2.20 (m, 16H, C*H*$_2$CH$_2$NH$_2$-A,B), 2.18–2.11 (m, 4H, OCH$_2$C*H*$_2$-A,B), 2.03–1.98 (m, 12H, H-3,5,7-adamantane-A,B), 1.74–1.68, 1.83–1.76 (2×m, 2×12H, H-4,6,10-adamantane-A,B), 1.65–1.58 (m, 24H, H-2,8,9-adamantane-A,B).
^{13}C NMR (125.8 MHz, CD_3OD): 77.55 (d, J = 7.2 Hz, O-*C*H$_2$-adamantane-A), 77.47 (d, J = 7.3 Hz, O-*C*H$_2$-adamantane-B), 64.34 (d, J = 6.4 Hz, O*C*H$_2$CH$_2$-B), 63.96 (d, J = 6.4 Hz, O*C*H$_2$CH$_2$-A), 51.05 (*C*H$_2$(CH$_2$)$_2$NH$_2$-A,B), 48.98 (PCH$_2$*C*H$_2$-A,B, overlapped by CD_3OD), 39.87 (*C*H-2,8,9-adamantane-A,B), 37.92 (*C*H-4,6,10-adamantane-A,B, *C*H$_2$NH$_2$-A,B), 32.33 (t, J = 6.6 Hz, OCH$_2$*C*H$_2$-B), 31.95 (t, J = 7.0 Hz, OCH$_2$*C*H$_2$-A), 29.45 (*C*H-3,5,7-adamantane-A,B), 29.45 (*C*H-3,5,7-adamantane-A,B), 23.24 (*C*H$_2$CH$_2$NH$_2$-A,B), 21.39 (d, J = 140.1 Hz, P*C*H$_2$-A), 21.29 (d, J = 139.4 Hz, P*C*H$_2$-B).
^{31}P{^{1}H} NMR (202.5 MHz, CD_3OD): 27.32.
IR ν_{max} (KBr) 2975 (s, br, sh), 2932 (s, sh), 2903 (vs), 2847 (s), 2675, 2656, 2636, 2035 (w, vbr), 1617 (m, br), 1517 (w, br, sh), 1464 (m), 1455 (m), 1404 (w, br), 1365 (vw), 1344 (vw), 1262 (m, sh), 1240 (m), 1226 (m), 1106 (vw), 1061 (m), 1015 (s), 999 (s), 987 (s), 975 (m, sh), 945 (w, sh), 923 (w), 809 (w), 437 (w).
HR-MS(ESI^{+}): For $C_{41}H_{81}N_6O_6P_2$ $(M+H)^+$ *m*/*z* calculated 815.56873, found 815.56785.

^{1}H NMR spectrum of compound **22**

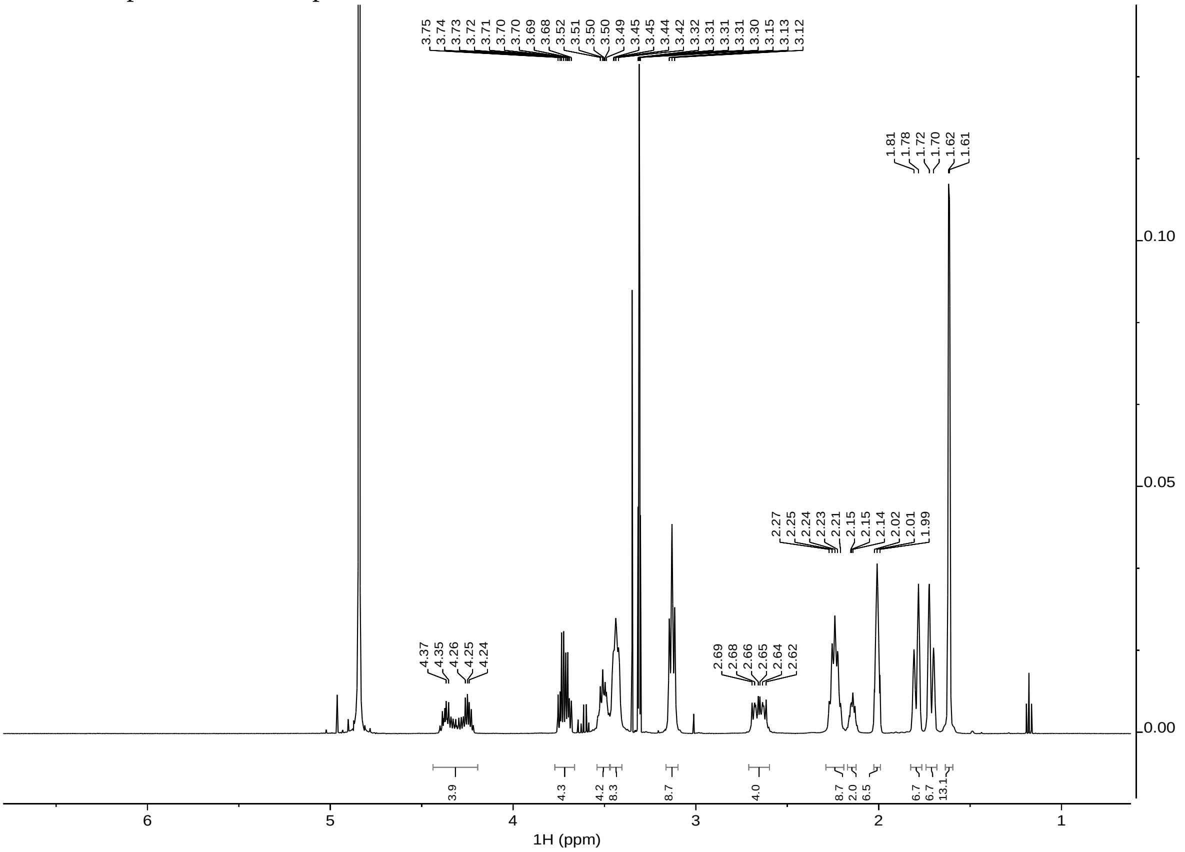

^{13}C NMR spectrum of compound **22**

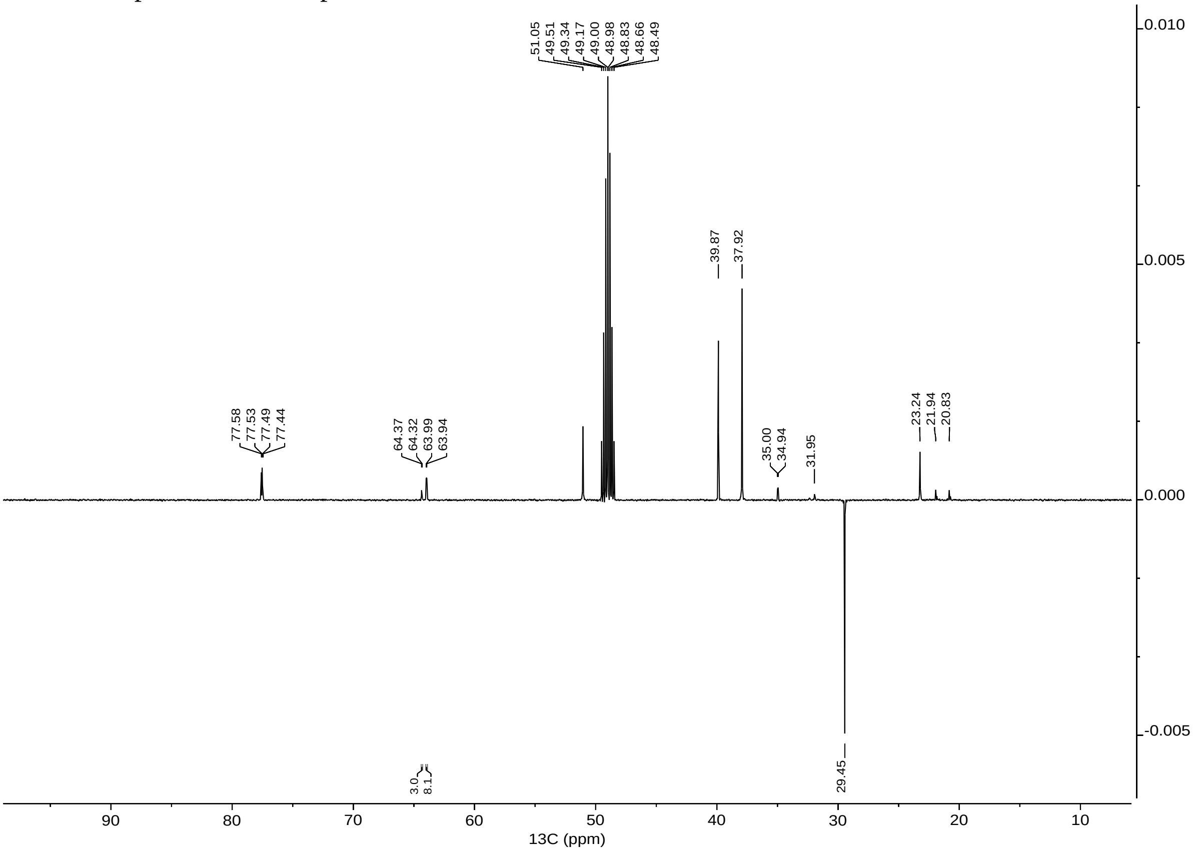

$^{31}P\{^{1}H\}$ NMR spectrum of compound **22**

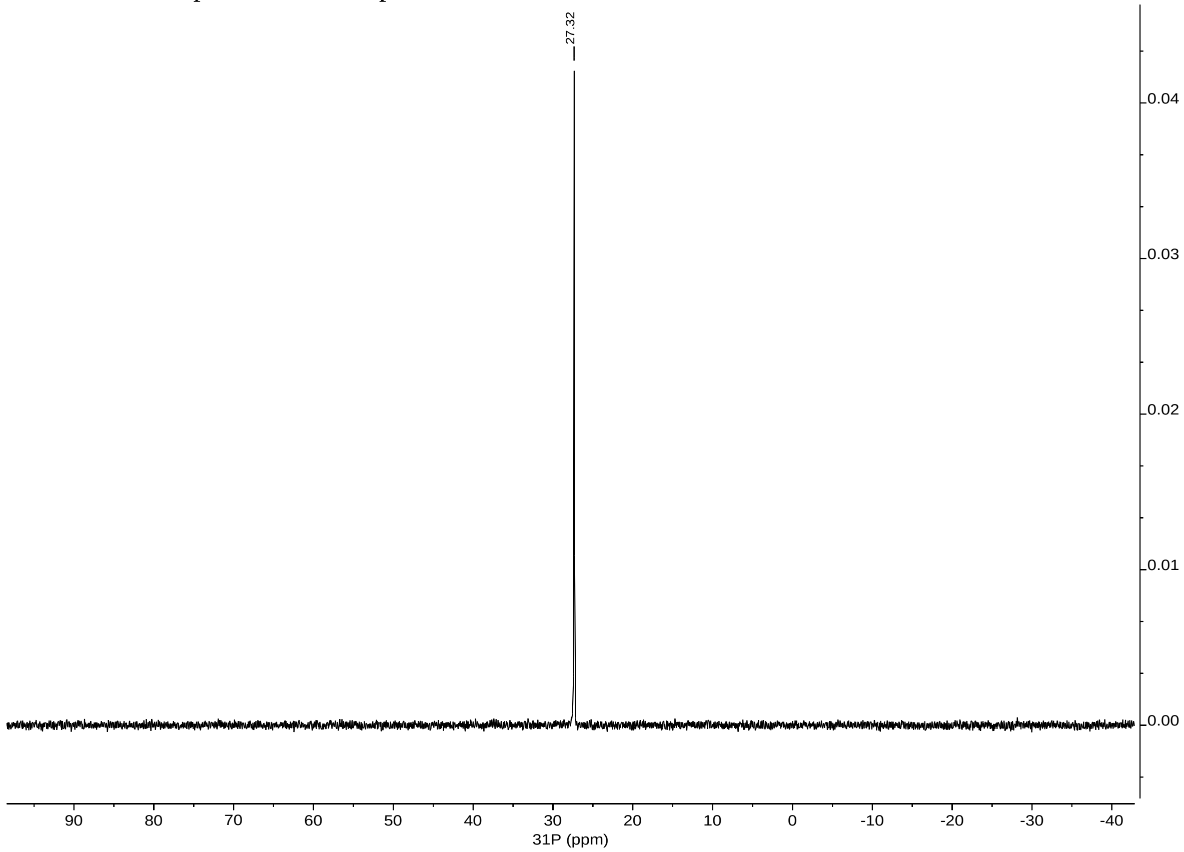

LC-MS chromatograms for compound **22**

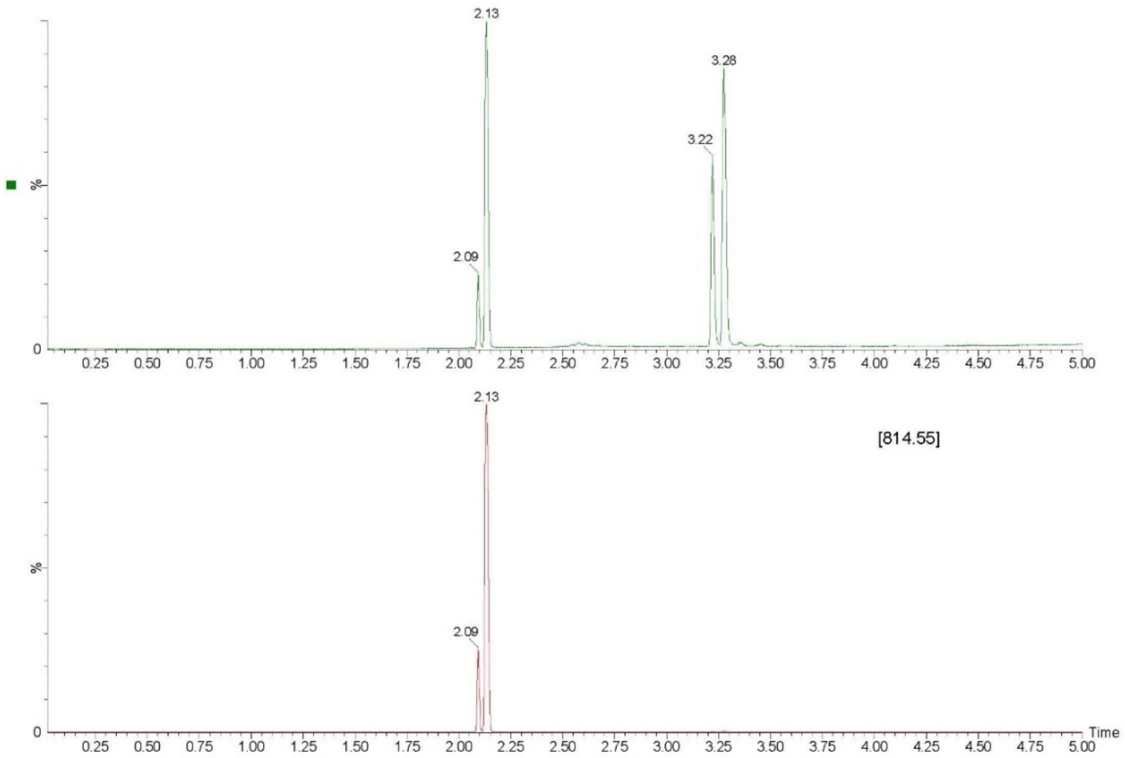

Bis(2-(adamantan-1-yl)ethyl) propane-1,3-diyl bis((2-(bis(3-aminopropyl)amino)ethyl)phosphonate) hexahydrochloride (23)

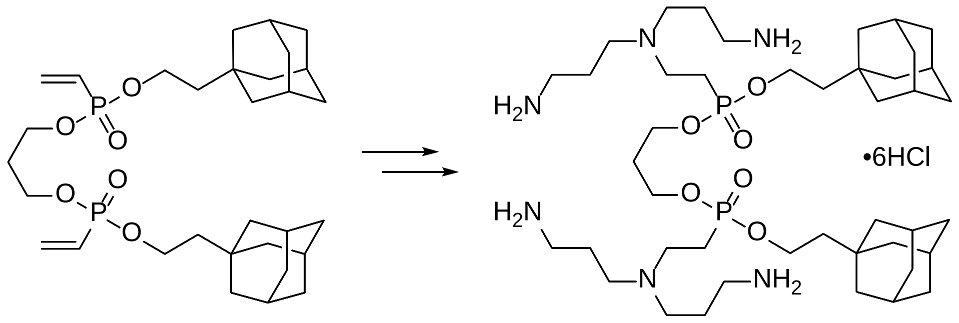

The title compound was prepared according to general methods **D** and **E** from vinylphosphonate dimer **S46** (0.62 g, 1.07 mmol) in 42% overall yield (0.48 g, 0.45 mmol) as a white solid.
Mixture of diastereoisomers.
^{1}H NMR (401 MHz, CD_3OD): 4.41–4.25 (m, 4H, OC*H*$_2$CH$_2$C*H*$_2$O), 4.28–4.16 (m, 4H, OC*H*$_2$CH$_2$C$_{quat}$), 3.54–3.46 (m, 4H, PCH$_2$C*H*$_2$), 3.46–3.39 (m, 8H, C*H*$_2$(CH$_2$)$_2$NH$_2$), 3.12 (t, *J* = 7.3 Hz, 8H, C*H*$_2$NH$_2$), 2.63 (dq, 4H, *J* = 16.2, 5.6 Hz, PC*H*$_2$), 2.22 (q, *J* = 8.0 Hz, 8H, C*H*$_2$CH$_2$NH$_2$), 2.17–2.10 (m, 2H, OCH$_2$C*H*$_2$CH$_2$O), 2.01–1.91 (m, 6H, C*H*), 1.81–1.65 (m, 12H, C$_{quat}$C*H*$_2$CH), 1.60 (d, 12H, *J* = 2.8 Hz, *c*-(CHC*H*$_2$)$_3$), 1.58–1.51 (m, 4H, OCH$_2$C*H*$_2$C$_{quat}$).
^{13}C NMR (101 MHz, CD_3OD): 64.83 (d, *J* = 6.4 Hz), 64.78 (d, *J* = 6.6 Hz, O*C*H$_2$CH$_2$C$_{quat}$), 64.27 (d, *J* = 6.4 Hz), 63.93 (d, *J* = 6.4 Hz, O*C*H$_2$CH$_2$*C*H$_2$O), 51.03 (*C*H$_2$(CH$_2$)$_2$NH$_2$), 48.91 (PCH$_2$*C*H$_2$), 45.56 (d, *J* = 5.5 Hz, OCH$_2$*C*H$_2$C$_{quat}$), 43.60 (*c*-(CH*C*H$_2$)$_3$), 38.01 (C$_{quat}$*C*H$_2$CH), 37.92 (*C*H$_2$NH$_2$), 32.91 (*C*$_{quat}$), 32.32 (d, *J* = 6.1 Hz), 31.98 (d, *J* = 5.4 Hz, OCH$_2$*C*H$_2$CH$_2$O), 30.03 (*C*H), 23.22 (*C*H$_2$CH$_2$NH$_2$), 21.57 (d, *J* = 139.9 Hz), 21.47 (d, *J* = 139.4 Hz, P*C*H$_2$).
^{31}P{^{1}H} NMR (162 MHz, CD_3OD): 28.93.
IR ν_{max} (KBr) 2950 (s, br), 2926 (vs, sh), 1906 (vs), 2847 (s), 1997 (vw, vbr), 2674, 1656, 2635, 2558, 1598 (w, br), 1509 (w, sh), 1470 (m), 1452 (m), 1404 (w, br), 1365 (vw, sh), 1345 (vw), 1252 (m, sh), 1228 (m), 1106 (w), 1072 (m), 1045 (m), 1013 (s), 998 (s), 987 (s), 972 (m, sh), 816 (w).
HR-MS(ESI$^+$): For $C_{43}H_{85}N_6O_6P_2$ $(M+H)^+$ *m*/*z* calculated 843.60003, found 843.59928.

^{1}H NMR spectrum of compound **23**

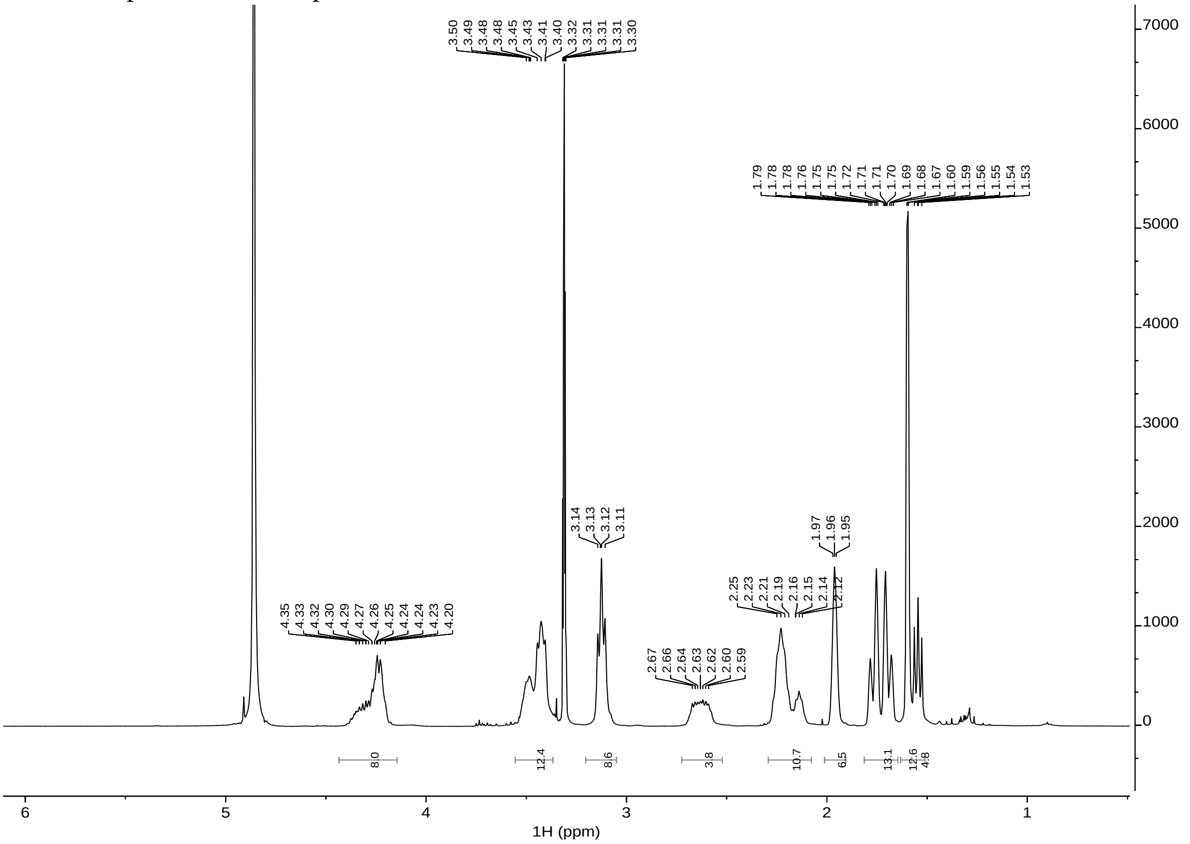

^{13}C NMR spectrum of compound **23**

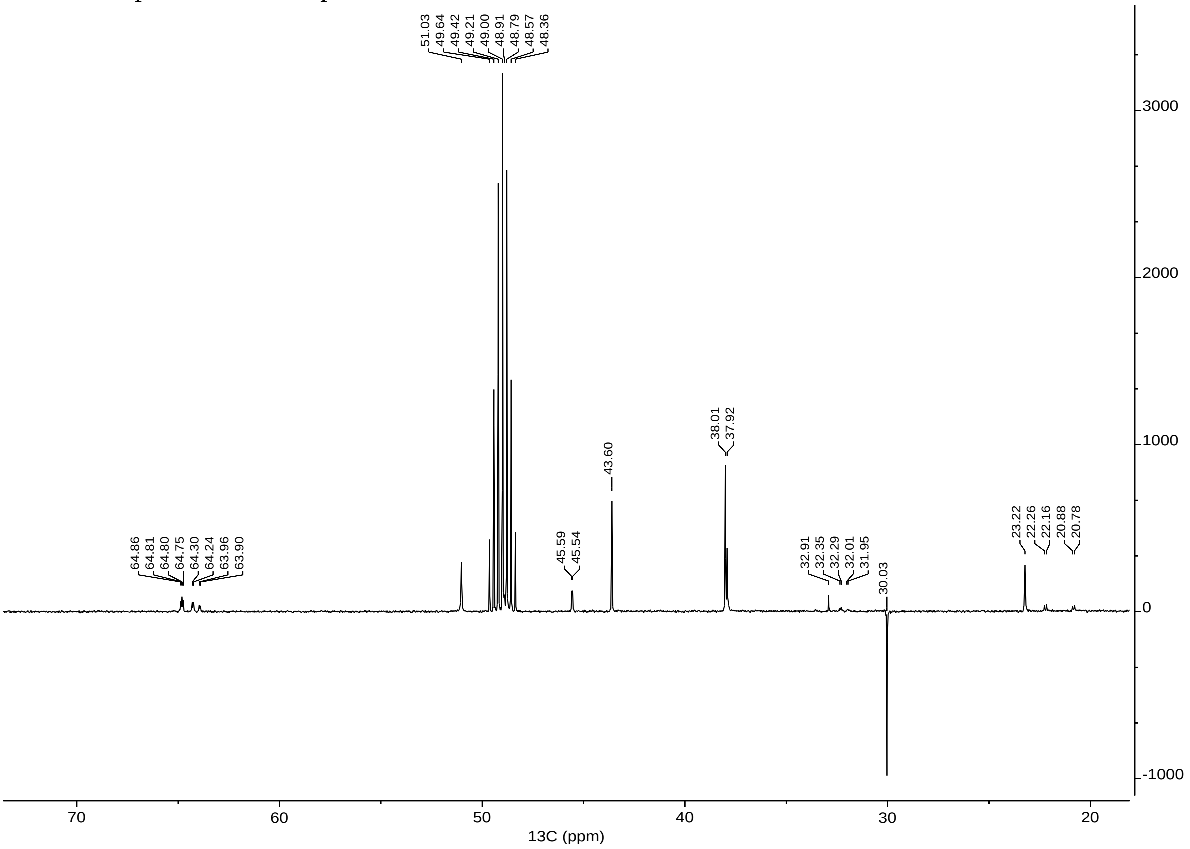

$^{31}P\{^{1}H\}$ NMR spectrum of compound **23**

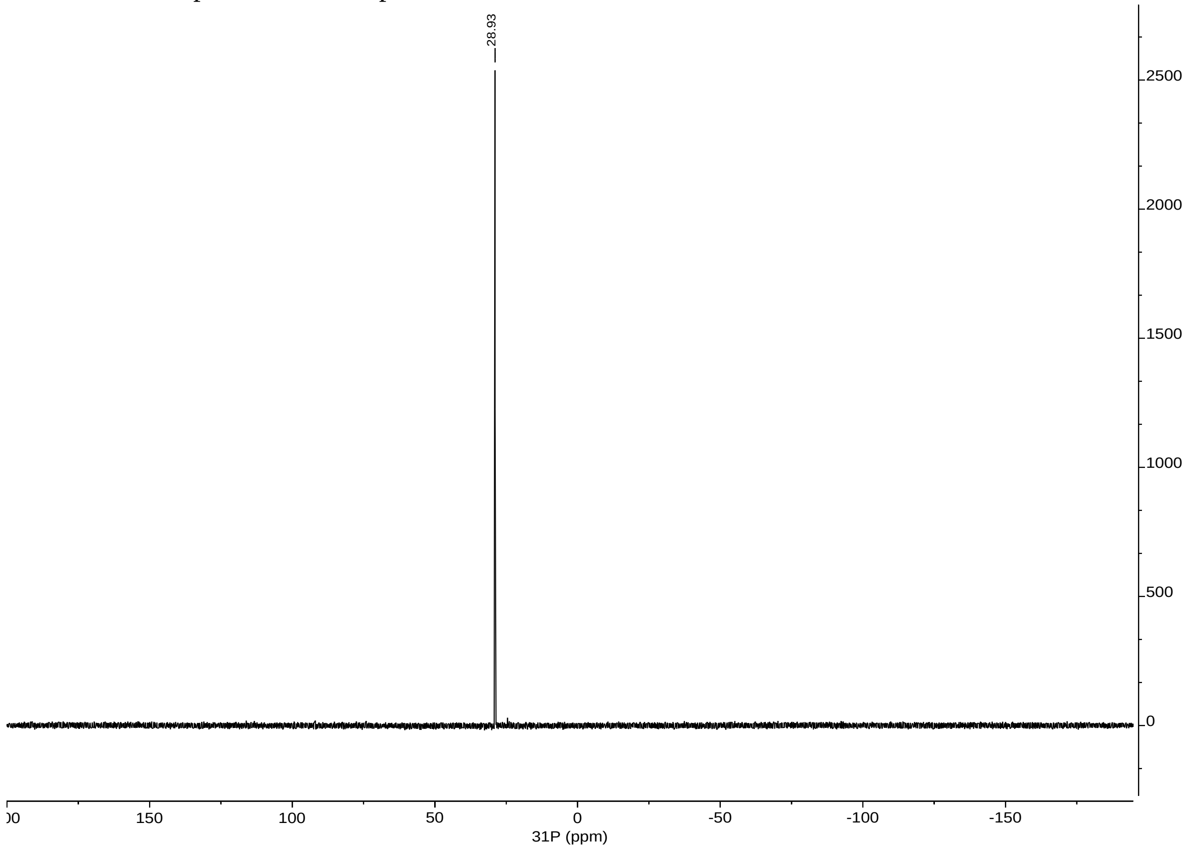

LC-MS chromatograms for compound **23**

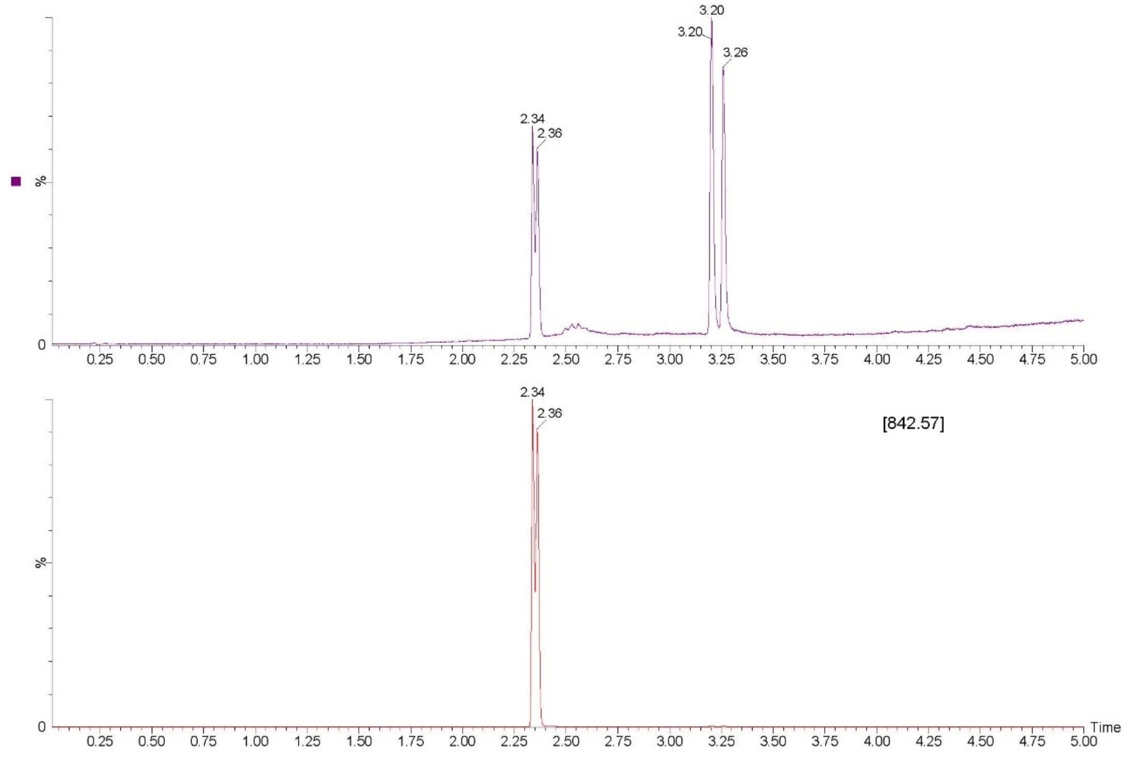

Butane-1,4-diyl diheptyl bis((2-(bis(3-aminopropyl)amino)ethyl)phosphonate) hexahydrochloride (24)

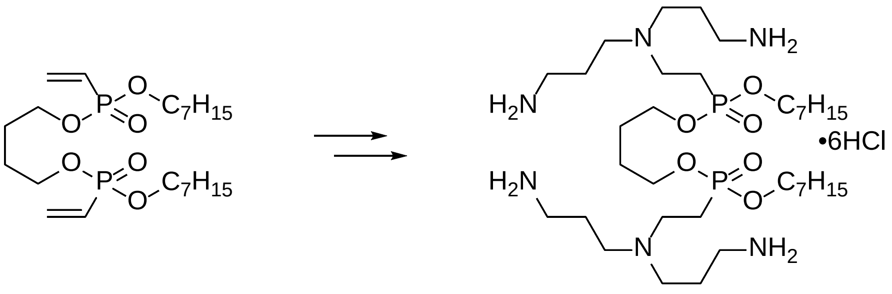

The title compound was prepared according to general methods **D** and **E** from vinylphosphonate dimer **S47** (0.32 g, 0.69 mmol) in 45% overall yield (0.29 g, 0.31 mmol) as a white solid.
Mixture of diastereoisomers.
^{1}H NMR (401 MHz, CD_3OD): 4.34–4.07 (m, 8H, OCH_2), 3.48 (dt, 4H, J = 10.4, 7.6 Hz, PCH_2CH_2), 3.44–3.39 (m, 8H, CH_2(CH_2)$_2$$NH_2$), 3.12 (t, J = 7.5 Hz, 8H, C$H_2$$NH_2$), 2.59 (ddt, 4H, J = 20.8, 7.4, 3.5 Hz, PCH_2), 2.30–2.14 (m, 8H, C$H_2$$CH_2NH_2$), 1.94–1.81 (m, 4H, OCH_2(CH_2)$_2$$CH_2O$), 1.72 (dt, 4H, J = 8.1, 6.4 Hz, CH_2(CH_2)$_4$$CH_3$), 1.48–1.24 (m, 16H, O($CH_2$)$_2$(C$H_2$)$_4$$CH_3$), 0.97–0.85 (m, 6H, C$H_3$).
^{13}C NMR (101 MHz, CD_3OD): 68.30 (d, J = 6.7 Hz), 68.29 (d, J = 6.7 Hz, O$C$$H_2$($CH_2$)$_5$$CH_3$), 67.65 (d, J = 6.6 Hz), 67.61 (d, J = 6.5 Hz, O$C$$H_2$($CH_2$)$_2$$C$$H_2$O), 51.04 ($C$$H_2$($CH_2$)$_2$$NH_2$), 48.87 ($PCH_2$$C$$H_2$), 37.90 ($C$$H_2$$NH_2$), 32.92 ($C$$H_2$($CH_2$)$_3$$CH_3$), 31.62 (d, J = 6.0 Hz, $C$$H_2$($CH_2$)$_4$$CH_3$), 30.00, 27.86 (d, J = 6.3 Hz), 27.82 (d, J = 6.2 Hz, OCH_2($C$$H_2$)$_2$$CH_2O$), 26.58, 23.68, 23.25 ($C$$H_2$$CH_2NH_2$), 21.42 (d, J = 140.3 Hz), 21.38 (d, J = 139.7 Hz, P$C$$H_2$), 14.43 ($C$$H_3$).
^{31}P{^{1}H} NMR (162 MHz, CD_3OD): 28.73.
IR ν_{max} (KBr) 2957 (vs), 2930 (vs), 2859 (s), 2740 (s, sh), 2627 (m), 2556 (m), 2002 (w, vbr), 1599 (m), 1469 (s), 1395 (m, br), 1254 (s, sh), 1229 (s), 1065 (s), 1007 (s), 984 (s, sh), 728 (w).
HR-MS(ESI$^+$): For $C_{34}H_{79}N_6O_6P_2$ $(M+H)^+$ m/z calculated 729.55308, found 729.55334.

^{1}H NMR spectrum of compound **24**

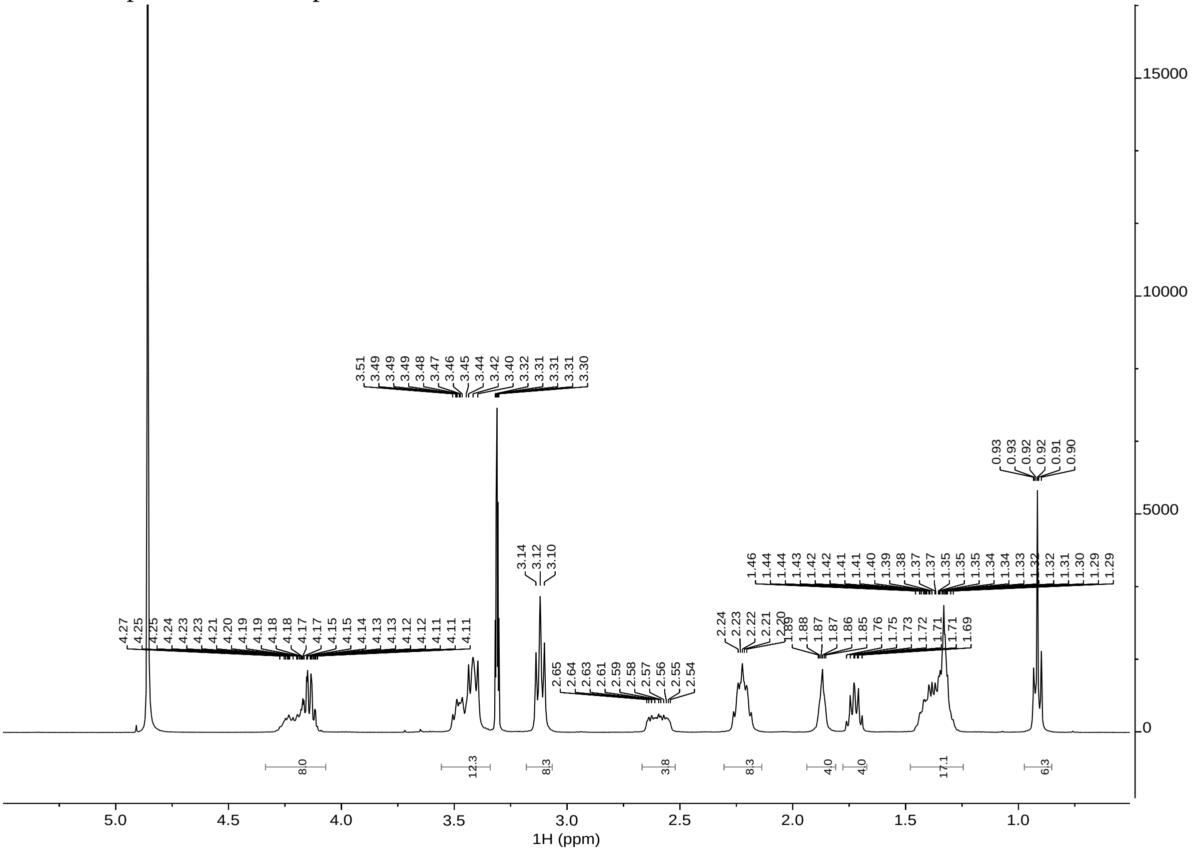

^{13}C NMR spectrum of compound **24**

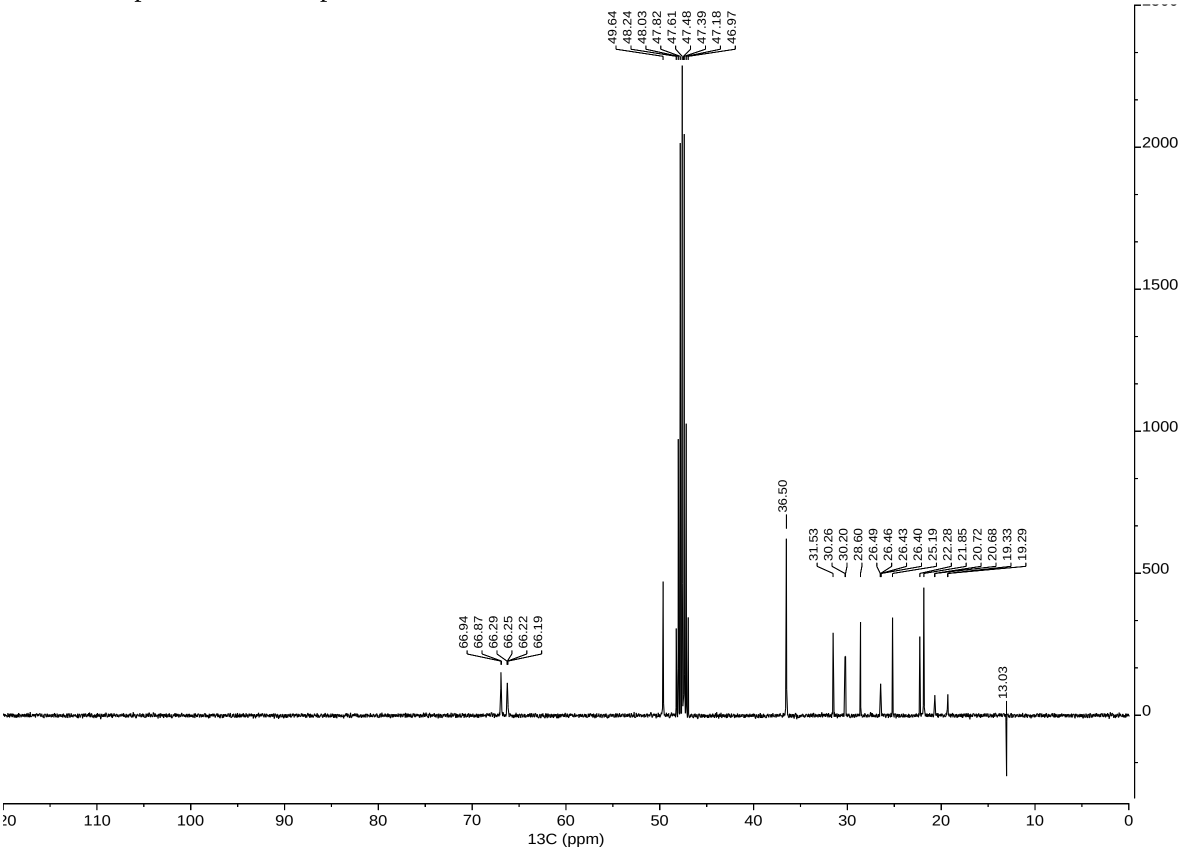

^{31}P{^{1}H} NMR spectrum of compound **24**

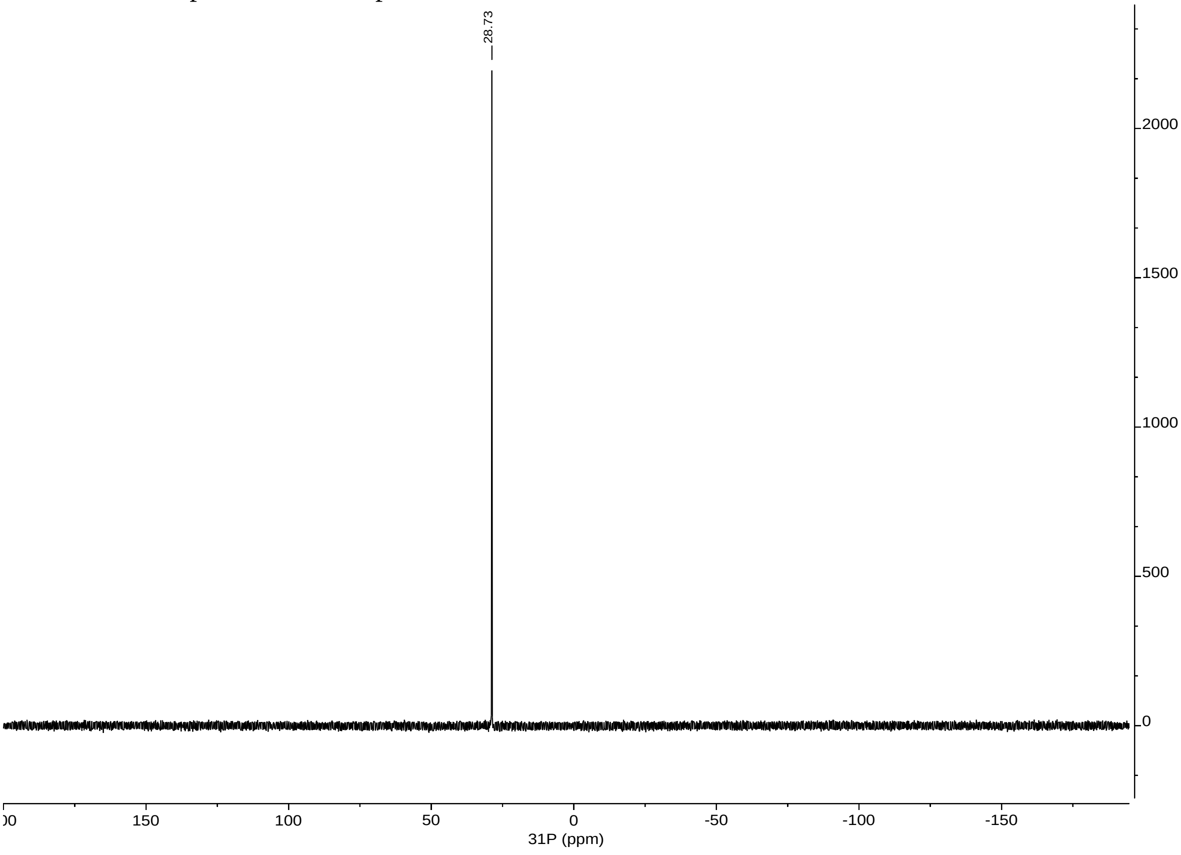

LC-MS chromatograms for compound **24**

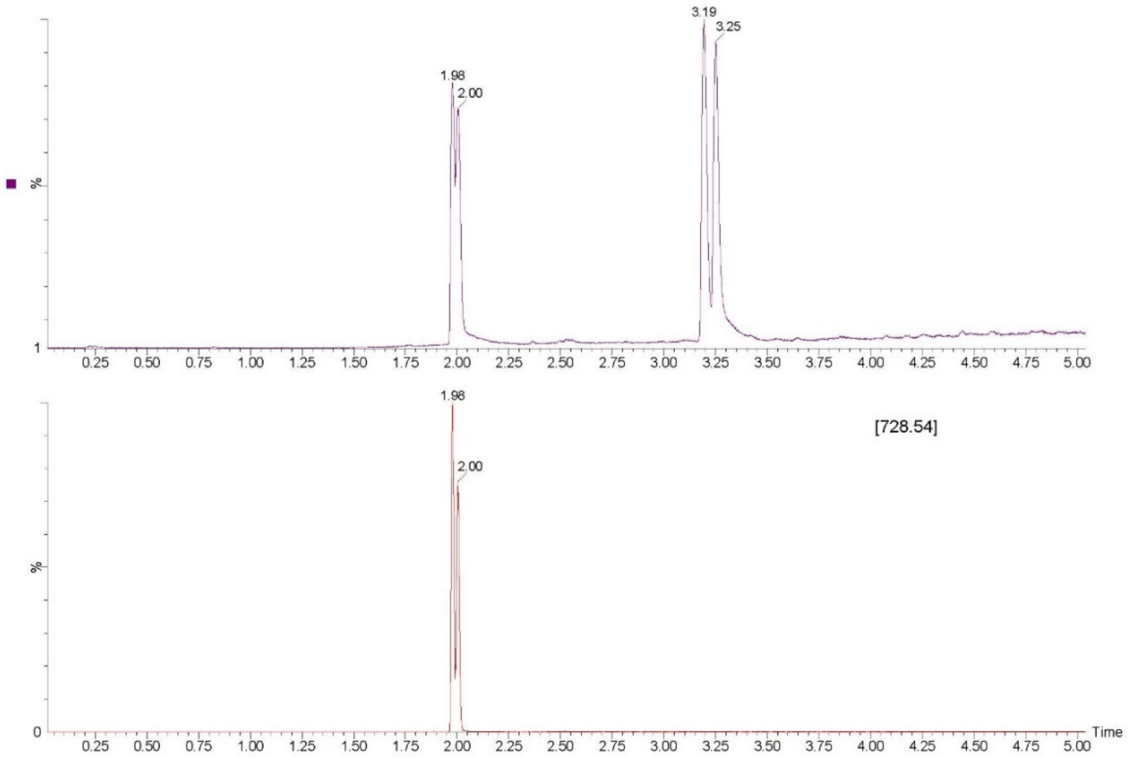

Butane-1,4-diyl dioctyl bis((2-(bis(3-aminopropyl)amino)ethyl)phosphonate) hexahydrochloride (25)

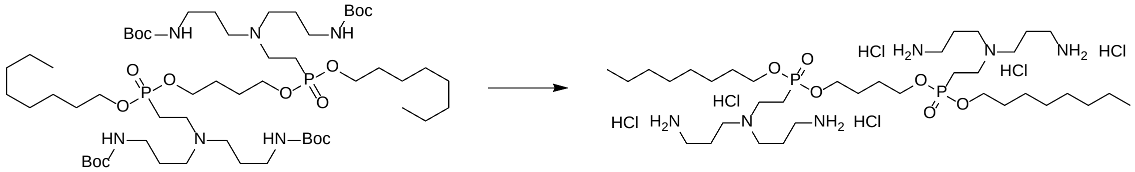

The title compound was prepared according to general method **E** from Boc derivative **S97** (0.85 g, 0.73 mmol) in 87% yield (0.62 g, 0.64 mmol) as a white solid.

Mixture of diastereoisomers.

^{1}H NMR (401 MHz, CD_3OD): 4.31–4.08 (m, 8H, C*H*$_2$O), 3.54–3.33 (m, 12H, C*H*$_2$N), 3.12 (t, 8H, *J* = 7.5 Hz C*H*$_2$NH$_2$), 2.68–2.51 (m, 4H, PC*H*$_2$), 2.29–2.13 (m, 8H, C*H*$_2$CH$_2$NH$_2$), 1.93–1.82 (m, 4H, OCH$_2$(C*H*$_2$)$_2$CH$_2$O), 1.77–1.68 (m, 4H, CH$_3$(CH$_2$)$_5$C*H*$_2$), 1.48–1.24 (m, 20H, CH$_3$(C*H*$_2$)$_5$), 0.96–0.84 (m, 6H, C*H*$_3$).

^{13}C NMR (101 MHz, CD_3OD): 68.29 (d, *J* = 6.6 Hz), 68.28 (d, *J* = 6.6 Hz, CH$_3$(CH$_2$)$_6$*C*H$_2$O), 67.64 (d, *J* = 6.6 Hz), 67.59 (d, *J* = 6.5 Hz, O*C*H$_2$(CH$_2$)$_2$*C*H$_2$O), 51.04 (*C*H$_2$(CH$_2$)$_2$NH$_2$), 48.86 (PCH$_2$*C*H$_2$), 37.92 (*C*H$_2$NH$_2$), 32.99 (CH$_3$CH$_2$*C*H$_2$), 31.62 (d, *J* = 5.9 Hz, CH$_3$(CH$_2$)$_5$*C*H$_2$CH$_2$O), 30.37, 30.30, 26.63, 27.86 (d, *J* = 6.2 Hz), 27.82 (d, *J* = 6.2 Hz, OCH$_2$(*C*H$_2$)$_2$CH$_2$O), 23.72 (CH$_3$*C*H$_2$), 23.26 (*C*H$_2$CH$_2$NH$_2$), 21.44 (d, *J* = 139.7 Hz), 21.40 (d, *J* = 139.8 Hz, P*C*H$_2$), 14.45 (*C*H$_3$).

^{31}P{^{1}H} NMR (162 MHz, CD_3OD): 28.76, 28.60.

IR ν_{max} (KBr) 3504 (w, br), 3000 (w, br), 2959 (s), 2933 (s), 2857 (s), 2745 (s, br), 2643 (s, br), 2561 (s, br), 2051 (w, br), 1609 (m), 1521 (w, sh), 1510 (m), 1229 (s, br), 1071 (s), 997 (s, br).

HR-MS(ESI$^+$): For $C_{36}H_{83}N_6O_6P_2$ $(M+H)^+$ *m/z* calculated 757.58438, found 757.58384.

^{1}H NMR spectrum of compound **25**

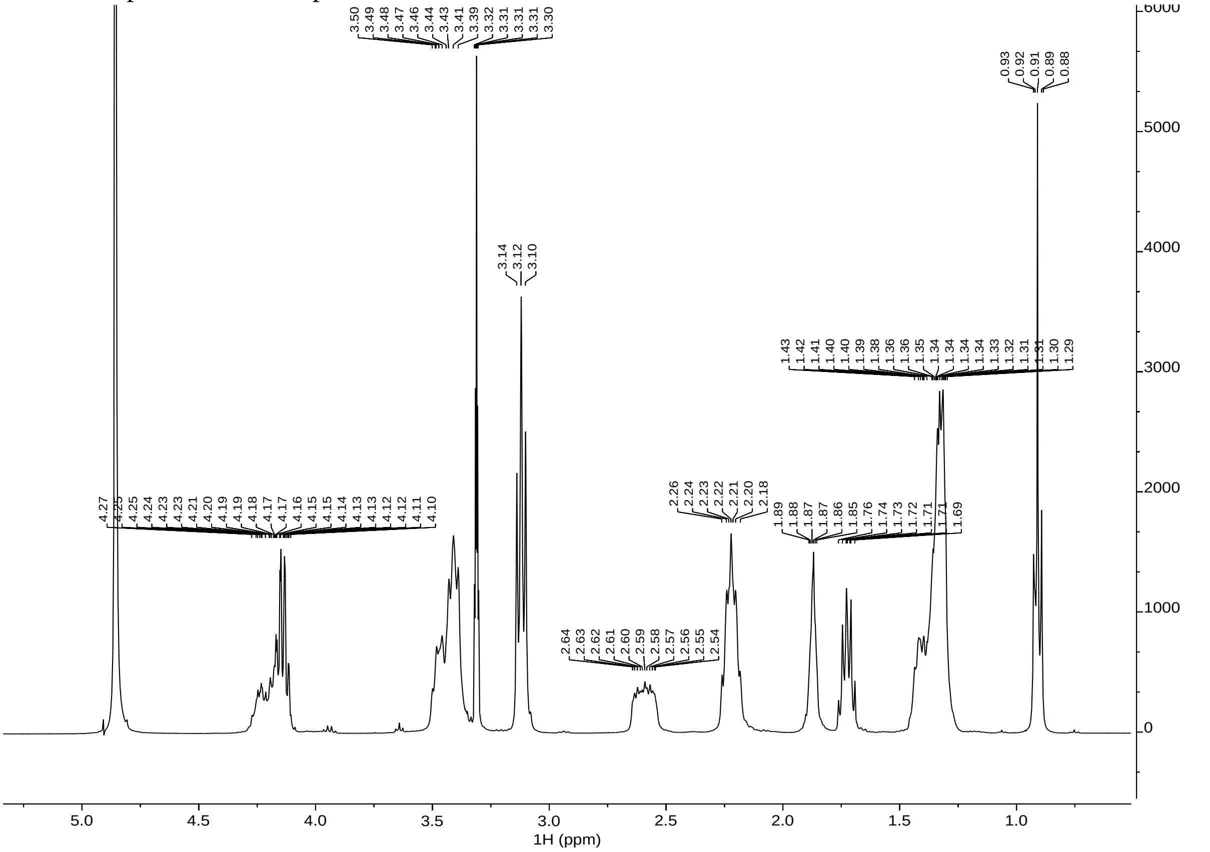

^{13}C NMR spectrum of compound **25**

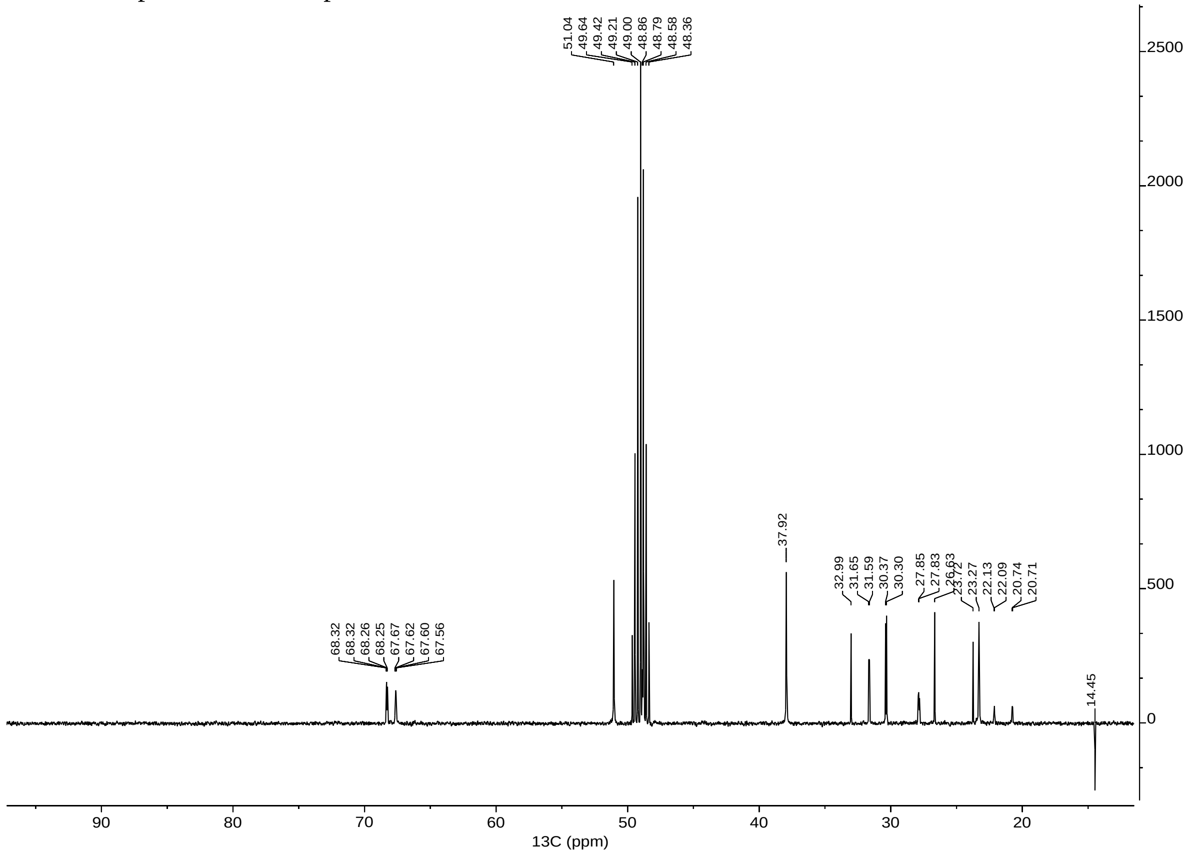

^{31}P{^{1}H} NMR spectrum of compound **25**

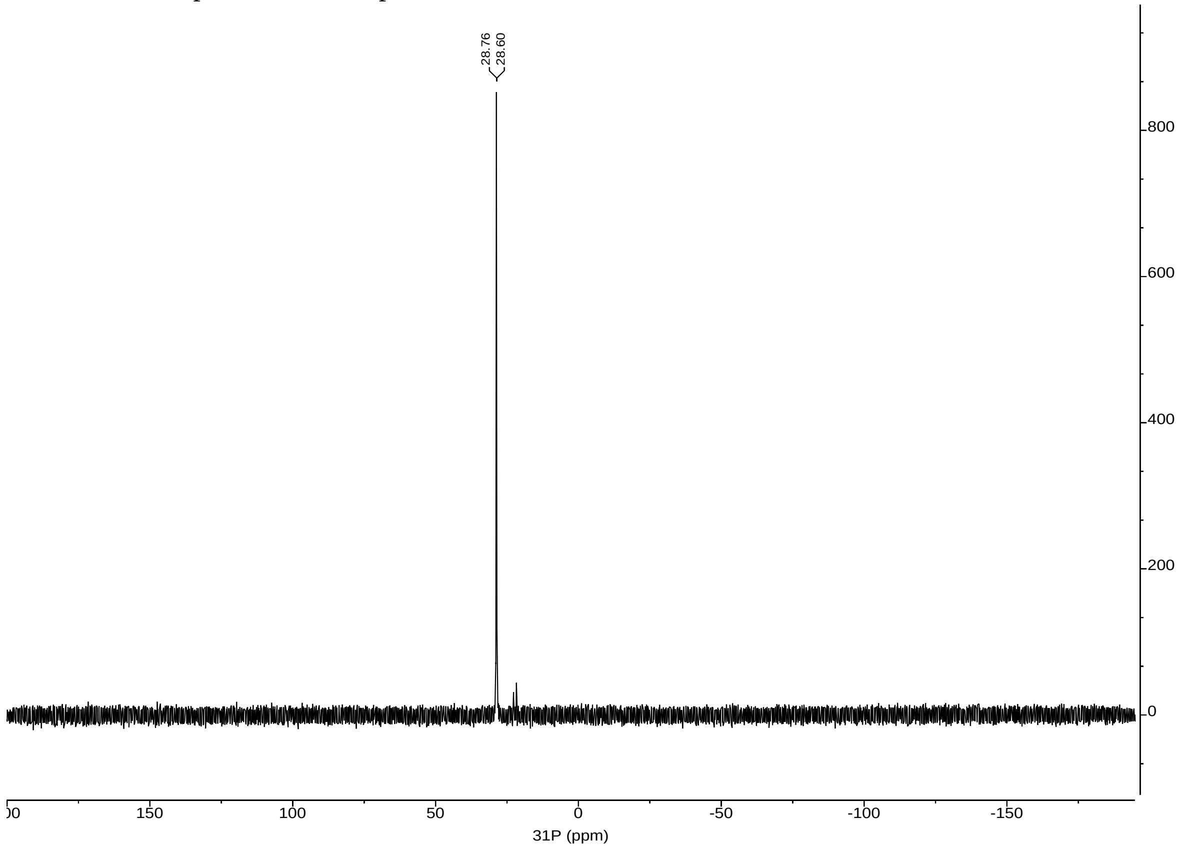

LC-MS chromatograms for compound **25**

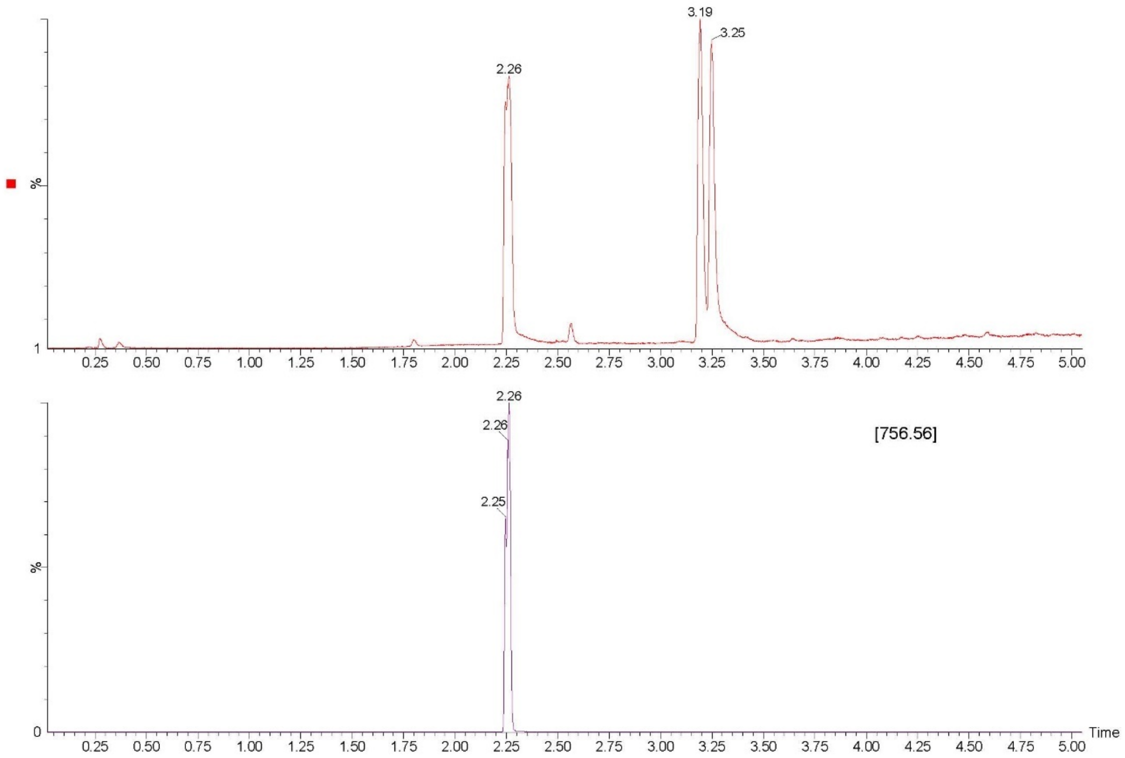

Bis((*Z*)-hept-3-en-1-yl) pentane-1,5-diyl bis((2-(bis(3-aminopropyl)amino)ethyl)phosphonate) hexahydrochloride (26)

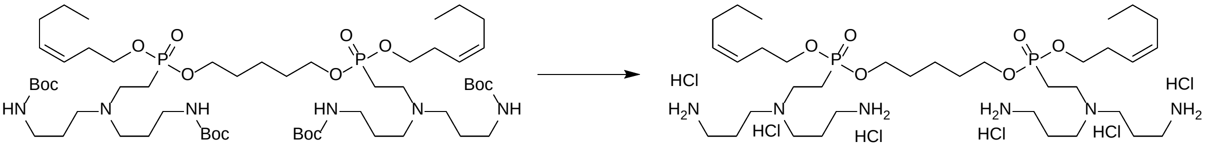

The title compound was prepared according to general method **E** from Boc derivative **S98** (0.83 g, 0.73 mmol) in 93% yield (0.65 g, 0.68 mmol) as a white solid.

^{1}H NMR (401 MHz, CD_3OD): 5.61–5.52 (m, 2H, $CH_3(CH_2)_2CH$), 5.48–5.39 (m, 2H, $CH(CH_2)_2O$), 4.26–4.08 (m, 8H, CH_2O), 3.53–3.36 (m, 12H, CH_2N), 3.12 (t, 8H, J = 7.5 Hz, CH_2NH_2), 2.66–2.45 (m, 8H, $CHCH_2CH_2O$, PCH_2), 2.28–2.17 (m, 8H, $CH_2CH_2NH_2$), 2.07 (qd, J = 7.4, 1.5 Hz, 4H, $CH_3CH_2CH_2$), 1.85–1.73 (m, 4H, $CH_2CH_2CH_2O$), 1.61–1.51 (m, 2H, $CH_2(CH_2)_2O$), 1.41 (h, 4H, J = 7.4 Hz, CH_3CH_2), 0.93 (t, 6H, J = 7.4 Hz, CH_3).

^{13}C NMR (101 MHz, CD_3OD): 134.14 ($CH_3(CH_2)_2CH$), 125.19 ($CH(CH_2)_2O$), 67.95 (d, J = 6.9 Hz), 67.61 (d, J = 6.6 Hz, CH_2O), 51.07 ($CH_2(CH_2)_2NH_2$), 37.95 (CH_2NH_2), 30.99 (d, J = 6.0 Hz), 30.96 (d, J = 6.4 Hz, $CH_2CH_2CH_2O$), 30.45 ($CH_3CH_2CH_2$), 30.97 (d, J = 6.2 Hz, $CHCH_2CH_2O$), 23.78 (CH_3CH_2), 23.35 ($CH_2CH_2NH_2$), 22.82 ($CH_2(CH_2)_2O$), 21.41 (d, J = 139.2 Hz (PCH_2), 14.15 (CH_3).

^{31}P{^{1}H} NMR (162 MHz, CD_3OD): 28.65.

IR ν_{max} 3013 (s), 3100–2500 (vs, br), 2958 (vs), 2933 (s), 2873 (s), 2017 (w), 1655 (w), 1601 (m), 1466 (m), 1379 (w), 1230 (s), 720 (w).

HR-MS(ESI^{+}): For $C_{35}H_{78}O_6N_6P_2$ $(M+2H)^{2+}$ $m/2z$ calculated 370.27236, found 370.27250.

^{1}H NMR spectrum of compound **26**

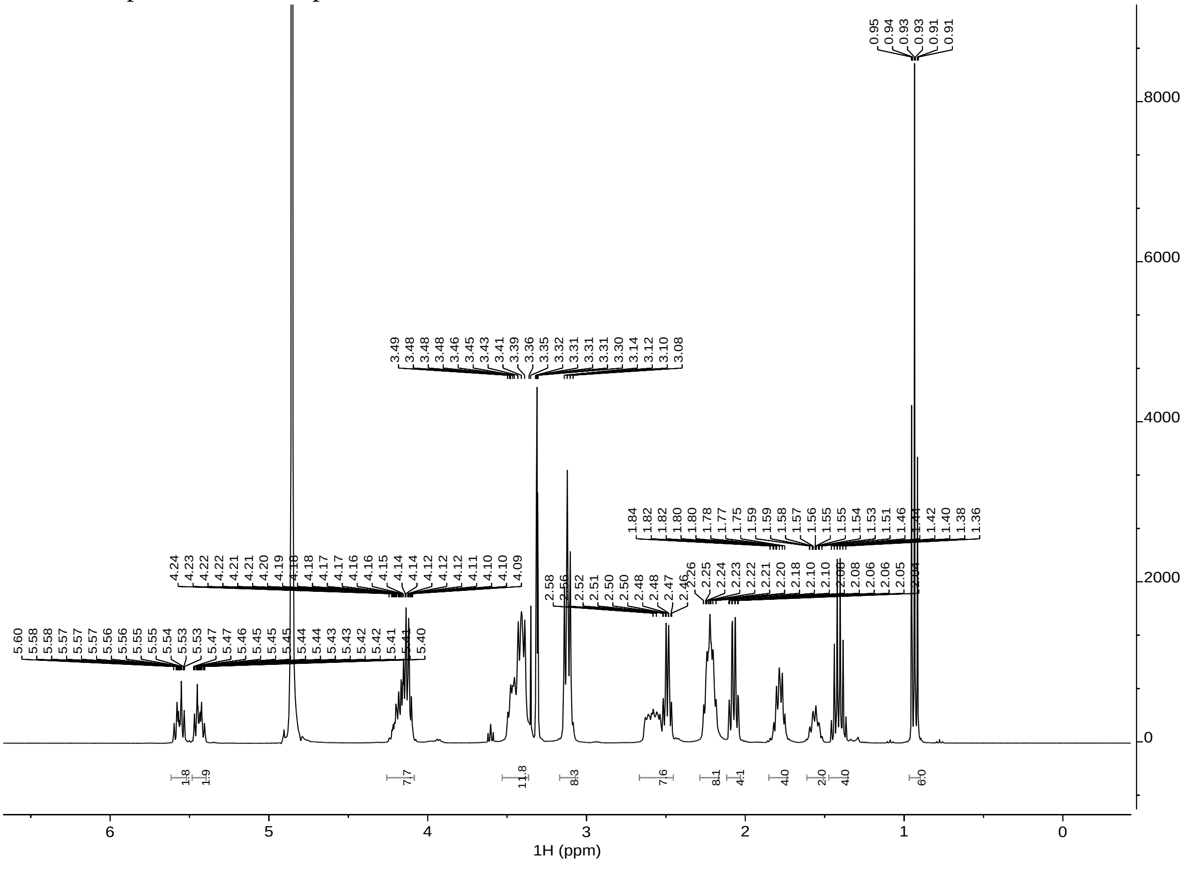

^{13}C NMR spectrum of compound **26**

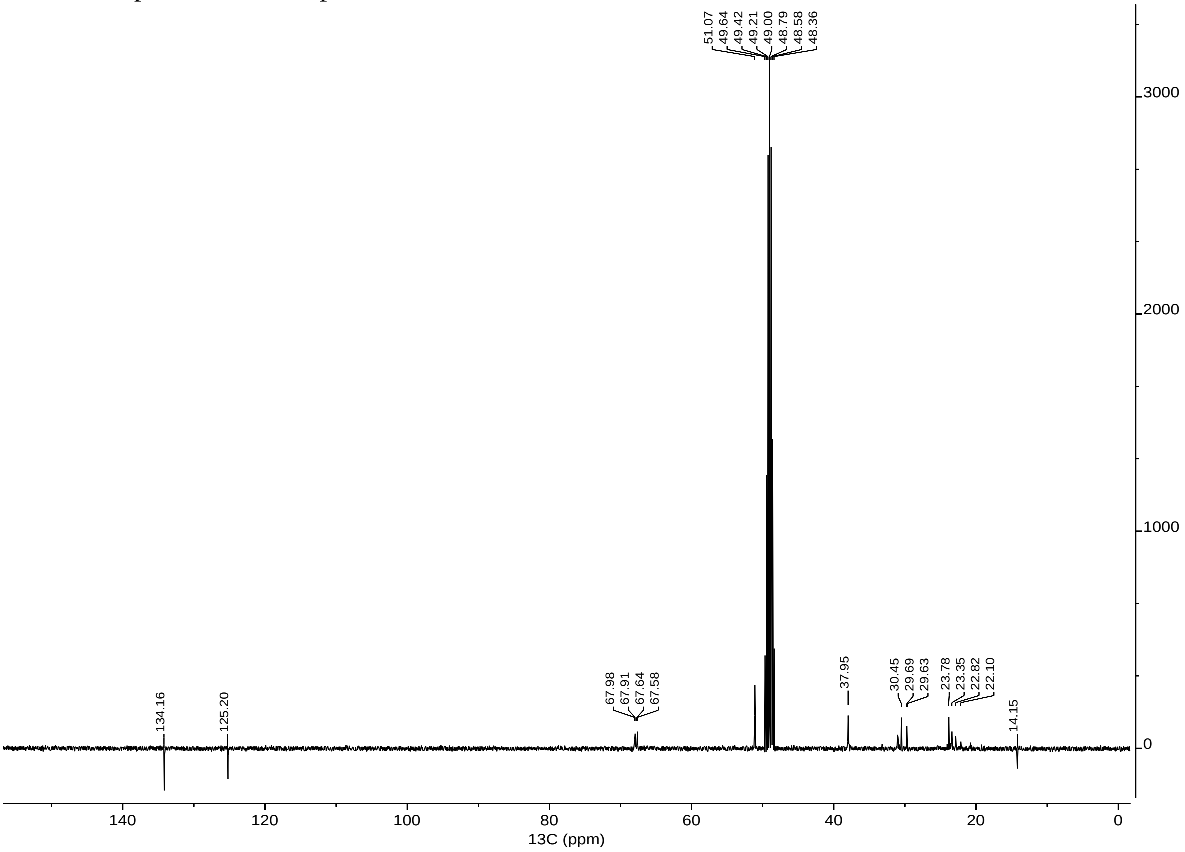

^{31}P{^{1}H} NMR spectrum of compound **26**

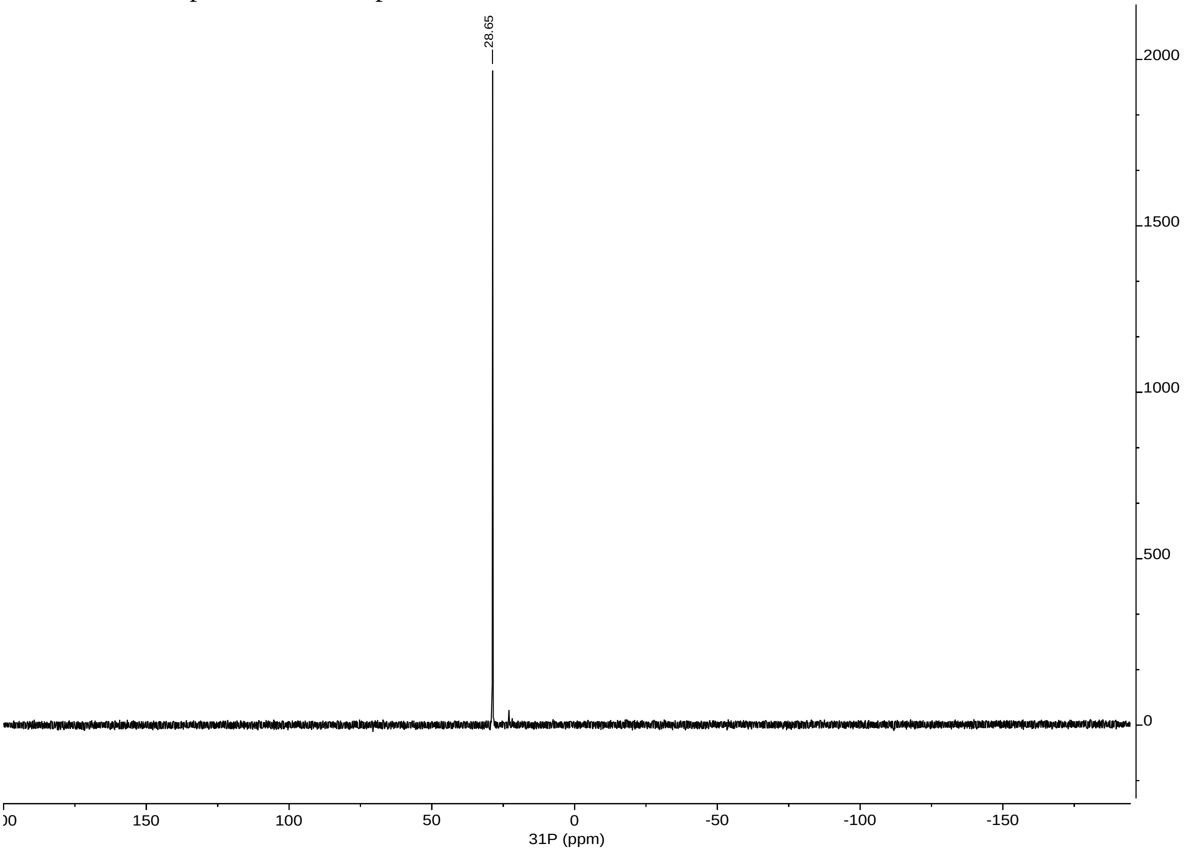

LC-MS chromatograms for compound **26**

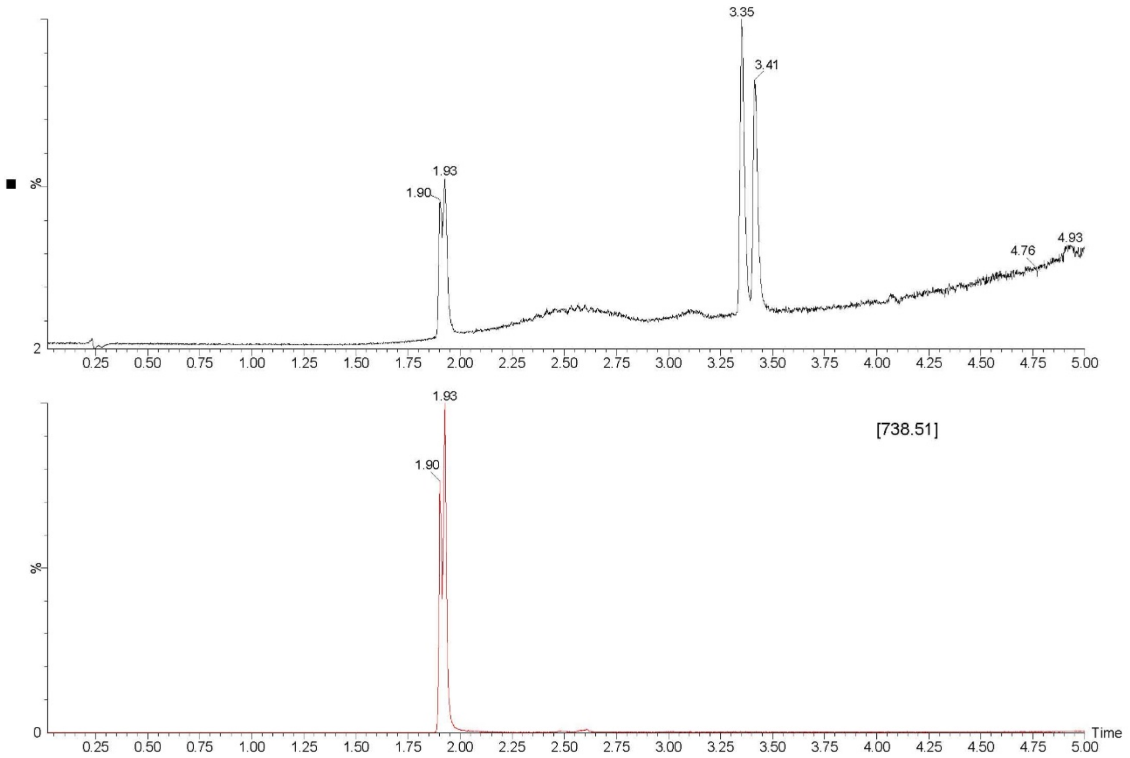

Bis((Z)-hept-4-en-1-yl) pentane-1,5-diyl bis((2-(bis(3-aminopropyl)amino)ethyl)phosphonate) hexahydrochloride (27)

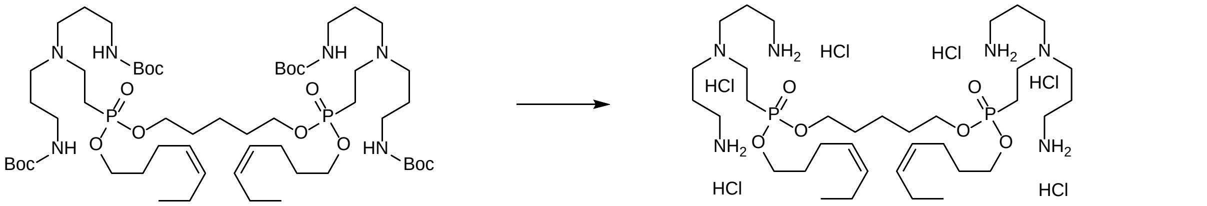

The title compound was prepared according to general method **E** from Boc derivative **S99** (0.48 g, 0.42 mmol) in 94% yield (0.38 g, 0.40 mmol) as a white solid.

Mixture of diastereoisomers.

^{1}H NMR (401 MHz, CD_3OD): 5.49–5.41 (m, 2H, CH_3CH_2CH), 5.39–5.31 (m, 2H, $CH(CH_2)_3O$), 4.25–4.09 (m, 8H, CH_2O), 3.54–3.37 (m, 12H, $CH_2(CH_2)_2NH_2$, PCH_2CH_2), 3.12 (t, 8H, J = 7.5 Hz, CH_2NH_2), 2.67–2.51 (m, 4H, PCH_2), 2.29–2.13 (m, 12H, $CH_2CH_2NH_2$, $CHCH_2(CH_2)_2O$), 2.13–2.03 (m, 4H, CH_3CH_2), 1.85–1.73 (m, 8H, CH_2CH_2O), 1.62–1.51 (m, 2H, $O(CH_2)_2CH_2(CH_2)_2O$), 0.98 (t, 6H, J = 7.5 Hz, CH_3).

^{13}C NMR (101 MHz, CD_3OD): 133.87 (CH_3CH_2CH), 128.47 ($CH(CH_2)_3O$), 68.00 (d, J = 6.7 Hz, $OCH_2(CH_2)_3CH_2O$), 67.71 (d, J = 6.7 Hz, $CH(CH_2)_2CH_2O$), 51.05 ($CH_2(CH_2)_2NH_2$), 48.87 (PCH_2CH_2), 37.89 (CH_2NH_2), 31.67 (d, J = 6.1 Hz, $CHCH_2CH_2CH_2O$), 31.00 (d, J = 6.2 Hz), 30.97 (d, J = 5.9 Hz, $OCH_2CH_2CH_2CH_2CH_2O$), 24.03 ($CHCH_2(CH_2)_2O$), 23.26 ($CH_2CH_2NH_2$), 22.81 (d, J = 2.9 Hz, $O(CH_2)_2CH_2(CH_2)_2O$), 21.50 (CH_3CH_2), 21.36 (d, J = 140.2 Hz), 21.35 (d, J = 140.0 Hz, PCH_2), 14.72 (CH_3).

^{31}P{^{1}H} NMR (162 MHz, CD_3OD): 28.72, 28.68.

IR ν_{max} 3010 (s, sh), 2961 (vs), 2931 (s), 2875 (s), 2040 (w), 1654 (w), 1404 (m), 1380 (w, sh), 1280 (w, sh), 1228 (m), 1067 (s), 997 (s), 840 (w), 756 (w).

HR-MS(ESI$^+$): For $C_{35}H_{78}O_6N_6P_2$ $(M+2H)^{2+}$ $m/2z$ calculated 370.27236, found 370.27254.

^{1}H NMR spectrum of compound **27**

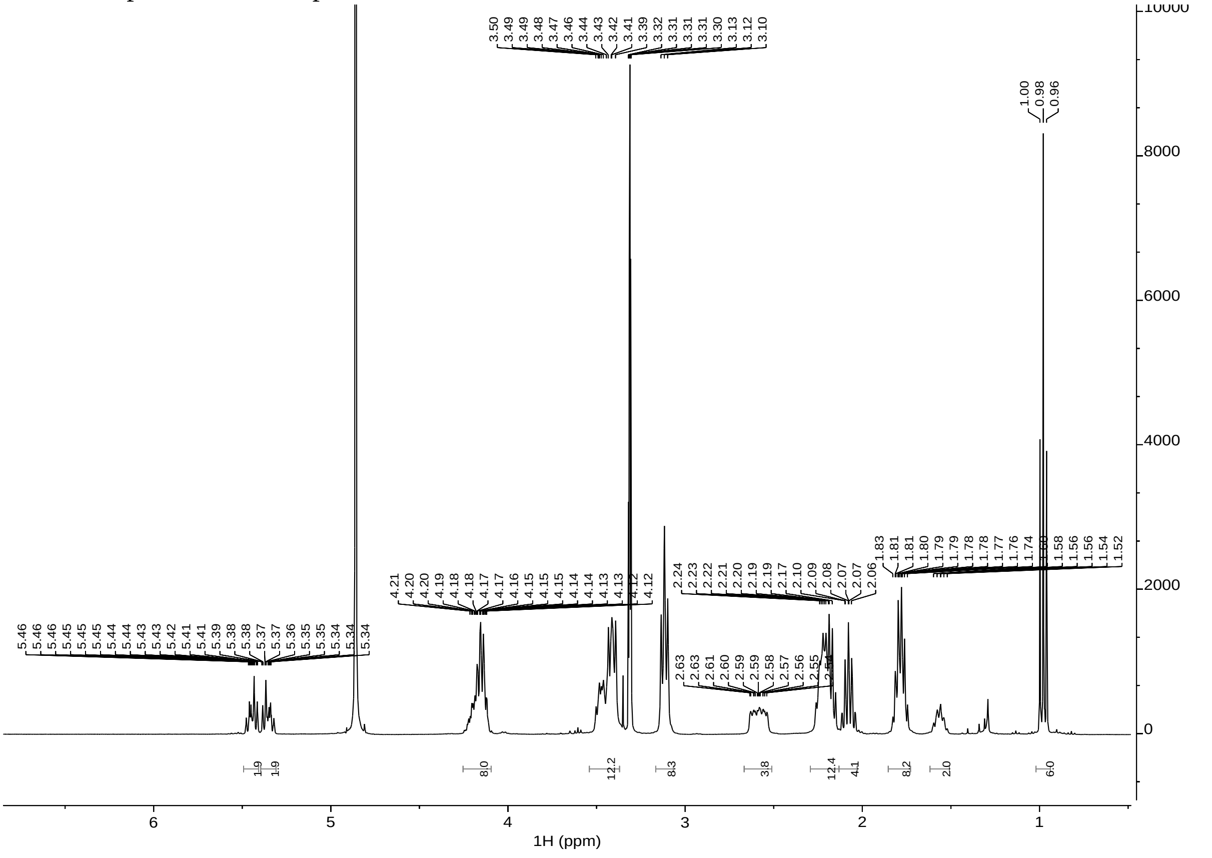

^{13}C NMR spectrum of compound **27**

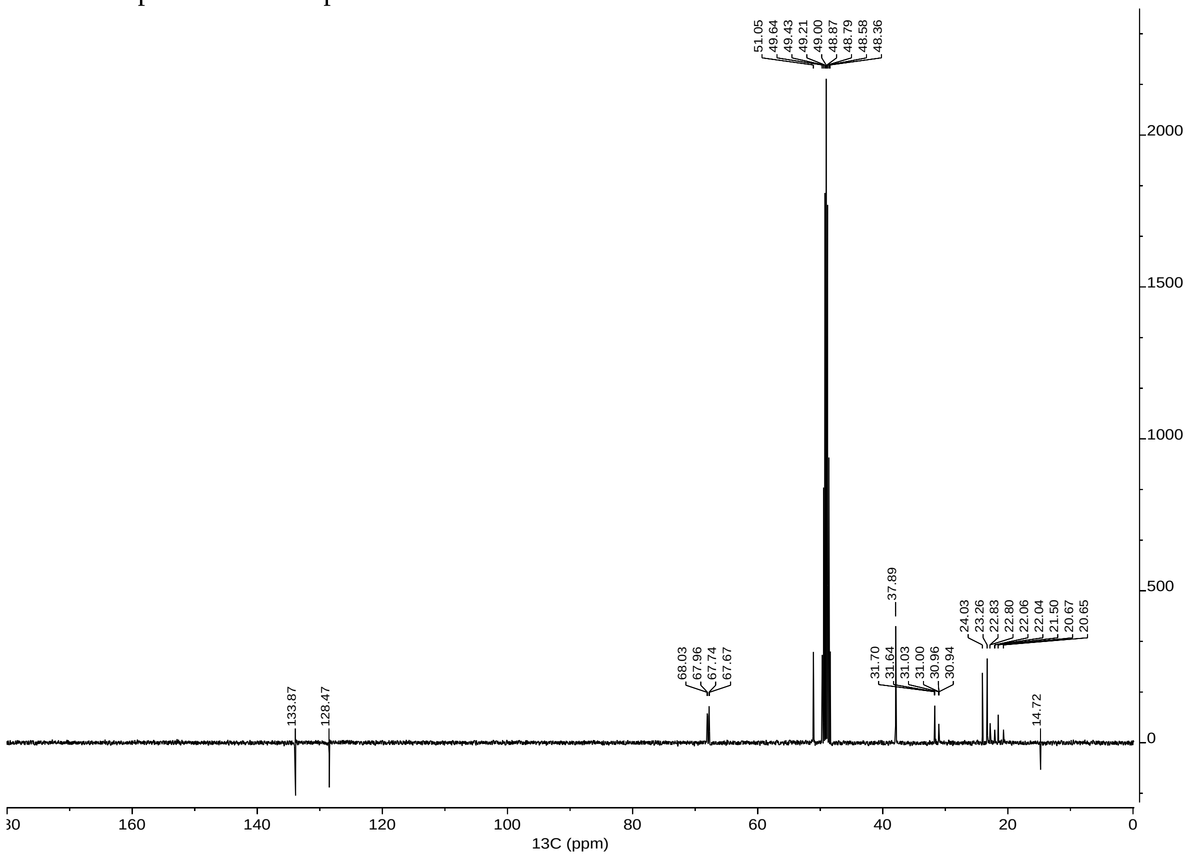

^{31}P{^{1}H} NMR spectrum of compound **27**

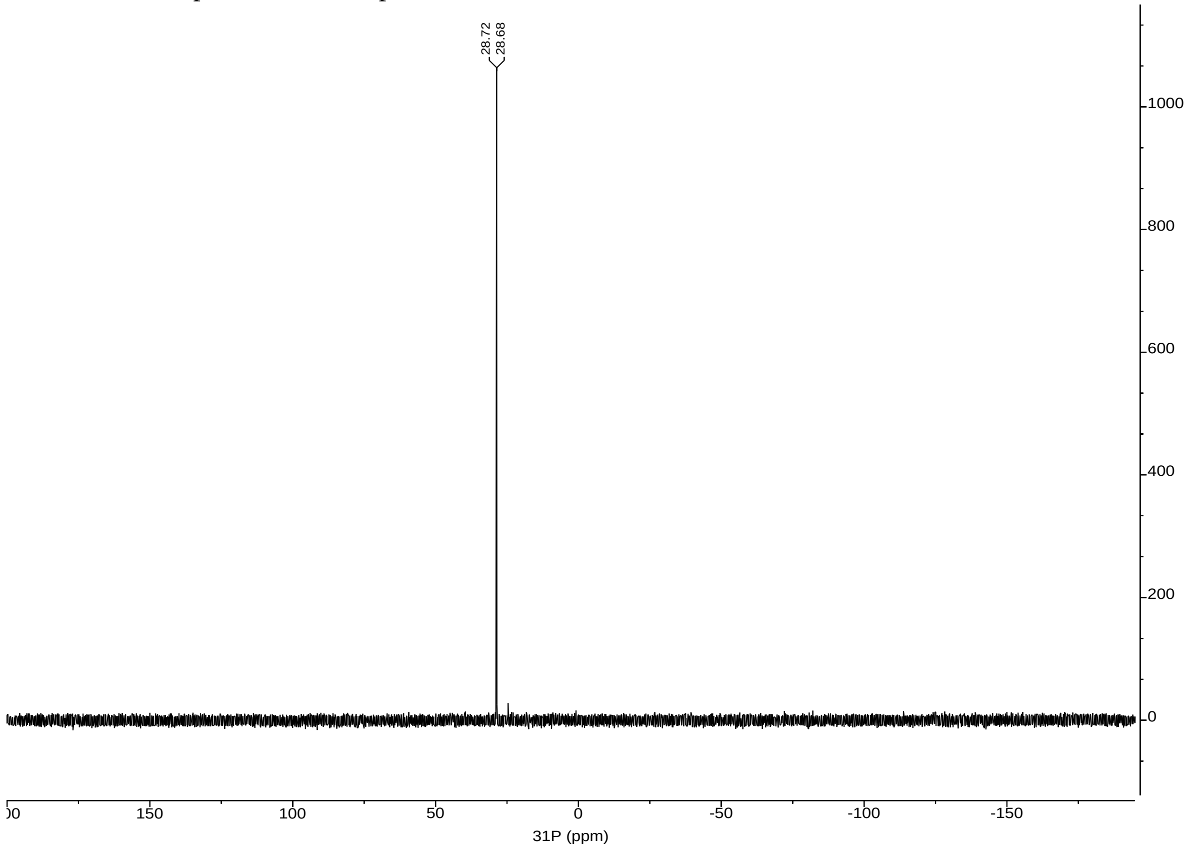

LC-MS chromatograms for compound **27**

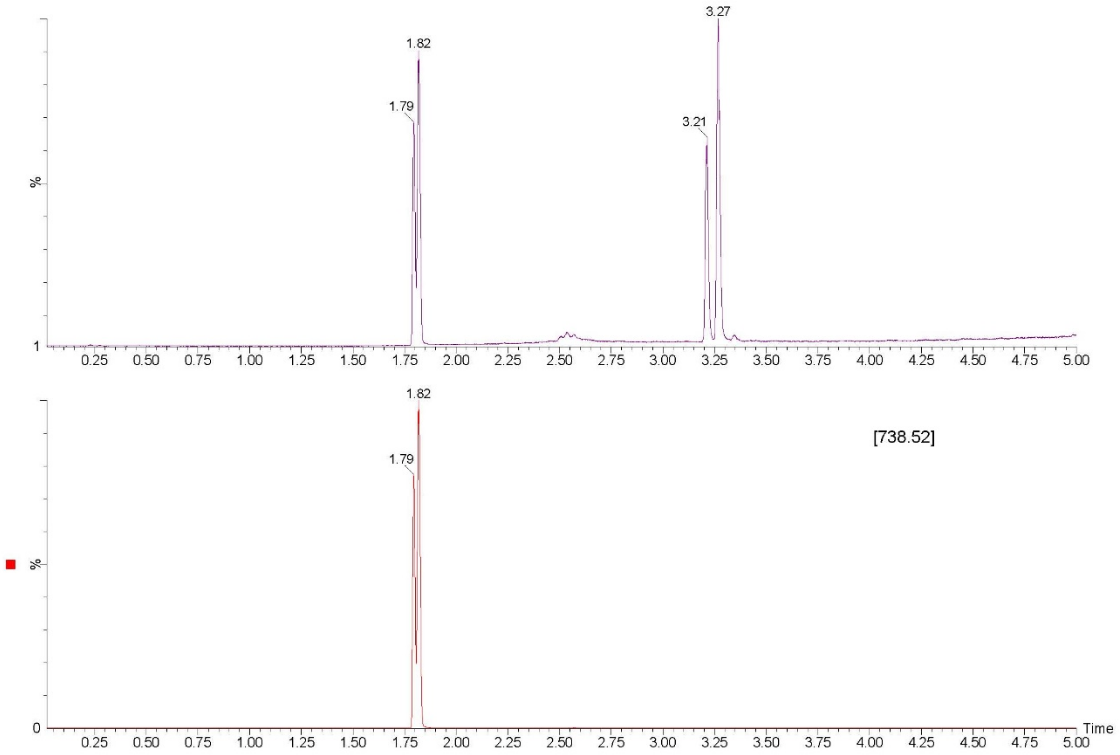

Bis((*Z*)-oct-3-en-1-yl) pentane-1,5-diyl bis((2-(bis(3-aminopropyl)amino)ethyl)phosphonate) hexahydrochloride (28)

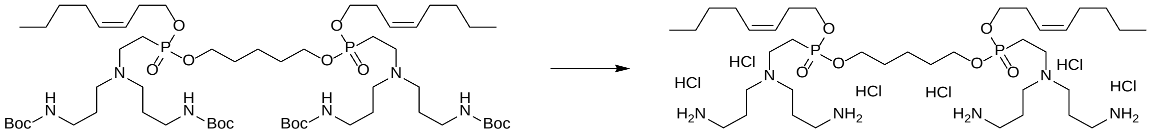

The title compound was prepared according to general method **E** from Boc derivative **S100** (0.67 g, 0.57 mmol) in 95% yield (0.54 g, 0.54 mmol) as a white solid.

Mixture of diastereoisomers.

^{1}H NMR (400 MHz, CD_3OD) δ 5.60–5.50 (m, 2H, $CH_3(CH_2)_3CH$), 5.46–5.37 (m, 2H, $CH(CH_2)_2O$), 4.25–4.06 (m, 8H, CH_2O), 3.52–3.37 (m, 12H, CH_2N), 3.14 (t, *J* = 7.3 Hz, 8H, CH_2NH_2), 2.68–2.54 (m, 4H, PCH_2), 2.48 (q, *J* = 6.9 Hz, 4H, $CHCH_2CH_2O$), 2.30–2.18 (m, 8H, $CH_2CH_2NH_2$), 2.12–2.04 (m, 4H, $CH_3(CH_2)_2CH_2$), 1.83–1.72 (m, 4H, $CH_2CH_2CH_2O$), 1.63–1.47 (m, 2H, $CH_2(CH_2)_2O$), 1.41–1.26 (m, 8H, $CH_3(CH_2)_2$), 0.97–0.85 (m, 6H, CH_3).

^{13}C NMR (101 MHz, CD_3OD) δ 134.20 ($CH_3(CH_2)_3CH$), 125.03 ($CH(CH_2)_2O$), 67.91 (d, *J* = 6.7 Hz, $(CH_2)_2CH_2O$), 67.57 (d, *J* = 6.7 Hz, $CHCH_2CH_2O$), 50.97 ($CH_2(CH_2)_2NH_2$), 48.88 (PCH_2CH_2), 37.93 (CH_2NH_2), 32.85 ($CH_3CH_2CH_2$), 30.90 (d, *J* = 6.0 Hz), 30.88 (d, *J* = 6.1 Hz, $CH_2CH_2CH_2O$), 29.58 (d, *J* = 6.0 Hz, $CHCH_2CH_2O$), 28.03 ($CH_3(CH_2)_2CH_2$), 23.31 (CH_3CH_2), 23.13 ($CH_2CH_2NH_2$), 22.77 ($CH_2(CH_2)_2O$), 22.75, 21.44 (d, *J* = 139.8 Hz), 21.43 (d, *J* = 139.7 Hz, PCH_2), 14.35 (CH_3).

^{31}P{^{1}H} NMR (162 MHz, CD_3OD): 28.45.

IR ν_{max} (KBr) 3435 (s, br), 3015 (m, sh), 2957 (m), 2927 (m), 2872 (m), 2857 (m), 2500–2300 (m, br), 1626 (w), 1467 (w), 1379 (w), 1275 (w, sh), 1226 (w), 1004 (m).

HR-MS(ESI$^+$): For $C_{37}H_{82}O_6N_6P_2$ $(M+2H)^{2+}$ *m*/2*z* calculated 384.28801, found 384.28824.

^{1}H NMR spectrum of compound **28**

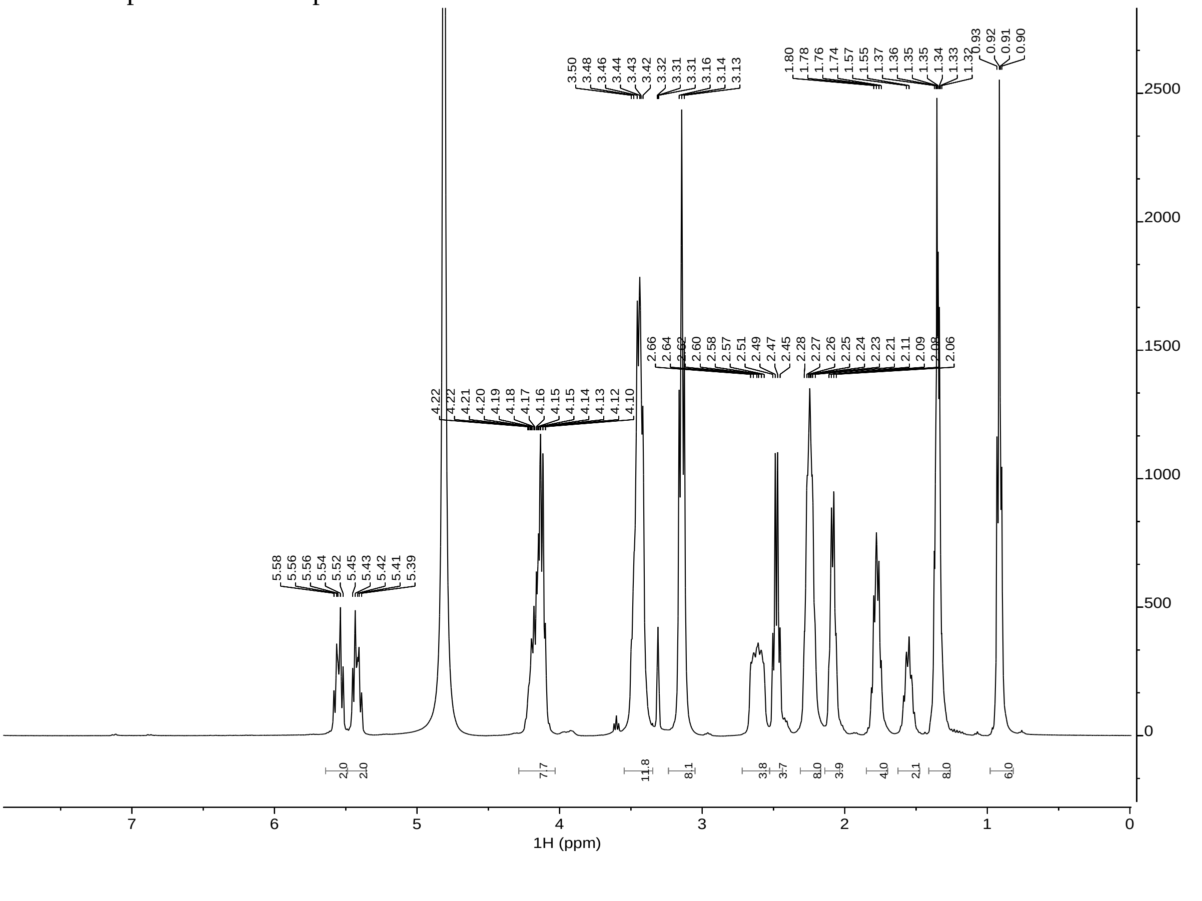

^{13}C NMR spectrum of compound **28**

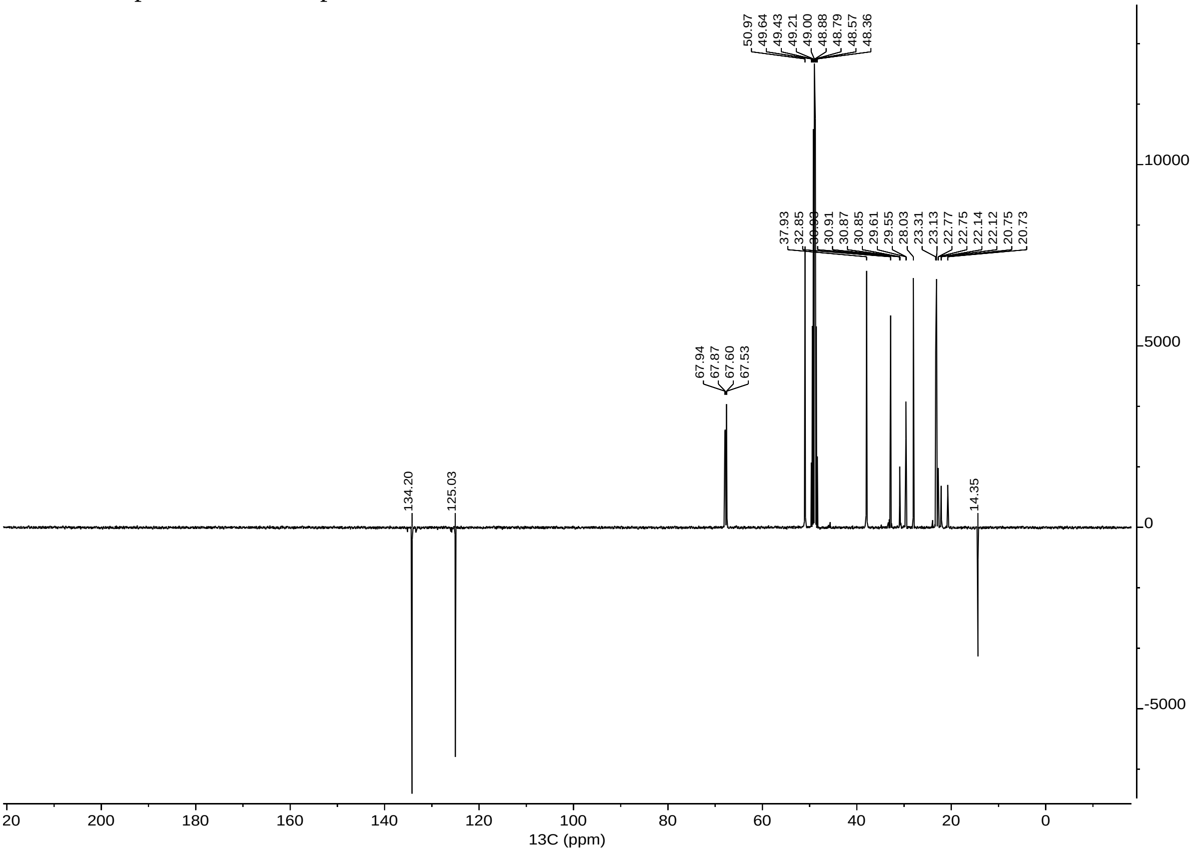

$^{31}P\{^{1}H\}$ NMR spectrum of compound **28**

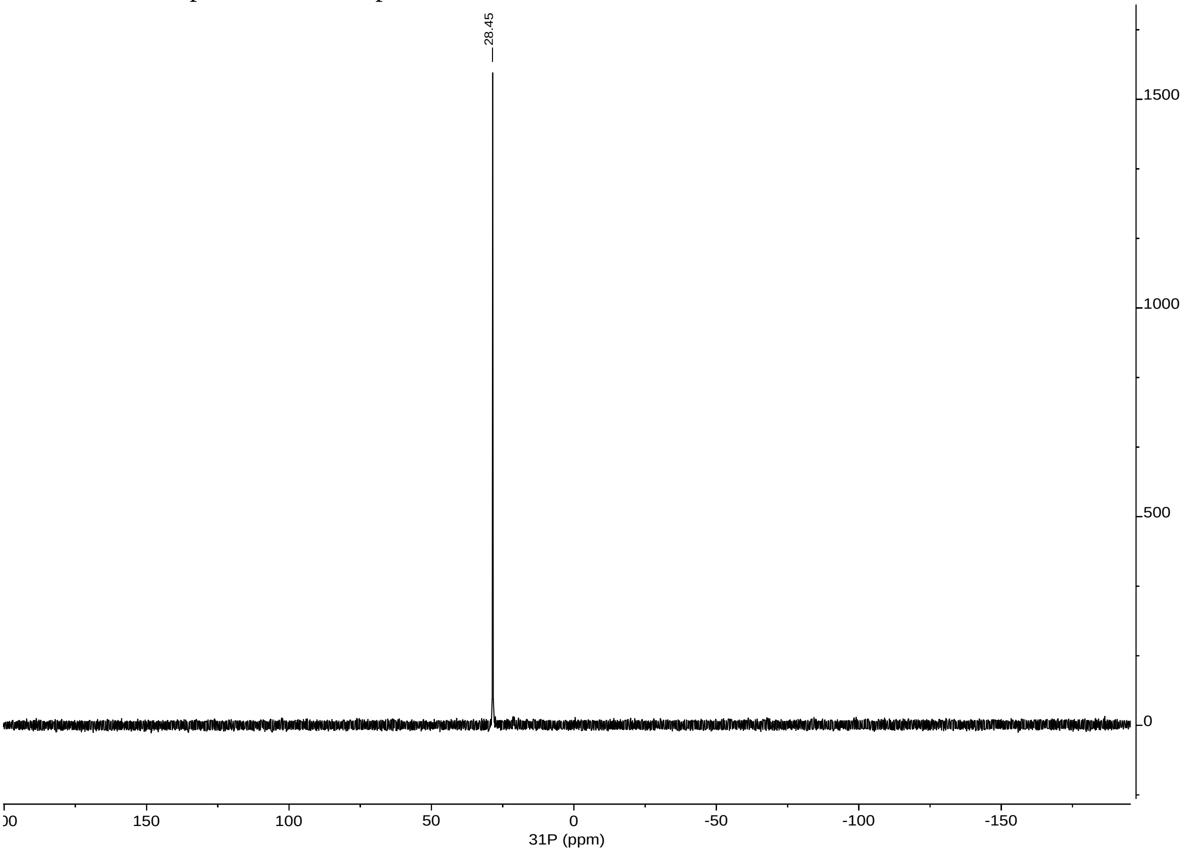

LC-MS chromatograms for compound **28**

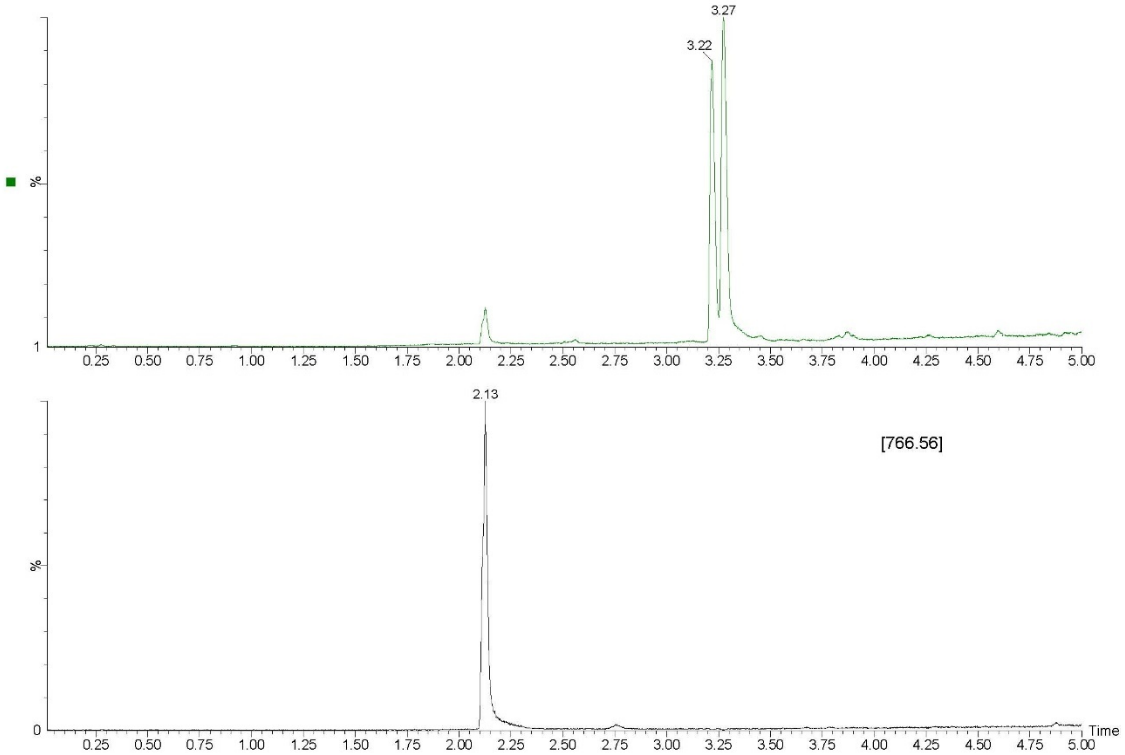

Bis((*Z*)-non-3-en-1-yl) pentane-1,5-diyl bis((2-(bis(3-aminopropyl)amino)ethyl)phosphonate) hexahydrochloride (29)

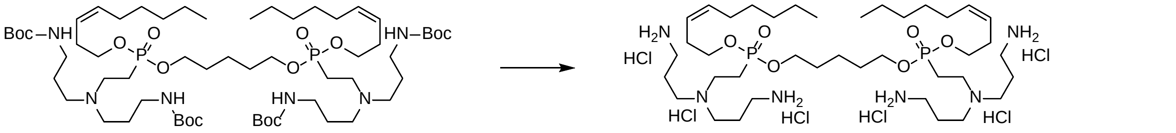

The title compound was prepared according to general method **E** from Boc derivative **S101** (0.99 g, 0.83mmol) in 97% yield (0.81 g, 0.80 mmol) as a white solid.

Mixture of diastereoisomers.

^{1}H NMR (401 MHz, CD_3OD): 5.63–5.51 (m, 2H, $CH_3(CH_2)_4CH$), 5.47–5.37 (m, 2H, $CH(CH_2)_2O$), 4.27–4.07 (m, 8H, CH_2O), 3.53–3.34 (m, 12H, CH_2N), 3.11 (t, 8H, J = 7.5 Hz, CH_2NH_2), 2.67–2.43 (m, 4H, $CHCH_2CH_2O$), 2.30–2.15 (m, 8H, $CH_2CH_2NH_2$), 2.15–2.04 (m, 4H, $CH_3(CH_2)_3CH_2$), 1.85–1.71 (m, 4H, $CH_2CH_2CH_2O$), 1.62–1.49 (m, 2H, $CH_2(CH_2)_2O$), 1.45–1.25 (m, 12H, $CH_3(CH_2)_3$), 0.97–0.86 (m, 6H, CH_3).

^{13}C NMR (101 MHz, CD_3OD): 134.41 ($CH_3(CH_2)_4CH$), 124.95 ($CH(CH_2)_2O$), 67.97 (d, J = 6.8 Hz, $OCH_2(CH_2)_3CH_2O$), 67.65 (d, J = 6.9 Hz, $CHCH_2CH_2O$), 49.65 ($CH_2(CH_2)_2NH_2$), 47.44 (PCH_2CH_2), 37.88 (CH_2NH_2), 32.66 ($CH_3CH_2CH_2$), 30.98 (d, J = 6.1 Hz), 30.95 (d, J = 6.3 Hz, $CH_2CH_2CH_2O$**)**, 30.40 ($CH_3(CH_2)_2CH_2$), 29.65 (d, J = 6.0 Hz, $CHCH_2CH_2O$), 28.37 ($CH_3(CH_2)_3CH_2$), 23.64 (CH_3CH_2), 23.25 ($CH_2CH_2NH_2$), 22.82, 22.79 ($CH_2(CH_2)_2O$), 21.37 (d, J = 140.1 Hz), 21.35 (d, J = 140.0 Hz, PCH_2), 14.44 (CH_3).

^{31}P{^{1}H} NMR (162 MHz, CD_3OD): 28.67, 28.63.

IR ν_{max} 3200–2800 (s), 2959 (s), 2929 (s), 2873 (s), 2859 (s), 2048 (m), 2800–2400 (s), 1650 (m, sh), 1617 (m), 1468 (m), 1380 (m), 1229 (m), 725 (m).

HR-MS(ESI$^+$): For $C_{39}H_{85}O_6N_6P_2$ $(M+H)^+$ m/z calculated 795.60003, found 795.59979.

^{1}H NMR spectrum of compound **29**

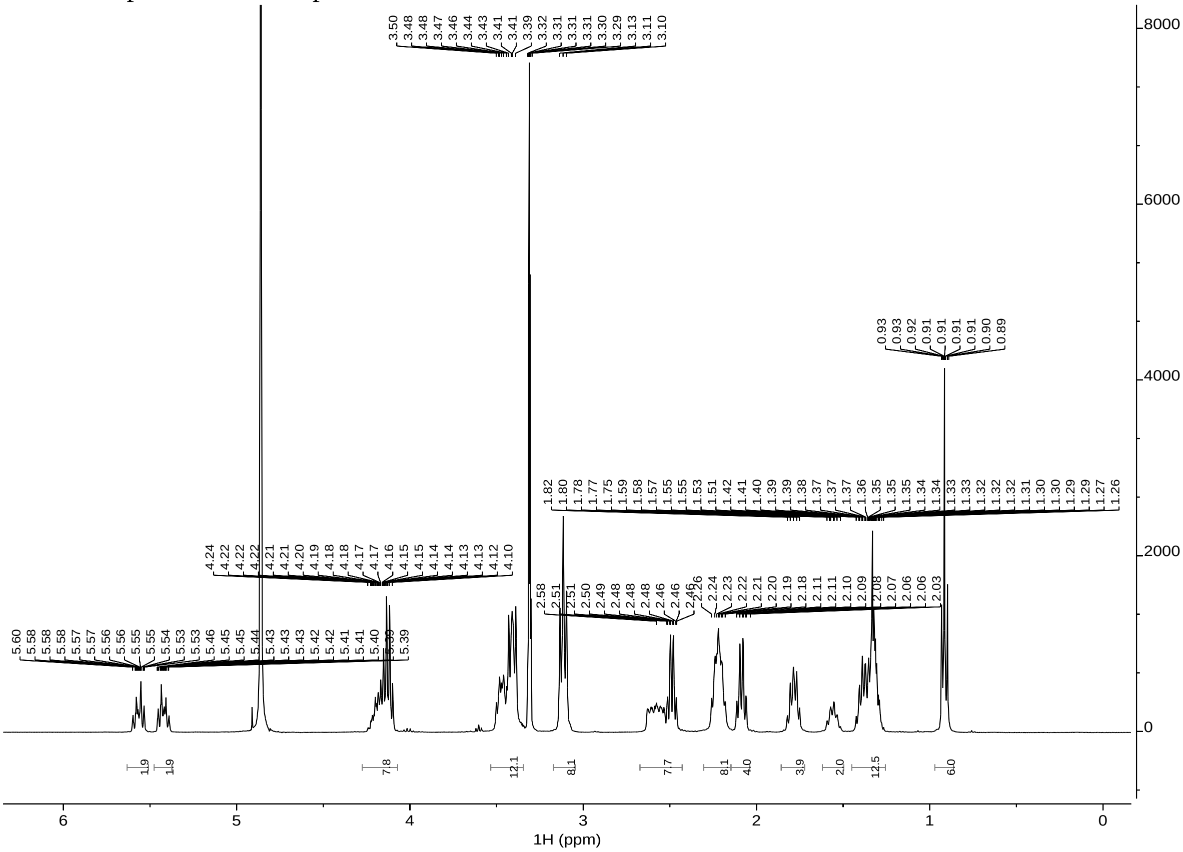

^{13}C NMR spectrum of compound **29**

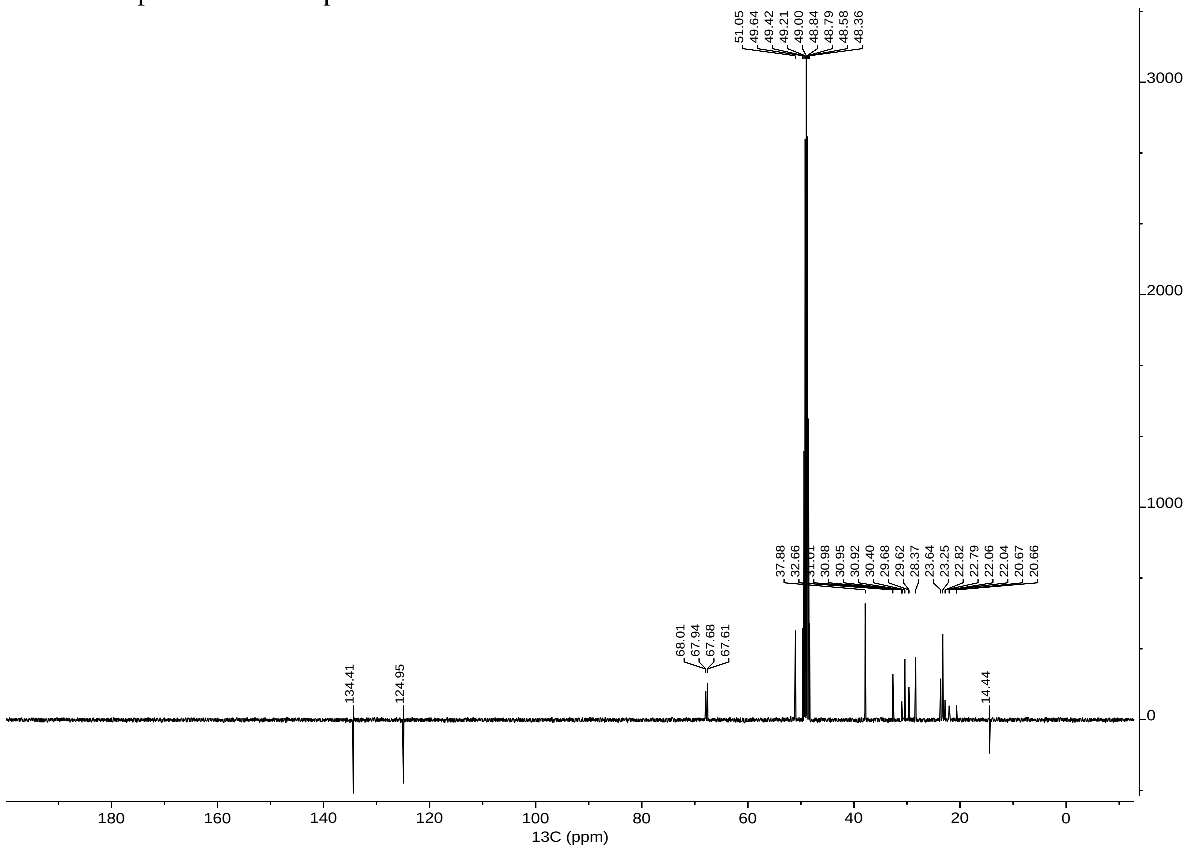

^{31}P{^{1}H} NMR spectrum of compound **29**

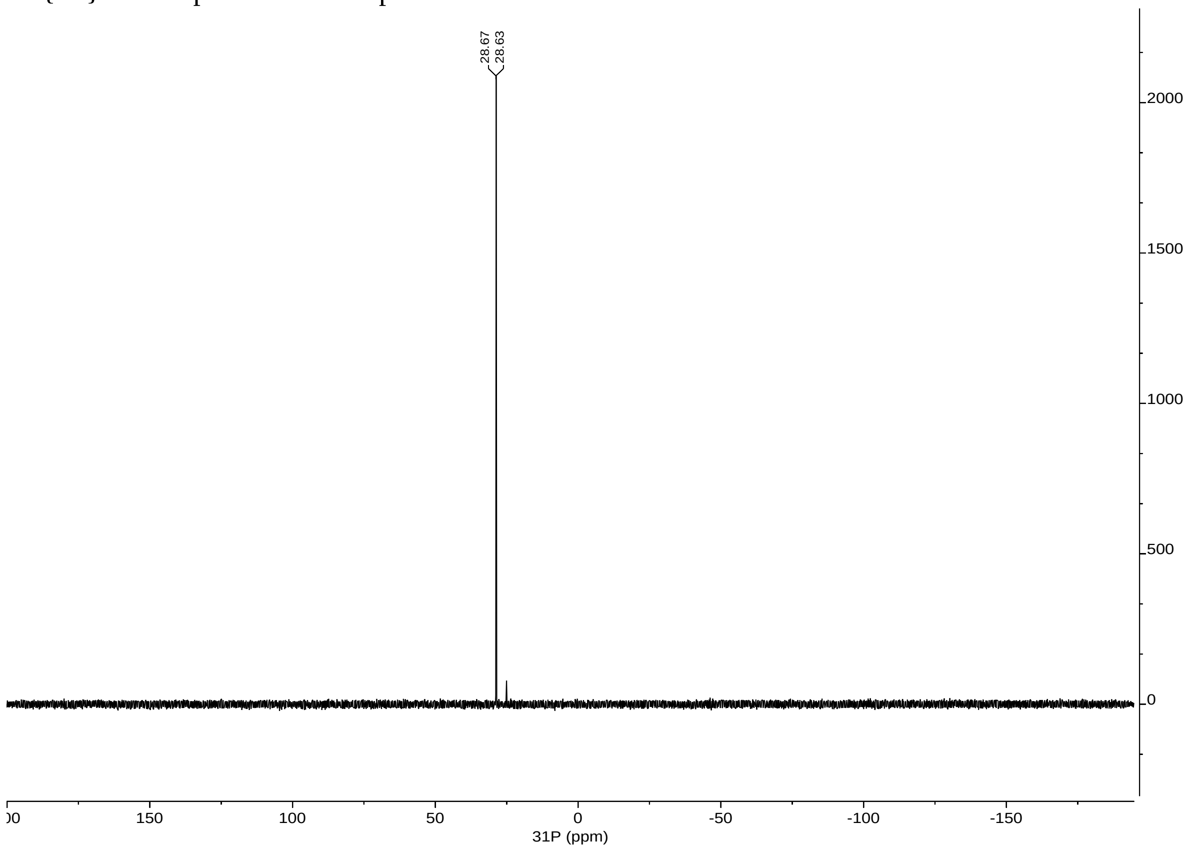

LC-MS chromatograms for compound **29**

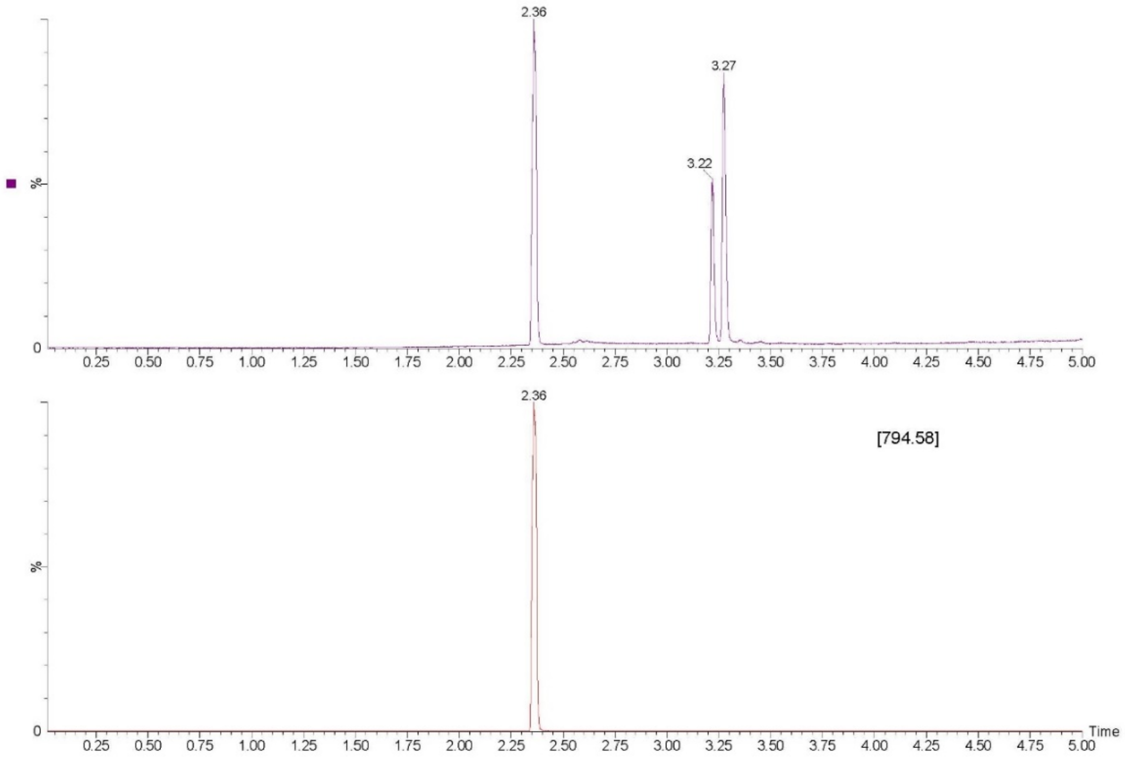

Bis((*Z*)-non-3-en-1-yl) pentane-1,5-diyl bis((2-(bis(3-guanidinopropyl)amino)ethyl)phosphonate) hexahydrochloride (30)

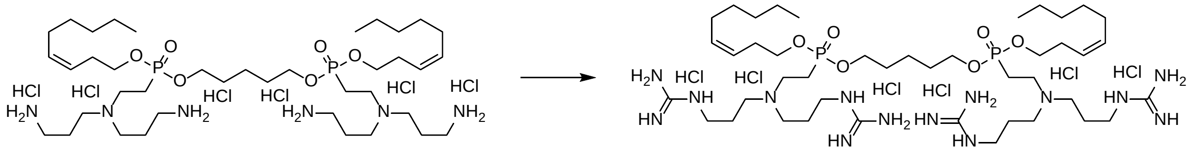

The title compound was prepared according to general method **F** from LPPO **29** (0.30 g, 0.30mmol) in 66% yield (0.23 g, 0.20 mmol) as a white solid.

Mixture of diastereoisomers.

^{1}H NMR (401 MHz, CD_3OD): 5.63–5.51 (m, 2H, $CH_3(CH_2)_4CH$), 5.49–5.36 (m, 2H, $CH(CH_2)_2O$), 4.24–4.05 (m, 8H, CH_2O), 3.52–3.40 (m, 4H, PCH_2CH_2), 3.41–3.32 (m, 16H, $CH_2CH_2CH_2NH$), 2.63–2.44 (m, 8H, $CHCH_2CH_2O$, PCH_2), 2.16–2.03 (m, 12H, $CH_3(CH_2)_3CH_2$, CH_2CH_2NH), 1.78 (p, 4H, J = 6.6 Hz, $CH_2CH_2CH_2O$), 1.61–1.49 (m, 2H, $CH_2(CH_2)_2O$), 1.44–1.26 (m, 12H, $CH_3(CH_2)_3$), 0.97–0.85 (m, 6H, CH_3).

^{13}C NMR (101 MHz, CD_3OD): 158.69 (C=NH), 134.42 ($CH_3(CH_2)_4CH$), 125.03 ($CH(CH_2)_2O$), 67.94 (d, J = 6.7 Hz, $CHCH_2CH_2O$), 67.63 (d, J = 6.7 Hz, $OCH_2(CH_2)_3CH_2O$), 51.57 (CH_2NH), 48.68 (PCH_2CH_2), 39.69 ($CH_2(CH_2)_2NH$), 32.66 ($CH_3CH_2CH_2$), 31.01 (d, J = 6.2 Hz), 30.99 (d, J = 5.8 Hz, $CH_2CH_2CH_2O$), 30.41 ($CH_3(CH_2)_2CH_2$), 29.65 (d, J = 6.1 Hz, $CHCH_2CH_2O$), 28.38 ($CH_3(CH_2)_3CH_2$), 24.78 ($CH_2CH_2NH_2$), 23.64, 22.81 ($CH_3(CH_2)_2$), 22.78 ($CH_2(CH_2)_2O$), 21.35 (d, J = 140.4 Hz, PCH_2), 14.46 (CH_3).

^{31}P{^{1}H} NMR (162 MHz, CD_3OD): 28.84, 28.81.

IR ν_{max} 3400–2400 (s), 3009 (m), 2956 (m), 2930 (m), 2871 (m), 2859 (s), 1666 (s), 1646 (s), 1619 (s), 1467 (m), 1378 (w), 1229 (m), 1203 (m), 1009 (m), 721 (w).

HR-MS(ESI^{+}): For $C_{43}H_{93}O_6N_{14}P_2$ $(M+H)^+$ m/z calculated 963.68723, found 963.68764.

^{1}H NMR spectrum of compound **30**

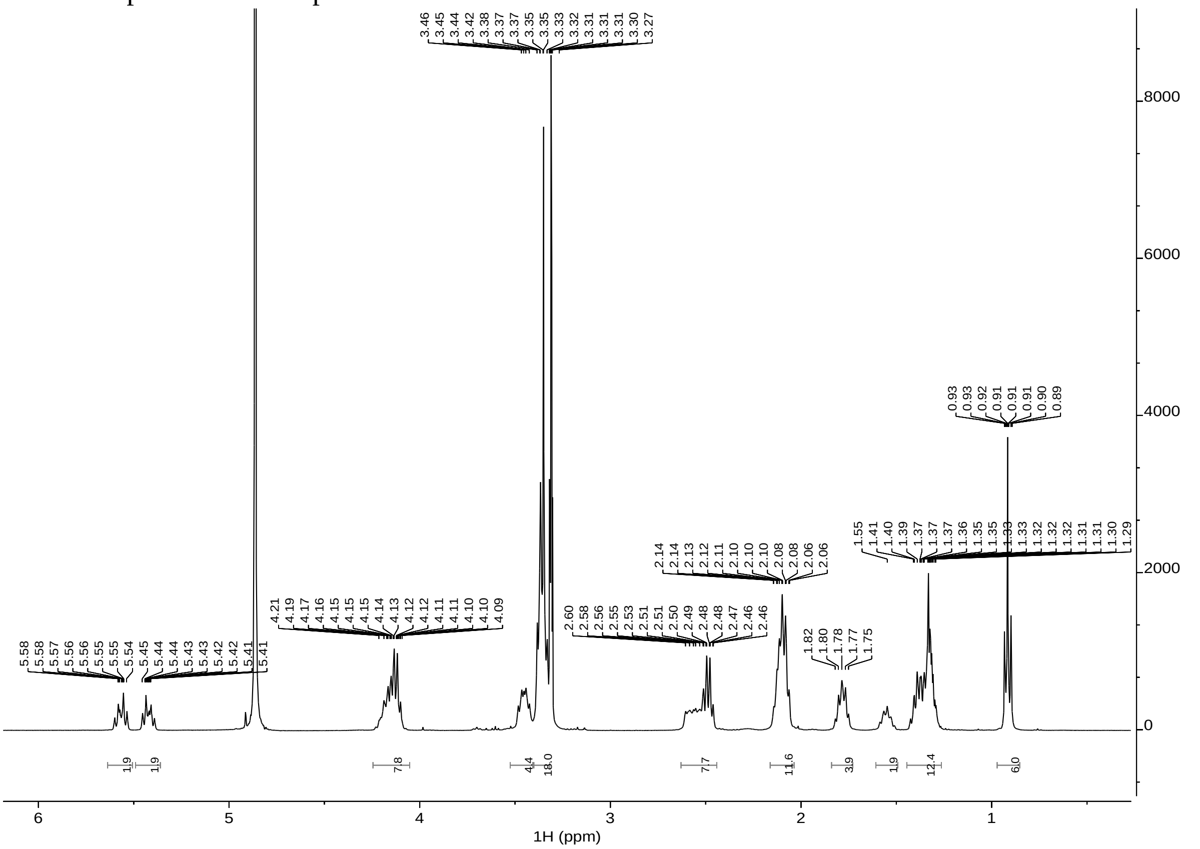

[13] C NMR spectrum of compound **30**

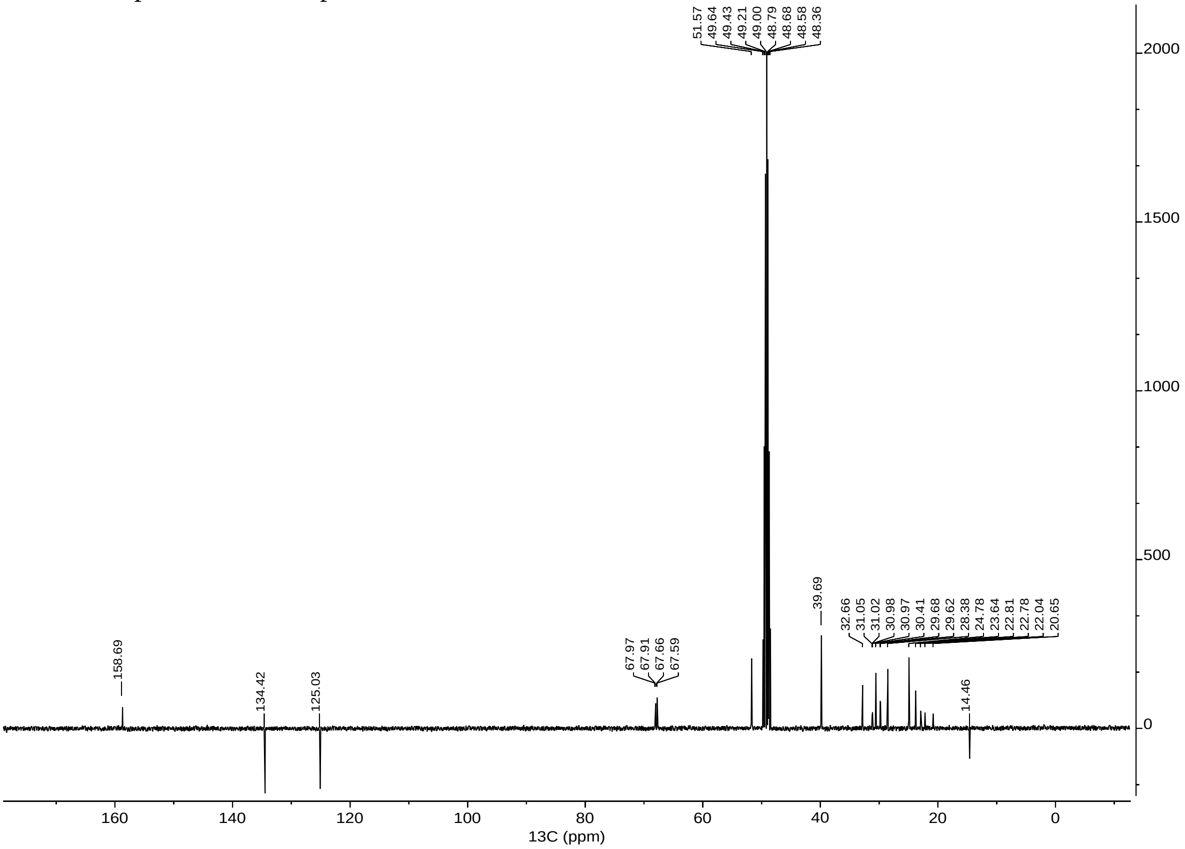

[31] P{[1] H} NMR spectrum of compound **30**

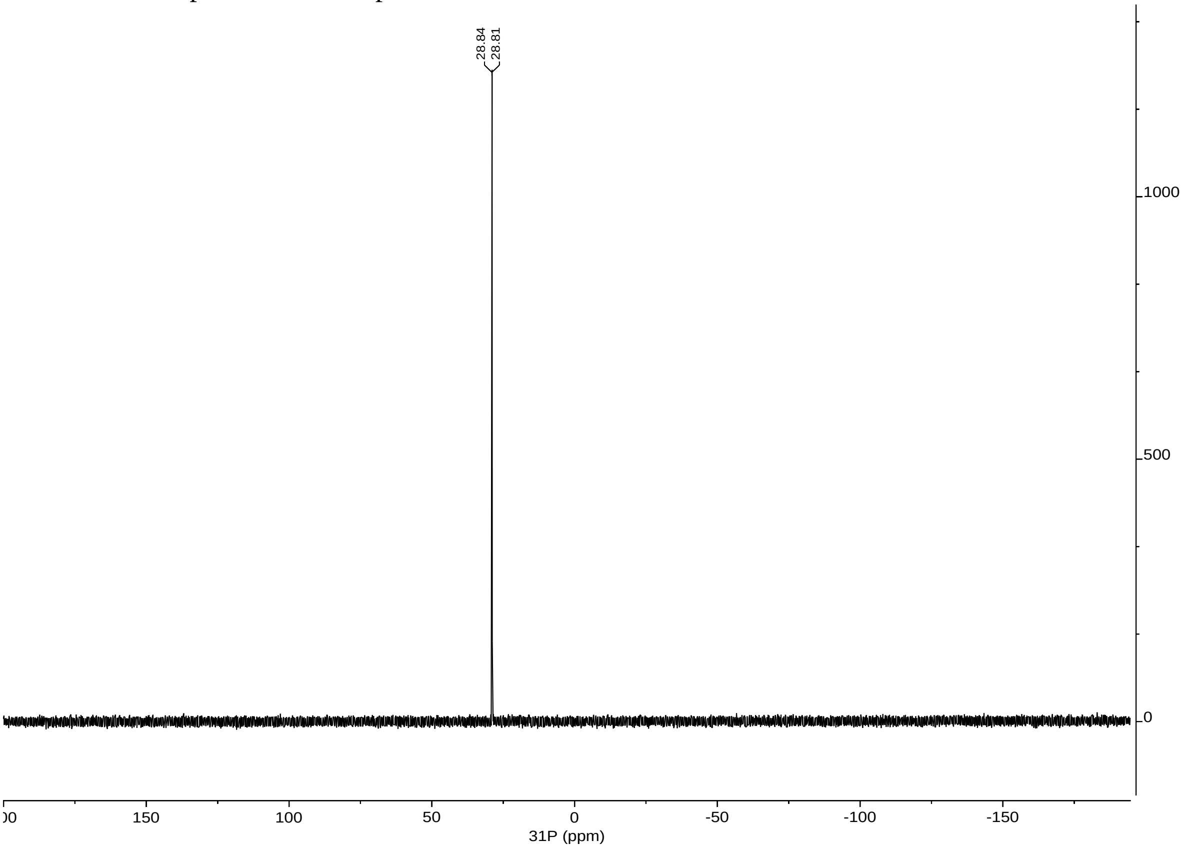

LC-MS chromatograms for compound **30**

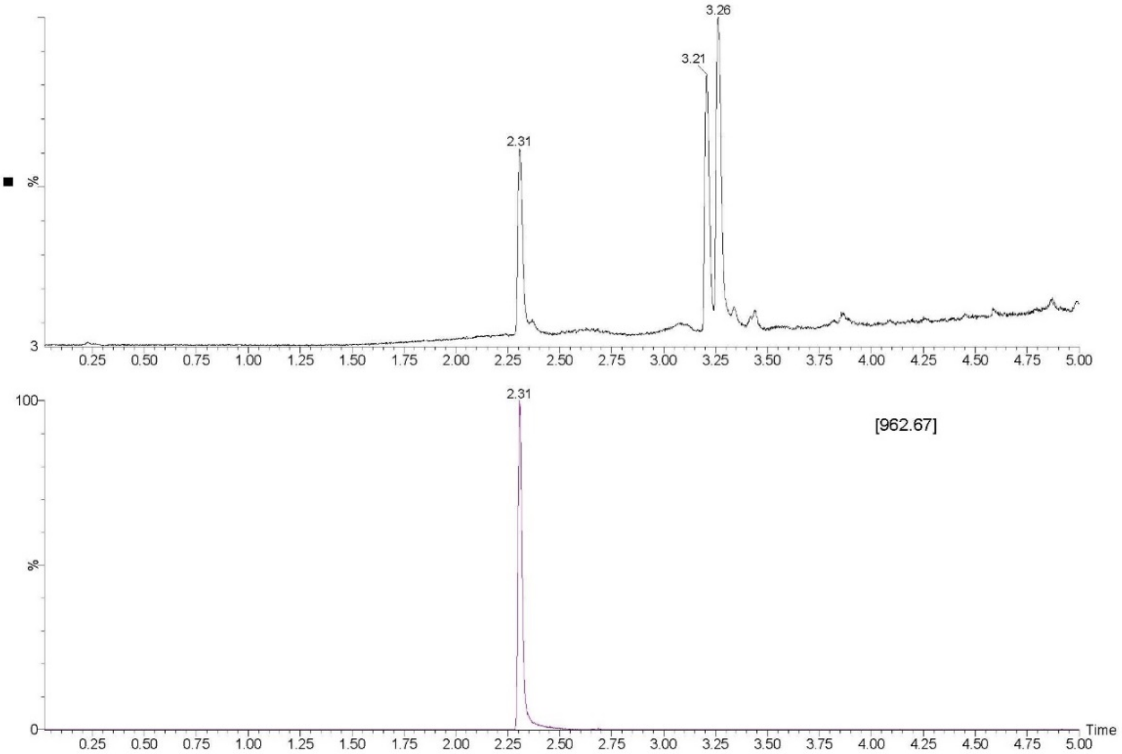

Bis((adamantan-1-yl)methyl) pentane-1,5-diyl bis((2-(bis(3-aminopropyl)amino)ethyl)phosphonate) hexahydrochloride (31)

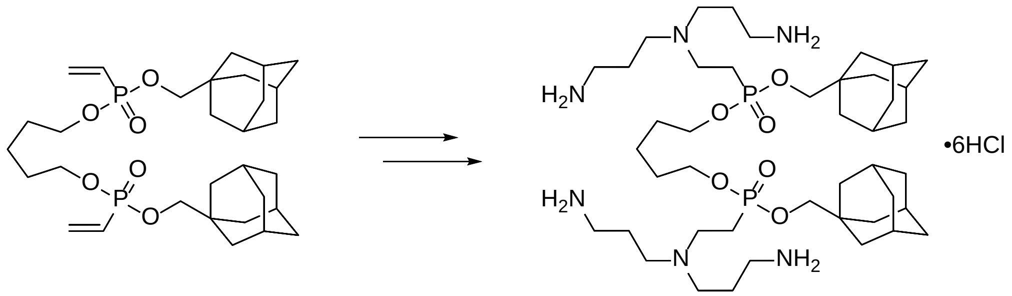

The title compound was prepared according to general methods **D** and **E** from vinylphosphonate dimer **S53** (1.40 g, 2.41 mmol) in 51% overall yield (1.32 g, 1.24 mmol) as a white solid.

Mixture of diastereoisomers.

^{1}H NMR (401 MHz, CD_3OD): 4.26–4.10 (m, 4H, OCH_2C$_{quat}$), 3.70 (qd, 4H 9.6, 5.6 Hz, OCH_2CH$_2$), 3.51–3.36 (m, 12H, NCH_2), 3.13 (t, 8H, *J* = 7.4 Hz, CH_2NH$_2$), 2.61 (dtd, 4H, *J* = 17.5, 9.0, 8.5, 4.8 Hz, PCH_2), 2.30–2.16 (m, 8H, CH_2CH$_2$NH$_2$), 2.01 (p, 6H, *J* = 3.1 Hz, C*H*), 1.85–1.67 (m, 16H, OCH$_2$CH_2, C$_{quat}$CH_2CH), 1.63–1.52 (m, 14H, *c*-(CHCH_2)$_3$, O(CH$_2$)$_2$CH_2).

^{13}C NMR (101 MHz, CD_3OD): 77.41 (d, *J* = 7.0 Hz, O*C*H$_2$C$_{quat}$), 68.02 (d, *J* = 6.8 Hz, O*C*H$_2$CH$_2$), 51.03 (*C*H$_2$(CH$_2$)$_2$NH$_2$), 48.93 (PCH$_2$*C*H$_2$), 39.88 (*c*-(CH*C*H$_2$)$_3$), 37.94 (C$_{quat}$*C*H$_2$CH), 37.89 (*C*H$_2$NH$_2$), 34.96 (d, *J* = 6.7 Hz, *C*$_{quat}$), 31.01 (d, *J* = 6.1 Hz), 30.97 (d, *J* = 6.1 Hz, OCH$_2$*C*H$_2$), 29.44 (*C*H), 23.22 (*C*H$_2$CH$_2$NH$_2$), 22.87, 22.84 (O(CH$_2$)$_2$*C*H$_2$), 21.23 (d, *J* = 140.1 Hz), 21.22 (d, *J* = 140.1 Hz, P*C*H$_2$).

^{31}P{^{1}H} NMR (162 MHz, CD_3OD) δ 28.77, 28.73.

IR ν_{max} (KBr) 3000 (vs, vbr), 2932 (vs, sh), 2903 (vs), 2847 (vs), 2800–2400 (s, vbr), 2676, 1656, 2043 (w, vbr), 1610 (m, br), 1515 (m, br, sh), 1474 (m, sh), 1464 (m), 1454 (m), 1397 (m, br), 1365 (w), 1344 (w), 1241 (m, sh), 1225 (m), 1155 (m), 1106 (m), 1061 (s), 1013 (s), 998 (s), 988 (s), 943 (m), 823 (m), 810 (w), 435 (w).

HR-MS(ESI$^+$): For $C_{43}H_{85}N_6O_6P_2$ $(M+H)^+$ *m*/*z* calculated 843.60003, found 843.59950.

^{1}H NMR spectrum of compound **31**

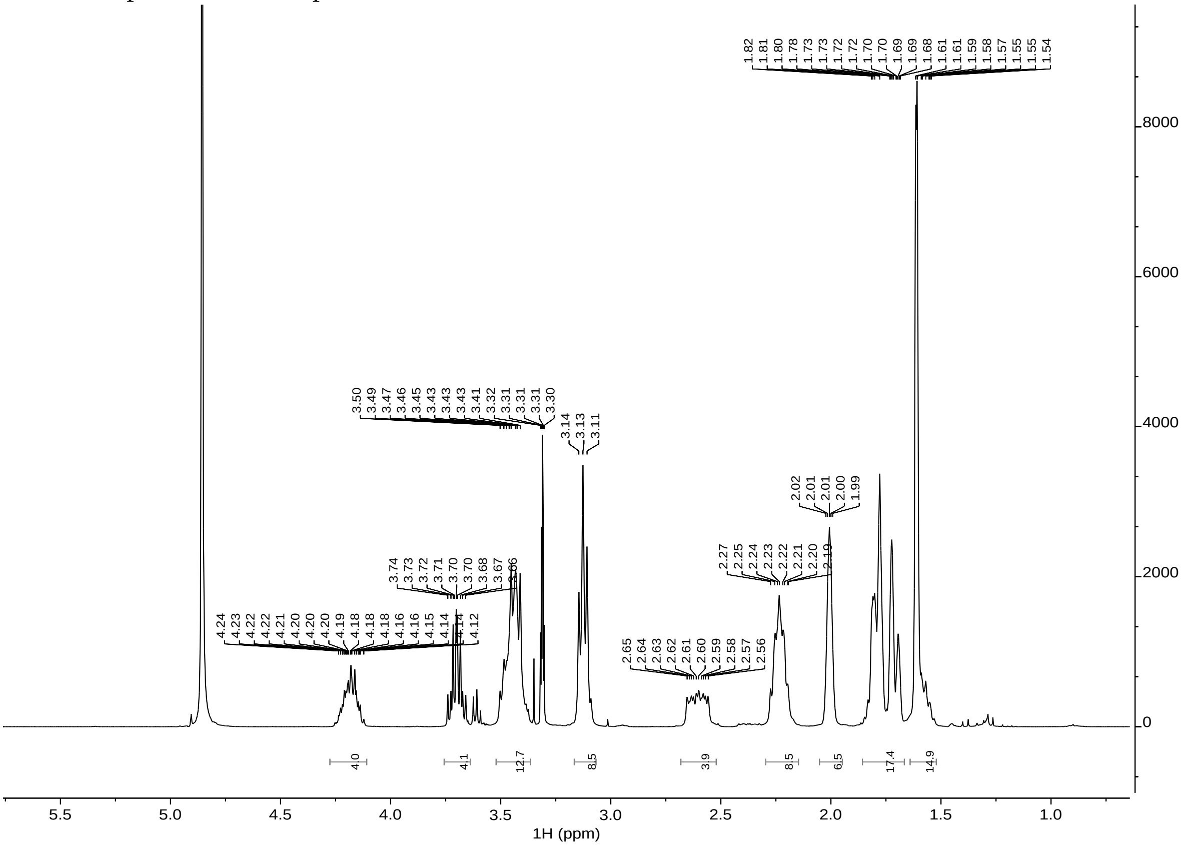

^{13}C NMR spectrum of compound **31**

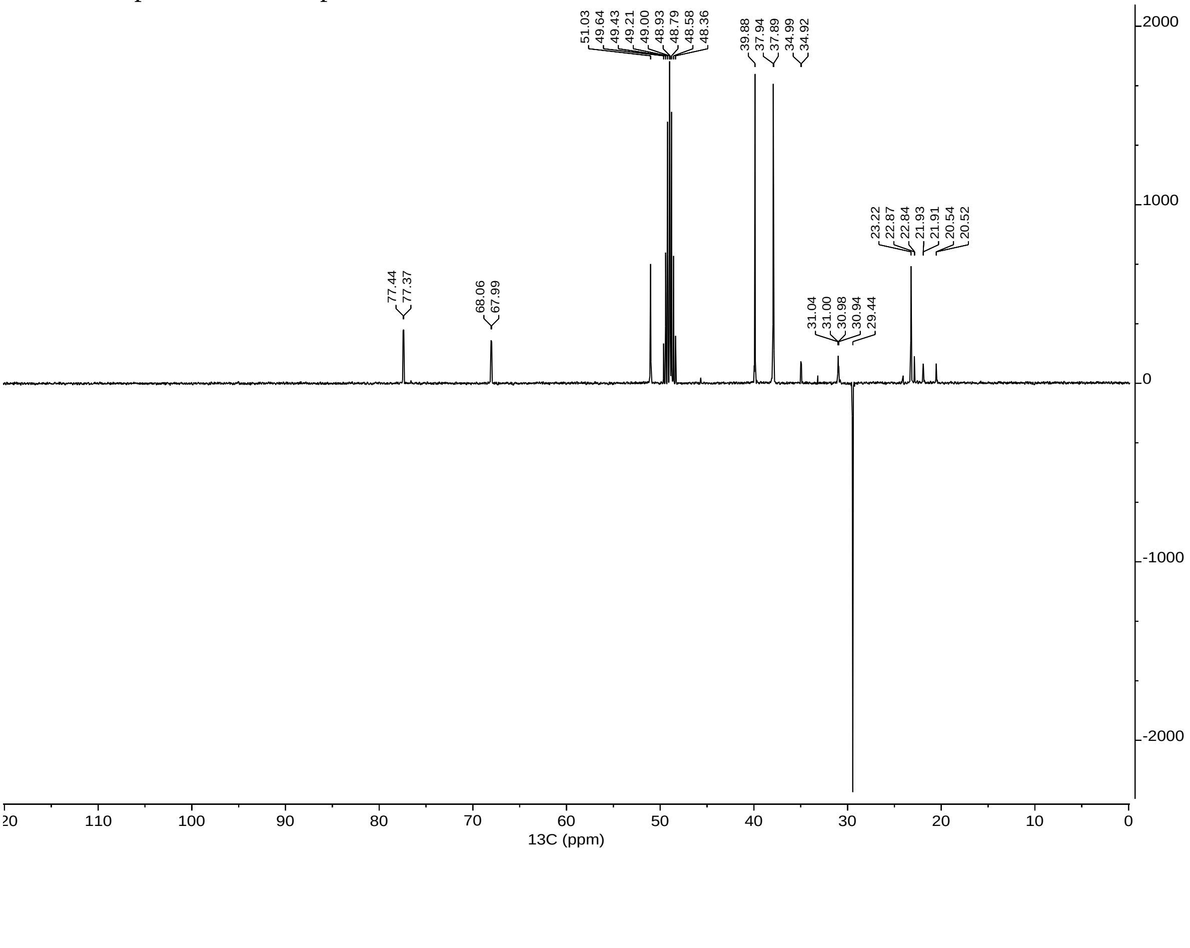

$^{31}P\{^1H\}$ NMR spectrum of compound **31**

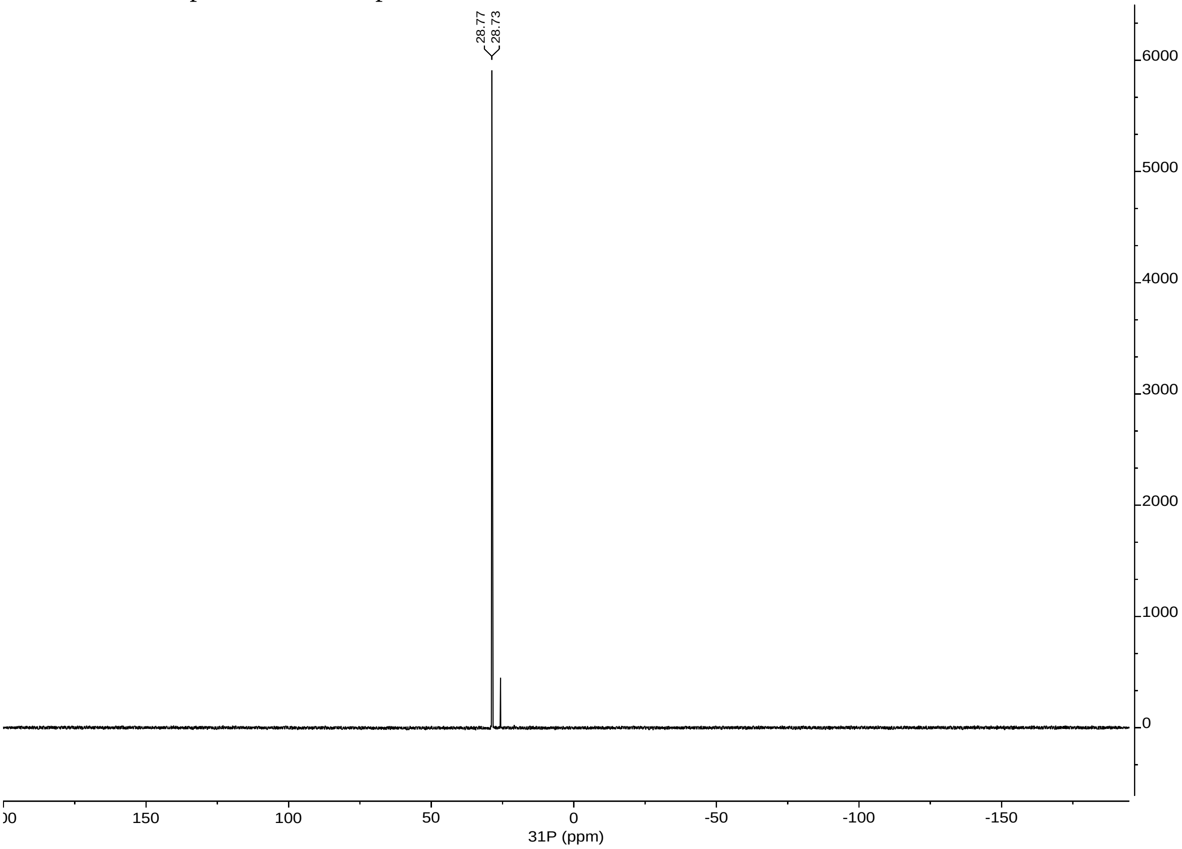

LC-MS chromatograms for compound **31**

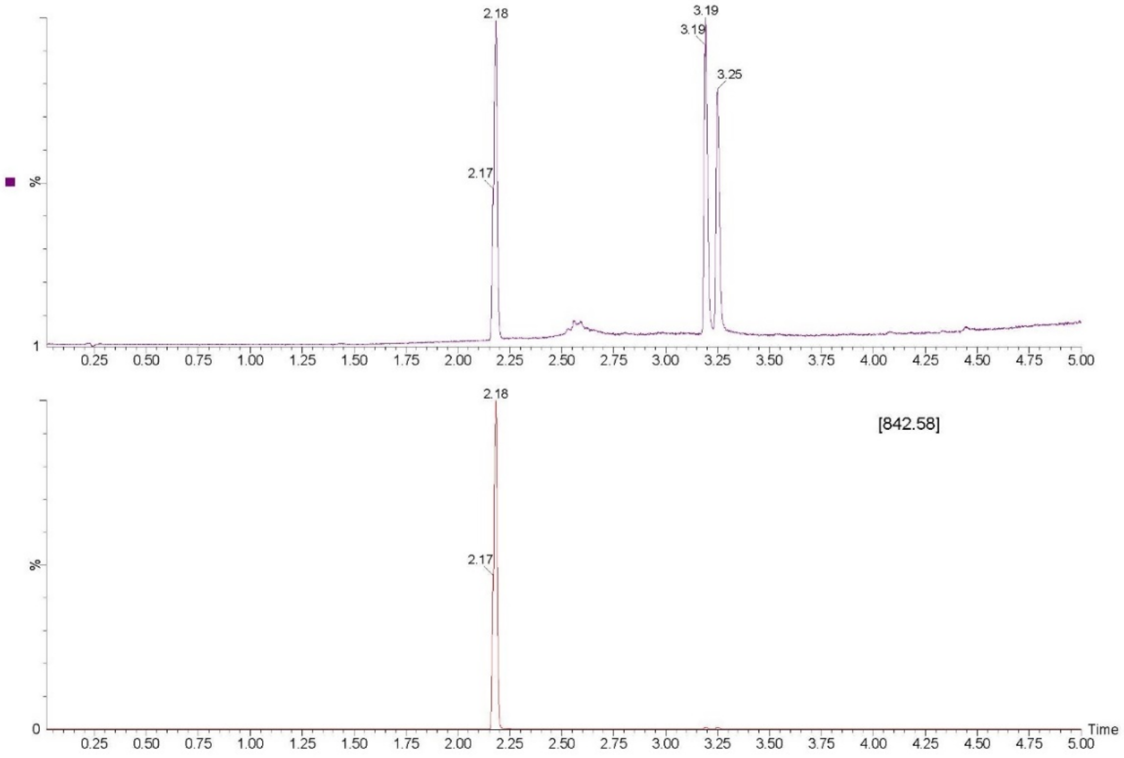

Bis((adamantan-1-yl)methyl) pentane-1,5-diyl bis((2-(bis(3-guanidinopropyl)amino)ethyl)phosphonate) hexahydrochloride (32)

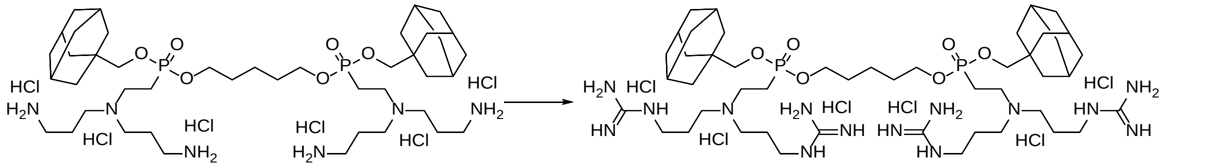

The title compound was prepared according to general method **F** from LPPO **31** (0.30 g, 0.28 mmol) in 72% yield (0.25 g, 0.20 mmol) as a white solid.
Mixture of diastereoisomers.
^{1}H NMR (500 MHz, CD_3OD) δ 4.25–4.12 (m, 4H, OC*H*$_2$CH$_2$), 3.69 (qd, *J* = 9.6, 5.5 Hz, 4H, OC*H*$_2$C), 3.50–3.41 (m, 4H, PCH$_2$C*H*$_2$), 3.40–3.32 (m, 16H, C*H*$_2$CH$_2$C*H*$_2$NH), 2.64–2.50 (m, 4H, PC*H*$_2$), 2.15–2.05 (m, 8H, C*H*$_2$CH$_2$NH), 2.01 (p, *J* = 3.1 Hz, 6H, CH$_2$C*H*), 1.84–1.67 (m, 16H, OCH$_2$C*H*$_2$, CHC*H*$_2$CH), 1.61 (d, *J* = 2.8 Hz, 12H), 1.59–1.51 (m, 2H, O(CH$_2$)$_2$C*H*$_2$).
^{13}C NMR (126 MHz, CD_3OD) δ 158.68 (*C*=NH), 77.38 (d, *J* = 7.2 Hz, O*C*H$_2$C), 68.01 (d, *J* = 6.7 Hz, O*C*H$_2$CH$_2$), 51.57 (*C*H$_2$NH), 48.62 (PCH$_2$*C*H$_2$), 39.92 (C*C*H$_2$CH), 39.68 (*C*H$_2$(CH$_2$)$_2$NH), 37.95 (CH*C*H$_2$CH), 34.98 (d, *J* = 6.8 Hz, OCH$_2$*C*), 31.06 (d, *J* = 5.8 Hz), 31.03 (d, *J* = 5.9 Hz, OCH$_2$*C*H$_2$), 29.46 (*C*H), 24.84, 22.83, 22.81 (O(CH$_2$)$_2$*C*H$_2$), 21.14 (d, *J* = 140.1 Hz, P*C*H$_2$).
^{31}P{^{1}H} NMR (202 MHz, CD_3OD) δ 26.30, 26.28.
IR ν_{max} 3400–2400 (w-s), 3322 (m), 3261 (m), 3143 (m), 2903 (m), 2847 (m), 1669 (s), 1646 (s), 1619 (s), 1453 (w), 1390 (w), 1366 (w), 1220 (m), 1014 (m), 721 (w).
HR-MS(ESI$^+$): For $C_{47}H_{94}O_6N_{14}P_2$ $(M+2H)^{2+}$ *m*/2*z* calculated 506.34725, found 506.34692.

^{1}H NMR spectrum of compound **32**

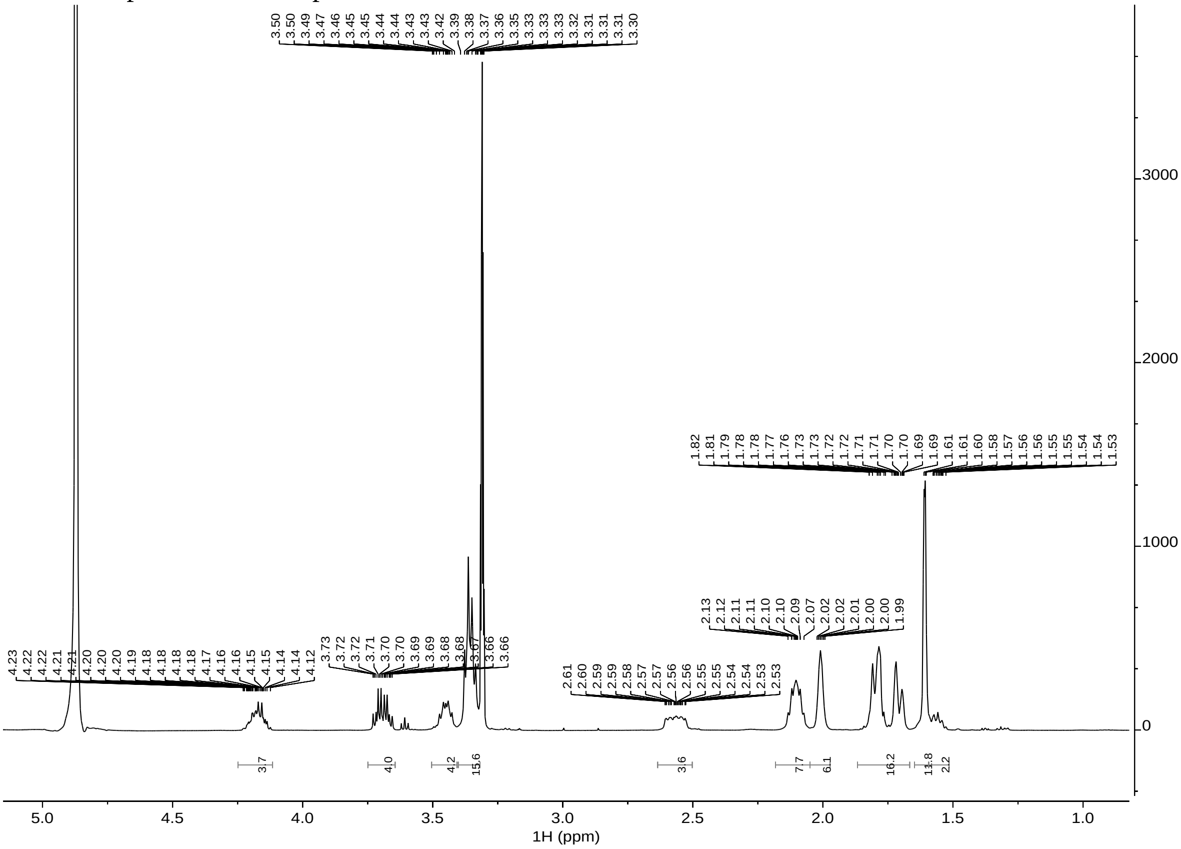

^{13}C NMR spectrum of compound **32**

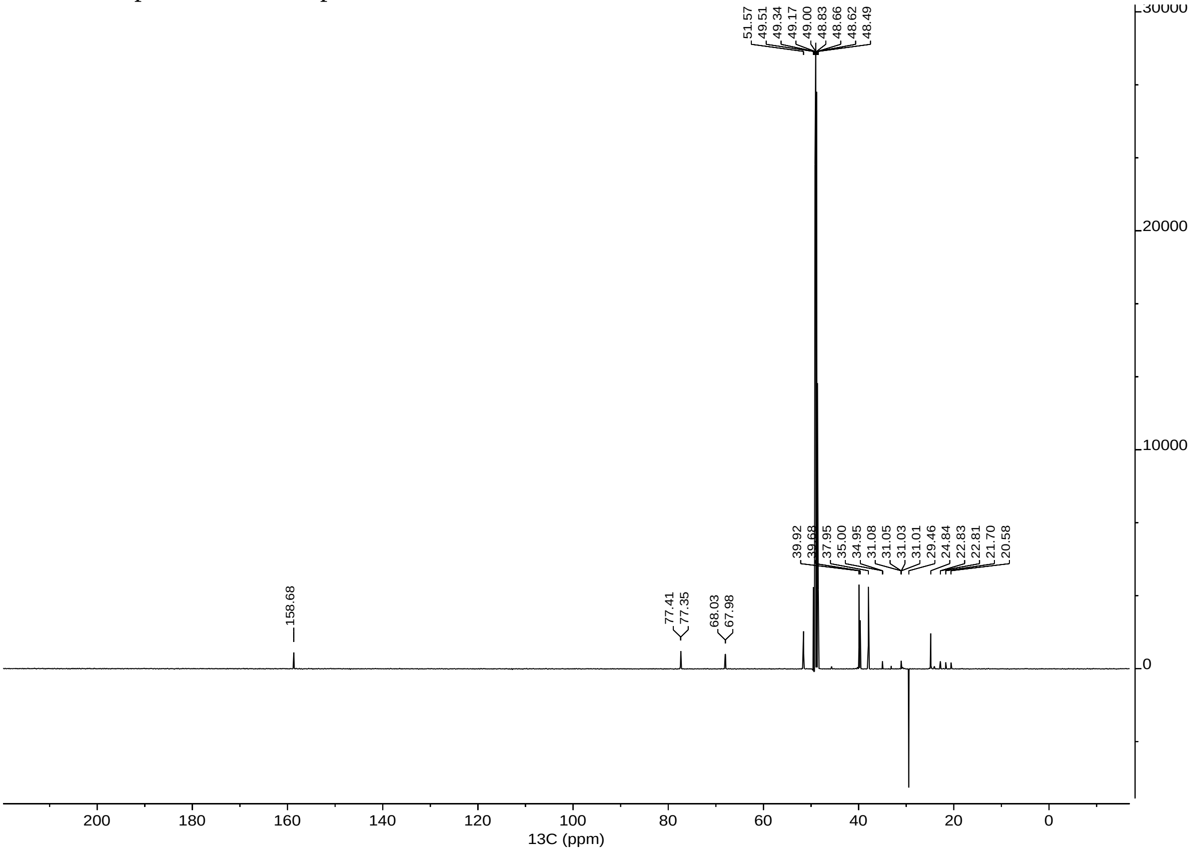

$^{31}P\{^{1}H\}$ NMR spectrum of compound **32**

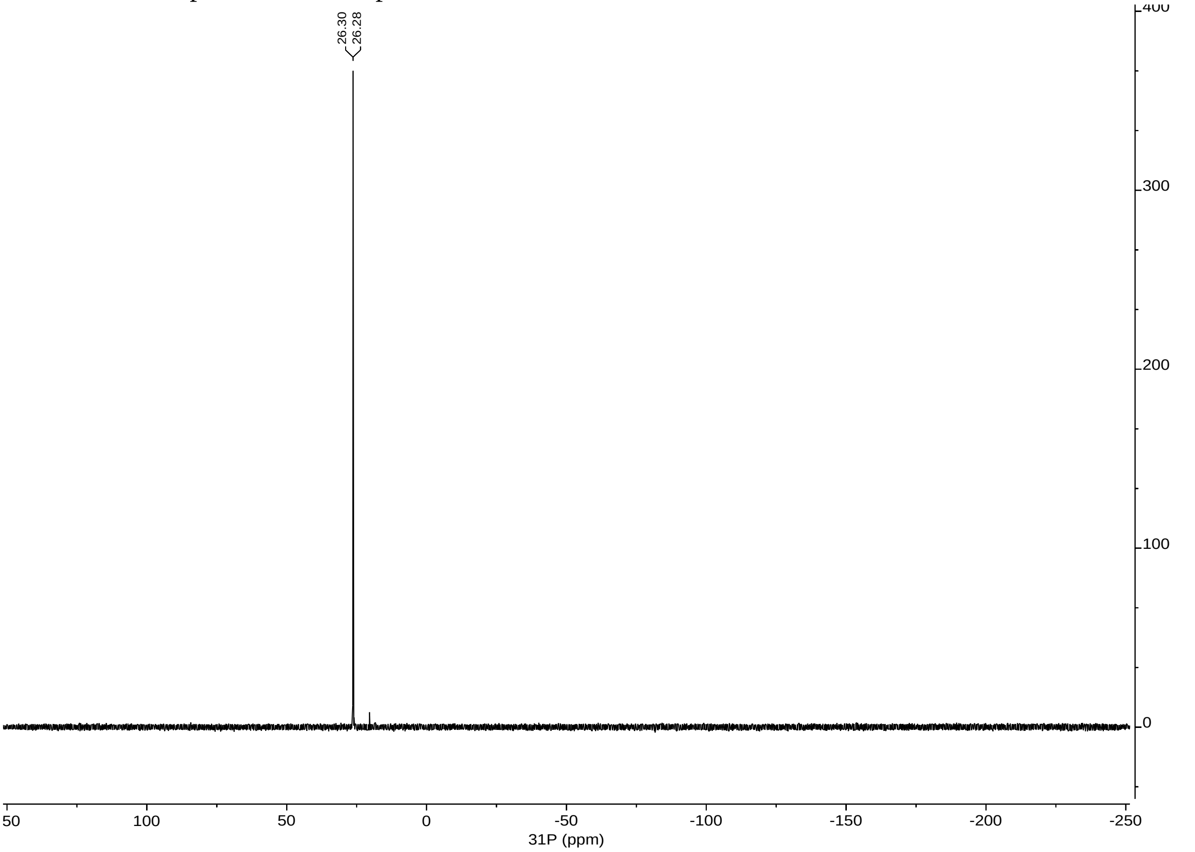

LC-MS chromatograms for compound **32**

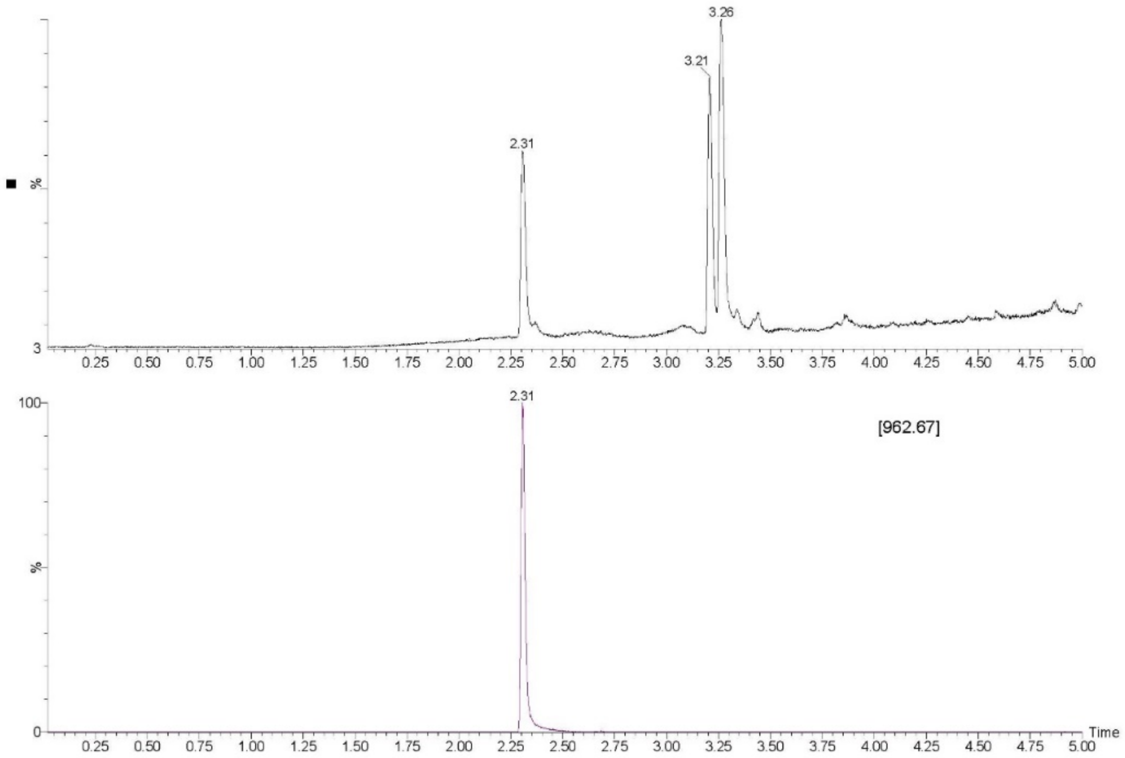

Hexane-1,6-diyl dihexyl bis((2-(bis(3-aminopropyl)amino)ethyl)phosphonate) hexahydrochloride (33)

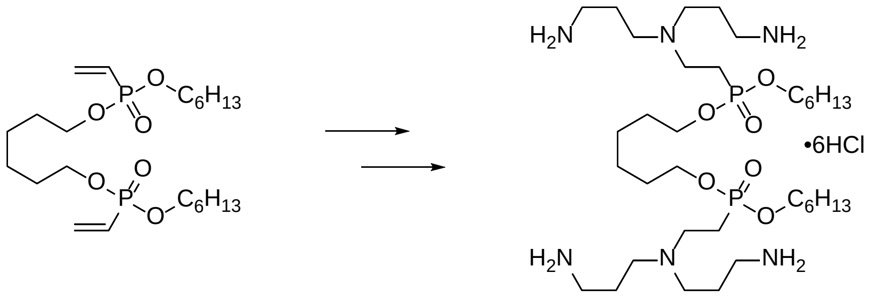

The title compound was prepared according to general methods **D** and **E** from vinylphosphonate dimer **S54** (0.85 g, 1.83 mmol) in 22% overall yield (0.39 g, 0.41 mmol) as a white solid.
Mixture of diastereoisomers.
^{1}H NMR (500.2 MHz, CD_3OD): 4.21–4.09 (m, 8H, C*H*$_2$O), 3.50–3.43 (m, 4H, PCH$_2$C*H*$_2$), 3.43–3.36 (m, 8H, C*H*$_2$(CH$_2$)$_2$NH$_2$), 3.43–3.36 (m, 8H, C*H*$_2$NH$_2$), 3.11 (t, 8H, *J* = 7.5 Hz, C*H*$_2$CH$_2$NH$_2$), 2.61–2.50 (m, 4H, PC*H*$_2$), 1.80–1.68 (m, 8H, C*H*$_2$CH$_2$O), 1.52–1.32 (m, 16H, CH$_3$(C*H*$_2$)$_2$, C*H*$_2$(CH$_2$)$_2$O), 0.95–0.90 (m, 6H, C*H*$_3$).
^{13}C NMR (125.8 MHz, CD_3OD): 68.25 (d, *J* = 6.7 Hz), 68.07 (d, *J* = 6.6 Hz), 68.05 (d, *J* = 6.6 Hz, *C*H$_2$O), 51.07 (*C*H$_2$(CH$_2$)$_2$NH$_2$), 48.87 (PCH$_2$*C*H$_2$), 37.90 (*C*H$_2$NH$_2$), 32.49 (CH$_3$CH$_2$*C*H$_2$), 31.55 (d, *J* = 5.9 Hz), 31.40 (d, *J* = 5.7 Hz), 31.37 (d, *J* = 5.7 Hz, *C*H$_2$CH$_2$O), 26.29. 26.15, 26.14, 23.81 (CH$_3$*C*H$_2$), 23.26 (*C*H$_2$CH$_2$NH$_2$), 21.35 (d, *J* = 140.4 Hz), 21.33 (d, *J* = 140.7 Hz, P*C*H$_2$), 14.37 (*C*H$_3$).
^{31}P{^{1}H} NMR (202.5 MHz, CD_3OD): 26.62.
IR ν_{max} ($CHCl_3$) 2957 (vs), 2933 (vs), 2860 (s), 2745 (s, br), 2640 (m, br), 2558 (m, br), 2051 (w, br), 1615 (m, br), 1513 (w, br), 1468 (m), 1397 (w, br), 1379 (w, sh), 1265 (m, sh), 1229 (m, br), 1060 (m), 1040 (m, sh), 998 (s).
HR-MS(ESI$^+$): For $C_{34}H_{80}O_6P_2$ $(M+2H)^{2+}$ *m*/2*z* calculated 365.28018, found 365.28030.

^{1}H NMR spectrum of compound **33**

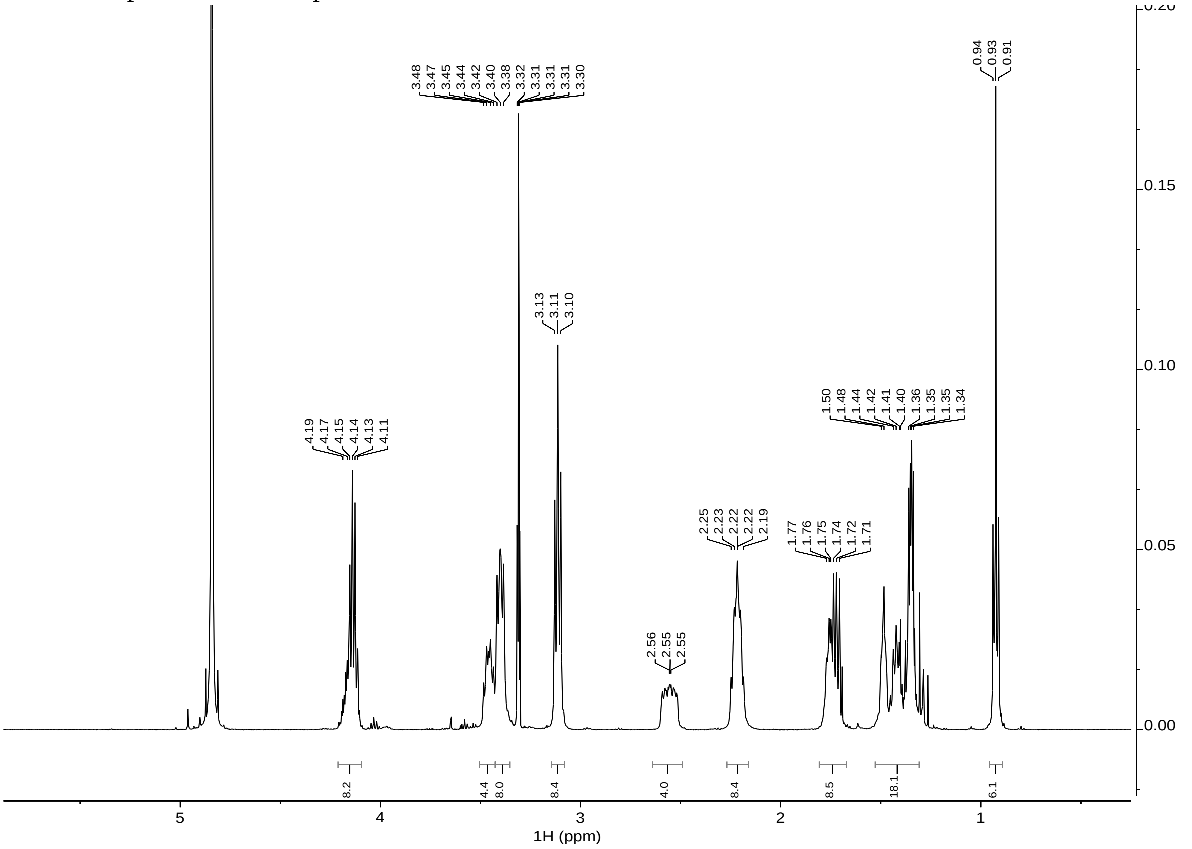

^{13}C NMR spectrum of compound **33**

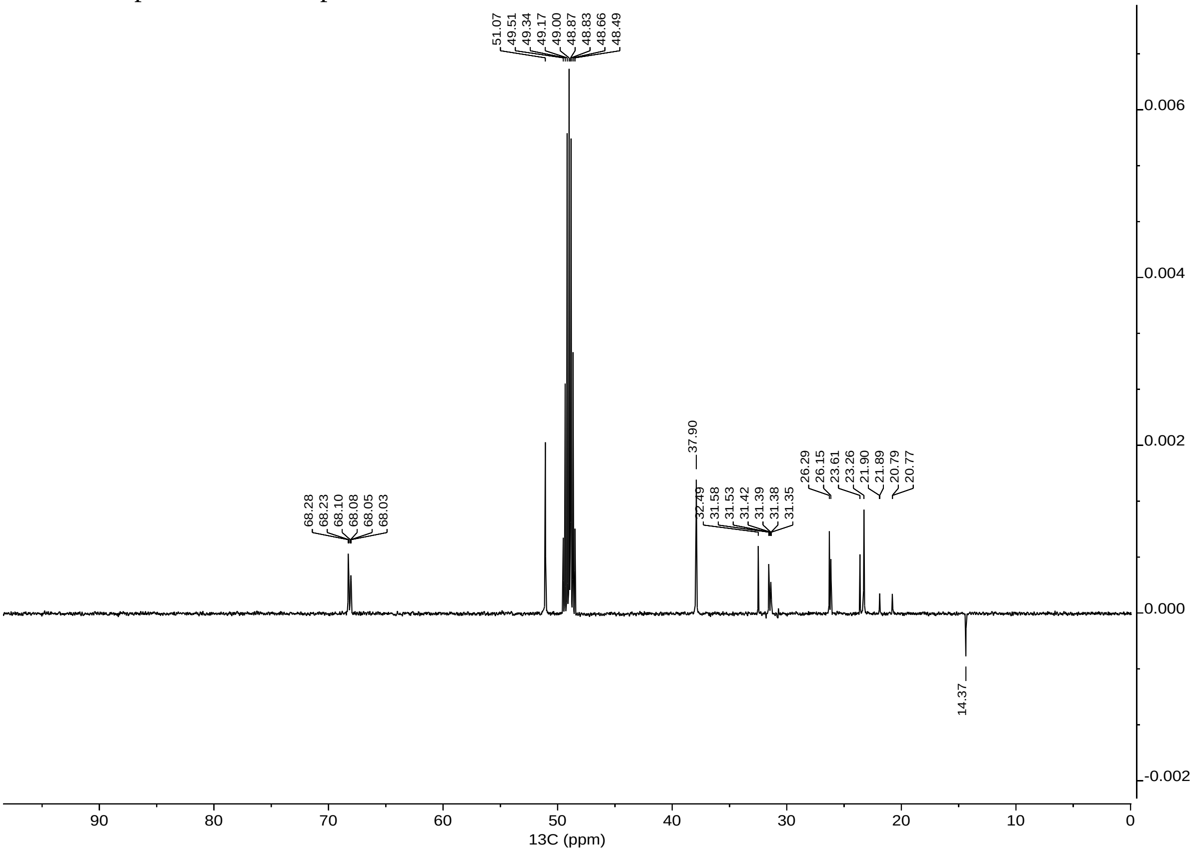

$^{31}P\{^1H\}$ NMR spectrum of compound **33**

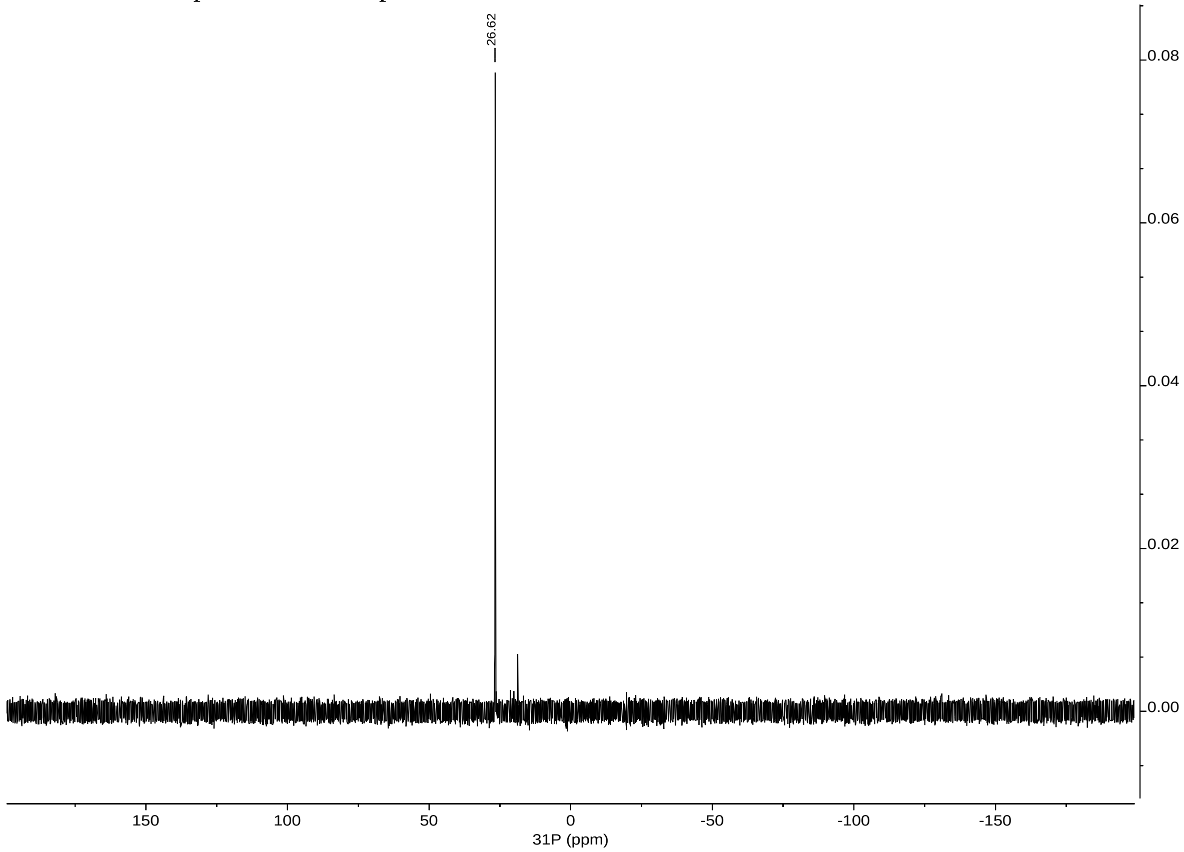

LC-MS chromatograms for compound **33**

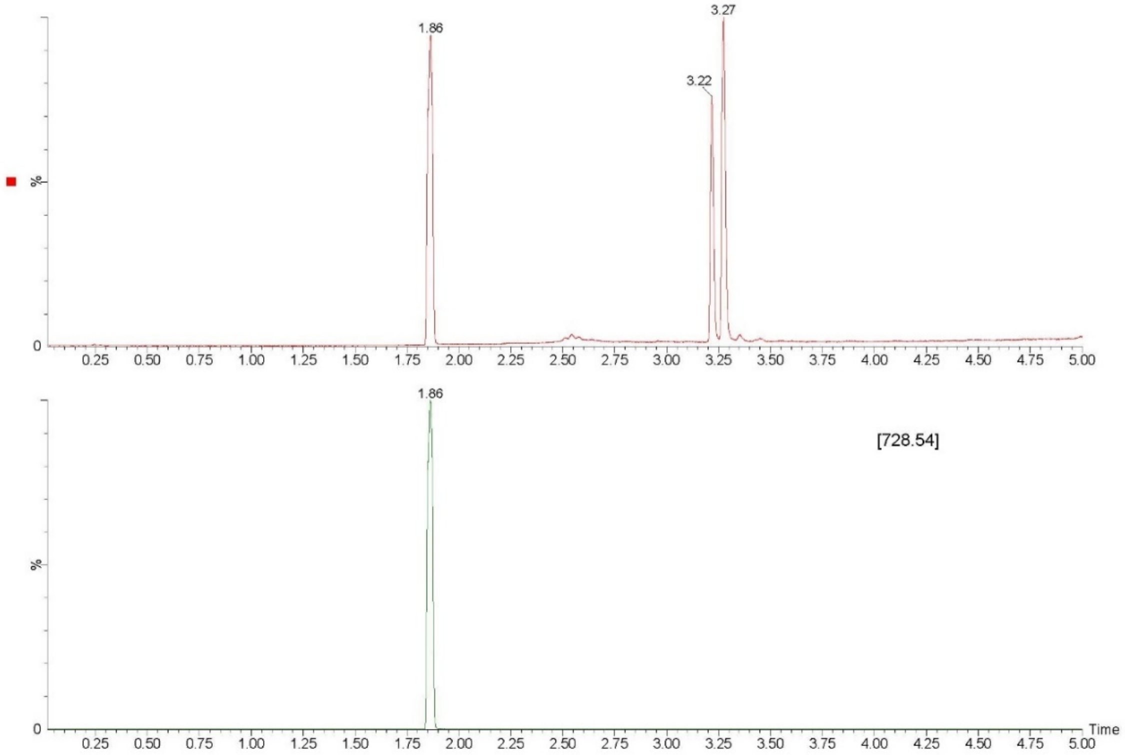

Bis(cyclopentylmethyl) hexane-1,6-diyl bis((2-(bis(3-aminopropyl)amino)ethyl)phosphonate) hexahydrochloride (34)

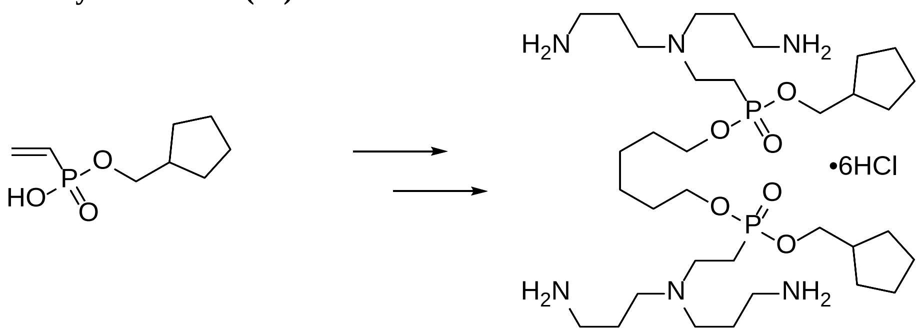

The title compound was prepared according to general methods **C**, **D** and **E** from mono cyclopentylmethyl vinylphosphonate **S24** in 15% overall yield (1.25 g, 1.22 mmol) as a white solid. Mixture of diastereoisomers.

^{1}H NMR (500.0 MHz, CD_3OD): 4.22–4.10 (m, 4H, OC*H*$_2$CH$_2$), 4.06–3.97 (m, 4H, OC*H*$_2$-cyclopent), 3.50–3.45 (m, 4H, PCH$_2$C*H*$_2$), 3.45–3.39 (m, 8H, C*H*$_2$(CH$_2$)$_2$NH$_2$), 3.12 (t, 8H, J = 7.4 Hz, C*H*$_2$NH$_2$), 2.63–2.52 (m, 4H, PC*H*$_2$), 2.28 (sep, 2H, J = 7.6 Hz, H-1-cyclopent), 2.27–2.18 (m, 8H, C*H*$_2$CH$_2$NH$_2$), 1.84–1.72 (m, 8H, H-2a,5a-cyclopent, OCH$_2$C*H*$_2$), 1.70–1.56 (m, 8H, *H*-3,4-cyclopent), 1.53–1.44 (m, 4H, O(CH$_2$)$_2$C*H*$_2$), 1.39–1.29 (m, 4H, H-2b,5b-cyclopent).

^{13}C NMR (125.7 MHz, CD_3OD): 71.77 (d, J = 6.9 Hz, OCH$_2$-cyclopent), 68.08 (d, $J_{C,P}$ = 6.7 Hz), 68.06 (d, $J_{C,P}$ = 6.7 Hz, O*C*H$_2$CH$_2$), 51.06 (*C*H$_2$(CH$_2$)$_2$NH$_2$), 48.92 (PCH$_2$*C*H$_2$), 41.33 (d, J = 6.1 Hz, CH-1-cyclopent), 37.88 (*C*H$_2$NH$_2$), 31.37 (d, J = 5.9 Hz), 31.35 (d, J = 5.9 Hz, OCH$_2$*C*H$_2$), 29.97, 29.96 (CH$_2$-2,5-cyclopent), 26.34 (CH$_2$-3,4-cyclopent), 26.13, 26.12 (O(CH$_2$)$_2$*C*H$_2$), 23.21 (*C*H$_2$CH$_2$NH$_2$), 21.36 (d, J = 140.0 Hz), 21.35 (d, J = 140.2 Hz, P*C*H$_2$).

^{31}P{^{1}H} NMR (202.4 MHz, CD_3OD): 26.70.

IR ν_{max} (KBr) 3000 (s, vbr), 2953 (vs), 2912 (vs, sh), 2868 (s), 2740 (s, br, sh), 2636 (s, vbr), 2559 (m, vbr), 2022 (w, vbr), 1604 (m, br), 1505 (m, sh, br), 1470 (m), 1396 (w), 1255 (m, br, sh), 1223 (m), 1075 (m, sh), 1025 (s, sh), 1000 (s).

HR-MS(ESI^{+}): For $C_{34}H_{75}N_6O_6P_2$ $(M+H)^{+}$ *m/z* calculated 725.52178, found 725.52100.

^{1}H NMR spectrum of compound **34**

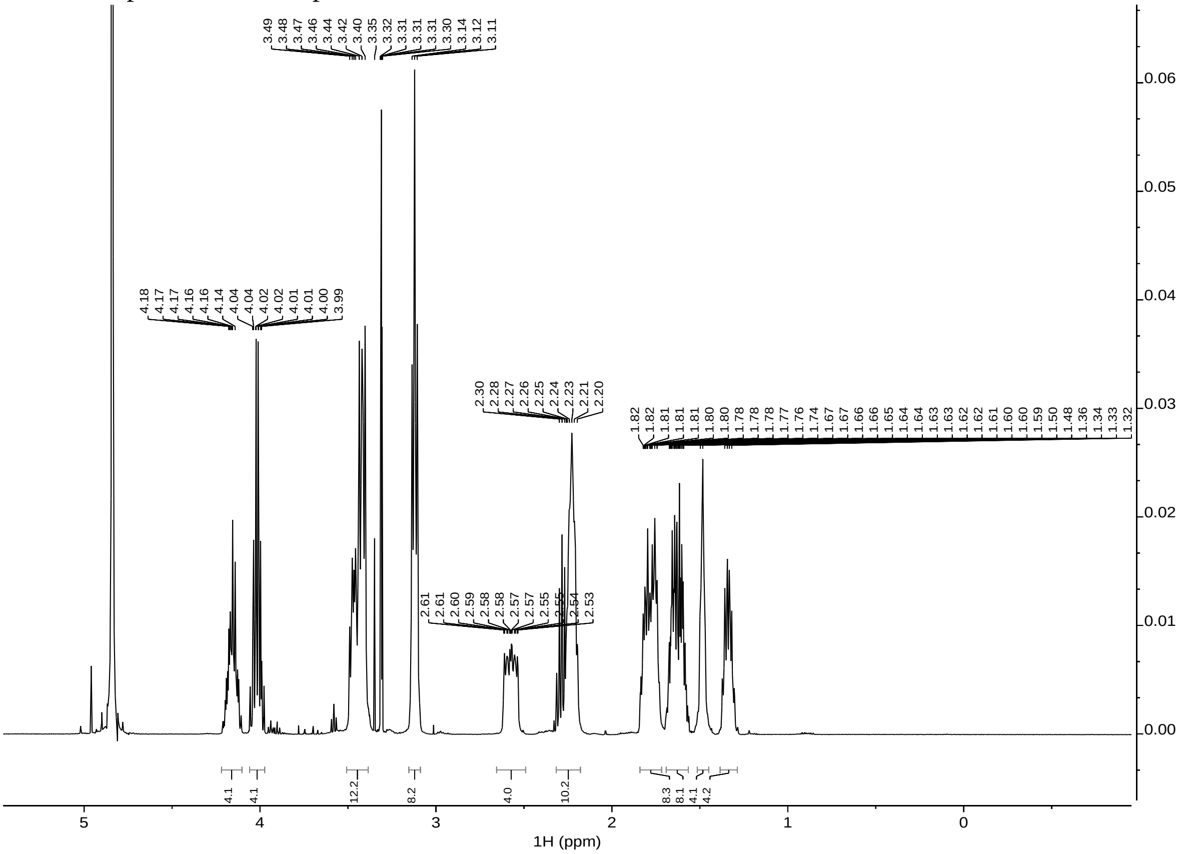

^{13}C NMR spectrum of compound **34**

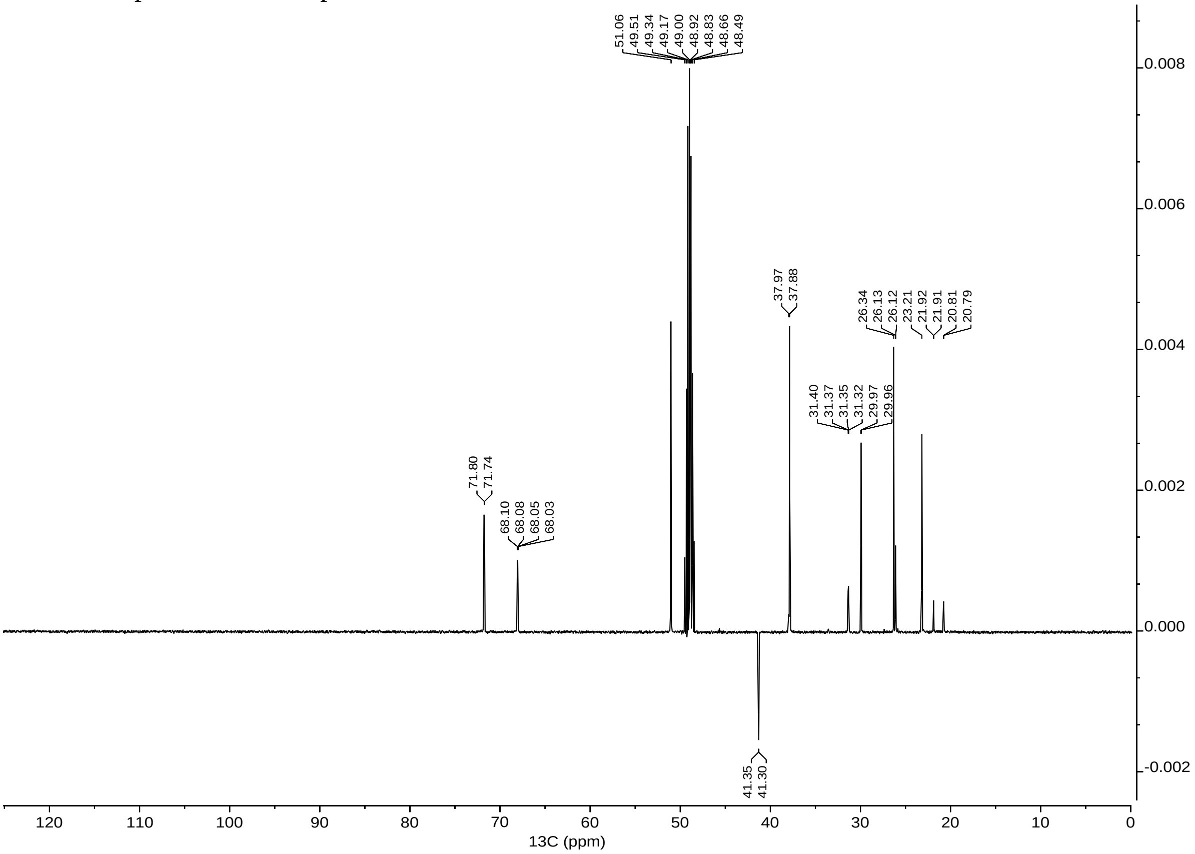

$^{31}P\{^1H\}$ NMR spectrum of compound **34**

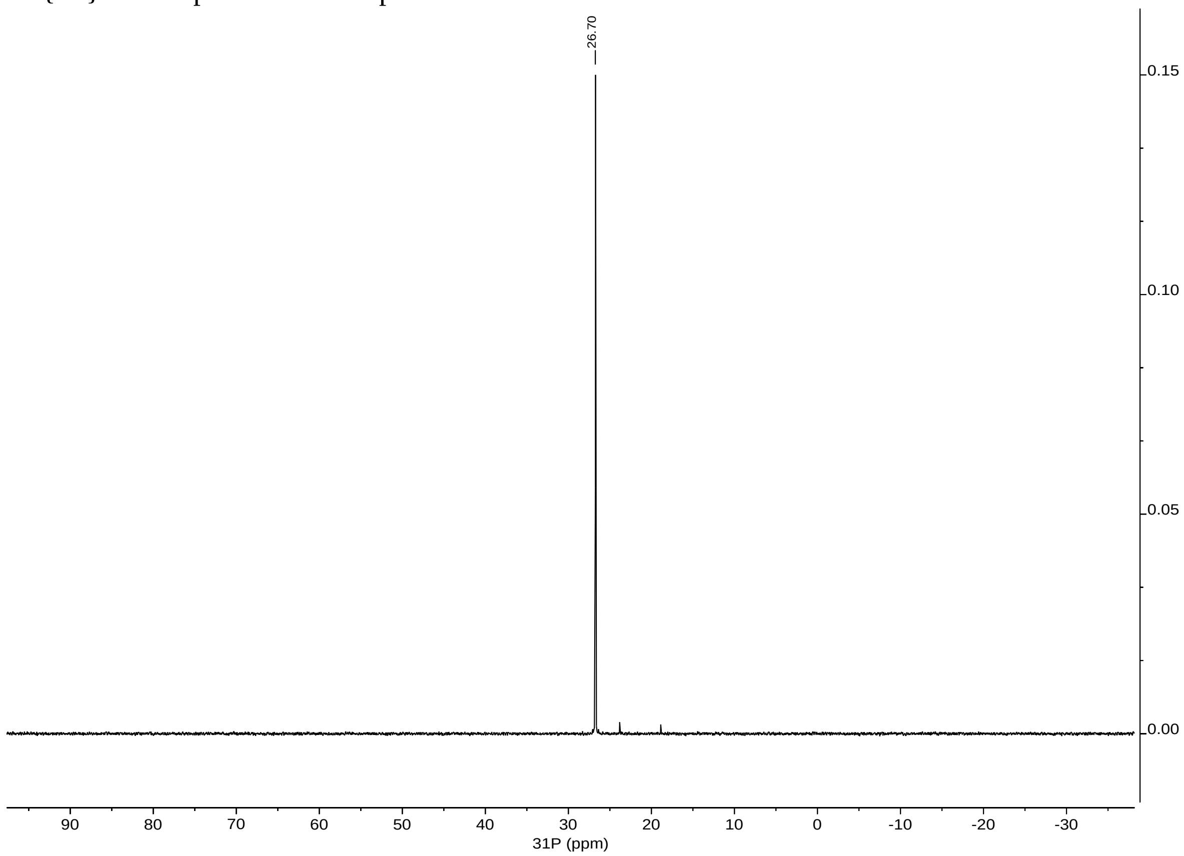

LC-MS chromatograms for compound **34**

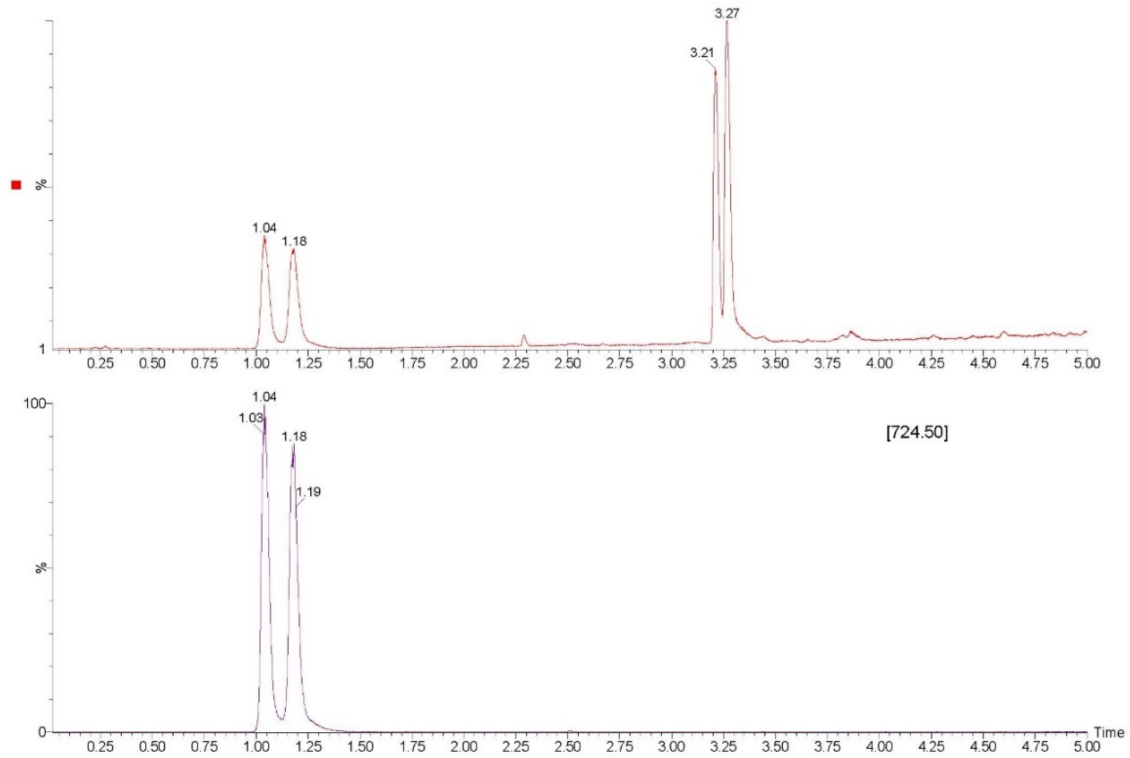

Bis((*Z*)-hept-3-en-1-yl) hexane-1,6-diyl bis((2-(bis(3-aminopropyl)amino)ethyl)phosphonate) hexahydrochloride (35)

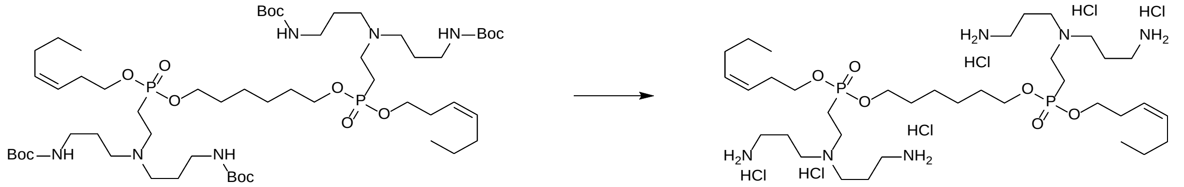

The title compound was prepared according to general method **E** from Boc derivative **S102** (0.81 g, 0.70 mmol) in 92% yield (0.58 g, 0.65 mmol) as a white solid.

Mixture of diastereoisomers.

^{1}H NMR (401 MHz, CD_3OD): 5.62–5.52 (m, 2H, $CH_3(CH_2)_2CH$), 5.48–5.39 (m, 2H, $CH(CH_2)_2O$), 4.24–4.06 (m, 8H, CH_2O), 3.52–3.35 (m, 12H, NCH_2), 3.11 (t, 8H, J = 7.6 Hz, CH_2NH_2), 2.63–2.45 (m, 8H, $CHCH_2CH_2O$, PCH_2), 2.27–2.15 (m, 8H, $CH_2CH_2NH_2$), 2.12–2.03 (m, 4H, $CH_3CH_2CH_2$), 1.81–1.70 (m, 4H, $CH_2CH_2CH_2O$), 1.51–1.46 (m, 4H, $CH_2(CH_2)_2O$), 1.46–1.36 (m, 4H, CH_3CH_2), 0.93 (t, 6H, J = 7.4 Hz, CH_3).

^{13}C NMR (101 MHz, CD_3OD): 134.14 ($CH_3(CH_2)_2CH$), 125.20 ($CH(CH_2)_2O$), 68.08 (d, J = 6.7 Hz), 68.06 (d, J = 6.7 Hz, $(CH_2)_2CH_2O$), 67.63 (d, J = 7.2 Hz, $CHCH_2CH_2O$), 51.05 ($CH_2(CH_2)_2NH_2$), 48.83, 48.82 (PCH_2CH_2), 37.88 (CH_2NH_2), 31.39 (d, J = 6.1 Hz), 31.36 (d, J = 6.0 Hz, $CH_2CH_2CH_2O$), 30.45, 29.66 (d, J = 6.2 Hz, $CHCH_2CH_2O$), 26.15 ($CH_2(CH_2)_2O$), 23.78 (CH_3CH_2), 23.26 ($CH_2CH_2NH_2$), 21.34 (d, J = 140.4 Hz), 21.32 (d, J = 140.4 Hz, PCH_2),14.15 (CH_3).

^{31}P{^{1}H} NMR (162 MHz, CD_3OD): 28.67.

IR ν_{max} (KBr) 3100–2500 (vs, vbr), 3013 (vs), 2957 (vs), 2932 (vs), 2872 (s), 1653 (w), 1609 (m), 1465 (m), 1379 (w, sh), 1230 (m), 1001 (s), 720 (w).

HR-MS(ESI$^+$): For $C_{36}H_{79}O_6N_6P_2$ $(M+H)^+$ *m*/*z* calculated 753.55308, found 753.55327.

^{1}H NMR spectrum of compound **35**

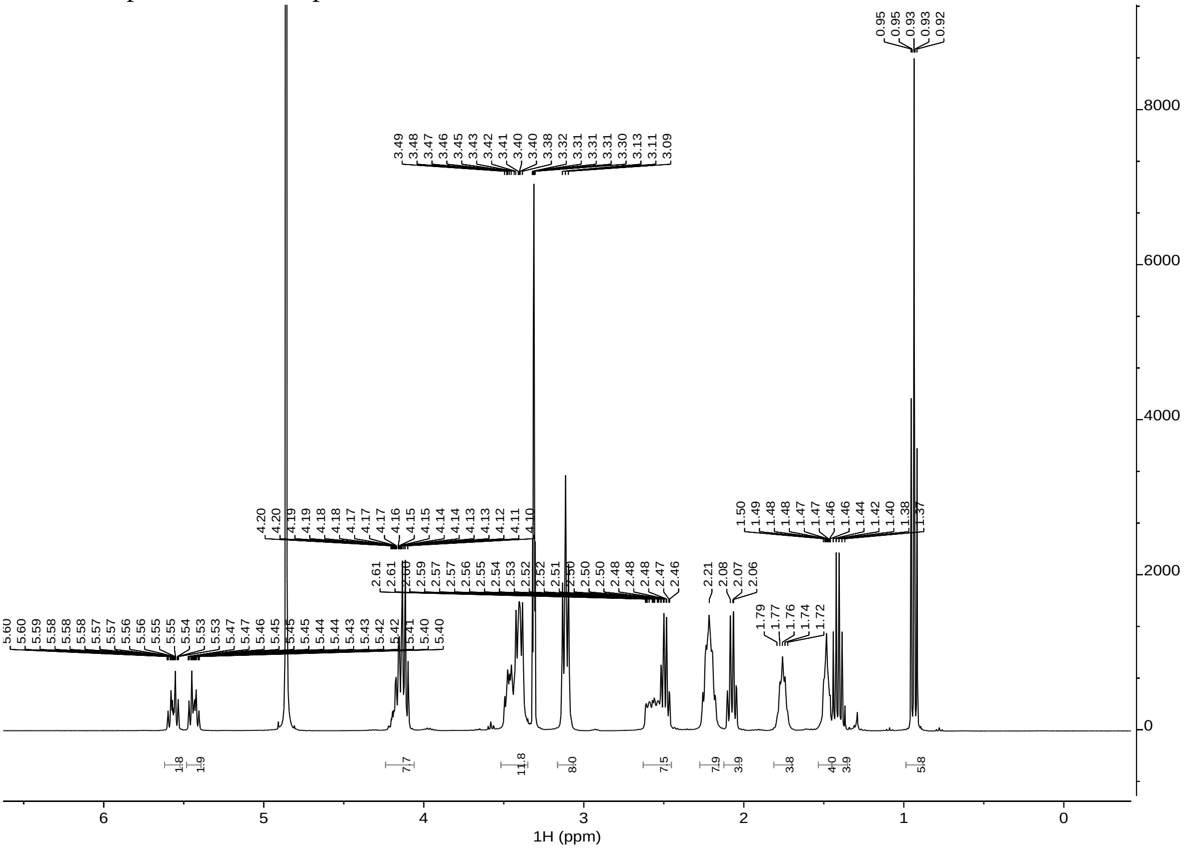

^{13}C NMR spectrum of compound **35**

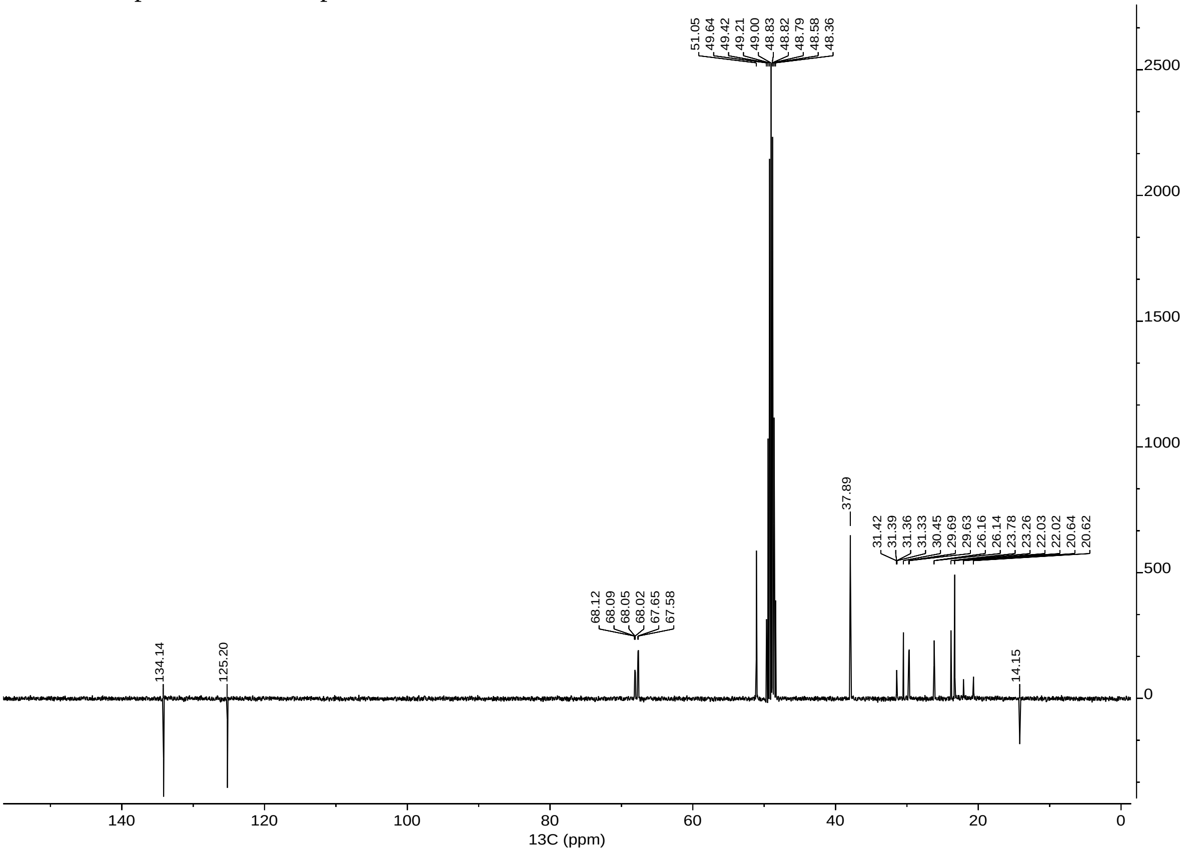

^{31}P{^{1}H} NMR spectrum of compound **35**

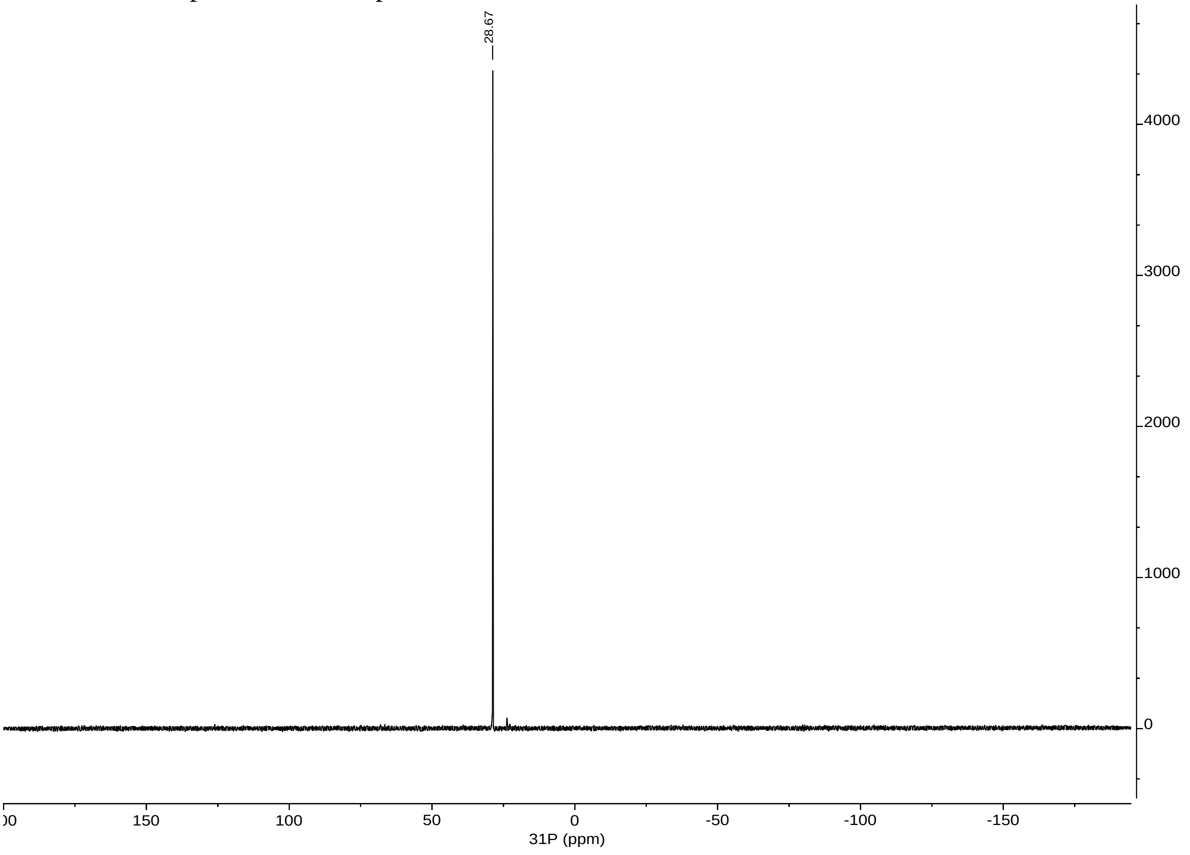

LC-MS chromatograms for compound **35**

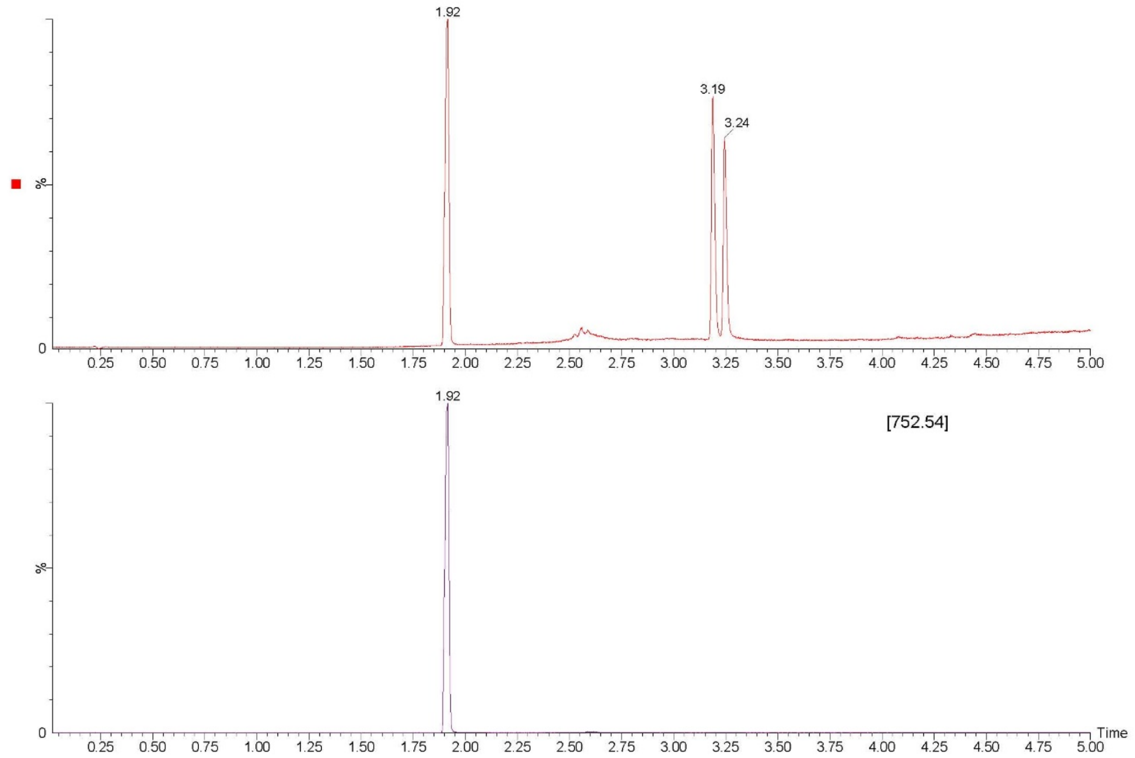

Bis((*Z*)-hept-3-en-1-yl) hexane-1,6-diyl bis((2-(bis(3-guanidinopropyl)amino)ethyl)phosphonate) hexahydrochloride (36)

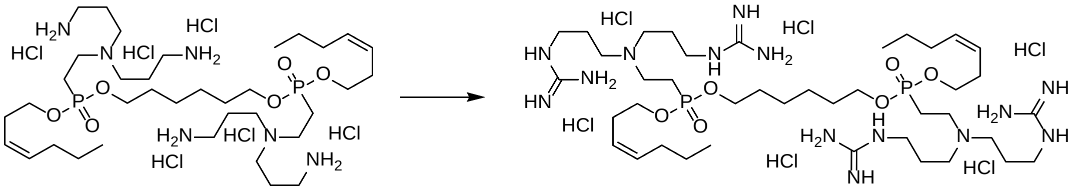

The title compound was prepared according to general method **F** from LPPO **35** (0.30 g, 0.33 mmol) in 65% yield (0.23 g, 0.22 mmol) as a white solid.

Mixture of diastereoisomers.

^{1}H NMR (401 MHz, CD_3OD) δ 5.61–5.52 (m, 2H, C*H*(CH_2)$_2$$CH_3$), 5.48–5.39 (m, 2H, O($CH_2$)$_2$C*H*), 4.18–4.08 (m, 8H, OC*H*$_2$), 3.48–3.40 (m, 4H, PCH_2C*H*$_2$), 3.40–3.31 (m, 16H, C*H*$_2$$CH_2$C*H*$_2$NH), 2.60–2.45 (m, 8H, PC*H*$_2$, OCH_2C*H*$_2$CH), 2.16–2.04 (m, 12H, $CH_3$$CH_2$C*H*$_2$, C*H*$_2$$CH_2$NH), 1.75 (p, J = 7.1, 6.4 Hz, 4H, OCH_2C*H*$_2$$CH_2$), 1.52–1.45 (m, 4H, O$CH_2$$CH_2$C*H*$_2$), 1.41 (q, J = 7.4 Hz, 4H, CH_3C*H*$_2$), 0.93 (t, J = 7.4 Hz, 6H, C*H*$_3$).

^{13}C NMR (101 MHz, CD_3OD) δ 158.70 (*C*=NH), 134.18 (*C*H(CH_2)$_2$$CH_3$), 125.27 (O($CH_2$)$_2$*C*H), 68.08 (d, J = 6.5 Hz), 68.05 (d, J = 6.7 Hz, O*C*H_2(CH_2)$_2$), 67.60 (d, J = 6.7 Hz, O*C*$H_2$$CH_2$CH), 51.59 (*C*$H_2$NH), 48.64 (P$CH_2$*C*$H_2$), 39.69 (*C*$H_2$($CH_2$)$_2$NH, 31.44 (d, J = 5.9 Hz), 31.41 (d, J = 6.0 Hz, OCH_2*C*$H_2$$CH_2$), 30.46 (*C*$H_2$$CH_2$$CH_3$), 29.65 (d, J = 6.0 Hz, OCH_2*C*H_2CH), 26.18 (d, J = 1.9 Hz, O(CH_2)$_2$*C*H_2), 24.80 (*C*$H_2$$CH_2$NH), 23.80 (*C*$H_2$$CH_3$), 21.31 (d, J = 140.4 Hz, P*C*H_2), 14.16 (*C*H_3).

^{31}P{^{1}H} NMR (162 MHz, CD_3OD) δ 28.95, 28.92.

IR ν_{max} 3400–3100 (s, br), 3008 (m), 2959 (m), 2925 (m), 2870 (m), 1670 (s), 1646 (s), 1625 (s, sh), 1465 (m), 1376 (m), 1224 (m), 1003 (m), 720 (w).

HR-MS(ESI$^+$): For $C_{40}H_{88}O_6N_{14}P_2$ (M+2H)$^{2+}$ *m*/2*z* calculated 461.32378, found 461.32363.

^{1}H NMR spectrum of compound **36**

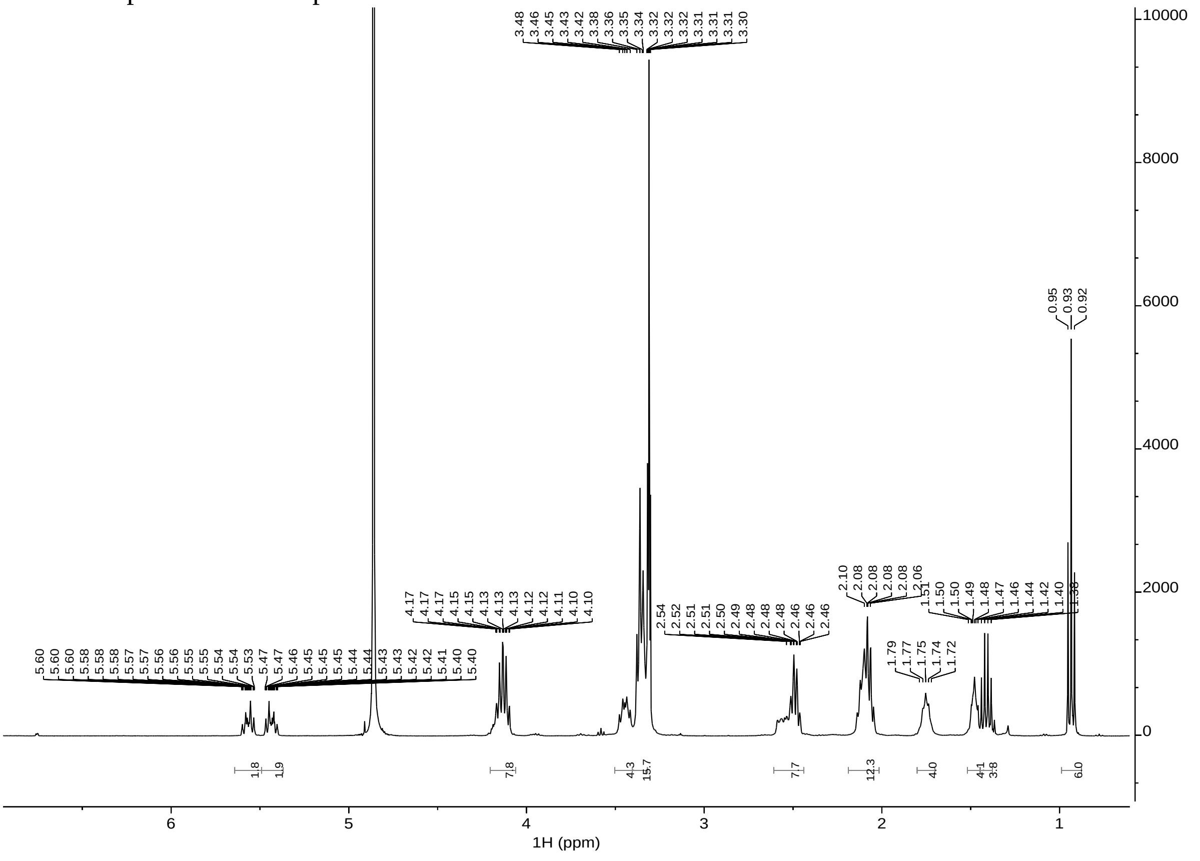

^{13}C NMR spectrum of compound **36**

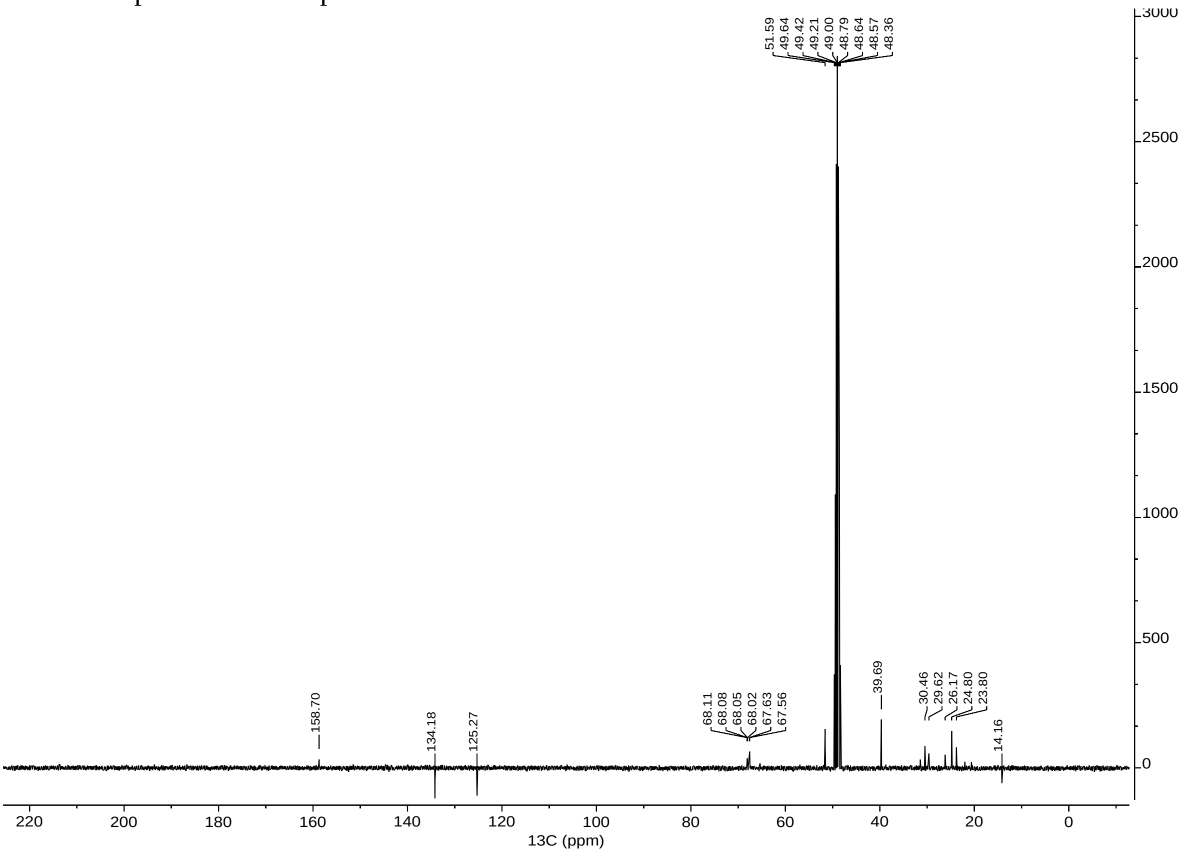

^{31}P{^{1}H} NMR spectrum of compound **36**

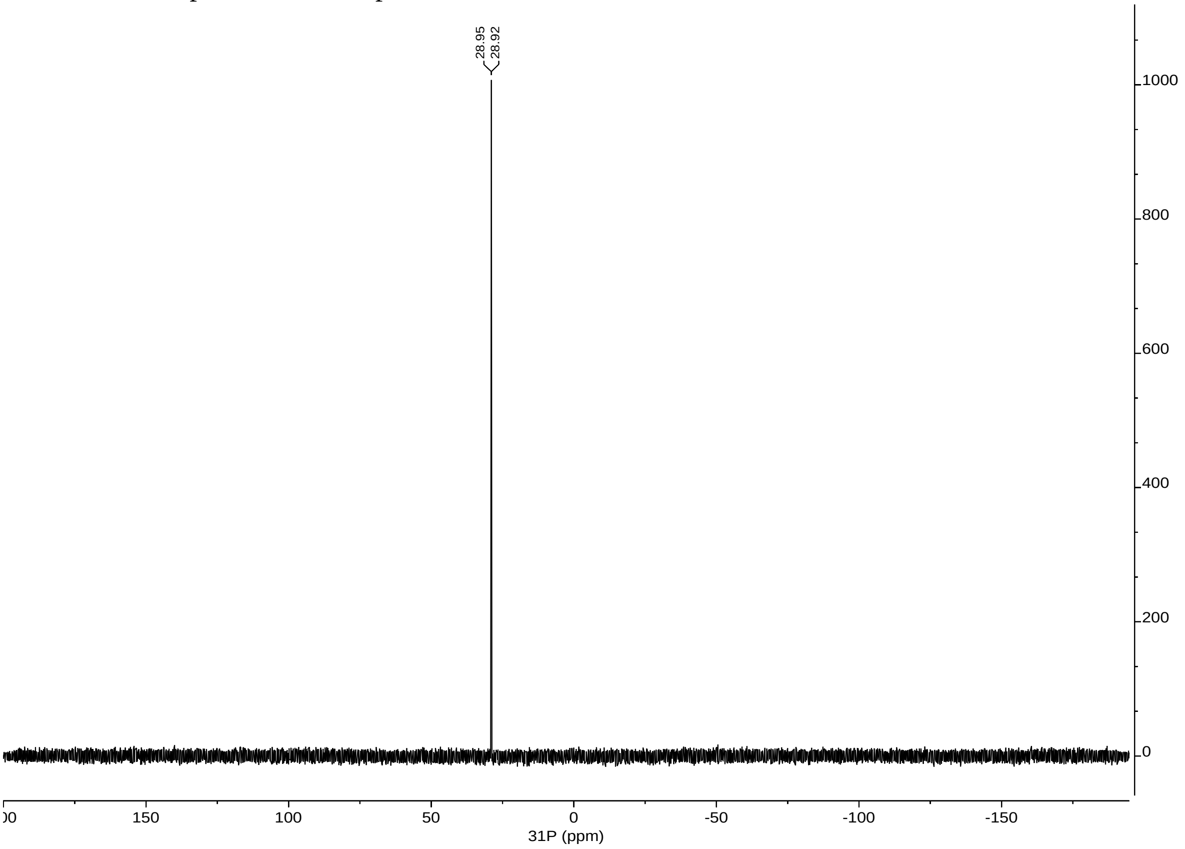

LC-MS chromatograms for compound **36**

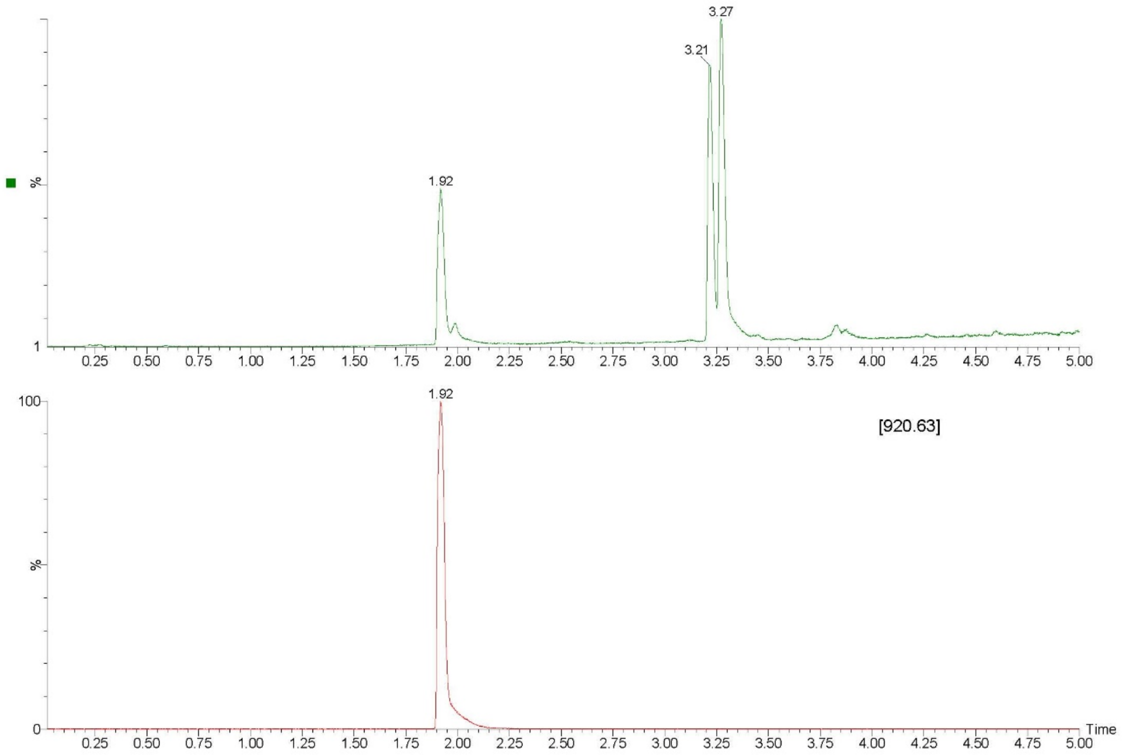

Bis((*Z*)-hept-4-en-1-yl) hexane-1,6-diyl bis((2-(bis(3-aminopropyl)amino)ethyl)phosphonate) hexahydrochloride (37)

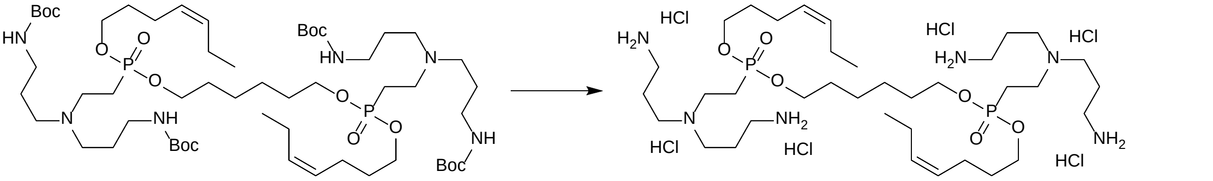

The title compound was prepared according to general method **E** from Boc derivative **S103** (0.54 g, 0.47 mmol) in 97% yield (0.44 g, 0.46 mmol) as a white solid.

Mixture of diastereoisomers.

^{1}H NMR (401 MHz, CD_3OD): 5.49–5.40 (m, 2H, CH_3CH_2CH), 5.39–5.31 (m, 2H, $CH(CH_2)_3O$), 4.22–4.08 (m, 8H, CH_2O), 3.52–3.35 (m, 12H, CH_2NH_2, PCH_2CH_2), 3.11 (t, 8H, J = 7.5 Hz, $CH_2(CH_2)_2NH$), 2.64–2.47 (m, 4H, PCH_2), 2.29–2.13 (m, 12H, $CH_2CH_2NH_2$, $CHCH_2(CH_2)_2O$), 2.13–2.01 (m, 4H, CH_3CH_2), 1.84–1.70 (m, 8H, CH_2CH_2O), 1.52–1.45 (m, 4H, $O(CH_2)_2(CH_2)_2(CH_2)_2O$), 0.98 (t, 6H, J = 7.5 Hz, CH_3).

^{13}C NMR (101 MHz, CD_3OD): 133.87 (CH_3CH_2CH), 128.47 ($CH(CH_2)_3O$), 68.11 (d, J = 6.8 Hz), 68.09 (d, J = 6.9 Hz, $OCH_2(CH_2)_4CH_2O$), 67.68 (d, J = 6.7 Hz, $CH(CH_2)_2CH_2O$), 51.06 ($CH_2(CH_2)_2NH_2$), 48.84 (PCH_2CH_2), 37.89 (CH_2NH_2), 31.67 (d, J = 6.0 Hz, $CHCH_2CH_2CH_2O$), 31.43 (d, J = 6.2 Hz), 31.40 (d, J = 6.1 Hz, $OCH_2CH_2(CH_2)_2CH_2CH_2O$), 26.18, 26.16 ($O(CH_2)_2(CH_2)_2(CH_2)_2O$), 24.03 ($CHCH_2(CH_2)_2O$), 23.29 ($CH_2CH_2NH_2$), 21.50 ($CH_3CH_2$), 21.31 (d, J = 140.4 Hz), 21.30 (d, J = 140.1 Hz, PCH_2), 14.73 (CH_3).

^{31}P{^{1}H} NMR (162 MHz, CD_3OD): 28.75.

IR ν_{max} 3010 (s, sh), 2961 (vs), 2935 (s), 2874 (s), 2034 (w), 1653 (w), 1404 (m), 1380 (w, sh), 1280 (sh), 1228 (m), 1066 (m), 1001 (s), 841 (w), 755 (w).

HR-MS(ESI$^+$): For $C_{36}H_{80}O_6N_6P_2$ $(M+2H)^{2+}$ $m/2z$ calculated 377.28018, found 377.28046.

^{1}H NMR spectrum of compound **37**

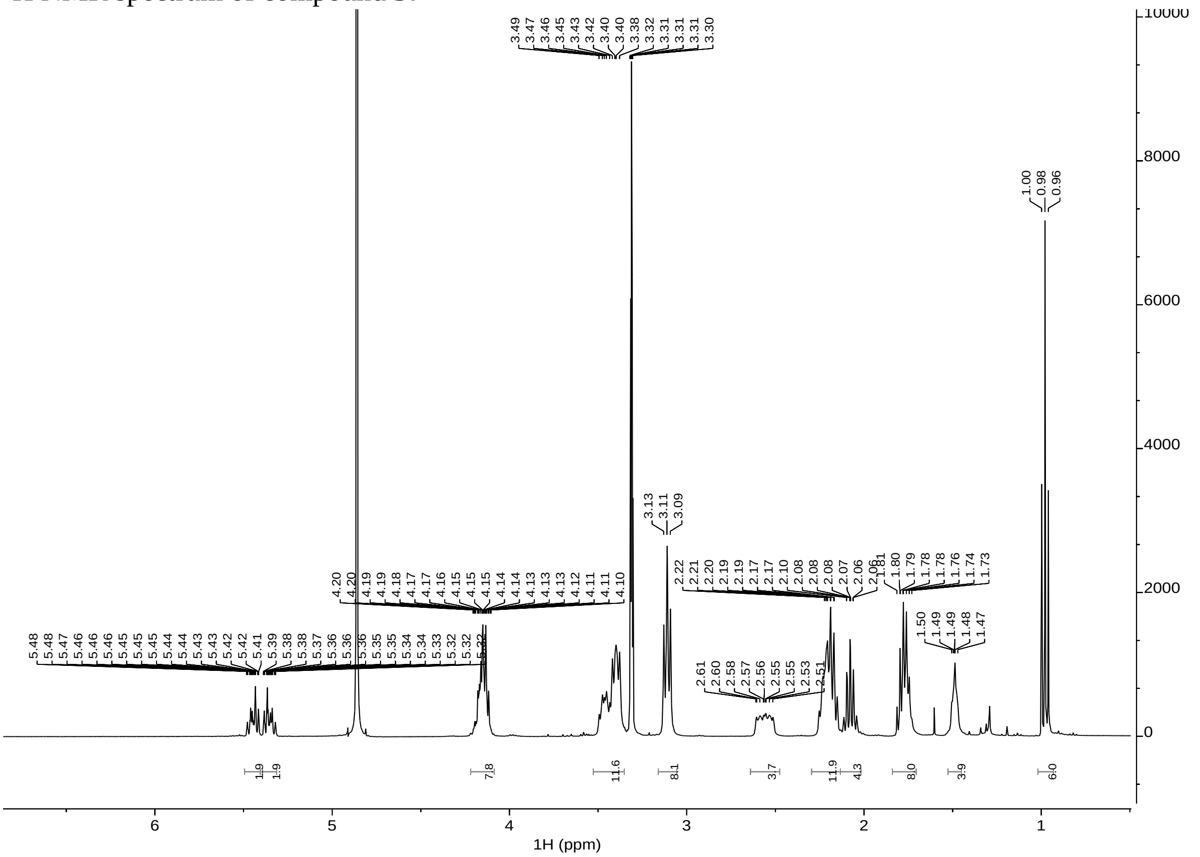

^{13}C NMR spectrum of compound **37**

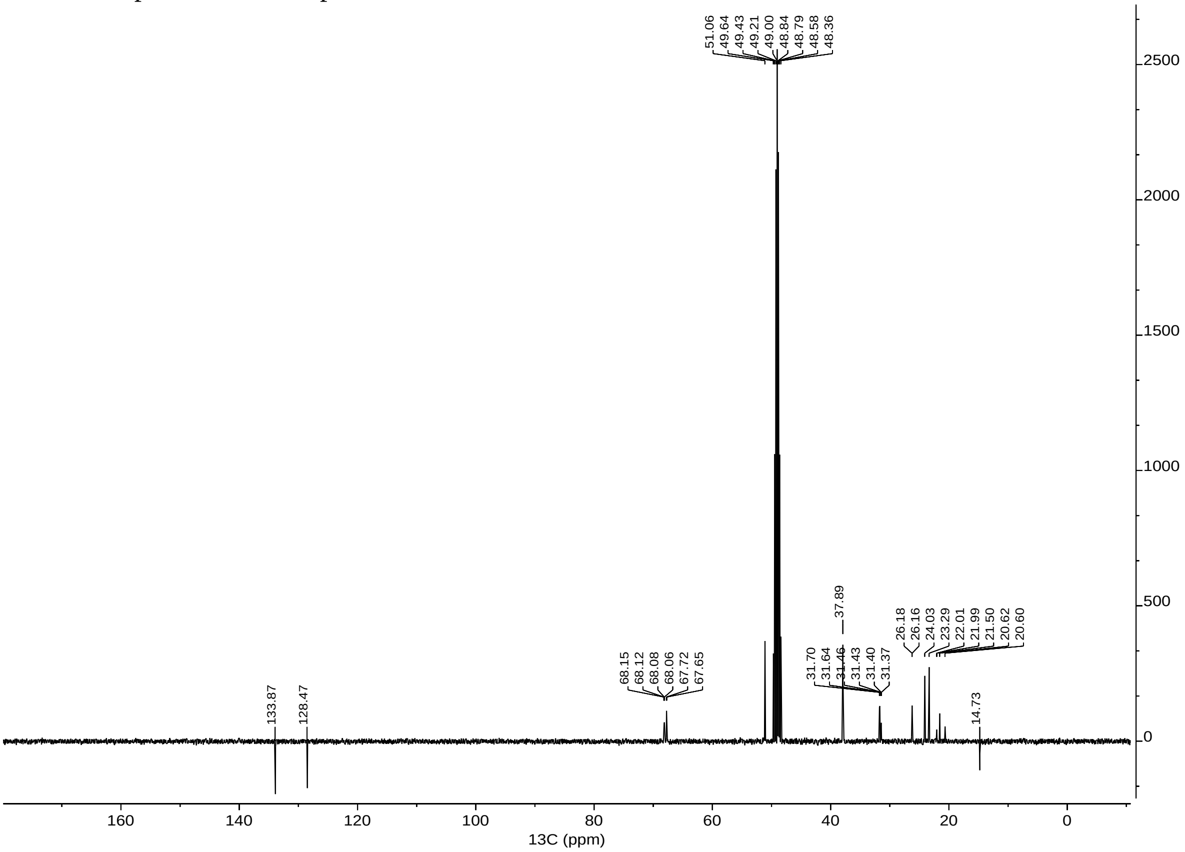

^{31}P{^{1}H} NMR spectrum of compound **37**

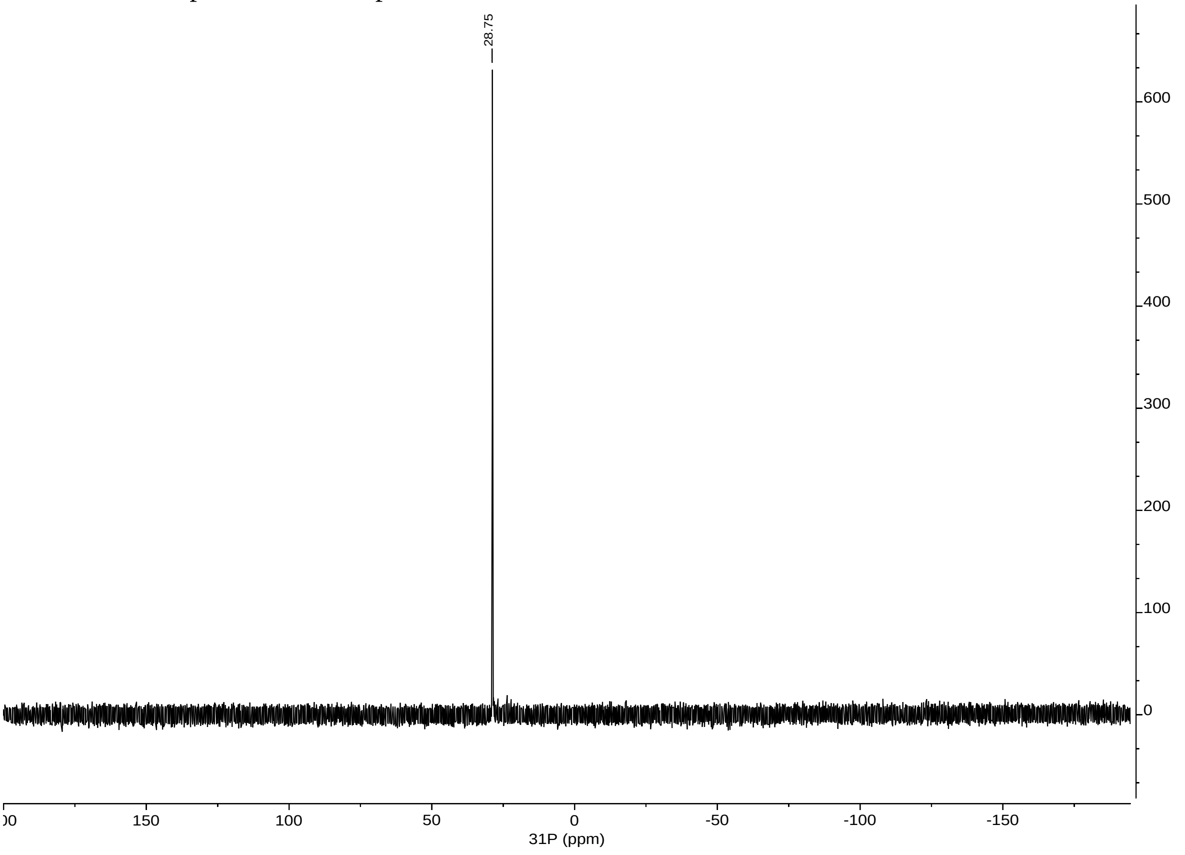

LC-MS chromatograms for compound **37**

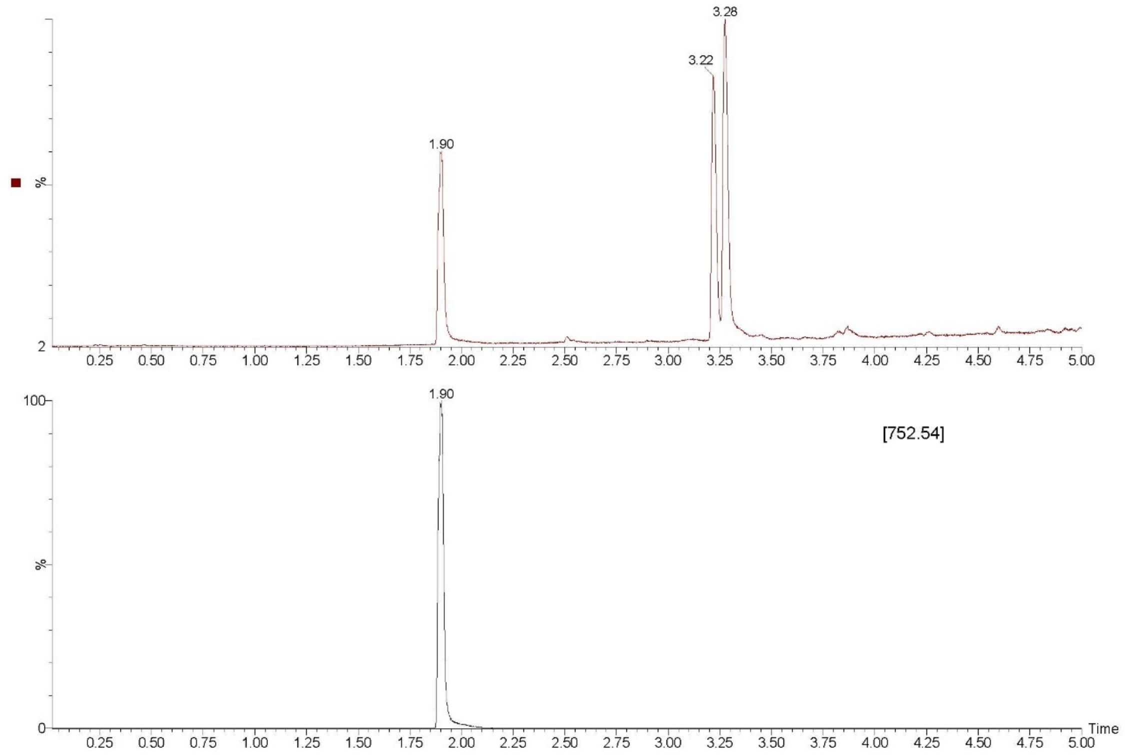

Hexane-1,6-diyl dioctyl bis((2-(bis(3-aminopropyl)amino)ethyl)phosphonate) hexahydrochloride (38)

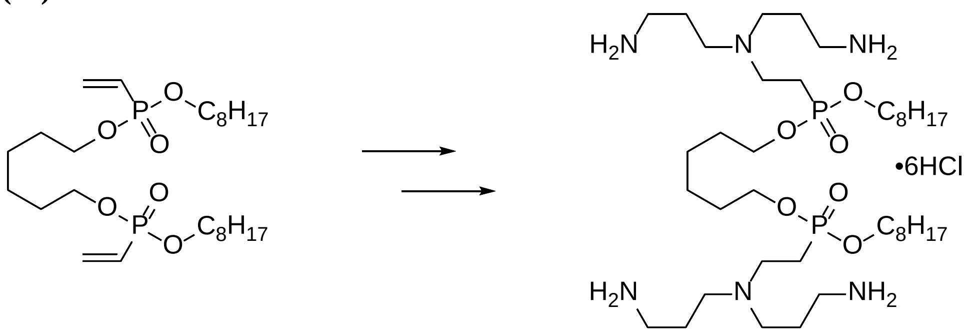

The title compound was prepared according to general methods **D** and **E** from vinylphosphonate dimer **S57** (1.14 g, 2.17 mmol) in 66% overall yield (1.44 g, 1.44 mmol) as a white solid.
Mixture of diastereoisomers.
^{1}H NMR (500.0 MHz, CD_3OD): 4.23–4.07 (m, 8H, C*H*$_2$O), 3.51–3.42 (m, 4H, PCH$_2$C*H*$_2$), 3.45–3.36 (m, 8H, C*H*$_2$(CH$_2$)$_2$NH$_2$), 3.11 (t, 8H, *J* = 7.5 Hz, C*H*$_2$NH$_2$), 2.62–2.52 (m, 4H, PC*H*$_2$), 2.27–2.17 (m, 8H, C*H*$_2$CH$_2$NH$_2$), 1.79–1.67 (m, 8H, C*H*$_2$CH$_2$O), 1.52–1.26 (m, 24H, CH$_3$(C*H*$_2$)$_4$, C*H*$_2$(CH$_2$)$_2$O), 0.93–0.88 (m, 6H, C*H*$_3$).
^{13}C NMR (125.7 MHz, CD_3OD): 68.22, 68.03, 68.01 (d, *J* = 6.6 Hz, *C*H$_2$O), 50.98 (*C*H$_2$(CH$_2$)$_2$NH$_2$), 48.83 (PCH$_2$*C*H$_2$), 37.86 (*C*H$_2$NH$_2$), 32.99 (CH$_3$CH$_2$*C*H$_2$), 31.60(d, *J* = 5.9 Hz), 31.39(d, *J* = 5.9 Hz), 31.36 (d, *J* = 6.0 Hz, *C*H$_2$CH$_2$O), 30.37, 30.28, 26.63, 26.16, 23.73 (CH$_3$(*C*H$_2$)$_4$, *C*H$_2$(CH$_2$)$_2$O), 23.21 (*C*H$_2$CH$_2$NH$_2$), 21.31(d, *J* = 140.2 Hz), 21.28 (d, *J* = 140.3 Hz, P*C*H$_2$), 14.47 (*C*H$_3$).
^{31}P{^{1}H} NMR (202.4 MHz, CD_3OD): 27.32.
IR ν_{max} (KBr) 2956 (vs), 2926 (vs), 2856 (s), 2748 (m, br, sh), 2644 (m, br), 2559 (m, br), 2055 (w, br), 1626 (s, br), 1513 (m, br), 1468 (s), 1398 (m, br), 1385 (vw), 1257 (m, sh), 1230 (s), 1071 (s, br, sh), 1001 (s, br), 724 (w).
HR-MS(ESI$^+$): For $C_{38}H_{88}N_6O_6P_2$ $(M+2H)^{2+}$ *m*/2*z* calculated 393.31148, found 393.31156.

^{1}H NMR spectrum of compound **38**

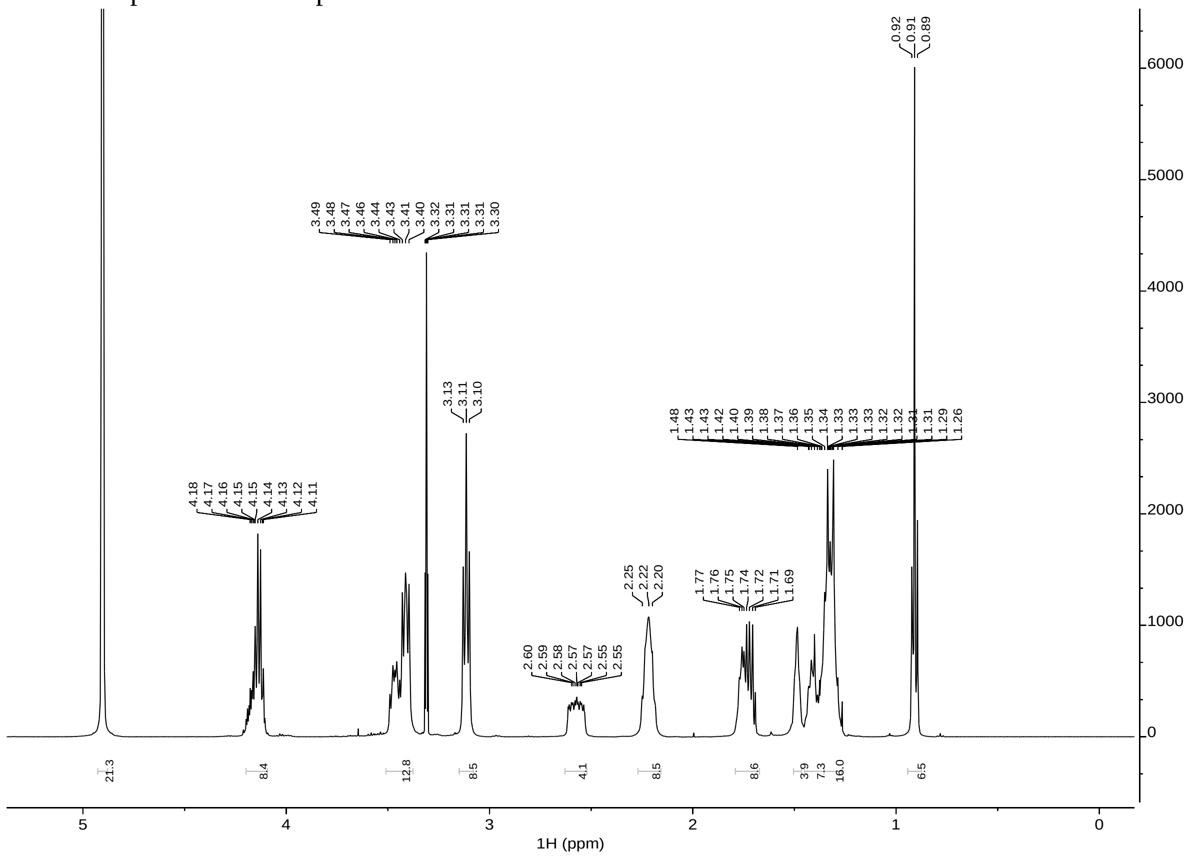

^{13}C NMR spectrum of compound **38**

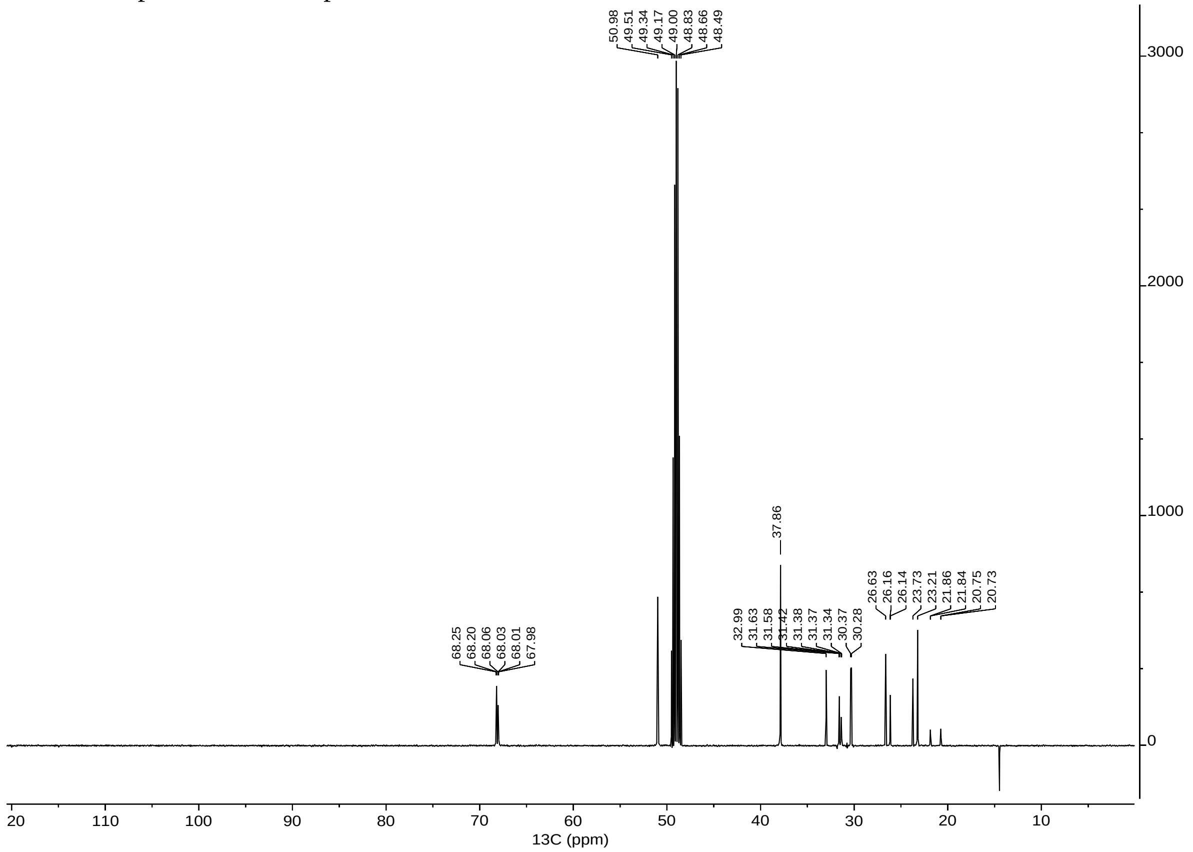

$^{31}P\{^1H\}$ NMR spectrum of compound **38**

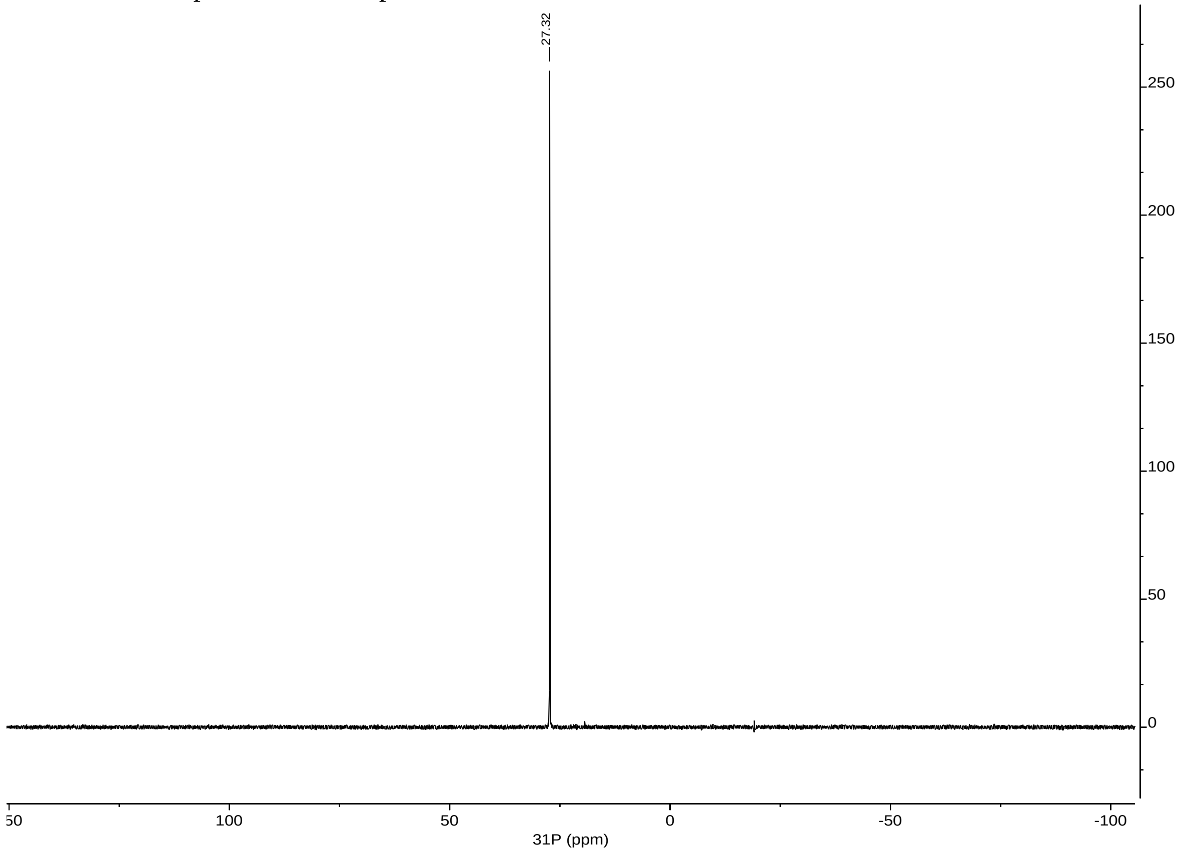

LC-MS chromatograms for compound **38**

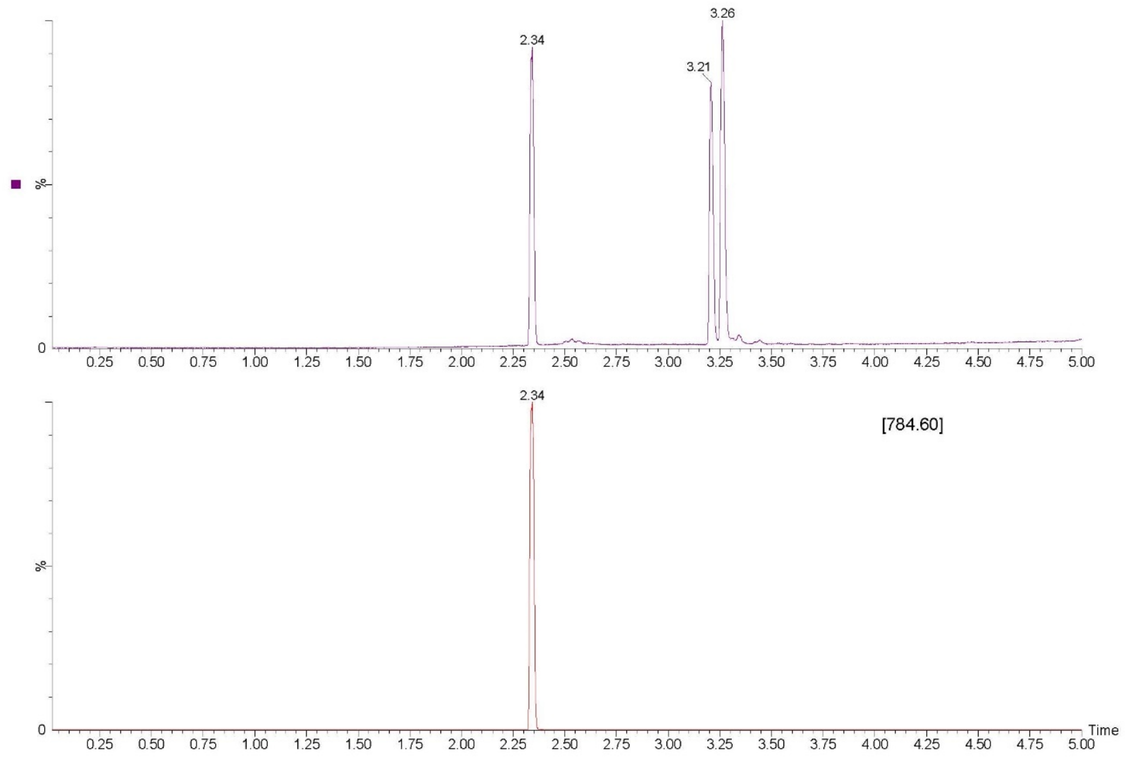

Hexane-1,6-diyl dioctyl bis((2-(bis(2-aminoethyl)amino)ethyl)phosphonate) hexahydrochloride (39)

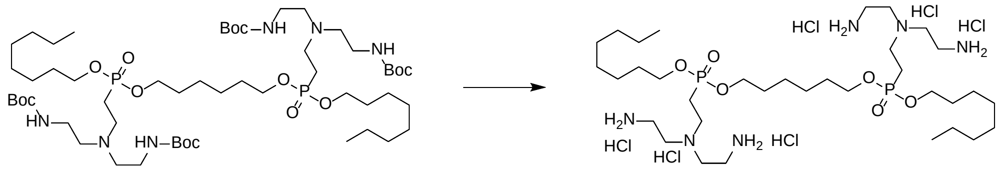

The title compound was prepared according to general method **E** from Boc derivative **S104** (0.61 g, 0.54 mmol) in 96% yield (0.47 g, 0.52 mmol) as a white solid.

Mixture of diastereoisomers.

^{1}H NMR (401 MHz, CD_3OD): 4.20–4.07 (m, 8H, C*H*$_2$O), 3.44–3.31 (m, 20H, C*H*$_2$N, C*H*$_2$$NH_2$), 2.50–2.35 (m, 4H, PC*H*$_2$), 1.81–1.67 (m, 8H, C*H*$_2$$CH_2$O), 1.53–1.46 (m, 4H, O($CH_2$)$_2$(C*H*$_2$)$_2$($CH_2$)$_2$O), 1.46–1.27 (m, 20H, CH_3(C*H*$_2$)$_5$(CH_2)$_2$O), 0.95–0.87 (m, 6H, C*H*$_3$(CH_2)$_7$O).

^{13}C NMR (101 MHz, CD_3OD): 67.97 (d, *J* = 6.9 Hz, CH_3(CH_2)$_6$*C*H_2O), 67.79 (d, *J* = 6.6 Hz), 67.77 (d, *J* = 6.9 Hz, O*C*H_2(CH_2)$_4$*C*H_2O), 51.46 (*C*$H_2$$CH_2$$NH_2$), 48.50 (P$CH_2$*C*$H_2$), 36.85 (*C*$H_2$$NH_2$), 32.98 ($CH_3$$CH_2$*C*$H_2$), 31.61 (d, *J* = 6.0 Hz, CH_3(CH_2)$_5$*C*H_2), 31.42 (d, *J* = 6.2 Hz), 31.40 (d, *J* = 6.0 Hz, OCH_2*C*H_2(CH_2)$_2$*C*$H_2$$CH_2$O), 30.36, 30.28, 26.66 (CH_3(CH_2)$_2$(*C*H_2)$_3$), 26.19 (O(CH_2)$_2$(*C*H_2)$_2$(CH_2)$_2$O), 23.72 (CH_3*C*H_2), 22.23 (d, *J* = 139.3 Hz, P*C*H_2), 14.45 *C*H_3).

^{31}P{^{1}H} NMR (162 MHz, CD_3OD): 32.26.

IR ν_{max} 3300–2500 (s), 2957 (s), 2928 (vs), 2856 (s), 2051 (w), 1609 (m), 1468 (s), 1379 (w), 1231 (m), 1002 (s), 848 (w), 770 (w), 724 (w).

HR-MS(ESI^+): For $C_{34}H_{80}O_6N_6P_2$ $(M+2H)^{2+}$ *m*/2*z* calculated 365.28018, found 365.28057.

^{1}H NMR spectrum of compound **39**

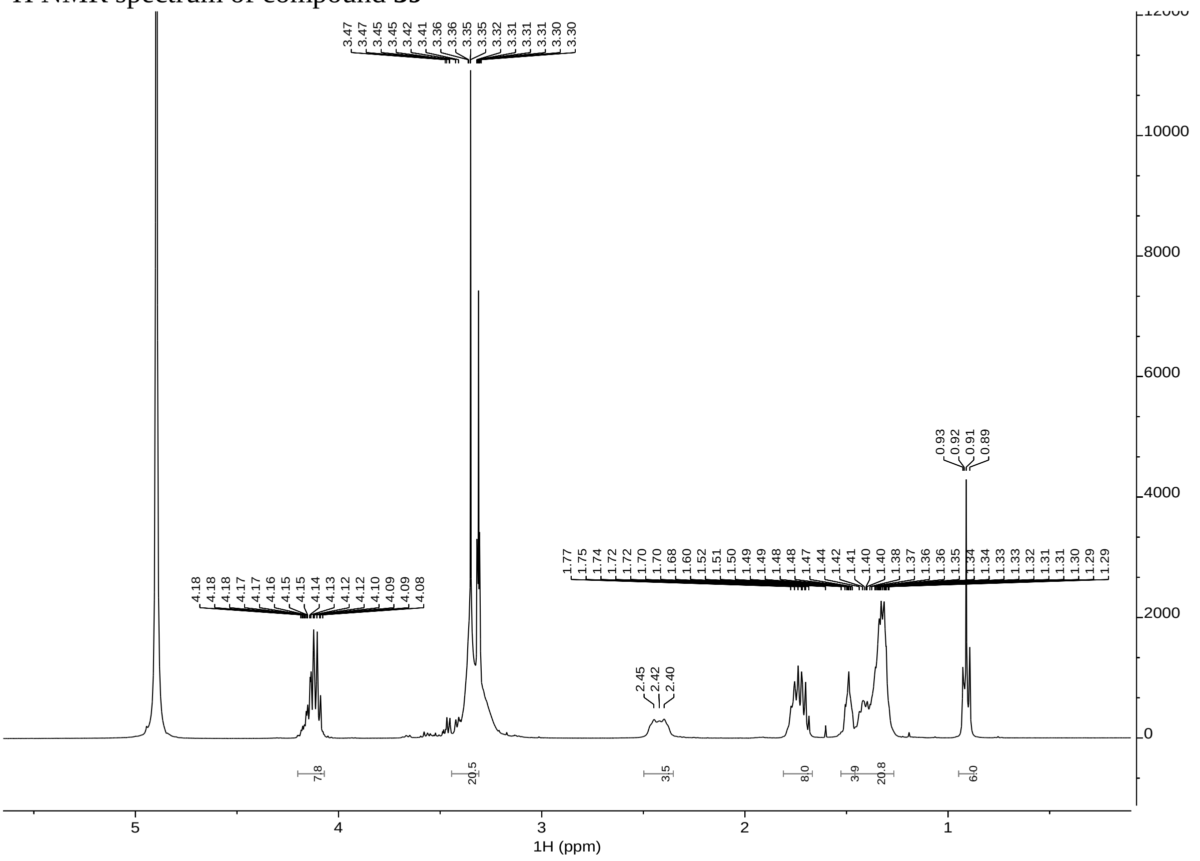

^{13}C NMR spectrum of compound **39**

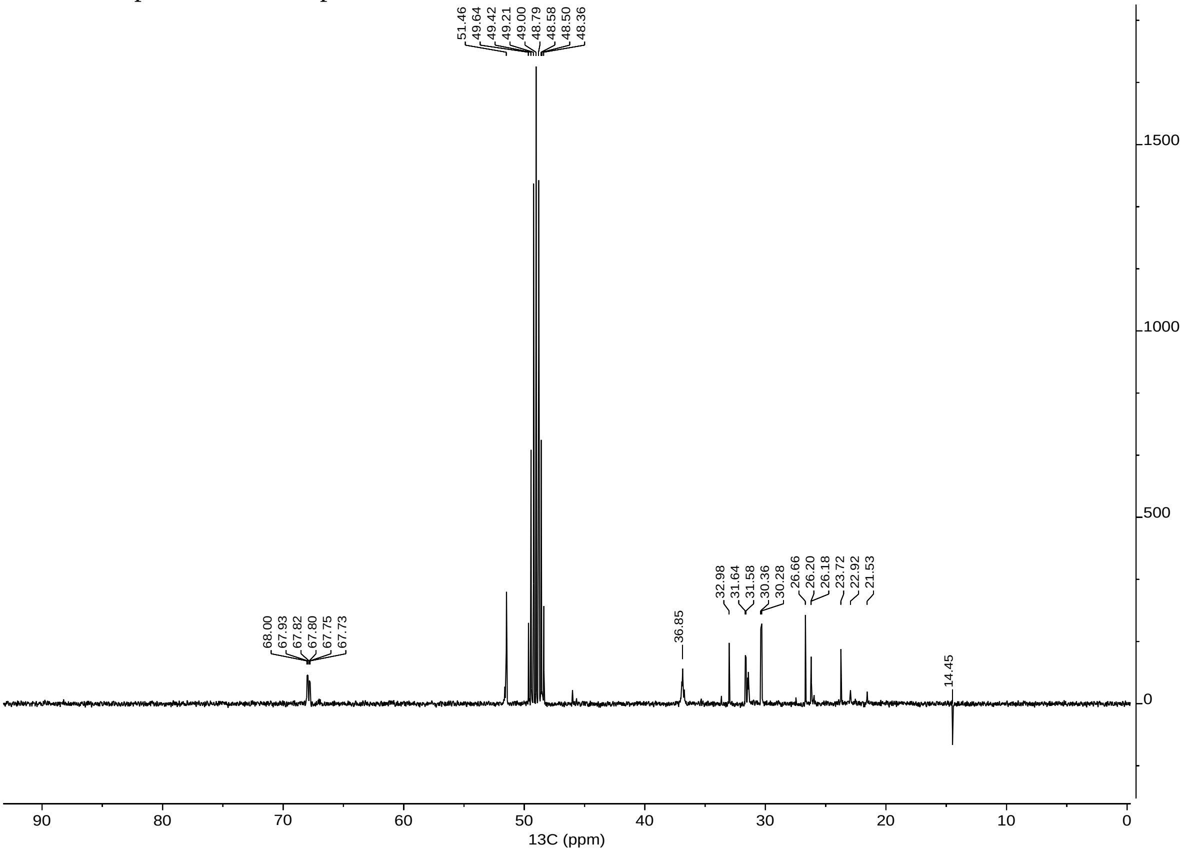

$^{31}P\{^{1}H\}$ NMR spectrum of compound **39**

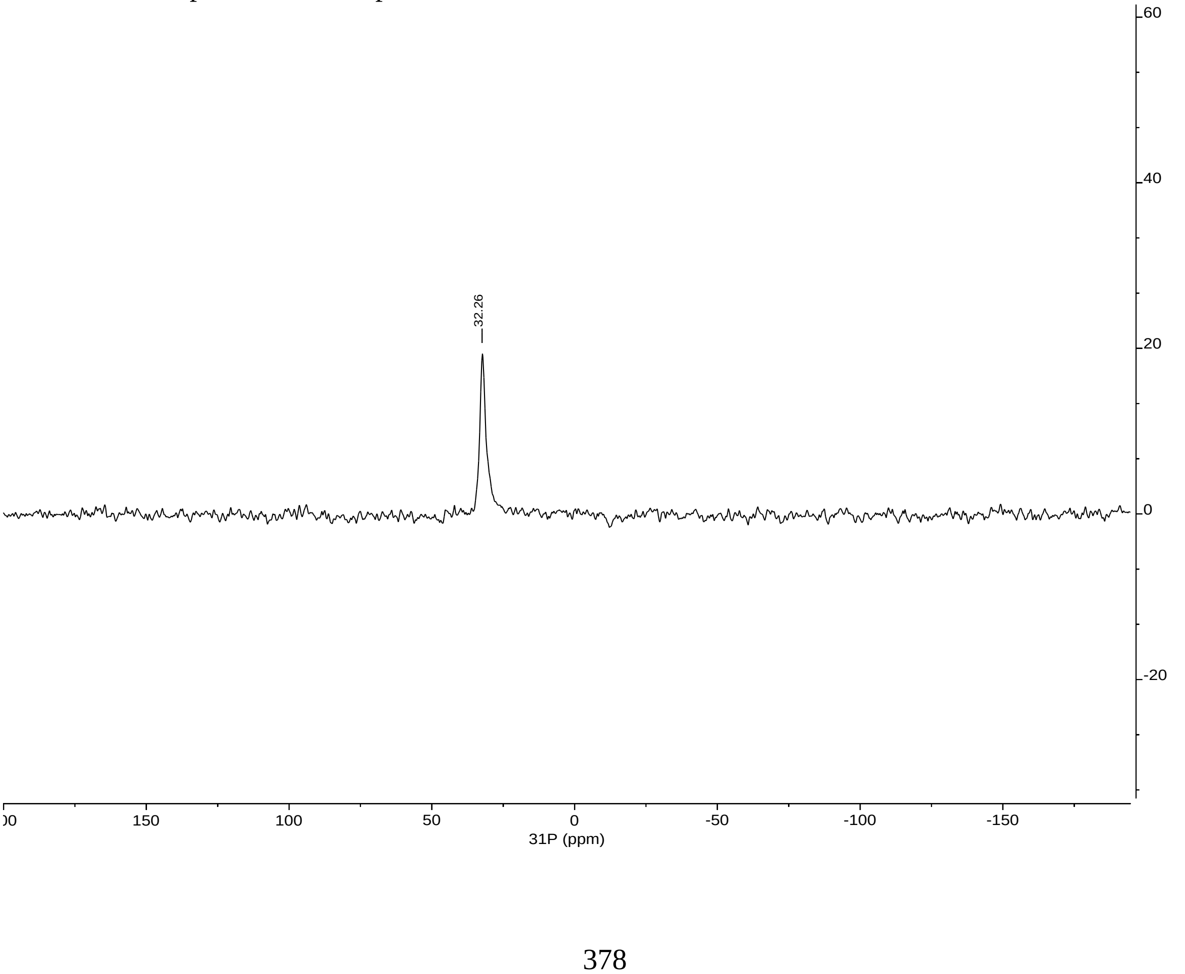

LC-MS chromatograms for compound **39**

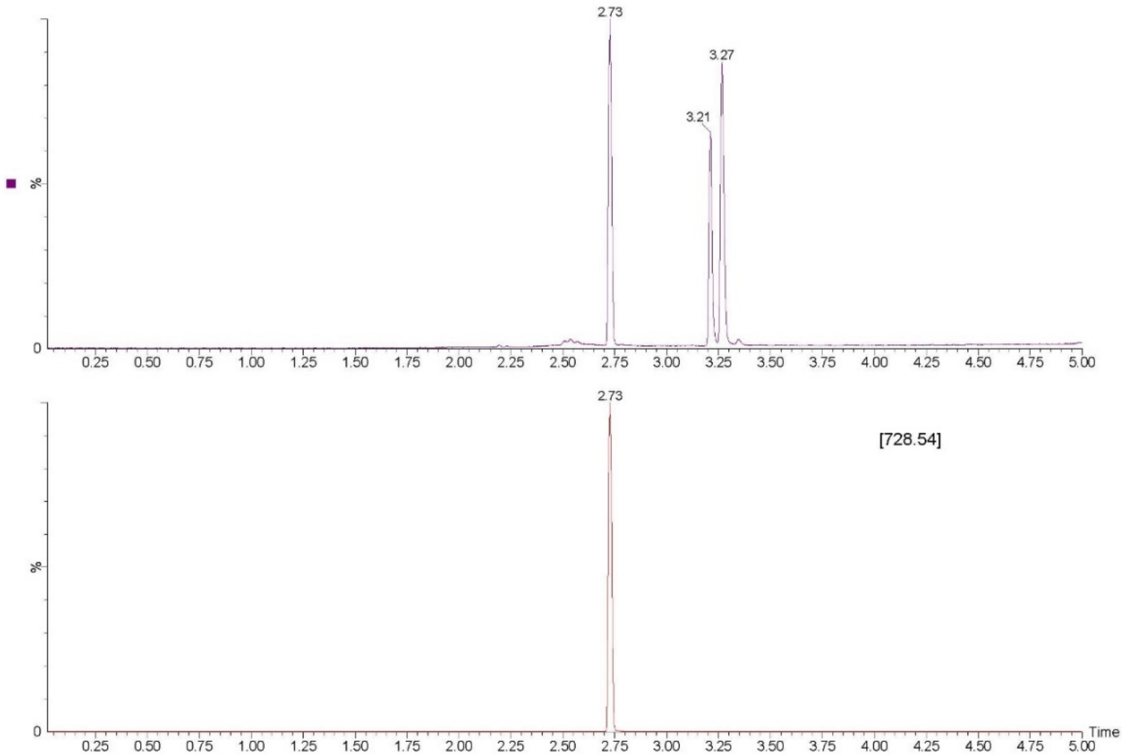

Bis(3-cyclohexylpropyl) hexane-1,6-diyl bis((2-(bis(3-aminopropyl)amino)ethyl)phosphonate) hexahydrochloride (40)

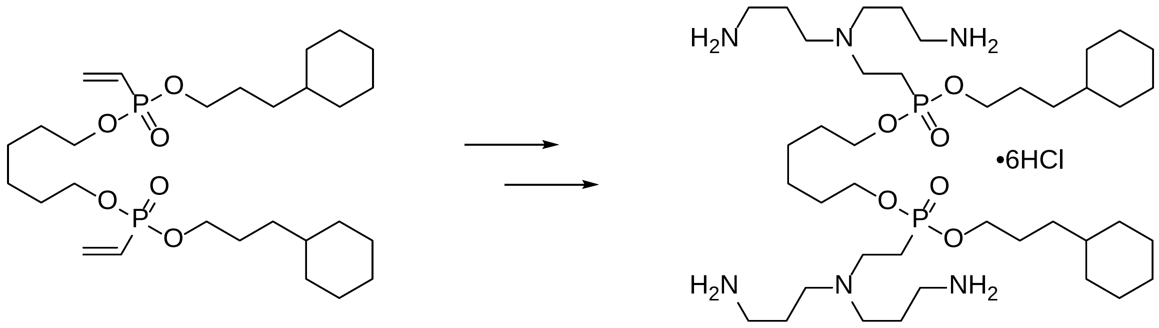

The title compound was prepared according to general methods **D** and **E** from vinylphosphonate dimer **S58** (0.82 g, 1.50 mmol) in 81% overall yield (1.25 g, 1.22 mmol) as a white solid.
Mixture of diastereoisomers.
^{1}H NMR (500.2 MHz, CD_3OD): 4.21–4.08 (m, 8H, OC*H*$_2$), 3.50–3.44 (m, 4H, PCH$_2$C*H*$_2$), 3.44–3.37 (m, 8H, C*H*$_2$(CH$_2$)$_2$NH$_2$), 3.12 (t, 8H, *J* = 7.4 Hz, C*H*$_2$NH$_2$), 2.61–2.50 (m, 4H, PC*H*$_2$), 2.28–2.16 (m, 8H, C*H*$_2$CH$_2$NH$_2$), 1.80–1.63 (m, 18H, 2a,3a,4a,5a,6a-Cy*H*, OCH$_2$C*H*$_2$), 1.52–1.45 (m, 4H, O(CH$_2$)$_2$(C*H*$_2$)$_2$(CH$_2$)$_2$O), 1.33–1.13 (m, 12H, 1,3b,4b,5b-Cy*H*, O(CH$_2$)C*H*$_2$Cy), 0.98–0.88 (m, 4H, 2b,6b-Cy*H*).
^{13}C NMR (125.8 MHz, CD_3OD): 68.60 (d, *J* = 6.7 Hz, O*C*H$_2$(CH$_2$)Cy), 68.09, 68.06 (d, *J* = 6.6 Hz, O*C*H$_2$(CH$_2$)$_4$*C*H$_2$O), 51.06 (*C*H$_2$(CH$_2$)$_2$NH$_2$), 48.89 (PCH$_2$*C*H$_2$), 38.61 (1-*C*H$_{Cy}$), 37.88 (*C*H$_2$NH$_2$), 34.46, 34.44 (2,6-(*C*H$_2$)$_{Cy}$), 34.26 (*C*H$_2$Cy), 31.40, 31.37 (d, *J* = 5.9 Hz, OCH$_2$*C*H$_2$(CH$_2$)$_2$*C*H$_2$CH$_2$O), 29.03 (d, *J* = 5.9 Hz, *C*H$_2$CH$_2$Cy), 27.69 (4-(*C*H$_2$)$_{Cy}$), 27.42 (3,5-(*C*H$_2$)$_{Cy}$), 26.17, 26.15 (O(CH$_2$)$_2$(*C*H$_2$)$_2$(CH$_2$)$_2$O), 23.23 (*C*H$_2$CH$_2$NH$_2$), 21.33, 21.31 (d, *J* = 140.0 Hz, P*C*H$_2$).
^{31}P{^{1}H} NMR (202.5 MHz, CD_3OD): 26.92.
IR ν_{max} (KBr) 3000 (v, vbr2970 (s, sh), 2923 (vs), 2851 (s), 2744 (m, br, sh), 2640 (m, vbr), 2559 (m, vbr), 2030 (w, vbr), 1608 (w, br), 1515 (w, sh), 1505 (w, sh), 1396 (w, br), 1346 (vw), 1260 (w, br, sh), 1227 (m), 1069 (m), 1053 (m, sh), 1002 (s).
HR-MS(ESI$^+$): For $C_{40}H_{87}N_6O_6P_2$ $(M+H)^+$ *m/z* calculated 809.61568, found 809.61578.

^{1}H NMR spectrum of compound **40**

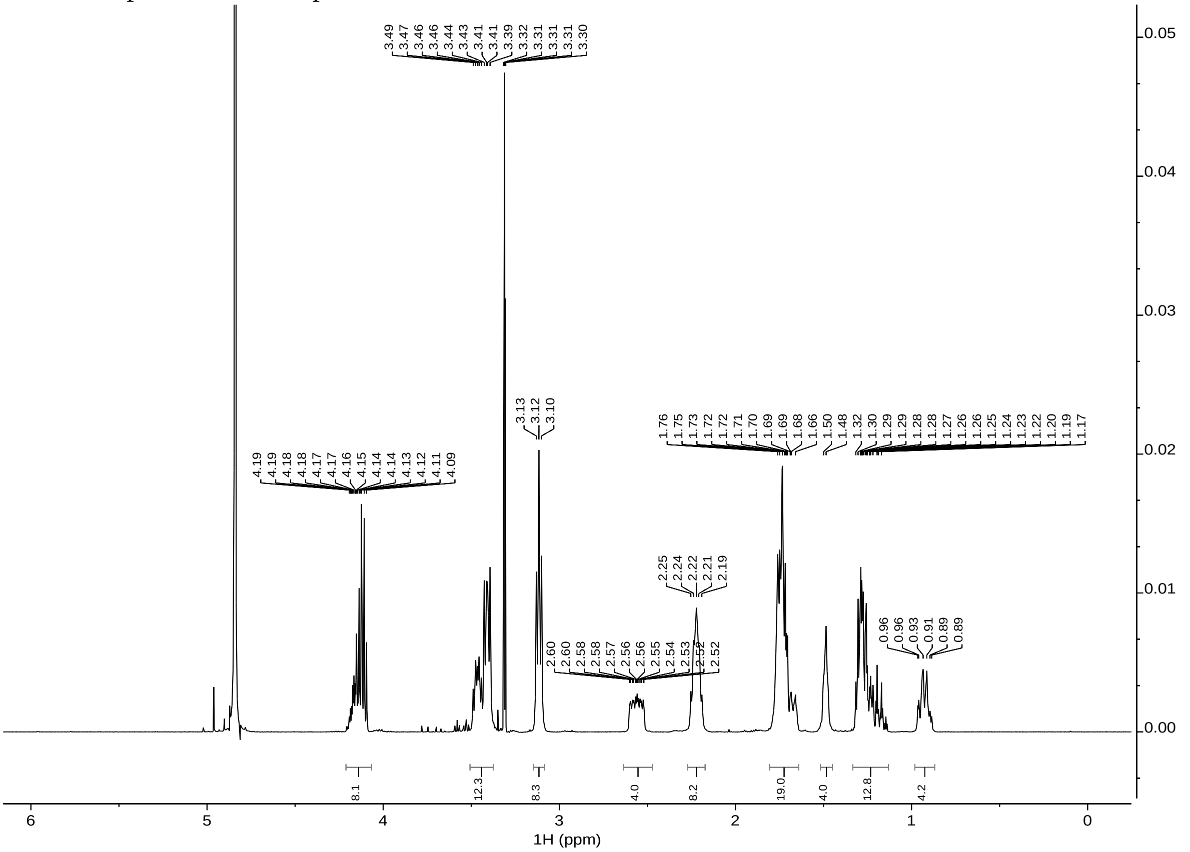

^{13}C NMR spectrum of compound **40**

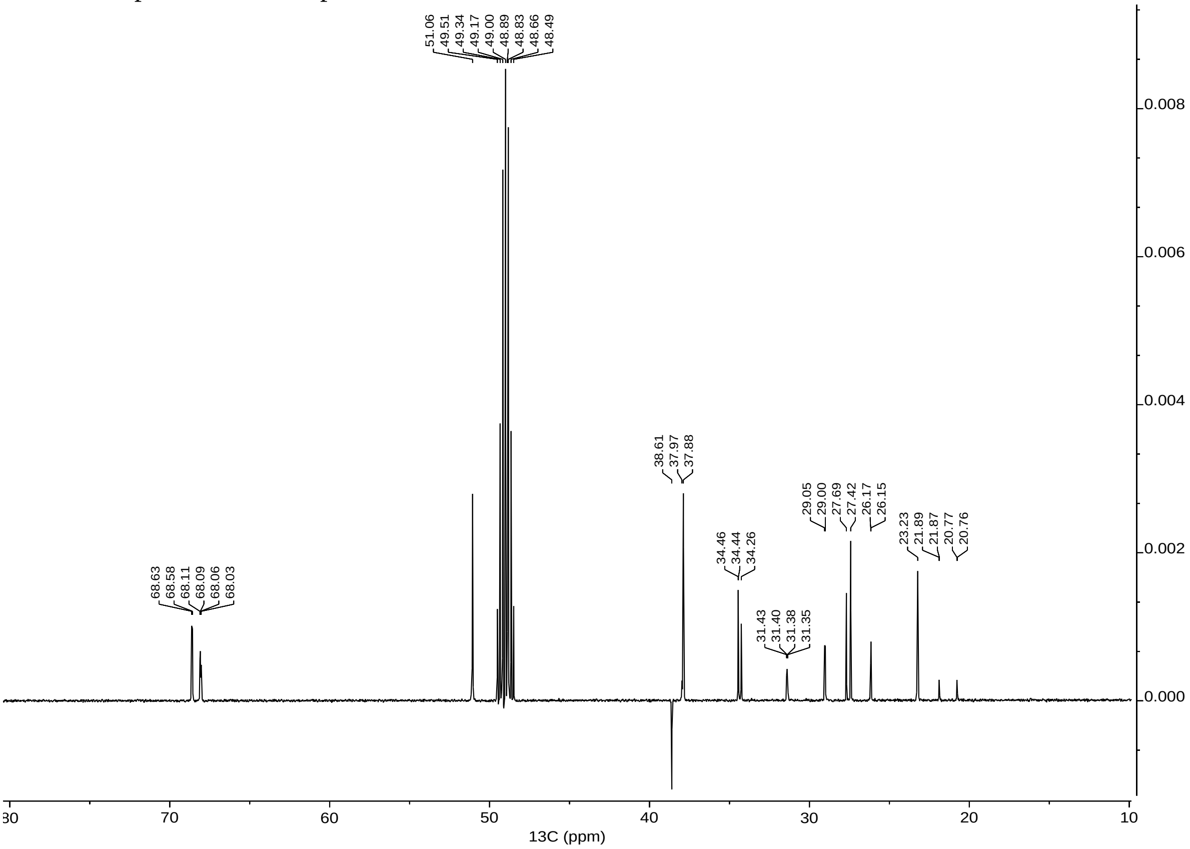

$^{31}P\{^1H\}$ NMR spectrum of compound **40**

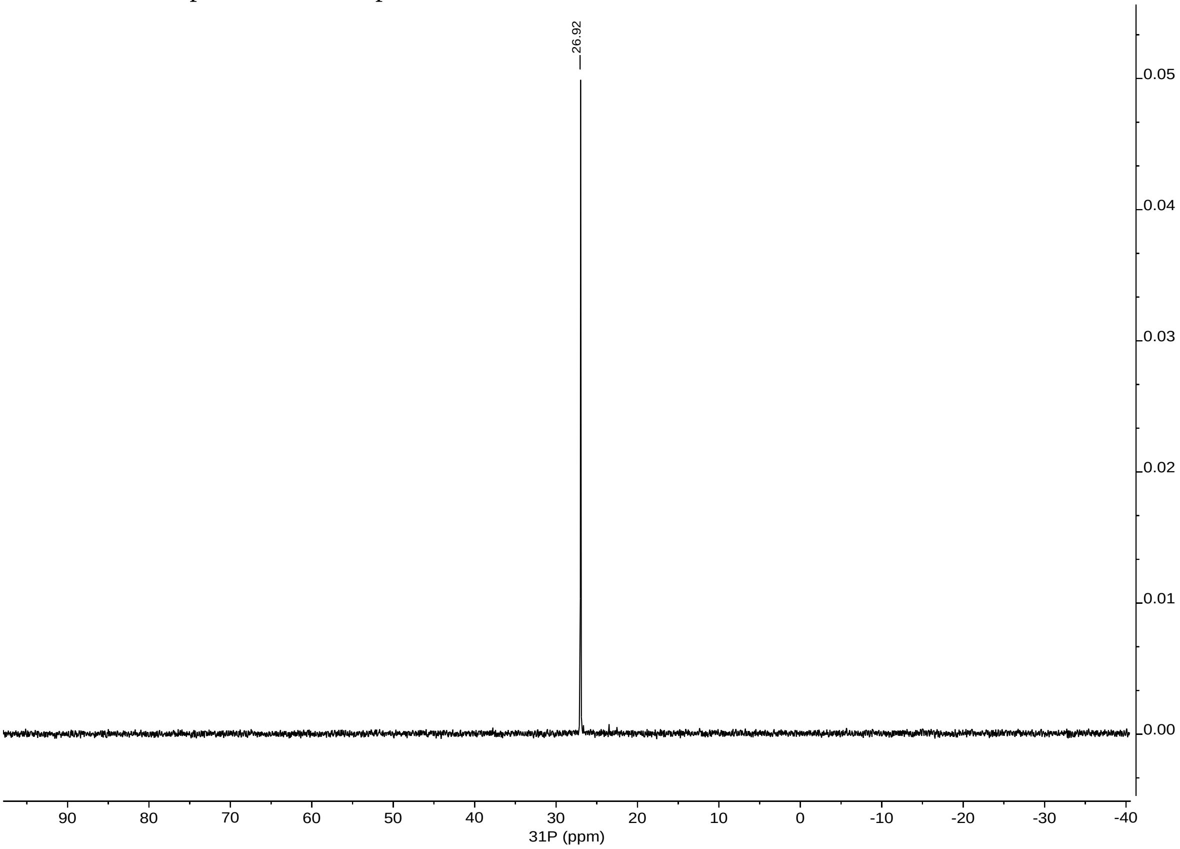

LC-MS chromatograms for compound **40**

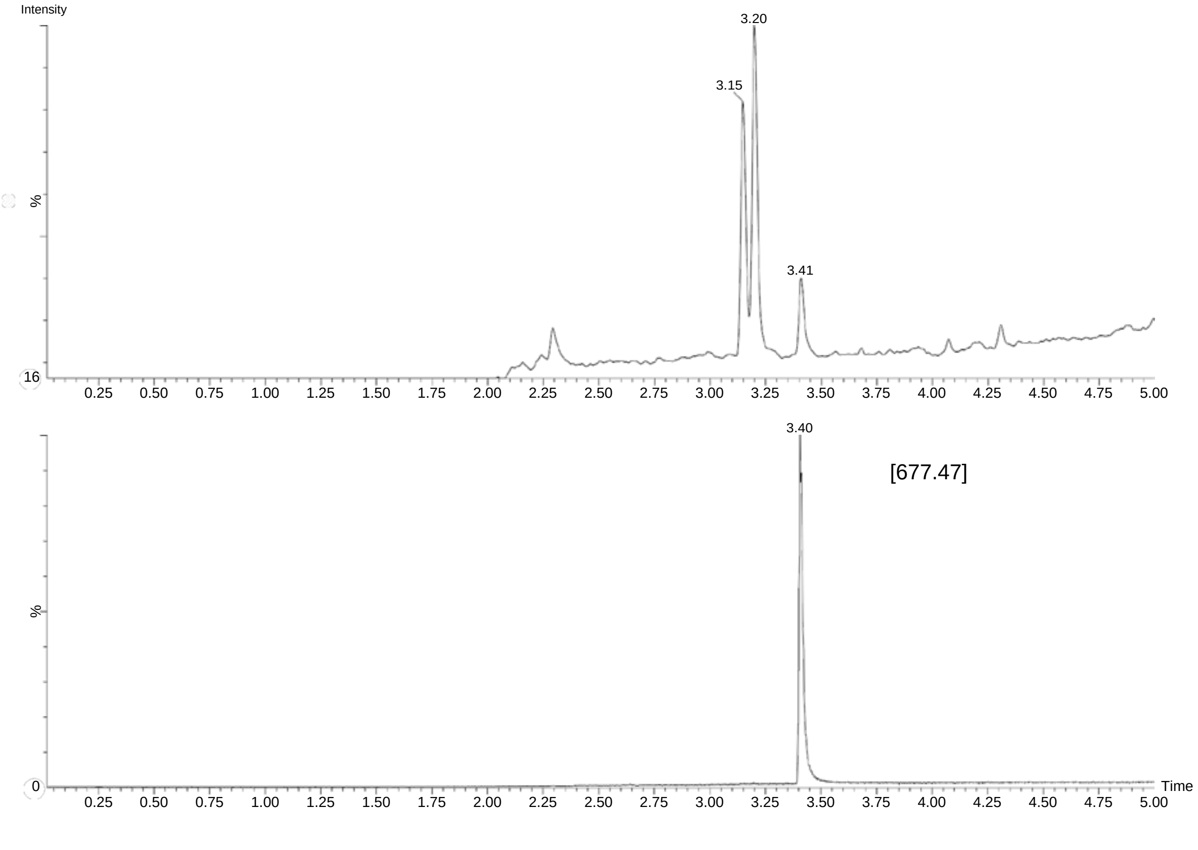

Hexane-1,6-diyl bis((*Z*)-oct-3-en-1-yl) bis((2-(bis(3-aminopropyl)amino)ethyl)phosphonate) hexahydrochloride (41)

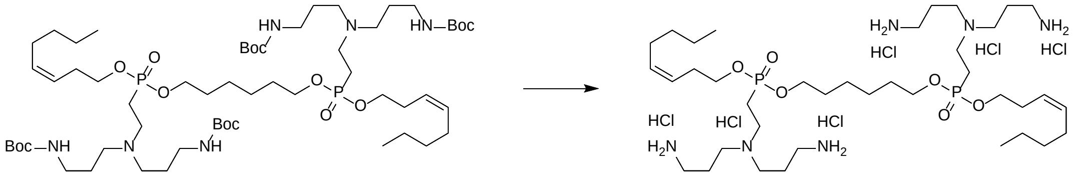

The title compound was prepared according to general method **E** from Boc derivative **S105** (0.85 g, 0.71 mmol) in 93% yield (0.66 g, 0.66 mmol) as a white solid.

Mixture of diastereoisomers.

^{1}H NMR (400 MHz, CD_3OD): 5.62–5.52 (m, 2H, $CH_3(CH_2)_3CH$), 5.48–5.35 (m, 2H, $CH(CH_2)_2O$), 4.23–4.05 (m, 8H, CH_2O), 3.52–3.33 (m, 12H, CH_2N), 3.11 (t, J = 7.5 Hz, 8H, CH_2NH_2), 2.61–2.51 (m, 4H, PCH_2), 2.51–2.44 (m, 4H, $CHCH_2CH_2O$), 2.28–2.15 (m, 8H, $CH_2CH_2NH_2$), 2.15–2.03 (m, 4H, $CH_3(CH_2)_2CH_2$), 1.81–1.70 (m, 4H, $CH_2CH_2CH_2O$), 1.54–1.44 (m, 4H, $CH_2(CH_2)_2O$), 1.42–1.29 (m, 8H, $CH_3(CH_2)_2$), 0.97–0.88 (m, 6H, CH_3).

^{13}C NMR (101 MHz, CD_3OD): 134.22 ($CH_3(CH_2)_3CH$), 125.02 ($CH(CH_2)_2O$), 68.01 (d, J = 6.6 Hz, $(CH_2)_2CH_2O$), 67.99 (d, J = 6.6 Hz, $(CH_2)_2CH_2O$), 67.56 (d, J = 6.8 Hz, $CHCH_2CH_2O$), 50.99 ($CH_2(CH_2)_2NH_2$), 48.88 (PCH_2CH_2), 37.93 (CH_2NH_2), 32.87 ($CH_3CH_2CH_2$), 31.33 (d, J = 6.0 Hz, $CH_2CH_2CH_2O$), 31.30 (d, J = 6.1 Hz, $CHCH_2CH_2O$), 29.61 (d, J = 6.0 Hz, $CHCH_2CH_2O$), 28.05 ($CH_3(CH_2)_2CH_2$), 26.11 ($CH_2(CH_2)_2O$), 23.33 (CH_3CH_2), 23.17 (CH_2CH_2NH), 21.41 (d, J = 140.0 Hz), 21.39 (d, J = 140.2 Hz, PCH_2), 14.35 (CH_3).

^{31}P{^{1}H} NMR (162 MHz, CD_3OD): 28.41.

IR ν_{max} (KBr) 3432 (m, br), 3015 (s), 2957 (vs), 2931 (vs), 2872 (s), 2500–2300 (vs, vbr), 1611 (m), 1467 (m), 1380 (w, sh), 1228 (m), 1055 (s), 1003 (s).

HR-MS(ESI$^+$): For $C_{38}H_{84}O_6N_6P_2$ $(M+2H)^{2+}$ $m/2z$ calculated 391.29583, found 391.29589.

^{1}H NMR spectrum of compound **41**

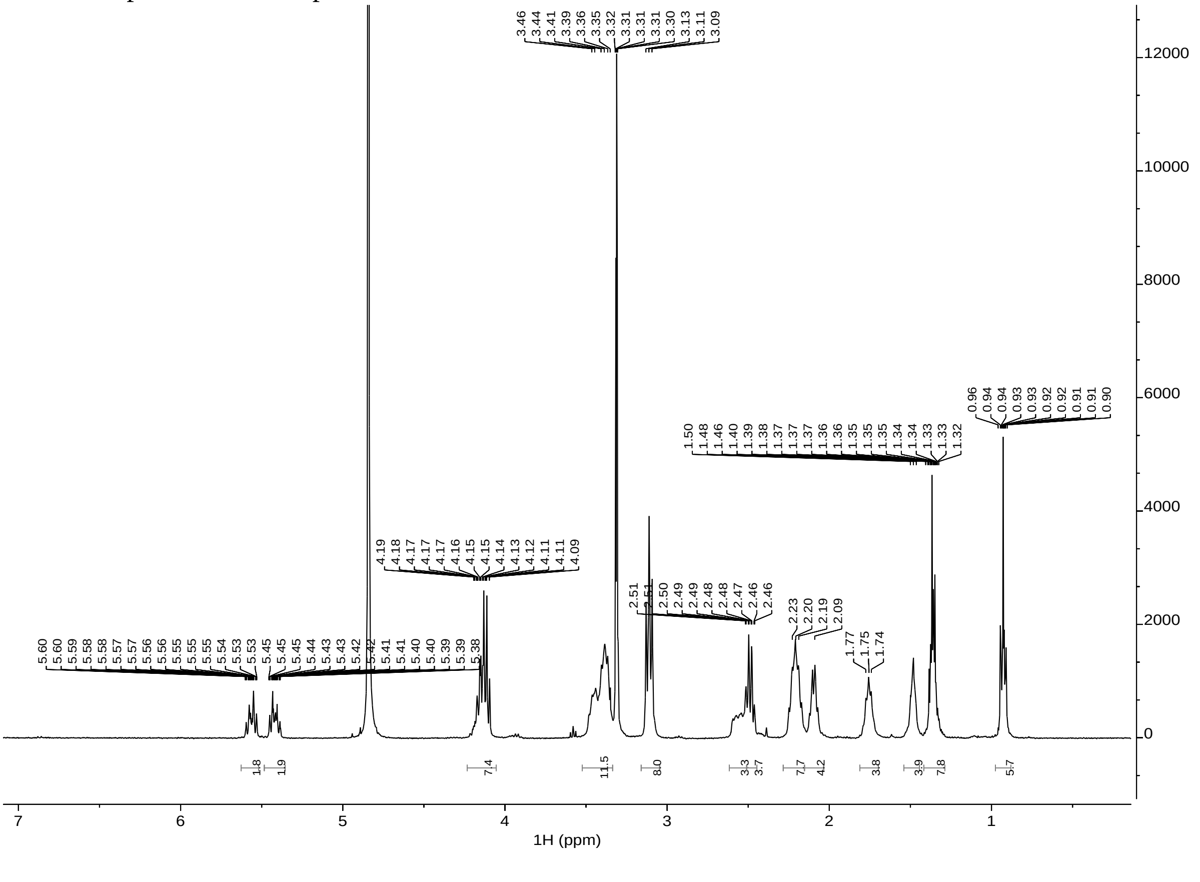

^{13}C NMR spectrum of compound **41**

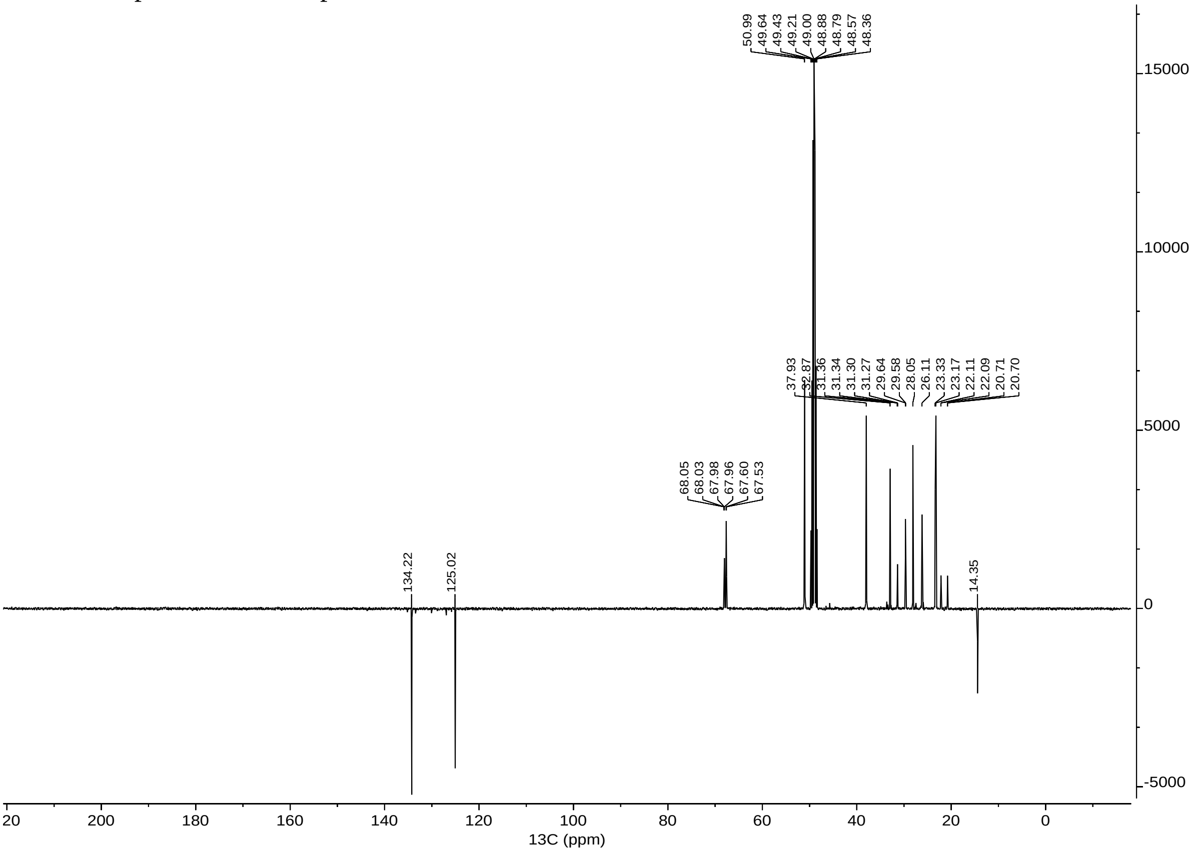

^{31}P{^{1}H} NMR spectrum of compound **41**

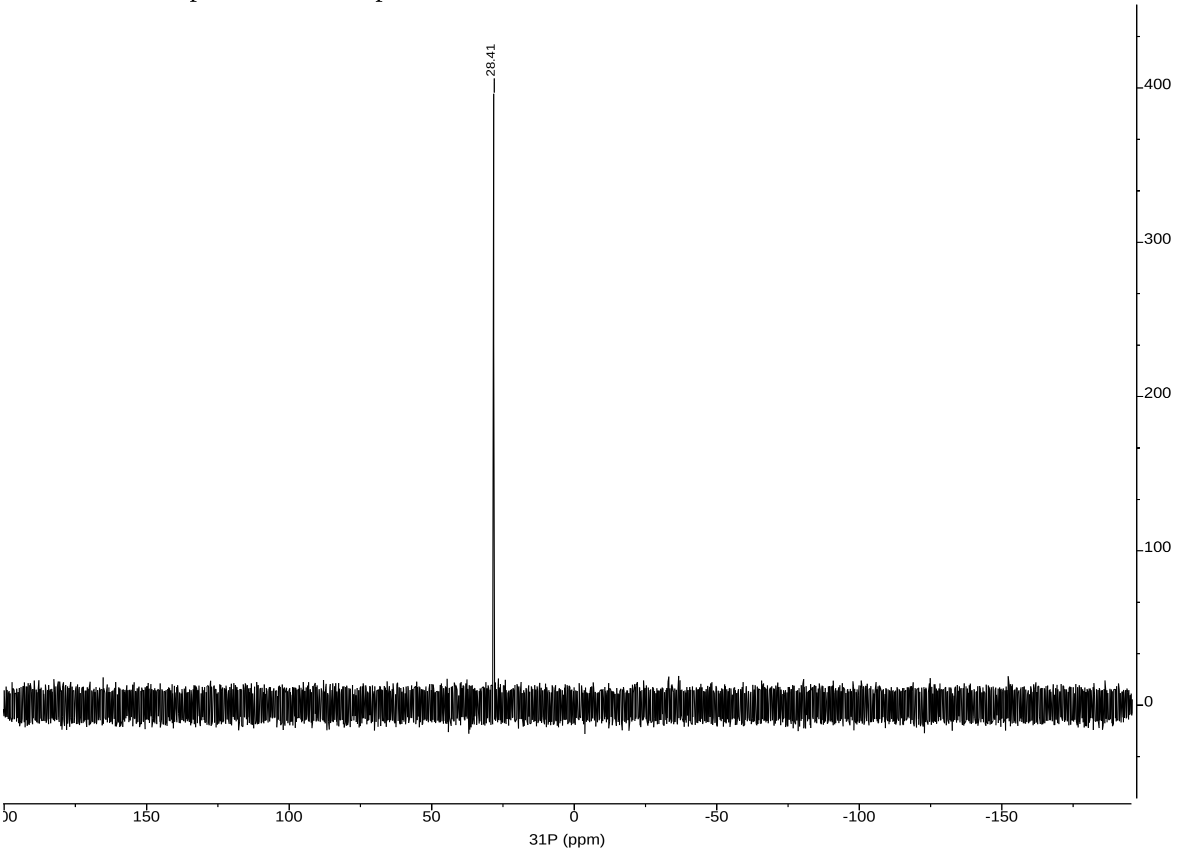

LC-MS chromatograms for compound **41**

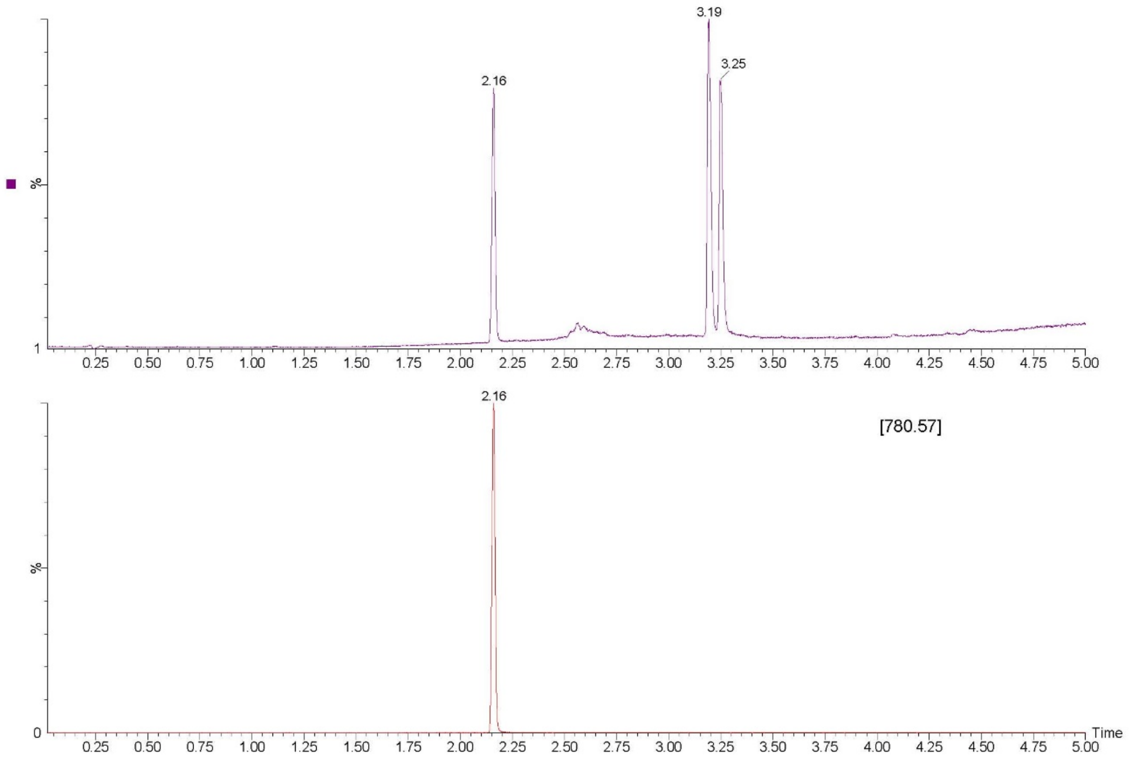

Hexane-1,6-diyl bis((*Z*)-oct-3-en-1-yl) bis((2-(bis(3-guanidinopropyl)amino)ethyl)phosphonate) hexahydrochloride (42)

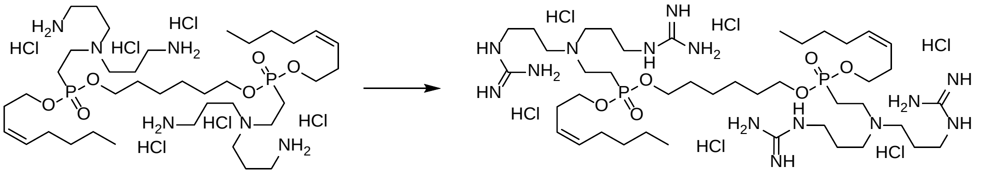

The title compound was prepared according to general method **F** from LPPO **41** (0.20 g, 0.20 mmol) in 67% yield (0.14 g, 0.13 mmol) as a white solid.
Mixture of diastereoisomers.
^{1}H NMR (400 MHz, CD_3OD): 5.62–5.51 (m, 2H, $CH_3(CH_2)_3CH$), 5.47–5.36 (m, 2H, $CH(CH_2)_2O$), 4.22–4.07 (m, 8H, CH_2O), 3.50–3.41 (m, 4H, PCH_2CH_2), 3.41–3.32 (m, 16H, $CH_2CH_2CH_2NH$), 2.64–2.43 (m, 8H, $CHCH_2CH_2O$, PCH_2), 2.18–2.03 (m, 12H, $CH_3(CH_2)_2CH_2$, CH_2CH_2NH), 1.80–1.69 (m, 4H, $OCH_2CH_2CH_2$), 1.52–1.43 (m, 4H, $O(CH_2)_2CH_2$), 1.41–1.30 (m, 8H, $CH_3(CH_2)_2$, 0.96–0.88 (m, 6H, CH_3).
^{13}C NMR (101 MHz, CD_3OD): 158.67 (*C*=NH), 134.33 ($CH_3(CH_2)_2CH$), 125.07 ($CH(CH_2)_2O$), 68.05 (d, J = 6.6 Hz), 68.02 (d, J = 6.6 Hz, $(CH_2)_2CH_2O$), 67.60 (d, J = 6.6 Hz, $CHCH_2CH_2O$), 51.55 (CH_2NH), 48.69 (PCH_2CH_2), 39.69 ($CH_2(CH_2)_2NH$), 32.93 (CH_3CH_2), 31.41 (d, J = 6.2 Hz), 31.38 (d, J = 6.2 Hz, $CH_2CH_2CH_2O$), 29.64 (d, J = 6.0 Hz, $CHCH_2CH_2O$), 28.11 ($CH_3(CH_2)_2CH_2$), 26.19, 26.17 ($CH_2(CH_2)_2O$), 24.78 (CH_2CH_2NH), 23.39 (CH_3CH_2), 21.35 (d, J = 140.2 Hz, PCH_2), 14.38 (CH_3).
^{31}P{^{1}H} NMR (162 MHz, CD_3OD): 28.80.
IR ν_{max} 3400–3200 (vs), 3316 (s), 3258 (s), 3148 (s), 3013 (w), 2956 (m), 2932 (m), 2871 (w), 1666 (vs), 1646 (s), 1619 (s, sh), 1466 (m), 1377 (m), 1224 (m), 1006 (s), 721 (w).
HR-MS(ESI^+): For $C_{42}H_{92}O_6N_{14}P_2$ $(M+2H)^{2+}$ $m/2z$ calculated 475.33943, found 475.33956.

^{1}H NMR spectrum of compound **42**

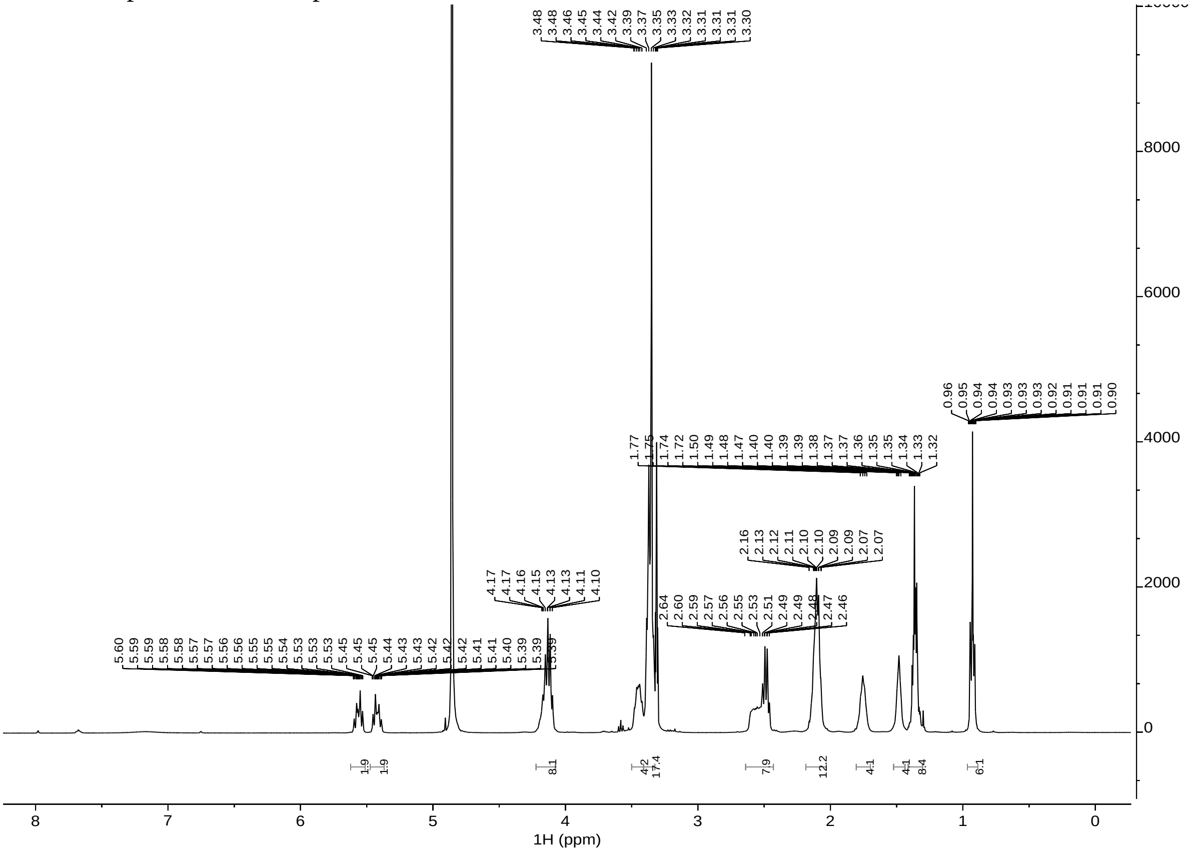

^{13}C NMR spectrum of compound **42**

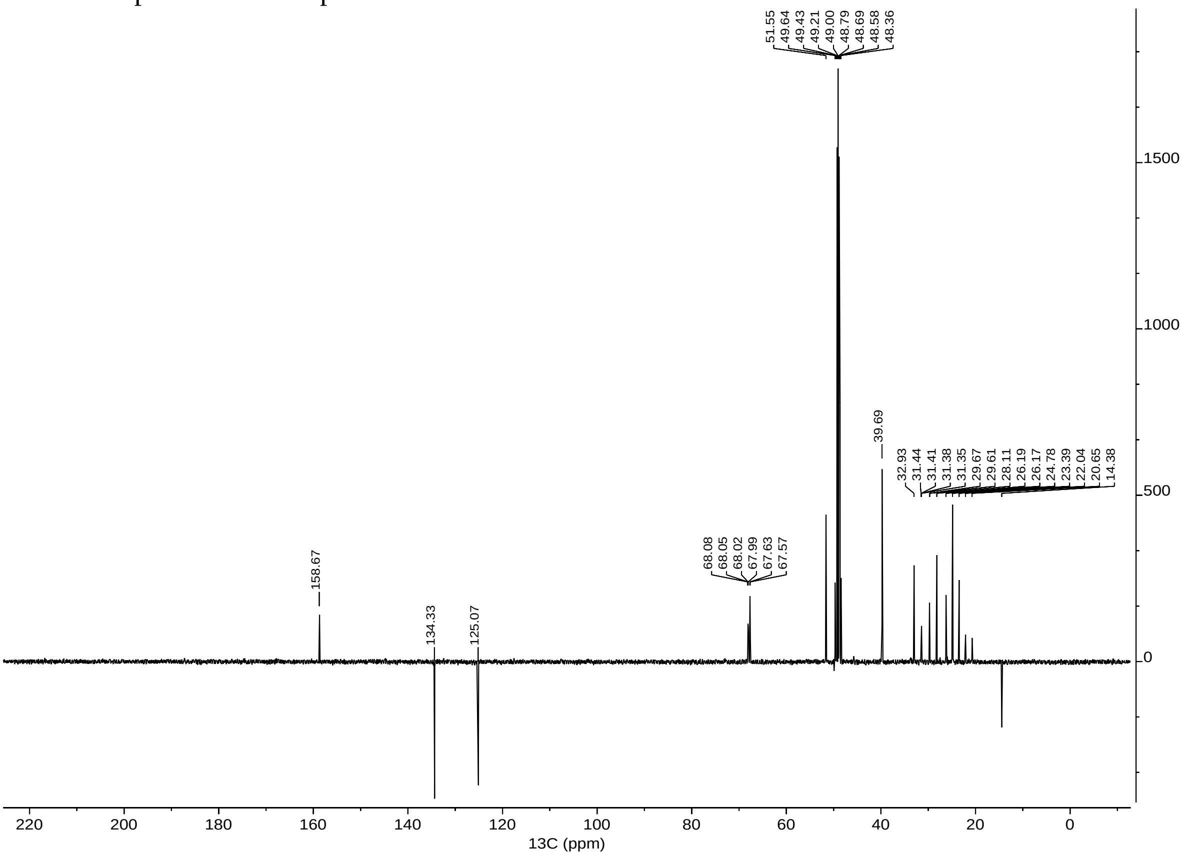

^{31}P{^{1}H} NMR spectrum of compound **42**

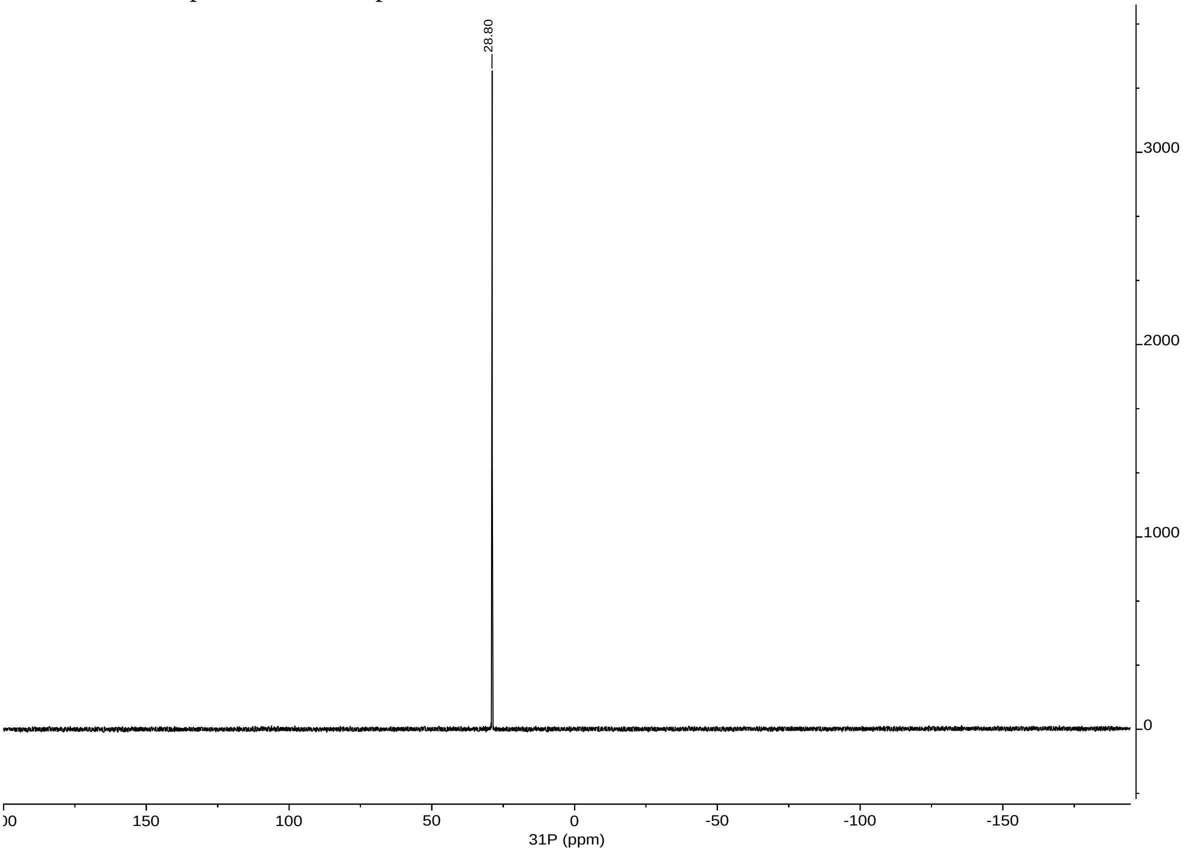

LC-MS chromatograms for compound **42**

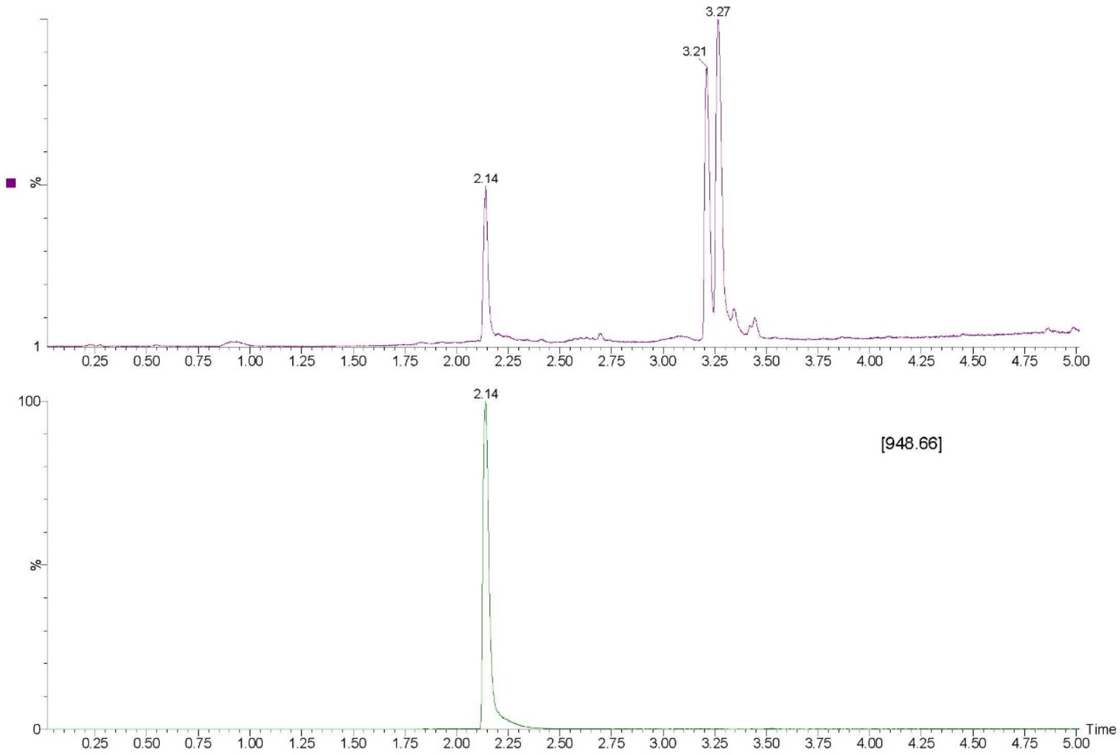

Hexane-1,6-diyl bis((Z)-oct-3-en-1-yl) bis((2-(bis(2-aminoethyl)amino)ethyl)phosphonate) hexahydrochloride (43)

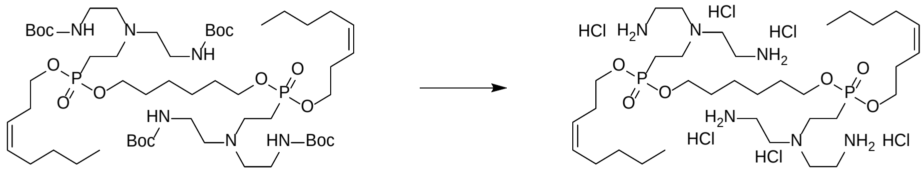

The title compound was prepared according to general method **E** from Boc derivative **S106** (0.38 g, 0.34 mmol) in 93% yield (0.30 g, 0.32 mmol) as a white solid.

Mixture of diastereoisomers.

^{1}H NMR (401 MHz, CD_3OD): 5.61–5.51 (m, 2H, $CH_3(CH_2)_3CH$), 5.47–5.37 (m, 2H, $CH(CH_2)_2O$), 4.17–4.03 (m, 8H, CH_2O), 3.24–3.14 (m, 8H, CH_2NH_2), 3.09–2.93 (m, 12H, PCH_2CH_2, CH_2N), 2.47 (q, 4H, J = 6.9 Hz, $CHCH_2CH_2O$), 2.33–2.18 (m, 4H, PCH_2), 2.15–2.04 (m, 4H, $CH_3(CH_2)_2CH_2$, 1.80–1.69 (m, 4H, $CH_2CH_2CH_2O$), 1.50–1.45 (m, 4H, $CH_2(CH_2)_2O$), 1.40–1.32 (m, 8H, $CH_3(CH_2)_2$), 0.96–0.89 (m, 6H, CH_3).

^{13}C NMR (101 MHz, CD_3OD): 134.30 ($CH_3(CH_2)_3CH$), 125.12 ($CH(CH_2)_2O$), 67.64 (d, J = 6.6 Hz, $(CH_2)_2CH_2O$), 67.19 (d, J = 6.7 Hz, $CHCH_2CH_2O$), 51.51 ($CH_2CH_2NH_2$), 37.67 (CH_2NH_2), 32.93 ($CH_3CH_2CH_2$), 31.49 (d, J = 6.1 Hz), 31.47 (d, J = 5.9 Hz, $CH_2CH_2CH_2O$), 29.65 (d, J = 6.2 Hz, $CHCH_2CH_2O$), 28.11 ($CH_3(CH_2)_2CH_2$), 26.24 ($CH_2(CH_2)_2O$), 23.40 (CH_3CH_2), 22.68 (d, J = 137.2 Hz, PCH_2), 14.37 (CH_3).

^{31}P{^{1}H} NMR (162 MHz, CD_3OD): 33.79.

IR ν_{max} (KBr) 3250–2500 (s), 3007 (s), 2959 (vs), 2930 (s), 2871 (s), 1652 (w), 1592 (m), 1469 (m), 1385 (w), 1227 (m), 1055 (s), 1007 (s), 790 (w).

HR-MS(ESI$^+$): For $C_{34}H_{75}O_6N_6P_2$ $(M+H)^+$ m/z calculated 725.52178, found 725.52136.

^{1}H NMR spectrum of compound **43**

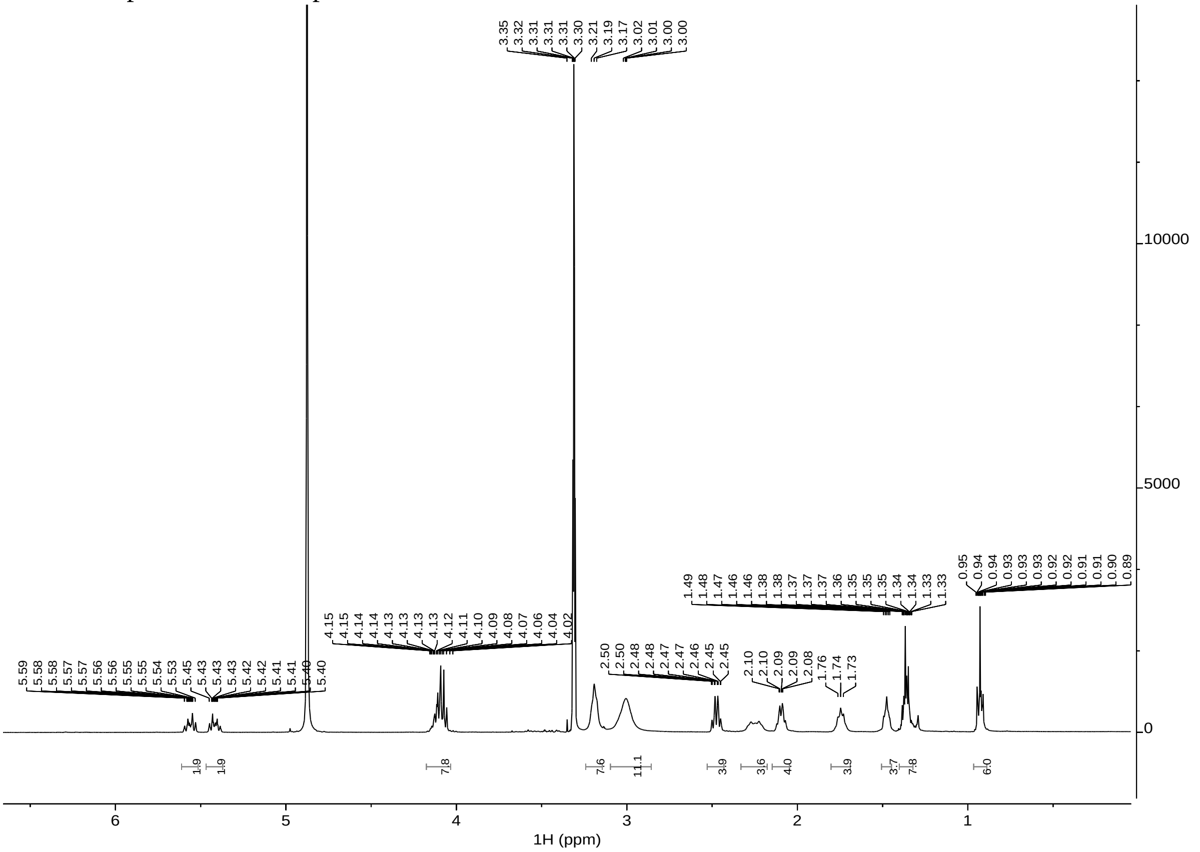

^{13}C NMR spectrum of compound **43**

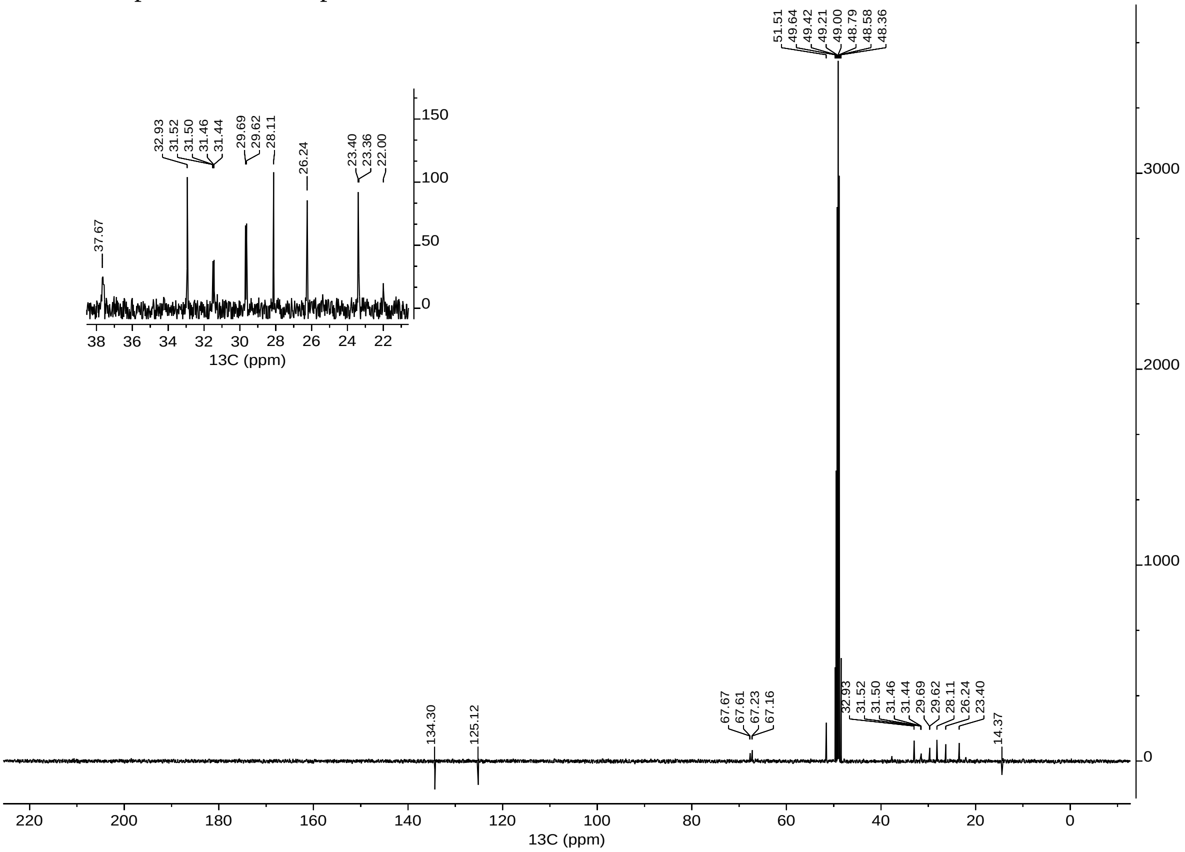

$^{31}P\{^1H\}$ NMR spectrum of compound **43**

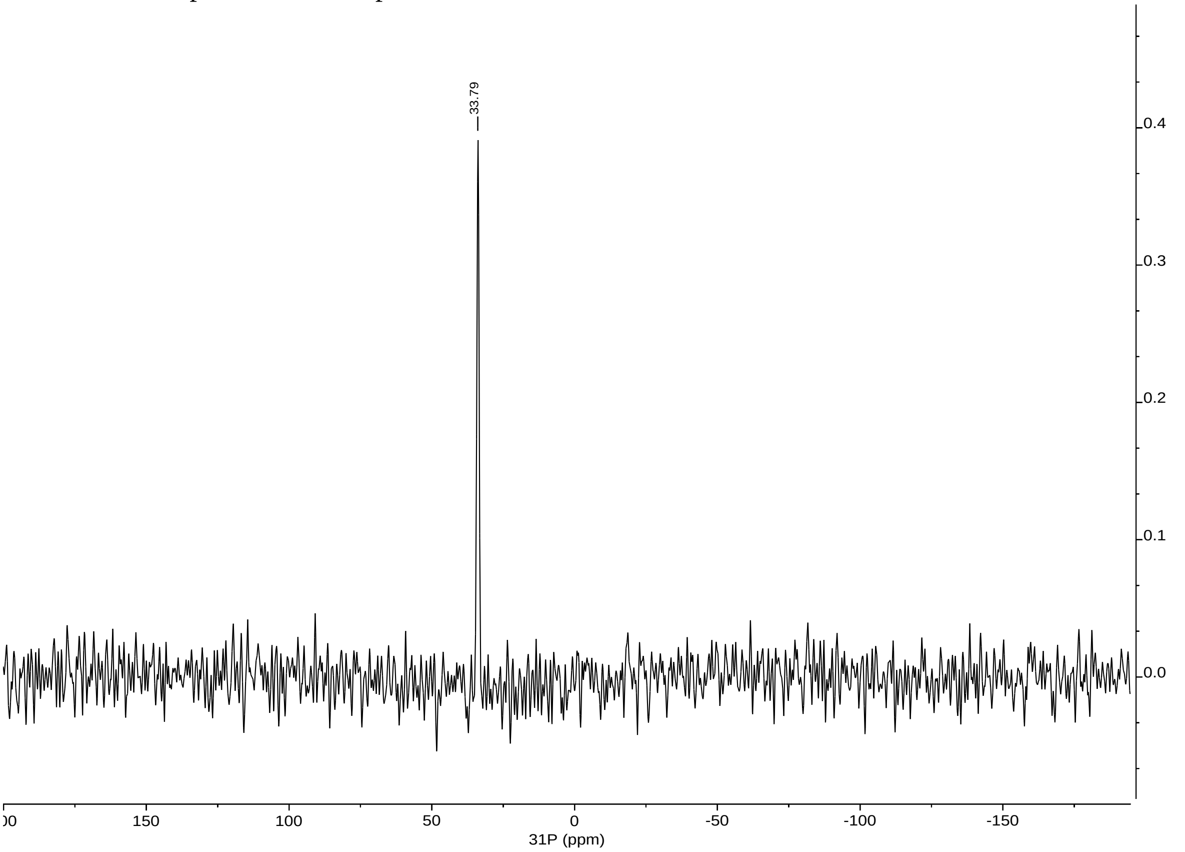

LC-MS chromatograms for compound **43**

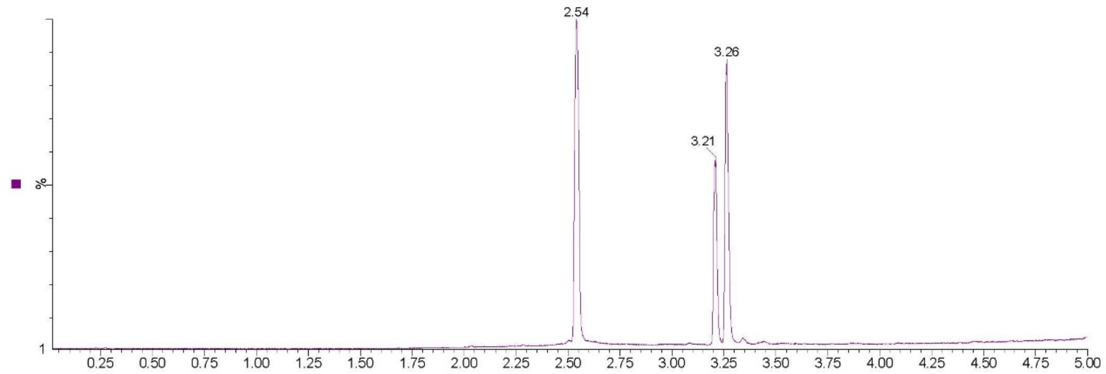

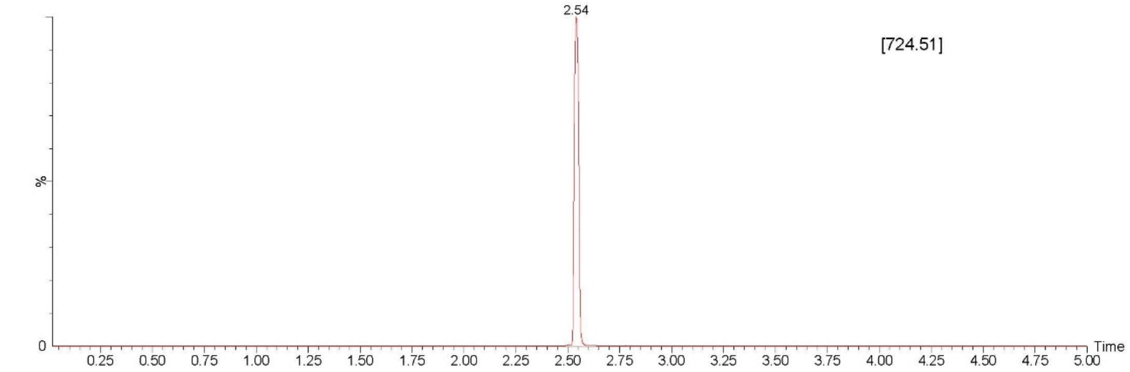

Hexane-1,6-diyl diphenethyl bis((2-(bis(3-aminopropyl)amino)ethyl)phosphonate) hexahydrochloride (44)

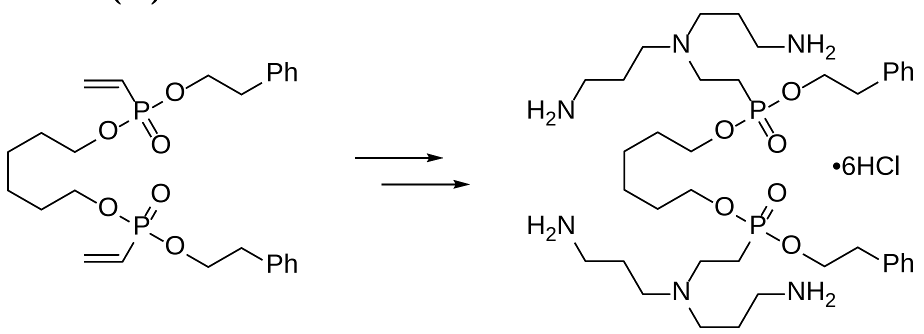

The title compound was prepared according to general methods **D** and **E** from vinylphosphonate dimer **S60** (1.14 g, 2.25 mmol) in 57% overall yield (1.27 g, 1.29 mmol) as a white solid.
Mixture of diastereoisomers.
^{1}H NMR (500.2 MHz, CD_3OD): 7.42–7.18 (m, 10H, Ph), 4.42–4.29 (m, 4H, C*H*$_2$CH$_2$Ph), 4.10–3.90 (m, 4H, OC*H*$_2$(CH$_2$)$_2$), 3.38–3.30 (m, 12H, NC*H*$_2$), 3.10 (t, 8H, *J* = 7.5 Hz, C*H*$_2$NH$_2$), 3.03 (t, 4H, *J* = 6.6 Hz, C*H*$_2$Ph), 2.48 (ddd, 4H, *J* = 21.5, 10.4, 5.6 Hz, PC*H*$_2$), 2.24–2.11 (m, 8H, C*H*$_2$CH$_2$NH$_2$), 1.65 (p, *J* = 6.5 Hz, 4H, OCH$_2$C*H*$_2$CH$_2$), 1.38 (td, *J* = 7.3, 6.8, 2.5 Hz, 4H, O(CH$_2$)$_2$C*H*$_2$).
^{13}C NMR (125.8 MHz, CD_3OD): 138.84 (C_{quat}), 130.27 (C_{ortho}), 129.71 (C_{meta}), 127.89 (C_{para}), 68.62 (d, *J* = 6.7 Hz, O*C*H$_2$CH$_2$Ph), 67.94 (d, *J* = 6.7 Hz), 67.91 (d, *J* = 7.1 Hz, O*C*H$_2$(CH$_2$)$_2$), 51.05 (*C*H$_2$(CH$_2$)$_2$NH$_2$), 48.72 (PCH$_2$*C*H$_2$), 37.87 (*C*H$_2$NH$_2$), 37.72 (d, *J* = 6.2 Hz, *C*H$_2$Ph), 31.28 (d, *J* = 5.8 Hz), 31.25 (d, *J* = 5.8 Hz, OCH$_2$*C*H$_2$CH$_2$), 26.04, 26.02 (O(CH$_2$)$_2$*C*H$_2$), 23.23 (*C*H$_2$CH$_2$NH$_2$), 21.25 (d, *J* = 140.1 Hz), 21.24 (d, *J* = 140.7 Hz, P*C*H$_2$).
^{31}P{^{1}H} NMR (202.5 MHz, CD_3OD): 26.43.
IR ν_{max} (KBr) 2950 (vs, vbr), 2930 (vs), 2858 (vs), 2033 (w, vbr), 2740 (s, br, sh), 2638 (s, br), 2559 (s, br), 1604 (s), 1521 (m, sh), 1497 (s), 1469 (s), 1454 (s), 1408 (m), 1395 (m), 1256 (s, br, sh), 1226 (s, br), 1157 (m), 1060 (s), 1009 (vs), 1000 (vs), 907 (m), 769 (m, sh), 753 (s), 702 (s), 621 (vw), 574 (m), 491 (m).
HR-MS(ESI$^+$): For $C_{38}H_{71}N_6O_6P_2$ $(M+H)^+$ *m/z* calculated 769.49048, found 769.48989.

^{1}H NMR spectrum of compound **44**

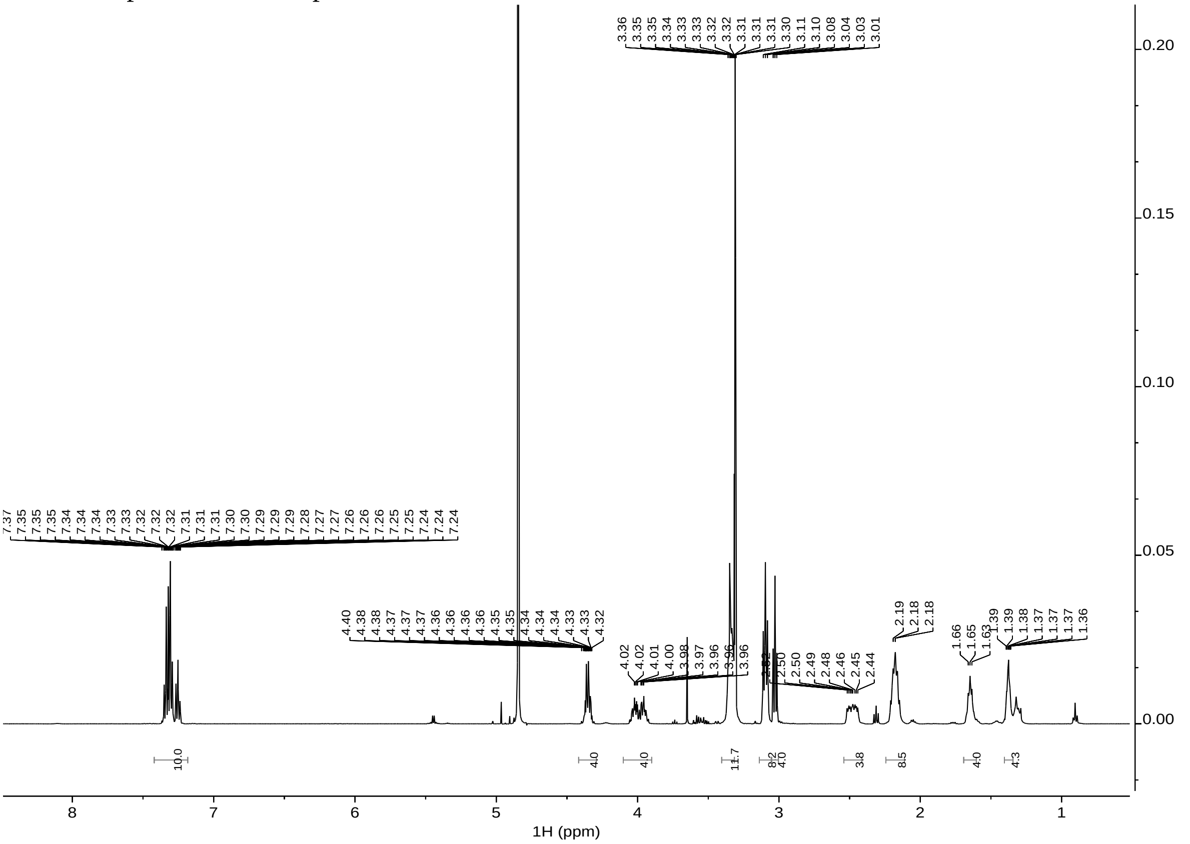

^{13}C NMR spectrum of compound **44**

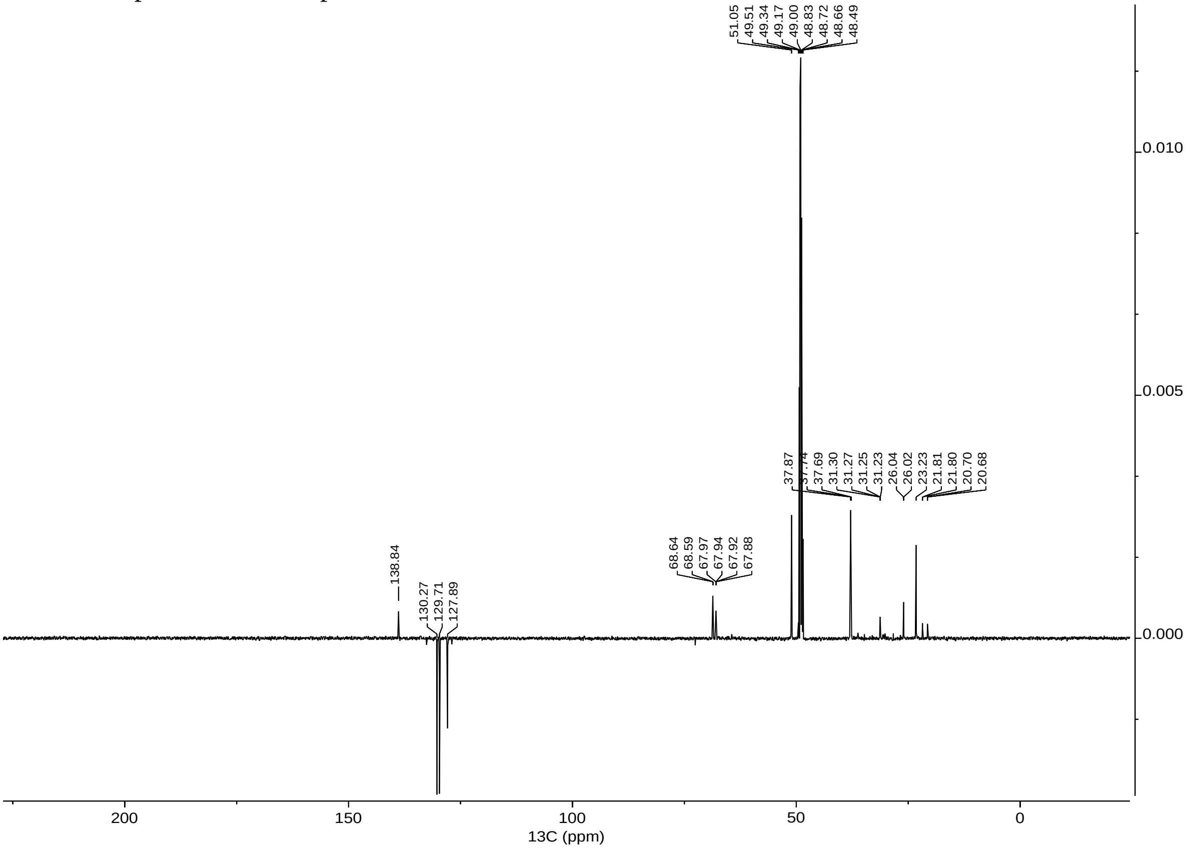

$^{31}P\{^{1}H\}$ NMR spectrum of compound **44**

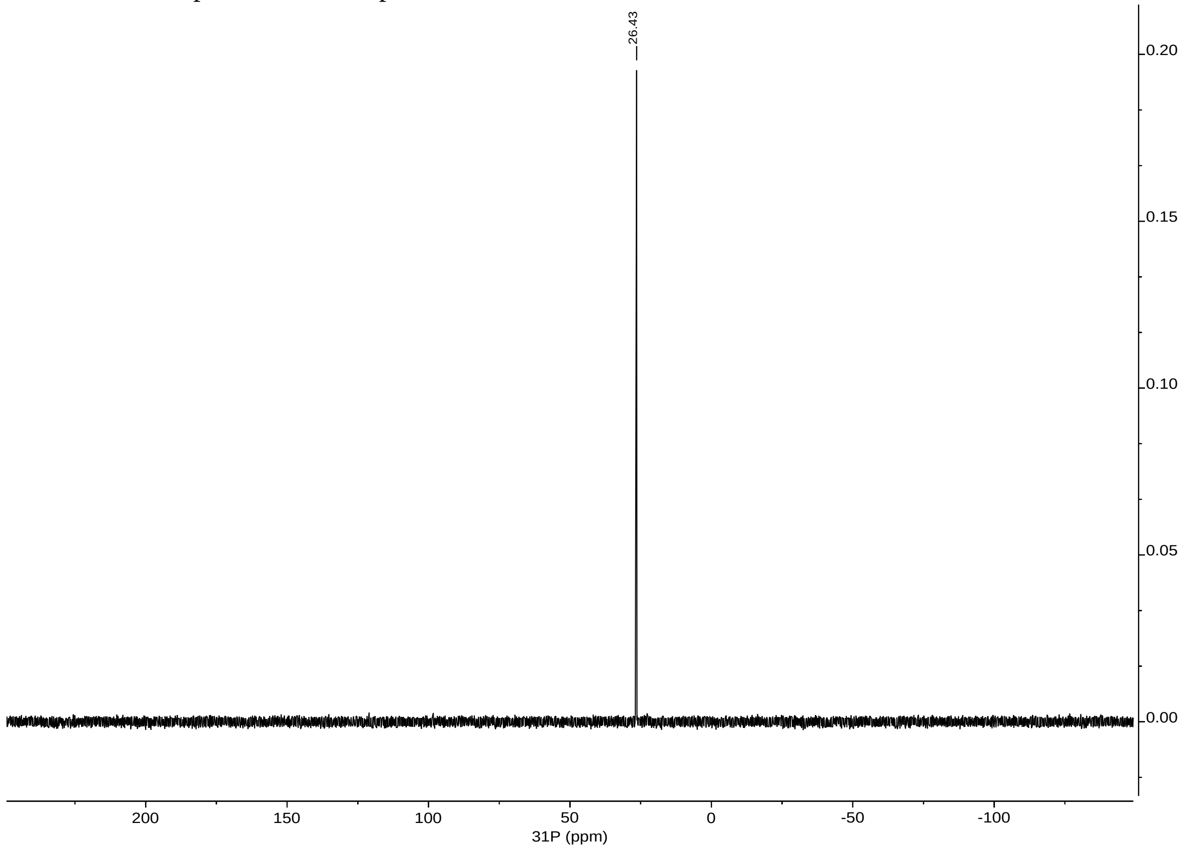

LC-MS chromatograms for compound **44**

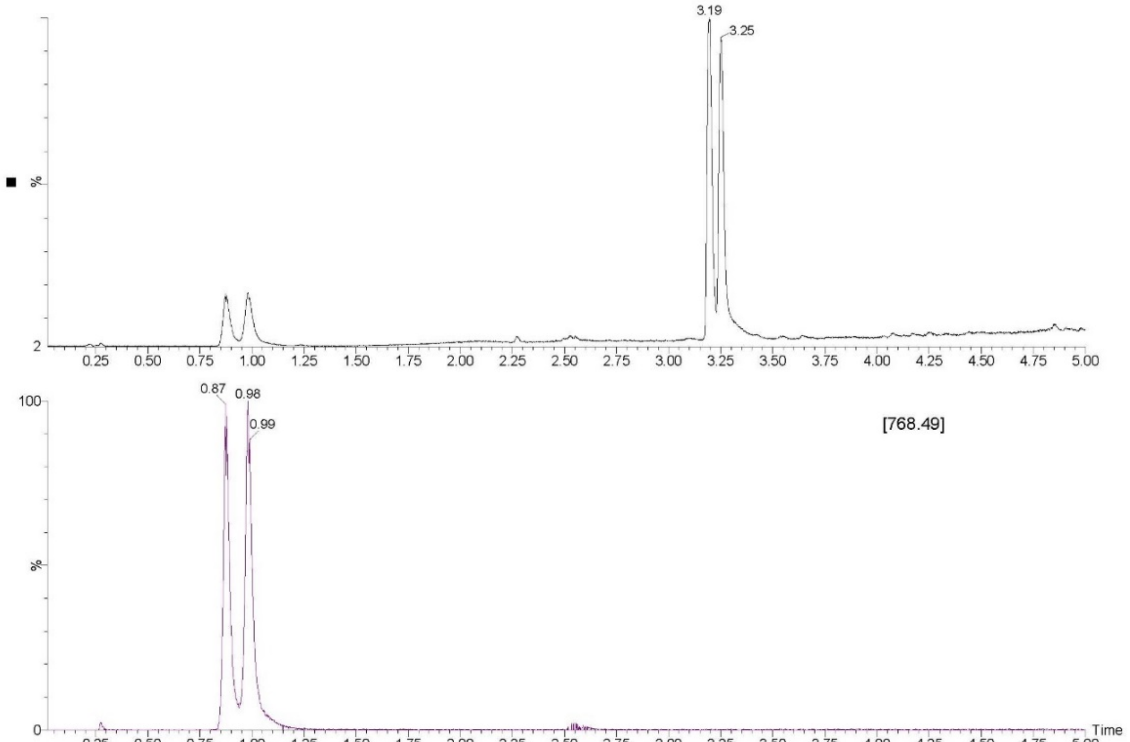

Hexane-1,6-diyl diphenethyl bis((2-(bis(2-aminoethyl)amino)ethyl)phosphonate) hexahydrochloride (45)

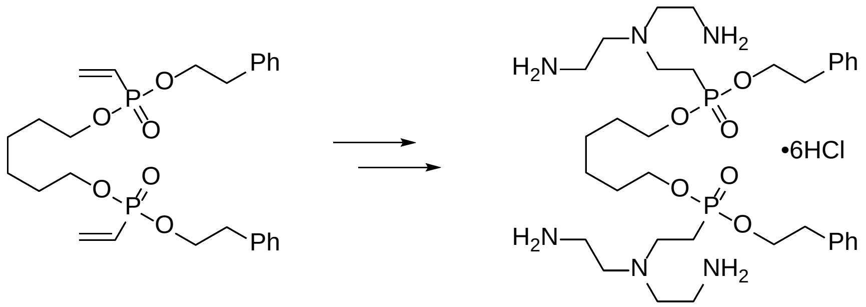

The title compound was prepared according to general methods **D** and **E** from vinylphosphonate dimer **S60** (0.47 g, 0.93 mmol) in 37% overall yield (0.26 g, 0.28 mmol) as a white solid.

^{1}H NMR (401 MHz, CD_3OD): 7.38–7.19 (m, 10H, Ph), 4.31 (q, 4H, J = 6.8 Hz, CH_2CH_2Ph), 4.04–3.87 (m, 4H, $OCH_2(CH_2)_4CH_2O$), 3.27–3.13 (m, 8H, (CH_2NH_2), 3.12–2.90 (m, 16H, NCH_2, CH_2Ph), 2.29–2.12 (m, 4H, PCH_2), 1.70–1.56 (m, 4H, $OCH_2CH_2CH_2$), 1.40–1.34 (m, 4H, $O(CH_2)_2CH_2$).

^{13}C NMR (101 MHz, CD_3OD): 138.94 (C_{quat}), 130.25 (C_{ortho}), 129.67 (C_{meta}), 127.87 (C_{para}), 68.31 (d, J = 6.6 Hz, CH_2CH_2Ph), 67.55 (d, J = 7.0 Hz), 67.54 (d, J = 6.8 Hz, $OCH_2(CH_2)_4CH_2O$), 51.47 ($CH_2CH_2NH_2$), 47.95 (PCH_2CH_2), 37.75 (d, J = 6.5 Hz, CH_2Ph), 37.30 (CH_2NH_2), 31.33 (d, J = 6.2 Hz), 31.31 (d, J = 6.3 Hz, $OCH_2CH_2CH_2$), 26.09, 26.08 ($O(CH_2)_2CH_2$), 22.41 (d, J = 138.5 Hz, PCH_2).

^{31}P{^{1}H} NMR (162 MHz, CD_3OD): 32.84.

IR ν_{max} (KBr) 3023 (vs), 2958 (vs), 2950 (vs, vbr), 2933 (vs), 2863 (s), 2635 (m, br, sh), 2554 (m, vbr), 1978 (vw, vbr)1602 (w), 1593 (w, br), 1496 (m), 1470 (m), 1454 (m), 1392 (w, br), 1258 (m, sh), 1225 (s, sh), 1212 (s), 1052 (s), 1013 (vs), 1001 (vs), 769 (w), 753 (m), 702 (m), 621 (vw), 574 (w), 489 (w).

HR-MS(ESI^{+}): For $C_{34}H_{63}N_6O_6P_2$ $(M+H)^{+}$ m/z calculated 713.42788, found 713.42754.

^{1}H NMR spectrum of compound **45**

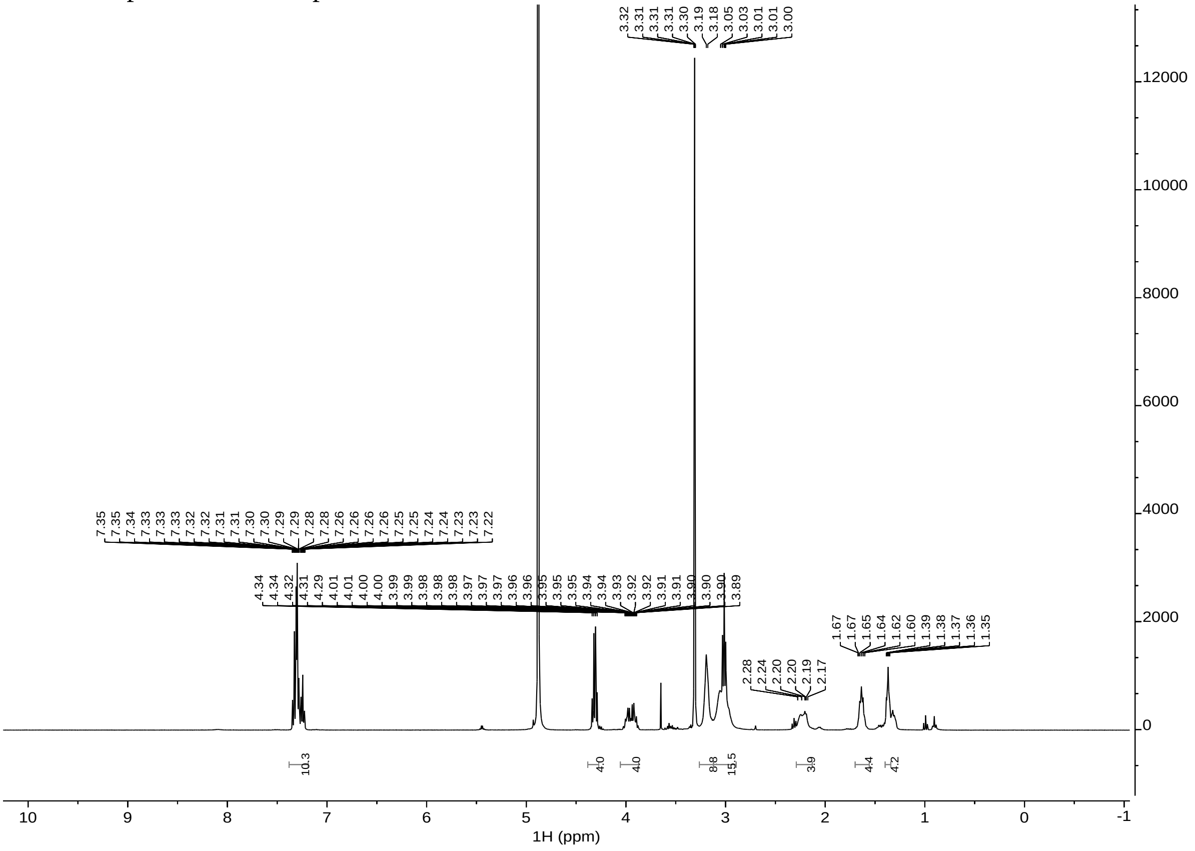

^{13}C NMR spectrum of compound **45**

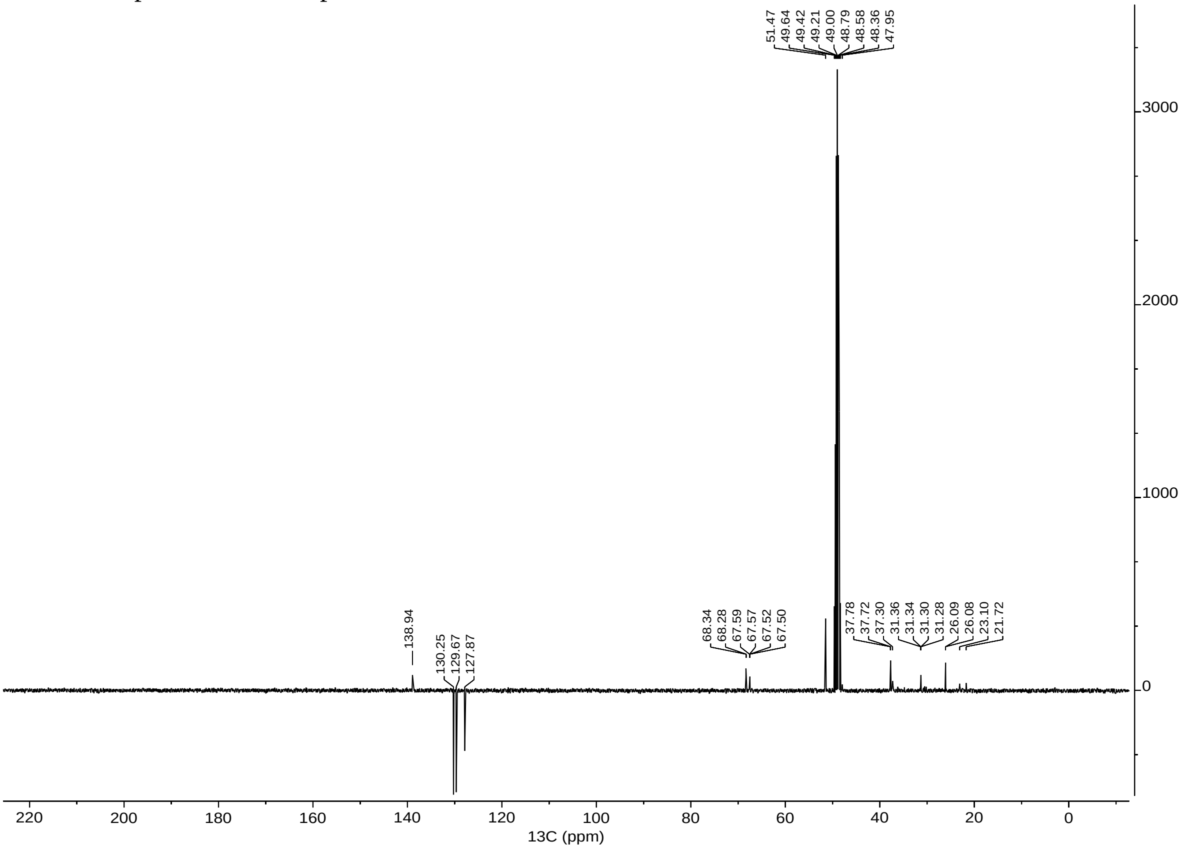

^{31}P{^{1}H} NMR spectrum of compound **45**

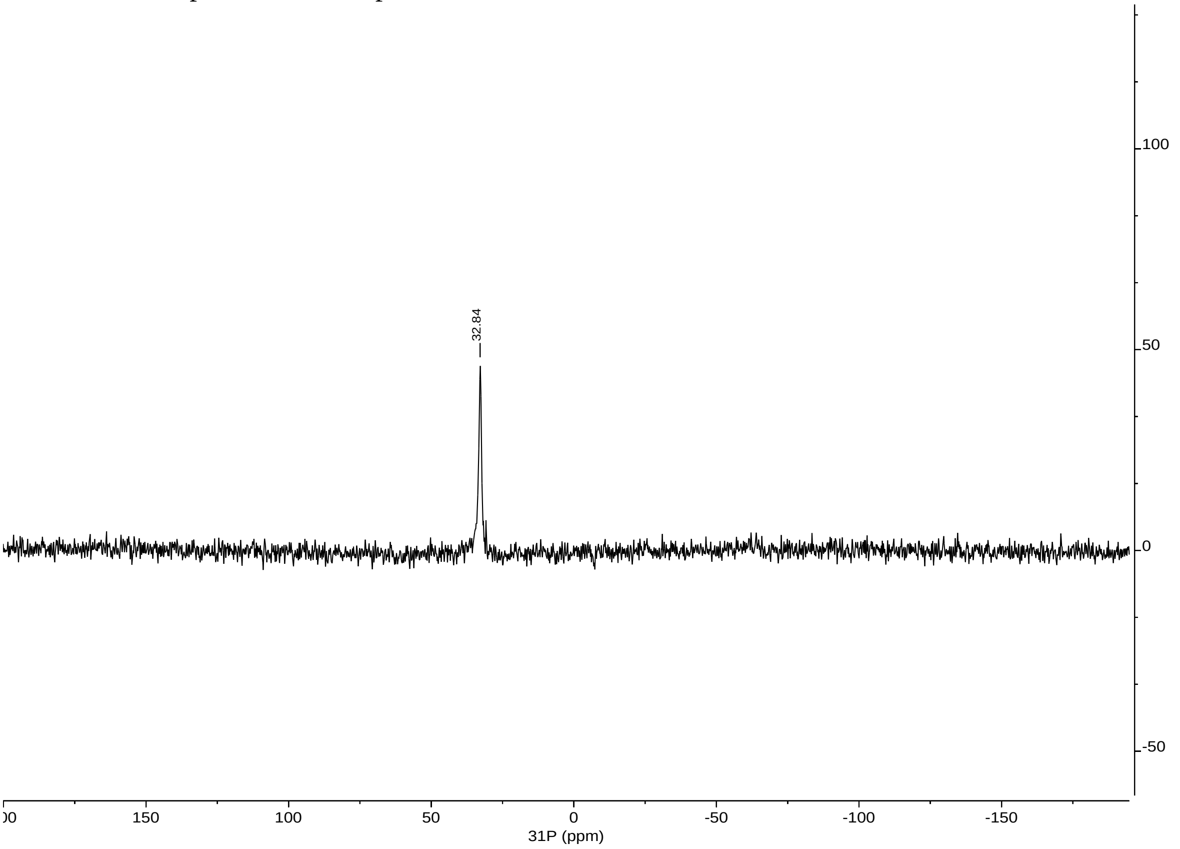

LC-MS chromatograms for compound **45**

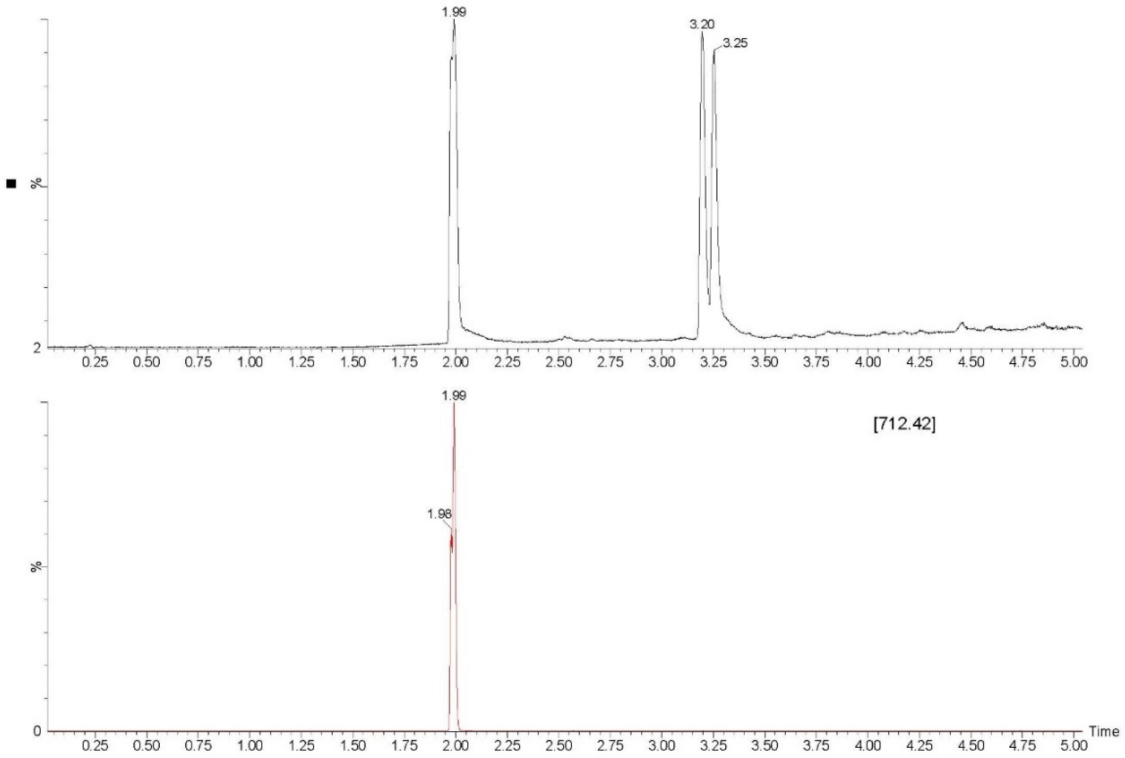

Hexane-1,6-diyl bis((*Z*)-non-3-en-1-yl) bis((2-(bis(3-aminopropyl)amino)ethyl)phosphonate) hexahydrochloride (46)

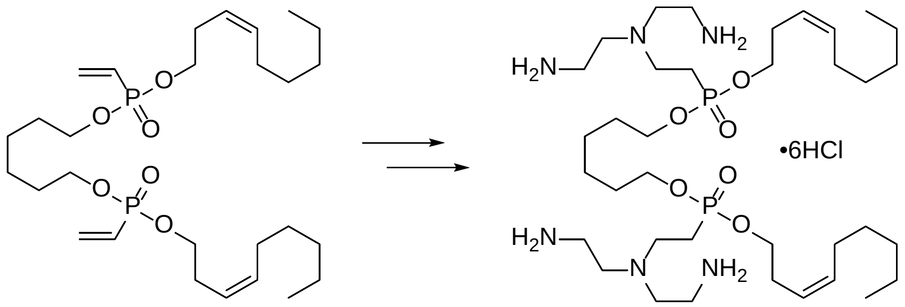

The title compound was prepared according to general methods **D** and **E** from vinylphosphonate dimer **S61** (1.09 g, 1.99 mmol) in 46% overall yield (0.95 g, 0.92 mmol) as a white solid.

^{1}H NMR (500.2 MHz, CD_3OD): 5.56 (dtt, 1H, J = 10.5, 7.3, ^{4}J = 1.6 Hz, $CH_3(CH_2)_4CH$), 5.42 (dtt, 1H, J = 10.5, 6.9, ^{4}J = 1.6 Hz, $CH(CH_2)_2O$), 4.21–4.09 (m, 8H, $CHCH_2CH_2O$, $OCH_2(CH_2)_2$), 3.50–3.44 (m, 4H, PCH_2CH_2), 3.44–3.37 (m, 8H, $CH_2(CH_2)_2NH_2$), 3.14–3.09 (m, 8H, CH_2NH_2), 2.62–2.52 (m, 4H, PCH_2), 2.48 (qd, 4H, J = 6.9, ^{4}J = 1.6 Hz, $CHCH_2CH_2O$), 2.27–2.18 (m, 8H, $CH_2CH_2NH_2$), 2.08 (qd, 4H, J = 7.3, ^{4}J = 1.6, $CH_3(CH_2)_3CH_2$), 1.81–1.72 (m, 4H, $OCH_2CH_2CH_2$), 1.45–1.51 (m, 4H, $O(CH_2)_2CH_2$), 1.42–1.27 (m, 12H, $CH_3(CH_2)_3$), 0.93–0.89 (m, 6H, CH_3).

^{13}C NMR (125.8 MHz, CD_3OD): 68.08, 68.06 (d, J = 6.7 Hz, $OCH_2(CH_2)_2$), 67.63 (d, J = 6.8 Hz, $CHCH_2CH_2O$), 51.06 ($CH_2(CH_2)_2NH_2$), 48.87 (PCH_2CH_2), 37.88 (CH_2NH_2), 32.62 (CH_3CH_2), 31.37, 31.34 (d, J = 5.9 Hz, $OCH_2CH_2CH_2$), 30.36 ($CH_3(CH_2)_2CH_2$), 29.64 (d, J = 6.0 Hz, $CHCH_2CH_2O$), 28.34 ($CH_3(CH_2)_3CH_2$), 26.13, 26.12 ($O(CH_2)_2CH_2$), 23.60 ($CH_3CH_2CH_2$), 23.21 ($CH_2CH_2NH_2$), 21.36, 21.35 (d, J = 140.3 Hz, PCH_2), 14.42 (CH_3).

^{31}P{^{1}H} NMR (202.5 MHz, CD_3OD): 26.94.

IR ν_{max} (KBr) 3010 (s, sh), 2957 (vs), 2930 (vs), 2872 (s), 2859 (s), 2009 (w, vbr), 2735 (m, sh), 2633 (m), 2558 (m), 1655 (vw, sh), 1601 (m), 1511 (m, sh), 1467 (m), 1406 (w), 1380 (w), 1252 (m, sh), 1227 (m), 1070 (s, sh), 1056 (s), 1003 (s), 808 (w).

HR-MS(ESI$^+$): For $C_{40}H_{88}N_6O_6P_2$ $(M+2H)^+$ $m/2z$ calculated 405.31148, found 405.31180.

^{1}H NMR spectrum of compound **46**

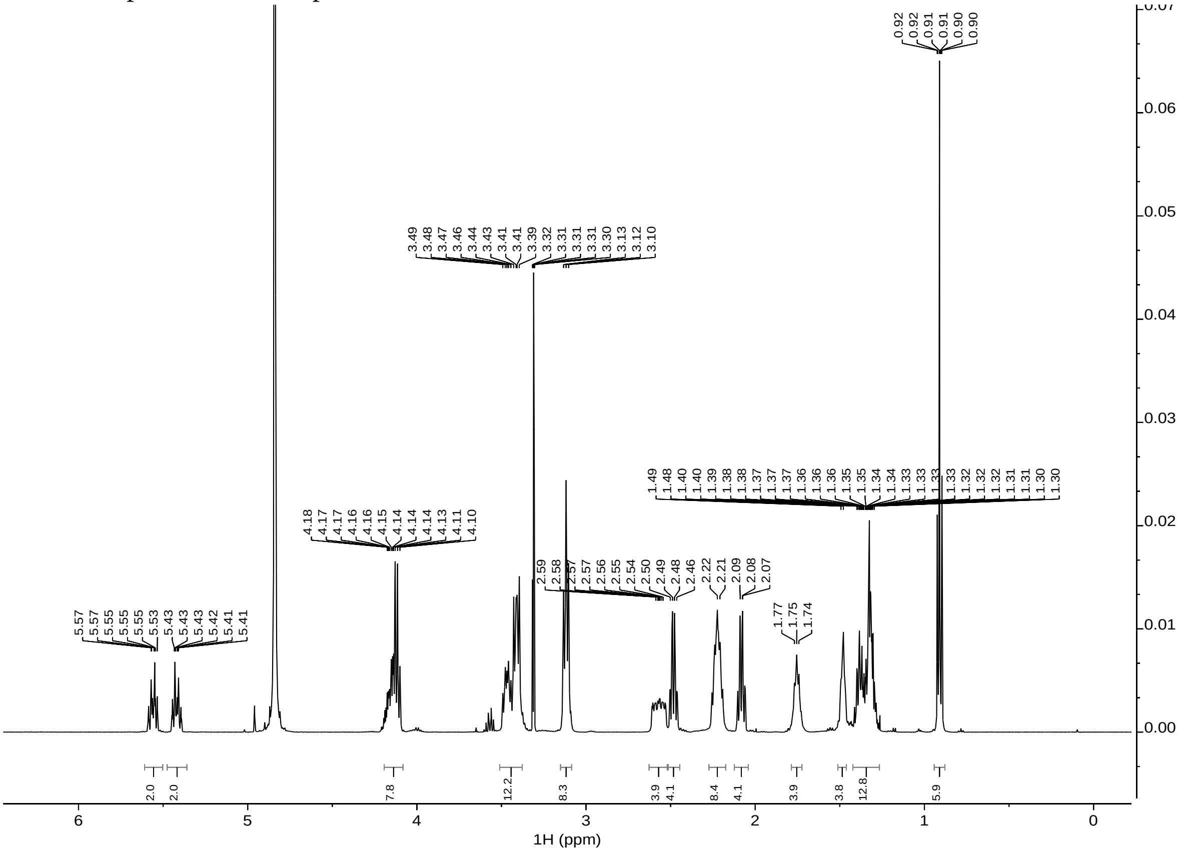

^{13}C NMR spectrum of compound **46**

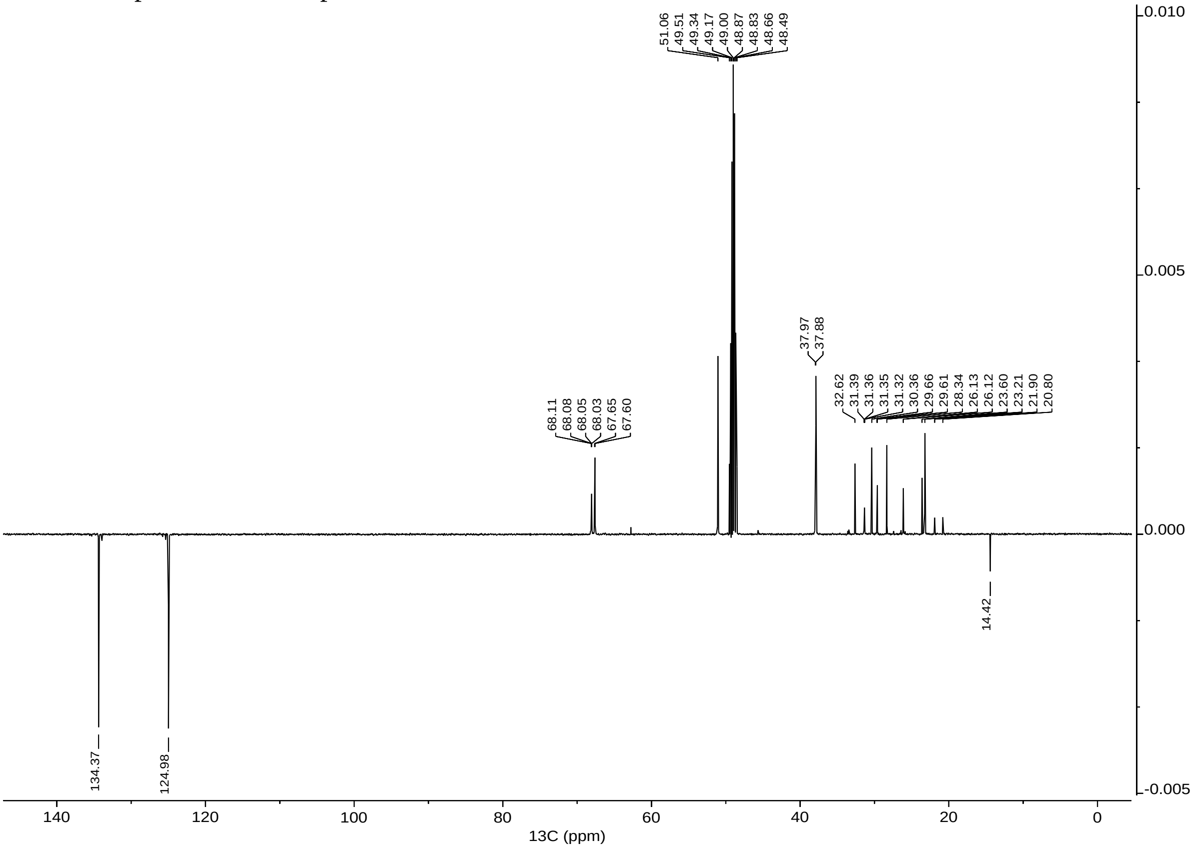

$^{31}P\{^{1}H\}$ NMR spectrum of compound **46**

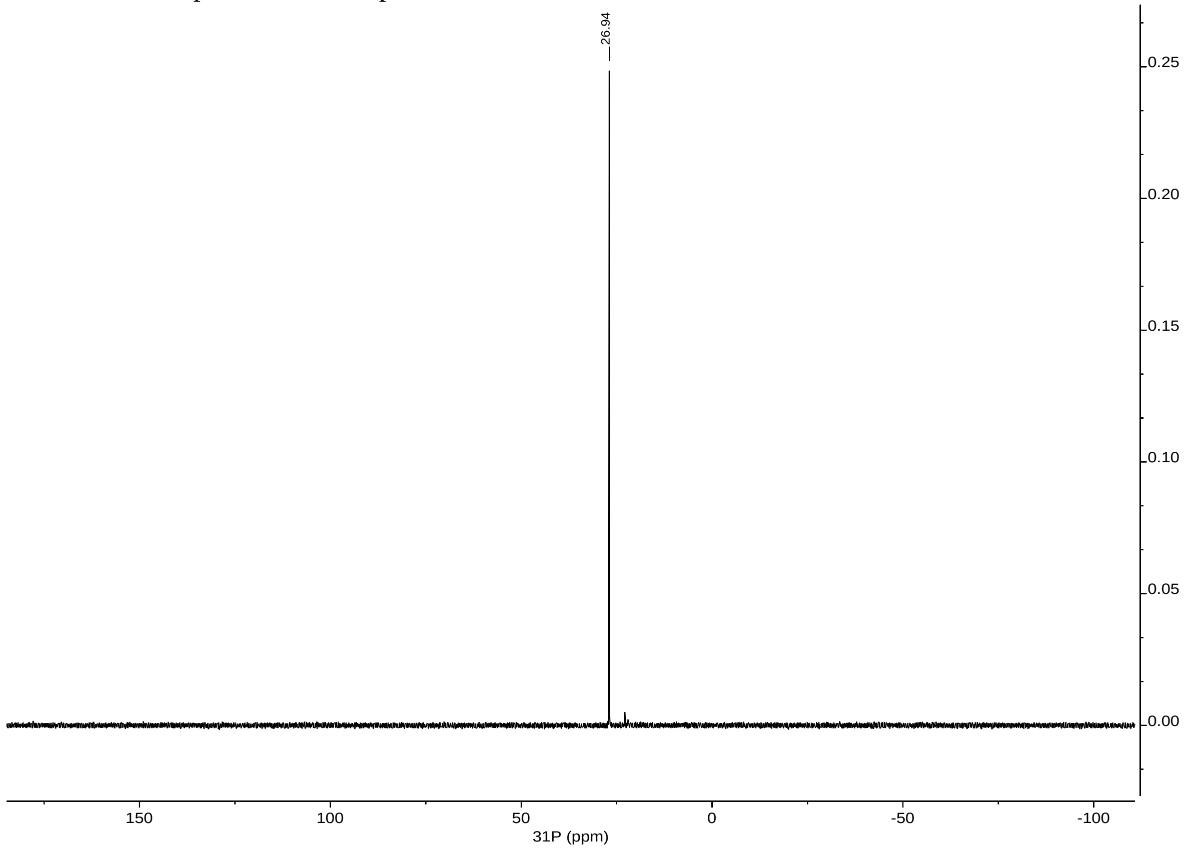

LC-MS chromatograms for compound **46**

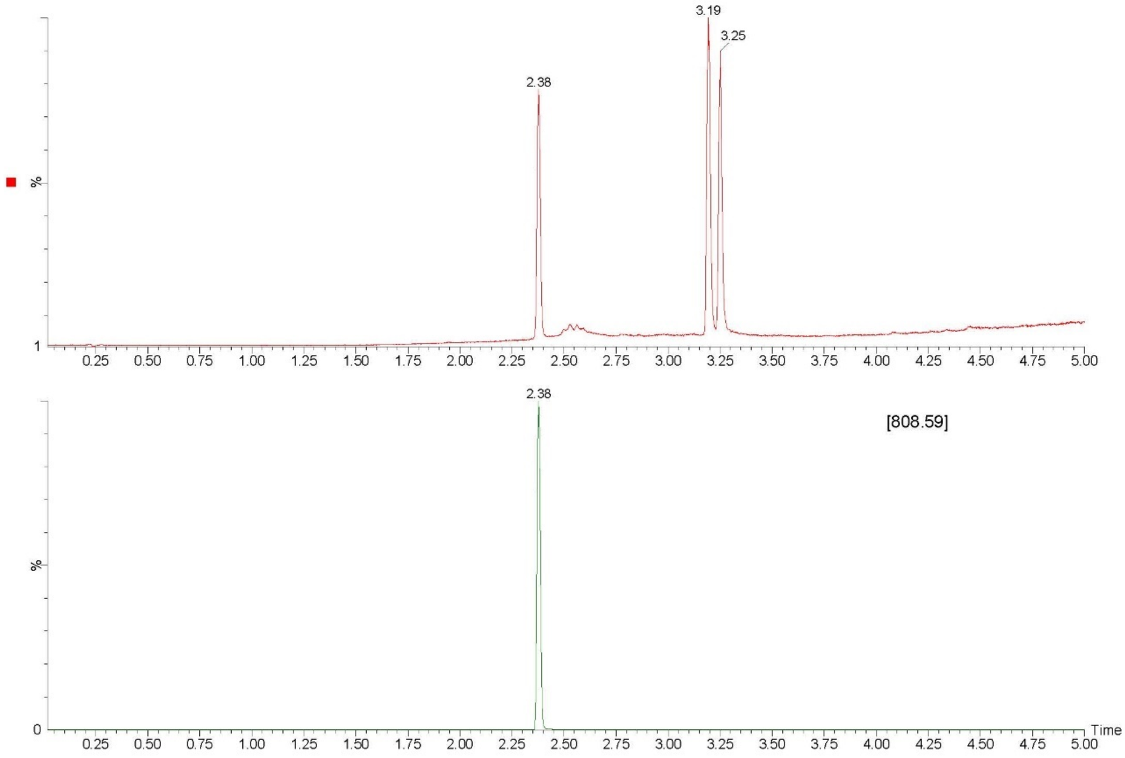

Didecyl hexane-1,6-diyl bis((2-(bis(3-aminopropyl)amino)ethyl)phosphonate) hexahydrochloride (47)

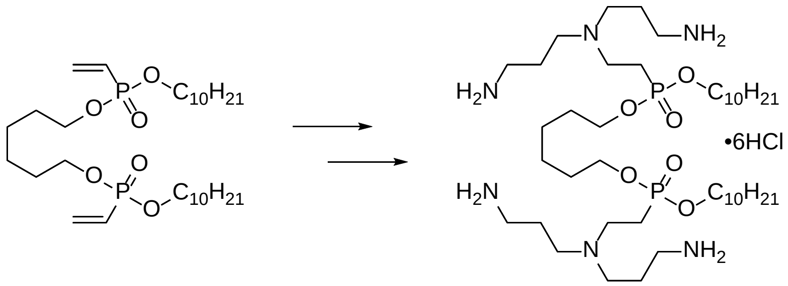

The title compound was prepared according to general methods **D** and **E** from vinylphosphonate dimer **S62** (1.20 g, 2.07 mmol) in 39% overall yield (0.85 g, 0.80 mmol) as a white solid.
Mixture of diastereoisomers.
^{1}H NMR (401 MHz, CD_3OD) δ 4.25–4.06 (m, 8H, OCH_2), 3.54–3.44 (m, 4H, PCH$_2$CH_2), 3.44–3.35 (m, 8H, CH_2(CH$_2$)$_2$NH$_2$), 3.12 (t, J = 7.5 Hz, 8H, CH_2NH$_2$), 2.64–2.50 (m, 4H, PCH_2), 2.22 (tt, J = 8.5, 6.0 Hz, 8H, CH_2CH$_2$NH$_2$), 1.84–1.65 (m, 8H, OCH$_2$CH_2), 1.49 (dt, J = 9.1, 2.9 Hz, 4H, O(CH$_2$)$_2$(CH_2)$_2$(CH$_2$)$_2$O), 1.44–1.36 (m, 4H, CH_2(CH$_2$)$_6$CH$_3$), 1.36–1.21 (m, 24H, (CH_2)$_6$CH$_3$), 0.96–0.84 (m, 6H, CH_3).
^{13}C NMR (101 MHz, CD_3OD) δ 68.24 (d, J = 6.8 Hz), 68.04 (d, J = 6.7 Hz, OCH$_2$(CH$_2$)$_4$$CH_2$O), 68.01 (d, J = 6.5 Hz, OCH$_2$(CH$_2$)$_8$CH$_3$), 51.01 (NCH$_2$(CH$_2$)$_2$NH$_2$), 48.87 (PCH$_2$$CH_2$), 37.88 ($CH_2NH_2$), 33.06 ($CH_2$(CH$_2$)$_5CH_3$), 31.61 (d, J = 5.9 Hz, CH$_2$(CH$_2$)$_7$CH$_3$), 31.39 (d, J = 5.8 Hz), 31.35 (d, J = 5.6 Hz, OCH$_2$$CH_2$(CH$_2$)$_2$$CH_2CH_2$O), 30.69, 30.45, 30.31, 26.62 (O(CH$_2$)$_2$(CH$_2$)$_2$(CH$_2$)$_2$O), 26.13 (CH$_2$(CH$_2$)$_6$CH$_3$), 23.73, 23.21 (CH$_2$CH$_2$NH$_2$), 21.32 (d, J = 139.1 Hz), 21.30 (d, J = 140.4 Hz, PCH$_2$), 14.45 (CH$_3$).
^{31}P{^{1}H} NMR (162 MHz, CD_3OD) δ 28.65, 28.64.
IR ν_{max} (KBr) 2956 (vs), 2926 (vs), 2855 (vs), 2740 (s, sh), 2629 (s, br), 2556 (m), 2010 (w, vbr), 1603 (m), 1521 (w, sh), 1468 (s), 1395 (m), 1254 (s, sh), 1228 (s), 1060 (s, sh), 1000 (vs, br), 723 (w).
HR-MS(ESI$^+$): For $C_{42}H_{95}N_6O_6P_2$ (M+H)$^+$ m/z calculated 841.67828, found 841.67850.

^{1}H NMR spectrum of compound **47**

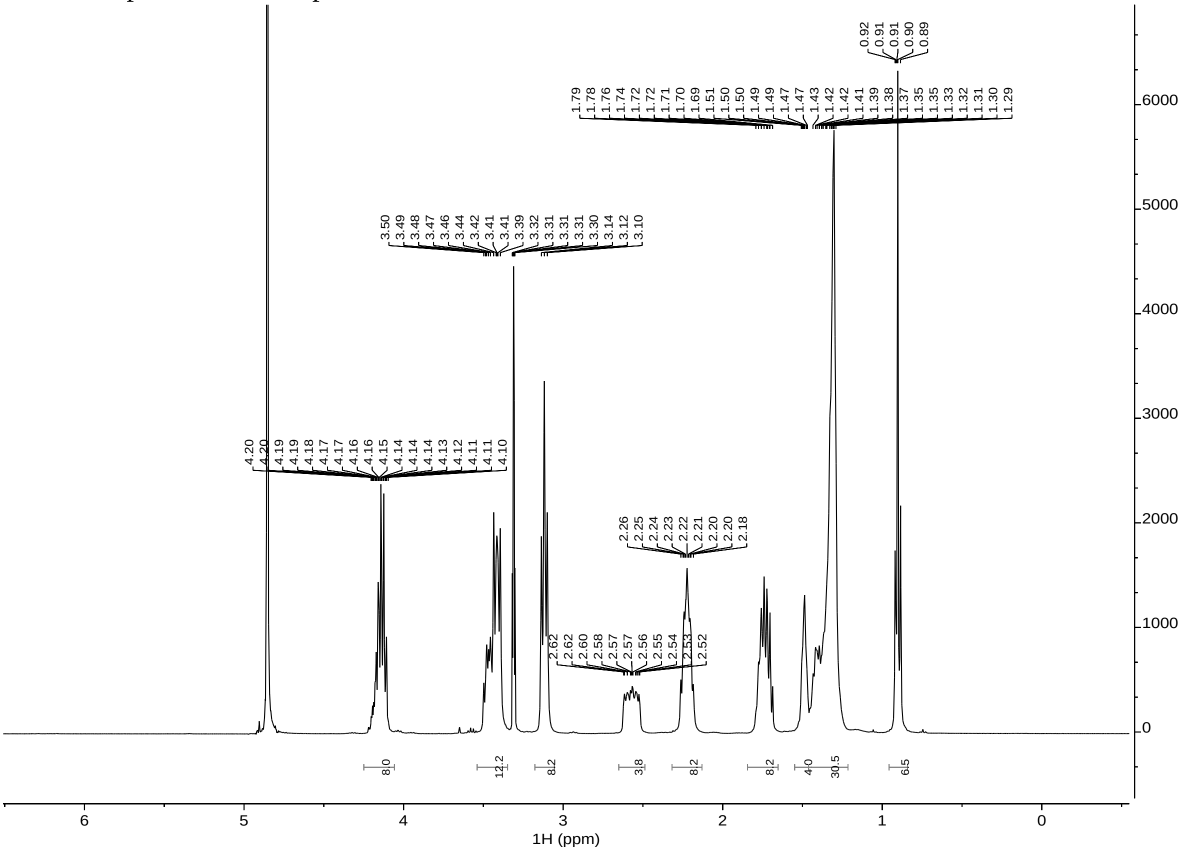

^{13}C NMR spectrum of compound **47**

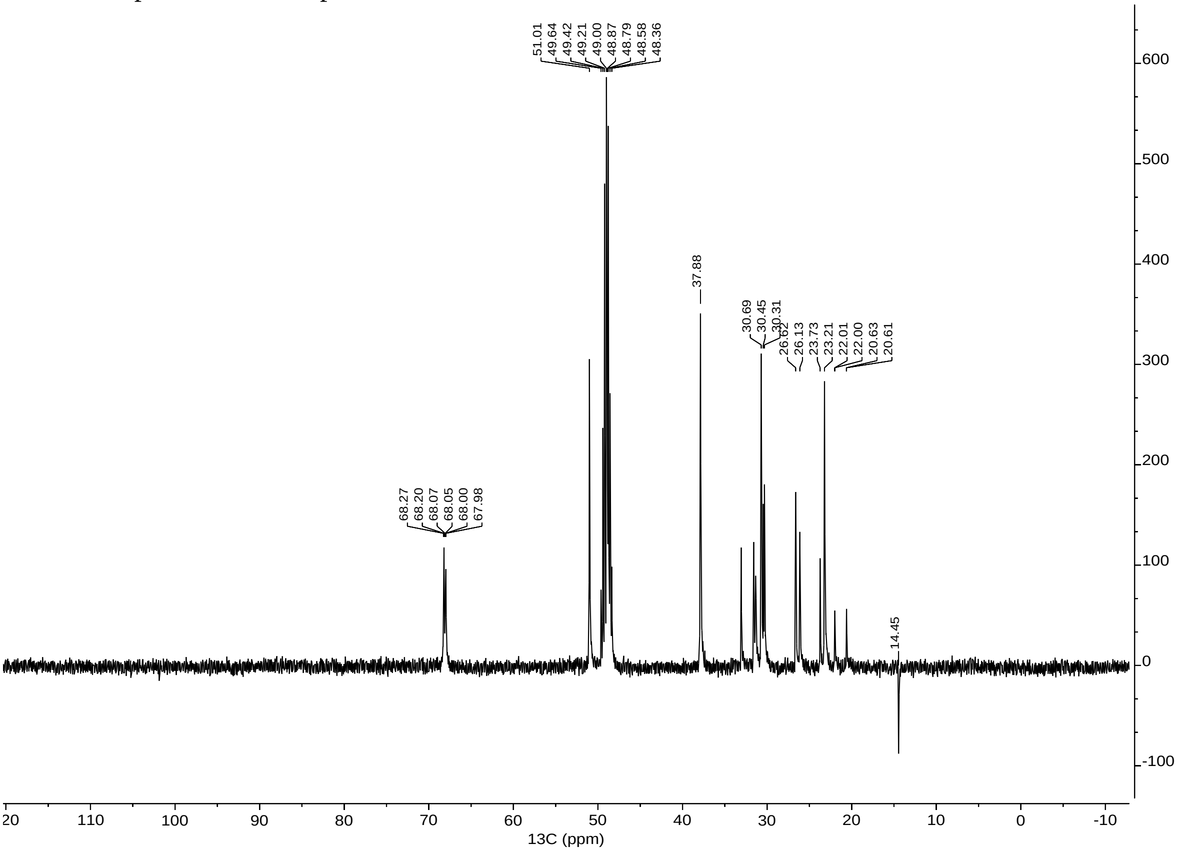

$^{31}P\{^1H\}$ NMR spectrum of compound **47**

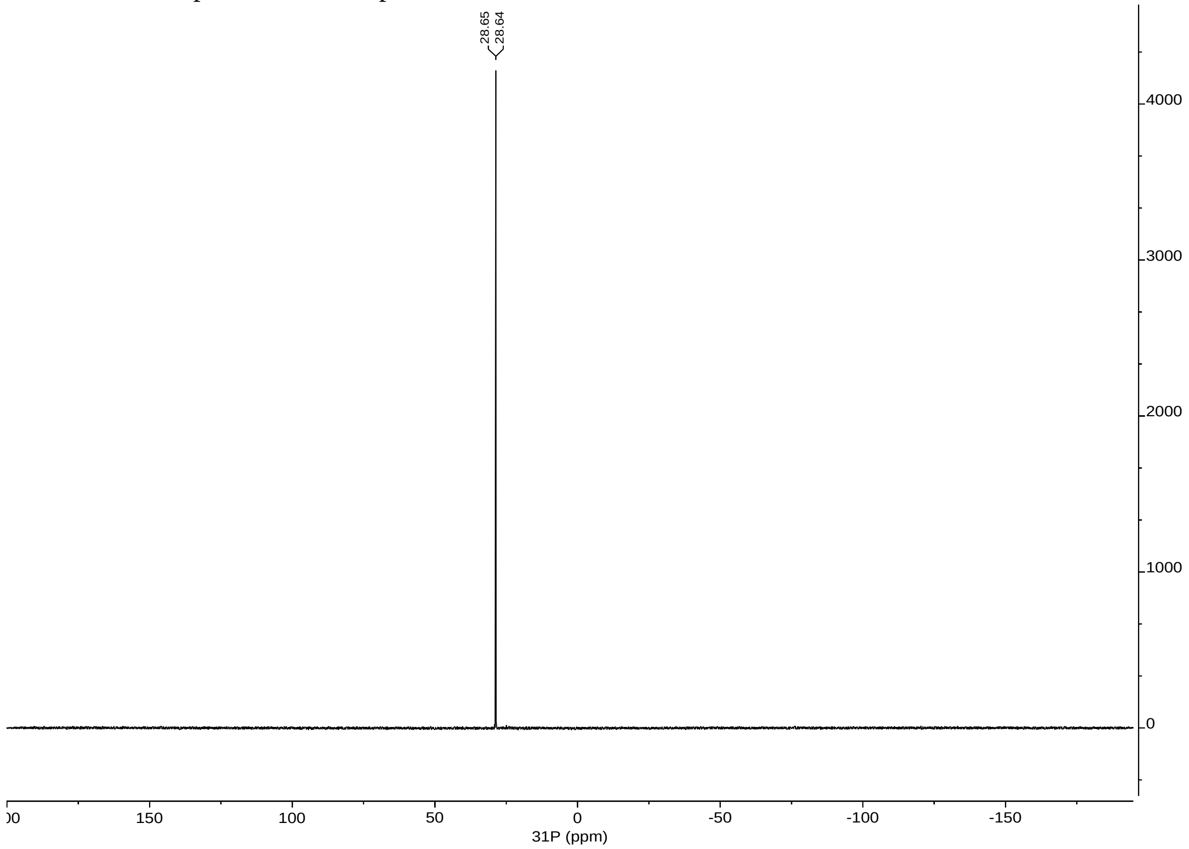

LC-MS chromatograms for compound **47**

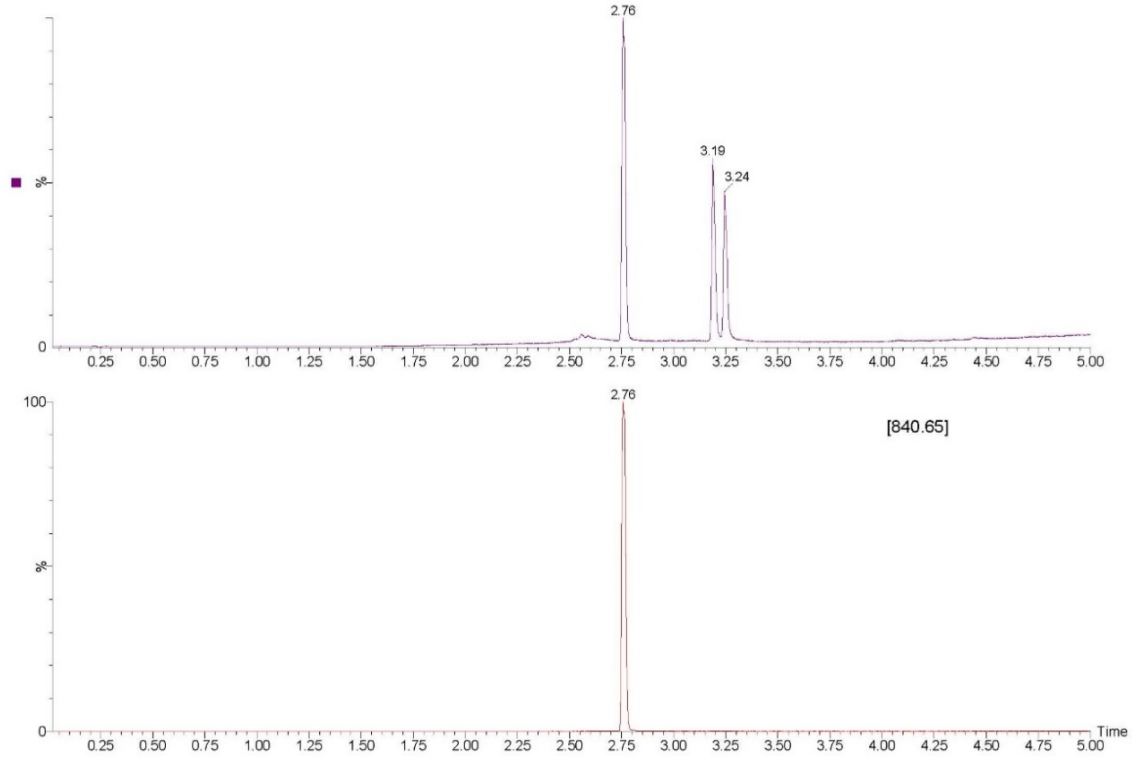

Bis((*Z*)-dec-4-en-1-yl) hexane-1,6-diyl bis((2-(bis(3-aminopropyl)amino)ethyl)phosphonate) hexahydrochloride (48)

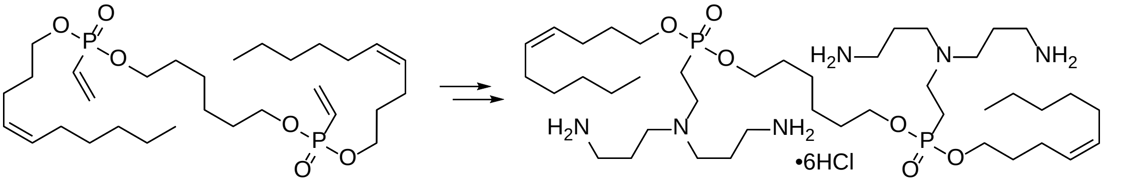

The title compound was prepared according to general methods **D** and **E** from vinylphosphonate dimer **S63** in 78% overall yield (290 mg, 0.29 mmol) as a white solid.

^{1}H NMR (500 MHz, CD_3OD) δ 5.49–5.35 (m, 4H, C*H*=C*H*), 4.22–4.09 (m, 8H, OC*H*$_2$), 3.50–3.42 (m, 4H, PCH_2CH_2), 3.42–3.35 (m, 8H, $PCH_2CH_2NCH_2$), 3.14–3.07 (m, 8H, CH_2NH_2), 2.55 (dtd, *J* = 19.3, 7.9, 7.4, 2.4 Hz, 4H, PCH_2), 2.27–2.14 (m, 12H, $CH_2CH_2NH_2$), 2.06 (q, *J* = 7.0 Hz, 4H, $CH_3(CH_2)_3CH_2$), 1.83–1.72 (m, 8H, OCH_2CH_2), 1.49 (tt, *J* = 4.4, 1.9 Hz, 4H, $O(CH_2)_2(CH_2)_2$), 1.44–1.24 (m, 12H, $CH_3(CH_2)_3$), 0.95–0.89 (m, 6H, CH_3).

^{13}C NMR (126 MHz, CD_3OD) δ 132.26 ($CH_3(CH_2)_4CH$), 129.08 ($O(CH_2)_3CH$), 68.08 (dd, *J* = 6.7, 2.8 Hz, $OCH_2(CH_2)_4CH_2O$), 67.75 (d, *J* = 6.7 Hz, $OCH_2(CH_2)_2CH$), 51.09 ($PCH_2CH_2NCH_2$), 48.83 (PCH_2CH_2), 37.92 (CH_2NH_2), 32.67 (CH_2CH_3), 31.71 (d, *J* = 5.9 Hz, $OCH_2CH_2CH_2CH$), 31.43 (dd, *J* = 5.8, 3.5 Hz, $OCH_2CH_2(CH_2)_2CH_2CH_2O$), 30.49 ($CH_3(CH_2)_2CH_2$), 28.20 ($CH_3(CH_2)_3CH_2$), 26.18 (d, *J* = 1.7 Hz, $(CH_2)_2(CH_2)_2O$), 24.19 ($O(CH_2)_2CH_2CH$), 23.65 ($CH_3CH_2CH_2$), 23.32 ($CH_2CH_2NH_2$), 21.35 (d, *J* = 139.9 Hz, PCH_2), 14.45 (CH_3).

^{31}P{^{1}H} NMR (202 MHz, CD_3OD) δ 27.07.

IR ν_{max} (film) 3395 (w), 3007 (s), 2956 (vs), 2927 (vs), 2872 (s), 2852 (s), 2560–2741 (m), 1655 (w), 1608 (m), 1467 (m), 1404 (m), 1379 (m), 1230 (m), 1003 (vs), 845 (m), 758 (w), 720 (w).

HR-MS(ESI$^+$): For $C_{42}H_{91}O_6N_6P_2$ $(M+H)^+$ *m*/*z* calculated 837.64698, found 837.64717. For $C_{42}H_{92}O_6N_6P_2$ $(M+2H)^{2+}$ *m*/2*z* calculated 419.32713, found 419.32717.

^{1}H NMR spectrum of compound **48**

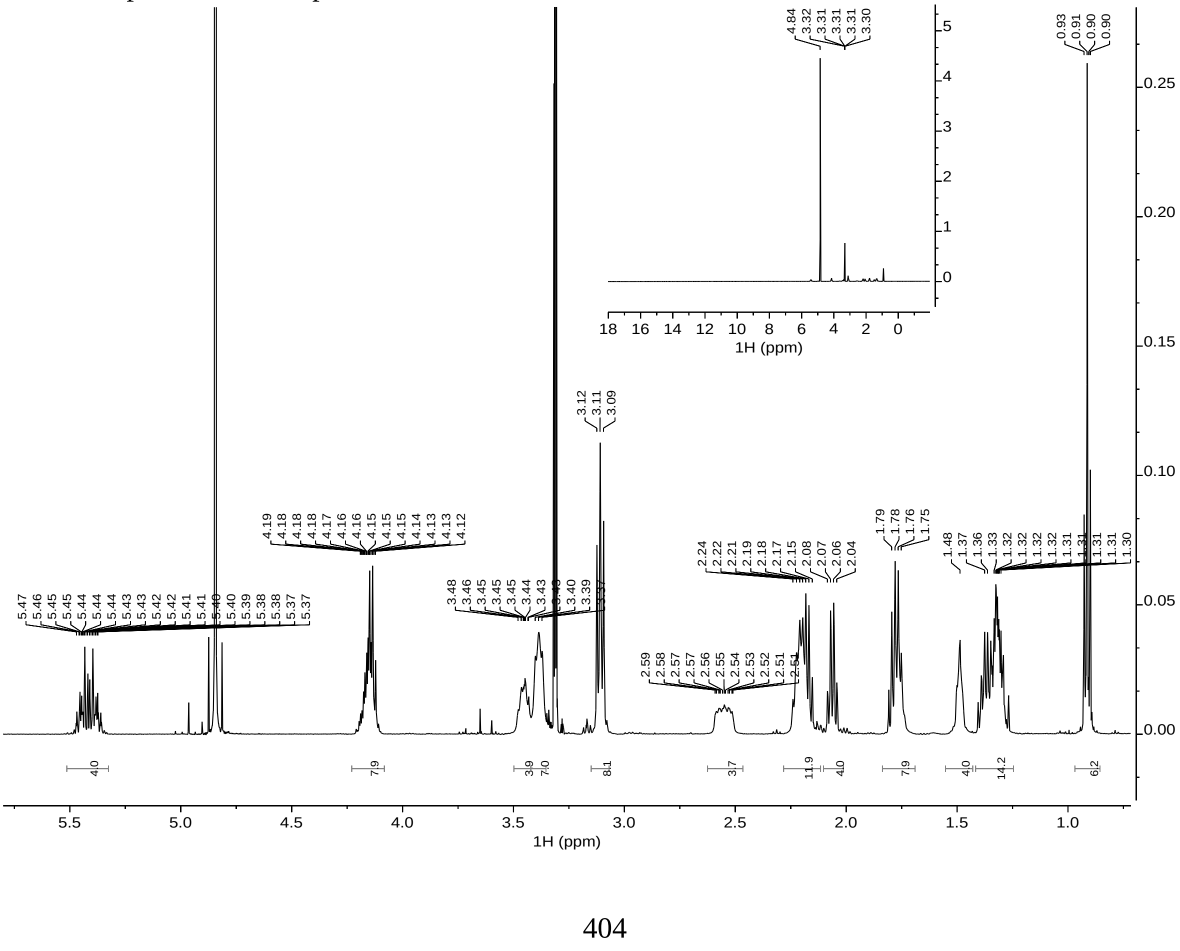

^{13}C NMR spectrum of compound **48**

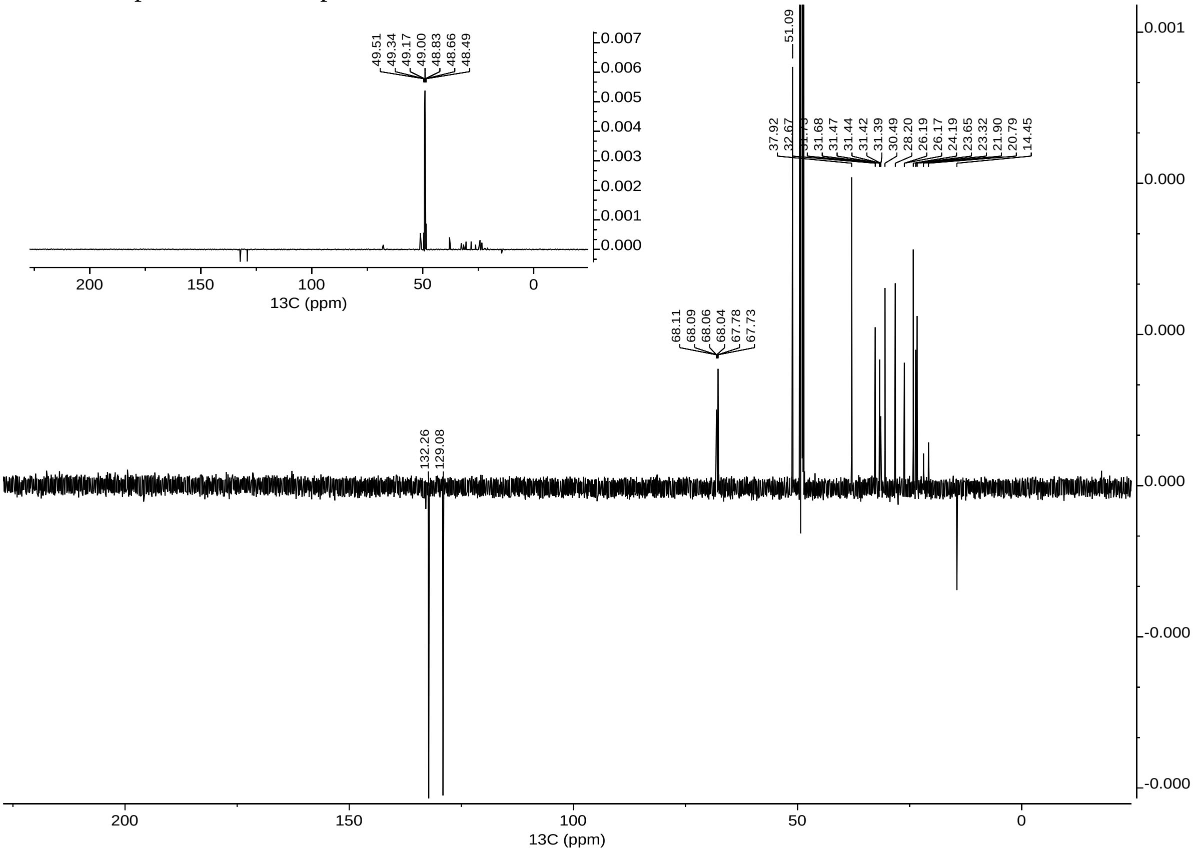

^{31}P{^{1}H} NMR spectrum of compound **48**

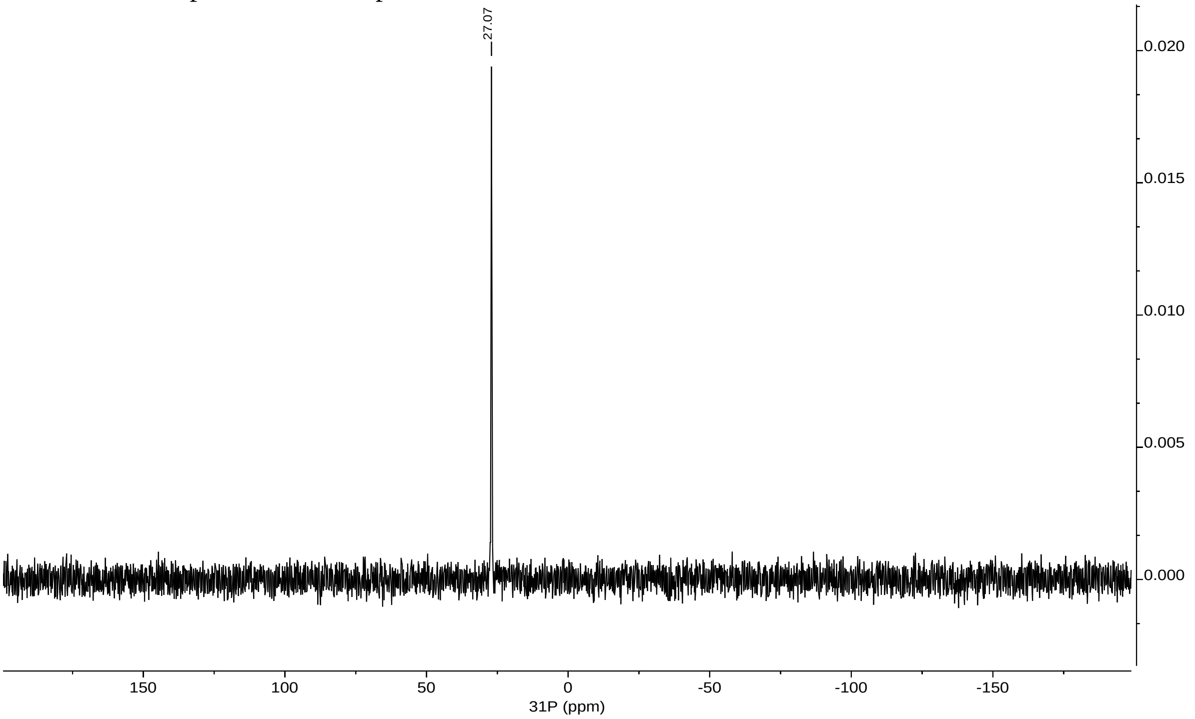

LC-MS chromatograms for compound **48**

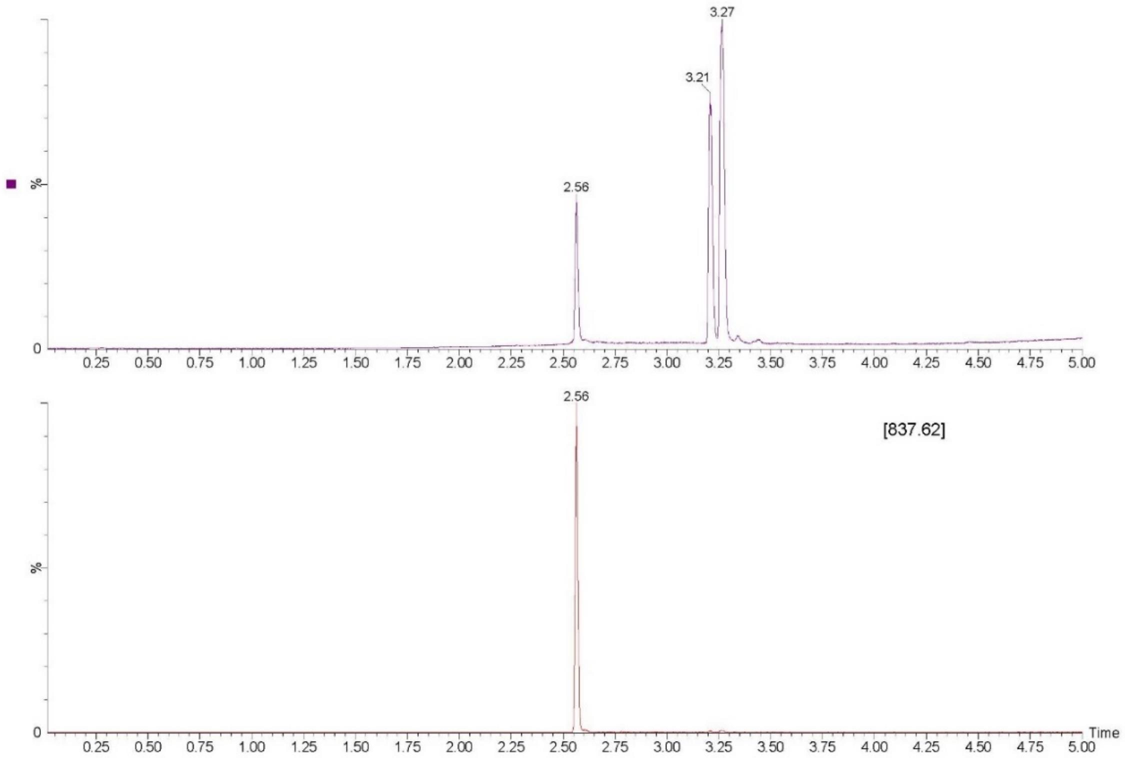

Bis(2-(adamantan-1-yl)ethyl) hexane-1,6-diyl bis((2-(bis(3-aminopropyl)amino)ethyl)phosphonate) hexahydrochloride (49)

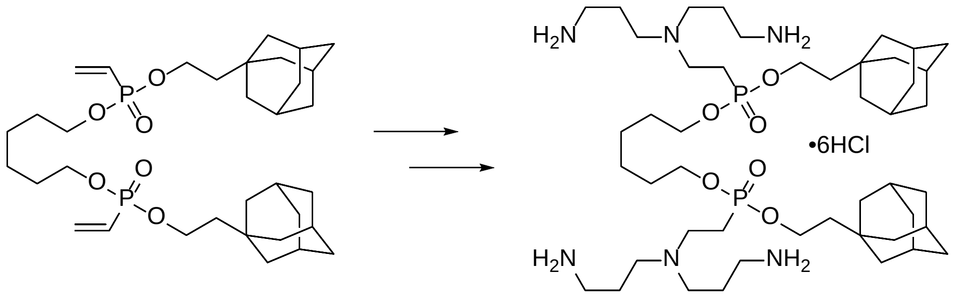

The title compound was prepared according to general methods **D** and **E** from vinylphosphonate dimer **S64** (2.58 g,4.14 mmol) in 54% overall yield (2.45 g, 2.22 mmol) as a white solid.
Mixture of diastereoisomers.

^{1}H NMR (401 MHz, CD_3OD): 4.25–4.09 (m, 8H, OC*H*$_2$), 3.46 (dd, 4H, *J* = 10.7, 5.9 Hz, PCH$_2$C*H*$_2$), 3.44–3.36 (m, 8H, C*H*$_2$(CH$_2$)$_2$NH$_2$), 3.11 (t, 8H, *J* = 7.5 Hz, C*H*$_2$NH$_2$), 2.64–2.48 (m, 4H, PC*H*$_2$), 2.27–2.15 (m, 8H, C*H*$_2$CH$_2$NH$_2$), 1.96 (p, 6H, *J* = 3.2 Hz, C*H*), 1.81–1.65 (m, 16H, C$_{quat}$C*H*$_2$CH, OCH$_2$C*H*$_2$CH$_2$), 1.59 (d, 12H, *J* = 2.9 Hz, *c*-(CHC*H*$_2$)$_3$), 1.57–1.45 (m, 8H, O(CH$_2$)$_2$C*H*$_2$, OCH$_2$C*H*$_2$C$_{quat}$).

^{13}C NMR (101 MHz, CD_3OD): 68.09 (d, *J* = 4.6 Hz), 68.06 (d, *J* = 3.9 Hz, O*C*H$_2$(CH$_2$)$_2$), 64.62 (d, *J* = 6.7 Hz, O*C*H$_2$CH$_2$C$_{quat}$), 51.04 (*C*H$_2$(CH$_2$)$_2$NH$_2$), 48.87 (d, *J* = 2.3 Hz, PCH$_2$*C*H$_2$), 45.55 (d, *J* = 5.7 Hz, OCH$_2$*C*H$_2$C$_{quat}$), 43.62 (*c*-(CH*C*H$_2$)$_3$), 38.02 (C$_{quat}$*C*H$_2$CH), 37.88 (*C*H$_2$NH$_2$), 32.92 (*C*$_{quat}$), 31.42 (d, *J* = 5.5 Hz), 31.40 (d, *J* = 5.1 Hz, OCH$_2$*C*H$_2$CH$_2$), 30.04 (*C*H), 26.22 (O(CH$_2$)$_2$*C*H$_2$), 23.26 (*C*H$_2$CH$_2$NH$_2$), 21.35 (d, *J* = 139.2 Hz), 21.33 (d, *J* = 140.2 Hz, P*C*H$_2$).

^{31}P{^{1}H} NMR (162 MHz, CD_3OD): 28.74.

IR ν_{max} (KBr) 2921 (vs), 2904 (vs), 2848 (vs), 2750 (s, br, sh), 2660 (s, br, sh), 2560 (m, br, sh), 2059 (w, vbr), 1507 (w, br), 1451 (m), 1345 (w), 1230 (m, vbr), 1099 (w), 1045 (m), 1010 (br, sh), 997 (s, vbr), 973 (m, sh), 813 (w).

HR-MS(ESI$^+$): For $C_{46}H_{91}N_6O_6P_2$ $(M+H)^+$ *m/z* calculated 885.64698, found 885.64665.

^{1}H NMR spectrum of compound **49**

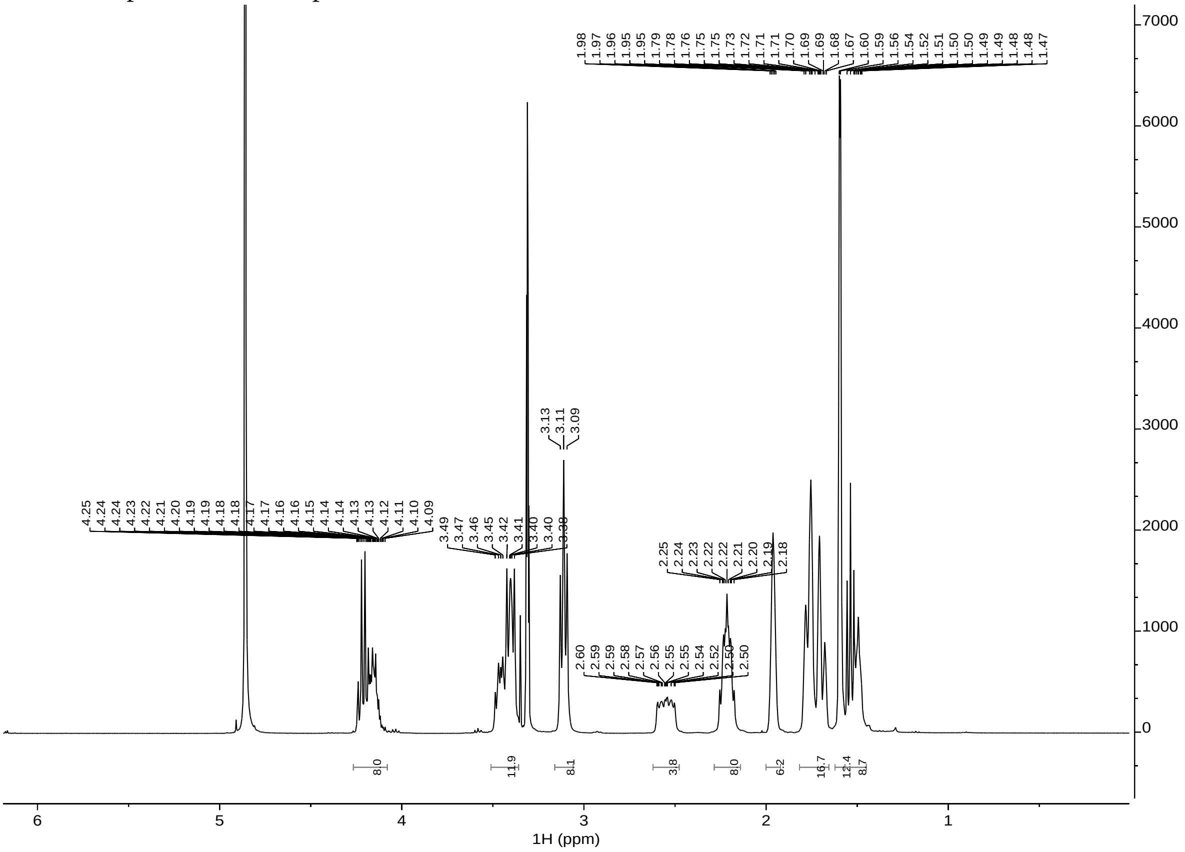

^{13}C NMR spectrum of compound **49**

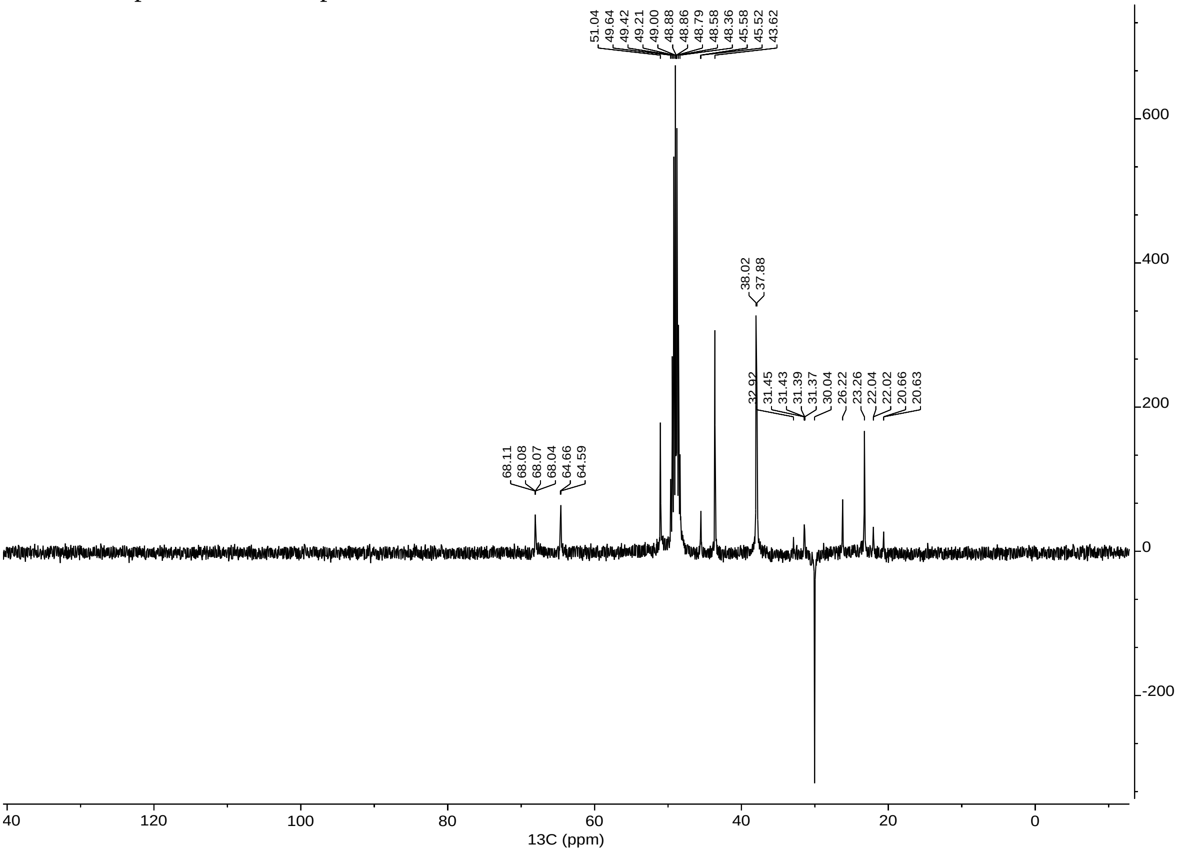

$^{31}P\{^{1}H\}$ NMR spectrum of compound **49**

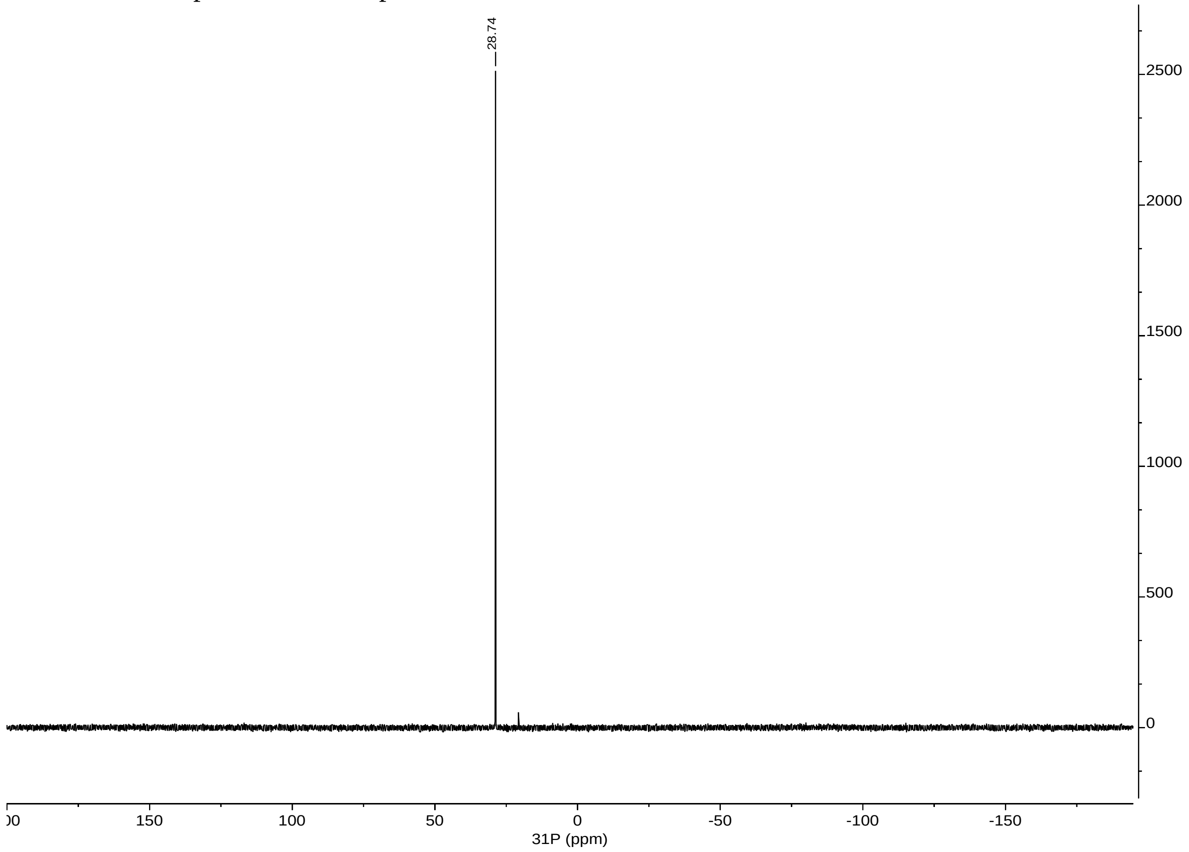

LC-MS chromatograms for compound **49**

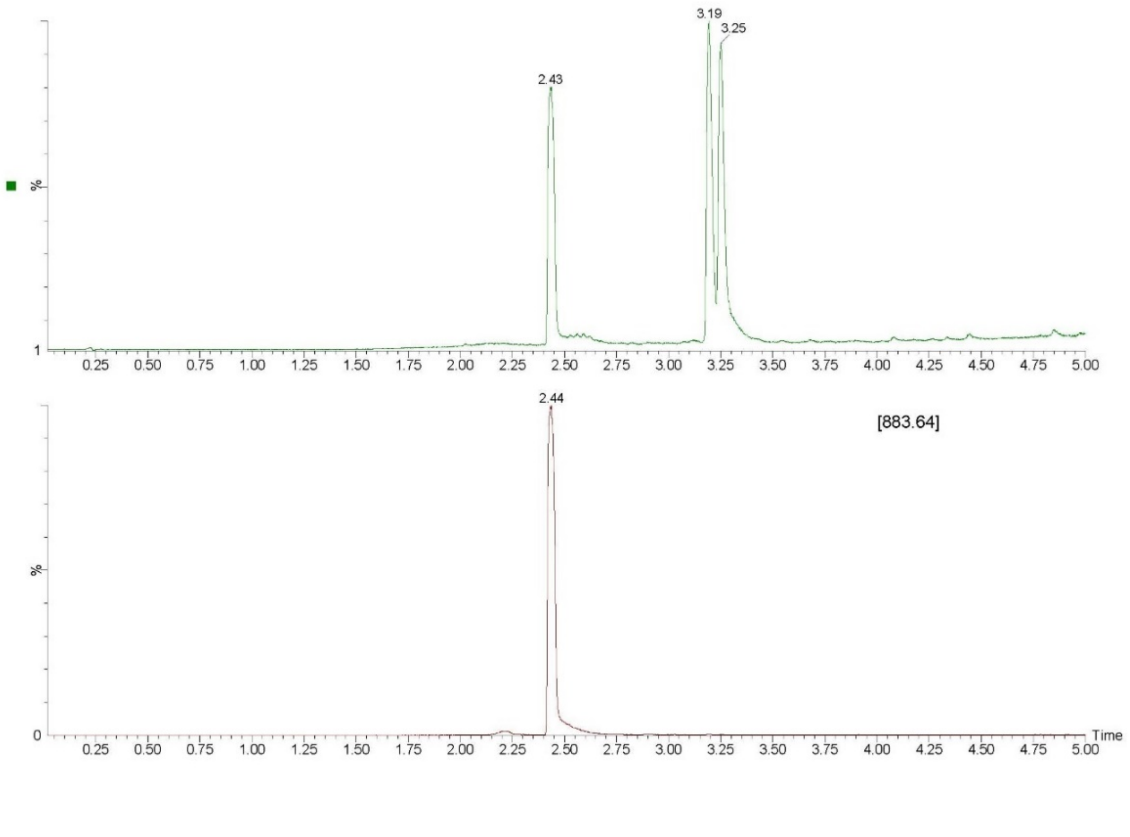

Heptane-1,7-diyl bis(4,4,4-trifluorobutyl) bis((2-(bis(3-aminopropyl)amino)ethyl)phosphonate) hexahydrochloride (50)

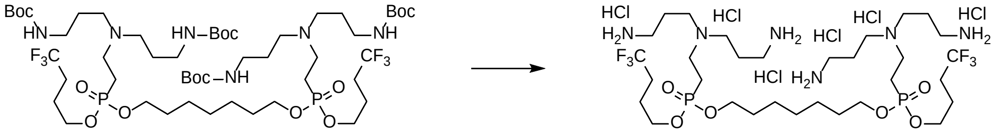

The title compound was prepared according to general method **E** from Boc derivative **S107** (0.79 g, 0.66 mmol) in 98% yield (0.66 g, 0.65 mmol) as a white solid.

^{1}H NMR (500 MHz, CD_3OD) δ 4.27–4.10 (m, 8H, C*H*$_2$O), 3.51–3.43 (m, 4H, PCH$_2$C*H*$_2$), 3.43–3.36 (m, 8H, C*H*$_2$(CH$_2$)$_2$NH$_2$), 3.10 (t, *J* = 7.5 Hz, 8H, C*H*$_2$NH$_2$), 2.63–2.52 (m, 4H, PC*H*$_2$), 2.41–2.28 (m, 4H, C*H*$_2$CF$_3$), 2.26–2.16 (m, 8H, C*H*$_2$CH$_2$NH$_2$), 2.03–1.94 (m, 4H, C*H*$_2$CH$_2$CF$_3$), 1.79–1.70 (m, 4H, OCH$_2$C*H*$_2$(CH$_2$)$_3$C*H*$_2$CH$_2$O), 1.51–1.37 (m, 6H, O(CH$_2$)$_2$(C*H*$_2$)$_3$(CH$_2$)$_2$O).

^{13}C NMR (126 MHz, CD_3OD) δ 128.66 (q, *J* = 275.2 Hz, *C*F$_3$), 68.38 (d, *J* = 6.8 Hz, O*C*H$_2$(CH$_2$)$_5$*C*H$_2$O), 66.41 (d, *J* = 6.4 Hz, O*C*H$_2$(CH$_2$)$_2$CF$_3$), 51.06 (PCH$_2$*C*H$_2$), 37.87 (*C*H$_2$CH$_2$NH$_2$), 31.47 (d, *J* = 5.8 Hz, OCH$_2$*C*H$_2$(CH$_2$)$_2$), 30.94 (q, *J* = 29.2 Hz, *C*H$_2$CF$_3$), 29.74 (O(CH$_2$)$_3$*C*H$_2$), 26.48 (O(CH$_2$)$_2$*C*H$_2$CH$_2$), 24.46 (dq, *J* = 6.3, 3.1 Hz, *C*H$_2$CH$_2$CF$_3$), 23.28 (*C*H$_2$CH$_2$NH$_2$), 21.23 (d, *J* = 140.3 Hz, P*C*H$_2$).

^{31}P{^{1}H} NMR (202 MHz, CD_3OD) δ 27.67.

^{19}F NMR (377 MHz, CD_3OD): -67.78 (t, *J* = 11.2 Hz).

IR ν_{max} 3100–2500 (vs), 2944 (vs), 2864 (s), 2019 (w), 1605 (m), 1519 (m, sh), 1393 (m), 1340 (m), 1256 (s), 1154 (s), 1134 (m), 1100–1000 (vs), 990 (s).

HR-MS(ESI+): For $C_{31}H_{68}O_6N_6F_6P_2$ $(M+2H)^{2+}$ *m*/2*z* calculated 398.22844, found 398.22800.

^{1}H NMR spectrum of compound **50**

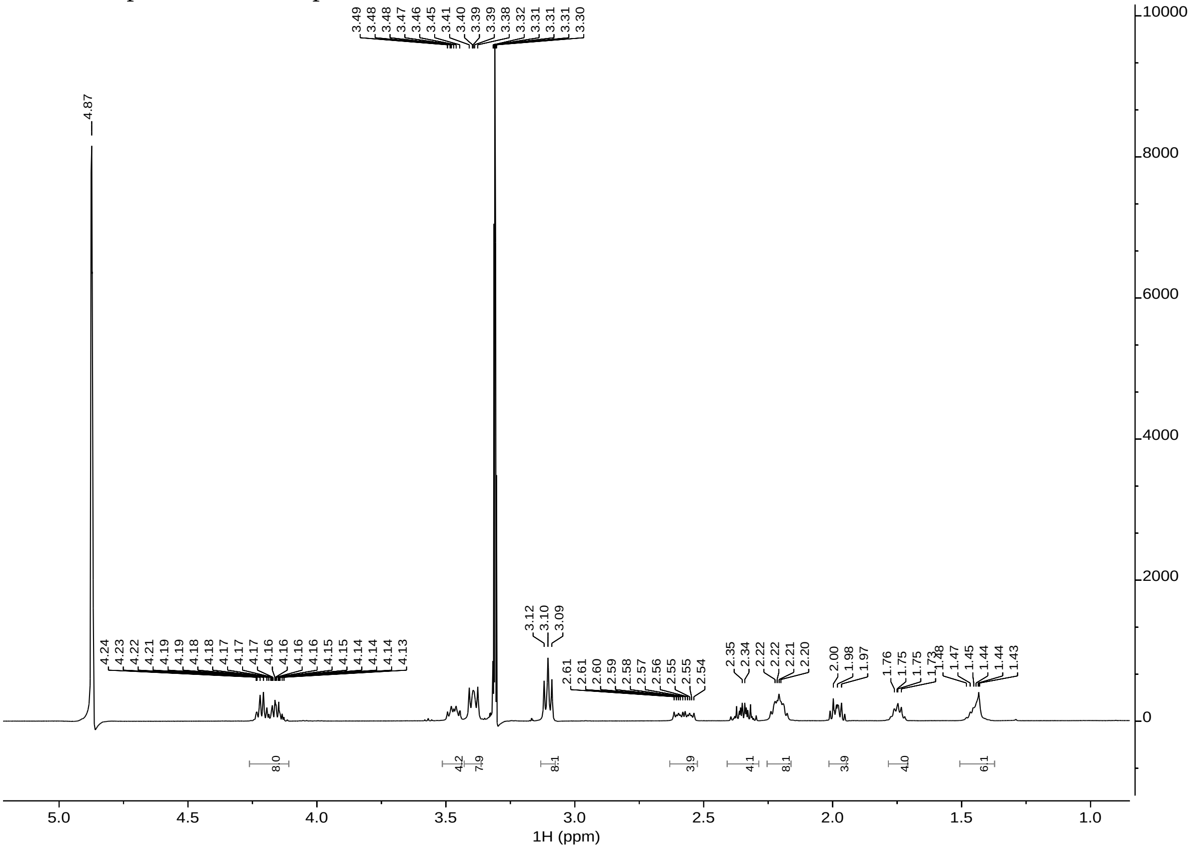

^{13}C NMR spectrum of compound **50**

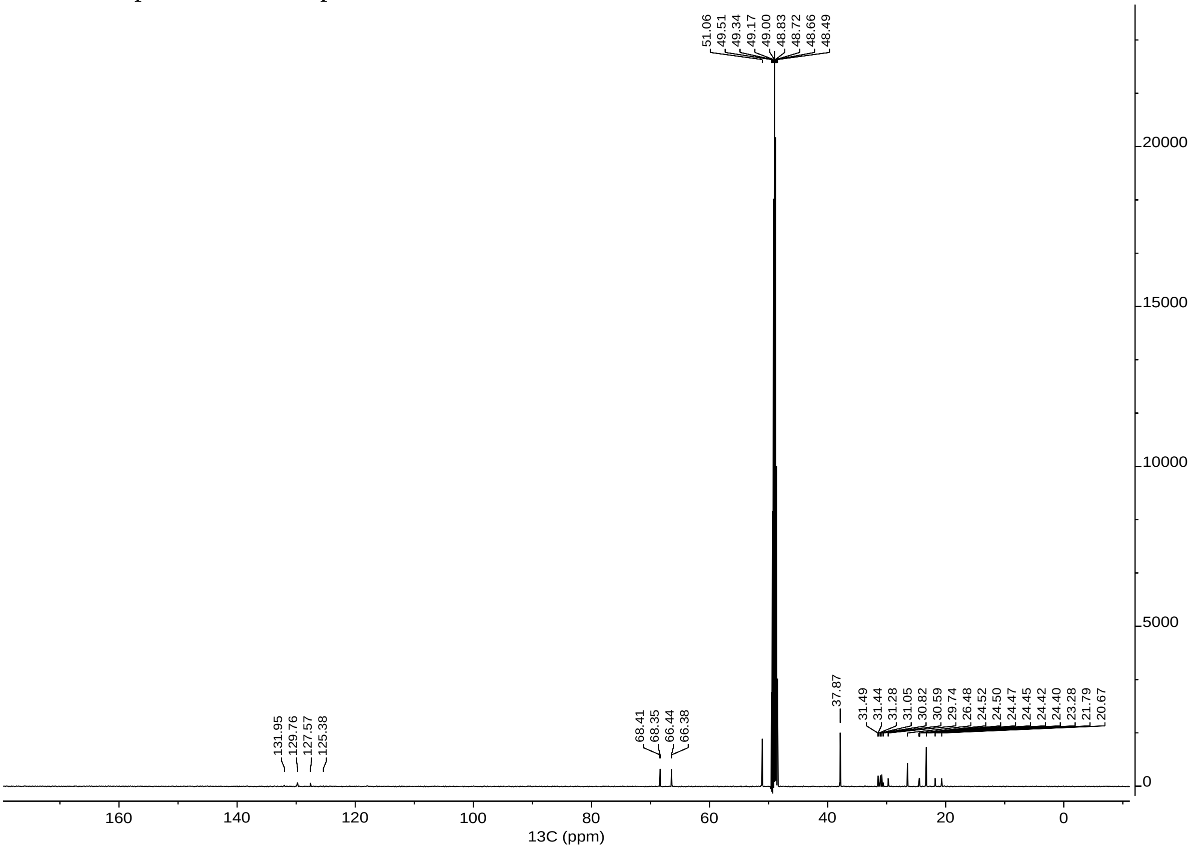

^{31}P{^{1}H} NMR spectrum of compound **50**

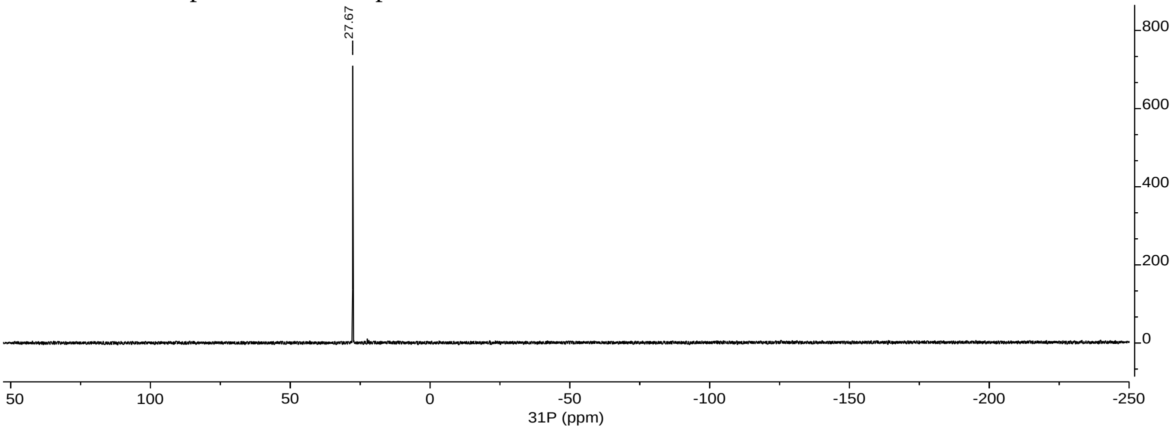

^{19}F NMR spectrum of compound **50**

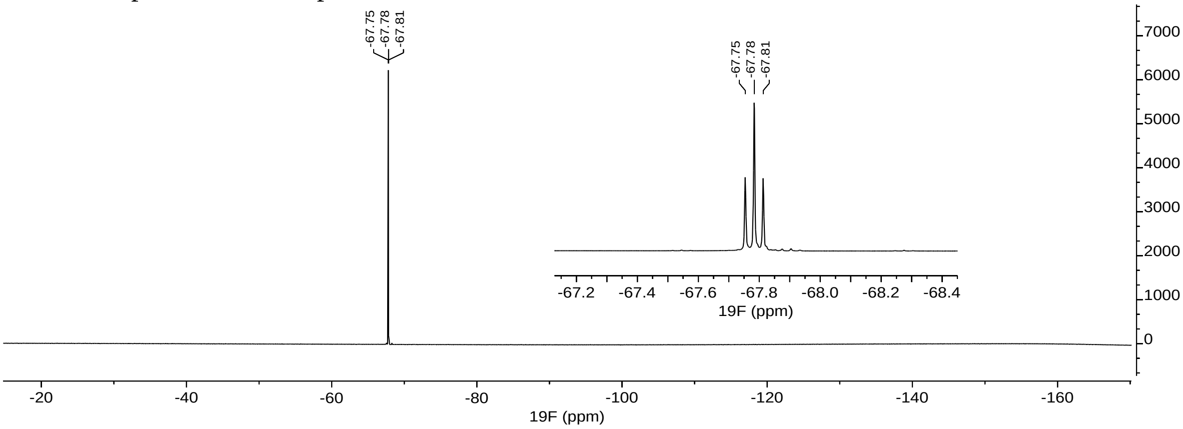

LC-MS chromatograms for compound **50**

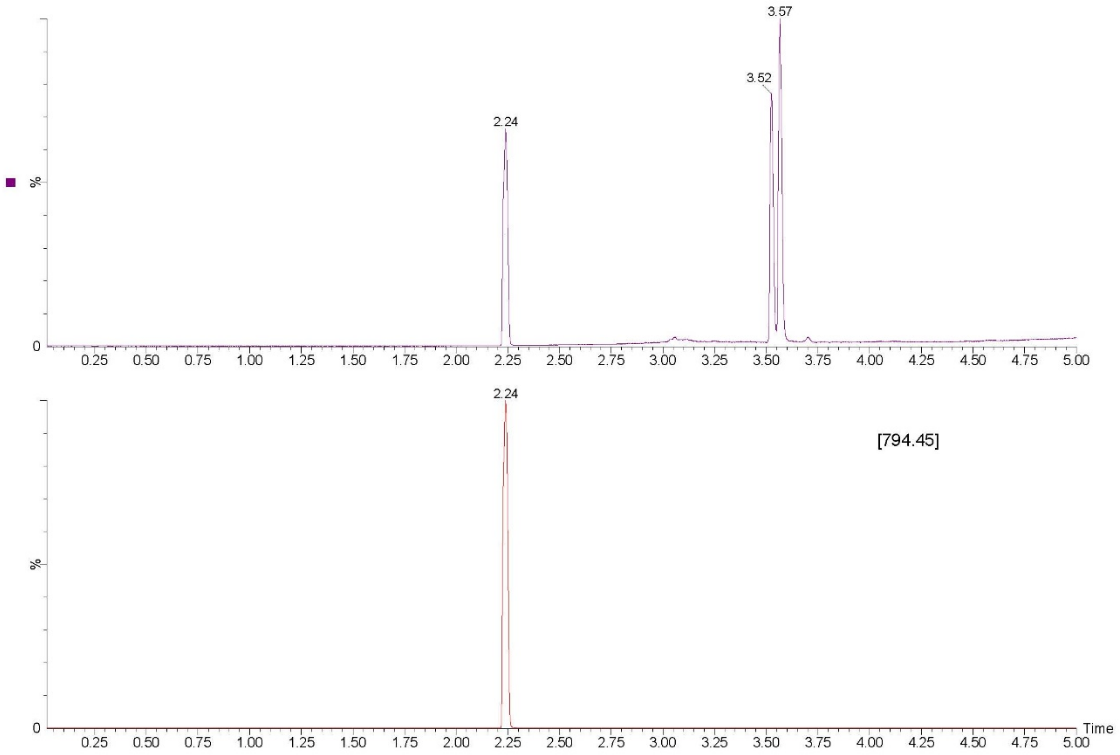

Heptane-1,7-diyl bis(4,4,4-trifluorobutyl) bis((2-(bis(3-guanidinopropyl)amino)ethyl)phosphonate) hexahydrochloride (51)

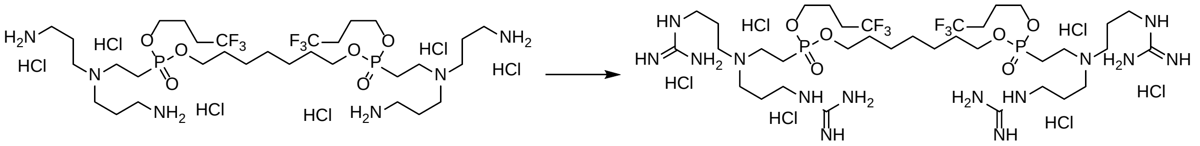

The title compound was prepared according to general method **F** from LPPO **50** (0.28 g, 0.28 mmol) in 64% yield (0.21 g, 0.18 mmol) as a white solid.

^{1}H NMR (401 MHz, CD_3OD): 4.26–4.09 (m, 8H, C*H*$_2$O), 3.51–3.42 (m, 4H, PCH$_2$C*H*$_2$), 3.42–3.32 (m, 16H, N(CH$_2$)$_2$C*H*$_2$NH, NC*H*$_2$(CH$_2$)$_2$NH), 2.68–2.52 (m, 4H, PC*H*$_2$CH$_2$N), 2.42–2.26 (m, 4H, CF$_3$C*H*$_2$(CH$_2$)$_2$O), 2.17–2.06 (m, 8H, NCH$_2$C*H*$_2$CH$_2$NH$_2$), 2.03–1.92 (m, 4H, CF$_3$CH$_2$C*H*$_2$CH$_2$O), 1.81–1.68 (m, 4H, OCH$_2$C*H*$_2$(CH$_2$)$_3$C*H*$_2$CH$_2$O), 1.50–1.39 (m, 6H, O(CH$_2$)$_2$(C*H*$_2$)$_3$(CH$_2$)$_2$O).

^{13}C NMR (101 MHz, CD_3OD): 158.65 (*C*=NH), 128.65 (q, *J* = 275.3 Hz, *C*F$_3$), 68.31 (d, *J* = 6.7 Hz, O*C*H$_2$(CH$_2$)$_3$), 66.37 (d, *J* = 6.3 Hz, CF$_3$(CH$_2$)$_2$*C*H$_2$O), 51.51 (*C*H$_2$NH$_2$), 48.64 (PCH$_2$*C*H$_2$), 39.67 (*C*H$_2$(CH$_2$)$_2$NH), 31.42 (d, *J* = 6.2 Hz, OCH$_2$*C*H$_2$(CH$_2$)$_2$), 30.94 (q, *J* = 29.2 Hz, CF$_3$*C*H$_2$), 29.71 (O(CH$_2$)$_3$*C*H$_2$), 26.47 (O(CH$_2$)$_2$*C*H$_2$CH$_2$), 24.74 (*C*H$_2$CH$_2$NH$_2$), 24.44 (dq, *J* = 6.2, 3.1 Hz, CF$_3$CH$_2$*C*H$_2$), 21.29 (d, *J* = 140.2 Hz, P*C*H$_2$).

^{31}P{^{1}H} NMR (162 MHz, CD_3OD): 29.13.

^{19}F NMR (377 MHz, CD_3OD): -67.70 (t, *J* = 11.1 Hz).

IR ν_{max} 3387 (s, vbr), 3159 (s, br), 2955 (m), 2859 (w), 2709 (w, vbr), 2597 (w, br), 2495 (w, br), 1671 (vs), 1645 (vs), 1620 (s), 1390 (m), 1341 (m), 1257 (s), 1236 (s), 1154 (s), 1132 (s), 1020 (s, br).

HR-MS(ESI$^+$): For $C_{35}H_{75}O_6N_{14}F_6P_2$ (M+H)$^+$ *m/z* calculated 963.53679, found 963.53701.

^{1}H NMR spectrum of compound **51**

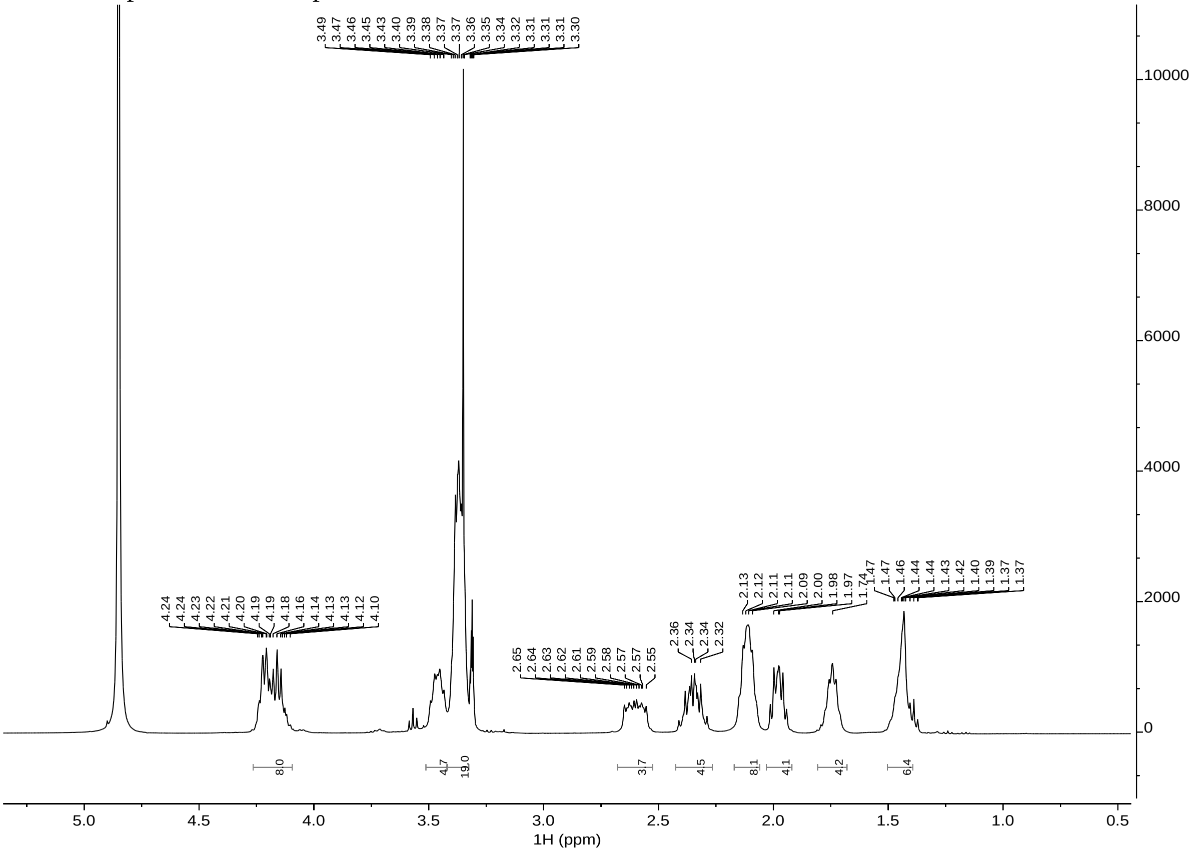

^{13}C NMR spectrum of compound **51**

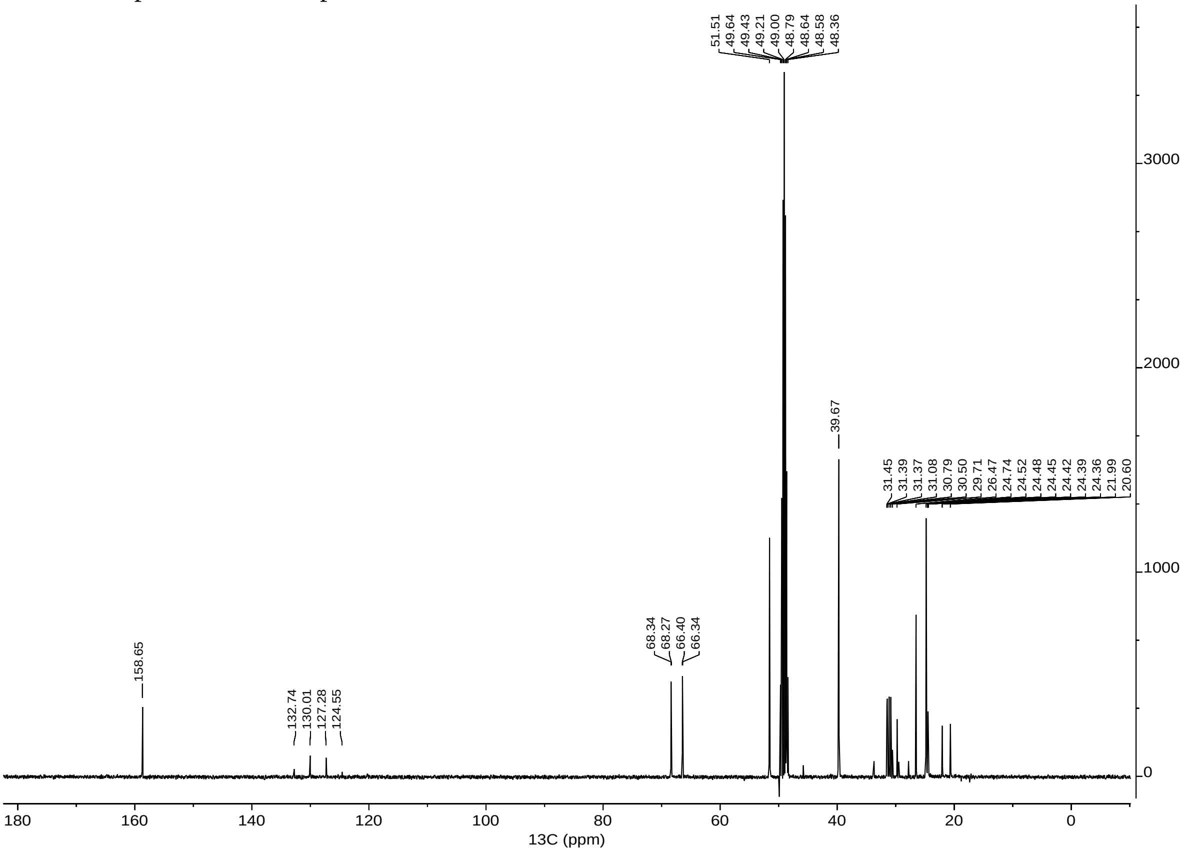

^{31}P{^{1}H} NMR spectrum of compound **51**

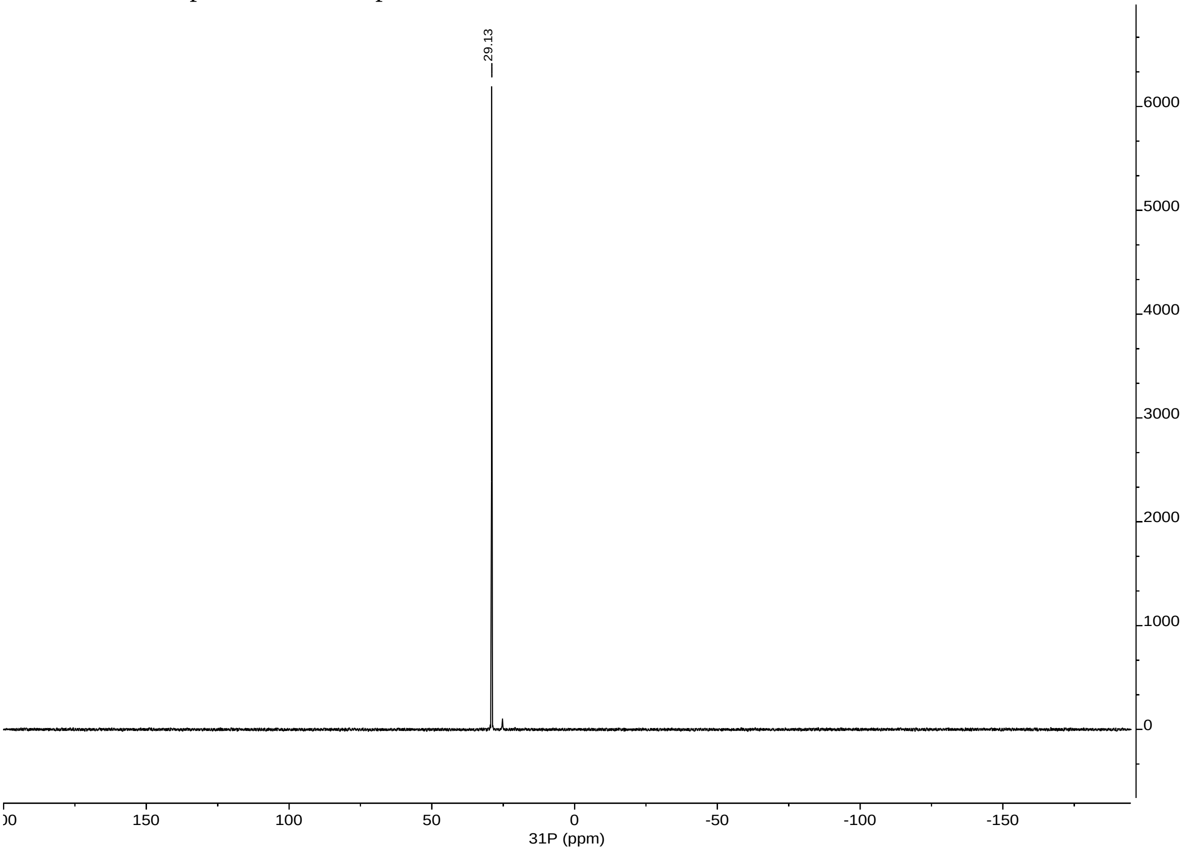

^{19}F NMR spectrum of compound **51**

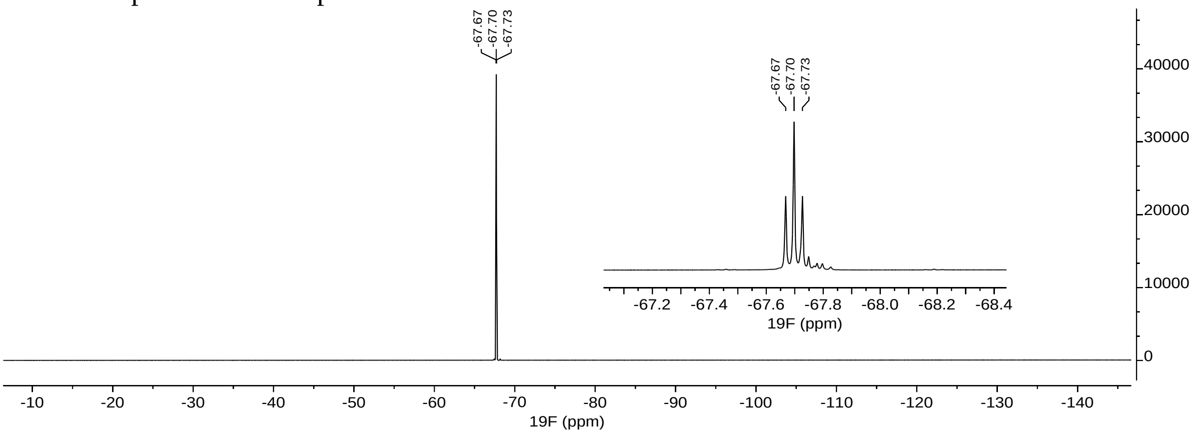

LC-MS chromatograms for compound **51**

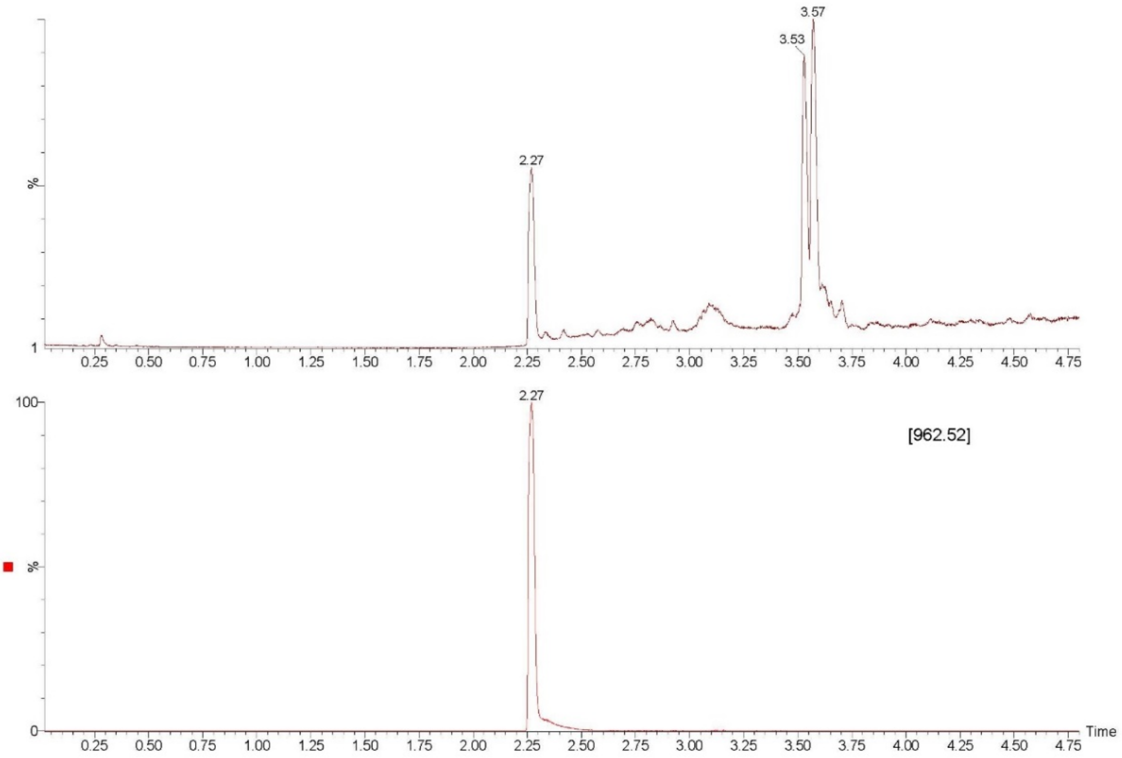

Heptane-1,7-diyl diheptyl bis((2-(bis(3-aminopropyl)amino)ethyl)phosphonate) hexahydrochloride (52)

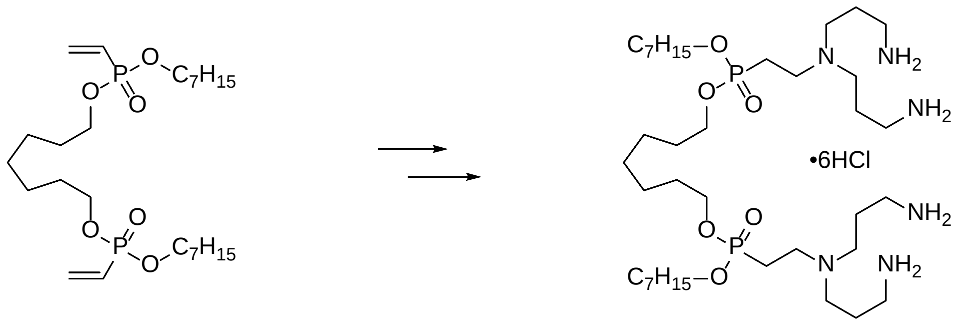

The title compound was prepared according to general methods **D** and **E** from vinylphosphonate dimer **S66** (190 mg, 374 µmol) in 78% overall yield (290 mg, 293 µmol) as a white solid.

^{1}H NMR (401 MHz, CD_3OD) δ 4.13 (q, *J* = 7.0 Hz, 8H, OC*H*$_2$), 3.43 (t, *J* = 6.5 Hz, 4H, PCH$_2$C*H*$_2$), 3.40–3.34 (m, 8H, P(CH$_2$)$_2$NC*H*$_2$), 3.09 (t, *J* = 7.5 Hz, 8H, C*H*$_2$NH$_2$), 2.50 (dtd, *J* = 16.8, 8.3, 4.8 Hz, 4H, PC*H*$_2$), 2.25–2.14 (m, 8H, C*H*$_2$CH$_2$NH$_2$), 1.78–1.67 (m, 8H, OCH$_2$C*H*$_2$), 1.49–1.25 (m, 22H), 0.97–0.87 (m, 6H, C*H*$_3$).

^{13}C NMR (101 MHz, CD_3OD) δ 68.26 (d, *J* = 6.7 Hz), 68.22 (d, *J* = 6.5 Hz, O*C*H$_2$), 51.10 (*C*H$_2$(CH$_2$)$_2$NH$_2$), 48.72 (PCH$_2$*C*H$_2$), 37.86 (*C*H$_2$NH$_2$), 32.93 (*C*H$_2$CH$_2$CH$_3$), 31.62 (d, *J* = 6.1 Hz), 31.55 (d, *J* = 6.1 Hz, OCH$_2$*C*H$_2$), 30.11, 29.98, 27.20, 26.60 (O(CH$_2$)$_2$(*C*H$_2$)$_2$), 23.67 (*C*H$_2$CH$_3$), 23.32 (*C*H$_2$CH$_2$NH$_2$), 21.19 (d, *J* = 140.9 Hz, P*C*H$_2$), 14.42 (*C*H$_3$).

^{31}P{^{1}H} NMR (162 MHz, CD_3OD) δ 28.68.

IR ν_{max} (film) ~3000–2500 (m–s, vbr), 2954 (vs), 2931 (vs), 2858 (s), 2748 (m), 2633 (m), 2559 (w), 2021 (w, vbr), 1602 (w), 1467 (m), 1395 (w), 1378 (w), 1251 (m), 1227 (m), 1063 (m), 1004 (s), 726 (w).

HR-MS(ESI$^+$): For $C_{37}H_{85}O_6N_6P_2$ $(M+H)^+$ *m*/*z* calculated 771.60003, found 771.59984. For $C_{37}H_{86}O_6N_6P_2$ $(M+2H)^{2+}$ *m*/2*z* calculated 386.30366, found 386.30356.

^{1}H NMR spectrum of compound **52**

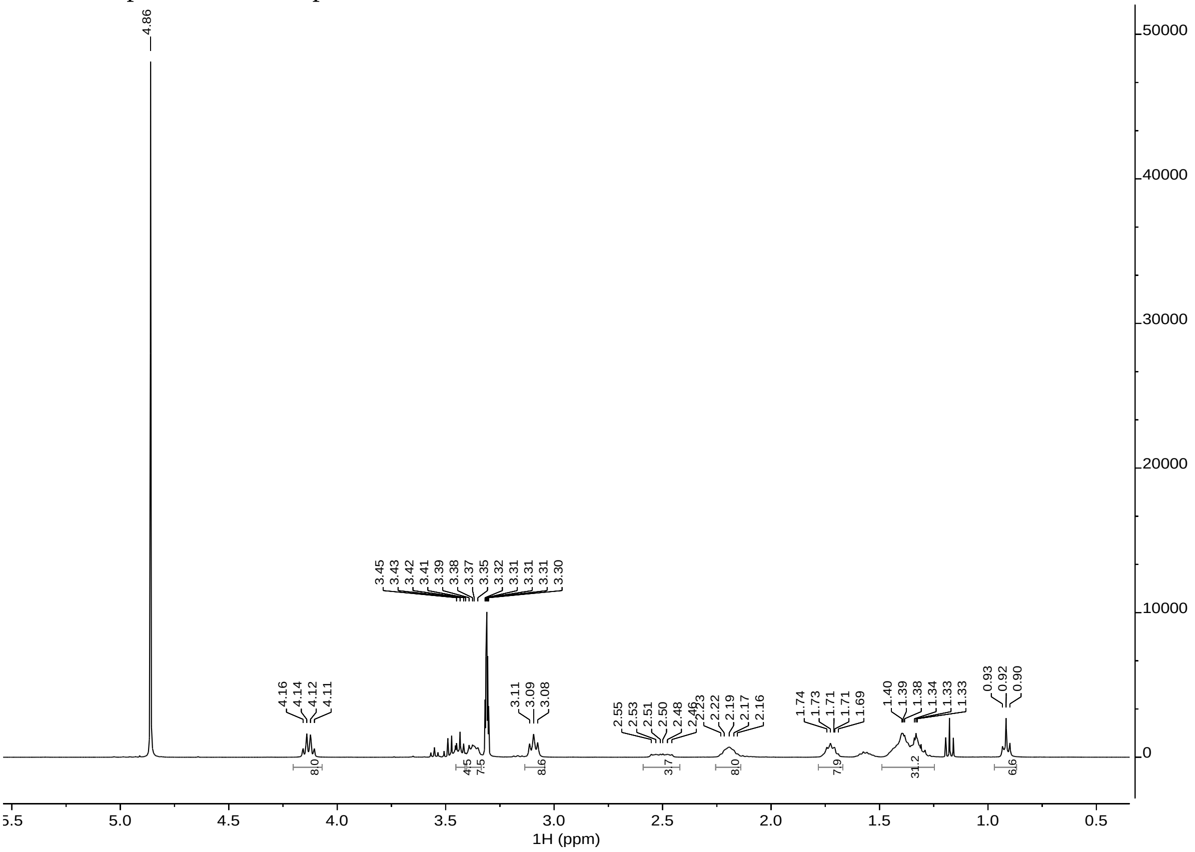

^{13}C NMR spectrum of compound **52**

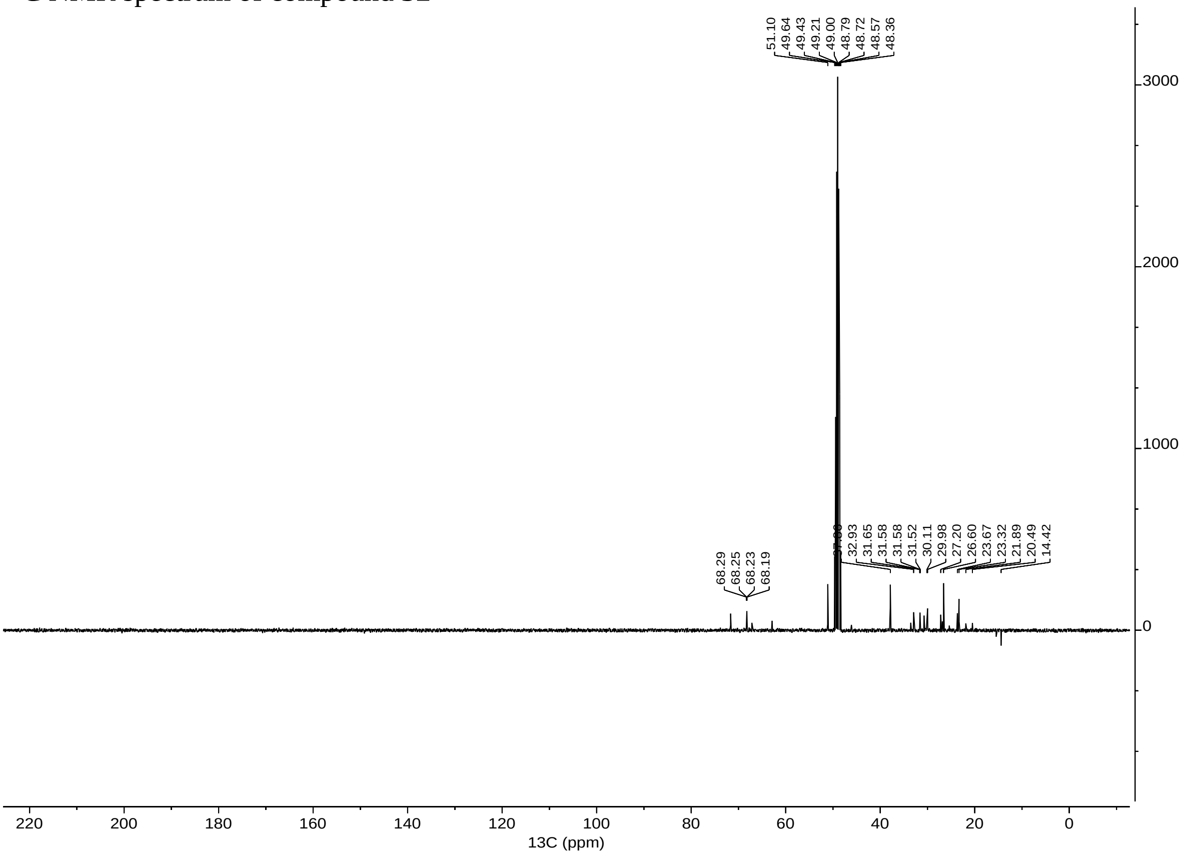

$^{31}P\{^{1}H\}$ NMR spectrum of compound **52**

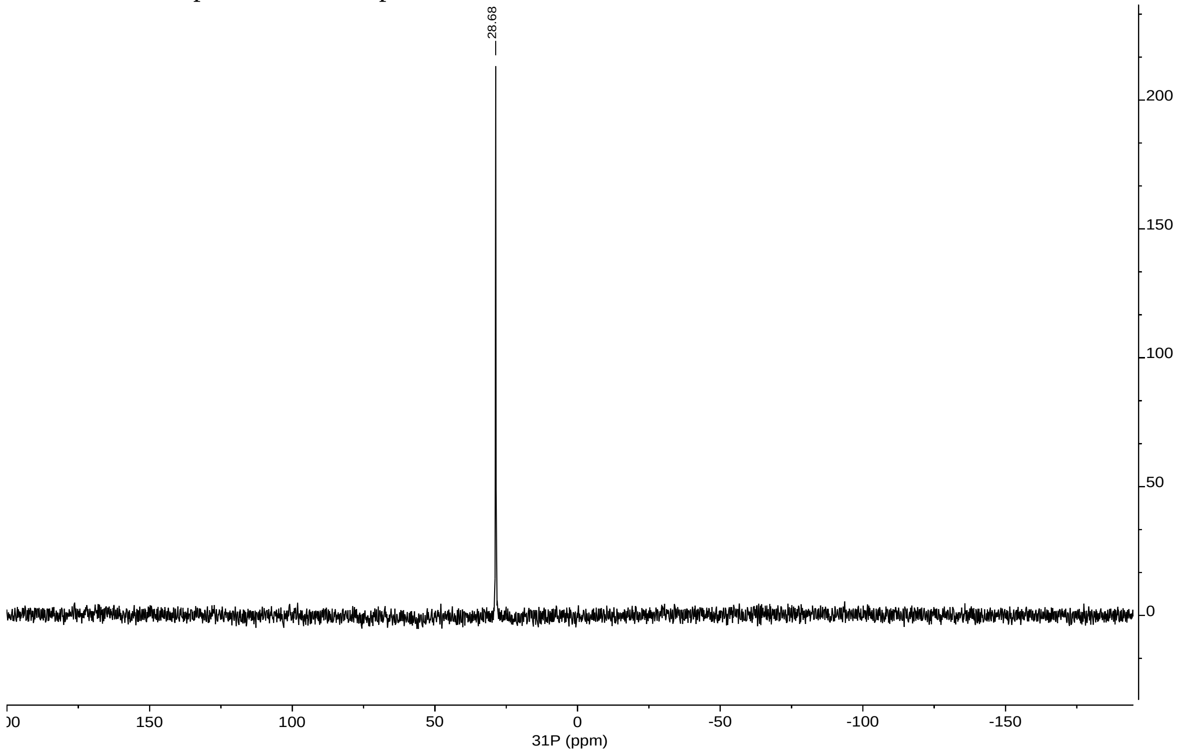

LC-MS chromatograms for compound **52**

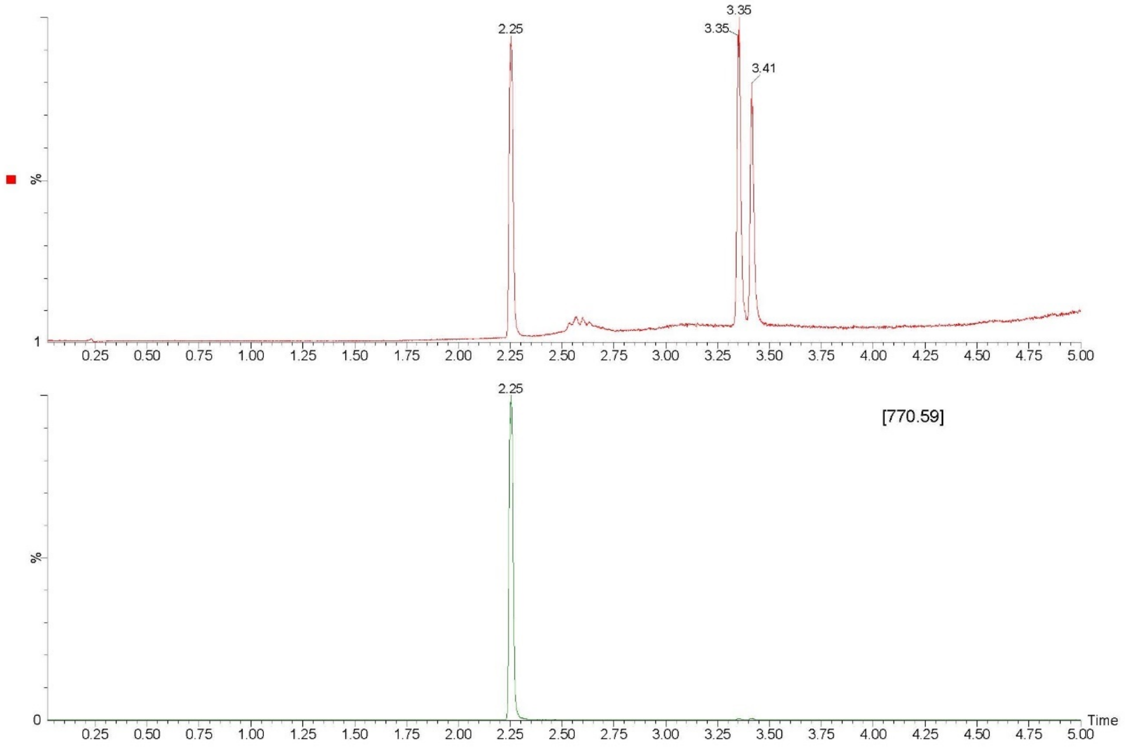

Bis((*Z*)-hept-3-en-1-yl) heptane-1,7-diyl bis((2-(bis(3-aminopropyl)amino)ethyl)phosphonate) hexahydrochloride (53)

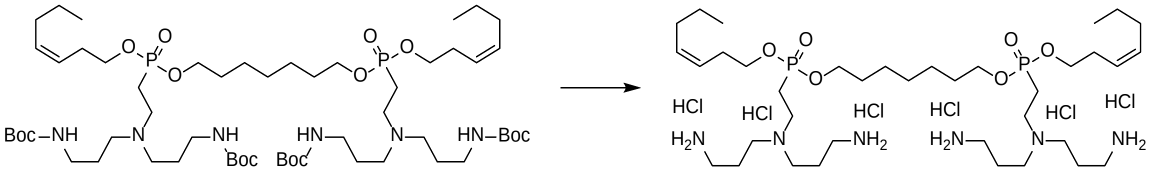

The title compound was prepared according to general method **E** from Boc derivative **S108** (0.55 g, 0.47 mmol) in 94% yield (0.43 g, 0.44 mmol) as a white solid.
Mixture of diastereoisomers.
^{1}H NMR (401 MHz, CD_3OD): 5.61–5.50 (m, 2H, $CH_3(CH_2)_2CH$), 5.49–5.37 (m, 2H, $CH(CH_2)_2O$), 4.21–4.07 (m, 8H, CH_2O), 3.52–3.35 (m, 12H, CH_2N), 3.12 (t, 8H, J = 7.3 Hz, CH_2NH_2), 2.65–2.44 (m, 8H, $CHCH_2CH_2O$, PCH_2), 2.28–2.17 (m, 8H, $CH_2CH_2NH_2$), 2.07 (qd, 4H, J = 7.4, 1.4 Hz, $CH_3CH_2CH_2$), 1.78–1.68 (m, 4H, $CH_2CH_2CH_2O$), 1.50–1.34 (m, 10H, CH_3CH_2, $(CH_2)_2(CH_2)_2O$), 0.93 (t, 6H, J = 7.4 Hz, CH_3).
^{13}C NMR (101 MHz, CD_3OD): 134.07 ($CH_3(CH_2)_2CH$), 125.22 ($CH(CH_2)_2O$), 68.14 (d, J = 6.8 Hz, $(CH_2)_2CH_2O$), 67.58 (d, J = 6.7 Hz, $CHCH_2CH_2O$), 51.00 ($CH_2(CH_2)_2NH_2$), 48.86 (PCH_2CH_2), 37.88 (CH_2NH_2), 31.43 (d, J = 6.0 Hz), 31.42 (d, J = 6.0 Hz, $CH_2CH_2CH_2O$), 30.41 ($CH_3CH_2CH_2$), 29.73, 29.71 ($CH_2(CH_2)_3O$), 29.64 (d, J = 6.0 Hz, $CHCH_2CH_2O$), 26.48, 26.47 ($CH_2(CH_2)_2O$), 23.75 (CH_3CH_2), 23.18 (CH_2CH_2NH), 21.32 (d, J = 140.2 Hz, PCH_2), 14.14 (CH_3).
^{31}P{^{1}H} NMR (162 MHz, CD_3OD): 28.63.
IR ν_{max} (KBr) 3100–2500 (vs, vbr), 3010 (s, sh), 2957 (vs), 2932 (s), 2871 (s), 2027 (w), 1652 (vw), 1604 (m), 1465 (m), 1379 (m), 1226 (m), 1003 (vs).
HR-MS(ESI$^+$): For $C_{37}H_{81}O_6N_6P_2$ (M+H)$^+$ m/z calculated 767.56873, found 767.65846.

^{1}H NMR spectrum of compound **53**

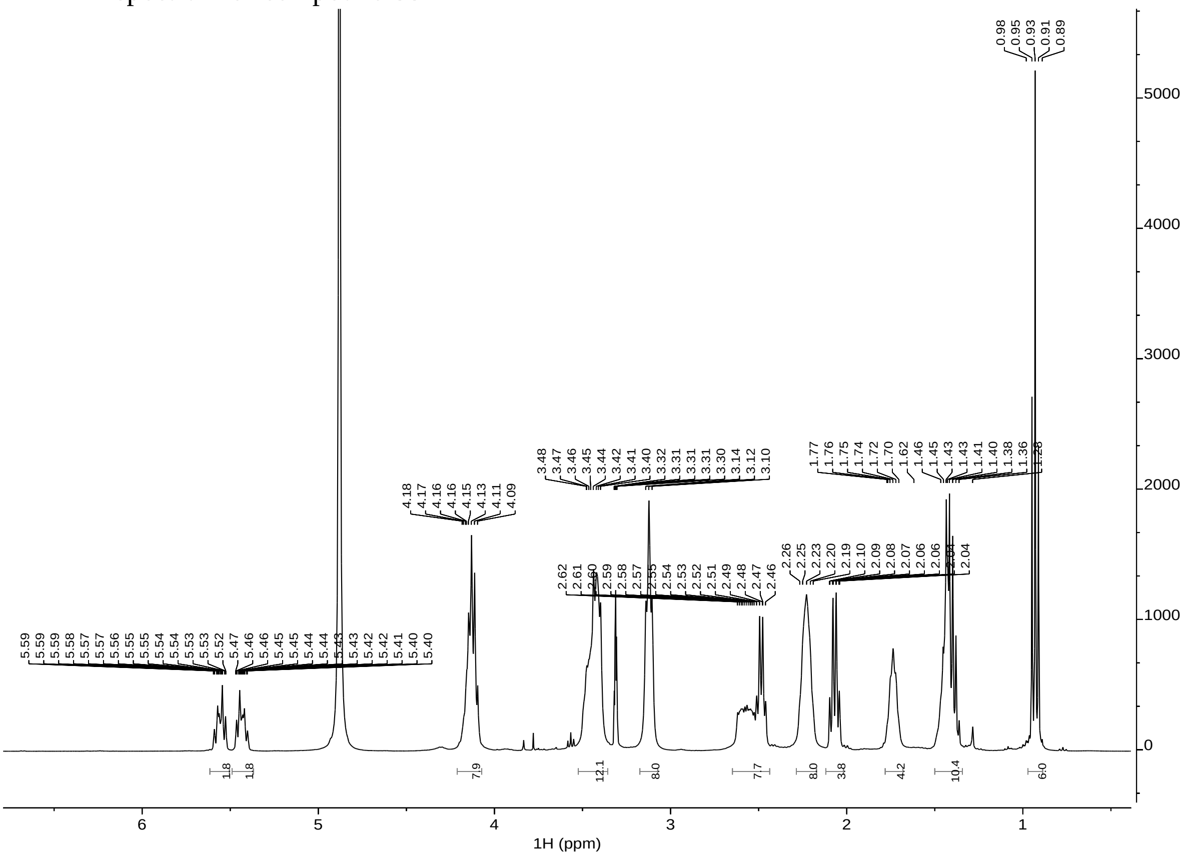

^{13}C NMR spectrum of compound **53**

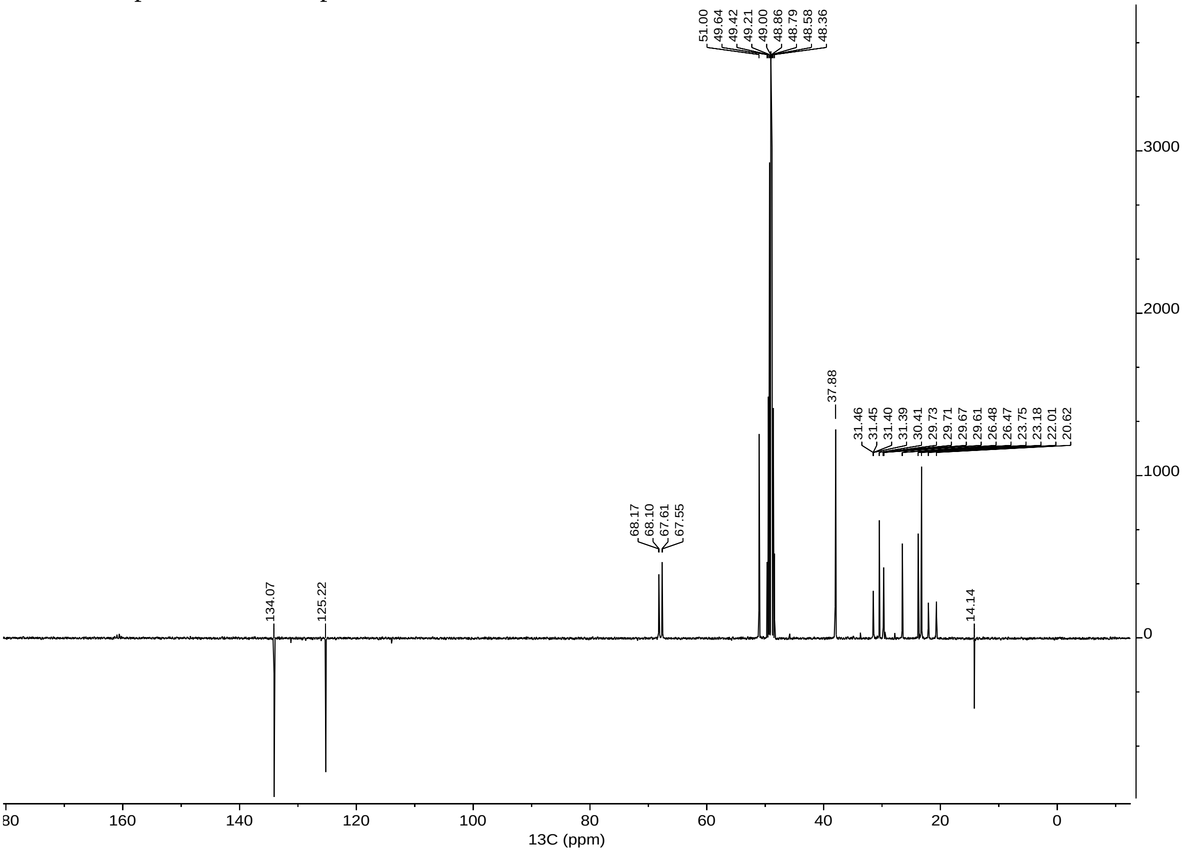

^{31}P{^{1}H} NMR spectrum of compound **53**

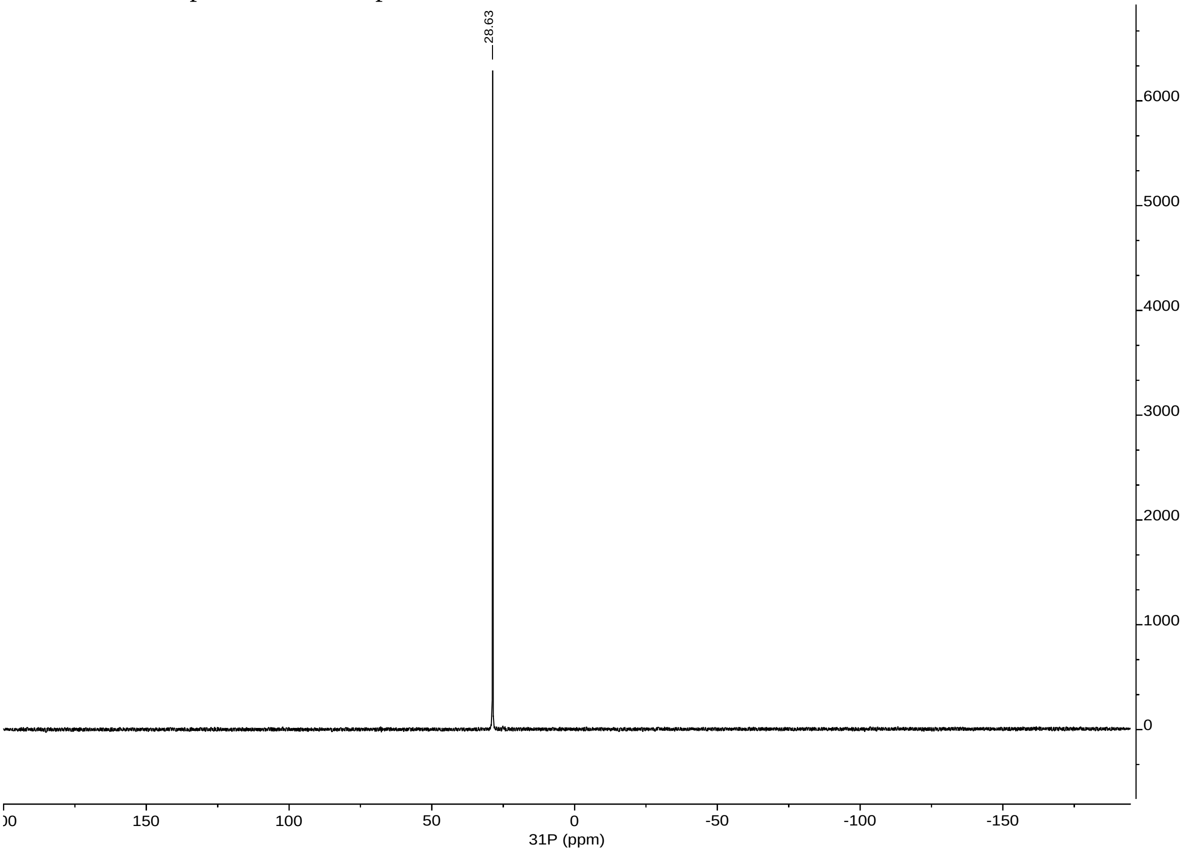

LC-MS chromatograms for compound **53**

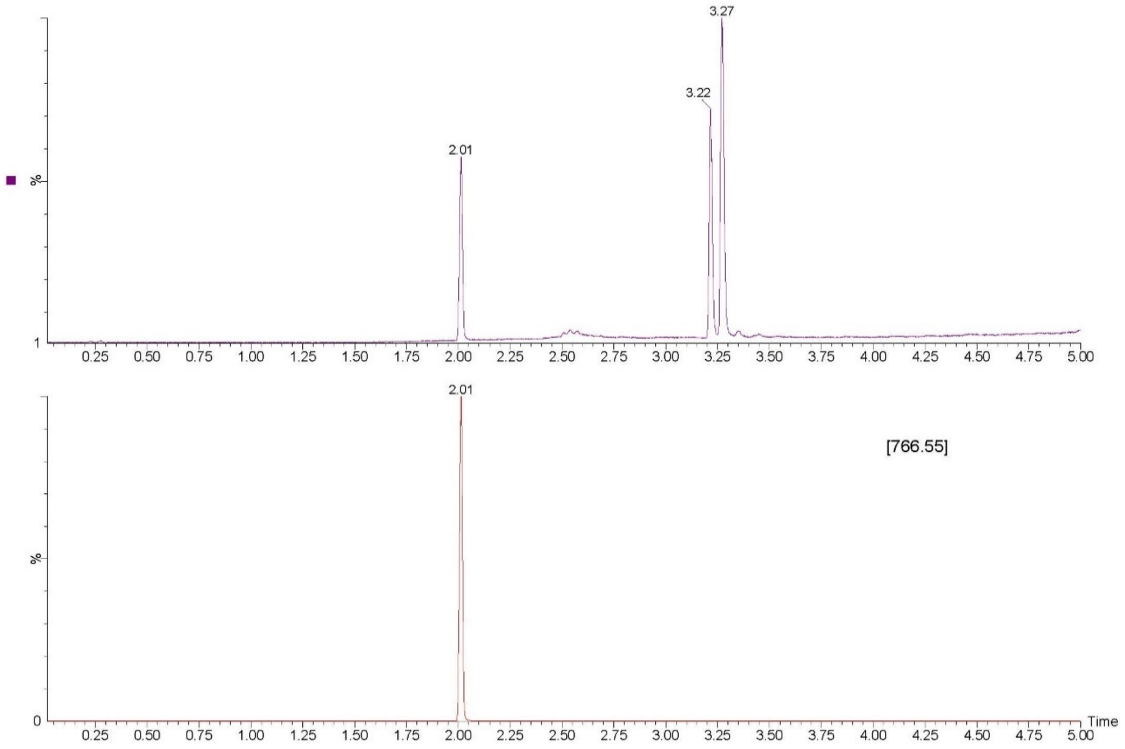

Bis((*Z*)-hept-3-en-1-yl) heptane-1,7-diyl bis((2-(bis(2-aminoethyl)amino)ethyl)phosphonate) hexahydrochloride (54)

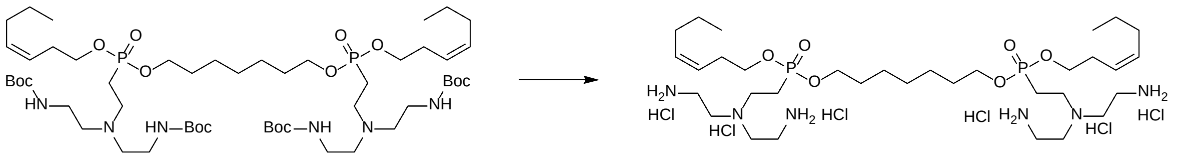

The title compound was prepared according to general method **E** from Boc derivative **S109** (0.30 g, 0.27 mmol) in 96% yield (0.24 g, 0.26 mmol) as a white solid.

Mixture of diastereoisomers.

^{1}H NMR (401 MHz, CD_3OD): 5.61–5.52 (m, 2H, $CH_3(CH_2)_2CH$), 5.48–5.38 (m, 2H, $CH(CH_2)_2O$), 4.19–4.06 (m, 8H, CH_2O), 3.40–3.21 (m, 20H, CH_2N, CH_2NH_2), 2.53–2.33 (m, 8H, $CHCH_2CH_2O$, PCH_2), 2.07 (q, 4H, *J* = 7.4 Hz, $CH_3CH_2CH_2$), 1.79–1.68 (m, 4H, $CH_2CH_2CH_2O$), 1.52–1.36 (m, 10H, CH_3CH_2, $(CH_2)_2(CH_2)_2O$), 0.93 (t, 6H, *J* = 7.4 Hz, CH_3).

^{13}C NMR (101 MHz, CD_3OD): 134.11 ($CH_3(CH_2)_2CH$, 125.28 ($CH(CH_2)_2O$), 67.93 (d, *J* = 6.6 Hz, $(CH_2)_2CH_2O$), 67.38 (d, *J* = 6.7 Hz, $CHCH_2CH_2O$), 51.48 ($CH_2CH_2NH_2$), 48.84 (PCH_2CH_2), 36.72 (CH_2NH_2), 31.47 (d, *J* = 6.0 Hz), 31.46 (d, *J* = 6.1 Hz, $CH_2CH_2CH_2O$), 30.44 ($CH_3CH_2CH_2$), 29.77, 29.75 ($CH_2(CH_2)_2O$), 29.66 (d, *J* = 6.2 Hz, $CHCH_2CH_2O$), 26.53 ($CH_2(CH_2)_3O$), 23.78 (CH_3CH_2), 22.18 (d, *J* = 139.0 Hz, PCH_2), 14.15 (CH_3).

^{31}P{^{1}H} NMR (162 MHz, CD_3OD): 31.44.

IR ν_{max} (KBr) 3100–2500 (s, vbr), 3010 (s), 2958 (vs), 2932 (s), 2871 (s), 2046 (w), 1653 (vw), 1601 (w), 1466 (m), 1379 (w), 1233 (m), 1004 (vs).

HR-MS(ESI^{+}): For $C_{33}H_{74}O_6N_6P_2$ $(M+2H)^{2+}$ *m*/2*z* calculated 356.25671, found 356.25675.

^{1}H NMR spectrum of compound **54**

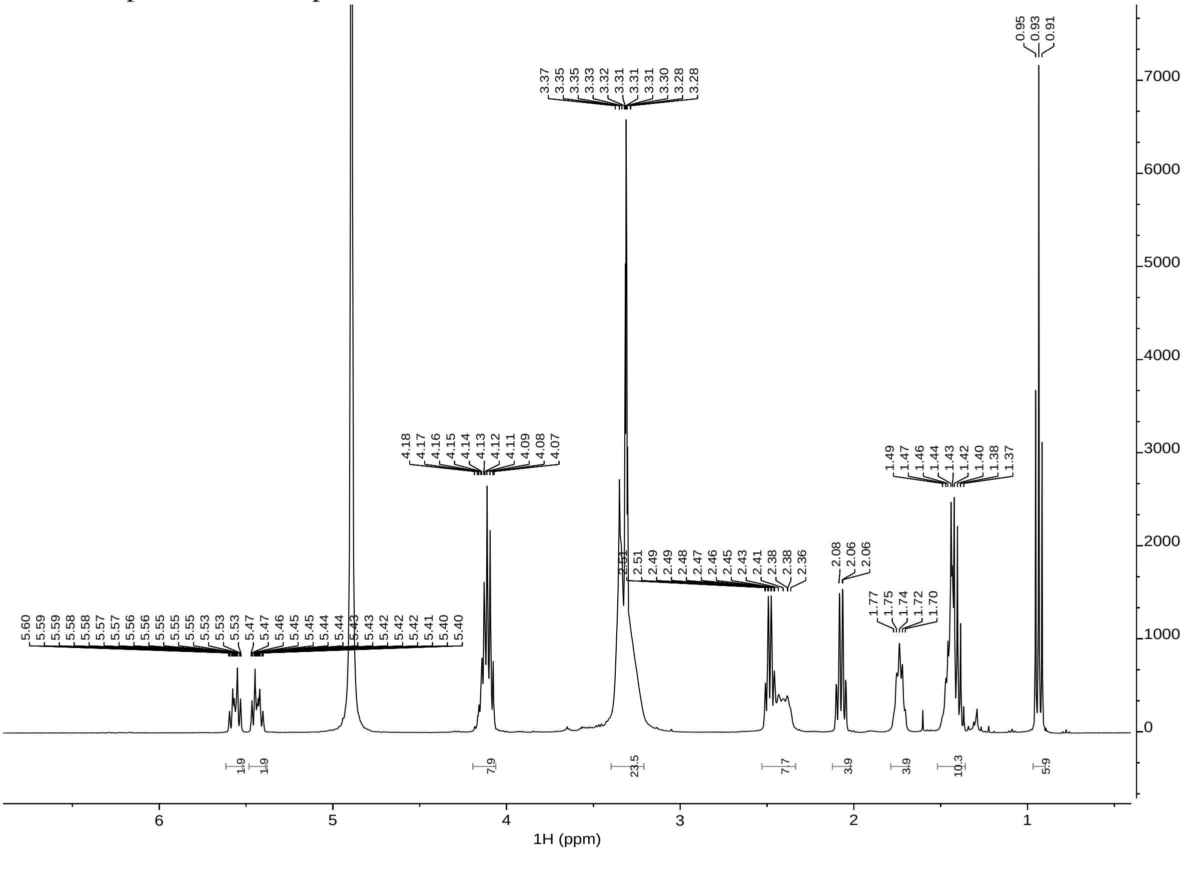

^{13}C NMR spectrum of compound **54**

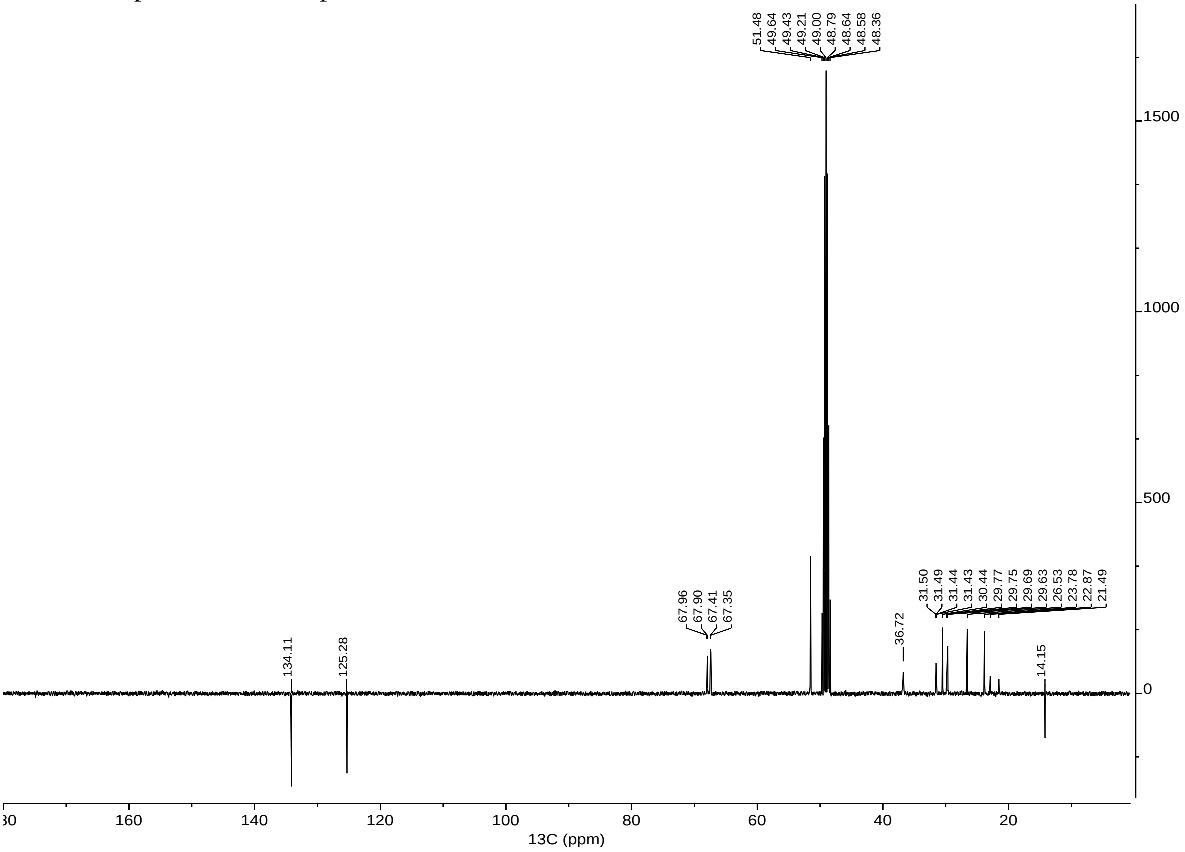

$^{31}P\{^{1}H\}$ NMR spectrum of compound **54**

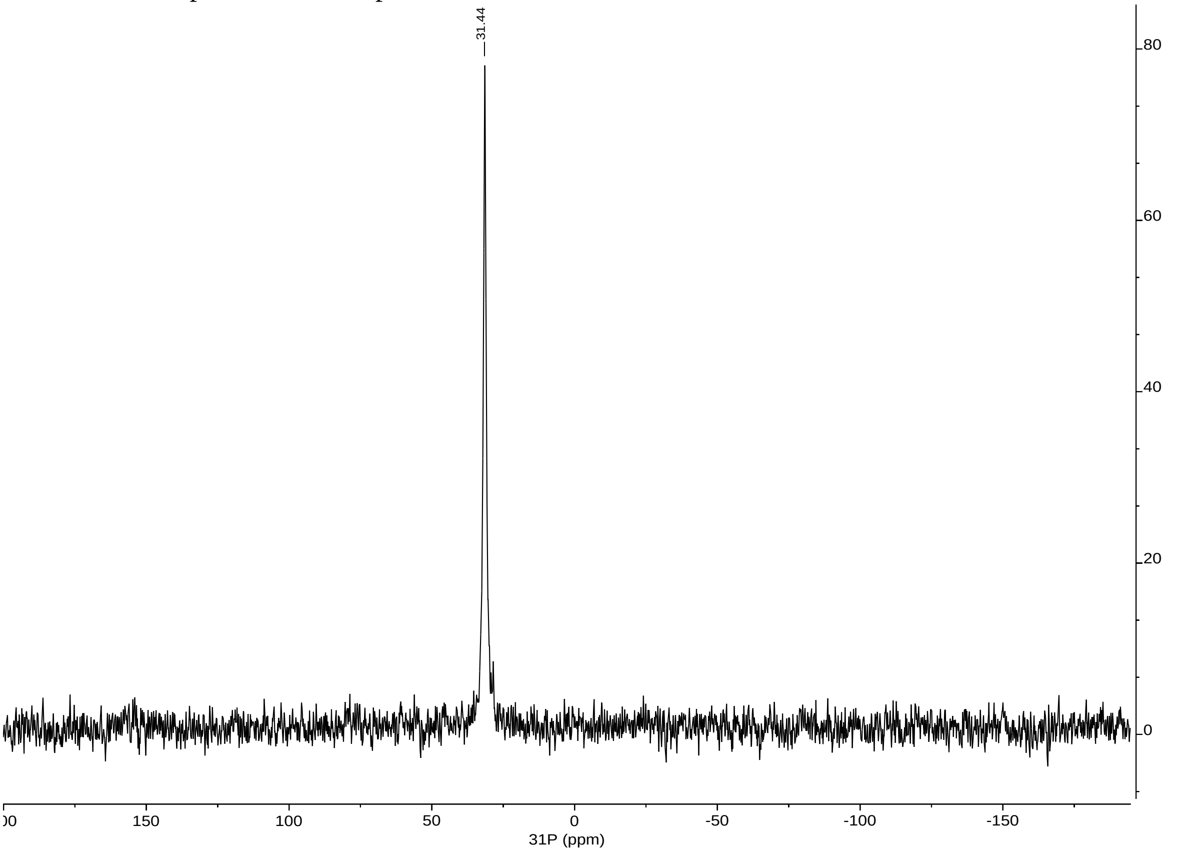

LC-MS chromatograms for compound **54**

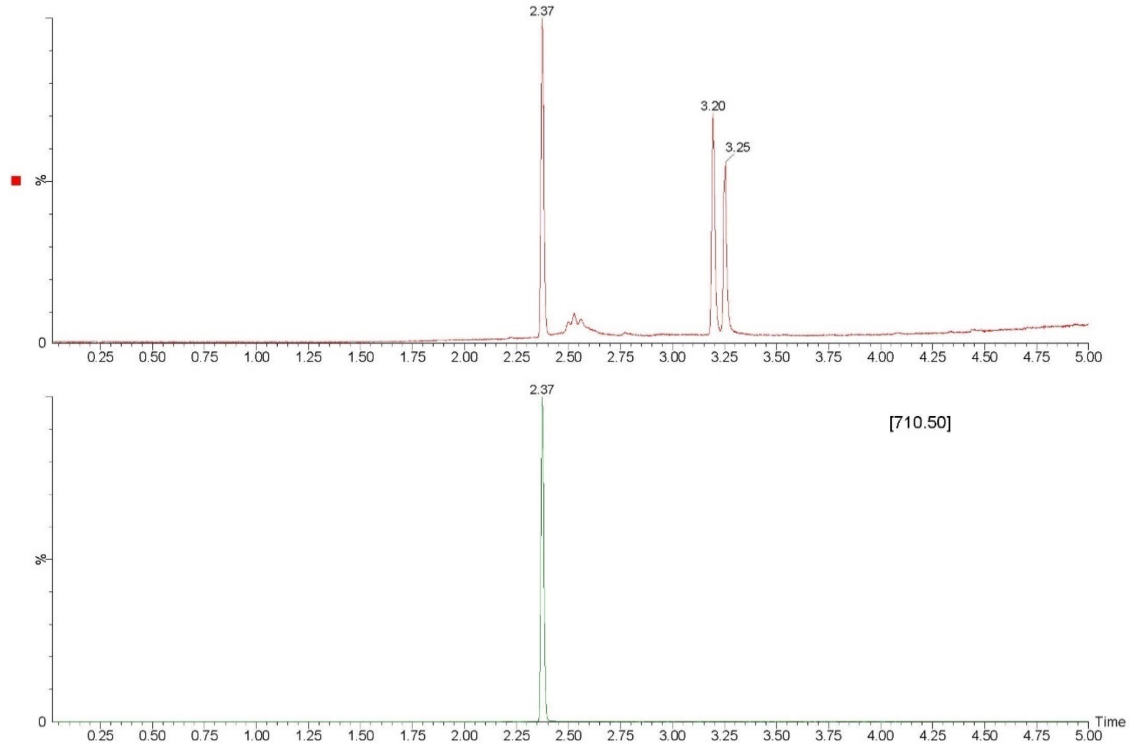

Diisobutyl octane-1,8-diyl bis((2-(bis(3-aminopropyl)amino)ethyl)phosphonate) hexahydrochloride (55)

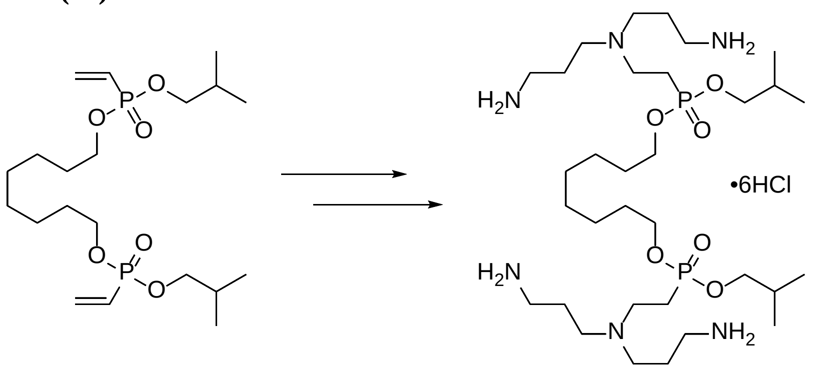

The title compound was prepared according to general methods **D** and **E** from vinylphosphonate dimer **S68** (1.36 g, 3.10 mmol) in 48% overall yield (1.37 g, 1.49 mmol) as a white solid.

^{1}H NMR (401 MHz, CD_3OD) δ 4.14 (t, *J* = 7.9, 6.6 Hz, 4H, OC*H*$_2$CH$_2$), 3.99–3.86 (m, 4H, OC*H*$_2$CH), 3.49–3.42 (m, 4H, PCH$_2$C*H*$_2$), 3.39 (dd, *J* = 9.2, 7.1 Hz, 8H, C*H*$_2$(CH$_2$)$_2$NH$_2$), 3.10 (t, *J* = 7.5 Hz, 8H, C*H*$_2$NH$_2$), 2.54 (dtd, *J* = 16.9, 8.4, 4.7 Hz, 4H, PC*H*$_2$), 2.27–2.14 (m, 8H, C*H*$_2$CH$_2$NH$_2$), 1.98 (dh, *J* = 13.3, 6.7 Hz, 2H, OCH$_2$C*H*), 1.73 (p, *J* = 6.6 Hz, 4H, OCH$_2$C*H*$_2$), 1.49–1.36 (m, 8H, O(CH$_2$)$_2$(C*H*$_2$)$_2$), 0.99 (d, *J* = 6.7 Hz, 12H, C*H*$_3$).

^{13}C NMR (101 MHz, CD_3OD) δ 73.92 (d, *J* = 7.0 Hz, O*C*H$_2$CH), 68.25 (d, *J* = 6.7 Hz, O*C*H$_2$CH$_2$), 51.07 (*C*H$_2$(CH$_2$)$_2$NH$_2$), 48.79 (PCH$_2$*C*H$_2$), 37.87 (*C*H$_2$NH$_2$), 31.56 (d, *J* = 5.9 Hz, OCH$_2$*C*H$_2$), 30.46 (d, *J* = 6.3 Hz, OCH$_2$*C*H), 30.16, 26.54 (O(CH$_2$)$_2$(*C*H$_2$)$_2$), 23.31 (*C*H$_2$CH$_2$NH$_2$), 21.20 (d, *J* = 140.6 Hz, P*C*H$_2$), 19.03 (*C*H$_3$).

^{31}P{^{1}H} NMR (162 MHz, CD_3OD) δ 28.60.

IR ν_{max} 2959–2558 (m, vbr), 2953 (vs), 2875 (s), 2754–2558 (m, vbr), 2046 (w), 1608 (w), 1517 (sh, w), 1470 (m), 1396 (w), 1369 (vw), 1227 (m), 1009 (vs), 913 (vw).

HR-MS(ESI$^+$): For $C_{32}H_{75}O_6N_6P_2$ $(M+H)^+$ *m/z* calculated 701.52178, found 701.52204, for $C_{32}H_{76}O_6N_6P_2$ $(M+2H)^{2+}$ *m*/2*z* calculated 351.26453, found 351.26459.

^{1}H NMR spectrum of compound **55**

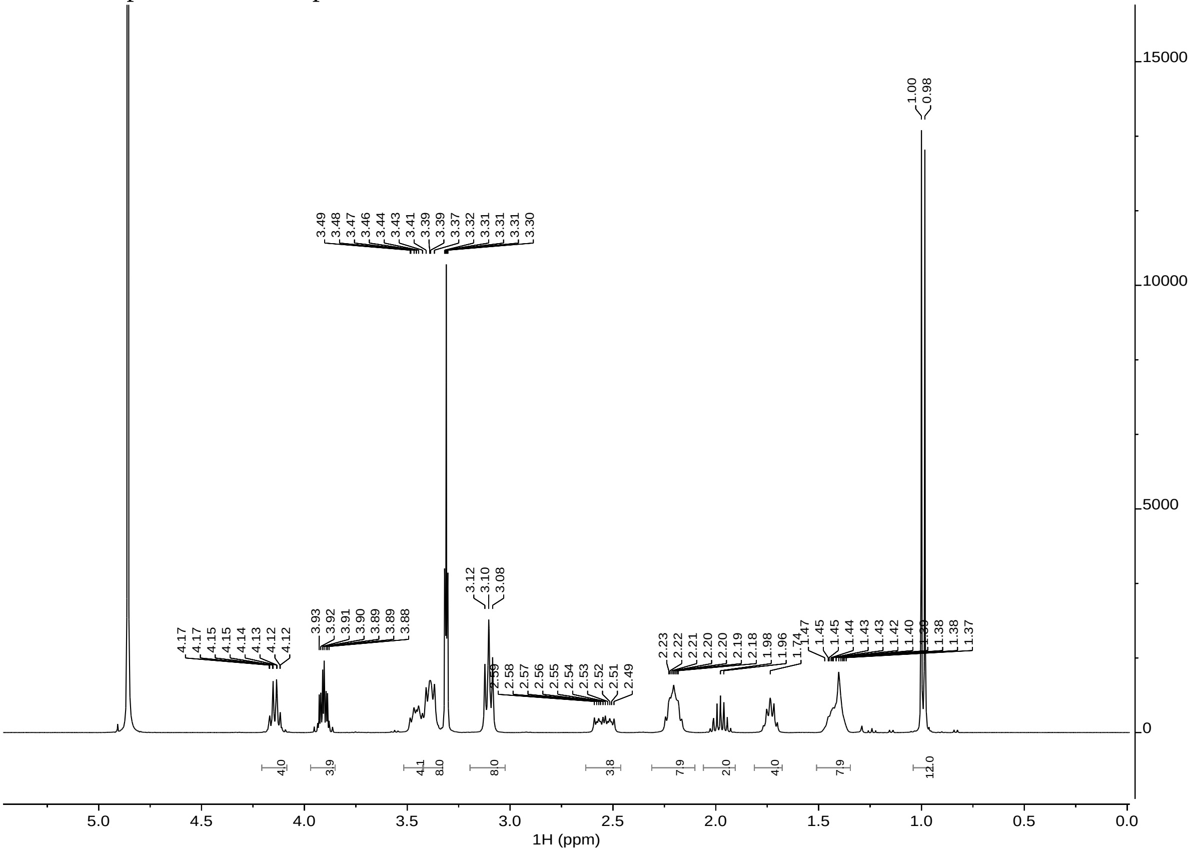

^{13}C NMR spectrum of compound **55**

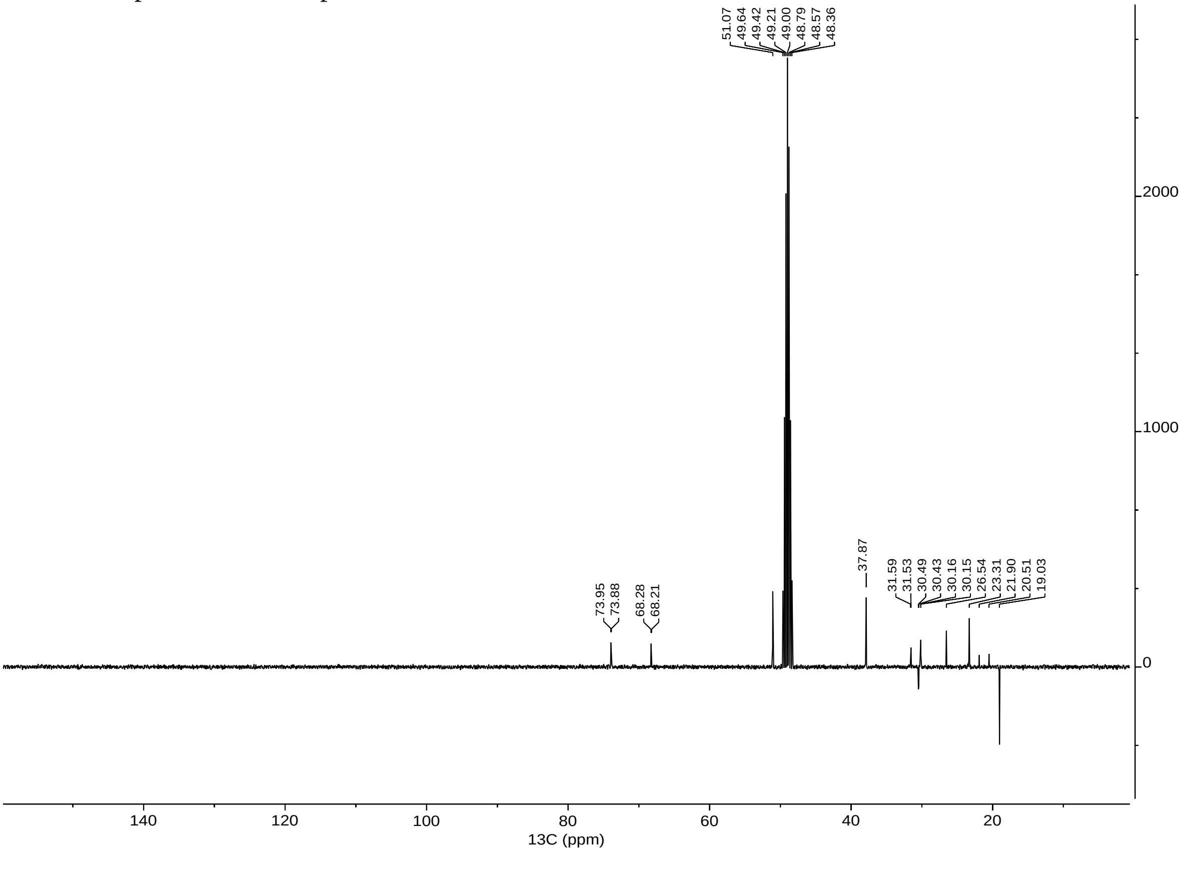

$^{31}P\{^1H\}$ NMR spectrum of compound **55**

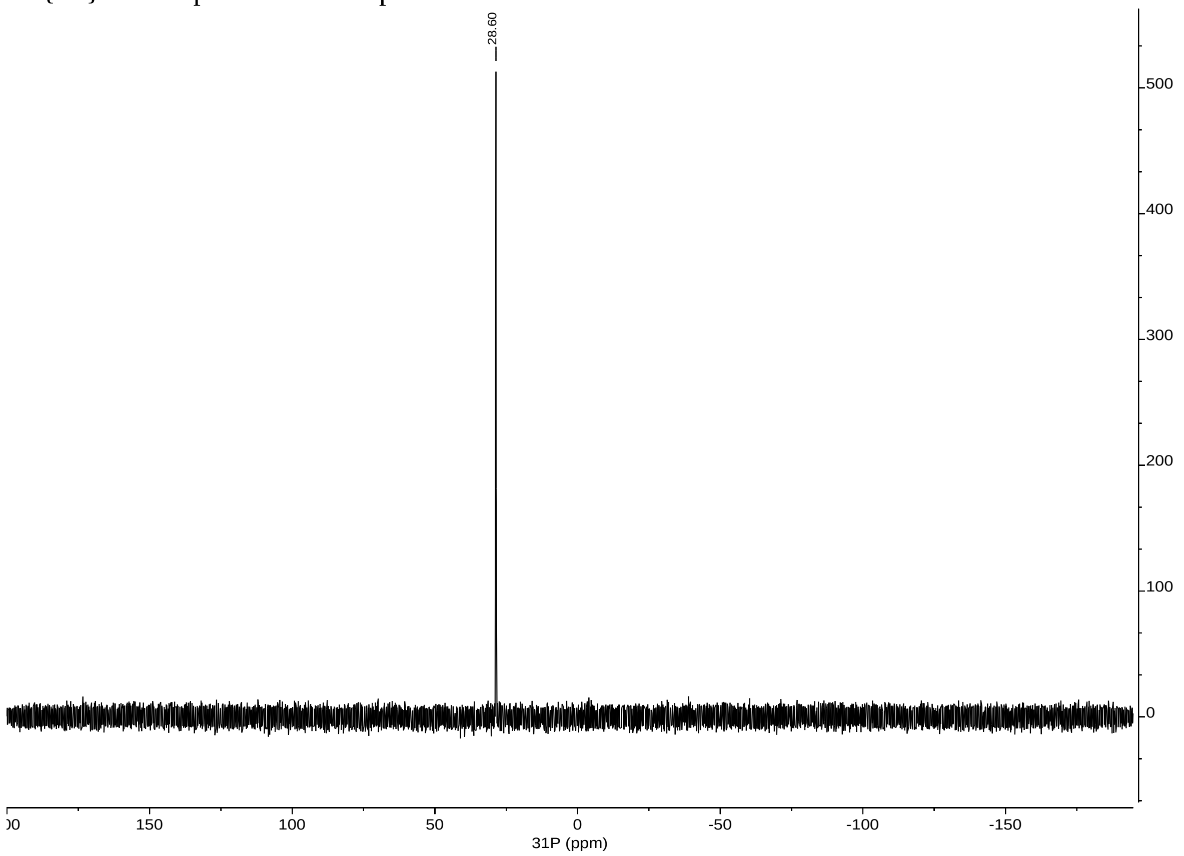

LC-MS chromatograms for compound **55**

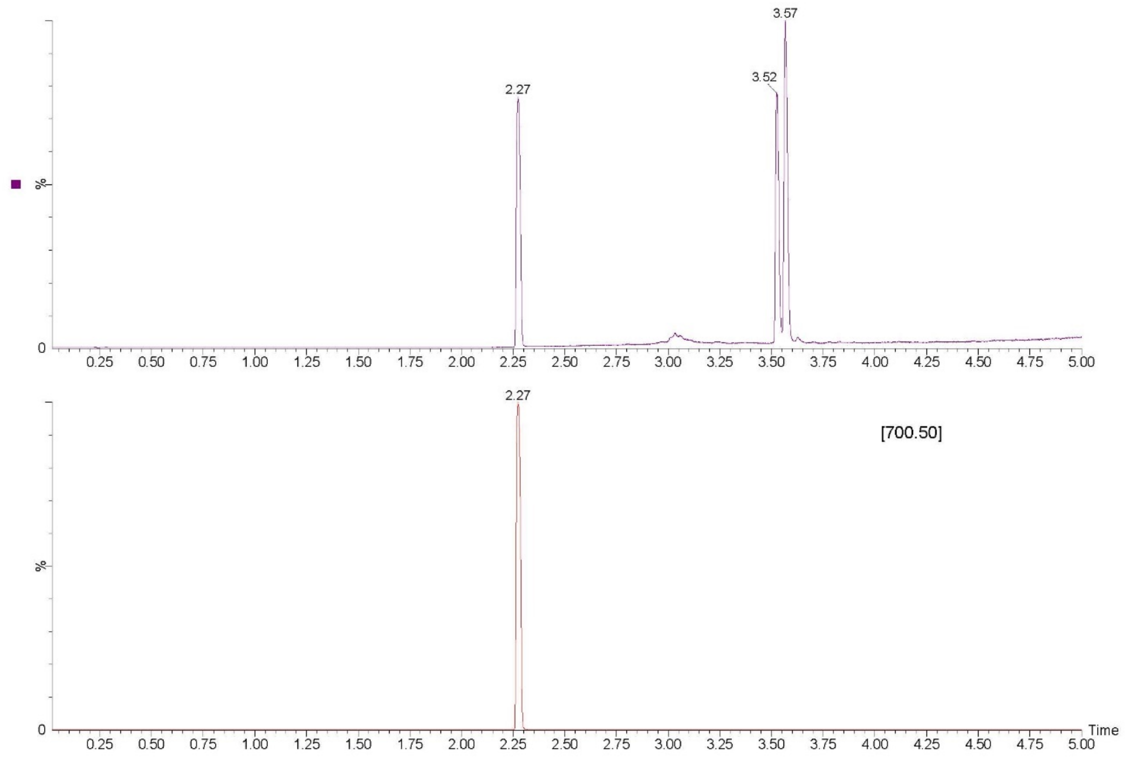

Dibutyl octane-1,8-diyl bis((2-(bis(3-aminopropyl)amino)ethyl)phosphonate) hexahydrochloride (56)

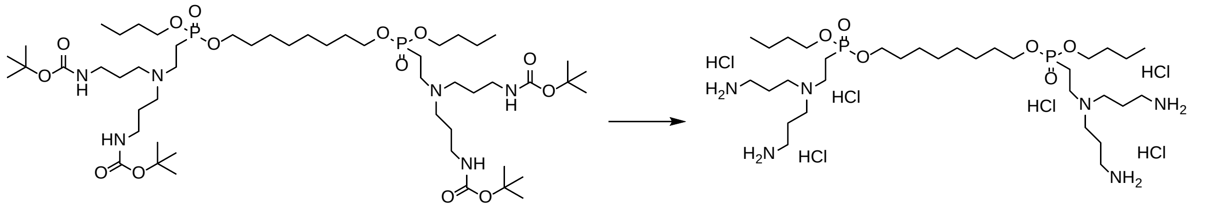

The title compound was prepared according to general method **E** from Boc derivative **S110** (0.69 g, 0.63 mmol) in 91% yield (0.53 g, 0.57 mmol) as a white solid.

^{1}H NMR (400 MHz, CD_3OD): 4.19–4.09 (m, 8H, CH_2O), 3.49–3.42 (m, 4H, PCH_2CH_2), 3.42–3.34 (m, 8H, $CH_2(CH_2)_2NH_2$), 3.10 (t, 8H, *J* = 7.5 Hz, CH_2NH_2), 2.60–2.46 (m, 4H, PCH_2), 2.27–2.14 (m, 8H, $CH_2CH_2NH_2$), 1.78–1.66 (m, 8H, CH_2CH_2O), 1.51–1.34 (m, 12H, CH_3CH_2, $(CH_2)_2(CH_2)_2O$), 0.98 (t, 6H, *J* = 7.4 Hz, CH_3).

^{13}C NMR (101 MHz, CD_3OD): 68.21 (d, *J* = 6.9 Hz), 67.91 (d, *J* = 6.7 Hz, CH_2O), 51.07 ($CH_2(CH_2)_2NH_2$), 48.84 (PCH_2CH_2), 37.90 (CH_2NH_2), 33.61 (d, *J* = 5.9 Hz), 31.54 (d, *J* = 5.8 Hz, CH_2CH_2O), 30.14, 26.53 ($(CH_2)_2(CH_2)_2O$), 23.29 ($CH_2CH_2NH_2$), 21.29 (d, *J* = 140.8 Hz, PCH_2), 19.79 (CH_3CH_2), 13.96 (CH_3).

^{31}P{^{1}H} NMR (162 MHz, CD_3OD): 28.65.

IR ν_{max} (KBr) 2954 (vs), 2740 (s, sh), 2632 (s), 2558 (s), 2007 (w), 1599 (m), 1510 (m, sh), 1467 (s), 1223 (s, br), 1064 (s), 1018 (vs, br), 995 (s, br).

HR-MS(ESI^{+}): For $C_{32}H_{76}N_6O_6P_2$ $(M+2H)^{2+}$ *m*/2*z* calculated 351.26453, found 351.26434.

^{1}H NMR spectrum of compound **56**

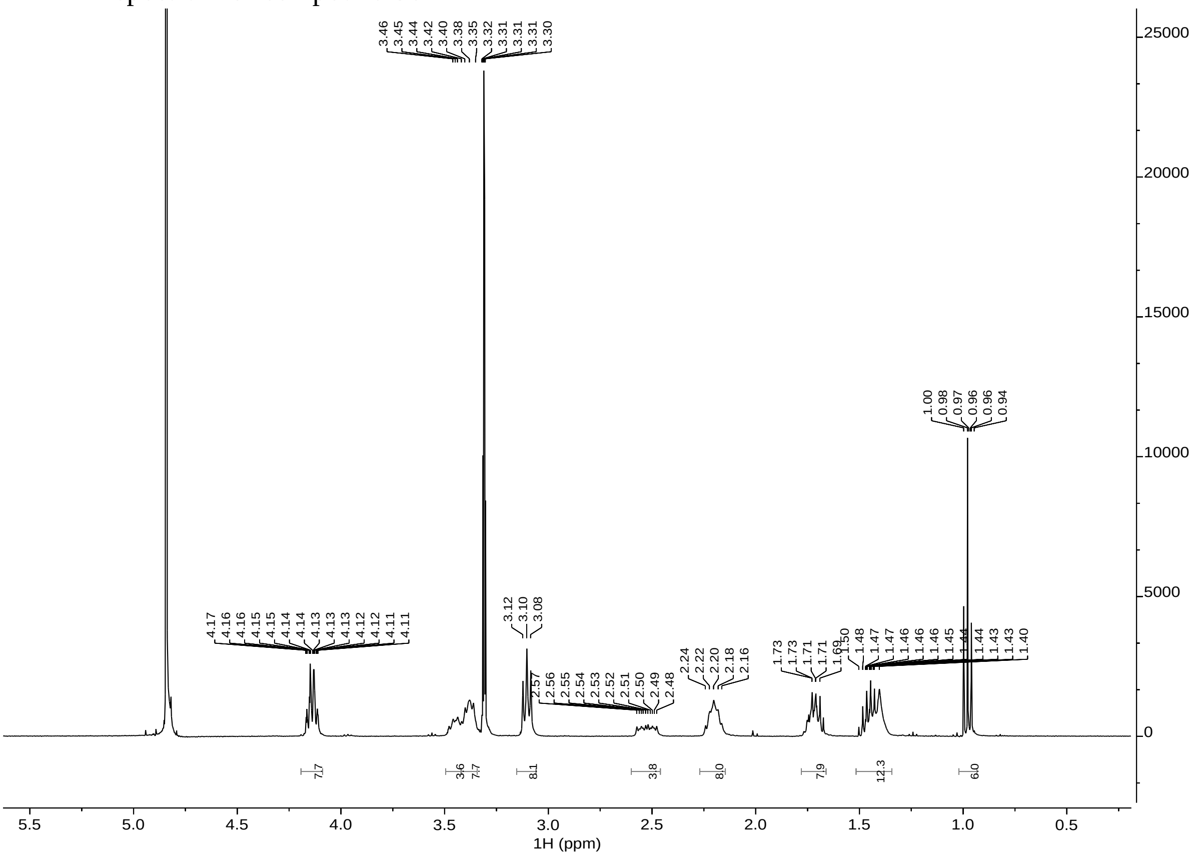

^{13}C NMR spectrum of compound **56**

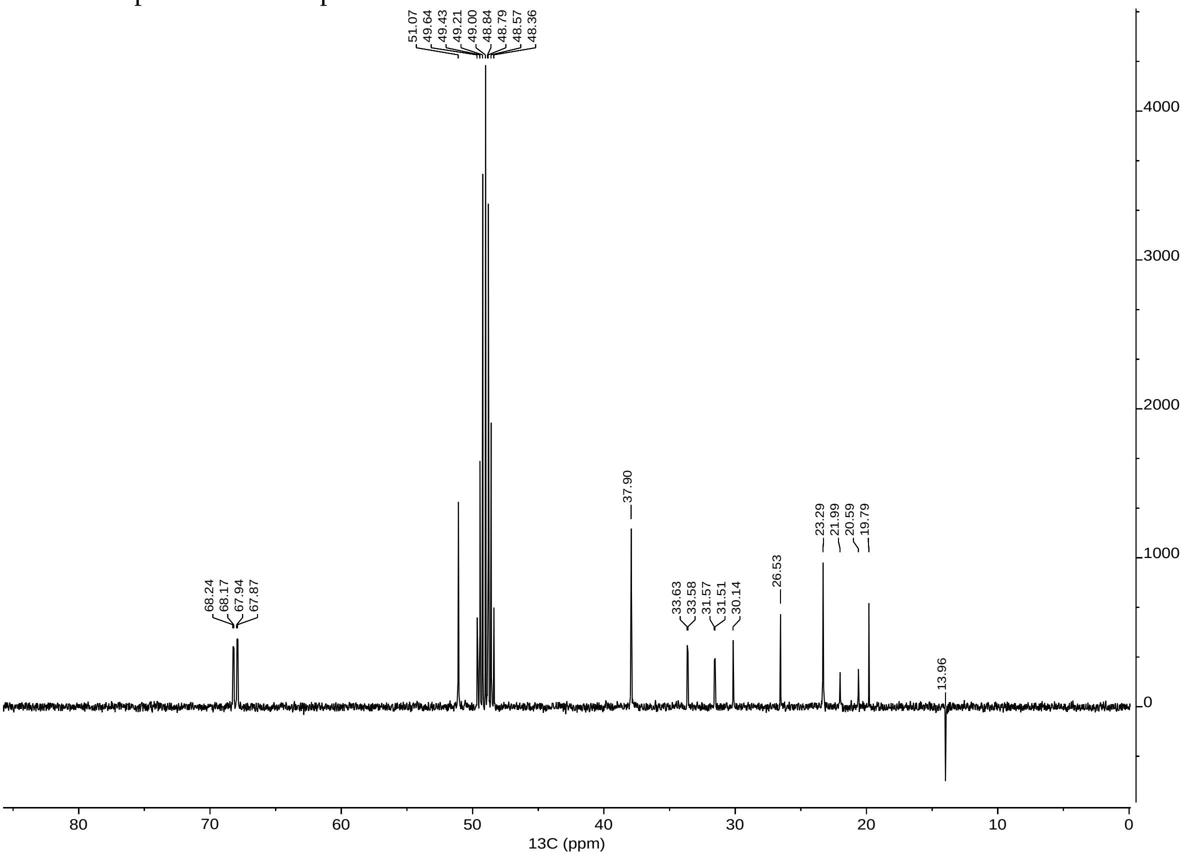

^{31}P{^{1}H} NMR spectrum of compound **56**

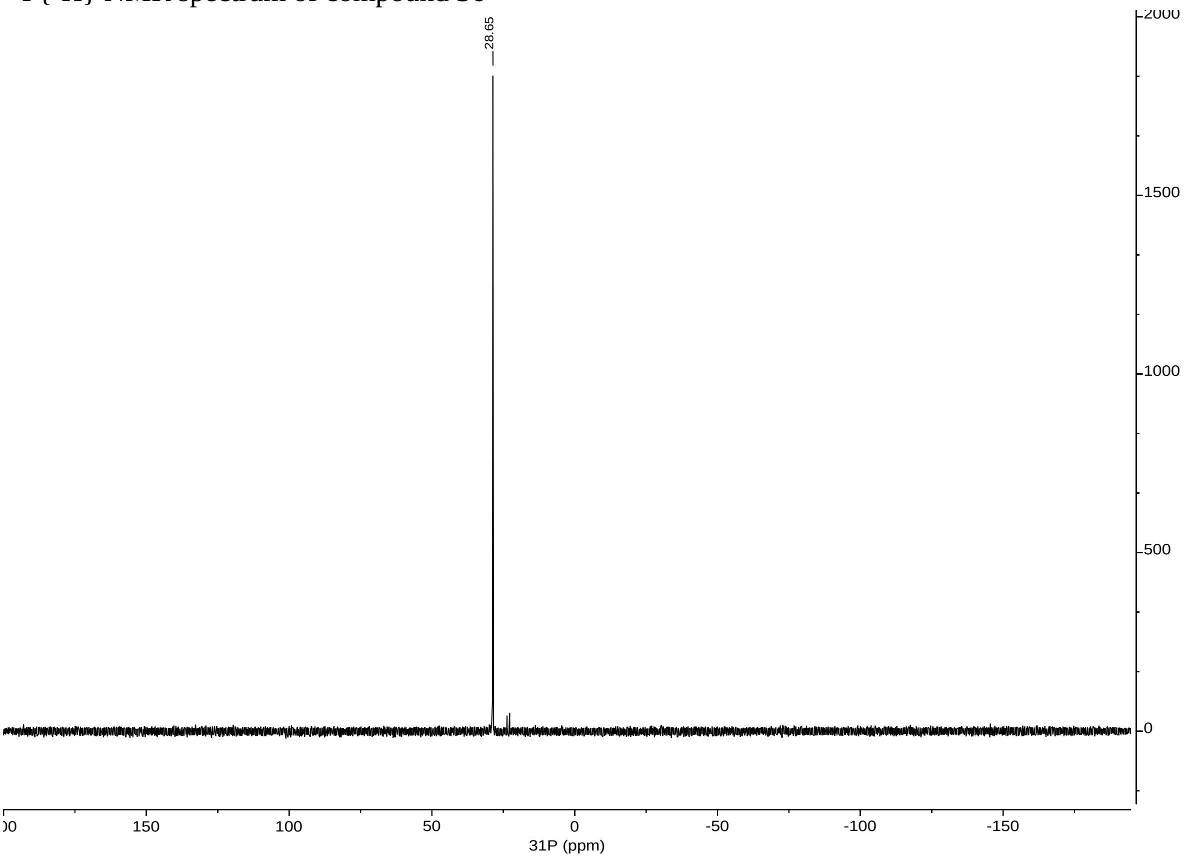

LC-MS chromatograms for compound **56**

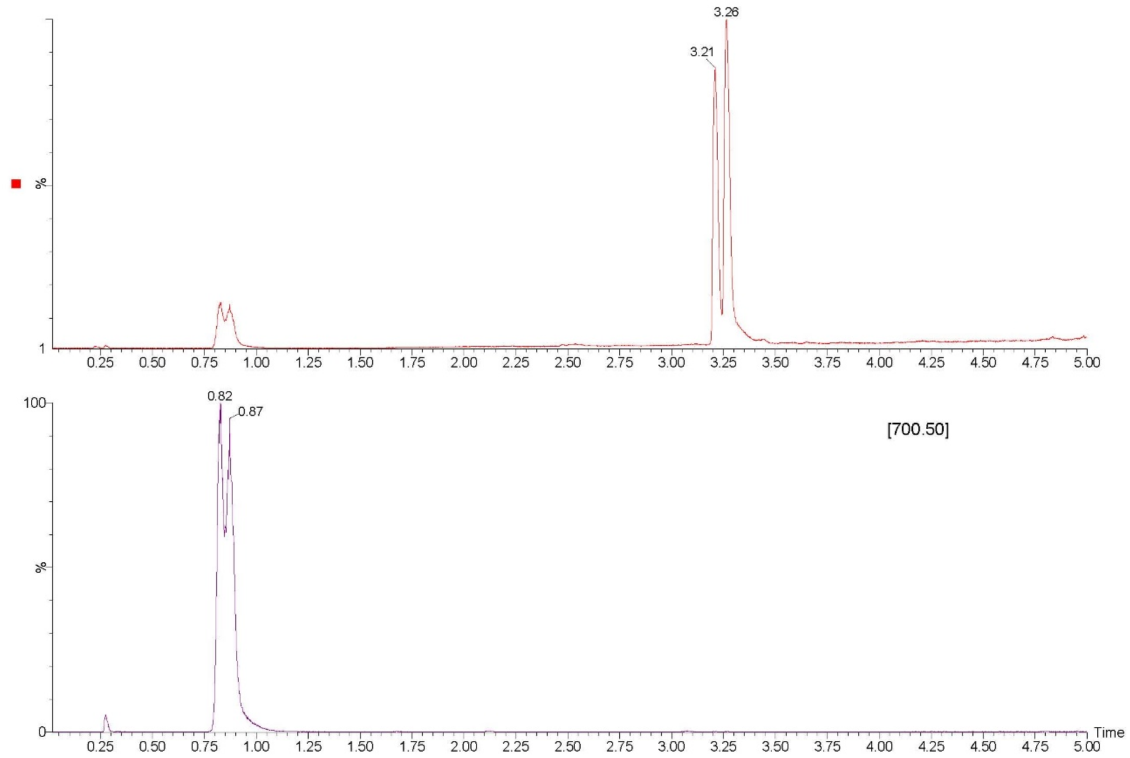

Octane-1,8-diyl dipentyl bis((2-(bis(3-aminopropyl)amino)ethyl)phosphonate) hexahydrochloride (57)

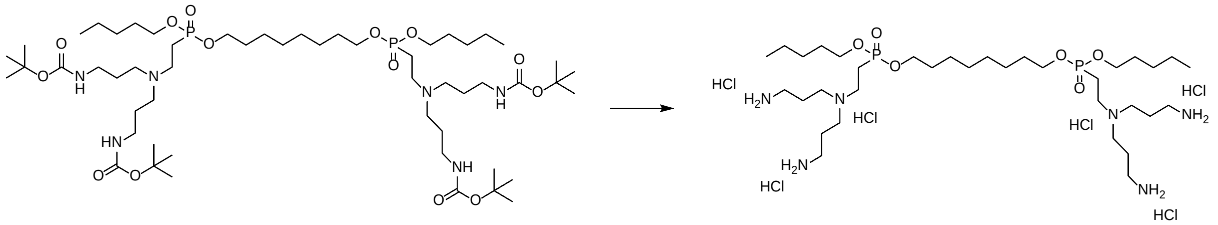

The title compound was prepared according to general method **E** from Boc derivative **S111** (0.70 g, 0.62 mmol) in 92% yield (0.54 g, 0.57 mmol) as a white solid.

^{1}H NMR (400 MHz, CD_3OD): 4.20–4.07 (m, 8H, CH_2O), 3.49–3.43 (m, 4H, PCH_2CH_2), 3.43–3.36 (m, 8H, $CH_2(CH_2)_2NH_2$), 3.11 (t, 8H, J = 7.5 Hz, CH_2NH_2), 2.61–2.47 (m, 4H, PCH_2), 2.27–2.15 (m, 8H, $CH_2CH_2NH_2$), 1.79–1.67 (m, 8H, CH_2CH_2O), 1.48–1.32 (m, 16H, $(CH_2)_2(CH_2)_2O$), 0.99–0.90 (m, 6H, CH_3).

^{13}C NMR (101 MHz, CD_3OD): 68.22 (d, J = 6.7 Hz, CH_2O), 51.07 ($CH_2(CH_2)_2NH_2$), 48.84 (PCH_2CH_2), 37.89 (CH_2NH_2), 31.56 (d, J = 5.9 Hz), 31.29 (d, J = 5.9 Hz, CH_2CH_2O), 30.16, 30.15, 28.79, 26.54, 23.29, 23.28 ($CH_2CH_2NH_2$), 21.28 (d, J = 140.4 Hz, PCH_2), 14.34 (CH_3).

^{31}P{^{1}H} NMR (162 MHz, CD_3OD): 28.40.

IR ν_{max} (KBr) 2933 (s), 2634 (s), 2558 (m), 1600 (m), 1467 (m), 1224 (m), 1173 (m, sh), 1070 (m, sh), 1047 (s), 997 (s).

HR-MS(ESI$^+$): For $C_{34}H_{79}N_6O_6P_2$ $(M+H)^+$ m/z calculated 729.55308, found 729.55290.

^{1}H NMR spectrum of compound **57**

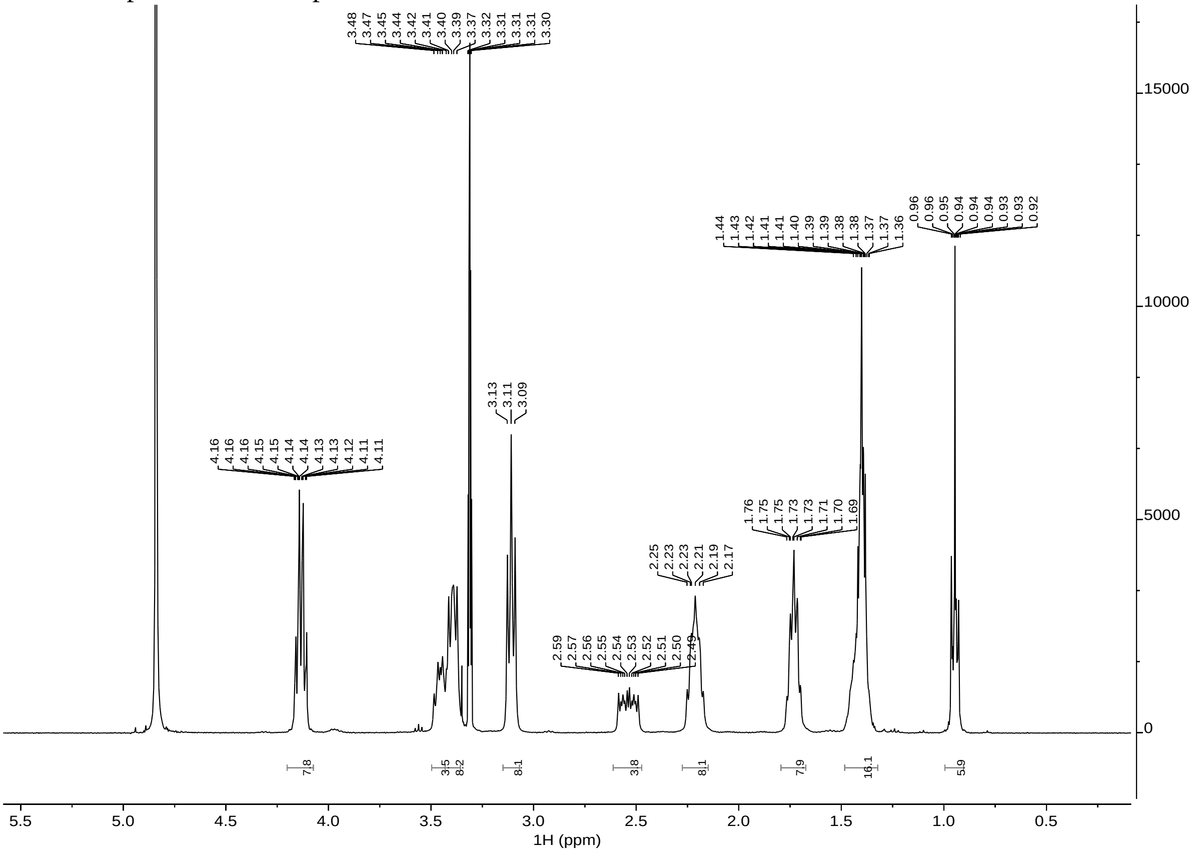

^{13}C NMR spectrum of compound **57**

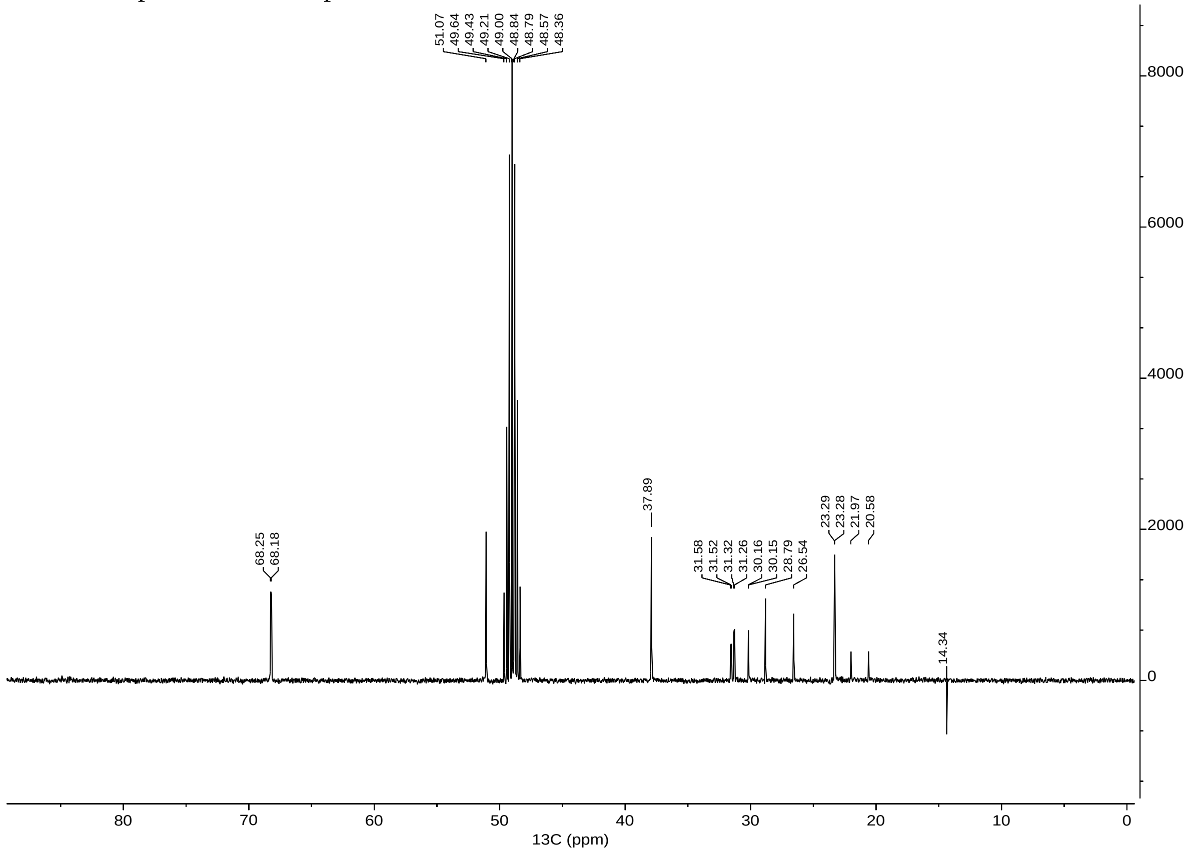

^{31}P{^{1}H} NMR spectrum of compound **57**

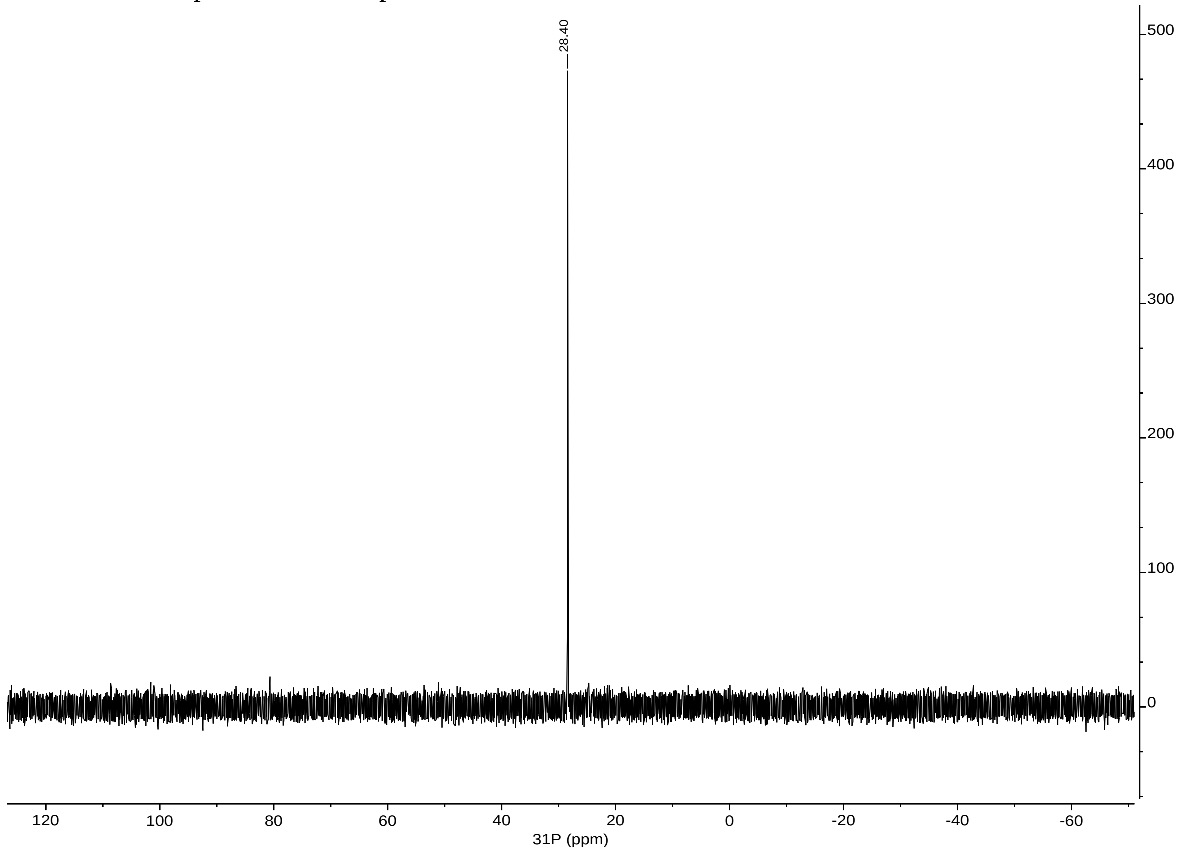

LC-MS chromatograms for compound **57**

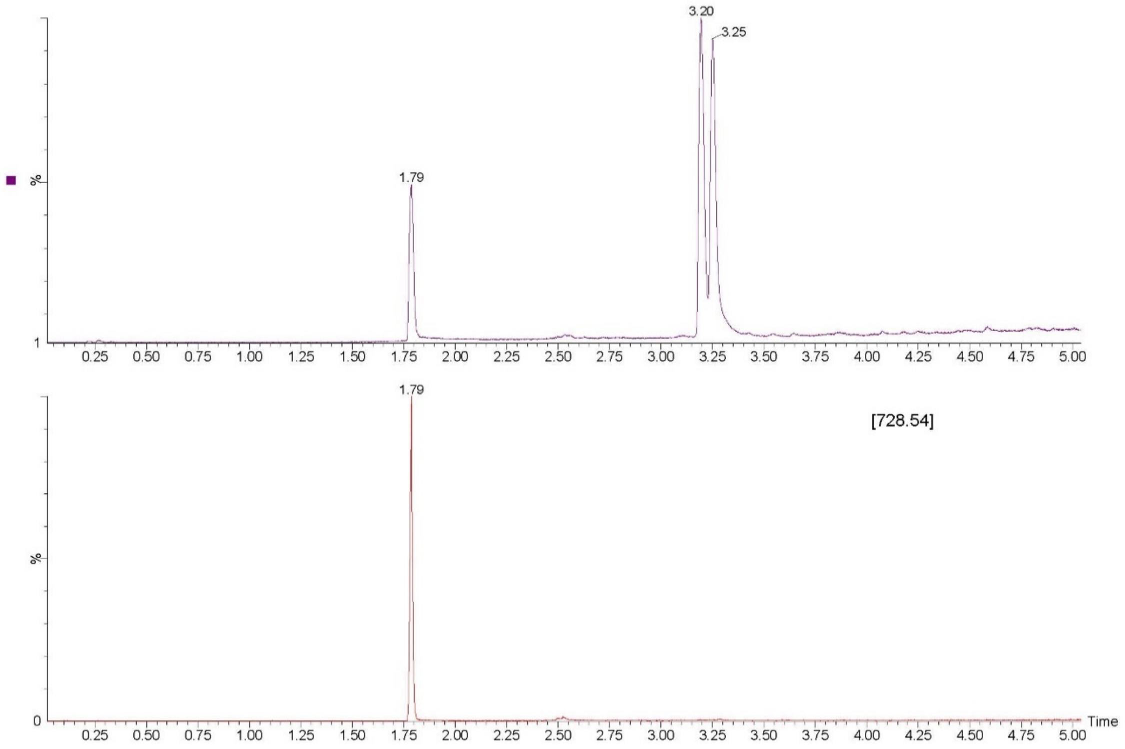

Octane-1,8-diyl dipentyl bis((2-(bis(2-aminoethyl)amino)ethyl)phosphonate) hexahydrochloride (58)

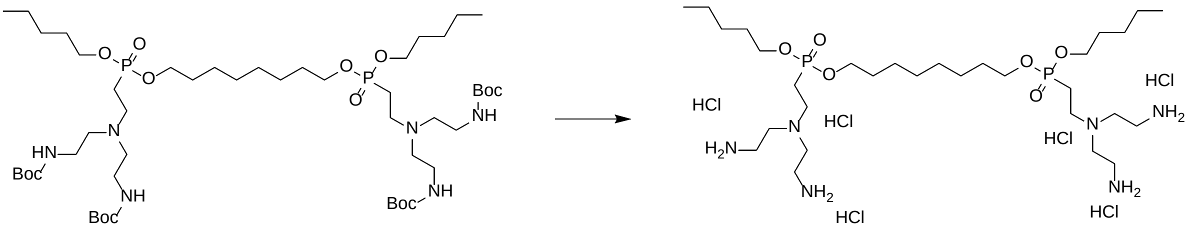

The title compound was prepared according to general method **E** from Boc derivative **S112** (0.62 g, 0.55 mmol) in 92% yield (0.45 g, 0.51 mmol) as a white solid.

^{1}H NMR (401 MHz, CD_3OD): 4.20–4.07 (m, 8H, CH_2O), 3.36–3.13 (m, 20H, $CH_2NCH_2CH_2NH_2$), 2.44–2.29 (m, 4H, PCH_2), 1.79–1.70 (m, 8H, CH_2CH_2O), 1.50–1.37 (m, 16H, $(CH_2)_2(CH_2)_2O$), 1.00–0.93 (m, 6H, CH_3).

^{13}C NMR (101 MHz, CD_3OD): 67.90 (d, J = 6.6 Hz, CH_2O), 51.50 ($CH_2CH_2NH_2$), 37.03 (CH_2NH_2), 31.57 (d, J = 6.1 Hz, $CH_3(CH_2)_2CH_2$), 31.30 (d, J = 5.9 Hz, $OCH_2CH_2(CH_2)_4CH_2CH_2O$), 30.18, 28.83, 26.59, 23.31 ($(CH_2)_2(CH_2)_2O$), 22.30 (d, J = 138.7 Hz, PCH_2), 14.35 (CH_3).

^{31}P{^{1}H} NMR (162 MHz, CD_3OD): 31.93.

IR ν_{max} 3000 (s, br), 2956 (vs), 2932 (vs), 2870 (s), 2859 (s), 2640 (m, br), 2543 (m, br), 2005 (w, br), 1666 (vs), 1594 (w), 1566 (w, sh), 1467 (m), 1390 (w), 1227 (m), 1017 (s), 1000 (s).

HR-MS(ESI$^+$): For $C_{30}H_{70}O_6N_6NaP_2$ $(M+Na)^+$ m/z calculated 695.47243, found 695.47269.

^{1}H NMR spectrum of compound **58**

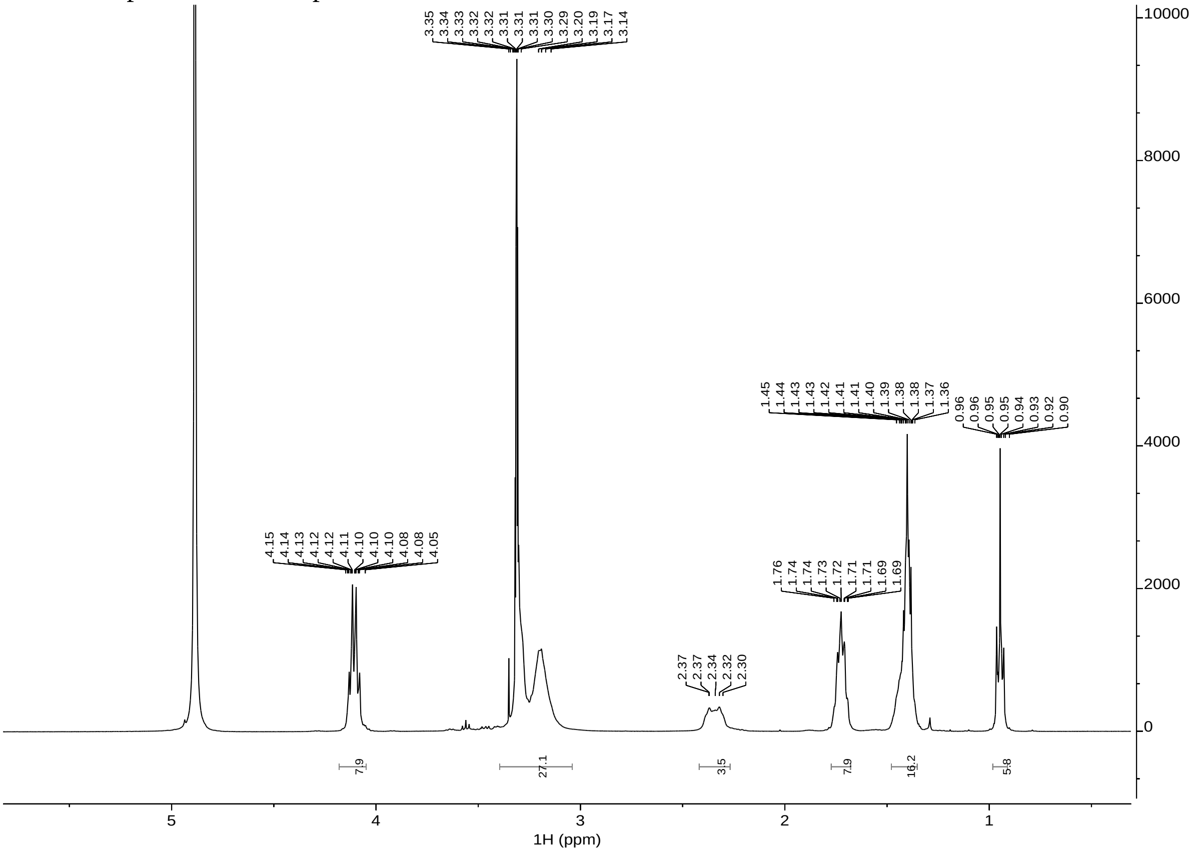

^{13}C NMR spectrum of compound **58**

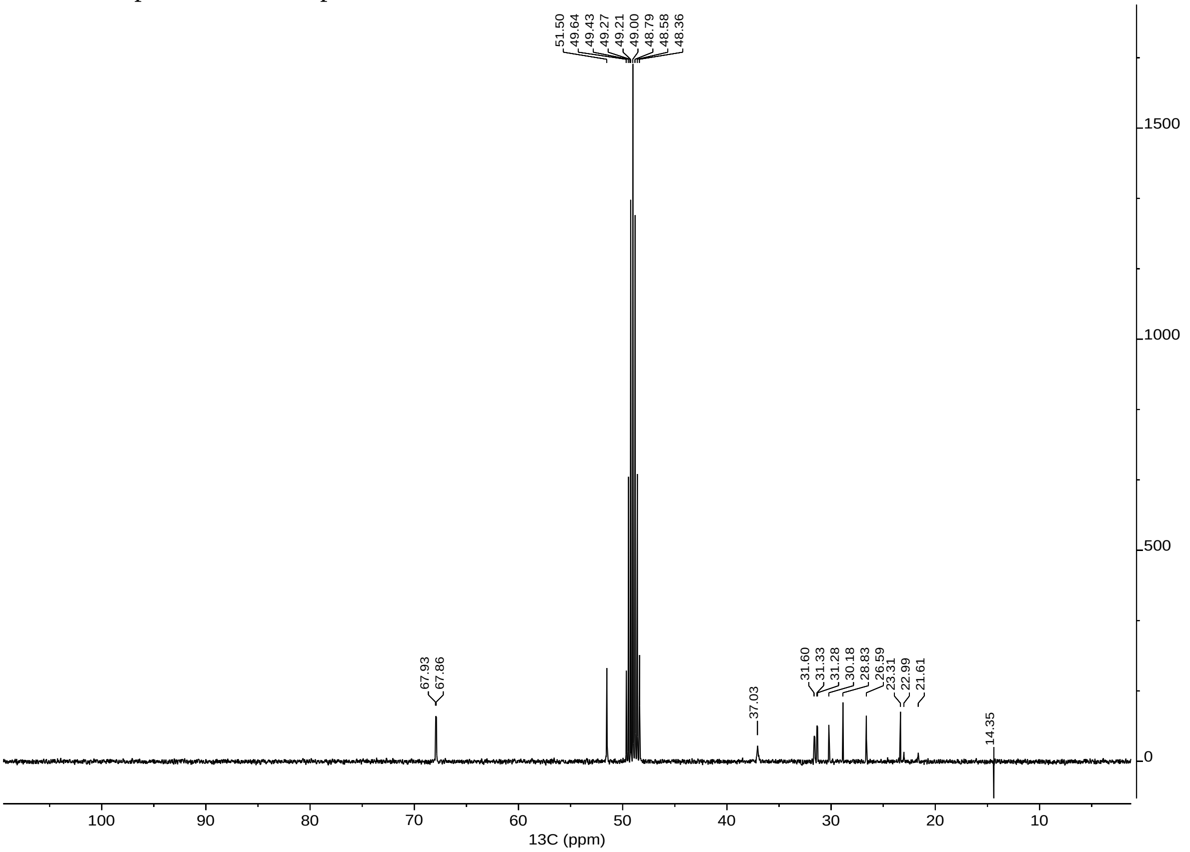

^{31}P{^{1}H} NMR spectrum of compound **58**

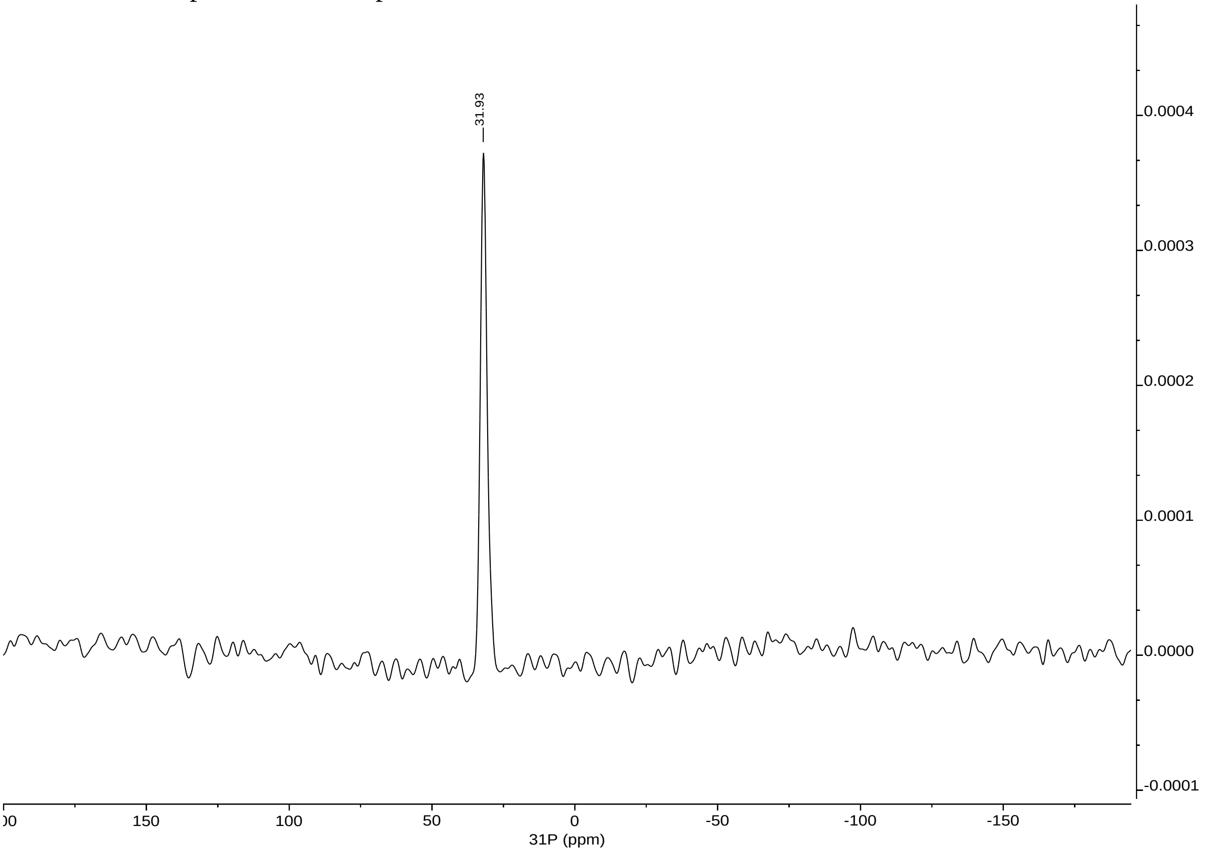

LC-MS chromatograms for compound **58**

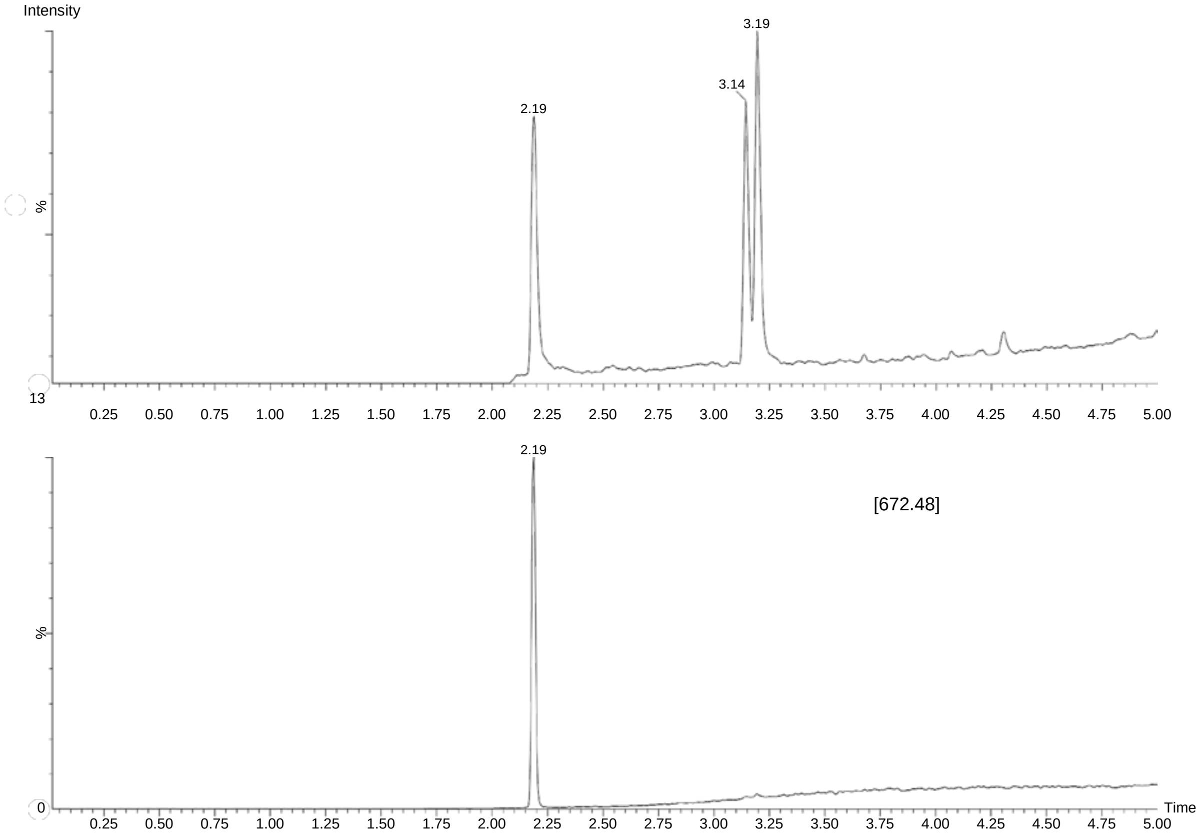

Dihexyl octane-1,8-diyl bis((2-(bis(3-aminopropyl)amino)ethyl)phosphonate) hexahydrochloride (59)

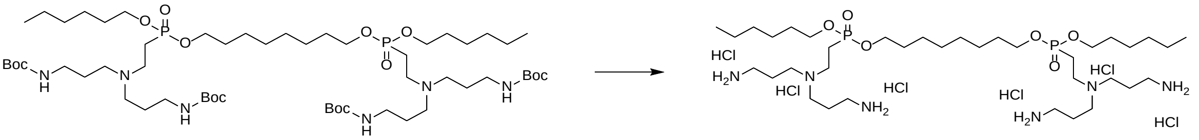

The title compound was prepared according to general method **E** from Boc derivative **S113** (1.10 g, 0.95 mmol) in 95% yield (0.88 g, 0.90 mmol) as a white solid.

^{1}H NMR (400 MHz, CD_3OD): 4.20–4.07 (m, 8H, CH_2O), 3.50–3.42 (m, 4H, PCH_2CH_2), 3.42–3.34 (m, 8H, CH_2(CH_2)$_2$$NH_2$), 3.11 (t, 8H, J = 7.5 Hz, C$H_2$$NH_2$), 2.62–2.45 (m, 4H, PC$H_2$), 2.28–2.14 (m, 8H, C$H_2$$CH_2NH_2$), 1.81–1.65 (m, 8H, C$H_2$$CH_2$O), 1.50–1.28 (m, 20H, CH_3CH_2, (CH_2)$_2$(CH_2)$_2$O), 0.98–0.88 (m, 6H, CH_3).

^{13}C NMR (101 MHz, CD_3OD): 68.24 (d, J = 6.8 Hz), 68.22 (d, J = 6.8 Hz, $C$$H_2$O), 51.08 ($C$$H_2$($CH_2$)$_2$$NH_2$), 48.82 ($PCH_2$$C$$H_2$), 37.89 ($C$$H_2NH_2$), 32.51 ($CH_3CH_2$$C$$H_2$), 31.57 (d, J = 6.0 Hz, $C$$H_2CH_2$O), 30.18, 26.56, 26.31, 23.63 ($CH_3$$C$$H_2$), 23.29 ($C$$H_2CH_2NH_2$), 21.27 (d, J = 140.1 Hz, P$C$$H_2$), 14.38 ($C$$H_3$).

^{31}P{^{1}H} NMR (162 MHz, CD_3OD): 28.45.

IR ν_{max} (KBr) 2966 (m), 2931 (m), 2872 (m), 2623 (w), 2553 (w), 1470 (m), 1384 (w), 1236 (w), 1050 (m), 1000 (m).

HR-MS(ESI$^+$): For $C_{36}H_{84}N_6O_6P_2$ (M+2H)$^{2+}$ $m/2z$ calculated 379.29583, found 379.29553.

^{1}H NMR spectrum of compound **59**

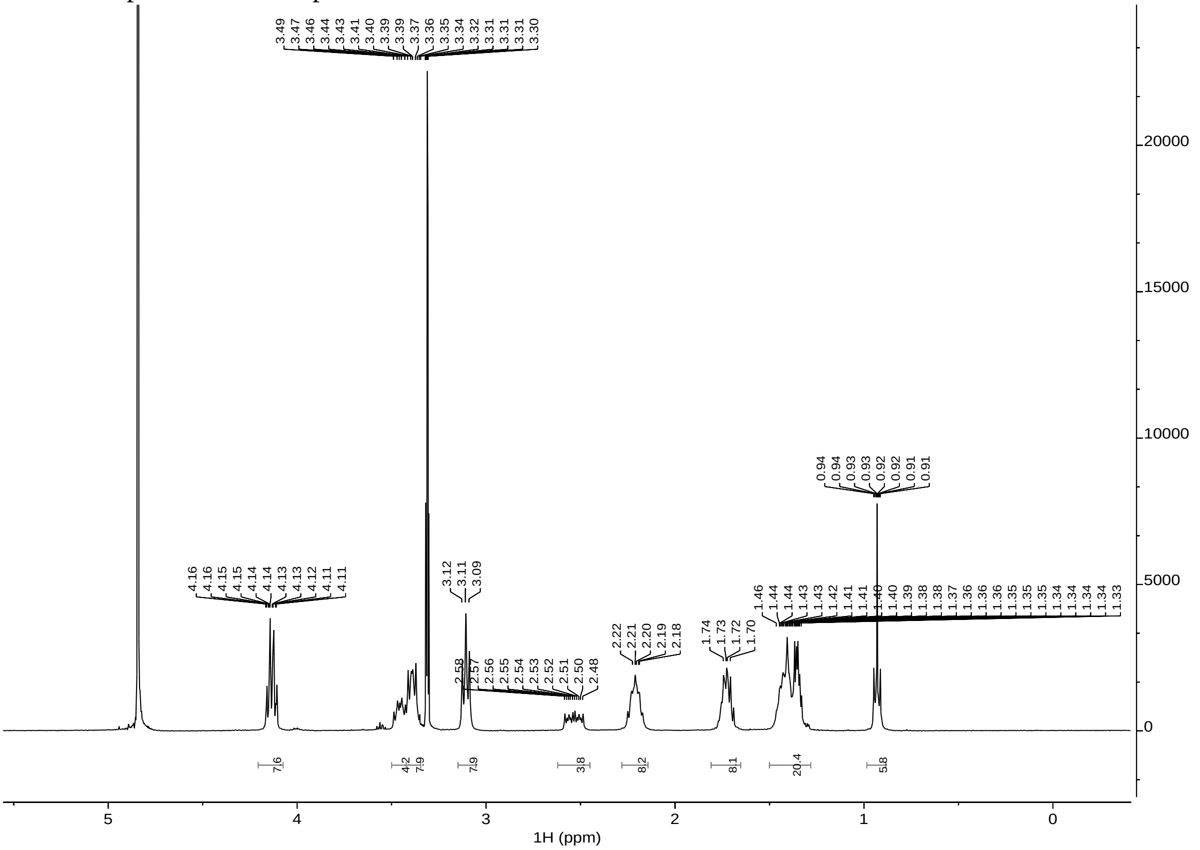

^{13}C NMR spectrum of compound **59**

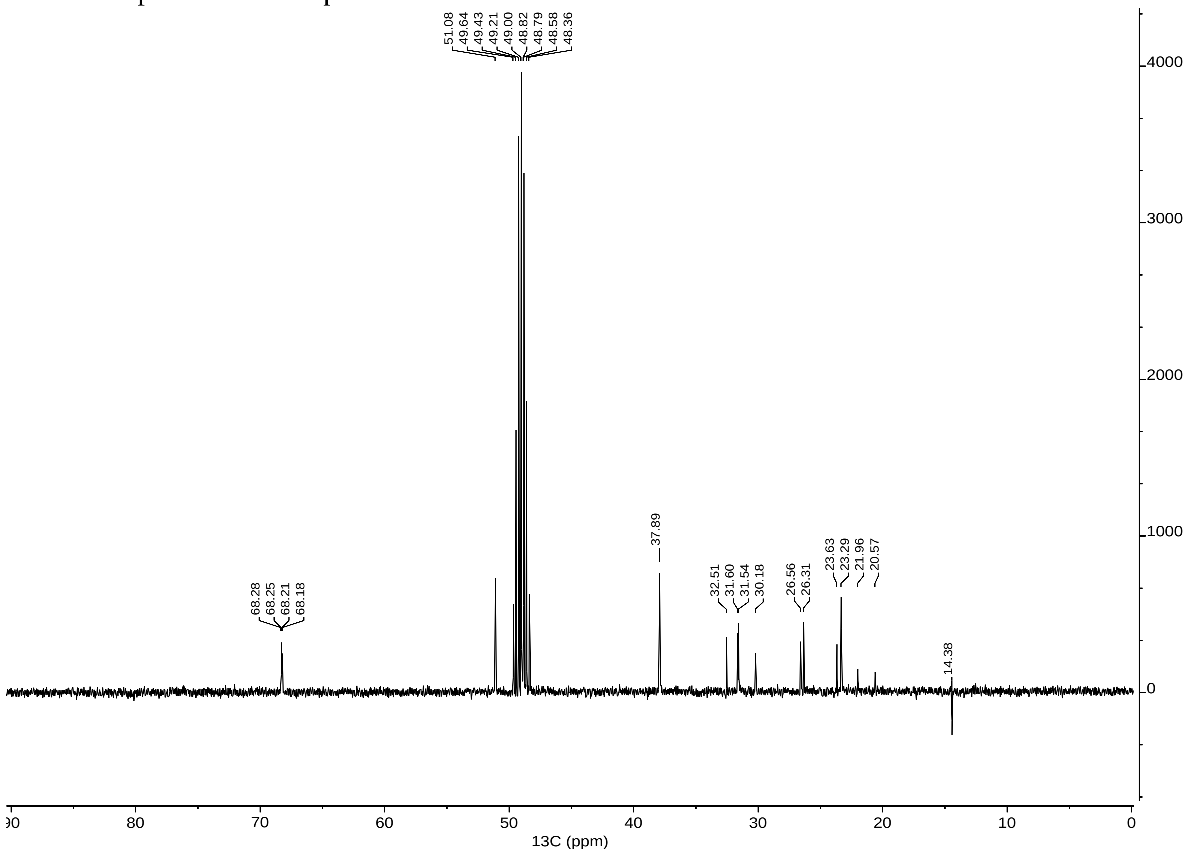

$^{31}P\{^1H\}$ NMR spectrum of compound **59**

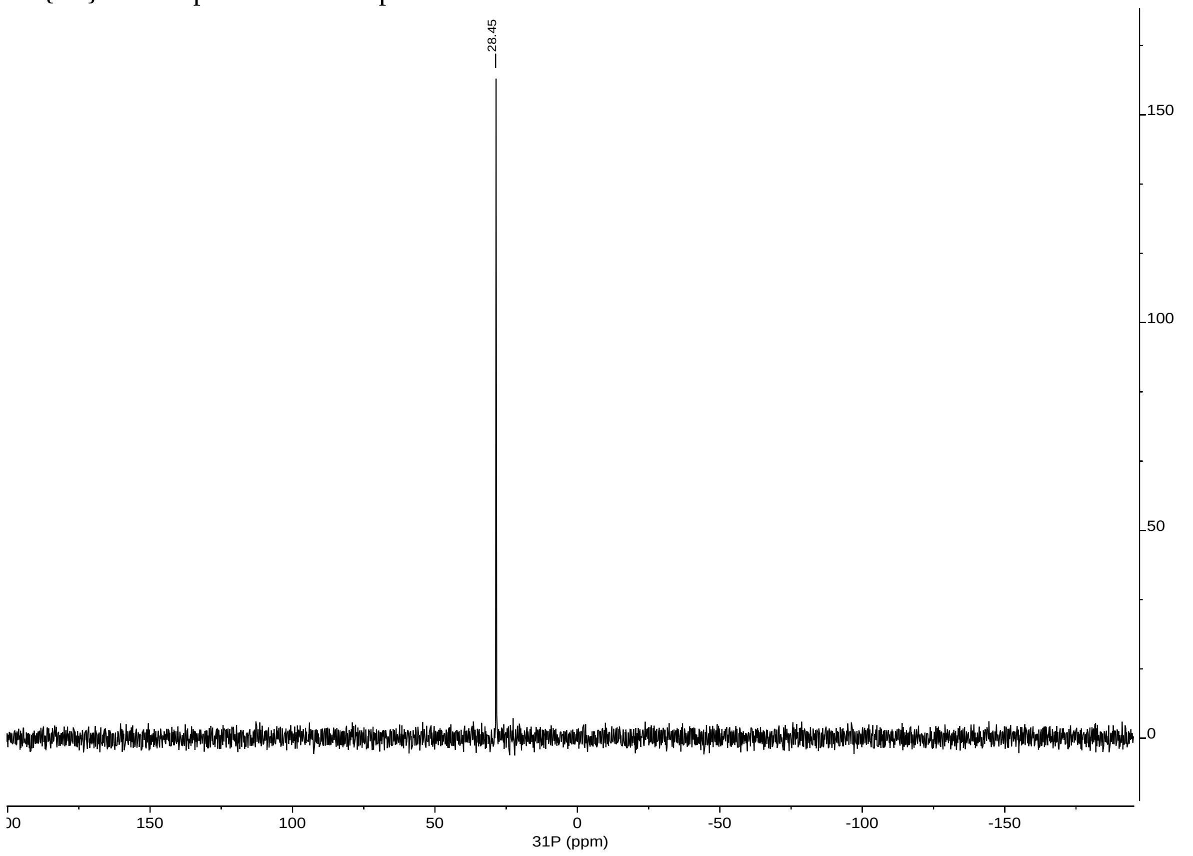

LC-MS chromatograms for compound **59**

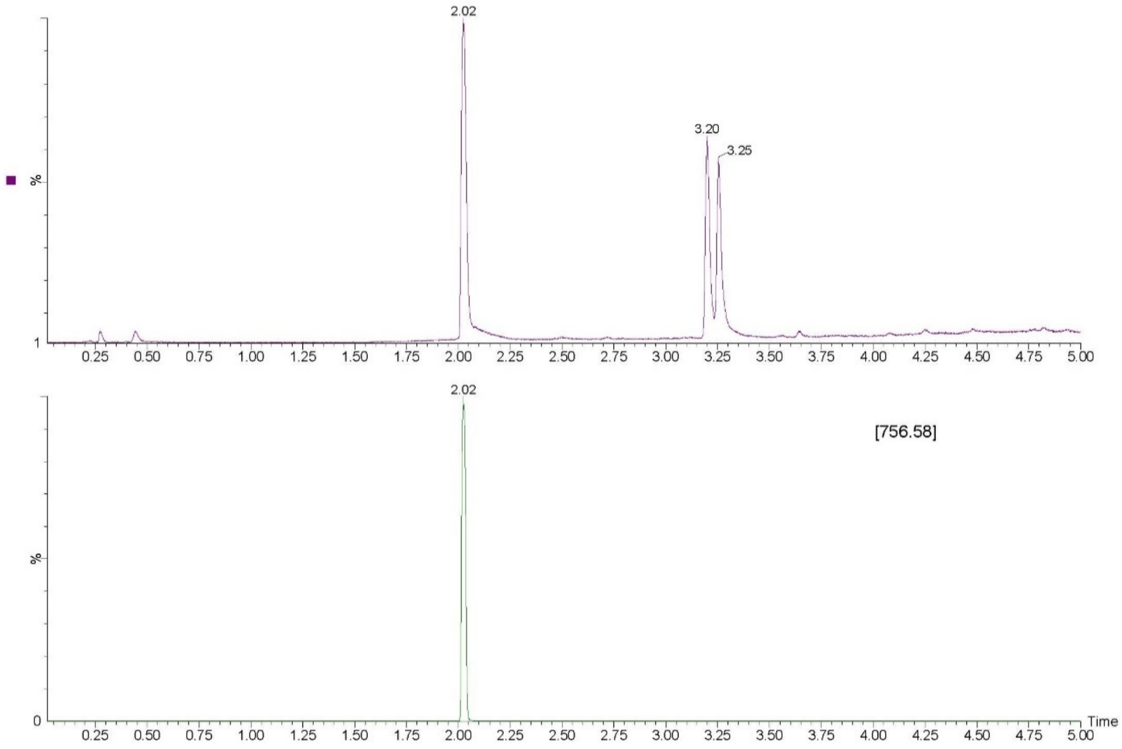

Dihexyl octane-1,8-diyl bis((2-(bis(3-guanidinopropyl)amino)ethyl)phosphonate) hexahydrochloride (60)

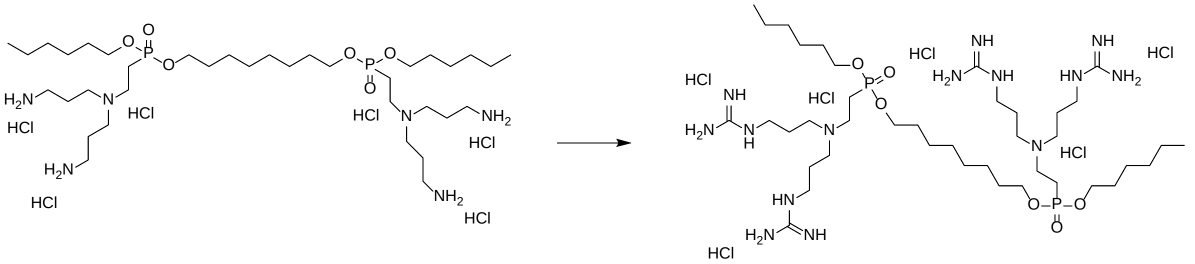

The title compound was prepared according to general method **F** from LPPO **59** (0.20 g, 0.21 mmol) in 84% yield (0.20 g, 0.17 mmol) as a white solid.

^{1}H NMR (401 MHz, CD_3OD): 4.21–4.06 (m, 8H, CH_2O), 3.52–3.39 (m, 4H, PCH_2CH_2), 3.39–3.32 (m, 16H, $CH_2CH_2CH_2NH$), 2.60–2.43 (m, 4H, PCH_2), 2.18–2.02 (m, 8H, CH_2CH$_2$NH), 1.79–1.66 (m, 8H, CH_2CH$_2$O), 1.50–1.26 (m, 20H, CH_3CH_2, $(CH_2)_2(CH_2)_2O$), 0.98–0.86 (m, 6H, CH_3).

^{13}C NMR (101 MHz, CD_3OD): 158.68 (*C*=NH), 68.21 (d, *J* = 6.8 Hz, *C*H$_2$O), 51.58 (*C*H$_2$NH), 48.63 (PCH$_2$*C*H$_2$), 39.67 (*C*H$_2$(CH$_2$)$_2$NH), 32.50 (CH$_3$CH$_2$*C*H$_2$), 31.57 (d, *J* = 6.1 Hz), 31.55 (d, *J* = 6.0 Hz, *C*H$_2$CH$_2$O), 30.20, 26.58, 26.32, 24.78 (*C*H$_2$CH$_2$NH$_2$), 23.64 (CH$_3$*C*H$_2$), 21.25 (d, *J* = 140.6 Hz, P*C*H$_2$), 14.40 (*C*H$_3$).

^{31}P{^{1}H} NMR (162 MHz, CD_3OD): 28.80.

IR ν_{max} (KBr) 3316 (s), 3260 (s), 3147 (s), 2955 (m), 2932 (m), 2858 (m), 1667 (vs), 1646 (s), 1619 (s, sh), 1467 (m), 1376 (w), 1218 (m), 1070–1000 (s), 723 (vw).

HR-MS(ESI^{+}): For $C_{40}H_{92}N_{14}O_6P_2$ $(M+2H)^{2+}$ *m/z* calculated 463.33943, found 463.33887.

^{1}H NMR spectrum of compound **60**

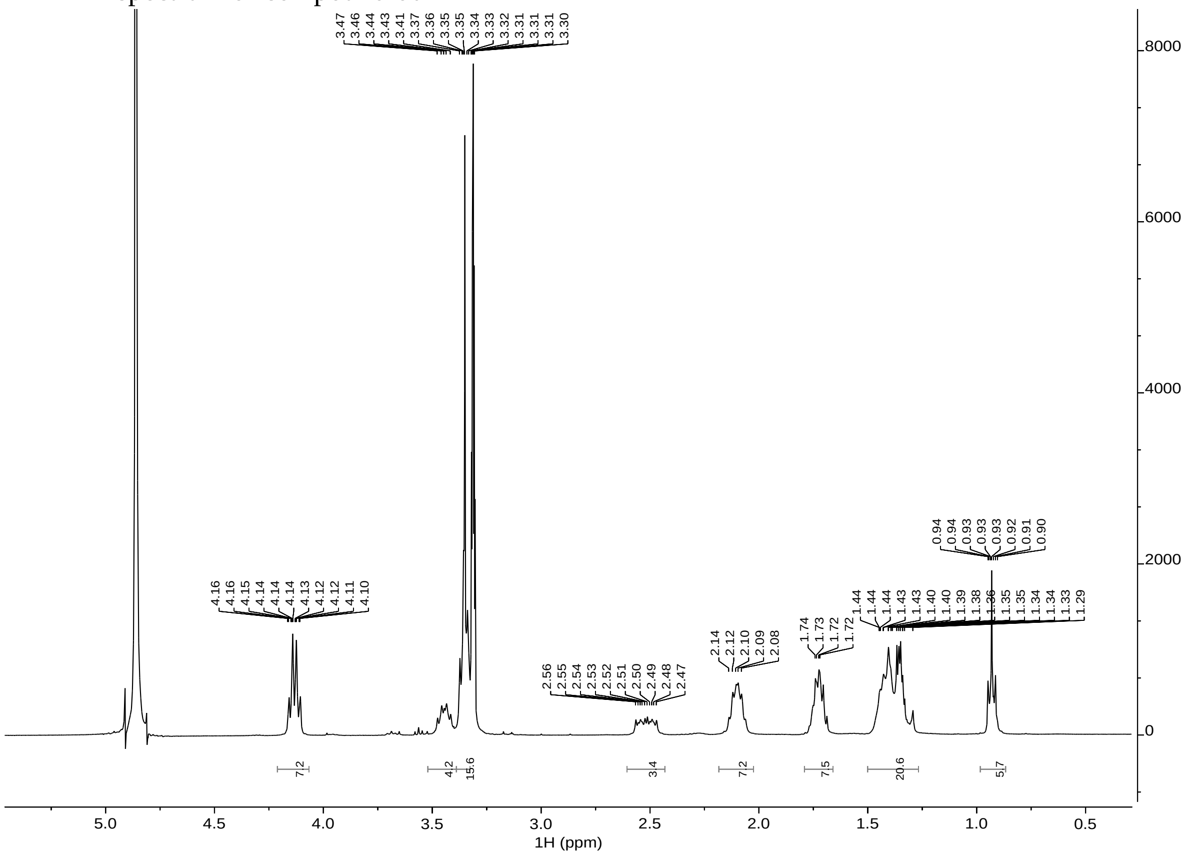

^{13}C NMR spectrum of compound **60**

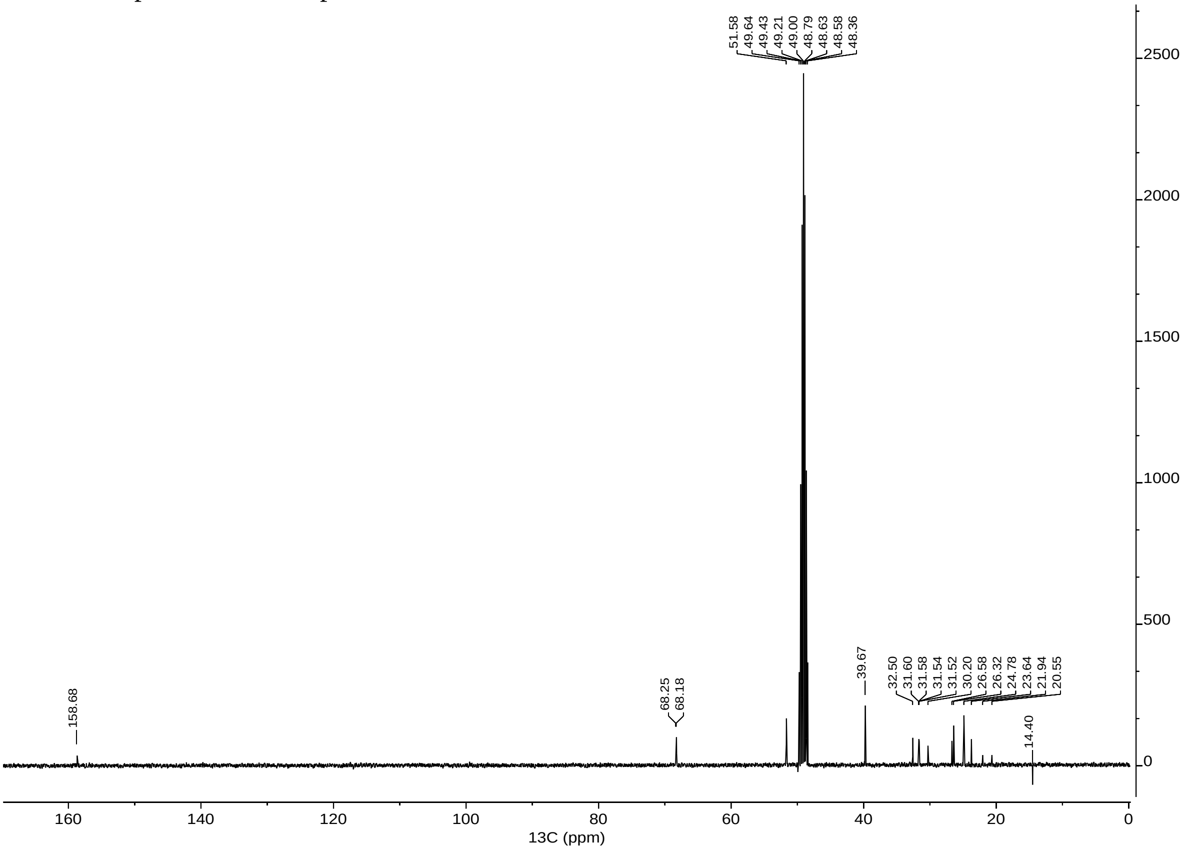

$^{31}P\{^{1}H\}$ NMR spectrum of compound **60**

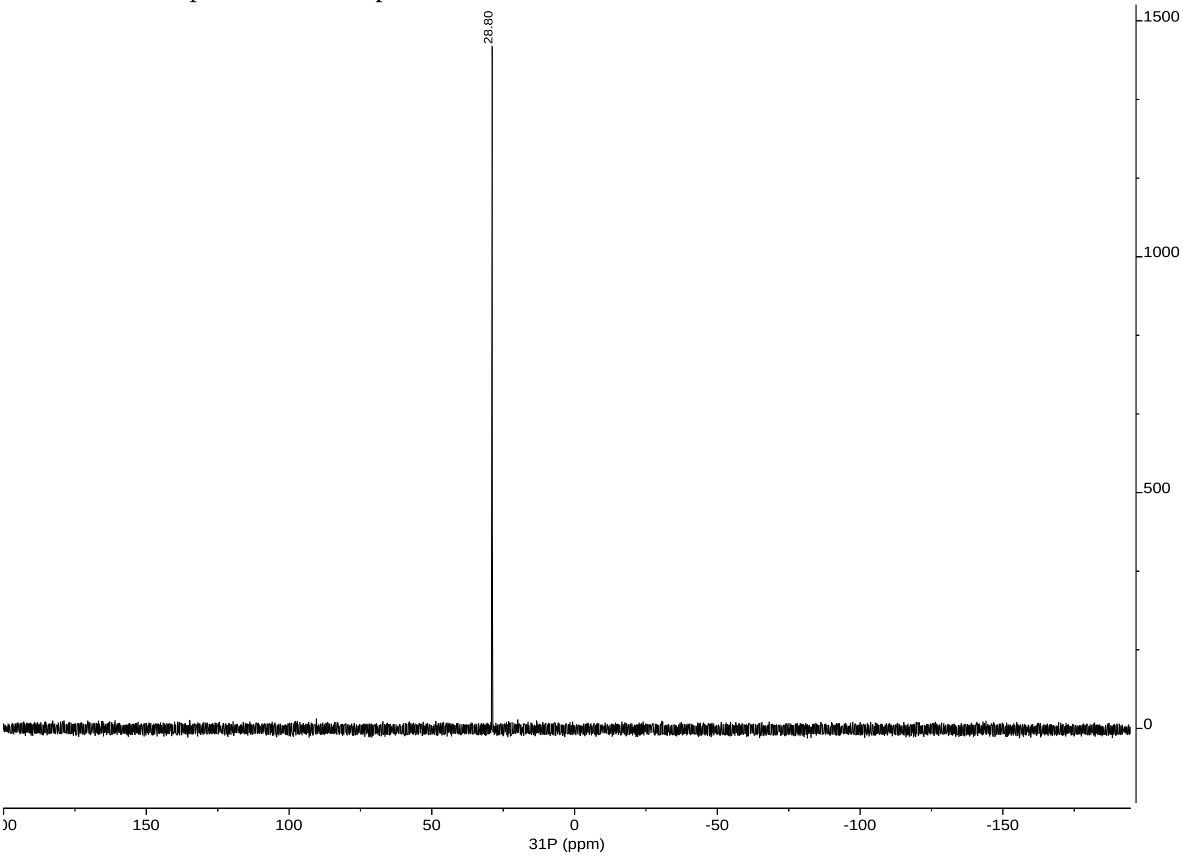

LC-MS chromatograms for compound **60**

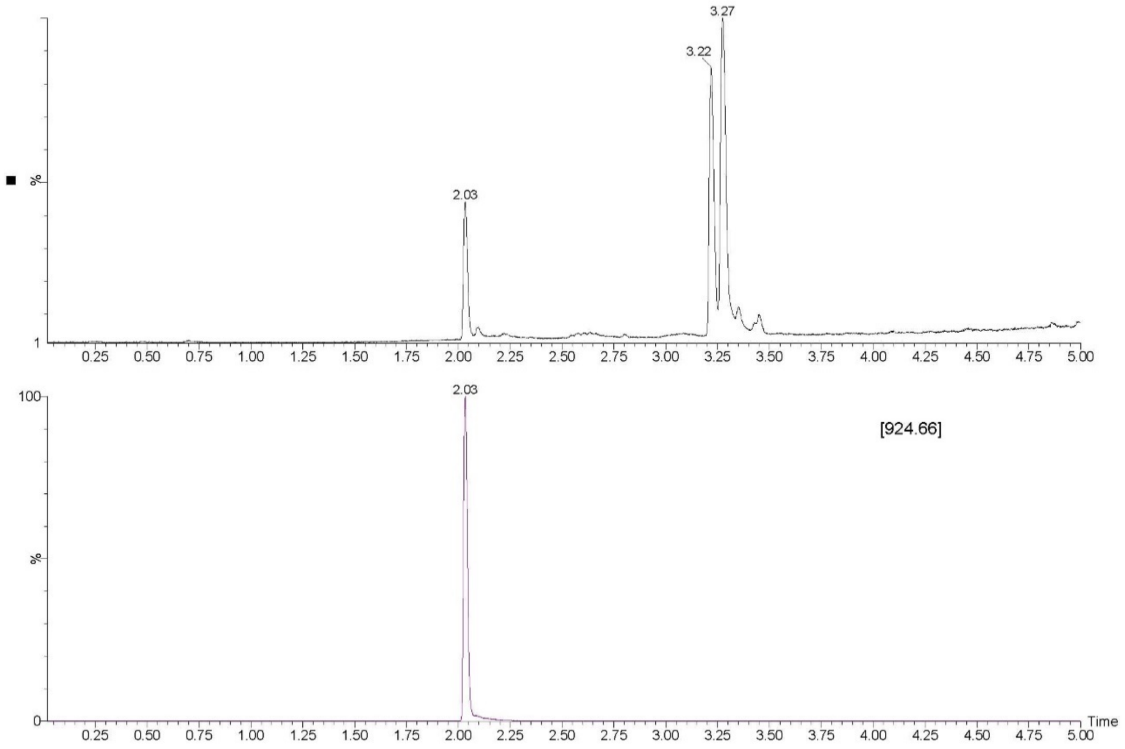

Dihexyl octane-1,8-diyl bis((2-(bis(2-aminoethyl)amino)ethyl)phosphonate) hexahydrochloride (61)

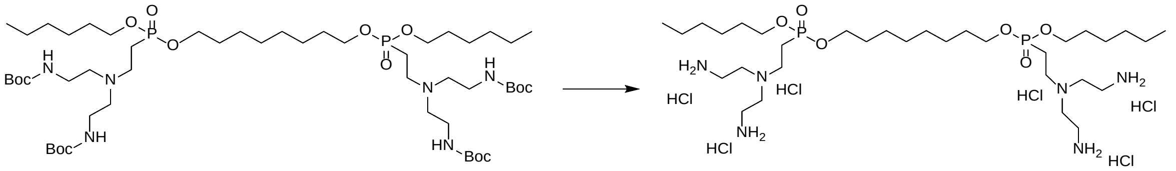

The title compound was prepared according to general method **E** from Boc derivative **S114** (1.13 g, 0.92 mmol) in 93% yield (0.79 g, 0.86 mmol) as a white solid.

^{1}H NMR (401 MHz, CD_3OD): 4.14–4.03 (m, 8H, CH_2O), 3.22–3.11 (m, 8H, CH_2NH_2), 3.05–2.88 (m, 12H, CH_2N), 2.28–2.14 (m, 4H, PCH_2), 1.79–1.64 (m, 8H, CH_2CH_2O), 1.46–1.23 (m, 20H, CH_3CH_2, $(CH_2)_2(CH_2)_2O$), 0.99–0.88 (m, 6H, CH_3).

^{13}C NMR (101 MHz, CD_3OD): 67.73 (d, J = 7.0 Hz), 67.72 (d, J = 6.7 Hz, CH$_2$O), 51.52 (CH$_2$CH$_2$NH$_2$), 47.66 (PCH$_2$$CH_2$), 37.76 ($CH_2NH_2$), 31.62 (d, J = 6.0 Hz), 31.58 (d, J = 6.1 Hz, CH$_2$CH$_2$O), 32.53, 30.27, 26.64, 26.36, 23.65 (CH$_3$$CH_2$, ($CH_2$)$_2$(CH$_2$)$_2$O), 22.71 (d, J = 139.2 Hz, PCH$_2$), 14.39 (CH$_3$).

^{31}P{^{1}H} NMR (162 MHz, CD_3OD): 31.90.

IR ν_{max} 2926 (m), 2793 (s), 2677 (m), 2617 (m), 2542 (s), 2438 (m), 1514 (m), 1467 (m), 1390 (m), 1228 (s), 1050 (s), 990 (s).

HR-MS(ESI$^+$): For $C_{32}H_{76}O_6N_6P_2$ $(M+2H)^{2+}$ $m/2z$ calculated 351.26453, found 351.26459.

^{1}H NMR spectrum of compound **61**

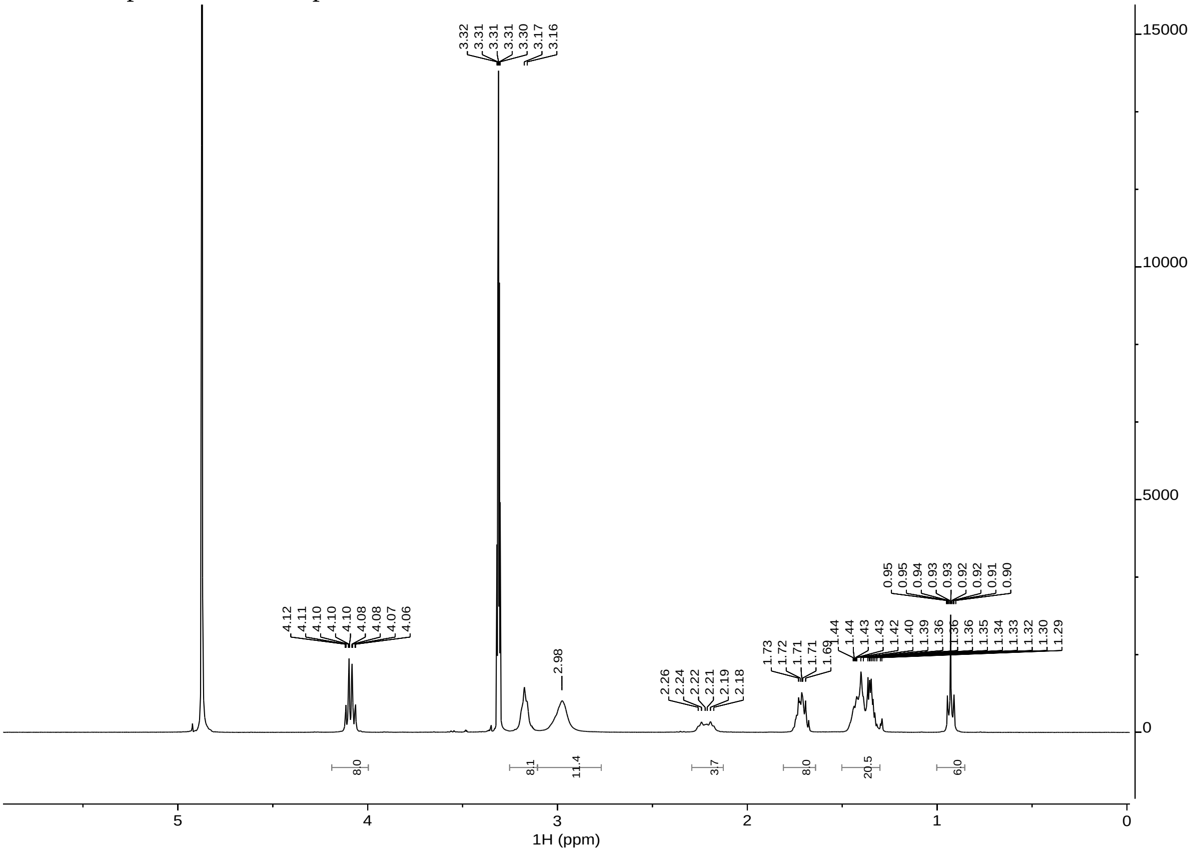

^{13}C NMR spectrum of compound **61**

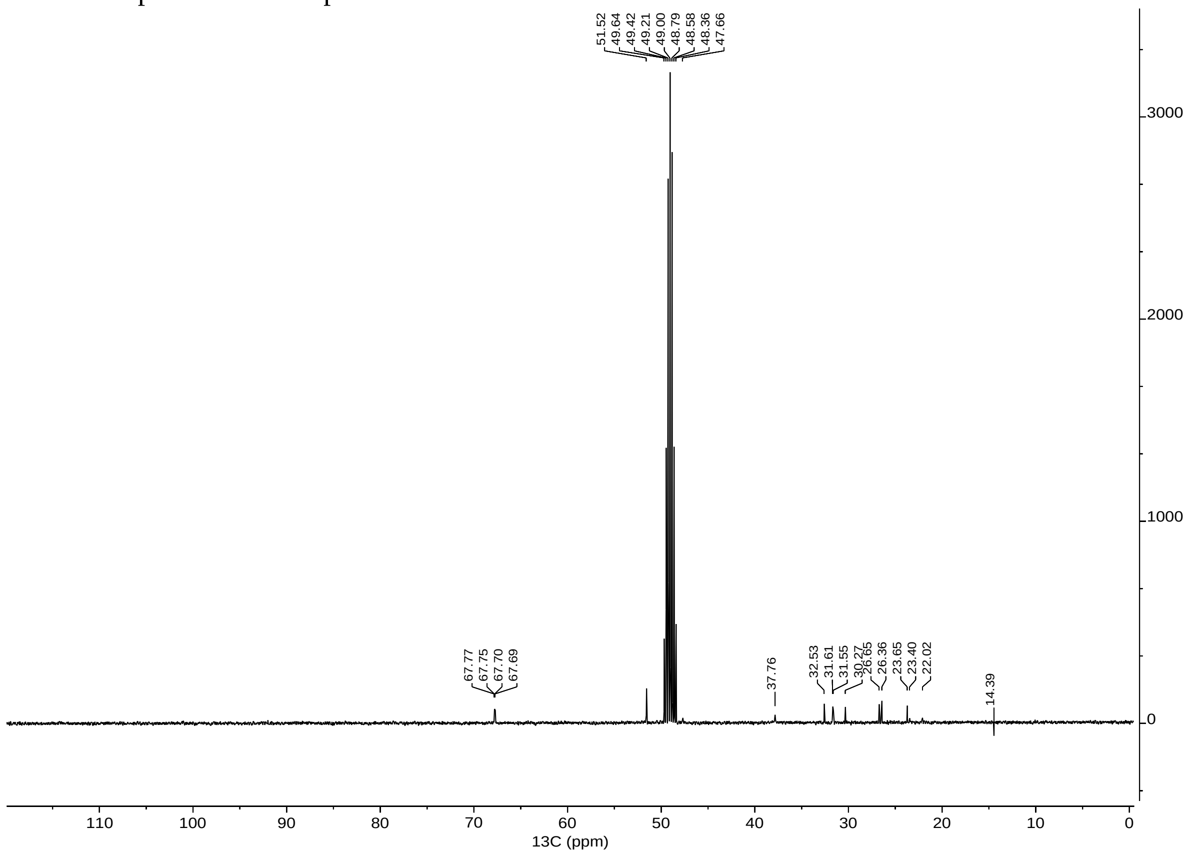

^{31}P{^{1}H} NMR spectrum of compound **61**

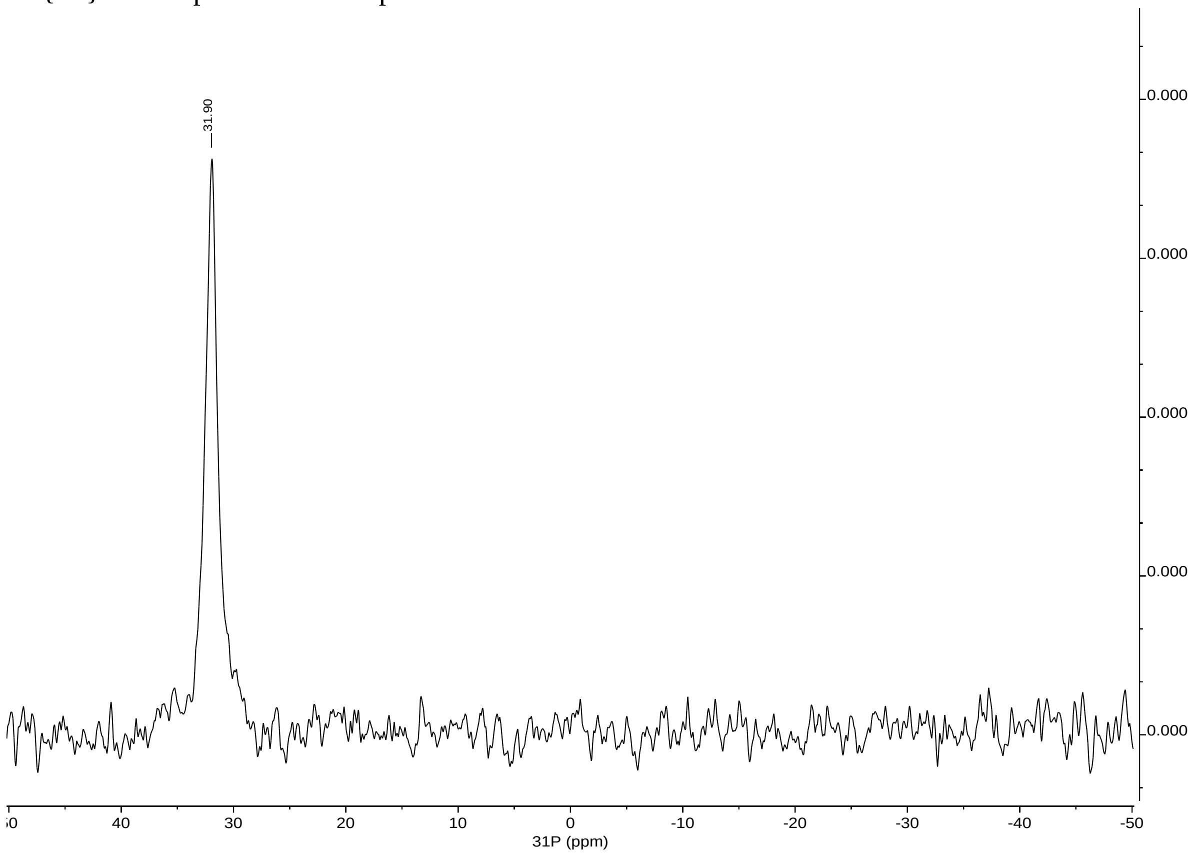

LC-MS chromatograms for compound **61**

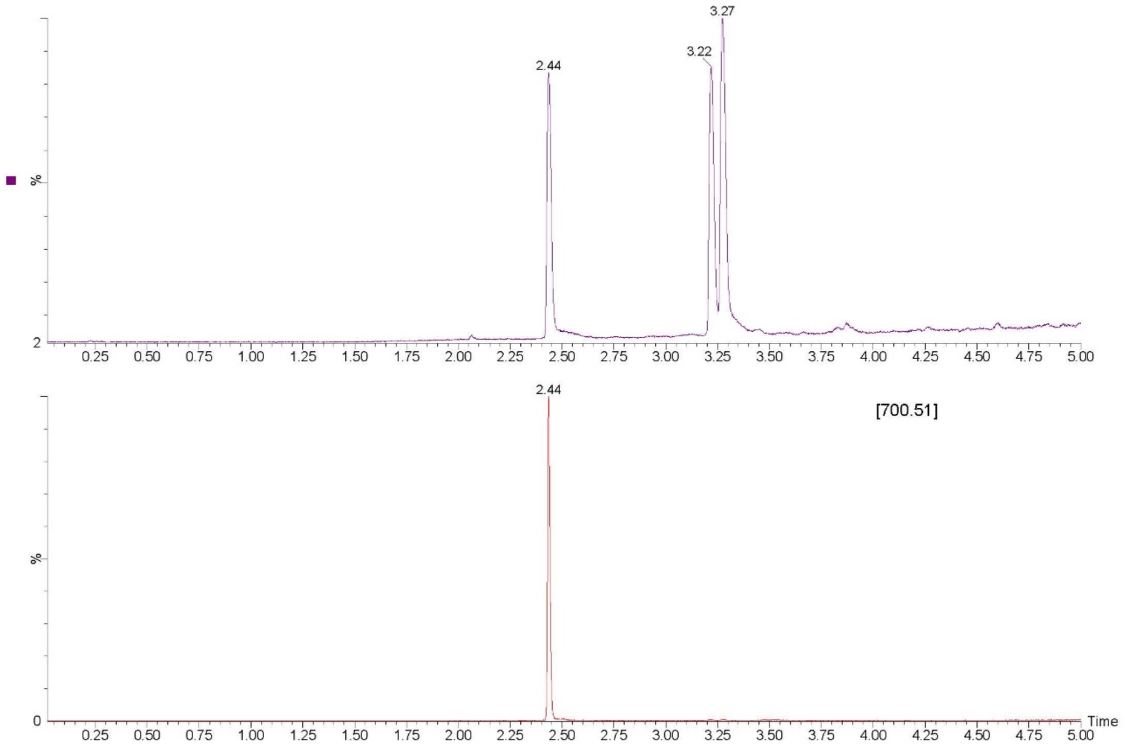

Bis((*Z*)-hept-3-en-1-yl) octane-1,8-diyl bis((2-(bis(3-aminopropyl)amino)ethyl)phosphonate) hexahydrochloride (62)

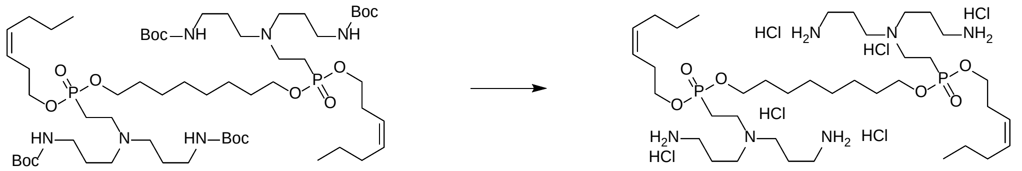

The title compound was prepared according to general method **E** from Boc derivative **S115** (0.65 g, 0.55 mmol) in 96% yield (0.54 g, 0.54 mmol) as a white solid.

^{1}H NMR (401 MHz, CD_3OD): 5.63–5.51 (m, 2H, $CH_3(CH_2)_2CH$), 5.49–5.39 (m, 2H, $CH(CH_2)_2O$), 4.20–4.05 (m, 8H, CH_2O), 3.52–3.34 (m, 12H, CH_2N), 3.10 (t, 8H, *J* = 7.5 Hz, CH_2NH_2), 2.63–2.42 (m, 8H, $CHCH_2CH_2O$, PCH_2), 2.27–2.13 (m, 8H, $CH_2CH_2NH_2$), 2.12–2.02 (m, 4H, $CH_3CH_2CH_2$), 1.73 (p, 4H, *J* = 6.8 Hz, $CH_2CH_2CH_2O$), 1.49–1.33 (m, 12H, CH_3CH_2, $(CH_2)_2(CH_2)_2O$), 0.93 (t, 6H, *J* = 7.4 Hz, CH_3).

^{13}C NMR (101 MHz, CD_3OD): 134.15 ($CH_3(CH_2)_2CH$), 125.18 ($CH(CH_2)_2O$), 68.23 (d, *J* = 6.8 Hz, $(CH_2)_2CH_2O$), 67.61 (d, *J* = 6.9 Hz, $CHCH_2CH_2O$), 51.06 ($CH_2(CH_2)_2NH_2$), 48.83 (PCH_2CH_2), 37.87 (CH_2NH_2), 31.55 (d, *J* = 6.0 Hz, $CH_2CH_2CH_2O$), 30.45 ($CH_3CH_2CH_2$), 30.18 (d, *J* = 1.2 Hz, $CH_2(CH_2)_2O$), 29.67 (d, *J* = 5.9 Hz, $CHCH_2CH_2O$), 26.55 ($CH_2(CH_2)_3O$), 23.79 (CH_3CH_2), 23.28 (CH_2CH_2NH), 21.25 (d, *J* = 140.5 Hz, PCH_2), 14.15 (CH_3).

^{31}P{^{1}H} NMR (162 MHz, CD_3OD): 28.67.

IR ν_{max} (KBr) 3100–2500 (vs, br), 3007 (s, sh), 2957 (vs), 2932 (s), 2871 (s), 2028 (w), 1657 (vw), 1605 (w), 1466 (m), 1228 (m), 1004 (s).

HR-MS(ESI$^+$): For $C_{38}H_{84}O_6N_6P_2$ $(M+2H)^{2+}$ *m*/2*z* calculated 391.29583, found 391.29558.

^{1}H NMR spectrum of compound **62**

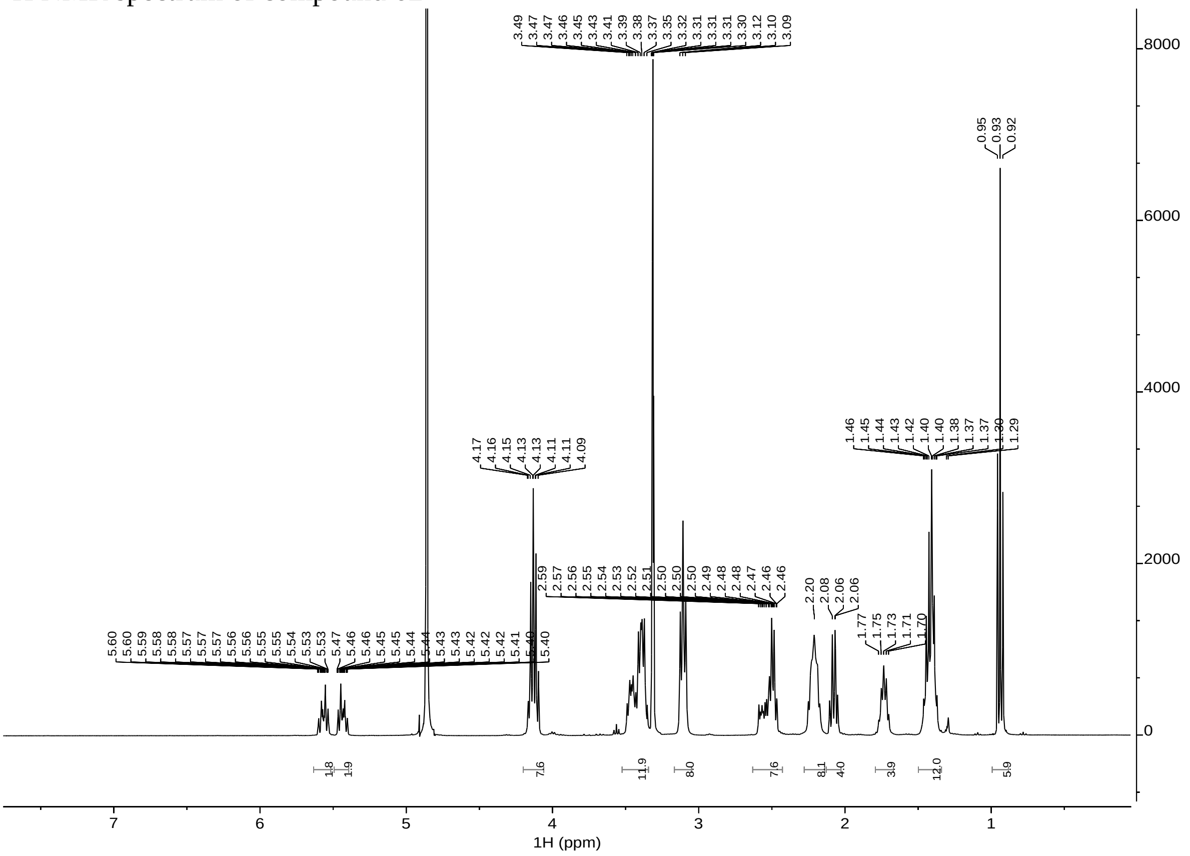

^{13}C NMR spectrum of compound **62**

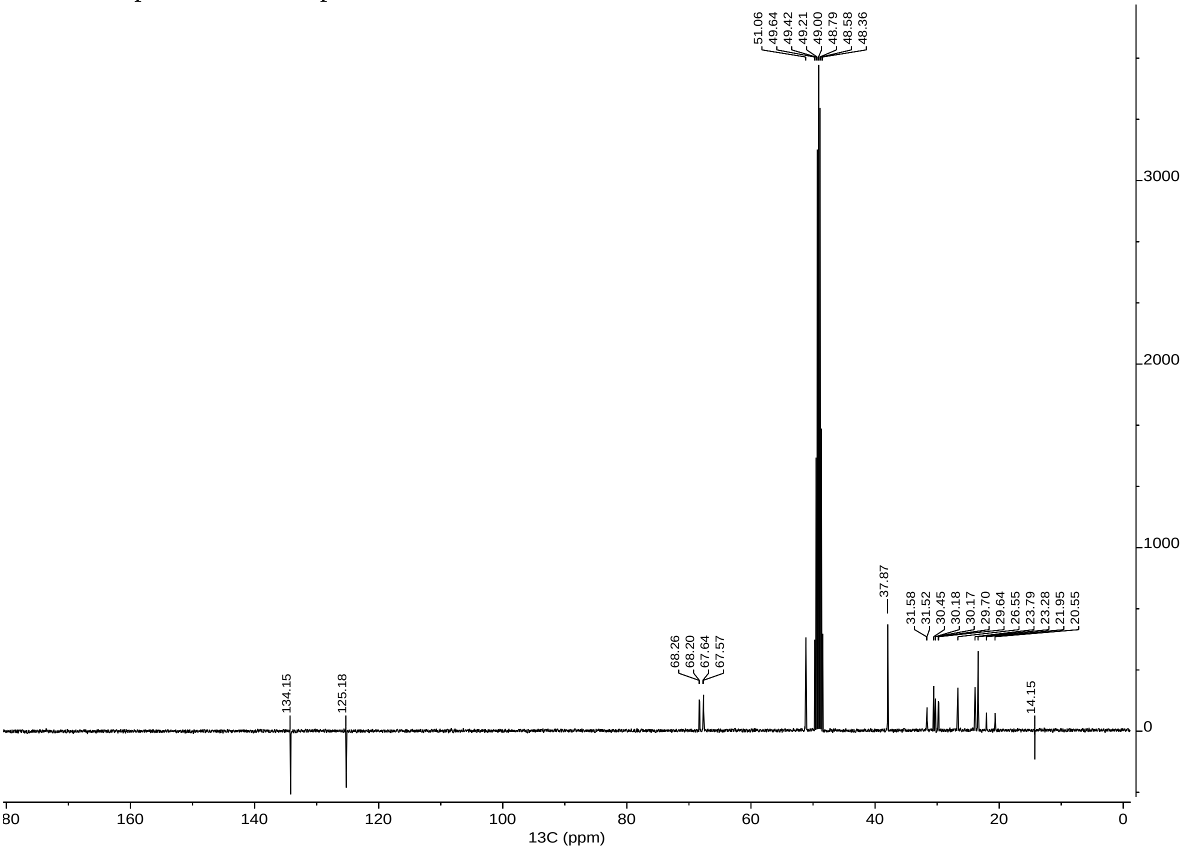

^{31}P{^{1}H} NMR spectrum of compound **62**

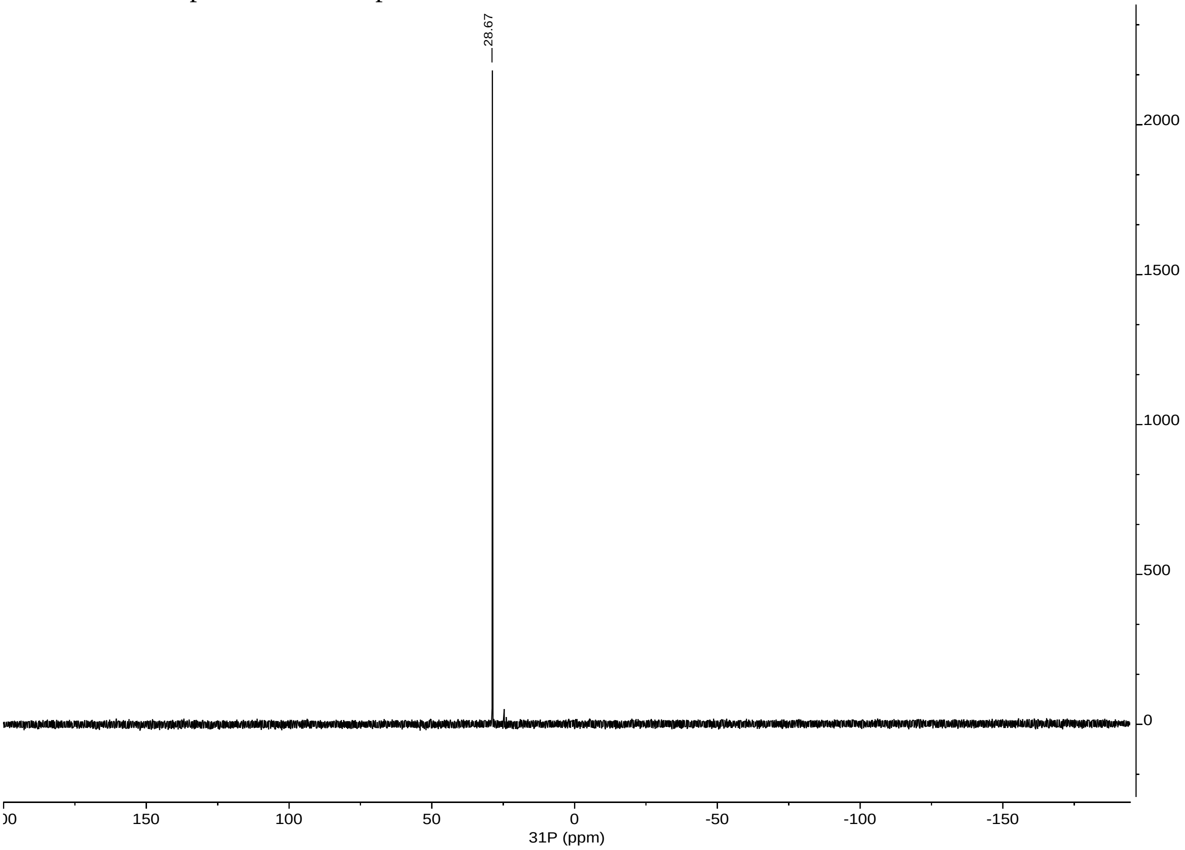

LC-MS chromatograms for compound **62**

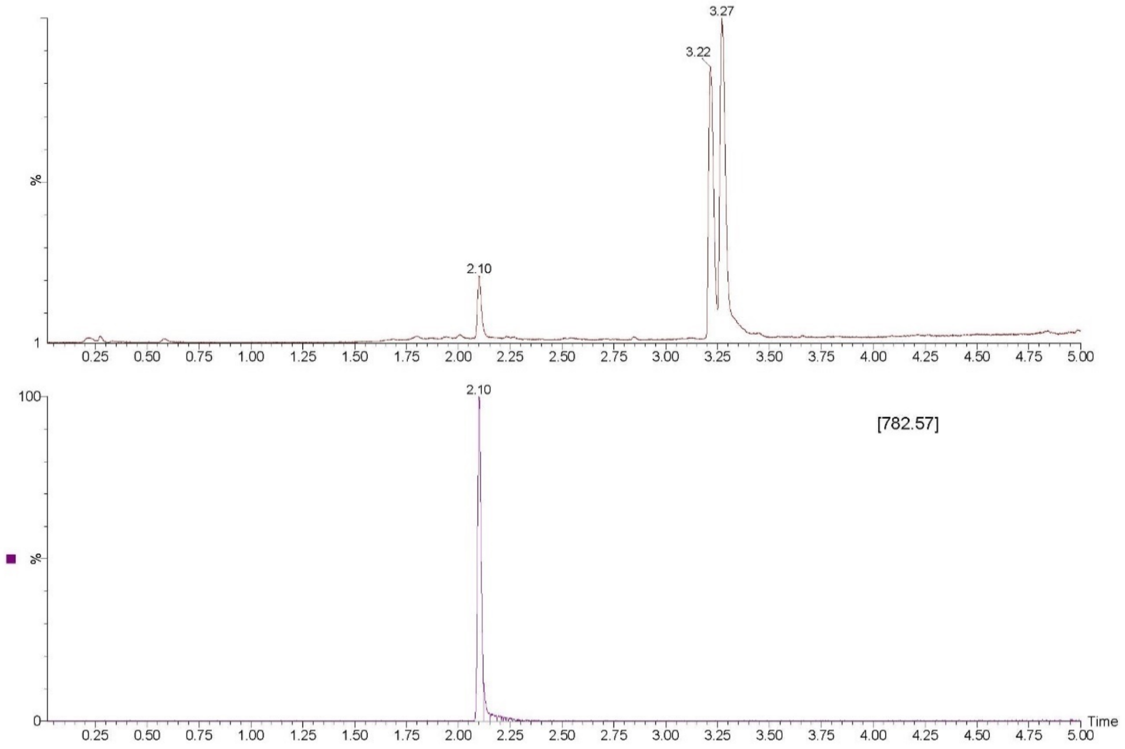

Octane-1,8-diyl dioctyl bis((2-(bis(3-aminopropyl)amino)ethyl)phosphonate) hexahydrochloride (63)

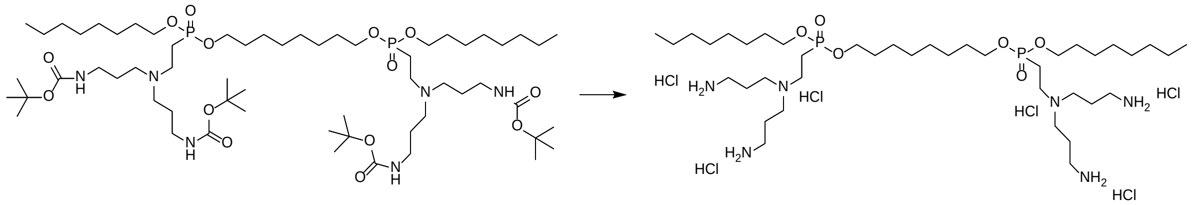

The title compound was prepared according to general method **E** from Boc derivative **S116** (0.90 g, 0.75 mmol) in 92% yield (0.64 g, 0.69 mmol) as a white solid.

^{1}H NMR (400 MHz, CD_3OD): 4.16–4.10 (CH_2O), 3.49–3.45 (m, 4H, PCH_2CH_2), 3.41–3.36 (m, 8H, C$H_2(CH_2)_2NH_2$), 3.11 (t, 8H, J = 7.6 Hz, CH_2NH_2), 2.57–2.48 (m, 4H, PCH_2), 2.25–2.16 (m, 8H, C$H_2CH_2NH_2$), 1.77–1.61 (m, 8H, CH_2CH_2O), 1.42–1.30 (m, 28H, $(CH_2)_2(CH_2)_2O$, $CH_3(CH_2)_3$), 0.92–0.89 (m, 6H, CH_3).

^{13}C NMR (101 MHz, CD_3OD): 68.28 (d, J = 6.9 Hz), 68.25 (d, J = 6.8 Hz, CH$_2$O), 51.07 (CH$_2(CH_2)_2NH_2$), 48.94 (PCH_2CH_2), 37.84 (CH$_2NH_2$), 32.94 ($CH_3CH_2CH_2$), 31.57 (d, J = 5.8 Hz), 31.54 (d, J = 6.0 Hz, CH_2CH_2O), 30.32, 30.22, 30.17 (d, J = 1.7 Hz), 26.60, 26.54, 23.68 (CH_3CH_2), 23.24 ($CH_2CH_2NH_2$), 21.25 (d, J = 139.8 Hz, PCH_2), 14.45 (CH_3).

^{31}P{^{1}H} NMR (162 MHz, CD_3OD): 28.52.

IR ν_{max} (KBr) 3000–2500 (vs, vbr), 2955 (s), 2925 (vs), 2855 (s), 2025 (w), 1599 (w), 1467 (m), 1378 (w), 1227 (m), 1015 (m), 996 (m), 721 (vw).

HR-MS(ESI^{+}): For $C_{40}H_{92}N_6O_6P_2$ (M+2H)$^{2+}$ $m/2z$ calculated 407.32713, found 407.32687.

^{1}H NMR spectrum of compound **63**

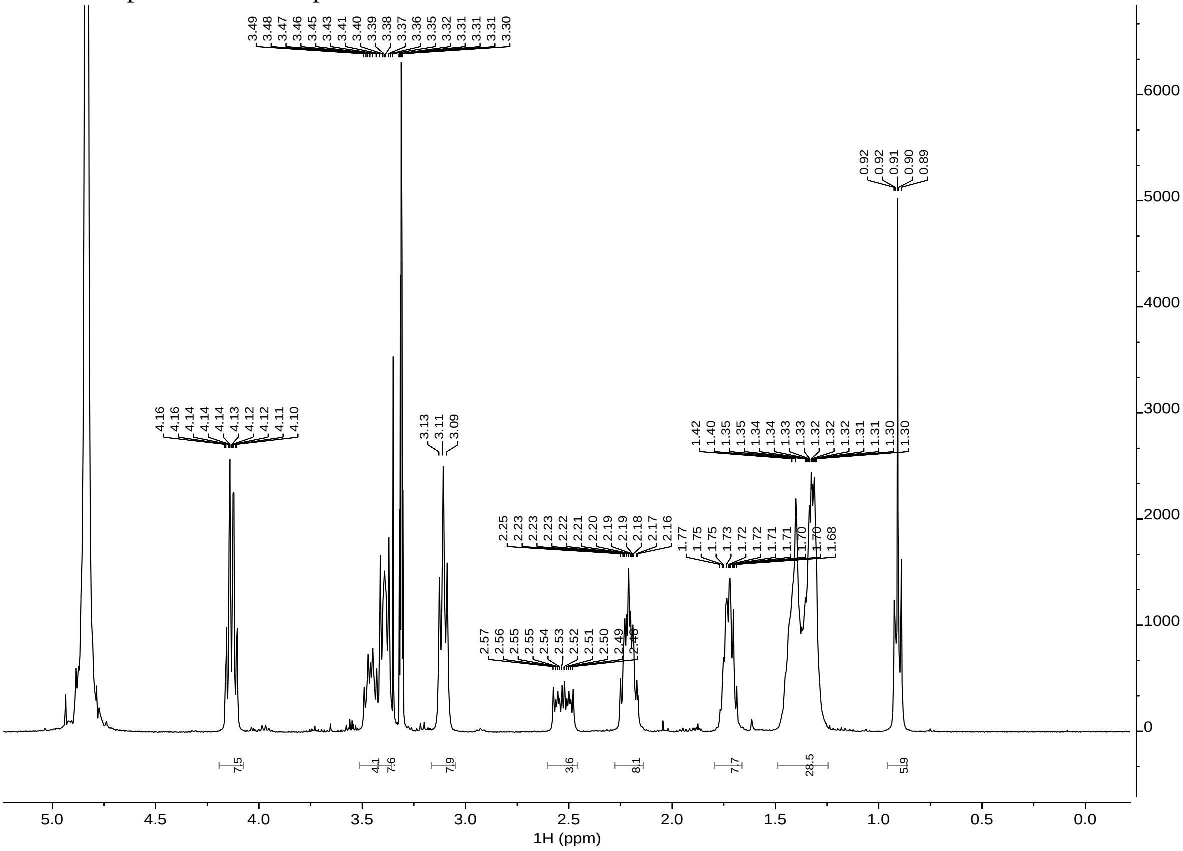

^{13}C NMR spectrum of compound **63**

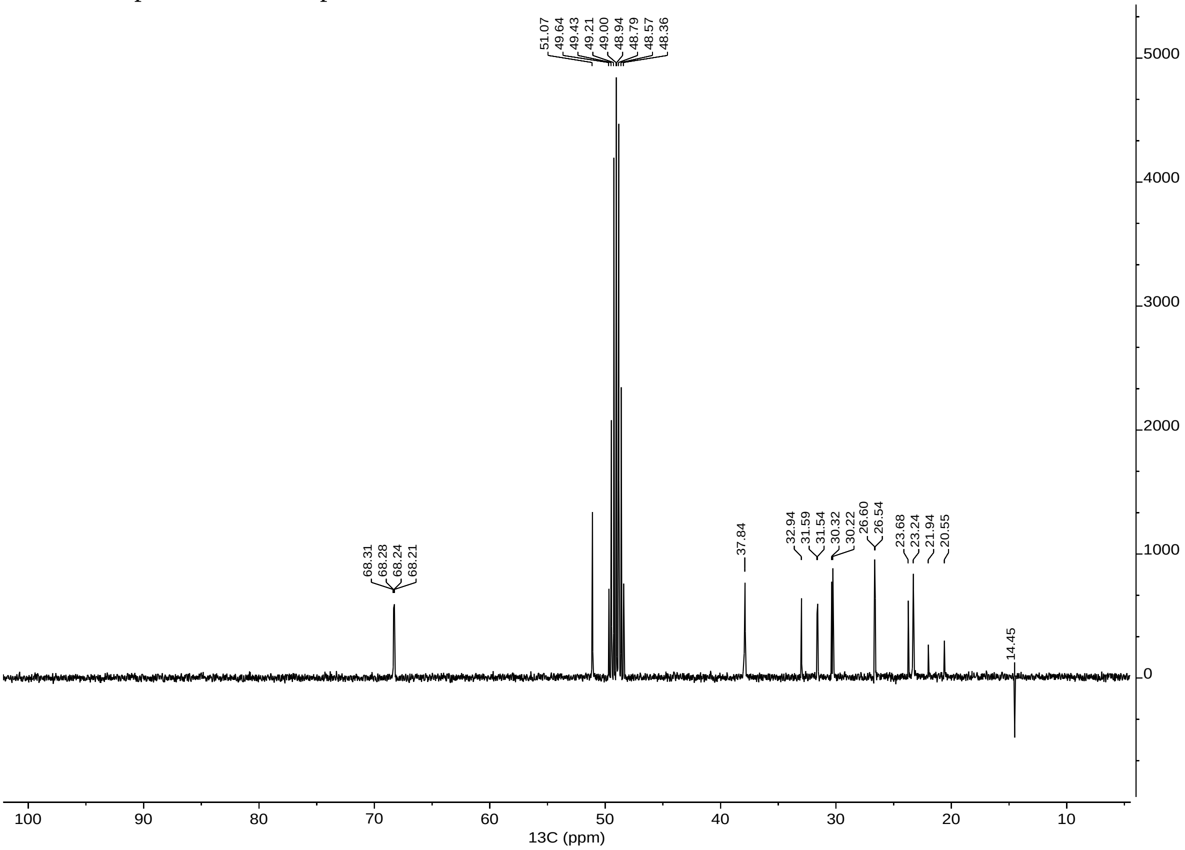

^{31}P{^{1}H} NMR spectrum of compound **63**

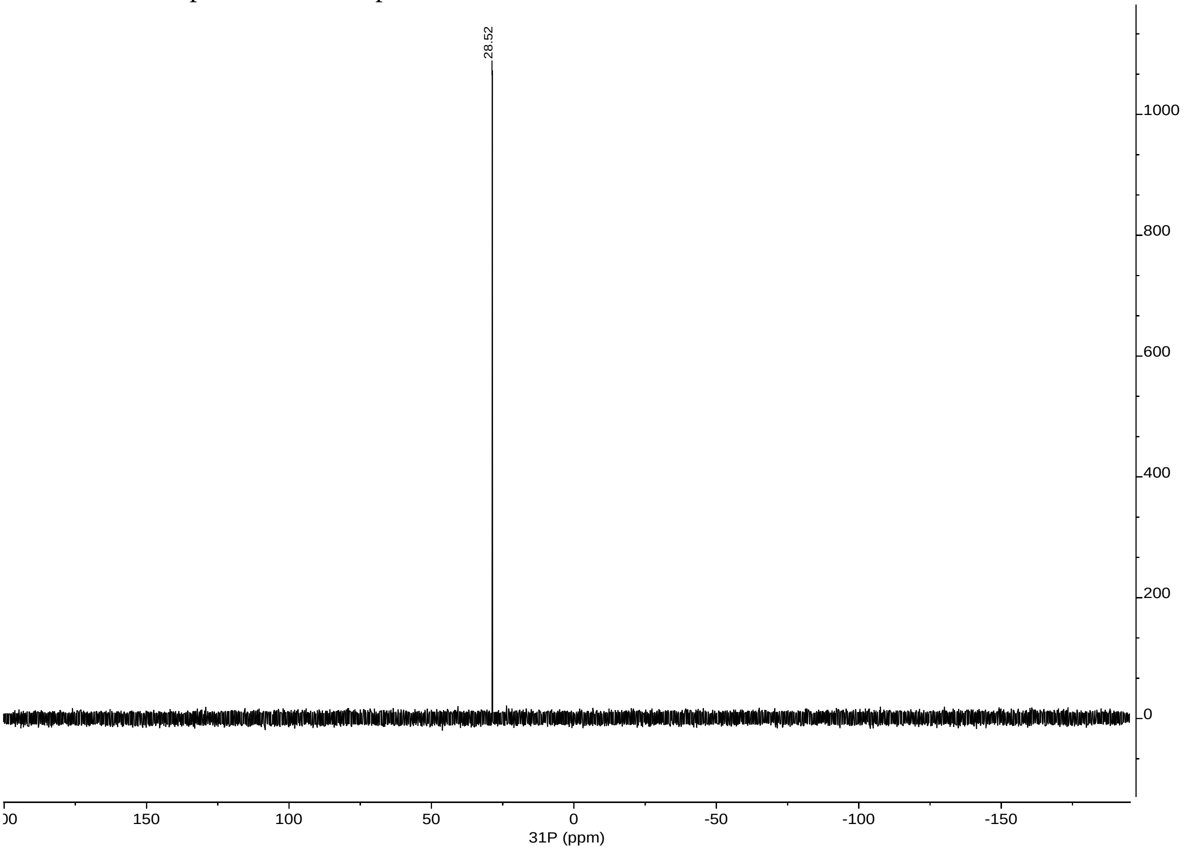

LC-MS chromatograms for compound **63**

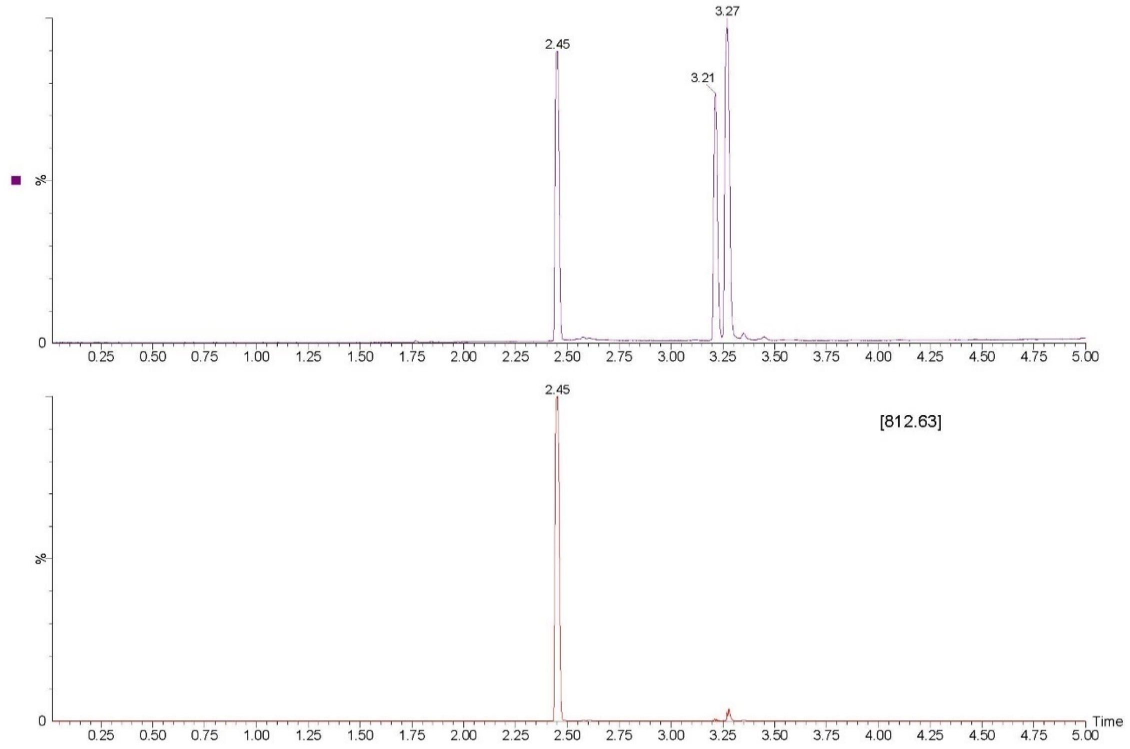

Octane-1,8-diyl dioctyl bis((2-(bis(2-aminoethyl)amino)ethyl)phosphonate) hexahydrochloride (64)

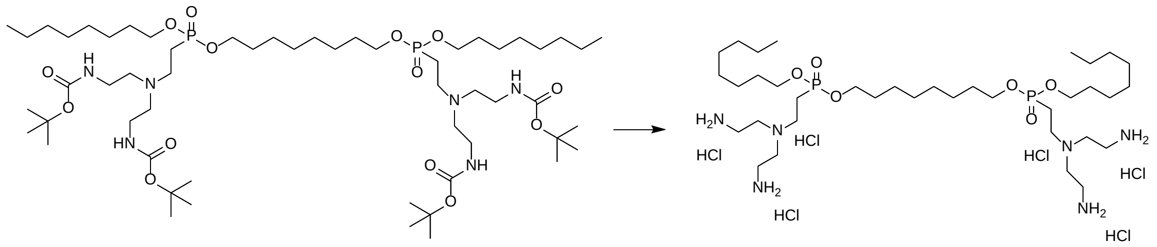

The title compound was prepared according to general method **E** from Boc derivative **S117** (0.60 g, 0.52 mmol) in 93% yield (0.47 g, 0.48 mmol) as a white solid.

^{1}H NMR (401 MHz, CD_3OD): 4.16–4.05 (m, 8H, CH_2O), 3.29–3.23 (m, 8H, CH_2NH_2), 3.23–3.05 (m, 12H, CH_2N), 2.40–2.25 (m, 4H, PCH_2), 1.77–1.66 (m, 8H, CH_2CH_2O), 1.49–1.27 (m, 28H, CH_3(CH_2)$_3$, (CH_2)$_2$(CH_2)$_2$O), 0.94–0.87 (m, 6H, CH_3).

^{13}C NMR (101 MHz, CD_3OD): 67.89 (d, J = 6.8 Hz), 67.86 (d, J = 6.8 Hz, CH$_2$O), 51.50 (CH$_2CH_2NH_2$), 48.24 (d, J = 4.6 Hz, P$CH_2$$CH_2$), 36.84 ($CH_2NH_2$), 32.99 ($CH_3CH_2$$CH_2$), 31.61 (d, J = 5.9 Hz), 31.59 (d, J = 6.0 Hz, CH$_2CH_2$O), 30.37, 30.28, 30.22, 26.67, 26.62, 23.72 ($CH_3$$CH_2$), 22.34 (d, J = 139.8 Hz, PCH$_2$), 14.46 CH$_3$).

^{31}P{^{1}H} NMR (162 MHz, CD_3OD): 32.43.

IR ν_{max} (KBr) 3300–2500 (s, br), 2956 (s), 2928 (s), 2856 (s), 1591 (m), 1468 (m), 1394 (w), 1378 (w), 1209 (m), 1071 (m), 1014 (s), 724 (w).

HR-MS(ESI^{+}): For $C_{36}H_{84}N_6O_6P_2$ $(M+2H)^{2+}$ $m/2z$ calculated 379.29583, found 379.29571.

^{1}H NMR spectrum of compound **64**

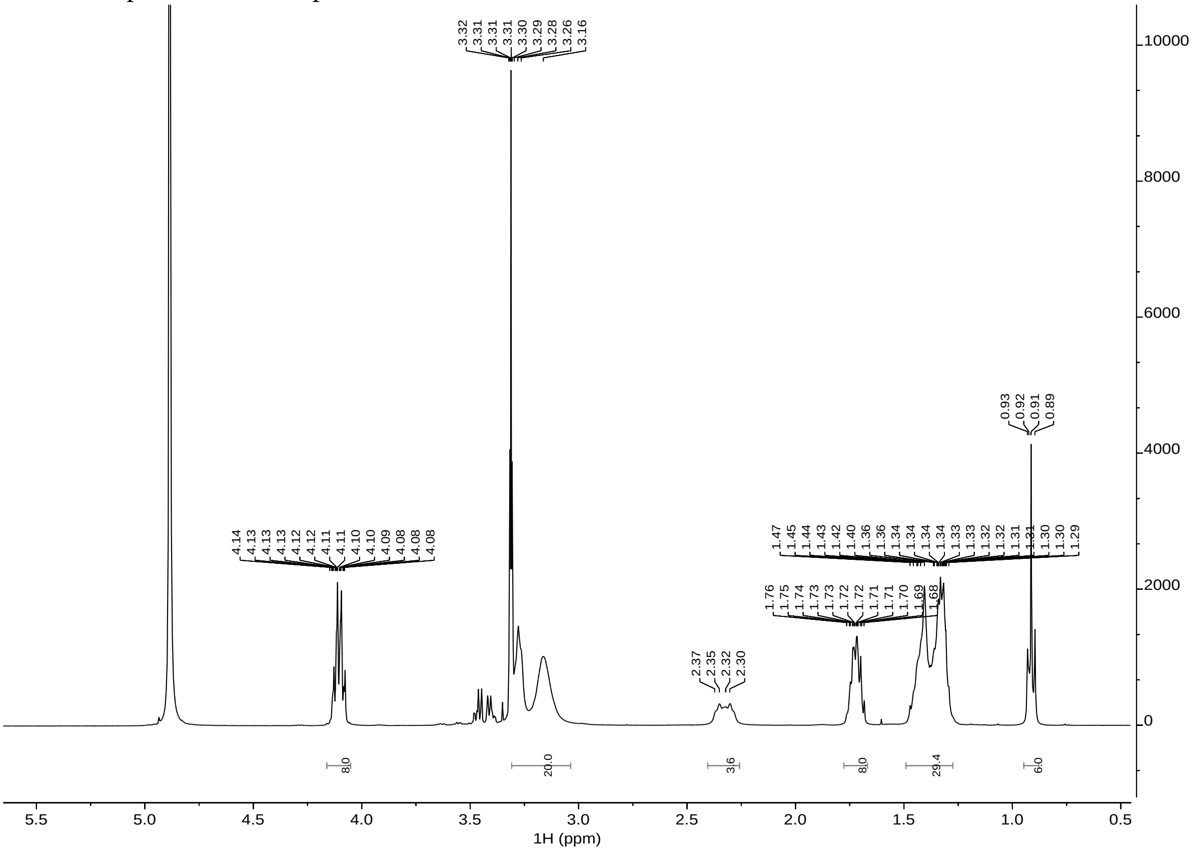

^{13}C NMR spectrum of compound **64**

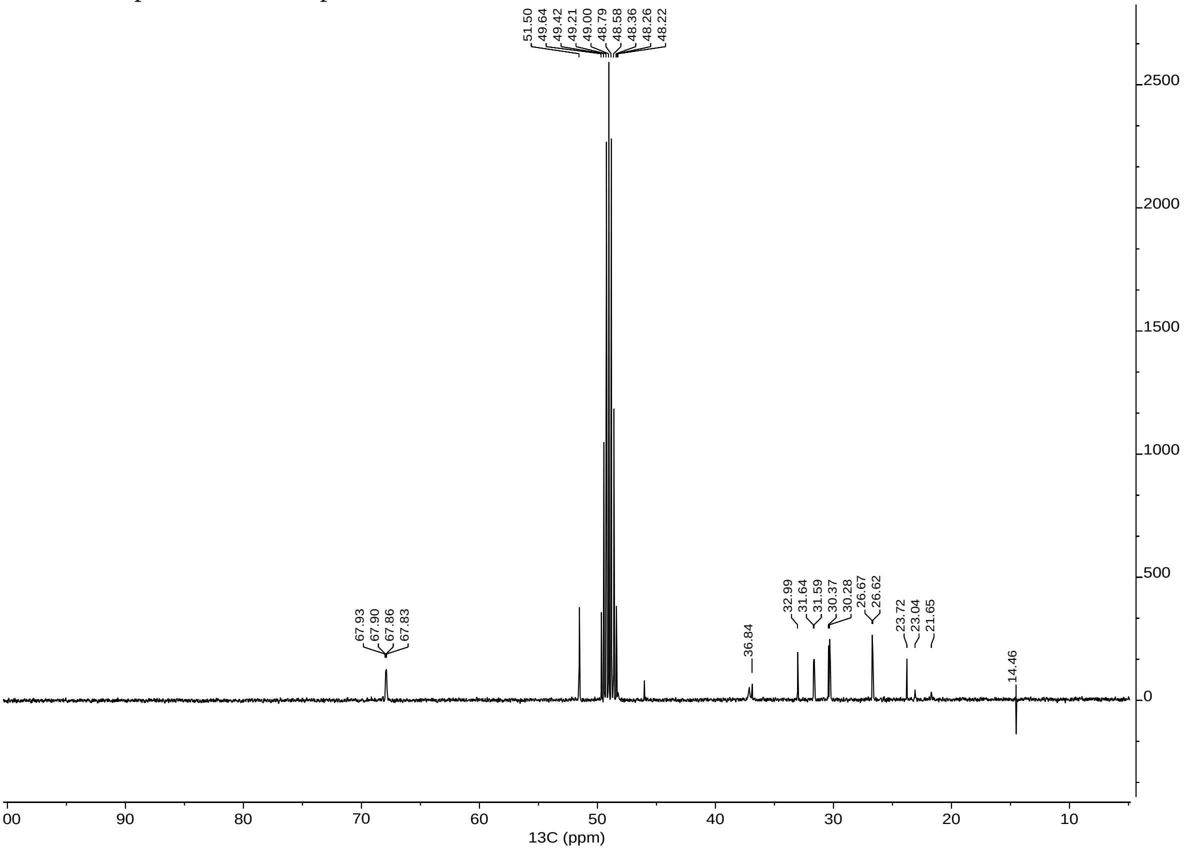

$^{31}P\{^1H\}$ NMR spectrum of compound **64**

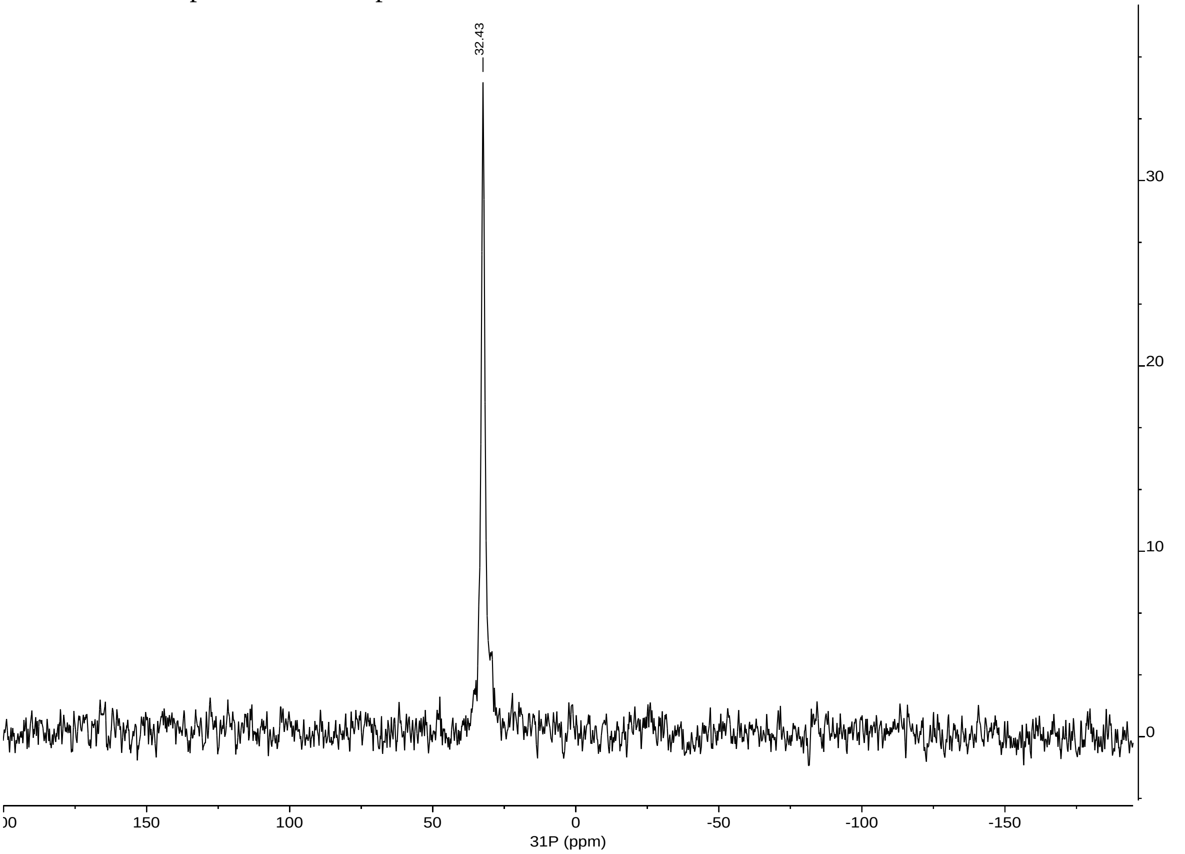

LC-MS chromatograms for compound **64**

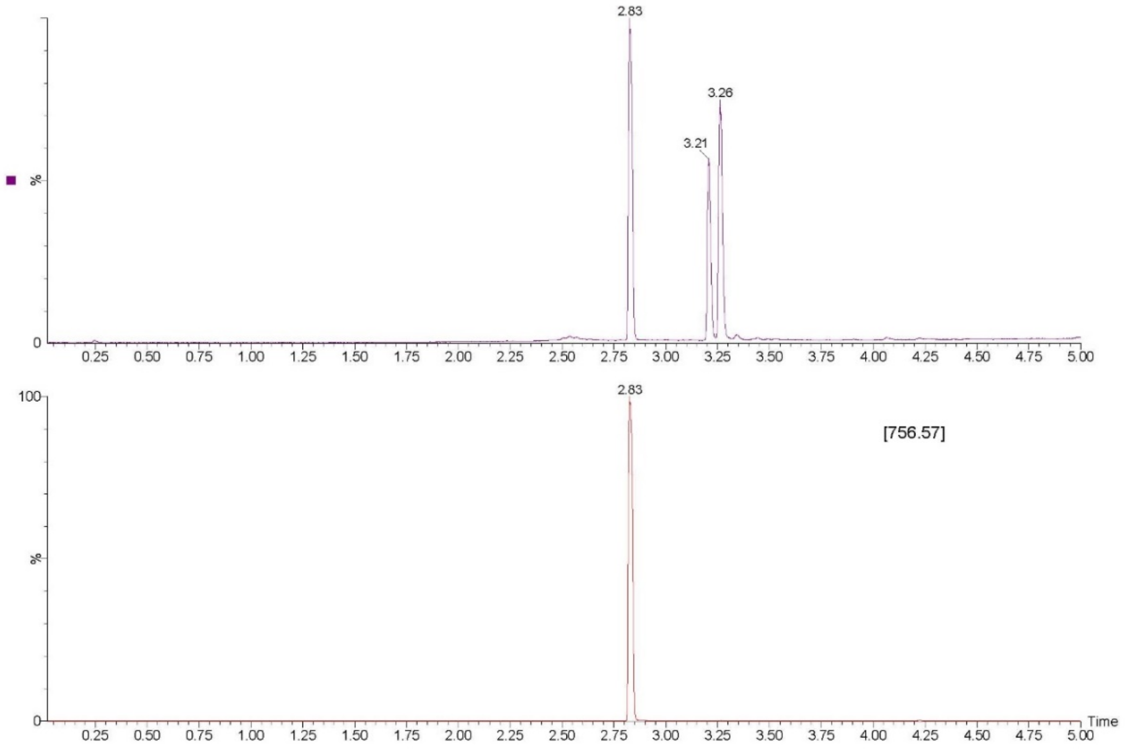

Octane-1,8-diyl diphenethyl bis((2-(bis(3-aminopropyl)amino)ethyl)phosphonate) hexahydro-chloride (65)

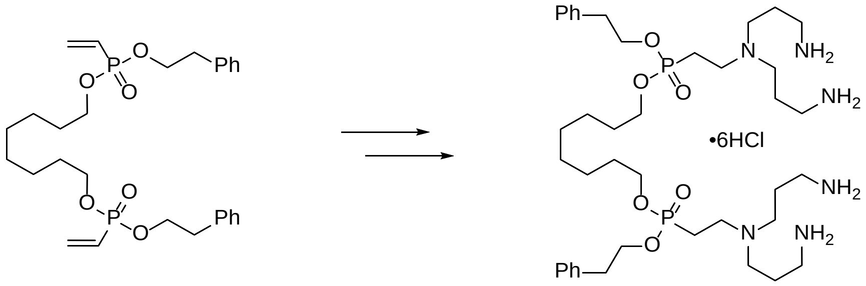

The title compound was prepared according to general methods **D** and **E** from vinylphosphonate dimer **S74** (0.15 g, 0.28 mmol) in 57% overall yield (0.16 g, 0.16 mmol) as a white solid.

^{1}H NMR (401 MHz, CD_3OD) δ 7.36–7.22 (m, 10H, *Ph*), 4.39–4.31 (m, 4H, OCH_2CH_2Ph), 4.06–3.90 (m, 4H, $OCH_2(CH_2)_3$), 3.39–3.31 (m, 12H, NCH_2), 3.09 (t, *J* = 7.5 Hz, 8H, CH_2NH_2), 3.03 (t, *J* = 6.6 Hz, 4H, CH_2Ph), 2.52–2.40 (m, 4H, PCH_2), 2.17 (ddd, *J* = 15.6, 9.1, 6.5 Hz, 8H, $CH_2CH_2NH_2$), 1.64 (p, *J* = 6.7 Hz, 4H, $OCH_2CH_2CH_2$), 1.39–1.28 (m, 8H, $O(CH_2)_2(CH_2)_2$).

^{13}C NMR (101 MHz, CD_3OD) δ 138.84 (C_{quat}), 130.26 (C_{ortho}), 129.70 (C_{meta}), 127.89 (C_{para}), 68.60 (d, *J* = 6.8 Hz, OCH_2CH_2Ph), 68.07 (d, *J* = 6.9 Hz, $OCH_2(CH_2)_3$), 51.02 ($CH_2(CH_2)_2NH_2$), 48.66 (d, *J* = 1.7 Hz, PCH_2CH_2), 37.85 (CH_2NH_2), 37.72 (d, *J* = 6.3 Hz, CH_2Ph), 31.45 (d, *J* = 6.1 Hz, $OCH_2CH_2CH_2$), 30.09 (d, *J* = 1.5 Hz, $O(CH_2)_2CH_2$), 26.45 ($O(CH_2)_3CH_2$), 23.24 ($CH_2CH_2NH_2$), 21.16 (d, *J* = 140.6 Hz, PCH_2).

^{31}P{^{1}H} NMR (162 MHz, CD_3OD) δ 28.50.

IR ν_{max} (KBr) 2960 (vs, sh), 2935 (vs), 2859 (vs), 2742 (s, sh), 2633 (s, br), 2557 (m), 2019 (w, vbr), 1604 (m), 1522 (m, sh), 1497 (m), 1468 (s), 1454 (s), 1405 (w), 1394 (w), 1258 (m, sh), 1227 (s), 1156 (w), 1060 (s), 1010 (vs), 970 (s, sh), 905 (w), 752 (m), 728 (w, sh), 701 (m), 574 (w), 491 (w).

HR-MS(ESI$^+$): For $C_{40}H_{75}N_6O_6P_2$ $(M+H)^+$ *m/z* calculated 797.52178, found 797.52232.

^{1}H NMR spectrum of compound **65**

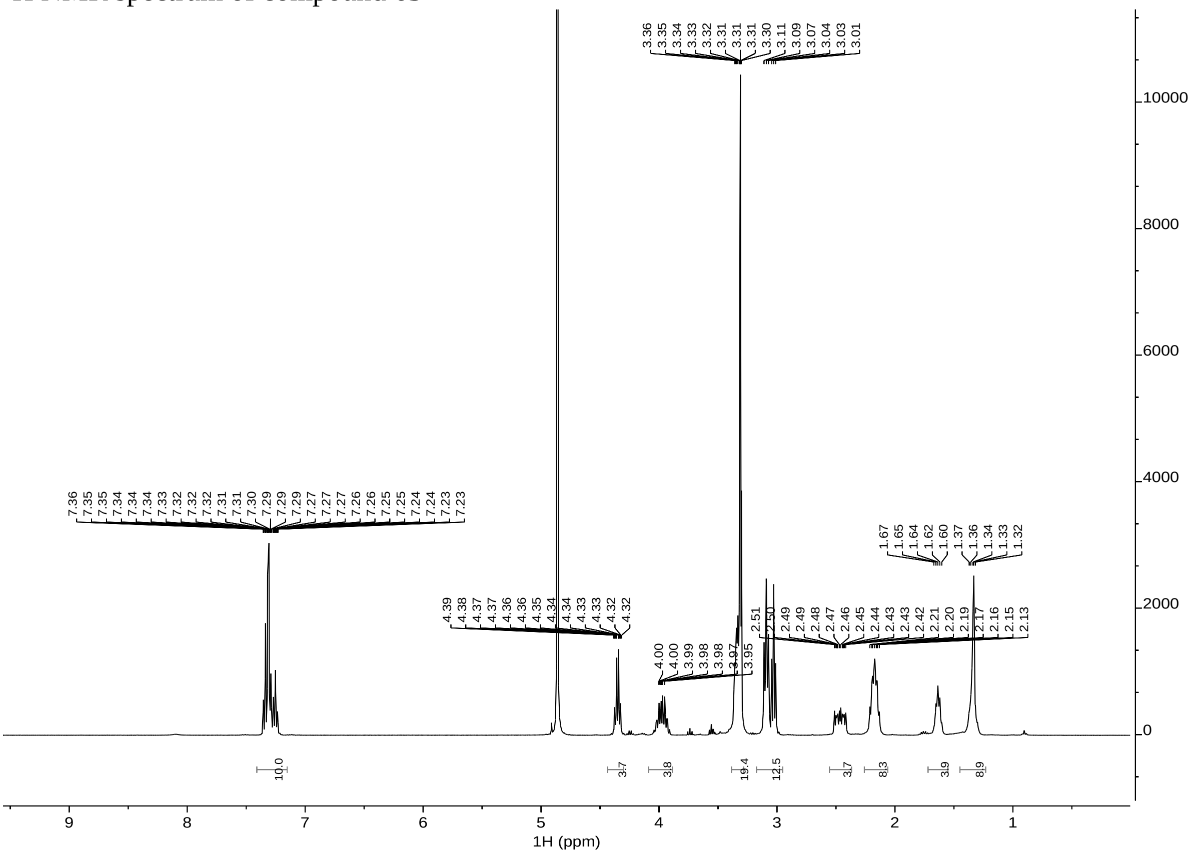

^{13}C NMR spectrum of compound **65**

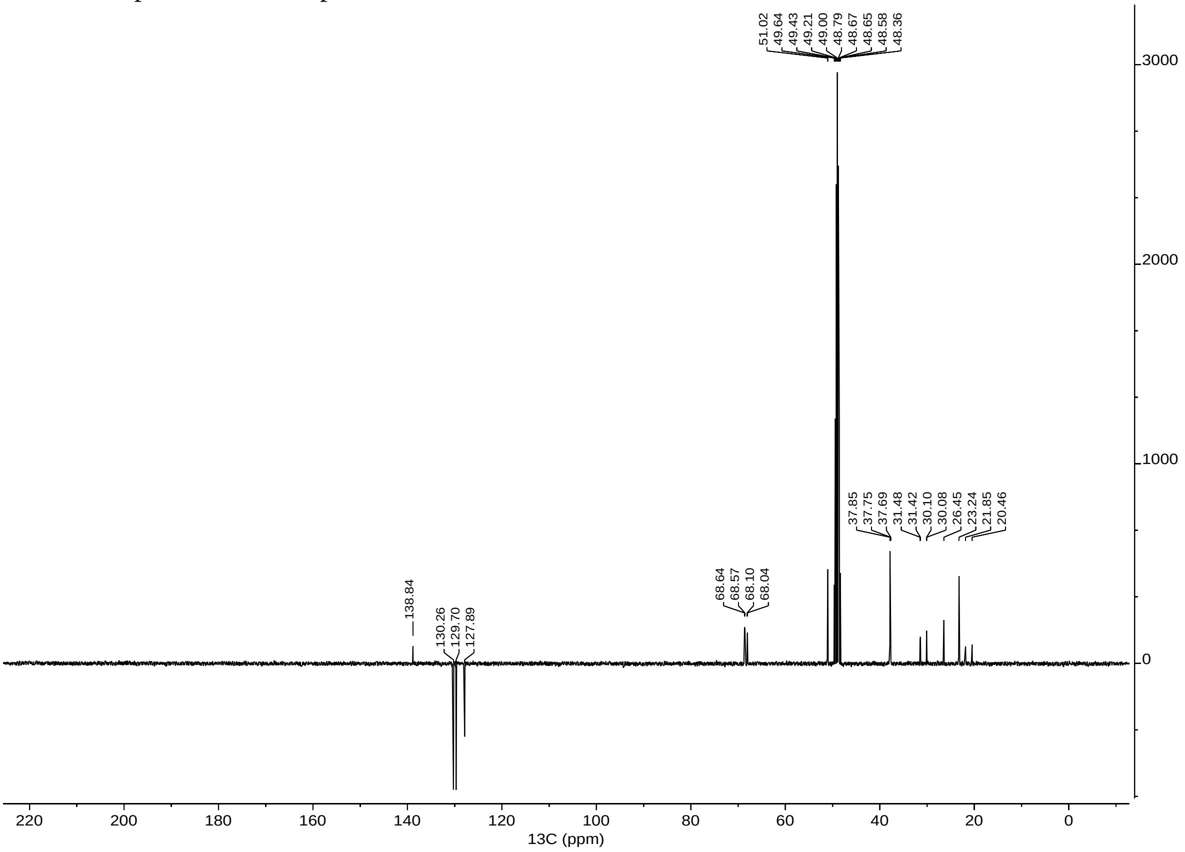

^{31}P{^{1}H} NMR spectrum of compound **65**

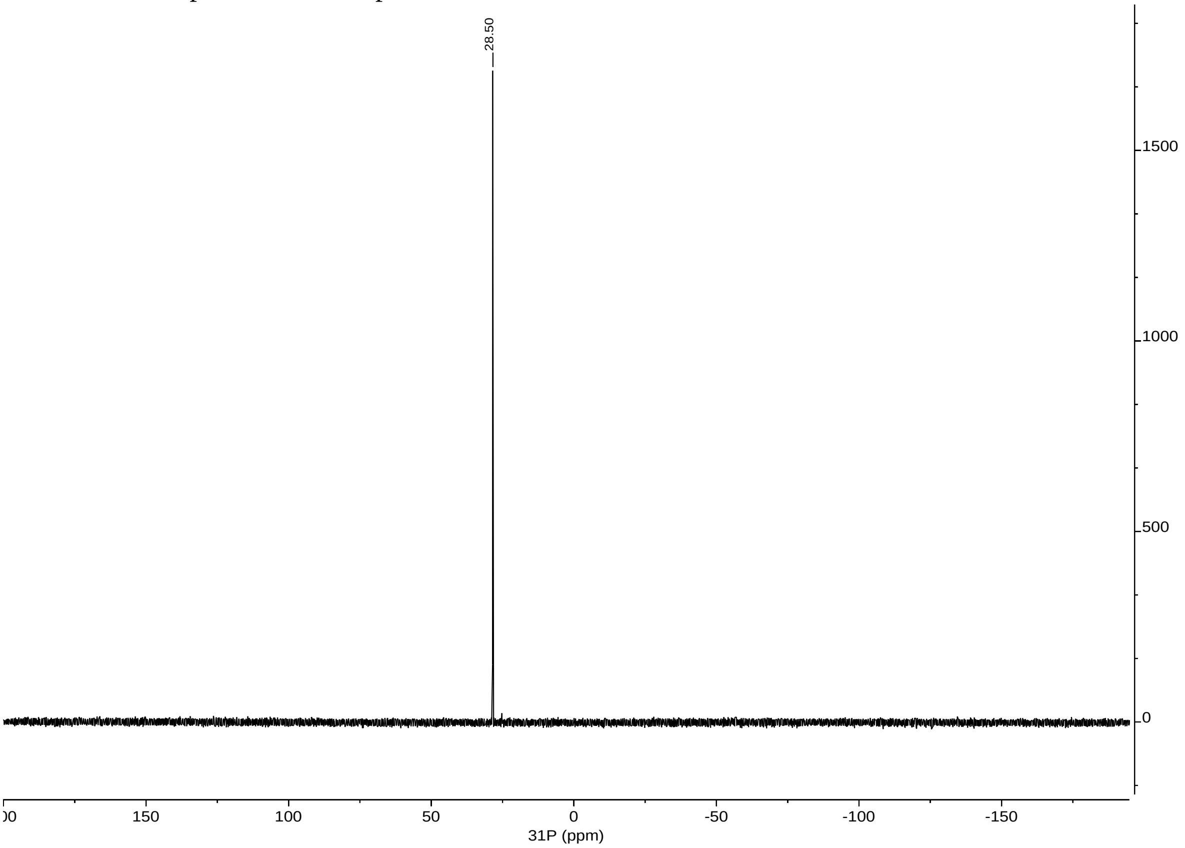

LC-MS chromatograms for compound **65**

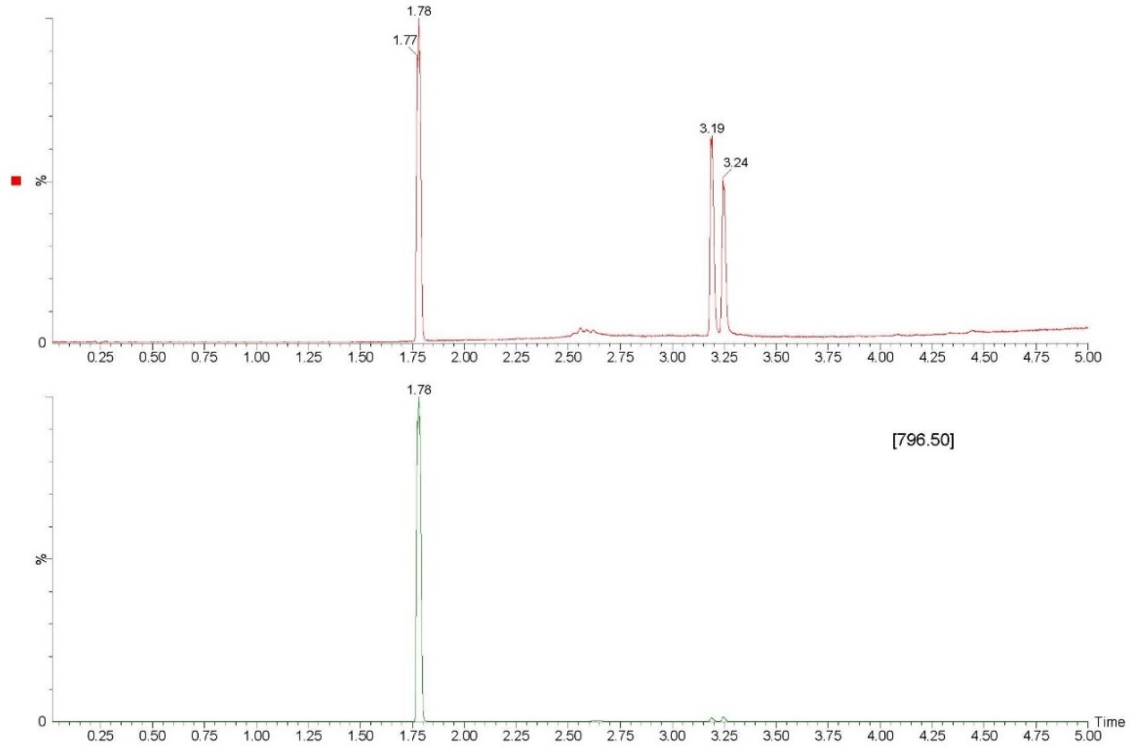

Decane-1,10-diyl diisobutyl bis((2-(bis(3-aminopropyl)amino)ethyl)phosphonate) hexahydro-chloride (66)

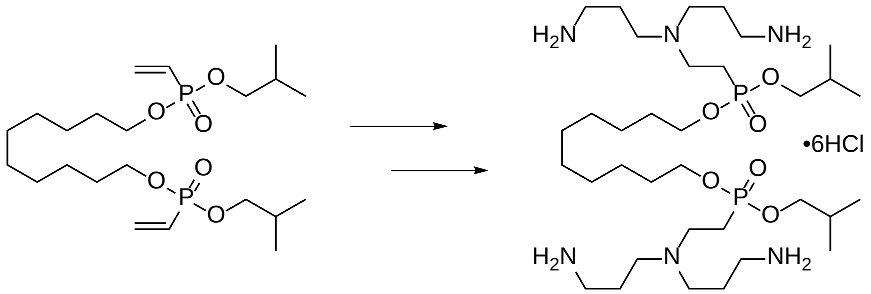

The title compound was prepared according to general methods **D** and **E** from vinylphosphonate dimer **S75** (416 mg, 0.89 mmol) in 31% overall yield (258 mg, 0.28 mmol) as a white solid.

^{1}H NMR (401 MHz, CD_3OD) δ 4.14 (q, J = 6.7 Hz, 4H, OC$H_2$$CH_2$), 3.90 (tt, J = 6.1, 3.2 Hz, 4H, OCH_2CH), 3.51–3.43 (m, 4H, $PCH_2C$$H_2$), 3.43–3.35 (m, 8H, C$H_2$$(CH_2)_2NH_2$), 3.11 (t, J = 7.5 Hz, 8H, C$H_2$$NH_2$), 2.74–2.47 (m, 4H, PC$H_2$), 2.21 (h, J = 6.4 Hz, 8H, C$H_2$$CH_2NH_2$), 1.98 (dt, J = 13.3, 6.6 Hz, 2H, OCH_2C*H*), 1.83–1.65 (m, 4H, $OCH_2C$$H_2$), 1.53–1.27 (m, 12H, $O(CH_2)_2(C$$H_2$$)_3$), 0.99 (d, J = 6.7 Hz, 12H, CH_3).

^{13}C NMR (101 MHz, CD_3OD) δ 73.92 (d, J = 7.1 Hz, O*C*H_2CH), 68.29 (d, J = 6.7 Hz, O*C*H_2CH_2), 51.09 (*C*$H_2(CH_2)_2NH_2$), 37.86 (*C*H_2NH_2), 31.63 (d, J = 5.9 Hz, OCH_2*C*H_2), 30.65, 30.47 (d, J = 6.4 Hz, OCH_2*C*H), 30.30, 26.63 ($O(CH_2)_2($*C*$H_2)_2$), 23.33 (*C*$H_2CH_2NH_2$), 21.16 (d, J = 139.8 Hz, P*C*H_2), 19.02 (*C*H_3).

^{31}P{^{1}H} NMR (162 MHz, CD_3OD) δ 28.59.

IR ν_{max} (KBr) 2961 (s), 2929 (s), 2855 (m), 3200–2700 (vs, vbr), 2700–2500 (m, vbr), 1610 (m), 1513 (m), 1468 (s), 1401 (m), 1369 (m), 1227 (vs), 1005 (vs), ~869 (m, sh), 851 (m), 767 (m), 725 (w).

HR-MS(ESI^{+}): For $C_{34}H_{79}O_6N_6P_2$ $(M+H)^+$ *m*/*z* calculated 729.55308, found 729.55268.

^{1}H NMR spectrum of compound **66**

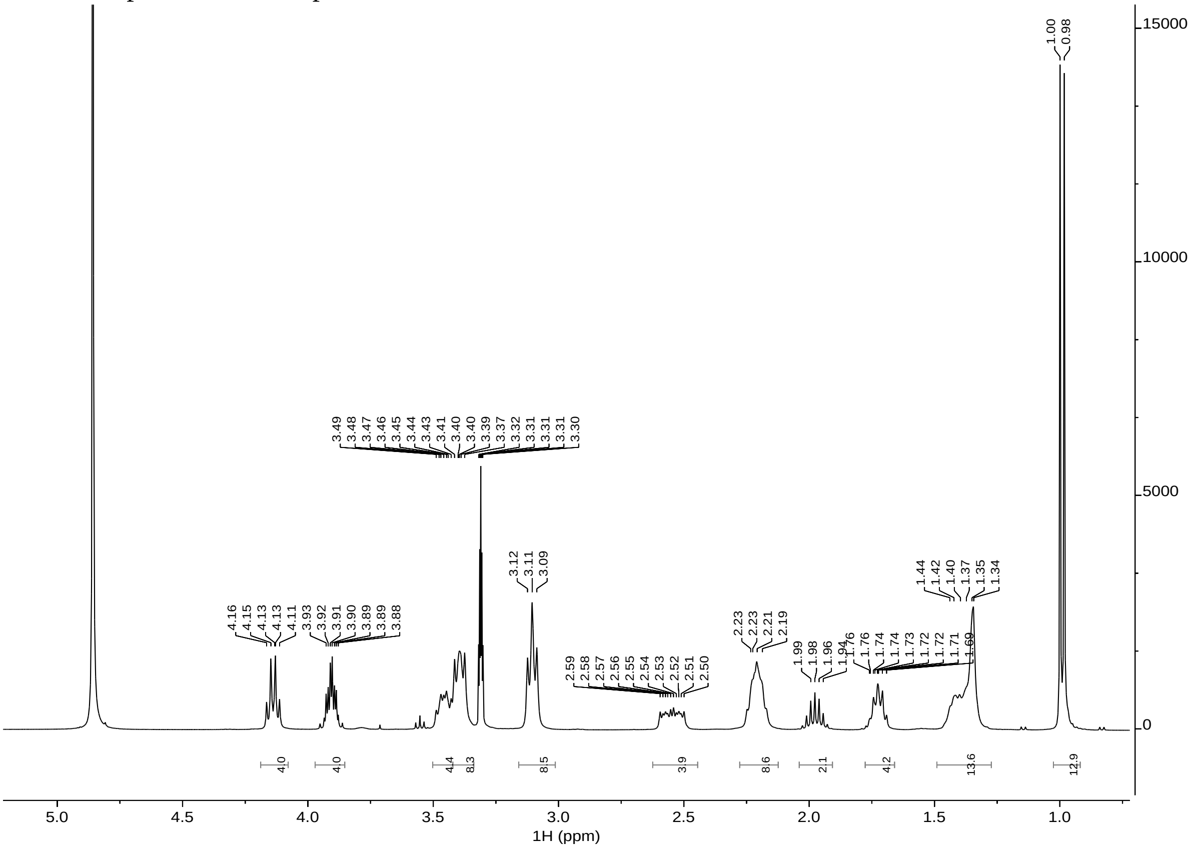

^{13}C NMR spectrum of compound **66**

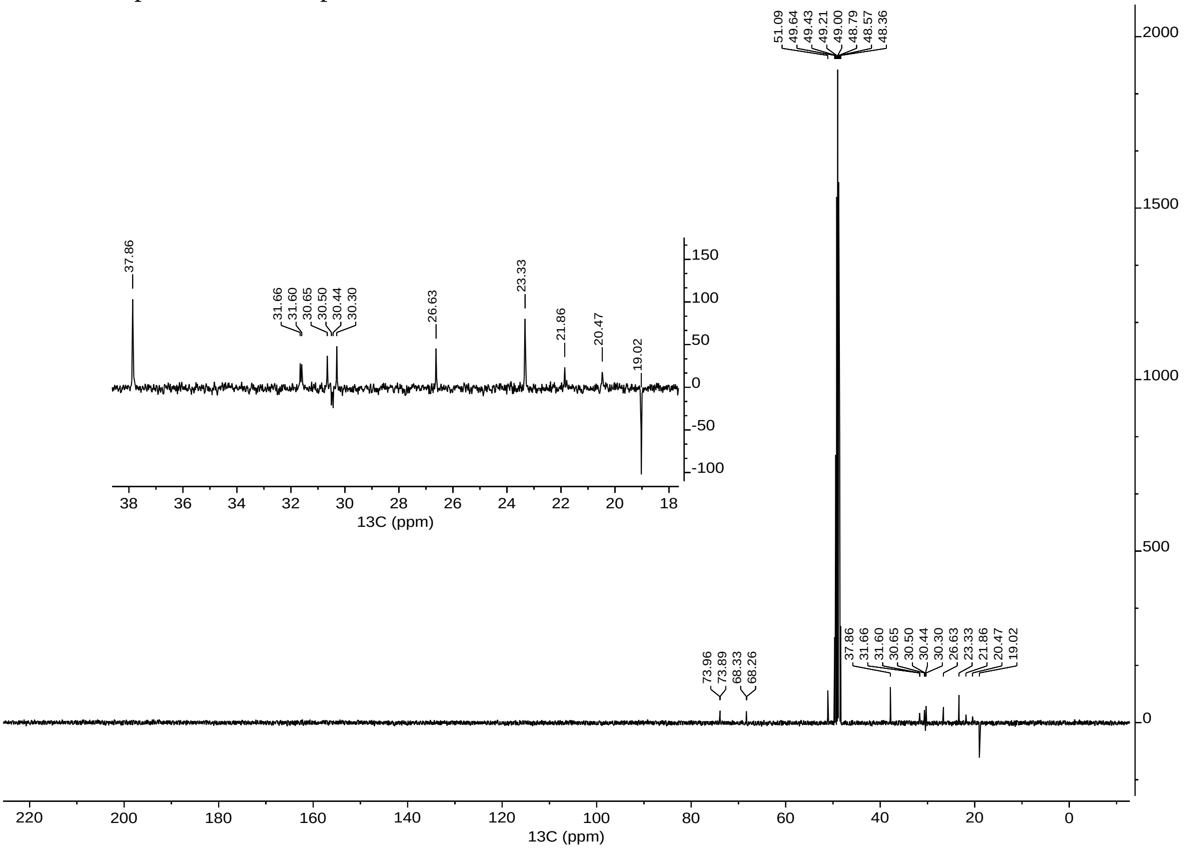

^{31}P{^{1}H} NMR spectrum of compound **66**

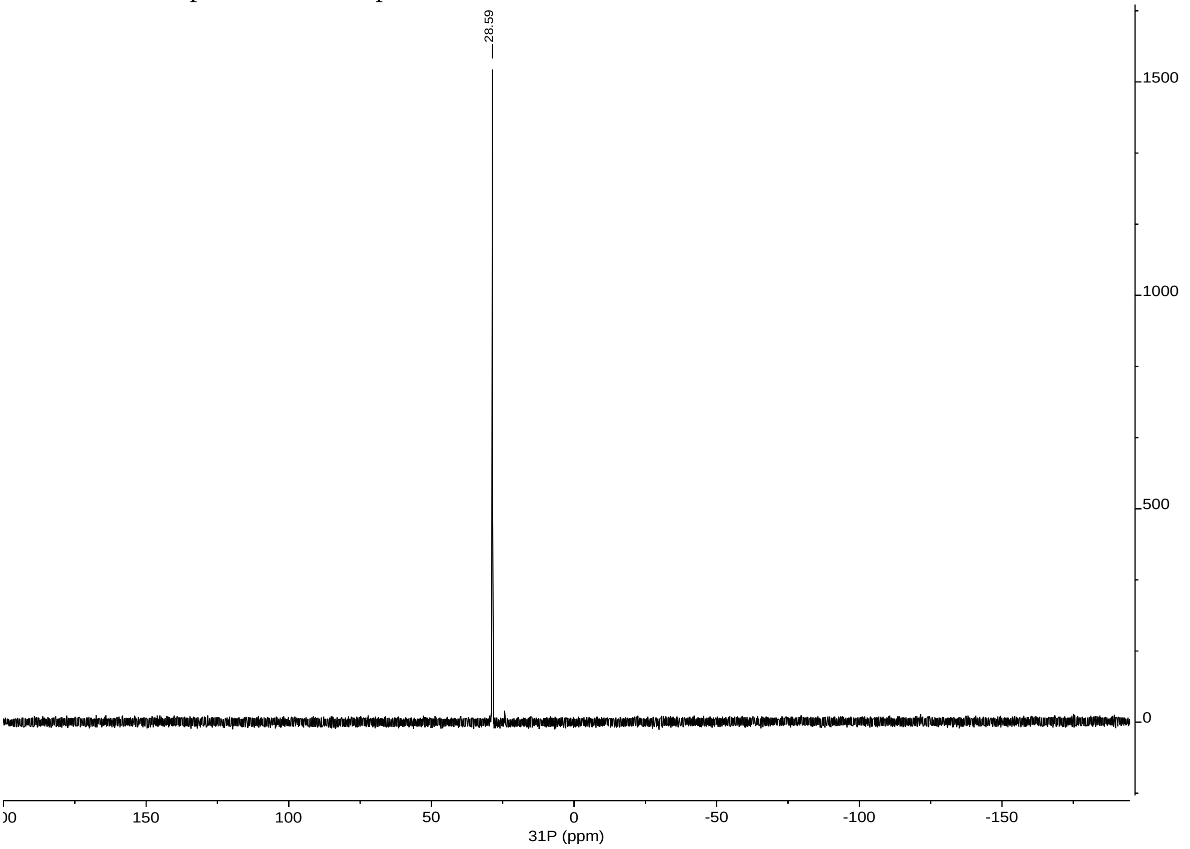

LC-MS chromatograms for compound **66**

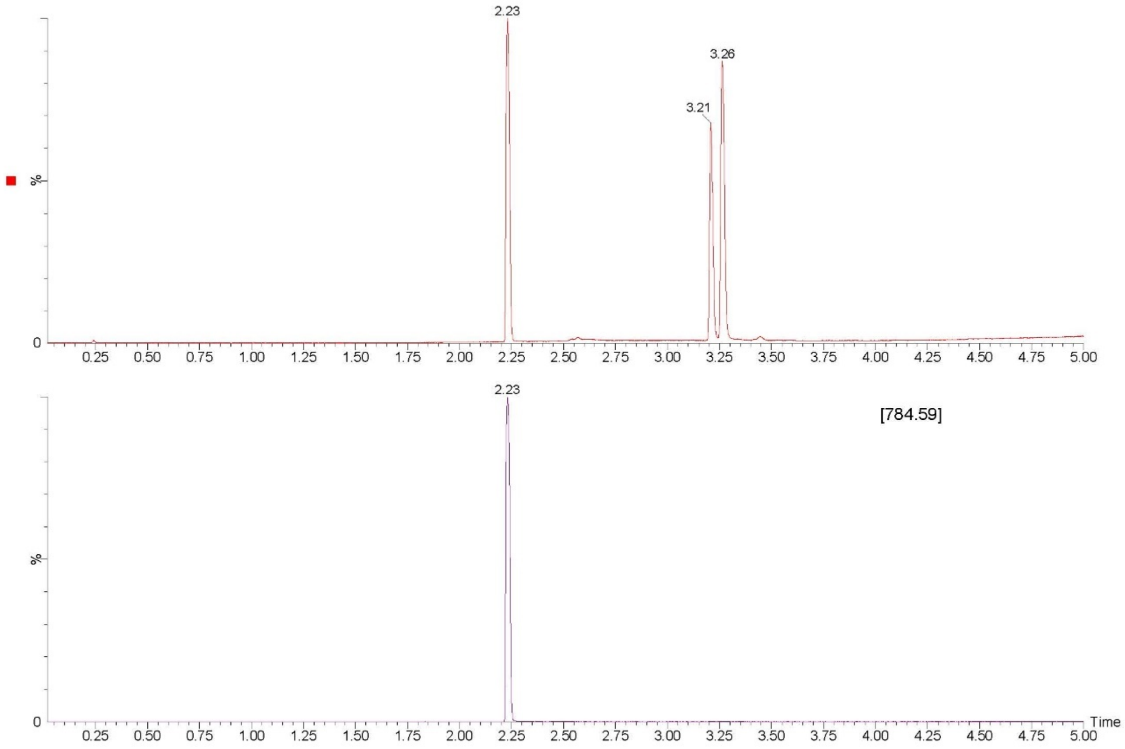

Dibutyl decane-1,10-diyl bis((2-(bis(3-aminopropyl)amino)ethyl)phosphonate) hexahydrochloride (67)

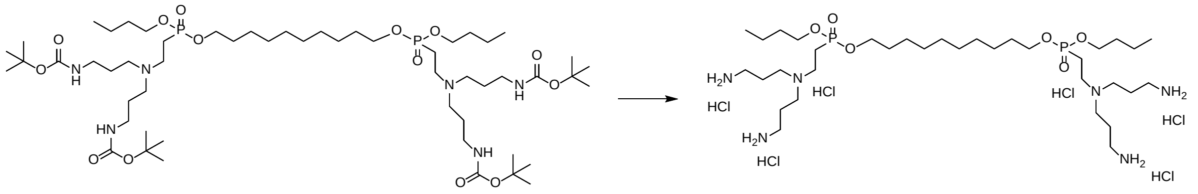

The title compound was prepared according to general method **E** from Boc derivative **S118** (0.80 g, 0.71 mmol) in 94% yield (0.63 g, 0.67 mmol) as a white solid.

^{1}H NMR (401 MHz, CD_3OD) δ 4.17–4.10 (m, 8H, C*H*$_2$O), 3.50–3.42 (m, 4H, PCH$_2$C*H*$_2$), 3.39 (dd, J = 10.2, 6.3 Hz, 8H, C*H*$_2$(CH$_2$)$_2$NH$_2$), 3.10 (t, J = 7.5 Hz, 8H, C*H*$_2$NH$_2$), 2.60–2.46 (m, 4H, PC*H*$_2$), 2.28–2.14 (m, 8H, C*H*$_2$CH$_2$NH$_2$), 1.78–1.66 (m, 8H, C*H*$_2$CH$_2$O), 1.51–1.31 (m, 16H, CH$_3$C*H*$_2$, (C*H*$_2$)$_3$(CH$_2$)$_2$O), 0.98 (t, J = 7.4 Hz, 6H, C*H*$_3$).

^{13}C NMR (101 MHz, CD_3OD) δ 68.24 (d, J = 6.8 Hz), 67.90 (d, J = 6.7 Hz, *C*H$_2$O), 51.05 (*C*H$_2$(CH$_2$)$_2$NH$_2$), 48.81 (PCH$_2$*C*H$_2$), 37.86 (*C*H$_2$NH$_2$), 33.61 (d, J = 6.0 Hz), 31.60 (d, J = 5.8 Hz, *C*H$_2$CH$_2$O), 30.62, 30.28, 26.61 ((*C*H$_2$)$_3$(CH$_2$)$_2$O), 23.27 (*C*H$_2$CH$_2$NH$_2$), 21.23 (d, J = 140.6 Hz, P*C*H$_2$), 19.80 (CH$_3$*C*H$_2$), 13.96 (*C*H$_3$).

^{31}P{^{1}H} NMR (162 MHz, CD_3OD) δ 28.66.

IR ν_{max} (KBr) 2932 (vs), 2632 (m), 2558 (m), 1598 (m), 1467 (m), 1224 (m), 1172 (m sh), 1070 (m, sh), 1063 (m), 1018 (vs), 997 (m, sh).

HR-MS(ESI$^+$): For $C_{34}H_{79}N_6O_6P_2$ $(M+H)^+$ *m/z* calculated 729.55308, found 729.55255.

^{1}H NMR spectrum of compound **67**

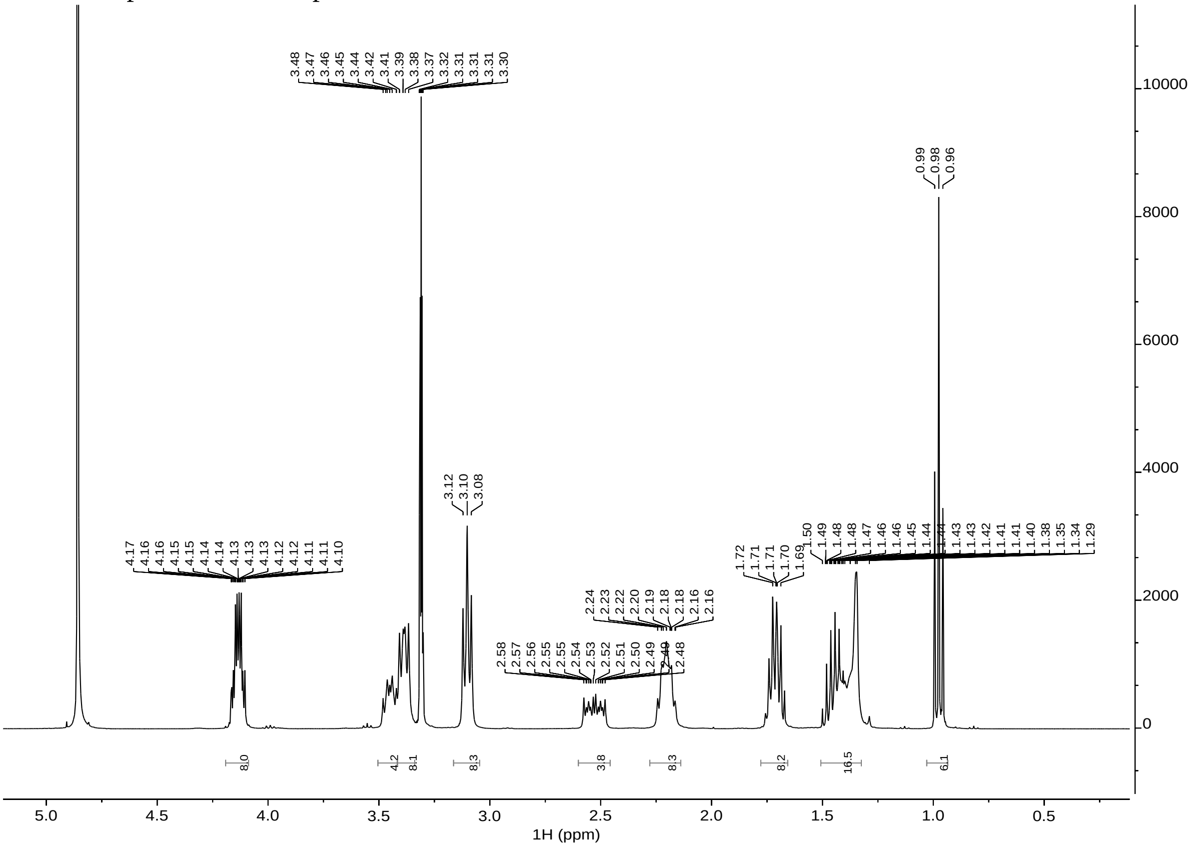

^{13}C NMR spectrum of compound **67**

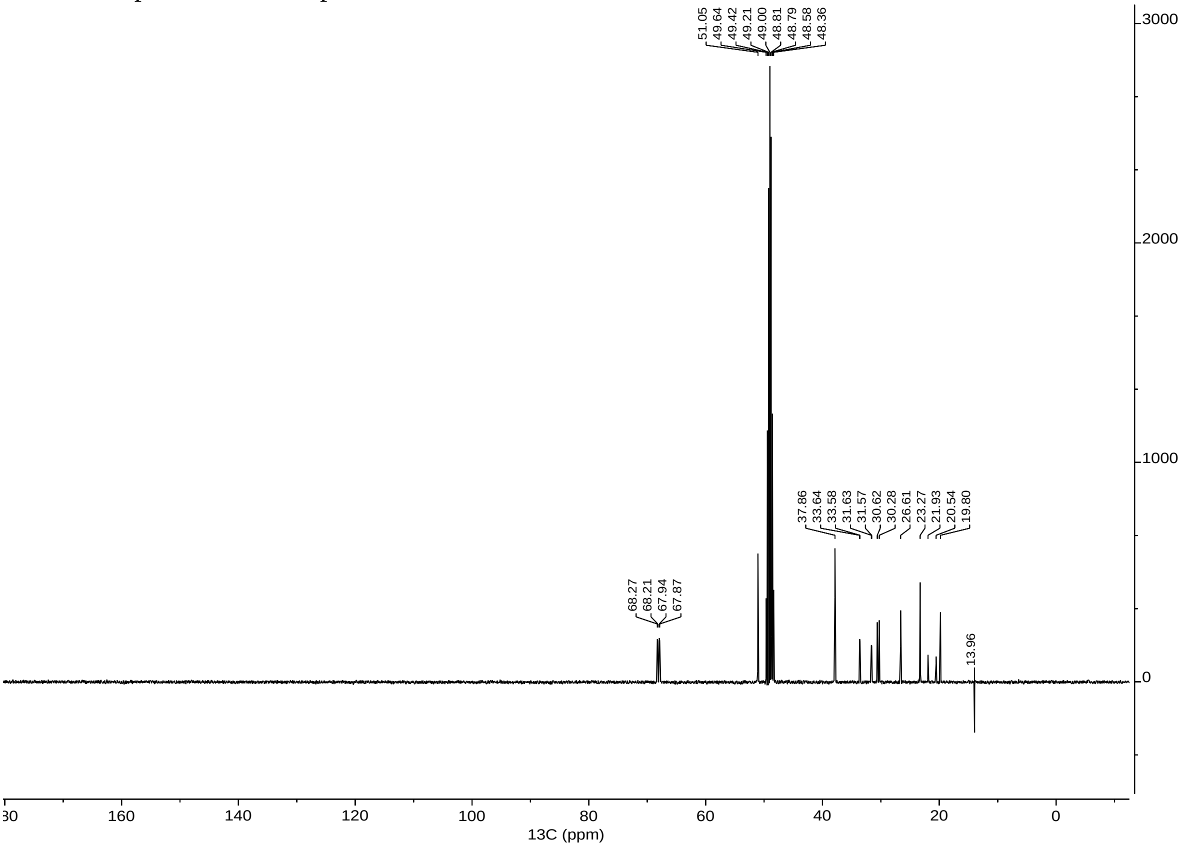

^{31}P{^{1}H} NMR spectrum of compound **67**

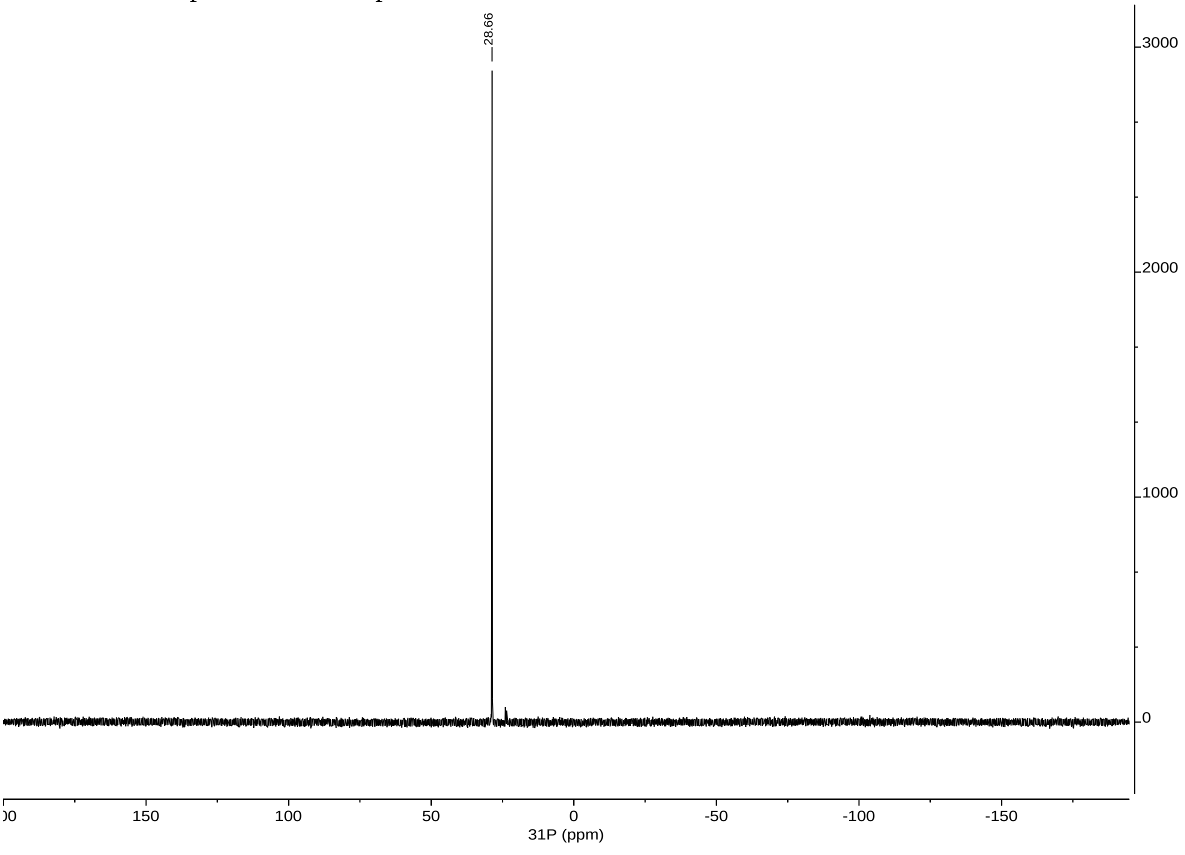

LC-MS chromatograms for compound **67**

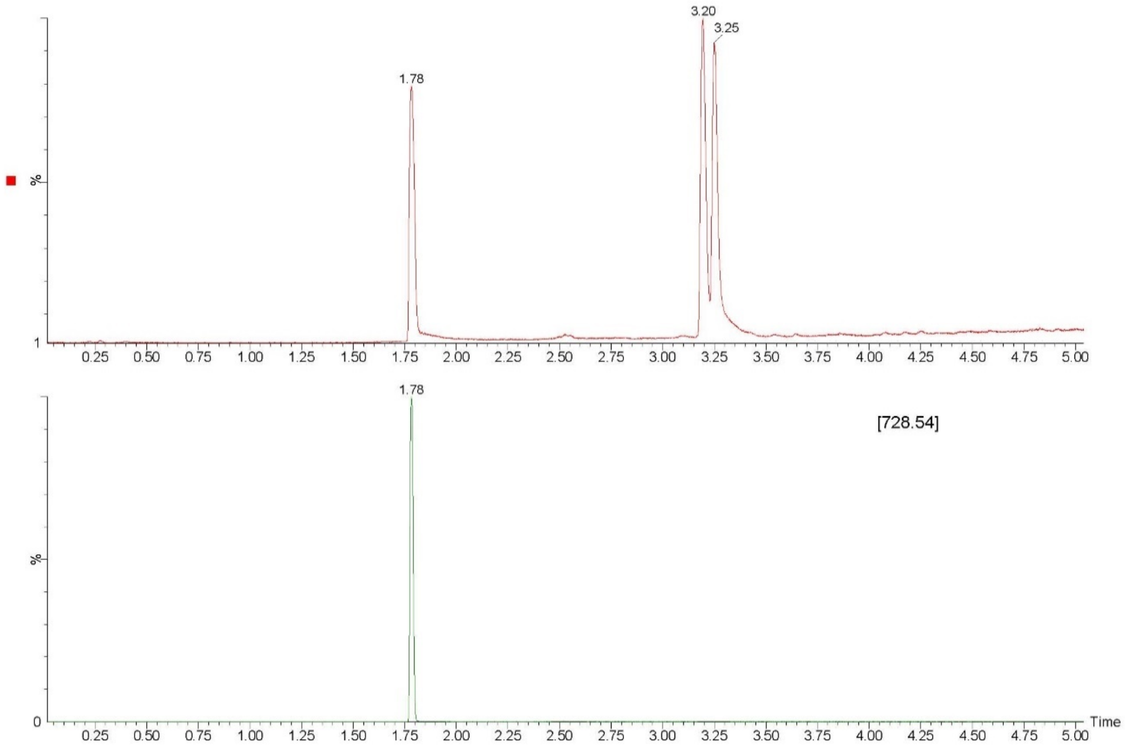

Dibutyl decane-1,10-diyl bis((2-(bis(2-aminoethyl)amino)ethyl)phosphonate) hexahydrochloride (68)

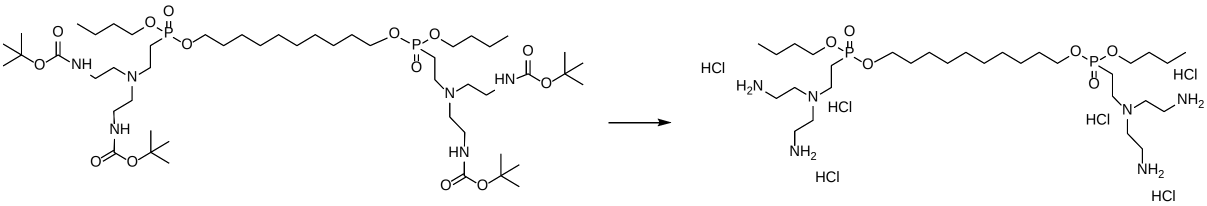

The title compound was prepared according to general method **E** from Boc derivative **S119** (0.56 g, 0.52 mmol) in 94% yield (0.43 g, 0.49 mmol) as a white solid.

^{1}H NMR (400 MHz, CD_3OD): 4.17–4.04 (m, 8H, C*H*$_2$O), 3.29–3.20 (m, 8H, C*H*$_2$NH$_2$), 3.17–3.04 (m, 12H, C*H*$_2$N), 2.36–2.22 (m, 4H, PC*H*$_2$), 1.78–1.63 (m, 8H, C*H*$_2$CH$_2$O), 1.52–1.30 (m, 16H, CH$_3$C*H*$_2$, (C*H*$_2$)$_3$(CH$_2$)$_2$O), 0.98 (t, 6H, *J* = 7.4 Hz, C*H*$_3$).

^{13}C NMR (101 MHz, CD_3OD): 67.96 (d, *J* = 6.8 Hz), 67.64 (d, *J* = 6.7 Hz, *C*H$_2$O), 51.50 (*C*H$_2$CH$_2$NH$_2$), 48.49 (PCH$_2$*C*H$_2$), 36.81 (*C*H$_2$NH$_2$), 33.62 (d, *J* = 6.1 Hz), 31.59 (d, *J* = 6.1 Hz, *C*H$_2$CH$_2$O), 30.59, 30.26, 26.63 ((*C*H$_2$)$_3$(CH$_2$)$_2$O), 20.81 (d, *J* = 139.2 Hz, P*C*H$_2$), 19.82 (CH$_3$*C*H$_2$), 13.96 (*C*H$_3$).

^{31}P{^{1}H} NMR (162 MHz, CD_3OD): 28.44.

IR ν_{max} (KBr) 2961 (m), 2926 (m), 2674 (m, sh), 2551 (m, br), 1613, (m), 1467 (m), 1391 (m), 1260 (m), 1236 (m), 1066 (m, sh), 991 (m, sh).

HR-MS(ESI$^+$): For $C_{30}H_{71}N_6O_6P_2$ $(M+H)^+$ *m/z* calculated 673.49048, found 673.49047.

^{1}H NMR spectrum of compound **68**

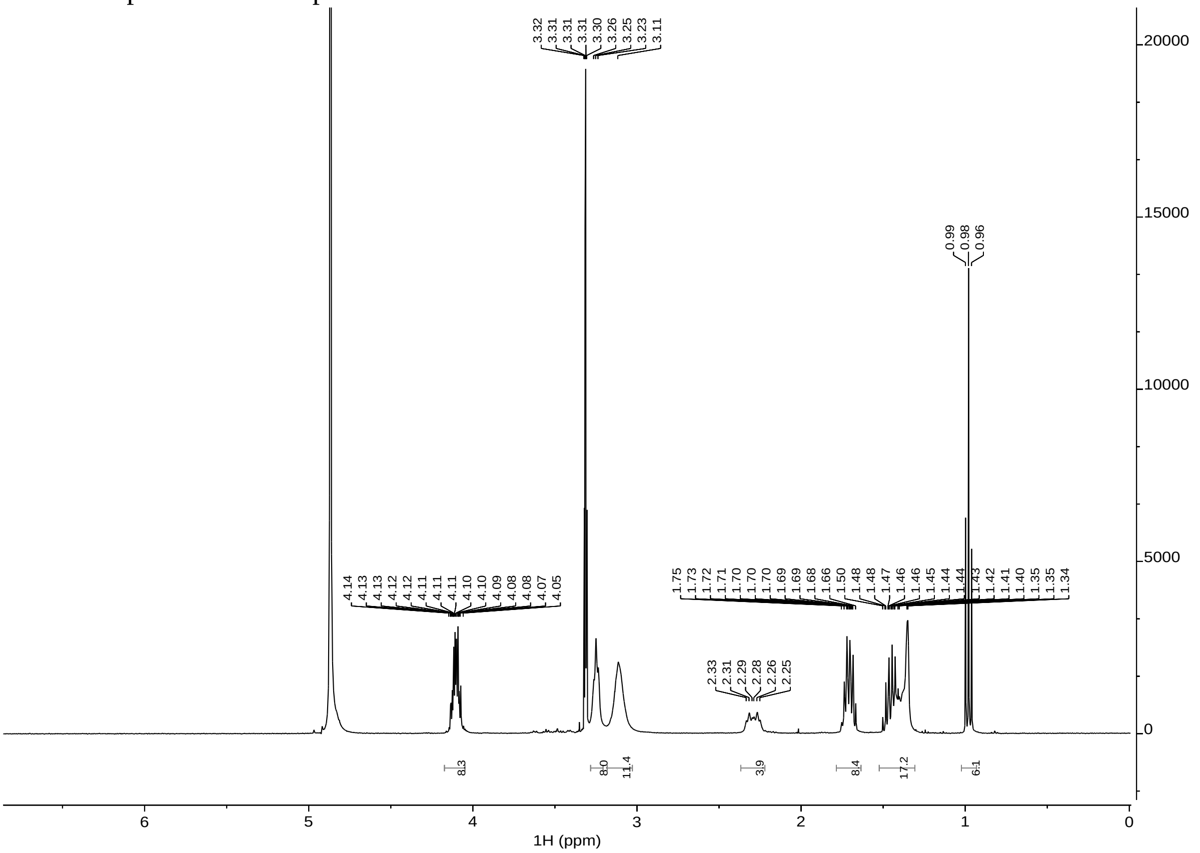

^{13}C NMR spectrum of compound **68**

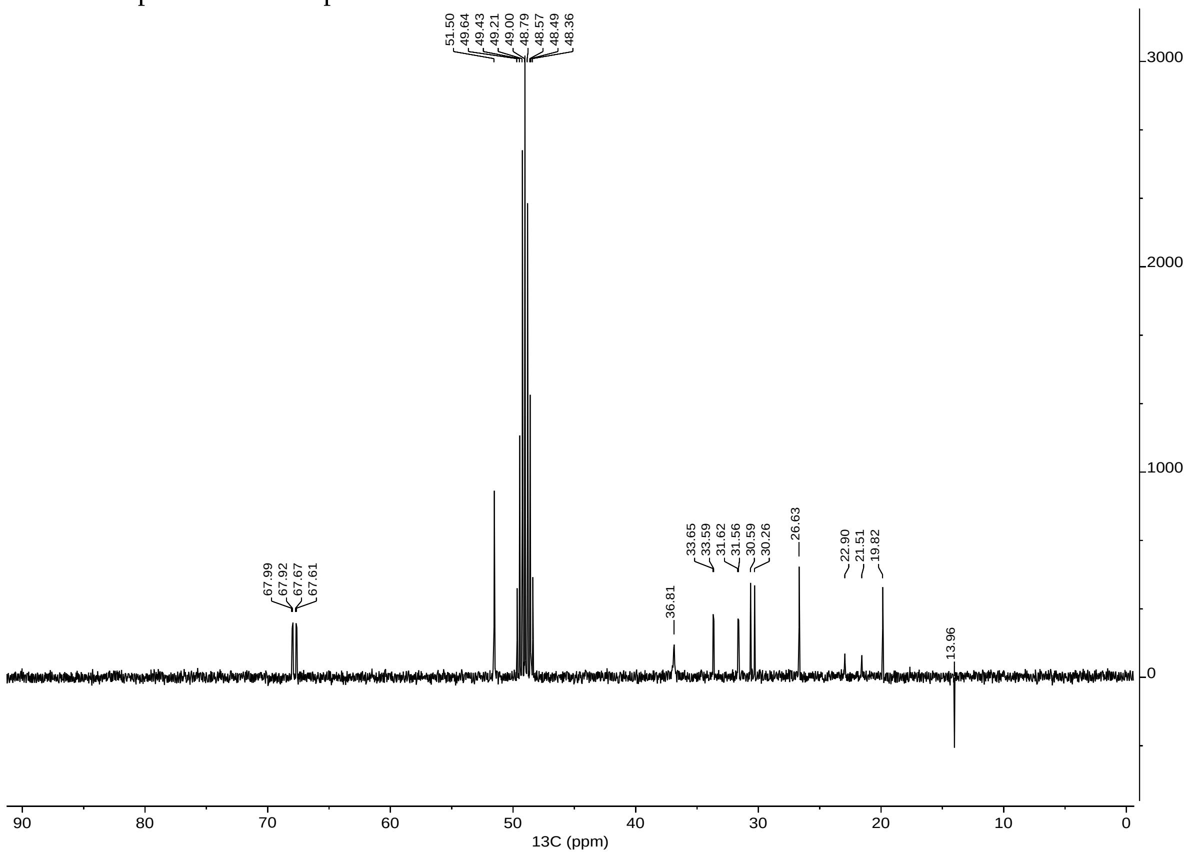

$^{31}P\{^{1}H\}$ NMR spectrum of compound **68**

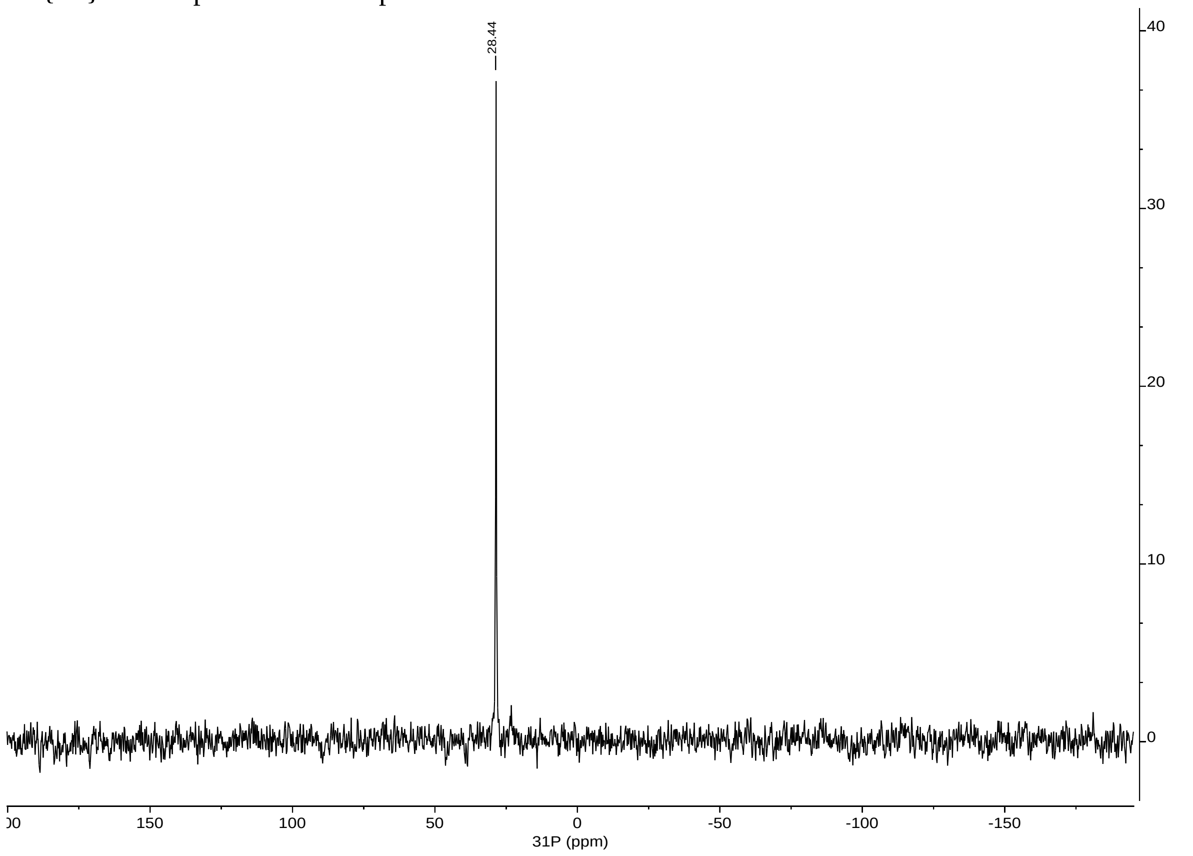

LC-MS chromatograms for compound **68**

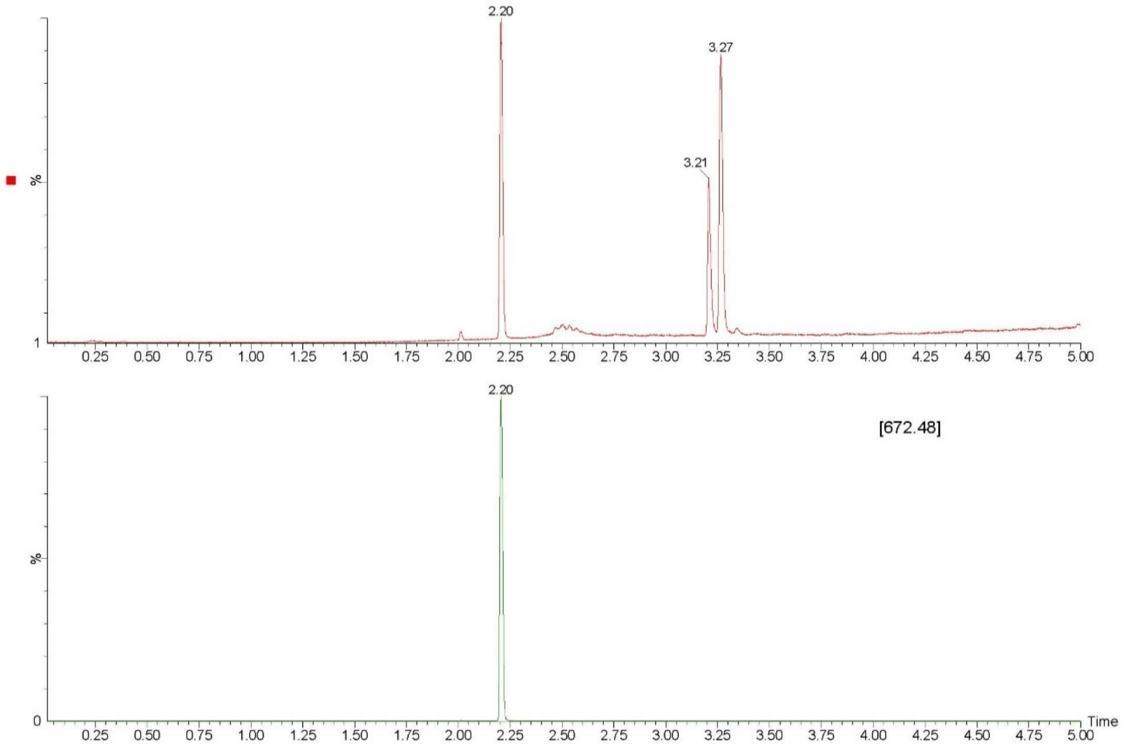

Decane-1,10-diyl dipentyl bis((2-(bis(3-aminopropyl)amino)ethyl)phosphonate) hexahydrochloride (69)

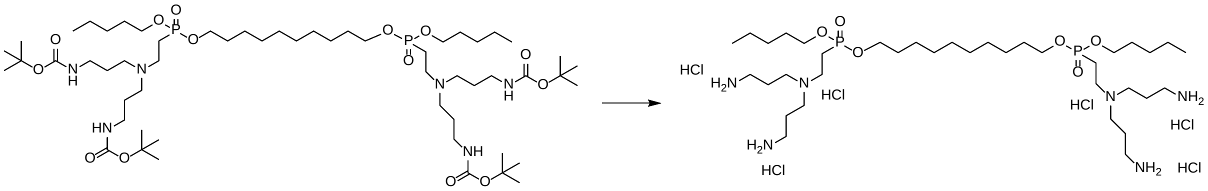

The title compound was prepared according to general method **E** from Boc derivative **S120** (0.90 g, 0.82 mmol) in 91% yield (0.73 g, 0.75 mmol) as a white solid.

^{1}H NMR (400 MHz, CD_3OD): 4.19–4.07 (m, 8H, CH_2O), 3.49–3.42 (m, 4H, PCH_2CH_2), 3.42–3.35 (m, 8H, C$H_2(CH_2)_2NH_2$), 3.10 (t, 8H, J = 7.5 Hz, CH_2NH_2), 2.61–2.45 (m, 4H, PCH_2), 2.28–2.14 (m, 8H, C$H_2CH_2NH_2$), 1.79–1.66 (m, 8H, CH_2CH_2O), 1.48–1.30 (m, 20H, $CH_3(CH_2)_2$, $(CH_2)_3(CH_2)_2O$), 1.00–0.89 (m, 6H, CH_3).

^{13}C NMR (101 MHz, CD_3OD): 69.20 (d, J = 6.8 Hz), 68.27 (d, J = 6.8 Hz, CH$_2$O), 51.07 (CH$_2(CH_2)_2NH_2$), 48.83 (PCH$_2C$H$_2$), 37.87 (CH$_2NH_2$), 31.61 (d, J = 5.9 Hz), 31.29 (d, J = 5.9 Hz, CH$_2CH_2O$), 30.63, 30.29, 28.80, 26.62, 23.30 ($CH_3(CH_2)_2$, $(CH_2)_3(CH_2)_2O$), 23.27 (CH$_2CH_2NH_2$), 21.25 (d, J = 140.5 Hz, PCH$_2$), 14.35 (CH$_3$).

^{31}P{^{1}H} NMR (162 MHz, CD_3OD): 28.41.

IR ν_{max} (KBr) 2929 (s), 2831 (m, sh), 2677–2543 (m), 1611 (m), 1514 (m), 1467 (m), 1390 (m), 1263 (m), 1228 (s), 1077–991 (s).

HR-MS(ESI$^+$): For $C_{36}H_{83}N_6O_6P_2$ $(M+H)^+$ m/z calculated 379.29583, found 379.29576.

^{1}H NMR spectrum of compound **69**

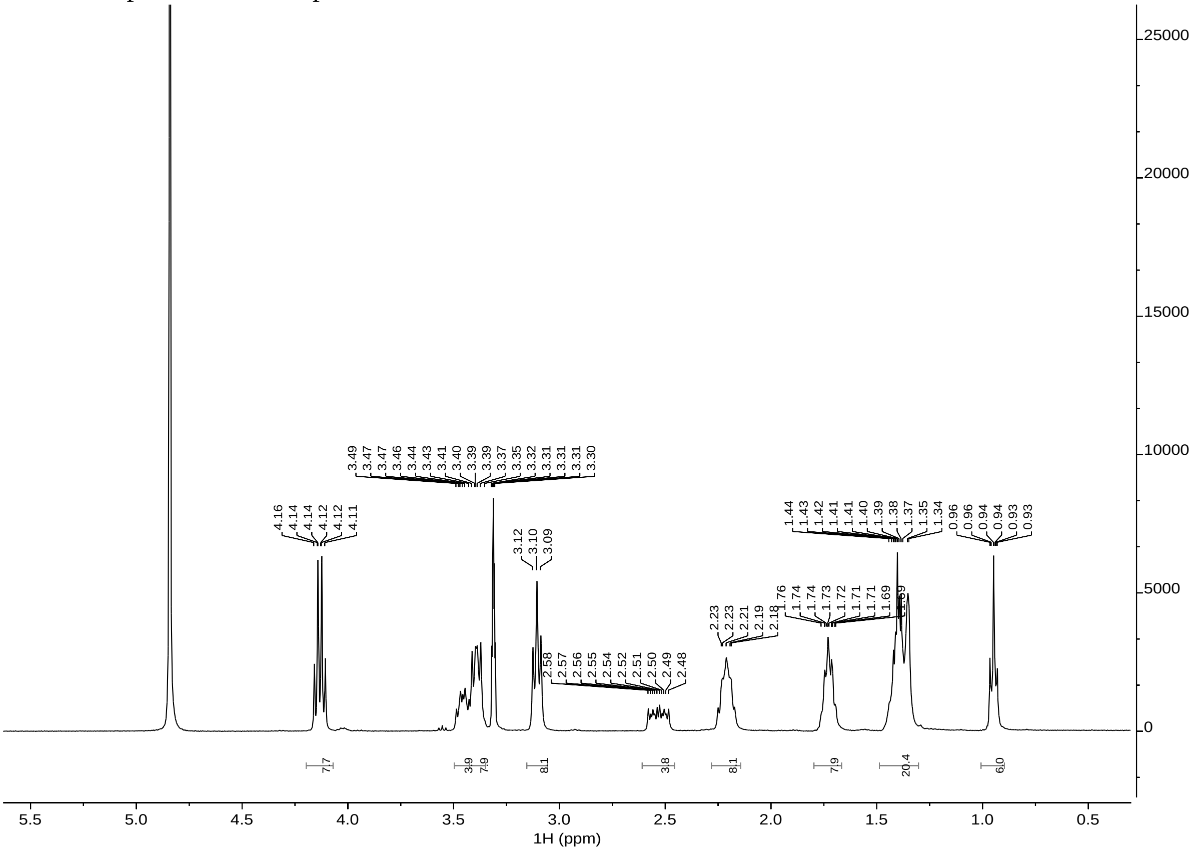

^{13}C NMR spectrum of compound **69**

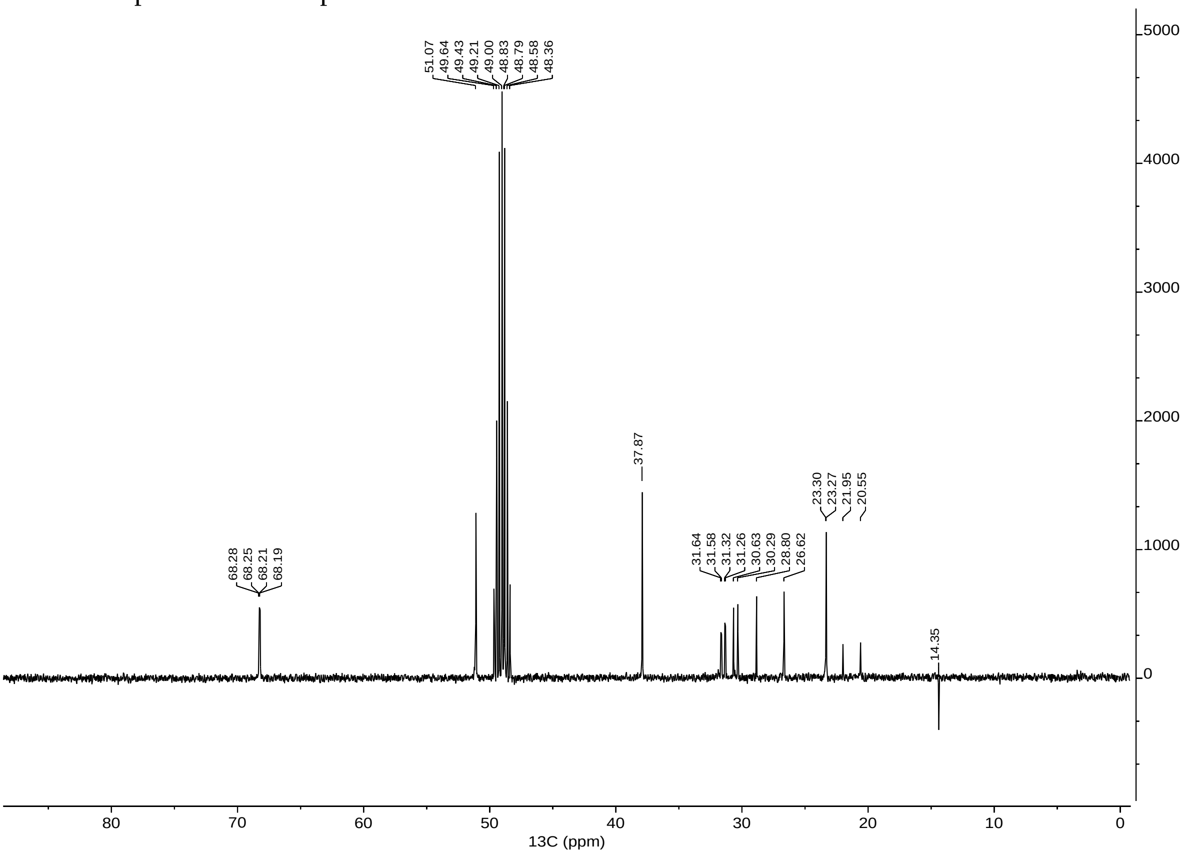

$^{31}P\{^{1}H\}$ NMR spectrum of compound **69**

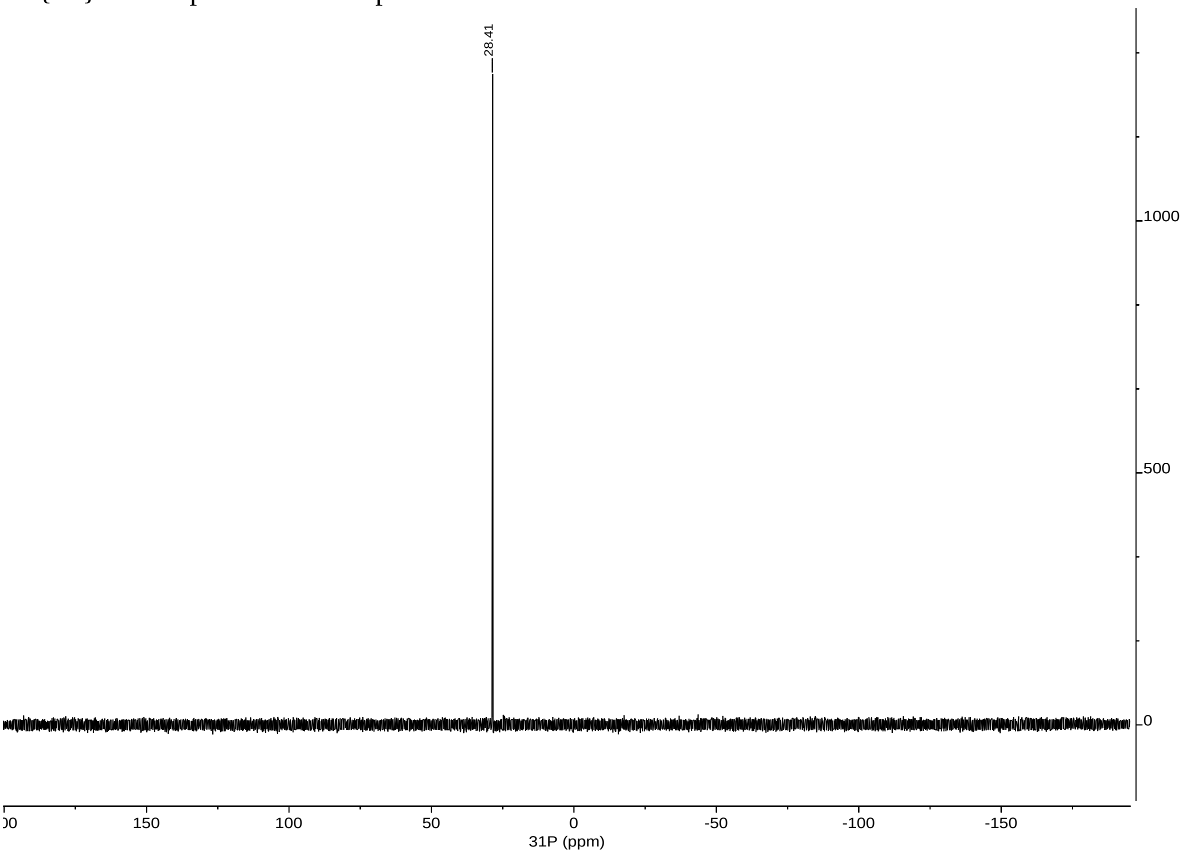

LC-MS chromatograms for compound **69**

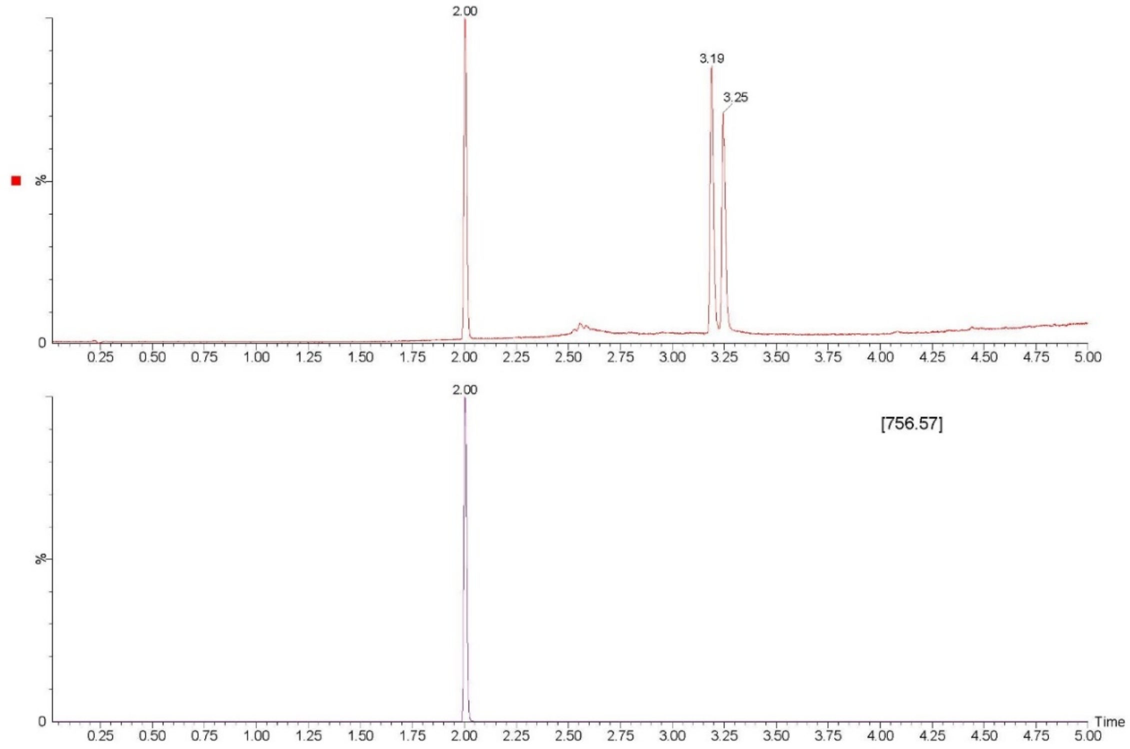

Decane-1,10-diyl dihexyl bis((2-(bis(3-aminopropyl)amino)ethyl)phosphonate) hexahydrochloride (70)

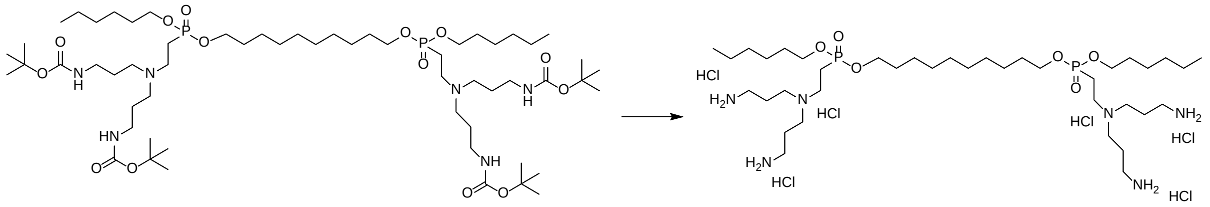

The title compound was prepared according to general method **E** from Boc derivative **S121** (1.0 g, 0.84 mmol) in 90% yield (0.70 g, 0.76 mmol) as a white solid.

^{1}H NMR (400 MHz, CD_3OD): 4.14 (t, J = 6.6 Hz, 4H), 4.12 (t, J = 6.5 Hz, 4H, CH_2O), 3.50–3.41 (m, 4H, PCH_2CH_2), 3.41–3.33 (m, 8H, C$H_2$$(CH_2)_2NH_2$), 3.10 (t, 8H, J = 7.5 Hz, C$H_2$$NH_2$), 2.59–2.44 (m, 4H, PC$H_2$), 2.27–2.13 (m, 8H, C$H_2$$CH_2NH_2$), 1.78–1.67 (m, 8H, C$H_2$$CH_2O$), 1.49–1.29 (m, 24H, (C$H_2$)$_3$$(CH_2)_2O$), 0.97–0.88 (m, 6H, C$H_3$).

^{13}C NMR (101 MHz, CD_3OD): 66.85 (d, J = 7.2 Hz, CH_2O), 51.08 ($CH_2(CH_2)_2NH_2$), 48.83 (PCH_2CH_2), 37.88 (CH_2NH_2), 32.51 ($CH_3CH_2CH_2$), 31.63 (d, J = 5.6 Hz), 31.57 (d, J = 5.7 Hz, CH_2CH_2O), 30.66, 30.31, 26.64, 26.31, 23.64 (CH_3CH_2), 23.31 ($CH_2CH_2NH_2$), 21.24 (d, J = 140.6 Hz, PCH_2), 14.38 (CH_3).

^{31}P{^{1}H} NMR (162 MHz, CD_3OD): 28.45.

IR ν_{max} (KBr) 2974 (m), 2927 (w), 2895 (w), 2635 (w), 2510 (w), 1601 (w, sh), 1383 (w), 1273 (w), 1089 (m), 1050 (m).

HR-MS(ESI^{+}): For $C_{38}H_{87}N_6O_6P_2$ $(M+H)^{+}$ m/z calculated 785.61568, found 785.61479

^{1}H NMR spectrum of compound **70**

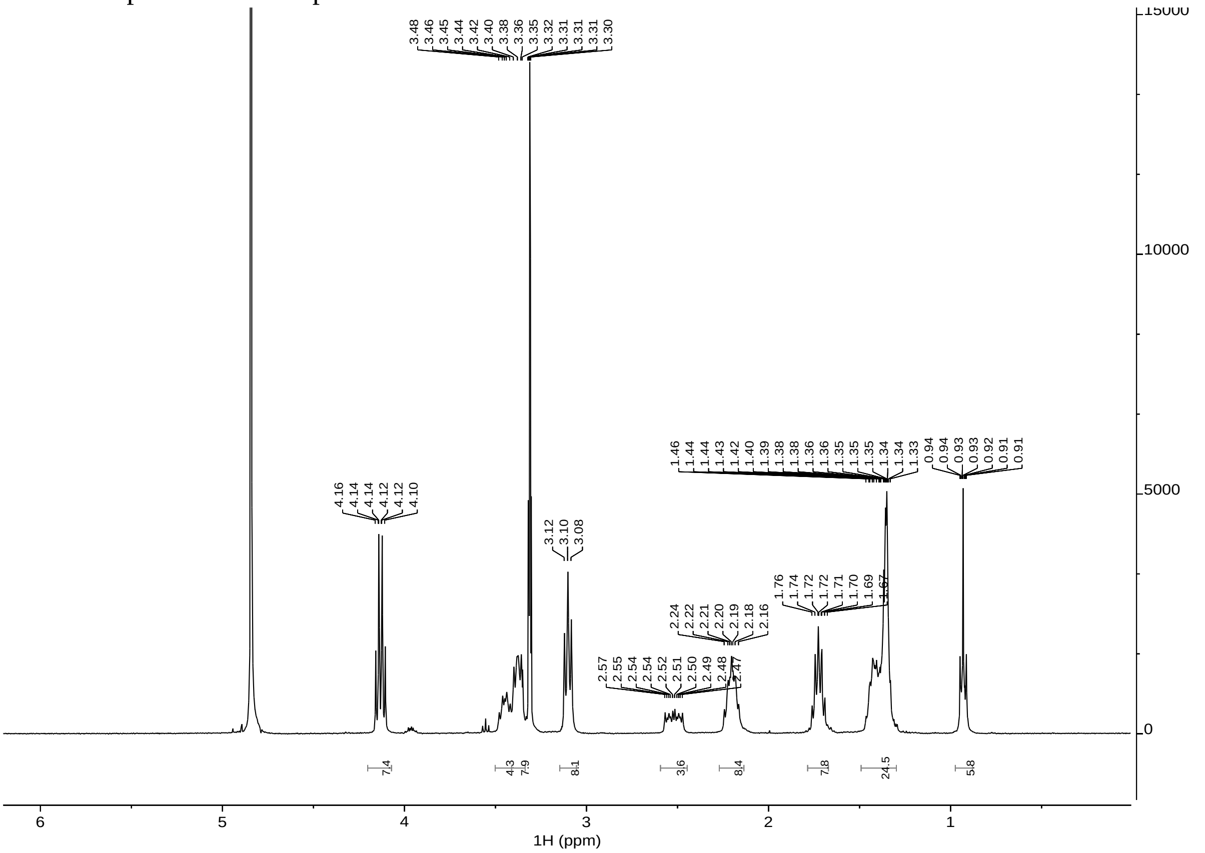

^{13}C NMR spectrum of compound **70**

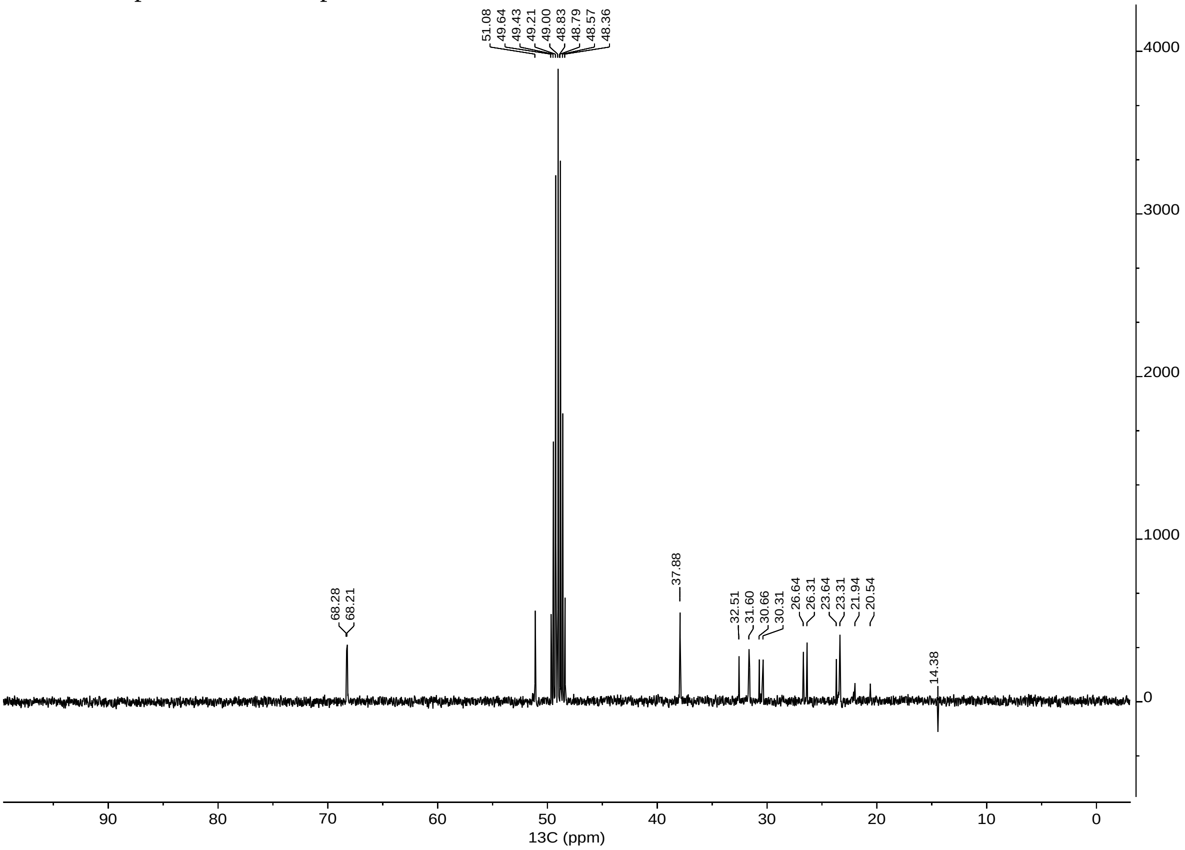

^{31}P{^{1}H} NMR spectrum of compound **70**

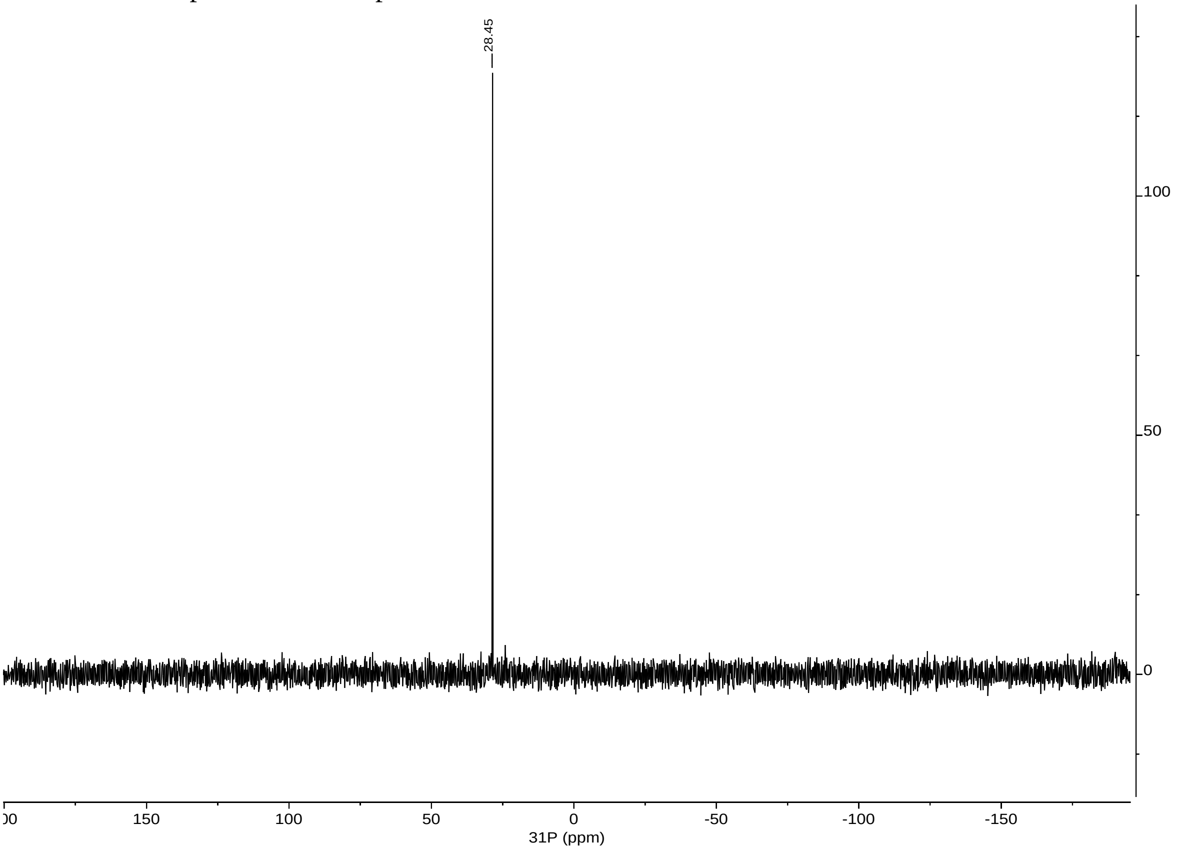

LC-MS chromatograms for compound **70**

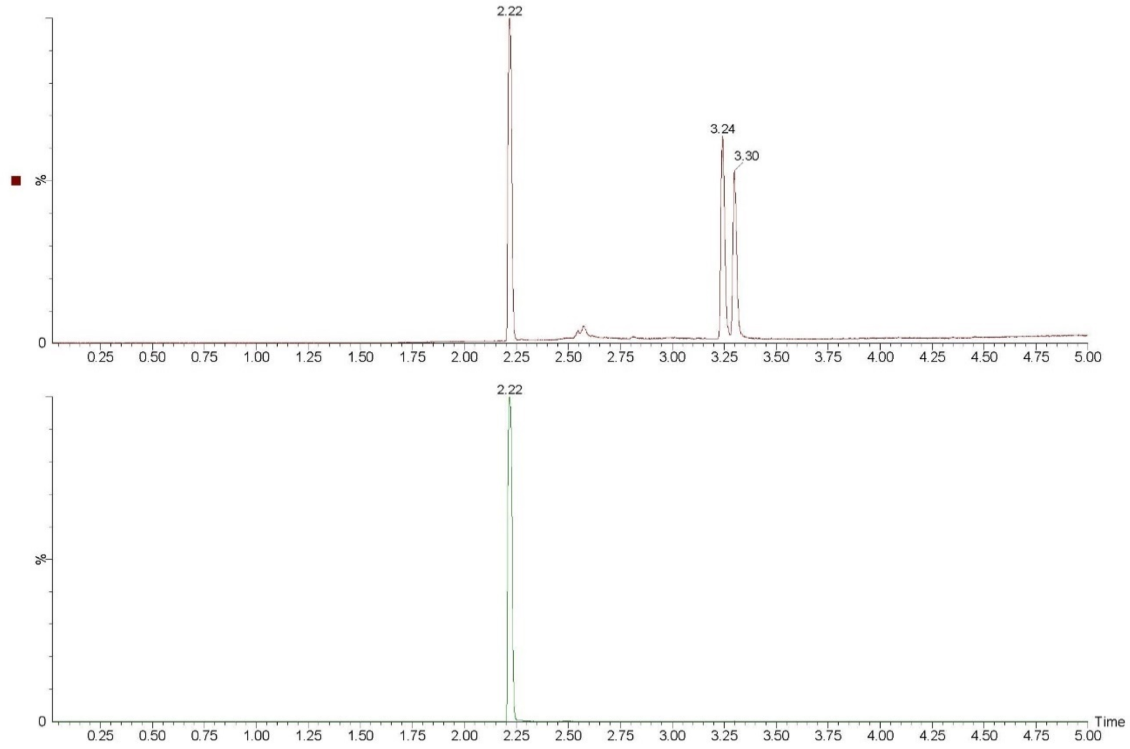

Decane-1,10-diyl dioctyl bis((2-(bis(3-aminopropyl)amino)ethyl)phosphonate) hexahydrochloride (71)

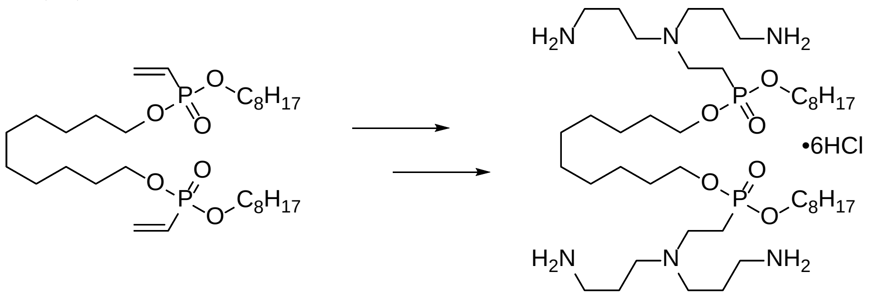

The title compound was prepared according to general methods **D** and **E** from vinylphosphonate dimer **S79** (0.91 g, 1.57 mmol) in 66% overall yield (1.1 g, 1.04 mmol) as a white solid.

^{1}H NMR (500.2 MHz, CD_3OD): 4.16–4.10 (m, 8H, CH_2O), 3.49–3.43 (m, 4H, PCH_2CH_2), 3.43–3.36 (m, 8H, C$H_2(CH_2)_2NH_2$), 3.11 (t, 8H, J = 7.5 Hz, CH_2NH_2), 2.59–2.49 (m, 4H, PCH_2), 2.26–2.17 (m, 8H, C$H_2CH_2NH_2$), 1.76–1.68 (m, 8H, CH_2CH_2O), 1.46–1.26 (m, 32H, $CH_3(CH_2)_2$, $(CH_2)_3(CH_2)_2O$), 0.93–0.88 (m, 6H, CH_3).

^{13}C NMR (125.8 MHz, CD_3OD): 68.23 (d, J = 6.7 Hz, CH_2O), 51.04 ($CH_2(CH_2)_2NH_2$), 48.85 (PCH_2CH_2), 37.88 (CH_2NH_2), 32.96 ($CH_3CH_2CH_2$), 31.59, 31.58 (d, J = 5.8 Hz, CH_2CH_2O), 30.62, 30.34, 30.29, 30.24, 26.64, 26.62 ($(CH_2)_3(CH_2)_2O$), 23.69 (CH_3CH_2), 23.22 ($CH_2CH_2NH_2$), 21.28 (d, J = 140.2 Hz, PCH_2), 14.45 (CH_3).

^{31}P{^{1}H} NMR (202.5 MHz, CD_3OD): 26.64.

IR ν_{max} (KBr) 2956 (s), 2925 (vs), 2675 (m), 2620 (m), 2546 (m), 2055 (w, br), 1626 (w), 1608 (w, sh), 1543 (w), 1511 (w), 1468 (m), 1460 (m), 1401 (w), 1390 (w), 1378 (w, sh), 1338 (vw), 1305 (vw), 1263 (m), 1227 (s), 1075 (m), 1022 (s, sh), 996 (s), 722 (w).

HR-MS(ESI^{+}): For $C_{42}H_{96}N_6O_6P_2$ $(M+2H)^{2+}$ $m/2z$ calculated 421.34278, found 421.34273.

^{1}H NMR spectrum of compound **71**

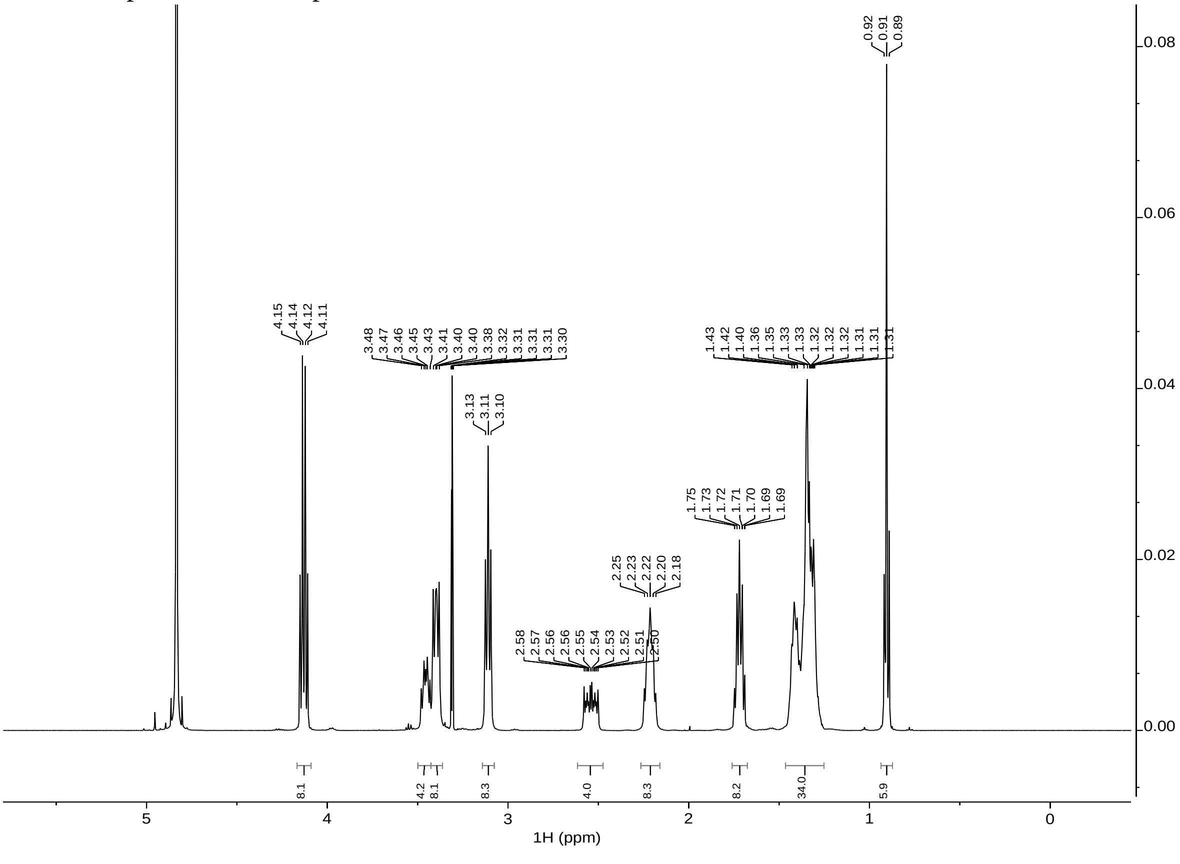

^{13}C NMR spectrum of compound **71**

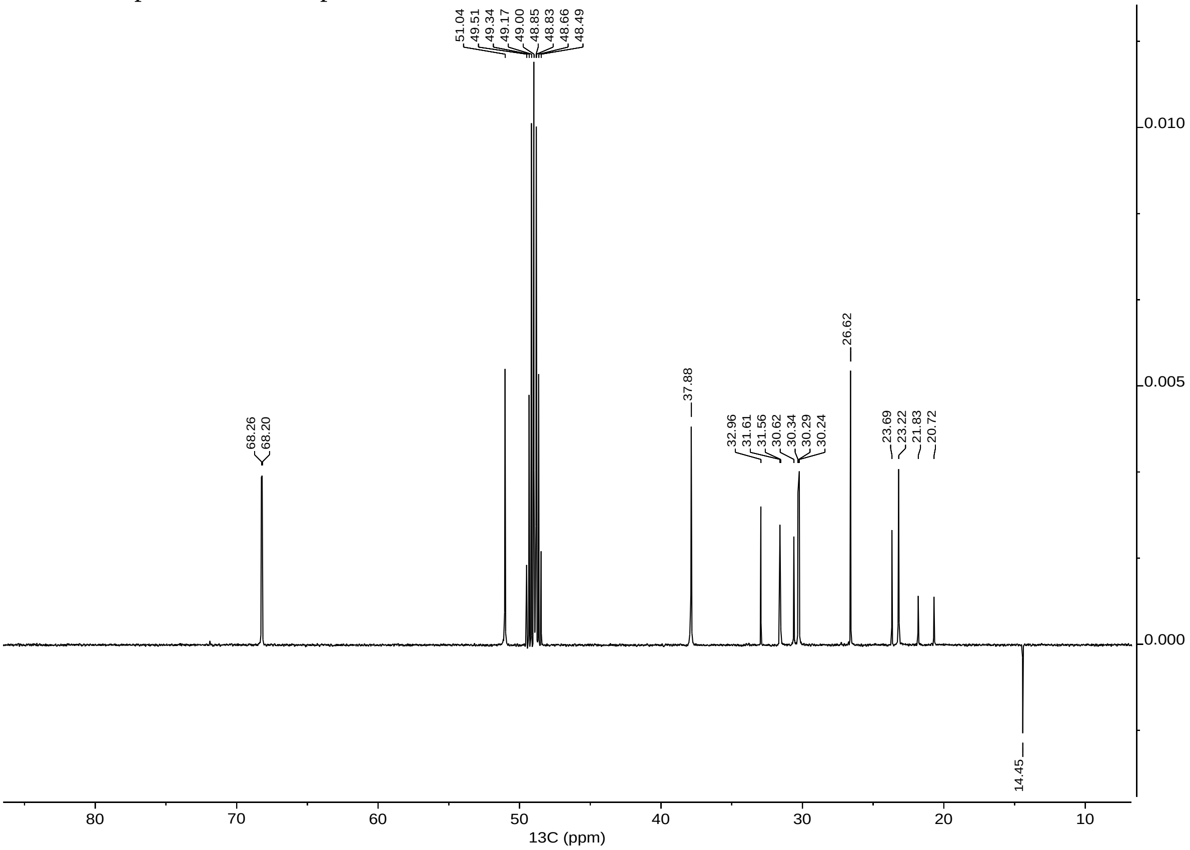

$^{31}P\{^{1}H\}$ NMR spectrum of compound **71**

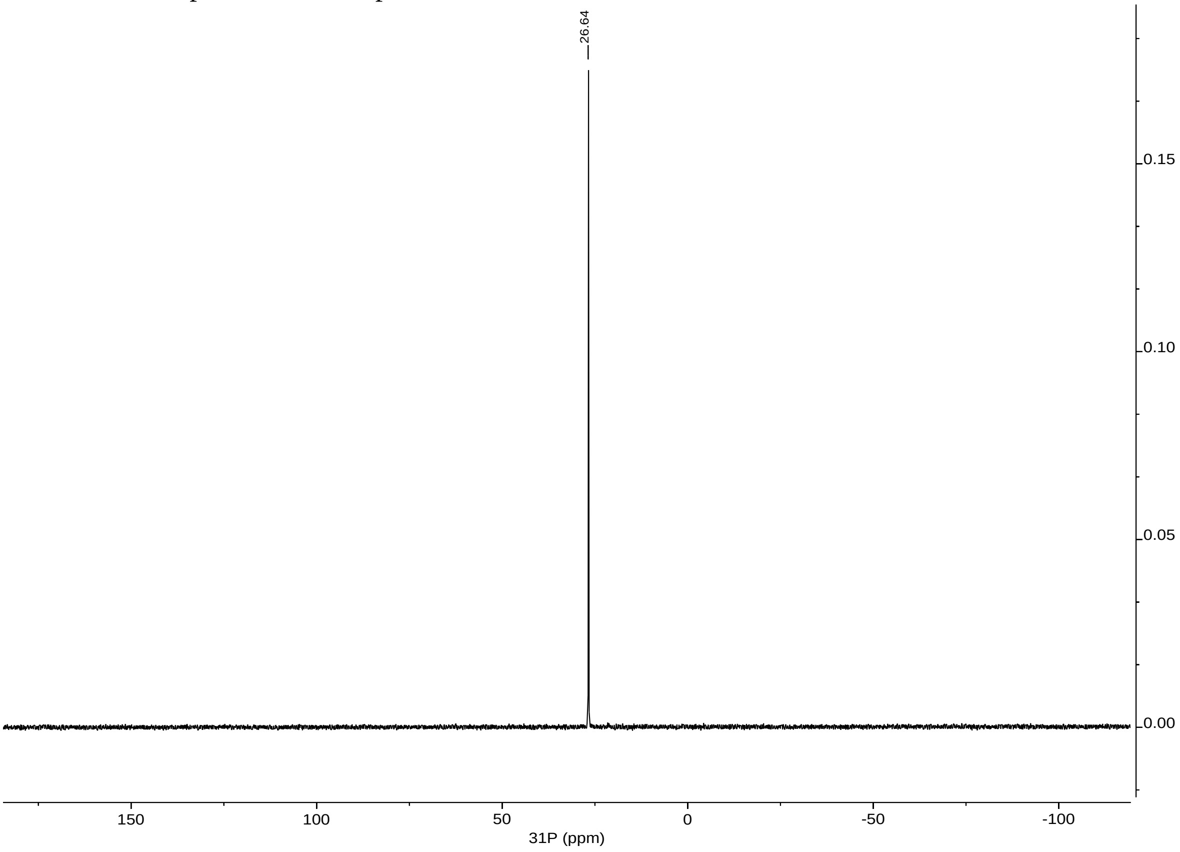

LC-MS chromatograms for compound **71**

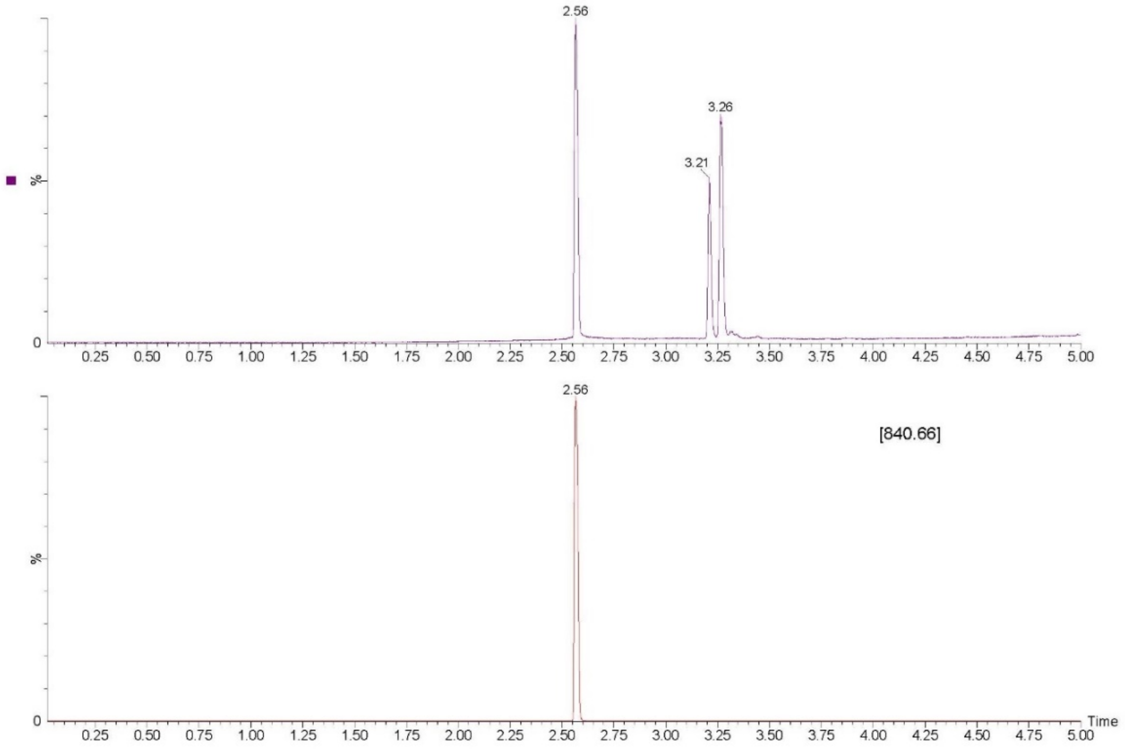

Decane-1,10-diyl diphenethyl bis((2-(bis(3-aminopropyl)amino)ethyl)phosphonate) hexahydro-chloride (72)

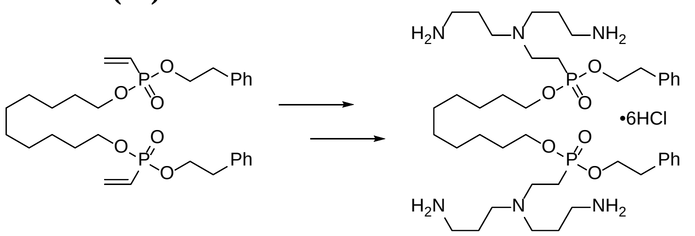

The title compound was prepared according to general methods **D** and **E** from vinylphosphonate dimer **S80** (0.33 g, 0.59 mmol) in 56% overall yield (0.34 g, 0.33 mmol) as a white solid.

^{1}H NMR (401 MHz, CD_3OD) δ 7.41–7.21 (m, 10H, Ph), 4.43–4.28 (m, 4H, CH_2CH$_2$Ph), 3.97 (dddd, *J* = 19.6, 10.0, 7.5, 3.3 Hz, 4H, OCH_2(CH$_2$)$_8$CH_2O), 3.38–3.31 (m, 12H, NCH_2), 3.09 (t, *J* = 7.5 Hz, 8H, CH_2NH$_2$), 3.03 (t, *J* = 6.6 Hz, 4H, CH_2Ph), 2.57–2.40 (m, 4H, PCH_2), 2.27–2.11 (m, 8H, CH_2CH$_2$NH$_2$), 1.69–1.58 (m, 4H, OCH$_2$CH_2CH$_2$), 1.48–1.25 (m, 12H, O(CH$_2$)$_2$(CH_2)$_3$).

^{13}C NMR (101 MHz, CD_3OD) δ 138.83 (C_{quat}), 130.25(C_{ortho}), 129.68(C_{meta}), 127.87(C_{para}), 68.58 (d, *J* = 6.9 Hz, CH$_2$CH$_2$Ph), 68.08 (d, *J* = 6.9 Hz, OCH$_2$(CH$_2$)$_4$), 51.00 (CH$_2$(CH$_2$)$_2$NH$_2$), 48.67 (PCH$_2$$CH_2$), 37.85 ($CH_2NH_2$), 37.71 (d, *J* = 6.2 Hz, CH$_2$Ph), 31.49 (d, *J* = 5.6 Hz, OCH$_2$$CH_2CH_2$), 30.57 (O(CH$_2$)$_2$$CH_2$), 30.23 (O(CH$_2$)$_3$$CH_2$), 26.53 (O(CH$_2$)$_4$$CH_2$), 23.21 ($CH_2CH_2NH_2$), 21.16 (d, *J* = 140.5 Hz, PCH$_2$).

^{31}P{^{1}H} NMR (162 MHz, CD_3OD) δ 28.52.

IR ν_{max} (KBr) 2965 (vs, sh), 2927 (vs), 2854 (vs), 2745 (s, sh), 2633 (s, br), 2558 (s), 2027 (w, vbr), 1615 (m, sh), 1605 (m), 1520 (m, sh), 1497 (m), 1470 (s), 1454 (s), 1412 (m), 1391 (m), 1256 (m), 1236 (s), 1156 (w), 1062 (s), 1088 (m, sh), 1009 (vs), 967 (s, sh), 902 (m), 750 (m), 725 (w, sh), 700 (m), 574 (w), 495 (m).

HR-MS(ESI$^+$): For $C_{42}H_{79}N_6O_6P_2$ $(M+H)^+$ *m/z* calculated 825.55308, found 825.553

^{1}H NMR spectrum of compound **72**

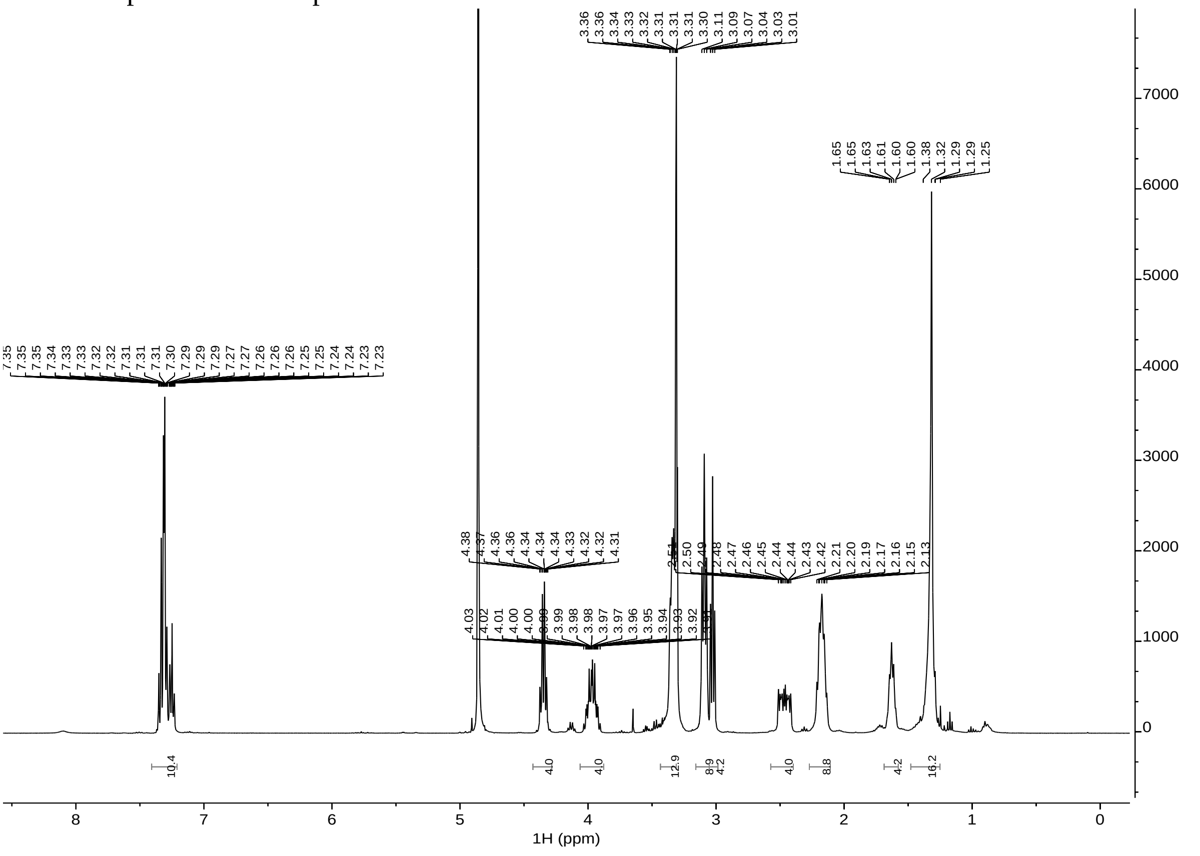

^{13}C NMR spectrum of compound **72**

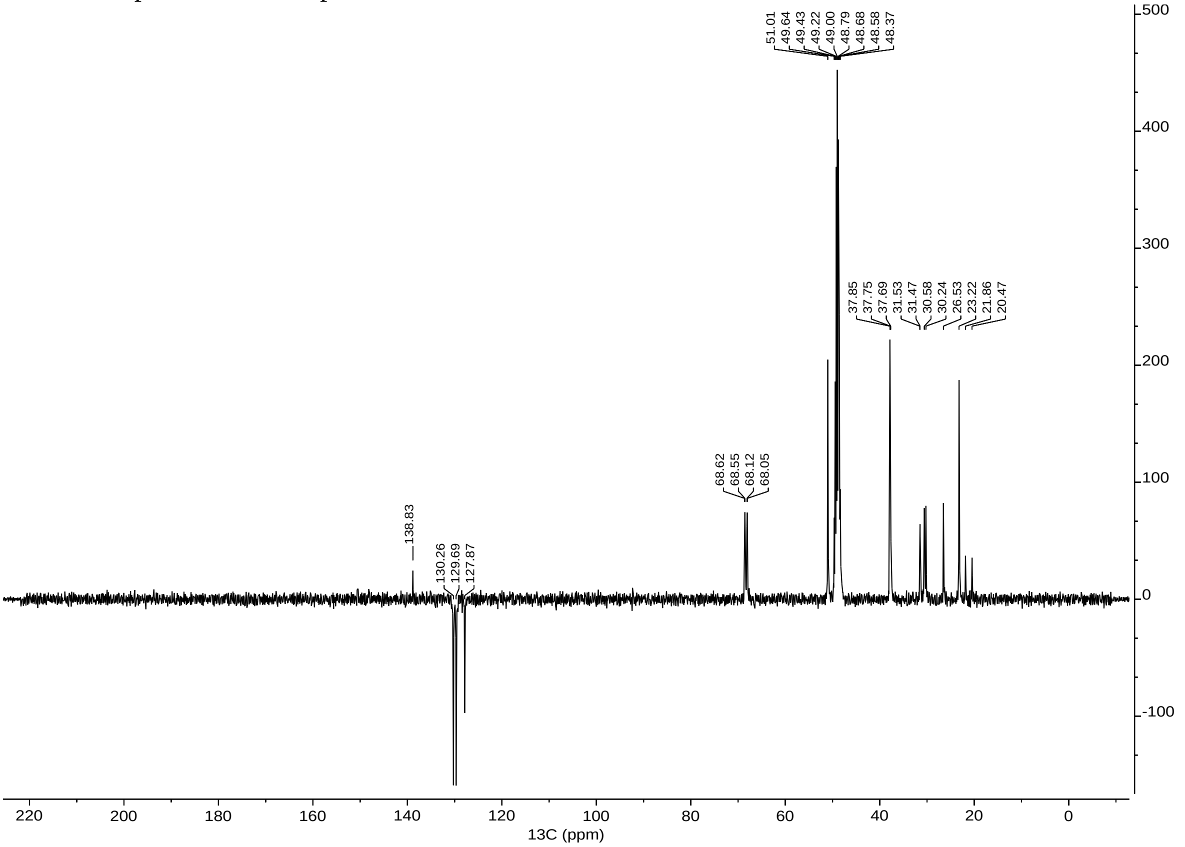

^{31}P{^{1}H} NMR spectrum of compound **72**

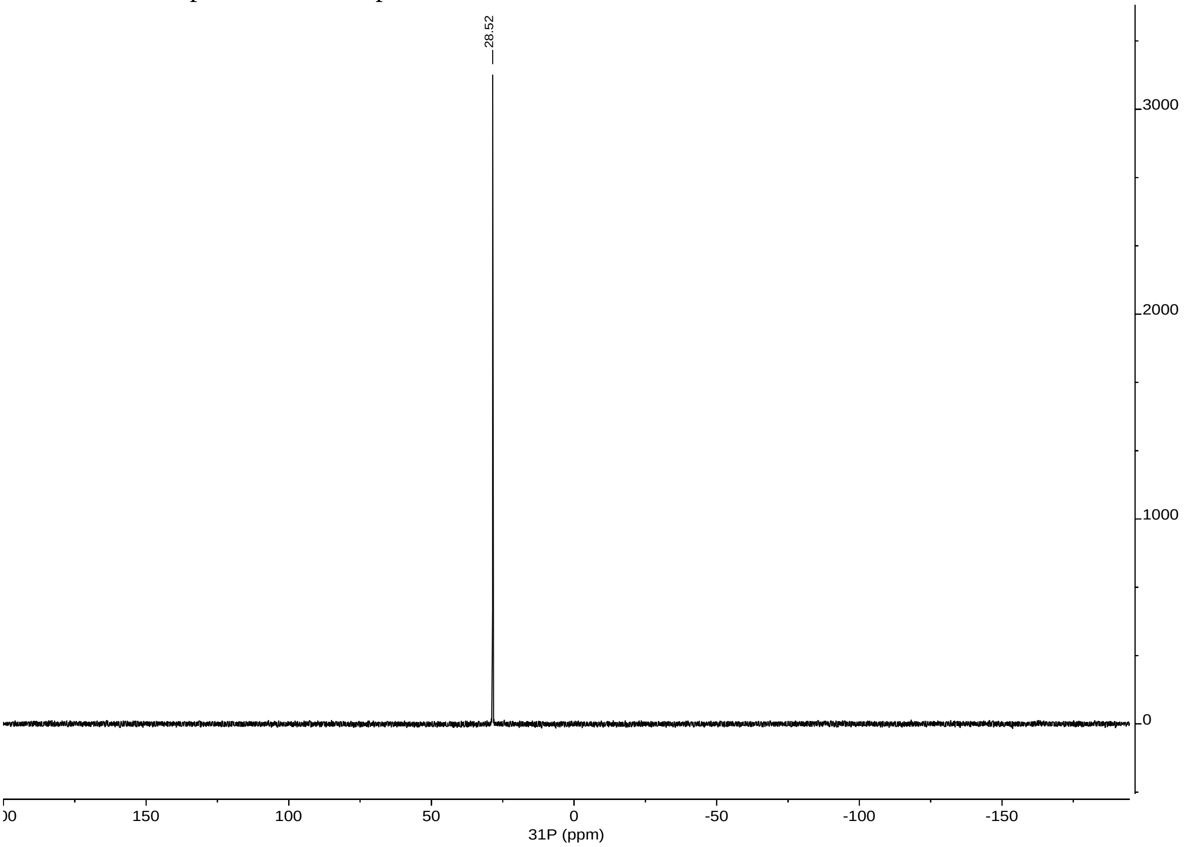

LC-MS chromatograms for compound **72**

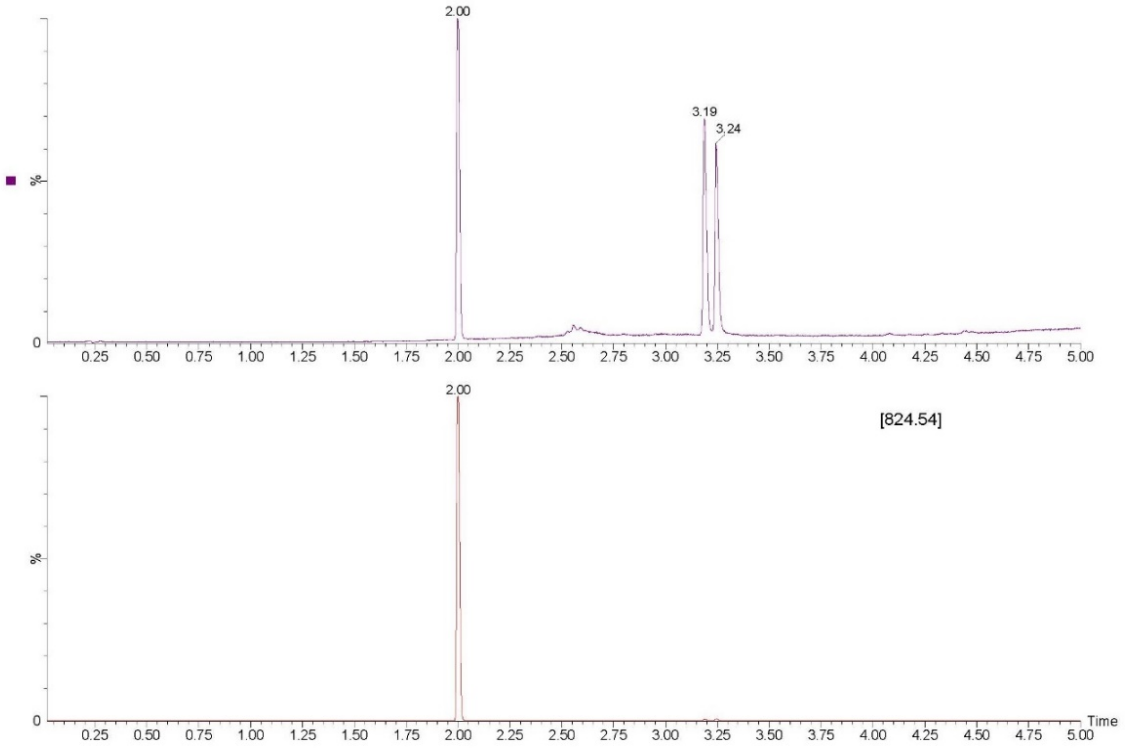

Decane-1,10-diyl didecyl bis((2-(bis(3-aminopropyl)amino)ethyl)phosphonate) hexahydrochloride (73)

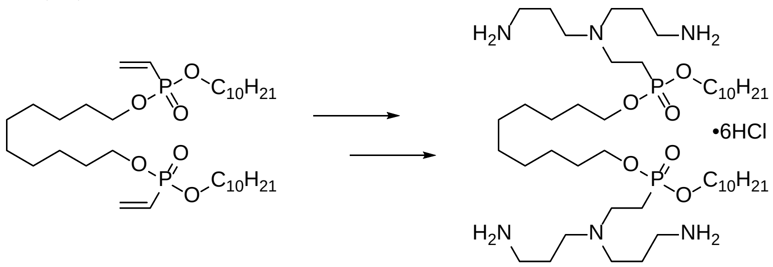

The title compound was prepared according to general methods **D** and **E** from vinylphosphonate dimer **S81** (0.60 g, 0.94 mmol) in 65% overall yield (0.68 g, 0.61 mmol) as a white solid.

^{1}H NMR (500.2 MHz, CD_3OD): 4.16–4.10 (m, 8H, CH_2O), 3.49–3.42 (m, 4H, PCH$_2$CH_2), 3.42–3.36 (m, 8H, CH_2(CH$_2$)$_2$NH$_2$), 3.11 (t, 8H, J = 7.5 Hz, CH_2NH$_2$), 2.58–2.49 (m, 4H, PCH_2), 2.25–2.16 (m, 8H, CH_2CH$_2$NH$_2$), 1.76–1.68 (m, 8H, CH_2CH$_2$O), 1.46–1.25 (m, 40H, CH$_3$(CH_2)$_4$, (CH_2)$_3$(CH$_2$)$_2$O), 0.93–0.88 (m, 6H, CH_3).

^{13}C NMR (125.8 MHz, CD_3OD): 68.24 (d, J = 6.6 Hz, CH$_2$O), 51.06 (CH$_2$(CH$_2$)$_2$NH$_2$), 48.83 (PCH$_2$$CH_2$), 37.87 ($CH_2NH_2$), 33.05 (CH$_3CH_2$$CH_2$), 31.62, 31.59 (d, J = 5.8 Hz, CH$_3$(CH$_2$)$_7$$CH_2CH_2O, OCH_2$$CH_2$(CH$_2$)$_6$$CH_2CH_2$O), 30.69, 30.66, 30.45, 30.31, 30.29, 26.64, 26.62, 23.72 (CH$_3$(CH$_2$)$_7$(CH$_2$)$_2$O, O(CH$_2$)$_2$(CH$_2$)$_6$(CH$_2$)$_2$O), 23.26 (NCH$_2$$CH_2CH_2NH_2$), 21.26 (d, J = 140.3 Hz, PCH$_2$CH$_2$N), 14.46 (CH$_3$(CH$_2$)$_8$CH$_2$O).

^{31}P{^{1}H} NMR (202.5 MHz, CD_3OD): 26.65.

IR ν_{max} (KBr) 2956 (vs), 2925 (vs), 2854 (vs), 2675 (m, br), 2620 (m, br), 2546 (s, br), 2059 (w, br), 1628 (m, sh), 1607 (m), 1542 (m), 1510 (m), 1484 (m), 1468 (m), 1456 (m, sh), 1401 (w), 1390 (w), 1377 (w, sh), 1338 (vw), 1305 (w), 1227 (s), 1075 (m), 1022 (s, sh), 992 (s), 722 (w).

HR-MS(ESI^{+}): For $C_{46}H_{104}N_6O_6P_2$ $(M+2H)^{2+}$ $m/2z$ calculated 449.37408, found 449.37398.

^{1}H NMR spectrum of compound **73**

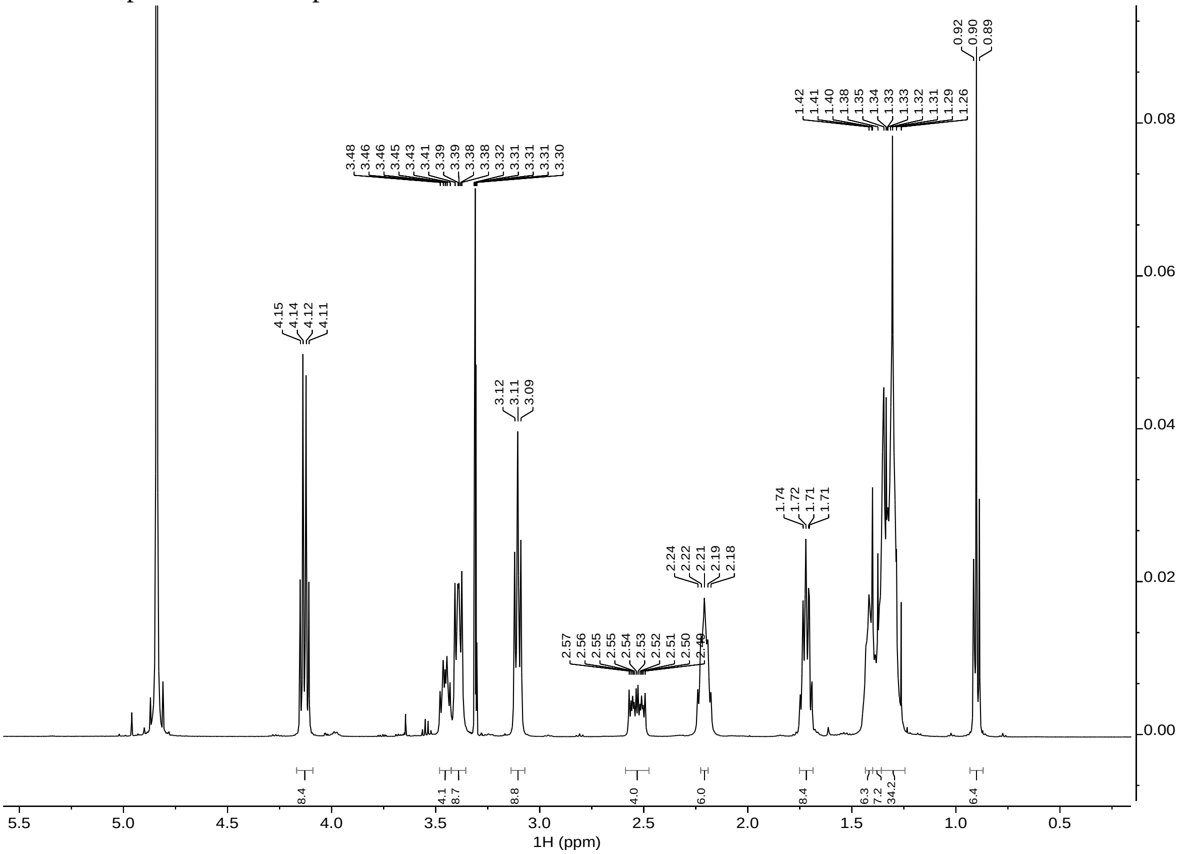

^{13}C NMR spectrum of compound **73**

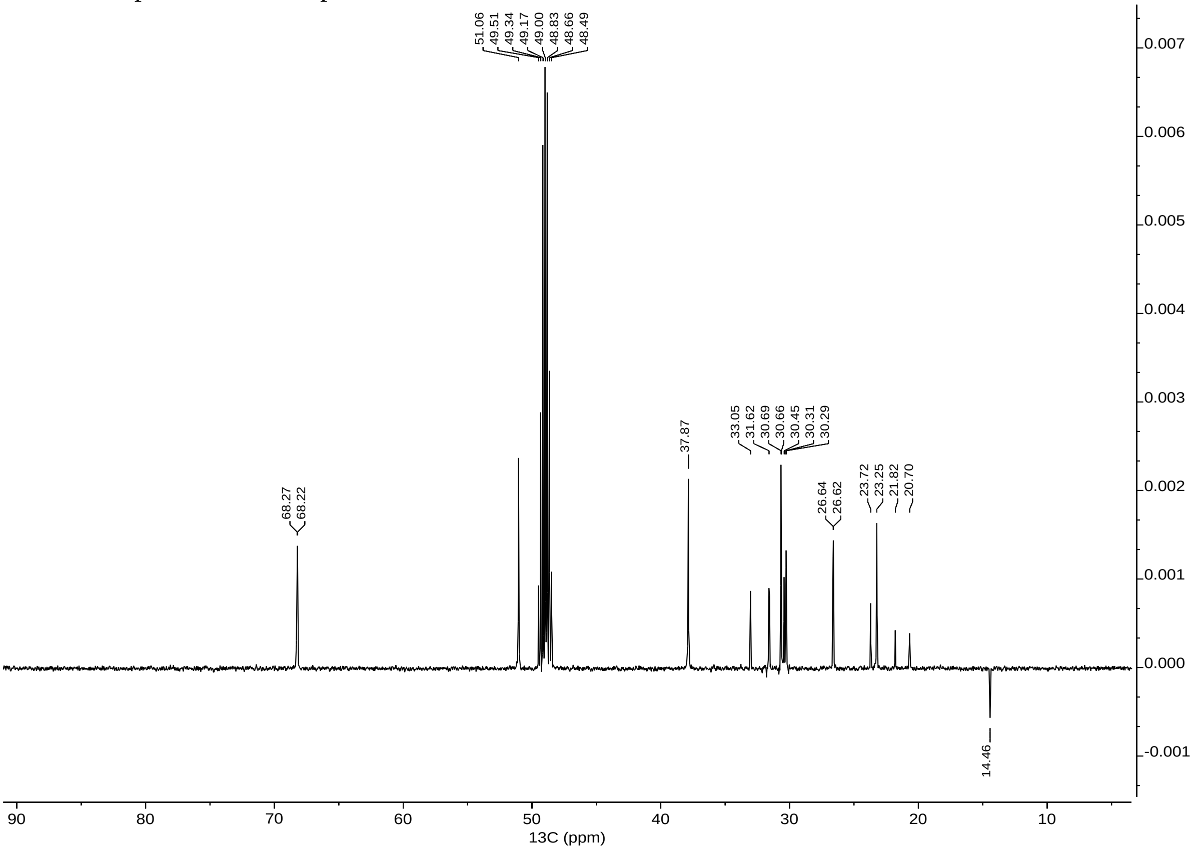

^{31}P{^{1}H} NMR spectrum of compound **73**

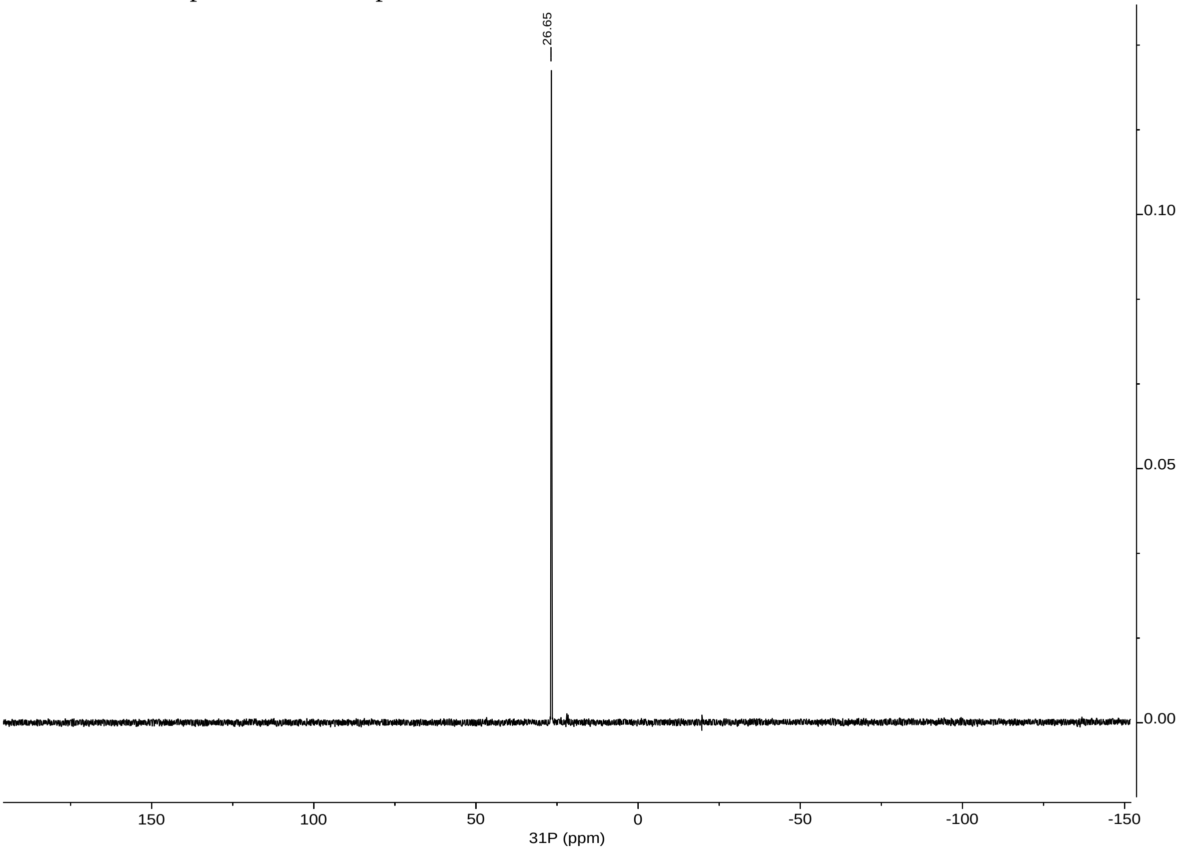

LC-MS chromatograms for compound **73**

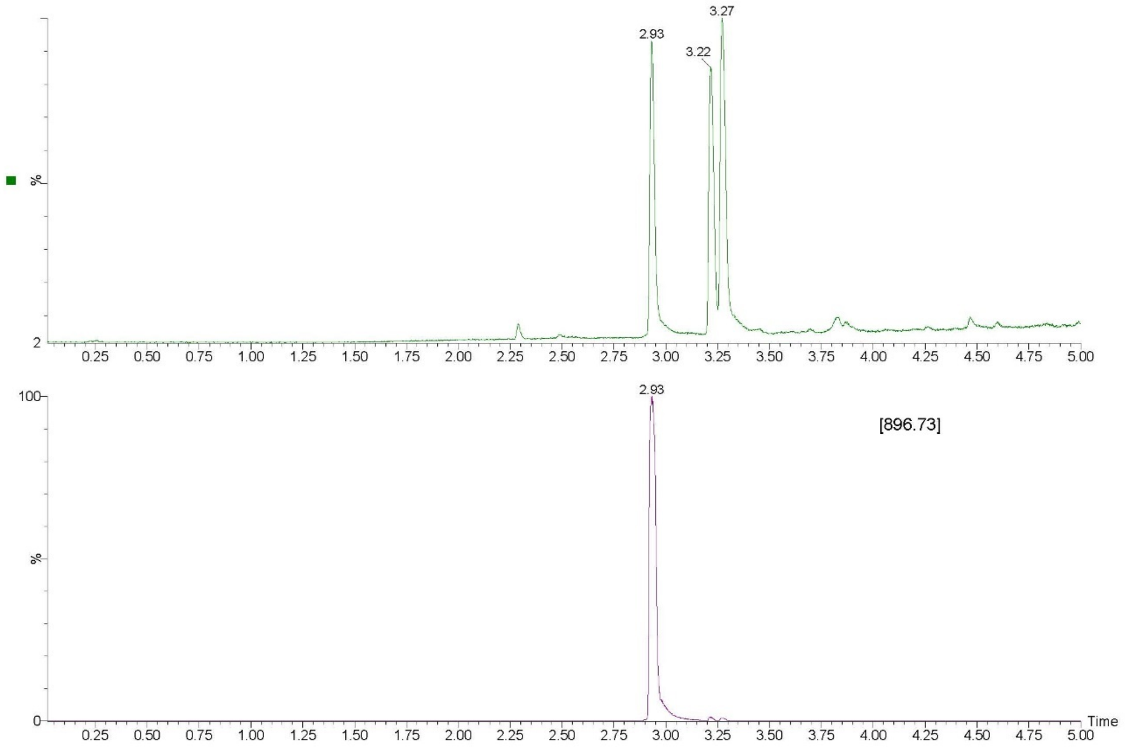

Diisobutyl dodecane-1,12-diyl bis((2-(bis(3-aminopropyl)amino)ethyl)phosphonate) hexahydrochloride (74)

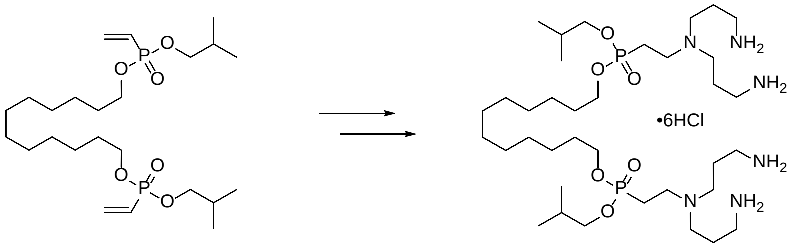

The title compound was prepared according to general methods **D** and **E** from vinylphosphonate dimer **S82** (360 mg, 0.728 mmol) in 6% overall yield (43 mg, 43.6 µmol) as a white solid.

^{1}H NMR (401 MHz, CD_3OD) δ 4.15 (dt, *J* = 7.8, 6.6 Hz, 4H, OC*H*$_2$CH$_2$), 3.92 (td, *J* = 6.6, 2.4 Hz, 4H, OC*H*$_2$CH), 3.55–3.42 (m, 4H, PCH$_2$C*H*$_2$), 3.43–3.34 (m, 8H, C*H*$_2$(CH$_2$)$_2$NH$_2$), 3.11 (t, *J* = 7.5 Hz, 8H, C*H*$_2$NH$_2$), 2.67–2.46 (m, 4H, PC*H*$_2$), 2.33–2.15 (m, 8H, C*H*$_2$CH$_2$NH$_2$), 1.99 (dp, *J* = 13.3, 6.7 Hz, 2H, OCH$_2$C*H*), 1.86–1.65 (m, 4H, OCH$_2$C*H*$_2$), 1.53–1.28 (m, 16H, O(CH$_2$)$_2$(C*H*$_2$)$_4$), 1.00 (d, *J* = 6.7 Hz, 12H, C*H*$_3$).

^{13}C NMR (101 MHz, CD_3OD) δ 73.92 (d, *J* = 6.6 Hz, O*C*H$_2$CH), 68.29 (d, *J* = 6.7 Hz, O*C*H$_2$CH$_2$), 51.09 (*C*H$_2$(CH$_2$)$_2$NH$_2$), 37.87 (*C*H$_2$NH$_2$), 31.63 (d, *J* = 5.5 Hz, OCH$_2$*C*H$_2$), 30.74, 30.71, 30.47 (d, *J* = 6.4 Hz, OCH$_2$*C*H), 30.32, 26.62 (O(CH$_2$)$_2$(*C*H$_2$)$_2$), 23.32 (*C*H$_2$CH$_2$NH$_2$), 21.19 (d, *J* = 140.6 Hz, P*C*H$_2$), 19.03 (*C*H$_3$).

^{31}P{^{1}H} NMR (162 MHz, CD_3OD) δ 27.12.

IR ν_{max}(KBr) 3200–2700 (vs, vbr), 2961 (s), 2927 (s), 2854 (m), 2700–2500 (m), 1607 (m), 1510 (w), 1470 (m), 1400 (w), 1369 (w), 1226 (m), 1002 (s), ~869 (w, sh), 851 (m), 768 (w), 724 (vw).

HR-MS(ESI$^+$): For $C_{36}H_{83}O_6N_6P_2$ (M+H)$^+$ *m/z* calculated 757.58438, found 757.58375.

^{1}H NMR spectrum of compound **74**

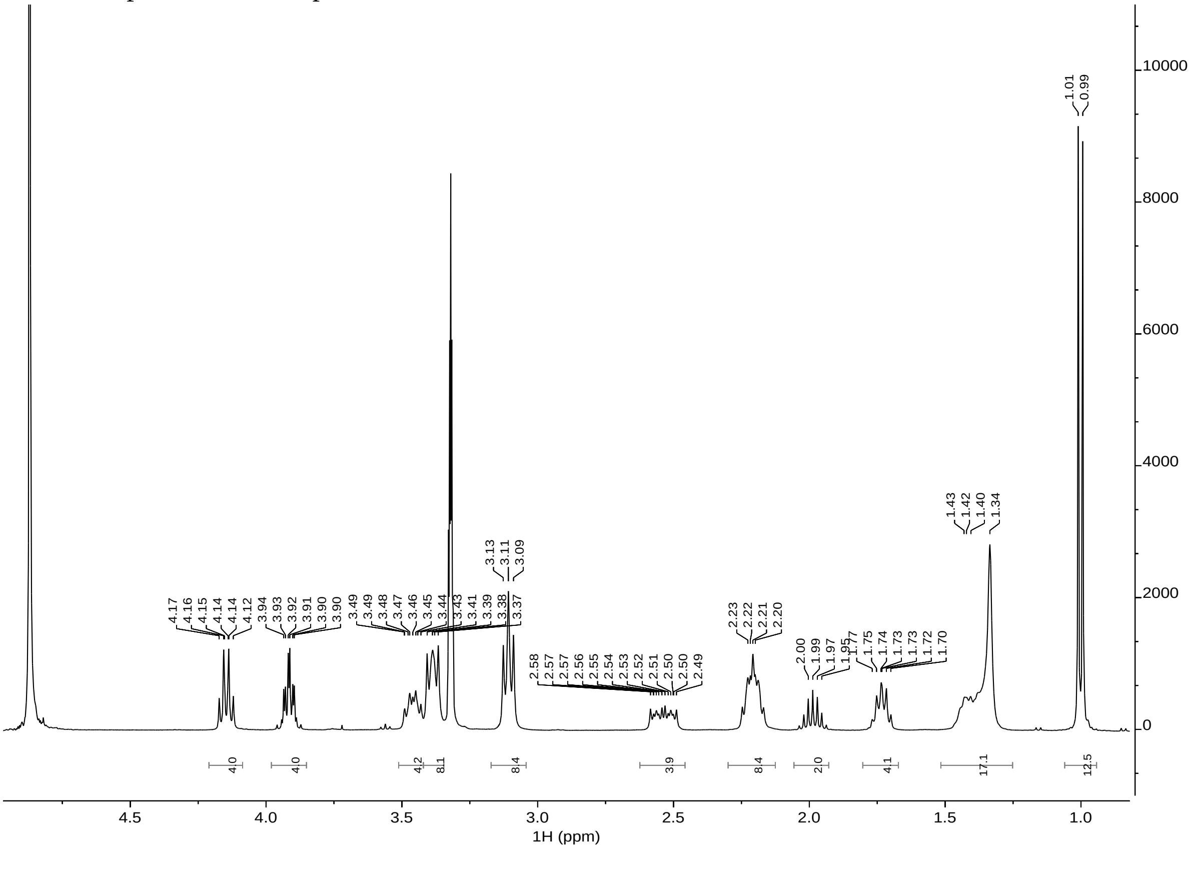

^{13}C NMR spectrum of compound **74**

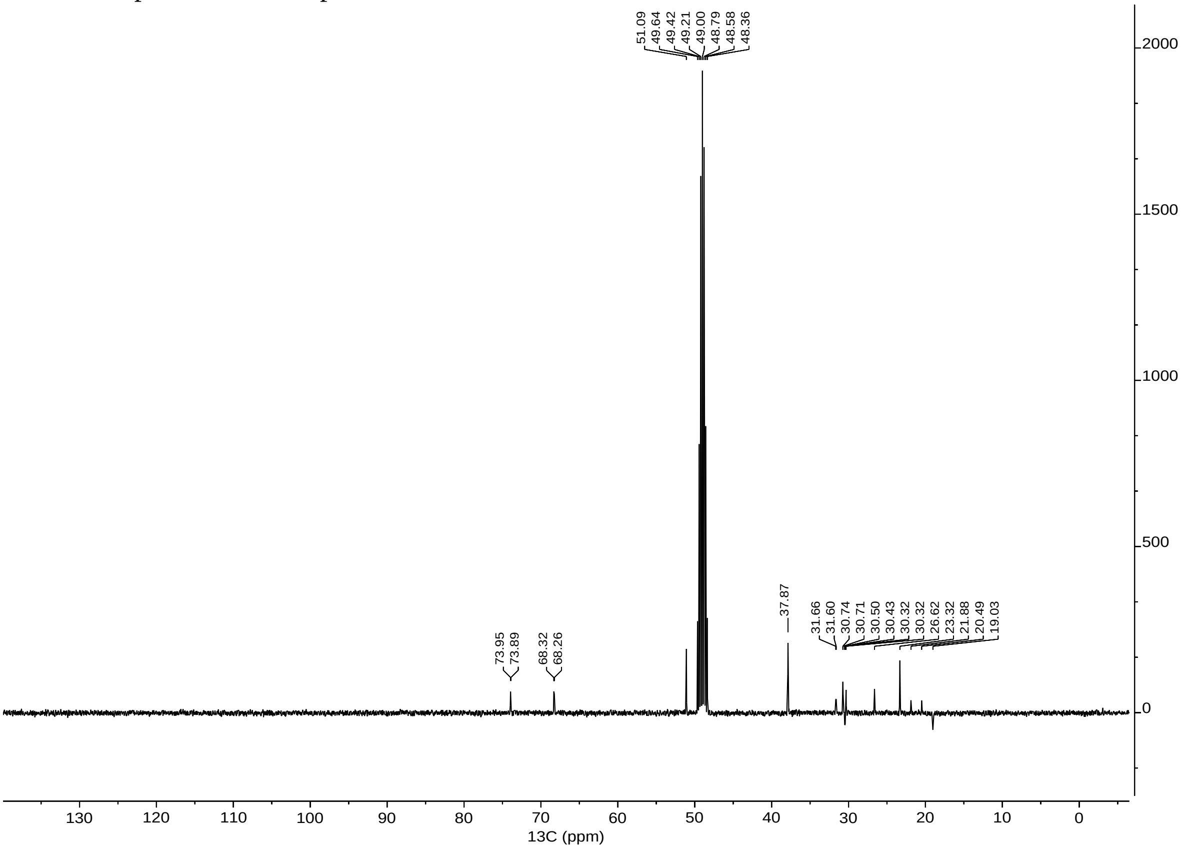

^{31}P{^{1}H} NMR spectrum of compound **74**

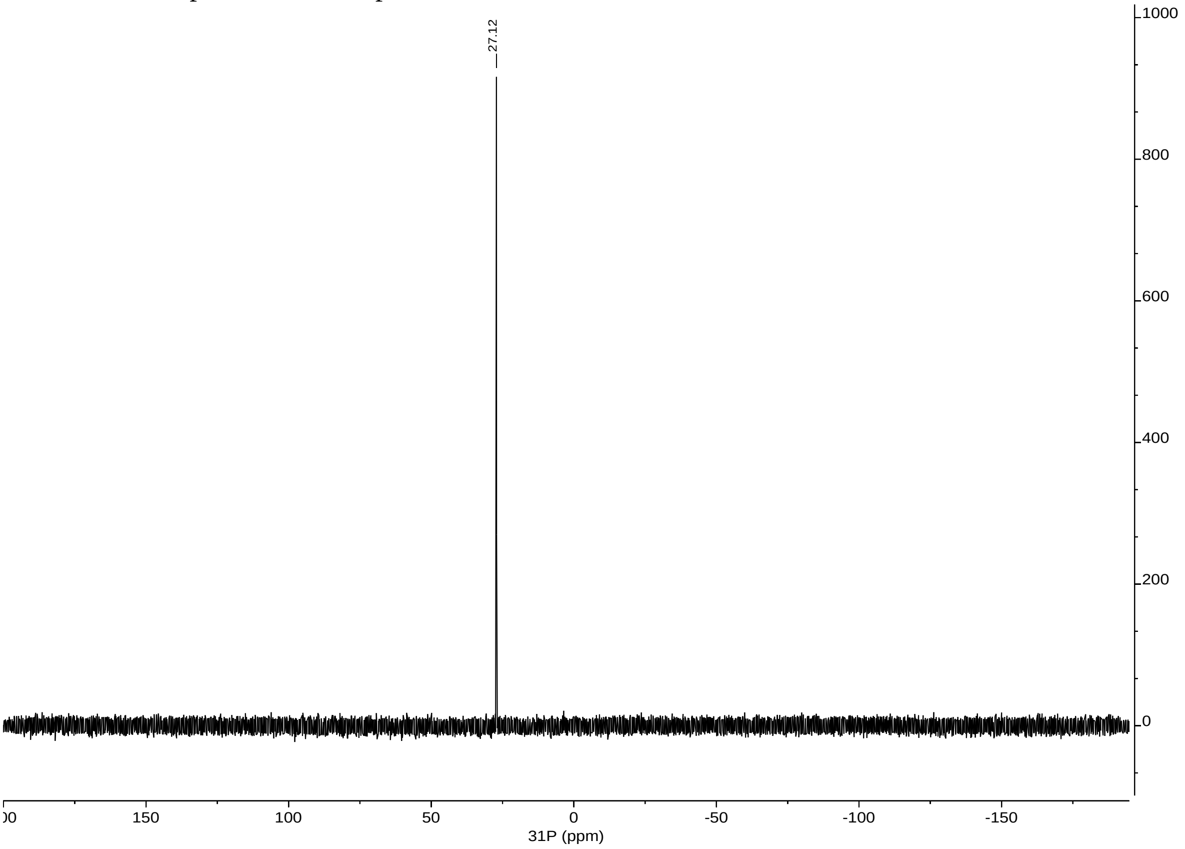

LC-MS chromatograms for compound **74**

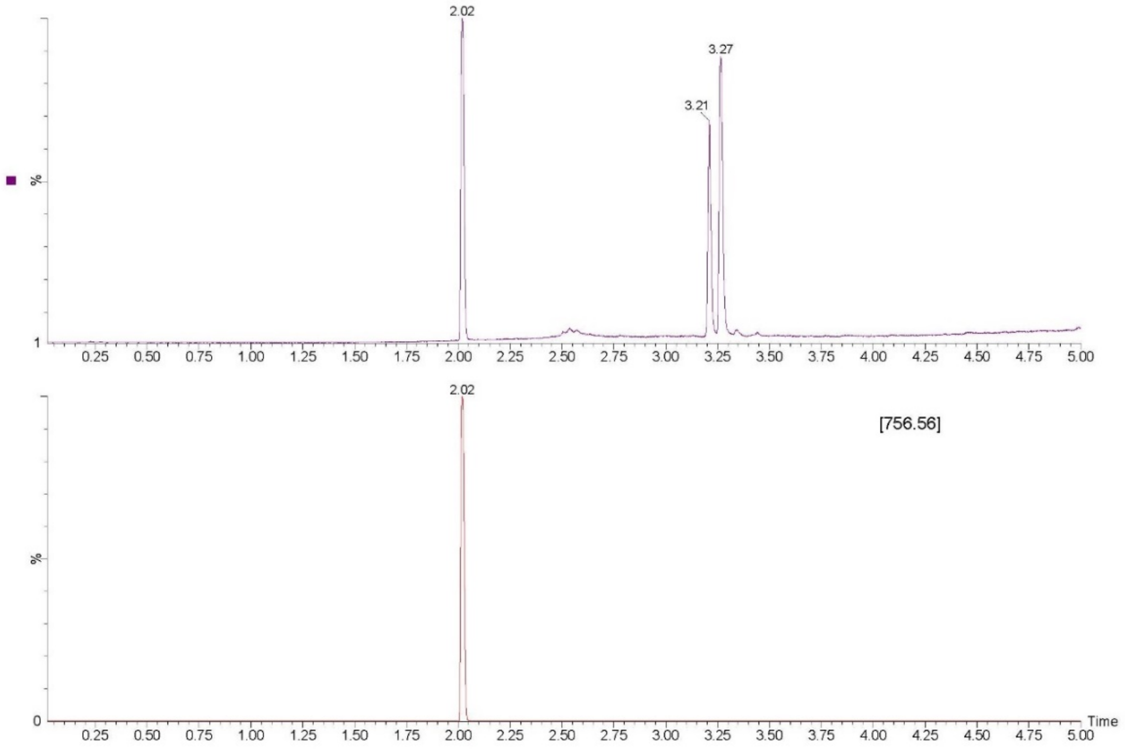

Dibutyl dodecane-1,12-diyl bis((2-(bis(3-aminopropyl)amino)ethyl)phosphonate) hexahydrochloride (75)

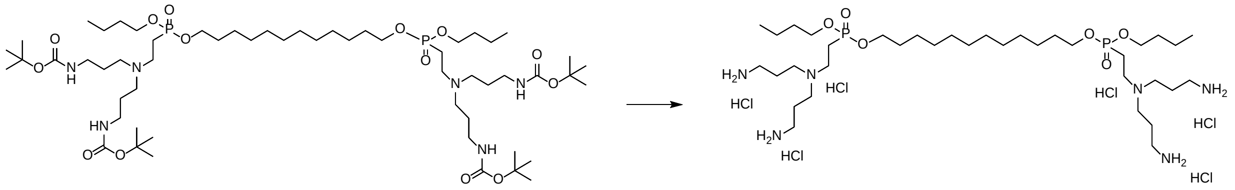

The title compound was prepared according to general method **E** from Boc derivative **S122** (1.0 g, 0.84 mmol) in 89% yield (0.73 g, 0.75 mmol) as a white solid.

^{1}H NMR (400 MHz, CD_3OD): 4.20–4.07 (m, 8H, CH_2O), 3.49–3.41 (m, 4H, PCH_2CH_2), 3.41–3.35 (m, 8H, CH_2(CH)$_2$NH$_2$), 3.10 (t, 8H, J = 7.5 Hz, CH_2NH$_2$), 2.61–2.43 (m, 4H, PCH_2), 2.28–2.10 (m, 8H, CH_2CH$_2$NH$_2$), 1.80–1.64 (m, 8H, CH_2CH$_2$O), 1.53–1.28 (m, 20H, CH$_3$CH_2, (CH_2)$_4$(CH$_2$)$_2$O), 0.98 (t, 6H, J = 7.4 Hz, CH_3).

^{13}C NMR (101 MHz, CD_3OD): 68.24 (d, J = 6.7 Hz), 67.90 (d, J = 6.8 Hz CH$_2$O), 51.05 (CH$_2$(CH$_2$)$_2$NH$_2$), 48.84 (PCH$_2$$CH_2$), 37.87 ($CH_2NH_2$), 33.60 (d, J = 6.0 Hz), 31.59 (d, J = 6.0 Hz, CH$_2$CH$_2$O), 30.70, 30.67, 30.29, 26.60 ((CH$_2$)$_4$(CH$_2$)$_2$O), 23.24 (CH$_2$CH$_2$NH$_2$), 21.27 (d, J = 140.8 Hz, PCH$_2$), 19.79 (CH$_3$$CH_2$), 13.96 ($CH_3$).

^{31}P{^{1}H} NMR (162 MHz, CD_3OD): 28.35.

IR ν_{max} (KBr) 2930 (s), 2780 (m), 2630 (m), 2557 (m), 1597 (m), 1467 (m), 1226 (m), 1168 (m, sh), 1070 (m, sh), 1064 (m), 1020 (s), 1004 (s).

HR-MS(ESI^{+}): For $C_{36}H_{83}N_6O_6P_2$ (M+H)$^{+}$ m/z calculated 757.58438, found 757.58402.

^{1}H NMR spectrum of compound **75**

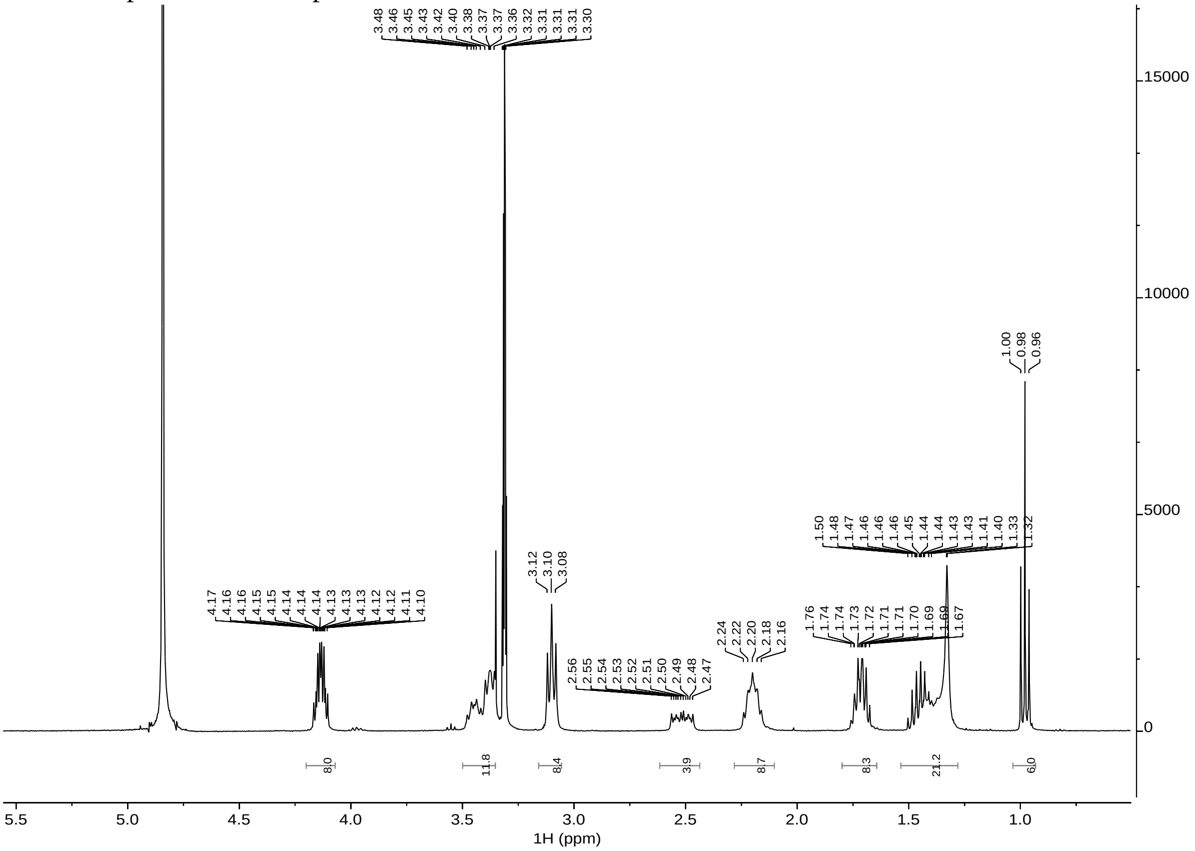

^{13}C NMR spectrum of compound **75**

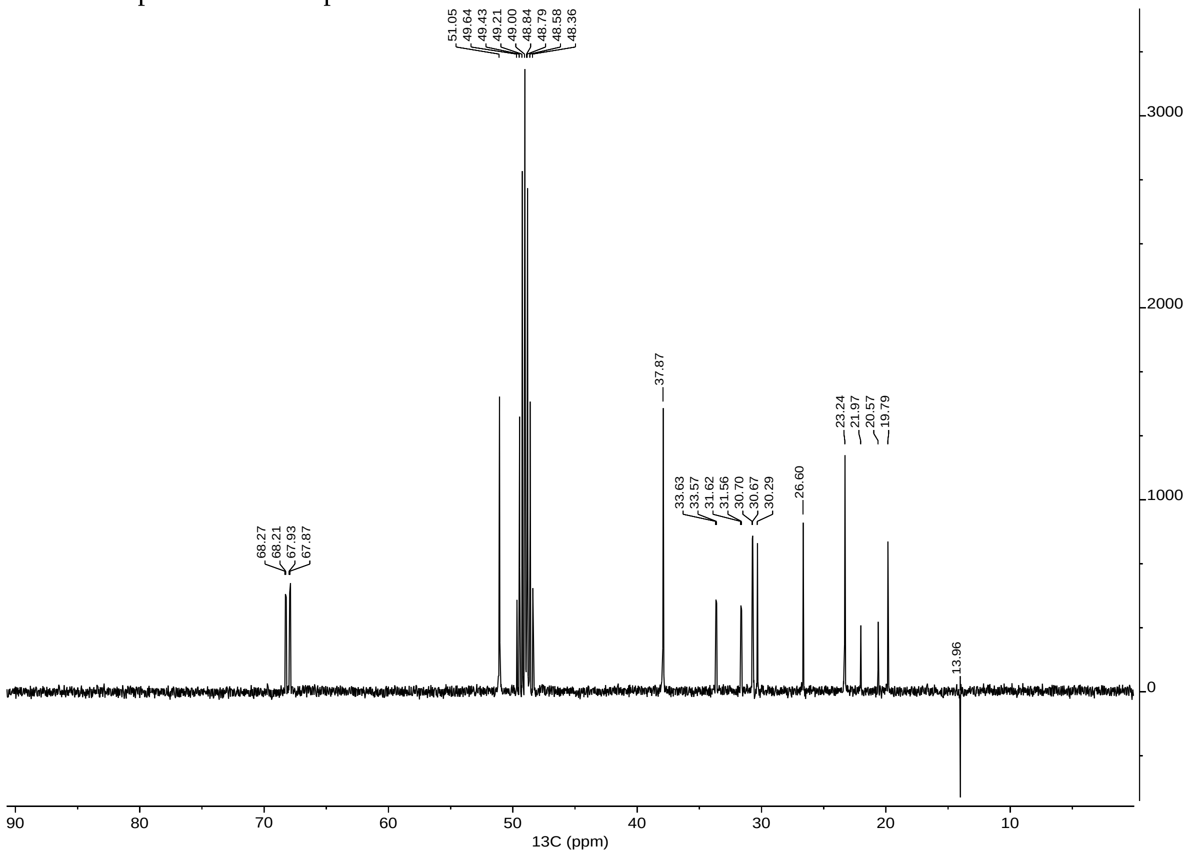

^{31}P{^{1}H} NMR spectrum of compound **75**

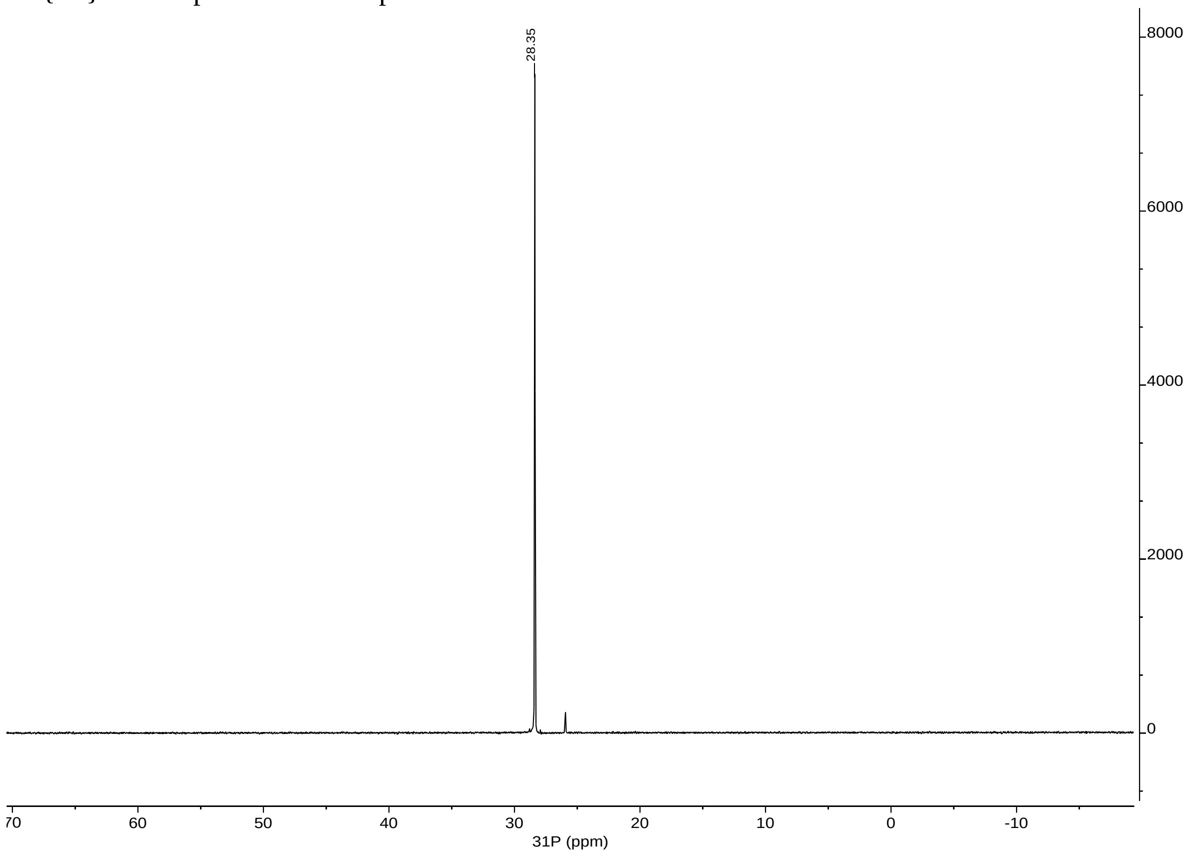

LC-MS chromatograms for compound **75**

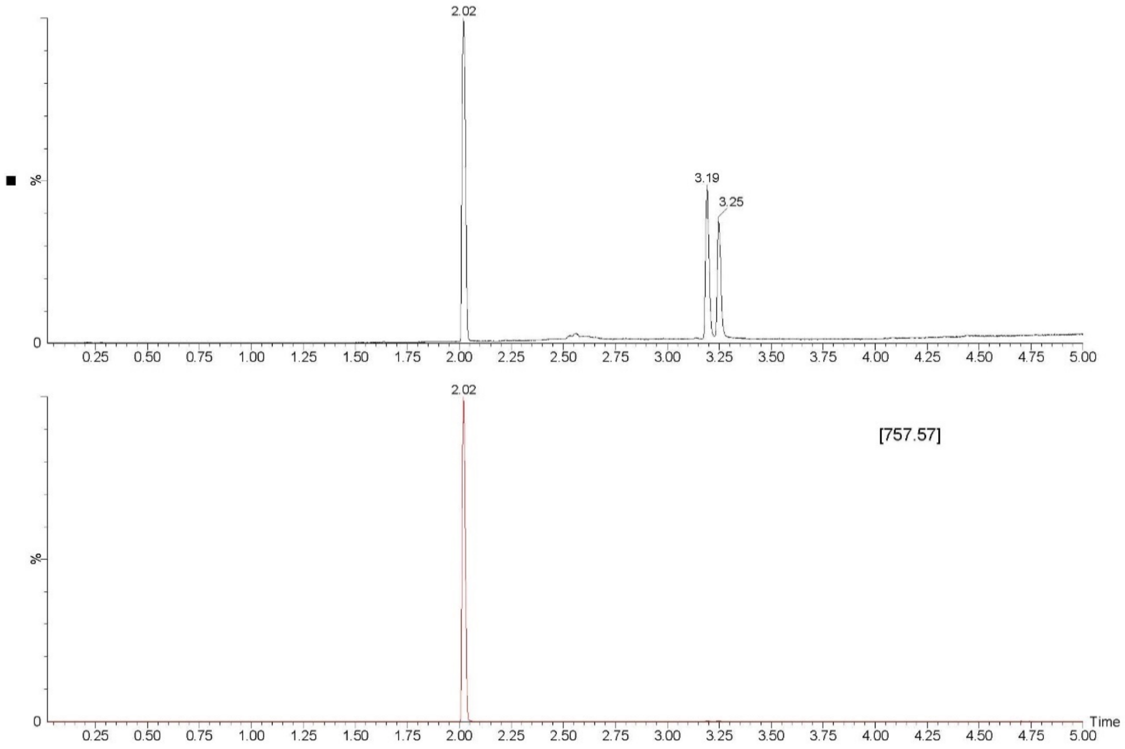

Dodecane-1,12-diyl dipentyl bis((2-(bis(3-aminopropyl)amino)ethyl)phosphonate) hexahydro-chloride (76)

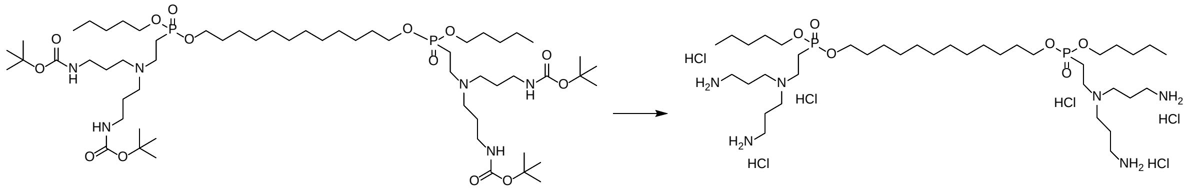

The title compound was prepared according to general method **E** from Boc derivative **S123** (1.10 g, 0.93 mmol) in 90% yield (0.78 g, 0.84 mmol) as a white solid.

^{1}H NMR (400 MHz, CD_3OD): 4.19–4.08 (m, 8H, C*H*$_2$O), 3.49–3.42 (m, 4H, PCH_2C*H*$_2$), 3.42–3.34 (m, 8H, C*H*$_2(CH_2)_2NH_2$), 3.10 (t, 8H, *J* = 7.5 Hz, C*H*$_2NH_2$), 2.61–2.45 (m, 4H, PC*H*$_2$), 2.28–2.13 (m, 8H, C*H*$_2CH_2NH_2$), 1.79–1.66 (m, 8H, C*H*$_2CH_2O$), 1.48–1.26 (m, 24H, CH_3(C*H*$_2$)$_2$, (C*H*$_2$)$_4(CH_2)_2O$), 0.99–0.89 (m, 6H, C*H*$_3$).

^{13}C NMR (101 MHz, CD_3OD): 68.26 (d, *J* = 6.8 Hz), 68.20 (d, *J* = 6.8 Hz, *C*H_2O), 51.05 (*C*$H_2(CH_2)_2NH_2$), 48.86 (PCH_2*C*H_2), 37.87 (*C*H_2NH_2), 31.60 (d, *J* = 5.8 Hz), 31.29 (d, *J* = 5.9 Hz, *C*H_2CH_2O), 30.71, 30.68, 30.30, 28.79, 26.62, 23.29 (CH_3(*C*H_2)$_2$, (*C*H_2)$_4(CH_2)_2O$), 23.24 (*C*$H_2CH_2NH_2$), 21.27 (d, *J* = 140.3 Hz, P*C*H_2), 14.35 (*C*H_3).

^{31}P{^{1}H} NMR (162 MHz, CD_3OD): 28.38.

IR ν_{max} (KBr) 2930 (vs), 2631 (m, sh), 2557 (m, sh), 1599 (m), 1467 (m), 1226 (s), 1170 (m, sh), 1065 (m, sh), 1052 (s, sh), 995 (s).

HR-MS(ESI^{+}): For $C_{38}H_{88}N_6O_6P_2$ $(M+2H)^{2+}$ *m*/2*z* calculated 393.31148, found 393.31141.

^{1}H NMR spectrum of compound **76**

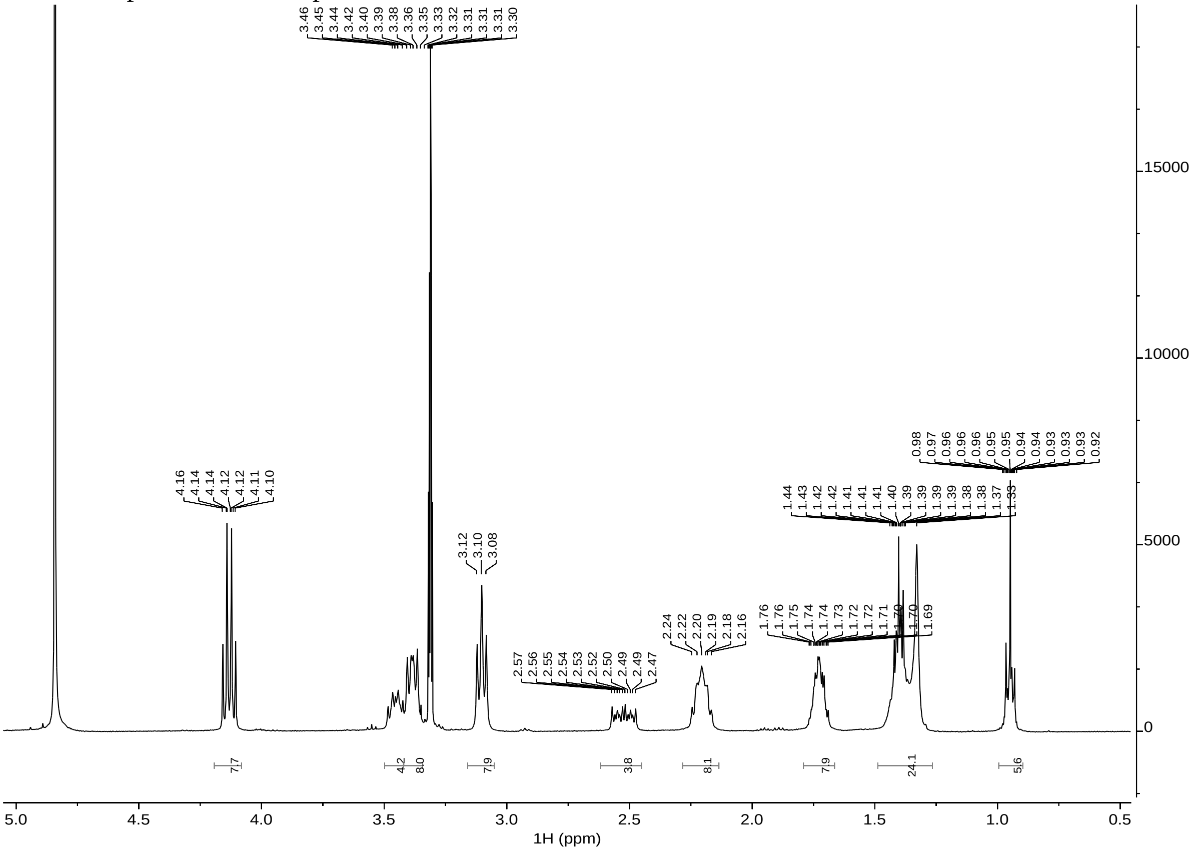

^{13}C NMR spectrum of compound **76**

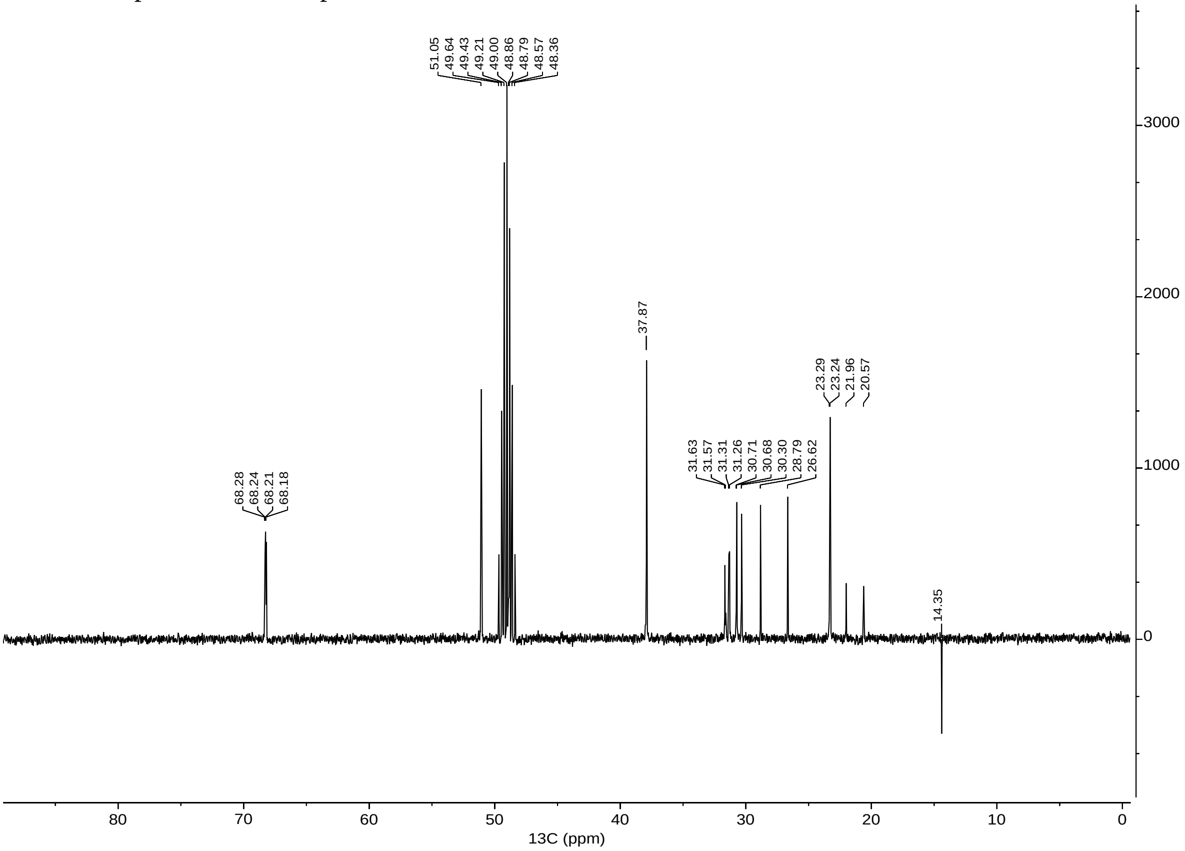

^{31}P{^{1}H} NMR spectrum of compound **76**

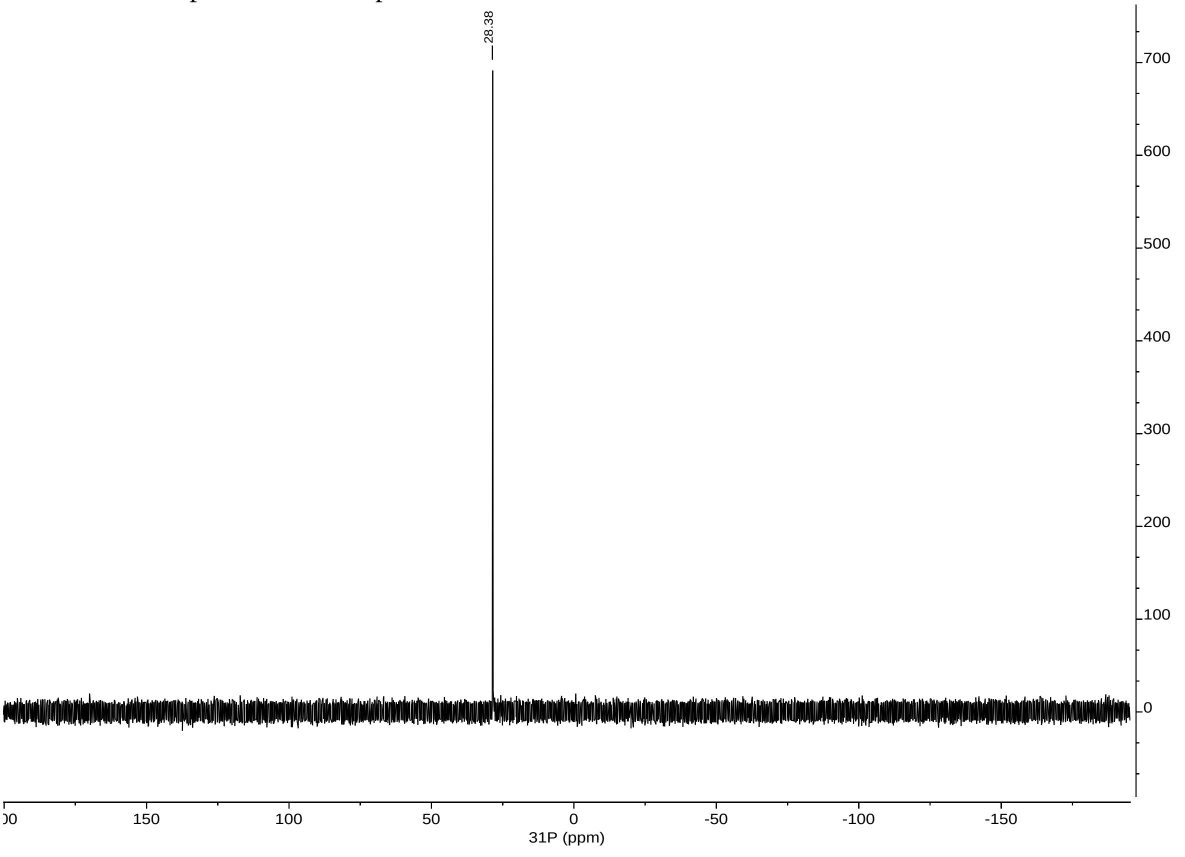

LC-MS chromatograms for compound **76**

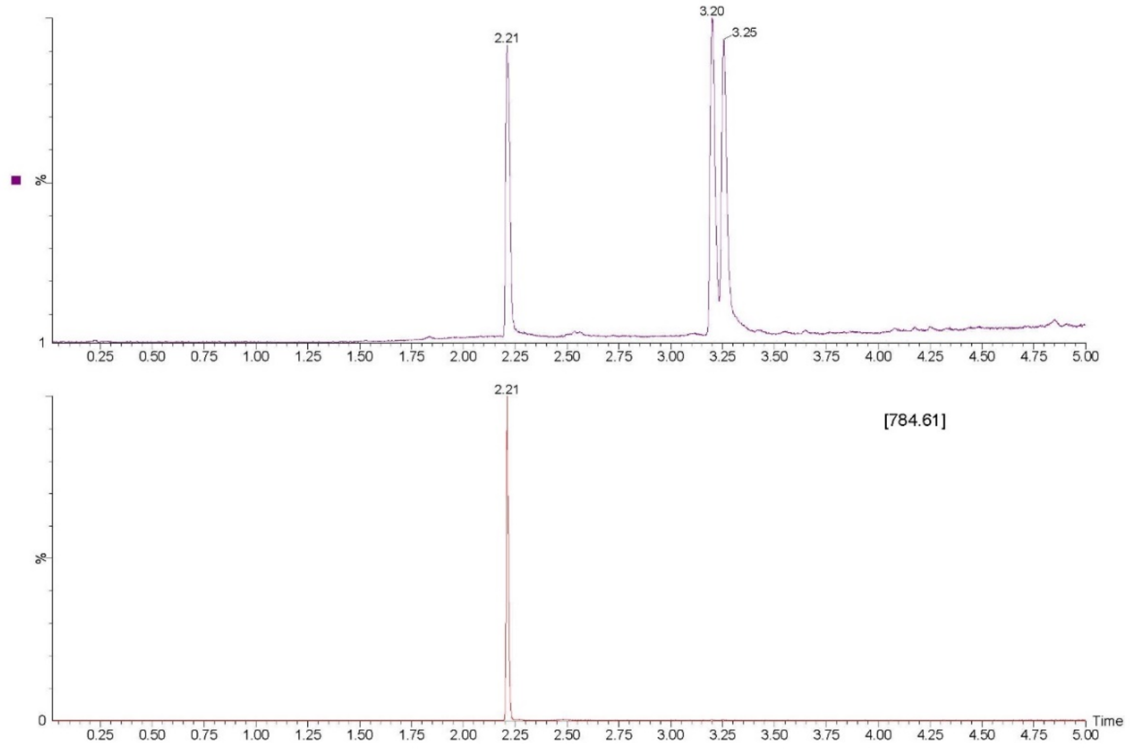

Dodecane-1,12-diyl dihexyl bis((2-(bis(3-aminopropyl)amino)ethyl)phosphonate) hexahydrochloride (77)

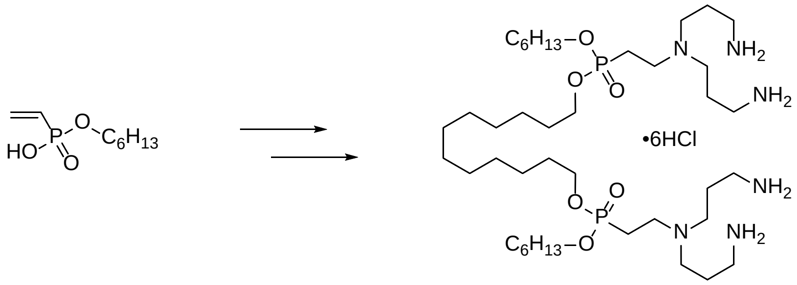

The title compound was prepared according to general methods **C**, **D** and **E** from mono hexyl vinylphosphonate **S23** (2.79 g, 14.5 mmol) and 1,12-dibromododecane (1.59 g, 4.84 mmol) in 16% overall yield (0.80 g, 0.78 mmol) as a white solid.

^{1}H NMR (401 MHz, CD_3OD) δ 4.13 (dt, *J* = 7.8, 6.6 Hz, 8H, OC*H*$_2$), 3.52–3.35 (m, 12H, NC*H*$_2$), 3.12 (t, *J* = 7.5 Hz, 8H, C*H*$_2$NH$_2$), 2.65–2.50 (m, 4H, PC*H*$_2$), 2.23 (tt, *J* = 9.0, 6.1 Hz, 8H, C*H*$_2$CH$_2$NH$_2$), 1.71 (dt, *J* = 8.4, 6.5 Hz, 8H, OCH$_2$C*H*$_2$), 1.47–1.25 (m, 28H, O(CH$_2$)$_2$(C*H*$_2$)$_8$(CH$_2$)$_2$O, O(CH$_2$)$_2$(C*H*$_2$)$_3$CH$_3$), 0.99–0.86 (m, 6H, C*H*$_3$).

^{13}C NMR (101 MHz, CD_3OD) δ 68.18 (d, *J* = 6.8 Hz, O*C*H$_2$), 50.95 (*C*H$_2$(CH$_2$)$_2$NH$_2$), 48.86 (d, *J* = 2.1 Hz, PCH$_2$*C*H$_2$), 37.86 (*C*H$_2$NH$_2$), 32.48, 31.57 (d, *J* = 4.4 Hz, OCH$_2$*C*H$_2$), 31.52 (d, *J* = 4.5 Hz, OCH$_2$*C*H$_2$), 30.69, 30.66, 30.28, 26.60 (O(CH$_2$)$_2$*C*H$_2$), 26.28 (O(CH$_2$)$_2$*C*H$_2$), 23.61, 23.13 (*C*H$_2$CH$_2$NH$_2$), 21.27 (d, *J* = 140.1 Hz, P*C*H$_2$), 14.40 (*C*H$_3$).

^{31}P{^{1}H} NMR (162 MHz, CD_3OD) δ 28.64.

IR ν_{max} (KBr) 3100-2500 (m-s, vbr), 2960 (m, br, sh), 2923 (m, br), 2855 (m), 2674 (m), 2617 (m), 2545 (m, br), 2040 (m, vbr), 1604 (s), 1542 (m), 1508 (m), 1483 (s), 1468 (s), 1459 (s), 1401 (m), 1379 (m, sh), 1226 (vs), 1074 (s), 1040 (s, sh), 1025 (vs, sh), 995 (vs), 964 (s, sh), 724 (w).

HR-MS(ESI$^+$): For $C_{40}H_{91}N_6O_6P_2$ (M+H)$^+$ *m*/*z* calculated 813.64698, found 813.64686.

^{1}H NMR spectrum of compound **77**

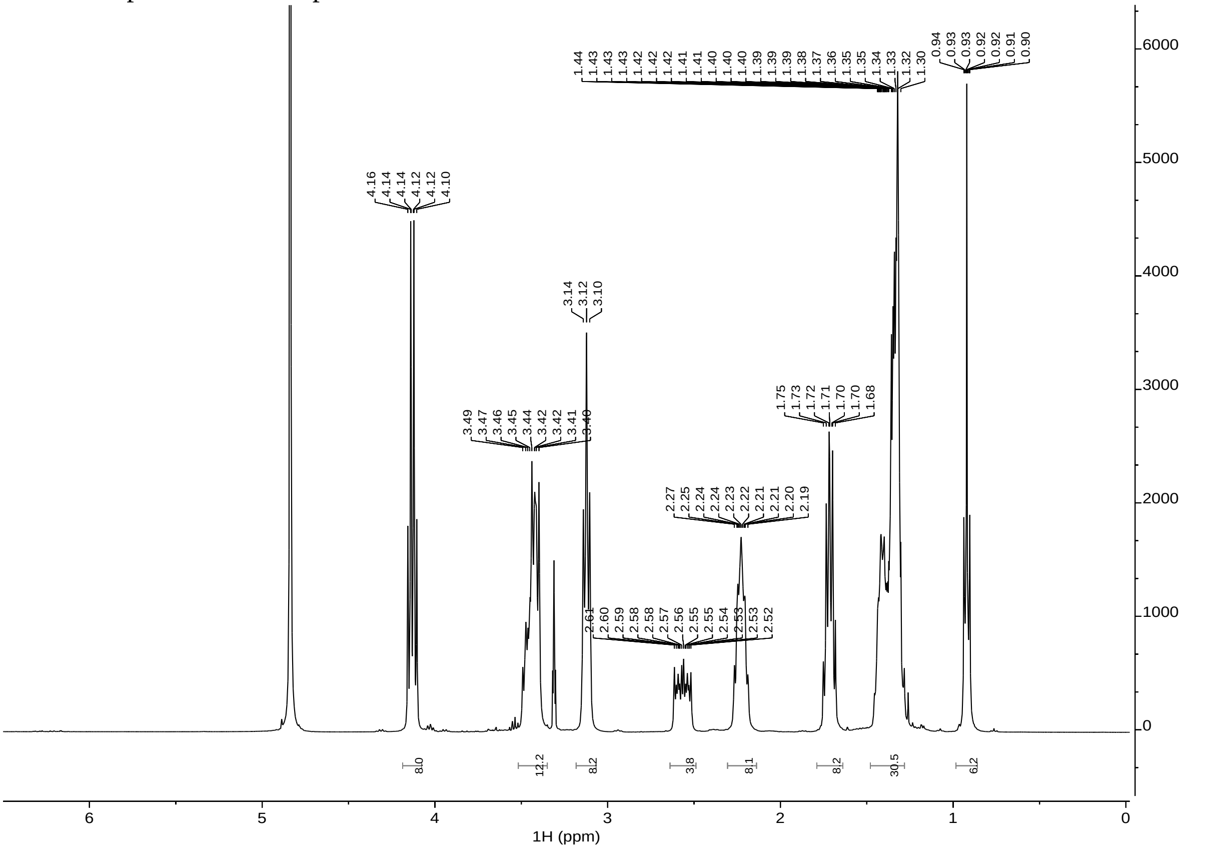

^{13}C NMR spectrum of compound **77**

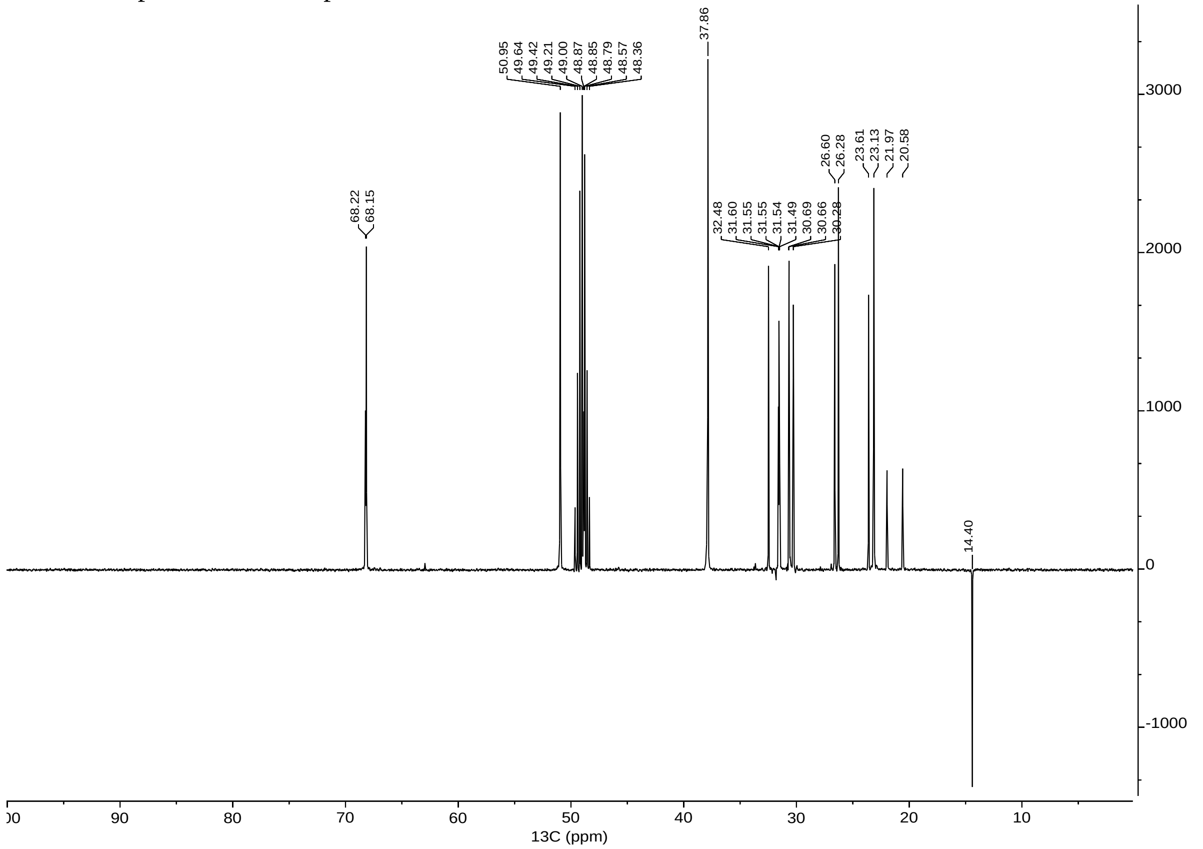

^{31}P{^{1}H} NMR spectrum of compound **77**

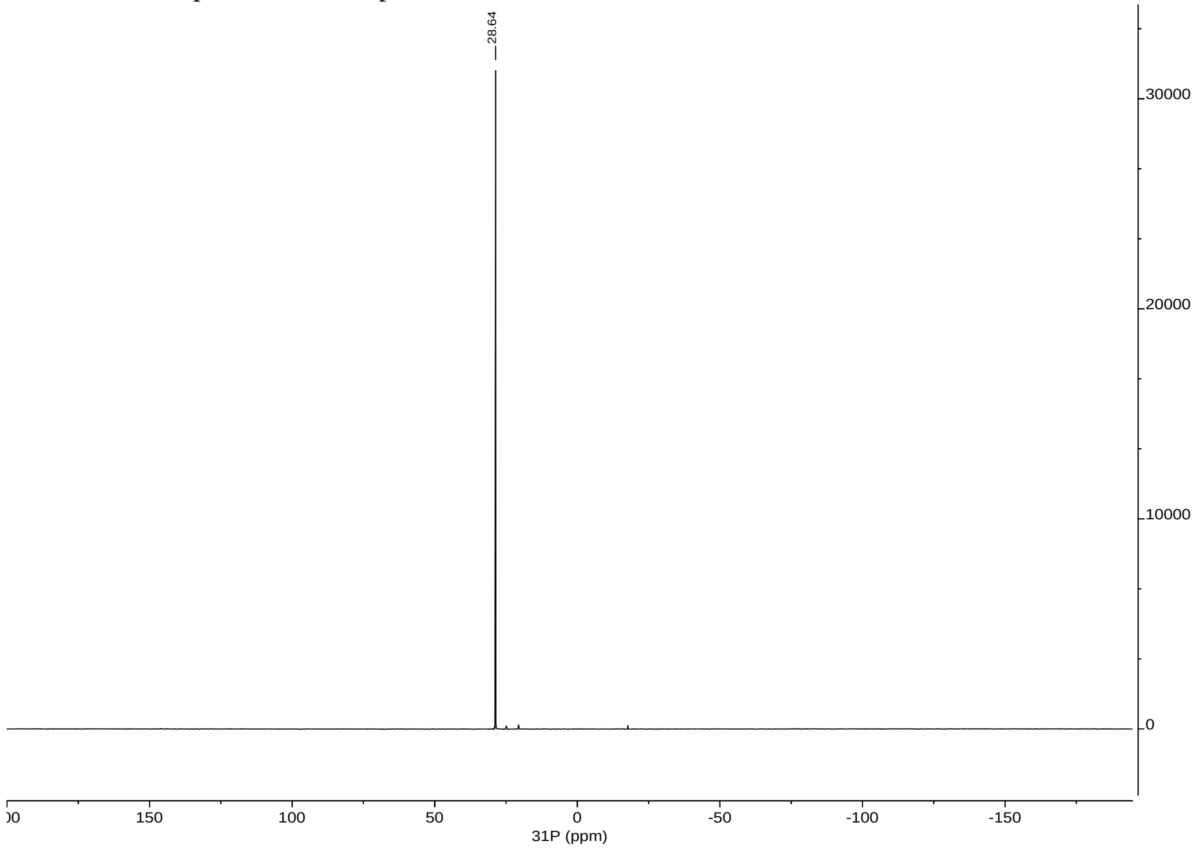

LC-MS chromatograms for compound **77**

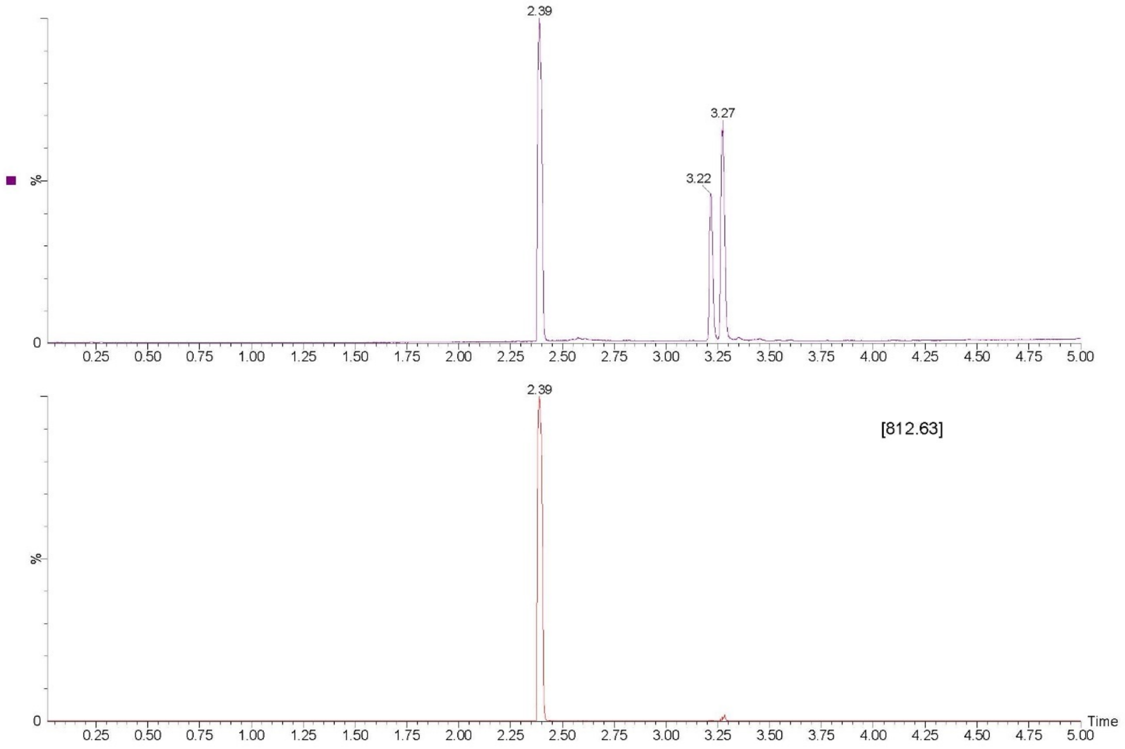

Dodecane-1,12-diyl dioctyl bis((2-(bis(3-aminopropyl)amino)ethyl)phosphonate) hexahydrochloride (78)

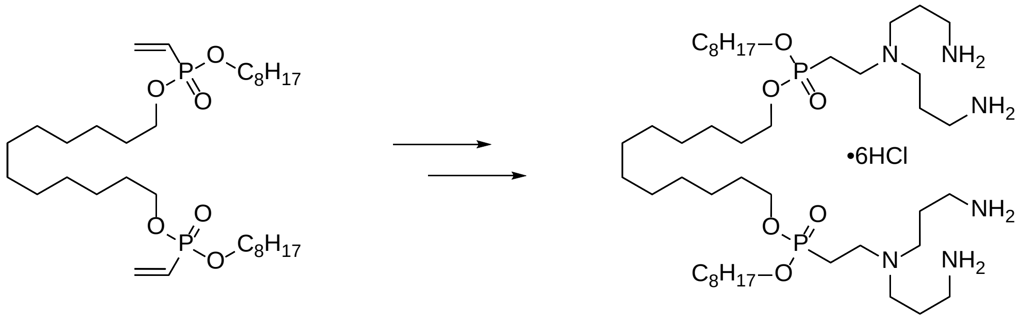

The title compound was prepared according to general methods **D** and **E** from vinylphosphonate dimer **S85** (0.54 g, 0.89 mmol) in 69% overall yield (0.67 g, 0.614 mmol) as a white solid.

^{1}H NMR (500.2 MHz, CD_3OD): 4.09–4.16 (m, 8H, CH_2O), 3.41–3.48 (m, 4H, PCH_2CH_2), 3.34–3.41 m, 8H, CH_2(CH_2)$_2$$NH_2$), 3.10 (t, 8H, J_{vic} = 7.5, C$H_2$$NH_2$), 2.46–2.57 (m, 4H, PC$H_2$), 2.15–2.25 (m, 8H, C$H_2$$CH_2NH_2$), 1.68–1.76 (m, 8H, C$H_2$$CH_2$O), 1.26–1.46 (m, 36H, CH_3CH_2, O(CH_2)$_2$(CH_2)$_4$), 0.88–0.93 (m, 6H, CH_3).

^{13}C NMR (125.8 MHz, CD_3OD): 68.26 (d, $J_{C,P}$ = 6.7, CH_2O), 51.08 ($CH_2(CH_2)_2NH_2$), 48.80 (PCH_2CH_2), 37.88 (CH_2NH_2), 32.98 ($CH_3CH_2CH_2$), 31.63 (d, $J_{C,P}$ = 5.5), 31.61 (d, $J_{C,P}$ = 5.7, CH_2CH_2O), 30.77 ($CH_3(CH_2)_5CH_2CH_2O$, O(CH_2)$_2$(CH_2)$_4$), 30.74, 30.36, 30.35, 30.26, 26.64, 26.64, 23.71 (CH_3CH_2), 23.29 ($CH_2CH_2NH_2$), 21.24 (d, $J_{C,P}$ = 140.4, PCH_2), 14.45 (CH_3).

^{31}P{^{1}H} NMR (202.5 MHz, CD_3OD): 26.67.

IR ν_{max} (KBr) 2956 (vs), 2925 (vs), 2854 (vs), 2675 (m), 2643 (m), 2619 (m), 2547 (m), 2059 (w, br), 1623 (m, sh), 1608 (m), 1543 (m), 1510 (m), 1484 (m), 1468 (m), 1460 (m), 1401 (w), 1390 (m), 1378 (w, sh), 1262 (m), 1227 (s), 1075 (m), 1022 (s), 995 (s), 722 (w).

HR-MS(ESI$^+$) For $C_{44}H_{100}N_6O_6P_2$ (M+2H)$^{2+}$ $m/2z$ calculated 435.35843, found 435.35823.

^{1}H NMR spectrum of compound **78**

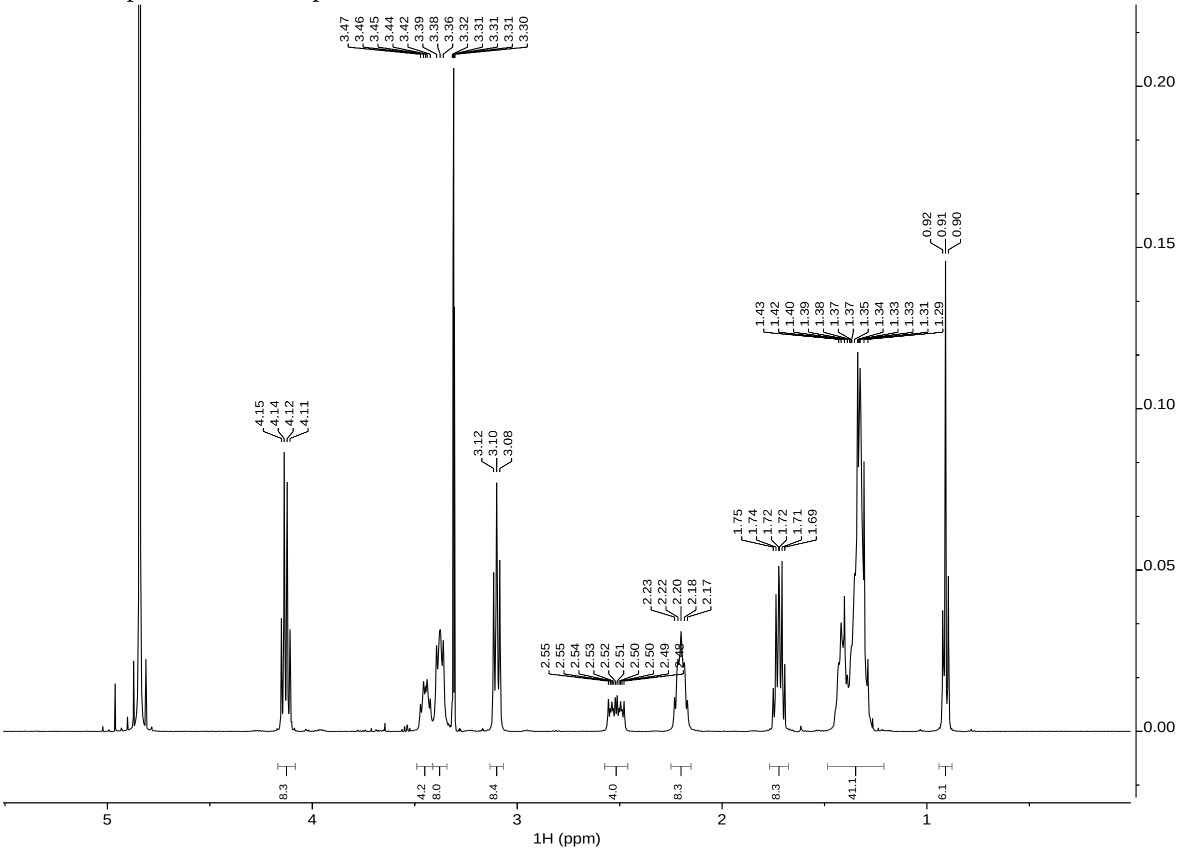

^{13}C NMR spectrum of compound **78**

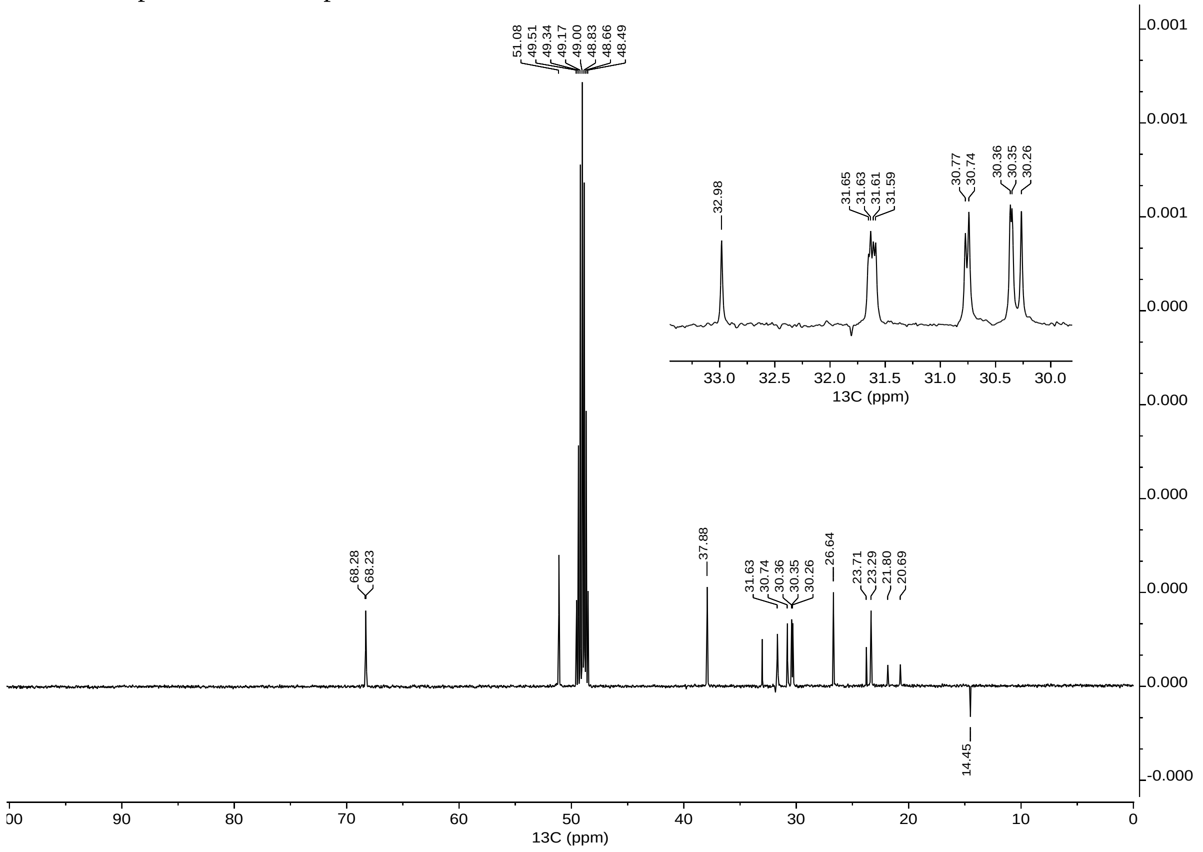

^{31}P{^{1}H} NMR spectrum of compound **78**

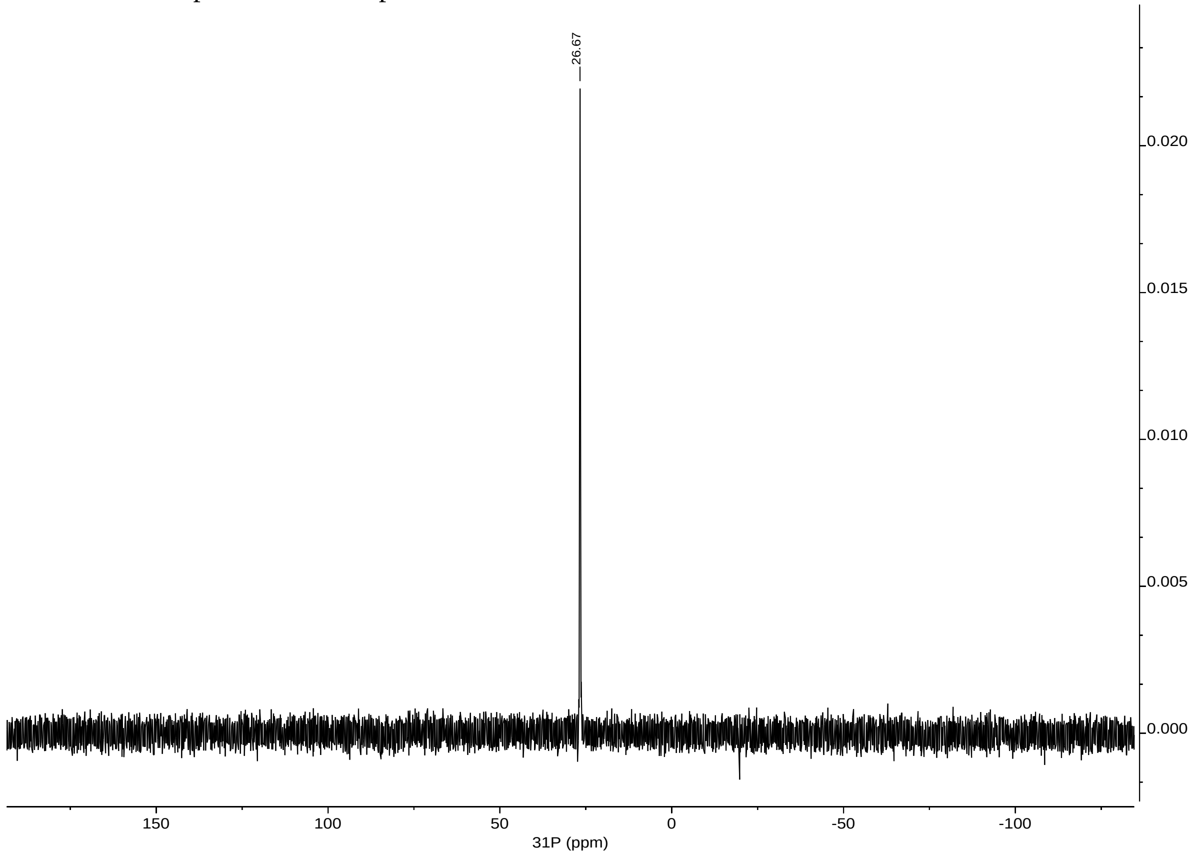

LC-MS chromatograms for compound **78**

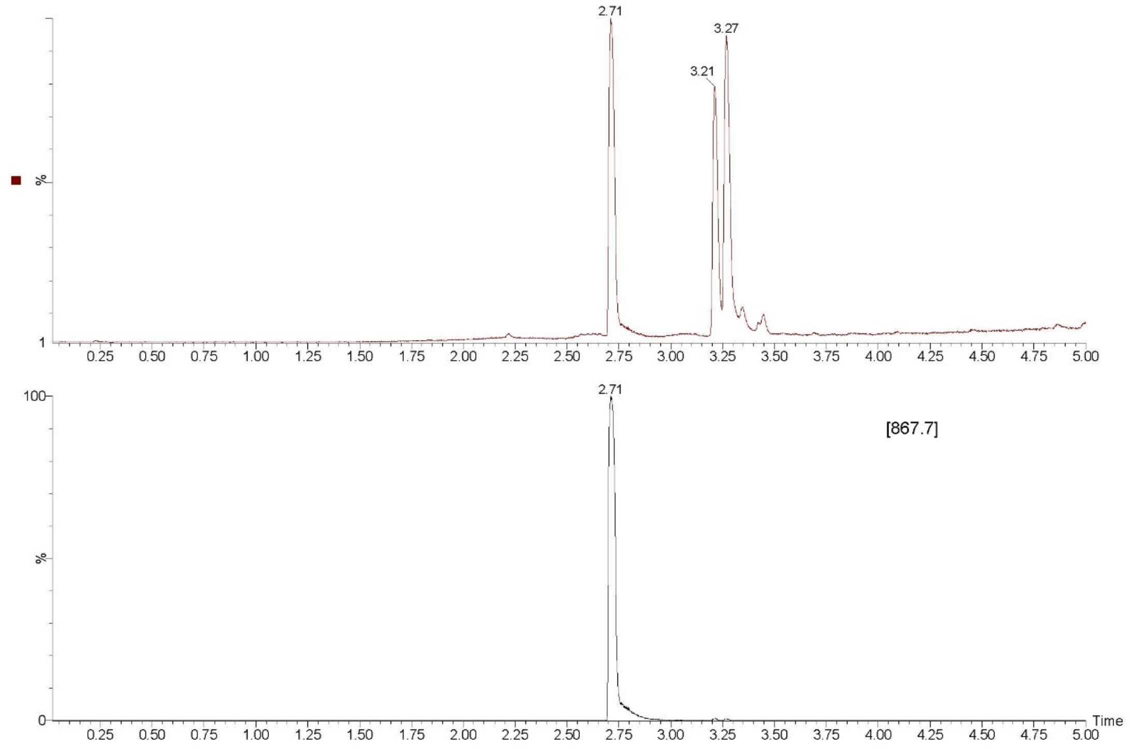

Dodecane-1,12-diyl diphenethyl bis((2-(bis(3-aminopropyl)amino)ethyl)phosphonate) hexahydrochloride (79)

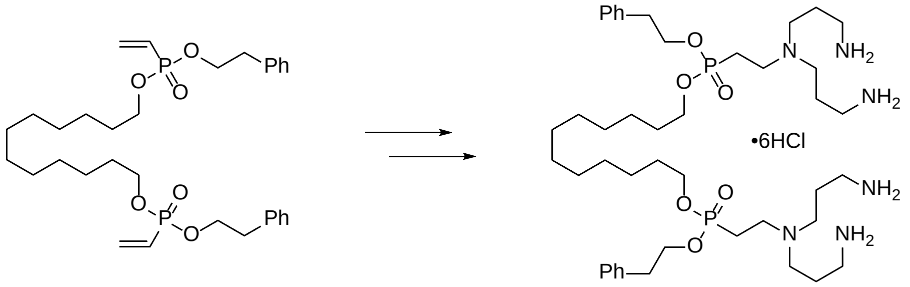

The title compound was prepared according to general methods **D** and **E** from vinylphosphonate dimer **S86** (0.81 g, 1.37 mmol) in 53% overall yield (0.78 g, 0.407 mmol) as a white solid.

^{1}H NMR (500.2 MHz, CD_3OD): 7.37–7.22 (m, 10H, Ph*H*) 4.30–4.40 (m, 4H, OC*H*$_2$CH$_2$Ph), 3.91–4.03 (m, 4H, OC*H*$_2$(CH$_2$)$_5$), 3.30–3.36 (m, 12H, C*H*$_2$(CH$_2$)$_2$NH$_2$, PCH$_2$C*H*$_2$), 3.09 (t, 8H, J_{vic} = 7.5, C*H*$_2$NH$_2$), 3.03 (t, 4H, J_{vic} = 6.6, C*H*$_2$Ph), 2.41–2.51 (m, 4H, PC*H*$_2$) , 2.13–2.22 (m, 8H, C*H*$_2$CH$_2$NH$_2$), 1.59–1.67 (m, 4H, OCH$_2$C*H*$_2$CH$_2$), 1.28–1.37 (m, 16H, O(CH$_2$)$_2$(C*H*$_2$)$_4$).

^{13}C NMR (125.8 MHz, CD_3OD): 138.83 (CH$_2$*C*$_{quat}$), 130.25, 129.68, 127.87 (H*C*$_{Ph}$), 68.58 (d, $J_{C,P}$ = 6.8, O*C*H$_2$CH$_2$Ph), 68.11 (d, $J_{C,P}$ = 6.8, O*C*H$_2$(CH$_2$)$_5$), 51.04 (*C*H$_2$(CH2)$_2$NH$_2$), 48.68 (PCH$_2$*C*H$_2$N), 37.86 (*C*H$_2$NH$_2$), 37.71 (d, $J_{C,P}$ = 6.3, OCH$_2$*C*H$_2$Ph), 31.50 (d, $J_{C,P}$ = 6.0, OCH$_2$*C*H$_2$CH$_2$) , 30.68, 30.64, 30.25, 26.53 (O(CH$_2$)$_2$(*C*H$_2$)$_4$), 23.23 (*C*H$_2$CH$_2$NH$_2$), 21.18 (d, $J_{C,P}$ = 140.5, P*C*H$_2$).

^{31}P{^{1}H} NMR (202.5 MHz, CD_3OD): 26.48.

IR ν_{max} 3087 (m), 3024 (s), 2961–2555 (m, vbr), 2924 (vs), 2853 (s), 2746–2555 (w, vbr), 2009 (w), 1603 (w), 1496 (w), 1466 (m), 1454 (m), 1394 (w), 1226 (m), 1203 (m), 1155 (vw), 1092 (sh, w), 1057–972 (w–m, br), 905 (vw), 750 (w), 699 (m), 550 (vw).

HR-MS(ESI^{+}): For $C_{44}H_{84}O_6N_6P_2$ (M+H)$^{+}$ *m*/2*z* calculated 427.29583, found 427.29547.

^{1}H NMR spectrum of compound **79**

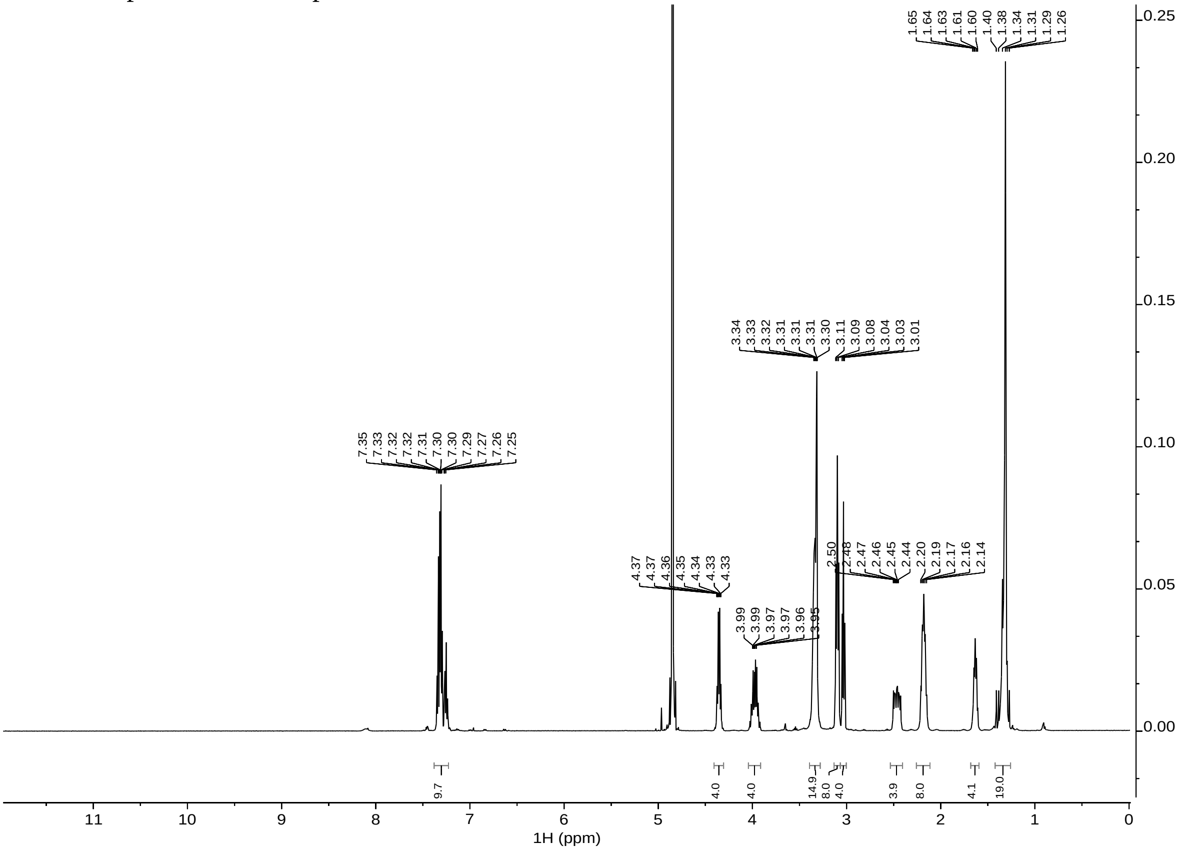

^{13}C NMR spectrum of compound **79**

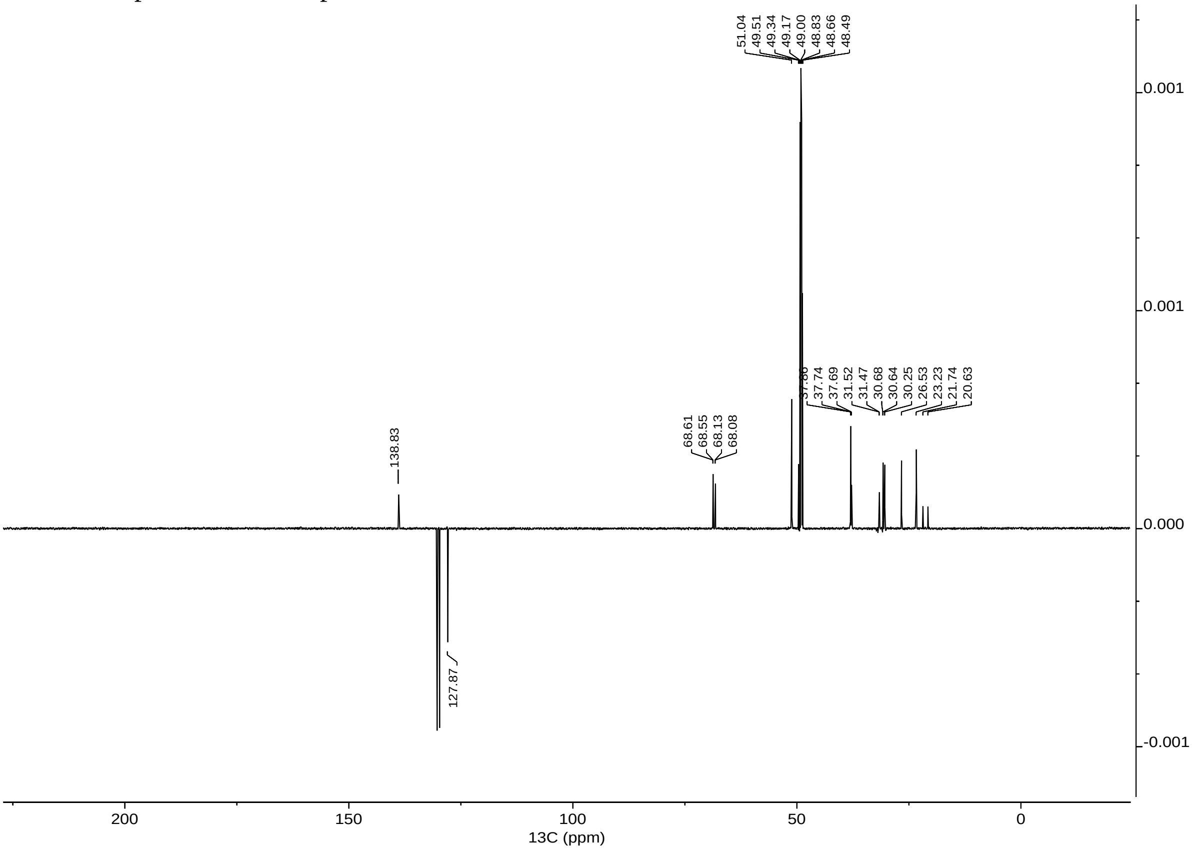

^{31}P{^{1}H} NMR spectrum of compound **79**

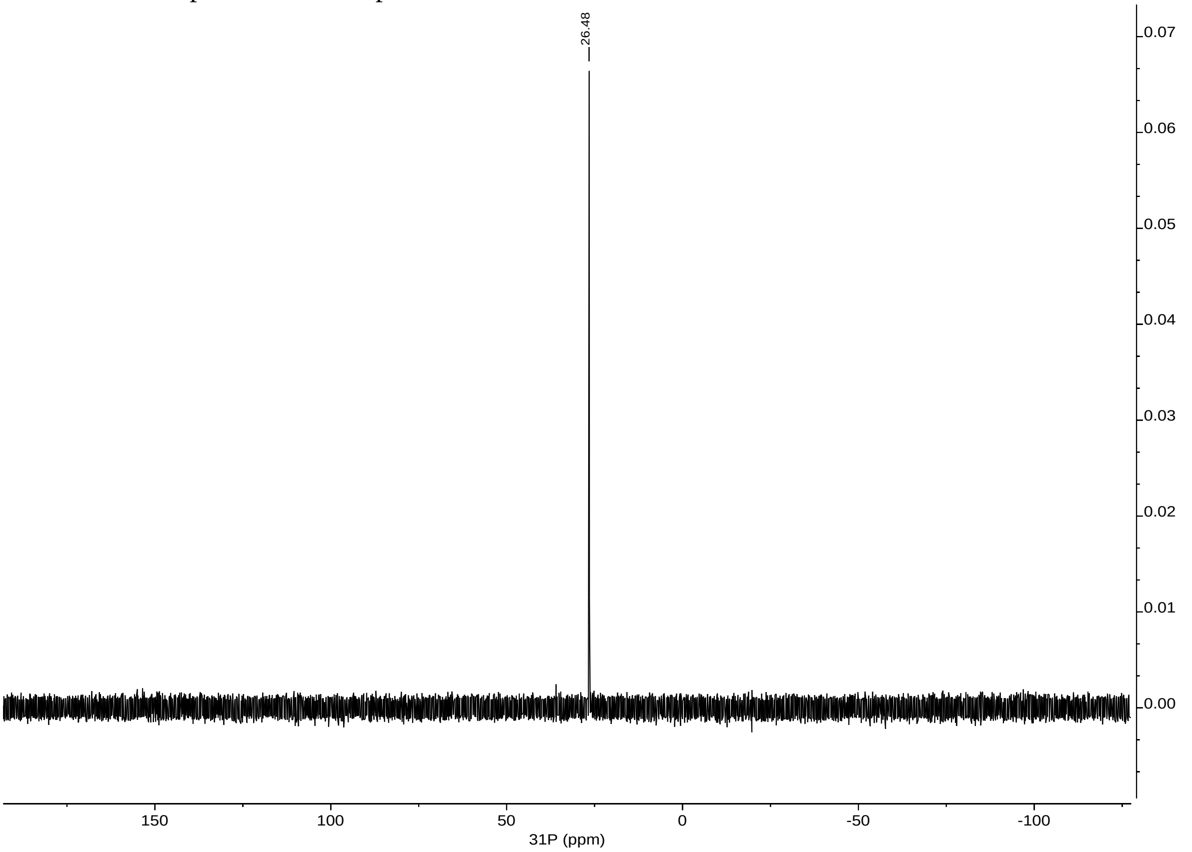

LC-MS chromatograms for compound **79**

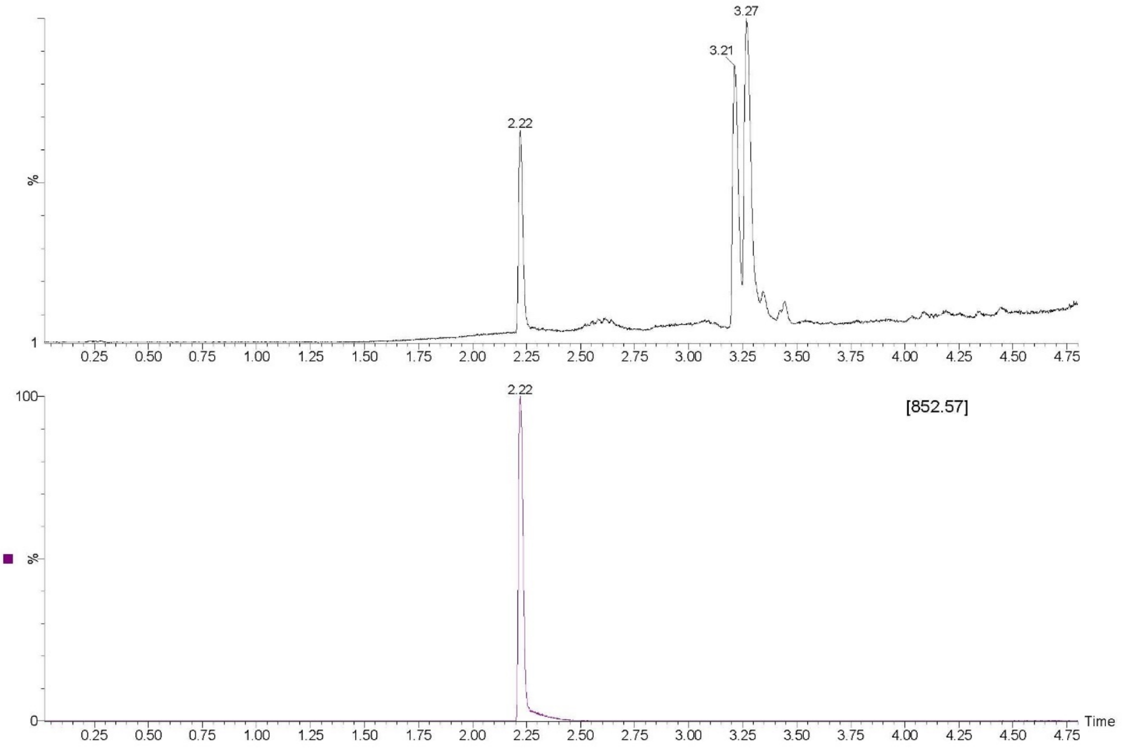

Diisobutyl tetradecane-1,14-diyl bis((2-(bis(3-aminopropyl)amino)ethyl)phosphonate) hexahydrochloride (80)

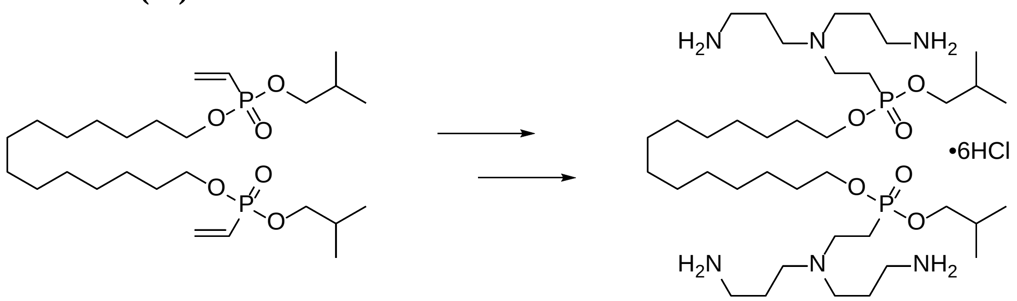

The title compound was prepared according to general methods **D** and **E** from vinylphosphonate dimer **S87** (496 mg, 0.949 mmol) in 43% overall yield (409 mg, 0.407 mmol) as a white solid.

^{1}H NMR (401 MHz, CD_3OD) δ 4.15 (dt, *J* = 7.7, 6.6 Hz, 4H, OC*H*$_2$CH$_2$), 3.92 (td, *J* = 6.6, 2.5 Hz, 4H, OC*H*$_2$CH), 3.52–3.43 (m, 4H, PCH$_2$C*H*$_2$), 3.43–3.36 (m, 8H, C*H*$_2$(CH$_2$)$_2$NH$_2$), 3.11 (t, *J* = 7.5 Hz, 8H, C*H*$_2$NH$_2$), 2.64–2.45 (m, 4H, PC*H*$_2$), 2.22 (h, *J* = 7.7, 7.1 Hz, 8H, C*H*$_2$CH$_2$NH$_2$), 1.99 (dp, *J* = 13.3, 6.6 Hz, 2H, OCH$_2$C*H*), 1.84–1.64 (m, 4H, OCH$_2$C*H*$_2$), 1.53–1.22 (m, 20H, O(CH$_2$)$_2$(C*H*$_2$)$_5$), 1.00 (d, *J* = 6.7 Hz, 12H, C*H*$_3$).

^{13}C NMR (101 MHz, CD_3OD) δ 73.92 (d, *J* = 7.0 Hz, O*C*H$_2$CH), 68.29 (d, *J* = 6.8 Hz, O*C*H$_2$CH$_2$), 51.07 (*C*H$_2$(CH$_2$)$_2$NH$_2$), 37.86 (*C*H$_2$NH$_2$), 31.62 (d, *J* = 5.8 Hz, OCH$_2$*C*H$_2$), 30.81, 30.76, 30.71, 30.46 (d, *J* = 6.3 Hz, OCH$_2$*C*H), 30.31, 26.62 (O(CH$_2$)$_2$(*C*H$_2$)$_5$), 23.30 (*C*H$_2$CH$_2$NH$_2$), 21.18 (d, *J* = 140.6 Hz, P*C*H$_2$), 19.03 (*C*H$_3$).

^{31}P{^{1}H} NMR (162 MHz, CD_3OD) δ 28.60.

IR ν_{max} (KBr) 3200–2700 (vs, vbr), 2960 (s), 2926 (vs), 2854 (s), 2700–2500 (m), 1608 (m), 1511 (m), 1468 (m), 1400 (m), 1369 (w), 1227 (s), 1004 (vs), 852 (m), 768 (w), ~725 (w, sh).

HR-MS(ESI$^+$): For $C_{38}H_{87}O_6N_6P_2$ (M+H)$^+$ *m*/*z* calculated 785.61568, found 785.61523.

^{1}H NMR spectrum of compound **80**

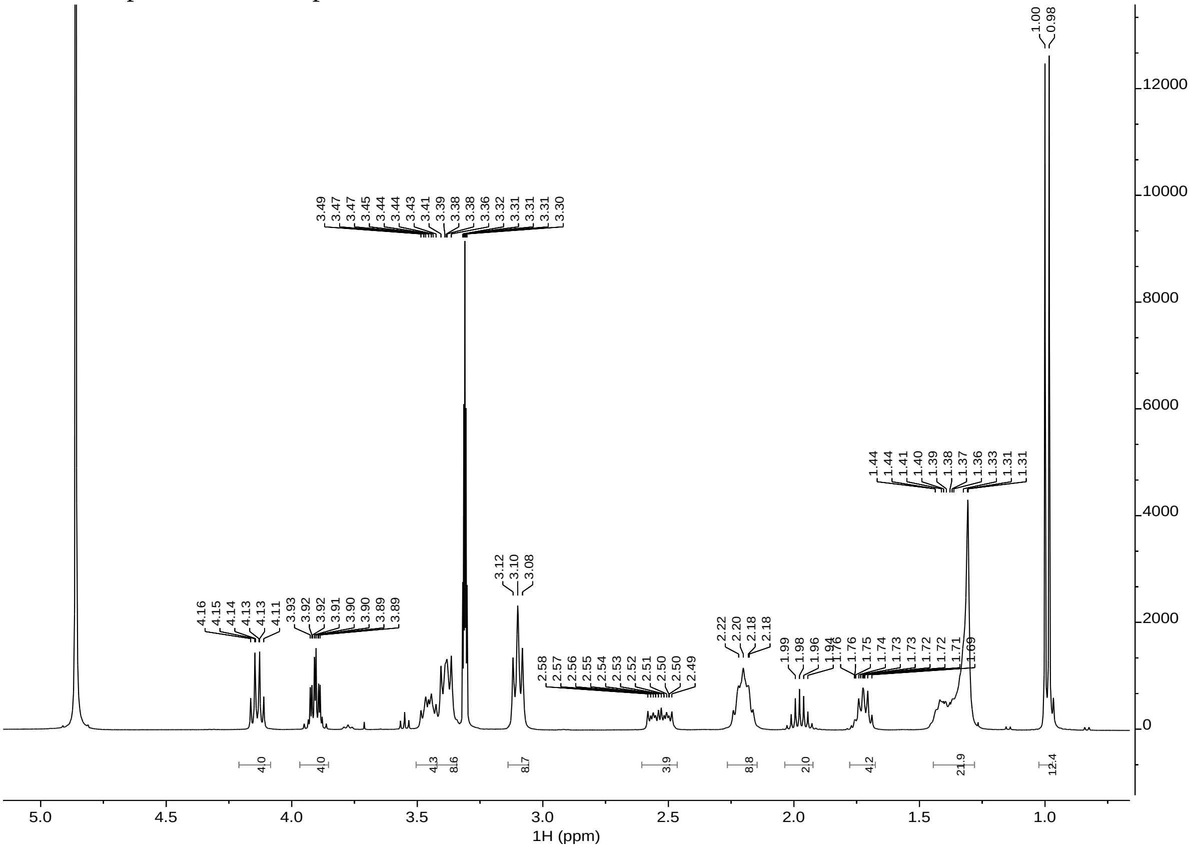

^{13}C NMR spectrum of compound **80**

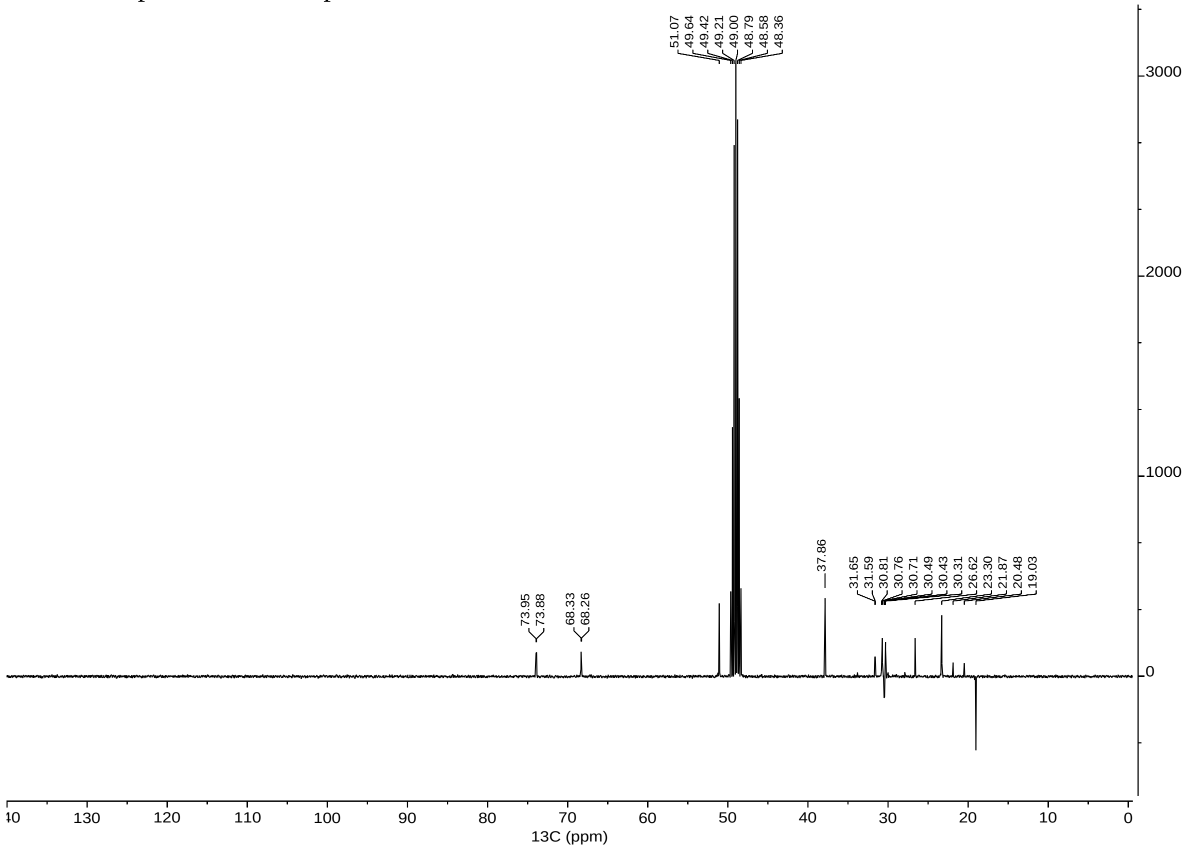

^{31}P{^{1}H} NMR spectrum of compound **80**

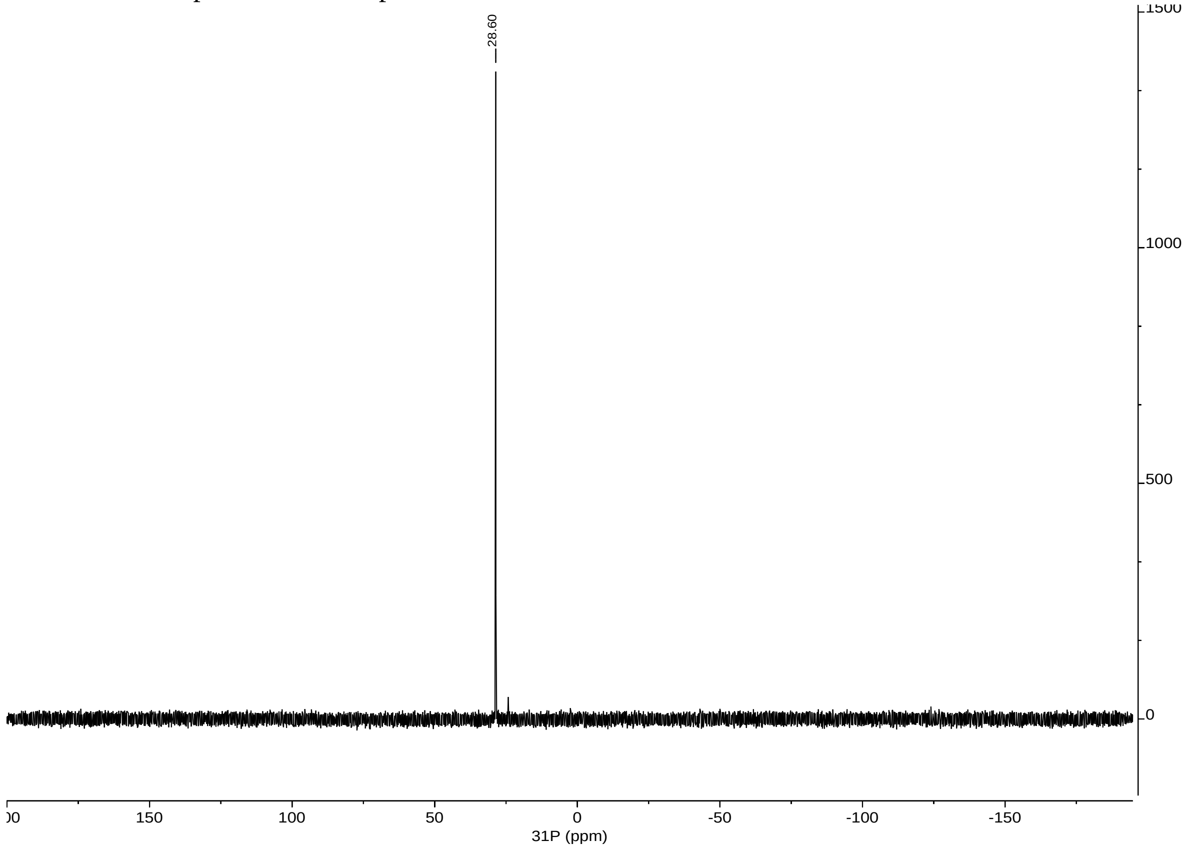

LC-MS chromatograms for compound **80**

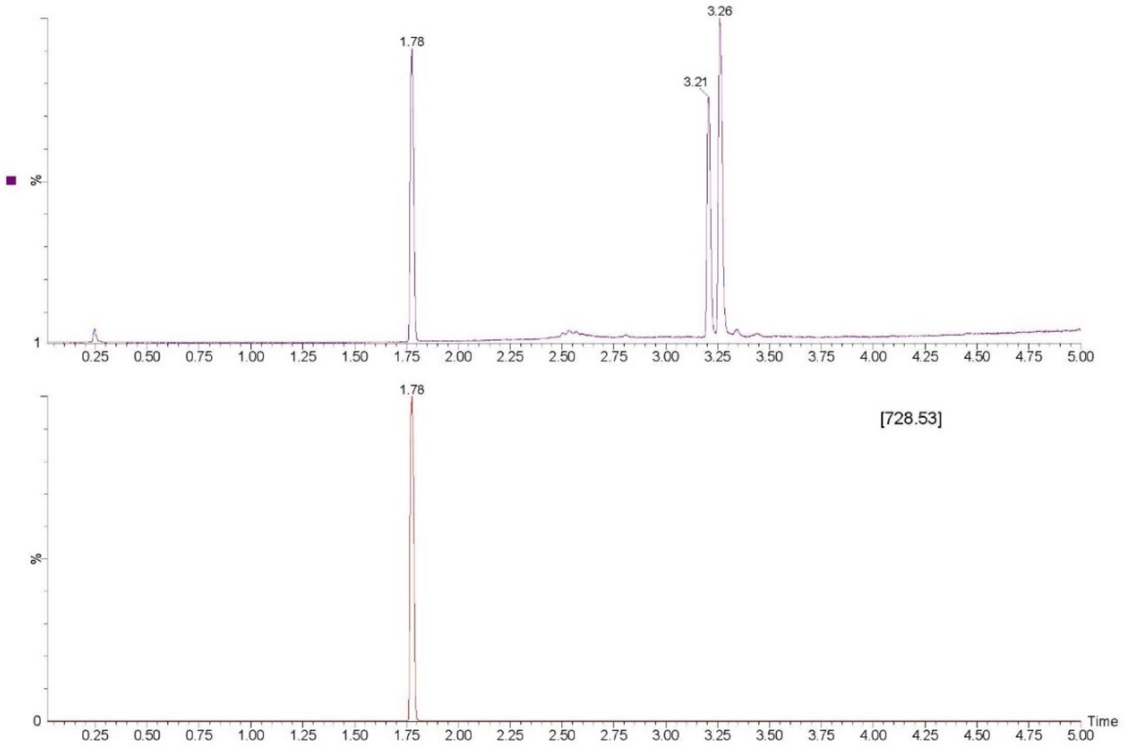

Dibutyl tetradecane-1,14-diyl bis((2-(bis(3-aminopropyl)amino)ethyl)phosphonate) hexahydrochloride (81)

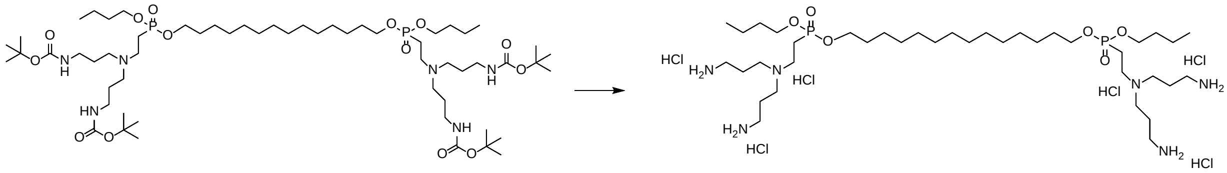

The title compound was prepared according to general method **E** from Boc derivative **S124** (0.65 g, 0.55 mmol) in 91% yield (0.50 g, 0.50 mmol) as a white solid.

^{1}H NMR (400 MHz, CD_3OD): 4.20–4.07 (m, 8H, C*H*$_2$O), 3.53–3.42 (m, 4H, PCH_2CH_2), 3.42–3.34 (m, 8H, C*H*$_2$(CH_2)$_2$$NH_2$), 3.10 (t, 8H, *J* = 7.5 Hz, C*H*$_2$$NH_2$), 2.60–2.45 (m, 4H, PC*H*$_2$), 2.27–2.13 (m, 8H, C*H*$_2$$CH_2NH_2$), 1.77–1.65 (m, 8H, C*H*$_2$$CH_2O$), 1.51–1.27 (m, 24H, CH_3C*H*$_2$, (C*H*$_2$)$_5$(CH_2)$_2$O), 0.98 (t, 6H, *J* = 7.4 Hz, C*H*$_3$).

^{13}C NMR (101 MHz, CD_3OD): 68.27 (d, *J* = 6.7 Hz), 67.92 (d, *J* = 6.8 Hz, *C*H_2O), 51.08 (*C*H_2(CH_2)$_2$$NH_2$), 48.85 ($PCH_2$*C*$H_2$), 37.86 (*C*$H_2NH_2$), 33.61 (d, *J* = 6.0 Hz), 31.59 (d, *J* = 5.9 Hz, *C*H_2CH_2O), 30.79, 30.75, 30.70, 30.30, 26.61 ((*C*H_2)$_5$(CH_2)$_2$O), 23.28 (*C*$H_2CH_2NH_2$), 21.24 (d, *J* = 140.7 Hz, P*C*H_2), 19.80 (CH_3*C*H_2), 13.96 (*C*H_3).

^{31}P{^{1}H} NMR (162 MHz, CD_3OD): 28.40.

IR ν_{max} (KBr) 2966 (m), 2928 (m), 2625 (m), 1604 (m), 1468 (m), 1384 (m), 1230 (m), 1050 (m), 1021 (m).

HR-MS(ESI$^+$): For $C_{38}H_{87}N_6O_6P_2$ $(M+H)^+$ calculated 785.61568, found 785.61506.

^{1}H NMR spectrum of compound **81**

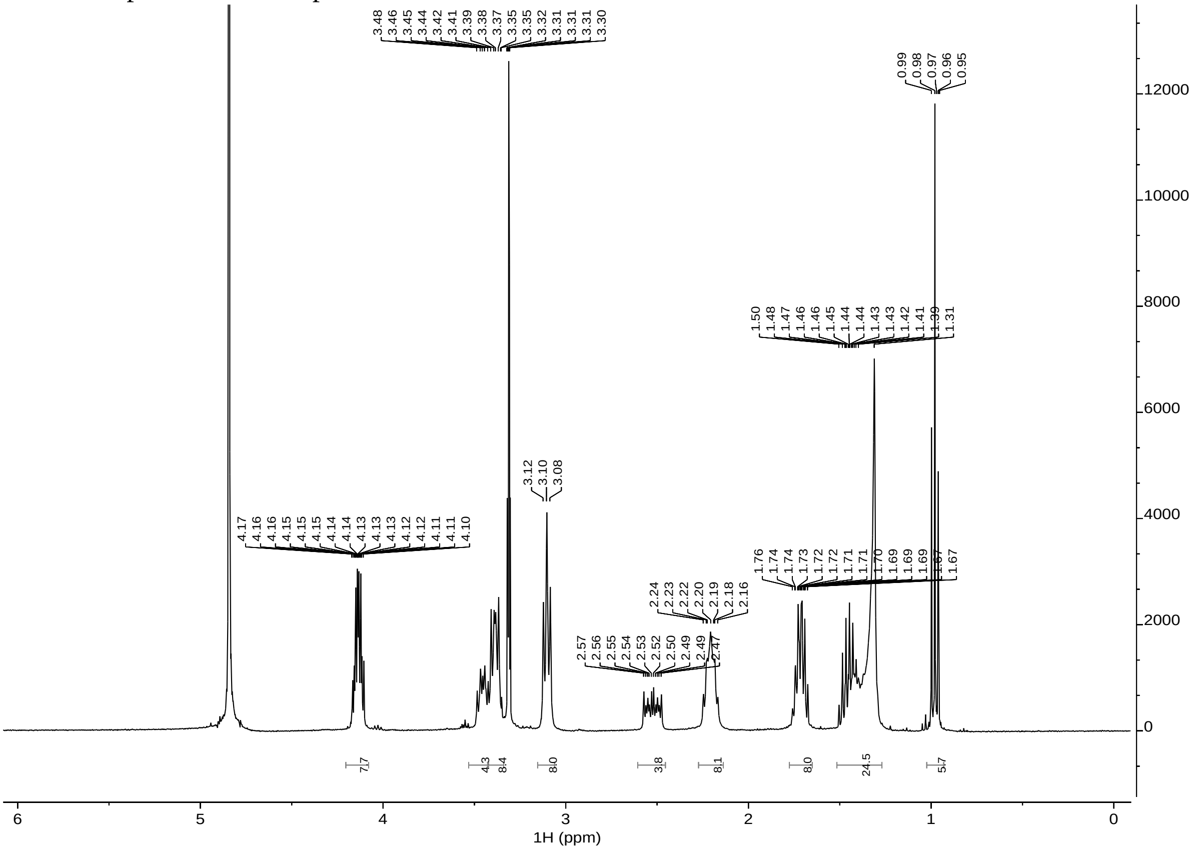

^{13}C NMR spectrum of compound **81**

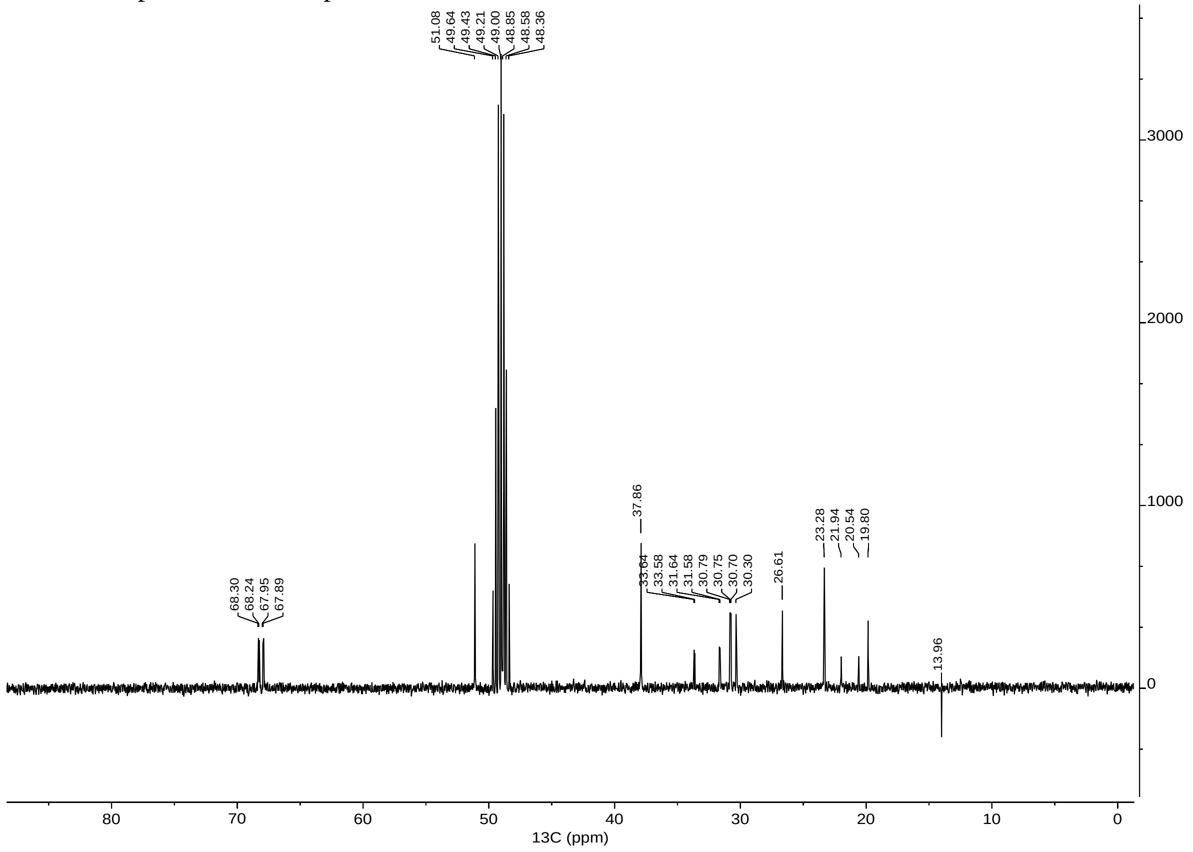

$^{31}P\{^{1}H\}$ NMR spectrum of compound **81**

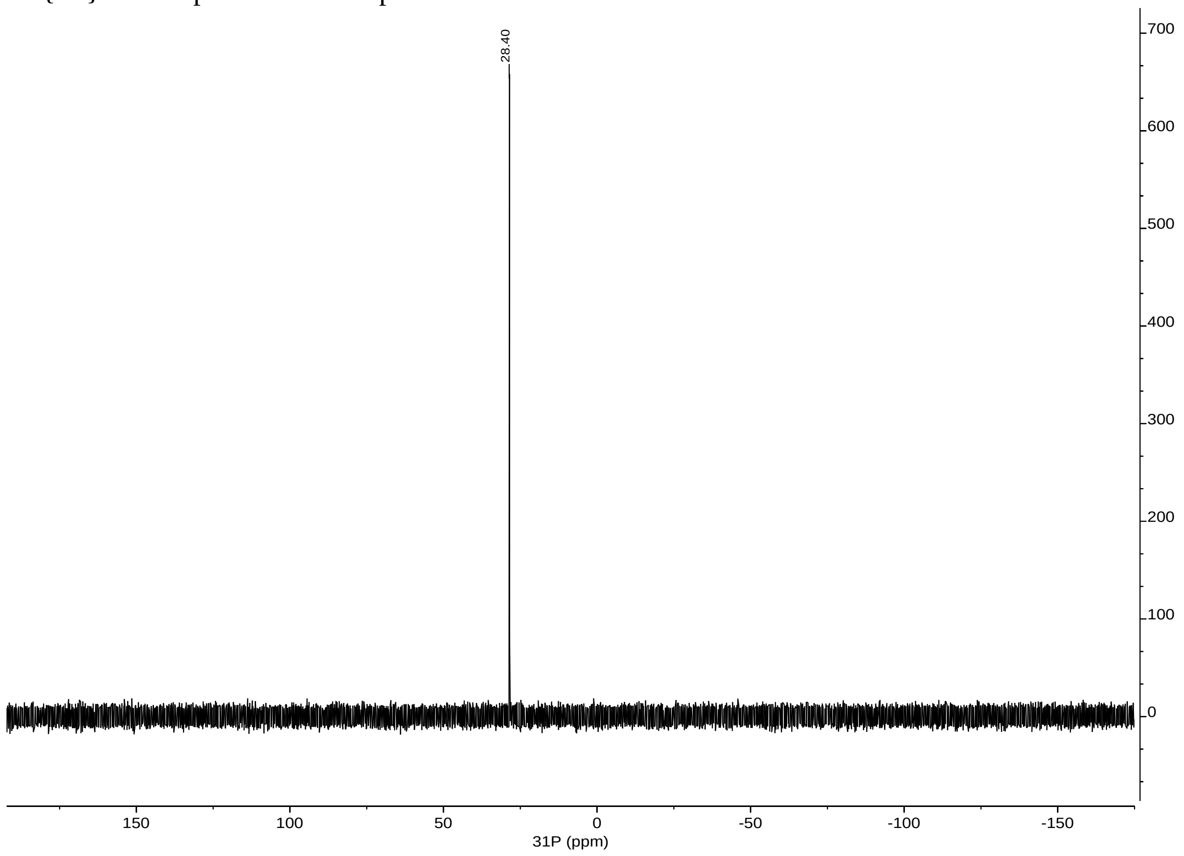

LC-MS chromatograms for compound **81**

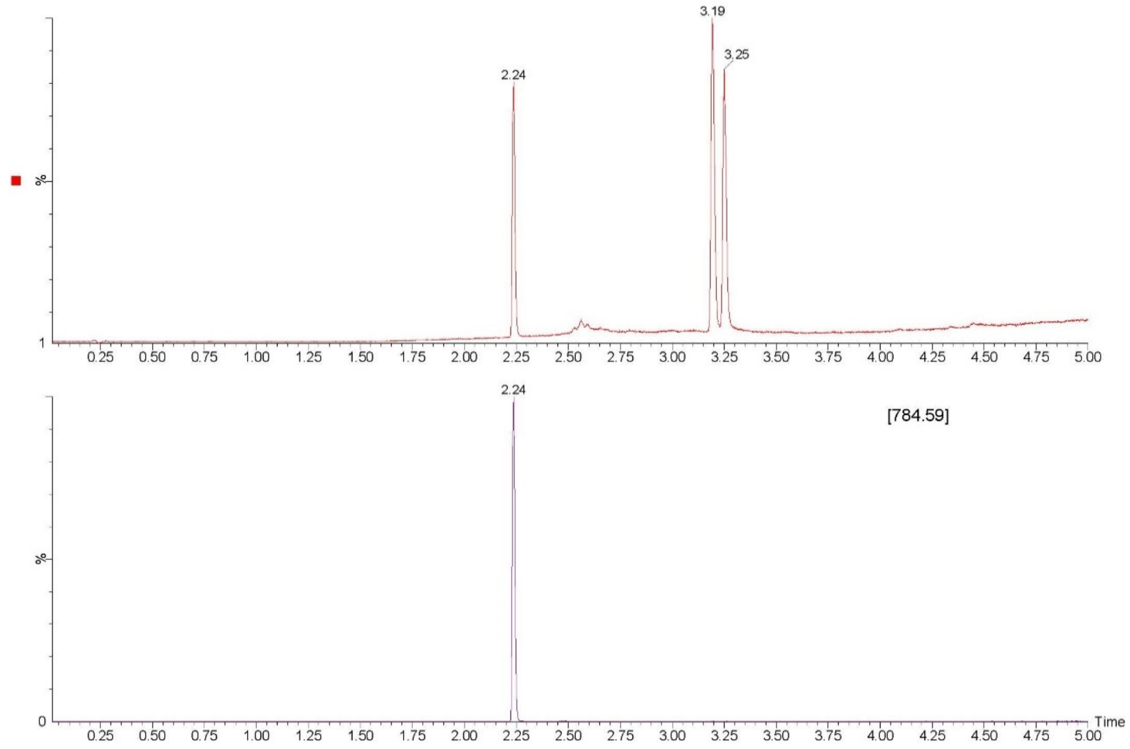

Dipentyl tetradecane-1,14-diyl bis((2-(bis(3-aminopropyl)amino)ethyl)phosphonate) hexahydrochloride (82)

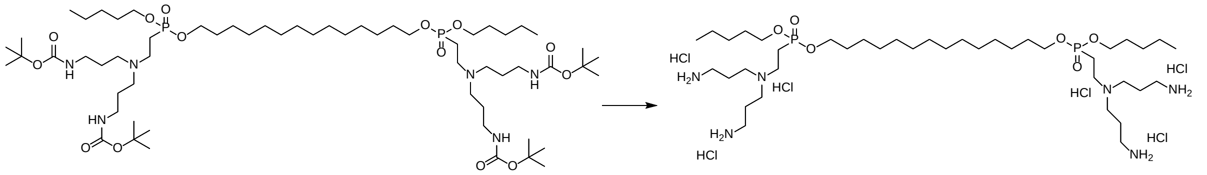

The title compound was prepared according to general method **E** from Boc derivative **S125** (0.85 g, 0.70 mmol) in 93% yield (0.68 g, 0.65 mmol) as a white solid.

^{1}H NMR (400 MHz, CD_3OD): 4.19–4.07 (m, 8H, CH_2O), 3.50–3.42 (m, 4H, PCH_2CH_2), 3.42–3.35 (m, 8H, $CH_2(CH_2)_2NH_2$), 3.10 (t, 8H, J = 7.5 Hz, CH_2NH_2), 2.62–2.45 (m, 4H, PCH_2), 2.28–2.14 (m, 8H, $CH_2CH_2NH_2$), 1.79–1.66 (m, 8H, CH_2CH_2O), 1.48–1.25 (m, 28H, $CH_3(CH_2)_2$, $(CH_2)_5(CH_2)_2O$), 1.00–0.89 (m, 6H, CH_3).

^{13}C NMR (101 MHz, CD_3OD): 68.27 (d, J = 6.8 Hz), 68.21 (d, J = 6.7 Hz, CH_2O), 51.06 ($CH_2(CH_2)_2NH_2$), 48.83 (PCH_2CH_2), 37.87 (CH_2NH_2), 31.61 (d, J = 5.8 Hz), 31.29 (d, J = 5.9 Hz, CH_2CH_2O), 30.79, 30.74, 30.70, 30.31, 28.80, 26.62, 23.30 ($CH_3(CH_2)_2$, $(CH_2)_5(CH_2)_2O$), 23.26 ($CH_2CH_2NH_2$), 21.25 (d, J = 140.5 Hz, PCH_2), 14.34 (CH_3).

^{31}P{^{1}H} NMR (162 MHz, CD_3OD): 28.41

IR ν_{max} (KBr) 2928 (s), 2623 (m), 2553 (m), 1598 (m), 1468 (m), 1230 (m), 1171 (m), 1080 (m, sh), 1047 (m), 995 (vs).

HR-MS(ESI$^+$): For $C_{40}H_{91}N_6O_6P_2$ $(M+H)^+$ m/z calculated 813.64698, found 813.64643.

^{1}H NMR spectrum of compound **82**

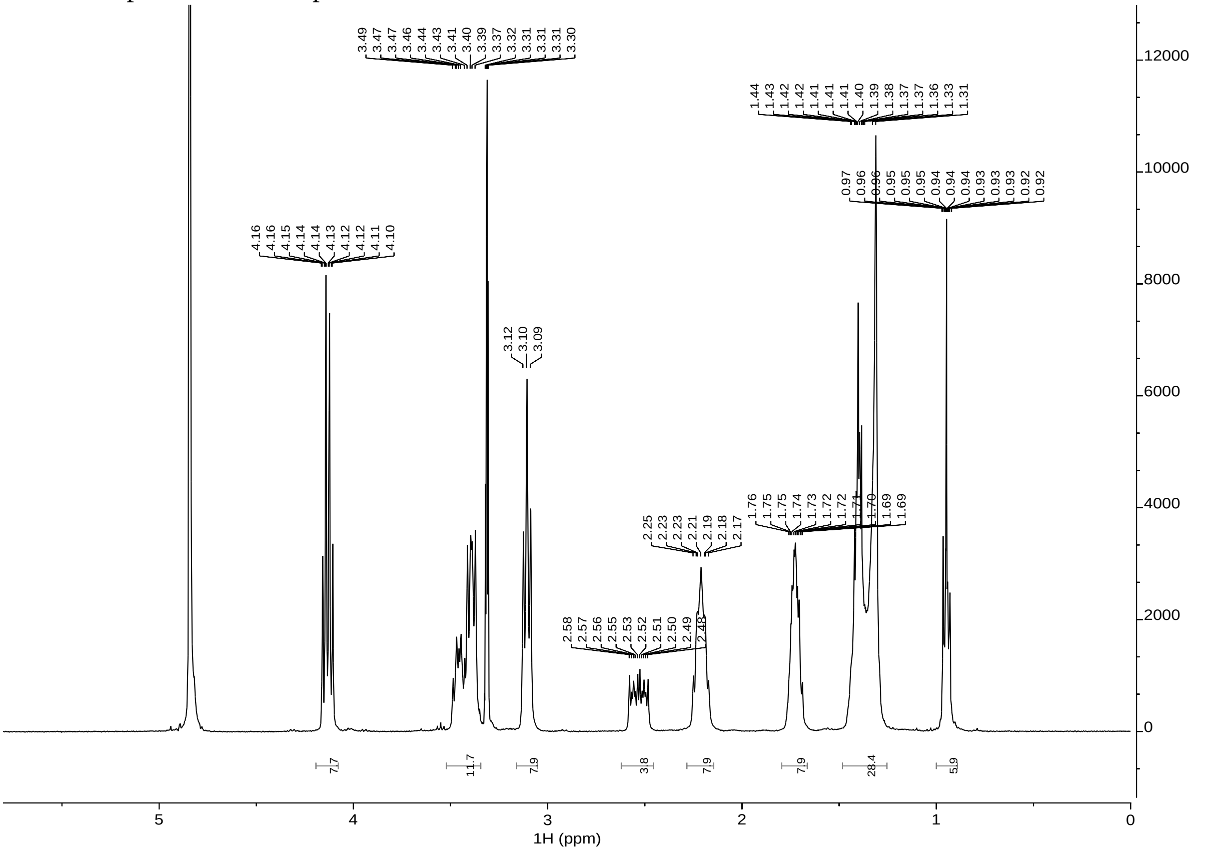

^{13}C NMR spectrum of compound **82**

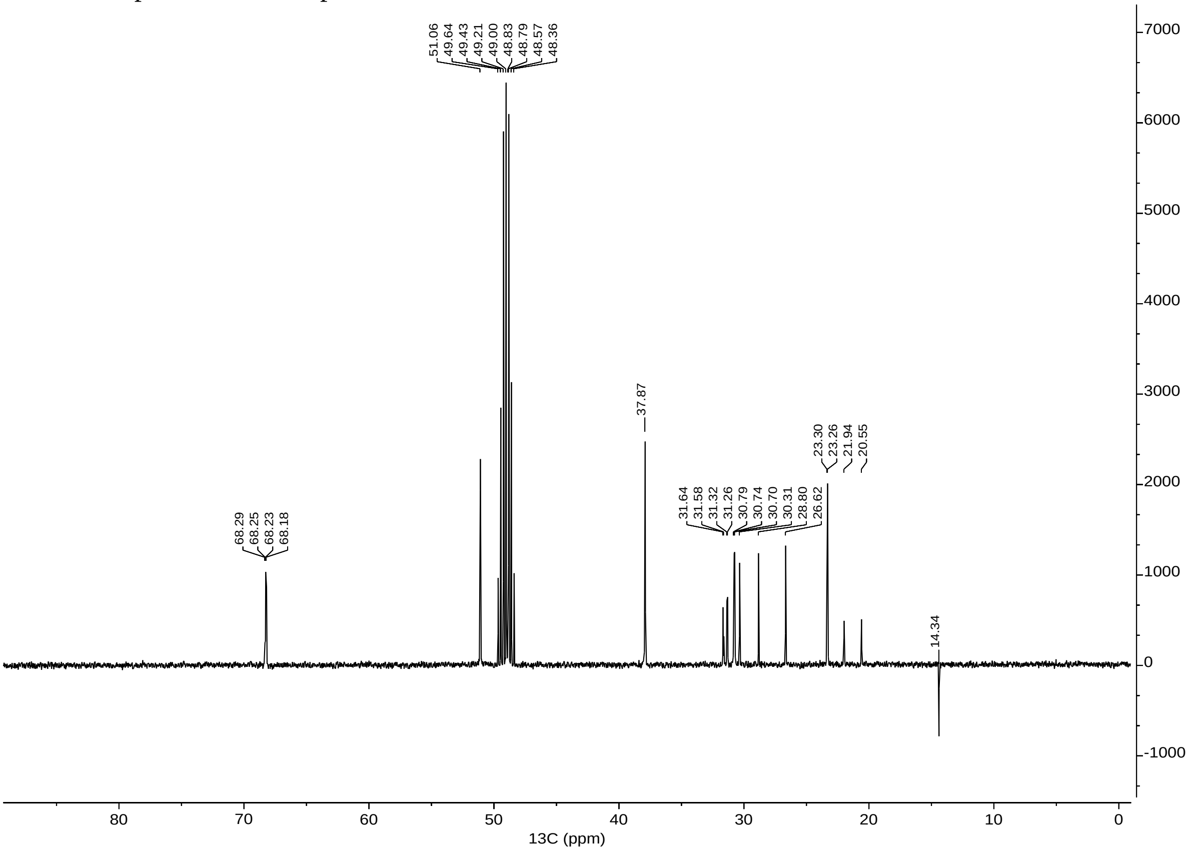

^{31}P{^{1}H} NMR spectrum of compound **82**

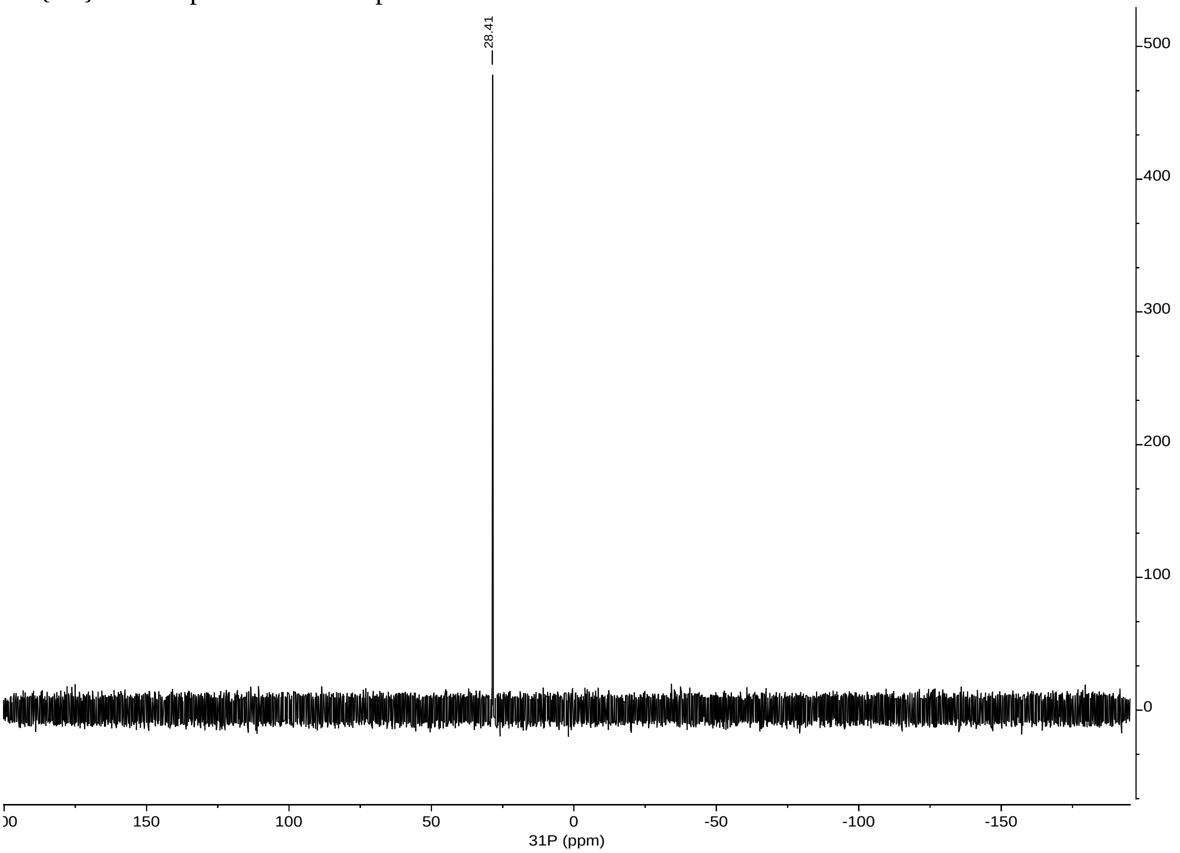

LC-MS chromatograms for compound **82**

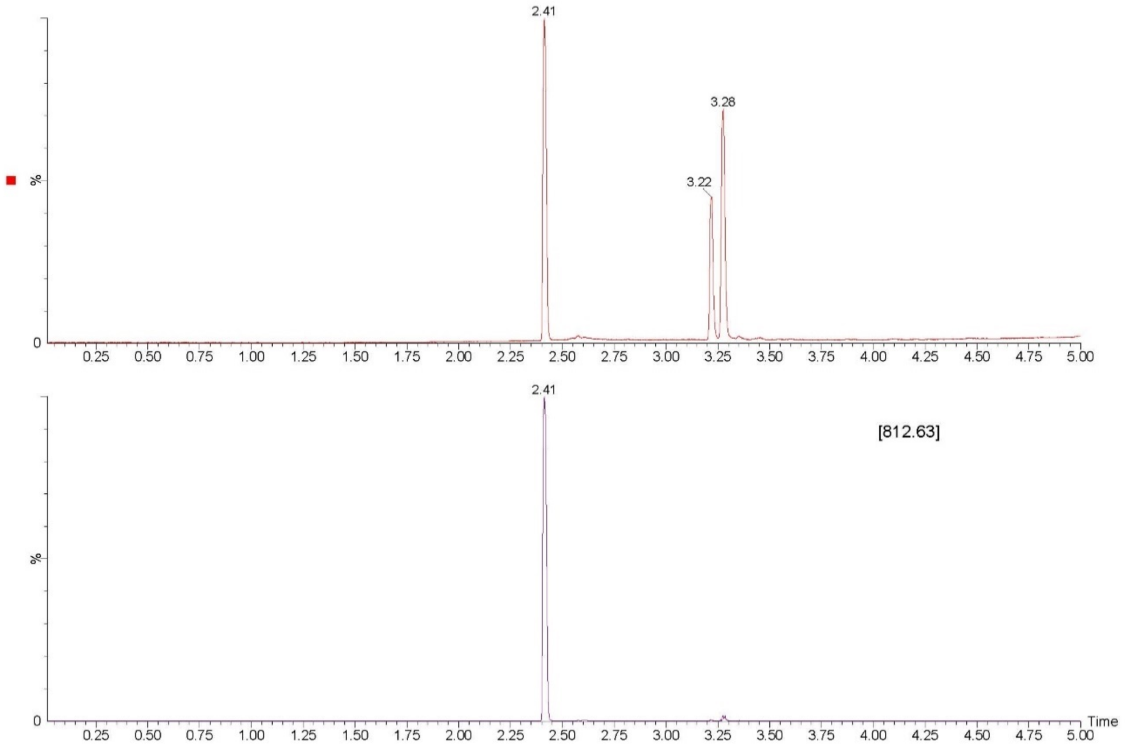

Dihexyl tetradecane-1,14-diyl bis((2-(bis(3-aminopropyl)amino)ethyl)phosphonate) hexahydro-chloride (83)

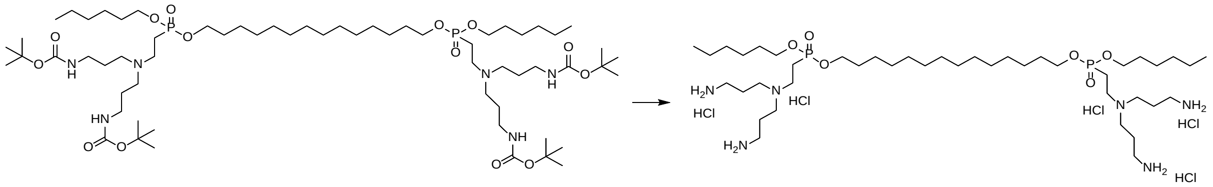

The title compound was prepared according to general method **E** from Boc derivative **S126** (1.30 g, 0.84 mmol) in 90% yield (0.93 g, 0.95 mmol) as a white solid.

^{1}H NMR (400 MHz, CD_3OD): 4.18–4.06 (m, 8H, C*H*$_2$O), 3.50–3.42 (m, 4H, PCH_2CH_2), 3.42–3.35 (m, 8H, C*H*$_2$(CH_2)$_2$$NH_2$), 3.10 (t, 8H, *J* = 7.5 Hz, C*H*$_2$$NH_2$), 2.61–2.45 (m, 4H, PC*H*$_2$), 2.28–2.14 (m, 8H, C*H*$_2$$CH_2NH_2$), 1.79–1.66 (m, 8H, C*H*$_2$$CH_2$O), 1.49–1.25 (m, 32H, CH_3(C*H*$_2$)$_3$, (C*H*$_2$)$_5$(CH_2)$_2$O), 0.97–0.87 (m, 6H, C*H*$_3$).

^{13}C NMR (101 MHz, CD_3OD) δ 68.26 (d, *J* = 6.9 Hz), 68.24 (d, *J* = 6.7 Hz, *C*H_2O), 51.06 (*C*H_2(CH_2)$_2$$NH_2$), 48.83 ($PCH_2$*C*$H_2$), 37.88 (*C*$H_2NH_2$), 32.51 ($CH_3CH_2$*C*$H_2$), 31.61 (d, *J* = 5.8 Hz), 31.57 (d, *J* = 6.0 Hz, *C*H_2CH_2O), 30.80, 30.76, 30.71, 30.32, 26.63, 26.31, 23.64 (CH_3*C*H_2), 23.27 (*C*$H_2CH_2NH_2$), 21.25 (d, *J* = 140.5 Hz, P*C*H_2), 14.39 (*C*H_3).

^{31}P{^{1}H} NMR (162 MHz, CD_3OD): 28.40.

IR ν_{max} (KBr) 2925 (s), 2957 (s), 2857 (s), 2678 (m), 2488 (m, sh), 1608 (m ,sh). 1489 (m), 1467 (m), 1391 (m), 1227 (s), 1037 (m), 992 (s).

HR-MS(ESI^+): For $C_{49}H_{96}N_6O_6P_2$ $(M+2H)^{2+}$ *m*/2*z* calculated 421.34278, found 421.34226.

^{1}H NMR spectrum of compound **83**

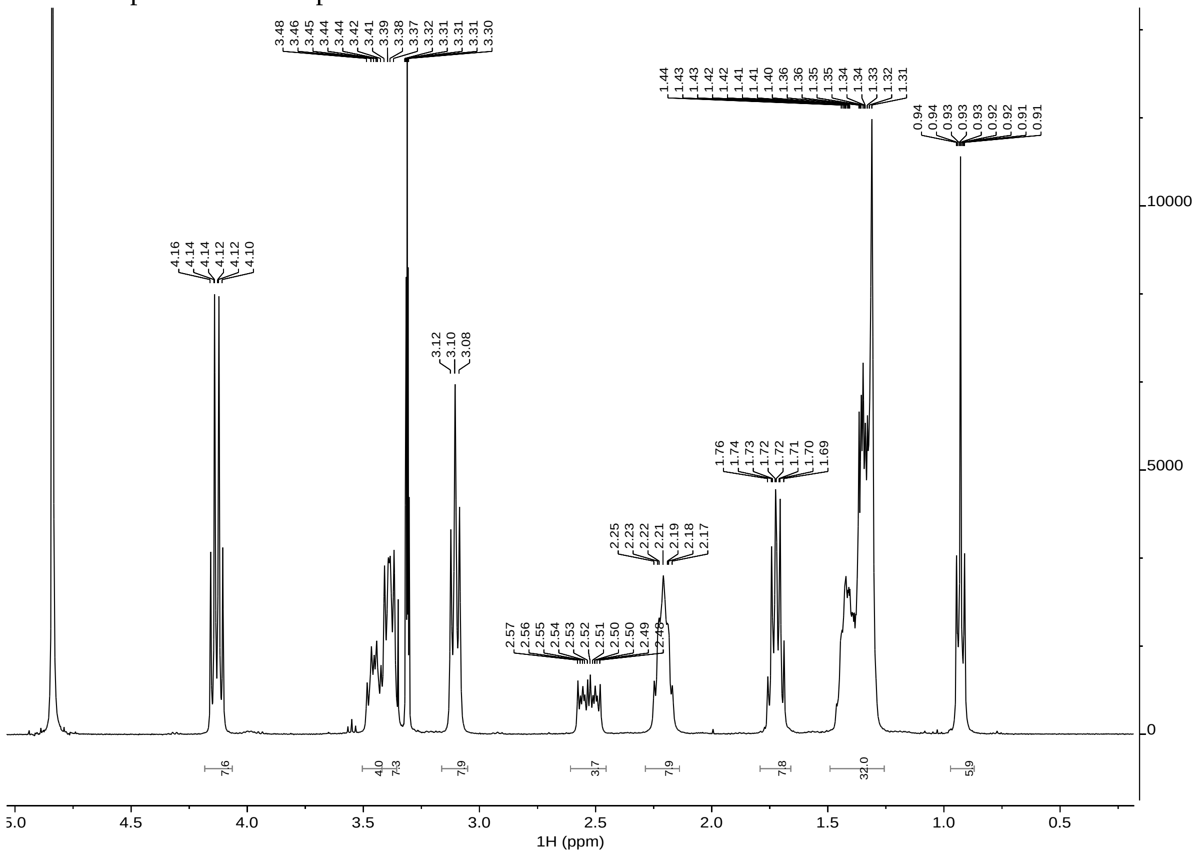

^{13}C NMR spectrum of compound **83**

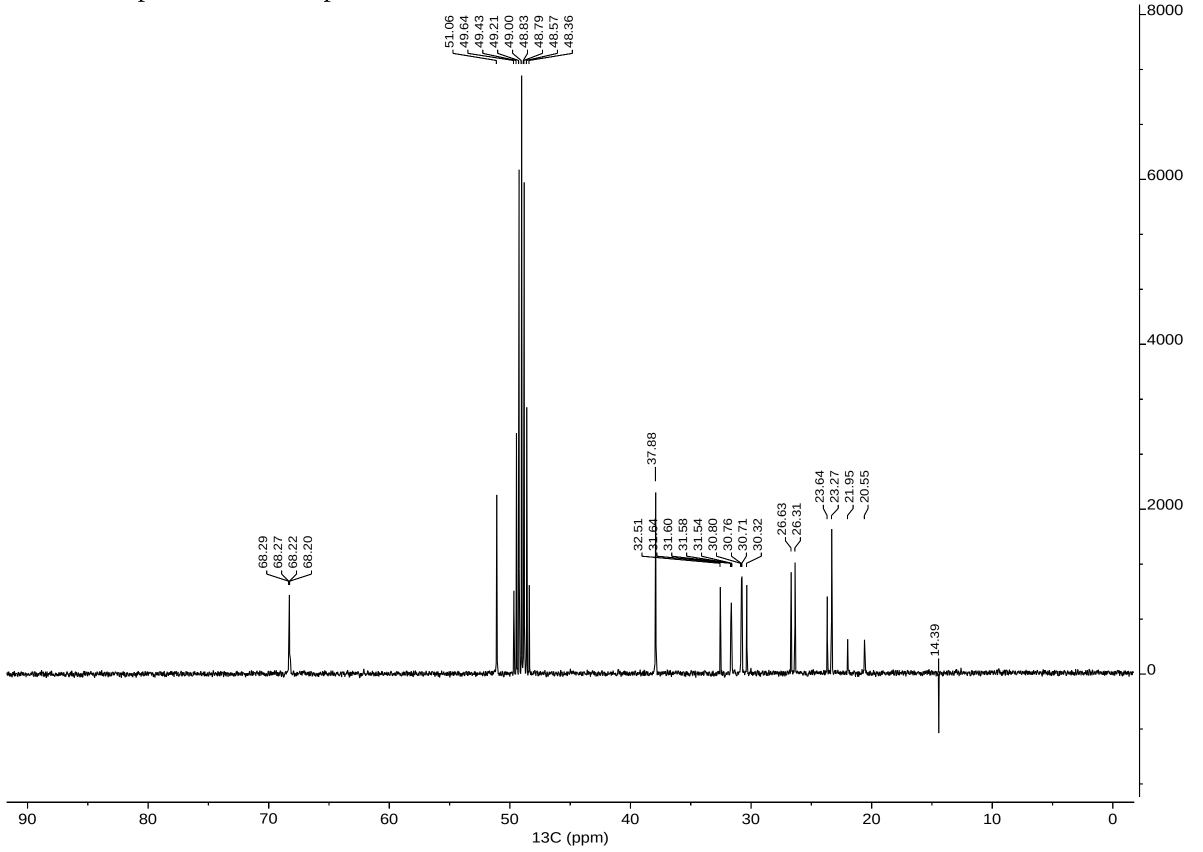

^{31}P{^{1}H} NMR spectrum of compound **83**

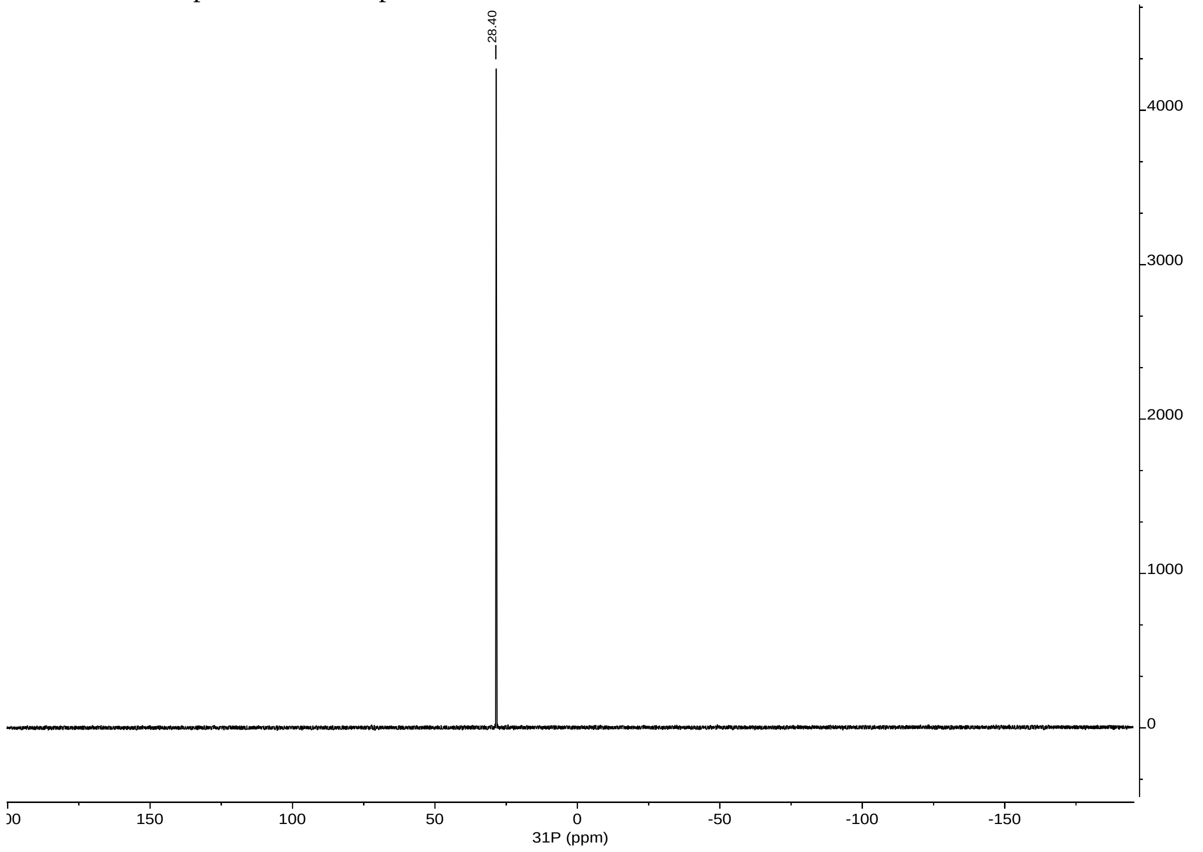

LC-MS chromatograms for compound **83**

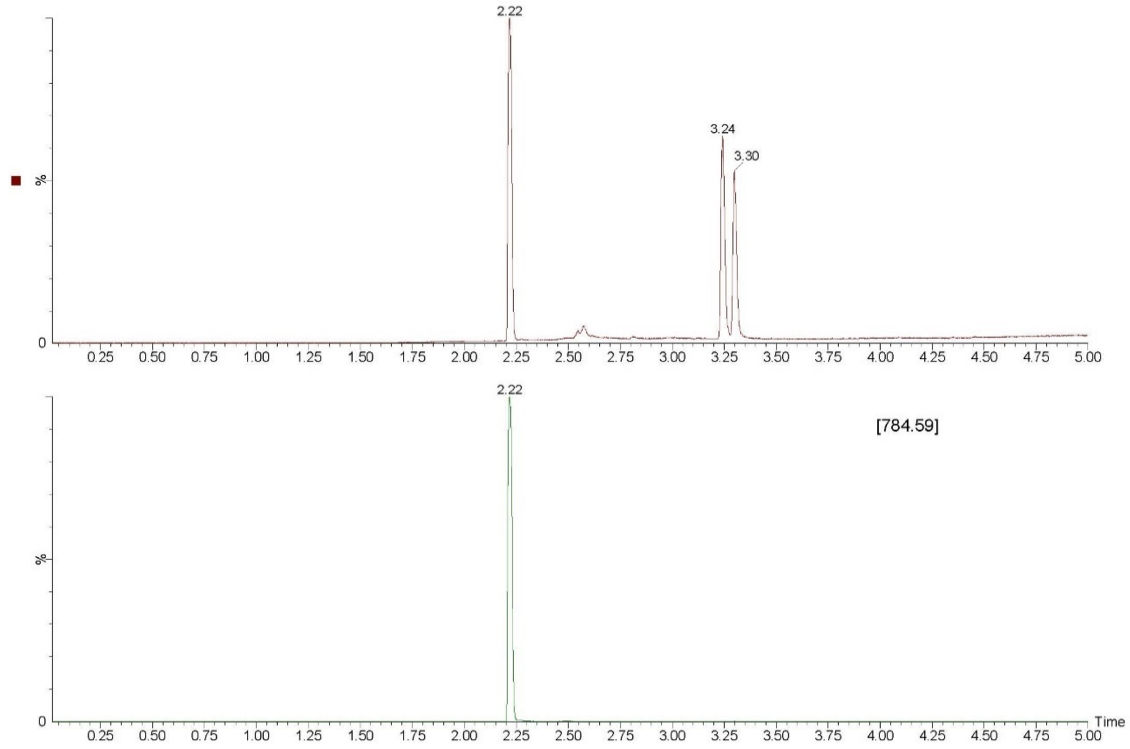

4. Tables S2 and S3

Table S2. *In vitro* skin irritability test.

Sample	Absorbance (OD) mean of 3 tissues	SD (%)	Viability (% of control)
**Negative control**	1.592	3.1	100.0
**Positive control**	0.059	28.0	3.7
**23** (20 mg/L)	1.762	7.7	110.7
**23** (200 mg/L)	1.638	7.1	102.9
**25** (20 mg/L)	1.854	3.7	116.5
**25** (200 mg/L)	1.900	6.7	119.4
**38** (20 mg/L)	1.982	4.3	124.5
**38** (200 mg/L)	1.926	5.2	121.0

Table S3. *In vitro* eye irritability test.

Sample	OD		mean OD	viability	difference of viability
	tissue 1	tissue 2			
Negative control	2.199	2.114	2.157	100.0%	3.9%
Positive control	0.958	0.848	0.903	41.9%	5.1%
**23** (20 mg/L)	2.000	1.974	1.987	92.1%	1.2%
**23** (200 mg/L)	2.142	2.104	2.123	98.5%	1.7%
**25** (20 mg/L)	2.157	2.125	2.141	99.3%	1.5%
**25** (200 mg/L)	2.066	2.125	2.096	97.2%	2.7%
**38** (20 mg/L)	2.014	2.134	2.074	96.2%	5.6%
**38** (200 mg/L)	2.000	2.034	2.017	93.5%	1.6%

5. Table S4

Table S4. Antibacterial activities of LEGO-LPPO.

Cmpd	MBC (mg/L)								
	Efa	Sau	Sep	SauR	EfnR	Eco	EcoR	Pae	PaeR
14	>128	>128	>128	>128	>128	>128	>128	>128	>128
15	>128	64	16	64	>128	>128	>128	>128	128
16	>128	128	32	128	>128	>128	>128	>128	>128
17	8	4	2	2	64	2	8	32	32
18	128	32	8	16	>128	>128	>128	>128	128
19	8	4	2	2	64	2	8	32	32
20	1	1	1	1	4	1	2	2	2
21	2	1	1	1	16	1	4	2	2
22	16	4	4	4	64	8	16	128	16
23	2	1	2	1	32	2	2	64	4
24	128	8	8	8	>128	8	128	>128	>128
25	4	2	1	2	64	2	4	8	4
26	8	1	1	1	16	1	2	8	8
27	>128	32	128	64	>128	>128	>128	>128	>128
28	>128	32	16	64	>128	128	>128	>128	>128
29	32	4	2	2	128	8	16	128	64
30	4	1	1	1	32	2	2	8	16
31	4	2	2	2	64	2	8	128	32
32	8	1	0.5	1	16	2	2	32	32
33	>128	16	8	16	>128	128	>128	>128	>128
34	>128	128	128	128	>128	>128	>128	>128	>128
35	>128	8	8	8	>128	32	128	>128	>128
36	128	2	2	2	>128	4	2	>128	>128
37	>128	16	8	16	>128	>128	128	>128	>128
38	2	1	0.5	1	8	0.5	1	2	2
39	1	0.5	0.5	0.5	8	1	2	2	8
40	2	1	1	1	16	1	2	4	4
41	16	2	1	2	64	4	8	64	64
42	16	1	1	1	32	4	4	32	32
43	4	1	1	1	64	1	4	64	64
44	>128	64	128	128	>128	>128	>128	>128	>128
45	>128	64	32	64	>128	>128	>128	>128	>128
46	4	1	2	1	16	1	4	8	4
47	1	1	1	1	1	1	1	2	2
48	2	0.5	0.5	0.5	4	1	2	2	2
49	1	0.5	0.5	0.5	4	0.5	1	1	4
50	>128	>128	>128	>128	>128	>128	>128	>128	>128
51	>128	8	16	16	>128	128	>128	>128	>128
52	4	1	0.5	1	128	1	2	16	8
53	128	4	4	4	>128	8	64	>128	128
54	32	2	1	2	>128	8	64	64	32
55	>128	>128	128	>128	>128	>128	>128	>128	>128
56	>128	>128	>128	>128	>128	>128	>128	>128	>128
57	>128	16	8	16	>128	>128	>128	>128	128
58	>128	8	4	8	>128	>128	>128	>128	>128
59	32	1	0.5	2	>128	4	8	128	64
60	16	0.5	1	1	32	1	0.5	16	8
61	16	2	2	2	128	32	64	128	128
62	128	8	4	8	>128	16	128	128	128
63	1	1	0.5	0.5	4	1	1	2	2
64	1	0.5	0.2	0.5	2	1	1	2	1
65	128	16	8	16	128	128	128	128	128
66	64	32	8	32	>128	128	>128	>128	128
67	>128	16	8	8	>128	>128	>128	>128	>128

**68**	**64**	**64**	**64**	**128**	**128**	**>128**	**>128**	**128**	**>128**
**69**	**32**	**2**	**1**	**2**	**128**	**64**	**128**	**32**	**16**
**70**	**4**	**1**	**0.5**	**1**	**32**	**2**	**8**	**8**	**8**
**71**	**0.5**	**0.5**	**0.5**	**1**	**1**	**0.5**	**1**	**1**	**1**
**72**	**32**	**4**	**2**	**2**	**128**	**16**	**64**	**128**	**128**
**73**	**2**	**2**	**1**	**2**	**2**	**2**	**2**	**4**	**4**
**74**	**64**	**4**	**2**	**8**	**>128**	**>128**	**>128**	**>128**	**16**
**75**	**32**	**4**	**1**	**8**	**>128**	**>128**	**>128**	**32**	**16**
**76**	**4**	**1**	**0.5**	**2**	**32**	**16**	**32**	**8**	**8**
**77**	**0.5**	**0,5**	**0,25**	**0.5**	**2**	**1**	**2**	**4**	**4**
**78**	**1**	**1**	**0.5**	**1**	**1**	**1**	**1**	**2**	**2**
**79**	**8**	**1**	**0.5**	**4**	**32**	**4**	**32**	**16**	**8**
**80**	**8**	**2**	**1**	**4**	**16**	**16**	**16**	**16**	**8**
**81**	**4**	**2**	**1**	**4**	**32**	**16**	**32**	**4**	**4**
**82**	**1**	**1**	**0.5**	**1**	**4**	**2**	**2**	**4**	**2**
**83**	**1**	**1**	**0.5**	**1**	**2**	**1**	**1**	**2**	**4**

MBC value experiments were performed in triplicates. *Enterococcus faecalis* ATCC 29212 = CCM 4224 (Efa), *Staphylococcus aureus* ATCC 29213 = CCM 4223 (Sau), *Staphylococcus epidermidis* CCM 7221 (Sep), *Staphylococcus aureus* 4591 (SauR), *Enterococcus faecium 419/ANA* (EfnR), *Escherichia coli* ATCC 25922 = CCM 3954 (Eco), *Escherichia coli* CE5556 (EcoR), *Pseudomonas aeruginosa* ATCC 27853 = CCM 3955 (Pae), *Pseudomonas aeruginosa* R (PaeR), BSA: in the presence of 4% BSA.
